# STAT3 Targets Suggest Mechanisms of Aggressive Tumorigenesis in Diffuse Large B-Cell Lymphoma

**DOI:** 10.1534/g3.113.007674

**Published:** 2013-10-18

**Authors:** Jennifer Hardee, Zhengqing Ouyang, Yuping Zhang, Anshul Kundaje, Philippe Lacroute, Michael Snyder

**Affiliations:** * Department of Genetics, Stanford University School of Medicine, Stanford, California 94305; † Department of Molecular, Cellular, and Developmental Biology, Yale University, New Haven, Connecticut 06520; ‡ Department of Computer Science, Stanford University School of Engineering, Stanford, California 94305

**Keywords:** genomics, next-generation sequencing, cancer signaling, signal transduction, oncogenic pathways

## Abstract

The signal transducer and activator of transcription 3 (STAT3) is a transcription factor that, when dysregulated, becomes a powerful oncogene found in many human cancers, including diffuse large B-cell lymphoma. Diffuse large B-cell lymphoma is the most common form of non-Hodgkin’s lymphoma and has two major subtypes: germinal center B-cell−like and activated B–cell—like. Compared with the germinal center B-cell−like form, activated B-cell−like lymphomas respond much more poorly to current therapies and often exhibit overexpression or overactivation of STAT3. To investigate how STAT3 might contribute to this aggressive phenotype, we have integrated genome-wide studies of STAT3 DNA binding using chromatin immunoprecipitation-sequencing with whole-transcriptome profiling using RNA-sequencing. STAT3 binding sites are present near almost a third of all genes that differ in expression between the two subtypes, and examination of the affected genes identified previously undetected and clinically significant pathways downstream of STAT3 that drive oncogenesis. Novel treatments aimed at these pathways may increase the survivability of activated B-cell−like diffuse large B-cell lymphoma.

The transcription factor signal transducer and activator of transcription 3 (STAT3) is a key signal transducer that regulates gene expression in response to interleukins and cytokines, as well as a wide variety of other extracellular signals (Zhong *et al.* 1994; Baker *et al.* 2007; Minegishi 2009). The binding of one of these messengers to its receptor launches a tyrosine phosphorylation cascade that results in the cytosolic activation and dimerization of STAT3, which is then imported to the nucleus, where it binds its target sequences. STAT3 mediates the expression of a large number of genes and plays a key role in many cellular processes, especially those related to cell growth and apoptosis (Baker *et al.* 2007).

As the result of these proliferative and antiapoptotic effects, STAT3 is also a powerful oncogene (Alvarez and Frank 2004). Constitutively active STAT3, caused by upstream dysregulation, is found in a large number of human cancers and is generally associated with a poorer prognosis (Benekli *et al.* 2003; Turkson 2004; Hodge *et al.* 2005). In particular, overactive STAT3 is frequently found in diffuse large B-cell lymphoma (DLBCL) and is associated with poorer outcomes (Ding *et al.* 2008; Wu *et al.* 2011). DLBCL is the most common form of lymphoma and comprises at least two subtypes: germinal center B-cell-like (GCB) and activated B-cell-like (ABC) (Alizadeh *et al.* 2000; Rosenwald *et al.* 2002; Wright *et al.* 2003; American Cancer Society 2012). These two subtypes have significant differences in three-year survival, which is nearly 85% for GCB but only 65–70% for patients with ABC (Fu *et al.* 2008; Lenz *et al.* 2008). High levels of STAT3 generally are found only in the activated B-cell−like subtype.

In the present study, we sought to further understand the difference in STAT3 function between these two subtypes through mapping its binding regions (BRs) and analyzing gene expression in GCB and ABC patient tumor-derived cell lines. We performed ChIP-Seq (chromatin immunoprecipitation followed by DNA sequencing) experiments to map STAT3 binding sites and RNA-Seq to analyze the global gene expression patterns. We then synthesized these data to determine which genetic loci show both differential STAT3 binding and differential mRNA expression. We found that STAT3 likely up-regulates a number of oncogenic pathways to promote aggressive tumor growth and migration.

## Materials and Methods

Cell lines were grown at 37° and 5% CO_2_. SU-DHL2, SU-DHL4, SU-DHL6, SU-DHL10, OCI-Ly7, and U-2932 were grown in RPMI 1640 media supplemented with 15% FBS and antibiotics. OCI-Ly3 and OCI-Ly10 were grown in IMDM media supplemented with 15% fetal bovine serum, antibiotics, and 55 µM beta-mercaptoethanol. Western blots were performed on whole-cell lysate, with equal protein loading in each lane, with the use of anti-STAT3 rabbit polyclonal antibody sc-482X (Santa Cruz Biotechnology, Inc.); anti-pSTAT3-Y705 mouse monoclonal antibody sc-8059X (Santa Cruz Biotechnology, Inc.); and anti-GAPDH mouse monoclonal antibody ab8245 (Abcam). ChIP-sequencing was performed with the anti-STAT3 antibody sc-482X on formaldehyde-crosslinked pellets of 1 × 10^6^ cells. DNA was mechanically sheared using a Branson sonicator, then immunoprecipitated for 16 hr. Bound DNA was recovered on protein A-agarose beads and purified via ethanol precipitation. mRNA for RNA-sequencing was isolated directly from whole cell lysate using magnetic poly-dT beads (Dynabeads mRNA DIRECT Kit; Invitrogen), then chemically fragmented (RNA Fragmentation Reagents; Ambion). cDNA was synthesized using random hexamer primers. For library preparation, standard Illumina GA-IIx primers were ligated and gel purification was used to size-select DNA in the 150- to 300-bp range. Single-ended 36-bp reads were generated for both ChIP- and RNA-sequencing runs.

### Statistical analysis

Sequencing results were mapped to the human genome (hg19) using Bowtie (Langmead *et al.* 2009). STAT3 ChIP-sequencing peaks were compared with a non-IP’d genomic DNA control, and identified using the SPP peak caller (Kharchenko *et al.* 2008). Replicates were analyzed using irreproducible discovery rate analysis to identify strong, repeatable peaks for each cell line (Li *et al.* 2011). These lists were combined and any overlapping or abutting peaks were merged into broader BRs (Kasowski *et al.* 2010). BRs that occurred in only one cell line were eliminated from further analysis. The ChIP-sequencing data for each line were rescored to determine how many fragments mapped to each BR. RNA-sequencing mapped reads were intersected with the RefSeq database to generate read counts for each annotated gene. Both data sets were then normalized to library size with DESeq, and to contrast the two subtypes, the DESeq parameterized negative binomial model was used to obtained the *P*-values for each BR or gene (Anders and Huber 2010). To correct for multiple comparisons, *P*-values were adjusted with the Benjamini-Hochberg procedure (Benjamini and Hochberg 1995). ChIP-Seq BRs were associated with genes that they might regulate via the Genomic Regions Enrichment of Annotations Tool using its default association settings (GREAT; McLean *et al.* 2010) . This list of genes associated with significant changes in STAT3 binding was compared with the list of genes with significant expression changes using the online tool Galaxy (Giardine *et al.* 2005; Blankenberg *et al.* 2010; Goecks *et al.* 2010). Extended materials and methods are available as Supporting Information, File S1, complete data tables are available as File S2. All ChIP-Seq and RNA-Seq data are available in the NCBI GEO public database as series accession no. GSE50724. Within this accession, ChIP-Seq data are found in the subseries GSE50723, and RNA-Seq data are in the subseries GSE50721. Individual data files are assigned the consecutive accession numbers GSM1227193-GSM1227214.

## Results and Discussion

### STAT3 is overexpressed and overactive in ABC DLBCL

In normal B-cells, STAT3 is expressed at low endogenous levels in the cytosol. High levels of STAT3 expression and activation frequently are found in ABC DLBCL, suggesting that it plays a major role in the oncogenesis of this subtype (Ding *et al.* 2008; Wu *et al.* 2011). To investigate the levels of activation of STAT3 in the treatment-responsive GCB lymphoma relative to ABC lymphoma, we selected eight patient tumor–derived cell lines for study and examined levels of total and activated STAT3 by using immunoblot analysis. Four of these cell lines have been classified by Drexler (2010) as GCB (SU-DHL4, SU-DHL6, SU-DHL10, OCI-Ly7) and four as ABC (SU-DHL2, OCI-Ly3, OCI-Ly10, U-2932).

**Figure 1:**
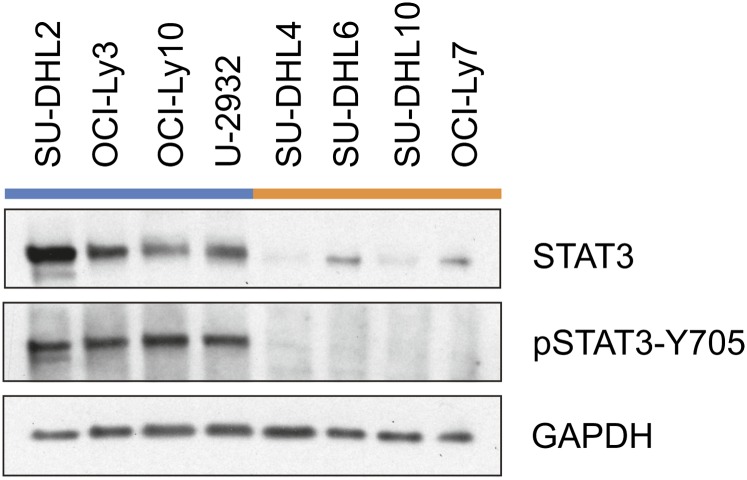
Western blot analysis of STAT3 in ABC and GCB cell lines. Western immunoblots were performed on whole-cell lysate from DLBCL cell lines probed with antibodies against STAT3, tyrosine-phosphorylated STAT3, and glyceraldehyde 3-phosphate dehydrogenase (GAPDH). Fold change was measured by densitometric quanitification using NIH ImageJ software, normalized to a GAPDH loading control.

Whole-cell lysates from each line were prepared and probed with antibodies that recognize total STAT3 as well as those that recognize only the phosphorylated (Tyr705) activated form (Shuai *et al.* 1993; Wen *et al.* 1995; Zhang *et al.* 1995). STAT3 protein is present in all of the GCB and ABC cell lines; however, it is at much greater levels (7.2-fold) in all four ABC cell lines (Figure 1). Furthermore, the phosphorylated form of the protein exhibits an even larger difference (56-fold) between the GCB and ABC lines (Figure 1).

### STAT3 shows differential binding in ABC relative to GCB cells

To better understand how STAT3 induces aggressive behavior in ABC cells, we next sought to determine the chromosomal targets of STAT3 in all eight cell lines. We performed ChIP-Seq by using an antibody prepared against the protein’s C-terminal region, the area most divergent among STAT family proteins (Baker *et al.* 2007). Immunoblot analyses indicate that this antibody recognizes a single band of the expected molecular mass, which was confirmed as STAT3 by immunoprecipitation followed by mass spectrometry (see Figure S1). We performed ChIP-Seq for all eight cell lines along with genomic DNA input controls. Two to nine biological replicates were carried out for each line; replicates were sequenced to an average depth of 18 million mapped reads (87% mapping; see Table S1). All replicates were used in the analysis.

The ChIP-Seq binding peaks were scored using the SPP peak caller and irreproducible discovery rate analysis (Kharchenko *et al.* 2008; Li *et al.* 2011), which resulted in the identification of 1000−6500 strongly reproducible peaks per cell line (Table 1). Overlapping or abutting peaks were merged into BRs to facilitate comparison between cell lines, yielding a total of 10,337 distinct STAT3 BRs that are occupied in at least two of the eight cell lines. These BRs occur throughout the genome, with no obvious hot spots.

**Table 1:** STAT3 peaks identified by IDR analysis by cell line

Name	Peaks by IDR
ABC cell lines	
SU-DHL2	5375
OCI-Ly3	2885
OCI-Ly10	3258
U-2932	5180
GCB cell lines	
SU-DHL4	6535
SU-DHL6	4570
SU-DHL10	1067
OCI-Ly7	6262

STAT3, signal transducer and activator of transcription 3; IDR, irreproducible discovery rate; ABC, activated B-cell-like; GCB, germinal center B-cell-like.

**Figure 2:**
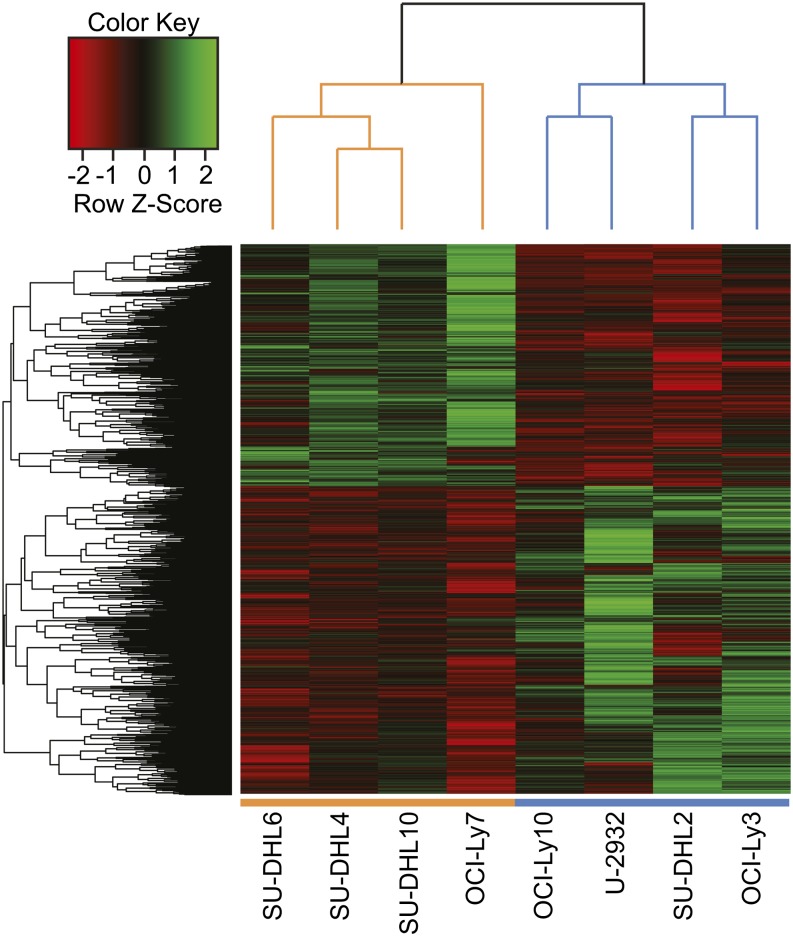
Hierarchical clustering analysis of STAT3 BRs. Clustering was performed on the 3524 regions that are differentially bound by STAT3 at FDR < 0.05 in ABC cell lines *vs.* GCB cell lines. Green and red blocks, respectively, represent high and low number of fragments sequenced in that BR relative to the average, whereas black blocks indicate no difference in expression. Fragment counts were log transformed to approximate a normal distribution, standardized by cell line, and each BR was normalized with sum of squares set to unity. GCB cell lines are represented by orange bars and ABC cell lines by blue bars. Generated using the heatmap.2 function in the R package gplots (R Development Core Team 2008; Warnes *et al.* 2011).

**Figure 3:**
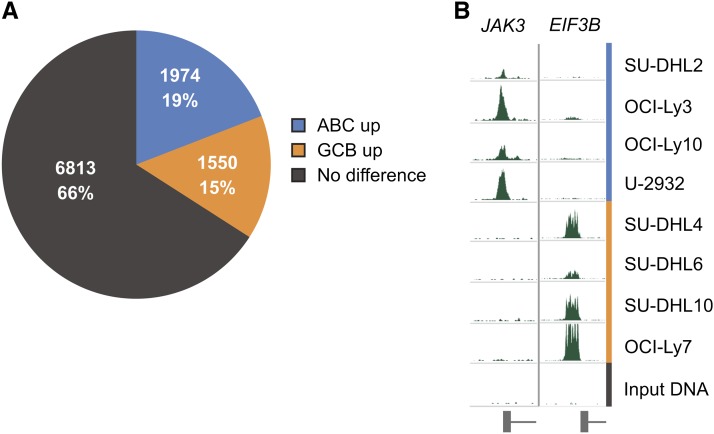
Differential STAT3 BRs. (A) STAT3 BRs divided by occupancy pattern. (B) Examples of differentially occupied STAT3 BRs. *JAK3* shows strong binding in the ABC cell lines: SU-DHL2, OCI-Ly3, OCI-Ly10, and U-2932. *EIF3B* shows strong binding in the GCB cell lines: SU-DHL4, SU-DHL6, SU-DHL10, and OCI-Ly7.

To identify target regions differentially bound by STAT3 in the ABC and GCB subtypes, we used the parameterized negative binomial model in DESeq (Anders and Huber 2010) to identify those BRs that displayed significantly different normalized fragment counts between the ABC and GCB cell lines, then corrected for multiple comparisons using the Benjamini-Hochberg procedure (Benjamini and Hochberg 1995). Of the 10,337 high-confidence BRs, one third (n = 3524) are differentially bound by STAT3 between the two subtypes at a false-discovery rate (FDR) < 0.05. (For a complete list of peaks by enriched subtype, see Table S4.) When these differentially bound peaks are clustered, the DLBCL cell lines cluster according to their GCB-ABC subtype (Figure 2). Consistently, more BRs are strongly bound in ABC than in GCB, although BRs with increased STAT3 binding occur surprisingly frequently in the GCB cell lines given their low level of STAT3 expression: 44% of differentially bound BRs (n = 1550) show more STAT3 binding in GCB, whereas 56% (n = 1974) are more strongly bound in ABC (Figure 3A). STAT3 protein levels are very low in GCB cell lines, so one possible explanation for its equivalent number of binding sites is simply that they represent lower occupancy regions. Indeed, we noticed the signal strength for the GCB lines was lower than that of the ABC cell lines and required additional ChIP-Seq replicate experiments to identify peaks.

STAT3 has a binding peak 14 bp upstream of the transcription start site (TSS) of *JAK3*, and this binding is much stronger (fold change = 12.47, FDR = 5.21 × 10^−27^) in the ABC subtype (Figure 3B). JAK3 associates with the interleukin (IL)-2 family γ-chain receptors, transducing the signal from cytokines such as IL-2, IL-7, IL-9, IL-15, and IL-21 (Suzuki *et al.* 2000). Although JAK2 is often regarded as the primary partner of STAT3, constitutively active JAK3:STAT3 signaling has been reported as a poor prognosis indicator in anaplastic large-cell lymphoma and in colon cancer cell lines (Lin *et al.* 2005; Han *et al.* 2006). Our results suggest that STAT3 activates key regulators of cytokine signaling.

Conversely, *EIF3B* shows increased STAT3 binding in the GCB subtype cell lines (fold change = 0.01, FDR = 2.21 × 10^−3^; Figure 3B). As with JAK3, the STAT3 binding peak is just upstream of the TSS, a common location for gene regulation by transcription factors. EIF3B is a major scaffolding subunit of the eukaryotic translation initiation factor 3 (eIF-3) complex, a complex required for the initiation of protein synthesis. Intriguingly, short hairpin RNA knockdown of eIF-3B induces apoptosis in glioblastoma (Liang *et al.* 2012), suggesting that eIF-3B may also play a role in GCB DLBCL.

### 

#### Systematic analyses reveal that STAT3 binding associates with oncogenic pathways in the ABC subtype:

After the BRs with significant binding differences were identified, we associated them with nearby genes and then analyzed enrichment across a variety of ontologies using GREAT (McLean *et al.* 2010). All but 19 BRs fall within the putative regulatory domain of at least one gene, and the majority are associated with one (n = 4203) or two (n = 6095) nearby genes.

**Figure 4:**
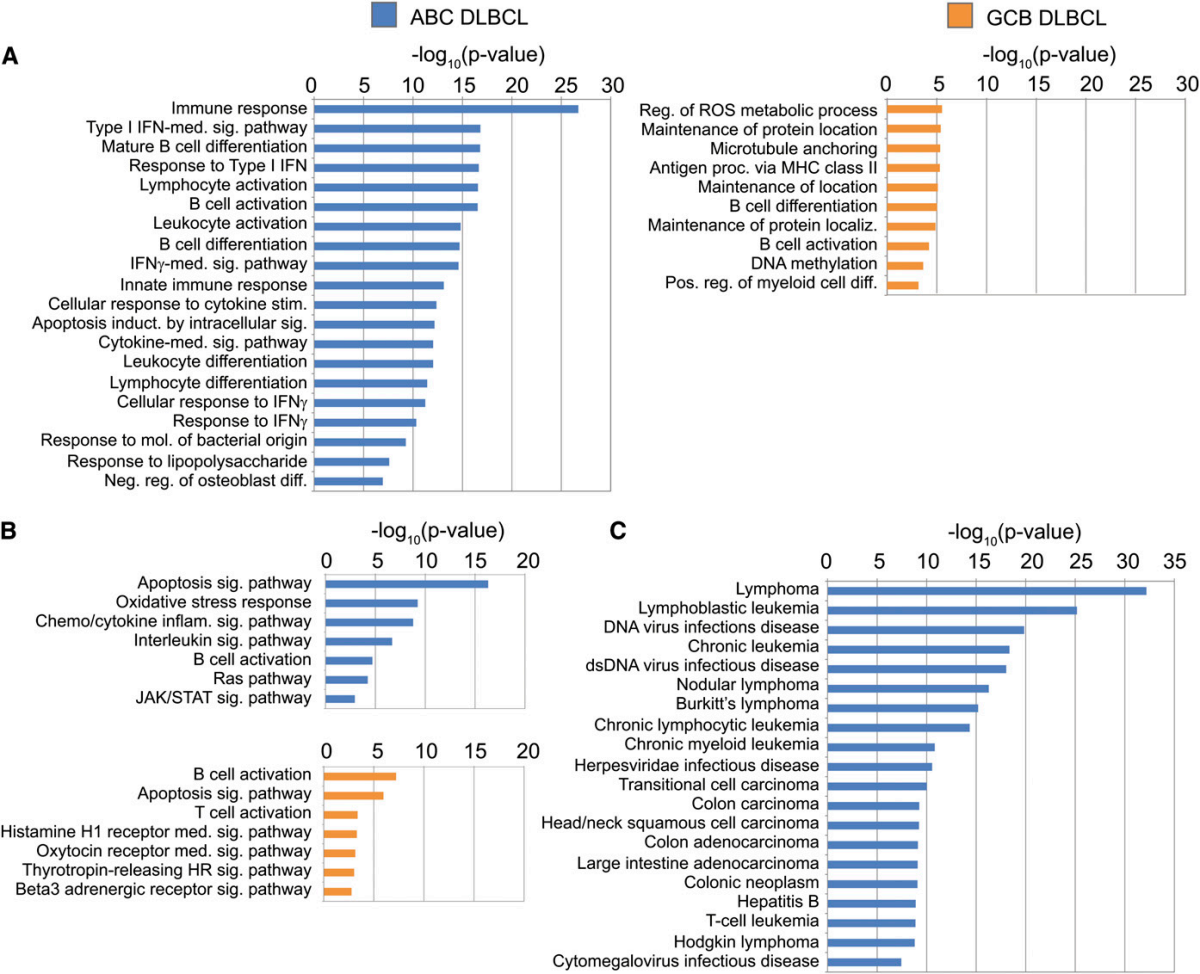
ChIP-Seq BR enrichment for ontological categories. Scale is the –log_10_(*P*-value) of the binomial *P*-value, equivalent to the exponent. (A) All enriched GO Biological Processes, *P* < 0.05. (B) All enriched PANTHER Pathways, *P* < 0.05. (C) All enriched Disease Ontology listings, *P* < 0.05. Data shown for ABC only, as GCB had no significantly enriched categories.

The Gene Ontology Biological Processes (GO BP) ontology shows that STAT3 BRs occupied in ABC DLBCL are located near genes involved in B-cell activation and mature B-cell differentiation (Figure 4A). This result is consistent with STAT3’s role in B-cell maturation and with the theory that more mature B-cells give rise to the ABC subtype (Angelin-Duclos *et al.* 2000; Falini *et al.* 2000; Fornek *et al.* 2006; Avery *et al.* 2010). The Disease Ontology analysis reveals that the 1974 ABC-enriched STAT3 BRs fall near genes associated with many forms of cancer, especially lymphomas and leukemias (Figure 4C).

Although there are nearly as many STAT3 BRs with strong binding in the GCB cell lines as in the ABC lines, these BRs and their associated genes do not point to a clear oncogenic program like the ABC BRs do. Far fewer categories are enriched, and at lower significance levels; furthermore, the 1550 GCB-specific BRs are not enriched for any disease categories. The enriched PANTHER Pathway category “apoptosis signaling pathway” primarily contains genes that induce apoptosis for the GCB-bound BRs, but the ABC subtype list includes genes that both up- and down-regulate apoptosis (Figure 4B). Taken together, these data show that in the ABC subtype, overactive STAT3 substantially changes its binding behavior and promotes a genetic program that blocks apoptotic signaling.

#### STAT3 binding sites in DLBCL are seen in other hematopoetic and cancer cell lines:

We compared the overall list of STAT3 BRs found in DLBCL with BRs identified in three other cell lines: GM12878, an EBV-transformed B lymphocyte line; HeLa S3, a suspension-tolerant clonal derivative of the HeLa cervical adenocarcinoma line; and LoVo, a colorectal adenocarcinoma cell line. GM12878 and HeLa S3 experiments were performed as part of the ENCODE project (The ENCODE Project Consortium *et al.* 2011) as GEO nos. GSM935557 and GSM935276, whereas the LoVo experiments were performed by Yan *et al.* (2013) as GEO no. GSM1208799.

On the basis of a 1-bp overlap, 3932 (38%) of the 10,337 STAT3 BRs observed in our DLBCL cell lines are also found in GM12878 cells, whereas 1143 (11%) are found in LoVo and 1063 (10%) in HeLa S3 (see Figure S2). It is not surprising that GM12878 has the greatest concordance with DLBCL, as it is the most similar cell type. DLBCL and GM12878 share 2958 BRs not found in either HeLa S3 or LoVo. As determined using GREAT (McLean *et al.* 2010), these BRs are found near genes enriched for type I interferon and cytokine signaling, many B-cell properties including activation and differentiation, DNA damage response, and the JAK/STAT pathway itself (including *JAK1* and *JAK3*; *STAT1*, *3*, *4*, *5A*, and *5B*; and the down-regulators *SOCS1*, *PIAS2*, and *PTPRC*). More than one-third (36%, n = 1406) of these regions are subtype-specific in DLBCL, and three quarters of those (74%, n = 1049) are more strongly bound in the ABC subtype. This bias toward ABC regions may be due to the presence of the Epstein-Barr virus used to transform GM12878; EBV is a well-known oncovirus that promotes the development of hematopoetic cancers (Pallesen *et al.* 1991; Yu *et al.* 1996; Siu *et al.* 2002; Brady *et al.* 2007; Castillo *et al.* 2008) and whose presence contributes to poorer survival in DLBCL specifically (Park *et al.* 2007).

The 1063 STAT3 binding sites shared between DLBCL and HeLa S3 are enriched for genes involved in a number of cancer-related functions: chromatin structure, single-stranded DNA binding, DNA damage checkpoints, abnormal interferon levels, and the apoptotic signaling pathway. One third (n = 353) of these regions are subtype-specific in DLBCL, and the vast majority (90%, n = 316) are more strongly bound in the ABC subtype. The ABC-specific BRs in HeLa S3 and GM12878 fall near genes that enrich many of the same ontological categories, including immune response, the JAK/STAT pathway, interferon signaling and response, and cytokine signaling and response. They also share Disease Ontology categories that indicate immune system activation, such as leukemias of various types, allergies, hepatitis, and response to infection. In contrast, the 1143 shared LoVo BRs are primarily enriched for genes involved in RNA processing, splicing, and translation, rather than cancer-related categories. Bias toward ABC-specific BRs is not seen in the LoVo colon carcinoma line, where BRs specific to each DLBCL subtype are equally represented. Only 143 STAT3 BRs are found in all four cell types. These are generally not near genes enriched for any pathways or ontological categories. However, there is overlap with a curated gene sets for genes that are targets of c-Myc.

### Subtypes show differential gene expression

To better understand the biology of the GCB and ABC subtypes and to relate their gene expression profiles to identified STAT3 targets, we examined global mRNA expression in both subtypes using RNA-Seq analysis (Morin *et al.* 2008). RNA-Seq was performed on mRNA isolated from all eight cell lines. Two biological replicates per cell line were analyzed with an average of 22 million mapped reads (86% mapping; see Table S2).

To identify genes that are differentially expressed in each subtype, we used the parameterized negative binomial model in DESeq (Anders and Huber 2010) to identify annotated RefSeq genes that displayed significantly different normalized read counts between the ABC and GCB cell lines, then corrected for multiple comparisons by using the Benjamini-Hochberg procedure (Benjamini and Hochberg 1995). We found many genes that exhibit differential gene expression in the GCB and ABC cell types, including several genes that play a role in apoptosis and cancer. This analysis also successfully re-identified many genes that have previously been shown to be biomarkers for each DLBCL subtype (Rosenwald *et al.* 2002; Tirado *et al.* 2012), including *BCL2*, *IRF4*, *MME* (*CD10*), and *BCL6* (Table 2). Surprisingly, the established ABC subtype biomarker *MUM1* (Hans *et al.* 2004; Sweetenham 2005; Anderson *et al.* 2009) showed no significant expression change (FDR = 0.92) but did have a STAT3 BR 344 bp upstream of its TSS with significantly more binding in GCB (fold change = 0.46, FDR = 4.89 × 10^−3^).

**Table 2:** Validated DLBCL subtype signature genes

Gene Name	Gene Description	GCB Read Counts	ABC Read Counts	Fold Change	FDR
ABC signature					
* BCL2*	B-cell CLL/lymphoma 2	4,599.52	22,162.49	4.82	4.90E-05
* CCND2*	Cyclin D2	31.20	3692.67	118.60	1.56E-18
* CFLAR*	CASP8 and FADD-like apoptosis regulator	460.63	6463.37	14.03	5.52E-09
* FOXP1*	forkhead box P1	1202.37	6588.56	5.46	1.13E-04
* IRF4*	Interferon regulatory factor 4	976.01	21,475.28	22.01	2.43E-14
* NFKBIZ*	Nuclear factor of kappa light polypeptide gene enhancer in B-cells inhibitor, zeta	962.34	8119.55	8.46	5.63E-06
GCB signature					
* BCL6*	B-cell CLL/lymphoma 6	15,105.68	5124.25	0.34	1.94E-02
* MAPK10*	Mitogen-activated protein kinase 10	1473.10	57.42	0.04	4.45E-05
* MME (CD10)*	Membrane metallo-endopeptidase	6163.64	663.16	0.11	6.01E-05
* MYBL1*	v-myb myeloblastosis viral oncogene homolog (avian)-like 1	2932.21	661.39	0.23	4.99E-02

DLBCL, diffuse large B-cell lymphoma; GCB, germinal center B-cell-like; ABC, activated B-cell-like; FDR, false-discovery rate; CLL, chronic lymphocytic leukemia.

**Figure 5:**
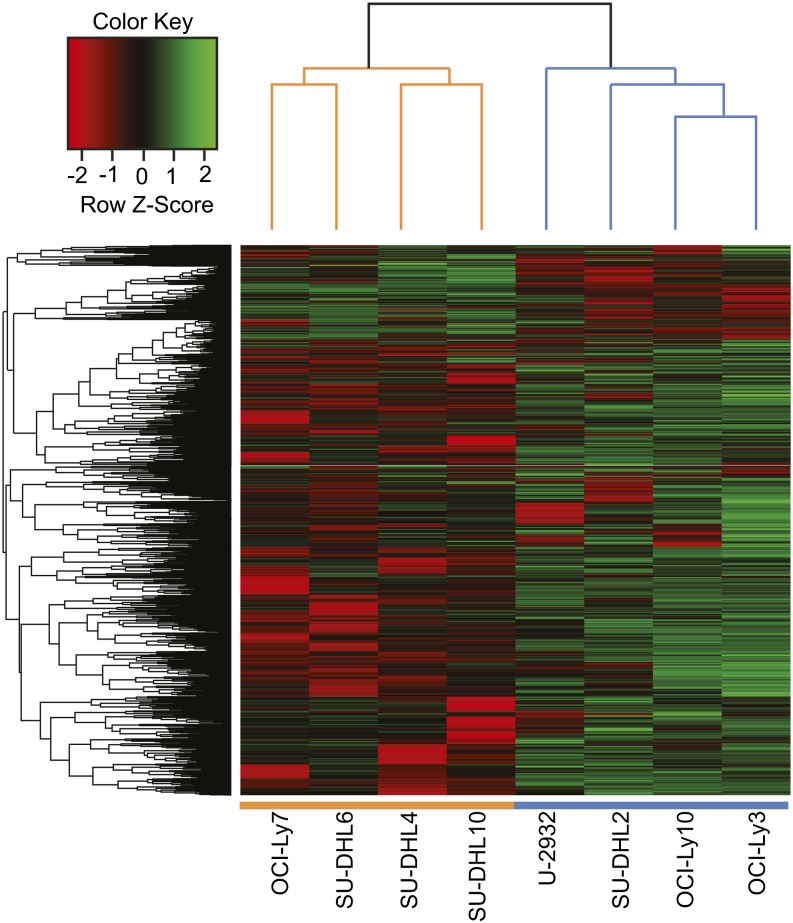
Hierarchical clustering analysis of gene expression. Clustering was performed on the 1545 genes that are differentially expressed at FDR < 0.05 in ABC cell lines *vs.* GCB cell lines. Green and red blocks, respectively, represent high and low signals in the BR relative to the average, whereas black blocks indicate no difference in expression. Read counts were log transformed to approximate a normal distribution, standardized by cell line, and each gene was normalized with sum of squares set to unity. GCB cell lines are represented by orange bars and ABC cell lines by blue bars. Generated using the heatmap.2 function in the R package gplots (R Development Core Team 2008; Warnes *et al.* 2011).

**Figure 6:**
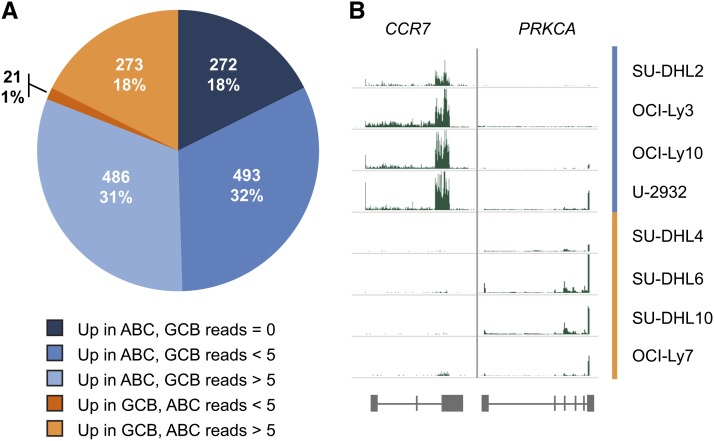
Differential gene expression. (A) RefSeq genes with differential expression (FDR < 0.05) divided by expression pattern. (B) Examples of differentially expressed genes. *CCR7* shows greater expression in the ABC cell lines: SU-DHL2, OCI-Ly3, OCI-Ly10, and U-2932. *PRKCA* shows greater expression in the GCB cell lines: SU-DHL4, SU-DHL6, SU-DHL10, and OCI-Ly7.

A total of 1545 genes are differentially expressed between subtypes at an FDR < 0.05 (Figure 5), which is approximately 7% of the transcriptome. The vast majority of these genes (81%, n = 1251) are more highly expressed in the ABC cell lines. In contrast, only 294 genes (19%) are more highly expressed in the GCB cell lines. Interestingly, half (n = 765) of the genes with greater ABC subtype expression demonstrate very low read counts (<5) in the GCB cell types. Conversely, only 21 genes that are more highly expressed in GCB are unique to that subtype (Figure 6A). The prevalence of such “on/off” genes indicates that the major differences between ABC and GCB DLBCL are due almost exclusively to additional gene expression in ABC, rather than the two subtypes having divergent but equally active genetic programs.

### 

#### Genes up-regulated in ABC subtype:

**Figure 7:**
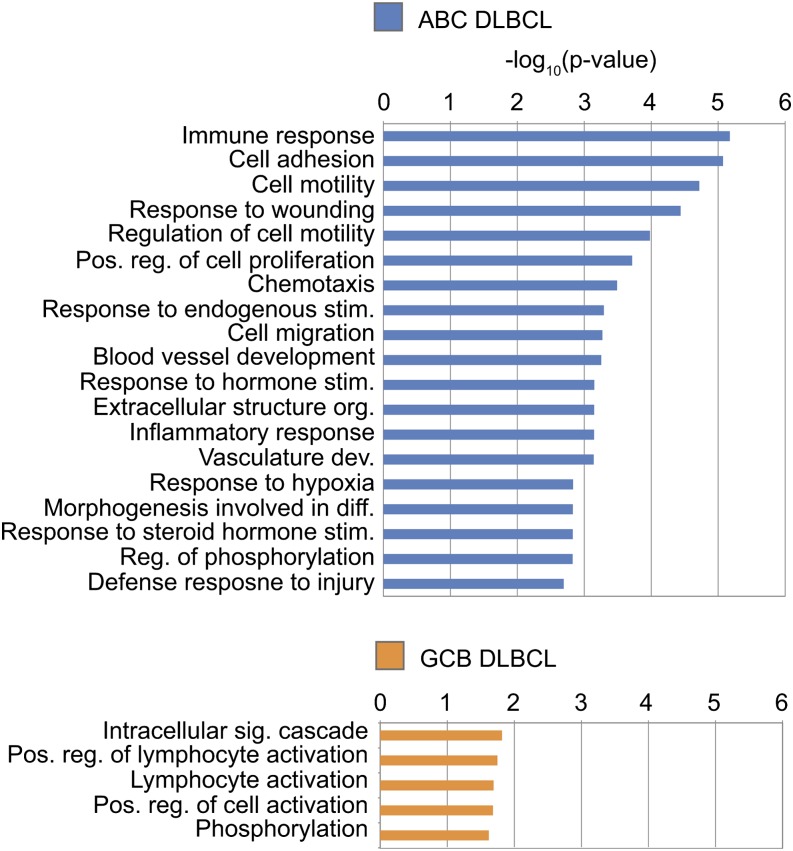
RNA-Seq gene enrichment for ontological categories. Scale is the –log_10_(*P*-value) of the binomial *P*-value, equivalent to the exponent. All enriched GO Biological Processes categories are shown, *P* < 0.05.

Genes with GO BP categories related to cell adhesion, motility, and chemotaxis are enriched in ABC DLBCL cells relative to GCB cells (Figure 7). The expression of these pathways would prime tumors to spread rapidly from their lymph nodes of origin, and may partially explain why ABC lymphomas are so much more aggressive than their GCB counterparts.

The underlying gene list includes a large number of chemokine ligands and receptors, including several with known roles in cancer (Table 3). *CCR7* shows expression limited to the ABC subtype (Figure 6B). Increased *CCR7* expression is clearly associated with metastasis to the lymph nodes in a broad variety of cancers (Schimanski *et al.* 2006; Kodama *et al.* 2007; Nakata *et al.* 2008; Pan *et al.* 2008; Arigami *et al.* 2009; Ishida *et al.* 2009). Furthermore, *CCR7* and *CXCR5* contribute to apoptosis resistance in B-cell−derived acute and chronic lymphocytic leukemia (Hu *et al.* 2004; Burkle *et al.* 2007).

**Table 3:** Selected differentially expressed genes

Gene Name	Gene Description	GCB Read Counts	ABC Read Counts	Fold Change	FDR
ABC up					
* ARNT2*	Aryl-hydrocarbon receptor nuclear translocator 2	0.00	386.14	N/A	2.25E-08
* CCR7*	Chemokine (C-C motif) receptor 7	46.30	8,252.02	178.25	4.72E-23
* CXCR5*	Chemokine (C-X-C motif) receptor 5	1,845.20	12,595.47	6.83	8.83E-07
* FGF2*	Fibroblast growth factor 2 (basic)	5.23	149.82	28.63	1.34E-02
* PDGFA*	Platelet-derived growth factor alpha polypeptide	3.25	106.66	32.83	1.59E-02
* PDGFRB*	Platelet-derived growth factor receptor, beta polypeptide	0.43	26.39	60.79	1.62E-02
* TGFA*	Transforming growth factor, alpha	4.50	132.35	29.42	2.46E-02
GCB up					
* BCL6*	B-cell CLL/lymphoma 6	15,105.68	5,124.25	0.34	1.94E-02
* PRKCA*	Protein kinase C, alpha	5,600.83	1,531.13	0.27	5.00E-04

GCB, germinal center B-cell-like; ABC, activated B-cell-like; FDR, false-discovery rate; N/A, not available; CLL, chronic lymphocytic leukemia.

Also up-regulated in the ABC subtype is transforming growth factor alpha (*TGFA*), which is a ligand for the epidermal growth factor receptor. Epidermal growth factor receptor in turn activates a signaling pathway for cell proliferation, differentiation, and development that significantly affects the progression of many human cancers, including brain, lung, breast, ovarian, prostate, and pancreatic tumors (Lu *et al.* 2001; Yang *et al.* 2011).

Genes in GO BP categories related to angiogenesis and hypoxia are also substantially enriched in the ABC cell lines (Figure 7). Angiogenesis is known to be important and prognostic in malignant lymphomas (Koster and Raemaekers 2005). The factors up-regulated in ABC DLBCL include *FGF2*, a major lymphangiogenic growth factor that promotes tumor growth and metastasis (Cao *et al.* 2008; Da *et al.* 2008). A number of growth stimulation factors and receptors are also up-regulated, such as platelet-derived growth factor alpha (*PDGFA*) and the beta subunit of the PDGF receptor (*PDGFRB*), both of which play essential roles in blood vessel development. Hypoxia induced genes include *ARNT2*, which has been reported to promote *VEGF* expression in teratomas and to protect neuronal cells from hypoxia (Maltepe *et al.* 2000). *ARNT2* expression has not been reported previously as a mechanism in non-Hodgkin lymphoma. Thus, these analyses suggest new molecules that may be involved in ABC tumorigenesis.

#### Genes up-regulated in GCB subtype:

GO analysis of the differentially expressed genes reveals that the GCB-enriched transcripts are involved in activation of lymphocytes (Figure 7). This list includes *BCL6*, a major biomarker for the GCB DLBCL subtype. Another notable GCB up-regulated gene is protein kinase C alpha (PKCα), which is encoded by *PRKCA* (Figure 6B). PKCα is a protein kinase that can act as an oncogene (Besson and Yong 2000; Huttemann *et al.* 2012). However, in response to inflammatory signaling, PKCα also can negatively regulate NF-κB−induced genes by promoting the synthesis of the inhibitor IκBα (Han *et al.* 1999). Constitutive NF-κB signaling is a prominent feature of ABC DLBCL and includes cross-talk with STAT3 via up-regulation of IL-6 and IL-10 (Motoyama *et al.* 2005). The high PKCα and low NF-κB levels in our GCB cell lines suggests that its predominant role in DLBCL may not be as an oncogene but rather as an inhibitor of the NF-κB pathway.

### STAT3 induces an aggressive genetic program in DLBCL

To better understand the downstream consequences of STAT3 binding, we next integrated the binding and gene expression results. Genes associated with STAT3 BRs were correlated with their expression levels from the RNA-Seq data. Both the STAT3 binding and mRNA expression were evaluated for statistical significance, resulting in a list of genes that shows both nearby STAT3 binding changes and an accompanying relative mRNA expression level for the two cell types.

**Figure 8:**
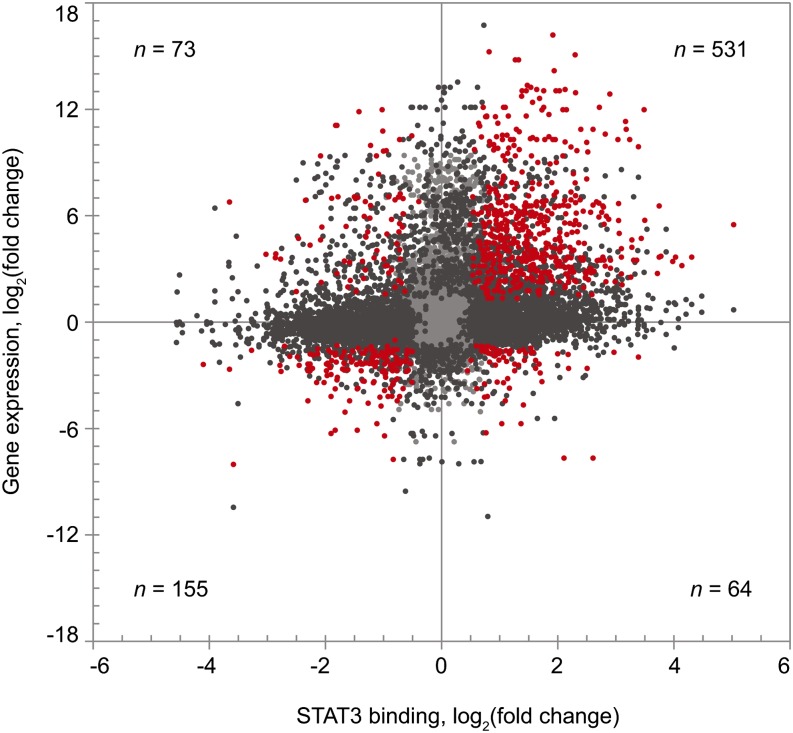
STAT3 binding correlated with gene expression change. Red dots indicate STAT3 BRs that have significant differences in binding (FDR < 0.05) between subtypes and are also associated with genes that display significant differences in expression (FDR < 0.05); dark gray dots indicate STAT3 BRs that either show significant differences in binding or whose associated genes display a significant difference in expression; and light gray dots indicate other BRs.

ChIP-Seq identified a total of 10,337 STAT3 BRs, whereas the RNA-Seq analysis compared the expression level of 20,992 genes. Using GREAT analysis, we found that more than half of these genes (60%, n = 12,631) have a STAT3 BR associated with them. Many of these genes have multiple nearby BRs; likewise, many BRs are associated with two genes because they are located in a region in which putative regulatory domains overlap. Intersecting the ChIP-Seq and RNA-Seq datasets resulted in a total of 16,341 gene/BR matches (see Table S4). Forty percent (n = 6580) showed significant differences (FDR < 0.05) in at least one category: either gene expression or STAT3 binding differed markedly between the two DLBCL subtypes. A total of 823 of these gene/BR matches, corresponding to 445 individual genes, were significantly different in both categories (Figure 8).

If STAT3 binding did not influence DLBCL gene expression, we would expect the nature of STAT3 binding near a gene to have no correlation with its expression level (Zhu *et al.* 2012). Instead, we observe that an increase in STAT3 binding is correlated with an increase in expression in most of these pairs (83%, n = 686), an association that is highly significant at *P* = 2.3 × 10^−28^ (Table 4). The strong association between binding and increased expression indicates that STAT3 binding is a significant contributor to the gene expression pattern in GCB and ABC DLBCL. The analysis indicates that STAT3 primarily functions as a transcriptional activator whereas a subset of its binding might contribute to transcriptional repression, which is consistent with its roles as previously described (Ouyang *et al.* 2009).

**Table 4:** STAT3 binding is correlated with gene expression by the χ^2^ test

STAT3 Binding	Gene Expression	Expected Frequency	Expected Counts	Observed Counts	$\frac{{\left(\text{obs.}—\text{exp}\right)}^{2}}{\text{exp}}$
ABC+	ABC+	55%•81% = 46.5%	382	531	57.19
ABC+	GCB+	55%•19% = 9.5%	78	64	2.51
GCB+	ABC+	45%•81% = 36.5%	300	155	70.08
GCB+	GCB+	45%•19% = 7.5%	61	73	1.95
			823	823	χ^2^ = 131.74
					d.f. = 3
					*P* = 2.3 × 10^−28^

STAT3, signal transducer and activator of transcription 3; TSS, transcription start site; ChIP, chromatin immunoprecipitation; FDR, false-discovery rate; ABC, activated B-cell-like; FDR, false-discovery rate; obs, observed counts; exp, expected counts.

### 

#### High STAT3 binding and gene expression in ABC:

The most enriched category of BR/gene pairs consists of genes with greater STAT3 binding in the ABC subtype as well as increased gene expression in ABC: 531 pairs correspond to 287 different genes. *STAT3* itself is among these, as are several of its known targets, including *PRDM1* and *SOCS3* (Table 5). We also confirm the findings from array CGH studies that *NFKBIZ* and the oncogene *PIM2* are elevated in ABC lymphoma (Tirado *et al.* 2012), but with the added information that STAT3 shows elevated levels of binding in the regulatory domains of these genes.

**Table 5:** Selected differentially expressed genes with associated STAT3 peaks

Gene Name	Peak Proximity to TSS	ChIP Fold Change	ChIP FDR	Expression Fold Change	Expression FDR
Increased ABC binding and expression					
* APOL2*	322	2.64	2.44E-06	36.76	6.80E-14
* BATF*	−7868	4.14	2.50E-14	18.51	5.48E-10
* BATF*	−7	3.73	4.97E-13	18.51	5.48E-10
* BCL2A1*	−7594	5.50	3.07E-19	21.56	3.25E-09
* BCL2A1*	−6328	2.28	1.25E-04	21.56	3.25E-09
* BCL2A1*	59	2.69	5.14E-06	21.56	3.25E-09
* CXCR5*	−43	3.29	4.84E-07	6.82	8.83E-07
* IL-6*	−28625	2.79	1.96E-07	10369.08	7.03E-05
* IL-10*	5883	2.45	7.48E-04	222.86	2.37E-11
* IL-10RA*	917	2.00	1.91E-03	4.96	7.26E-04
* IL-12A*	−63	2.41	9.49E-05	28133.47	2.60E-07
* IL-2RB*	27623	4.69	1.33E-19	935.76	6.45E-16
* SOCS3*	−65	9.45	2.66E-33	1251.98	2.14E-15
* STAT3*	−308	17.63	1.05E-09	9.00	1.39E-07
* PIM2*	−25	1.78	2.74E-03	12.30	2.78E-10
* PRDM1*	11587	2.17	1.16E-03	127.12	3.21E-16
* NFKBIZ*	−95	4.59	7.14E-16	8.46	5.63E-06
Increased GCB binding and expression					
* ABI3*	424	0.61	4.9E-02	0.19	2.55E-02
* MYBL1*	−199	0.66	3.48E-02	0.23	4.99E-02
* TRIB2*	−144	0.37	6.71E-06	0.07	6.81E-08
Mixed binding and expression					
* CD22*	−138	0.30	1.21E-07	0.25	8.10E-04
* CD22*	17,622	2.91	8.27E-09	0.25	8.10E-04
* CD22*	18,556	4.65	7.74E-11	0.25	8.10E-04

STAT3, signal transducer and activator of transcription 3; TSS, transcription start site; ChIP, chromatin immunoprecipitation; FDR, false-discovery rate; ABC, activated B-cell-like; FDR, false-discovery rate.

**Figure 9:**
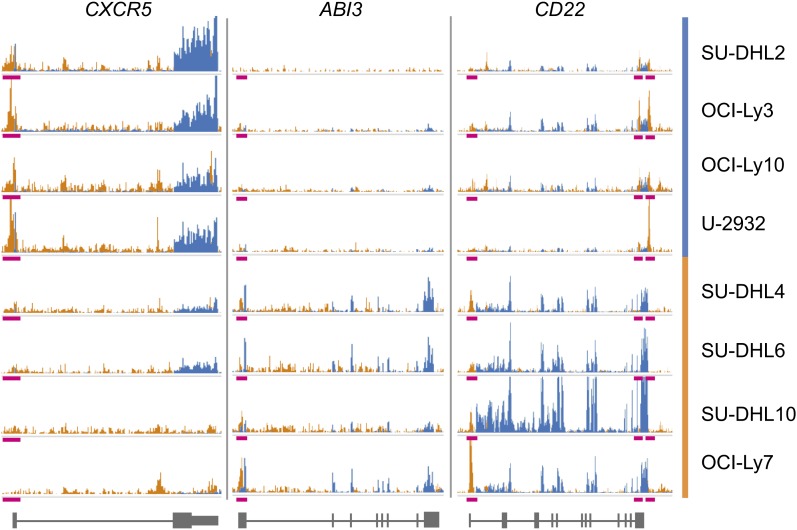
STAT3 peaks associated with gene expression changes. Examples of differentially occupied STAT3 BRs are shown. Blue tracks represent gene expression; orange tracks represent STAT3 binding; pink bars show noteworthy STAT3 BRs. *CXCR5* shows increased gene expression correlated with STAT3 binding (chr11: 118,754,259−118,754,737) in the ABC cell lines: SU-DHL2, OCI-Ly3, OCI-Ly10, and U-2932. *ABI3* shows strong increased gene expression correlated with STAT3 binding (chr17: 47,287,858−47,288,168) in the GCB cell lines: SU-DHL4, SU-DHL6, SU-DHL10, and OCI-Ly7. *CD22* shows increased gene expression in the GCB cell lines, but a mixture of STAT3 binding behavior: STAT3 has more GCB binding near the TSS (chr19: 35,819,821−35,820,060) but more ABC binding at two sites near the 3′ end (chr19: 35,837,564—35,837,836; chr19: 35,838,409−35,838,860).

STAT3 also binds near many genes that are up-regulated in ABC cells relative to GCB cells; these genes have a wide range of functions that are pertinent to oncogenesis, especially apoptosis and cellular signaling. The *CXCR5* chemokine receptor, which contributes to apoptosis resistance in B-cell—derived leukemias, is 6.83-fold more expressed in the ABC subtype (FDR = 8.83 × 10^−7^) and also has a distinct STAT3 peak 43 bp upstream of its TSS that is occupied only in ABC cell lines (Figure 9). STAT3 is also characterized as regulating the BCL2 protein family, which influences apoptosis. We specifically identify *BCL2A1* (formerly *BFL1*), which is associated with three strongly bound STAT3 BRs and demonstrates a 4.43-fold increase in expression. High levels of BCL2A1 have been proposed as a mechanism of chemoresistance in chronic lymphocytic leukemia (Olsson *et al.* 2007).

STAT3 also has a binding peak just 7 bp upstream of the TSS of *BATF*, which is highly expressed in ABC cells. BATF belongs to the AP-1/ATF superfamily of transcription factors and dimerizes with the Jun family, acting as a negative regulator (Echlin *et al.* 2000). Although BATF typically promotes growth arrest and terminal differentiation (Liao *et al.* 2011a), chronically elevated BATF levels often are seen in lymphoid tumors and promote an impaired response to apoptotic signals, apparently mediated by the BCL2 family (Logan *et al.* 2012). Because normal levels of BATF stop cell division, this pathway presents a compelling target for drug modification.

We also identify *APOL2* as a potential target of STAT3, suggesting a novel mechanism of apoptosis resistance in ABC DLBCL cells. A STAT3 BR is located 322 bp downstream of the *APOL2* TSS (fold change = 2.64, FDR = 2.44 × 10^−6^), and its mRNA expression level rises 36.76-fold in the ABC subtype (FDR = 6.80 × 10^−14^). APOL2 has recently been found to protect endothelial cells from IFNγ cytotoxic signaling (Liao *et al.* 2011b), and could play the same role in DLBCL in response to inflammatory signals.

Many interleukins and interleukin receptors show both elevated expression and STAT3 binding, including *IL-6*, *IL-10*, *IL-10RA*, and *IL-12A*. Both IL-6 and IL-10 are known to induce signaling via STAT3 upon binding their extracellular receptors; in turn, STAT3 up-regulates expression of the *IL-10* and *IL-6* genes to form an activation loop. *NFKBIZ*, which is also upregulated by STAT3, is a nuclear IκB protein that activates *IL-6* transcription and may also play a role in this loop (Motoyama *et al.* 2005). Polymorphisms in *IL-10* and its receptor subunit *IL-10RA*, also up-regulated in this study, have been implicated in the development of non-Hodgkin lymphoma generally and B-cell lymphoma specifically (Lan *et al.* 2006; Rothman *et al.* 2006), suggesting that IL-10 pathway dysregulation promotes hematopoetic tumors.

*IL-2RB* produces the beta subunit of the IL-2 cytokine receptor. Its expression level increases 935.76-fold from GCB to ABC (FDR = 6.45 × 10^−16^) and is associated with increased STAT3 binding at a distal site about 27 kb downstream (FDR = 1.33 × 10^−19^). *IL-2RA*, which encodes the other half of the IL-2 receptor complex, is also up-regulated in the ABC subtype, although it lacks a proximal STAT3 peak (fold change = 8.00, FDR = 1.95 × 10^−3^). These findings point to a potential therapy target for refractory cases of ABC DLBCL. The existing anticancer treatment denileukin diftitox (trade name Ontak) is an engineered protein combining IL-2 and diphtheria toxin, which binds to IL-2 receptors and introduces the diphtheria toxin into cells, killing them (Lansigan *et al.* 2010). Ontak has been specifically approved by the Food and Drug Administration for the treatment of cutaneous T-cell lymphoma but should be effective in treating any lymphoma that displays an elevated number of IL-2 receptors. Furthermore, increased expression of the *IL-2RB* gene is known to increase the efficacy of Ontak treatment (Shao *et al.* 2002).

#### High STAT3 binding and gene expression in GCB:

A total of 95 genes have greater STAT3 binding and gene expression in GCB cells. Among these is *MYBL1*, a known biomarker for the GCB subtype (Blenk *et al.* 2007), which is overexpressed as expected in GCB cell lines and also has a statistically significant STAT3 peak 199 bp upstream of its TSS. Also included in this list are two tumor suppressor genes: *ABI3* and *TRIB2*. In thyroid tumors, *ABI3* expression blocks tumor progression on many fronts, including reducing anchorage-independent growth and *in vivo* tumor formation while increasing cellular senescence (Latini *et al.* 2011). In the GCB subtype, it demonstrates greater expression that corresponds to the presence of STAT3 424 bp downstream of its TSS (Figure 9). TRIB2 inhibits JNK, which ultimately reduces the antiapoptotic activity of BCL2 (Gilby *et al.* 2010). The loss of *TRIB2* expression in ABC DLBCL is particularly problematic, as STAT3 increases the expression of BCL2 and its family members.

#### Anticorrelated STAT3 binding and gene expression:

Seventeen percent (n = 137) of BR/gene pairs have strong STAT3 binding in one subtype but strong gene expression in the other. These 107 genes may be regulated by other factors, or STAT3 may serve as a repressor at these sites, potentially by blocking the binding of other factors. Fully 40% (n = 43) of these potential anticorrelated genes occur due to a mixture of STAT3 binding behavior: the gene is associated with many BRs, some of which are more occupied by STAT3 in ABC while others show stronger binding GCB.

We observe greater *CD22* expression in GCB DLBCL, associated with a STAT3 peak 138 bp upstream of its TSS. However, *CD22* also has two STAT3 BRs near its 3′ end that are more strongly bound in ABC (Figure 9). This finding suggests that STAT3 binding at the *CD22* promoter drives transcription, whereas binding at the two downstream sites suppresses it. CD22 is a cell surface antigen expressed in a wide variety of non-Hodgkin lymphomas, and anti-CD22 monoclonal antibody treatments currently are being developed (Lu *et al.* 2006; Li *et al.* 2013). However, they may be less successful in treating ABC DLBCL due to lowered expression of this antigen.

*BCL6* is the most extreme example of this mixed binding phenomenon. It is the primary biomarker for the GCB subtype of DLBCL and is known to be regulated by STAT3. We found that expression of *BCL6* is extremely high in GCB lines, three times the level found in our ABC cell lines. However, its regulation is complex: there are 24 STAT3 BRs within the putative regulatory domain of *BCL6*, which includes a large upstream gene desert that contains considerable STAT3 binding activity. Seven of these BRs show a significant binding differential between the subtypes. Two of them are more strongly bound in ABC, but the other five are more strongly bound by STAT3 in GCB (see Figure S3 and Table S3). This complexity suggests that the overall sum of STAT3 binding contributes to the final regulation of BCL6.

**Figure 10:**
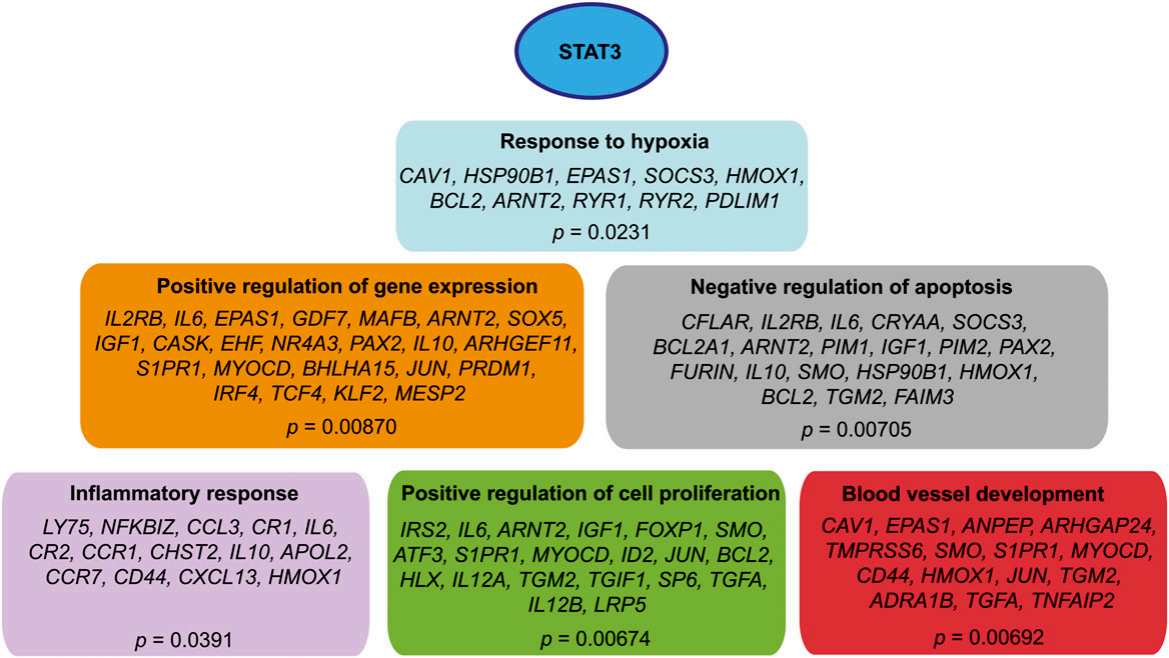
Downstream pathways and genes induced by STAT3 in ABC DLBCL. Examples of the top GO Biological Processes categories enriched among genes with increased expression and increased STAT3 binding in ABC DLBCL. All *P*-values are corrected for multiple comparisons using the Benjamini-Hochberg procedure.

The combination of whole-genome profiling approaches shows differential STAT3 binding sites near almost a third of all genes that differ in expression between GCB and ABC DLBCL, leaving little doubt that STAT3 is a regulator of the aggressive clinical phenotype and drug resistance displayed by the ABC subtype. The STAT3 binding and expression results suggest possible models by which STAT3 regulates biological pathways to promote aggressive oncogenic behavior in ABC DLBCL. Ontological analysis of the genes up-regulated in ABC DLBCL with increased nearby STAT3 binding indicates that STAT3 promotes cell migration, blood vessel development, and the inflammatory response (Figure 10). Furthermore, it ensures its own continued high expression by up-regulating its pathway partners and upstream signaling molecules, such as IL-6 and IL-10, and promotes cross-talk with the nuclear factor-κB pathway. Most critically, STAT3 strongly up-regulates many mechanisms of apoptosis resistance. These effects synergize and likely lead to increased cell proliferation and distributions of cells, which are hallmarks of aggressive lymphomas. These results provide novel insights into the genetics of oncogenesis in the ABC form of diffuse large B-cell lymphoma and identify a substantial number of plausible downstream mechanisms for the treatment-resistant phenotype of this subtype.

## Supplementary Material

Supporting Information
