# Supplementary material for: STAT3 Targets Suggest Mechanisms of Aggressive Tumorigenesis in Diffuse Large B-Cell Lymphoma
Source: G3 (Bethesda). 2013 Oct 18;3(12):2173–85. doi: 10.1534/g3.113.007674 (PMC3852380; doi:10.1534/g3.113.007674)
Supplement: Supporting Information [file supp_3_12_2173__index.html]

STAT3 Targets Suggest Mechanisms of Aggressive Tumorigenesis in Diffuse Large B-Cell Lymphoma — Supporting Information 

# STAT3 Targets Suggest Mechanisms of Aggressive Tumorigenesis in Diffuse Large B-Cell Lymphoma

## Supporting Information for Hardee *et al.*, 2013

**Files in this Data Supplement:**

- Supporting Information - Figures S1-S3, Files S1-S2, and Tables S1-S4 (PDF, 2 MB)
- Figure S1 - STAT3 immunoblotting and immunoprecipitation with sc-482. (PDF, 343 KB)
- Figure S2 - DLBCL STAT3 binding regions shared with other cell lines. (PDF, 319 KB)
- Figure S3 - The *BCL6* gene locus with STAT3 ChIP-seq peaks. (PDF, 361 KB)
- File S1 - Extended Materials and Methods (PDF, 355 KB)
- Table S1 - ChIP-Seq sequencing statistics for all replicates. (PDF, 320 KB)
- Table S2 - RNA-Seq sequencing statistics for all replicates. (PDF, 314 KB)
- Table S3 - BCL6-associated STAT3 binding regions. (PDF, 333 KB)
- Table S4 - BRs by binding. (PDF, 1 MB)
- File S2 - Data tables for ChIP-sequencing and RNA-sequencing analysis (.xls, 18 MB)
