# Supplementary material for: STAT3 Targets Suggest Mechanisms of Aggressive Tumorigenesis in Diffuse Large B-Cell Lymphoma
Source: G3 (Bethesda). 2013 Oct 18;3(12):2173–85. doi: 10.1534/g3.113.007674 (PMC3852380; doi:10.1534/g3.113.007674)
Supplement: Supporting Information [file supp_g3.113.007674_007674SI.pdf]

G3: Genes | Genomes | Genetics

STAT3 Targets Suggest Mechanisms of Aggressive Tumorigenesis in Diffuse Large B Cell Lymphoma

Jennifer Hardee[*,§], Zhengqing Ouyang[*,1,2,3], Yuping Zhang[*,4] , Anshul Kundaje[*,†], Philippe Lacroute[*], Michael Snyder[*,5]

*Department of Genetics, Stanford University School of Medicine, Stanford, CA 94305; §Department of Molecular, Cellular, and Developmental Biology, Yale University, New Haven, CT 06520; and †Department of Computer Science, Stanford University School of Engineering, Stanford, CA 94305

[1]The Jackson Laboratory for Genomic Medicine, Farmington, CT 06030

[2]Department of Biomedical Engineering, University of Connecticut, Storrs, CT 06269

[3]Department of Genetics and Developmental Biology, University of Connecticut Health Center, Farmington, CT 06030

[4]Department of Biostatistics, Yale School of Public Health, Yale University, New Haven, CT 06520

[5]Corresponding author: Department of Genetics, Stanford University School of Medicine, Stanford, CA 94305. Email: mpsnyder@stanford.edu

DOI: 10.1534/g3.113.007674

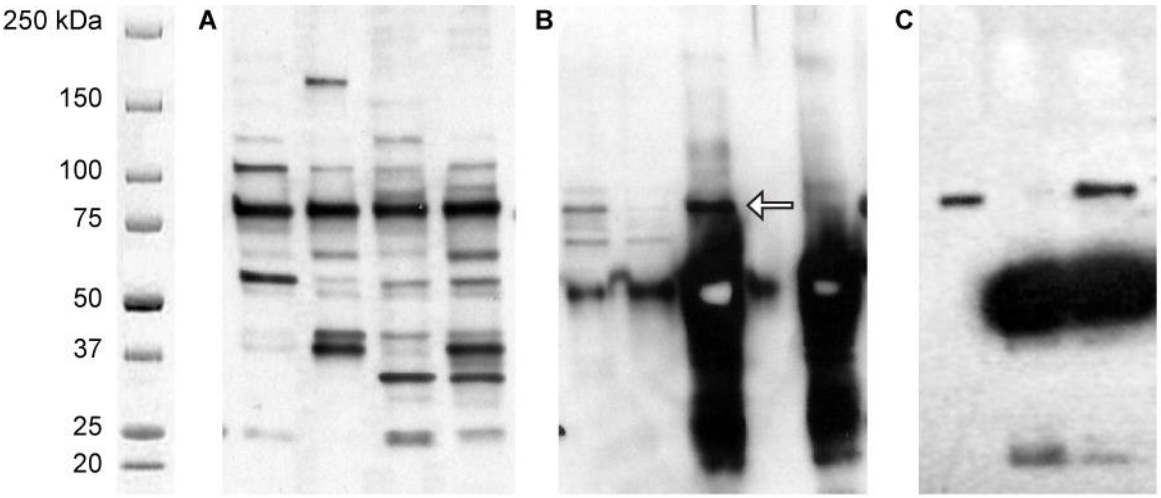

Figure S1 STAT3 immunoblotting and immunoprecipitation with sc-482. Western blot and IPs show a band consistent with expected size (88 kDa) of STAT3. (A) Western blot using antibody sc-482 versus nuclear lysates. Lanes contain (from left to right) lysate from K562 cells, GM12878 cells, HeLa S3 cells, and HepG2 cells. (B) IP of STAT3 using sc-482 in HeLa S3 cells. *Lane 1:* input nuclear lysate; *lane 2:* unbound material from IP with sc-482; *lane 3:* material IP'd with sc-482; *lane 4:* material IP'd using control rabbit IgG. Arrow indicates the band of interest. (C) IP of STAT3 using sc-482 in K562 cells. *Lane 1:* input nuclear lysate; *lane 2:* material IP'd using control rabbit IgG; *lane 3:* material IP'd with sc-482.

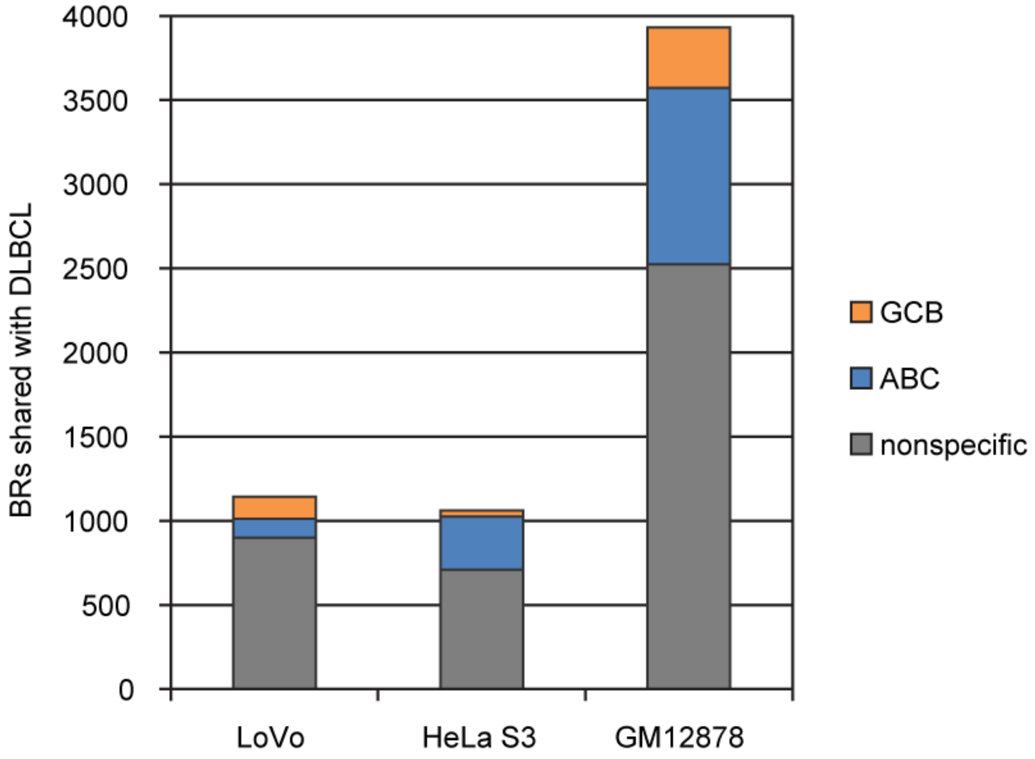

Figure S2 DLBCL STAT3 binding regions shared with other cell lines. 10,337 DLBCL high-confidence STAT3 binding regions (BRs) were compared to STAT3 BR lists generated by other studies in the cell lines LoVo, HeLa S3, and GM12878. Orange bars represent BRs that are preferentially bound by STAT3 in the GCB subtype of DLBCL; blue bars represent BRs preferentially bound in the ABC subtype of DLBLC; gray bars represent BRs that show no DLBCL subtype binding bias.

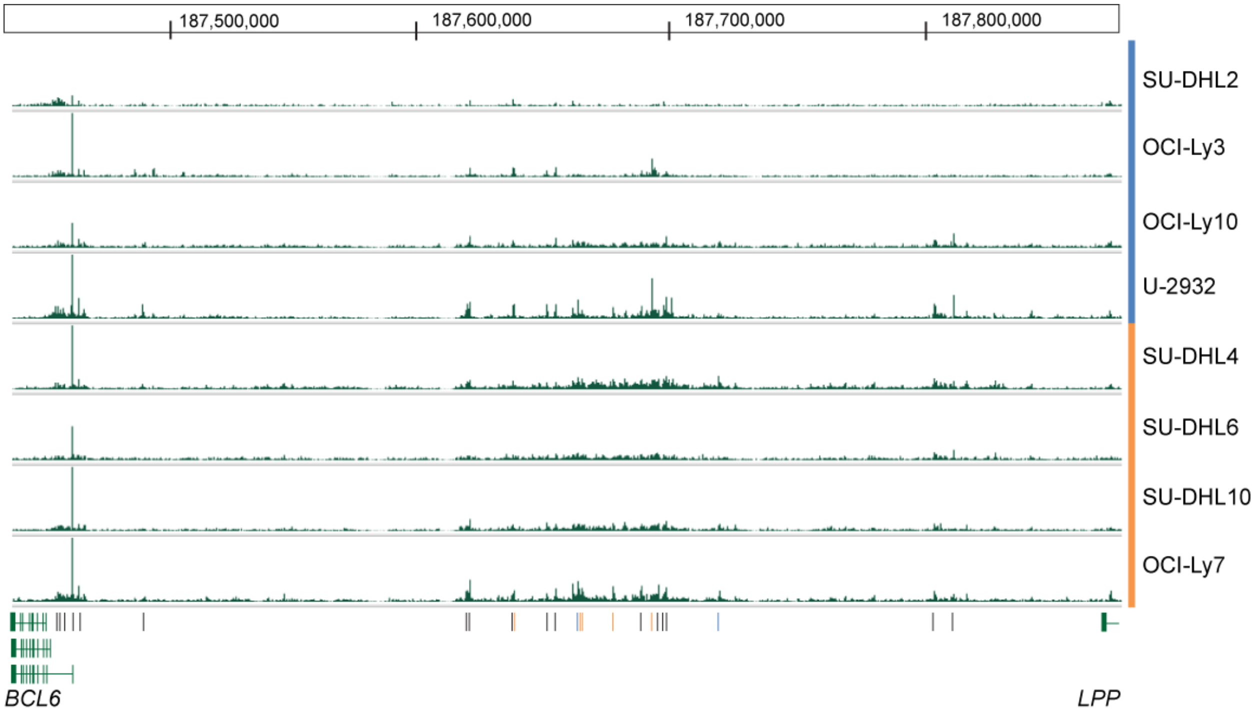

Figure S3 The *BCL6* gene locus with STAT3 ChIP-seq peaks. All 24 STAT3 BRs are shown within the upstream putative regulatory region of *BCL6* (chr3: 187,436,000-187,876,700). Orange bars represent loci with enriched STAT3 binding in the GCB subtype at FDR < 0.05 (*n* = 5); blue bars represent loci with enriched STAT3 binding in the ABC subtype (*n* = 2); dark gray bars represent peaks with no statistically significant difference in binding between the two subtypes (*n* = 17). *BL6* is encoded on the minus strand at left, and *LPP* encoded on the plus strand at right (only TSS shown).

File S1

EXTENDED MATERIALS AND METHODS

Cell culture: OCI-Ly7, SU-DHL4, SU-DHL6, SU-DHL10, and U-2932 cell lines were purchased from Deutsche Sammlung von Mikroorganismen und Zellkulturen (DSMZ). OCI-Ly3, OCI-Ly10, and SU-DHL2 cells were generously donated by Dr. Louis Staudt's laboratory at the NIH National Cancer Institute. Cell lines OCI-Ly7, SU-DHL2, SU-DHL4, SU-DHL6, SU-DHL10, and U-2932 were cultured in suspension in RPMI 1640 (Gibco-Invitrogen) containing L-glutamine and HEPES, supplemented with heat-inactivated fetal bovine serum (15%) and antibiotics (Penicillin Streptomycin, Gibco-Invitrogen), at 37° and 5% CO_2. Cell lines OCI-Ly3 and OCI-Ly10 were cultured in suspension in IMDM (Gibco-Invitrogen) containing L-glutamine and HEPES, supplemented with heat-inactivated fetal bovine serum (15%), beta-mercaptoethanol (55 μM, Gibco-Invitrogen), and antibiotics (Penicillin Streptomycin, Gibco-Invitrogen), at 37° and 5% CO_2.

Western blotting: Cells were washed with PBS, then lysed for 30 minutes on ice in cold RIPA buffer containing 1 mM DTT, 0.5 mM PMSF, protease inhibitors (Complete Protease Inhibitor Cocktail Tablets, Roche), and phosphatase inhibitors (Phosphatase Inhibitor Cocktail 2 and 3, Sigma Aldrich). Cell debris was removed by centrifugation. Protein concentration of the resulting whole cell lysate was measured by Bradford assay (Precision Assay solution, BioRad).

Whole cell lysate was mixed with an equal volume of 2x Laemmli SDS loading buffer and incubated at 70° for 15 minutes to denature the proteins. Then, equal amounts of protein were loaded into each lane of a 4-20% SDS-PAGE gel (Mini-PROTEAN TGX Precast Gel, BioRad), which was run at 100 volts for 1 hour (running buffer: 25 mM Tris, 192 mM glycine, 0.1% SDS). The gel was transferred to a PVDF membrane (transfer buffer: 25 mM Tris, 192 mM glycine, 20% methanol, 0.1% SDS) at 250 mAmps for 1 hour. The membrane was then sliced horizontally at the 50 kDa marker to separate the upper portion containing STAT3 protein (88 kDa) from that containing the GAPDH loading control (40 kDa).

PVDF membranes were blocked in 5% BSA at 4° overnight on an orbital shaker. Blots were then probed using the SNAP i.d. 2.0 system (Millipore). Primary antibodies were diluted in 0.5% BSA in PBS-T (0.1% Tween): anti-STAT3 antibody sc-482X and anti-pSTAT3-Y705 antibody sc-8059X (Santa Cruz Biotechnology) were diluted 1:200; anti-GAPDH antibody ab8245 (Abcam) was diluted 1:5000. The membranes were incubated with primary antibody for 20 minutes at room temperature, then washed three times with PBS-T. HRP-conjugated secondary antibodies were diluted in 0.5% BSA in PBS-T: goat anti-rabbit was diluted

1:10,000; rabbit anti-mouse was diluted 1:8000. Blots were incubated with secondary antibody for 10 minutes at room temperature, then washed three times with PBS-T.

Blots were treated with enhanced chemiluminescent (ECL) substrate in a ratio of 70:30 pico to femto (SuperSignal West Chemiluminescent Substrate, Pierce) and visualized using BioMax Maximum Resolution film (Carestream). Fold change was measured by densitometric quanitification using NIH ImageJ software, normalized to the GAPDH loading control (Schneider *et al.* 2012).

Chromatin immunoprecipitation (ChIP): Cells were suspended in their growing media at $1x10^6$ per mL and crosslinked with formaldehyde at a final concentration of 1% for 10 minutes at room temperature, followed by the addition of glycine in PBS at a final concentration of 125 mM for 5 minutes. Cells were collected by centrifugation, washed twice in PBS, then snap frozen in liquid nitrogen and stored at -80° in aliquots of $1x10^8$ cells.

Frozen aliquots were thawed in 5 mL of cold PBS plus protease inhibitors (Complete Protease Inhibitor Cocktail Tablets, Roche) at 4° for 30 minutes with rotation. Cells were collected by centrifugation, then swollen on ice for 10 minutes in a hypotonic buffer (20 mM HEPES, pH 7.9; 10 mM KCl; 1 mM EDTA, pH 8.0; 10% glycerol; 1 mM DTT; 0.5 mM PMSF; and protease inhibitors). Cells were broken by dounce homogenization, and the nuclei were pelleted by mild centrifugation. The nuclei were washed one with hypotonic buffer, then lysed in RIPA buffer (10 mM Tris-Cl, pH 8.0; 140 mM NaCl; 0.025% sodium azide; 1% Triton X-100; 0.1% SDS; 1% deoxycholic acid; 0.5 mM PMSF; 1 mM DTT; 0.1 mM sodium orthovanadate; and protease inhibitors) on ice for at least 30 minutes with repeated vortexing. The nuclear lysate was sonicated with a Branson 250 Sonifier to shear the chromatin (output 20%, 100% duty cycle, 12 20-second pulses each followed by a two minute rest on ice). The samples were clarified by centrifugation at 14,000 rpm at 4° for 15 minutes. 200 μL was removed from each sample to act as a pre-immunoprecipitation genomic DNA control ("input" control) and stored at 4°. Then STAT3-DNA complexes were immunoprecipitated with an anti-STAT3 C-terminal antibody (sc-482X, Santa Cruz Biotechnology) for 16 hours at 4° on a multimix rotator (VWR International).

Each immunoprecipitated sample was incubated with protein A-agarose beads (Upstate Biotechnology) for 2 hours at 4° on a multimix rotator, followed by three washes with RIPA buffer and one wash with PBS. The antibody-STAT3-DNA complexes were eluted from the beads by addition of 1% SDS in TE (10 mM Tris-Cl, pH 7.6; 1 mM EDTA, pH 8.0) and incubation at 65° for 16 minutes with repeated vortexing. The beads were removed by centrifugation and the supernatant reserved. The beads were

resuspended in 0.67% SDS in TE and incubated at 65° for a further 16 minutes with repeated vortexing. The beads were removed by centrifugation and the supernatant combined with that from the 1% SDS elution. The input DNA control aliquots were removed from 4° storage and 300 uL of 1% SDS in TE was added. Both the input controls and the bead elutions were incubated at 65° overnight to reverse the crosslinking. To purify the DNA, RNase A was added to a final concentration of 200 μg/mL, and the samples were incubated at 37° for 30 minutes. Proteinase K was added to a final concentration of 200 μg/mL, and the samples were incubated at 45° for 30 minutes. This was followed by an ethanol precipitation and DNA purification column (Qiagen) to recover the DNA.

To test the efficacy of the IP, ChIP'd DNA was amplified using primers against an empirical STAT3 binding site in its own promoter region, coordinates chr17: 40,540,721-40,540,905 (build hg19). An annealing temperature of 60° and 30 PCR cycles were used. Primer sequence:

Left primer: 5'-GCTGGCTGTTCCGACAGT-3'

Right primer: 5'-CAGGAGGGAGCTGTATCAGG-3'

mRNA isolation for RNA-Seq: An aliquot of $4x10^6$ cells were washed once with PBS and lysed. The lysate was incubated with magnetic poly-dT beads (Dynabeads mRNA DIRECT Kit, Invitrogen) to directly isolate poly-A RNA using the "standard" wash volumes. Beads were serially washed in Washing Buffer A (10 mM Tris-HCl, pH 7.5; 0.15 M LiCl; 1 mM EDTA; 0.1% LiDS) and Washing Buffer B (10 mM Tris-HCl, pH 7.5; 0.15 M LiCl; 1 mM EDTA; 10 mM Tris-HCl). The mRNA was eluted from the beads using 10 mM Tris-HCl, pH 7.5.

To reduce contamination by ribosomal RNA, the purified poly-A mRNA was then bound to the same beads, washed, and eluted a second time. After the second purification, it was fragmented to approximately 300 bp in length (RNA Fragmentation Reagents, Ambion), treated with DNase I, and purified via Qiagen's RNeasy MinElute Cleanup kit.

As the kit did not supply enough Buffer B to accommodate all optional additional wash steps, extra buffer was prepared based on the ingredients listed in the kit instructions. There was no difference between samples prepared using the pre-made kit buffer versus those prepared using homemade buffer.

cDNA synthesis: Double-stranded cDNA was synthesized from the isolated mRNA using the Invitrogen SuperScript Double-Stranded cDNA Labeling Kit. mRNA was incubated with random hexamer primers at 70° for 10 minutes, then incubated at 45° for 1 hour with SuperScript II RT to synthesize the first strand of cDNA. RNase H was added to degrade the existing RNA, and then the second strand of cDNA was synthesized in the presence of DNA Polymerase I and DNA Ligase at 16° for 2 hours. The final product was purified using Qiagen's QIAquick PCR Purification kit.

DNA library preparation for sequencing: Sheared DNA was converted to blunt-ended fragments (End-It Repair Kit, Epicenter-Illumina). Then a single overhanging dATP was added via a Klenow fragment, and Illumina adapters were ligated onto the ends. Adapter sequence:

Left primer (1.1): 5'-AATGATACGGCGACCACCGAGATCTACACTCTTTCCCTACACGAC-3'

Right primer (2.1): 5'-CAAGCAGAAGAGGGCATACGAGCTCTTCCGATCT-3'

To remove excess non-ligated adapters, the DNA was gel purified using a 2% agarose gel with a size selection from 150-300 base pairs. Then the DNA was PCR amplified using Illumina primers and Phusion high-fidelity DNA polymerase master mix (New England BioLabs) under the following reaction conditions: 98° for 30 seconds; 15 cycles of 98° for 10 seconds, 65° for 30 seconds, 72° for 30 seconds; and 72° for 5 minutes. The PCR product was gel purified again using a 2% agarose gel with a size selection from 150-300 base pairs.

ChIP-Seq peak calling & replicate analysis: We used the Irreproducible Discovery Rate (IDR) framework (Li *et al.* 2011) with the SPP peak caller (Kharchenko *et al.* 2008) in order to identify high confidence and reliable regions of enrichments (peaks) in the ChIP-seq datasets. Specifically, we followed the ENCODE uniform processing pipeline as outlined at http://anshul.kundaje.net/projects/idr. Briefly, the SPP peak caller was used with a relaxed peak calling threshold (FDR = 0.9) to obtain a large number of peaks (maximum of 300,000) that span true signal as well as noise (false identifications). Peaks in each replicate were ranked based on their SPP signal score. The IDR method analyzes ranked peak calls for a pair of replicates, and considers peaks that are present in both replicates to belong to one of two populations: a reproducible signal group or an irreproducible noise group. The method adaptively learns the degree of peak-rank consistency in the signal component and the proportion of peaks belonging to each component. The model can then be used to infer an IDR score for every peak that is

found in both replicates. The IDR score of a peak represents the expected probability that the peak belongs to the noise component, and is based on its ranks in the two replicates. Hence, low IDR scores represent high-confidence peaks. The number of peaks with IDR scores better than 0.02 (2%) was used as the cross-replicate peak rank threshold. If a dataset had more than two replicates, all pairs of replicates were analyzed using the IDR method. The maximum peak rank threshold across all pairwise analyses was used as the final cross-replicate peak rank threshold. Any thresholds based on reproducibility of peak calling between biological replicates are bounded by the quality and enrichment of the worst replicate.

In cases where a cell line dataset had at least one replicate that was significantly worse in data quality than another replicate, a rescue pipeline was used in order to balance data quality between the set of replicates. Mapped reads were pooled across all replicates of a dataset, and then randomly sampled (without replacement) to generate two pseudo-replicates with equal numbers of reads. This sampling strategy tends to transfer signal from stronger replicates to the weaker replicates, thereby balancing cross-replicate data quality and sequencing depth. SPP was used to with relaxed thresholds (FDR 0.9) to call peaks on these pseudo-replicates. The peaks were ranked by SPP signal score. The two sets of ranked peaks from the pseudo-replicates were then compared using IDR and the number of peaks passing an IDR threshold of 0.0025 (0.25%) was used as a rescue peak rank threshold. Ultimately, for each dataset, the best of the cross-replicate and rescue peak rank thresholds were used to obtain an optimal peak rank threshold. Finally, mapped reads from all replicate datasets were pooled and SPP was once again used to call peaks on the pooled data with a relaxed FDR of 0.9. These peaks on the pooled data were ranked by the SPP signal-score. The optimal peak rank threshold (N) was then used on select the top N peaks ranked set of pooled-data peaks.

Once the characteristic high confidence STAT3 binding sites were identified for each cell line with IDR, all the peak lists were combined. Any overlapping or abutting peaks were merged into broader "binding regions" (BRs) to facilitate comparison between cell lines (as in KASOWSKI *et al.* 2010). Binding regions that occurred in only one cell line were eliminated from further analysis. The ChIP-Seq data for each line was then rescored to determine how many fragments mapped to each BR.

Next, the ChIP-Seq datasets were normalized using DESeq (ANDERS and HUBER 2010). The effective library size of each sample was estimated based on the pooled count data. First, a reference sample was defined in which the reference count of each BR is its geometric mean over all samples. Second, for each sample, a vector was calculated as the ratios of the read counts over the reference counts for all the BRs. Third, the median of these ratios across all the BRs was defined as the "size factor" of each sample. Lastly, each sample was normalized by dividing the real counts by its size factor.

We then compared the four ABC cell lines to the four GCB cell lines to identity BRs that were more strongly occupied in one subtype of DLBCL. We compared 24 GCB replicates versus 11 ABC replicates. To assess the significance levels of differential

STAT3 binding in ChIP-Seq data, we applied the negative binomial model in DESeq. The dispersion parameter in the model is estimated from the data by examining the relationship between the mean and variance of read counts across all the BRs. To contrast two conditions, we used the parameterized negative binomial model for each gene and obtained the p-values. To correct for multiple comparison, we adjusted the p-values with the Benjamini-Hochberg procedure which controls false discovery rate (Benjamini and Hochberg 1995).

RNA-Seq replicate analysis: Single-ended 36 bp reads were generated and the raw reads were mapped to the human genome (build hg19) using Bowtie (Langmead *et al.* 2009). The top two best mappings were kept, tolerating two or fewer mismatched bases per read. Then a custom script was used to select the better of these two unique mappings. We also filtered reads that mapped to human ribosomal RNAs. The positions of the filtered reads were intersected with the coordinates of 26,322 annotated RefSeq genes, and the read count for each gene was generated. The RNA-Seq datasets were then normalized similarly to the ChIP-Seq data using DESeq (Anders and Huber 2010).

Two biological replicates were performed for each line, and their normalized read counts were compared on a gene by gene basis. We then compared the four ABC cell lines to the four GCB cell lines to identity genes that were more highly expressed in one subtype of DLBCL. Because of the high degree of similarity between RNA-Seq replicates, we chose to collapse the replicates by summing the read counts of two replicates for each cell line, then compare the four GCB lines versus four ABC lines. To assess the significance levels of differential gene expression in RNA-Seq data, we applied the negative binomial model in DESeq (Anders and Huber 2010). The dispersion parameter in the model is estimated from the data by examining the relationship between the mean and variance of read counts across all the genes. To contrast two conditions, we used the parameterized negative binomial model for each gene and obtained the p-values. To correct for multiple comparison, we adjusted the p-values with the Benjamini-Hochberg procedure which controls false discovery rate (Benjamini and Hochberg 1995).

ChIP-Seq gene associations: ChIP-Seq binding regions (BRs) were associated with genes that they might regulate via the Genomic Regions Enrichment of Annotations Tool (GREAT, McLean *et al.* 2010). To determine whether a BR and a given gene are linked, GREAT first determines a putative regulatory domain for every gene, which consists of a basal domain plus an extension. The basal regulatory domain (BRD) consists of the region spanning 5 kb upstream to 1 kb downstream of the gene's

transcription start site (TSS), regardless of overlapping nearby genes. The gene's extended regulatory domain (ERD) is then calculated by elongating the BRD both upstream and downstream for 1000 kb or until reaching another gene's BRD, whichever occurs first.

Once this set of extended regulatory domains is established, GREAT associates the list of ChIP-Seq BRs with all of the genes whose regulatory domains they overlap. In many cases, BRs fall in regions where two regulatory domains overlap; consequently, GREAT associates these BRs with both genes. For purposes of downstream comparison, we treated these multiple associations as separate database entries, to allow the greatest sensitivity in detecting associations with gene expression data. We then compared this list of genes associated with significant changes in STAT3 binding to the list of genes with significant expression changes using the online tool Galaxy (Giardine *et al.* 2005; Blankenberg *et al.* 2010; Goecks *et al.* 2010).

REFERENCES FOR EXTENDED METHODS

Anders, S., and W. Huber, 2010 Differential expression analysis for sequence count data. Genome Biol 11**:** R106.

Benjamini, Y., and Y. Hochberg, 1995 Controlling the false discovery rate: a practical and powerful approach to multiple testing. Journal of the Royal Statistical Society Series B (Methodological) 57**:** 289-300.

Blankenberg, D., G. Von Kuster, N. Coraor, G. Ananda, R. Lazarus *et al.*, 2010 Galaxy: a web-based genome analysis tool for experimentalists. Curr Protoc Mol Biol Chapter 19**:** Unit 19 10 11-21.

Giardine, B., C. Riemer, R. C. Hardison, R. Burhans, L. Elnitski *et al.*, 2005 Galaxy: a platform for interactive large-scale genome analysis. Genome Res 15**:** 1451-1455.

Goecks, J., A. Nekrutenko and J. Taylor, 2010 Galaxy: a comprehensive approach for supporting accessible, reproducible, and transparent computational research in the life sciences. Genome Biol 11**:** R86.

Kasowski, M., F. Grubert, C. Heffelfinger, M. Hariharan, A. Asabere *et al.*, 2010 Variation in transcription factor binding among humans. Science 328**:** 232-235.

Kharchenko, P. V., M. Y. Tolstorukov and P. J. Park, 2008 Design and analysis of ChIP-seq experiments for DNA-binding proteins. Nat Biotechnol 26**:** 1351-1359.

Langmead, B., C. Trapnell, M. Pop and S. L. Salzberg, 2009 Ultrafast and memory-efficient alignment of short DNA sequences to the human genome. Genome Biol 10**:** R25.

Li, Q., J. B. Brown, H. Huang and P. J. Bickel, 2011 Measuring reproducibility of high-throughput experiments. Annals of Applied Statistics 5**:** 1752-1779.

McLean, C. Y., D. Bristor, M. Hiller, S. L. Clarke, B. T. Schaar *et al.*, 2010 GREAT improves functional interpretation of cis-regulatory regions. Nat Biotechnol 28**:** 495-501.

Schneider, C. A., W. S. Rasband and K. W. Eliceiri, 2012 NIH Image to ImageJ: 25 years of image analysis. Nat Methods 9**:** 671-675.

File S2

Data tables for ChIP-sequencing and RNA-sequencing analysis

File S1 is available for download at http://www.g3journal.org/lookup/suppl/doi:10.1534/g3.113.007674/-/DC1.

Table S1 ChIP-Seq sequencing statistics for all replicates

Cell line	Replicate	Total reads	Mapped reads	Percent mapping
**ABC subtype**				
SU-DHL2	1	13,512,260	12,288,255	90.94%
	2	13,362,953	12,424,630	92.98%
OCI-Ly3	1	13,064,810	10,257,726	78.51%
	2	18,089,134	16,571,735	91.61%
	3	18,897,921	17,974,794	95.12%
	4	24,373,222	22,970,681	94.25%
OCI-Ly10	1	37,395,608	33,290,027	89.02%
	2	44,875,156	37,749,797	84.12%
U-2932	1	16,507,416	15,223,231	92.22%
	2	17,272,908	16,148,051	93.49%
	3	18,302,385	14,800,735	80.87%
**GCB subtype**				
SU-DHL4	1	20,378,155	17,826,440	87.48%
	2	20,023,227	18,458,102	92.18%
	3	19,006,085	17,222,037	90.61%
	4	6,186,894	4,950,382	80.01%
	5	26,300,058	21,093,459	80.20%
	6	26,764,265	23,361,452	87.29%
	7	12,400,324	10,817,576	87.24%
	8	22,072,217	20,667,096	93.63%
	9	1,217,646	597,943	49.11%
SU-DHL6	1	10,546,526	8,980,307	85.15%
	2	18,604,899	14,101,371	75.79%
	3	31,175,126	28,350,143	90.94%
	4	21,166,960	19,002,685	89.78%
	5	20,685,171	18,677,393	90.29%
	6	16,917,103	15,262,894	90.22%
	7	16,835,815	15,109,722	89.75%
	8	18,277,246	15,785,997	86.37%
SU-DHL10	1	28,470,759	26,816,509	94.19%
	2	27,785,404	26,145,200	94.10%
	3	16,827,217	15,260,782	90.69%
	4	22,196,262	21,051,264	94.84%
OCI-Ly7	1	25,139,778	23,903,936	95.08%
	2	21,659,272	20,741,470	95.76%
	3	17,519,050	16,590,574	94.70%

Table S2 RNA-Seq sequencing statistics for all replicates

Cell line	Replicate	Total reads	Mapped reads	Percent mapping
**ABC subtype**				
SU-DHL2	1	28,204,354	24,615,267	87.27%
	2	25,329,137	21,547,373	85.07%
OCI-Ly3	1	30,939,496	24,755,240	80.01%
	2	28,502,235	21,678,611	76.06%
OCI-Ly10	1	32,056,465	28,628,279	89.31%
	2	29,305,171	26,100,549	89.06%
U-2932	1	17,600,699	14,810,651	84.15%
	2	15,868,465	13,051,886	82.25%
**GCB subtype**				
SU-DHL4	1	18,071,332	15,607,255	86.36%
	2	18,930,836	16,772,051	88.60%
SU-DHL6	1	29,374,231	24,974,330	85.02%
	2	33,740,415	28,752,594	85.22%
SU-DHL10	1	27,520,478	24,673,323	89.65%
	2	28,931,874	25,673,326	88.74%
OCI-Ly7	1	26,992,682	23,664,656	87.67%
	2	27,520,987	24,460,363	88.88%

Table S3   BCL6-associated STAT3 binding regions

Coordinates	Relative to TSS	GCB mean	ABC mean	Fold change	FDR
**Proximal to TSS**					
chr3: 187465839-187466149	-2519	23.60	29.70	1.26	6.34E-01
chr3: 187463162-187463620	+84	269.83	217.87	0.81	6.49E-01
chr3: 187459876-187460179	+3447	17.18	21.34	1.24	3.08E-01
chr3: 187457782-187458196	+5486	20.85	25.02	1.20	5.76E-01
chr3: 187456628-187456917	+6702	13.52	21.93	1.62	8.55E-02
**Differential STAT3 binding, FDR < 0.05**					
chr3: 187718164-187718648	-254931	21.57	8.52	0.39	5.39E-05
chr3: 187691802-187692078	-228465	17.20	46.39	2.70	5.67E-08
chr3: 187676418-187676807	-213138	30.48	17.19	0.56	5.18E-03
chr3: 187664324-187664610	-200992	18.02	9.28	0.51	6.92E-04
chr3: 187663823-187664237	-200555	23.78	13.13	0.55	2.81E-03
chr3: 187662495-187662971	-199258	37.25	21.87	0.59	2.16E-02
chr3: 187637464-187637777	-174146	12.62	24.54	1.94	2.94E-03
**No binding change, FDR ≥ 0.05**					
chr3: 187810509-187810889	-347224	21.64	27.38	1.27	1.02E-01
chr3: 187802985-187803493	-339764	26.97	27.44	1.02	8.82E-01
chr3: 187697374-187697744	-234084	31.46	32.09	1.02	8.22E-01
chr3: 187696442-187697009	-233251	31.25	26.35	0.84	5.75E-01
chr3: 187694135-187694445	-230815	21.84	16.36	0.75	9.11E-02
chr3: 187687424-187687774	-224124	26.22	22.90	0.87	4.75E-01
chr3: 187653559-187654015	-190312	32.38	30.23	0.93	7.86E-01
chr3: 187650339-187650713	-187051	20.27	22.18	1.09	9.32E-01
chr3: 187636865-187637222	-173569	19.12	24.24	1.27	4.71E-01
chr3: 187619873-187620465	-156694	34.76	34.35	0.99	7.57E-01
chr3: 187618642-187619059	-155376	26.63	24.31	0.91	9.59E-01
chr3: 187491071-187491347	-27734	10.74	17.00	1.58	7.11E-02

Table S4 BRs by binding

Chromosome	Start	End	foldChange	FDR	Gene1	Peak1	Gene2	Peak2	Gene3	Peak3	Gene4	Peak4
**Unique to ABC**												
chr9	5920972	5921248	19.49	1.76E-51	MLANA	30201	RANBP6	94508				
chr6	133383848	133384124	16.78	3.73E-51	EYA4	-178509	RPS12	248278				
chr14	103785780	103785965	37.93	1.60E-46	EIF5	-14620	TNFAIP2	193209				
chr1	59512477	59512749	15.88	3.58E-45	JUN	-262828	FGGY	-250012				
chr12	11802461	11802706	17.46	6.36E-45	ETV6	-204						
chr5	40502911	40503187	14.97	6.36E-45	PTGER4	-176983						
chr9	98614870	98615154	17.90	6.36E-45	PTCH1	-344181	C9orf102	-22888				
chr3	172230265	172230541	11.00	4.49E-42	GHSR	-64200	TNFSF10	10866				
chr2	165610733	165611009	13.24	3.50E-40	GRB14	-132511	SLC38A11	201164				
chr5	95263659	95263935	12.54	6.10E-40	GLRX	-105220	ELL2	33978				
chr2	165766681	165766957	16.01	6.52E-39	GRB14	-288459	SLC38A11	45216				
chr10	24672659	24672970	9.79	2.58E-38	KIAA1217	175095	ARHGAP21	339782				
chr18	55768528	55768814	14.46	4.53E-37	NEDD4L	57052	ALPK2	527518				
chr3	186706720	186706996	11.39	5.07E-37	ST6GAL1	-32807	ADIPOQ	146395				
chr18	42087971	42088270	16.39	2.40E-35	SETBP1	-172742						
chr12	102330026	102330319	11.25	3.06E-35	DRAM1	59068	CCDC53	125726				
chr21	37915128	37915368	11.69	4.54E-35	SIM2	-156743	CLDN14	-76523				
chr18	55618482	55618718	17.64	5.30E-35	ATP8B1	-219561	NEDD4L	-93019				
chr18	55598391	55598656	21.69	3.25E-34	ATP8B1	-199485	NEDD4L	-113095				
chr18	49479891	49480173	7.98	7.01E-34	MEX3C	-756342	DCC	-386539				
chr18	52908371	52908616	11.26	7.01E-34	TCF4	347366	RAB27B	412654				
chr17	76356081	76356364	10.94	2.66E-33	SOCS3	-65						
chr12	103899120	103899424	8.78	4.30E-33	STAB2	-81797	ASCL1	547820				
chr19	57781319	57781595	8.84	4.62E-33	ZNF460	-10396	ZNF805	29404				
chr11	123350217	123350500	9.49	4.17E-32	ASAM	-284352	GRAMD1B	-46169				
chr17	76274427	76274711	8.90	4.17E-32	LOC283999	47178	SOCS3	81589				
chr21	47012333	47012610	7.46	2.83E-31	PCBP3	-257403	SLC19A1	-50121				
chr17	37640480	37640844	13.15	3.83E-31	CRKRS	22923	NEUROD2	123513				
chr17	78228617	78228904	9.17	8.30E-31	RNF213	-84965	SLC26A11	34524				
chr18	53273053	53273434	10.94	9.43E-31	TCF4	-17384						
chr15	64194186	64194462	9.17	1.59E-30	HERC1	-68177	DAPK2	144197				
chr11	27255648	27255970	8.81	2.63E-30	BBOX1	193300	LGR4	238525				
chr12	24895354	24895664	8.83	3.81E-30	SOX5	-792872	BCAT1	206799				
chr20	49133153	49133436	8.09	1.50E-29	PARD6B	-214786	PTPN1	6404				
chr19	47689713	47689953	7.72	1.53E-29	BBC3	46190	SAE1	55753				
chr18	67616215	67616491	7.02	2.16E-29	CD226	7879	DOK6	548062				

chr18	9069588	9069938	9.75	2.79E-29	NDUFV2	-32912	RAB12	460320
chr18	53915940	53916195	8.19	9.96E-29	TCF4	-660208	TXNL1	390202
chr21	23595785	23596105	8.19	1.50E-28				
chr3	146187214	146187502	7.70	1.70E-27	PLSCR4	-218392	PLSCR2	26364
chr16	76610954	76611198	8.60	3.41E-27	LOC100130958	-622273	CNTNAP4	267341
chr19	17958725	17958984	12.46	5.21E-27	JAK3	-14		
chr19	28474885	28475161	11.68	5.21E-27				
chr21	19833298	19833603	7.80	6.93E-27	PRSS7	-57481		
chr12	40707757	40708036	8.67	8.78E-27	CNTN1	-378461	LRRK2	89084
chr3	177061573	177061895	7.80	1.27E-26	TBL1XR1	-146686		
chr7	150019533	150019809	5.85	1.27E-26	LRRC61	-625		
chr20	49163433	49163709	6.28	2.20E-26	PARD6B	-184510	PTPN1	36680
chr18	55816177	55816489	7.52	2.94E-26	NEDD4L	104714	ALPK2	479856
chr18	60952649	60952914	7.44	4.02E-26	BCL2	33831	PHLPP1	570048
chr11	123985983	123986265	9.52	5.72E-26	OR10G7	-76416	X64983	-70227
chr7	30839247	30839523	6.53	6.32E-26	GHRHR	-164251	FAM188B	28352
chr19	45257633	45257915	8.43	6.36E-26	CBLC	-23352	BCL3	5796
chr18	53988700	53989075	7.30	6.90E-26	TCF4	-733028	TXNL1	317382
chr16	10007393	10007669	14.08	1.50E-25	USP7	-950190	GRIN2A	268732
chr4	78508118	78508401	8.24	2.75E-25	CXCL13	75353	CNOT6L	232284
chr19	50426565	50426859	6.94	3.22E-25	IL4I1	-26565	ATF5	-5262
chr21	45274947	45275285	7.72	1.23E-24	AGPAT3	-10000	RRP1	65698
chr2	8518458	8518734	7.76	1.30E-24	ID2	-303388		
chr11	356254	356530	5.87	1.78E-24	IFITM3	-35478	B4GALNT4	-13403
chr1	155163072	155163348	5.89	2.89E-24	MUC1	-510		
chr2	181971576	181971871	12.81	2.91E-24	ITGA4	-349895	UBE2E3	126612
chr20	42379852	42380155	6.71	2.91E-24	TOX2	-163488	GTSF1L	-24362
chr3	194210661	194210934	9.72	3.50E-24	ATP13A3	-21830	TMEM44	143315
chr18	51811452	51811847	5.70	1.16E-23	POLI	15801	STARD6	69293
chr11	35062228	35062511	6.52	4.36E-23	APIP	-124431	CD44	-98047
chr18	53256911	53257261	6.84	4.81E-23	TCF4	-1226		
chr3	186229027	186229271	7.35	5.76E-23	DGKG	-149126	CRYGS	33018
chr11	111238440	111238680	9.14	6.47E-23	C11orf92	-68021	POU2AF1	11597
chr18	77139122	77139372	6.26	6.59E-23	NFATC1	-21079	ATP9B	309850
chr11	118712198	118712510	6.42	6.75E-23	DDX6	-50382	CXCR5	-42187
chr11	35364825	35365101	10.77	8.02E-23	SLC1A2	76142	CD44	204546
chr7	130756852	130757141	6.96	9.81E-23	KLF14	-338137	MKLN1	-255598
chr12	10902813	10903089	10.98	1.18E-22	CSDA	-26998	TAS2R7	52275

chr8	134058583	134058968	5.94	1.41E-22	SLA	13827	TG	179571
chr14	20959936	20960218	7.49	1.60E-22	RNASE10	-18554	NP	22535
chr18	41143819	41144095	6.14	1.63E-22	SYT4	-286342		
chr18	60828337	60828587	8.24	2.75E-22	BCL2	158151	PHLPP1	445728
chr6	45798305	45798591	9.73	3.15E-22	SUPT3H	-452778	CLIC5	249637
chr6	64229244	64229520	7.96	3.82E-22	LGSN	-199500	PTP4A1	-52538
chr19	13010666	13010972	6.10	9.03E-22	GCDH	8845	SYCE2	19267
chr6	471273	471549	5.42	1.79E-21	IRF4	79659	EXOC2	221698
chr2	112236658	112236934	16.33	2.27E-21	BCL2L11	358305	ANAPC1	404945
chr7	130645126	130645370	10.87	2.27E-21	MKLN1	-367347	KLF14	-226388
chr15	101137048	101137324	7.60	3.02E-21	ALDH1A3	-282823	LASS3	-52261
chr16	57023276	57023555	4.82	3.02E-21	NLRC5	6		
chr19	1132195	1132453	10.92	3.73E-21	GPX4	28388	SBNO2	41958
chr1	94108121	94108380	6.33	3.86E-21	BCAR3	39134	FNBP1L	194412
chrX	15866783	15867080	10.79	5.41E-21	AP1S2	6168	ZRSR2	58358
chr4	72978329	72978632	6.37	5.77E-21	NPFFR2	80960	ADAMTS3	456035
chr2	216882193	216882494	5.11	7.95E-21	MREG	-3998		
chr22	50328892	50329181	7.71	8.77E-21	PIM3	-25106	CRELD2	16754
chr8	126614195	126614551	5.97	1.08E-20	TRIB1	171810	FAM84B	956093
chr3	188027437	188027745	7.24	1.90E-20	TPRG1	-862172	LPP	96870
chr14	76009196	76009569	10.06	2.60E-20	FLVCR2	-35557	BATF	20599
chr18	53043702	53043990	5.81	2.85E-20	TCF4	212014	RAB27B	548006
chr5	158325184	158325510	5.60	2.85E-20	EBF1	201441		
chr6	161171259	161171567	5.49	2.85E-20	MAP3K4	-241409	PLG	48139
chr11	26936587	26936863	6.16	3.11E-20	SLC5A12	-193151	FIBIN	-78903
chr17	56756796	56757072	9.17	3.52E-20	SEPT4	-150271	TEX14	12482
chr3	150803233	150803511	6.50	3.69E-20	MED12L	-1213		
chr5	54061661	54061905	10.09	4.23E-20	ESM1	219631	SNX18	248190
chr10	63808265	63808541	6.14	4.70E-20	ARID5B	146960	RTKN2	220063
chr17	37926197	37926473	5.12	4.85E-20	GRB7	32148	IKZF3	94106
chr2	60641027	60641323	6.82	6.44E-20	BCL11A	139458		
chr3	194928900	194929420	4.87	1.05E-19	LSG1	-535954	C3orf21	62735
chr11	35051554	35051835	5.66	1.10E-19	APIP	-113756	CD44	-108722
chr4	140099087	140099416	5.42	1.12E-19	ELF2	-38601	C4orf49	102240
chr22	37518196	37518481	4.93	1.33E-19	TMPRSS6	-18646	IL2RB	27623
chr12	103924220	103924464	5.95	1.35E-19	STAB2	-56727	ASCL1	572890
chr1	118206975	118207304	5.46	1.68E-19	WDR3	-265232	MAN1A2	297055
chr18	52935290	52935745	5.50	1.89E-19	TCF4	320342	RAB27B	439678

chr3	122283022	122283298	6.30	2.20E-19	DTX3L	-25	PARP9	-13
chr19	16453161	16453466	7.40	3.05E-19	KLF2	17663	EPS15L1	129448
chr15	80271082	80271391	5.73	3.07E-19	ZFAND6	-80784	BCL2A1	-7594
chr5	55290877	55291153	5.07	3.38E-19	IL6ST	-252		
chr17	45949642	45949897	5.43	3.77E-19	SP2	-23746	SP6	-16530
chr5	66502878	66503154	6.27	3.87E-19	CD180	-10399		
chr18	24002714	24002999	9.21	7.73E-19	KCTD1	125643	TAF4B	196448
chr19	47685545	47685788	7.12	8.62E-19	BBC3	50356	SAE1	51587
chr2	231526363	231526639	4.39	8.76E-19	CAB39	-51056	SP100	245630
chr1	150539864	150540108	9.88	1.13E-18	MCL1	12150	ADAMTSL4	18088
chr1	75323810	75324086	6.54	1.20E-18	LHX8	-270171	CRYZ	-124856
chr17	74188028	74188315	5.45	1.27E-18	FOXJ1	-51527	RNF157	48218
chr18	57625285	57625595	9.95	1.30E-18	PMAIP1	58248	MC4R	414561
chr3	142800899	142801352	4.98	1.31E-18	CHST2	-37542	SR140	80754
chr15	90325681	90325995	5.25	1.71E-18	MESP2	6249	ANPEP	32234
chr18	55897860	55898357	4.74	1.93E-18	NEDD4L	186490	ALPK2	398080
chr3	150811023	150811319	7.60	2.46E-18	MED12L	6586	GPR171	109817
chr17	57863867	57864130	19.32	2.74E-18	TMEM49	79136	TUBD1	106297
chrX	100663308	100663584	6.73	2.94E-18	GLA	-445	HNRNPH2	255
chr4	167760854	167761222	5.66	3.47E-18	SPOCK3	394703	TLL1	966628
chr4	102728126	102728402	4.78	3.82E-18	BANK1	16500	SLC39A8	538391
chr17	67112134	67112580	5.13	5.01E-18	ABCA9	-55221	ABCA6	25658
chr8	133991289	133991585	5.82	5.73E-18	SLA	81166	TG	112232
chr6	20769220	20769496	6.17	6.29E-18	SOX4	-824614	CDKAL1	234670
chr17	25871616	25871942	5.23	6.47E-18	LGALS9	-86395	KSR1	72743
chr3	195919187	195919427	4.43	1.02E-17	TFRC	-110275	ZDHHC19	18993
chr9	134503373	134503679	6.71	1.13E-17	UCK1	-96864	RAPGEF1	81703
chr7	106065467	106065818	4.42	1.37E-17	PIK3CG	-440281	NAMPT	-140005
chr7	52894253	52894497	6.78	1.56E-17				
chr22	38672278	38672663	6.35	1.59E-17	TMEM184B	-3455		
chr21	36260882	36261158	4.70	1.63E-17	C21orf96	150703	CLIC6	219332
chr18	3586721	3587008	5.60	1.75E-17	TGIF1	135274	DLGAP1	293275
chr5	158883837	158884114	5.25	2.34E-17	ADRA1B	-459764	IL12B	-126495
chr17	63911001	63911277	6.19	2.35E-17	AXIN2	-353399	APOH	314417
chr18	55758445	55758926	4.76	2.43E-17	NEDD4L	47067	ALPK2	537503
chr17	57906607	57906910	5.24	2.62E-17	TUBD1	63537	TMEM49	121896
chr21	44584860	44585253	4.65	3.04E-17	CRYAA	-4084		
chr3	101778418	101778732	5.02	3.45E-17	ZPLD1	-375284	NFKBIZ	210217

chr17	30842915	30843191	6.03	3.60E-17	CDK5R1	28948	MYO1D	360849
chr8	129076524	129076968	4.60	3.60E-17	MYC	328431		
chr8	60060281	60060582	5.45	4.41E-17	TOX	-28665		
chr11	82680949	82681251	5.18	4.82E-17	C11orf82	68363	RAB30	101784
chr9	98585149	98585429	6.52	6.67E-17	PTCH1	-314458	C9orf102	-52611
chr7	17199160	17199534	5.37	7.26E-17	AGR3	-277734	AHR	-138929
chr17	80408637	80408940	5.27	9.13E-17	C17orf62	-95	C17orf62	-95
chr2	208637244	208637520	9.77	9.13E-17	FZD5	-3239		
chr18	55682322	55682624	8.87	1.45E-16	ATP8B1	-283434	NEDD4L	-29146
chr13	107222809	107223143	6.08	1.96E-16	EFNB2	-35639		
chr14	103577238	103577522	5.54	3.35E-16	CDC42BPB	-53638	TNFAIP2	-15284
chr19	947785	948075	5.13	4.95E-16	GRIN3B	-52507	ARID3A	21893
chr18	53052197	53052520	8.08	5.23E-16	TCF4	203501	RAB27B	556519
chr7	17441828	17442104	7.05	6.45E-16	AHR	103690	SNX13	538165
chr3	99657398	99657698	6.81	6.80E-16	C3orf26	120867	FILIP1L	175801
chr3	101568124	101568402	4.90	7.14E-16	NFKBIZ	-95		
chr5	32211270	32211546	4.40	7.14E-16	GOLPH3	-36983	MTMR12	101706
chr12	8782112	8782415	4.28	7.30E-16	AICDA	-16822	MFAP5	33169
chr11	102459815	102460091	5.32	8.21E-16	MMP7	-58475	MMP20	36110
chr3	72034174	72034474	5.17	8.26E-16	PROK2	-199967	RYBP	461450
chr18	51790374	51790662	5.24	9.54E-16	MBD2	-39360	POLI	-5331
chr3	177538503	177538779	6.73	1.30E-15	KCNMB2	-715583	TBL1XR1	-623593
chr10	125157823	125158099	5.05	1.35E-15	GPR26	-267910	BUB3	244201
chr5	54082032	54082317	6.24	1.35E-15	ESM1	199239	SNX18	268582
chr3	186239236	186239578	6.15	1.37E-15	DGKG	-159384	CRYGS	22760
chr3	119041904	119042185	4.57	1.48E-15	CDGAP	28825	TMEM39A	140426
chr8	135575999	135576285	4.66	1.52E-15	ST3GAL1	-991959	ZFAT	149139
chr1	198171533	198171809	7.43	1.75E-15	NEK7	45563	ATP6V1G3	338404
chr18	61185718	61186054	4.57	2.38E-15	SERPINB12	-37507	SERPINB5	41742
chr10	45474119	45474459	6.02	2.45E-15	C10orf10	41		
chr6	4343088	4343332	6.30	2.92E-15	CDYL	-433470	PECI	-207379
chr3	178686801	178687077	4.51	2.96E-15	ZMAT3	102645	KCNMB2	432715
chr15	90931108	90931384	3.86	3.43E-15	IQGAP1	-227		
chr12	103134475	103134751	8.15	3.61E-15	IGF1	-260235	PAH	176768
chr9	127632536	127632820	5.37	4.14E-15	ARPC5L	1194	GOLGA1	70708
chr18	57567119	57567370	4.70	4.16E-15	PMAIP1	53		
chr18	56737727	56737995	5.01	4.31E-15	SEC11C	-69264	ZNF532	207800
chr6	76636067	76636367	6.30	4.83E-15	IMPG1	146118	MYO6	177308

chr9	140732688	140732955	4.59	5.01E-15	CACNA1B	-39419	EHMT1	219378
chr12	122429206	122429550	5.48	5.16E-15	BCL7A	-30483	PSMD9	102732
chr1	236767725	236768005	5.90	6.06E-15	HEATR1	-51		
chr2	8628202	8628579	4.44	6.22E-15	ID2	-193593		
chr18	9066351	9066627	7.63	6.64E-15	NDUFV2	-36186	RAB12	457046
chr18	77134829	77135111	6.26	7.51E-15	NFATC1	-25356	ATP9B	305573
chr22	50978023	50978388	5.89	7.68E-15	TYMP	-9948	CPT1B	38672
chr9	134505751	134506027	5.66	1.12E-14	UCK1	-99227	RAPGEF1	79340
chr10	63752508	63752796	4.86	1.13E-14	ARID5B	91209	RTKN2	275814
chr1	12079252	12079532	4.26	1.14E-14	TNFRSF8	-44042	MFN2	39154
chr20	4793719	4793995	4.52	1.18E-14	RASSF2	1912	PRND	91301
chr2	8685138	8685431	4.23	1.20E-14	ID2	-136699		
chr12	93639397	93639650	4.66	1.22E-14	EEA1	-316417	NUDT4	-132177
chr13	27705357	27705700	4.37	1.34E-14	GPR12	-370607	USP12	40500
chr12	96602599	96602933	4.45	1.44E-14	ELK3	14559	PCTK2	191457
chr14	106284244	106284695	5.69	1.44E-14	IGHE	44992	TMEM121	291517
chr12	57452345	57452623	5.96	1.46E-14	MYO1A	-8594	TMEM194A	20090
chr17	53658151	53658462	8.74	1.50E-14	MMD	-158966	TMEM100	141918
chr5	54198683	54198969	4.67	1.67E-14	ESM1	82588	SNX18	385233
chrX	118751811	118752133	6.21	1.90E-14	NKRF	-12159	SEPT6	75361
chr17	76642055	76642351	5.43	2.00E-14	DNAH17	-74799	CYTH1	136173
chr14	75957936	75958263	4.97	2.13E-14	BATF	-30684	JDP2	59263
chr14	75980794	75981038	4.35	2.50E-14	BATF	-7868	JDP2	82079
chr7	2934119	2934459	4.48	2.98E-14	GNA12	-50330	CARD11	149290
chr18	55626531	55626853	3.86	3.07E-14	ATP8B1	-227653	NEDD4L	-84927
chr1	75277088	75277365	5.67	3.35E-14	LHX8	-316892	CRYZ	-78135
chr18	43554102	43554393	3.96	3.49E-14	PSTPIP2	98002	SIGLEC15	148703
chr4	167583447	167583751	4.28	3.49E-14	SPOCK3	572142	TLL1	789189
chr5	130729884	130730186	4.39	3.57E-14	CDC42SE2	130333	RAPGEF6	240894
chr3	33091609	33091885	8.93	4.22E-14	TMPPE	46567	CCR4	98681
chr9	139298056	139298350	3.98	4.44E-14	SNAPC4	-5314	SDCCAG3	6851
chr12	8801257	8801538	5.12	4.48E-14	AICDA	-35956	MFAP5	14035
chrX	48795970	48796293	4.07	5.12E-14	PIM2	-19719	OTUD5	18761
chr19	893184	893439	11.84	5.32E-14	MED16	-94		
chr22	38852011	38852287	4.32	5.33E-14	KCNJ4	-12116	KDELR3	-11934
chr20	36722770	36723055	4.29	5.78E-14	KIAA0406	-61080	TGM2	70787
chr1	159046984	159047340	11.92	6.12E-14	AIM2	-515		
chr22	35807052	35807328	4.02	6.23E-14	RASD2	-130162	MCM5	11074

chr16	29831611	29831887	3.75	6.83E-14	MVP	-38		
chr16	87731868	87732190	3.99	7.34E-14	KLHDC4	67513	JPH3	95530
chr15	55581878	55582175	4.09	8.17E-14	PIGB	-29106	RAB27A	-18920
chr21	36584792	36585075	6.09	8.38E-14	CBR1	-857351	RUNX1	-163339
chr7	28191532	28191834	4.27	8.82E-14	JAZF1	28754	TAX1BP1	411945
chr18	55879535	55879825	4.12	8.95E-14	NEDD4L	168061	ALPK2	416509
chr2	8683509	8684110	4.07	1.10E-13	ID2	-138174		
chr2	198081273	198081549	3.93	1.10E-13	ANKRD44	-18649	SF3B1	218360
chr4	145556148	145556424	6.51	1.15E-13	GYPA	-494382	HHIP	-10887
chr8	135704533	135704786	6.04	1.30E-13	ZFAT	20621		
chr12	104872163	104872439	4.16	1.33E-13	CHST11	21523	SLC41A2	450171
chr3	126690874	126691201	3.58	1.41E-13	TXNRD3	-317093	PLXNA1	-16464
chr1	42133024	42133344	3.62	1.51E-13	EDN2	-182840	HIVEP3	251312
chr3	194850201	194850615	4.23	1.79E-13	LSG1	-457202	C3orf21	141487
chr18	56459347	56459623	5.22	2.01E-13	ZNF532	-70576	MALT1	120867
chr18	36520830	36521147	4.38	2.15E-13				
chr11	121289397	121289644	4.99	2.34E-13	SORL1	-33440	SC5DL	126133
chr2	160735690	160735966	6.26	2.42E-13	LY75	25434	MARCH7	166818
chr9	140205075	140205362	5.74	2.85E-13	NRARP	-8516	EXD3	112495
chr18	56633584	56633830	4.95	2.88E-13	SEC11C	-173418	ZNF532	103646
chr12	132258048	132258409	3.56	3.25E-13	MMP17	-54712	SFRS8	62594
chr4	105981783	105982087	5.35	3.42E-13	CXXC4	-569468	TET2	-86008
chr11	3071072	3071377	3.82	4.31E-13	NAP1L4	-57618	CARS	7446
chr19	47792751	47793210	3.66	4.64E-13	C5AR1	-20123	PRR24	14839
chr19	57791082	57791392	5.57	4.69E-13	ZNF460	-616		
chr8	133932323	133932606	7.45	4.89E-13	TG	53260	SLA	140138
chr18	60809034	60809314	5.10	4.90E-13	BCL2	177439	PHLPP1	426440
chr14	75988637	75988917	3.84	4.97E-13	BATF	-7		
chr2	25633308	25633584	4.02	5.00E-13	DNMT3A	-68672	DTNB	263057
chr12	125259140	125259419	3.96	5.55E-13	NCOR2	-239123	SCARB1	89239
chr6	2857881	2858303	4.21	6.05E-13	SERPINB1	-16011	SERPINB9	45453
chr2	46137030	46137320	5.00	6.40E-13	EPAS1	-387388	PRKCE	258132
chr6	24940632	24940908	3.78	6.63E-13	FAM65B	-29575	DKFZp686H12134	197281
chr20	21284245	21284521	3.60	6.95E-13	XRN2	441		
chr11	103800264	103800526	4.24	7.06E-13	DDI1	-106913	DYNC2H1	820235
chr9	98029043	98029322	5.32	7.13E-13	FANCC	50808	C9orf3	540189
chr2	55361016	55361315	4.74	7.85E-13	RPS27A	-97899	RTN4	-83432
chr2	10951059	10951358	3.68	1.13E-12	PDIA6	1751	ATP6V1C2	89434

chr3	150886135	150886411	4.61	1.13E-12	GPR171	34715	MED12L	81688
chr12	11903384	11903660	4.33	1.85E-12	BCL2L14	-320356	ETV6	100734
chr18	12839733	12840009	3.97	1.85E-12	PTPN2	44463	PSMG2	136807
chr21	44582645	44583046	3.88	1.85E-12	U2AF1	-55158	CRYAA	-6295
chr12	112568372	112568685	3.80	2.21E-12	TRAFD1	5180	C12orf51	175509
chr18	3593934	3594260	4.80	2.55E-12	TGIF1	142506	DLGAP1	286043
chr19	2475691	2475971	3.93	2.66E-12	GADD45B	-304		
chr21	26561044	26561320	4.11	2.69E-12	MRPL39	418619		
chr19	56632703	56633053	3.71	2.89E-12	ZNF787	-229		
chr19	10381574	10381850	3.91	3.27E-12	ICAM1	195		
chr5	140090676	140090985	3.88	3.91E-12	PCDHAC2	-255281	ZMAT2	10799
chr22	27054911	27055216	3.94	4.35E-12	CRYBA4	37136		
chr17	76339741	76340093	3.98	4.86E-12	SOCS3	16241	LOC283999	112526
chr4	141712037	141712421	4.51	5.70E-12	TBC1D9	-34758	RNF150	342387
chr17	12154233	12154577	4.74	5.71E-12	MYOCD	-414802	MAP2K4	230270
chr9	82749497	82749750	6.86	5.98E-12	TLE4	562746		
chr3	32480507	32480768	4.08	6.27E-12	CMTM7	47475	CMTM6	63765
chr16	48492304	48492580	4.80	7.21E-12	SIAH1	-73213	N4BP1	151678
chr2	242067139	242067386	3.87	8.46E-12	PASK	21615	SNED1	129008
chrX	48794319	48794614	5.56	9.61E-12	PIM2	-18054	OTUD5	20426
chr11	123349780	123350169	4.08	9.88E-12	ASAM	-283968	GRAMD1B	-46553
chr19	36202156	36202502	4.76	9.89E-12	ZBTB32	-1501		
chr1	150551940	150552220	4.04	1.02E-11	MCL1	56		
chr17	12762960	12763279	4.59	1.15E-11	RICH2	70291	ELAC2	158239
chr16	19865609	19865913	5.71	1.20E-11	C16orf88	-136269	GPRC5B	30390
chr18	53073121	53073436	4.86	1.20E-11	TCF4	182581	RAB27B	577439
chr1	205282831	205283155	4.50	1.23E-11	NUAK2	7890	TMCC2	85902
chr17	73872156	73872432	3.56	1.29E-11	UNC13D	-31496	TRIM47	2362
chr3	183309748	183310075	6.15	1.29E-11	KLHL24	-43499	KLHL6	-36413
chr9	130533626	130533902	3.44	1.29E-11	TOR2A	-36160	SH2D3C	7284
chr4	164512786	164513133	4.42	1.32E-11	TKTL2	-117913	MARCH1	21816
chr5	98363130	98363415	3.83	1.33E-11	CHD1	-101035		
chr14	73689739	73690050	4.21	1.37E-11	PAPLN	-14310	PSEN1	86752
chr12	27373199	27373475	5.75	1.62E-11	STK38L	-23741	MED21	197854
chr12	66696106	66696418	3.14	1.67E-11	HELB	-94		
chr5	131826354	131826730	3.87	1.85E-11	IRF1	-77		
chr18	73029727	73030003	5.69	2.20E-11	TSHZ1	107134	C18orf62	109724
chr19	53510549	53510793	3.52	2.58E-11	AK127846	-94		

chr10	12164709	12164977	4.30	2.65E-11	SEC61A2	-6797	DHTKD1	53909
chr20	20432914	20433236	4.24	2.71E-11	INSM1	84310	RALGAPA2	260191
chr1	207752765	207753058	3.98	2.72E-11	CR1L	-65546	CR1	83439
chr8	129115768	129116245	3.36	3.01E-11	MYC	367692		
chr15	31638213	31638489	4.10	3.07E-11	KLF13	19268	OTUD7A	309191
chr22	38656280	38656604	3.38	3.09E-11	TMEM184B	12574	MAFF	46900
chr12	24972604	24972854	5.46	3.18E-11	SOX5	-870092	BCAT1	129579
chr1	54355376	54355670	3.47	3.53E-11	DIO1	-4338	YIPF1	-70
chr11	103751780	103752056	5.25	3.56E-11	DDI1	-155390	DYNC2H1	771758
chr18	33765217	33765493	4.71	3.56E-11	MOCOS	-2125		
chr18	60708352	60708695	4.96	3.56E-11	BCL2	278089	PHLPP1	325790
chr17	80256027	80256343	3.69	3.63E-11	CSNK1D	-24612	CD7	19295
chr5	110590427	110590690	3.64	3.65E-11	CAMK4	30477	STARD4	257598
chr18	57069734	57070046	4.67	3.96E-11	LMAN1	-43382	CCBE1	294754
chr15	40339935	40340280	3.92	4.04E-11	SRP14	-8719	BMF	60967
chr22	37484081	37484357	4.30	4.04E-11	TMPRSS6	15474	KCTD17	36440
chr15	42750126	42750402	3.65	4.78E-11	ZFP106	-534		
chr16	10008221	10008542	4.85	4.78E-11	USP7	-951041	GRIN2A	267881
chr18	56631000	56631311	3.56	4.96E-11	SEC11C	-175969	ZNF532	101095
chr12	46502541	46502857	4.03	4.99E-11	SFRS2IP	-118298	SLC38A1	160509
chr20	51590641	51590911	5.80	5.08E-11	TSHZ2	1899	ZNF217	608931
chr15	85279599	85279996	3.65	5.10E-11	SEC11A	-20124	ZNF592	-12020
chr18	60683545	60683822	3.36	5.24E-11	PHLPP1	300950	BCL2	302929
chr10	53459582	53459858	3.82	5.35E-11	CSTF2T	-365		
chr1	17518908	17519184	6.62	5.64E-11	PADI2	-73098	PADI1	-12575
chr6	221118	221400	3.63	6.83E-11	DUSP22	-70842		
chr19	35838409	35838860	4.65	7.74E-11	FFAR1	-3810		
chr18	55789154	55789539	3.21	8.13E-11	NEDD4L	77728	ALPK2	506842
chr2	58135895	58136173	9.33	9.81E-11	VRK2	-137695		
chr18	12879837	12880113	4.01	1.03E-10	PTPN2	4359	PSMG2	176911
chr7	105410455	105410762	4.07	1.14E-10	FLJ23834	-193048	RINT1	238077
chr18	55549133	55549495	3.39	1.18E-10	NEDD4L	-162305	ATP8B1	-150275
chr3	169774598	169775113	3.68	1.26E-10	GPR160	19121	PHC3	124681
chr1	172750683	172750995	4.12	1.31E-10	FASLG	122654	TNFSF18	269264
chr12	27361042	27361303	4.95	1.36E-10	STK38L	-35905	MED21	185690
chr17	58469540	58469900	3.65	1.41E-10	USP32	-134		
chr11	111921490	111921772	5.03	1.47E-10	DLAT	26093	TIMM8B	35828
chr1	150534560	150534903	22.57	1.51E-10	ADAMTSL4	12834	MCL1	17404

chr17	73316262	73316615	3.45	1.55E-10	SLC25A19	-30909	GRB2	85351
chr5	177659706	177659999	3.22	1.66E-10	AGXT2L2	-50		
chr12	122470522	122470805	3.49	1.79E-10	MLXIP	-46096	BCL7A	10803
chr20	20444732	20445010	3.31	1.95E-10	INSM1	96106	RALGAPA2	248395
chr16	59627200	59627456	5.99	2.10E-10	GOT2	-859082		
chr3	132036014	132036274	4.18	2.16E-10	ACPP	-67		
chr14	81937533	81937853	4.07	2.17E-10	STON2	-72769	SEL1L	62512
chr16	18937824	18938131	3.31	2.30E-10	SMG1	-252		
chr20	49461297	49461541	4.96	2.31E-10	BCAS4	49952	ADNP	86108
chr11	118709620	118710084	3.49	2.33E-10	DDX6	-47880	CXCR5	-44689
chr2	64281274	64281627	2.95	2.54E-10	VPS54	-35237	PELI1	90154
chr17	77005714	77006079	2.99	2.59E-10	CANT1	2		
chr4	77120836	77121100	3.03	2.66E-10	NUP54	-51313	SCARB2	14067
chr19	50850609	50850885	3.30	2.83E-10	NAPSB	-2742		
chr4	185395779	185396086	3.21	3.03E-10	IRF2	-207		
chr18	57364534	57365024	4.49	3.06E-10	CCBE1	-135		
chr16	71887376	71887667	3.39	3.31E-10	ATXN1L	7623	ZNF821	29890
chr20	33735017	33735300	3.06	3.42E-10	EDEM2	2		
chr11	65188730	65189022	5.83	3.50E-10	SCYL1	-103672	FRMD8	34835
chr18	60251605	60251879	3.34	3.76E-10	PHLPP1	-130992	ZCCHC2	61084
chr7	50418962	50419259	3.66	3.92E-10	IKZF1	74733	FIGNL1	98977
chrX	134248462	134248761	7.57	3.98E-10	FAM127A	82279	ZNF75D	229345
chr6	119670952	119671232	3.56	4.12E-10	MAN1A1	-166		
chr1	221219537	221219813	3.52	4.21E-10	HLX	166932	DUSP10	695786
chr6	37018002	37018458	3.83	4.67E-10	PIM1	-119692	FGD2	44807
chr1	185572412	185572724	3.48	4.79E-10	IVNS1ABP	-286107	HMCN1	-131115
chr8	24244967	24245326	3.69	4.98E-10	ADAM7	-53362	ADAMDEC1	3349
chr1	66736898	66737174	3.42	5.18E-10	SGIP1	-262789	PDE4B	478180
chr19	45504370	45504688	2.79	5.21E-10	RELB	-183		
chr13	99959570	99959848	3.57	5.38E-10	GPR183	40		
chr7	28744959	28745240	3.44	5.59E-10	TRIL	252929	CREB5	292956
chr3	69132039	69132354	3.46	5.66E-10	UBA3	-2673	ARL6IP5	-1893
chr8	59864471	59864747	4.23	6.06E-10	NSMAF	-292643	TOX	167158
chr19	28884977	28885253	3.17	6.17E-10	UQCRFS1	819021		
chr14	102241151	102241491	3.01	6.18E-10	DYNC1H1	-189544	PPP2R5C	13186
chr9	6689964	6690243	3.74	6.23E-10	KDM4C	-67537	GLDC	-44412
chr1	204422311	204422556	3.63	6.40E-10	PPP1R15B	-41490	PIK3C2B	37040
chr16	76997540	76997859	3.11	7.21E-10	LOC100130958	-235649	CNTNAP4	653965

chr1	145421707	145422001	3.57	7.30E-10	TXNIP	-16608	HFE2	8663
chr1	226297951	226298264	3.85	8.19E-10	H3F3B	47687	ACBD3	76315
chr1	236319825	236320287	3.53	9.14E-10	GPR137B	14224	ERO1LB	125283
chr6	84937181	84937457	3.26	9.52E-10	KIAA1009	16		
chr15	91137632	91138004	4.33	1.01E-09	BLM	-122761	CRTC3	64620
chr17	40540661	40540981	17.89	1.05E-09	STAT3	-308		
chr6	75308159	75308431	5.67	1.05E-09	COL12A1	607328	CD109	902787
chr11	116933890	116934216	4.17	1.10E-09	APOA1	-225715	SIK3	34940
chr2	10829990	10830311	3.21	1.10E-09	NOL10	-39		
chr19	12904296	12904672	4.06	1.11E-09	JUNB	2174	PRDX2	8210
chr8	129062987	129063334	3.62	1.11E-09	MYC	314846		
chr5	137514518	137514870	3.15	1.15E-09	BRD8	-336	KIF20A	277
chr5	54060028	54060272	3.81	1.24E-09	ESM1	221264	SNX18	246557
chr18	42305616	42305895	3.11	1.26E-09	SLC14A2	-889010	SETBP1	44893
chr3	119357759	119358033	3.65	1.30E-09	POPDC2	21508	PLA1A	41174
chr1	235147342	235147988	4.95	1.37E-09	IRF2BP2	-402394	TOMM20	144591
chr1	245027774	245028122	3.17	1.37E-09	HNRNPU	-121		
chr1	152007086	152007362	3.26	1.38E-09	S100A10	-40510	S100A11	2287
chr12	121665107	121665366	4.37	1.38E-09	P2RX4	17573	CAMKK2	70874
chr2	222419996	222420272	3.65	1.42E-09	EPHA4	16876		
chr18	56203866	56204148	2.94	1.53E-09	ALPK2	92182	NEDD4L	492388
chr1	3573348	3573594	5.54	1.57E-09	TP73	4342	KIAA0495	90415
chr19	1897590	1897897	3.79	1.58E-09	FAM108A1	-12226	SCAMP4	-7629
chr18	60822684	60823034	4.16	1.64E-09	BCL2	163754	PHLPP1	440125
chr18	67861552	67861870	3.71	1.68E-09	CD226	-237479	RTTN	11251
chr3	126630348	126630598	3.20	1.71E-09	TXNRD3	-256528	PLXNA1	-77029
chr3	126673717	126674115	3.40	1.71E-09	TXNRD3	-299971	PLXNA1	-33586
chr4	150959801	150960168	4.65	1.71E-09	DCLK2	-40095		
chr8	24151467	24151772	3.25	1.75E-09	ADAM28	40		
chr3	15403116	15403532	3.21	1.78E-09	SH3BP5	-29220	METTL6	65718
chr16	11269682	11269998	3.63	1.79E-09	SOCS1	80199	CIITA	298785
chr17	49205441	49205717	4.16	1.81E-09	NME1-NME2	-25341	SPAG9	-7353
chr1	205761844	205762120	4.02	1.86E-09	RAB7L1	-17372	SLC41A1	20179
chr18	53100836	53101264	3.53	1.91E-09	TCF4	154810	RAB27B	605210
chr1	156163604	156163903	4.50	1.95E-09	SLC25A44	24		
chr7	134320855	134321138	4.27	1.96E-09	BPGM	-10534	AKR1B15	70764
chrX	71528752	71529028	6.24	2.10E-09	CITED1	-3126		
chr14	24630225	24630563	12.26	2.20E-09	IRF9	665		

chr15	70678921	70679275	3.29	2.20E-09	TLE3	-288842	UACA	376752
chr9	114747789	114748089	4.55	2.20E-09	UGCG	88733	SUSD1	189617
chr17	76272222	76272515	4.03	2.38E-09	LOC283999	44978	SOCS3	83789
chr11	57333640	57333928	3.61	2.44E-09	UBE2L6	1396	SMTNL1	24805
chr15	75016350	75016680	2.96	2.51E-09	EDC3	-28129	CYP1A1	1362
chr19	660313	660795	3.13	2.56E-09	RNF126	2673	FGF22	20628
chr3	88198798	88199089	3.02	2.66E-09	C3orf38	51		
chr2	111977031	111977361	3.46	2.67E-09	BCL2L11	98705	ANAPC1	664545
chr20	42381133	42381460	3.27	2.79E-09	TOX2	-162195	GTSF1L	-25655
chr2	165617783	165618064	4.48	2.80E-09	GRB14	-139564	SLC38A11	194111
chr2	225531724	225532052	3.53	2.80E-09	CUL3	-81778	DOCK10	375442
chr14	75886112	75886397	3.24	2.83E-09	JDP2	-12582	FOS	140774
chr3	112395694	112395971	3.21	2.84E-09	CCDC80	-35856	CD200R1L	168964
chr21	43947941	43948241	2.89	2.93E-09	PDE9A	-125771	SLC37A1	28349
chr18	48362882	48363158	3.78	3.00E-09	ME2	-42415	MRO	-16586
chr2	225906093	225906379	3.62	3.22E-09	CUL3	-456126	DOCK10	1094
chr10	63803225	63803587	3.21	3.26E-09	ARID5B	141963	RTKN2	225060
chr4	84101567	84101849	4.85	3.80E-09	COQ2	104256	COPS4	145469
chr18	44811084	44811445	3.19	3.83E-09	CORL2	-35711	SMAD2	646250
chr8	129186456	129186748	3.97	4.08E-09	MYC	438287		
chr2	145113956	145114206	4.57	4.26E-09	GTDC1	-62021	ZEB2	163835
chr13	42490688	42491071	3.36	4.33E-09	KIAA0564	44341	C13orf15	459338
chr7	105406861	105407114	5.08	4.33E-09	FLJ23834	-196669	RINT1	234456
chr19	49588237	49588531	3.20	4.36E-09	SNRNP70	-81	SNRNP70	-81
chr12	7261732	7262008	2.83	4.55E-09	C1RL	-77		
chr15	76867756	76868035	5.02	5.10E-09	ISL2	238749	SCAPER	308321
chr9	4464909	4465227	3.34	5.40E-09	GLIS3	-165033	SLC1A1	-25376
chr3	191013868	191014178	3.06	5.50E-09	CCDC50	-32851	OSTN	83701
chr13	20438927	20439203	3.90	5.61E-09	ZMYM5	-1289		
chr11	68180307	68180729	2.87	5.66E-09	SAPS3	-47668	LRP5	100410
chr4	40538082	40538372	3.47	5.68E-09	RBM47	93656	CHRNA9	200758
chr22	29999259	29999535	3.19	6.09E-09	NF2	-148		
chr12	94954622	94954995	3.39	6.10E-09	TMCC3	89515	PLXNC1	412310
chr8	66856731	66857060	4.59	6.11E-09	PDE7A	-155575	DNAJC5B	-76895
chr2	201988436	201988887	3.06	6.14E-09	CASP10	-58959	CFLAR	7846
chr18	44471757	44472037	3.12	6.25E-09	ST8SIA5	-134858	PIAS2	25569
chr1	212740495	212740829	3.18	6.93E-09	ATF3	1965	BATF3	132665
chr8	125774149	125774425	4.52	7.02E-09	ZNF572	-211252	MTSS1	-33557

chr14	76015470	76015817	2.85	7.76E-09	FLVCR2	-29296	BATF	26860
chr2	209176057	209176348	3.83	8.21E-09	PTH2R	-95353	PIKFYVE	45212
chr19	35837564	35837836	2.91	8.27E-09	FFAR1	-4745		
chr3	185255112	185255392	3.56	8.41E-09	TMEM41A	-38407	LIPH	15117
chr11	109898061	109898306	3.96	8.46E-09	ZC3H12C	-65903	C11orf87	605309
chr2	61989396	61989674	5.03	8.80E-09	XPO1	-224117	CCT4	126256
chr17	73291754	73292151	4.22	9.02E-09	SLC25A19	-6423	GRB2	109837
chr9	128463169	128463445	3.30	9.02E-09	MAPKAP1	6206	GAPVD1	439196
chr13	100072601	100072951	2.72	9.52E-09	GPR183	-113027	TM9SF2	-80952
chr1	51985027	51985355	2.44	9.82E-09	EPS15	-196		
chr15	70571058	70571357	3.04	9.82E-09	TLE3	-180952	UACA	484642
chr9	138022182	138022469	2.85	9.86E-09	KIAA0649	-349322	OLFM1	55237
chr16	81782040	81782392	3.90	1.06E-08	PLCG2	-30714	CMIP	303441
chr12	105035733	105035984	4.40	1.10E-08	CHST11	185081	SLC41A2	286613
chr6	11461633	11461909	3.26	1.13E-08	NEDD9	-228856	TMEM170B	-76740
chr2	161560800	161561088	3.96	1.24E-08	TANK	-432522	RBMS1	-210626
chr2	185228050	185228340	3.34	1.26E-08	ZNF804A	-234898		
chr15	81403881	81404173	3.35	1.30E-08	MESDC2	-121822	IL16	-85192
chr3	150945556	150945832	2.90	1.33E-08	GPR171	-24706	P2RY14	50536
chr12	26624401	26624711	2.98	1.38E-08	SSPN	276050	ITPR2	361575
chr16	4303781	4304079	3.05	1.38E-08	SRL	-11849	TFAP4	19071
chr2	158325910	158326225	3.43	1.38E-08	CYTIP	-25464	ACVR1C	159331
chr13	99911248	99911522	3.17	1.41E-08	GPR18	-545		
chr11	34675500	34675810	3.43	1.45E-08	PDHX	-262022	EHF	32987
chr8	142192433	142192745	3.01	1.45E-08	PTK2	-181257	SLC45A4	46084
chr7	141310557	141310833	4.81	1.46E-08	WEE2	-97458	AGK	59617
chr1	149969580	149969873	2.99	1.70E-08	MTMR11	-60936	OTUD7B	12959
chr4	3087073	3087349	3.90	1.93E-08	RGS12	-228663	HTT	10803
chr18	55297398	55297687	3.18	2.02E-08	NARS	-8366	ATP8B1	101496
chr3	188002398	188002646	3.58	2.04E-08	TPRG1	-887241	LPP	71801
chr4	130453925	130454201	5.64	2.05E-08	SCLT1	-439299		
chr11	82708249	82708631	3.07	2.10E-08	RAB30	74444	C11orf82	95703
chr12	113508013	113508349	3.18	2.18E-08	DTX1	12519	RASAL1	65840
chr14	96164115	96164459	2.55	2.18E-08	TCL1A	16246	GLRX5	162964
chr2	61034485	61034768	2.42	2.18E-08	REL	-74125	PAPOLG	51244
chr12	11989223	11989779	4.01	2.27E-08	BCL2L14	-234377	ETV6	186713
chr17	56446323	56446606	3.17	2.37E-08	SUPT4H1	-16902	RNF43	48466
chr9	140733682	140734061	3.03	2.43E-08	CACNA1B	-38369	EHMT1	220428

chr7	128603507	128603751	3.39	2.47E-08	IRF5	25635	TNPO3	91569
chr5	75885946	75886222	4.08	2.50E-08	F2RL2	33156	IQGAP2	186935
chr4	144298980	144299270	4.13	2.56E-08	SMARCA5	-135491	GAB1	41142
chr11	65239601	65239910	2.60	2.72E-08	SCYL1	-52792	FRMD8	85715
chr20	32156194	32156482	2.89	2.92E-08	C20orf134	-97966	CBFA2T2	78410
chr11	100551832	100552206	3.27	3.02E-08	FLJ32810	-6388		
chr3	142164885	142165161	4.48	3.17E-08	GK5	-220595	XRN1	1830
chr17	60730748	60731083	3.52	3.31E-08	MRC2	26154	MARCH10	154789
chr2	102239235	102239510	3.40	3.34E-08	RFX8	-148208	MAP4K4	-75115
chr13	50940985	50941333	3.34	3.39E-08	RNASEH2B	-542733	ST13	195005
chr3	107819846	107820164	3.49	3.43E-08	CD47	-10070	IFT57	121412
chr20	39657060	39657353	2.83	3.47E-08	TOP1	-255		
chr12	51751189	51751453	3.07	3.57E-08	CELA1	-10858	GALNT6	33879
chr5	65121780	65122028	3.35	3.66E-08	ERBB2IP	-100480	NLN	103819
chr21	44772532	44773056	3.16	3.83E-08	SIK1	74208	CRYAA	183653
chr8	142168247	142168491	2.83	3.87E-08	PTK2	-157037	SLC45A4	70304
chr2	222067794	222068216	3.55	3.92E-08	EPHA4	369005		
chr4	79671321	79671766	3.37	4.35E-08	BMP2K	-25988	ANXA3	198802
chr16	85449975	85450310	2.82	4.39E-08	KIAA0182	-196781	KIAA0513	388733
chr9	140221137	140221494	3.11	4.46E-08	NRARP	-24613	EXD3	96398
chr1	208351083	208351386	4.18	4.68E-08	CD34	-266552	PLXNA2	66430
chr1	212113305	212113554	3.07	5.32E-08	LPGAT1	-109316	INTS7	95454
chr3	177055507	177055856	2.92	5.42E-08	TBL1XR1	-140634		
chr5	10562498	10562878	2.61	5.44E-08	ROPN1L	120679	DAP	198699
chr16	85932527	85932920	2.87	5.67E-08	IRF8	-50		
chr18	60189950	60190269	3.06	5.67E-08	ZCCHC2	-548		
chr3	187691802	187692078	2.70	5.67E-08	LPP	-238781	BCL6	-228465
chr8	129083285	129083719	2.59	5.82E-08	MYC	335187		
chr2	191884967	191885324	4.89	6.35E-08	STAT1	-6170	STAT4	130779
chr9	33081266	33081542	2.64	6.67E-08	SMU1	-4690		
chr18	22932063	22932339	2.58	6.83E-08	ZNF521	13		
chr2	54865925	54866219	3.60	6.83E-08	EML6	-86077	DKFZp547I014	109713
chr8	136935355	136935621	4.62	7.26E-08	KHDRBS3	465772		
chr20	32829094	32829400	3.60	7.32E-08	EIF2S2	-129162	ASIP	-18924
chr1	17565902	17566193	2.19	7.49E-08	PADI3	-9545	PADI1	34427
chr9	139776192	139776500	2.76	7.49E-08	TRAF2	-4619		
chr21	43889425	43889744	3.31	7.65E-08	RSPH1	26816	UBASH3A	65566
chr3	15598020	15598324	3.56	7.82E-08	COLQ	-34914	HACL1	44958

chr20	50021347	50021623	3.06	7.88E-08	KCNG1	-381810	NFATC2	137773
chr13	100150814	100151183	3.11	8.08E-08	TM9SF2	-2729		
chr20	24404481	24404725	4.24	9.35E-08	GGTLC1	-435187	TMEM90B	-45232
chr3	138048567	138048843	2.90	9.77E-08	TXNDC6	23		
chr9	134604864	134605182	3.26	9.87E-08	RAPGEF1	-19794	MED27	350230
chr12	123893863	123894152	4.23	1.05E-07	SETD8	25304	RILPL2	27256
chr4	139111526	139112060	2.92	1.14E-07	PCDH18	-658164	SLC7A11	51710
chr4	185189151	185189640	3.02	1.14E-07	ENPP6	-50282	IRF2	206330
chr1	110786327	110786610	3.06	1.18E-07	RBM15	-95476	KCNC4	32404
chr11	34255653	34255965	2.97	1.22E-07	ABTB2	122993	NAT10	128698
chr11	70280402	70280696	2.79	1.32E-07	CTTN	35937	SHANK2	655293
chr2	24713502	24713885	3.13	1.33E-07	ITSN2	-130297	NCOA1	-93651
chr12	11912068	11912362	3.58	1.38E-07	BCL2L14	-311663	ETV6	109427
chr1	146555942	146556340	3.20	1.49E-07	PDZK1	-624101	PRKAB2	87988
chr17	16879687	16879977	3.29	1.50E-07	TNFRSF13B	-4430		
chr9	102821102	102821383	3.27	1.50E-07	ERP44	40087	STX17	152328
chr21	37661500	37661741	2.84	1.54E-07	MORC3	-30866	DOPEY2	124782
chr1	195164625	195164911	3.55	1.55E-07				
chr12	92797649	92797939	3.45	1.59E-07	PLEKHG7	-332471	BTG1	-258121
chr15	31558049	31558469	3.00	1.60E-07	TRPM1	-164335	KLF13	-60824
chr18	61089546	61090098	4.43	1.62E-07	VPS4B	-70		
chr20	52276816	52277092	4.10	1.62E-07	ZNF217	-77247	BCAS1	410350
chr17	28033450	28033780	2.90	1.63E-07	GIT1	-117005	SSH2	223403
chr12	24872449	24872696	3.77	1.64E-07	SOX5	-769936	BCAT1	229735
chr5	172483174	172483482	2.71	1.64E-07	C5orf41	-42		
chr8	134070312	134070731	2.49	1.66E-07	SLA	2081	TG	191317
chr18	56317338	56317687	3.06	1.72E-07	ALPK2	-21324	MALT1	-21105
chr20	38458360	38458789	3.00	1.72E-07	MAFB	859301	DHX35	867575
chr10	37717723	37717994	4.70	1.92E-07	ANKRD30A	303074	ZNF248	428627
chr19	12947122	12947403	2.28	1.92E-07	MAST1	-1996	RTBDN	-1033
chr7	22737969	22738312	2.90	1.96E-07	MGC87042	-198343	IL6	-28625
chr5	150439558	150439815	2.83	2.01E-07	TNIP1	21310	GPX3	39688
chr11	73231856	73232132	4.95	2.05E-07	PLEKHB1	-126600	RELT	144589
chr16	10023248	10023524	4.78	2.10E-07	USP7	-966045	GRIN2A	252877
chr2	135498022	135498298	3.66	2.18E-07	ACMSD	-98026	TMEM163	-21589
chr1	192595703	192596030	2.97	2.31E-07	RGS13	-9415	RGS1	51010
chr7	116651960	116652225	3.09	2.36E-07	ST7	58712	WNT2	311250
chr6	134557178	134557472	2.98	2.43E-07	SLC2A12	-183536	SGK1	81871

chr3	13129564	13129870	2.43	2.57E-07	IQSEC1	-15100	NUP210	332092
chr1	169662345	169662632	3.97	2.61E-07	SELP	-63112	SELL	18348
chr16	75109188	75109464	3.19	2.61E-07	LDHD	41339	ZNRF1	76411
chrX	39719541	39719841	3.33	2.61E-07	BCOR	237028		
chr18	2641685	2641984	2.50	2.65E-07	SMCHD1	-14051	NDC80	70325
chr2	69016772	69017107	2.89	2.65E-07	ARHGAP25	54972	BMP10	81709
chr18	60926651	60927142	3.38	2.69E-07	BCL2	59716	PHLPP1	544163
chr2	238795272	238795566	3.45	2.74E-07	UBE2F	-80281	RAMP1	27232
chr16	87942945	87943286	2.84	2.78E-07	SLC7A5	-40016	CA5A	26996
chr11	87070566	87070857	3.66	2.82E-07	TMEM135	321647	RAB38	837887
chr11	61348854	61349179	2.58	2.83E-07	SYT7	-719		
chr7	155137218	155137514	2.43	3.13E-07	EN2	-113458	INSIG1	47880
chr5	157288358	157288646	3.02	3.19E-07	CLINT1	-2334		
chr15	99191499	99191775	2.88	3.31E-07	IGF1R	-1124		
chr9	2241915	2242296	3.54	3.36E-07	VLDLR	-379687	SMARCA2	226764
chr16	28935262	28935601	2.84	3.44E-07	RABEP2	1100	ATP2A1	45623
chr6	157886272	157886548	3.90	3.44E-07	SNX9	-357884	ZDHHC14	83853
chr16	27442359	27442635	3.13	3.48E-07	IL21R	3918	GTF3C1	118754
chr11	75524223	75524490	4.07	3.55E-07	UVRAG	-1855		
chr7	137686939	137687203	2.83	3.67E-07	CREB3L2	-225		
chr20	43367517	43367793	2.99	3.68E-07	KCNK15	-6833	WISP2	23770
chr13	99358785	99359058	3.74	3.72E-07	STK24	-129526	SLC15A1	46007
chr20	42310643	42311045	4.38	3.72E-07	MYBL2	15135	GTSF1L	44798
chr3	175236307	175236641	3.91	3.73E-07	NAALADL2	659363		
chr2	8666941	8667319	2.98	3.74E-07	ID2	-154854		
chr18	54318303	54318684	3.23	3.76E-07	WDR7	-122		
chr1	11741117	11741611	2.76	3.77E-07	MAD2L2	10314	FBXO6	17214
chr7	130645914	130646192	2.38	3.78E-07	MKLN1	-366542	KLF14	-227193
chr16	87889867	87890143	2.81	3.84E-07	KLHDC4	-90463	SLC7A5	13095
chr2	64487988	64488299	2.47	4.11E-07	HSPC159	-193183	PELI1	-116539
chr12	47749984	47750421	2.86	4.12E-07	AMIGO2	-276469	RPAP3	349641
chr7	138802952	138803267	3.12	4.22E-07	TTC26	-15380	ZC3HAV1	-8645
chrX	64062392	64062722	3.43	4.27E-07	MTMR8	-447246	ZC4H2	133779
chr6	70591741	70592027	2.60	4.28E-07	COL19A1	15436	COL9A1	420902
chr12	24992222	24992597	2.76	4.47E-07	SOX5	-889773	BCAT1	109898
chr19	36231289	36231565	2.72	4.50E-07	TMEM149	2093	MLL4	22506
chr11	118754259	118754737	3.34	4.84E-07	CXCR5	-43		
chr15	99396471	99396748	2.08	5.05E-07	LOC145814	152275	IGF1R	203849

chr2	45955608	45955956	2.60	5.05E-07	EPAS1	-568781	PRKCE	76739
chr19	46018192	46018547	3.05	5.26E-07	VASP	7682	OPA3	69752
chr17	33390652	33390922	3.03	5.40E-07	RFFL	25507	LIG3	83249
chr1	8487639	8487985	2.59	5.47E-07	SLC45A1	103422	RERE	389887
chr16	4664859	4665171	2.66	5.55E-07	C16orf5	-76199	MGRN1	-9811
chr20	47541055	47541331	3.72	5.68E-07	CSE1L	-121645	ARFGEF2	2918
chr8	28232897	28233177	2.51	6.00E-07	ZNF395	10940	PNOC	58388
chr2	37862881	37863162	2.60	6.09E-07	CDC42EP3	36304	QPCT	291269
chr12	26241974	26242233	2.78	6.16E-07	BHLHE41	35899	RASSF8	130135
chr19	52068112	52068398	3.94	6.16E-07	SIGLEC6	-33191	ZNF175	-6276
chr5	106724806	106725117	2.58	6.52E-07	EFNA5	281634		
chr15	95128021	95128339	3.76	6.55E-07	MCTP2	286750		
chr15	74695266	74695625	2.81	6.70E-07	CYP11A1	-35365	SEMA7A	30853
chr1	209846654	209846971	3.98	6.85E-07	G0S2	-1857		
chr16	68504459	68504735	3.02	6.95E-07	KIAA1954	-68590	SMPD3	-22188
chr7	37826921	37827288	3.02	7.16E-07	TXNDC3	-61094	GPR141	47109
chr18	9066822	9067355	3.15	7.85E-07	NDUFV2	-35586	RAB12	457646
chr20	61530410	61530686	3.67	7.91E-07	TCFL5	-37433	DIDO1	38726
chr1	208005215	208005459	3.63	7.98E-07	CD34	79346	CD46	79935
chr19	39893279	39893555	2.61	8.18E-07	G0S24	-4081	ZFP36	-4070
chr10	112263920	112264196	3.21	8.27E-07	SMC3	-63391	DUSP5	6433
chr6	378171	378540	2.53	8.35E-07	IRF4	-13396	DUSP22	86255
chr3	16953971	16954247	3.40	8.45E-07	DAZL	-307103	PLCL2	-20473
chr12	71054719	71054968	3.49	8.80E-07	PTPRB	-23625	PTPRR	259740
chr6	135191795	135192075	3.58	9.30E-07	SGK1	-552739	ALDH8A1	79309
chr16	81771642	81771928	2.87	9.40E-07	PLCG2	-41145	CMIP	293010
chr18	10950923	10951238	3.29	9.41E-07	GNAL	-738055	C18orf58	-60391
chr19	47123034	47123274	2.70	9.79E-07	PTGIR	5200	CALM3	18642
chr3	177063420	177063735	2.83	9.94E-07	TBL1XR1	-148530		
chr9	140632642	140632952	2.12	1.01E-06	CACNA1B	-139444	EHMT1	119353
chr1	230259552	230259858	2.91	1.02E-06	GALNT2	56749	PGBD5	253662
chr2	223792334	223792594	4.11	1.02E-06	KCNE4	-124398	ACSL3	66732
chr1	240851163	240851407	3.98	1.03E-06	GREM2	-75823	RGS7	669193
chr15	70785210	70785486	2.48	1.04E-06	TLE3	-395092	UACA	270502
chr16	21518322	21518641	2.65	1.06E-06	DKFZp547E087	-19492	SLC7A5P1	13283
chr18	56806705	56807022	3.68	1.10E-06	SEC11C	-261		
chr12	40015274	40015590	4.33	1.12E-06	ABCD2	-1589		
chr19	47760038	47760331	2.69	1.19E-06	BBC3	-24162	PRR24	-17957

chr10	125151745	125152074	3.76	1.22E-06	GPR26	-273961	BUB3	238150
chr18	12314668	12314949	2.46	1.27E-06	TUBB6	6552	AFG3L2	62385
chr16	29611472	29611748	2.81	1.29E-06	IMAA	-4901		
chr3	171825356	171825636	2.59	1.38E-06	FNDC3B	68078	GHSR	340707
chr18	56197049	56197474	2.37	1.39E-06	ALPK2	98927	NEDD4L	485643
chr18	60191883	60192276	2.32	1.40E-06	PHLPP1	-190654	ZCCHC2	1422
chr19	50845917	50846195	3.33	1.44E-06	KCNC3	-13422	NAPSB	1949
chr12	72233304	72233719	2.38	1.46E-06	TBC1D15	25		
chr19	16438419	16438734	3.18	1.46E-06	KLF2	2926	EPS15L1	144185
chr21	34641136	34641412	3.17	1.47E-06	IFNAR1	-55940	IFNAR2	39043
chrX	23791737	23792019	3.21	1.48E-06	ACOT9	-30471	SAT1	-9397
chr5	17269382	17269632	2.87	1.50E-06	BASP1	51757		
chr3	53782768	53783064	3.40	1.54E-06	CHDH	97503	CACNA1D	253885
chr7	138794414	138794775	2.54	1.59E-06	ZC3HAV1	-130		
chr3	177069582	177069959	3.26	1.65E-06	TBL1XR1	-154723		
chr15	22899417	22899743	3.67	1.70E-06	CYFIP1	6896	NIPA2	134828
chr16	57073059	57073690	3.14	1.73E-06	NLRC5	49965	FAM192A	146601
chr11	84260343	84260619	3.01	1.75E-06				
chr17	26662425	26662823	3.29	1.84E-06	IFT20	-129	TNFAIP1	76
chr17	73110758	73111043	2.65	1.85E-06	ARMC7	4819	NT5C	16976
chr3	150983719	150984047	2.80	1.96E-06	GPR171	-62895	P2RY14	12347
chr3	71179992	71180268	3.53	1.97E-06	FOXP1	453010		
chr19	44258002	44258333	2.59	2.02E-06	C19orf61	974		
chr14	94442468	94442810	2.93	2.07E-06	OTUB2	-50085	ASB2	-18872
chr4	144280193	144280544	3.20	2.10E-06	SMARCA5	-154247	GAB1	22386
chr2	64955141	64955468	2.87	2.12E-06	SLC1A4	-261151	SERTAD2	-74259
chr12	75724023	75724299	2.29	2.14E-06	GLIPR1L1	-4302	CAPS2	-325
chr11	109870843	109871119	3.14	2.15E-06	ZC3H12C	-93106	C11orf87	578106
chr4	109254731	109255010	4.34	2.22E-06	RPL34	-286851	LEF1	-165293
chr17	79702466	79702750	2.94	2.25E-06	GCGR	-59418	SLC25A10	23237
chr4	124897468	124897776	2.76	2.27E-06	SPRY1	579666		
chr9	130673558	130673829	2.91	2.28E-06	ST6GALNAC6	-11823	ST6GALNAC4	5611
chr19	14317584	14317860	1.95	2.29E-06	LPHN1	-725		
chr8	135701345	135701647	2.57	2.32E-06	ZFAT	23785		
chr9	37382689	37382998	2.76	2.32E-06	GRHPR	-39863	ZCCHC7	262375
chr7	148204221	148204465	2.66	2.41E-06	CUL1	-191590	NM_001126313	72704
chr12	24894253	24894532	3.80	2.43E-06	SOX5	-791756	BCAT1	207915
chr7	114706143	114706428	2.69	2.43E-06	MDFIC	144077	TFEC	964512

chr22	36635513	36635843	2.72	2.44E-06	APOL2	322		
chr2	25640733	25641023	2.49	2.50E-06	DNMT3A	-76104	DTNB	255625
chr11	75946831	75947122	2.82	2.58E-06	WNT11	-29403	PRKRIR	144903
chr7	105449664	105449963	2.54	2.59E-06	FLJ23834	-153843	RINT1	277282
chr7	55584896	55585162	2.55	2.65E-06	VOPP1	55171	LANCL2	151888
chr13	51168576	51168852	2.53	2.78E-06	RNASEH2B	-315178	ST13	422560
chr2	74413096	74413378	2.67	2.87E-06	MTHFD2	-12453	MOBKL1B	-7242
chr15	91293461	91293737	3.16	2.90E-06	FURIN	-118286	BLM	33020
chr1	147806576	147806895	3.15	2.99E-06	NBPF11	-196648	PPIAL4A	148683
chr16	87808350	87808658	2.82	3.04E-06	KLHDC4	-8962	SLC7A5	94596
chr19	39894462	39894929	2.47	3.04E-06	G0S24	-2802	ZFP36	-2791
chr13	66712643	66713107	2.94	3.05E-06				
chr20	30945636	30946103	3.43	3.07E-06	ASXL1	-283		
chr2	225893374	225893659	2.85	3.08E-06	CUL3	-443407	DOCK10	13813
chr19	45256567	45256951	3.07	3.09E-06	CBLC	-24367	BCL3	4781
chr1	16291663	16292120	2.49	3.28E-06	ZBTB17	10735	SPEN	117533
chr11	128338904	128339461	2.60	3.34E-06	ETS1	118270		
chr5	95157713	95157989	2.30	3.53E-06	GLRX	726		
chr2	111966723	111967023	2.75	3.54E-06	BCL2L11	88382	ANAPC1	674868
chr10	37718249	37718541	4.47	3.56E-06	ANKRD30A	303610	ZNF248	428091
chr1	173387388	173387635	2.96	3.57E-06	TNFSF4	-211041	PRDX6	-58974
chr11	109855620	109855896	3.20	3.64E-06	ZC3H12C	-108329	C11orf87	562883
chr17	38755560	38755868	2.46	3.78E-06	CCR7	-33990	SMARCE1	48389
chr2	8442152	8442509	2.37	3.89E-06	ID2	-379653		
chr8	126163982	126164226	3.96	3.89E-06	TRIB1	-278459	NSMCE2	60021
chr2	96811819	96812099	2.28	3.95E-06	DUSP2	-780		
chr5	124271548	124271914	2.20	4.06E-06	ZNF608	-190866		
chr12	105368484	105368758	2.53	4.18E-06	SLC41A2	-46149	ALDH1L2	109720
chr1	67223802	67224193	2.94	4.22E-06	INSL5	42941	SGIP1	224173
chr3	101510795	101511159	3.18	4.22E-06	NFKBIZ	-57381	FAM55C	12948
chr1	220267237	220267513	2.53	4.25E-06	BPNT1	-4184	IARS2	-80
chr9	123693846	123694255	2.43	4.28E-06	TRAF1	-4878		
chr13	28054636	28054887	3.12	4.33E-06	MTIF3	-30051	LNX2	139958
chr11	68138220	68138660	2.39	4.36E-06	SAPS3	-89746	LRP5	58332
chr15	85861553	85861918	3.08	4.54E-06	AKAP13	-62135	PDE8A	336531
chr13	99881036	99881293	2.75	4.63E-06	UBAC2	28486	GPR18	29675
chr4	14179690	14179977	2.96	4.63E-06	CPEB2	-825688	BOD1L	-550506
chr1	171221689	171221999	2.70	4.70E-06	FMO4	-61642	FMO1	4181

chr13	34253320	34253692	2.52	4.78E-06	STARD13	-393605	RFC3	-138700
chr22	37618773	37619049	2.61	4.86E-06	SSTR3	-10558	RAC2	21394
chrX	71526720	71526996	3.85	4.87E-06	CITED1	-1094		
chr4	123553887	123554159	3.44	5.02E-06	BBS12	-99834	IL21	-11812
chr15	80263453	80263714	2.77	5.14E-06	BCL2A1	59		
chr20	46115873	46116335	2.90	5.15E-06	ZMYND8	-130630	NCOA3	-14553
chr1	161509925	161510191	2.84	5.21E-06	FCGR3A	9760	HSPA6	16022
chr21	45510290	45510607	2.49	5.29E-06	PWP2	-16759	TRAPPC10	78243
chr9	114774712	114775027	2.55	5.77E-06	UGCG	115664	SUSD1	162686
chr1	206955807	206956197	2.50	5.84E-06	IL19	-16213	IL10	-10163
chr6	52362369	52362736	2.32	5.95E-06	EFHC1	77559	TRAM2	79309
chr4	86478468	86478867	2.76	5.97E-06	ARHGAP24	82384	MAPK10	797129
chr12	40502051	40502420	3.10	6.05E-06	SLC2A13	-2575		
chr12	48201891	48202167	2.03	6.13E-06	HDAC7	11734	SLC48A1	35062
chr1	235243072	235243348	3.55	6.37E-06	IRF2BP2	-497939	TOMM20	49046
chr11	120215121	120215397	2.67	6.54E-06	GRIK4	-315719	ARHGEF12	7313
chr21	43299041	43299324	2.17	6.57E-06	PRDM15	408		
chr3	171818879	171819292	2.55	6.57E-06	FNDC3B	61668	GHSR	347117
chr7	28194590	28194906	1.91	6.67E-06	JAZF1	25689	TAX1BP1	415010
chr17	76333575	76333920	2.41	6.93E-06	SOCS3	22410	LOC283999	106357
chr15	85523509	85523819	2.04	7.17E-06	PDE8A	-1541		
chr14	87533455	87533751	3.34	7.71E-06	GALC	926304		
chr19	14629452	14629747	2.33	8.14E-06	DNAJB1	-399		
chr3	30681482	30681753	3.02	8.16E-06	TGFBR2	33624	GADL1	254535
chr2	238777679	238777923	2.94	8.67E-06	UBE2F	-97899	RAMP1	9614
chr2	172176006	172176303	2.61	8.78E-06	DCAF17	-114704	TLK1	-88331
chr3	13125425	13125788	2.16	8.86E-06	IQSEC1	-10990	NUP210	336202
chr20	24405980	24406421	3.35	9.14E-06	GGTLC1	-436785	TMEM90B	-43634
chr16	27210196	27210440	3.11	9.16E-06	NSMCE1	69795		
chr17	12334654	12334938	3.28	9.41E-06	MYOCD	-234411	MAP2K4	410661
chr3	18464860	18465143	2.23	9.42E-06	TBC1D5	-680762	SATB1	15250
chr3	143747664	143747940	2.40	9.42E-06	C3orf58	57162		
chr6	116340730	116340978	3.06	9.53E-06	FRK	41067		
chr12	108962635	108962881	2.87	9.55E-06	ISCU	6464	TMEM119	29136
chr4	55181759	55182055	3.73	1.02E-05	KIT	-342188	PDGFRA	86643
chr12	11899031	11899391	2.74	1.05E-05	BCL2L14	-324667	ETV6	96423
chr11	57192237	57192541	2.37	1.06E-05	PRG3	-43766	SLC43A3	2664
chr13	42951889	42952204	2.79	1.06E-05	TNFSF11	-196244	AKAP11	105758

chr11	65262035	65262279	3.38	1.07E-05	SCYL1	-30391	FRMD8	108116
chr15	52240926	52241213	2.61	1.10E-05	LEO1	22888	TMOD3	119181
chr16	31885779	31886055	2.39	1.11E-05	ZNF267	838		
chr19	18485205	18485470	2.42	1.12E-05	GDF15	-11630	PGPEP1	33930
chr9	114790934	114791345	2.30	1.15E-05	UGCG	131934	SUSD1	146416
chr7	125301877	125302134	2.86	1.20E-05	POT1	-731969		
chr15	99143155	99143431	4.12	1.24E-05	IGF1R	-49468		
chr1	179050853	179051181	2.19	1.25E-05	TOR3A	-95		
chr15	81316109	81316362	2.87	1.28E-05	IL16	-172983	MESDC2	-34031
chr19	50528496	50529123	3.47	1.28E-05	ZNF473	-402	VRK3	-5
chr22	37838564	37838935	3.66	1.28E-05	ELFN2	-15245	MFNG	43635
chr11	65192665	65192939	2.25	1.29E-05	SCYL1	-99746	FRMD8	38761
chr7	101855189	101855443	2.50	1.32E-05	SH2B2	-73089	CUX1	394434
chr1	199010627	199010996	2.74	1.34E-05	NR5A2	-985958	PTPRC	402675
chr12	93561641	93561887	3.01	1.34E-05	EEA1	-238657	NUDT4	-209937
chr1	207103751	207104035	2.21	1.36E-05	FAIM3	-8515	PIGR	15918
chr17	76272592	76272846	2.33	1.36E-05	LOC283999	45328	SOCS3	83439
chr19	5974904	5975373	2.33	1.41E-05	RANBP3	3181	CAPS	60946
chr18	52901917	52902193	2.28	1.44E-05	TCF4	353805	RAB27B	406215
chr11	9738359	9738638	2.65	1.63E-05	SWAP70	52871	SBF2	577255
chr11	18790023	18790316	2.94	1.64E-05	PTPN5	23219	TMEM86A	69819
chr19	54975277	54975785	2.06	1.64E-05	LENG9	-637		
chr2	170998455	170998728	2.12	1.65E-05	MYO3B	-36063	UBR3	314574
chr4	87919230	87919633	2.43	1.68E-05	SLC10A6	-149016	AFF1	-8721
chr4	170332012	170332259	2.66	1.68E-05	SH3RF1	-139887	NEK1	201478
chr17	16891409	16891783	2.63	1.70E-05	KIAA0864	-176378	TNFRSF13B	-16194
chr5	4879857	4880133	2.65	1.73E-05	ADAMTS16	-260448		
chr21	35392317	35392611	2.33	1.75E-05	SLC5A3	-53359	ITSN1	377680
chr3	50628266	50628572	3.05	1.96E-05	CISH	20843	HEMK1	21510
chr3	185902925	185903293	2.58	1.97E-05	ETV5	-76208	DGKG	176914
chr6	11730876	11731244	2.29	1.97E-05	C6orf105	48220	TMEM170B	192549
chr12	48591845	48592136	2.22	1.99E-05	OR10AD1	5084	C12orf68	14625
chr17	37914201	37914546	2.46	1.99E-05	GRB7	20187	IKZF3	106067
chr9	92761994	92762297	2.99	1.99E-05	GADD45G	542219	DIRAS2	642962
chr17	48226225	48226501	2.33	2.00E-05	PPP1R9B	1514	PDK2	53667
chr22	27006351	27006603	2.86	2.00E-05	TPST2	-45107	CRYBB1	7514
chr2	24299098	24299490	3.14	2.04E-05	SF3B14	20		
chr1	150535239	150535588	4.93	2.07E-05	ADAMTSL4	13516	MCL1	16722

chr13	100028387	100028660	2.23	2.10E-05	TM9SF2	-125204	GPR183	-68775
chrX	41301603	41301867	2.34	2.10E-05	NYX	-4978		
chr15	85855701	85855961	2.88	2.12E-05	AKAP13	-68040	PDE8A	330626
chr1	158985343	158985740	2.40	2.15E-05	IFI16	5860	AIM2	61105
chr15	74677434	74677678	2.26	2.17E-05	CYP11A1	-17475	SEMA7A	48743
chr18	55887673	55887974	2.86	2.21E-05	NEDD4L	176205	ALPK2	408365
chr2	240322602	240322978	1.98	2.21E-05	HDAC4	-147		
chr17	78237237	78237527	2.01	2.29E-05	RNF213	-76344	SLC26A11	43145
chr12	96883149	96883459	2.47	2.31E-05	NEDD1	-417697	PCTK2	-89081
chr4	124246415	124246725	2.86	2.33E-05	SPRY1	-71386	SPATA5	402345
chr11	33964650	33964954	2.97	2.35E-05	CAPRIN1	-108428	LMO2	-50966
chr1	231748526	231748802	2.44	2.43E-05	DISC1	-13897	TSNAX	84265
chr8	81260150	81260410	2.62	2.44E-05	TPD52	-267270	ZBTB10	-138168
chr14	21776900	21777176	2.27	2.54E-05	RPGRIP1	20902	SUPT16H	75387
chr19	3721399	3721692	1.98	2.55E-05	PIP5K1C	-21101	TJP3	-6828
chr7	73157287	73157577	2.03	2.59E-05	ABHD11	-4242		
chr16	87958135	87958473	2.50	2.62E-05	SLC7A5	-55204	CA5A	11808
chr22	35767910	35768160	2.91	2.64E-05	HMOX1	-9052	TOM1	72238
chr14	70163614	70163890	4.21	2.71E-05	SFRS5	-70082	KIAA0247	85442
chr22	29188172	29188448	2.42	2.80E-05	XBP1	8250	HSCB	50267
chr1	212781899	212782205	2.47	2.85E-05	ATF3	43355	BATF3	91275
chr20	16555414	16555690	1.92	2.89E-05	KIF16B	-1474		
chr2	179886051	179886335	2.64	2.90E-05	CCDC141	-136374	SESTD1	243157
chr14	65770449	65771019	2.77	2.91E-05	MAX	-201507	FUT8	-108801
chr12	1663484	1663955	2.10	2.93E-05	FBXL14	39611	ERC1	563316
chr11	76385000	76385285	2.16	3.06E-05	LRRC32	-4099		
chr12	11883824	11884077	2.88	3.06E-05	BCL2L14	-339927	ETV6	81163
chr3	188107747	188108070	2.20	3.09E-05	TPRG1	-781854	LPP	177188
chr3	195906437	195907150	2.48	3.09E-05	TFRC	-97762	ZDHHC19	31506
chr17	73031078	73031378	2.45	3.12E-05	ATP5H	11846	ICT1	22448
chr13	69162845	69163108	2.39	3.13E-05				
chr13	100153431	100153786	2.92	3.14E-05	TM9SF2	-119		
chr15	85524444	85524765	2.10	3.17E-05	PDE8A	-600		
chr19	56116663	56117003	1.90	3.19E-05	ZNF524	5103	ZNF784	19108
chr16	11082260	11082504	3.07	3.25E-05	CIITA	111327	SOCS1	267657
chrX	24531606	24531880	4.22	3.27E-05	PDK3	48399	PCYT1B	133712
chr11	18415887	18416168	2.53	3.32E-05	LDHA	92		
chr7	105925647	105926006	2.13	3.37E-05	NAMPT	-189		

chr19	45542568	45542844	2.44	3.38E-05	SFRS16	408		
chr3	61623108	61623431	2.42	3.40E-05	PTPRG	76027	FEZF2	735920
chr8	74903416	74903715	2.69	3.40E-05	LY96	-21		
chr20	47463457	47463767	3.00	3.42E-05	ARFGEF2	-74663	PREX1	-19192
chr19	48972031	48972342	2.21	3.44E-05	CYTH2	-278		
chr21	42734285	42734566	2.97	3.44E-05	MX2	476		
chr22	24407502	24407957	2.33	3.45E-05	CABIN1	-35		
chr17	76254665	76254945	3.46	3.47E-05	LOC283999	27414	SOCS3	101353
chr18	72728042	72728302	3.28	3.47E-05	ZADH2	193109	ZNF407	385249
chr1	150459561	150459894	2.89	3.50E-05	TARS2	-192		
chr4	10724701	10724983	2.81	3.69E-05	CLNK	-38456	HS3ST1	705695
chr8	103818542	103818832	2.27	3.70E-05	KLF10	-150704	AZIN1	57710
chr12	96972356	96972632	3.39	3.72E-05	NEDD1	-328507	PCTK2	-178271
chr3	183299565	183299899	2.84	3.75E-05	KLHL24	-53679	KLHL6	-26233
chr9	38319073	38319408	2.28	3.81E-05	SHB	-250031	ALDH1B1	-73461
chr15	63796576	63796886	2.38	3.82E-05	USP3	-79		
chr19	52074238	52074616	2.49	3.82E-05	ZNF175	-104		
chr6	27560449	27560754	2.79	3.84E-05	ZNF184	-119705	HIST1H2BL	215107
chr15	91191854	91192142	2.64	3.97E-05	BLM	-68581	CRTC3	118800
chr19	42633490	42634077	5.05	3.97E-05	POU2F2	2846	ZNF574	53494
chr10	31389998	31390279	3.48	3.98E-05	ZEB1	-217962	ZNF438	-69273
chr2	196426087	196426331	2.72	4.01E-05	SLC39A10	-95323		
chr22	49776313	49776787	2.46	4.16E-05	BRD1	441902	FAM19A5	891262
chr9	131902270	131902592	2.26	4.20E-05	METTL11A	-486004	PPP2R4	29187
chr12	26676709	26676971	2.55	4.24E-05	ITPR2	309291	SSPN	328334
chr11	67203880	67204257	3.07	4.27E-05	PTPRCAP	1084	RPS6KB2	8134
chr13	41350894	41351170	2.25	4.41E-05	SLC25A15	-12515	MRPS31	-5685
chr17	29035763	29036089	1.82	4.52E-05	LRRC37B2	100478	CRLF3	115852
chr21	30257362	30257858	2.61	4.55E-05	N6AMT1	83		
chr7	89872280	89872526	2.88	4.56E-05	C7orf63	-2085		
chr2	237478167	237478512	2.63	4.58E-05	CXCR7	-40		
chr1	237899998	237900242	4.18	4.65E-05	ZP4	153815	RYR2	694418
chr1	149982559	149982835	2.19	4.91E-05	OTUD7B	-11		
chr19	54974914	54975238	1.88	4.96E-05	LENG9	-182		
chr17	1620792	1621068	2.55	5.03E-05	PRPF8	-32754	SERPINF2	-25200
chr9	139796926	139797244	2.17	5.10E-05	TRAF2	16120	FBXW5	42088
chr1	157536414	157536704	2.48	5.22E-05	FCRL5	-14249	FCRL4	31311
chr5	133890151	133890417	2.24	5.45E-05	PHF15	28486	SAR1B	78243

chr15	91330079	91330385	2.58	5.50E-05	FURIN	-81653	BLM	69653
chr22	34317106	34317382	2.49	5.51E-05	LARGE	-828		
chr15	81594207	81594490	2.18	5.59E-05	STARD5	22175	IL16	105130
chr19	42625835	42626168	2.05	5.60E-05	POU2F2	10628	ZNF574	45712
chr11	128627789	128628059	2.58	5.63E-05	FLI1	64111	KCNJ1	84439
chr12	14547365	14547649	2.18	5.63E-05	ATF7IP	28896	PLBD1	173284
chr3	15847302	15847580	2.79	5.63E-05	ANKRD28	53612	BTD	204186
chr14	61969946	61970314	2.55	5.64E-05	HIF1A	-191989	PRKCH	181615
chr1	185308407	185308666	2.56	5.86E-05	HMCN1	-395146	IVNS1ABP	-22076
chr14	95731875	95732253	2.59	5.97E-05	DICER1	-123979	CLMN	54181
chr19	38754267	38754511	3.57	6.08E-05	SPINT2	-813		
chr11	125773006	125773255	2.35	6.09E-05	DDX25	-1141	PUS3	-15
chr5	17257900	17258170	2.43	6.28E-05	BASP1	40285		
chr2	25502153	25502444	1.95	6.33E-05	POMC	-110740	DNMT3A	62475
chr3	36953966	36954338	2.47	6.35E-05	TRANK1	-51741	EPM2AIP1	80643
chr1	145455343	145455679	2.17	6.42E-05	RBM8A	-52127	TXNIP	17049
chr13	100089167	100089411	2.01	6.48E-05	GPR183	-129540	TM9SF2	-64439
chr1	87264630	87264888	2.45	6.49E-05	SH3GLB1	94502	SEP15	115348
chr3	169772998	169773398	2.54	6.60E-05	GPR160	17463	PHC3	126339
chr9	101892400	101892693	2.46	6.60E-05	TGFBR1	25135	ALG2	91699
chr2	17994966	17995316	2.63	6.66E-05	MSGN1	-2645		
chr8	66989984	66990236	3.37	6.77E-05	TRIM55	-49168	DNAJC5B	56319
chr2	192482326	192482656	2.66	6.91E-05	OBFC2A	-60370	MYO1B	372384
chr3	186761900	186762400	2.32	6.91E-05	ST6GAL1	22485	RPL39L	95113
chrX	150565389	150565760	2.09	6.91E-05	VMA21	-130		
chr13	48760494	48760738	3.55	6.93E-05	MED4	-91376	ITM2B	-46658
chr16	89126721	89126977	2.25	6.93E-05	CBFA2T3	-83448	ACSF3	-33405
chr12	102155243	102155487	3.69	6.94E-05	SYCP3	-22118	GNPTAB	69267
chr17	36857204	36857452	2.90	7.01E-05	MLLT6	-4545		
chr18	56325450	56325786	4.12	7.09E-05	ALPK2	-29429	MALT1	-13000
chr15	63773328	63773590	3.21	7.10E-05	CA12	-99384	USP3	-23351
chr20	48552634	48552921	1.82	7.13E-05	RNF114	-136		
chr3	170074062	170074331	2.11	7.27E-05	SKIL	-1276		
chr13	115079733	115080074	2.22	7.40E-05	ZNF828	-61		
chr1	235114579	235114867	2.19	7.48E-05	IRF2BP2	-369452	TOMM20	177533
chr3	176914555	176914935	2.28	7.50E-05	TBL1XR1	303		
chr3	127609473	127609728	3.19	7.55E-05	MGLL	-67550	KBTBD12	-32301
chr3	150863869	150864207	2.17	7.71E-05	GPR171	56950	MED12L	59453

chr3	150484135	150484538	2.61	7.83E-05	SIAH2	-3074		
chr2	31479025	31479415	2.33	7.85E-05	EHD3	22340	XDH	158391
chr12	95114990	95115266	2.50	7.89E-05	TMCC3	-70804	NDUFA12	282383
chr2	136891619	136892057	2.50	7.89E-05	THSD7B	-856624	CXCR4	-16113
chr18	55908523	55908799	3.12	7.95E-05	NEDD4L	197042	ALPK2	387528
chr6	134568664	134568942	2.64	7.98E-05	SLC2A12	-195014	SGK1	70393
chr3	5020288	5020574	2.57	7.99E-05	BHLHE40	-666		
chrX	53741741	53742047	2.69	8.06E-05	HUWE1	-28221	PHF8	328713
chr2	97303993	97304303	2.76	9.26E-05	FER1L5	-4426		
chr18	56613474	56613723	2.07	9.33E-05	SEC11C	-193526	ZNF532	83538
chr3	150811780	150812069	2.11	9.33E-05	MED12L	7340	GPR171	109063
chr13	22009997	22010341	2.59	9.49E-05	SKA3	-259459	ZDHHC20	23254
chr3	159706361	159706770	2.52	9.49E-05	IL12A	-63		
chr9	137283737	137284078	2.25	9.67E-05	COL5A1	-249744	RXRA	65592
chr5	88030121	88030378	2.84	9.68E-05	TMEM161B	-465585	MEF2C	149052
chr11	82904586	82904874	2.22	9.71E-05	PCF11	36593	CCDC90B	92647
chr9	33988554	33988804	2.95	9.75E-05	DCAF12	138092	UBE2R2	171497
chr4	71544158	71544434	2.46	1.01E-04	IGJ	-11948	UTP3	-9900
chr18	54305733	54306204	3.02	1.01E-04	TXNL1	301		
chr13	50265515	50265791	2.04	1.02E-04	EBPL	-30		
chr22	40988805	40989069	2.50	1.03E-04	MKL1	43753	SGSM3	222342
chr19	55896879	55897574	2.52	1.05E-04	RPL28	-73		
chr17	57915137	57915447	2.46	1.05E-04	TUBD1	55004	TMEM49	130429
chr3	37949304	37949726	3.10	1.06E-04	VILL	-85563	CTDSPL	45846
chr2	10054829	10055094	2.51	1.06E-04	GRHL1	-36830	TAF1B	71391
chr17	35767201	35767477	2.15	1.08E-04	ACACA	-437	TADA2A	27
chr6	53096537	53096788	3.13	1.08E-04	GCM1	-83039	ELOVL5	117279
chr3	136471129	136471514	2.53	1.10E-04	STAG1	-77		
chr7	138818353	138818636	1.97	1.12E-04	TTC26	5		
chr11	76758649	76758976	2.25	1.12E-04	CAPN5	-19179	B3GNT6	13378
chr14	35873004	35873290	2.06	1.12E-04	NFKBIA	813		
chr11	43902142	43902393	1.87	1.14E-04	ALKBH3	-89		
chr1	20452609	20452886	2.41	1.14E-04	PLA2G2F	-13075	PLA2G2D	-6740
chr22	38857585	38857876	2.09	1.15E-04	KCNJ4	-17698	KDELR3	-6352
chr18	42339336	42339614	2.40	1.16E-04	SLC14A2	-855291	SETBP1	78612
chr2	217087847	217088109	2.72	1.16E-04	XRCC5	113958	MARCH4	148772
chr18	56431567	56431843	2.23	1.19E-04	ZNF532	-98356	MALT1	93087
chr12	92458870	92459162	2.13	1.20E-04	DCN	-885657	BTG1	80657

chr15	101667708	101667984	2.24	1.20E-04	CHSY1	124280	LRRK1	208386
chr15	31645543	31645965	2.21	1.21E-04	KLF13	26671	OTUD7A	301788
chr11	76385698	76386048	2.29	1.21E-04	LRRC32	-4829		
chr3	176919502	176919800	2.22	1.21E-04	TBL1XR1	-4603		
chr19	47791700	47791955	2.08	1.22E-04	C5AR1	-21276	PRR24	13686
chr19	52207430	52207738	2.21	1.23E-04	SIGLEC14	-57452	HAS1	19637
chr1	178575179	178575489	2.28	1.23E-04	RALGPS2	-118966	C1orf49	93122
chr11	85925832	85926144	3.23	1.25E-04	PICALM	-145880	EED	-29827
chr15	80269840	80270102	2.36	1.25E-04	ZFAND6	-82050	BCL2A1	-6328
chr2	38949925	38950169	2.40	1.26E-04	SFRS7	28589	GALM	56995
chr7	142960429	142960725	2.06	1.28E-04	GSTK1	55		
chr8	116439901	116440250	2.15	1.29E-04	TRPS1	241152		
chr14	91830492	91830838	2.20	1.30E-04	GPR68	-110441	CCDC88C	53468
chr2	20251709	20251966	2.08	1.31E-04	LAPTM4A	-49		
chr16	84122355	84122683	1.95	1.32E-04	SLC38A8	-46757	MBTPS1	27998
chr14	75959850	75960215	2.22	1.34E-04	BATF	-28751	JDP2	61196
chr3	156892542	156892923	3.89	1.34E-04	PTX3	-261847	CCNL1	-14251
chr17	36858317	36858658	2.18	1.35E-04	MLLT6	-3385		
chr1	224390950	224391199	2.80	1.36E-04	DEGS1	20147	NVL	126797
chr11	123941665	123942007	3.49	1.39E-04	X64983	-114515	OR10G7	-32128
chr17	74260741	74261093	2.15	1.40E-04	RNF157	-24527	QRICH2	42844
chr19	41035010	41035329	1.96	1.43E-04	SHKBP1	-47587	SPTBN4	62044
chr8	19674610	19675019	2.54	1.43E-04	INTS10	-103		
chr8	30601796	30602072	2.18	1.45E-04	UBXN8	244		
chr9	137282481	137282798	2.66	1.45E-04	COL5A1	-251012	RXRA	64324
chr2	98643539	98643827	2.30	1.47E-04	CNGA3	-318935	TMEM131	-31329
chr11	120796705	120796994	2.79	1.49E-04	TBCEL	-97963	GRIK4	265872
chr2	196424302	196424676	2.33	1.49E-04	SLC39A10	-97043		
chr3	19988397	19988700	1.99	1.54E-04	RAB5A	-23		
chr1	154973227	154973537	1.92	1.56E-04	ZBTB7B	-1730		
chr17	76352510	76352802	2.11	1.56E-04	SOCS3	3502	LOC283999	125265
chr16	23706298	23706628	2.49	1.57E-04	PLK1	16262	ERN2	18358
chr2	20565074	20565422	2.82	1.58E-04	RHOB	-81587	PUM2	-38109
chr2	25642595	25642871	2.40	1.61E-04	DNMT3A	-77959	DTNB	253770
chr1	145382671	145383082	2.97	1.61E-04	HFE2	-30314	NBPF10	89506
chr20	37501713	37502053	2.28	1.63E-04	FAM83D	-53072	PPP1R16B	67535
chr2	191878853	191879311	2.25	1.69E-04	STAT1	-106		
chr9	114794447	114794733	2.45	1.70E-04	UGCG	135384	SUSD1	142966

chr15	74286735	74287113	1.97	1.77E-04	STOML1	-2289	PML	-90
chr8	107072665	107072958	2.50	1.81E-04	OXR1	-209661	ZFPM2	741665
chr4	40187375	40187651	2.35	1.83E-04	ACOT7L	-128694	RHOH	-11014
chr18	56448828	56449104	2.34	1.85E-04	ZNF532	-81095	MALT1	110348
chr15	57591995	57592275	2.36	1.86E-04	CGNL1	-76570	TCF12	381302
chr5	139017055	139017338	2.11	1.89E-04	CXXC5	-11104	UBE2D2	76446
chr9	124412539	124412815	2.94	1.90E-04	DAB2IP	83278	TTLL11	443208
chr19	49866797	49867151	1.99	1.92E-04	TEAD2	-1260	DKKL1	-68
chr19	7198061	7198314	1.79	1.93E-04	INSR	95823	ZNF557	128717
chr1	16292543	16292887	2.22	1.94E-04	ZBTB17	9912	SPEN	118356
chr1	145507294	145507677	2.24	1.95E-04	RBM8A	-152		
chr7	138803748	138804022	1.83	1.96E-04	TTC26	-14605	ZC3HAV1	-9420
chr5	115912778	115913054	2.88	1.99E-04	SEMA6A	-2365		
chr20	37678743	37679024	2.50	2.01E-04	DHX35	87884		
chr3	186251911	186252187	2.34	2.03E-04	DGKG	-172026	CRYGS	10118
chr9	140117878	140118192	1.89	2.03E-04	RNF208	-2260		
chr7	22925574	22925891	1.94	2.03E-04	TOMM7	-63312	FAM126A	128037
chr17	19659170	19659463	3.03	2.04E-04	ALDH3A1	-10545	ULK2	111922
chr9	37197143	37197486	2.02	2.07E-04	GRHPR	-225392	ZCCHC7	76846
chr11	117686050	117686466	2.21	2.09E-04	DSCAML1	-18282	FXYD2	9201
chr8	9008062	9008338	1.97	2.10E-04	PPP1R3B	20		
chr4	40517379	40517655	2.29	2.11E-04	RBM47	114366	CHRNA9	180048
chr5	123370612	123370949	2.34	2.12E-04	CSNK1G3	522988	ZNF608	710084
chr13	109814553	109814879	2.30	2.12E-04	MYO16	566216	IRS2	624198
chr3	186759656	186760103	2.36	2.13E-04	ST6GAL1	20215	RPL39L	97383
chr3	72213543	72213825	2.01	2.14E-04	PROK2	-379327	RYBP	282090
chr3	150317403	150317670	2.67	2.22E-04	SELT	-3529		
chr11	73702386	73703003	2.27	2.22E-04	UCP2	-8806	UCP3	17587
chr14	60794170	60794565	2.12	2.24E-04	PPM1A	81898	C14orf39	158396
chrX	53122380	53122658	2.25	2.24E-04	TSPYL2	10970	KDM5C	132085
chr22	29138452	29138970	2.30	2.26E-04	CHEK2	-889	HSCB	668
chr1	145395448	145395742	2.36	2.27E-04	HFE2	-17596	NBPF10	102224
chr11	60677683	60678045	2.02	2.33E-04	PRPF19	-3803	TMEM109	-3507
chr2	24800377	24800673	2.92	2.34E-04	ITSN2	-217128	NCOA1	-6820
chr11	47236379	47236674	2.05	2.35E-04	DDB2	34		
chr1	53144830	53145128	1.93	2.44E-04	C1orf163	19059	FAM159A	45913
chr3	112532809	112533105	2.84	2.44E-04	CCDC80	-172980	CD200R1L	31840
chr7	70226778	70227071	2.65	2.44E-04	WBSCR17	-370864		

chr19	50400130	50400572	2.04	2.49E-04	IL4I1	-204				
chr4	37140009	37140271	3.09	2.49E-04	RELL1	547859	DTHD1	856896		
chr5	126112279	126112610	2.14	2.49E-04	LMNB1	-388				
chr16	21566123	21566445	2.74	2.51E-04	SLC7A5P1	-34519	IGSF6	97688		
chr19	5827874	5828252	1.62	2.51E-04	NRTN	4245	FUT6	11679		
chr2	8692637	8693051	2.36	2.52E-04	ID2	-129140				
chr5	110087960	110088303	2.72	2.52E-04	TSLP	-319258	SLC25A46	13378		
chr3	9210046	9210290	2.39	2.61E-04	RAD18	-205022	SRGAP3	81143		
chr7	155534537	155534838	1.97	2.62E-04	SHH	70279	RBM33	97485		
chr2	55459577	55459933	2.39	2.65E-04	C2orf63	-306	RPS27A	690		
chr2	17935180	17935463	1.81	2.68E-04	SMC6	-226	GEN1	145		
chr19	44269609	44269950	2.14	2.68E-04	C19orf61	-10638	KCNN4	15629		
chr13	50705316	50705597	2.12	2.68E-04	ST13	-40697	KCNRG	116067		
chr10	104153742	104154018	2.04	2.69E-04	NFKB2	-459				
chr3	13053084	13053399	2.01	2.82E-04	RPL32	-171293	IQSEC1	61375		
chr2	239762938	239763182	2.30	2.86E-04	TWIST2	6334	HDAC4	559583		
chr14	103993757	103993999	2.57	2.88E-04	CKB	-4708	TRMT61A	-1631	TRMT61A	-1631
chr15	31558605	31558938	2.09	2.90E-04	TRPM1	-164848	KLF13	-60311		
chr9	136651561	136651868	1.93	2.97E-04	SARDH	-48236	VAV2	205731		
chr16	78133276	78133586	2.31	2.98E-04	WWOX	-120				
chr4	2747919	2748167	1.98	3.04E-04	TNIP2	10060	RNF4	277236		
chr4	140222435	140222725	1.85	3.05E-04	NARG1	-96				
chr18	56563565	56563882	2.17	3.10E-04	SEC11C	-243401	ZNF532	33663		
chr9	112736728	112737034	1.99	3.17E-04	PALM2-AKAP2	194304	TXN	281897		
chr9	37403095	37403522	2.64	3.19E-04	GRHPR	-19398	ZCCHC7	282840		
chr2	194340882	194341158	2.53	3.22E-04						
chr22	34317843	34318119	2.61	3.35E-04	LARGE	-1565				
chr19	44037339	44037595	1.78	3.40E-04	ZNF575	127				
chr1	153935883	153936162	2.11	3.50E-04	CRTC2	-4980	CREB3L4	-4374		
chr19	6244017	6244293	2.42	3.64E-04	MLLT1	35804	ACSBG2	108445		
chr8	1904145	1904389	3.01	3.72E-04	KBTBD11	-17777	ARHGEF10	132118		
chr6	7313354	7313713	2.21	3.74E-04	SSR1	7				
chr4	103748939	103749550	2.40	3.89E-04	UBE2D3	-537				
chr8	53206801	53207060	2.49	3.96E-04	PCMTD1	-395196	ST18	115508		
chr2	11893655	11894000	2.29	3.99E-04	TRIB2	-963170	LPIN1	7088		
chr1	203259424	203259940	2.32	4.06E-04	CHIT1	-60822	BTG2	-14982		
chr7	116660104	116660348	2.60	4.08E-04	ST7	66845	WNT2	303117		
chr8	131054592	131055005	2.19	4.09E-04	GSDMC	-255665	ASAP1	359417		

chr15	52554100	52554350	2.22	4.14E-04	GNB5	-70660	MYO5C	33745
chr3	141217697	141217995	2.08	4.15E-04	RNF7	-239205	RASA2	11920
chr6	12011378	12011783	2.03	4.25E-04	HIVEP1	-1143		
chr7	116223805	116224049	3.18	4.25E-04	MET	-88532	CAV1	59088
chr17	4278290	4278741	2.24	4.26E-04	SPNS3	-58703	UBE2G1	-8547
chr12	91775165	91775441	2.74	4.32E-04	DCN	-201944	BTG1	764370
chr16	11391374	11391731	2.37	4.33E-04	C16orf75	-47758	PRM1	-16361
chr6	154674062	154674378	2.95	4.34E-04	IPCEF1	3680	OPRM1	313777
chr11	128159960	128160380	2.24	4.35E-04	ETS1	297283		
chr9	96725250	96725542	1.91	4.35E-04	PTPDC1	-121350	BARX1	-7788
chr7	50308605	50308913	2.19	4.42E-04	ZPBP	-175899	IKZF1	-35619
chr2	45957102	45957385	2.10	4.42E-04	EPAS1	-567319	PRKCE	78201
chr11	115093871	115094147	2.38	4.44E-04	CADM1	281232	FAM55B	544809
chr11	65355636	65356020	1.77	4.45E-04	KCNK7	7639	SSSCA1	17885
chr1	45276081	45276391	1.81	4.46E-04	BTBD19	2082	PTCH2	32380
chr4	8393743	8394012	2.04	4.50E-04	ACOX3	48574	HTRA3	122386
chr5	32583741	32583990	2.03	4.58E-04	SUB1	-1739		
chr13	48878677	48879012	2.40	4.63E-04	RB1	962		
chr3	67050441	67050769	2.07	4.64E-04	KBTBD8	1878	SUCLG2	654433
chr15	78441334	78441622	1.78	4.65E-04	IDH3A	-241		
chr3	157147562	157147824	2.97	4.66E-04	CCNL1	-269211	PTX3	-6887
chr1	9486340	9486686	2.17	4.66E-04	SLC25A33	-113015	SPSB1	133572
chr11	128421735	128422158	2.29	4.75E-04	ETS1	35506		
chr19	16413155	16413413	2.64	4.76E-04	KLF2	-22367	AP1M1	104619
chr19	12902016	12902428	1.99	4.77E-04	JUNB	-88		
chr18	23795638	23795966	2.03	4.77E-04	TAF4B	-10607	PSMA8	81986
chr13	100195088	100195585	2.30	4.78E-04	CLYBL	-63599	TM9SF2	41609
chr2	176033408	176033688	2.48	4.80E-04	ATF2	-651		
chr14	59642183	59642485	2.40	4.82E-04	DAAM1	-13065	DACT1	537577
chr3	106021412	106021657	2.79	5.04E-04	CBLB	-433648		
chr1	174126371	174126616	2.26	5.05E-04	RABGAP1L	-2140		
chr19	16189680	16189979	2.14	5.13E-04	RAB8A	-32660	TPM4	11513
chr11	62389486	62389873	2.07	5.16E-04	B3GAT3	-232		
chr12	77171504	77171780	2.02	5.16E-04	ZDHHC17	13788	CSRP2	101157
chr3	185960388	185960664	2.75	5.22E-04	ETV5	-133625	DGKG	119497
chr1	8763370	8763658	1.50	5.23E-04	RERE	114185	SLC45A1	379124
chr2	101769340	101769576	2.46	5.28E-04	TBC1D8	-1612		
chr13	99626341	99626633	3.16	5.37E-04	SLC15A1	-221558	DOCK9	112173

chr19	37288171	37288475	2.24	5.40E-04	BC052603	-24607	ZNF790	40961
chr6	27125726	27126100	2.11	5.52E-04	PRSS16	-89595	HIST1H2AH	11005
chr20	49186984	49187250	2.17	5.52E-04	PARD6B	-160964	PTPN1	60226
chr12	125264329	125264670	1.86	5.57E-04	NCOR2	-244343	SCARB1	84019
chr13	91709108	91709372	2.70	5.58E-04	GPC5	-341695		
chr1	2322936	2323212	1.83	5.65E-04	RER1	-140		
chr1	25348771	25349242	2.20	5.78E-04	RUNX3	-92237	SYF2	210006
chr20	43331467	43331880	1.96	5.78E-04	ADA	-51298	WISP2	-12211
chr17	37911036	37911331	2.33	5.97E-04	GRB7	16997	IKZF3	109257
chr19	1038120	1038564	2.09	6.10E-04	ABCA7	-1760		
chr7	64146884	64147228	2.11	6.21E-04	ZNF138	-107715	ZNF107	20545
chr11	35187538	35187895	2.57	6.31E-04	CD44	27300	SLC1A2	253388
chr17	72510506	72510775	2.27	6.33E-04	CD300LB	16972	CD300A	48119
chr2	100851561	100852000	1.96	6.33E-04	AFF3	-129736	LONRF2	87414
chr5	118754485	118754754	2.29	6.34E-04	HSD17B4	-33528	TNFAIP8	63024
chr16	22218427	22218766	1.95	6.40E-04	POLR3E	-90144	EEF2K	1005
chr3	159640866	159641157	2.59	6.41E-04	IL12A	-65617	SCHIP1	649468
chr17	38771460	38771792	1.95	6.51E-04	CCR7	-49902	SMARCE1	32477
chr17	76640575	76640819	2.19	6.51E-04	DNAH17	-73293	CYTH1	137679
chr2	68917067	68917457	2.33	6.55E-04	ARHGAP25	-44706	PROKR1	44468
chrX	1599679	1600148	2.29	6.61E-04	ASMTL	-28070	P2RY8	56123
chr3	186244136	186244383	1.97	6.61E-04	DGKG	-164237	CRYGS	17907
chr12	53297179	53297455	2.67	6.62E-04	KRT78	-54539	KRT8	1551
chr14	76006510	76006765	2.14	6.68E-04	FLVCR2	-38302	BATF	17854
chr7	44530315	44530681	2.05	6.72E-04	CAMK2B	-165268	NPC1L1	50416
chr7	45020088	45020415	2.00	6.72E-04	MYO1G	-1548		
chr1	206291766	206292103	2.44	6.72E-04	C1orf186	-3288		
chr19	52110332	52110608	2.03	6.74E-04	hCG_2008157	-12840	SIGLEC5	23257
chr7	26004139	26004614	2.10	6.76E-04	NPVF	-736272	NFE2L3	-187470
chr11	62380421	62380708	2.04	6.91E-04	EML3	-328	ROM1	352
chr17	79450301	79450545	2.05	6.95E-04	ACTG1	29404	BAHCC1	76883
chr1	27021988	27022346	1.97	7.13E-04	ARID1A	-355		
chr20	48236835	48237261	2.34	7.26E-04	PTGIS	-52341	B4GALT5	93373
chr11	115093203	115093496	2.37	7.27E-04	CADM1	281891	FAM55B	544150
chr17	56736299	56736696	2.29	7.45E-04	SEPT4	-129835	TEX14	32918
chr1	206939833	206940078	2.62	7.48E-04	IL10	5883	MAPKAPK2	81667
chrX	48538894	48539199	2.53	7.56E-04	WAS	-3139		
chr8	141467747	141468023	1.69	7.60E-04	TRAPPC9	793		

chr6	15662538	15662929	2.24	7.61E-04	DTNBP1	537		
chr11	130036388	130036661	2.14	7.66E-04	ST14	6843	ZBTB44	148082
chr13	99828857	99829133	1.84	7.66E-04	DOCK9	-90335	UBAC2	-23684
chr21	44710291	44710635	2.25	7.84E-04	CRYAA	121322	SIK1	136539
chr5	95672142	95672418	1.98	7.98E-04	ELL2	-374505	PCSK1	96672
chr12	9803319	9803629	2.02	8.07E-04	KLRB1	-42977	CLEC2D	-18835
chr1	161359620	161359896	2.22	8.08E-04	FCGR2A	-115447	SDHC	75592
chr1	22192486	22192915	2.01	8.09E-04	LDLRAD2	53943	HSPG2	71049
chr3	113724335	113724604	2.18	8.10E-04	QTRTD1	-51141	ZDHHC23	57722
chr2	20876620	20876929	1.81	8.22E-04	GDF7	10351	APOB	390170
chr19	42101678	42101967	2.15	8.36E-04	CEACAM21	19292	CEACAM4	31619
chr8	131000355	131000614	2.29	8.61E-04	GSDMC	-201351	ASAP1	413731
chr19	6604860	6605191	2.21	8.74E-04	CD70	-13863	TNFSF14	65573
chr6	414639	414875	2.49	8.74E-04	IRF4	23005	EXOC2	278352
chr18	77385186	77385476	2.44	8.80E-04	CTDP1	-54470	NFATC1	225005
chr7	102065270	102065577	1.82	9.01E-04	ORAI2	-8572	PRKRIP1	28620
chr18	33709668	33709966	1.86	9.13E-04	SLC39A6	-460	ELP2	-70
chr3	169762747	169763023	2.03	9.13E-04	GPR160	7150	PHC3	136652
chr13	31613412	31613694	2.72	9.46E-04	HSPH1	122564	ALOX5AP	303884
chr20	62611365	62611614	2.20	9.52E-04	PRPF6	-941		
chr1	150336011	150336409	2.05	9.58E-04	RPRD2	-780		
chr1	207098645	207098951	2.07	9.66E-04	FAIM3	-3420		
chr1	161582390	161582656	1.93	9.72E-04	HSPA7	6674	FCGR3B	18635
chr16	4012776	4013037	2.58	9.73E-04	CREBBP	-82786	ADCY9	153279
chr17	61919985	61920334	2.25	9.73E-04	SMARCD2	191		
chr10	26661801	26662210	1.98	9.79E-04	APBB1IP	-65260	GAD2	156770
chr8	67005479	67005732	2.70	9.83E-04	TRIM55	-33672	DNAJC5B	71815
chr17	60802246	60802582	2.05	9.85E-04	MARCH10	83291	MRC2	97652
chr12	9917423	9917667	1.90	9.85E-04	CD69	-4048		
chr12	92865396	92865672	2.23	9.91E-04	BTG1	-325861	PLEKHG7	-264731
chr8	26429599	26429921	1.99	9.93E-04	PNMA2	-58277	DPYSL2	-5661
chr19	40926648	40927060	1.98	9.98E-04	PRX	-7583	SERTAD1	5078
chr18	33709264	33709589	1.80	1.01E-03	ELP2	-460	SLC39A6	-70
chr3	71060779	71061053	2.97	1.01E-03	FOXP1	572224		
chr22	27011198	27011478	2.01	1.03E-03	TPST2	-49968	CRYBB1	2653
chr8	56885616	56885865	2.09	1.04E-03	LYN	93355	RPS20	101399
chr10	91648545	91648931	2.58	1.05E-03	KIF20B	187371	HTR7	968933
chr1	211700390	211700685	2.84	1.06E-03	RD3	-34279	SLC30A1	51561

chr11	60134505	60134833	2.39	1.10E-03	MS4A7	-11289	MS4A6E	32314
chr5	124040609	124040949	2.04	1.10E-03	ZNF608	40086		
chrX	53100837	53101247	1.88	1.10E-03	TSPYL2	-10507	GPR173	22536
chr19	55166422	55166669	2.13	1.11E-03	LILRB4	-7578	LILRB1	24578
chr2	86221135	86221449	2.17	1.13E-03	ST3GAL5	-105135	POLR1A	111986
chr6	106545581	106545982	2.21	1.16E-03	PRDM1	11587	ATG5	227913
chr2	25584973	25585286	1.93	1.16E-03	DNMT3A	-20356	DTNB	311373
chr3	195633244	195633520	1.97	1.17E-03	TNK2	-10950	SDHALP1	83768
chr20	23331181	23331458	1.97	1.17E-03	NXT1	-53		
chr5	77656082	77656411	1.79	1.18E-03	SCAMP1	-92		
chr15	31751257	31751601	1.87	1.18E-03	KLF13	132346	OTUD7A	196113
chr17	259826	260265	1.88	1.18E-03	RPH3AL	-57470	VPS53	358050
chr13	95201807	95202118	2.05	1.19E-03	DCT	-70027	TGDS	46548
chr19	42618622	42618919	1.77	1.19E-03	POU2F2	17859	ZNF574	38481
chr18	56435686	56435997	2.08	1.20E-03	ZNF532	-94219	MALT1	97224
chr19	50401011	50401289	1.92	1.22E-03	IL4I1	-1003		
chr7	69204231	69204483	2.21	1.23E-03				
chr1	206912640	206913088	2.08	1.23E-03	IL10	32975	MAPKAPK2	54575
chr19	46209701	46210036	1.98	1.23E-03	QPCTL	14128	FBXO46	24282
chr9	12958765	12959071	2.55	1.25E-03	TYRP1	265532	MPDZ	291453
chr12	4647515	4647803	1.70	1.25E-03	RAD51AP1	-291		
chr17	8130237	8130481	2.66	1.28E-03	AURKB	-16476	C17orf68	21054
chr3	58182238	58182501	2.07	1.28E-03	DNASE1L3	18028	FLNB	188243
chr9	140655489	140655777	2.54	1.28E-03	CACNA1B	-116608	EHMT1	142189
chr4	114604324	114604707	2.84	1.29E-03	CAMK2D	78567	ANK2	633731
chr17	80828905	80829447	1.77	1.30E-03	ZNF750	-31245	B3GNTL1	180510
chr3	183245144	183245396	2.18	1.32E-03	MCF2L2	-99415	KLHL6	28229
chr1	36814922	36815244	1.80	1.36E-03	FAM176B	-25328	STK40	36402
chr4	124317723	124317999	2.02	1.39E-03	SPRY1	-95		
chr13	78596391	78596733	2.09	1.39E-03	EDNRB	-46898	POU4F1	581133
chr19	50529166	50529476	2.04	1.40E-03	VRK3	-516	ZNF473	109
chr3	183272113	183272439	1.97	1.41E-03	MCF2L2	-126421	KLHL6	1223
chr22	22307125	22307375	2.26	1.42E-03	PPM1F	-33		
chr11	58984357	58984655	2.02	1.42E-03	MPEG1	-4012		
chr11	65189537	65189881	1.86	1.45E-03	SCYL1	-102839	FRMD8	35668
chr15	41708998	41709344	1.88	1.46E-03	RTF1	-131		
chr3	177038667	177038974	2.02	1.46E-03	TBL1XR1	-123773		
chr1	9129690	9130086	2.10	1.46E-03	SLC2A5	-1		

chr19	14317995	14318469	1.32	1.47E-03	LPHN1	-1235		
chrX	152127365	152127653	1.85	1.47E-03	PNMA3	-97257	ZNF185	44512
chr3	138296877	138297378	2.05	1.47E-03	CEP70	16001	ESYT3	143713
chr15	83735874	83736159	1.71	1.49E-03	BTBD1	89		
chr2	136964223	136964620	2.23	1.53E-03	THSD7B	-784040	CXCR4	-88697
chr14	22750386	22750736	2.11	1.55E-03	TCRDV2	-177528	TRA	274797
chr4	102711663	102711928	1.80	1.56E-03	BANK1	32		
chr1	205744227	205744760	2.19	1.59E-03	RAB7L1	116		
chr9	130732692	130732990	1.93	1.60E-03	DPM2	-32078	NAIF1	96758
chr17	43430466	43430731	2.47	1.61E-03	MAP3K14	-36185	ARHGAP27	72413
chr3	185000593	185000837	2.11	1.61E-03	MAP3K13	-80255	EHHADH	-28878
chr1	157010672	157010932	3.33	1.62E-03	ARHGEF11	4360	PEAR1	147279
chr19	49588584	49588884	1.74	1.62E-03	SNRNP70	269	SNRNP70	269
chr12	53765023	53765299	1.86	1.64E-03	SP7	-35624	SP1	-8818
chr1	79658909	79659388	2.21	1.64E-03	ELTD1	-186654		
chr4	87857329	87857597	2.43	1.69E-03	SLC10A6	-87047	AFF1	-70690
chr1	175158069	175158573	2.10	1.75E-03	TNN	121327	TNR	554431
chr15	90324379	90324654	2.04	1.75E-03	MESP2	4928	ANPEP	33555
chr15	70796970	70797299	1.81	1.75E-03	TLE3	-406879	UACA	258715
chr20	48315885	48316190	2.23	1.79E-03	PTGIS	-131331	B4GALT5	14383
chr16	87734591	87734924	1.73	1.80E-03	KLHDC4	64784	JPH3	98259
chr19	49256099	49256375	2.27	1.85E-03	FGF21	-2911	FUT1	2410
chr4	56814790	56815091	1.86	1.85E-03	CEP135	-96		
chr3	179156581	179156852	2.27	1.85E-03	GNB4	12654	MFN1	91237
chr1	110950195	110950521	1.94	1.86E-03	HBXIP	188		
chr17	62502224	62502550	1.98	1.87E-03	CCDC45	-771	DDX5	97
chr22	18278557	18279001	2.45	1.88E-03	BID	-22000	MICAL3	228546
chr6	291589	292060	1.98	1.89E-03	DUSP22	-276		
chr7	73515790	73516078	1.92	1.91E-03	EIF4H	-72772	LIMK1	17778
chr11	117857864	117858182	2.06	1.91E-03	IL10RA	917		
chr3	15387279	15387575	1.69	1.94E-03	SH3BP5	-13323	METTL6	81615
chr12	96589362	96589638	2.21	1.94E-03	ELK3	1293	PCTK2	204723
chr22	20104644	20104884	1.67	1.95E-03	RANBP1	-260	TRMT2A	4
chr10	27026627	27026920	2.03	1.96E-03	PDSS1	40179	ABI1	123185
chr12	104322801	104323077	2.03	1.97E-03	HSP90B1	-1250	GNN	1050
chr14	102342913	102343259	1.96	2.00E-03	DYNC1H1	-87779	PPP2R5C	114951
chr11	117881734	117882048	2.19	2.00E-03	TMPRSS4	-65836	IL10RA	24785
chr9	140082936	140083246	1.84	2.02E-03	ANAPC2	-34	SSNA1	37

chr12	90320940	90321242	2.06	2.05E-03	ATP2B1	-271247		
chr4	143326555	143326799	2.79	2.05E-03	INPP4B	440927	IL15	768923
chr1	28879303	28879613	1.70	2.10E-03	TRNAU1AP	-71		
chr2	202022846	202023090	2.01	2.11E-03	CASP10	-24653	CFLAR	42152
chr6	13274059	13274416	2.04	2.11E-03	TBC1D7	54532	PHACTR1	556405
chr4	185655264	185655574	2.51	2.11E-03	MLF1IP	-133		
chr19	42612707	42612990	1.80	2.18E-03	POU2F2	23781	ZNF574	32559
chr22	21355953	21356245	1.89	2.20E-03	THAP7	305		
chr15	89183378	89183624	1.76	2.21E-03	ACAN	-163173	ISG20	1462
chr12	90343717	90343993	2.25	2.22E-03	ATP2B1	-294011		
chr18	72166955	72167258	2.06	2.22E-03	CNDP1	-34585	CNDP2	3510
chr11	111259717	111259993	2.08	2.24E-03	POU2AF1	-9698	BTG4	123209
chr19	7770557	7770861	2.25	2.24E-03	FCER2	-3677		
chr9	37378256	37378528	2.14	2.24E-03	GRHPR	-44315	ZCCHC7	257923
chr3	171824820	171825084	1.91	2.27E-03	FNDC3B	67534	GHSR	341251
chr17	40215742	40216083	1.74	2.31E-03	ZNF385C	-35206	DHX58	48838
chr6	159128951	159129228	2.25	2.32E-03	SYTL3	58044	EZR	110250
chr3	121733612	121733917	2.23	2.35E-03	ILDR1	7265	SLC15A2	120594
chr11	118972755	118973040	1.99	2.35E-03	DPAGT1	-113		
chrX	52963879	52964219	1.98	2.37E-03	FAM156B	21904	FAM156B	35964
chr3	195264826	195265191	1.92	2.39E-03	ACAP2	-101192	PPP1R2	5215
chr5	88028037	88028357	1.96	2.40E-03	TMEM161B	-463532	MEF2C	151105
chr19	14693675	14693966	2.05	2.42E-03	CLEC17A	-75		
chr3	194854319	194854655	2.15	2.42E-03	LSG1	-461281	C3orf21	137408
chr19	58962717	58963038	1.69	2.42E-03	ZNF324B	-93		
chr7	47977820	47978119	1.82	2.45E-03	PKD1L1	10067	C7orf69	143081
chr1	36770171	36770424	2.08	2.46E-03	FAM176B	19457	THRAP3	80281
chr2	233946933	233947269	2.06	2.47E-03	ATG16L1	-213116	INPP5D	22065
chr10	52384261	52384572	1.69	2.47E-03	SGMS1	-680		
chr11	86013041	86013370	2.02	2.49E-03	C11orf73	-47		
chr13	52188700	52188976	2.10	2.51E-03	WDFY2	30354	DHRS12	189455
chr18	76828983	76829292	1.80	2.55E-03	ATP9B	-259		
chr19	47730794	47731110	1.83	2.58E-03	BBC3	5071	SAE1	96872
chr18	55288920	55289248	1.98	2.58E-03	NARS	93		
chr1	212661978	212662302	2.22	2.60E-03	ATF3	-76557	NENF	55911
chr3	150804448	150804807	1.84	2.62E-03	MED12L	43		
chr7	150132433	150132720	2.00	2.63E-03	GIMAP8	-15385	ZNF775	56171
chr2	158325357	158325700	2.12	2.63E-03	CYTIP	-24925	ACVR1C	159870

chr18	12407779	12408110	1.49	2.65E-03	SLMO1	50		
chr12	104321996	104322272	1.95	2.69E-03	HSP90B1	-2055	GNN	1855
chr3	184231552	184231828	2.29	2.70E-03	EPHB3	-47897	CHRD	133829
chr4	164585044	164585320	2.09	2.71E-03	MARCH1	-50406		
chrX	48776232	48776644	1.81	2.74E-03	PIM2	-25		
chr2	9563448	9563807	1.68	2.74E-03	CPSF3	-240	ITGB1BP1	15
chr1	234658332	234658699	1.91	2.77E-03	TARBP1	-43667	IRF2BP2	86755
chr9	139662591	139662924	1.63	2.77E-03	LCN15	-3793		
chr8	130951952	130952249	1.65	2.78E-03	GSDMC	-152967	ASAP1	462115
chr1	235098956	235099266	1.97	2.79E-03	IRF2BP2	-353840	TOMM20	193145
chr3	186718480	186718918	2.15	2.84E-03	ST6GAL1	-20966	ADIPOQ	158236
chr20	16554000	16554295	1.65	2.85E-03	KIF16B	-70		
chr3	42632021	42632300	1.69	2.90E-03	NKTR	-9986	SEC22C	-8733
chr10	80827039	80827338	1.76	2.91E-03	ZMIZ1	-1603		
chr1	192544625	192544958	1.85	2.93E-03	RGS1	-65		
chr4	74124640	74125046	1.92	2.93E-03	ANKRD17	-341		
chr3	187637464	187637777	1.94	2.94E-03	LPP	-293100	BCL6	-174146
chr8	103836554	103836871	2.11	2.95E-03	KLF10	-168730	AZIN1	39684
chr5	34499164	34499431	2.57	2.99E-03	C1QTNF3	-455981	RAI14	-157298
chr19	58987297	58987775	1.86	3.00E-03	ZNF446	-259		
chr7	148198841	148199165	1.79	3.01E-03	CUL1	-196930	NM_001126313	67364
chr22	18077412	18077703	1.98	3.03E-03	ATP6V1E1	34030	SLC25A18	34375
chr7	134832616	134832918	2.02	3.05E-03	TMEM140	1		
chr19	56189497	56189881	1.91	3.06E-03	EPN1	1698	NLRP9	60079
chr3	169658263	169658608	1.98	3.07E-03	LRRC31	-70776	SEC62	-26144
chr4	74455907	74456347	2.16	3.08E-03	RASSF6	30213	AFM	108665
chr21	48055231	48055601	1.79	3.09E-03	PRMT2	-111		
chr1	150132556	150132942	2.07	3.14E-03	PLEKHO1	10579	ANP32E	75755
chr9	132175901	132176201	1.60	3.14E-03	METTL11A	-212384	PPP2R4	302807
chr11	118691354	118691845	2.57	3.15E-03	CXCR5	-62941	DDX6	-29628
chrX	347842	348156	1.76	3.16E-03	PPP2R3B	-372		
chr3	47823362	47823619	1.78	3.17E-03	SMARCC1	-86		
chr1	145396850	145397126	1.96	3.17E-03	HFE2	-16203	NBPF10	103617
chr3	190963874	190964123	1.90	3.18E-03	CCDC50	-82875	OSTN	33677
chr2	220363479	220363786	1.71	3.19E-03	GMPPA	20		
chr1	145399174	145399494	2.01	3.20E-03	HFE2	-13857	NBPF10	105963
chr12	50505880	50506272	1.74	3.21E-03	C12orf62	176		
chr16	77224581	77225246	2.04	3.26E-03	LOC100130958	-8435	CNTNAP4	881179

chr2	112462829	112463144	1.68	3.31E-03	ANAPC1	178754	BCL2L11	584496
chr12	46384440	46384765	1.89	3.33E-03	SFRS2IP	-202		
chr19	57862485	57862738	1.86	3.35E-03	ZNF304	-33		
chr10	67261028	67261289	2.33	3.35E-03				
chr9	139434133	139434569	1.97	3.39E-03	NOTCH1	5887	C9orf163	56404
chr8	53626928	53627233	1.71	3.42E-03	RB1CC1	-55		
chr5	58423523	58423799	1.67	3.49E-03	RAB3C	544722	PDE4D	765960
chr9	95640211	95640576	1.90	3.49E-03	ZNF484	-104		
chr12	12878273	12878676	1.53	3.51E-03	APOLD1	-376		
chr6	18328843	18329119	1.94	3.51E-03	DEK	-64182	RNF144B	-58613
chr8	96036933	96037209	1.86	3.58E-03	C8orf38	-150		
chr4	87882822	87883098	1.91	3.58E-03	SLC10A6	-112544	AFF1	-45193
chr11	117356943	117357243	1.93	3.60E-03	CEP164	158522	DSCAML1	310883
chr1	197170520	197170911	1.79	3.69E-03	ZBTB41	-1044		
chr15	23033425	23033701	2.06	3.72E-03	NIPA2	845		
chr17	76702407	76702657	1.77	3.74E-03	DNAH17	-135128	CYTH1	75844
chr11	63993604	63993884	1.73	3.75E-03	DNAJC4	-4009	TRPT1	-18
chr7	140624715	140624992	1.81	3.80E-03	BRAF	-290		
chr19	19266343	19266705	2.33	3.86E-03	TMEM161A	-17257	MEF2B	14574
chr19	5791094	5791357	1.79	3.91E-03	DUS3L	23		
chr2	218087301	218087577	1.92	3.93E-03	TNP1	-362657	TNS1	721357
chr2	232572762	232573105	1.58	3.94E-03	PTMA	-301		
chr19	4581733	4582009	1.94	3.95E-03	SEMA6B	-23370	C19orf10	88544
chr4	146981054	146981370	1.87	3.99E-03	ZNF827	-121605	LSM6	-115623
chr1	2232533	2232875	1.97	4.00E-03	RER1	-90510	SKI	72570
chr10	52383633	52383931	1.58	4.00E-03	SGMS1	-45		
chr9	88555634	88555880	1.73	4.04E-03	MAK10	-300		
chr1	220263029	220263394	2.03	4.10E-03	IARS2	-4243	BPNT1	-21
chr1	144533961	144534325	3.26	4.10E-03	PPIAL4A	-169897	PDE4DIP	460879
chr19	947094	947401	1.83	4.11E-03	GRIN3B	-53189	ARID3A	21211
chr2	98644103	98644429	1.57	4.12E-03	CNGA3	-318352	TMEM131	-31912
chr5	77590368	77590837	1.70	4.13E-03	AP3B1	-75		
chr8	133288601	133288911	1.80	4.15E-03	HHLA1	-171244	KCNQ3	204248
chr5	64357289	64357649	2.07	4.15E-03	SDCCAG10	292714	ADAMTS6	420235
chrX	70585693	70585971	1.82	4.20E-03	TAF1	-282		
chr1	235123380	235123628	2.39	4.21E-03	IRF2BP2	-378233	TOMM20	168752
chr12	58292965	58293337	1.71	4.28E-03	CTDSP2	-52404	XRCC6BP1	-42294
chr17	77336754	77337298	1.99	4.32E-03	HRNBP3	141654	ENGASE	266007

chr2	10554148	10554472	1.74	4.36E-03	ODC1	34143	HPCAL1	111270		
chr16	89831320	89831652	1.68	4.36E-03	ZNF276	43534	FANCA	51579		
chr7	50332012	50332256	2.03	4.36E-03	ZPBP	-199274	IKZF1	-12244		
chr10	101754168	101754412	1.81	4.38E-03	DNMBP	15386	ABCC2	211827		
chr2	74756660	74757085	1.84	4.39E-03	DQX1	-3465	AUP1	101	HTRA2	341
chr3	15419692	15420042	1.80	4.39E-03	SH3BP5	-45763	METTL6	49175		
chr1	31226586	31226889	1.79	4.48E-03	MATN1	-30306	LAPTM5	3945		
chr2	70417700	70417987	1.74	4.50E-03	TIA1	57935	PCBP1	103259		
chr19	39050229	39050584	1.68	4.50E-03	MAP4K1	58236	RYR1	126067		
chr17	41445179	41445456	2.05	4.55E-03	ARL4D	-31035	TMEM106A	81424		
chr19	59084313	59084666	1.48	4.56E-03	MZF1	452				
chr4	144325777	144326021	2.12	4.67E-03	SMARCA5	-108717	GAB1	67916		
chr18	43830396	43830737	1.78	4.69E-03	RNF165	-83620	HAUS1	146269		
chr1	40652194	40652470	1.82	4.72E-03	TMCO2	-61241	RLF	25291		
chr11	65641059	65641410	1.59	4.75E-03	EFEMP2	-895				
chr7	105332520	105332817	2.01	4.78E-03	FLJ23834	-270988	RINT1	160137		
chr3	178789103	178789380	2.21	4.78E-03	ZMAT3	342				
chr20	37471192	37471520	2.13	4.82E-03	FAM83D	-83599	PPP1R16B	37008		
chr6	43142252	43142599	1.80	4.85E-03	CUL9	-7496	SRF	3506		
chr1	221951137	221951413	1.93	4.85E-03	DUSP10	-35814	HHIPL2	770169		
chr2	69875329	69875626	1.99	4.87E-03	AAK1	-4501				
chr2	185309023	185309344	1.97	4.87E-03	ZNF804A	-153909				
chr12	26266794	26267109	1.94	4.91E-03	BHLHE41	11051	RASSF8	154983		
chr1	206290163	206290588	2.38	4.93E-03	C1orf186	-1729				
chr19	35949907	35950234	1.89	4.96E-03	FFAR2	9454	KRTDAP	31285		
chr11	118741356	118741830	1.92	4.96E-03	DDX6	-79621	CXCR5	-12948		
chr15	80310021	80310274	2.21	4.99E-03	BCL2A1	-46505	ZFAND6	-41873		
chr1	249168218	249168608	1.92	5.01E-03	ZNF692	-15142				
chr12	12764615	12764894	1.68	5.03E-03	CREBL2	-76				
chr2	232526884	232527235	1.82	5.03E-03	NMUR1	-131878	PTMA	-46175		
chr15	22541907	22542314	1.88	5.06E-03	GOLGA6L1	-194135	OR4N2	128649		
chr4	109541524	109541808	1.86	5.12E-03	RPL34	-56				
chr4	184427339	184427669	1.65	5.12E-03	ING2	1284	ENPP6	711610		
chr9	128395007	128395322	1.96	5.13E-03	MAPKAP1	74348	GAPVD1	371054		
chr17	76635122	76635568	1.79	5.16E-03	DNAH17	-67941	CYTH1	143031		
chr3	15689160	15689470	2.23	5.18E-03	BTD	46060	ANKRD28	211738		
chrX	24168434	24168692	2.03	5.18E-03	ZFX	-1245				
chr5	77655651	77655939	1.68	5.28E-03	SCAMP1	-544				

chr2	39719234	39719565	2.11	5.30E-03	TMEM178	-173693	MAP4K3	-55181
chr12	56584009	56584281	2.10	5.30E-03	SMARCC2	-794		
chr17	1359501	1359763	1.56	5.36E-03	CRK	-88		
chr1	145589284	145589630	1.90	5.37E-03	NUDT17	-22		
chr8	8610515	8610881	2.12	5.45E-03	CLDN23	51032	MFHAS1	140433
chr16	85532132	85532564	1.61	5.49E-03	KIAA0182	-114576	KIAA0513	470938
chr17	8099437	8099701	2.17	5.50E-03	C17orf59	-6005	AURKB	14314
chr5	60615601	60615917	1.76	5.53E-03	C5orf43	-157457	ZSWIM6	-12341
chr19	49956249	49956561	1.57	5.53E-03	PIH1D1	-1290	ALDH16A1	-68
chr1	15898248	15898583	2.04	5.67E-03	AGMAT	13189	DNAJC16	45064
chr4	141075292	141075572	1.54	5.69E-03	MAML3	-199		
chr22	17738876	17739239	1.97	5.70E-03	CECR2	-217572	CECR1	-48279
chr15	63766286	63766577	1.66	5.71E-03	CA12	-92357	USP3	-30378
chr16	56950940	56951242	1.81	5.76E-03	HERPUD1	-14657	SLC12A3	51972
chr7	151168800	151169100	1.87	5.78E-03	RHEB	48060	NUB1	130092
chr4	23335610	23336081	1.98	5.83E-03	PPARGC1A	555854	GBA3	641298
chr7	22485063	22485401	1.63	5.84E-03	RAPGEF5	-88699	MGC87042	54566
chr7	47976914	47977278	1.88	5.87E-03	PKD1L1	10941	C7orf69	142207
chr12	25150986	25151389	1.94	5.89E-03	LRMP	-54053	BCAT1	-48880
chr1	235116767	235117051	2.18	5.89E-03	IRF2BP2	-371638	TOMM20	175347
chr11	128422917	128423248	1.95	5.89E-03	ETS1	34370		
chr2	65280408	65280688	1.73	5.98E-03	CEP68	-2947		
chr2	197024786	197025138	1.98	6.02E-03	DNAH7	-91426	STK17B	11374
chr12	59507616	59507892	2.16	6.04E-03	SLC16A7	-575372	LRIG3	-193492
chr18	9102341	9102663	1.84	6.04E-03	NDUFV2	-173		
chr2	97202813	97203091	1.87	6.07E-03	ARID5A	488		
chr11	62319233	62319695	2.13	6.08E-03	AHNAK	-5132	EEF1G	21996
chr11	82773012	82773278	2.16	6.10E-03	RAB30	9739	C11orf82	160408
chr2	39355179	39355448	1.69	6.10E-03	SOS1	-7710	CDKL4	101359
chr16	27388720	27388967	1.98	6.11E-03	IL21R	-49735	IL4R	63593
chr7	29317166	29317444	1.75	6.11E-03	PRR15	-286122	CHN2	83184
chr17	72510947	72511191	1.93	6.19E-03	CD300LB	16544	CD300A	48547
chr10	96996105	96996455	2.02	6.26E-03	C10orf129	42323	PDLIM1	54501
chr5	118679470	118679746	1.88	6.31E-03	TNFAIP8	-11988		
chr4	87995037	87995288	1.77	6.31E-03	AFF1	67010	KLHL8	146511
chr3	58477531	58477819	1.78	6.34E-03	KCTD6	-148		
chr2	21022750	21023132	1.78	6.34E-03	GDF7	156517	APOB	244004
chr19	58919673	58920106	1.42	6.38E-03	ZNF584	-173		

chr13	99128765	99129017	2.05	6.40E-03	RNF113B	-299370	STK24	100505
chr9	112178471	112178741	1.93	6.48E-03	EPB41L4B	-95585	PTPN3	81987
chr7	128786235	128786679	1.94	6.73E-03	SMO	-42256	TSPAN33	1745
chr6	53409734	53410050	1.65	6.76E-03	GCLC	-61		
chr4	119199568	119200119	1.80	6.76E-03	PRSS12	74078	NDST3	244344
chr9	130878080	130878325	1.97	6.82E-03	PTGES2	12271	SLC25A25	47724
chr3	122417137	122417381	2.16	6.84E-03	PARP14	17587	HSPBAP1	95391
chr10	70319909	70320185	1.98	6.89E-03	TET1	-70		
chrX	47077075	47077502	1.62	6.98E-03	PCTK1	-826		
chr12	113772740	113773081	1.64	6.99E-03	SLC24A6	14		
chr12	1913725	1914001	1.59	7.00E-03	LRTM2	-15570	ADIPOR2	113616
chr19	7744182	7744585	1.75	7.00E-03	TRAPPC5	-1323		
chr8	142216658	142216906	1.80	7.06E-03	PTK2	-205450	SLC45A4	21891
chr17	45569950	45570220	2.05	7.08E-03	NPEPPS	-38359	C17orf57	168735
chr2	97523600	97523900	1.47	7.10E-03	ANKRD23	-13992	SEMA4C	11985
chr5	124270762	124271107	1.74	7.18E-03	ZNF608	-190070		
chr9	117160066	117160371	1.88	7.22E-03	AKNA	-3534		
chr3	152016798	152017237	1.68	7.32E-03	MBNL1	-176		
chr9	101943818	101944285	1.83	7.35E-03	ALG2	40194	TGFBR1	76640
chr1	52344461	52344737	1.71	7.36E-03	NRD1	10		
chr20	5059164	5059459	1.82	7.36E-03	SLC23A2	-68373	C20orf30	34421
chr8	135613841	135614465	1.67	7.53E-03	ZFAT	111128		
chr19	58978220	58978471	1.66	7.54E-03	ZNF324	-117		
chr21	36599556	36599922	1.89	7.57E-03	CBR1	-842546	RUNX1	-178144
chr5	68628501	68628745	1.82	7.61E-03	TAF9	36786	CDK7	98001
chr6	28180625	28180958	2.05	7.62E-03	ZNF193	-12281	ZNF192	71076
chr17	36908750	36909162	1.94	7.66E-03	PCGF2	-4395	PSMB3	-46
chr15	30917875	30918174	2.07	7.70E-03	ARHGAP11B	-854		
chr14	32030424	32030760	1.76	7.72E-03	NUBPL	1		
chr17	3461877	3462153	1.76	7.74E-03	TRPV3	-726		
chr17	77397208	77397484	1.87	7.86E-03	HRNBP3	81334	ENGASE	326327
chr18	39694333	39694609	1.72	7.88E-03	PIK3C3	159272		
chr10	102672386	102672648	1.78	7.92E-03	SEMA4G	-59769	PAX2	167049
chr2	25585634	25585878	1.69	7.96E-03	DNMT3A	-20982	DTNB	310747
chr1	161016398	161016678	2.18	7.98E-03	USF1	-781		
chr14	24836387	24836663	1.47	8.02E-03	NFATC4	380		
chr17	55681347	55681643	1.97	8.04E-03	MRPS23	245904	MSI2	347564
chrX	77359209	77359599	1.86	8.04E-03	PGK1	-262		

chr19	909174	909557	1.75	8.12E-03	MED16	-16148	C19orf22	3859
chr19	55629041	55629326	1.53	8.14E-03	PPP1R12C	-257		
chr16	85790002	85790293	1.81	8.18E-03	GINS2	-67560	COX4NB	43000
chr14	65818560	65818866	2.07	8.23E-03	MAX	-249486	FUT8	-60822
chr12	66626115	66626408	1.98	8.39E-03	HELB	-70094	IRAK3	43284
chr3	138089962	138090218	2.02	8.40E-03	ESYT3	-63325	MRAS	22582
chr11	75484607	75484948	1.91	8.40E-03	UVRAG	-41434	DGAT2	5000
chr21	43955154	43955576	1.84	8.41E-03	PDE9A	-118497	SLC37A1	35623
chr20	52543087	52543500	1.93	8.53E-03	ZNF217	-343587	BCAS1	144010
chr2	68989821	68990214	1.75	8.60E-03	ARHGAP25	28050	BMP10	108631
chr2	240189126	240189402	1.65	8.61E-03	HDAC4	133379	TWIST2	432538
chr7	73625266	73625598	1.49	8.63E-03	LAT2	1345	RFC2	43306
chr9	114424647	114424923	2.20	8.73E-03	UGCG	-234421	DNAJC25	31153
chr2	216880706	216881011	1.79	8.75E-03	MREG	-2513		
chr19	51399901	51400205	1.67	8.77E-03	KLK4	13941	KLK2	23364
chr3	122824479	122824774	1.70	8.82E-03	SEC22A	-96147	PDIA5	38662
chr1	26947298	26947608	1.55	8.90E-03	ARID1A	-75069	RPS6KA1	75110
chr2	3714873	3715137	1.76	8.94E-03	ALLC	9219		
chr3	177077539	177078016	2.20	8.94E-03	TBL1XR1	-162730		
chr3	186703865	186704146	1.94	8.94E-03	ST6GAL1	-35659	ADIPOQ	143543
chr1	154531168	154531574	1.96	9.00E-03	UBE2Q1	-251		
chr6	134700246	134700678	1.71	9.00E-03	SGK1	-61266	ALDH8A1	570782
chr1	31227545	31227972	2.29	9.11E-03	MATN1	-31327	LAPTM5	2924
chr2	100863964	100864335	1.79	9.17E-03	AFF3	-142105	LONRF2	75045
chr2	88315793	88316092	1.97	9.20E-03	RGPD1	-190628	SMYD1	-51439
chr7	55601110	55601408	1.85	9.27E-03	VOPP1	38941	LANCL2	168118
chrX	153607212	153607629	1.25	9.35E-03	FLNA	-4415	EMD	-176
chr12	108079356	108079645	1.68	9.41E-03	PWP1	-89		
chr11	128343482	128343888	1.66	9.45E-03	ETS1	113768		
chr19	38524247	38524807	1.88	9.53E-03	SIPA1L3	126659	DPF1	190363
chr2	98629081	98629423	1.90	9.60E-03	CNGA3	-333366	TMEM131	-16898
chr1	66867747	66868269	1.68	9.64E-03	SGIP1	-131817	PDE4B	609152
chr17	17566627	17567327	1.63	9.65E-03	PEMT	-71983	RAI1	-17810
chr19	17633941	17634502	1.97	9.78E-03	GLT25D1	-32289	PGLS	11790
chr16	22203587	22203865	1.84	9.80E-03	EEF2K	-13866	VWA3A	99863
chr1	211870716	211871109	1.51	9.83E-03	NEK2	-21946	LPGAT1	133201
chr6	132819763	132820055	2.14	9.96E-03	MOXD1	-97245	STX7	14428
chr5	65850082	65850358	1.75	9.97E-03	MAST4	-274384	SFRS12	410135

chr22	39541546	39541818	1.91	1.00E-02	CBX7	6856	APOBEC3H	48392
chr6	4776455	4776731	1.68	1.01E-02	CDYL	-87		
chr19	10676605	10676874	1.52	1.02E-02	CDKN2D	2891	ATG4D	22093
chr15	75339281	75339754	1.66	1.02E-02	PPCDC	23591	DNM1P33	255788
chr4	185235517	185235793	1.81	1.02E-02	ENPP6	-96541	IRF2	160071
chr12	111882730	111883037	1.84	1.02E-02	SH2B3	39132	ATXN2	154596
chr5	169758190	169758462	2.23	1.03E-02	LCP2	-33504	KCNIP1	-22555
chr1	150265913	150266470	1.84	1.03E-02	MRPS21	-77		
chr13	48809222	48809508	2.08	1.03E-02	RB1	-68518	ITM2B	2091
chr11	121440741	121441065	1.60	1.04E-02	SORL1	117942	BLID	546020
chr2	70702455	70702743	2.13	1.04E-02	FAM136A	-173379	TGFA	78506
chr5	10700127	10700502	1.62	1.05E-02	DAP	61072	ROPN1L	258306
chr5	139018000	139018278	1.61	1.07E-02	CXXC5	-10162	UBE2D2	77388
chr6	53412697	53412987	1.56	1.07E-02	GCLC	-3011		
chr17	42425572	42425933	1.72	1.07E-02	GRN	3262	FAM171A2	15482
chr19	59055357	59055834	1.49	1.07E-02	TRIM28	-240		
chr11	121460678	121461251	1.97	1.07E-02	SORL1	138004	BLID	525958
chr18	31382994	31383275	2.07	1.07E-02	ASXL3	224594	NOL4	420311
chr17	40400575	40401027	1.78	1.07E-02	GHDC	-54251	STAT5B	27623
chr15	33162700	33162976	1.86	1.07E-02	GREM1	152633	FMN1	197247
chr1	32421650	32421969	1.70	1.08E-02	KHDRBS1	-57681	PTP4A2	-17822
chr12	123868360	123868648	1.63	1.09E-02	SETD8	-200		
chr15	91329374	91329826	1.79	1.10E-02	FURIN	-82285	BLM	69021
chr12	104322453	104322697	1.78	1.11E-02	HSP90B1	-1614	GNN	1414
chr22	39925188	39925498	1.87	1.11E-02	RPS19BP1	3517	ATF4	8774
chr15	44068330	44068735	1.68	1.12E-02	ELL3	969		
chr3	100427964	100428465	1.79	1.13E-02	TFG	55		
chr15	43212986	43213391	1.59	1.13E-02	TTBK2	-182		
chr19	45519610	45520057	1.65	1.14E-02	SFRS16	-22464	RELB	15122
chr2	8713733	8714009	1.60	1.14E-02	ID2	-108113		
chr2	231467386	231467687	1.92	1.14E-02	CAB39	-110020	SP100	186666
chr5	109257497	109257807	2.16	1.15E-02	MAN2A1	232496	TMEM232	804729
chr2	96890816	96891154	1.69	1.15E-02	STARD7	-16412	TMEM127	40747
chr11	117688973	117689250	2.13	1.16E-02	DSCAML1	-21136	FXYD2	6347
chr15	66111282	66111565	1.87	1.17E-02	RAB11A	-50372	DENND4A	-26793
chr10	105437954	105438233	1.63	1.18E-02	SH3PXD2A	177070	NEURL	184359
chr3	17236699	17237111	1.99	1.18E-02	PLCL2	262323	TBC1D5	547335
chr12	90250298	90250574	1.46	1.18E-02	ATP2B1	-200592		

chr3	122295034	122295365	2.01	1.18E-02	PARP15	-1249		
chr11	64633091	64633504	1.73	1.19E-02	CDC42BPG	-21257	EHD1	12893
chr19	11146509	11146985	1.91	1.19E-02	LDLR	-53310	SMARCA4	75149
chr5	33440691	33440971	1.78	1.20E-02	TARS	-67		
chr19	49955016	49955371	1.88	1.20E-02	ALDH16A1	-1279	PIH1D1	-79
chr1	159037709	159038319	1.98	1.20E-02	AIM2	8633	IFI16	58332
chr19	56825753	56826103	1.39	1.20E-02	ZSCAN5A	-86269	ZNF542	-53540
chr2	89137752	89138205	1.69	1.20E-02	O1/O11 and JK2	-752583	RPIA	146803
chr16	2961748	2962018	1.41	1.20E-02	FLYWCH1	-97		
chr14	24578045	24578389	1.66	1.21E-02	DCAF11	-5689	PCK2	14734
chr6	30456933	30457298	1.77	1.21E-02	HLA-E	-155		
chr13	79768481	79768776	1.82	1.22E-02	RNF219	-535315	RBM26	211294
chr17	56708946	56709245	1.91	1.22E-02	SEPT4	-102433	TEX14	60320
chr2	3522726	3523036	1.54	1.23E-02	ADI1	469		
chr18	77580121	77580424	1.80	1.23E-02	KCNG2	-43395	CTDP1	140472
chr1	80133585	80133861	2.07	1.24E-02	ELTD1	-661228		
chr8	33330584	33330933	1.46	1.24E-02	FUT10	-95		
chr19	58666445	58666767	1.74	1.24E-02	ZNF329	-4458		
chr3	197439219	197439548	1.78	1.24E-02	BDH1	-156526	KIAA0226	24389
chr19	36606307	36606637	1.53	1.27E-02	POLR2I	-266	TBCB	584
chr2	42332512	42332832	1.76	1.27E-02	EML4	-63818	SGK493	57511
chr3	186236758	186237174	1.63	1.27E-02	DGKG	-156943	CRYGS	25201
chr3	195620386	195620680	1.64	1.28E-02	MUC4	-81385	TNK2	1899
chr3	120314933	120315333	1.64	1.29E-02	NDUFB4	5		
chr15	45020905	45021419	2.42	1.29E-02	TRIM69	-7378	B2M	17477
chr17	53315651	53315904	1.86	1.30E-02	HLF	-26543	STXBP4	269652
chr2	6485693	6486026	1.68	1.31E-02	CMPK2	520076	SOX11	653061
chr3	188156876	188157244	1.67	1.32E-02	TPRG1	-732703	LPP	226339
chr5	1499042	1499374	1.67	1.32E-02	SLC6A3	-53670	LPCAT1	24868
chr16	3202823	3203124	1.88	1.32E-02	OR1F1	-51273	caspace	8754
chr9	102016113	102016543	1.66	1.34E-02	NR4A3	-572681	SEC61B	31758
chr19	54605905	54606206	1.67	1.35E-02	OSCAR	-1814	NDUFA3	-104
chr13	95253617	95254083	1.69	1.36E-02	GPR180	-254		
chr15	86233838	86234109	1.85	1.36E-02	KLHL25	104215	AKAP13	310103
chr17	37778214	37778524	1.66	1.37E-02	PPP1R1B	-4810		
chr1	154580537	154580947	1.56	1.38E-02	ADAR	-60		
chr14	102310025	102310475	1.51	1.38E-02	DYNC1H1	-120615	PPP2R5C	82115
chr7	55637485	55637761	1.76	1.40E-02	VOPP1	2577	LANCL2	204482

chr1	245084313	245084603	2.30	1.40E-02	HNRNPU	-56631	EFCAB2	-48713
chr12	14518796	14519076	1.63	1.40E-02	ATF7IP	325		
chr19	49999404	49999708	1.60	1.41E-02	RPS11	-78		
chr14	103572140	103572416	1.74	1.43E-02	CDC42BPB	-48536	TNFAIP2	-20386
chr2	173941046	173941353	2.18	1.43E-02	ZAK	635		
chr9	138853102	138853464	1.41	1.43E-02	UBAC1	-57		
chr13	52158332	52158595	1.47	1.44E-02	WDFY2	-20		
chr14	62129233	62129511	1.93	1.44E-02	HIF1A	-32747	PRKCH	340857
chr3	186250597	186250907	2.05	1.44E-02	DGKG	-170729	CRYGS	11415
chr16	89638488	89638732	2.13	1.45E-02	CPNE7	-3566		
chr17	3614240	3614739	2.36	1.45E-02	P2RX5	-14907	GSG2	-12707
chr3	181418270	181418534	1.99	1.46E-02	DNAJC19	-710872	SOX2	-11320
chr5	96271542	96271852	1.60	1.46E-02	LNPEP	-22459	ERAP2	60053
chr8	141474244	141474713	1.72	1.47E-02	CHRAC1	-46922	TRAPPC9	-5801
chr2	211035370	211035634	1.63	1.50E-02	ACADL	54713	RPE	168150
chr2	201983150	201983515	1.67	1.50E-02	CASP10	-64288	CFLAR	2517
chr19	45970816	45971064	1.43	1.50E-02	FOSB	-313		
chr11	93474542	93474921	1.75	1.51E-02	TAF1D	-70	C11orf54	-61
chr8	135703566	135703898	1.66	1.51E-02	ZFAT	21549		
chr11	67035896	67036195	1.77	1.52E-02	SSH3	-34873	ADRBK1	2141
chr2	192419826	192420102	1.75	1.52E-02	OBFC2A	-122897	MYO1B	309857
chr2	24148887	24149178	1.59	1.53E-02	ATAD2B	901		
chr16	2557069	2557329	1.86	1.53E-02	ATP6V0C	-6754	TBC1D24	32052
chr16	87843176	87843526	1.57	1.54E-02	KLHDC4	-43809	SLC7A5	59749
chr19	6502345	6502602	2.16	1.54E-02	TUBB4	-144		
chr3	188414919	188415237	2.13	1.54E-02	TPRG1	-474685	LPP	484357
chr1	42928752	42929028	1.93	1.55E-02	PPIH	-195158	PPCS	6717
chr20	48285064	48285322	2.05	1.57E-02	PTGIS	-100486	B4GALT5	45228
chr19	47616945	47617191	1.51	1.57E-02	ZC3H4	-59		
chr2	3643673	3643923	1.96	1.57E-02	ALLC	-61988	COLEC11	1161
chr19	1269529	1269821	1.63	1.57E-02	CIRBP	408		
chr12	31812094	31812389	1.61	1.58E-02	C12orf72	-367		
chr19	38469433	38469714	1.77	1.61E-02	SIPA1L3	71706	DPF1	245316
chr19	11375151	11375405	1.84	1.61E-02	DOCK6	-2121		
chr17	61851132	61851478	1.71	1.61E-02	DDX42	-262	CCDC47	-217
chr7	4721684	4721970	1.57	1.62E-02	FOXK1	-103		
chr18	51750879	51751490	1.63	1.65E-02	MBD2	-27		
chr18	45206303	45206562	1.92	1.65E-02	CORL2	-430879	SMAD2	251082

chr2	25524375	25524654	1.72	1.66E-02	POMC	-132956	DNMT3A	40259
chrX	1640560	1640832	1.83	1.67E-02	ASMTL	-68852	P2RY8	15341
chr11	62476956	62477323	1.61	1.68E-02	BSCL2	-94		
chr3	107241595	107241871	1.57	1.68E-02	BBX	-50		
chr11	119039283	119039572	1.80	1.68E-02	NLRX1	-12		
chr18	3178002	3178278	1.60	1.69E-02	LPIN2	-166195	MYOM1	41966
chr16	57578212	57578569	1.63	1.69E-02	GPR56	-75519	GPR114	1790
chr7	76831779	76832083	1.94	1.69E-02	FGL2	-2781		
chr1	221950093	221950393	1.47	1.70E-02	DUSP10	-34782	HHIPL2	771201
chr19	57900923	57901332	1.63	1.71E-02	ZNF548	-90		
chr13	41006172	41006469	1.67	1.72E-02	FOXO1	234413	COG6	776557
chr4	99850183	99850707	1.74	1.72E-02	EIF4E	-202		
chr22	29102904	29103161	2.31	1.73E-02	TTC28	-27180	CHEK2	34789
chr12	2113561	2113833	1.48	1.73E-02	DCP1B	-20		
chr19	56652148	56652566	1.50	1.74E-02	ZNF444	-199		
chr11	34018612	34018892	1.99	1.74E-02	LMO2	-104916	CAPRIN1	-54478
chr12	116997618	116997887	1.83	1.75E-02	MAP1LC3B2	567		
chr15	74676158	74676480	1.74	1.75E-02	CYP11A1	-16238	SEMA7A	49980
chr4	114486142	114486463	1.66	1.77E-02	CAMK2D	196780	ANK2	515518
chr4	1340485	1340960	1.47	1.78E-02	CRIPAK	-44617	MAEA	57051
chr9	135257500	135257902	1.82	1.78E-02	SETX	-27329	TTF1	24520
chr11	76383726	76384155	2.03	1.78E-02	LRRC32	-2897		
chr19	57874683	57874995	1.58	1.79E-02	ZNF547	-40		
chr20	50016951	50017199	1.73	1.80E-02	KCNG1	-377400	NFATC2	142183
chr1	145397743	145398052	1.73	1.80E-02	HFE2	-15293	NBPF10	104527
chr2	8617973	8618333	1.70	1.80E-02	ID2	-203831		
chr11	133765179	133765470	1.81	1.81E-02	SPATA19	-49933	IGSF9B	61555
chr19	49990617	49990994	1.62	1.81E-02	RPL13A	-59		
chr3	49275742	49276200	1.66	1.81E-02	KLHDC8B	66903	USP4	101565
chr4	81912085	81912361	1.72	1.82E-02	BMP3	-39896	FGF5	724481
chr6	28849079	28849380	2.16	1.84E-02	SCAND3	-294118	TRIM27	42538
chr2	54326952	54327280	1.64	1.84E-02	PSME4	-129139	ACYP2	-15294
chr7	105332176	105332430	1.64	1.84E-02	FLJ23834	-271354	RINT1	159771
chr18	72264980	72265220	1.78	1.86E-02	ZNF407	-77823	CNDP1	63408
chr12	90647603	90647952	1.50	1.87E-02	ATP2B1	-597934	EPYC	751025
chr21	26946216	26946470	1.74	1.87E-02	MRPL39	33458		
chr15	80748204	80748503	1.80	1.88E-02	FAM108C1	-239298	ARNT2	51662
chr7	138915719	138916057	1.48	1.89E-02	LUC7L2	-128746	TTC26	97398

chr9	88969046	88969518	1.58	1.90E-02	ZCCHC6	87		
chr9	124705839	124706132	1.65	1.91E-02	TTLL11	149899	DAB2IP	376587
chr19	58874148	58874389	1.24	1.92E-02	ZNF497	-149		
chr1	192485968	192486285	1.87	1.92E-02	RGS1	-58730	RGS21	200005
chr18	77748493	77748796	1.94	1.92E-02	TXNL4A	-113		
chr1	161500896	161501173	1.70	1.93E-02	HSPA6	6999	FCGR3A	18783
chr19	37019107	37019515	1.68	1.95E-02	ZNF260	-141		
chr17	30850294	30850676	1.71	1.95E-02	CDK5R1	36380	MYO1D	353417
chr22	19842367	19842616	1.57	1.96E-02	GNB1L	-30		
chr10	97457225	97457469	2.28	2.00E-02	TCTN3	-3447		
chr9	34098341	34098704	1.75	2.02E-02	DCAF12	28248	UBE2R2	281341
chr3	183892380	183892674	1.55	2.03E-02	AP2M1	-107		
chr1	207510558	207510865	1.92	2.03E-02	CR2	-116958	CD55	15895
chr19	41770121	41770361	1.43	2.03E-02	HNRNPUL1	121		
chr9	137828646	137828906	1.73	2.04E-02	OLFM1	-138313	FCN1	-18967
chr12	69202555	69202831	1.59	2.05E-02	MDM2	722		
chrX	129300076	129300352	1.41	2.05E-02	AIFM1	-406		
chr22	35806203	35806530	1.64	2.05E-02	RASD2	-130985	MCM5	10251
chr4	113656590	113656869	2.35	2.06E-02	ANK2	-314055	LARP7	98118
chr2	31392963	31393275	1.79	2.08E-02	GALNT14	-31548	CAPN14	47292
chr15	63767107	63767463	1.66	2.09E-02	CA12	-93210	USP3	-29525
chr19	55919532	55919816	1.58	2.10E-02	UBE2S	-349		
chr7	140098231	140098515	1.68	2.10E-02	SLC37A3	-62		
chr1	37754437	37754895	1.58	2.14E-02	GRIK3	-254822	ZC3H12A	-185453
chr3	143393026	143393503	1.78	2.15E-02	SLC9A9	174081	CHST2	554597
chr13	76270010	76270286	2.23	2.17E-02	LMO7	-64649	UCHL3	146221
chr17	77967381	77967689	2.01	2.18E-02	CBX4	-154322	TBC1D16	42112
chr3	186783139	186783621	1.96	2.20E-02	ST6GAL1	43715	RPL39L	73883
chr16	2014614	2015146	1.73	2.20E-02	RNF151	-1995	RPS2	-53
chr19	40790328	40790604	2.12	2.22E-02	AKT2	799		
chr17	75136447	75136784	1.59	2.22E-02	SEC14L1	-389		
chr9	139001495	139001808	1.48	2.23E-02	NACC2	-14521	LHX3	93352
chr12	123850010	123850490	1.73	2.23E-02	SETD8	-18454	SBNO1	-15262
chr2	177628634	177629120	1.56	2.24E-02	HNRNPA3	-448545	MTX2	494754
chr15	45684339	45684691	1.75	2.30E-02	GATM	-13535	SPATA5L1	-10004
chr3	57261593	57261903	1.70	2.30E-02	APPL1	-17		
chr7	11579966	11580244	1.47	2.30E-02	THSD7A	291719	PHF14	566589
chr12	56754086	56754396	2.12	2.31E-02	STAT2	-332		

chrX	30907547	30907859	1.46	2.31E-02	MAP3K7IP3	-192		
chr16	15684733	15685074	1.92	2.32E-02	KIAA0430	52105	C16orf45	156579
chr14	68973478	68973766	1.68	2.34E-02	ZFP36L1	286163	RAD51L1	687113
chr12	57916484	57916736	1.43	2.37E-02	DDIT3	-2310	MBD6	-49
chr16	2770946	2771228	1.89	2.37E-02	PRSS27	-535		
chr10	89622023	89622355	1.43	2.37E-02	PTEN	-1006		
chr3	38179941	38180312	1.67	2.39E-02	ACAA1	-1394	MYD88	158
chr5	118625963	118626373	1.58	2.40E-02	TNFAIP8	-65428		
chr14	104073491	104073767	1.73	2.40E-02	BAG5	-45015	KLC1	-21896
chr4	57844875	57845119	1.46	2.42E-02	C4orf14	-1171	POLR2B	-112
chr12	31835100	31835417	1.62	2.42E-02	C12orf72	22650	H3F3C	109916
chr19	48248590	48249049	1.58	2.42E-02	GLTSCR2	27		
chr9	31519020	31519348	1.72	2.45E-02	ACO1	-865417		
chr12	12509877	12510209	1.58	2.47E-02	MANSC1	-6874	DUSP16	205274
chr22	24186640	24186931	1.45	2.48E-02	SLC2A11	-13255	DERL3	-5587
chr12	46465990	46466266	1.68	2.48E-02	SFRS2IP	-81727	SLC38A1	197080
chr7	55602025	55602420	2.08	2.49E-02	VOPP1	37977	LANCL2	169082
chr2	46717781	46718038	1.86	2.51E-02	LOC388946	11206	ATP6V1E2	29186
chr12	43505304	43505697	1.70	2.51E-02	PRICKLE1	-628085	ADAMTS20	440223
chr19	1021054	1021314	1.68	2.52E-02	C19orf6	-43		
chr6	37016447	37016816	1.72	2.52E-02	PIM1	-121290	FGD2	43209
chr16	85981462	85981825	1.61	2.54E-02	FOXF1	-562489	IRF8	48870
chr9	95900677	95901103	1.48	2.54E-02	NINJ1	-4320		
chr7	97829705	97830048	1.71	2.54E-02	BHLHA15	-11691	LMTK2	93680
chr7	26008325	26008599	1.45	2.55E-02	NPVF	-740357	NFE2L3	-183385
chr5	173314625	173314939	1.64	2.57E-02	CPEB4	-549		
chr11	65341611	65342055	1.54	2.57E-02	SSSCA1	3890	KCNK7	21634
chr17	77778326	77778611	2.02	2.57E-02	CBX8	-7579	CBX4	34744
chr6	87864941	87865304	1.59	2.58E-02	ZNF292	-146		
chr12	123911571	123911914	1.56	2.58E-02	RILPL2	9521	SETD8	43039
chr9	36993852	36994236	1.64	2.60E-02	PAX5	40432	MELK	421139
chr12	121991439	121991742	1.60	2.60E-02	KDM2B	27329	RNF34	153689
chr6	347291	347589	1.90	2.60E-02	IRF4	-44312	DUSP22	55339
chr11	119194234	119194510	1.86	2.61E-02	RNF26	-10865	MCAM	-6532
chr2	65662835	65663126	1.49	2.62E-02	SPRED2	-3325		
chr3	98241744	98242055	1.81	2.62E-02	CLDND1	10		
chr4	170679024	170679334	1.77	2.66E-02	C4orf27	-86		
chr2	27712528	27712902	1.57	2.66E-02	IFT172	-144		

chr15	74258214	74258474	1.92	2.67E-02	STOML1	26291	LOXL1	39555
chr19	50143480	50143880	1.32	2.67E-02	SCAF1	-1702	RRAS	-280
chr3	184190884	184191150	1.57	2.67E-02	EPHB3	-88570	CHRD	93156
chr2	3652966	3653312	1.34	2.67E-02	ALLC	-52647	COLEC11	10502
chr9	136810392	136810997	1.72	2.68E-02	SARDH	-207216	VAV2	46751
chr3	183003651	183003895	1.72	2.69E-02	B3GNT5	32741	MCF2L2	142082
chr1	169863015	169863495	1.77	2.70E-02	SCYL3	-179		
chr20	49996954	49997264	1.78	2.70E-02	KCNG1	-357434	NFATC2	162149
chr19	39109559	39110008	1.63	2.72E-02	MAP4K1	-1141	EIF3K	62
chr17	80477152	80477557	1.44	2.72E-02	FOXK2	-239		
chr5	88567331	88567575	1.93	2.73E-02	MEF2C	-388151		
chr8	8860072	8860481	1.59	2.73E-02	ERI1	-37		
chr20	30946819	30947216	1.80	2.74E-02	ASXL1	865		
chr20	5591481	5591949	1.52	2.75E-02	RP5-1022P6.2	-43		
chr20	43996860	43997236	1.82	2.76E-02	SYS1	5347	TP53TG5	9909
chr15	93375657	93375960	1.76	2.76E-02	FAM174B	-176778	CHD2	-67742
chr8	28198601	28198918	1.84	2.77E-02	PNOC	24111	ZNF395	45217
chr15	90640809	90641056	1.81	2.77E-02	IDH2	4775	ZNF710	96181
chr19	1240306	1240578	1.55	2.78E-02	C19orf26	-2452	ATP5D	-1307
chr22	20861694	20861939	1.74	2.78E-02	MED15	-69		
chr11	64667776	64668020	2.23	2.79E-02	PPP2R5B	-24282	EHD1	-21707
chr7	135665538	135665869	1.79	2.80E-02	MTPN	-3500		
chr1	27018675	27019154	1.59	2.81E-02	ARID1A	-3607		
chr12	56617985	56618274	1.49	2.81E-02	RNF41	-2426	OBFC2B	5
chr22	45683886	45684130	1.52	2.81E-02	FAM118A	-21777	UPK3A	3119
chr4	122722290	122722600	1.46	2.82E-02	EXOSC9	-27		
chr8	125539513	125539793	1.47	2.82E-02	TATDN1	11676	RNF139	52645
chr2	185235797	185236046	1.87	2.84E-02	ZNF804A	-227171		
chr13	78271707	78271986	1.48	2.84E-02	SCEL	162038	EDNRB	277817
chr9	126973085	126973365	1.72	2.85E-02	NEK6	-47018	LHX2	199336
chr4	693233	693488	1.66	2.85E-02	MFSD7	-10388	PCGF3	-6212
chr19	1344752	1345060	1.55	2.86E-02	MUM1	-10070	EFNA2	58738
chr9	129233377	129233635	1.76	2.86E-02	LMX1B	-143242	FAM125B	144378
chr19	38495222	38495505	1.52	2.87E-02	SIPA1L3	97496	DPF1	219526
chr19	46149333	46149649	1.69	2.89E-02	EML2	-765		
chr2	8443585	8443977	1.66	2.90E-02	ID2	-378203		
chr18	44702571	44702882	1.62	2.91E-02	IER3IP1	18		
chr15	85956718	85957036	1.54	2.93E-02	AKAP13	33006	KLHL25	381312

chr12	122418084	122418455	1.77	2.94E-02	BCL7A	-41591	PSMD9	91624
chr7	50356947	50357223	1.72	2.94E-02	IKZF1	12707	FIGNL1	161003
chr1	226850600	226851053	2.00	2.95E-02	ITPKB	76049	C1orf95	114326
chr10	329790	330149	1.72	2.95E-02	ZMYND11	149546	DIP2C	405638
chr19	57751858	57752137	1.87	2.96E-02	ZNF805	-55		
chr20	60982602	60982940	1.75	2.96E-02	CABLES2	-432		
chr8	33342543	33342841	1.80	2.96E-02	MAK16	7		
chr11	122588123	122588477	1.66	2.97E-02	CRTAM	-120955	UBASH3B	61902
chr5	179233797	179234073	1.25	2.97E-02	MGAT4B	17		
chr22	19132163	19132427	1.54	2.98E-02	DGCR14	-105		
chr19	58898346	58898806	1.86	3.01E-02	RPS5	-60		
chrX	16737506	16737799	1.72	3.02E-02	SYAP1	-102		
chr5	114545006	114545389	1.83	3.06E-02	TRIM36	-28955	PGGT1B	53371
chr11	70116501	70116809	1.60	3.07E-02	PPFIA1	-168		
chr11	117198377	117198653	1.60	3.07E-02	CEP164	-56		
chr11	118938086	118938587	1.62	3.09E-02	VPS11	-156		
chr10	18948071	18948470	1.47	3.11E-02	ARL5B	-42		
chr19	16739889	16740429	1.57	3.11E-02	MED26	-1144		
chr9	130541566	130541831	1.51	3.11E-02	SH2D3C	-651		
chr3	150102718	150103003	1.90	3.12E-02	PFN2	-414120	TSC22D2	-23927
chr17	37910349	37910942	1.73	3.13E-02	GRB7	16459	IKZF3	109795
chr5	60954403	60954679	2.02	3.13E-02	KIF2A	-647448	FLJ37543	20905
chr12	26266250	26266537	1.80	3.15E-02	BHLHE41	11609	RASSF8	154425
chr7	127291850	127292174	1.51	3.16E-02	SND1	-190		
chr15	45747090	45747400	1.66	3.18E-02	C15orf48	24482	SLC30A4	67757
chr1	101713480	101713817	1.60	3.18E-02	S1PR1	11344	OLFM3	749141
chrX	41593198	41593520	1.75	3.18E-02	GPR82	9951	CASK	188928
chr7	150132816	150133177	1.57	3.19E-02	GIMAP8	-14965	ZNF775	56591
chr7	43689449	43689725	1.95	3.20E-02	STK17A	66895	C7orf44	79496
chr18	43547064	43547523	1.81	3.22E-02	PSTPIP2	104956	SIGLEC15	141749
chr15	65596987	65597487	1.88	3.23E-02	PARP16	-18219	IGDCC3	73141
chr8	126656743	126657124	1.87	3.23E-02	TRIB1	214371	FAM84B	913532
chr5	141704537	141704786	1.76	3.25E-02	SPRY4	-42		
chr19	3180384	3180660	1.90	3.26E-02	NCLN	-5353	S1PR4	1756
chr11	116969151	116969401	1.50	3.26E-02	SIK3	-283		
chr3	191846733	191847007	1.92	3.26E-02	FGF12	279968	PYDC2	667918
chr20	42356889	42357169	1.73	3.28E-02	GTSF1L	-1387		
chr6	149079653	149080024	1.90	3.28E-02	MAP3K7IP2	-559224	UST	11568

chr3	185973360	185973716	1.49	3.29E-02	ETV5	-146637	DGKG	106485
chr2	242275754	242276108	1.96	3.29E-02	FARP2	-19780	SEPT2	21208
chr12	40620078	40620361	1.86	3.29E-02	CNTN1	-466138	LRRK2	1407
chr2	174739636	174739995	1.69	3.31E-02	SP3	90247	CDCA7	520255
chr19	37157631	37157920	2.07	3.31E-02	ZNF461	-37		
chr9	139981167	139981445	1.56	3.32E-02	MAN1B1	-111		
chr17	73030502	73030746	1.71	3.33E-02	ATP5H	12450	ICT1	21844
chr11	111957345	111957643	1.66	3.33E-02	SDHD	-77	TIMM8B	-35
chr15	81586179	81586681	1.89	3.33E-02	STARD5	30094	IL16	97211
chr2	89145311	89145802	1.58	3.33E-02	O1/O11 and JK2	-745005	RPIA	154381
chr5	43020159	43020490	1.54	3.34E-02	C5orf39	20122	GHR	596299
chr15	79299325	79299749	1.48	3.37E-02	CTSH	-62117	RASGRF1	83678
chr15	43398150	43398478	1.71	3.38E-02	UBR1	-28		
chr2	133339586	133339925	2.66	3.38E-02	LYPD1	88725	GPR39	165609
chr4	77069450	77069760	1.64	3.38E-02	NUP54	50		
chr1	118148259	118148566	1.52	3.38E-02	WDR3	-323959	MAN1A2	238328
chr17	34417496	34417954	1.83	3.39E-02	CCL3	-219		
chr7	154997318	154997588	1.68	3.40E-02	INSIG1	-92033	HTR5A	134907
chr1	157670603	157670913	1.68	3.41E-02	FCRL3	-111		
chr10	105439172	105439470	1.60	3.43E-02	SH3PXD2A	175843	NEURL	185586
chr11	61809576	61809913	1.67	3.44E-02	INCENP	-81700	FTH1	-74613
chr17	36754846	36755186	1.39	3.44E-02	SNIP	7167	ARHGAP23	141372
chr11	118991679	118992278	1.72	3.45E-02	HINFP	-309		
chr9	130565141	130565453	1.85	3.46E-02	FPGS	143		
chr7	106511546	106511791	1.92	3.48E-02	PRKAR2B	-173509	PIK3CG	5745
chr2	74408858	74409146	1.66	3.48E-02	MOBKL1B	-3007		
chr12	107303961	107304358	1.59	3.51E-02	C12orf23	-45384	RIC8B	135761
chr1	27019498	27019838	1.67	3.52E-02	ARID1A	-2854		
chr5	61601492	61601914	1.64	3.54E-02	KIF2A	-286		
chr19	1042231	1042517	1.87	3.54E-02	HMHA1	-24800	ABCA7	2272
chr19	41282983	41283357	1.55	3.57E-02	RAB4B	-1001		
chr3	13373716	13374108	1.66	3.59E-02	IQSEC1	-259295	NUP210	87897
chr20	30286925	30287260	1.77	3.60E-02	BCL2L1	23563	COX4I2	61402
chr11	118766376	118766628	1.56	3.61E-02	CXCR5	11961	BCL9L	15111
chr2	16894387	16894648	1.71	3.61E-02	VSNL1	-827289	FAM49A	-47422
chr2	122407417	122407789	1.61	3.63E-02	CLASP1	-551		
chr3	111258605	111258881	1.91	3.64E-02	CD96	-2183		
chr19	48833364	48833747	1.44	3.64E-02	EMP3	4927	TMEM143	33630

chr17	41560993	41561411	1.75	3.64E-02	DHX8	-132		
chr19	50922823	50923113	1.77	3.64E-02	SPIB	773		
chr19	50181531	50181910	1.43	3.64E-02	C19orf76	-10221	PRMT1	1225
chr22	42840118	42840394	1.29	3.69E-02	SERHL	-56329	NFAM1	-11855
chr6	6856817	6857068	1.63	3.69E-02	RREB1	-251245	LY86	268009
chr14	55569036	55569327	1.71	3.70E-02	LGALS3	-26690	SOCS4	75338
chr12	31899759	31900051	1.58	3.71E-02	H3F3C	45270	C12orf72	87296
chr19	1940718	1941140	1.35	3.72E-02	CSNK1G2	-232		
chr5	178971532	178971808	1.63	3.74E-02	RUFY1	-5901	AX747985	20222
chr3	193411658	193411977	1.78	3.75E-02	HES1	-442116	OPA1	100885
chr2	96829524	96829834	1.63	3.76E-02	DUSP2	-18500	STARD7	44894
chr12	124086516	124086962	1.55	3.81E-02	DDX55	67		
chr19	2289755	2290036	1.62	3.83E-02	AX747191	-103		
chr2	25433119	25433475	1.74	3.85E-02	POMC	-41738	DNMT3A	131477
chrX	100662887	100663303	1.69	3.86E-02	HNRNPH2	-96	GLA	-94
chr1	247095432	247095691	1.56	3.88E-02	AHCTF1	-13883	ZNF695	75793
chr12	58008446	58008722	1.43	3.89E-02	SLC26A10	-5109	GEFT	4621
chr3	184925708	184926186	1.56	3.89E-02	EHHADH	45890	VPS8	396016
chr12	133485053	133485509	1.44	3.90E-02	CHFR	-21077	ZNF605	47587
chr3	156202119	156202411	1.76	3.90E-02	SSR3	70670	KCNAB1	363928
chr1	51972262	51972596	1.75	3.90E-02	TTC39A	-161644	EPS15	12566
chr14	55541364	55541676	1.90	3.92E-02	LGALS3	-54352	SOCS4	47676
chr2	61198551	61198808	1.90	3.92E-02	PEX13	-46132	REL	89928
chr10	23012178	23012493	1.67	3.94E-02	ARMC3	-204618	PIP4K2A	-8833
chr7	94250062	94250316	1.59	3.94E-02	SGCE	35332	CASD1	111019
chr12	9822126	9822429	1.70	3.94E-02	CLEC2D	-31		
chr7	25894723	25894976	1.67	3.96E-02	NPVF	-626745	NFE2L3	-296997
chr11	126081432	126081799	1.43	3.96E-02	RPUSD4	-29		
chr4	40843048	40843330	1.54	3.99E-02	NSUN7	91275	APBB2	373446
chr20	23342426	23342792	1.41	4.02E-02	GZF1	-2391		
chr9	136688795	136689147	1.56	4.03E-02	SARDH	-85492	VAV2	168475
chr5	175843488	175843793	1.49	4.07E-02	CLTB	-101		
chr3	37000195	37000519	1.63	4.10E-02	TRANK1	-97946	EPM2AIP1	34438
chr18	51884078	51884664	1.54	4.11E-02	STARD6	-3428	C18orf54	-2526
chr13	99830900	99831243	1.55	4.13E-02	DOCK9	-92412	UBAC2	-21607
chr4	3049239	3049571	1.55	4.13E-02	HTT	-27003	GRK4	84062
chr5	94417210	94417529	2.14	4.14E-02	MCTP1	202909	ANKRD32	462979
chr19	58816527	58816818	1.51	4.14E-02	ZSCAN22	-21712	ZNF8	26355

chr19	45873813	45874067	1.50	4.15E-02	ERCC2	-95		
chr19	49375397	49375791	1.37	4.15E-02	PLEKHA4	-3710	PPP1R15A	-55
chr11	65208358	65208734	1.66	4.15E-02	SCYL1	-84002	FRMD8	54505
chr13	46739676	46739943	1.67	4.18E-02	CPB2	-60599	LCP1	16649
chr17	74264344	74264632	1.60	4.18E-02	RNF157	-28098	QRICH2	39273
chr19	1904345	1904734	1.65	4.20E-02	ADAT3	-833	SCAMP4	-833
chr2	231454261	231454526	1.70	4.20E-02	CAB39	-123163	SP100	173523
chr16	64355850	64356132	1.62	4.21E-02	CDH11	799928		
chr11	125462243	125462722	1.64	4.24E-02	STT3A	-256		
chr17	61819095	61819508	1.45	4.24E-02	STRADA	-87		
chr7	129133958	129134280	1.81	4.24E-02	NRF1	-117436	AHCYL2	269255
chr8	10850883	10851170	1.58	4.25E-02	SOX7	-263005	XKR6	207848
chr3	51534108	51534380	1.40	4.26E-02	VPRBP	-243		
chr16	89877726	89878056	1.41	4.27E-02	FANCA	5174	ZNF276	89939
chr7	148680681	148680957	1.81	4.27E-02	EZH2	-99405	PDIA4	44963
chr15	72371295	72371611	1.67	4.29E-02	MYO9A	38969	NR2E3	268559
chr20	37504150	37504624	1.83	4.31E-02	FAM83D	-50568	PPP1R16B	70039
chrX	15692861	15693105	1.58	4.31E-02	CA5BP	-56		
chr19	50145034	50145426	1.53	4.33E-02	RRAS	-1830	SCAF1	-152
chr20	4970261	4970574	1.61	4.35E-02	RASSF2	-174649	SLC23A2	20521
chr1	167691015	167691291	1.74	4.35E-02	MPZL1	-34		
chr9	114688854	114689123	1.51	4.35E-02	UGCG	29783	SUSD1	248567
chr17	79068751	79068998	2.29	4.36E-02	BAIAP2	59928	AATK	70997
chr21	37692286	37692612	1.41	4.37E-02	MORC3	-38		
chr12	44152567	44152863	1.44	4.38E-02	PUS7L	-153	IRAK4	-32
chr19	40791325	40791849	1.79	4.39E-02	AKT2	-322		
chr3	46120363	46120639	1.63	4.39E-02	XCR1	-51522	CCR1	129331
chr5	123919511	123919780	1.75	4.41E-02	ZNF608	161219		
chr22	32026371	32026647	1.69	4.42E-02	PISD	301		
chr2	216979416	216979744	1.58	4.44E-02	XRCC5	5560	MARCH4	257170
chr17	73150478	73150903	1.54	4.47E-02	HN1	84		
chr7	22486638	22486915	1.80	4.48E-02	RAPGEF5	-90244	MGC87042	53021
chr19	40949071	40949371	1.50	4.49E-02	SERTAD1	-17289	SERTAD3	1061
chr16	17510572	17511153	1.65	4.52E-02	XYLT1	53875		
chr5	143569483	143569727	1.81	4.52E-02	KCTD16	19168		
chr7	129254928	129255194	1.61	4.53E-02	NRF1	3506	UBE2H	337728
chr7	111202508	111202826	1.30	4.54E-02	IMMP2L	-320		
chr11	10879223	10879499	1.57	4.56E-02	ZBED5	259		

chr1	235114097	235114454	1.63	4.56E-02	IRF2BP2	-369005	TOMM20	177980
chr4	175849845	175850335	1.64	4.56E-02	ADAM29	10581		
chr15	89019848	89020168	2.00	4.56E-02	MRPS11	9324	DET1	69904
chr13	99852761	99853130	1.50	4.58E-02	UBAC2	267		
chr2	27593234	27593510	1.67	4.58E-02	EIF2B4	-453	SNX17	-17
chr7	108210019	108210439	1.52	4.61E-02	DNAJB9	-127	THAP5	-62
chr6	14910954	14911190	1.76	4.61E-02	JARID2	-335455	CD83	793207
chr8	109455439	109455862	1.52	4.61E-02	TTC35	-202		
chr1	212588205	212588654	1.60	4.63E-02	TMEM206	-163		
chr22	19159033	19159308	1.58	4.65E-02	GSC2	-21375	SLC25A1	7130
chr3	61880738	61881044	1.74	4.65E-02	PTPRG	333648	FEZF2	478299
chr5	124082973	124083293	1.64	4.65E-02	ZNF608	-2268		
chr6	27640152	27640438	1.87	4.66E-02	ZNF184	-199398	HIST1H2BL	135414
chr8	29087058	29087392	2.00	4.66E-02	KIF13B	33385	HMBOX1	339314
chr4	110736415	110736765	1.50	4.68E-02	GAR1	-76		
chr2	37458671	37458930	1.66	4.68E-02	CEBPZ	-61	C2orf56	27
chr1	206304394	206304639	1.80	4.69E-02	C1orf186	-15870	CTSE	-12942
chr10	79789213	79789520	1.50	4.72E-02	RPS24	-4151	POLR3A	-108
chr7	36325905	36326181	1.76	4.74E-02	ANLN	-103389	EEPD1	133207
chr4	3079608	3079907	1.61	4.76E-02	RGS12	-236116	HTT	3350
chr15	75082393	75082722	1.66	4.77E-02	LMAN1L	-22636	CSK	8133
chr4	57253444	57253777	1.61	4.79E-02	AASDH	27		
chr19	6801862	6802106	1.64	4.80E-02	EMR1	-85598	VAV1	29262
chr14	81425829	81426123	1.58	4.82E-02	TSHR	4107	GTF2A1	261318
chr19	45829431	45829689	1.63	4.83E-02	CKM	-3426		
chr1	241696247	241696497	1.81	4.85E-02	KMO	692		
chr2	64751222	64751470	1.41	4.85E-02	AFTPH	-119		
chr19	10535983	10536268	1.68	4.91E-02	PDE4A	4793	KEAP1	77928
chr22	29168514	29168856	1.46	4.91E-02	XBP1	27875	HSCB	30642
chr17	73179007	73179268	1.61	4.93E-02	HN1	-28363	NUP85	-22459
chr17	1726758	1727067	1.90	4.94E-02	SMYD4	6262	SERPINF1	61654
chr22	40573740	40574068	1.76	4.96E-02	TNRC6B	-25		
chr18	9475243	9475617	1.31	4.96E-02	RALBP1	-100		
chr11	118757427	118757813	1.60	4.99E-02	CXCR5	3079	BCL9L	23993
chr6	119763935	119764260	1.74	4.99E-02	MAN1A1	-93172		
chr7	116502331	116502677	1.40	4.99E-02	CAPZA2	-59		
chr12	25539066	25539310	1.34	4.99E-02	KRAS	-135334	IFLTD1	167029
chr2	33497086	33497375	1.70	4.99E-02	RASGRP3	-241711	LTBP1	324839

Unique to GCB								
chr10	71712594	71712830	0.06	2.96E-28	H2AFY2	-99645	COL13A1	151068
chr2	234135329	234135613	0.06	7.65E-26	ATG16L1	-24746	INPP5D	210435
chr1	115885979	115886289	0.06	9.24E-25	VANGL1	-298440	NGF	-5277
chr3	48700231	48700578	0.08	5.64E-24	TMEM89	-41216	CELSR3	9576
chr7	26137839	26138170	0.08	6.41E-24	NPVF	-869900	NFE2L3	-53842
chr18	5978408	5978718	0.07	1.45E-23	TMEM200C	-86460	L3MBTL4	436347
chr2	120939650	120939886	0.11	2.87E-23	TMEM185A	41216	EPB41L5	169099
chr3	34304544	34304854	0.08	1.03E-22	PDCD6IP	464633		
chr1	110166411	110166721	0.09	1.60E-22	GSTM4	-32132	AMPD2	3290
chr1	54821700	54821900	0.07	1.62E-22	SSBP3	50292	MRPL37	155960
chr20	62918217	62918527	0.09	5.99E-22	PCMTD2	31324		
chr16	24113514	24113763	0.16	9.77E-22	CACNG3	-153237	PRKCB	266339
chr3	12758081	12758318	0.14	1.03E-21	RAF1	-52500	TMEM40	42608
chr3	15259407	15259587	0.13	1.94E-21	CAPN7	11764	SH3BP5	114607
chr3	11623776	11624007	0.04	3.73E-21	VGLL4	61506	ATG7	309882
chrX	48754406	48754649	0.13	9.81E-21	PQBP1	-667	TIMM17B	898
chr16	4511348	4511608	0.12	1.36E-20	NMRAL1	13418	DNAJA3	35620
chr15	37392240	37392440	0.09	1.88E-19	MEIS2	364		
chr19	12991419	12991701	0.17	3.07E-19	DNASE2	775		
chr19	55737020	55737291	0.09	3.55E-19	PTPRH	-16282	TMEM86B	3476
chr10	112483725	112484035	0.09	6.08E-19	PDCD4	-147716	RBM20	79725
chr7	99967329	99967665	0.11	1.24E-18	PILRA	-3571		
chr2	145427230	145427540	0.13	1.70E-18	ZEB2	-149469		
chr14	95953760	95954020	0.10	2.14E-18	GLRX5	-47433	C14orf49	-11717
chr5	692251	692561	0.10	6.27E-18	TPPP	1104	CEP72	80001
chr7	16955788	16955988	0.10	6.94E-18	AHR	-382388	AGR3	-34275
chrX	33146367	33146620	0.16	9.04E-18	DMD	83179		
chr7	10032	10276	0.07	1.09E-17	FAM20C	-182815		
chr7	22213949	22214149	0.14	1.09E-17	CDCA7L	-228507	RAPGEF5	182484
chr6	35231302	35231612	0.09	1.13E-17	DEF6	-34138	ZNF76	3947
chr5	130967696	130967937	0.18	2.04E-17	RAPGEF6	3112	CDC42SE2	368115
chr12	56696472	56696683	0.14	2.48E-17	CS	-2403		
chr19	49652931	49653277	0.08	3.34E-17	HRC	5577	PPFIA3	30441
chr1	198607119	198607364	0.20	4.33E-17	PTPRC	-895		
chr14	75699356	75699666	0.13	4.94E-17	TMED10	-56162	FOS	-45970
chr13	50074476	50074786	0.14	5.07E-17	PHF11	4830	RCBTB1	85088
chr4	186401482	186401734	0.16	6.78E-17	CCDC110	-8695	PDLIM3	55104

chr18	10033	10281	0.13	7.52E-17	USP14	-148326		
chr10	121920644	121920954	0.11	9.70E-17	PPAPDC1A	-295667	SEC23IP	268576
chr2	66661998	66662258	0.15	1.16E-16	MEIS1	-404		
chr20	62918583	62918893	0.12	1.30E-16	PCMTD2	31690		
chr11	83393368	83393633	0.09	2.36E-16	CCDC90B	-396124		
chr11	76093316	76093590	0.21	3.66E-16	PRKRIR	-1573		
chr19	12917616	12917868	0.23	4.78E-16	RNASEH2A	314		
chr8	56922647	56922947	0.17	5.01E-16	RPS20	64343	LYN	130411
chr1	20730816	20731142	0.07	5.18E-16	CAMK2N1	81749	VWA5B1	113567
chr6	139337557	139337793	0.22	7.23E-16	HECA	-118574	REPS1	-28277
chr13	49942913	49943149	0.15	7.98E-16	CAB39L	32704	CDADC1	120928
chr17	30466148	30466381	0.25	1.18E-15	RHOT1	-3208		
chr1	214601269	214601505	0.18	1.35E-15	PTPN14	123255	SMYD2	146822
chr2	122246953	122247263	0.18	1.35E-15	TFCP2L1	-204330	CLASP1	159944
chr10	115720472	115720838	0.06	1.46E-15	ADRB1	-83151	NHLRC2	106235
chr19	44084545	44084855	0.17	2.21E-15	XRCC1	-4970	BC071811	4
chr1	26437509	26437745	0.15	2.23E-15	PDIK1L	-641		
chrX	48857998	48858260	0.19	2.33E-15	GRIPAP1	546		
chr6	159477130	159477421	0.19	2.38E-15	FNDC1	-113153	TAGAP	-11092
chr15	64889460	64889683	0.14	2.43E-15	ZNF609	97953	OAZ2	105890
chr10	94003108	94003334	0.14	4.83E-15	CPEB3	47623	BTAF1	319485
chr16	25771331	25771567	0.17	5.17E-15	HS3ST4	68102		
chr6	45485797	45486033	0.13	5.46E-15	SUPT3H	-140245	CLIC5	562170
chr19	42721166	42721408	0.16	6.23E-15	ZNF526	-3205	DEDD2	526
chr2	89534569	89534879	0.05	6.28E-15	O1/O11 and JK2	-355838	RPIA	543548
chr12	109747504	109747748	0.17	8.47E-15	FOXN4	-601		
chr18	12703863	12704102	0.16	8.69E-15	CEP76	-1280	PSMG2	919
chr20	61069632	61069911	0.22	1.02E-14	SLCO4A1	-204025	GATA5	-18746
chr13	41226762	41227008	0.15	1.95E-14	FOXO1	13849	COG6	997121
chr2	143629080	143629327	0.16	5.21E-14	LRP1B	-739934	KYNU	-5991
chr8	48109337	48109690	0.16	5.24E-14	CEBPD	541212		
chr20	57738831	57739132	0.24	6.57E-14	SLMO2	-121081	ZNF831	-27093
chr16	788144	788388	0.20	7.67E-14	NARFL	2731	HAGHL	11000
chr7	101497750	101498060	0.19	8.96E-14	SH2B2	-430500	CUX1	37023
chrX	19002520	19002756	0.21	9.16E-14	PHKA2	-158		
chr2	75062388	75062700	0.22	1.08E-13	POLE4	-123231	HK2	2762
chr5	110221682	110221992	0.30	1.21E-13	TSLP	-185553	SLC25A46	147083
chr14	52118144	52118434	0.14	1.55E-13	FRMD6	-287		

chr8	42128468	42128704	0.23	1.69E-13	IKBKB	-243		
chr12	113772146	113772390	0.30	1.75E-13	SLC24A6	657		
chr10	76349059	76349369	0.14	1.75E-13	MYST4	-237165	ADK	438249
chr12	49594931	49595241	0.22	2.28E-13	TUBA1B	-69782	TUBA1C	-63779
chr8	29743626	29743862	0.18	2.36E-13	DUSP4	-535559	TMEM66	196905
chr17	70506592	70506828	0.16	2.58E-13	SOX9	389549	SLC39A11	582143
chr7	10395515	10395825	0.11	3.42E-13	NDUFA4	584143		
chr1	3636696	3636932	0.17	3.51E-13	KIAA0495	27072	TP73	67685
chr1	6550557	6550820	0.19	3.65E-13	TNFRSF25	-24434	PLEKHG5	6795
chr11	118799894	118800111	0.24	3.84E-13	UPK2	-27023	BCL9L	-18390
chr3	44040583	44040864	0.06	3.86E-13	C3orf23	-339220	ABHD5	308349
chr5	154137060	154137370	0.23	4.10E-13	LARP1	2326	C5orf4	92998
chr17	61926317	61926582	0.13	4.35E-13	SMARCD2	-6099	CSH2	24639
chr12	56615074	56615316	0.21	4.64E-13	OBFC2B	-2930	RNF41	509
chrX	37853094	37853442	0.04	5.80E-13	SYTL5	-39519	CXorf27	3198
chrX	43513845	43514151	0.21	6.05E-13	MAOA	-1411		
chr8	41435838	41436082	0.13	6.38E-13	AGPAT6	253		
chr21	40875065	40875375	0.22	6.59E-13	B3GALT5	-154034	SH3BGR	51440
chr19	19517209	19517445	0.22	7.07E-13	GATAD2A	20685	TSSK6	109142
chrX	49020301	49020537	0.23	7.24E-13	PLP2	-7854	MAGIX	1238
chr18	74207095	74207584	0.15	7.85E-13	ZNF236	-328776	ZNF516	-32243
chr11	57103015	57103215	0.21	8.09E-13	P2RX3	-2834	SSRP1	236
chr12	95833495	95833816	0.19	8.55E-13	METAP2	-34166	VEZT	222134
chr9	119522900	119523228	0.18	9.20E-13	TRIM32	73483	ASTN2	654253
chr13	30989760	30990009	0.14	9.23E-13	KATNAL1	-108722	HMGB1	50196
chr16	70099879	70100140	0.23	9.26E-13	PDXDC2	-75899	PDPR	-47519
chr7	99789329	99789639	0.16	1.04E-12	PVRIG	-27388	STAG3	13946
chr15	38943978	38944251	0.18	1.09E-12	THBS1	-929165	RASGRP1	-87108
chr5	148737146	148737406	0.16	1.10E-12	PCYOX1L	-294		
chr1	6052055	6052343	0.22	1.13E-12	NPHP4	332		
chr5	147162255	147162608	0.22	1.19E-12	JAKMIP2	-180		
chr11	66034752	66035025	0.17	1.29E-12	RAB1B	-1167		
chr7	99790549	99790859	0.12	1.42E-12	PVRIG	-26168	STAG3	15166
chr6	150325657	150326005	0.19	2.24E-12	RAET1L	20837	ULBP1	40688
chr1	24872202	24872512	0.11	2.33E-12	C1orf130	-10245	RCAN3	42970
chr3	31986673	31986932	0.21	2.42E-12	OSBPL10	36435	STT3B	412312
chrX	48768356	48768599	0.32	2.50E-12	SLC35A2	757		
chr15	42411146	42411456	0.21	2.60E-12	PLA2G4D	-24549	PLA2G4F	37509

chr1	206732659	206732903	0.25	2.85E-12	RASSF5	51902	LGTN	53123
chr14	56046523	56046833	0.24	3.04E-12	KTN1	-247		
chr18	658156	658416	0.20	3.50E-12	C18orf56	54	TYMS	682
chr7	134832157	134832395	0.11	3.61E-12	TMEM140	-490		
chr1	179335556	179335866	0.19	3.68E-12	SOAT1	72694	NPHS2	209373
chr3	126105525	126105835	0.23	3.84E-12	KLF15	-29444	ZXDC	89082
chr19	10203844	10204154	0.25	4.18E-12	ANGPTL6	9426	RDH8	80074
chr7	142503655	142503965	0.17	5.16E-12	EPHB6	-48982	TCRBC2	9443
chr11	45921389	45921683	0.17	5.54E-12	C11orf94	7297	MAPK8IP1	14334
chr17	7137432	7137680	0.26	6.14E-12	DVL2	307		
chr10	73868615	73868925	0.19	7.21E-12	SPOCK2	-19980	ASCC1	106924
chr8	30659229	30659539	0.25	7.26E-12	PPP2CB	10968	UBXN8	57694
chr1	19922891	19923128	0.30	8.40E-12	C1orf151	-457		
chr2	87303435	87303715	0.22	8.72E-12	PLGLB1	-744031	LOC285074	-25886
chr12	105745389	105745627	0.17	9.70E-12	APPL2	-115500	NUAK1	788303
chr14	22902192	22902592	0.17	1.09E-11	TCRDV2	-25697	TRA	426628
chr18	78016187	78016436	0.13	1.13E-11	PARD6G	-10915		
chr11	62474952	62475202	0.26	1.15E-11	GNG3	-37		
chr17	62031864	62032174	0.27	1.18E-11	CD79B	-22315	SCN4A	18259
chr7	25016715	25017025	0.13	1.20E-11	DFNA5	-219787	OSBPL3	2890
chr1	78295660	78295896	0.10	1.21E-11	NEXN	-58422	FAM73A	50469
chr12	113569378	113569642	0.27	1.21E-11	RASAL1	4511	DTX1	73848
chr11	67123796	67124181	0.28	1.22E-11	POLD4	-2972		
chr13	115077452	115077784	0.19	1.23E-11	ZNF828	-2347		
chr18	2570806	2571116	0.24	1.32E-11	NDC80	-549	METTL4	528
chr1	155349484	155349735	0.04	1.47E-11	RUSC1	58970	ASH1L	182714
chrX	131623217	131623527	0.21	1.50E-11	MBNL3	-49030	HS6ST2	472051
chr1	31364698	31365008	0.14	1.83E-11	LAPTM5	-134170	SDC3	16627
chr10	64028340	64028607	0.20	1.84E-11	RTKN2	-8		
chr10	90640603	90640857	0.22	2.26E-11	STAMBPL1	704		
chr22	24129644	24129919	0.19	2.39E-11	SMARCB1	632		
chr14	22984483	22984784	0.14	2.39E-11	TCRDV2	56545	DAD1	73509
chr1	87916339	87916649	0.24	2.47E-11	LMO4	122343		
chr5	66300262	66300540	0.24	2.77E-11	MAST4	175797	CD180	192216
chr20	55819946	55820277	0.08	2.98E-11	BMP7	21595	TFAP2C	615754
chr18	12420992	12421308	0.14	3.54E-11	SLMO1	13255	SPIRE1	236762
chr17	56199307	56199617	0.24	3.60E-11	OR4D1	-33053	DYNLL2	38682
chr19	55678138	55678521	0.07	3.62E-11	TNNI3	-9230	SYT5	13390

chr2	219741653	219741969	0.27	3.66E-11	WNT10A	-3444		
chr17	58042180	58042424	0.18	3.85E-11	RNFT1	-185		
chr4	38818140	38818551	0.26	4.07E-11	TLR1	-11934	TLR6	12812
chr10	111768443	111768753	0.20	4.12E-11	ADD3	887		
chr17	7743288	7743624	0.05	4.66E-11	KDM6B	221		
chrX	129304624	129304890	0.22	4.96E-11	AIFM1	-4949	RAB33A	-1016
chr19	49649470	49649720	0.13	5.00E-11	HRC	9086	PPFIA3	26932
chr1	150244732	150245042	0.24	5.35E-11	APH1A	-3355	C1orf54	-315
chr13	115078731	115079005	0.19	6.17E-11	ZNF828	-1097		
chr10	112201476	112201796	0.12	6.78E-11	SMNDC1	-136929	DUSP5	-55989
chr4	41258692	41259005	0.05	7.22E-11	UCHL1	-49		
chr6	160114377	160114665	0.15	7.34E-11	SOD2	-168		
chrX	49022733	49023065	0.10	7.74E-11	PLP2	-5374	MAGIX	3718
chr12	52517568	52518033	0.27	7.85E-11	C12orf44	54043	KRT80	67983
chr10	103879292	103879602	0.25	9.23E-11	LDB1	763		
chr1	9910301	9910611	0.26	9.23E-11	CLSTN1	-25906	CTNNBIP1	59860
chr16	31880808	31881125	0.28	1.02E-10	ZNF267	-4112		
chr2	87303889	87304199	0.26	1.02E-10	PLGLB1	-743562	LOC285074	-26355
chr12	32286586	32286902	0.17	1.05E-10	FGD4	-368297	BICD1	26559
chr6	35104031	35104341	0.20	1.12E-10	TCP11	5001	ANKS1A	247148
chr9	135285272	135285594	0.23	1.13E-10	TTF1	-3212		
chr2	219151818	219152070	0.34	1.14E-10	TMBIM1	5336	PNKD	16829
chr12	120104298	120104534	0.22	1.25E-10	PRKAB1	-1345		
chr1	161691191	161691501	0.28	1.27E-10	FCRLB	-1097		
chr11	111749425	111749661	0.32	1.31E-10	C11orf1	-405	FDXACB1	610
chr8	10857080	10857416	0.20	1.37E-10	SOX7	-269226	XKR6	201627
chr22	46984311	46984628	0.15	1.41E-10	CELSR1	-51403	GRAMD4	-38178
chr2	71357871	71358229	0.20	1.49E-10	MCEE	-656	MPHOSPH10	606
chr7	73089113	73089423	0.24	1.57E-10	VPS37D	7094	DNAJC30	8513
chr6	57037344	57037594	0.13	1.65E-10	BAG2	365		
chr12	54149300	54149610	0.27	1.65E-10	HOXC13	-183121	CALCOCO1	-28148
chr12	121974430	121974630	0.18	1.75E-10	KDM2B	44390	RNF34	136628
chrX	48595889	48596179	0.21	1.76E-10	GATA1	-48948	SUV39H1	40903
chr1	193506539	193506849	0.33	1.78E-10	B3GALT2	-350951		
chr1	100817777	100817973	0.20	1.81E-10	CDC14A	-148		
chr14	76657670	76657980	0.26	1.96E-10	ESRRB	-179865	C14orf118	39566
chr2	86946773	86947180	0.25	1.99E-10	RMND5A	-437		
chr1	1677446	1678011	0.06	1.99E-10	CDC2L1	-21954	NADK	32180

chr19	1490482	1490760	0.14	2.05E-10	REEP6	-544	PCSK4	-214
chrX	40856435	40856745	0.27	2.49E-10	MED14	-261786	USP9X	-88298
chr2	86947262	86947406	0.29	2.50E-10	RMND5A	-80		
chr5	40679466	40679666	0.27	2.57E-10	PTGER4	-466		
chr13	44849644	44849954	0.23	2.81E-10	ENOX1	-646186	SERP2	-98179
chr14	68750691	68751001	0.24	2.85E-10	RAD51L1	464337	ZFP36L1	508939
chr12	32086529	32086839	0.24	2.96E-10	BICD1	-173501	H3F3C	-141509
chr16	29678019	29678270	0.17	3.14E-10	QPRT	-12296	SPN	3845
chr6	157138635	157138871	0.16	3.69E-10	ARID1B	39667	C6orf35	606040
chrX	47479226	47479470	0.20	3.83E-10	SYN1	-92		
chr2	66794118	66794354	0.23	3.92E-10	ETAA1	-830206	MEIS1	131704
chr3	57262507	57262799	0.18	4.30E-10	APPL1	888		
chr5	180671155	180671425	0.20	4.37E-10	GNB2L1	-384		
chr7	8009504	8009814	0.25	4.47E-10	GLCCI1	1236	ICA1	292023
chrY	22897227	22897552	0.17	4.51E-10	RPS4Y2	-20564	EIF1AY	159779
chr17	36955628	36955938	0.19	4.61E-10	PIP4K2B	375		
chr17	7476709	7477098	0.23	5.19E-10	CD68	-5901	EIF4A1	1302
chr6	31797511	31797821	0.23	5.38E-10	HSPA1B	2154	NEU1	33043
chr12	67010166	67010476	0.26	5.69E-10	GRIP1	62604	HELB	313965
chr20	56477369	56477679	0.21	5.72E-10	PMEPA1	-192493	PPP4R1L	406971
chr6	155739544	155739953	0.24	5.72E-10	TFB1M	-104123	NOX3	37288
chr6	32121583	32121909	0.13	5.92E-10	PRRT1	-2026	PPT2	445
chr6	33291061	33291297	0.20	6.06E-10	DAXX	-386		
chr1	182614109	182614419	0.28	6.23E-10	RGS16	-40716	RGS8	27803
chr6	35300175	35300485	0.19	6.33E-10	PPARD	-10005	DEF6	34735
chr8	29743294	29743530	0.24	6.70E-10	DUSP4	-535227	TMEM66	197237
chr1	19229014	19229386	0.06	7.65E-10	ALDH4A1	93		
chr7	99792882	99793192	0.24	7.67E-10	PVRIG	-23835	STAG3	17499
chr1	36621275	36621606	0.20	8.72E-10	MAP7D1	-362		
chr7	98667788	98668024	0.18	9.84E-10	SMURF1	73817	TRRAP	191793
chr17	48980951	48981430	0.27	1.05E-09	TOB1	-39778	SPAG9	217035
chr7	68958982	68959278	0.23	1.08E-09				
chr6	160183220	160183599	0.30	1.16E-09	ACAT2	421		
chr7	7222453	7222763	0.28	1.25E-09	C1GALT1	362		
chr1	205717949	205718259	0.35	1.35E-09	SLC45A3	-68474	NUCKS1	1257
chr1	204283440	204283689	0.32	1.40E-09	GOLT1A	-100345	PPP1R15B	97379
chr22	37988561	37988798	0.34	1.47E-09	GGA1	-15823	LGALS2	-12656
chr15	74340719	74340955	0.27	1.54E-09	ISLR2	-81906	PML	53823

chr8	61824308	61824549	0.29	1.62E-09	RLBP1L1	-376096	CHD7	233090
chr4	153589026	153589262	0.33	1.68E-09	FBXW7	-132959	TMEM154	12047
chr2	230999416	230999652	0.30	1.93E-09	SLC16A14	-65915	SP110	85293
chr7	40873618	40873930	0.18	1.94E-09	C7orf10	699199	INHBA	868932
chr9	119526035	119526345	0.21	1.99E-09	TRIM32	76609	ASTN2	651127
chr2	89985855	89986381	0.20	2.01E-09	O1/O11 and JK2	95556		
chr8	79428243	79428561	0.15	2.08E-09	PKIA	66		
chr14	100070576	100070844	0.22	2.20E-09	HHIPL1	-40770	CCNK	122971
chr7	140396235	140396490	0.27	2.31E-09	NDUFB2	-118		
chr2	217277312	217277556	0.21	2.38E-09	SMARCAL1	-39		
chr1	147013117	147013364	0.32	2.42E-09	BCL9	59		
chr1	10125	10395	0.17	2.43E-09	OR4F5	-58831		
chr8	23077251	23077561	0.37	2.47E-09	TNFRSF10D	-55866	TNFRSF10A	5233
chr16	30196400	30196644	0.27	2.57E-09	GIYD2	-8695	CORO1A	1596
chr7	90338955	90339231	0.14	2.67E-09	PFTK1	381		
chr19	18108599	18108966	0.15	2.67E-09	KCNN1	46672	IL12RB1	88959
chr3	71191606	71191864	0.38	2.68E-09	FOXP1	441405		
chr18	10446	10756	0.17	2.69E-09	USP14	-147882		
chr17	62770406	62770716	0.23	2.70E-09	SMURF2	-112175	LOC146880	7061
chrX	47880760	47881070	0.23	2.76E-09	SPACA5	17181	ZNF630	49593
chr20	52551088	52551398	0.28	2.79E-09	ZNF217	-351536	BCAS1	136061
chr12	56224351	56224638	0.16	2.80E-09	DNAJC14	-1997		
chr17	40594279	40594589	0.26	2.81E-09	PTRF	-19160	ATP6V0A1	-16428
chr17	16189918	16190228	0.32	3.03E-09	CENPV	66739	PIGL	69564
chr5	65220511	65220821	0.35	3.08E-09	ERBB2IP	-1718		
chr2	42793198	42793508	0.28	3.36E-09	MTA3	-2318		
chr6	154582828	154583064	0.16	3.42E-09	IPCEF1	94954	OPRM1	222503
chr5	153569905	153570215	0.25	3.71E-09	GALNT10	-235		
chr19	18633440	18633702	0.31	4.02E-09	ELL	-634		
chr1	38259466	38259776	0.23	4.14E-09	MANEAL	-153		
chr7	129073919	129074158	0.31	4.17E-09	NRF1	-177516	AHCYL2	209175
chr20	633826	634136	0.29	4.18E-09	SRXN1	-91		
chr7	136088390	136088700	0.23	4.54E-09	CHRM2	-465287	MTPN	-426341
chr1	87221549	87221859	0.33	4.94E-09	SH3GLB1	51447	SEP15	158403
chr7	27774243	27774509	0.26	4.99E-09	HIBADH	-71774	TAX1BP1	-5362
chr5	177557800	177558070	0.20	5.04E-09	N4BP3	17379	NHP2	23026
chr14	96119085	96119395	0.30	5.10E-09	TCL1A	61293	GLRX5	117917
chr3	10029004	10029240	0.39	5.23E-09	TMEM111	-600		

chr2	191513273	191513583	0.31	5.66E-09	NAB1	-420		
chr19	58326051	58326292	0.29	5.68E-09	ZNF552	109		
chr5	79571246	79571556	0.25	6.11E-09	SPZ1	-44389	SERINC5	-19531
chr1	52869227	52869537	0.26	6.19E-09	PRPF38A	-837	ORC1L	749
chr1	26437965	26438233	0.29	6.33E-09	PDIK1L	-169		
chr11	47290634	47290942	0.21	6.41E-09	MADD	-411		
chr13	67460285	67460595	0.32	6.41E-09	PCDH9	344028		
chrX	129065452	129065688	0.25	6.64E-09	BCORL1	-51099	UTP14A	25411
chr11	911036	911449	0.28	6.72E-09	CHID1	-369		
chr8	62844218	62844463	0.21	7.51E-09	NKAIN3	-317160	ASPH	-217142
chr17	55629539	55629816	0.36	7.81E-09	MSI2	295747	MRPS23	297721
chr17	58428455	58428765	0.29	7.89E-09	USP32	40976	CA4	201308
chr1	204476873	204477183	0.39	8.07E-09	PIK3C2B	-17554	MDM4	-8483
chr19	42927265	42927591	0.12	8.58E-09	CNFN	-32984	LIPE	4150
chr2	97628179	97628489	0.27	8.98E-09	SEMA4C	-92599	FAHD2B	132248
chr11	34460697	34460933	0.29	9.02E-09	CAT	337		
chr3	150127782	150128029	0.30	9.28E-09	EIF2A	-136668	TSC22D2	1118
chr12	6570515	6570825	0.35	9.82E-09	VAMP1	9173	TAPBPL	9493
chr6	152623349	152623585	0.26	1.07E-08	SYNE1	335067	NR3A1	459735
chr2	39102818	39103084	0.35	1.10E-08	DHX57	70		
chr9	100749836	100750146	0.27	1.26E-08	NANS	-68691	ANP32B	4502
chr1	15944398	15944727	0.31	1.30E-08	DDI2	493		
chr14	21944571	21944826	0.38	1.31E-08	TOX4	-636	RAB2B	433
chr3	31975586	31975896	0.18	1.36E-08	OSBPL10	47497	STT3B	401250
chr6	33378494	33378730	0.32	1.37E-08	PHF1	-161		
chr16	82608986	82609231	0.16	1.43E-08	MPHOSPH6	-405280	CDH13	-51469
chr9	112676641	112676954	0.30	1.44E-08	PALM2-AKAP2	134221	TXN	341980
chr12	54892217	54892527	0.32	1.48E-08	NCKAP1L	877		
chr15	45997735	45998009	0.31	1.58E-08	SQRDL	70616		
chr8	70025944	70026188	0.24	1.71E-08	SULF1	-352793		
chr7	99699522	99699809	0.31	1.81E-08	MCM7	-239	AP4M1	536
chr8	42995232	42995544	0.30	1.83E-08	HGSNAT	-204		
chr7	64712221	64712658	0.29	1.86E-08	ERV3	-245319	ZNF92	-126328
chr17	4851991	4852227	0.31	2.01E-08	ENO3	-2277	PFN1	-284
chr16	15736655	15736936	0.32	2.04E-08	KIAA0430	213		
chr7	27963370	27963606	0.19	2.10E-08	TAX1BP1	183750	JAZF1	256949
chr9	112888351	112888897	0.26	2.15E-08	TXN	130154	PALM2-AKAP2	346047
chr10	99078707	99079055	0.12	2.18E-08	FRAT1	-141		

chr11	67417513	67417829	0.41	2.18E-08	ACY3	459		
chr6	13262009	13262274	0.29	2.21E-08	TBC1D7	66628	PHACTR1	544309
chr19	55690657	55690967	0.26	2.25E-08	SYT5	908		
chr10	99094457	99094798	0.15	2.27E-08	FRAT2	-170		
chr18	63533	63843	0.16	2.38E-08	USP14	-94795		
chr11	63535179	63535489	0.34	2.42E-08	C11orf95	779		
chr17	76151007	76151293	0.23	2.42E-08	SYNGR2	-13521	C17orf99	8716
chr10	63656598	63656882	0.30	2.47E-08	ARID5B	-4703		
chrX	49969025	49969358	0.35	2.55E-08	AKAP4	-3528		
chr8	59562280	59562516	0.33	2.59E-08	NSMAF	9568	SDCBP	96670
chr7	43691290	43691622	0.26	2.83E-08	STK17A	68764	C7orf44	77627
chr1	68201644	68201883	0.34	2.98E-08	GADD45A	50881	GNG12	97378
chr8	107669769	107670093	0.14	3.08E-08	ABRA	112541	OXR1	387458
chr1	198633340	198633650	0.39	3.13E-08	PTPRC	25358		
chr2	166450833	166451143	0.26	3.16E-08	CSRNP3	22098	GALNT3	199815
chr22	26828588	26828898	0.32	3.27E-08	ASPHD2	3463	HPS4	46808
chr9	126959523	126959901	0.31	3.37E-08	NEK6	-60531	LHX2	185823
chr1	31224958	31225268	0.33	3.41E-08	MATN1	-28681	LAPTM5	5570
chrX	47225338	47225843	0.14	3.62E-08	ZNF157	-4408		
chr12	121974117	121974353	0.29	3.84E-08	KDM2B	44685	RNF34	136333
chr3	196949745	196949981	0.26	3.86E-08	MFI2	-193177	DLG1	75584
chr9	141054147	141054457	0.23	4.04E-08	TUBB4Q	-15149	CACNA1B	282061
chr3	42201200	42201653	0.36	4.06E-08	TRAK1	68681	CCK	104972
chr12	54120982	54121218	0.37	4.11E-08	CALCOCO1	207		
chr5	137203311	137203621	0.26	4.76E-08	MYOT	-79		
chr11	75479467	75479791	0.16	4.85E-08	DGAT2	-149		
chr7	101497208	101497547	0.24	5.10E-08	SH2B2	-431027	CUX1	36496
chr14	88470807	88471117	0.39	5.41E-08	GPR65	-534		
chr3	127278870	127279180	0.37	5.44E-08	TPRA1	30543	PLXNA1	571523
chr15	38369137	38369447	0.29	5.77E-08	SPRED1	-175760	TMCO5A	141834
chrX	129220928	129221238	0.30	5.80E-08	ELF4	23605	BCORL1	104414
chr18	21596620	21596930	0.40	5.86E-08	CABYR	-122180	LAMA3	327213
chr7	4792927	4793173	0.22	5.89E-08	KIAA0415	-22214	FOXK1	71120
chr12	50643628	50643938	0.37	6.14E-08	LASS5	-82686	LIMA1	33570
chr7	92219100	92219534	0.24	6.29E-08	PEX1	-61472	CDK6	246624
chr17	21189891	21190201	0.22	6.53E-08	KCNJ12	-89653	MAP2K3	2078
chr15	42787190	42787434	0.29	6.68E-08	SNAP23	-523		
chr1	64060072	64060382	0.34	7.17E-08	ROR1	-179463	PGM1	1280

chr15	98689770	98689970	0.37	7.20E-08	IGF1R	-502891		
chr11	121246623	121246936	0.30	7.23E-08	SORL1	-76181	SC5DL	83392
chr12	106721294	106721604	0.25	7.49E-08	CKAP4	-79736	POLR3B	-29987
chr12	111538597	111538907	0.24	7.49E-08	SH2B3	-305000	CUX2	66923
chr6	132816731	132817041	0.39	8.07E-08	MOXD1	-94222	STX7	17451
chr7	101532054	101532290	0.18	8.15E-08	SH2B2	-396233	CUX1	71290
chr3	20227417	20227662	0.35	8.20E-08	SGOL1	143		
chr20	57365394	57365633	0.26	8.70E-08	GNAS	-49281	NPEPL1	97652
chr18	23878291	23878589	0.23	9.09E-08	TAF4B	72031	KCTD1	250060
chr9	695233	695543	0.25	9.18E-08	DMRT1	-146302	KANK1	190685
chr7	5862418	5862889	0.15	9.34E-08	OCM	-57775	RNF216	-41362
chr9	27386852	27387162	0.33	9.90E-08	IFNK	-137305	C9orf11	-89870
chr19	2398779	2399089	0.29	1.01E-07	TMPRSS9	9150	TIMM13	28941
chrX	48755245	48755595	0.17	1.02E-07	TIMM17B	6	PQBP1	225
chr19	2678175	2678485	0.22	1.07E-07	GNG7	24416	GADD45B	202195
chr12	49628968	49629212	0.26	1.10E-07	TUBA1B	-103786	TUBA1C	-29775
chr7	67079885	67080195	0.16	1.18E-07	TYW1	618223		
chr19	35819821	35820060	0.30	1.21E-07	CD22	-138		
chr8	38644496	38644745	0.18	1.33E-07	TACC1	-101		
chr8	96960636	96960946	0.30	1.37E-07	GDF6	212229	PLEKHF2	814753
chr19	53898358	53898682	0.37	1.45E-07	ZNF765	123		
chr8	17013247	17013528	0.13	1.49E-07	ZDHHC2	-448		
chr20	25667403	25667715	0.33	1.51E-07	NANP	-62911	ZNF337	9910
chr15	70600760	70601077	0.28	1.55E-07	TLE3	-210663	UACA	454931
chr12	56769384	56769772	0.33	1.59E-07	APOF	-12995	TIMELESS	73622
chrX	12976609	12976919	0.20	1.60E-07	TMSB4X	-16461	TLR8	52006
chr1	53924470	53924721	0.17	1.61E-07	DMRTB1	-476		
chr1	228325969	228326279	0.30	1.61E-07	GUK1	-1805		
chr15	40972387	40972732	0.28	1.61E-07	RAD51	-14767	CASC5	86113
chr12	13254096	13254406	0.28	1.66E-07	EMP1	-95351	GSG1	-5542
chr14	45603423	45603709	0.18	1.66E-07	FANCM	-1570	FKBP3	443
chr7	104909474	104909803	0.18	1.69E-07	SRPK2	119702	MLL5	255002
chrX	118827060	118827458	0.31	1.74E-07	SEPT6	74		
chr1	24235411	24235680	0.27	1.88E-07	FUCA1	-40725	CNR2	4271
chr10	5882911	5883324	0.21	1.88E-07	FBXO18	-53231	GDI2	-27606
chr11	85021591	85021830	0.17	2.17E-07	DLG2	316603		
chr17	40439364	40439627	0.31	2.17E-07	STAT5A	-69		
chr1	43609182	43609500	0.30	2.18E-07	SLC2A1	-184494	EBNA1BP2	28900

chr19	59118741	59119051	0.24	2.25E-07	MZF1	-33954		
chr17	64487385	64487695	0.25	2.26E-07	CACNG5	-385808	PRKCA	188614
chr11	65667816	65668126	0.25	2.33E-07	FOSL1	26		
chr16	85587297	85587533	0.36	2.37E-07	KIAA0182	-59509	KIAA0513	526005
chr16	31707001	31707311	0.27	2.44E-07	CSDAP1	-126311	ZNF720	-17410
chr11	71938757	71938993	0.38	2.45E-07	INPPL1	2993	PHOX2A	16345
chr19	44123790	44124031	0.30	2.60E-07	ZNF428	103		
chr3	46968392	46968775	0.28	2.69E-07	NBEAL2	-52589	PTH1R	49348
chrX	155260155	155260399	0.19	2.76E-07	WASH1	10310		
chr1	9970711	9970955	0.33	2.79E-07	CTNNBIP1	-517		
chr19	17866760	17867059	0.31	2.91E-07	B3GNT3	-39009	MAP1S	36607
chr21	45196135	45196419	0.35	2.99E-07	CSTB	-18		
chr10	126336445	126336755	0.30	2.99E-07	FAM53B	96330	LHPP	186188
chr12	44200270	44200580	0.33	3.23E-07	TWF1	-247		
chr4	74088760	74088996	0.41	3.27E-07	COX18	-153406	ANKRD17	35624
chr22	18539055	18539367	0.37	3.29E-07	MICAL3	-31886	PEX26	-21475
chr17	7741785	7742095	0.41	3.38E-07	KDM6B	-1295		
chr15	44580003	44580313	0.46	3.47E-07	CASC4	-771		
chr10	131733033	131733283	0.24	3.53E-07	EBF3	28933	MGMT	467704
chr9	130667483	130667793	0.34	3.55E-07	ST6GALNAC6	-5767	ST6GALNAC4	11667
chr10	112033977	112034333	0.21	3.61E-07	SMNDC1	30552	MXI1	66792
chr1	43833963	43834273	0.30	3.80E-07	ELOVL1	-419		
chr19	3854321	3854582	0.35	4.05E-07	MATK	-68037	ZFR2	14575
chr12	1704438	1704682	0.29	4.08E-07	FBXL14	-1229		
chr6	15586142	15586459	0.24	4.08E-07	DTNBP1	76970	JARID2	339774
chrX	48858635	48858940	0.21	4.13E-07	GRIPAP1	-113		
chr8	59988715	59989087	0.35	4.24E-07	NSMAF	-416935	TOX	42866
chr1	11538921	11539200	0.25	4.44E-07	PTCHD2	-234		
chr16	24550502	24550810	0.19	4.44E-07	RBBP6	-252		
chr9	98268179	98268453	0.33	4.63E-07	FANCC	-188325	PTCH1	2515
chr2	179313645	179313955	0.30	4.72E-07	DFNB59	-2363	PRKRA	2158
chr12	65059309	65059509	0.34	4.75E-07	RASSF3	55116	GNS	93817
chr21	34863791	34864119	0.22	5.15E-07	DNAJC28	68		
chr6	159638769	159639104	0.32	5.36E-07	FNDC1	48508	SOD2	475416
chr17	75720699	75721009	0.23	5.40E-07	TNRC6C	-279464	SEPT9	443362
chr10	116286633	116286991	0.19	5.46E-07	AFAP1L2	-122297	ABLIM1	131246
chr19	36869827	36870107	0.37	5.48E-07	ZFP14	-11094	ZFP82	39583
chr17	56065722	56066032	0.33	5.68E-07	VEZF1	-262		

chr16	3301220	3301476	0.21	5.79E-07	ZNF200	-15892	MEFV	5279
chr7	101458285	101458650	0.38	5.89E-07	CUX1	-2414		
chr1	44302037	44302482	0.21	6.12E-07	ARTN	-99394	ST3GAL3	129042
chr7	7310813	7311113	0.21	6.20E-07	C1GALT1	88717	COL28A1	264497
chr1	84211947	84212257	0.39	6.26E-07	TTLL7	252731		
chr5	156644554	156645081	0.40	6.47E-07	CYFIP2	-48273	ITK	36911
chr19	56150477	56150713	0.37	6.78E-07	ZNF581	-4391	ZNF580	-1797
chr16	89227745	89228055	0.27	6.83E-07	CDH15	-10263	ACSF3	67646
chr6	36087072	36087334	0.21	6.84E-07	MAPK13	-11059	MAPK14	91749
chr4	42634724	42635034	0.31	6.89E-07	ATP8A1	24243	SHISA3	235023
chr16	17463298	17463747	0.33	7.23E-07	XYLT1	101215	AK310228	998526
chr9	130769962	130770331	0.46	7.77E-07	DPM2	-69384	NAIF1	59452
chr10	75571515	75571792	0.13	7.89E-07	NDST2	-65		
chr6	107437824	107438134	0.44	7.96E-07	C6orf203	88572	PDSS2	342800
chr1	93426096	93426348	0.30	8.15E-07	FAM69A	857		
chr2	153574505	153574741	0.30	8.35E-07	PRPF40A	-648	ARL6IP6	200
chr8	125538875	125539125	0.36	8.89E-07	TATDN1	12329	RNF139	51992
chr15	48624076	48624360	0.20	8.91E-07	DUT	597		
chr3	9438296	9438596	0.16	9.07E-07	THUMPD3	33729	LHFPL4	157040
chrX	48534942	48535252	0.26	9.25E-07	WAS	-7089	WDR13	79196
chr1	160807327	160807656	0.31	9.30E-07	CD244	25153	LY9	41564
chr17	37123525	37123809	0.33	9.52E-07	LASP1	97555	PLXDC1	184235
chrX	53024645	53025136	0.20	9.81E-07	GPR173	-53615	FAM156B	-38938
chr10	95255914	95256224	0.32	1.02E-06	CEP55	-300		
chr6	111804323	111804779	0.31	1.03E-06	REV3L	-119		
chr5	1313088	1313398	0.34	1.12E-06	TERT	-18081	CLPTM1L	31759
chr9	6757103	6757384	0.32	1.23E-06	KDM4C	-397		
chr16	29972998	29973319	0.18	1.26E-06	TMEM219	-192		
chr3	105975433	105975743	0.38	1.30E-06	CBLB	-387701		
chrX	47420671	47420999	0.19	1.40E-06	ARAF	257		
chr10	27530621	27530931	0.42	1.43E-06	ACBD5	-968		
chr1	32757334	32757619	0.15	1.44E-06	HDAC1	-231		
chr17	77774363	77774673	0.37	1.49E-06	CBX8	-3628		
chr19	35739208	35739934	0.06	1.49E-06	LSR	12		
chrX	47077788	47078220	0.24	1.49E-06	PCTK1	-111		
chr17	73043144	73043411	0.29	1.52E-06	ATP5H	-204	KCTD2	-1
chr5	40755289	40755607	0.37	1.55E-06	TTC33	624		
chr3	4544799	4545177	0.38	1.55E-06	BHLHE40	-476109	ITPR1	9954

chr17	62963768	62964078	0.29	1.58E-06	LRRC37A3	-48935	GNA13	88997
chr19	40790908	40791261	0.31	1.63E-06	AKT2	180		
chr1	24648820	24649130	0.41	1.68E-06	NIPAL3	-93270	GRHL3	3094
chr5	137088520	137088830	0.31	1.68E-06	KLHL3	-16896	HNRNPA0	1364
chr7	5436160	5436509	0.27	1.68E-06	TNRC18	26842	SLC29A4	113774
chr7	10979778	10980088	0.36	1.72E-06	NDUFA4	-120		
chr3	55760355	55760672	0.39	1.78E-06	WNT5A	-239183	ERC2	741877
chr11	72525088	72525447	0.19	1.79E-06	ATG16L2	-183		
chr2	27603503	27603757	0.42	1.82E-06	ZNF513	-37		
chr14	39736295	39736619	0.21	1.87E-06	CTAGE5	129		
chr2	166532001	166532350	0.34	1.87E-06	CSRNP3	103286	GALNT3	118627
chr15	102520957	102521270	0.23	1.95E-06	WASH2P	8010		
chr2	113299430	113299787	0.21	1.95E-06	POLR1B	117		
chr6	112408759	112409069	0.39	1.95E-06	TUBE1	-163		
chr3	133292566	133292876	0.32	2.02E-06	CDV3	287		
chr1	208037246	208037556	0.47	2.03E-06	CD34	47282	CD46	111999
chr3	152005787	152006038	0.24	2.13E-06	MBNL1	-11281	SUCNR1	414476
chr1	39456727	39456994	0.13	2.14E-06	AKIRIN1	-55		
chr1	35667672	35668010	0.38	2.14E-06	ZMYM4	-66727	SFPQ	-9098
chr6	41990137	41990562	0.30	2.15E-06	CCND3	-80798	TAF8	-27901
chr2	46925604	46925927	0.26	2.19E-06	SOCS5	-333		
chr6	43214327	43214637	0.37	2.36E-06	SLC22A7	-51516	TTBK1	3260
chr12	1703905	1704236	0.23	2.40E-06	FBXL14	-740		
chr3	32726271	32726557	0.20	2.42E-06	CNOT10	-284		
chr22	50690637	50691039	0.31	2.43E-06	HDAC10	-1004		
chr16	81897414	81897848	0.36	2.43E-06	PLCG2	84701	SDR42E1	147462
chr16	23875022	23875474	0.35	2.47E-06	CACNG3	-391628	PRKCB	27948
chr16	85148564	85148800	0.40	2.48E-06	KIAA0182	-498242	KIAA0513	87272
chr6	36971819	36972193	0.36	2.66E-06	FGD2	-1417		
chr8	81050846	81051156	0.44	2.69E-06	ZBTB10	-347447	TPD52	-57991
chr7	43688046	43688282	0.38	2.92E-06	STK17A	65472	C7orf44	80919
chr8	99058274	99058596	0.32	2.94E-06	RPL30	-662		
chr3	33700526	33700930	0.12	2.95E-06	UBP1	-218831	CLASP2	59120
chr4	87927981	87928259	0.22	3.01E-06	AFF1	-33		
chrX	128977703	128978013	0.34	3.02E-06	ZDHHC9	-390		
chr8	49860126	49860436	0.25	3.08E-06	SNTG1	-964316	SNAI2	-26293
chr15	94774285	94774592	0.40	3.12E-06	MCTP2	-66991		
chr2	131863717	131864027	0.42	3.15E-06	POTEE	-112052	PLEKHB2	1452

chr7	2595109	2595556	0.13	3.28E-06	IQCE	-3299	C7orf27	-187
chr12	124272594	124272838	0.20	3.35E-06	ZNF664	-184954	DNAH10	25674
chr19	10339134	10339444	0.26	3.35E-06	DNMT1	-33534	S1PR2	2659
chr5	612106	612416	0.32	3.41E-06	CEP72	-144		
chr1	228651417	228651838	0.38	3.41E-06	RHOU	-219241	HIST3H2BB	5820
chr10	85974355	85974665	0.41	3.50E-06	LRIT2	10774	PCDH21	19993
chr22	26702618	26702935	0.41	3.50E-06	ASPHD2	-122503	SEZ6L	137297
chr16	22368857	22369103	0.30	3.53E-06	CDR2	16958	POLR3E	60239
chr4	104119522	104119838	0.20	3.57E-06	CENPE	-114		
chr16	11903923	11904289	0.18	3.64E-06	ZC3H7A	-27698	RSL1D1	41336
chr10	126228393	126228942	0.40	3.74E-06	LHPP	78256	FAM53B	204262
chr1	46016412	46016727	0.36	3.75E-06	AKR1A1	72		
chr18	46472798	46473108	0.31	3.76E-06	SMAD7	4128	KIAA0427	407526
chr11	47291467	47291746	0.30	3.93E-06	MADD	408		
chr5	1525163	1525473	0.42	4.41E-06	LPCAT1	-1242		
chr3	194406307	194406678	0.14	4.46E-06	LSG1	-13287	C3orf21	585402
chr19	49653516	49653954	0.16	4.49E-06	HRC	4946	PPFIA3	31072
chr16	85855209	85855596	0.38	4.61E-06	IRF8	-77371	COX4I1	22207
chr19	5864065	5864375	0.37	4.67E-06	FUT3	-12735	FUT5	6414
chr1	32404024	32404274	0.21	4.74E-06	PTP4A2	-161		
chr19	35738545	35739067	0.36	4.86E-06	LSR	-753		
chr21	34426895	34427139	0.30	4.92E-06	OLIG1	-15433	OLIG2	28778
chr11	85913828	85914198	0.43	4.93E-06	PICALM	-133905	EED	-41802
chr9	21802429	21802827	0.33	5.03E-06	MTAP	-7		
chr16	11633548	11633859	0.38	5.03E-06	LITAF	47102	C16orf75	194393
chr6	44215410	44215729	0.27	5.11E-06	HSP90AB1	721		
chr12	113623261	113623564	0.23	5.14E-06	DDX54	-129		
chrX	118708320	118708572	0.25	5.14E-06	UBE2A	-53		
chr12	56727550	56727855	0.31	5.14E-06	IL23A	-4960	PAN2	-174
chr2	86790038	86790376	0.23	5.14E-06	RNF103	60771	KDM3A	121623
chr10	135524491	135524801	0.29	5.26E-06	LOC653545	40968		
chr3	67096528	67096863	0.37	5.31E-06	KBTBD8	47969	SUCLG2	608342
chr11	568595	568844	0.22	5.44E-06	PHRF1	-7763	RASSF7	7270
chr11	3876027	3876263	0.43	5.66E-06	STIM1	-788		
chr13	43701994	43702242	0.23	5.82E-06	DNAJC15	104756	ENOX1	501495
chr20	36661581	36661903	0.29	5.84E-06	KIAA0406	91		
chr19	48972862	48973172	0.35	5.85E-06	CYTH2	552		
chr10	61645424	61645734	0.42	5.92E-06	SLC16A9	-175930	CCDC6	21239

chr9	112729287	112729747	0.43	6.10E-06	PALM2-AKAP2	186940	TXN	289261
chr1	85459809	85460119	0.33	6.13E-06	LPAR3	-101068	MCOLN2	2832
chr7	154794820	154795084	0.19	6.33E-06	PAXIP1	-270		
chr16	31119656	31120079	0.38	6.37E-06	BCKDK	206		
chr20	814201	814527	0.28	6.37E-06	FAM110A	8		
chr4	36252	36638	0.33	6.60E-06	ZNF718	-16782		
chr1	183442124	183442537	0.44	6.65E-06	SMG7	697		
chr2	12856699	12857009	0.35	6.71E-06	TRIB2	-144		
chr4	42629906	42630216	0.28	6.76E-06	ATP8A1	29061	SHISA3	230205
chr22	42765333	42765643	0.34	7.04E-06	TCF20	-154043	NFAM1	62913
chr15	65020385	65020696	0.45	7.30E-06	OAZ2	-25079	RBPMS2	47229
chr19	898707	899017	0.36	7.41E-06	MED16	-5644	C19orf22	14363
chr19	1650587	1650897	0.32	7.45E-06	TCF3	-456		
chr7	50161003	50161260	0.25	7.50E-06	IKZF1	-183246	ZPBP	-28272
chr12	50794224	50794599	0.12	7.71E-06	LARP4	-238		
chr17	8093329	8093749	0.32	7.91E-06	C17orf59	25		
chrX	47050006	47050289	0.18	7.98E-06	UBA1	-51		
chr20	57463993	57464307	0.39	8.08E-06	TH1L	-92161	GNAS	36114
chr1	6259746	6260042	0.24	8.13E-06	RPL22	-215		
chr8	54755717	54756085	0.42	8.17E-06	ATP6V1H	-51		
chr2	134877034	134877424	0.47	8.30E-06	NCKAP5	-551198	MGAT5	-134601
chr6	42018226	42018551	0.29	8.34E-06	TAF8	138		
chrX	19002911	19003250	0.42	8.67E-06	PHKA2	-601		
chr19	23945424	23945792	0.30	8.72E-06	ZNF681	-3915	RPSA	-208
chr7	87849144	87849676	0.18	8.76E-06	SRI	-17		
chr8	62661038	62661348	0.37	8.95E-06	NKAIN3	-500308	ASPH	-33994
chr16	81861600	81861923	0.38	9.49E-06	PLCG2	48832	SDR42E1	183331
chr20	3335141	3335415	0.29	9.65E-06	SLC4A11	-116905	ATRN	-116387
chr8	17941616	17941913	0.20	1.01E-05	ASAH1	114		
chr9	131903915	131904225	0.44	1.01E-05	METTL11A	-484365	PPP2R4	30826
chr11	44626928	44627238	0.30	1.05E-05	TSPAN18	-300875	CD82	39942
chr19	2679482	2679792	0.24	1.05E-05	GNG7	23109	GADD45B	203502
chr8	142106635	142106987	0.39	1.05E-05	PTK2	-95479	SLC45A4	131862
chr16	15068552	15068873	0.35	1.06E-05	PDXDC1	-120		
chr19	38826993	38827433	0.36	1.06E-05	CATSPERG	770		
chr7	16685467	16685777	0.10	1.06E-05	ANKMY2	-224	BZW2	-137
chr17	65990614	65990942	0.29	1.08E-05	C17orf58	-1013		
chr11	118796569	118796855	0.15	1.09E-05	UPK2	-30314	BCL9L	-15099

chr2	173145654	173145967	0.41	1.09E-05	DLX2	-178333	ITGA6	-146503
chr12	106696251	106696579	0.20	1.13E-05	POLR3B	-55021	CKAP4	-54702
chr1	31223742	31224069	0.40	1.15E-05	MATN1	-27474	LAPTM5	6777
chr14	21571909	21572250	0.18	1.17E-05	ZNF219	-4907		
chr6	114292271	114292720	0.17	1.18E-05	HDAC2	-142		
chr15	40074952	40075267	0.39	1.25E-05	FSIP1	-71		
chr7	101513004	101513525	0.37	1.32E-05	SH2B2	-415140	CUX1	52383
chr17	71228306	71228598	0.37	1.37E-05	C17orf80	-324		
chr7	130754046	130754356	0.24	1.37E-05	KLF14	-335341	MKLN1	-258394
chr2	230038186	230038496	0.17	1.38E-05	SPHKAP	-991980	PID1	97716
chr2	60779702	60780090	0.44	1.40E-05	BCL11A	737		
chr12	113377709	113378067	0.45	1.42E-05	OAS2	-38386	OAS3	1639
chr11	11283841	11284351	0.48	1.44E-05	ZBED5	-404476	CSNK2A1	90808
chr14	74208607	74208917	0.31	1.44E-05	PNMA1	-27634	C14orf43	45134
chr2	220408488	220408767	0.35	1.46E-05	CHPF	-141	TMEM198	-117
chr22	38710971	38711219	0.35	1.46E-05	TMEM184B	-42079	CSNK1E	2994
chr4	13486004	13486322	0.19	1.49E-05	RAB28	-174		
chr3	12791008	12791318	0.48	1.49E-05	RAF1	-85463	TMEM40	9645
chr6	36953935	36954215	0.26	1.52E-05	MTCH1	-126		
chr2	153574818	153575123	0.26	1.53E-05	PRPF40A	-996	ARL6IP6	548
chr16	25061945	25062255	0.49	1.57E-05	LCMT1	-60947	ARHGAP17	-35425
chr8	110551522	110551859	0.42	1.58E-05	EBAG9	-238		
chr14	31494508	31494818	0.42	1.58E-05	AP4S1	-20	STRN3	944
chr17	2614009	2614319	0.38	1.65E-05	KIAA0664	763		
chr10	23055140	23055514	0.42	1.65E-05	ARMC3	-161627	PIP4K2A	-51824
chr1	145122825	145123061	0.44	1.68E-05	NOTCH2NL	-86168	SEC22B	26536
chr16	10037187	10037456	0.47	1.68E-05	USP7	-979981	GRIN2A	238941
chr2	136889506	136889770	0.43	1.70E-05	THSD7B	-858824	CXCR4	-13913
chr22	24530155	24530465	0.32	1.73E-05	SUSD2	-47134	CABIN1	122545
chr10	43913604	43914109	0.33	1.74E-05	ZNF487	-18717	HNRNPF	-10558
chr15	72978202	72978555	0.38	1.77E-05	BBS4	-147		
chr4	186347079	186347430	0.14	1.83E-05	UFSP2	-116		
chr6	32590290	32590581	0.33	1.87E-05	HLA-DRB5	-92430	HLA-DQA1	-14747
chr7	97601394	97601773	0.36	1.91E-05	ASNS	-99730	OCM2	17832
chr2	239197484	239197813	0.25	1.92E-05	PER2	-442		
chr10	35379416	35379689	0.30	1.97E-05	CUL2	-259		
chr20	48247016	48247458	0.43	1.97E-05	PTGIS	-62530	B4GALT5	83184
chr7	127743849	127744159	0.33	1.99E-05	LEP	-137327	LRRC4	-73002

chrX	153640911	153641221	0.25	2.07E-05	DNASE1L1	-639		
chr1	156119412	156119687	0.39	2.09E-05	SEMA4A	-3838		
chr10	98031096	98031428	0.44	2.10E-05	BLNK	71		
chr3	113462129	113462439	0.31	2.12E-05	ATP6V1A	-3624	NAT13	2812
chr1	236447707	236448047	0.55	2.13E-05	ERO1LB	-2538		
chr1	11072402	11072760	0.14	2.17E-05	TARDBP	-98		
chr1	26197537	26197848	0.22	2.17E-05	PAQR7	51		
chr15	53022397	53022688	0.33	2.20E-05	ARPP19	-161330	ONECUT1	59666
chr16	20911795	20912154	0.20	2.20E-05	DCUN1D3	-414	LYRM1	-100
chr2	171738402	171738712	0.38	2.20E-05	GORASP2	-47154	GAD1	65357
chr18	3261823	3262113	0.27	2.21E-05	MYL12B	-143		
chr16	47526460	47526779	0.40	2.30E-05	PHKB	31410	ABCC12	654061
chr11	129817398	129817708	0.43	2.31E-05	NFRKB	-54649	PRDM10	55177
chr7	151329365	151329639	0.33	2.33E-05	RHEB	-112492	PRKAG2	244814
chr6	137416771	137417007	0.51	2.35E-05	IL20RA	-50591	IL22RA2	77896
chr8	97273839	97274142	0.17	2.38E-05	MTERFD1	-195	PTDSS1	-176
chr10	81947266	81947680	0.37	2.39E-05	ANXA11	17855	PLAC9	55215
chr12	27031733	27032043	0.43	2.39E-05	ITPR2	-45757	C12orf11	59366
chr18	3448973	3449287	0.44	2.45E-05	TGIF1	-2461		
chr7	21985517	21985842	0.17	2.53E-05	CDCA7L	-138		
chr14	92587983	92588367	0.18	2.60E-05	CPSF2	-154	NDUFB1	-22
chr17	7748108	7748520	0.41	2.67E-05	TMEM88	-10070	KDM6B	5079
chr17	36956038	36956427	0.16	2.71E-05	PIP4K2B	-75		
chr9	130953864	130954202	0.38	2.73E-05	CIZ1	-165		
chr21	45144666	45144977	0.44	2.73E-05	PDXK	5844	CSTB	51437
chr20	32581424	32581698	0.27	2.80E-05	RALY	-171		
chr7	50352249	50352609	0.38	2.84E-05	IKZF1	8051	FIGNL1	165659
chr17	66221284	66221560	0.35	2.90E-05	AMZ2	-22723	KPNA2	189574
chr12	104850510	104850754	0.50	2.91E-05	CHST11	-146		
chr1	147142405	147142758	0.39	2.94E-05	ACP6	52		
chr19	4763126	4763370	0.42	3.00E-05	DPP9	-39393	FEM1A	-28480
chr7	99724730	99725047	0.16	3.02E-05	MBLAC1	569		
chr5	1255146	1255456	0.30	3.05E-05	SLC6A18	29831	TERT	39861
chr19	12900780	12901070	0.21	3.11E-05	JUNB	-1385		
chr5	81147337	81148024	0.30	3.11E-05	ATG10	-120163	SSBP2	-100609
chr19	48281642	48281960	0.22	3.12E-05	SEPW1	-41		
chr12	49947348	49947658	0.41	3.17E-05	MCRS1	12719	KCNH3	14563
chr6	114179941	114180190	0.39	3.29E-05	MARCKS	1539	HDAC2	112288

chr8	27951245	27951481	0.32	3.38E-05	ELP3	779		
chr10	112327085	112327481	0.14	3.43E-05	SMC3	-166		
chr7	43909016	43909275	0.19	3.45E-05	MRPS24	-1		
chr7	28609885	28610162	0.43	3.45E-05	CREB5	157880	TRIL	388005
chr12	113500564	113500874	0.38	3.46E-05	DTX1	5057	RASAL1	73302
chr14	57046213	57046570	0.20	3.47E-05	C14orf101	-119		
chr3	44087398	44087732	0.35	3.47E-05	C3orf23	-292379	ABHD5	355190
chr22	27905799	27906111	0.30	3.48E-05	PITPNB	409300	CRYBA4	888027
chr1	154934231	154934676	0.36	3.49E-05	PYGO2	-196		
chr22	40574546	40574856	0.50	3.49E-05	TNRC6B	772		
chr6	109703686	109704043	0.13	3.56E-05	CD164	-103		
chr12	119984216	119984526	0.32	3.60E-05	LOC387890	-46893	HSPB8	367776
chr1	54291068	54291378	0.36	3.61E-05	GLIS1	-91346	TMEM48	12952
chr1	203267878	203268188	0.42	3.61E-05	CHIT1	-69173	BTG2	-6631
chr1	177091996	177092313	0.39	3.69E-05	ASTN1	41869	PAPPA2	659848
chr10	104040048	104040358	0.34	3.78E-05	NFKB2	-114136	GBF1	34894
chr19	5865212	5865456	0.44	3.79E-05	FUT3	-13849	FUT5	5300
chr3	9821445	9821755	0.38	3.88E-05	CAMK1	-9939	TADA3	12820
chr20	60718569	60718805	0.39	3.97E-05	PSMA7	-213	SS18L1	-135
chr6	31550115	31550425	0.43	4.16E-05	LST1	-3701	LTB	-68
chr16	21530331	21530641	0.41	4.18E-05	DKFZp547E087	-31496	SLC7A5P1	1279
chr5	173070952	173071262	0.52	4.34E-05	CPEB4	-244224	BOD1	-27441
chr14	102834086	102834396	0.40	4.40E-05	CINP	-4988		
chr7	139025874	139026209	0.23	4.49E-05	LUC7L2	-18592	TTC26	207552
chr19	10205073	10205383	0.32	4.52E-05	ANGPTL6	8197	RDH8	81303
chr22	39052331	39052690	0.19	4.69E-05	CBY1	-147		
chr16	16001753	16002209	0.48	4.70E-05	ABCC1	-41453	C16orf63	-19534
chr2	149893086	149893535	0.45	4.94E-05	LYPD6B	-1670		
chr22	37415398	37415908	0.39	4.99E-05	TST	-162	MPST	-49
chr13	76111958	76112285	0.18	5.03E-05	COMMD6	-131		
chr2	73441071	73441381	0.46	5.03E-05	SMYD5	-140		
chr9	34637697	34638070	0.25	5.04E-05	SIGMAR1	-116		
chr10	13850975	13851215	0.31	5.04E-05	PRPF18	222156	FRMD4A	521771
chr1	37420571	37420925	0.30	5.06E-05	CSF3R	-472239	GRIK3	79096
chr19	50167510	50167839	0.43	5.27E-05	BCL2L12	-724		
chr5	149790023	149790291	0.45	5.27E-05	CD74	2175	TCOF1	52955
chr6	30524212	30524478	0.26	5.34E-05	PRR3	-411	GNL1	663
chr8	50097413	50097723	0.25	5.35E-05	SNTG1	-727029	SNAI2	-263580

chr19	10443501	10444021	0.16	5.36E-05	RAVER1	553		
chr3	187718164	187718648	0.39	5.39E-05	BCL6	-254931	LPP	-212315
chr8	22926552	22926788	0.52	5.41E-05	TNFRSF10B	30		
chr19	36134369	36134716	0.21	5.42E-05	COX6B1	-4612		
chr19	18315061	18315509	0.30	5.49E-05	RAB3A	-411		
chr10	76970271	76970581	0.42	5.80E-05	VDAC2	-137		
chr8	144679584	144679874	0.22	5.93E-05	TIGD5	-492	EEF1D	116
chr6	10533990	10534369	0.46	6.01E-05	TFAP2A	-118710	GCNT2	-51813
chr3	50358887	50359228	0.21	6.05E-05	HYAL1	-18054	HYAL2	1223
chrX	118699223	118699594	0.38	6.07E-05	CXorf56	-45		
chr6	32909318	32909626	0.46	6.16E-05	HLA-DMB	-655		
chr3	191900884	191901274	0.38	6.23E-05	FGF12	225759	PYDC2	722127
chr16	75143644	75143884	0.34	6.33E-05	LDHD	6901	ZNRF1	110849
chr1	151431839	151432401	0.46	6.38E-05	POGZ	-188		
chr19	12912566	12912954	0.15	6.50E-05	RNASEH2A	-4668	PRDX2	-66
chr14	31926004	31926314	0.49	6.56E-05	C14orf126	521		
chr9	91537060	91537441	0.38	6.60E-05	C9orf47	-68527	SPIN1	533954
chr11	842550	842867	0.26	6.84E-05	POLR2L	-180	TSPAN4	-115
chr16	30363153	30363463	0.28	7.13E-05	CD2BP2	3374	SULT1A3	152772
chr6	35888700	35889160	0.34	7.25E-05	SRPK1	26		
chr8	41502523	41502833	0.28	7.35E-05	NKX6-3	2197	AGPAT6	66971
chr2	120070548	120070801	0.27	7.40E-05	DBI	-53829	STEAP3	89291
chr10	99894165	99894401	0.46	7.41E-05	CRTAC1	-103698	LOXL4	133724
chr16	2563659	2564054	0.28	7.46E-05	ATP6V0C	-96		
chr4	42659228	42659595	0.16	7.63E-05	ATP8A1	-290		
chr14	107259167	107259477	0.39	7.73E-05	ADAM6	-820964	IGHV7-81	23961
chr6	74171241	74171624	0.46	7.74E-05	MTO1	-21		
chr14	39901338	39901708	0.18	7.89E-05	CTAGE5	165195		
chr9	127624180	127624450	0.23	7.89E-05	RPL35	-75		
chr8	86102109	86102563	0.20	7.93E-05	CA13	-55380	E2F5	12717
chr12	46797176	46797486	0.38	8.04E-05	SLC38A2	-30686	SLC38A4	422449
chr13	49945818	49946128	0.47	8.07E-05	CAB39L	29762	CDADC1	123870
chr19	49621984	49622297	0.33	8.34E-05	PPFIA3	-522		
chr12	32260875	32261185	0.43	8.57E-05	BICD1	845		
chr3	129813036	129813415	0.52	8.59E-05	COL29A1	-251133	ALG1L2	12552
chr14	23000529	23000839	0.53	8.60E-05	DAD1	57459	TCRDV2	72595
chr14	105124220	105124530	0.49	8.91E-05	TMEM179	-53278	INF2	-31568
chr8	131675372	131675628	0.23	9.12E-05	ASAP1	-261284	ADCY8	377335

chr1	174231978	174232329	0.47	9.67E-05	GPR52	-185058	RABGAP1L	103520
chr19	7410517	7410991	0.37	9.96E-05	INSR	-116743	ARHGEF18	-93820
chr10	127511808	127512165	0.16	1.02E-04	UROS	-150	BCCIP	-117
chr1	1624096	1624743	0.09	1.02E-04	LOC728661	-177		
chr1	175194144	175194613	0.40	1.02E-04	TNN	157385	TNR	518373
chr8	27326027	27326337	0.44	1.03E-04	CHRNA2	10631	PTK2B	143128
chr7	138791783	138792035	0.45	1.04E-04	KIAA1549	-187339	ZC3HAV1	2556
chr1	241933541	241933777	0.54	1.05E-04	OPN3	-129958	EXO1	-77876
chr3	160472883	160473269	0.17	1.08E-04	PPM1L	-920		
chr22	19466686	19466977	0.25	1.08E-04	CDC45L	-584	UFD1L	-94
chr19	18334779	18335015	0.23	1.09E-04	RAB3A	-20023	PDE4C	24113
chr12	53594612	53594922	0.40	1.09E-04	ITGB7	6233	ZNF740	20232
chr6	57036831	57037141	0.46	1.09E-04	BAG2	-118		
chr8	59570989	59571299	0.39	1.09E-04	NSMAF	822		
chr12	6536092	6536438	0.31	1.12E-04	CD27	-17786	LTBR	42908
chr19	9945335	9945645	0.46	1.13E-04	PIN1	-509		
chr4	48917609	48917913	0.41	1.14E-04	CWH43	-70504	OCIAD2	-8946
chr8	43018259	43018588	0.39	1.15E-04	HGSNAT	22832		
chr7	107204135	107204444	0.44	1.17E-04	DUS4L	-142	COG5	669
chr9	137000827	137001227	0.43	1.18E-04	WDR5	-183		
chr17	76730880	76731387	0.47	1.21E-04	DNAH17	-163730	CYTH1	47242
chr5	88179272	88179582	0.44	1.22E-04	MEF2C	-125		
chr8	48873182	48873505	0.12	1.23E-04	PRKDC	-601	MCM4	-150
chr5	140887063	140887333	0.37	1.25E-04	PCDHGC5	18390	DIAPH1	111424
chr4	37070	37357	0.25	1.26E-04	ZNF718	-16013		
chr22	24820798	24821141	0.40	1.28E-04	ADORA2A	-2560		
chr8	61824712	61824991	0.34	1.30E-04	RLBP1L1	-375673	CHD7	233513
chr6	106958372	106958757	0.42	1.31E-04	AIM1	-740		
chr19	46850594	46850904	0.53	1.31E-04	PPP5C	455		
chr6	31795150	31795466	0.19	1.32E-04	HSPA1B	-204		
chr5	10250005	10250449	0.26	1.32E-04	FAM173B	-213	CCT5	-55
chr5	179921127	179921406	0.21	1.36E-04	CNOT6	-150		
chr8	22832849	22833233	0.42	1.40E-04	PEBP4	-47620	RHOBTB2	-20320
chr16	70285727	70286133	0.38	1.42E-04	EXOSC6	-97		
chr19	36391492	36391802	0.49	1.46E-04	HCST	-1735	NFKBID	-95
chr12	25104743	25105057	0.63	1.47E-04	BCAT1	-2592		
chr7	158312139	158312400	0.44	1.52E-04	PTPRN2	68212		
chr1	98386892	98387208	0.32	1.56E-04	DPYD	-435		

chr9	117150158	117150511	0.51	1.57E-04	AKNA	6350	ORM1	65032
chr6	42858495	42858794	0.21	1.57E-04	PTCRA	-25082	RPL7L1	10974
chr1	28501826	28502202	0.38	1.63E-04	EYA3	-86883	PTAFR	1177
chr11	576121	576471	0.17	1.64E-04	PHRF1	-187		
chr19	24006698	24007023	0.43	1.66E-04	ZNF254	-263115	RPSA	61045
chr7	1199745	1200127	0.35	1.67E-04	ZFAND2A	-81		
chr1	154916717	154917027	0.53	1.76E-04	PMVK	-7388	PBXIP1	11695
chr6	27791550	27791862	0.52	1.76E-04	HIST1H4J	-197		
chr14	93579709	93580019	0.51	1.80E-04	ITPK1	2399	CHGA	190419
chrX	153686359	153686693	0.41	1.86E-04	PLXNA3	-97		
chr6	31869850	31870434	0.41	1.86E-04	EHMT2	-4678	ZBTB12	-373
chr22	18484273	18484602	0.30	1.88E-04	BID	-227659	MICAL3	22887
chr8	8800161	8800471	0.48	1.88E-04	ERI1	-59998	MFHAS1	-49185
chr6	151006999	151007347	0.55	1.92E-04	MTHFD1L	-179518	PLEKHG1	86174
chr1	91487707	91487943	0.50	1.92E-04	ZNF644	-154		
chr18	9136399	9136716	0.25	1.94E-04	ANKRD12	-200		
chr12	70300111	70300549	0.46	1.94E-04	C12orf28	-20107	RAB3IP	167699
chr17	40518709	40519019	0.48	1.97E-04	STAT3	21649	STAT5A	79299
chr17	49008318	49008628	0.49	1.98E-04	TOB1	-67060	SPAG9	189753
chr19	17865659	17866151	0.42	2.01E-04	B3GNT3	-40014	MAP1S	35602
chr6	42237311	42237655	0.39	2.01E-04	MRPS10	-51850	TRERF1	182300
chr11	111284975	111285350	0.47	2.19E-04	POU2AF1	-35006	BTG4	97901
chr19	7124857	7125167	0.28	2.22E-04	ZNF557	55541	INSR	168999
chr14	75348235	75348695	0.21	2.25E-04	DLST	-129		
chr12	120739958	120740374	0.26	2.27E-04	SIRT4	3		
chr1	155658553	155658903	0.26	2.28E-04	DAP3	-146	YY1AP1	63
chr5	132202098	132202385	0.29	2.29E-04	GDF9	-1765	UQCRQ	-77
chr19	7413586	7414063	0.33	2.32E-04	INSR	-119814	ARHGEF18	-90749
chr1	42512475	42512785	0.30	2.35E-04	HIVEP3	-128134	GUCA2B	-106462
chr13	51483841	51484151	0.48	2.39E-04	RNASEH2B	104		
chr15	89786961	89787289	0.28	2.40E-04	FANCI	-69		
chr18	32738969	32739279	0.55	2.41E-04	ZNF397	-81874	MAPRE2	180916
chr7	8230059	8230354	0.54	2.48E-04	ICA1	71475	GLCCI1	221784
chr8	74903852	74904275	0.56	2.60E-04	LY96	477		
chr8	125616841	125617151	0.43	2.65E-04	NDUFB9	65653	MTSS1	123734
chr11	126334849	126335159	0.49	2.68E-04	ST3GAL4	108908	KIRREL3	535762
chr10	75385616	75385926	0.44	2.80E-04	USP54	-50338	MYOZ1	15744
chr17	6915557	6915975	0.22	2.80E-04	C17orf49	-1698	RNASEK	-32

chr1	54335163	54335473	0.50	2.83E-04	TMEM48	-31143	YIPF1	20135
chr3	195636236	195636556	0.33	2.86E-04	TNK2	-13964	SDHALP1	80754
chr21	44394069	44394412	0.20	2.87E-04	PKNOX1	-402		
chr10	15001045	15001355	0.41	2.90E-04	MEIG1	-238		
chr7	5270753	5271093	0.36	2.97E-04	SLC29A4	-51638	WIPI2	41088
chr2	227770469	227770786	0.45	3.00E-04	RHBDD1	69855	COL4A4	258647
chr5	114880833	114881143	0.49	3.04E-04	FEM1C	-397		
chr22	21368527	21368837	0.39	3.07E-04	P2RX6	-782		
chr17	80550601	80551323	0.04	3.09E-04	FOXK2	73368	RAB40B	105636
chr6	33289802	33290235	0.56	3.11E-04	ZBTB22	-4300	DAXX	774
chr6	31670603	31671335	0.20	3.19E-04	LY6G6F	-3715	BAT5	119
chr1	213224280	213224663	0.53	3.28E-04	RPS6KC1	-116		
chr9	140162437	140162747	0.42	3.28E-04	C9orf167	-9688	COBRA1	12833
chr6	107939876	107940233	0.44	3.32E-04	SOBP	128738	SCML4	205466
chr22	30988121	30988403	0.29	3.34E-04	PES1	-368		
chr6	116593491	116593801	0.42	3.35E-04	TSPYL4	-18385	TSPYL1	7634
chr17	7258318	7258562	0.44	3.48E-04	TMEM95	-57		
chr8	8245770	8246084	0.42	3.49E-04	CLDN23	-313739	PRAGMIN	-6670
chr10	72335731	72336158	0.43	3.52E-04	NODAL	-134480	PRF1	26586
chr12	62653895	62654184	0.24	3.60E-04	USP15	-147		
chr19	50353849	50354492	0.22	3.60E-04	PTOV1	-245		
chr10	98038815	98039228	0.42	3.66E-04	DNTT	-25063	BLNK	-7689
chrX	134478174	134478484	0.35	3.74E-04	ZNF75D	-372	ZNF449	-367
chr12	92801803	92802113	0.42	3.78E-04	PLEKHG7	-328307	BTG1	-262285
chr19	45909587	45909954	0.19	3.84E-04	PPP1R13L	-1459	CD3EAP	304
chr1	46598338	46598901	0.21	3.88E-04	PIK3R3	-240		
chr1	21845598	21845908	0.42	3.93E-04	ALPL	9895	RAP1GAP	132595
chr4	103746719	103747298	0.48	4.08E-04	MANBA	-64858	UBE2D3	1699
chr12	53614414	53614775	0.52	4.09E-04	ITGB7	-13595	RARG	11441
chr16	81040488	81040847	0.22	4.15E-04	CENPN	565		
chr7	30066011	30066542	0.33	4.18E-04	PLEKHA8	-2011	FKBP14	-9
chr6	2886230	2886540	0.46	4.20E-04	SERPINB1	-44304	SERPINB9	17160
chr3	15248396	15248772	0.54	4.26E-04	CAPN7	851		
chr19	8275391	8275775	0.40	4.34E-04	LASS4	1366	CD320	97656
chr2	198571132	198571442	0.43	4.44E-04	MARS2	1200	BOLL	79651
chr3	9690760	9691303	0.24	4.47E-04	MTMR14	-112		
chr12	1313885	1314195	0.56	4.47E-04	ERC1	213636	FBXL14	389291
chr14	68141325	68141648	0.41	4.60E-04	VTI1B	115		

chr17	67651082	67651374	0.37	4.68E-04	KCNJ16	-420198	MAP2K6	240390
chr16	4674309	4674731	0.24	4.70E-04	MGRN1	-306		
chr22	48466650	48466964	0.41	4.70E-04	FAM19A5	-418481		
chr11	3915505	3915824	0.43	4.73E-04	RRM1	-200259	STIM1	38732
chr8	67974311	67974635	0.26	4.77E-04	CSPP1	-2130	COPS5	89
chr21	44782090	44782400	0.63	4.82E-04	SIK1	64757	CRYAA	193104
chr1	27816605	27817135	0.20	4.88E-04	WASF2	-201		
chr19	18740669	18740979	0.42	4.90E-04	KLHL26	-7014	TMEM59L	17142
chr9	35815121	35815494	0.47	4.94E-04	SPAG8	-3049	HINT2	-266
chr5	159895049	159895434	0.36	4.94E-04	PTTG1	46377	ATP10B	383977
chr14	69259857	69260178	0.49	4.97E-04	ZFP36L1	-233		
chr1	158217960	158218270	0.57	5.04E-04	CD1A	-5812	CD1D	68378
chr2	135169448	135169893	0.44	5.10E-04	MGAT5	157841	TMEM163	306900
chr1	217804387	217804736	0.25	5.26E-04	GPATCH2	-153	SPATA17	-133
chr7	101528196	101528600	0.50	5.27E-04	SH2B2	-400007	CUX1	67516
chr6	33245660	33245965	0.35	5.31E-04	B3GALT4	896		
chr12	56551941	56552295	0.39	5.32E-04	MYL6	18		
chr22	39926687	39926997	0.48	5.46E-04	RPS19BP1	2018	ATF4	10273
chr17	28258319	28258627	0.47	5.48E-04	SSH2	-1455		
chr1	151319440	151319754	0.50	5.59E-04	RFX5	172		
chr19	18100324	18100569	0.41	5.61E-04	KCNN1	38336	IL12RB1	97295
chr7	55433620	55434082	0.27	5.68E-04	LANCL2	710		
chr2	64044131	64044441	0.29	5.69E-04	UGP2	-24728	MDH1	228164
chr9	95087895	95088230	0.29	5.74E-04	NOL8	-187	CENPP	322
chr14	39644140	39644468	0.18	5.76E-04	TRAPPC6B	-4670	PNN	-83
chr12	96537277	96537587	0.50	6.10E-04	LTA4H	-108067	ELK3	-50775
chr5	173344906	173345276	0.25	6.11E-04	HMP19	-127633	CPEB4	29760
chr19	1648405	1648794	0.46	6.26E-04	UQCR	-43169	TCF3	1686
chr1	32292911	32293283	0.43	6.30E-04	SPOCD1	-11517	PTP4A2	110891
chr3	169939794	169940297	0.12	6.36E-04	PRKCI	-174		
chr1	85461137	85461373	0.62	6.40E-04	LPAR3	-102359	MCOLN2	1541
chr3	30694495	30694812	0.54	6.42E-04	TGFBR2	46660	GADL1	241499
chr8	38625808	38626131	0.48	6.42E-04	RNF5	-167195	TACC1	-18752
chr19	36050158	36050409	0.29	6.54E-04	ATP4A	4276	TMEM147	13739
chr17	54974379	54974773	0.42	6.58E-04	TRIM25	16833	DGKE	63116
chr1	62902450	62902686	0.53	6.60E-04	USP1	593		
chr20	35807413	35807758	0.32	6.67E-04	RPN2	130	C20orf132	388
chr1	231115173	231115483	0.51	6.70E-04	TTC13	-721	ARV1	505

chr3	197900190	197900444	0.35	6.76E-04	LMLN	213246		
chr5	71615975	71616404	0.30	6.76E-04	MRPS27	-106	PTCD2	-10
chrX	147691519	147691829	0.55	6.78E-04	AFF2	109535	IDS	895190
chr12	56498408	56498718	0.49	6.91E-04	PA2G4	460		
chr3	187664324	187664610	0.51	6.92E-04	LPP	-266254	BCL6	-200992
chr11	36153342	36153604	0.49	6.99E-04	PRR5L	-244062	LDLRAD3	187861
chr2	146508234	146508516	0.50	7.02E-04				
chr6	32862086	32862448	0.51	7.02E-04	PSMB9	40329	HLA-DMB	46550
chr22	51059507	51059817	0.47	7.08E-04	ARSA	6945	MAPK8IP2	18100
chr5	115177076	115177511	0.39	7.14E-04	AP3S1	-325	ATG12	205
chr19	10613376	10613640	0.24	7.16E-04	KEAP1	546		
chr2	128378417	128378727	0.29	7.26E-04	GPR17	-24867	MYO7B	85194
chr7	100202114	100202487	0.42	7.49E-04	MOSPD3	-7813	PCOLCE	2419
chr6	32915652	32915909	0.52	7.55E-04	HLA-DMB	-6964	HLA-DMA	5118
chr1	8021456	8021773	0.21	7.56E-04	PARK7	-99		
chr12	125399903	125400157	0.48	7.57E-04	UBC	-453		
chr9	140567501	140567811	0.45	7.66E-04	CACNA1B	-204585	EHMT1	54212
chrX	48900700	48901015	0.52	7.67E-04	TFE3	132		
chr7	50306442	50306752	0.54	7.74E-04	ZPBP	-173737	IKZF1	-37781
chr4	670082	670427	0.42	7.77E-04	ATP5I	-2133	MYL5	-1456
chr1	222817408	222817757	0.39	7.85E-04	MIA3	26139	AIDA	68281
chr5	140998615	140998886	0.37	7.98E-04	DIAPH1	-129		
chr5	64807986	64808296	0.48	8.04E-04	ADAMTS6	-30437	CENPK	50854
chr5	6717874	6718184	0.39	8.07E-04	ADCY2	-678314	POLS	3311
chr11	85955471	85955877	0.20	8.07E-04	EED	-141		
chr16	58451671	58452107	0.49	8.09E-04	NDRG4	-45660	GINS3	25591
chr18	29523551	29523861	0.48	8.09E-04	KIAA1012	-615		
chr4	81355351	81355661	0.25	8.10E-04	BMP3	-596613	FGF5	167764
chr11	57434878	57435256	0.26	8.16E-04	ZDHHC5	-407		
chr3	179040560	179040920	0.47	8.21E-04	ZNF639	-811		
chr5	163343297	163343607	0.64	8.34E-04	MAT2B	410867		
chr12	40618740	40619181	0.48	8.56E-04	LRRK2	148		
chr11	8935949	8936199	0.37	8.73E-04	C11orf17	3373	ASCL3	28506
chr13	45009348	45009654	0.42	8.73E-04	SERP2	61523	TSC22D1	141200
chr13	27359348	27359739	0.47	8.74E-04	GPR12	-24622	USP12	386485
chr7	98973681	98974083	0.41	8.74E-04	ARPC1B	1553	PDAP1	32410
chr10	6389270	6389580	0.48	8.79E-04	PFKFB3	144585	PRKCQ	232813
chr3	135912137	135912447	0.41	8.81E-04	MSL2	2396	PPP2R3A	227725

chr10	75403868	75404180	0.47	9.03E-04	MYOZ1	-2509		
chr11	126174157	126174467	0.51	9.12E-04	DCPS	665		
chr1	46768997	46769457	0.24	9.17E-04	UQCRH	-153		
chr20	2633017	2633313	0.31	9.18E-04	NOP56	-89		
chr18	24018891	24019368	0.54	9.20E-04	KCTD1	109370	TAF4B	212721
chr15	76136551	76136861	0.46	9.20E-04	FBXO22	-59494	UBE2Q2	1084
chr16	81554747	81555057	0.41	9.26E-04	PLCG2	-258028	CMIP	76127
chr8	52849976	52850272	0.50	9.32E-04	PCMTD1	-38389	ST18	472315
chr19	14607017	14607327	0.42	9.36E-04	GIPC1	-228		
chr16	4555425	4555831	0.59	9.38E-04	HMOX2	29287	C16orf5	33188
chr19	49141439	49141928	0.21	9.41E-04	DBP	-1045	Sec1	388
chr4	153001090	153001379	0.40	9.44E-04	PET112L	-319089	FBXW7	454950
chr3	9834386	9834786	0.20	9.66E-04	TADA3	-166		
chr12	4257582	4257921	0.57	9.80E-04	PARP11	-275144	CCND2	-125150
chr8	134512317	134512627	0.57	1.07E-03	NDRG1	-202925	ST3GAL1	71711
chr17	8079012	8079322	0.42	1.09E-03	TMEM107	547		
chr7	8007930	8008240	0.48	1.10E-03	GLCCI1	-338		
chr7	123388952	123389331	0.28	1.10E-03	WASL	-26		
chr10	17628733	17629043	0.44	1.10E-03	ST8SIA6	-132634	PTPLA	30485
chr15	67546924	67547235	0.17	1.11E-03	AAGAB	-6		
chr7	111846380	111846708	0.44	1.12E-03	ZNF277	-99	DOCK4	-82
chr2	6641974	6642284	0.48	1.12E-03	CMPK2	363807	SOX11	809330
chr10	90146932	90147216	0.41	1.13E-03	KILLIN	-523880	RNLS	196008
chr16	7403706	7403966	0.31	1.14E-03	A2BP1	21085		
chr16	13484004	13484398	0.43	1.15E-03	ERCC4	-529813	SHISA9	488724
chr2	44271368	44271678	0.40	1.15E-03	PPM1B	-124477	LRPPRC	-48379
chr10	126107372	126107796	0.36	1.15E-03	OAT	-65		
chr9	15510916	15511306	0.24	1.17E-03	PSIP1	-108		
chr12	42076803	42077113	0.50	1.18E-03	PDZRN4	245417	GLT8D3	461715
chr16	88869899	88870204	0.51	1.20E-03	CDT1	-134		
chr4	114315919	114316229	0.54	1.21E-03	ANK2	345289	CAMK2D	367009
chr2	201676069	201676611	0.27	1.22E-03	BZW1	-307		
chr15	66252968	66253278	0.48	1.23E-03	RAB11A	91327	MEGF11	292952
chr20	5751653	5751949	0.40	1.23E-03	RP5-1022P6.2	-160129	CHGB	-140173
chr20	52824360	52824691	0.30	1.26E-03	PFDN4	24		
chr17	71952261	71952518	0.39	1.26E-03	SDK2	-312163	RPL38	-247405
chr12	105352446	105352757	0.52	1.26E-03	SLC41A2	-30130	ALDH1L2	125739
chr6	31461599	31461891	0.43	1.27E-03	MICB	-4110		

chr3	11313924	11314327	0.49	1.27E-03	ATG7	116		
chr4	83933972	83934482	0.34	1.27E-03	LIN54	-2245		
chr2	20297913	20298272	0.53	1.29E-03	LAPTM4A	-46304	SDC1	126834
chr17	17653235	17653584	0.56	1.31E-03	RAI1	68623	SREBF1	86915
chr6	34759419	34759898	0.47	1.36E-03	SNRPC	34347	TAF11	96160
chrX	53449084	53449556	0.48	1.37E-03	SMC1A	298		
chr19	6737216	6737600	0.17	1.37E-03	TRIP10	-2299	GPR108	225
chrX	47221488	47221798	0.48	1.38E-03	ZNF157	-8356	USP11	129329
chr5	176433723	176434000	0.33	1.39E-03	UIMC1	-419		
chr17	72451363	72451687	0.47	1.39E-03	CD300A	-10997	GPRC5C	21689
chr19	18210238	18210582	0.57	1.40E-03	PIK3R2	-53606	MAST3	1807
chr10	122062060	122062370	0.32	1.40E-03	PPAPDC1A	-154251	SEC23IP	409992
chr3	182762338	182762660	0.54	1.41E-03	DCUN1D1	-64173	MCCC1	54866
chr16	58764978	58765292	0.48	1.42E-03	SLC38A7	-46461	GOT2	3111
chr8	120927555	120927838	0.25	1.42E-03	COL14A1	-209655	DEPDC6	41797
chr17	9479061	9479421	0.53	1.43E-03	WDR16	-703	STX8	34
chr7	87503428	87503738	0.50	1.44E-03	DBF4	-1961		
chr11	47421839	47422269	0.52	1.44E-03	SPI1	-21927	SLC39A13	-7997
chr1	226841113	226841536	0.50	1.45E-03	ITPKB	85551	C1orf95	104824
chr19	2163737	2163985	0.27	1.51E-03	DOT1L	-287		
chr19	50381161	50381397	0.59	1.51E-03	AKT1S1	-667	TBC1D17	450
chr11	82612438	82612855	0.26	1.53E-03	PRCP	-1090	C11orf82	-90
chr5	111496321	111496632	0.18	1.54E-03	TIGA1	251		
chr19	35786898	35787216	0.50	1.56E-03	CD22	-33022	MAG	4019
chr12	133396684	133397080	0.47	1.59E-03	ANKLE2	-58431	GOLGA3	8406
chr11	117049382	117049692	0.55	1.61E-03	SIDT2	-402		
chr10	104172841	104173151	0.36	1.61E-03	PSD	5905	NFKB2	18657
chr14	71122174	71122506	0.47	1.62E-03	TTC9	13836	MAP3K9	153548
chr19	7600464	7600849	0.42	1.62E-03	KIAA1543	-60131	PNPLA6	1614
chr21	19191702	19192271	0.24	1.64E-03	CHODL	-425163	BTG3	-206719
chr8	67443979	67444340	0.42	1.68E-03	MYBL1	81320	ADHFE1	99442
chr6	24910644	24911104	0.50	1.69E-03	FAM65B	321		
chr22	41777464	41777809	0.48	1.70E-03	TEF	-326		
chr7	22005539	22005933	0.51	1.74E-03	CDCA7L	-20194	RAPGEF5	390797
chr8	38243546	38244040	0.46	1.75E-03	WHSC1L1	-4003	LETM2	-227
chr6	32122434	32122744	0.54	1.79E-03	PRRT1	-2869	PPT2	1288
chr13	30948661	30949002	0.41	1.80E-03	KATNAL1	-67669	HMGB1	91249
chr19	50354740	50355050	0.38	1.81E-03	PTOV1	479		

chr6	116892286	116892670	0.50	1.86E-03	RWDD1	-105		
chr9	130538553	130538984	0.24	1.86E-03	TOR2A	-41165	SH2D3C	2279
chr16	21964296	21964776	0.23	1.89E-03	UQCRC2	-73		
chr20	44600078	44600577	0.26	1.90E-03	ZNF335	505		
chr10	103578111	103578744	0.14	1.91E-03	MGEA5	-206		
chr22	21213048	21213326	0.28	1.93E-03	PI4KA	-117	SNAP29	-105
chr4	114374362	114374672	0.47	1.94E-03	CAMK2D	308566	ANK2	403732
chr10	74091709	74092058	0.43	1.96E-03	DNAJB12	23023	DDIT4	58207
chr7	64868995	64869305	0.39	1.97E-03	VKORC1L1	-469107	ZNF92	30382
chr7	27170445	27170693	0.43	1.98E-03	HOXA4	-170		
chr11	125932702	125933240	0.26	1.99E-03	CDON	216		
chr6	6610579	6611002	0.53	2.00E-03	RREB1	-497397	LY86	21857
chr1	155532702	155533070	0.37	2.02E-03	ASH1L	-562		
chr5	137673390	137673976	0.17	2.04E-03	KDM3B	-14602	CDC25C	-6167
chr17	17140447	17141116	0.19	2.05E-03	FLCN	-280		
chr7	6487538	6487950	0.49	2.07E-03	DAGLB	-101		
chr6	150326201	150326587	0.57	2.07E-03	RAET1L	20274	ULBP1	41251
chr1	154910092	154910402	0.55	2.08E-03	PMVK	-763		
chr20	42219216	42219610	0.31	2.08E-03	IFT52	-166		
chr19	871559	871869	0.39	2.10E-03	CFD	12049	MED16	21504
chr19	12847870	12848373	0.32	2.15E-03	ASNA1	-184		
chr1	114414208	114414764	0.46	2.15E-03	PTPN22	-111		
chr2	29503360	29503658	0.51	2.15E-03	C2orf71	-206382	ALK	640923
chr6	36410527	36410834	0.32	2.18E-03	KCTD20	137		
chr7	2393704	2394496	0.10	2.21E-03	EIF3B	-374		
chr12	129306561	129306871	0.58	2.23E-03	SLC15A4	1825	TMEM132C	407425
chr11	129872182	129872804	0.18	2.24E-03	PRDM10	237		
chr2	33302920	33303230	0.55	2.24E-03	RASGRP3	-435867	LTBP1	130683
chr12	122241080	122241403	0.54	2.25E-03	SETD1B	-1388		
chr1	174245268	174245539	0.50	2.26E-03	GPR52	-171808	RABGAP1L	116770
chr17	7589286	7590038	0.15	2.28E-03	WRAP53	273		
chr4	139936578	139936924	0.30	2.28E-03	CCRN4L	-192		
chr1	151319779	151320107	0.50	2.28E-03	RFX5	-174		
chr4	57301852	57302385	0.40	2.32E-03	PPAT	-274	PAICS	-150
chr18	3247504	3247856	0.22	2.33E-03	MYL12A	152		
chr19	10305560	10305917	0.16	2.35E-03	DNMT1	16		
chr7	44086277	44086587	0.45	2.37E-03	DBNL	2193	PGAM2	18731
chr3	113933975	113934309	0.59	2.38E-03	DRD3	-36243	ZNF80	22283

chr9	130007541	130007851	0.56	2.38E-03	ANGPTL2	-122652	GARNL3	-19060
chrX	4264814	4265058	0.33	2.38E-03	PRKX	-633275		
chr7	66144021	66144331	0.49	2.40E-03	RABGEF1	-60807	KCTD7	50286
chr6	32952603	32952913	0.44	2.40E-03	BRD2	16321	HLA-DOA	24631
chr19	10522905	10523218	0.57	2.40E-03	CDC37	-8791	PDE4A	-8271
chr1	172501538	172502098	0.39	2.42E-03	C1orf9	-442		
chr14	107154857	107155207	0.46	2.46E-03	ADAM6	-716674	IGHV7-81	128251
chr1	153571333	153571704	0.37	2.48E-03	S100A2	-33213	S100A16	13995
chr2	86226323	86226633	0.53	2.52E-03	ST3GAL5	-110321	POLR1A	106800
chr5	156310274	156310784	0.57	2.52E-03	PPP1R2P3	32980	TIMD4	79737
chr3	16647465	16647775	0.51	2.54E-03	DAZL	-614		
chr8	68305090	68305400	0.58	2.56E-03	ARFGEF1	-49333	CPA6	353375
chr11	3926378	3926688	0.32	2.57E-03	RRM1	-189391	STIM1	49600
chr1	186051497	186051741	0.58	2.62E-03	PRG4	-213786	HMCN1	347936
chr3	118960284	118960594	0.51	2.63E-03	B4GALT4	-687		
chr4	140684861	140685174	0.62	2.70E-03	MGST2	98096	MAML3	390215
chr7	69161071	69161409	0.52	2.71E-03				
chr5	64064383	64064819	0.34	2.72E-03	SDCCAG10	-154	SFRS12IP1	-105
chr3	194457268	194457622	0.46	2.72E-03	LSG1	-64239	C3orf21	534450
chr12	54019853	54020506	0.23	2.77E-03	ATF7	19		
chr17	56429480	56429789	0.44	2.78E-03	SUPT4H1	-72		
chr3	12748005	12748372	0.55	2.80E-03	RAF1	-42489	TMEM40	52619
chr3	187663823	187664237	0.55	2.81E-03	LPP	-266691	BCL6	-200555
chr9	132346425	132346755	0.68	2.91E-03	METTL11A	-41845	PPP2R4	473346
chr12	110436753	110437252	0.17	2.92E-03	GIT2	-2809		
chr16	84759460	84759941	0.57	2.92E-03	CRISPLD2	-93886	USP10	26146
chr19	3500568	3500999	0.23	2.93E-03	DOHH	-163		
chr22	38967008	38967320	0.48	2.94E-03	DMC1	-975		
chr20	524456	524810	0.28	3.01E-03	CSNK2A1	-151		
chr15	89181920	89182338	0.35	3.01E-03	ISG20	90		
chr7	2354269	2354563	0.52	3.03E-03	SNX8	-317		
chr6	33290486	33290815	0.57	3.04E-03	ZBTB22	-4932	DAXX	142
chr17	49337306	49337834	0.24	3.06E-03	UTP18	-327	MBTD1	-143
chr2	233940657	233941091	0.61	3.07E-03	ATG16L1	-219343	INPP5D	15838
chr8	54934805	54935115	0.52	3.08E-03	TCEA1	48		
chr5	149790854	149791240	0.56	3.09E-03	CD74	1285	TCOF1	53845
chr2	86332977	86333351	0.27	3.14E-03	PTCD3	-141	POLR1A	114
chr16	2255222	2255822	0.35	3.16E-03	MLST8	72		

chr6	6587344	6587699	0.51	3.16E-03	LY86	-1412		
chr6	43149598	43149960	0.54	3.18E-03	CUL9	-143		
chr6	3356509	3356819	0.58	3.19E-03	TUBB2B	-128696	SLC22A23	100129
chr11	9623330	9623714	0.57	3.19E-03	SWAP70	-62106	WEE1	28294
chr19	50316793	50317153	0.38	3.20E-03	MED25	-4573	FUZ	-505
chr6	42016177	42016615	0.49	3.21E-03	TAF8	-1855		
chr19	42806521	42807232	0.17	3.27E-03	PAFAH1B3	75		
chr5	157825704	157826014	0.36	3.34E-03	CLINT1	-539691	EBF1	700929
chr1	2574520	2574844	0.60	3.37E-03	ACTRT2	-363364	MMEL1	-10201
chr10	75910523	75910833	0.49	3.46E-03	ADK	-287	AP3M1	148
chr18	47883606	47883916	0.51	3.48E-03	CXXC1	-69069	SKA1	-17631
chr4	77870704	77870940	0.49	3.48E-03	SEPT11	-73		
chr9	140470522	140470859	0.62	3.51E-03	ZMYND19	14246	MRPL41	24382
chr18	74827500	74828138	0.38	3.52E-03	MBP	16955	ZNF236	291703
chr6	57086897	57087207	0.65	3.56E-03	RAB23	26		
chr2	97589986	97590296	0.51	3.56E-03	SEMA4C	-54406	FAHD2B	170441
chr10	49876138	49876528	0.50	3.59E-03	ARHGAP22	-63195	WDFY4	-17185
chr6	139696997	139697355	0.55	3.60E-03	CITED2	-1391		
chr6	31620336	31620742	0.45	3.67E-03	APOM	-3132	BAT3	-369
chr15	38364647	38364957	0.56	3.67E-03	SPRED1	-180250	TMCO5A	137344
chr14	36325215	36325612	0.61	3.72E-03	BRMS1L	29817	MBIP	464468
chr3	148961248	148961671	0.56	3.72E-03	CP	-21628	TM4SF18	89959
chr10	11706139	11706439	0.59	3.74E-03	USP6NL	-132015	ECHDC3	-78067
chr17	64563352	64563670	0.51	3.74E-03	CACNG5	-309837	PRKCA	264585
chr21	46530138	46530448	0.55	3.75E-03	ADARB1	35800	POFUT2	177518
chr7	75544221	75544524	0.17	3.76E-03	POR	-47		
chr4	31292501	31292832	0.52	3.76E-03	PCDH7	570630		
chr13	114100197	114100664	0.40	3.81E-03	GRTP1	-81968	ADPRHL1	7408
chr12	68382963	68383284	0.51	3.86E-03	IFNG	170397	DYRK2	340612
chr5	79486721	79487075	0.49	3.88E-03	SERINC5	64972	THBS4	155907
chr12	67295014	67295324	0.50	3.90E-03	CAND1	-367892	GRIP1	-222244
chr9	71255197	71255608	0.49	3.90E-03	C9orf71	-99620	PIP5K1B	-65213
chr18	20808228	20808613	0.53	3.91E-03	CABLES1	92694	C18orf45	209504
chr10	25304961	25305271	0.65	4.04E-03	THNSL1	-392	ENKUR	-86
chr9	129483830	129484078	0.46	4.05E-03	ZBTB43	-83331	LMX1B	107206
chr15	65117795	65118467	0.31	4.06E-03	PIF1	-293		
chr7	66202721	66202990	0.42	4.08E-03	RABGEF1	-2127		
chr2	234240298	234240608	0.50	4.14E-03	DGKD	-22700	SAG	24144

chr18	18551790	18552117	0.47	4.15E-03	ROCK1	139858		
chr19	13215186	13215779	0.44	4.15E-03	LYL1	-1802		
chr2	85173272	85173634	0.62	4.23E-03	KCMF1	-24778	TMSB10	40690
chr20	56255352	56255662	0.55	4.24E-03	ZBP1	-59875	PMEPA1	29524
chr9	92116319	92116674	0.39	4.27E-03	GADD45G	-103430	SEMA4D	-21886
chr7	8215830	8216274	0.55	4.28E-03	ICA1	85630	GLCCI1	207629
chr20	31332721	31333090	0.65	4.32E-03	COMMD7	-1092		
chr6	30484358	30484693	0.59	4.34E-03	PRR3	-40230	HLA-E	27255
chr16	29874544	29875049	0.23	4.34E-03	CDIPT	-219		
chr2	164202409	164202811	0.64	4.37E-03	KCNH7	-507370	FIGN	389903
chr5	158239407	158239717	0.48	4.38E-03	CLINT1	-953394	EBF1	287226
chrX	152602501	152602737	0.30	4.38E-03	ZFP92	-81162	ZNF275	3006
chr15	65136815	65137150	0.53	4.45E-03	ANKDD1A	-67118	PIF1	-19145
chr12	105131170	105131496	0.33	4.48E-03	SLC41A2	191139	CHST11	280555
chr3	51533361	51533608	0.73	4.52E-03	VPRBP	516		
chr5	137438650	137438974	0.57	4.55E-03	WNT8A	19038	NME5	36320
chr7	102920444	102920680	0.39	4.59E-03	DPY19L2P2	197		
chr15	57551678	57551992	0.58	4.64E-03	CGNL1	-116870	TCF12	341002
chr1	158921745	158922078	0.55	4.65E-03	IFI16	-57770	PYHIN1	20570
chr16	17554097	17554464	0.56	4.68E-03	XYLT1	10457		
chr12	122101657	122102035	0.62	4.72E-03	TMEM120B	-48812	ORAI1	37391
chr1	226537731	226538074	0.48	4.79E-03	LIN9	-40705	PARP1	57898
chr7	45018636	45018947	0.56	4.81E-03	MYO1G	-88		
chr22	23296927	23297171	0.56	4.82E-03	GNAZ	-115620	IGL@	67089
chr12	113859716	113860026	0.53	4.83E-03	SDSL	-345		
chr11	111285470	111286014	0.63	4.85E-03	POU2AF1	-35585	BTG4	97322
chr9	117379328	117379646	0.53	4.86E-03	C9orf91	5781	TNFSF15	188921
chr2	85765852	85766336	0.31	4.88E-03	MAT2A	-194		
chr19	1354510	1354754	0.46	4.89E-03	MUM1	-344		
chr12	6873431	6873758	0.53	4.95E-03	PTMS	-1946		
chrX	77041539	77042003	0.58	4.96E-03	ATRX	-52		
chr6	2989794	2990147	0.31	4.98E-03	SERPINB6	-17881	NQO2	-10096
chr17	34135981	34136520	0.18	5.03E-03	TAF15	-237		
chr7	1032822	1033108	0.54	5.08E-03	GPR146	-64176	CYP2W1	10130
chr3	187676418	187676807	0.56	5.18E-03	LPP	-254108	BCL6	-213138
chr19	17186205	17186543	0.22	5.21E-03	MYO9B	-217	HAUS8	-31
chr8	103825946	103826399	0.48	5.28E-03	KLF10	-158190	AZIN1	50224
chr17	73257690	73258010	0.61	5.32E-03	GGA3	-173	MRPS7	101

chr16	31191129	31191598	0.25	5.42E-03	FUS	-89		
chr8	42979587	42979823	0.43	5.42E-03	HGSNAT	-15887	SGK196	31048
chr6	106967895	106968313	0.41	5.46E-03	AIM1	8799	RTN4IP1	109269
chr3	121744243	121744518	0.55	5.49E-03	ILDR1	-3351		
chr1	207981068	207981378	0.54	5.51E-03	CD46	55821	CD34	103460
chr9	127533613	127533973	0.29	5.63E-03	NR6A1	-217		
chr2	12172558	12172857	0.53	5.64E-03	TRIB2	-684290	LPIN1	285968
chr1	24239715	24240129	0.46	5.65E-03	CNR2	-105		
chr19	38538817	38539317	0.44	5.73E-03	SIPA1L3	141199	DPF1	175823
chr11	44578508	44578818	0.65	5.74E-03	ALX4	-246947	CD82	-8478
chr14	60558346	60558832	0.53	5.80E-03	C14orf135	-40		
chr6	28219905	28220232	0.54	5.81E-03	ZKSCAN4	-67		
chr20	311166	311516	0.61	5.85E-03	NRSN2	-16029	SOX12	5102
chr4	963176	963486	0.36	5.87E-03	DGKQ	4013	TMEM175	37069
chr12	45937608	45937918	0.58	5.95E-03	ARID2	-185857	ANO6	327887
chr19	42927679	42928108	0.53	6.04E-03	CNFN	-33450	LIPE	3684
chr12	116309598	116309908	0.59	6.06E-03	MED13L	405238		
chr9	134270116	134270426	0.45	6.07E-03	BAT2L	-35206	PPAPDC3	105190
chr19	1449951	1450282	0.48	6.08E-03	APC2	-31		
chr13	76123464	76123937	0.27	6.10E-03	UCHL3	-226		
chr8	67578405	67578820	0.57	6.10E-03	VCPIP1	839		
chr2	234327255	234327531	0.44	6.10E-03	DGKD	64240	USP40	146843
chr1	147071572	147071886	0.53	6.14E-03	BCL9	58547	ACP6	70905
chr14	69152136	69152446	0.57	6.15E-03	ZFP36L1	107494	RAD51L1	865782
chr11	64901950	64902744	0.22	6.17E-03	SYVN1	-344		
chr6	31703947	31704374	0.52	6.25E-03	MSH5	-3613		
chr10	18483238	18483494	0.48	6.25E-03	CACNB2	53760	NSUN6	457184
chr20	8000350	8000660	0.55	6.26E-03	TMX4	-112		
chr11	66049923	66050240	0.41	6.31E-03	CNIH2	4386	YIF1A	6556
chr6	37786734	37787325	0.25	6.32E-03	ZFAND3	-277		
chr12	53493183	53493493	0.51	6.32E-03	SOAT2	-3936		
chr19	56330278	56330588	0.54	6.35E-03	NLRP4	-17511	RFPL4A	59926
chr17	73781050	73781360	0.41	6.35E-03	UNK	524		
chr8	48110226	48110514	0.48	6.37E-03	CEBPD	540356		
chr3	42003486	42003807	0.23	6.44E-03	ULK4	13		
chr11	11251675	11251985	0.35	6.48E-03	ZBED5	-372210	CSNK2A1	123074
chr3	193822024	193822446	0.28	6.49E-03	HES1	-31699	OPA1	511302
chr17	43225039	43225593	0.36	6.55E-03	HEXIM1	632		

chr17	1388198	1388508	0.55	6.57E-03	CRK	-28809	MYO1C	7648
chr15	75642247	75642551	0.58	6.74E-03	NEIL1	3068	MAN2C1	18542
chr19	7402263	7402574	0.45	6.74E-03	INSR	-108408	ARHGEF18	-102155
chr8	30013776	30014086	0.55	6.74E-03	DCTN6	118		
chr15	43802790	43803341	0.50	6.75E-03	TP53BP1	-17712	MAP1A	-6740
chr13	111366364	111366674	0.60	6.90E-03	ING1	-840		
chr7	5571518	5571780	0.52	6.93E-03	ACTB	-1417		
chr1	154833062	154833342	0.42	6.96E-03	ADAR	-252520	KCNN3	9552
chr15	63792562	63792798	0.44	7.08E-03	USP3	-4130		
chr2	44589708	44590018	0.59	7.17E-03	PREPL	-2974		
chr5	78907838	78908176	0.54	7.32E-03	PAPD4	-236		
chr2	114361682	114361992	0.54	7.46E-03	RABL2A	-22980	WASH1	8192
chr13	115046020	115046295	0.58	7.54E-03	UPF3A	-920		
chr17	16341923	16342406	0.25	7.59E-03	C17orf45	-136		
chr6	31528204	31528455	0.53	7.60E-03	LTA	-11546	NFKBIL1	12977
chr1	150039664	150040002	0.58	7.60E-03	VPS45	491		
chr19	53607006	53607317	0.69	7.62E-03	ZNF160	-475		
chr15	89010332	89011059	0.31	7.66E-03	MRPL46	-63	MRPS11	12
chr18	77384645	77384977	0.70	7.74E-03	CTDP1	-54990	NFATC1	224485
chr19	46975137	46975447	0.52	7.77E-03	CCDC8	-58373	AK094504	129165
chr7	1067908	1068177	0.41	7.77E-03	GPR146	-29098	CYP2W1	45208
chr17	21226215	21226585	0.62	7.80E-03	KCNJ12	-53299	MAP2K3	38432
chr8	57154531	57154777	0.63	7.86E-03	CHCHD7	30339	SDR16C5	78587
chr11	117103078	117103388	0.56	7.88E-03	PCSK7	-422	RNF214	-171
chr2	220019761	220020071	0.55	7.88E-03	IHH	-94727	NHEJ1	5671
chr2	69135017	69135339	0.55	7.89E-03	BMP10	-36529	GKN2	44924
chr17	37356071	37356636	0.29	7.93E-03	CACNB1	-2453	RPL19	-182
chr11	114030601	114030990	0.60	7.94E-03	NNMT	-135739	ZBTB16	99508
chr14	107259683	107260110	0.53	7.94E-03	ADAM6	-821539	IGHV7-81	23386
chr2	44223084	44223566	0.30	7.97E-03	LRPPRC	-181		
chr8	96281744	96282322	0.44	8.03E-03	PLEKHF2	135995	GDF6	890987
chr17	7108126	7108475	0.66	8.05E-03	ASGR1	-25418	DLG4	12627
chr22	29663674	29664328	0.31	8.16E-03	RHBDD3	-87	EWSR1	3
chr1	91966367	91966631	0.58	8.16E-03	CDC7	95		
chr15	89878009	89878398	0.22	8.20E-03	POLG	-178		
chr7	101582433	101582945	0.55	8.21E-03	SH2B2	-345716	CUX1	121807
chr2	214092819	214093129	0.46	8.21E-03	IKZF2	-77916	SPAG16	-56142
chr6	150943931	150944241	0.67	8.31E-03	MTHFD1L	-242605	PLEKHG1	23087

chr17	76126647	76126957	0.66	8.56E-03	TMC6	-1941	TMC8	-57
chr6	111580162	111580627	0.47	8.60E-03	KIAA1919	-87		
chr17	64188243	64188563	0.57	8.66E-03	AXIN2	-630663	APOH	37153
chr1	114471897	114472333	0.52	8.75E-03	HIPK1	119		
chr22	47010285	47010771	0.54	8.86E-03	CELSR1	-77461	GRAMD4	-12120
chr7	5572505	5572974	0.56	9.06E-03	ACTB	-2508		
chr9	116698625	116698887	0.59	9.27E-03	ZNF618	60194	AMBP	141996
chr10	126290033	126290288	0.48	9.35E-03	LHPP	139749	FAM53B	142769
chr13	46869560	46869889	0.64	9.38E-03	LRRC63	83642	C13orf18	91910
chr19	5130385	5130771	0.49	9.39E-03	KDM4B	161454	PTPRS	210236
chr8	67579712	67580031	0.60	9.43E-03	VCPIP1	-420		
chr1	150583984	150584294	0.54	9.45E-03	MCL1	-32003	ENSA	17959
chr14	55658175	55658634	0.57	9.49E-03	DLGAP5	-9		
chr10	14644009	14644319	0.59	9.49E-03	FRMD4A	-271298	CDNF	235819
chr13	42033076	42033320	0.50	9.49E-03	C13orf15	1656	KIAA0564	502023
chr6	32920723	32921266	0.50	9.57E-03	HLA-DMA	-96		
chr1	76250285	76250595	0.54	9.78E-03	RABGGTB	-1446		
chr1	26185977	26186283	0.49	9.87E-03	PAQR7	11614	FAM54B	39733
chr19	50093437	50093848	0.56	9.89E-03	PRR12	-1269		
chr1	32014600	32014910	0.59	1.00E-02	TINAGL1	-27331	SERINC2	128792
chr11	72853476	72853908	0.29	1.00E-02	FCHSD2	-549		
chr8	30014343	30014674	0.51	1.00E-02	DCTN6	696		
chr14	64702713	64703163	0.65	1.02E-02	ESR2	58190	SYNE2	383255
chr22	20817536	20817846	0.47	1.04E-02	SCARF2	-25545	KLHL22	32431
chr6	32939919	32940159	0.47	1.04E-02	BRD2	3602	HLA-DOA	37350
chr1	178994906	178995409	0.53	1.05E-02	FAM20B	84		
chr6	144164072	144164548	0.60	1.05E-02	PHACTR2	165208	PLAGL1	165231
chr16	84544907	84545217	0.60	1.05E-02	KIAA1609	-6774	COTL1	106607
chr17	76248420	76248715	0.60	1.06E-02	LOC283999	21177	SOCS3	107590
chr14	52642715	52642966	0.69	1.06E-02	NID2	-106895	PTGDR	-91590
chr14	93472870	93473180	0.47	1.07E-02	CHGA	83580	ITPK1	109238
chr12	53693326	53693764	0.54	1.07E-02	C12orf10	75		
chr6	88889445	88889751	0.57	1.07E-02	CNR1	-13831	RNGTT	783750
chr18	23670531	23671058	0.32	1.10E-02	SS18	-184		
chr12	95325	95647	0.12	1.10E-02	IQSEC3	-91056		
chr7	63781418	63781728	0.57	1.10E-02	AK301806	7181	ZNF680	241932
chr20	3748966	3749276	0.57	1.11E-02	SPEF1	12981	HSPA12B	35765
chr19	21105886	21106263	0.52	1.11E-02	ZNF85	-5		

chr18	47339908	47340218	0.61	1.12E-02	ACAA2	188		
chr10	33260615	33260929	0.52	1.12E-02	ITGB1	-13479	NRP1	363061
chr17	9953986	9954263	0.40	1.13E-02	RCVRN	-145441	GAS7	147743
chr6	43192117	43192502	0.57	1.13E-02	C6orf108	4901	CUL9	42388
chr2	19547708	19548049	0.62	1.14E-02	NT5C1B	-777041	OSR1	10493
chr16	22637920	22638208	0.42	1.14E-02	HS3ST2	-187796	NPIPL3	113180
chr15	64455160	64455726	0.36	1.14E-02	PPIB	-89		
chr13	41271891	41272171	0.61	1.15E-02	FOXO1	-31297	MRPS31	73316
chr4	38862469	38862782	0.58	1.15E-02	TLR6	-31468	FAM114A1	-6811
chr14	45366051	45366369	0.65	1.15E-02	FSCB	-389711	KLHL28	64969
chr7	37392536	37392783	0.54	1.16E-02	AOAH	-628507	ELMO1	95851
chr10	1095383	1095768	0.58	1.17E-02	IDI1	-515		
chr20	55892962	55893292	0.54	1.17E-02	BMP7	-51420	SPO11	-11704
chr8	38627617	38627861	0.56	1.18E-02	RNF5	-168964	TACC1	-16983
chr12	130443013	130443261	0.38	1.18E-02	FZD10	-203895	TMEM132D	-54925
chr10	111967178	111967470	0.70	1.19E-02	MXI1	-39		
chr6	116600990	116601362	0.58	1.19E-02	DSE	-107	TSPYL1	104
chr6	150338113	150338362	0.56	1.20E-02	RAET1L	8430	ULBP1	53095
chr5	150613307	150613596	0.58	1.20E-02	ANXA6	-76085	GM2A	-19161
chr19	36544703	36545058	0.53	1.20E-02	THAP8	783		
chr8	103540746	103541092	0.60	1.23E-02	UBR5	-116424	ODF1	-22929
chr1	154909128	154909815	0.22	1.24E-02	PMVK	12		
chr12	52463112	52463796	0.29	1.25E-02	C12orf44	-304		
chr4	149903321	149903640	0.52	1.25E-02	NR3C2	-539838		
chr21	43483274	43483638	0.59	1.25E-02	ZNF295	-52960	UMODL1	-7970
chr1	32729339	32729604	0.49	1.27E-02	LCK	-10240	EIF3I	41513
chr16	67581694	67582004	0.61	1.27E-02	CTCF	-14615	FAM65A	19095
chr8	95961551	95961828	0.53	1.28E-02	TP53INP1	-75		
chr10	17685859	17686169	0.65	1.28E-02	STAM	-110		
chr3	184374765	184375075	0.43	1.28E-02	VPS8	-155011	EPHB3	95333
chr11	29540349	29540726	0.71	1.31E-02	KCNA4	497950		
chr12	51319027	51319337	0.68	1.31E-02	METTL7A	648		
chr19	17246213	17246523	0.55	1.31E-02	USE1	-79787	MYO9B	59777
chr6	36733702	36734057	0.60	1.32E-02	CPNE5	73340	CDKN1A	87421
chr6	109416344	109416775	0.59	1.32E-02	SESN1	-1283		
chr12	68385080	68385356	0.65	1.33E-02	IFNG	168303	DYRK2	342706
chr9	126979043	126979326	0.70	1.33E-02	NEK6	-41058	LHX2	205296
chr7	99588554	99588805	0.60	1.35E-02	ZKSCAN1	-24539	BC022382	10295

chr13	47170212	47170522	0.63	1.35E-02	LRCH1	43071	ESD	201000
chr7	135242429	135242706	0.61	1.35E-02	NUP205	-94		
chr7	24860574	24861403	0.48	1.38E-02	DFNA5	-63906	OSBPL3	158771
chr17	71587538	71587863	0.64	1.39E-02	CDC42EP4	-279558	SDK2	52526
chr7	27779303	27779888	0.52	1.40E-02	TAX1BP1	-142		
chr10	496902	497175	0.46	1.43E-02	DIP2C	238569	ZMYND11	316615
chr7	100270899	100271403	0.26	1.43E-02	GNB2	-212		
chr8	74206324	74206654	0.58	1.43E-02	RDH10	-776	RPL7	-620
chr17	7387287	7387916	0.34	1.43E-02	POLR2A	-248	ZBTB4	-34
chr5	1134568	1134912	0.60	1.44E-02	SLC6A19	-66970	SLC12A7	-22568
chr2	47917255	47917565	0.51	1.46E-02	KCNK12	-119940	MSH6	-92811
chr5	133706606	133707152	0.38	1.47E-02	CDKL3	-4114	UBE2B	9
chr6	31543217	31543502	0.53	1.49E-02	TNF	-932	TNF	10
chr2	88927318	88927882	0.54	1.50E-02	EIF2AK3	-606		
chr19	47103868	47104392	0.30	1.50E-02	CALM3	-382	AK094504	327
chr1	165567264	165567740	0.69	1.51E-02	MGST3	-32948	LRRC52	54024
chr15	56830090	56830442	0.54	1.51E-02	MNS1	-72931	ZNF280D	195521
chr2	61765229	61765590	0.41	1.52E-02	XPO1	8		
chr12	109592505	109592815	0.59	1.52E-02	ACACB	15458	FOXN4	154365
chr3	16474192	16474520	0.65	1.52E-02	RFTN1	80866	OXNAD1	167642
chr1	44412317	44412627	0.63	1.53E-02	IPO13	-6		
chr7	75947278	75947589	0.50	1.53E-02	HSPB1	15559	YWHAG	40908
chr17	61774863	61775173	0.67	1.55E-02	LIMD2	2501	MAP3K3	75217
chr1	26613963	26614273	0.54	1.58E-02	SH3BGRL3	7905	UBXN11	19077
chr4	1714069	1714583	0.44	1.60E-02	SLBP	-296		
chr14	107119936	107120449	0.60	1.60E-02	ADAM6	-681835	IGHV7-81	163090
chr20	52226223	52226667	0.59	1.61E-02	ZNF217	-26738	BCAS1	460859
chr6	161794945	161795204	0.43	1.62E-02	AGPAT4	-99968		
chr6	34436254	34436506	0.69	1.62E-02	PACSIN1	2475	SPDEF	87711
chr1	161146835	161147218	0.58	1.62E-02	B4GALT3	287		
chr6	44214062	44214337	0.54	1.63E-02	HSP90AB1	-649		
chr2	10588340	10588702	0.55	1.63E-02	ODC1	-68		
chr11	85780433	85780810	0.59	1.65E-02	PICALM	-514		
chr6	14211228	14211551	0.51	1.65E-02	CD83	93525		
chr7	1177945	1178181	0.51	1.66E-02	C7orf50	-170		
chr1	228354036	228354378	0.52	1.67E-02	C1orf69	778		
chr2	153032236	153032546	0.69	1.68E-02	STAM2	115		
chr14	102553288	102553615	0.40	1.68E-02	HSP90AA1	52634	DYNC1H1	122587

chr6	43741109	43741419	0.58	1.70E-02	VEGFA	3311	MRPL14	353927
chr19	20149953	20150197	0.50	1.71E-02	ZNF682	202		
chr20	8256059	8256575	0.68	1.72E-02	PLCB4	-820615	PLCB1	143021
chr17	3595703	3596013	0.62	1.73E-02	P2RX5	3725	TMEM93	23768
chr22	30831675	30832046	0.57	1.73E-02	SEC14L3	36173	SEC14L2	38928
chr11	94964429	94964899	0.51	1.74E-02	SESN3	-418		
chr6	6588842	6589152	0.66	1.74E-02	LY86	63		
chr6	42714685	42715039	0.60	1.74E-02	TBCC	-978		
chr2	47922171	47922804	0.47	1.74E-02	KCNK12	-125018	MSH6	-87733
chr16	9184996	9185384	0.56	1.74E-02	USP7	-127849		
chr6	27805117	27805385	0.59	1.75E-02	HIST1H2BN	-1189	HIST1H2AK	866
chr7	138212420	138212730	0.48	1.77E-02	TRIM24	67496	SVOPL	151215
chr6	24721100	24721679	0.29	1.79E-02	C6orf62	-1987		
chr19	47939969	47940279	0.63	1.80E-02	MEIS3	-17339	SLC8A2	35183
chr10	90145406	90145966	0.58	1.80E-02	KILLIN	-522492	RNLS	197396
chr1	224517811	224518163	0.68	1.81E-02	NVL	-115		
chr7	39989290	39989746	0.62	1.81E-02	CDC2L5	-441		
chr1	209929309	209929619	0.68	1.81E-02	TRAF3IP3	-60		
chr16	23529655	23530113	0.70	1.81E-02	GGA2	-8076	EARS2	38812
chr18	12702586	12703034	0.55	1.82E-02	PSMG2	-254	CEP76	-107
chr19	4723805	4724175	0.28	1.83E-02	DPP9	-135		
chr6	31548645	31548919	0.57	1.88E-02	LTB	1420	TNF	4490
chr6	35704101	35704367	0.59	1.88E-02	C6orf81	-625		
chr6	33560986	33561253	0.63	1.89E-02	ITPR3	-28041	BAK1	-13050
chr1	157743844	157744154	0.65	1.91E-02	FCRL3	-73352	FCRL2	2923
chr17	56591810	56592120	0.58	1.91E-02	HSF5	-26206	MTMR4	3286
chr12	117240870	117241281	0.54	1.92E-02	RNFT2	64980	HRK	78156
chr11	19454949	19455259	0.51	1.93E-02	NAV2	-279777	E2F8	-192597
chr14	107256632	107256900	0.61	1.94E-02	ADAM6	-818408	IGHV7-81	26517
chr1	155949393	155949703	0.55	1.94E-02	ARHGEF2	-1212		
chr22	38029042	38029484	0.53	1.95E-02	SH3BP1	-5794	GGA1	24760
chr19	19477546	19477856	0.58	1.95E-02	GATAD2A	-18941	KIAA0892	46071
chr8	57124216	57124512	0.65	1.96E-02	PLAG1	-505	CHCHD7	49
chr7	101673966	101674229	0.57	1.96E-02	SH2B2	-254307	CUX1	213216
chr9	106856281	106856591	0.58	1.98E-02	SMC2	-105		
chr7	87562972	87563397	0.58	1.98E-02	ADAM22	-517		
chr19	36706419	36706729	0.62	1.99E-02	ZNF565	-1008		
chr3	191849739	191849984	0.53	2.00E-02	FGF12	276976	PYDC2	670910

chr6	36727006	36727279	0.49	2.00E-02	CPNE5	80077	CDKN1A	80684
chr7	18560191	18560501	0.62	2.00E-02	HDAC9	24461	TWIST1	596949
chr7	5371034	5371319	0.52	2.02E-02	SLC29A4	48616	TNRC18	92000
chr1	52499365	52499675	0.68	2.02E-02	KTI12	-48		
chr8	22832149	22832401	0.57	2.02E-02	PEBP4	-46854	RHOBTB2	-21086
chr18	21589671	21589980	0.59	2.02E-02	CABYR	-129129	LAMA3	320264
chr18	3247211	3247503	0.54	2.04E-02	MYL12A	-171		
chr6	3024105	3024362	0.66	2.04E-02	RIPK1	-52764	NQO2	24167
chr1	39735377	39735703	0.63	2.05E-02	MACF1	-61270	NDUFS5	243534
chr17	39852271	39852590	0.69	2.05E-02	GAST	-16182	EIF1	7304
chr7	33102602	33102988	0.60	2.05E-02	NT5C3	-386		
chr7	66205374	66205731	0.56	2.08E-02	RABGEF1	570		
chr1	11029217	11029500	0.56	2.08E-02	CASZ1	-172652	TARDBP	-43320
chr17	18804262	18804573	0.67	2.09E-02	SLC5A10	-51060	PRPSAP2	42926
chr14	71583171	71583593	0.64	2.09E-02	SIPA1L1	-412647	PCNX	209260
chr2	84686369	84686736	0.55	2.11E-02	SUCLG1	33		
chr19	14629143	14629387	0.51	2.11E-02	DNAJB1	-64		
chr15	60973556	60973828	0.47	2.13E-02	RORA	-53963		
chr7	36231505	36231941	0.60	2.14E-02	ANLN	-197709	EEPD1	38887
chr3	16557346	16557844	0.70	2.15E-02	RFTN1	-2373		
chr17	65362600	65362910	0.75	2.16E-02	PSMD12	-34		
chr7	7221733	7222129	0.53	2.16E-02	C1GALT1	-315		
chr3	187662495	187662971	0.59	2.16E-02	LPP	-267988	BCL6	-199258
chr20	49575031	49575379	0.43	2.18E-02	MOCS3	-158	DPM1	-145
chr11	102980041	102980313	0.61	2.20E-02	DYNC2H1	17		
chr15	68522166	68522609	0.61	2.20E-02	CLN6	-308		
chr6	30698601	30698960	0.54	2.24E-02	TUBB	10624	FLOT1	11672
chr19	50179598	50180064	0.36	2.26E-02	PRMT1	-665		
chr6	31409456	31409702	0.50	2.26E-02	HCP5	-21378	MICA	38208
chr13	41722872	41723182	0.51	2.26E-02	KBTBD6	-16091	KBTBD7	45675
chr6	32659868	32660112	0.58	2.27E-02	HLA-DQA2	-49173	HLA-DQB1	-25524
chr17	56083834	56084144	0.61	2.31E-02	SFRS1	718		
chr3	56817942	56818196	0.82	2.31E-02	ERC2	-315678	ARHGEF3	295267
chr4	47916441	47916784	0.65	2.31E-02	NFXL1	20		
chr8	42249804	42250152	0.59	2.37E-02	VDAC3	632		
chr2	136874550	136875038	0.67	2.38E-02	CXCR4	931		
chr6	41286576	41286885	0.55	2.38E-02	TREM1	-32274	NCR2	-16797
chr10	3853662	3853972	0.68	2.39E-02	KLF6	-26344		

chr14	89290690	89291000	0.59	2.39E-02	TTC8	-73		
chr7	150594934	150595300	0.60	2.42E-02	ABP1	45544	KCNH2	79897
chr1	175288633	175288943	0.76	2.42E-02	TNN	251794	TNR	423964
chr3	134204488	134205101	0.36	2.44E-02	CEP63	-252	ANAPC13	68
chr7	915778	916171	0.53	2.45E-02	C7orf20	-216		
chr8	42195884	42196212	0.52	2.46E-02	POLB	18		
chr1	162467389	162467699	0.63	2.48E-02	UHMK1	-111		
chr11	60929105	60929544	0.68	2.49E-02	VPS37C	-409		
chr3	39425623	39426109	0.56	2.56E-02	RPSA	-22338	SLC25A38	1051
chr5	150535126	150535375	0.61	2.58E-02	TNIP1	-74254	ANXA6	2116
chr11	67423822	67424132	0.64	2.58E-02	ACY3	-5847	ALDH3B2	24708
chr17	55979060	55979306	0.63	2.59E-02	MRPS23	-51784	VEZF1	86432
chr6	5260820	5261301	0.61	2.60E-02	FARS2	-523	LYRM4	107
chr19	11190711	11190963	0.44	2.60E-02	LDLR	-9220	SMARCA4	119239
chr6	99873180	99873563	0.60	2.61E-02	SFRS18	-165		
chr11	111779939	111780189	0.68	2.64E-02	HSPB2	-3396	CRYAB	2409
chr1	40282698	40283298	0.61	2.67E-02	BMP8B	-28465	TRIT1	66179
chr12	56321689	56321951	0.59	2.67E-02	DGKA	-3992	WIBG	-123
chr5	140892655	140892974	0.60	2.69E-02	PCDHGC5	24007	DIAPH1	105807
chr11	94987490	94987817	0.61	2.70E-02	CEP57	-535988	SESN3	-23408
chr6	31763518	31763829	0.56	2.71E-02	VARS	38		
chr9	130497619	130497929	0.60	2.71E-02	TOR2A	-170		
chr13	51086192	51086547	0.70	2.71E-02	RNASEH2B	-397522	ST13	340216
chr8	48872516	48872951	0.62	2.76E-02	MCM4	-760	PRKDC	9
chr6	5261496	5261806	0.62	2.77E-02	LYRM4	-483	FARS2	67
chr10	104404631	104405065	0.57	2.77E-02	TRIM8	596		
chr2	232537693	232538022	0.54	2.78E-02	NMUR1	-142676	PTMA	-35377
chr3	194392900	194393401	0.35	2.83E-02	LSG1	55		
chr8	41480475	41480843	0.59	2.85E-02	NKX6-3	24216	AGPAT6	44952
chr6	155739126	155739462	0.62	2.85E-02	TFB1M	-103668	NOX3	37743
chr10	60144854	60145413	0.63	2.86E-02	TFAM	-42		
chr1	76192276	76192586	0.61	2.87E-02	RABGGTB	-59455	ACADM	2388
chr3	48342594	48343055	0.63	2.88E-02	NME6	23		
chr6	13526826	13527389	0.56	2.88E-02	SIRT5	-47767	GFOD1	-39321
chr11	22647337	22647693	0.68	2.92E-02	FANCF	-128		
chr5	90676792	90677105	0.54	2.95E-02	ARRDC3	2200	GPR98	822332
chr4	180911257	180911567	0.57	2.95E-02				
chr10	122610658	122611313	0.59	2.97E-02	BRWD2	291		

chr6	32383276	32383521	0.60	2.97E-02	HLA-DRA	-24248	BTNL2	-8499
chr8	120921463	120921773	0.49	3.01E-02	COL14A1	-215734	DEPDC6	35718
chr2	60782394	60782902	0.58	3.01E-02	BCL11A	-2015		
chr10	74094664	74094969	0.58	3.04E-02	DNAJB12	20090	DDIT4	61140
chr3	52444980	52445290	0.56	3.04E-02	BAP1	-1126	PHF7	608
chr15	68076698	68077014	0.62	3.05E-02	LBXCOR1	-41085	MAP2K5	241835
chr5	150503659	150504164	0.63	3.08E-02	TNIP1	-42915	ANXA6	33455
chr13	50071109	50071419	0.81	3.08E-02	PHF11	1463	RCBTB1	88455
chr16	30470121	30470465	0.62	3.09E-02	ITGAL	-13690	SEPHS2	-13069
chr6	89791274	89791604	0.51	3.09E-02	PNRC1	1010	SFRS13B	36361
chr12	94815945	94816255	0.54	3.09E-02	TMCC3	228224	PLXNC1	273601
chr6	24919843	24920153	0.66	3.13E-02	FAM65B	-8803	DKFZp686H12134	218053
chr5	40755784	40756228	0.64	3.13E-02	TTC33	66		
chr14	64970111	64970421	0.66	3.14E-02	ZBTB1	-1026	ZBTB25	288
chr5	6633174	6633441	0.68	3.14E-02	SRD5A1	-192	NSUN2	-151
chr2	6450063	6450400	0.54	3.15E-02	CMPK2	555704	SOX11	617433
chr7	66386979	66387404	0.56	3.17E-02	C7orf42	989		
chr19	42784441	42784751	0.66	3.18E-02	CIC	-4221		
chr7	64459687	64460102	0.66	3.19E-02	ZNF117	-8481	ERV3	7226
chr5	95159933	95160243	0.57	3.19E-02	GLRX	-1511		
chr17	73201535	73201859	0.69	3.19E-02	NUP85	100		
chr12	125348254	125348564	0.55	3.19E-02	SCARB1	110		
chr17	77970866	77971252	0.77	3.26E-02	CBX4	-157846	TBC1D16	38588
chr6	33216108	33216384	0.60	3.26E-02	VPS52	23416	RING1	39960
chr10	103815863	103816173	0.59	3.27E-02	C10orf76	-86		
chr6	24936016	24936326	0.66	3.29E-02	FAM65B	-24976	DKFZp686H12134	201880
chr1	32254116	32254426	0.68	3.34E-02	BAI2	-24623	SPOCD1	27309
chr2	61067985	61068686	0.48	3.35E-02	REL	-40416	PAPOLG	84953
chr16	84633372	84633744	0.58	3.38E-02	KIAA1609	-95270	COTL1	18111
chr6	89351163	89351554	0.55	3.39E-02	CNR1	-475592	RNGTT	321989
chr7	35577063	35577373	0.64	3.41E-02	TBX20	-283976	HERPUD2	157554
chr16	69830251	69830561	0.73	3.42E-02	CLEC18A	-154220	WWP2	34132
chr7	2749368	2749736	0.58	3.42E-02	AMZ1	30389	GNA12	134407
chr18	77782749	77783002	0.76	3.42E-02	TXNL4A	-34344	C18orf22	-11482
chr20	62580539	62580910	0.55	3.45E-02	UCKL1	7043	DNAJC5	54207
chr10	38299198	38299508	0.68	3.46E-02	ZNF33A	-225		
chr8	67525476	67525881	0.65	3.48E-02	MYBL1	-199		
chr11	3875624	3875917	0.68	3.51E-02	STIM1	-1162		

chr6	41701857	41702217	0.63	3.51E-02	TFEB	761		
chr1	24286075	24286418	0.55	3.53E-02	PNRC2	-54		
chr6	34216839	34217124	0.67	3.59E-02	C6orf1	-78		
chr2	27579318	27579782	0.57	3.60E-02	GTF3C2	318		
chr6	47215240	47215550	0.72	3.60E-02	GPR110	-205313	TNFRSF21	62285
chr4	25225396	25225706	0.62	3.60E-02	SEPSECS	-63347	PI4K2B	-10102
chr12	121124194	121124830	0.47	3.61E-02	MLEC	-437		
chr7	5534320	5534596	0.53	3.63E-02	TNRC18	-71281	FBXL18	18941
chr7	90338157	90338449	0.66	3.68E-02	PFTK1	-409		
chr11	64615678	64616012	0.54	3.69E-02	CDC42BPG	-3804		
chr12	8113308	8113670	0.67	3.75E-02	FOXJ2	-71870	SLC2A3	-24597
chr10	102289546	102289951	0.66	3.81E-02	NDUFB8	-113		
chr6	42713947	42714495	0.65	3.83E-02	TBCC	-337		
chr20	34252765	34253120	0.69	3.87E-02	RBM12	-95	CPNE1	-95
chr14	94429399	94429739	0.69	3.88E-02	OTUB2	-63155	ASB2	-5802
chr5	132387361	132387823	0.42	3.90E-02	HSPA4	-70		
chr6	159515557	159516032	0.59	3.90E-02	FNDC1	-74634	TAGAP	-49611
chr12	2921513	2921823	0.68	3.92E-02	ITFG2	-195		
chr7	100183648	100184218	0.46	3.94E-02	FBXO24	-3263	LRCH4	-157
chr12	656192	656447	0.54	3.95E-02	B4GALNT3	86777	NINJ2	116435
chr3	52755022	52755277	0.45	3.99E-02	SPCS1	15293	NEK4	49801
chr7	91875147	91875507	0.60	4.01E-02	ANKIB1	-221	KRIT1	87
chr19	7405939	7406273	0.58	4.02E-02	INSR	-112095	ARHGEF18	-98468
chr17	7117255	7117565	0.63	4.05E-02	ASGR1	-34527	DLG4	3518
chr7	24859905	24860236	0.57	4.06E-02	DFNA5	-62988	OSBPL3	159689
chr10	5882240	5882596	0.62	4.09E-02	FBXO18	-53931	GDI2	-26906
chr2	65597807	65598117	0.58	4.10E-02	SPRED2	61694	ACTR2	143133
chr7	73148870	73149327	0.61	4.14E-02	STX1A	-15111	ABHD11	4091
chr1	205564572	205564926	0.68	4.14E-02	MFSD4	26637	SLC45A3	84881
chr11	57508680	57508938	0.65	4.15E-02	C11orf31	87		
chr19	2086587	2086897	0.65	4.15E-02	MKNK2	-35499	MOBKL2A	9527
chr12	46660745	46661050	0.75	4.18E-02	SFRS2IP	-276497	SLC38A1	2310
chr3	9791409	9791734	0.60	4.20E-02	OGG1	-56		
chr19	10946955	10947265	0.66	4.20E-02	TMED1	-127		
chr12	118813924	118814322	0.68	4.21E-02	TAOK3	-3373	SUDS3	-235
chr17	74456697	74456954	0.59	4.24E-02	UBE2O	-7538	AANAT	-6825
chr8	59720535	59720771	0.61	4.25E-02	NSMAF	-148687	TOX	311114
chr7	31162235	31162495	0.51	4.25E-02	ADCYAP1R1	70223	NEUROD6	218173

chr21	35014038	35014348	0.70	4.30E-02	ITSN1	-591	CRYZL1	-33
chr19	44281962	44282272	0.49	4.33E-02	C19orf61	-22975	KCNN4	3292
chr12	6532656	6533095	0.67	4.36E-02	CD27	-21175	LTBR	39519
chr13	44817382	44817714	0.64	4.38E-02	ENOX1	-613935	SERP2	-130430
chr17	40168367	40168616	0.48	4.43E-02	NKIRAS2	-3595	DNAJC7	1179
chr7	92219587	92219864	0.61	4.50E-02	PEX1	-61881	CDK6	246215
chr1	36689290	36689976	0.57	4.53E-02	THRAP3	-384		
chr8	96243546	96243814	0.69	4.54E-02	PLEKHF2	97642	GDF6	929340
chr11	48040166	48040476	0.60	4.56E-02	OR4B1	-198041	PTPRJ	38211
chr6	15901310	15901718	0.66	4.57E-02	DTNBP1	-238243	MYLIP	-227803
chr1	115259262	115259792	0.62	4.58E-02	NRAS	-12		
chr8	102063952	102064220	0.50	4.63E-02	YWHAZ	-98865	ZNF706	153874
chr8	38853799	38854231	0.59	4.64E-02	ADAM9	-490	TM2D2	26
chr20	34042392	34042765	0.66	4.74E-02	CEP250	-571		
chr13	53226822	53227233	0.59	4.74E-02	SUGT1	197		
chr1	204490360	204490791	0.66	4.74E-02	MDM4	5065	LRRN2	164021
chr5	74632606	74633077	0.57	4.77E-02	HMGCR	-151		
chr7	5271433	5271697	0.59	4.77E-02	SLC29A4	-50996	WIPI2	41730
chr8	38324185	38324732	0.67	4.77E-02	FGFR1	1893	LETM2	80439
chr20	57987907	57988217	0.63	4.79E-02	PHACTR3	-191541	EDN3	112563
chr10	69834585	69834936	0.70	4.81E-02	HERC4	342		
chr12	131355441	131355751	0.71	4.90E-02	RAN	-1021		
chr8	42911303	42911550	0.62	4.90E-02	FNTA	-15		
chr2	27651117	27651608	0.65	4.90E-02	NRBP1	178		
chr6	30181105	30181449	0.62	4.91E-02	TRIM26	-124		
chr6	41978131	41978566	0.54	4.93E-02	CCND3	-68797	TAF8	-39902
chr6	31509612	31510213	0.58	4.95E-02	BAT1	312		
chr8	98655781	98656146	0.71	4.96E-02	MTDH	-443		
chr6	111136343	111136757	0.55	4.98E-02	CDC2L6	-138		
chr17	47287858	47288168	0.60	4.98E-02	GNGT2	-1270	ABI3	424
chr19	7239592	7239997	0.61	4.99E-02	INSR	54216	ZNF557	170324

Nonsignificant								
chr5	140943769	140944022	1.59	5.00E-02	DIAPH1	54726	PCDHGC5	75088
chr20	30640591	30640887	1.54	5.00E-02	HCK	682		
chrX	155259740	155260033	0.68	5.02E-02	WASH1	9920		
chr12	94070928	94071221	1.45	5.03E-02	CRADD	-76		
chr17	16877172	16877416	1.66	5.05E-02	TNFRSF13B	-1892		
chr19	50380554	50381084	0.39	5.05E-02	AKT1S1	-207	TBC1D17	-10
chr17	6939034	6939293	1.44	5.06E-02	SLC16A13	-230		
chr19	21688206	21688482	0.57	5.07E-02	ZNF429	-93		
chr6	134568316	134568603	1.65	5.07E-02	SLC2A12	-194671	SGK1	70736
chr4	185202564	185202864	1.75	5.07E-02	ENPP6	-63600	IRF2	193012
chr13	92001186	92001488	1.40	5.07E-02	GPC5	-49598		
chr19	48763379	48763684	1.67	5.08E-02	ZNF114	-10970	CARD8	-10607
chr19	17862079	17862484	0.63	5.08E-02	B3GNT3	-43637	MAP1S	31979
chr6	1861080	1861519	1.71	5.09E-02	FOXC1	250619	GMDS	384546
chr11	66568550	66568853	1.81	5.09E-02	SPTBN2	-79832	RCE1	-42181
chr17	80415912	80416192	1.52	5.10E-02	NARF	-488		
chr17	39844723	39845274	0.62	5.11E-02	EIF1	-128		
chr19	50934885	50935195	1.62	5.11E-02	MYBPC2	-1120		
chr4	106629687	106630058	1.49	5.11E-02	GSTCD	-2097	INTS12	8
chr6	26197307	26197703	0.72	5.11E-02	HIST1H2BF	-2282	HIST1H3D	1959
chr3	38178426	38178890	1.42	5.13E-02	MYD88	-1311	ACAA1	75
chr12	94542959	94543203	0.47	5.13E-02	PLXNC1	582		
chr12	132628798	132629091	1.48	5.13E-02	DDX51	-65	NOC4L	-48
chr14	107287443	107287687	0.56	5.15E-02	IGHV7-81	-4282		
chr17	45000216	45000633	0.74	5.16E-02	GOSR2	-61		
chr11	110198870	110199202	2.24	5.16E-02	FDX1	-101558	RDX	-31599
chr7	140179138	140179433	1.60	5.17E-02	MKRN1	83		
chr8	41521399	41521709	0.53	5.18E-02	NKX6-3	-16679	ANK1	232726
chr9	116037536	116037986	1.54	5.18E-02	PRPF4	-213	CDC26	108
chr7	101948772	101949051	0.58	5.19E-02	PRKRIP1	-87892	SH2B2	20507
chr11	66035741	66036100	1.52	5.20E-02	RAB1B	-135		
chr3	38125871	38126107	0.66	5.20E-02	DLEC1	45293	ACAA1	52744
chr20	2082737	2083356	0.36	5.25E-02	STK35	-367		
chr11	269224	269645	0.56	5.25E-02	NLRP6	-9135	PSMD13	32627
chr15	100052206	100052542	1.51	5.25E-02	MEF2A	-53759	LRRC28	260722
chr15	72766388	72766727	0.68	5.26E-02	ARIH1	-109		
chr13	99739848	99740204	1.32	5.27E-02	DOCK9	-1366		

chr20	57482868	57483187	0.60	5.28E-02	TH1L	-73283	GNAS	54992
chr12	48276969	48277303	1.56	5.29E-02	HDAC7	-63373	VDR	21678
chr1	45189161	45189435	0.54	5.30E-02	KIF2C	-16192	C1orf228	48904
chr6	43692852	43693096	0.64	5.30E-02	VEGFA	-44979	MRPS18A	-37446
chr7	108166345	108167093	1.62	5.31E-02	PNPLA8	-81		
chr17	4920214	4920648	0.66	5.37E-02	GPR172B	18296	KIF1C	19155
chr2	179279401	179279677	1.54	5.38E-02	PRKRA	36419	OSBPL6	94568
chr10	105230937	105231285	0.73	5.39E-02	CALHM1	-12463	CALHM3	7886
chr8	52814691	52814977	0.74	5.40E-02	PCMTD1	-3099		
chr1	144918027	144918303	1.88	5.40E-02	PPIAL4A	-553919	PDE4DIP	76857
chr20	43214984	43215362	1.64	5.41E-02	PKIG	54737	ADA	65203
chr19	47734503	47734826	1.45	5.41E-02	BBC3	1358	SAE1	100585
chr14	77428116	77428541	1.69	5.44E-02	C14orf4	66705	C14orf166B	135604
chr16	74401812	74402289	0.57	5.44E-02	LOC283922	102		
chr12	120730644	120731107	2.00	5.45E-02	PXN	-27313	SIRT4	-9287
chr1	6086213	6086519	1.50	5.45E-02	KCNAB2	-14		
chr2	27294467	27294781	1.33	5.46E-02	LOC100128731	-57		
chr1	38388546	38388892	0.62	5.46E-02	MTF1	-63427	INPP5B	24010
chr1	173379837	173380368	1.56	5.46E-02	TNFSF4	-203632	PRDX6	-66383
chr20	30623230	30623519	1.59	5.46E-02	HCK	-16682	XKR7	67570
chr7	128095584	128096043	1.63	5.47E-02	C7orf68	-70		
chr12	8796938	8797250	1.65	5.48E-02	AICDA	-31652	MFAP5	18339
chr9	140024070	140024342	1.46	5.49E-02	DPP7	-15011	GRIN1	-9403
chr3	170075170	170075520	1.44	5.50E-02	SKIL	-128		
chr11	130051331	130051621	1.60	5.50E-02	ST14	21794	ZBTB44	133131
chr7	44790614	44790911	1.47	5.51E-02	PPIA	-45478	ZMIZ2	2233
chr19	37019597	37020064	1.62	5.51E-02	ZNF260	-661		
chr4	25880327	25880655	0.70	5.54E-02	C4orf52	-35323	SEL1L3	-15881
chr7	140090722	140090985	1.50	5.54E-02	JHDM1D	-214113	SLC37A3	7457
chr14	74181082	74181403	0.46	5.55E-02	PNMA1	-115		
chr10	105880649	105880915	1.59	5.55E-02	GSTO1	-133896	COL17A1	-35144
chr2	26101229	26101772	1.41	5.56E-02	ASXL2	-189		
chr19	1407195	1407590	1.42	5.56E-02	DAZAP1	-191		
chr19	57791584	57791931	1.42	5.56E-02	ZNF460	-95		
chr22	43583507	43583855	1.52	5.56E-02	TTLL12	-574		
chr3	177076500	177076744	1.59	5.57E-02	TBL1XR1	-161574		
chr2	8822076	8822319	1.49	5.57E-02	ID2	214		
chrX	148622375	148622619	1.68	5.57E-02	CXorf40A	-60	CXorf40A	-46

chr2	32235856	32236340	1.49	5.58E-02	MEMO1	-400		
chr10	101774149	101774434	1.55	5.59E-02	DNMBP	-4616		
chr1	247553549	247553825	1.56	5.59E-02	ZNF496	-58642	NLRP3	-25771
chr17	79829134	79829475	1.59	5.62E-02	ARHGDIA	-67		
chr7	92439078	92439351	1.62	5.63E-02	PEX1	-281370	CDK6	26726
chr5	173248181	173248470	1.81	5.63E-02	BOD1	-204660	CPEB4	-67005
chr5	180402381	180402684	1.77	5.64E-02	BTNL3	-13368	BTNL8	76456
chr3	133207817	133208088	0.64	5.64E-02	CDV3	-84481	BFSP2	89163
chr11	64618690	64619096	1.61	5.65E-02	CDC42BPG	-6852	EHD1	27298
chr4	185249187	185249851	1.53	5.65E-02	ENPP6	-110405	IRF2	146207
chr1	14029298	14029638	1.48	5.65E-02	PRDM2	-1882		
chr12	117299247	117299598	1.84	5.66E-02	HRK	19809	RNFT2	123327
chr1	112912289	112912618	0.58	5.67E-02	KCND3	-380677	CTTNBP2NL	-26346
chr15	65184418	65184696	1.65	5.67E-02	PIF1	-66719	ANKDD1A	-19544
chr4	57623944	57624254	0.76	5.68E-02	HOPX	-101411	SPINK2	63794
chr15	77480161	77480471	1.92	5.69E-02	TSPAN3	-116803	SGK269	97017
chr1	145516101	145516377	1.64	5.70E-02	PEX11B	-144		
chr11	118077230	118077540	0.51	5.70E-02	SCN2B	-30048	AMICA1	18424
chr16	30645446	30645967	0.68	5.70E-02	FBRS	-30071	ZNF689	-24025
chr4	76439374	76439795	0.68	5.71E-02	THAP6	-69	RCHY1	43
chr1	65329700	65330025	0.63	5.72E-02	JAK1	102324	RAVER2	119085
chr6	53658399	53658684	1.40	5.77E-02	LRRC1	-992		
chr2	20550620	20550877	1.50	5.77E-02	RHOB	-96086	PUM2	-23610
chr1	54738706	54738979	1.45	5.77E-02	MRPL37	73003	SSBP3	133249
chr6	21593755	21594157	0.64	5.80E-02	SOX4	-16		
chr3	319975	320258	1.68	5.81E-02	CNTN6	-814512	CHL1	81467
chr19	5968040	5968289	0.71	5.82E-02	RANBP3	10155	CAPS	53972
chr7	46508427	46508663	0.63	5.82E-02	IGFBP3	-547674		
chr12	14766704	14767039	1.61	5.85E-02	PLBD1	-46081	GUCY2C	82647
chr18	20513134	20513725	0.65	5.85E-02	RBBP8	135		
chr5	124084379	124084689	0.66	5.85E-02	ZNF608	-3669		
chr15	64752507	64752858	1.39	5.86E-02	ZNF609	-38936	TRIP4	72663
chr4	129209152	129209454	1.44	5.88E-02	PGRMC2	-355		
chr20	36156254	36156564	0.69	5.88E-02	BLCAP	-106		
chr20	43093924	43094172	0.55	5.89E-02	TTPAL	-10499	HNF4A	64124
chr6	151086371	151086840	1.50	5.89E-02	MTHFD1L	-100085	PLEKHG1	165607
chr14	22749534	22749873	0.64	5.89E-02	TCRDV2	-178385	TRA	273940
chr10	101605635	101605879	1.52	5.89E-02	ABCC2	63294	DNMBP	163919

chr11	121300943	121301310	1.56	5.90E-02	SORL1	-21834	SC5DL	137739
chr19	16370436	16370827	1.49	5.90E-02	KLF2	-65019	AP1M1	61967
chr4	82483342	82483618	1.81	5.90E-02	RASGEF1B	-90419	HNRNPD	811669
chr2	226140979	226141271	2.12	5.90E-02	DOCK10	-233795		
chr3	196369978	196370483	1.60	5.91E-02	PIGX	-69015	LRRC33	3575
chr9	115141905	115142364	1.52	5.91E-02	HSDL2	-216		
chr19	45594792	45595045	0.62	5.92E-02	GEMIN7	12401	NKPD1	68489
chr4	84133654	84134077	1.32	5.93E-02	COQ2	72098	COPS4	177627
chr18	46986220	46986530	0.70	5.94E-02	DYM	704		
chr13	27998540	27998913	1.37	5.94E-02	GTF3A	46		
chr2	44706562	44706853	1.44	5.95E-02	SIX3	-462329	PREPL	-119819
chr7	105697916	105698290	0.75	5.99E-02	SYPL1	54954	FLJ23834	94446
chrX	1600645	1600994	1.57	6.04E-02	ASMTL	-28976	P2RY8	55217
chr11	66886454	66886855	1.31	6.04E-02	KDM2A	-85		
chr11	19575336	19575646	0.58	6.05E-02	E2F8	-312984	NAV2	-159390
chr6	138132585	138132831	1.58	6.05E-02	OLIG3	-317177	TNFAIP3	-55873
chr19	57946500	57946837	1.33	6.06E-02	ZNF749	-24		
chr9	37294083	37294386	1.50	6.06E-02	GRHPR	-128472	ZCCHC7	173766
chr19	48018334	48018939	1.50	6.06E-02	NAPA	-140		
chr3	42054160	42054809	0.58	6.07E-02	TRAK1	-78261	ULK4	-50825
chr12	14537833	14538129	1.38	6.08E-02	ATF7IP	19370	PLBD1	182810
chr11	119205051	119205295	1.55	6.09E-02	RNF26	-64		
chr10	14640449	14640740	1.61	6.09E-02	FRMD4A	-267729	CDNF	239388
chr21	34587894	34588204	1.64	6.13E-02	IFNAR2	-14182	OLIG1	145599
chr6	33575588	33575845	0.55	6.13E-02	BAK1	-27647	ITPR3	-13444
chr3	23705417	23705727	0.44	6.13E-02	UBE2E1	-141867	UBE2E2	460788
chr7	135194425	135194787	0.70	6.14E-02	CNOT4	245		
chr11	66115444	66115817	1.44	6.18E-02	BRMS1	-3049	B3GNT1	-470
chr16	27437744	27438054	1.72	6.20E-02	IL21R	-680		
chr6	107978974	107979241	0.66	6.21E-02	SCML4	166413	SOBP	167791
chr2	10588774	10589018	1.29	6.22E-02	ODC1	-443		
chr16	85603893	85604169	1.62	6.22E-02	KIAA0182	-42893	KIAA0513	542621
chr2	88301252	88301562	0.71	6.23E-02	RGPD1	-176092	SMYD1	-65975
chr19	8260052	8260401	0.68	6.26E-02	FBN3	-47846	LASS4	-13990
chr7	104585523	104585914	0.56	6.28E-02	MLL5	-68918	LHFPL3	616615
chr19	40030689	40030959	1.47	6.28E-02	EID2	14		
chr11	17297927	17298410	0.67	6.29E-02	NUCB2	-117		
chr10	74008576	74008913	0.61	6.29E-02	DDIT4	-24932	C10orf104	32939

chr4	139163397	139163695	1.65	6.31E-02	SLC7A11	-43		
chr7	30485484	30485788	0.62	6.32E-02	NOD1	32757	ZNRF2	161713
chr3	71613596	71613864	1.60	6.32E-02	FOXP1	19410		
chr22	48494099	48494452	1.80	6.33E-02	FAM19A5	-391012		
chr11	128563712	128564041	1.78	6.34E-02	FLI1	64		
chr15	57025721	57025997	1.76	6.38E-02	ZNF280D	-72		
chr18	72166454	72166716	1.75	6.39E-02	CNDP1	-35107	CNDP2	2988
chr14	60679264	60679574	1.77	6.39E-02	DHRS7	-47208	PPM1A	-33051
chr20	55926137	55926394	1.48	6.40E-02	RAE1	-352		
chr14	107238866	107239256	0.65	6.40E-02	ADAM6	-800703	IGHV7-81	44222
chr15	40857158	40857434	1.58	6.43E-02	RPUSD2	-4241		
chr20	18774536	18774819	1.51	6.43E-02	C20orf79	-19692	DTD1	206122
chr11	95524126	95524479	0.80	6.43E-02	CEP57	661		
chr1	6052683	6052959	1.45	6.44E-02	NPHP4	-290		
chr2	8374799	8375185	1.53	6.44E-02	ID2	-446992		
chr13	21714473	21714739	1.38	6.46E-02	SAP18	-47		
chr3	111697694	111697958	1.47	6.46E-02	ABHD10	-2		
chr5	157023467	157023799	1.68	6.46E-02	ADAM19	-20865	SOX30	55795
chr7	100157431	100157702	0.65	6.46E-02	SAP25	13874	AGFG2	20733
chr3	42623564	42623874	0.60	6.48E-02	SEC22C	-291		
chrX	53449684	53449960	1.49	6.49E-02	SMC1A	-204		
chr19	19030183	19030567	1.52	6.49E-02	COPE	-176	DDX49	-119
chr10	4178401	4178659	0.57	6.49E-02	AKR1E2	-689872	KLF6	-351057
chr22	43165705	43166029	1.50	6.49E-02	A4GALT	-48991	ARFGAP3	87541
chr3	13493268	13493569	1.49	6.51E-02	NUP210	-31610	HDAC11	-28296
chr14	74238430	74238918	0.63	6.51E-02	PNMA1	-57546	C14orf43	15222
chr11	111944617	111945150	1.62	6.53E-02	TIMM8B	12575	DLAT	49346
chr19	47291683	47291955	1.44	6.54E-02	SLC1A5	23		
chr19	51611335	51611886	1.42	6.56E-02	CTU1	36		
chr9	135817924	135818234	0.77	6.58E-02	C9orf98	-63881	TSC1	1941
chr6	137540453	137540731	1.58	6.60E-02	IFNGR1	-25		
chr15	62637153	62637397	1.61	6.60E-02	TLN2	-302235	C2CD4B	-179793
chr7	105172262	105172943	0.59	6.63E-02	RINT1	71		
chr19	54960131	54960468	1.45	6.65E-02	LENG8	235		
chr19	7294269	7294550	1.25	6.68E-02	INSR	-399		
chr8	145026257	145026542	1.42	6.68E-02	PLEC1	-1356		
chr1	158979475	158979862	1.71	6.69E-02	IFI16	-13		
chr19	49378543	49379073	1.48	6.71E-02	PPP1R15A	3159	TULP2	23188

chr17	4046182	4046601	0.63	6.71E-02	ZZEF1	-139	CYB5D2	-70
chr15	31522984	31523594	1.79	6.71E-02	TRPM1	-129365	KLF13	-95794
chr13	99141022	99141507	1.48	6.71E-02	RNF113B	-311744	STK24	88131
chr6	30582653	30583047	0.72	6.72E-02	MRPS18B	-2636	PPP1R10	2170
chr20	44563766	44564042	1.31	6.75E-02	PCIF1	587		
chr1	212208299	212208720	1.67	6.76E-02	DTL	-409	INTS7	374
chr17	63096425	63096903	0.73	6.76E-02	GNA13	-43744	RGS9	-36885
chr2	43360127	43360426	1.50	6.76E-02	HAAO	-340526	ZFP36L2	93468
chr6	70506692	70507151	0.61	6.77E-02	LMBRD1	-34		
chr10	70360028	70360304	1.43	6.77E-02	CCAR1	-120805	TET1	40049
chr14	106048121	106048712	1.53	6.78E-02	TMEM121	55464	IGHE	281045
chr1	11968177	11968696	1.86	6.78E-02	NPPB	-49445	KIAA2013	18043
chr7	10980174	10980454	0.66	6.79E-02	NDUFA4	-501		
chr10	91403610	91404016	0.62	6.79E-02	SLC16A12	-108500	PANK1	1402
chr14	65753214	65753496	1.54	6.81E-02	MAX	-184128	FUT8	-126180
chr5	135547898	135548144	0.63	6.86E-02	SMAD5	79485	TRPC7	145052
chr7	73482156	73482469	0.68	6.86E-02	LIMK1	-15843	ELN	39886
chr8	91640054	91640338	0.74	6.86E-02	CALB1	-545089	TMEM64	17937
chr15	50736871	50737143	1.56	6.88E-02	USP8	20428	USP50	101895
chr19	3626767	3627221	0.69	6.89E-02	C19orf29	-181		
chr11	120042984	120043260	1.77	6.89E-02	POU2F3	-67829	TRIM29	-34259
chr20	31533646	31534055	0.69	6.93E-02	SPAG4L	58388	EFCAB8	87122
chr17	76238552	76238873	0.62	6.93E-02	LOC283999	11322	SOCS3	117445
chr8	61911668	61912000	0.67	6.93E-02	RLBP1L1	-288691	CHD7	320495
chr16	75498400	75498893	0.65	6.94E-02	TMEM170A	-63		
chr2	182177185	182177658	1.48	7.00E-02	ITGA4	-144197	UBE2E3	332310
chr9	139378169	139378502	1.29	7.02E-02	SEC16A	-829	C9orf163	389
chr2	60666598	60666886	1.62	7.02E-02	BCL11A	113891		
chr1	178577611	178577917	0.88	7.04E-02	RALGPS2	-116536	C1orf49	95552
chr17	48944742	48945157	0.61	7.04E-02	TOB1	-3537		
chr9	893741	894216	1.53	7.07E-02	DMRT3	-82985	DMRT1	52289
chr1	197115759	197116214	0.72	7.07E-02	ASPM	-163		
chr4	120375714	120376012	1.45	7.07E-02	AX746903	-173082	FABP2	-132547
chrX	11381219	11381463	1.46	7.08E-02	AMELX	69808	ARHGAP6	302480
chrX	13105402	13105669	1.78	7.09E-02	FAM9C	-42736	ATXN3L	232982
chr3	187491071	187491347	1.58	7.11E-02	LPP	-439512	BCL6	-27734
chr8	22831249	22831635	0.66	7.13E-02	PEBP4	-46021	RHOBTB2	-21919
chr11	64851434	64851748	1.53	7.14E-02	ZFPL1	-103	CDCA5	24

chr5	90675755	90676126	0.60	7.15E-02	ARRDC3	3208	GPR98	821324
chr19	19144510	19144857	1.53	7.16E-02	SFRS14	-304	ARMC6	213
chr1	87379786	87380228	0.70	7.17E-02	HS2ST1	-328	SEP15	100
chr17	7739136	7739455	0.64	7.18E-02	KDM6B	-3939		
chr1	31971645	31971953	1.56	7.19E-02	TINAGL1	-70287	SERINC2	85836
chr6	30524904	30525274	0.70	7.23E-02	GNL1	-81	PRR3	333
chr8	67419473	67419749	1.51	7.23E-02	ADHFE1	74893	MYBL1	105869
chr1	2516873	2517155	1.52	7.26E-02	C1orf93	-1235		
chr6	43139047	43139294	0.62	7.26E-02	SRF	251		
chr9	82186638	82186986	1.42	7.26E-02	TLE4	-66		
chr13	111124153	111124464	1.65	7.26E-02	RAB20	89762	COL4A2	164678
chr19	16229367	16229719	1.52	7.27E-02	HSH2D	-15295	RAB8A	7053
chr1	33502531	33502876	0.65	7.28E-02	AK2	-212		
chr15	42066278	42066834	0.56	7.28E-02	MAPKBP1	-76		
chr17	79670238	79670593	1.49	7.28E-02	SLC25A10	-8955	HGS	19396
chr2	232180656	232180945	0.77	7.28E-02	B3GNT7	-79534	ARMC9	117459
chr7	107844015	107844405	1.38	7.29E-02	LAMB4	-73409	NRCAM	252616
chr16	67701818	67702148	0.76	7.30E-02	C16orf48	-1355		
chr19	42746830	42747107	1.45	7.31E-02	GSK3A	-233		
chr19	59084677	59085102	1.36	7.32E-02	MZF1	52		
chr14	58894189	58894682	0.54	7.33E-02	TIMM9	-204		
chr11	85339318	85339817	1.58	7.36E-02	DLG2	-1254	TMEM126B	-94
chr7	2681036	2681334	1.45	7.36E-02	AMZ1	-37978	TTYH3	9582
chr5	17278060	17278405	2.04	7.36E-02	BASP1	60483		
chr17	29648554	29649032	0.79	7.36E-02	EVI2A	-76		
chr19	50321205	50321640	1.28	7.36E-02	FUZ	-4955	MED25	-123
chr12	64943308	64943618	0.76	7.39E-02	RASSF3	-60830	TBK1	97526
chr11	119076478	119076754	1.49	7.40E-02	CBL	-374		
chr4	175849374	175849729	1.70	7.41E-02	ADAM29	10043		
chr6	8435677	8436147	0.68	7.41E-02	SLC35B3	-118		
chr5	179398012	179398279	1.81	7.42E-02	TBC1D9B	-63290	RNF130	100963
chr6	24721757	24722184	0.61	7.43E-02	C6orf62	-2568		
chr11	34126921	34127252	1.57	7.44E-02	NAT10	-24		
chr6	32812306	32812649	0.76	7.44E-02	PSMB8	-662		
chr3	112709847	112710269	1.52	7.45E-02	GTPBP8	258		
chr8	11349449	11349920	1.83	7.45E-02	BLK	-1836		
chr12	112820186	112820608	0.72	7.45E-02	C12orf51	-76359	RPL6	27046
chr12	6339951	6340249	1.51	7.46E-02	PLEKHG6	-79502	CD9	30545

chr7	50186268	50186616	0.64	7.48E-02	IKZF1	-157936	ZPBP	-53582
chr5	137688034	137688420	0.69	7.48E-02	KDM3B	-58		
chr11	81580729	81581084	1.77	7.48E-02				
chr1	156389770	156390046	1.65	7.48E-02	C1orf61	9432	RHBG	50905
chr16	3285084	3285334	1.61	7.49E-02	ZNF200	247		
chr2	74259626	74259999	1.39	7.50E-02	TET3	-13637	DGUOK	105860
chr4	190628401	190628645	1.52	7.51E-02	FRG1	-233451		
chr3	14166241	14166610	1.55	7.51E-02	CHCHD4	-55	TMEM43	-14
chr19	45251685	45251991	1.43	7.53E-02	BCL3	-140		
chr6	75994532	75994822	1.51	7.56E-02	TMEM30A	-45		
chr12	129163043	129163317	1.66	7.56E-02	SLC15A4	145361	TMEM132C	263889
chr1	25256733	25257140	0.60	7.57E-02	RUNX3	-167		
chr3	16472722	16473050	0.63	7.58E-02	RFTN1	82336	OXNAD1	166172
chr19	46365831	46366119	1.78	7.59E-02	FOXA3	-1543	SYMPK	573
chr20	52456041	52456400	0.58	7.60E-02	ZNF217	-256514	BCAS1	231083
chr11	67121064	67121516	1.50	7.60E-02	POLD4	-273		
chr11	58912117	58912411	1.53	7.61E-02	FAM111A	12		
chr1	243419189	243419452	1.59	7.62E-02	CEP170	-613	SDCCAG8	1
chr11	66379094	66379404	0.64	7.64E-02	RBM14	-4804		
chr20	40246911	40247243	0.76	7.66E-02	CHD6	56		
chr2	64954200	64954476	1.49	7.66E-02	SLC1A4	-262118	SERTAD2	-73292
chr11	73096158	73096527	1.55	7.67E-02	PLEKHB1	-262251	RELT	8938
chr17	73029866	73030118	1.60	7.68E-02	ATP5H	13082	ICT1	21212
chr7	100209384	100209841	0.62	7.69E-02	MOSPD3	-501		
chr9	34376680	34377186	0.64	7.69E-02	KIAA1161	-39		
chr1	176176675	176176980	1.63	7.71E-02	RFWD2	-458		
chr19	16427485	16427721	0.56	7.71E-02	KLF2	-8048	AP1M1	118938
chr9	93536988	93537310	1.58	7.71E-02	DIRAS2	-132041	SYK	-26863
chr11	71814389	71814744	1.57	7.75E-02	C11orf59	-245		
chr3	71613067	71613403	1.41	7.75E-02	FOXP1	19905		
chr14	23340847	23341157	0.63	7.76E-02	LRP10	42		
chr17	79565503	79565824	1.68	7.76E-02	C17orf70	-46238	NPLOC4	38474
chr2	37193904	37194178	1.30	7.77E-02	STRN	-426		
chr1	148241412	148241731	2.04	7.77E-02	NBPF15	-319275	NBPF14	-215709
chr3	113871354	113871689	1.70	7.78E-02	DRD3	26377	QTRTD1	95911
chr3	101617684	101618010	1.69	7.79E-02	ZPLD1	-536012	NFKBIZ	49489
chr8	6565651	6565969	1.43	7.80E-02	AGPAT5	-68		
chr7	35946084	35946363	0.65	7.82E-02	EEPD1	-246612	SEPT7	105597

chr14	24483221	24483497	1.71	7.82E-02	DHRS4L1	-22351	DHRS4	60394
chr1	146714019	146714355	1.33	7.85E-02	CHD1L	-104		
chr1	202858186	202858430	1.46	7.85E-02	RABIF	-45		
chr9	127703291	127703531	1.44	7.85E-02	GOLGA1	-25		
chr3	57969569	57969852	0.71	7.85E-02	FLNB	-24416	SLMAP	226537
chr18	67872789	67873100	1.50	7.86E-02	RTTN	17		
chr7	27702368	27702803	0.58	7.86E-02	HIBADH	16		
chr3	179322385	179322637	1.54	7.87E-02	MRPL47	-77	NDUFB5	-64
chr20	52556489	52556734	1.46	7.90E-02	ZNF217	-356905	BCAS1	130692
chr5	124238030	124238340	1.47	7.93E-02	ZNF608	-157320		
chr2	37311448	37311726	1.42	7.93E-02	HEATR5B	-102	CCDC75	-7
chr4	186134967	186135280	0.58	7.93E-02	SNX25	3840	LRP2BP	165028
chr7	44499951	44500213	0.73	7.93E-02	CAMK2B	-134852	NPC1L1	80832
chr1	111758808	111759118	0.68	7.94E-02	DRAM2	-76125	CHI3L2	-11318
chr1	1655692	1655986	1.50	7.98E-02	CDC2L1	-64		
chr3	194024154	194024488	0.81	8.02E-02	CPN2	47736	HES1	170387
chr6	33280546	33280853	1.51	8.07E-02	RGL2	-13962	TAPBP	1289
chr14	95961922	95962202	1.59	8.09E-02	GLRX5	-39261	C14orf49	-19889
chr19	55690084	55690394	1.42	8.09E-02	TNNI3	-21139	SYT5	1481
chr9	132751389	132751636	1.52	8.09E-02	FNBP1	53960	USP20	153817
chr7	100459245	100459514	1.55	8.10E-02	TRIP6	-5570	SLC12A9	9022
chr20	47131838	47132172	1.34	8.11E-02	SULF2	-717197	PREX1	312415
chr20	32700010	32700317	1.42	8.11E-02	EIF2S2	-79		
chr19	49894208	49894627	1.51	8.11E-02	CCDC155	2912	PTH2	32280
chr3	105572984	105573299	1.78	8.11E-02	CBLB	14745	ALCAM	487429
chr5	150576195	150576598	0.58	8.11E-02	GM2A	-56216	ANXA6	-39030
chr1	210001132	210001497	1.60	8.12E-02	C1orf107	-18		
chr20	34824194	34824470	1.66	8.14E-02	C20orf4	-115		
chr3	69101394	69101714	1.49	8.15E-02	TMF1	-70		
chr1	9686240	9686611	1.43	8.15E-02	PIK3CD	-25364	TMEM201	37449
chr5	179245969	179246252	1.39	8.19E-02	SQSTM1	-1731		
chr1	974130	974440	0.56	8.19E-02	AGRN	18782	C1orf159	77451
chr19	14685080	14685337	1.39	8.21E-02	NDUFB7	-2323		
chr3	169775894	169776240	1.69	8.22E-02	GPR160	20332	PHC3	123470
chr17	12921291	12921735	1.42	8.22E-02	ELAC2	-154		
chr6	157019449	157019744	1.69	8.24E-02	ARID1B	-79489		
chr5	134240097	134240463	0.65	8.24E-02	PCBD2	-530		
chr9	34171806	34172082	1.60	8.24E-02	DCAF12	-45173	UBAP1	-7067

chr3	101947960	101948236	1.68	8.24E-02	ZPLD1	-205761	NFKBIZ	379740
chr6	26055638	26055938	0.71	8.25E-02	HIST1H1C	911		
chr3	194304586	194304862	1.35	8.25E-02	ATP13A3	-115756	TMEM44	49389
chr2	69135602	69135928	1.55	8.26E-02	BMP10	-37116	GKN2	44337
chr3	186784351	186784808	1.60	8.29E-02	ST6GAL1	44915	RPL39L	72683
chr10	12084894	12085351	0.71	8.29E-02	UPF2	-315		
chr1	228296882	228297126	1.53	8.32E-02	MRPL55	9		
chr2	47168784	47169242	0.74	8.33E-02	TTC7A	700		
chr11	117817714	117818023	1.65	8.33E-02	IL10RA	-39237	TMPRSS13	-17754
chr17	43212696	43213066	1.39	8.35E-02	PLCD3	-2990	ACBD4	-133
chr9	3525769	3526064	0.62	8.37E-02	RFX3	66		
chr7	48031374	48031764	0.58	8.37E-02	HUS1	-12323	SUNC1	37147
chr10	74033431	74033759	0.58	8.39E-02	DDIT4	-82		
chr3	37284558	37284885	1.41	8.40E-02	GOLGA4	-16		
chr11	119544599	119545114	1.61	8.40E-02	THY1	-250611	PVRL1	54578
chr12	3334681	3334937	1.71	8.41E-02	PRMT8	-265616	TSPAN9	148252
chr14	23355940	23356310	0.59	8.41E-02	REM2	3693	RBM23	32271
chr12	12878686	12878935	1.33	8.41E-02	APOLD1	-40		
chr16	85587694	85588048	1.50	8.44E-02	KIAA0182	-59053	KIAA0513	526461
chr15	75335207	75335839	1.63	8.44E-02	PPCDC	19596	DNM1P33	259783
chr8	135844308	135844777	1.44	8.44E-02	KHDRBS3	-625173	ZFAT	-119262
chr2	131099769	131100079	0.68	8.44E-02	IMP4	-565	CCDC115	-2
chr19	40502764	40503077	1.52	8.45E-02	ZNF546	-22		
chr15	85872841	85873204	1.65	8.45E-02	AKAP13	-50848	PDE8A	347818
chr17	66494075	66494426	1.76	8.47E-02	WIPI1	-40598	PRKAR1A	-13859
chr15	66585598	66586103	0.57	8.49E-02	DIS3L	-69		
chr2	3381498	3381832	1.55	8.49E-02	TTC15	-1781	TSSC1	-12
chr1	150669493	150669915	1.51	8.49E-02	GOLPH3L	-32		
chr11	65893630	65893908	1.70	8.49E-02	KLC2	-131405	PACS1	55945
chr7	50307782	50308058	1.62	8.49E-02	ZPBP	-175060	IKZF1	-36458
chr2	182275800	182276110	0.63	8.49E-02	ITGA4	-45664	UBE2E3	430843
chr1	156710863	156711178	1.67	8.51E-02	MRPL24	-98		
chr19	46295934	46296462	0.68	8.51E-02	DMWD	-138		
chr2	25194590	25195185	1.43	8.51E-02	DNAJC27	-64		
chr14	104145722	104146062	0.74	8.51E-02	XRCC3	35931	KLC1	50367
chr19	47551828	47552191	1.33	8.51E-02	TMEM160	-128		
chr3	187456628	187456917	1.62	8.55E-02	RTP2	-36428	BCL6	6702
chr2	99952846	99953128	1.47	8.55E-02	EIF5B	-847	TXNDC9	-127

chr16	87425643	87425909	1.46	8.57E-02	MAP1LC3B	-25		
chr13	41038527	41038776	0.59	8.57E-02	FOXO1	202082	COG6	808888
chr19	48836034	48836624	1.43	8.57E-02	EMP3	7700	TMEM143	30857
chr21	44596572	44597066	1.41	8.57E-02	CRYAA	7678	SIK1	250183
chr19	59010799	59011109	1.45	8.58E-02	SLC27A5	12478	ZNF446	23159
chr8	56685815	56686125	0.63	8.58E-02	TMEM68	-85	TGS1	179
chr12	123849646	123849982	1.31	8.61E-02	SETD8	-18890	SBNO1	-14826
chr11	60128264	60128574	0.51	8.62E-02	MS4A7	-17539	MS4A6E	26064
chr6	47445073	47445487	0.68	8.63E-02	CD2AP	-245		
chr16	20915804	20916114	1.76	8.67E-02	DCUN1D3	-4398	LYRM1	3884
chr1	23670770	23671122	1.40	8.73E-02	HNRNPR	-93		
chr1	36396649	36397010	0.73	8.73E-02	EIF2C3	58		
chr6	32811637	32812018	1.46	8.73E-02	PSMB8	-12		
chr15	40885907	40886560	1.64	8.75E-02	CASC5	-213		
chr3	152094423	152094779	1.94	8.76E-02	P2RY1	-458135	TMEM14E	-35822
chr12	53773075	53773434	0.67	8.77E-02	SP1	-724		
chr11	60244476	60244838	1.56	8.78E-02	MS4A12	-15594	MS4A1	21375
chr13	74541949	74542200	1.68	8.78E-02	KLF12	165991	KLF5	908933
chr8	135612393	135612904	1.64	8.79E-02	ZFAT	112632		
chr19	47164415	47164705	1.11	8.80E-02	GNG8	-26621	PRKD2	55824
chr3	127317109	127317369	1.38	8.89E-02	MCM2	-14		
chr19	47354119	47354491	1.61	8.94E-02	AP2S1	-102		
chr1	16939970	16940439	0.52	8.97E-02	NBPF1	-223		
chr11	61582599	61583121	1.51	8.97E-02	FADS1	1669	FEN1	22710
chr2	47211063	47211674	0.63	8.98E-02	TTC7A	43056	CALM2	192371
chr9	14688314	14688624	0.75	9.02E-02	NFIB	-374524	ZDHHC21	5000
chr11	67195721	67196051	1.49	9.02E-02	RPS6KB2	-49		
chr7	4815025	4815383	0.63	9.02E-02	KIAA0415	-60		
chr8	142046133	142046481	0.76	9.03E-02	PTK2	-34975	SLC45A4	192366
chr1	1072116	1072380	0.49	9.06E-02	TTLL10	-37038	C1orf159	-20512
chr11	117924134	117924418	1.59	9.07E-02	TMPRSS4	-23451	IL10RA	67170
chr15	55977489	55977799	1.71	9.09E-02	PYGO1	-96594	PRTG	57533
chr9	101946762	101947191	1.50	9.09E-02	ALG2	37269	TGFBR1	79565
chr11	66112496	66112769	1.45	9.09E-02	BRMS1	-51		
chr8	38571855	38572107	0.76	9.10E-02	RNF5	-113206	TACC1	-72741
chr19	19517802	19518056	0.77	9.10E-02	GATAD2A	21287	TSSK6	108540
chr3	187694135	187694445	0.75	9.11E-02	LPP	-236431	BCL6	-230815
chr1	156426348	156426675	1.56	9.13E-02	C1orf61	-27172	MEF2D	44017

chr8	61821344	61821695	0.68	9.15E-02	RLBP1L1	-379005	CHD7	230181
chr8	87520852	87521124	0.62	9.17E-02	FAM82B	21		
chr22	22337050	22337417	1.69	9.18E-02	TOP3B	-87		
chr12	102333641	102333958	0.74	9.21E-02	DRAM1	62695	CCDC53	122099
chr15	44084070	44084314	1.34	9.21E-02	ELL3	-14690	SERINC4	8063
chr3	151986341	151987002	0.60	9.21E-02	MBNL1	-30522	SUCNR1	395235
chr2	32390708	32391055	1.61	9.22E-02	SLC30A6	-51		
chr17	56709343	56709616	1.41	9.23E-02	SEPT4	-102817	TEX14	59936
chr7	1543950	1544206	0.62	9.24E-02	INTS1	-60		
chr11	118306965	118307250	1.48	9.26E-02	MLL	-97		
chr2	3605858	3606161	1.45	9.29E-02	RNASEH1	-70		
chr15	75069750	75070137	1.58	9.31E-02	CSK	-4481		
chr3	174991883	174992254	1.74	9.31E-02	NAALADL2	414958		
chr17	8076590	8076988	1.89	9.31E-02	VAMP2	-10496	TMEM107	2925
chr16	66785504	66785814	0.67	9.32E-02	DYNC1LI2	-134		
chr12	16351801	16352171	1.48	9.32E-02	MGST1	-148593	DERA	287800
chr17	40761321	40761583	1.36	9.34E-02	FAM134C	-7	TUBG1	94
chr20	30135012	30135311	1.48	9.35E-02	ID1	-57930	HM13	32921
chr11	64781272	64782098	0.66	9.35E-02	ARL2	99		
chr3	128444748	128445043	1.48	9.36E-02	RAB7A	-83		
chr12	9860562	9861061	0.66	9.38E-02	CLECL1	25048	CLEC2D	38503
chr9	9612254	9612646	1.35	9.38E-02				
chr8	73920723	73921270	0.70	9.41E-02	TERF1	-100		
chr7	107383849	107384160	0.66	9.42E-02	CBLL1	-274		
chr20	30956783	30957186	1.56	9.42E-02	ASXL1	10832	COMMD7	374829
chr14	20895906	20896240	1.77	9.42E-02	TEP1	-14494	KLHL33	2521
chr4	8430096	8430349	1.32	9.43E-02	ACOX3	12229	HTRA3	158731
chr9	114837064	114837320	1.76	9.43E-02	SUSD1	100364	UGCG	177986
chr22	40831901	40832327	1.65	9.44E-02	SGSM3	65519	MKL1	200576
chr1	154600332	154600651	1.36	9.44E-02	ADAR	-19810	KCNN3	242262
chr19	14714885	14715140	1.70	9.44E-02	CLEC17A	21117	EMR3	70717
chr2	24270249	24270524	1.32	9.44E-02	FKBP1B	-2197	C2orf44	-91
chr9	102581566	102582002	1.43	9.44E-02	NR4A3	-7225	SEC61B	597214
chr15	31750031	31750356	1.44	9.45E-02	KLF13	131111	OTUD7A	197348
chr13	73356121	73356541	1.39	9.45E-02	DIS3	13		
chr12	25158187	25158691	1.55	9.47E-02	BCAT1	-56131	LRMP	-46802
chr20	19299501	19299747	0.61	9.47E-02	RIN2	-570586	SLC24A3	106334
chr19	6577346	6578175	1.75	9.48E-02	CD70	13402	TNFSF9	46751

chr6	44281001	44281311	0.69	9.49E-02	AARS2	-93		
chr22	23484050	23484409	0.69	9.49E-02	RAB36	-3283	RTDR1	11
chr8	28243630	28244081	1.15	9.51E-02	ZNF395	121		
chr9	137822290	137822667	1.66	9.51E-02	OLFM1	-144610	FCN1	-12670
chr7	73668584	73669009	1.29	9.52E-02	RFC2	-59		
chr2	68290040	68290289	1.50	9.52E-02	C1D	-6		
chr2	153573834	153574352	0.77	9.53E-02	ARL6IP6	-330	PRPF40A	-118
chr11	34072887	34073197	0.64	9.53E-02	CAPRIN1	-188		
chr1	246729415	246729840	1.56	9.53E-02	TFB2M	-63	C1orf71	-11
chr1	228353319	228353598	1.46	9.54E-02	C1orf69	30		
chr17	79818719	79819012	1.35	9.54E-02	P4HB	-322		
chr1	4404109	4404419	0.71	9.54E-02	AJAP1	-310841	DFFB	630419
chr17	75461689	75462106	0.71	9.56E-02	TNRC6C	-538420	SEPT9	184406
chr6	30650322	30650643	0.68	9.56E-02	DHX16	-9653	KIAA1949	4610
chr12	110905938	110906455	1.53	9.59E-02	GPN3	-133		
chr19	42747231	42747528	1.48	9.60E-02	GSK3A	-644		
chr2	60581016	60581387	1.57	9.63E-02	BCL11A	199431		
chr7	20256944	20257212	0.63	9.64E-02	MACC1	-65		
chr16	2802193	2802437	1.56	9.65E-02	SRRM2	-15		
chr19	10330391	10330698	0.67	9.65E-02	DNMT1	-24790	S1PR2	11403
chr13	31017999	31018312	0.64	9.66E-02	KATNAL1	-136993	HMGB1	21925
chr17	62103174	62103418	0.68	9.73E-02	ICAM2	-5302	ERN1	104206
chr2	32502681	32503140	1.57	9.74E-02	YIPF4	-47		
chr21	36237925	36238538	1.50	9.74E-02	C21orf96	173491	CLIC6	196544
chr4	8194484	8194827	1.45	9.74E-02	ABLIM2	-34097	SH3TC1	-6404
chr1	45275198	45275527	1.55	9.74E-02	BTBD19	1209	PTCH2	33253
chr6	27636286	27636530	1.86	9.74E-02	ZNF184	-195511	HIST1H2BL	139301
chr1	155210962	155211227	1.53	9.76E-02	GBA	-42		
chr6	26055994	26056247	0.77	9.76E-02	HIST1H1C	578		
chr5	81574148	81574458	0.74	9.77E-02	RPS23	-68		
chr11	65275015	65275301	1.62	9.81E-02	SCYL1	-17390	FRMD8	121117
chr18	2972197	2972457	1.60	9.82E-02	LOC727896	-25706	LPIN2	39618
chr1	1590403	1590645	1.25	9.83E-02	MMP23B	22964	LOC728661	33719
chr15	83513510	83513867	0.62	9.83E-02	WHAMM	35716	HOMER2	107784
chr20	25677404	25677666	1.45	9.83E-02	ZNF337	-66		
chr3	45730195	45730690	1.58	9.83E-02	SACM1L	-311		
chr3	169531554	169531896	1.57	9.83E-02	LRRC34	-1413		
chr13	26760229	26760537	1.53	9.83E-02	SHISA2	-135185	RNF6	35595

chr1	145208845	145209212	1.47	9.83E-02	NOTCH2NL	-82		
chr4	177116547	177116900	1.34	9.84E-02	SPATA4	98		
chr1	161155080	161155375	0.74	9.84E-02	B4GALT3	-7914	ADAMTS4	13617
chr5	56471451	56471716	2.11	9.85E-02	MIER3	-223630	GPBP1	-38317
chr9	130981050	130981297	1.25	9.88E-02	DNM1	15511	GOLGA2	57094
chr4	74454957	74455352	1.52	9.90E-02	RASSF6	31185	AFM	107693
chr20	49547800	49548227	0.67	9.92E-02	ADNP	-487		
chr12	14436384	14436725	1.76	9.92E-02	GRIN2B	-303533	ATF7IP	-82056
chr3	67048400	67048665	1.44	9.97E-02	KBTBD8	-194		
chr17	80407120	80407707	1.78	9.97E-02	C17orf101	-30952	C17orf62	1280
chr17	57439749	57440025	1.46	9.97E-02	DHX40	-202999	YPEL2	30834
chr10	98478067	98478399	0.50	9.99E-02	TM9SF3	-131424	PIK3AP1	2046
chr3	185216519	185217017	1.46	1.00E-01	TMEM41A	77		
chr19	45640535	45640782	1.53	1.00E-01	NKPD1	22749	GEMIN7	58141
chrX	153626429	153626895	1.45	1.00E-01	RPL10	91		
chr1	42800905	42801366	0.66	1.00E-01	FOXJ3	-233		
chr13	28051436	28051860	1.48	1.00E-01	MTIF3	-26937	LNX2	143072
chr5	180600566	180600842	1.52	1.00E-01	OR2V2	18761	TRIM7	31473
chr16	11836694	11837151	1.31	1.00E-01	TXNDC11	-275		
chr11	118122663	118123183	1.65	1.01E-01	MPZL3	88		
chr15	68502196	68502506	0.62	1.01E-01	CALML4	-3903		
chr17	74268154	74268669	1.65	1.01E-01	RNF157	-32022	QRICH2	35349
chr2	111814735	111815045	0.75	1.01E-01	BCL2L11	-63601	ACOXL	324740
chr7	72722729	72723066	0.62	1.01E-01	NSUN5	-75		
chr1	28832145	28832665	1.65	1.01E-01	RCC1	-12340	PHACTR4	136312
chr11	118888793	118889435	1.55	1.01E-01	TRAPPC4	-127	RPS25	-57
chr16	84627730	84628222	1.68	1.01E-01	KIAA1609	-89688	COTL1	23693
chr20	20032993	20033416	1.31	1.01E-01	CRNKL1	3485	NAT5	35268
chr7	25902045	25902399	1.32	1.01E-01	NPVF	-634117	NFE2L3	-289625
chr15	51199169	51199517	1.46	1.01E-01	AP4E1	-1603		
chr16	2318022	2318501	1.44	1.01E-01	RNPS1	-465		
chr2	38834920	38835236	1.52	1.02E-01	HNRPLL	-4900		
chr19	12792620	12792886	1.46	1.02E-01	DHPS	-76		
chr5	271451	271709	1.39	1.02E-01	PDCD6	-156		
chr9	35618290	35618612	1.45	1.02E-01	CD72	-27		
chr4	114683730	114684135	1.42	1.02E-01	CAMK2D	-850		
chr19	47852370	47852668	1.33	1.02E-01	DHX34	-27		
chr17	29035235	29035537	1.21	1.02E-01	LRRC37B2	99938	CRLF3	116392

chr3	187810509	187810889	1.27	1.02E-01	BCL6	-347224	LPP	-120022		
chr4	141805636	141805903	1.47	1.02E-01	TBC1D9	-128299	RNF150	248846		
chr7	64940700	64941058	0.60	1.03E-01	VKORC1L1	-397378	ZNF92	102111		
chr1	150185850	150186395	1.44	1.03E-01	ANP32E	22381	PLEKHO1	63953		
chr1	236135118	236135402	1.63	1.03E-01	LYST	-105040	NID1	93221		
chr18	43266812	43267106	0.76	1.03E-01	SLC14A1	-37133	SLC14A2	72193		
chr7	87505670	87505967	0.69	1.03E-01	SLC25A40	-151	DBF4	275		
chr2	106342331	106342685	2.19	1.03E-01	FHL2	-326827	NCK2	-125797		
chr5	95194448	95194724	1.64	1.03E-01	GLRX	-36009	ELL2	103189		
chr8	135724785	135725058	1.52	1.03E-01	ZFAT	359				
chr8	103823087	103823422	0.70	1.03E-01	KLF10	-155272	AZIN1	53142		
chr19	14116970	14117280	0.68	1.03E-01	RFX1	9				
chr2	152628102	152628402	1.48	1.03E-01	NEB	-37263	ARL5A	56757		
chr3	48754571	48754881	0.62	1.03E-01	DKFZp564L0678	194	IP6K2	194		
chr4	76870299	76870568	1.64	1.03E-01	NAAA	-8268	SDAD1	41679		
chr8	126446920	126447196	1.37	1.03E-01	TRIB1	4495				
chrX	12968276	12968556	1.54	1.04E-01	TMSB4X	-24809	TLR8	43658		
chr13	39612048	39612486	0.68	1.04E-01	NHLRC3	-188				
chr2	179387689	179388029	1.56	1.04E-01	PLEKHA3	42660	TTN	284291		
chr7	99942472	99942731	1.43	1.04E-01	PILRB	-13024	PVRIG	125730		
chr16	3124945	3125189	0.61	1.04E-01	IL32	9754	ZSCAN10	17794		
chr3	133185823	133186347	0.70	1.04E-01	CDV3	-106349	BFSP2	67295		
chr21	43430005	43430437	1.62	1.05E-01	ZNF295	275				
chr17	41393077	41393458	1.51	1.05E-01	ARL4D	-83085	TMEM106A	29374		
chr1	42194422	42194790	1.48	1.05E-01	EDN2	-244262	HIVEP3	189890		
chr13	50656111	50656420	1.49	1.05E-01	ST13	-89888	KCNRG	66876		
chr6	10460392	10460682	0.75	1.05E-01	GCNT2	-125456	TFAP2A	-45067		
chr20	44519753	44520107	1.46	1.05E-01	C20orf165	-3692	NEURL2	-29	CTSA	339
chr12	112452386	112452660	1.48	1.05E-01	TMEM116	-1588	ERP29	1371		
chr19	39348076	39348392	1.51	1.05E-01	HNRNPL	-7617	RINL	20660		
chr19	13001634	13001986	0.71	1.06E-01	KLF1	-3793	GCDH	-164		
chr16	1470660	1471175	1.49	1.06E-01	UNKL	-6218	C16orf91	8427		
chr6	31036663	31036921	0.56	1.06E-01	C6orf15	43540	MUC21	85307		
chr11	6256266	6256577	0.61	1.06E-01	CNGA4	-3908	FAM160A2	-481		
chr11	65278270	65278514	0.70	1.06E-01	SCYL1	-14156	FRMD8	124351		
chr4	76912025	76912348	1.38	1.06E-01	SDAD1	-74				
chr12	57636516	57636936	0.73	1.06E-01	STAC3	8250	SHMT2	13174		
chr14	58666532	58666868	0.59	1.06E-01	ACTR10	-133				

chr6	79577163	79577778	0.74	1.06E-01	IRAK1BP1	282		
chr11	111234790	111235029	0.97	1.06E-01	C11orf92	-64371	POU2AF1	15247
chr11	67253323	67253633	0.69	1.07E-01	AIP	2973	PITPNM1	19365
chr7	1063942	1064245	0.61	1.07E-01	GPR146	-33047	CYP2W1	41259
chr1	243418592	243418919	1.60	1.07E-01	SDCCAG8	-564	CEP170	-48
chr13	77903232	77903624	1.58	1.07E-01	MYCBP2	-2251		
chr7	99818881	99819225	0.65	1.07E-01	PILRB	-136573	PVRIG	2181
chr12	12966096	12966470	1.44	1.07E-01	DDX47	142		
chr11	108535416	108535885	1.38	1.07E-01	DDX10	-165		
chr2	100867151	100867486	1.33	1.07E-01	AFF3	-145274	LONRF2	71876
chr1	156308143	156308568	1.53	1.07E-01	CCT3	-161		
chr1	198650948	198651272	1.69	1.07E-01	PTPRC	42973		
chr1	203274217	203274497	1.47	1.08E-01	BTG2	-307		
chr9	4792488	4792853	0.60	1.08E-01	RCL1	-163		
chr17	27065685	27066034	1.53	1.08E-01	TRAF4	-5163	NEK8	10028
chr16	27415829	27416139	0.71	1.08E-01	IL21R	-22595	IL4R	90733
chr11	76155808	76156188	1.50	1.09E-01	C11orf30	-71		
chr12	109027450	109027797	1.48	1.09E-01	SELPLG	46		
chr15	65133537	65134006	1.36	1.09E-01	ANKDD1A	-70329	PIF1	-15934
chr9	127420509	127420838	1.33	1.09E-01	NR5A1	-150975	NR6A1	112902
chr2	65610998	65611246	0.80	1.09E-01	SPRED2	48534	ACTR2	156293
chr7	139616290	139616581	1.29	1.09E-01	TBXAS1	87413	PARP12	147085
chr2	70313444	70313754	0.69	1.09E-01	PCBP1	-986		
chr7	99686388	99686664	0.71	1.09E-01	COPS6	-57		
chr3	187956872	187957257	0.69	1.09E-01	TPRG1	-932698	LPP	26344
chr12	30848656	30849088	1.38	1.09E-01	IPO8	-121		
chr14	106328224	106328600	1.58	1.09E-01	IGHE	1050	TMEM121	335459
chr15	40397383	40397627	1.49	1.10E-01	SRP14	-66116	BMF	3570
chr5	177540315	177540693	1.23	1.10E-01	N4BP3	-52		
chr12	96608962	96609337	1.48	1.10E-01	ELK3	20943	PCTK2	185073
chr20	32366829	32367078	0.64	1.10E-01	CHMP4B	-32165	ZNF341	47146
chr4	148787996	148788254	1.71	1.10E-01	ARHGAP10	134672	NR3C2	575518
chr19	13278440	13278765	0.71	1.10E-01	IER2	17321	CACNA1A	338671
chr15	52758355	52758697	1.45	1.11E-01	MYO5C	-170556	MYO5A	62721
chr20	47894975	47895399	1.60	1.11E-01	ZNFX1	-431		
chr1	11866040	11866343	1.42	1.11E-01	MTHFR	-77	CLCN6	-15
chr14	68734895	68735223	1.57	1.11E-01	RAD51L1	448550	ZFP36L1	524726
chr1	61542507	61542994	0.62	1.11E-01	NFIA	-4783		

chr17	56582305	56582668	1.63	1.11E-01	HSF5	-16728	MTMR4	12764
chr8	81209269	81209649	0.65	1.12E-01	TPD52	-216449	ZBTB10	-188989
chr11	2407130	2407395	0.60	1.12E-01	CD81	8716	TRPM5	37012
chr8	103543024	103543347	0.62	1.12E-01	UBR5	-118691	ODF1	-20662
chr7	29961099	29961368	0.64	1.12E-01	SCRN1	68671	WIPF3	86893
chr1	155278218	155278768	1.44	1.12E-01	FDPS	-46		
chr1	45265809	45266279	1.29	1.12E-01	PLK3	8		
chr7	100781840	100782086	1.44	1.12E-01	AP1S1	-15723	SERPINE1	11584
chr10	75541594	75541917	0.73	1.12E-01	KIAA0913	-3849	CHCHD1	-52
chr1	183447198	183447442	0.63	1.12E-01	SMG7	5686	NCF2	112726
chr12	32059701	32060107	0.73	1.13E-01	BICD1	-200281	H3F3C	-114729
chr7	44646447	44646918	0.71	1.13E-01	OGDH	512		
chr1	194258684	194259087	1.61	1.13E-01				
chr16	2301542	2301787	1.43	1.13E-01	DCI	-62		
chr19	40023409	40023725	1.35	1.14E-01	EID2B	-73		
chr6	31830570	31830894	0.65	1.14E-01	NEU1	-23		
chr17	73660061	73660433	1.61	1.14E-01	SAP30BP	-3152	RECQL5	3022
chr4	57843674	57843931	1.59	1.14E-01	POLR2B	-1306	C4orf14	23
chr11	128341074	128341540	1.49	1.14E-01	ETS1	116146		
chr16	56985321	56985747	1.45	1.14E-01	CETP	-10301	HERPUD1	19786
chr17	56407559	56407901	1.48	1.14E-01	BZRAP1	-1578		
chr11	67275370	67275882	0.67	1.14E-01	PITPNM1	-2783	CDK2AP2	476
chr12	57632913	57633177	0.68	1.14E-01	SHMT2	9493	STAC3	11931
chr3	121721943	121722368	1.52	1.14E-01	ILDR1	18874	SLC15A2	108985
chr14	102771417	102771661	1.41	1.14E-01	RAGE	-8		
chr10	105881687	105882184	0.71	1.14E-01	GSTO1	-132742	COL17A1	-36298
chr5	99870848	99871133	1.30	1.14E-01	FAM174A	-133		
chr6	32982600	32982844	0.76	1.15E-01	HLA-DOA	-5333	HLA-DPA1	58827
chr12	1193089	1193325	0.71	1.16E-01	ERC1	92803	FBXL14	510124
chr5	96270733	96271159	1.38	1.16E-01	LNPEP	-23210	ERAP2	59302
chr2	190305661	190305952	1.43	1.16E-01	WDR75	-352		
chr7	43622294	43622604	0.70	1.16E-01	STK17A	-243		
chr6	36630138	36630448	0.63	1.16E-01	CDKN1A	-16166	SFRS3	68203
chr15	85293791	85294175	0.82	1.17E-01	ALPK3	-65928	ZNF592	2165
chr19	10541861	10542180	1.34	1.17E-01	PDE4A	10688	KEAP1	72033
chr16	734250	734596	1.42	1.17E-01	STUB1	4308	FBXL16	21385
chr9	95432383	95432789	1.39	1.17E-01	IPPK	-39		
chr2	240323017	240323484	1.32	1.17E-01	HDAC4	-608		

chr15	85197274	85197606	0.69	1.18E-01	NMB	4362	SCAND2	22749
chr9	130217783	130218215	0.85	1.18E-01	RPL12	-4315	LRSAM1	4207
chr2	157192103	157192361	1.58	1.18E-01	NR4A2	-2945		
chr20	18447789	18448133	0.75	1.18E-01	C20orf12	-132	POLR3F	-72
chr1	200589927	200590203	1.40	1.18E-01	KIF14	-203		
chr18	42683363	42683619	1.51	1.19E-01	SLC14A2	-511275	SETBP1	422628
chr22	28315142	28315508	1.36	1.19E-01	PITPNB	-70		
chr7	54826821	54827125	1.52	1.19E-01	SEC61G	-34		
chr7	35767311	35767566	0.65	1.19E-01	SEPT7	-73188	HERPUD2	-32667
chr16	30197145	30197468	0.79	1.19E-01	GIYD2	-7910	CORO1A	2381
chr12	109531222	109531544	1.50	1.19E-01	UNG	-4032	ALKBH2	-90
chr4	140216827	140217137	1.52	1.19E-01	NDUFC1	132		
chr13	47371203	47371629	1.45	1.19E-01	ESD	-49		
chr7	99613006	99613316	0.70	1.19E-01	ZKSCAN1	-58		
chr6	31527718	31528067	0.70	1.19E-01	LTA	-11983	NFKBIL1	12540
chr7	45040763	45041358	0.61	1.19E-01	CCM2	1274	NACAD	87432
chr7	128084422	128084751	1.31	1.20E-01	IMPDH1	-34551	C7orf68	-11297
chr9	130890472	130890782	0.77	1.20E-01	LOC389791	-181	PTGES2	-153
chr7	50346905	50347215	0.72	1.20E-01	IKZF1	2682	FIGNL1	171028
chr5	150635484	150635805	0.68	1.20E-01	GM2A	3032	SLC36A3	47689
chr19	18811278	18811539	1.41	1.20E-01	CRTC1	16984	COMP	90705
chr10	14690220	14690526	0.70	1.20E-01	FRMD4A	-317507	CDNF	189610
chr10	97517674	97518013	1.39	1.20E-01	CCNJ	-285315	ENTPD1	46308
chr12	68151976	68152234	0.67	1.20E-01	DYRK2	109593	IFNG	401416
chr15	91593067	91593334	1.91	1.20E-01	SV2B	-175902	VPS33B	-27368
chr7	23145127	23145720	0.58	1.20E-01	KLHL7	71		
chr8	119291731	119292025	1.51	1.20E-01	EXT1	-167820	TNFRSF11B	672505
chr2	170655247	170655557	0.84	1.21E-01	SSB	13		
chr11	102187840	102188506	1.70	1.21E-01	BIRC3	-21		
chr5	81045434	81045744	0.71	1.21E-01	ACOT12	-355601	SSBP2	1483
chr14	56662463	56662749	1.48	1.21E-01	C14orf101	-383905	PELI2	77513
chr19	49217597	49217884	1.28	1.21E-01	MAMSTR	5235	FUT2	18513
chr2	113931262	113931703	0.65	1.21E-01	PSD4	-77		
chr14	102305582	102305858	1.60	1.22E-01	DYNC1H1	-125145	PPP2R5C	77585
chr4	140004027	140004286	1.52	1.22E-01	ELF2	56494	CCRN4L	67214
chr3	13152143	13152425	1.37	1.22E-01	IQSEC1	-37667	NUP210	309525
chr4	128886280	128886524	1.37	1.22E-01	C4orf29	-59	MFSD8	737
chr22	24236370	24236729	1.51	1.22E-01	MIF	-15		

chr17	65809358	65809657	0.67	1.22E-01	BPTF	-12272	NOL11	95447
chr8	47868637	47868946	0.60	1.22E-01	CEBPD	781934		
chr16	29674861	29675171	0.61	1.23E-01	SPN	716		
chr7	12250744	12250987	0.67	1.23E-01	TMEM106B	18		
chr12	15942299	15942575	1.26	1.23E-01	EPS8	73		
chr17	7210725	7210987	1.42	1.23E-01	EIF5A	538		
chr3	113775209	113775797	1.52	1.23E-01	QTRTD1	-108		
chr11	65430262	65430836	1.51	1.23E-01	RELA	-106		
chr2	192540508	192540881	1.50	1.23E-01	OBFC2A	-2166		
chr3	169482818	169483179	1.56	1.24E-01	MECOM	-101436	ARPM1	4684
chr9	140149593	140149857	1.41	1.24E-01	COBRA1	-34		
chr3	10157196	10157440	1.32	1.24E-01	C3orf10	-15		
chr8	28195982	28196278	1.54	1.24E-01	PNOC	21481	ZNF395	47847
chr7	75272270	75272529	0.66	1.24E-01	PMS2L3	-114947	HIP1	95879
chr1	167468994	167469274	1.45	1.24E-01	CD247	18713	POU2F1	278991
chr2	128577670	128577982	0.58	1.24E-01	WDR33	-9081	POLR2D	37903
chr1	37349812	37350122	0.63	1.25E-01	CSF3R	-401458	GRIK3	149877
chr4	140187915	140188194	1.47	1.25E-01	ELF2	-127404	C4orf49	13437
chr19	12251896	12252243	0.65	1.25E-01	ZNF20	-930		
chr19	59066420	59066673	1.26	1.25E-01	CHMP2A	-61		
chr19	56110931	56111497	1.37	1.25E-01	ZNF524	-516	FIZ1	-321
chr6	11144641	11144895	0.71	1.25E-01	HERV-FRD	-32809	NEDD9	88147
chr15	69744736	69745203	0.77	1.25E-01	RPLP1	-189		
chr19	36422441	36422859	1.48	1.25E-01	TYROBP	-23464	LRFN3	-5372
chr1	9488888	9489180	1.35	1.26E-01	SLC25A33	-110494	SPSB1	136093
chr19	42636342	42636787	0.73	1.26E-01	POU2F2	65		
chr19	5680536	5680793	1.36	1.26E-01	HSD11B1L	-370	C19orf70	246
chr11	65990162	65990472	1.47	1.26E-01	KLC2	-34857	PACS1	152493
chr22	39898179	39898468	1.54	1.26E-01	SMCR7L	40		
chr19	54694082	54694426	1.25	1.26E-01	TSEN34	-850	MBOAT7	-521
chr19	8258824	8259134	0.83	1.26E-01	FBN3	-46598	LASS4	-15238
chr6	31164756	31165066	0.72	1.26E-01	POU5F1	-26460	HLA-C	74944
chr15	40390971	40391336	1.54	1.26E-01	SRP14	-59765	BMF	9921
chr6	33560293	33560615	0.72	1.26E-01	ITPR3	-28707	BAK1	-12384
chr6	57181504	57181755	0.73	1.27E-01	PRIM2	-792		
chr7	1126159	1126524	0.70	1.27E-01	GPER	-1381		
chr3	44803030	44803307	1.47	1.27E-01	KIF15	-40		
chr11	118271756	118272293	0.76	1.27E-01	ATP5L	-79		

chr5	96152120	96152430	0.88	1.28E-01	ERAP2	-59369	ERAP1	-8383		
chr4	78783698	78784164	1.50	1.28E-01	MRPL1	126				
chr5	139646397	139646857	1.41	1.28E-01	PFDN1	36062	C5orf53	141106		
chr6	474850	475110	1.52	1.29E-01	IRF4	83228	EXOC2	218129		
chr1	185126007	185126459	1.42	1.29E-01	C1orf25	-117				
chr19	55770540	55770893	1.43	1.29E-01	SAPS1	-679				
chr7	1550462	1550702	1.31	1.29E-01	MAFK	-19786	INTS1	-6564		
chr4	300359	300740	1.50	1.29E-01	ZNF141	-31046	ZNF732	-10606		
chr7	99717133	99717851	0.58	1.29E-01	TAF6	-513	CNPY4	227		
chr10	80828486	80828862	1.08	1.29E-01	ZMIZ1	-118				
chr19	47729463	47729952	1.33	1.29E-01	BBC3	6315	SAE1	95628		
chr6	27860729	27861156	1.42	1.30E-01	HIST1H3J	-2373	HIST1H2BO	-260	HIST1H2AM	20
chr20	44509699	44509976	1.49	1.30E-01	ZSWIM1	-10				
chr1	32782149	32782395	0.58	1.30E-01	MARCKSL1	19562	HDAC1	24564		
chr11	62358960	62359344	1.64	1.30E-01	TUT1	-43				
chr7	44121893	44122146	0.66	1.30E-01	POLM	109				
chr9	100684420	100684692	1.51	1.30E-01	C9orf156	296				
chr2	45878091	45878401	1.36	1.30E-01	PRKCE	-797				
chr3	194992100	194992602	0.63	1.30E-01	C3orf21	-456				
chr7	5553201	5553600	0.65	1.30E-01	FBXL18	-2				
chr12	56040006	56040260	1.36	1.30E-01	METTL7B	-35197	OR10P1	9457		
chr19	47759637	47759949	1.36	1.30E-01	BBC3	-23770	PRR24	-18349		
chr11	63334613	63335056	1.49	1.30E-01	RARRES3	30562	PLA2G16	47106		
chr7	128502682	128502973	1.48	1.31E-01	ATP6V1F	-70				
chr6	4018797	4019419	0.68	1.31E-01	PRPF4B	-2461				
chr11	59318419	59318858	1.46	1.32E-01	OR4D9	36253	OSBP	64978		
chr19	46405859	46406259	1.28	1.32E-01	MYPOP	-197				
chr5	180615830	180616084	1.51	1.32E-01	TRIM7	16220	OR2V2	34014		
chr1	153918484	153918794	1.50	1.32E-01	GATAD2B	-23188	CRTC2	12404		
chr19	17887362	17887637	1.44	1.32E-01	B3GNT3	-18419	MAP1S	57197		
chr6	26595997	26596307	0.71	1.33E-01	ABT1	-1028				
chr19	49137687	49137940	1.29	1.33E-01	Sec1	-3482	DBP	2825		
chr19	46234062	46234354	1.28	1.33E-01	FBXO46	-57				
chr6	42694312	42694622	0.74	1.33E-01	PRPH2	-4109				
chr5	61708433	61708681	1.40	1.33E-01	DIMT1L	-8829	IPO11	-6154		
chr2	68942555	68942847	1.50	1.33E-01	ARHGAP25	-19267	PROKR1	69907		
chr16	31471141	31471411	1.33	1.34E-01	ARMC5	959				
chr13	45694446	45694715	1.46	1.34E-01	GTF2F2	-50				

chr8	102217932	102218233	0.55	1.34E-01	ZNF706	-123			
chr3	176915594	176916077	1.44	1.35E-01	TBL1XR1	-788			
chr11	62420703	62421013	1.69	1.35E-01	INTS5	-84			
chr4	54614528	54614941	1.39	1.35E-01	LNX1	-157011	CHIC2	316053	
chr11	102158764	102159011	1.91	1.35E-01	BIRC3	-29306	YAP1	177678	
chr15	101739882	101740241	1.67	1.35E-01	CHSY1	52064	LRRK1	280602	
chr8	118452006	118452451	0.63	1.35E-01	MED30	-80736	SLC30A8	304892	
chr14	65879100	65879508	0.71	1.35E-01	FUT8	-231			
chr17	17942380	17942757	1.32	1.35E-01	ATPAF2	-89			
chr6	24774881	24775377	0.65	1.35E-01	GMNN	-35			
chrX	153990784	153991110	1.35	1.35E-01	DKC1	-84			
chr12	125399038	125399664	1.43	1.35E-01	UBC	226			
chr21	43954557	43954954	0.75	1.36E-01	PDE9A	-119106	SLC37A1	35014	
chr9	124921853	124922211	0.78	1.36E-01	NDUFA8	66			
chr1	218458403	218458748	1.45	1.36E-01	RRP15	-53			
chr19	1241421	1241831	1.43	1.36E-01	C19orf26	-3636	ATP5D	-123	
chr9	136283052	136283441	1.38	1.36E-01	ADAMTS13	-3873	REXO4	-83	
chr2	46796345	46796764	1.44	1.36E-01	RHOQ	26688	PIGF	47696	
chr7	23412860	23413666	0.56	1.37E-01	IGF2BP3	96732	GPNMB	126947	
chr9	102582040	102582395	1.17	1.37E-01	NR4A3	-6791	SEC61B	597648	
chr6	14095697	14095968	1.48	1.38E-01	CD83	-22032	RNF182	170630	
chr6	30646774	30647051	0.63	1.38E-01	DHX16	-6083	KIAA1949	8180	
chr11	65029128	65029446	1.48	1.38E-01	POLA2	-145			
chr12	114404035	114404325	1.38	1.38E-01	RBM19	-4			
chr2	73460346	73460596	1.18	1.38E-01	CCT7	-934	C2orf7	-115	
chr9	114798094	114798387	1.38	1.38E-01	UGCG	139035	SUSD1	139315	
chr7	130792751	130793010	1.28	1.39E-01	KLF14	-374021	MKLN1	-219714	
chr20	62259697	62259976	1.52	1.39E-01	GMEB2	-1456			
chr6	27834338	27834738	0.71	1.39E-01	HIST1H1B	821			
chr20	39640239	39640697	1.55	1.39E-01	MAFB	-322592	TOP1	-16994	
chr3	32509299	32509599	1.37	1.40E-01	CMTM6	34954	CMTM7	76286	
chr6	33281638	33281993	0.77	1.40E-01	TAPBP	173			
chr13	46756134	46756453	1.54	1.40E-01	LCP1	165			
chr20	23338631	23338892	1.30	1.40E-01	GZF1	-6238	NXT1	7389	
chr9	139010439	139010949	1.35	1.40E-01	NACC2	-23563	LHX3	84310	
chr7	152133751	152134027	1.18	1.41E-01	MLL3	-799			
chr4	787934	788370	0.60	1.41E-01	CPLX1	31793	PCGF3	88579	
chr21	45626630	45626991	0.79	1.41E-01	ICOSLG	34023	C21orf33	73317	

chr2	232092968	232093296	0.69	1.41E-01	B3GNT7	-167203	ARMC9	29790
chr1	145058991	145059284	1.50	1.41E-01	PDE4DIP	-64116	SEC22B	-37269
chr7	64023286	64023606	0.66	1.41E-01	ZNF680	59		
chr17	38738524	38738849	0.67	1.41E-01	CCR7	-16963	SMARCE1	65416
chr2	6985514	6985824	0.73	1.41E-01	CMPK2	20267		
chr3	5163729	5164085	1.33	1.41E-01	ARL8B	-23		
chr6	159230018	159230310	0.65	1.42E-01	EZR	9176	SYTL3	159118
chr11	1873998	1874414	0.68	1.42E-01	LSP1	6		
chr12	48206958	48207348	1.28	1.42E-01	HDAC7	6610	SLC48A1	40186
chr19	55787525	55788060	1.37	1.42E-01	SAPS1	-17755	HSPBP1	3958
chr6	130005163	130005469	1.65	1.42E-01	ARHGAP18	26054	LAMA2	801030
chr8	49862139	49862446	0.75	1.42E-01	SNTG1	-962304	SNAI2	-28305
chr6	41301749	41302042	0.77	1.42E-01	NCR2	-1632		
chr1	228674589	228674899	0.66	1.42E-01	RHOU	-196125	HIST3H2BB	28936
chr11	111712221	111712497	1.33	1.42E-01	PPP2R1B	-75190	ALG9	29946
chr1	19536730	19537204	1.36	1.42E-01	UBR4	-221		
chr15	70489048	70489294	1.40	1.43E-01	TLE3	-98915	UACA	566679
chr2	61371961	61372299	1.23	1.43E-01	PUS10	-126765	C2orf74	-17499
chr1	16162372	16162657	1.37	1.43E-01	UQCRHL	-28321	SPEN	-11844
chr3	14692961	14693435	1.59	1.43E-01	GRIP2	-109610	C3orf20	-23456
chr1	153895586	153895869	1.42	1.43E-01	GATAD2B	-277		
chr7	912968	913278	0.70	1.43E-01	C7orf20	-3068		
chr20	48732413	48732701	1.61	1.43E-01	TMEM189-UBE2V1	37778	SNAI1	133030
chr3	128399544	128399801	1.53	1.43E-01	RAB7A	-45306	RPN1	-29954
chr19	46366392	46366717	1.32	1.43E-01	FOXA3	-963	SYMPK	-7
chr7	23312739	23312989	0.71	1.44E-01	GPNMB	26548	IGF2BP3	197131
chr3	119182339	119182637	1.45	1.44E-01	TMEM39A	-17		
chr11	9779625	9779921	0.75	1.44E-01	SWAP70	94145	SBF2	535981
chr3	169489829	169490180	1.43	1.44E-01	ARPM1	-2322	MYNN	-848
chr7	48019050	48019375	0.72	1.44E-01	HUS1	33		
chr14	75716227	75716488	0.74	1.45E-01	TMED10	-73009	FOS	-29123
chr1	1550642	1550886	1.20	1.45E-01	MIB2	-120		
chr8	41347794	41348166	0.62	1.45E-01	GOLGA7	-101		
chr3	124714309	124714562	1.62	1.45E-01	MUC13	-60856	HEG1	60366
chr6	53583718	53584002	1.67	1.45E-01	LRRC1	-75674	KLHL31	-53354
chr17	29865156	29865467	0.73	1.46E-01	RAB11FIP4	146670	C17orf79	321014
chr5	43043242	43043583	0.59	1.46E-01	C5orf39	-2966		
chr8	95731899	95732205	0.77	1.46E-01	DPY19L4	-51		

chr1	25075130	25075586	1.55	1.46E-01	CLIC4	3598	RUNX3	181412				
chr19	36236312	36236623	1.43	1.46E-01	LIN37	-3044	TMEM149	-2948	U2AF1L4	-132	PSENEN	-26
chr11	108338115	108338373	1.28	1.46E-01	KDELC2	30915	ATM	244685				
chr7	5013421	5013893	0.74	1.46E-01	RBAK	-71896	MMD2	-14813				
chr1	226315190	226315541	1.47	1.47E-01	ACBD3	59057	H3F3B	64945				
chr8	38662850	38663249	0.71	1.47E-01	PLEKHA2	-95703	TACC1	18328				
chr4	79697002	79697249	1.32	1.47E-01	BMP2K	-406						
chr1	20142272	20142661	1.45	1.47E-01	RNF186	-696						
chr5	140079756	140080029	1.50	1.47E-01	ZMAT2	-139						
chr7	129845117	129845399	1.29	1.47E-01	C7orf45	-2446	TMEM209	80				
chr13	28024062	28024524	1.44	1.47E-01	MTIF3	418						
chr19	1021329	1021837	1.63	1.47E-01	CNN2	-4715	C19orf6	-442				
chr6	131520886	131521214	0.66	1.47E-01	ARG1	-373315	AKAP7	54625				
chr1	206808538	206808926	1.55	1.48E-01	DYRK3	-149						
chr7	106193258	106193539	1.33	1.48E-01	PIK3CG	-312525	NAMPT	-267761				
chr13	27825315	27825691	0.74	1.48E-01	RPL21	-189						
chr18	18691635	18691968	0.64	1.48E-01	ROCK1	10						
chr9	134553904	134554148	1.49	1.48E-01	UCK1	-147364	RAPGEF1	31203				
chr1	185286530	185286884	1.37	1.48E-01	IVNS1ABP	-246						
chr6	42713585	42713923	0.67	1.48E-01	TBCC	130						
chr2	136964871	136965336	1.30	1.48E-01	THSD7B	-783358	CXCR4	-89379				
chr10	121356518	121356763	0.59	1.48E-01	TIAL1	-100						
chr7	25219804	25220062	0.60	1.48E-01	CYCS	-54978	NPVF	48172				
chr19	1103672	1103945	1.28	1.48E-01	GPX4	-127						
chr22	43010678	43011043	1.26	1.48E-01	POLDIP3	101						
chr2	30369117	30369744	1.31	1.48E-01	ALK	-224999	LBH	-84966				
chr2	37899292	37899532	1.41	1.48E-01	CDC42EP3	-86						
chr9	132032494	132032787	1.55	1.48E-01	METTL11A	-355794	PPP2R4	159397				
chr3	150583164	150583437	1.53	1.49E-01	SIAH2	-102038	CLRN1	107485				
chr7	140623932	140624175	1.39	1.49E-01	BRAF	510						
chr19	49457828	49458188	1.42	1.49E-01	BAX	-109						
chr11	74660059	74660390	1.36	1.49E-01	SPCS2	-67	XRRA1	7				
chr20	25262293	25262569	1.40	1.49E-01	PYGB	33725	ABHD12	109046				
chr1	155915996	155916336	1.66	1.50E-01	RXFP4	4686	ARHGEF2	32170				
chr14	91782283	91782527	1.53	1.50E-01	GPR68	-62181	CCDC88C	101728				
chr10	69609073	69609360	1.58	1.51E-01	SIRT1	-35210	DNAJC12	-11280				
chr19	39971266	39971542	1.37	1.51E-01	TIMM50	352						
chr6	91085244	91085620	0.60	1.51E-01	BACH2	-78870	MAP3K7	211475				

chr1	26868691	26869047	1.29	1.52E-01	RPS6KA1	-3474		
chr4	121670691	121671015	1.23	1.52E-01	MAD2L1	-682840	PRDM5	173160
chr1	93441146	93441501	1.51	1.52E-01	MTF2	-103468	FAM69A	-14245
chr19	45981715	45982236	0.69	1.52E-01	FOSB	10723	RTN2	18337
chr1	70876592	70876921	0.72	1.53E-01	CTH	-198		
chr9	140356355	140356614	1.62	1.53E-01	NELF	-2699		
chr15	75315729	75316099	1.34	1.53E-01	PPCDC	-13		
chr17	77752639	77752953	1.29	1.53E-01	CBX2	803		
chr15	64445641	64446037	0.70	1.53E-01	SNX22	1923	PPIB	9515
chr7	128649522	128649778	1.45	1.53E-01	TNPO3	45548	IRF5	71656
chr18	42792733	42793012	1.54	1.53E-01	SLC14A2	-401893	SETBP1	532010
chr4	2263952	2264375	0.55	1.53E-01	MXD4	-425		
chr20	43607963	43608214	1.45	1.53E-01	STK4	12969	KCNS1	121664
chr17	76836922	76837204	1.32	1.53E-01	USP36	-94		
chr9	77703775	77704140	0.64	1.53E-01	C9orf95	-825	OSTF1	560
chr1	70820373	70820649	1.47	1.54E-01	ANKRD13C	-94	HHLA3	18
chr15	52548612	52548917	1.50	1.54E-01	GNB5	-65200	MYO5C	39205
chr2	231466258	231466598	1.64	1.54E-01	CAB39	-111129	SP100	185557
chr8	103875152	103875581	0.74	1.54E-01	KLF10	-207384	AZIN1	1030
chr3	128598201	128598556	1.40	1.54E-01	ACAD9	46		
chr4	164509024	164509342	1.54	1.54E-01	TKTL2	-114136	MARCH1	25593
chr19	5790445	5790946	1.57	1.54E-01	DUS3L	553		
chr22	37641484	37641867	1.53	1.54E-01	RAC2	-1371		
chr13	100027658	100028087	1.55	1.54E-01	TM9SF2	-125855	GPR183	-68124
chr3	177075679	177075972	1.57	1.54E-01	TBL1XR1	-160778		
chr2	69066414	69066727	0.67	1.55E-01	BMP10	32078	ARHGAP25	104603
chr11	93517204	93517537	1.36	1.55E-01	MED17	-34		
chr4	71553918	71554339	1.44	1.55E-01	UTP3	-67		
chr15	59279712	59279960	1.46	1.55E-01	RNF111	-29		
chr19	16683181	16683512	1.64	1.56E-01	SLC35E1	-154		
chrX	9431124	9431535	1.37	1.56E-01	TBL1X	-1871		
chr11	134094365	134094601	1.42	1.56E-01	VPS26B	-78	NCAPD3	-57
chr8	54983541	54983837	0.69	1.56E-01	TCEA1	-48681	LYPLA1	30888
chr3	187934896	187935310	0.70	1.56E-01	TPRG1	-954660	LPP	4382
chr3	40498616	40498878	1.43	1.56E-01	RPL14	-54		
chr12	113634799	113635073	0.62	1.56E-01	TPCN1	-24324	DDX54	-11652
chr4	26809050	26809297	0.68	1.57E-01	STIM2	-53190	TBC1D19	223628
chr3	177043048	177043317	1.58	1.57E-01	TBL1XR1	-128135		

chr17	38256911	38257155	1.33	1.57E-01	NR1D1	-60		
chr12	31559158	31559468	0.87	1.57E-01	OVOS1	-200225	DENND5B	184639
chr8	75262543	75262918	0.67	1.57E-01	GDAP1	113		
chr17	17380136	17380406	1.28	1.57E-01	MED9	-29		
chr20	43991550	43992154	0.68	1.57E-01	SYS1	151		
chr3	128968142	128968510	1.57	1.57E-01	COPG	-127		
chr6	41691248	41691558	0.72	1.57E-01	TFEB	61		
chr3	169663118	169663470	1.43	1.57E-01	LRRC31	-75634	SEC62	-21286
chr7	81475747	81476058	0.69	1.57E-01	HGF	-76451	CACNA2D1	597128
chr9	137029898	137030237	1.40	1.57E-01	RXRA	-188248	WDR5	28858
chr17	7338442	7338732	1.24	1.58E-01	FGF11	-4102	TMEM102	-175
chr11	64546263	64546709	1.43	1.58E-01	SF1	-245		
chr11	125757401	125757661	1.44	1.58E-01	HYLS1	4022	PUS3	15585
chr20	52539472	52539798	1.64	1.58E-01	ZNF217	-339928	BCAS1	147669
chr21	30364821	30365175	0.76	1.58E-01	RNF160	279		
chr17	74259145	74259393	0.57	1.58E-01	RNF157	-22879	QRICH2	44492
chr3	45637790	45638227	1.31	1.58E-01	SACM1L	-92745	LIMD1	1686
chr8	101965485	101965863	0.66	1.58E-01	YWHAZ	-453		
chr12	262348	262658	0.70	1.58E-01	SLC6A12	60868	IQSEC3	75961
chr9	96328704	96329098	1.19	1.58E-01	PHF2	-10008	FAM120A	114728
chr2	42588333	42588657	0.77	1.59E-01	COX7A2L	493		
chr1	28499163	28499484	0.61	1.59E-01	EYA3	-84193	PTAFR	3867
chr1	145096177	145096498	1.58	1.59E-01	SEC22B	-69		
chr9	37374913	37375249	0.69	1.59E-01	GRHPR	-47626	ZCCHC7	254612
chr11	119978952	119979199	1.44	1.59E-01	PVRL1	-379641	TRIM29	29787
chr9	71394783	71395038	1.31	1.59E-01	PIP5K1B	74295	PRKACG	234128
chr2	122512975	122513285	0.74	1.59E-01	TSN	9		
chr11	46722099	46722548	1.50	1.60E-01	ARHGAP1	-204	ZNF408	-44
chr13	53029366	53029718	0.61	1.60E-01	VPS36	-4779	CKAP2	47
chr1	231473519	231473863	1.44	1.60E-01	EXOC8	-113	C1orf124	9
chr17	19209599	19210106	1.54	1.60E-01	b9	56193	EPN2	69163
chr5	176784694	176784982	1.50	1.60E-01	RGS14	-6		
chr17	37912049	37912682	1.84	1.60E-01	GRB7	18179	IKZF3	108075
chr22	39842397	39842795	0.76	1.60E-01	MGAT3	-40633	MAP3K7IP1	46837
chr3	133209931	133210329	0.69	1.60E-01	CDV3	-82304	BFSP2	91340
chr5	159507898	159508313	0.61	1.60E-01	FABP6	-106268	TTC1	71926
chr3	62804005	62804383	1.51	1.60E-01	FEZF2	-445004	CADPS	56870
chr9	116172424	116172867	1.55	1.61E-01	POLE3	383		

chr9	94877500	94877922	1.41	1.62E-01	SPTLC1	-21		
chr5	133436216	133436472	1.36	1.62E-01	VDAC1	-95911	TCF7	-15256
chr6	56224114	56224390	1.44	1.62E-01	COL21A1	-111874	DST	595161
chr3	14732832	14733142	0.74	1.62E-01	FGD5	-127482	C3orf20	16333
chr10	37177967	37178214	1.29	1.62E-01	ANKRD30A	-236694		
chr10	74020521	74020802	0.65	1.62E-01	DDIT4	-13015	C10orf104	44856
chr16	89355883	89356123	1.20	1.62E-01	ZNF778	71892	ANKRD11	200966
chr19	2631464	2631753	0.64	1.62E-01	GNG7	71137	GADD45B	155474
chr7	99102628	99102938	0.68	1.62E-01	ZNF394	-4906	ZKSCAN5	510
chr18	24229663	24230000	1.50	1.63E-01	KCTD1	-101332	AQP4	215884
chr15	57511405	57511715	1.87	1.63E-01	CGNL1	-157145	TCF12	300727
chr2	70418022	70418339	1.20	1.63E-01	TIA1	57598	PCBP1	103596
chr14	94434025	94434340	1.50	1.64E-01	OTUB2	-58541	ASB2	-10416
chr16	11782978	11783258	1.52	1.64E-01	SNN	20817	TXNDC11	53530
chr3	101405492	101405777	1.55	1.64E-01	RPL24	-72		
chr13	103683811	103684280	0.81	1.64E-01	SLC10A2	35150	ERCC5	224550
chr1	27668308	27668584	1.17	1.64E-01	SYTL1	-67		
chr10	98479547	98479857	0.60	1.65E-01	PIK3AP1	577		
chr7	19748495	19748797	0.63	1.65E-01	TWISTNB	14		
chr19	36822569	36822845	0.77	1.65E-01	ZNF565	-117141	ZFP14	36166
chr1	156024319	156024732	1.45	1.65E-01	UBQLN4	-1010	ROBLD3	9
chr17	7307374	7307954	1.37	1.65E-01	NLGN2	-3838	C17orf61	-246
chr8	56852179	56852459	0.71	1.65E-01	LYN	59933	RPS20	134821
chr6	83924902	83925201	1.63	1.65E-01	PGM3	-22117	ME1	215727
chr9	35072493	35072900	0.70	1.66E-01	VCP	42		
chr7	26404712	26405030	0.66	1.66E-01	SNX10	73356	SKAP2	499470
chr12	133562770	133563236	1.41	1.67E-01	ZNF26	-53		
chr4	113174573	113174883	0.63	1.67E-01	AP1AR	21833	TIFA	32331
chrX	1510463	1510707	1.43	1.67E-01	SLC25A6	413		
chr2	202122954	202123316	1.49	1.67E-01	CASP8	381		
chr3	8398941	8399204	1.23	1.67E-01	LMCD1	-144438		
chr13	47557233	47557664	0.68	1.67E-01	HTR2A	-87080		
chr19	8454759	8455009	1.52	1.68E-01	RAB11B	-321		
chr17	7155308	7155588	1.31	1.68E-01	DULLARD	-453	C17orf81	-103
chr3	15091952	15092199	1.30	1.68E-01	MRPS25	14740	NR2C2	102840
chr3	185655061	185655408	1.37	1.68E-01	TRA2B	689		
chr14	64805490	64805929	0.79	1.69E-01	MTHFD1	-49049	ESR2	-44582
chr9	134955186	134955437	1.36	1.69E-01	MED27	-59		

chr10	102295394	102295760	1.56	1.69E-01	HIF1AN	-64		
chr5	139015286	139015562	1.37	1.69E-01	CXXC5	-12877	UBE2D2	74673
chr10	112174366	112174744	0.77	1.70E-01	SMNDC1	-109848	DUSP5	-83070
chr9	131038311	131038654	1.31	1.70E-01	GOLGA2	-215		
chr1	40071840	40072150	0.74	1.70E-01	PABPC4	-29474	HEYL	33353
chr1	153962969	153963363	0.72	1.70E-01	RAB13	-4360	RPS27	-73
chr12	10766049	10766359	1.28	1.70E-01	MAGOHB	-21		
chr2	232578485	232578815	0.70	1.70E-01	PTMA	5415	PDE6D	67324
chr3	189679289	189679633	1.58	1.70E-01	LEPREL1	159447	TP63	330245
chr6	143206676	143206920	1.63	1.70E-01	HIVEP2	59540	GPR126	583742
chr19	52692598	52693688	1.42	1.70E-01	PPP2R1A	-48		
chr10	89623088	89623514	0.65	1.70E-01	KILLIN	-107	PTEN	106
chr16	74729801	74730045	1.43	1.70E-01	RFWD3	-29144	MLKL	4866
chr7	24758848	24759182	0.58	1.70E-01	DFNA5	38068	MPP6	145930
chr16	11877222	11877651	1.37	1.70E-01	ZC3H7A	-1029		
chr16	89034538	89035041	1.34	1.70E-01	LOC390748	-101776	CBFA2T3	8611
chr12	56323188	56323514	0.73	1.71E-01	DGKA	-2461	WIBG	-1654
chr11	118800912	118801297	1.57	1.71E-01	UPK2	-25921	BCL9L	-19492
chr2	74007122	74007475	1.23	1.71E-01	DUSP11	-15		
chr19	8334018	8334338	1.47	1.71E-01	CD320	39061	LASS4	59961
chr6	26216597	26216973	0.64	1.71E-01	HIST1H2AE	-363	HIST1H2BG	87
chr19	41120447	41120723	1.24	1.72E-01	LTBP4	17444	NUMBL	75971
chr16	30546302	30546584	1.35	1.72E-01	ZNF747	-249		
chr7	3018095	3018517	1.34	1.72E-01	GNA12	-134347	CARD11	65273
chr13	49789177	49789447	0.84	1.72E-01	MLNR	-5162	FNDC3A	239264
chr2	47935930	47936240	1.57	1.72E-01	KCNK12	-138615	MSH6	-74136
chr13	46734679	46735040	1.64	1.73E-01	CPB2	-55649	LCP1	21599
chr7	134331375	134331724	1.26	1.73E-01	BPGM	19		
chr16	30420793	30421071	0.72	1.73E-01	ZNF771	2197	DCTPP1	20441
chr22	23277754	23278152	1.46	1.73E-01	GNAZ	-134716	IGL@	47993
chr22	29137613	29137951	1.48	1.73E-01	HSCB	-261	CHEK2	40
chr19	16295984	16296229	1.28	1.73E-01	FAM32A	-128		
chr17	27279312	27279721	1.57	1.73E-01	PHF12	-1009		
chr17	72869336	72869633	1.22	1.73E-01	FDXR	-329		
chr2	27357325	27357682	1.33	1.73E-01	PREB	38		
chr3	119217148	119217409	1.47	1.73E-01	C3orf1	-89		
chr8	96149933	96150256	0.85	1.73E-01	PLEKHF2	4057		
chr1	186344326	186344720	1.41	1.73E-01	C1orf27	-367	TPR	-66

chr3	14220065	14220546	1.33	1.74E-01	XPC	-134	LSM3	-31
chr12	22778689	22779065	0.78	1.74E-01	ETNK1	801		
chr10	26745959	26746255	1.40	1.74E-01	PDSS1	-240488	APBB1IP	18841
chr3	169530699	169530943	1.54	1.74E-01	LRRC34	-509		
chr4	170581219	170581553	0.81	1.74E-01	CLCN3	39664	C4orf27	97707
chr19	50490417	50490752	1.22	1.75E-01	SIGLEC11	-26156	VRK3	38220
chr18	54684405	54684715	1.43	1.75E-01	ST8SIA3	-335161	WDR7	365944
chr15	67378104	67378631	1.57	1.75E-01	SMAD3	20173	AAGAB	168706
chr11	67416685	67416995	0.78	1.75E-01	TBX10	-9809	ACY3	1290
chr16	30705160	30705505	0.66	1.75E-01	SRCAP	-5129	FBRS	29555
chr3	129158728	129159152	1.40	1.75E-01	MBD4	-88	IFT122	-28
chr6	26305596	26305863	1.59	1.76E-01	BTN3A2	-59668	HIST1H4H	-20003
chr17	4272894	4273318	1.45	1.76E-01	UBE2G1	-3137		
chr15	66681881	66682127	0.66	1.76E-01	MAP2K1	2793	SNAPC5	108142
chr8	67341070	67341380	0.61	1.76E-01	ADHFE1	-3493	RRS1	-38
chr3	9005076	9005355	1.20	1.77E-01	RAD18	-70		
chr11	118436659	118436968	1.31	1.77E-01	C11orf60	-64		
chr12	111872747	111873221	1.19	1.77E-01	SH2B3	29232	ATXN2	164496
chr22	38022089	38022362	1.54	1.77E-01	SH3BP1	-12831	GGA1	17723
chr15	40397824	40398119	1.39	1.77E-01	SRP14	-66583	BMF	3103
chr19	18392473	18392709	1.23	1.77E-01	JUND	-159		
chr2	242383306	242383597	1.52	1.77E-01	STK25	64582	FARP2	87741
chr19	34850116	34850612	1.38	1.77E-01	GPI	-5705	LSM14A	187012
chr6	88464548	88464917	0.78	1.78E-01	SPACA1	-292774	AKIRIN2	-52748
chr12	132434342	132434595	1.22	1.78E-01	EP400	-39		
chr8	86089264	86089612	0.73	1.78E-01	E2F5	-181		
chr1	171282969	171283290	1.66	1.78E-01	FMO4	-356		
chr1	161493547	161493852	1.66	1.79E-01	HSPA6	-336		
chr1	173446081	173446338	1.43	1.79E-01	PRDX6	-276		
chr19	12792106	12792382	1.42	1.79E-01	DHPS	433		
chr19	12035334	12035750	0.81	1.79E-01	ZNF700	-358		
chrX	40943631	40944069	1.25	1.79E-01	USP9X	-1038		
chr7	150020130	150020611	0.70	1.79E-01	LRRC61	75		
chr13	67798350	67798635	1.35	1.79E-01	PCDH9	5975		
chr11	20408834	20409269	0.72	1.79E-01	PRMT3	-24		
chr2	65258417	65258751	1.27	1.79E-01	CEP68	-24911	SLC1A4	42128
chr16	21369596	21369998	1.39	1.79E-01	CRYM	-80140	NPIPL3	66902
chr17	44270014	44270395	0.81	1.80E-01	LRRC37A	-102292	KIAA1267	-20607

chr11	68823404	68823991	1.36	1.80E-01	CCND1	-632175	TPCN2	7348
chr16	56390352	56390707	1.22	1.80E-01	AMFR	68914	GNAO1	165279
chr16	24025610	24025909	1.41	1.80E-01	CACNG3	-241116	PRKCB	178460
chr19	11039269	11039579	1.37	1.80E-01	YIPF2	-67		
chr19	52328082	52328392	0.65	1.81E-01	FPR3	29826	ZNF577	62992
chr3	167452525	167453044	1.62	1.81E-01	SERPINI1	-647	PDCD10	-134
chr5	131758449	131758744	1.38	1.81E-01	SLC22A5	53196	IRF1	67868
chr11	67159211	67159488	1.41	1.81E-01	RAD9A	-73		
chr9	98256305	98256572	1.28	1.81E-01	FANCC	-176448	PTCH1	14392
chr16	30933675	30934029	1.26	1.81E-01	FBXL19	-2044		
chr16	67260850	67261131	1.49	1.81E-01	LRRC29	-90	TMEM208	-25
chr20	43150701	43151048	0.76	1.81E-01	SERINC3	-149		
chr7	124569809	124570058	1.31	1.82E-01	POT1	103		
chr7	37375853	37376168	1.52	1.82E-01	AOAH	-611858	ELMO1	112500
chr10	112116370	112116716	1.42	1.82E-01	DUSP5	-141082	SMNDC1	-51836
chr2	207024195	207024565	1.32	1.82E-01	NDUFS1	-193	EEF1B2	62
chr1	26946837	26947202	1.39	1.82E-01	ARID1A	-75502	RPS6KA1	74677
chr6	131949268	131949628	0.74	1.82E-01	MED23	-85		
chr11	45053507	45053958	0.83	1.82E-01	TP53I11	-81125	SYT13	254151
chrX	12974916	12975284	0.92	1.82E-01	TMSB4X	-18125	TLR8	50342
chr1	40804400	40804745	0.75	1.82E-01	SMAP2	-35155	COL9A2	-21592
chr9	97488824	97489077	1.29	1.82E-01	C9orf3	-43		
chr1	66797256	66797532	1.21	1.82E-01	SGIP1	-202431	PDE4B	538538
chr11	63741801	63742180	1.43	1.83E-01	COX8A	-88		
chr8	38590089	38590380	0.69	1.83E-01	RNF5	-131460	TACC1	-54487
chr22	23480149	23480405	1.40	1.83E-01	RTDR1	3964	GNAZ	67608
chr5	126113962	126114285	1.31	1.83E-01	LMNB1	1291	MARCH3	252316
chr5	159435945	159436340	1.42	1.83E-01	TTC1	-37		
chr20	4954303	4954547	1.21	1.84E-01	RASSF2	-158656	SLC23A2	36514
chr19	7694481	7694767	1.34	1.84E-01	XAB2	-185		
chr17	27475913	27476157	1.38	1.84E-01	TIAF1	-73408	MYO18A	31372
chr1	234659372	234659624	1.61	1.84E-01	TARBP1	-44649	IRF2BP2	85773
chr9	37004657	37005181	1.43	1.84E-01	PAX5	29557	MELK	432014
chr7	77326724	77326975	0.69	1.84E-01	RSBN1L	1107	TMEM60	100897
chr13	99551370	99551637	0.65	1.84E-01	SLC15A1	-146575	DOCK9	187156
chr13	77901215	77901561	1.38	1.84E-01	MYCBP2	-211		
chr11	9710761	9711071	0.76	1.84E-01	SWAP70	25288	SBF2	604838
chr9	33297302	33297804	1.47	1.84E-01	NFX1	7043	AQP7	104964

chr19	2783288	2783772	1.34	1.85E-01	THOP1	-1976	SGTA	-176
chr8	61839149	61839437	0.64	1.85E-01	RLBP1L1	-361232	CHD7	247954
chr2	234312954	234313270	0.57	1.85E-01	DGKD	49959	USP40	161124
chr3	16553411	16553815	1.51	1.85E-01	RFTN1	1609	OXNAD1	246899
chr6	16205625	16206050	0.68	1.85E-01	GMPR	-32973	MYLIP	76521
chr7	100895370	100895844	0.80	1.85E-01	FIS1	-7236	RABL5	69486
chr6	32096032	32096299	0.76	1.86E-01	ATF6B	-149		
chr15	69167988	69168499	1.47	1.86E-01	ANP32A	-54983	NOX5	-54620
chr19	5622632	5623141	1.40	1.87E-01	SAFB	-277	SAFB2	51
chr16	66550709	66551081	1.49	1.87E-01	TK2	33420	BEAN	89655
chr2	197664303	197664608	1.51	1.87E-01	GTF3C3	-31		
chr13	51374606	51374864	1.57	1.88E-01	RNASEH2B	-109157	ST13	628581
chr7	130804246	130804556	0.63	1.89E-01	KLF14	-385541	MKLN1	-208194
chr8	134512856	134513268	1.38	1.89E-01	NDRG1	-203515	ST3GAL1	71121
chr3	129118376	129118693	1.56	1.89E-01	H1FX	-83415	MBD4	40317
chr17	7386590	7386869	1.57	1.89E-01	POLR2A	-1120	ZBTB4	838
chr3	185378265	185378555	1.48	1.89E-01	SENP2	74379	IGF2BP2	164417
chr1	244615433	244615747	1.54	1.89E-01	ADSS	-177		
chr12	118454307	118454802	0.66	1.89E-01	RFC5	47		
chr15	72343642	72343927	0.63	1.89E-01	MYO9A	66637	NR2E3	240891
chr14	21077476	21077752	1.34	1.90E-01	RNASE12	-18632	OR6S1	32236
chr12	133656656	133657333	1.49	1.90E-01	ZNF140	-42		
chr1	163291450	163291782	1.62	1.90E-01	NUF2	-107		
chr8	56792199	56792493	0.60	1.90E-01	LYN	-40		
chr14	91882601	91882889	1.46	1.90E-01	GPR68	-162521	CCDC88C	1388
chr20	10414771	10415047	1.27	1.90E-01	C20orf94	-1042	MKKS	-43
chr1	161135644	161136424	0.73	1.90E-01	PPOX	-147		
chr17	884273	884600	0.68	1.90E-01	NXN	-1427		
chr13	114519991	114520249	0.69	1.91E-01	GAS6	46926	FAM70B	57904
chr3	52481092	52481348	0.68	1.91E-01	SEMA3G	-2177		
chr18	33077515	33078089	1.64	1.91E-01	INO80C	153		
chr3	140911077	140911390	1.21	1.91E-01	ACPL2	-39448	SPSB4	140491
chr15	67813260	67813568	1.22	1.92E-01	C15orf61	-108		
chr16	68108586	68108916	0.71	1.92E-01	DDX28	-50792	NFATC3	-10624
chr19	52154472	52154718	1.44	1.92E-01	SIGLEC14	-4463		
chr16	71518305	71518710	1.34	1.92E-01	ZNF23	-22391	ZNF19	4746
chr6	20291983	20292293	0.83	1.92E-01	E2F3	-109999	MBOAT1	-79468
chr7	99698799	99699321	0.61	1.92E-01	AP4M1	-70	MCM7	367

chr5	167913270	167913580	1.49	1.93E-01	RARS	-38		
chr2	203130274	203130580	1.30	1.93E-01	NOP58	-88		
chr2	85581589	85581902	1.36	1.93E-01	ELMOD3	-97	RETSAT	75
chr21	35288034	35288278	1.33	1.93E-01	SLC5A3	-157667	ITSN1	273372
chr11	118797032	118797329	1.54	1.93E-01	UPK2	-29845	BCL9L	-15568
chr17	34454642	34454974	0.59	1.93E-01	CCL4	23588	CCL3L1	69348
chr1	109642559	109642937	0.69	1.93E-01	KIAA1324	-13785	TMEM167B	9345
chr10	74124074	74124384	0.75	1.93E-01	DNAJB12	-9322	CBARA1	261670
chr7	42923503	42923913	0.71	1.94E-01	GLI3	-647090	C7orf25	28443
chr12	122430136	122430412	1.59	1.94E-01	BCL7A	-29587	PSMD9	103628
chr19	1383091	1383591	1.51	1.94E-01	NDUFS7	-366		
chr20	1415564	1415877	1.37	1.94E-01	FKBP1A	-41905	NSFL1C	32696
chr5	1485140	1485417	1.07	1.94E-01	SLC6A3	-39741	LPCAT1	38797
chr9	86322953	86323321	1.47	1.95E-01	AK300656	-99	UBQLN1	31
chr11	102906754	102907030	1.65	1.95E-01	MMP13	-80430	DYNC2H1	-73268
chr19	29080937	29081240	1.53	1.95E-01	UQCRFS1	623047		
chr2	1618469	1618713	1.76	1.95E-01	PXDN	129700	TPO	201358
chr8	123685748	123686006	0.74	1.95E-01	ZHX2	-108024		
chr19	29110782	29111092	1.23	1.95E-01	UQCRFS1	593199		
chr10	5894326	5894602	1.39	1.95E-01	FBXO18	-41885	GDI2	-38952
chr17	8022131	8022664	0.82	1.96E-01	ALOXE3	-538		
chr7	140376167	140376463	1.28	1.96E-01	NDUFB2	-20166	ADCK2	3362
chr19	18668329	18668662	1.37	1.96E-01	C19orf50	-108		
chr9	125693765	125694088	1.18	1.96E-01	ZBTB26	-148		
chr2	234257843	234258108	1.24	1.96E-01	DGKD	-5177	SAG	41667
chr6	28908708	28909036	1.48	1.96E-01	TRIM27	-17104	ZNF311	64163
chr11	34073671	34073911	1.47	1.96E-01	CAPRIN1	561		
chr5	117842336	117842624	0.58	1.97E-01	TNFAIP8	-849116		
chr7	100026372	100026809	0.76	1.97E-01	MEPCE	-938	ZCWPW1	-289
chr7	156685847	156686088	1.16	1.97E-01	LMBR1	-66		
chr17	27278521	27278962	1.46	1.97E-01	PHF12	-234		
chr1	230031300	230031630	0.66	1.97E-01	GALNT2	-171491	URB2	269484
chr19	36618668	36619041	1.32	1.97E-01	POLR2I	-12649	CAPNS1	-12063
chr2	190648722	190649265	0.69	1.97E-01	ORMDL1	103	PMS1	183
chr19	16181265	16181597	0.76	1.97E-01	RAB8A	-41059	TPM4	3114
chr11	72862981	72863327	0.71	1.97E-01	P2RY2	-66190	FCHSD2	-10011
chr15	75230474	75230726	1.34	1.97E-01	COX5A	-105		
chr2	89157545	89158020	1.26	1.98E-01	O1/O11 and JK2	-732779	RPIA	166607

chr2	182321345	182321656	0.75	1.98E-01	ITGA4	-118		
chr13	34320071	34320478	0.80	1.98E-01	STARD13	-460374	RFC3	-71931
chr10	27531878	27532498	1.43	1.98E-01	ACBD5	-2380		
chr19	41816077	41816502	1.45	1.98E-01	TGFB1	43526	HNRNPUL1	46170
chr16	19535009	19535259	1.32	1.98E-01	GDE1	-1684	CP110	-45
chr7	100076755	100077155	0.63	1.98E-01	TSC22D4	-53		
chr21	43880973	43881323	1.48	1.98E-01	RSPH1	35253	UBASH3A	57129
chr13	101327190	101327544	1.27	1.98E-01	TMTC4	-264		
chr15	84624780	84625091	0.75	1.98E-01	ZSCAN2	-519313	ADAMTSL3	302098
chr1	249167877	249168192	1.51	1.98E-01	ZNF692	-14764		
chr3	133293173	133293483	0.66	1.99E-01	CDV3	894		
chr19	59031120	59031452	1.30	1.99E-01	ZBTB45	-365		
chr5	142814860	142815162	1.52	1.99E-01	NR3C1	-30966	YIPF5	735267
chr2	232539681	232540121	0.66	1.99E-01	NMUR1	-144719	PTMA	-33334
chr11	67124318	67124621	1.49	1.99E-01	POLD4	-3453		
chr14	105531755	105532014	1.60	1.99E-01	GPR132	-131		
chr9	133919141	133919451	0.91	1.99E-01	AIF1L	-52616	LAMC3	34792
chr5	108744629	108745214	0.75	1.99E-01	PJA2	753		
chr1	244816017	244816354	1.37	1.99E-01	FAM36A	-182453	C1orf101	191513
chr3	160603533	160603865	1.62	2.00E-01	PPM1L	129703	B3GALNT1	218984
chr6	52860333	52860843	0.93	2.00E-01	GSTA4	-410		
chr9	128469444	128469702	1.22	2.00E-01	MAPKAP1	-60		
chr1	197898088	197898398	0.87	2.00E-01	NEK7	-227865	LHX9	11726
chr6	26286636	26286911	0.78	2.01E-01	HIST1H4H	-1047		
chr6	139540446	139540756	0.68	2.01E-01	TXLNB	72607	HECA	84352
chr2	150444098	150444571	0.83	2.01E-01	MMADHC	-5		
chr1	208108546	208108896	1.80	2.02E-01	CD34	-24038	PLXNA2	308944
chr1	109288932	109289273	1.25	2.02E-01	STXBP3	-182		
chr8	102149357	102149750	0.74	2.02E-01	YWHAZ	-184333	ZNF706	68406
chr13	49066674	49067037	0.80	2.02E-01	LPAR6	-79203	RCBTB2	40460
chr1	24127247	24127525	1.38	2.02E-01	GALE	-1326		
chr12	9102550	9102892	1.33	2.02E-01	M6PR	-469		
chr2	29093043	29093400	1.38	2.02E-01	TRMT61B	-47		
chr1	33282783	33283156	1.34	2.02E-01	S100PBP	-206	YARS	663
chr1	226216709	226216968	1.43	2.02E-01	LEFTY2	-87919	H3F3B	-33582
chr12	110486271	110486640	1.39	2.02E-01	IFT81	-75684	GIT2	-52262
chr9	125027005	125027252	1.30	2.02E-01	MRRF	-18	RBM18	14
chr9	140130929	140131239	1.30	2.03E-01	TUBB2C	-4627		

chr7	120590845	120591364	1.31	2.03E-01	ING3	288		
chr20	49545850	49546235	1.43	2.03E-01	ADNP	1484	BCAS4	134576
chr5	180618546	180618950	1.53	2.03E-01	TRIM7	13429	OR2V2	36805
chrX	152110050	152110331	1.22	2.04E-01	PNMA3	-114575	ZNF185	27194
chr5	123985846	123986270	1.46	2.04E-01	ZNF608	94807		
chr9	139702151	139702470	1.37	2.04E-01	C9orf86	119		
chr1	154947274	154947657	1.24	2.04E-01	SHC1	-4243	CKS1B	348
chr11	64037373	64037732	1.38	2.04E-01	BAD	14623	PLCB3	18431
chr18	21977880	21978215	0.73	2.04E-01	OSBPL1A	-258		
chr1	153763556	153763980	1.52	2.04E-01	SLC27A3	16000	GATAD2B	131683
chr11	125757958	125758268	0.74	2.05E-01	HYLS1	4604	PUS3	15003
chr22	37638490	37638823	1.35	2.05E-01	SSTR3	-30304	RAC2	1648
chr9	136890411	136890667	1.15	2.05E-01	VAV2	-33093	BRD3	42602
chr2	228189800	228190078	1.56	2.05E-01	MFF	-2289		
chr17	2907571	2907964	0.72	2.05E-01	OR1D4	59187	RAP1GAP2	208036
chr3	52443918	52444173	1.29	2.05E-01	PHF7	-481	BAP1	-37
chr3	48229790	48230258	0.72	2.05E-01	CDC25A	-223		
chr19	36438350	36438660	1.52	2.05E-01	SDHAF1	-47596	LRFN3	10483
chr19	11302532	11302939	0.67	2.06E-01	LOC55908	-46081	SPC24	-36252
chr20	45891245	45891706	0.77	2.06E-01	ZMYND8	93998	EYA2	368213
chr6	26045521	26045831	1.29	2.06E-01	HIST1H2BB	-1791	HIST1H3C	37
chr19	18632877	18633187	0.72	2.06E-01	ELL	-95		
chr14	81444783	81445171	1.33	2.06E-01	TSHR	23108	GTF2A1	242317
chr1	19578013	19578323	0.76	2.06E-01	KIAA0090	-122	MRTO4	93
chr6	157744457	157744707	0.77	2.07E-01	C6orf35	211		
chr14	106873540	106873864	0.91	2.07E-01	ADAM6	-435344	IGHV7-81	409581
chr6	56954648	56955049	0.73	2.07E-01	ZNF451	21		
chr10	12285925	12286207	1.33	2.07E-01	CAMK1D	-105517	NUDT5	-47923
chr11	60673848	60674276	1.30	2.07E-01	PRPF19	-1		
chr15	77197638	77197898	1.39	2.07E-01	RCN2	-26194	SCAPER	-21551
chr12	76741946	76742455	0.69	2.07E-01	BBS10	21		
chr19	6588037	6588313	1.17	2.08E-01	CD70	2988	TNFSF9	57165
chr7	66093379	66093751	0.76	2.08E-01	KCTD7	-325		
chr15	52526699	52527034	0.75	2.08E-01	GNB5	-43302	MYO5C	61103
chr12	50038390	50038635	0.77	2.08E-01	PRPF40B	14185	FMNL3	62684
chr7	44678836	44679080	1.28	2.08E-01	ZMIZ2	-109572	OGDH	32787
chr14	55737859	55738169	0.77	2.09E-01	DLGAP5	-79618	KIAA0831	140562
chr11	65868190	65868546	0.72	2.09E-01	KLC2	-156806	PACS1	30544

chr14	23388215	23388798	0.70	2.09E-01	RBM23	-111		
chr19	34801242	34801552	0.61	2.09E-01	GPI	-54672	LSM14A	138045
chr19	42721862	42722287	1.38	2.09E-01	ZNF526	-2417	DEDD2	-262
chr17	47755292	47755627	1.34	2.09E-01	SPOP	65		
chr11	72863903	72864268	1.50	2.09E-01	P2RY2	-65258	FCHSD2	-10943
chr6	143265577	143265913	1.55	2.09E-01	HIVEP2	593		
chr19	41256155	41256573	1.26	2.09E-01	SNRPA	-415		
chr11	61730624	61730919	1.41	2.09E-01	FTH1	4360	BEST1	13416
chr7	101504448	101504860	0.66	2.09E-01	SH2B2	-423751	CUX1	43772
chr17	1552074	1552417	1.24	2.10E-01	SCARF1	-3205		
chr22	38859361	38859637	1.60	2.10E-01	KDELR3	-4584		
chr19	984146	984458	1.23	2.10E-01	GRIN3B	-16135	ARID3A	58265
chr22	37703086	37703330	0.58	2.10E-01	CYTH4	24713	ELFN2	120297
chr17	42976777	42977162	0.77	2.10E-01	YQ028	-3157	EFTUD2	23
chr7	48149806	48150050	0.72	2.10E-01	ABCA13	-61129	UPP1	21573
chr19	50979446	50979820	1.21	2.10E-01	C19orf63	-103		
chr2	223776765	223777196	1.48	2.10E-01	KCNE4	-139881	ACSL3	51249
chr6	150262911	150263193	1.39	2.10E-01	ULBP2	-84		
chr5	122733975	122734316	0.72	2.11E-01	CEP120	25106	PRDM6	309305
chr18	33729914	33730348	0.81	2.11E-01	MOCOS	-37349	ELP2	20244
chr12	49245879	49246317	1.37	2.11E-01	DDX23	-141		
chr2	11970212	11970654	1.21	2.11E-01	TRIB2	-886565	LPIN1	83693
chr11	130786297	130786593	1.36	2.11E-01	SNX19	-63		
chr12	1425035	1425312	1.66	2.11E-01	FBXL14	278157	ERC1	324770
chr3	126793714	126794143	0.81	2.11E-01	PLXNA1	86427	TPRA1	515639
chr14	99947603	99947847	1.40	2.12E-01	CCNK	-14		
chr19	58090192	58090551	1.35	2.12E-01	ZNF416	-129		
chr9	95491598	95491908	0.72	2.12E-01	IPPK	-59206	BICD2	35330
chr17	18759488	18759761	1.45	2.12E-01	PRPSAP2	-1867		
chr11	111288520	111288862	1.49	2.13E-01	POU2AF1	-38534	BTG4	94373
chr19	3133476	3133872	1.27	2.13E-01	GNA15	-2517		
chr3	3221265	3221565	1.52	2.13E-01	CRBN	-25		
chr5	98264875	98265491	1.37	2.13E-01	CHD1	-2945		
chr1	154810121	154810435	1.27	2.13E-01	ADAR	-229596	KCNN3	32476
chr2	131862516	131862899	0.70	2.13E-01	PLEKHB2	288		
chr6	43238957	43239269	1.45	2.13E-01	SLC22A7	-26885	TTBK1	27891
chr11	118955265	118955704	1.37	2.13E-01	HMBS	-102		
chr1	160752079	160752389	1.25	2.13E-01	LY9	-13694	SLAMF7	43157

chr1	222885970	222886339	1.33	2.14E-01	AIDA	-291	C1orf58	249
chr1	202896303	202896622	1.50	2.14E-01	KLHL12	-92		
chr17	35305997	35306267	1.21	2.14E-01	AATF	-43		
chr18	72219236	72219480	1.77	2.14E-01	ZNF407	-123565	CNDP1	17666
chr2	43037487	43037862	1.35	2.14E-01	HAAO	-17924	ZFP36L2	416070
chr2	8714419	8714694	1.17	2.14E-01	ID2	-107427		
chr17	40608859	40609116	1.39	2.14E-01	ATP6V0A1	-1874		
chr15	67155221	67155531	0.78	2.14E-01	SMAD3	-202819	SMAD6	160702
chr16	67514952	67515385	1.24	2.14E-01	ATP6V0D1	-80		
chr3	185826843	185827136	1.35	2.14E-01	ETV5	-89		
chr8	42698124	42698781	0.72	2.14E-01	THAP1	21		
chr17	29821258	29821595	1.53	2.15E-01	RAB11FIP4	102785	C17orf79	364899
chr17	7464320	7464768	0.74	2.15E-01	SENP3	-775		
chr11	47884755	47885191	1.28	2.15E-01	PTPRJ	-117137	NUP160	-14916
chr2	8821022	8821627	1.48	2.15E-01	ID2	-659		
chr18	48680276	48680683	1.31	2.15E-01	MEX3C	43210	SMAD4	123897
chr8	71314252	71314684	0.76	2.16E-01	PRDM14	-330906	NCOA2	1552
chr20	305555	306150	0.70	2.16E-01	SOX12	-386		
chr22	26931457	26931804	0.79	2.16E-01	TFIP11	-23194	TPST2	29739
chr19	47730312	47730588	1.35	2.16E-01	BBC3	5573	SAE1	96370
chr7	150755032	150755318	1.25	2.17E-01	SLC4A2	-1482	CDK5	-179
chr10	98460450	98460760	0.74	2.17E-01	TM9SF3	-113796	PIK3AP1	19674
chr14	107239538	107239913	0.82	2.17E-01	ADAM6	-801368	IGHV7-81	43557
chr1	40264900	40265245	0.91	2.17E-01	BMP8B	-10540	TRIT1	84104
chr7	102072284	102072569	0.71	2.17E-01	ORAI2	-1569		
chr15	90602764	90603121	1.41	2.17E-01	IDH2	42765	ZNF710	58191
chr20	42839327	42839774	1.37	2.17E-01	JPH2	-23333	FITM2	100338
chr7	45026237	45026555	0.67	2.17E-01	CCM2	-13391	MYO1G	-7692
chr2	231069780	231070125	1.53	2.18E-01	SLC16A14	-136334	SP110	14874
chr17	78388854	78389134	1.27	2.18E-01	FLJ35220	27		
chr17	7146209	7146487	1.38	2.18E-01	PHF23	-3523	GABARAP	-595
chr14	62019622	62019923	1.32	2.18E-01	HIF1A	-142346	PRKCH	231258
chr3	113233916	113234246	1.27	2.18E-01	CCDC52	-47		
chr2	70120893	70121213	1.45	2.18E-01	SNRNP27	-37		
chr19	59025265	59025559	1.46	2.18E-01	SLC27A5	-1980		
chr9	3526215	3526673	0.78	2.18E-01	RFX3	-461		
chr1	149870947	149871223	1.36	2.19E-01	BOLA1	-70		
chr7	150924214	150924598	1.38	2.19E-01	ABCF2	-89		

chr1	231347112	231347461	1.47	2.19E-01	GNPAT	-29632	TRIM67	48613
chr17	78120892	78121178	1.25	2.19E-01	EIF4A3	-97		
chr1	26868400	26868682	0.73	2.19E-01	RPS6KA1	-3802		
chr4	169753034	169753283	0.72	2.19E-01	CBR4	178263	PALLD	334942
chr12	7060255	7060580	0.72	2.19E-01	PTPN6	4678	PHB2	19498
chr17	74178371	74178673	1.43	2.19E-01	FOXJ1	-41877	RNF157	57868
chr22	26879664	26879938	1.35	2.19E-01	HPS4	-4250	SRRD	-49
chr13	92000690	92000997	1.34	2.19E-01	GPC5	-50091		
chr6	36648227	36648503	1.51	2.19E-01	CDKN1A	1906	CPNE5	158855
chr17	43394678	43395279	1.36	2.19E-01	MAP3K14	-565		
chr7	106810117	106810442	1.36	2.19E-01	HBP1	820		
chr4	89241842	89242155	1.35	2.20E-01	HERC6	-57892	PPM1K	-36111
chr7	66767467	66767900	0.61	2.20E-01	TYW1	305867		
chr6	44306308	44306588	0.83	2.20E-01	CDC5L	-48854	AARS2	-25385
chr10	111985489	111985780	1.38	2.20E-01	MXI1	18272	SMNDC1	79072
chr9	116341172	116341575	0.68	2.20E-01	ZNF618	-297188	RGS3	134363
chr6	26056415	26056792	0.76	2.20E-01	HIST1H1C	95		
chr6	43337200	43337560	0.64	2.20E-01	ZNF318	-199		
chr14	50065423	50065983	0.75	2.20E-01	PPIL5	288		
chr2	148778301	148778654	1.43	2.21E-01	ORC4L	658		
chr7	23571623	23571897	0.65	2.21E-01	TRA2A	-104		
chr16	2009369	2009702	1.38	2.21E-01	RPL3L	-4857	NDUFB10	19
chr16	72206248	72206579	0.89	2.21E-01	PMFBP1	-350		
chr6	6008747	6009090	0.63	2.21E-01	NRN1	-1286		
chr4	107237413	107237723	0.88	2.21E-01	TBCK	-145	AIMP1	-96
chr10	104941071	104941315	1.54	2.21E-01	NT5C2	11863	CNNM2	263079
chr7	50485788	50486041	0.69	2.21E-01	FIGNL1	32173	IKZF1	141537
chr11	117820975	117821341	1.66	2.21E-01	IL10RA	-35948	TMPRSS13	-21043
chr1	25345925	25346229	1.35	2.22E-01	RUNX3	-89307	SYF2	212936
chr4	77997387	77997641	1.37	2.22E-01	CCNI	-389		
chr11	44622623	44622867	1.35	2.22E-01	TSPAN18	-305213	CD82	35604
chr17	1619735	1620015	1.26	2.23E-01	PRPF8	-31699	SERPINF2	-26255
chr3	188139653	188140021	1.45	2.23E-01	TPRG1	-749926	LPP	209116
chr19	39926509	39926763	1.38	2.23E-01	RPS16	-18		
chr6	28909283	28909559	1.58	2.23E-01	TRIM27	-17653	ZNF311	63614
chr1	16694148	16694458	0.73	2.23E-01	FBXO42	-15355	SPATA21	69616
chr6	32163530	32163974	0.70	2.23E-01	GPSM3	-452		
chr12	1900326	1900740	0.64	2.23E-01	LRTM2	-28900	ADIPOR2	100286

chr2	118813680	118813990	1.44	2.23E-01	INSIG2	-32215	DDX18	241580
chr19	54704448	54704764	1.26	2.23E-01	RPS9	-120		
chr2	112463388	112463939	1.51	2.23E-01	ANAPC1	178077	BCL2L11	585173
chr3	44379780	44380402	0.74	2.23E-01	C3orf23	147		
chr17	17876025	17876344	1.26	2.23E-01	TOM1L2	-401	LRRC48	58
chr8	107282205	107282520	1.93	2.24E-01	OXR1	-110		
chr1	110927807	110928182	0.73	2.24E-01	SLC16A4	5641	RBM15	46050
chr19	13056398	13056806	1.42	2.24E-01	RAD23A	-52		
chr17	16440117	16440467	0.69	2.24E-01	ZNF287	32228	C17orf45	97991
chr21	46707792	46708036	1.32	2.24E-01	POFUT2	-103		
chr18	58546001	58546275	1.29	2.25E-01	CDH20	-611497	MC4R	-506137
chr8	67039017	67039352	0.77	2.25E-01	TRIM55	-93		
chr11	57424909	57425307	1.41	2.25E-01	CLP1	-108		
chr19	7402696	7403015	0.75	2.25E-01	INSR	-108845	ARHGEF18	-101718
chr5	131722310	131722672	0.88	2.25E-01	SLC22A5	17090	IRF1	103974
chr7	40174069	40174581	0.73	2.25E-01	C7orf10	-250	C7orf11	-67
chr10	71267654	71267898	0.74	2.25E-01	TSPAN15	56550	NEUROG3	65346
chr3	14473690	14474139	1.29	2.25E-01	SLC6A6	29809	GRIP2	109673
chr19	37663509	37663870	1.31	2.25E-01	ZNF585A	-75		
chr8	24224813	24225202	1.51	2.26E-01	ADAMDEC1	-16790	ADAM28	73428
chr13	91999681	92000082	1.28	2.26E-01	GPC5	-51053		
chr13	114992263	114992523	0.83	2.26E-01	RASA3	-94298	CDC16	-7969
chr22	27068557	27069182	1.46	2.26E-01	CRYBA4	50942		
chr8	87354558	87354838	1.20	2.26E-01	WWP1	-296		
chr19	39390192	39390686	1.30	2.27E-01	NFKBIB	-176	SIRT2	-78
chr13	41635468	41635891	0.85	2.27E-01	WBP4	-17		
chr22	42062675	42062937	1.34	2.27E-01	NHP2L1	22107	XRCC6	45511
chr3	114866429	114866689	1.59	2.27E-01	ZBTB20	-432		
chr16	70834926	70835218	1.24	2.28E-01	VAC14	-11		
chr2	65357296	65357570	1.29	2.28E-01	RAB1A	2		
chr1	155145667	155145927	1.35	2.28E-01	TRIM46	-563	KRTCAP2	7
chrX	1620174	1620452	0.71	2.28E-01	ASMTL	-48469	P2RY8	35724
chr4	26859124	26859430	1.29	2.28E-01	STIM2	-3087		
chr6	52528496	52528786	0.68	2.28E-01	TRAM2	-86779	TMEM14A	-7243
chr8	42010138	42010499	1.43	2.28E-01	AP3M2	-145		
chr1	99337084	99337443	1.26	2.28E-01	PAP2D	133185	SNX7	210028
chr8	37593998	37594260	1.28	2.28E-01	ERLIN2	32		
chr7	22893638	22893990	1.20	2.28E-01	TOMM7	-31393	FAM126A	159956

chr5	180478018	180478328	0.77	2.28E-01	OR2V2	-103770	BTNL9	10948
chr6	159290906	159291160	1.29	2.28E-01	OSTCL	-12369	TAGAP	175151
chr6	33176018	33176409	0.65	2.29E-01	RING1	-72		
chr1	24517448	24517770	0.71	2.29E-01	IL28RA	-3858		
chr12	7052963	7053554	1.62	2.29E-01	PTPN6	-2481		
chr3	170587839	170588223	1.41	2.29E-01	RPL22L1	14		
chr14	65785421	65785701	0.61	2.29E-01	MAX	-216334	FUT8	-93974
chr14	91885152	91885435	0.76	2.30E-01	CCDC88C	-1161		
chr7	64838627	64839053	0.72	2.30E-01	ZNF92	72		
chr10	104401692	104402136	0.68	2.30E-01	TRIM8	-2338		
chr12	52512102	52512529	1.30	2.30E-01	C12orf44	48558	KRT80	73468
chr2	120934714	120935099	0.82	2.30E-01	TMEM185A	46077	EPB41L5	164238
chr11	111250284	111250661	1.26	2.30E-01	POU2AF1	-316		
chr20	30102012	30102457	1.25	2.30E-01	HM13	-6		
chr20	60876886	60877196	0.75	2.30E-01	ADRM1	-986		
chr8	56986975	56987308	0.77	2.30E-01	RPS20	-2		
chr6	44144881	44145125	1.48	2.31E-01	SLC29A1	-46293	CAPN11	18455
chr19	38405587	38405977	0.86	2.31E-01	SIPA1L3	7914	DPF1	309108
chr15	86232926	86233236	0.82	2.31E-01	KLHL25	105108	AKAP13	309210
chr5	143550149	143550433	1.40	2.31E-01	KCTD16	-146	YIPF5	-13
chr1	211556002	211556288	1.32	2.31E-01	TRAF5	55997	RD3	110114
chr8	78042887	78043220	0.57	2.31E-01	PXMP3	-130530		
chr19	39902622	39903376	1.48	2.32E-01	PLEKHG2	-751		
chr9	37290430	37290688	0.59	2.32E-01	GRHPR	-132148	ZCCHC7	170090
chr12	129019503	129019748	1.49	2.32E-01	TMEM132C	120335	SLC15A4	288915
chr2	231068070	231068346	1.56	2.32E-01	SLC16A14	-134589	SP110	16619
chr5	78810337	78810631	1.44	2.32E-01	HOMER1	-784		
chr6	159137014	159137324	0.77	2.32E-01	SYTL3	66123	EZR	102171
chr1	155907938	155908266	0.84	2.33E-01	RXFP4	-3378		
chr19	10514147	10514428	1.42	2.33E-01	CDC37	-17		
chr22	41844435	41844794	0.66	2.33E-01	TOB2	-1588		
chr22	43486198	43486516	1.27	2.33E-01	TTLL1	-923		
chr9	130186427	130186827	1.37	2.33E-01	ZNF79	-26		
chr17	48981602	48981856	0.76	2.33E-01	TOB1	-40316	SPAG9	216497
chr19	50220791	50221101	0.67	2.33E-01	CPT1C	26545	TSKS	45569
chr2	27502543	27503053	0.71	2.33E-01	TRIM54	-2802		
chr14	51280295	51280551	0.69	2.34E-01	SAV1	-145400	NIN	17416
chr6	2765565	2765927	0.77	2.34E-01	WRNIP1	80		

chr2	95787503	95787813	0.81	2.34E-01	MRPS5	96		
chr3	193788334	193788805	1.30	2.34E-01	HES1	-65364	OPA1	477637
chr3	196594584	196594878	1.23	2.34E-01	SENP5	4		
chr1	23866268	23866578	0.68	2.34E-01	E2F2	-8710	ID3	19899
chr11	67416309	67416683	0.69	2.34E-01	TBX10	-9465	ACY3	1634
chr10	94050780	94051079	1.21	2.35E-01	CPEB3	-86	MARCH5	10
chr5	130506408	130506694	1.29	2.35E-01	LYRM7	-90		
chr17	27224244	27224715	1.26	2.35E-01	FLOT2	235		
chr9	134127974	134128284	1.55	2.35E-01	PPAPDC3	-36952	NUP214	127148
chr1	28573726	28574086	0.65	2.35E-01	SESN2	-12100	ATPIF1	11295
chr18	21498466	21498776	0.76	2.35E-01	CABYR	-220334	LAMA3	229059
chr11	111293780	111294056	1.39	2.35E-01	POU2AF1	-43761	BTG4	89146
chr10	105127568	105127864	1.48	2.36E-01	TAF5	-8		
chr18	77438964	77439557	1.39	2.36E-01	CTDP1	-540		
chr2	3383281	3383708	1.35	2.36E-01	TSSC1	-1842	TTC15	49
chr22	22465206	22465669	1.45	2.36E-01	VPREB1	-133762	TOP3B	-128291
chr6	32912182	32912449	0.83	2.36E-01	HLA-DMB	-3499		
chr10	103911849	103912186	0.70	2.36E-01	NOLC1	85		
chr15	74833146	74833480	1.38	2.36E-01	ARID3B	-235		
chr19	45393602	45393904	1.39	2.36E-01	TOMM40	-724		
chr6	159290363	159290826	0.79	2.36E-01	OSTCL	-11931	TAGAP	175589
chrX	53710991	53711470	1.18	2.36E-01	HSD17B10	-249908	HUWE1	2442
chr17	30676978	30677284	1.36	2.36E-01	ZNF207	-26		
chr12	49463623	49464019	1.17	2.37E-01	RHEBL1	-46		
chr16	47175619	47175897	0.64	2.37E-01	DNAJA2	-168133	NETO2	2150
chr15	90777191	90777466	1.19	2.37E-01	CIB1	-50		
chr7	73515445	73515770	1.36	2.37E-01	EIF4H	-73098	LIMK1	17452
chr1	236118004	236118520	1.32	2.37E-01	LYST	-88042	NID1	110219
chr15	59698669	59698963	1.36	2.37E-01	GCNT3	-205166	MYO1E	-33745
chr7	75988553	75989004	1.34	2.37E-01	YWHAG	-437		
chr4	83295105	83295540	1.32	2.37E-01	HNRNPD	-174		
chr7	24875668	24876010	0.72	2.37E-01	DFNA5	-78756	OSBPL3	143921
chr6	17706189	17706491	0.80	2.37E-01	NUP153	478		
chr9	80912310	80912761	0.81	2.37E-01	PSAT1	477		
chr1	45196923	45197257	1.50	2.37E-01	KIF2C	-8400	C1orf228	56696
chr1	181056653	181056944	1.38	2.37E-01	CACNA1E	-395917	MR1	53660
chr19	47633791	47634303	1.31	2.37E-01	SAE1	-33		
chr13	41054776	41055226	0.79	2.37E-01	FOXO1	185733	COG6	825237

chr11	111287905	111288270	1.46	2.38E-01	POU2AF1	-37931	BTG4	94976
chr22	41985661	41986207	0.69	2.39E-01	PMM1	-63		
chr14	68162223	68162590	0.70	2.39E-01	RDH11	103		
chr11	85753021	85753283	1.52	2.39E-01	SYTL2	-315641	PICALM	26956
chr5	74807513	74807818	1.25	2.39E-01	POLK	9	COL4A3BP	140
chr2	160106563	160106807	1.59	2.39E-01	WDSUB1	36551	TANC1	281539
chr12	6982385	6983021	0.73	2.39E-01	SPSB2	-254		
chr9	130668815	130669074	1.38	2.39E-01	ST6GALNAC6	-7074	ST6GALNAC4	10360
chr1	108440112	108440354	0.75	2.39E-01	VAV3	67312	NTNG1	757604
chr2	70475688	70476035	1.21	2.39E-01	TIA1	-83		
chr14	74868054	74868576	0.70	2.40E-01	TMEM90A	24490	C14orf115	53149
chr1	174968346	174968985	1.35	2.40E-01	CACYBP	-226		
chr19	2061541	2061851	0.69	2.40E-01	MKNK2	-10453	MOBKL2A	34573
chr15	41583330	41583603	1.47	2.40E-01	OIP5	41352	CHP	60030
chr20	44746746	44747112	1.41	2.41E-01	CD40	23		
chr11	18655822	18656127	0.78	2.41E-01	TMEM86A	-64376	UEVLD	-45694
chr8	56756655	56756981	0.86	2.41E-01	TMEM68	-70933	LYN	-35568
chr21	43298037	43298582	0.82	2.41E-01	RIPK4	-111061	PRDM15	1281
chrX	64754690	64754988	1.30	2.41E-01	LAS1L	-172		
chr19	13318970	13319231	1.42	2.42E-01	IER2	57819	CACNA1A	298173
chr3	169649643	169650299	1.52	2.42E-01	LRRC31	-62311	SEC62	-34609
chr3	16306254	16306682	0.76	2.42E-01	OXNAD1	-246	DPH3	28
chr12	25209603	25210034	0.80	2.42E-01	LYRM5	-138331	LRMP	4578
chr22	21983612	21983869	1.26	2.42E-01	SDF2L1	-12801	UBE2L3	61784
chr6	31515165	31515518	0.65	2.42E-01	ATP6V1G2	-715	NFKBIL1	-11
chr20	39570845	39571155	0.96	2.43E-01	MAFB	-253124	TOP1	-86462
chr19	56186441	56186699	1.26	2.43E-01	EPN1	-1421		
chr17	4843281	4843542	1.17	2.43E-01	RNF167	-218	SLC25A11	-94
chr2	27886588	27886848	1.45	2.43E-01	SUPT7L	-269	SLC4A1AP	380
chr21	45358922	45359221	1.43	2.43E-01	TRAPPC10	-73134	AGPAT3	73956
chr6	35995167	35995681	0.67	2.43E-01	SLC26A8	-3047	MAPK14	-30
chr17	38689614	38689950	1.41	2.44E-01	TNS4	-31928	CCR7	31942
chr6	84569061	84569428	1.39	2.44E-01	CYB5R4	-125		
chr3	15106705	15106983	1.37	2.44E-01	MRPS25	-28		
chr2	204103516	204103923	1.47	2.44E-01	CYP20A1	556		
chr21	46529707	46530003	1.58	2.44E-01	ADARB1	35362	POFUT2	177956
chr16	1358957	1359252	1.19	2.45E-01	UBE2I	-75		
chr11	111742148	111742424	1.54	2.45E-01	ALG9	19		

chr17	30228603	30228934	1.29	2.45E-01	UTP6	-40		
chr9	565529	565914	0.82	2.46E-01	DMRT1	-275968	KANK1	61019
chr12	133287165	133287505	1.23	2.46E-01	PGAM5	-101		
chr2	24149893	24150206	1.24	2.46E-01	ATAD2B	-116		
chr5	180648949	180649197	1.57	2.47E-01	TRIM41	-1233		
chr16	4524477	4524835	0.88	2.47E-01	HMOX2	-1685	NMRAL1	240
chr3	14443698	14444094	1.21	2.47E-01	SLC6A6	-210		
chr11	71823597	71824021	1.46	2.48E-01	C11orf51	13		
chr6	58287638	58287931	0.73	2.48E-01				
chr19	54618877	54619286	1.40	2.48E-01	TFPT	-27	PRPF31	292
chr13	28713432	28713676	1.20	2.48E-01	FLT3	-38825	PAN3	-34855
chr11	18548320	18548630	0.84	2.48E-01	TSG101	14		
chr2	74618983	74619383	0.74	2.48E-01	DCTN1	-11708	RTKN	49877
chr11	59383644	59384218	0.83	2.48E-01	OSBP	-314		
chr12	12868780	12869107	1.51	2.49E-01	CDKN1B	-1358		
chr2	183943340	183943658	1.19	2.49E-01	DUSP19	212		
chr12	96455715	96455995	1.51	2.50E-01	ELK3	-132352	LTA4H	-26490
chr4	1857812	1858248	1.20	2.50E-01	LETM1	-56		
chr5	139937032	139937320	1.26	2.50E-01	SRA1	502		
chr17	28050654	28050980	0.73	2.50E-01	GIT1	-134207	SSH2	206201
chr5	173043678	173044011	1.26	2.50E-01	BOD1	-179		
chr20	1373706	1374107	1.38	2.50E-01	FKBP1A	-91		
chr16	4401227	4401517	1.32	2.50E-01	VASN	-20477	GLIS2	19156
chr12	57081956	57082446	0.63	2.51E-01	PTGES3	-123		
chr7	139910085	139910479	1.47	2.51E-01	JHDM1D	-33541	SLC37A3	188029
chr1	84464764	84465028	0.59	2.51E-01	TTLL7	-63		
chr1	160759476	160759827	1.18	2.51E-01	LY9	-6276	SLAMF7	50575
chr22	42486854	42487167	0.74	2.51E-01	NDUFA6	-123		
chr7	35749124	35749369	0.82	2.51E-01	SEPT7	-91380	HERPUD2	-14475
chr9	114393356	114393828	1.25	2.51E-01	DNAJC25	-40		
chr19	46087818	46088445	1.36	2.52E-01	OPA3	-10		
chr6	53174352	53174777	0.76	2.52E-01	GCM1	-160941	ELOVL5	39377
chr9	125987676	125988057	1.27	2.53E-01	STRBP	42976	GPR21	191021
chr8	11275375	11275771	0.69	2.53E-01	BLK	-75948	AMAC1L2	87078
chr16	30934301	30934789	1.54	2.54E-01	FBXL19	-1351		
chr7	157179905	157180201	1.51	2.54E-01	DNAJB6	50343		
chr7	56019380	56019693	0.75	2.54E-01	GBAS	-12759	ZNF713	39209
chr11	6640537	6640908	0.83	2.54E-01	TPP1	-31		

chr6	53109827	53110137	0.75	2.54E-01	GCM1	-96358	ELOVL5	103960
chr15	75917977	75918302	0.82	2.54E-01	SNUPN	579		
chr21	34681110	34681473	0.72	2.54E-01	IFNAR1	-15922	IFNAR2	79061
chr11	72526751	72527074	0.77	2.55E-01	ATG16L2	1462	FCHSD2	326230
chr16	68318835	68319145	0.77	2.55E-01	PRMT7	-25955	SLC7A6	20567
chr3	107843250	107843507	1.68	2.55E-01	CD47	-33444	IFT57	98038
chr1	144932168	144932581	1.36	2.55E-01	PPIAL4A	-568129	PDE4DIP	62647
chr11	61124649	61124978	1.43	2.56E-01	CYBASC3	-453		
chr15	63795442	63795738	1.22	2.56E-01	USP3	-1220		
chr10	63759903	63760260	1.23	2.56E-01	ARID5B	98639	RTKN2	268384
chr2	220114263	220114575	0.82	2.57E-01	GLB1L	-4288	TUBA4B	-3546
chr2	201828370	201828615	1.42	2.57E-01	ORC2L	-83		
chr1	110359152	110359426	1.49	2.57E-01	CSF1	-93944	EPS8L3	-52725
chr13	28021112	28021422	0.83	2.57E-01	MTIF3	3444	GTF3A	22586
chr2	86422460	86422746	1.42	2.57E-01	MRPL35	-3953	IMMT	290
chr19	49956610	49957004	1.22	2.57E-01	PIH1D1	-1692	ALDH16A1	334
chr20	52346132	52346450	1.24	2.57E-01	ZNF217	-146584	BCAS1	341013
chr11	57298174	57298565	1.35	2.58E-01	TIMM10	-138		
chr9	100684720	100684992	1.24	2.58E-01	C9orf156	-4		
chr1	41174829	41175199	0.73	2.58E-01	KCNQ4	-74670	NFYC	17772
chr3	142937695	142938012	1.47	2.58E-01	CHST2	99186	SLC9A9	629492
chr5	149380057	149380301	1.39	2.58E-01	HMGXB3	10	TIGD6	38
chr11	36310856	36311166	1.34	2.58E-01	PRR5L	-86524	LDLRAD3	345399
chr3	108308096	108308736	0.75	2.58E-01	KIAA1524	75	DZIP3	79
chr3	9773077	9773509	1.24	2.59E-01	BRPF1	-141		
chr16	87756893	87757203	1.39	2.59E-01	KLHDC4	42494	JPH3	120549
chr13	46038828	46039126	1.21	2.59E-01	COG3	-94		
chr19	1076978	1077327	1.33	2.59E-01	HMHA1	9979	POLR2E	18238
chr9	116385177	116385549	1.38	2.59E-01	ZNF618	-253199	RGS3	178352
chr17	74523657	74523928	1.23	2.59E-01	RHBDF2	-26285	CYGB	9989
chr6	26538190	26538685	0.78	2.59E-01	HMGN4	-134		
chr22	43019512	43019863	1.17	2.59E-01	POLDIP3	-8726	CYB5R3	25717
chr13	47180364	47180664	1.48	2.59E-01	LRCH1	53218	ESD	190853
chr7	2178023	2178299	1.17	2.59E-01	MAD1L1	94422	ELFN1	429363
chr22	43507211	43507500	1.20	2.59E-01	BIK	599		
chr21	47706054	47706329	1.39	2.60E-01	MCM3AP	-956	C21orf57	-75
chr19	42364092	42364587	1.36	2.60E-01	RPS19	352		
chr19	58912647	58913017	1.10	2.60E-01	ZNF584	-7231	RPS5	14196

chr3	183966585	183966906	1.36	2.60E-01	ECE2	-699	ALG3	13
chr6	88589276	88589649	1.26	2.60E-01	AKIRIN2	-177478	SPACA1	-168044
chr5	157156938	157157188	1.81	2.61E-01	THG1L	-1260		
chr17	27046791	27047150	1.37	2.61E-01	RAB34	-1685	RPL23A	-29
chr6	42710160	42710524	0.80	2.61E-01	PRPH2	-19984	TBCC	3542
chr5	151129200	151129448	1.11	2.61E-01	SPARC	-62807	ATOX1	8886
chr3	179065217	179065628	1.25	2.61E-01	MFN1	-57		
chr1	93645865	93646270	0.66	2.62E-01	TMED5	178		
chr1	16212769	16213013	0.64	2.62E-01	SPEN	38532	ZBTB17	89736
chr14	92905985	92906268	1.24	2.63E-01	RIN3	-73998	SLC24A4	115975
chr19	46061727	46062014	1.27	2.63E-01	OPA3	26251	VASP	51183
chr3	133291995	133292239	1.42	2.63E-01	CDV3	-317		
chr11	65307652	65307968	0.70	2.63E-01	SCYL1	15262	LTBP3	17889
chr20	30628173	30628502	1.33	2.63E-01	HCK	-11719	XKR7	72533
chr19	41769355	41770068	0.64	2.63E-01	HNRNPUL1	-408		
chr10	70091408	70091922	0.72	2.63E-01	HNRNPH3	-103		
chr1	11969598	11970034	1.38	2.63E-01	NPPB	-50824	KIAA2013	16664
chr2	27304099	27304358	1.33	2.63E-01	KHK	-5382	EMILIN1	2723
chr16	28835318	28835753	1.23	2.63E-01	ATXN2L	1122	TUFM	22193
chr6	44064426	44064744	0.71	2.63E-01	MRPL14	30606	VEGFA	326632
chr18	12990958	12991268	0.66	2.63E-01	CEP192	-248		
chr3	169846336	169846693	1.39	2.63E-01	PHC3	53022	GPR160	90780
chr12	62996583	62996938	1.31	2.63E-01	MON2	136164	PPM1H	331904
chr3	15140551	15140829	1.33	2.63E-01	ZFYVE20	-35		
chr1	203734096	203734406	0.85	2.63E-01	LAX1	-33		
chr2	55920812	55921272	1.57	2.63E-01	PNPT1	-31		
chr3	42641778	42642097	1.31	2.63E-01	NKTR	-209		
chr15	45490702	45491038	1.33	2.63E-01	SHF	2503	DUOX1	68678
chr3	172468239	172468580	1.42	2.64E-01	ECT2	-3889		
chr1	222628509	222628767	1.45	2.64E-01	DUSP10	-713177	HHIPL2	92806
chr19	45681425	45681937	1.20	2.64E-01	BLOC1S3	-322	TRAPPC6A	-196
chr2	99797345	99797743	1.35	2.64E-01	MITD1	-52	MRPL30	-34
chr7	112580019	112580314	1.31	2.65E-01	TMEM168	-149689	GPR85	146375
chr7	129592502	129593063	1.18	2.65E-01	UBE2H	6		
chr4	378151	378725	0.68	2.65E-01	PIGG	-114551	ZNF141	46842
chr5	139944168	139944434	1.41	2.65E-01	SLC35A4	-119	APBB3	-112
chr7	65579686	65579934	0.82	2.65E-01	CRCP	5		
chr1	228290942	228291229	1.49	2.66E-01	MRPL55	5927	ARF1	20725

chr1	160001690	160001975	1.24	2.66E-01	PIGM	-50		
chr19	50919005	50919246	1.36	2.66E-01	SPIB	-3069		
chr6	31802210	31802820	0.77	2.66E-01	HSPA1B	7003	NEU1	28194
chr5	112312085	112312425	1.46	2.67E-01	DCP2	-178		
chr4	114468726	114469129	1.31	2.67E-01	CAMK2D	214155	ANK2	498143
chr14	90187142	90187542	0.70	2.67E-01	FOXN3	-101848	C14orf143	233747
chr6	133135307	133135690	0.73	2.67E-01	RPS12	-209		
chr7	11251802	11252087	0.67	2.67E-01	PHF14	238429	THSD7A	619879
chr11	96076387	96076697	1.40	2.68E-01	MAML2	-198		
chr4	159593138	159593792	1.37	2.68E-01	ETFDH	188		
chr1	16302485	16302785	1.37	2.68E-01	ZBTB17	-8		
chr12	54673825	54674135	1.40	2.68E-01	HNRNPA1	-508		
chr12	101673741	101673989	1.38	2.68E-01	UTP20	-40		
chr3	158362100	158362560	1.39	2.68E-01	GFM1	13		
chr4	113152520	113152774	1.36	2.68E-01	AP1AR	-248		
chr4	119512655	119512965	0.81	2.68E-01	METTL14	-93764	CEP170L	75315
chr13	31182068	31182364	1.48	2.69E-01	HMGB1	-142135	USPL1	-9614
chr17	56494892	56495216	1.20	2.69E-01	RNF43	-123		
chr18	47814018	47814493	1.45	2.70E-01	CXXC1	436		
chr5	37371067	37371320	1.31	2.70E-01	NUP155	3		
chr1	167684697	167684987	1.44	2.70E-01	CREG1	-161786	MPZL1	-6345
chr6	126102026	126102414	0.79	2.70E-01	NCOA7	-9781	HEY2	31488
chr19	13990971	13991363	1.32	2.70E-01	NANOS3	3217	C19orf57	25742
chr3	71493178	71493460	0.72	2.70E-01	FOXP1	139821		
chr1	244898763	244899096	0.73	2.70E-01	FAM36A	-99709	C1orf101	274257
chr2	158380286	158380613	0.80	2.70E-01	CYTIP	-79846	ACVR1C	104949
chr22	19406777	19407087	0.68	2.70E-01	CLTCL1	-127693	HIRA	12287
chr6	42738646	42739117	0.79	2.70E-01	RPL7L1	-108789	TBCC	-24998
chr1	245205998	245206308	1.47	2.70E-01	KIF26B	-112134	EFCAB2	72982
chr7	158387819	158388029	0.49	2.70E-01	PTPRN2	-7442	NCAPG2	109596
chr19	51307737	51308174	0.83	2.70E-01	ACPT	14284	KLK1	19087
chr17	46018622	46018979	0.86	2.71E-01	PNPO	-88		
chr21	35296373	35296665	0.66	2.71E-01	SLC5A3	-149304	ITSN1	281735
chr6	30689079	30689385	0.76	2.71E-01	MDC1	-3774	TUBB	1075
chr19	40476639	40477087	1.29	2.71E-01	PSMC4	-210		
chr6	1739923	1740219	1.44	2.71E-01	FOXC1	129390	GMDS	505775
chr9	136811592	136811845	1.52	2.71E-01	SARDH	-208240	VAV2	45727
chr11	114309944	114310189	1.32	2.71E-01	REXO2	-41		

chr4	146019082	146019533	1.27	2.71E-01	ANAPC10	60	ABCE1	152
chr11	67771552	67771796	1.42	2.72E-01	UNC93B1	-81		
chr2	55746558	55746883	1.22	2.72E-01	CCDC88A	-99664	SMEK2	98392
chr19	50934512	50934879	1.30	2.72E-01	MYBPC2	-1464		
chr19	50083729	50084086	1.40	2.72E-01	PRRG2	-679	NOSIP	-105
chr16	15949188	15949720	0.87	2.72E-01	MYH11	1433	NDE1	205371
chr11	67374227	67374514	1.31	2.72E-01	NDUFV1	48		
chr3	129143138	129143475	1.56	2.72E-01	H1FX	-108187	MBD4	15545
chr10	102241286	102241591	0.66	2.73E-01	WNT8B	18627	SEC31B	38156
chr17	7212213	7212545	0.73	2.73E-01	EIF5A	2061	GPS2	6595
chr8	38240334	38240927	0.84	2.73E-01	LETM2	-3389	WHSC1L1	-841
chr12	49208377	49208683	0.70	2.73E-01	CACNB3	-3982		
chr16	30483820	30484181	0.77	2.73E-01	ITGAL	18		
chr13	77913296	77913725	0.81	2.74E-01	SCEL	-196298	MYCBP2	-12334
chr22	23408742	23409009	1.49	2.75E-01	GNAZ	-3793		
chr6	43196933	43197360	0.75	2.75E-01	C6orf108	64		
chr19	48867103	48867893	1.39	2.75E-01	TMEM143	-312	SYNGR4	-159
chr12	104350850	104351449	1.37	2.75E-01	C12orf73	-157		
chr11	73000496	73000806	1.30	2.75E-01	ARHGEF17	-19012	P2RY6	17404
chr2	136996389	136996712	1.26	2.76E-01	THSD7B	-751911	CXCR4	-120826
chr20	10415129	10415380	1.19	2.76E-01	C20orf94	-696	MKKS	-389
chr7	106505687	106506015	0.84	2.76E-01	PIK3CG	-73		
chr5	10607763	10608307	1.22	2.76E-01	DAP	153352	ROPN1L	166026
chr8	145133322	145133632	1.45	2.76E-01	GPAA1	-4047	EXOSC4	-45
chrX	2439360	2439710	1.59	2.76E-01	CD99	-169693	ZBED1	-20520
chr19	52097551	52097955	1.19	2.76E-01	hCG_2008157	-123		
chr19	6587374	6587811	0.69	2.76E-01	CD70	3570	TNFSF9	56583
chr7	104624269	104624707	0.71	2.77E-01	MLL5	-30149	LHFPL3	655384
chr3	112361804	112362088	1.45	2.77E-01	CCDC80	-1969		
chr6	24522639	24523067	0.65	2.77E-01	ALDH5A1	27656	KIAA0319	123530
chr6	25992773	25993020	0.78	2.78E-01	HIST1H1A	25143	TRIM38	29826
chr16	89038229	89038523	1.35	2.78E-01	LOC390748	-105362	CBFA2T3	5025
chr17	4167086	4167566	0.81	2.78E-01	ANKFY1	-52		
chr14	76127436	76127682	1.24	2.78E-01	TTLL5	-62	C14orf1	-27
chr17	1990974	1991294	1.46	2.78E-01	HIC1	31530	SMG6	215935
chrX	102941505	102941904	1.34	2.78E-01	MORF4L2	1381	TCEAL1	57813
chr16	85752267	85752650	1.38	2.78E-01	GINS2	-29871	COX4NB	80689
chr11	65337422	65338041	1.47	2.78E-01	SSSCA1	-211		

chr1	165797311	165797621	0.81	2.79E-01	UCK2	576		
chr8	1840636	1840892	1.60	2.79E-01	KBTBD11	-81280	ARHGEF10	68615
chr1	182602970	182603248	1.18	2.79E-01	RGS16	-29561	RGS8	38958
chr13	46951686	46951996	1.44	2.79E-01	C13orf18	9794	LRRC63	165758
chr8	41402595	41402932	0.67	2.79E-01	AGPAT6	-32943	GINS4	16039
chr1	224180230	224180474	1.35	2.79E-01	DEGS1	-190576	TP53BP2	-146678
chr4	113208287	113208597	0.75	2.80E-01	TIFA	-1383		
chr5	154134404	154134809	0.71	2.80E-01	LARP1	-282		
chr6	153304437	153304757	1.21	2.80E-01	FBXO5	-394		
chr3	27410853	27411163	0.71	2.80E-01	NEK10	-96		
chr3	10431881	10432205	0.80	2.80E-01	SEC13	-69185	ATP2B2	115225
chr7	5258265	5258541	0.79	2.80E-01	SLC29A4	-64158	WIPI2	28568
chr1	203764539	203764830	1.34	2.80E-01	ZC3H11A	-66		
chr8	125551194	125551527	0.72	2.80E-01	TATDN1	-32	NDUFB9	18
chr10	112115795	112116079	1.38	2.81E-01	DUSP5	-141688	SMNDC1	-51230
chr1	165599940	165600339	1.48	2.81E-01	MGST3	-310		
chr11	118901492	118901895	1.34	2.81E-01	SLC37A4	-78		
chr1	156391377	156391713	1.17	2.81E-01	C1orf61	7795	RHBG	52542
chr8	103819759	103820027	0.93	2.81E-01	KLF10	-151910	AZIN1	56504
chr22	22711710	22712039	1.37	2.81E-01	VPREB1	112675	ZNF280B	151630
chr11	77705662	77706312	1.38	2.81E-01	INTS4	-270		
chr3	128995287	128995637	1.34	2.81E-01	COPG	27009	H1FX	39658
chr9	115983403	115983821	1.16	2.81E-01	SLC31A1	-196	FKBP15	64
chr6	159525193	159525433	0.65	2.81E-01	FNDC1	-65116	TAGAP	-59129
chr22	41682854	41683130	1.47	2.81E-01	RANGAP1	-776		
chr16	23345784	23346190	0.92	2.82E-01	SCNN1B	32396	COG7	118516
chr2	61108002	61108364	1.28	2.82E-01	REL	-569		
chr20	44718323	44718744	1.28	2.83E-01	NCOA5	46		
chr7	144532949	144533313	1.31	2.83E-01	TPK1	15		
chr3	32501621	32501963	0.72	2.83E-01	CMTM6	42611	CMTM7	68629
chr9	2242456	2242732	1.24	2.83E-01	VLDLR	-379199	SMARCA2	227252
chr2	68668812	68669146	1.42	2.83E-01	APLF	-25712	PLEK	76657
chr1	231664065	231664380	0.84	2.83E-01	TSNAX	-176		
chr5	139781279	139781616	1.23	2.83E-01	ANKHD1-EIF4EBP3	19		
chr2	10885743	10886021	1.45	2.83E-01	ATP6V1C2	24107	PDIA6	67078
chr11	47198356	47198751	1.20	2.84E-01	ARFGAP2	-135		
chr3	187960190	187960551	1.33	2.84E-01	TPRG1	-929392	LPP	29650
chr22	24256082	24256514	1.41	2.84E-01	BC036909	-15181	GSTT2	47095

chr6	36409218	36409564	0.74	2.84E-01	KCTD20	-1153				
chr12	53574198	53574458	1.38	2.85E-01	ZNF740	-207	CSAD	102		
chr8	67837592	67837890	0.66	2.85E-01	C8orf45	54757	LRRC67	103045		
chr20	56051219	56051491	0.79	2.85E-01	HMGB1L1	12728	RBM38	84892		
chr4	115524674	115524936	1.35	2.85E-01	UGT8	5194	NDST4	510227		
chr7	7680161	7680595	0.65	2.85E-01	COL28A1	-104918	RPA3	77860		
chr6	156885557	156885814	0.65	2.86E-01	ARID1B	-213400				
chr19	11545831	11546141	0.81	2.86E-01	PRKCSH	-283				
chr15	67385835	67386208	0.71	2.86E-01	SMAD3	27827	AAGAB	161052		
chr19	1258983	1259447	1.34	2.86E-01	CIRBP	-10052	MIDN	10663		
chr14	21457972	21458251	1.34	2.86E-01	METT11D1	147				
chr6	167527397	167527728	0.72	2.86E-01	CCR6	2268	GPR31	43756		
chr7	123197778	123198035	1.31	2.87E-01	NDUFA5	51				
chr16	89787213	89787491	1.42	2.87E-01	ZNF276	-600	C16orf7	42		
chr9	130829989	130830340	1.22	2.87E-01	NAIF1	-566	SLC25A25	-314		
chr12	14410571	14410990	1.36	2.87E-01	GRIN2B	-277759	ATF7IP	-107830		
chr6	10694734	10695242	0.71	2.88E-01	PAK1IP1	-200				
chr14	77787567	77787820	1.31	2.88E-01	POMT2	-469	GSTZ1	464		
chr16	11402721	11403050	1.21	2.88E-01	C16orf75	-36425	PRM1	-27694		
chr22	50946488	50946762	1.06	2.88E-01	LMF2	-490	NCAPH2	-20		
chr7	98976914	98977172	0.70	2.88E-01	ARPC1B	4714	PDAP1	29249		
chr2	62266496	62266783	1.53	2.88E-01	B3GNT2	-156622	COMMD1	133837		
chr2	241981203	241981513	0.76	2.88E-01	SNED1	43103	PASK	107520		
chr17	17739457	17739833	1.23	2.89E-01	SREBF1	680				
chr11	64889536	64889838	1.30	2.89E-01	ZNHIT2	-4517	MRPL49	-41	FAU	-27
chr3	58626403	58626651	0.65	2.89E-01	FAM107A	-63036	FAM3D	26034		
chr19	42787734	42788233	1.42	2.89E-01	CIC	-833				
chr2	47630155	47630465	1.41	2.89E-01	MSH2	47				
chr6	32098026	32098325	0.77	2.89E-01	ATF6B	-2159	FKBPL	-109		
chr8	28258590	28259008	1.17	2.89E-01	FZD3	-92974	ZNF395	-14822		
chr9	139131842	139132086	1.25	2.89E-01	LHX3	-36960	QSOX2	5723		
chr5	115420964	115421285	1.28	2.90E-01	COMMD10	398				
chr1	174998888	174999198	1.41	2.90E-01	TNN	-37951	MRPS14	-6482		
chr6	43635032	43635276	0.68	2.90E-01	MRPS18A	20374	RSPH9	22344		
chr1	156698481	156698773	1.56	2.91E-01	ISG20L2	-922	C1orf66	364		
chr11	63448726	63449017	1.24	2.91E-01	RTN3	-50				
chr17	7961104	7961388	1.37	2.91E-01	ALOX15B	18888	ALOX12B	29775		
chr16	81855119	81855465	0.73	2.91E-01	PLCG2	42362	SDR42E1	189801		

chr2	6483173	6483563	1.25	2.91E-01	CMPK2	522568	SOX11	650569
chr3	186524086	186524599	1.34	2.92E-01	RFC4	-53		
chr19	7587182	7587513	1.29	2.92E-01	MCOLN1	-164		
chr6	30028828	30029113	0.87	2.92E-01	ZNRD1	-65		
chr16	3208775	3209019	1.73	2.92E-01	OR1F1	-45350	caspace	14677
chr8	12622846	12623158	1.41	2.92E-01	C8orf79	-180181	LONRF1	-10010
chr9	93589455	93589790	1.51	2.92E-01	SYK	25611	AUH	534583
chr21	43483739	43484146	0.66	2.92E-01	ZNF295	-53447	UMODL1	-7483
chr13	25875595	25875893	1.38	2.92E-01	NUPL1	78		
chr22	46983602	46983912	1.29	2.92E-01	CELSR1	-50690	GRAMD4	-38891
chr18	52989000	52989236	0.76	2.93E-01	TCF4	266742	RAB27B	493278
chr16	80574675	80574919	1.33	2.93E-01	DYNLRB2	-57		
chr17	37950319	37950914	1.65	2.93E-01	GRB7	56430	IKZF3	69824
chr9	5438827	5439111	0.81	2.93E-01	C9orf46	-1109		
chr11	19454095	19454405	1.10	2.93E-01	NAV2	-280631	E2F8	-191743
chr10	12110619	12110924	1.19	2.93E-01	DHTKD1	-162		
chr13	74864521	74864856	0.83	2.93E-01	KLF12	-156623		
chr17	4736525	4736775	1.33	2.93E-01	MINK1	-33		
chr2	74681897	74682269	1.51	2.94E-01	WBP1	-3494	INO80B	-116
chr3	187954145	187954525	0.77	2.94E-01	TPRG1	-935428	LPP	23614
chr19	39935910	39936379	1.32	2.94E-01	SUPT5H	-41		
chr9	98224847	98225167	1.26	2.94E-01	FANCC	-145016	PTCH1	45824
chr2	9747966	9748378	1.45	2.94E-01	ADAM17	-52255	YWHAQ	22934
chr9	133454786	133455041	1.39	2.95E-01	FUBP3	-46		
chr15	91361868	91362184	1.22	2.95E-01	FURIN	-49859	BLM	101447
chr12	67835626	67835877	0.87	2.95E-01	DYRK2	-206760	CAND1	172691
chr13	45563493	45563810	1.47	2.95E-01	NUFIP1	-39		
chr15	64943784	64944060	0.66	2.95E-01	OAZ2	51540	ZNF609	152303
chr7	148638097	148638766	1.36	2.96E-01	EZH2	-57018	PDIA4	87350
chr9	34899625	34899869	0.69	2.96E-01	C9orf144B	-170212	KIAA1045	-58445
chr15	93374913	93375360	1.39	2.96E-01	FAM174B	-176106	CHD2	-68414
chr3	122512570	122512844	1.38	2.96E-01	DIRC2	-1194	HSPBAP1	-57
chr15	75081089	75081498	1.43	2.96E-01	LMAN1L	-23900	CSK	6869
chr20	30795520	30795764	1.09	2.97E-01	PLAGL2	-96	POFUT1	-54
chr11	33942800	33943168	1.32	2.97E-01	CAPRIN1	-130246	LMO2	-29148
chr1	22366310	22366642	0.70	2.97E-01	CDC42	-12644	CELA3B	63058
chr5	43008138	43008464	0.80	2.97E-01	C5orf39	32146	GHR	584275
chr12	93381773	93382085	1.20	2.98E-01	NUDT4	-389772	EEA1	-58822

chr3	183903412	183903899	1.30	2.98E-01	ABCF3	-207		
chr8	74888329	74888573	0.70	2.98E-01	TCEB1	-4105	TMEM70	21
chr7	100136287	100136808	1.31	2.98E-01	AGFG2	-286		
chr15	57998684	57999026	1.28	2.98E-01	GCOM1	114741	ALDH1A2	359051
chr15	67373166	67373811	1.36	2.98E-01	SMAD3	15294	AAGAB	173585
chr22	43263735	43264041	1.22	2.99E-01	ARFGAP3	-10480	PACSIN2	79159
chr10	6205504	6205910	1.07	2.99E-01	PFKFB3	-39133	RBM17	74758
chr15	90590410	90590655	1.30	2.99E-01	ZNF710	45781	IDH2	55175
chr19	45157725	45157973	1.32	2.99E-01	CEACAM19	-16875	PVR	10751
chr2	238877293	238877606	1.21	2.99E-01	SCLY	-92182	UBE2F	1750
chr12	57852742	57853050	0.72	2.99E-01	GLI1	-1022		
chr4	40307149	40307646	1.41	2.99E-01	CHRNA9	-30071	RHOH	108871
chr4	6641943	6642395	1.28	2.99E-01	MRFAP1	-276		
chr9	131266802	131267052	1.20	2.99E-01	GLE1	-44		
chr6	31125943	31126435	0.73	3.00E-01	CCHCR1	-623	TCF19	-114
chr11	67252373	67252864	0.81	3.00E-01	AIP	2114	PITPNM1	20224
chr8	123799976	123800291	0.70	3.00E-01	ZHX2	6233	DERL1	254514
chr7	35734669	35734996	0.66	3.00E-01	HERPUD2	-61		
chr3	127293758	127294086	1.22	3.00E-01	TPRA1	15646	PLXNA1	586420
chr15	91565746	91566015	1.54	3.00E-01	VPS33B	-48		
chr17	79908480	79908891	1.53	3.01E-01	MYADML2	-3577		
chr3	14473425	14473669	1.30	3.01E-01	SLC6A6	29441	GRIP2	110041
chr15	68923925	68924245	1.12	3.01E-01	CORO2B	52512	ANP32A	189176
chr5	131746491	131746864	1.28	3.01E-01	SLC22A5	41277	IRF1	79787
chr8	28259254	28259623	1.18	3.01E-01	FZD3	-92334	ZNF395	-15462
chr6	26271365	26271728	0.80	3.01E-01	HIST1H2BI	-1657	HIST1H3G	65
chr10	11726748	11727088	0.79	3.01E-01	USP6NL	-152644	ECHDC3	-57438
chr4	141444947	141445392	1.25	3.01E-01	ELMOD2	-182		
chr3	128952397	128952740	1.29	3.01E-01	CNBP	-49759	COPG	-15884
chr19	13029993	13030363	1.23	3.01E-01	SYCE2	-92		
chr20	2854012	2854252	1.12	3.02E-01	PTPRA	-49721	VPS16	32759
chr13	34116578	34117079	1.41	3.02E-01	RFC3	-275377	STARD13	-256928
chr1	160162928	160163226	1.26	3.02E-01	PEA15	-12048	CASQ1	2792
chr16	17395493	17395913	1.19	3.02E-01	XYLT1	169035	AK310228	930706
chr20	60757864	60758209	1.40	3.02E-01	GTPBP5	-44		
chr12	108954860	108955178	0.79	3.02E-01	ISCU	-1275	SART3	146
chr16	88103073	88103404	1.27	3.02E-01	ZNF469	-390640	BANP	118201
chr17	41438383	41438824	1.54	3.02E-01	ARL4D	-37749	TMEM106A	74710

chr9	134000836	134001090	1.22	3.02E-01	NUP214	-18		
chr2	38760334	38760653	1.24	3.02E-01	ATL2	-156062	HNRPLL	69684
chr16	71842869	71843390	1.54	3.03E-01	AP1G1	-154		
chr19	49122393	49122920	1.31	3.03E-01	RPL18	-224	SPHK2	109
chr1	156182635	156182991	0.88	3.03E-01	PMF1	29		
chr16	87984449	87985119	1.36	3.03E-01	BANP	-254		
chr4	39460307	39460741	1.27	3.03E-01	RPL9	-317	LIAS	-141
chr19	19779094	19779504	0.70	3.04E-01	ATP13A1	-4796	ZNF101	-364
chr10	43951069	43951456	1.27	3.04E-01	ZNF487	18689	ZNF239	118803
chr5	176943893	176944176	1.29	3.04E-01	DDX41	-68		
chr1	212208770	212209207	1.28	3.04E-01	INTS7	-105	DTL	70
chr2	85843087	85843354	1.37	3.04E-01	USP39	-62		
chr1	219347011	219347610	1.37	3.04E-01	LYPLAL1	119		
chr6	149753150	149753460	0.79	3.05E-01	TFL	52725	MAP3K7IP2	114242
chr17	38083744	38084061	1.31	3.05E-01	ORMDL3	-49		
chr3	121977734	121978015	1.45	3.05E-01	CSTA	-66136	CASR	75345
chr7	102045044	102045354	0.85	3.05E-01	ORAI2	-28797	PRKRIP1	8395
chr19	10764381	10764852	1.51	3.05E-01	ILF3	-320		
chr6	139613101	139613528	0.78	3.06E-01	TXLNB	-107		
chr9	139685601	139685883	1.25	3.06E-01	TMEM141	-35		
chr1	169454901	169455343	0.76	3.06E-01	SLC19A2	86		
chr17	57970120	57970556	1.37	3.06E-01	RPS6KB1	-105	TUBD1	-42
chrX	52949852	52950260	1.00	3.06E-01	FAM156B	21971	FAM156B	35897
chr12	102224453	102224799	1.36	3.07E-01	GNPTAB	6		
chr4	108951559	108951893	1.37	3.07E-01	HADH	40786	LEF1	137852
chr20	44420291	44420660	1.38	3.07E-01	DNTTIP1	-100	WFDC3	71
chr21	46369742	46370018	1.31	3.08E-01	ADARB1	-124613	C21orf67	-10052
chr3	187459876	187460179	1.24	3.08E-01	RTP2	-39683	BCL6	3447
chr12	104323150	104323661	1.20	3.08E-01	HSP90B1	-783	GNN	583
chr15	59978582	59978864	1.40	3.08E-01	GTF2A2	-28986	BNIP2	2919
chr19	42924161	42924471	0.91	3.08E-01	CNFN	-29872	LIPE	7262
chr22	17700175	17700487	1.17	3.08E-01	CECR2	-256299	CECR1	-9552
chr19	19249230	19249496	1.36	3.08E-01	TMEM161A	-96		
chr1	45251818	45252088	0.63	3.08E-01	BEST4	1473	RPS8	10707
chr20	49416714	49417127	0.72	3.08E-01	BCAS4	5454	ADNP	130606
chr2	120517211	120517628	0.71	3.09E-01	PTPN4	213		
chr17	73780604	73780861	1.15	3.09E-01	H3F3B	-4873	UNK	52
chr3	186285087	186285430	1.31	3.09E-01	DNAJB11	-2693	TBCCD1	-118

chr14	68283325	68283626	1.32	3.09E-01	RAD51L1	-3033	ZFYVE26	-170
chr7	25109644	25109911	0.75	3.09E-01	OSBPL3	-90018	CYCS	55177
chr12	9800758	9801292	1.29	3.09E-01	KLRB1	-40528	CLEC2D	-21284
chr1	1838913	1839219	1.21	3.10E-01	GNB1	-16571	CALML6	-7200
chr9	126102467	126102792	0.76	3.10E-01	STRBP	-71787	CRB2	-15818
chr9	139780635	139780920	1.22	3.10E-01	TRAF2	-187		
chr6	29933768	29934076	0.71	3.10E-01	HLA-F	-40295	HLA-A	23591
chr11	118763180	118763456	1.49	3.11E-01	CXCR5	8777	BCL9L	18295
chr19	18518872	18519116	1.23	3.11E-01	SSBP4	-11227	LRRC25	-10579
chr21	33984777	33985169	0.74	3.11E-01	C21orf59	-307		
chr22	39715424	39715848	1.29	3.11E-01	RPL3	34		
chr17	77770866	77771111	1.13	3.11E-01	CBX8	-99		
chr8	101734629	101735069	0.69	3.11E-01	PABPC1	-534		
chr3	10266537	10266853	1.21	3.11E-01	TATDN2	-23482	IRAK2	60132
chr14	75937150	75937548	0.68	3.11E-01	BATF	-51435	JDP2	38512
chr21	45627301	45627611	0.78	3.11E-01	ICOSLG	33378	C21orf33	73962
chr13	80622989	80623250	1.67	3.12E-01	SPRY2	291966	NDFIP2	567861
chr19	6772434	6772840	1.38	3.12E-01	VAV1	-85		
chr19	40931562	40931918	1.22	3.12E-01	SERTAD1	192		
chr1	52000739	52000983	0.62	3.12E-01	OSBPL9	-81685	EPS15	-15866
chr19	2236249	2236507	1.22	3.12E-01	SF3A2	-438	PLEKHJ1	573
chr1	71546690	71547074	0.78	3.12E-01	ZRANB2	-137		
chr9	110045311	110045900	1.21	3.12E-01	RAD23B	62		
chr6	45649561	45649930	0.88	3.12E-01	SUPT3H	-304076	CLIC5	398339
chr16	27214639	27215326	1.33	3.13E-01	NSMCE1	65130		
chr8	142099590	142099840	0.73	3.13E-01	PTK2	-88383	SLC45A4	138958
chr6	52926559	52926803	0.82	3.13E-01	FBXO9	-3115	ICK	-81
chr7	50535752	50536056	0.82	3.13E-01	FIGNL1	-17816	DDC	92864
chr2	64834161	64834535	1.30	3.13E-01	SERTAD2	46698	AFTPH	82883
chr7	26416644	26416920	0.67	3.13E-01	SNX10	85267	SKAP2	487559
chr6	88411547	88412052	0.74	3.13E-01	AKIRIN2	185		
chr7	35839974	35840298	0.71	3.13E-01	SEPT7	-491		
chr12	116997284	116997528	1.33	3.13E-01	MAP1LC3B2	220		
chr11	59317921	59318231	1.39	3.13E-01	OR4D9	35690	OSBP	65541
chr13	34302926	34303253	1.53	3.13E-01	STARD13	-443189	RFC3	-89116
chr17	79791063	79791334	1.21	3.14E-01	P4HB	27345	GCGR	29173
chr15	74913404	74913699	1.27	3.14E-01	CLK3	6217	EDC3	74834
chrX	10087584	10087905	1.32	3.14E-01	CLCN4	-37240	SHROOM2	333249

chr19	42748861	42749187	1.32	3.14E-01	GSK3A	-2288		
chr9	114799771	114800324	0.89	3.14E-01	SUSD1	137508	UGCG	140842
chr11	33499809	33500165	0.77	3.14E-01	C11orf41	-63890	HIPK3	220819
chr2	166049760	166050105	1.24	3.14E-01	SLC38A11	-237898	SCN3A	10644
chr5	52095697	52095992	1.22	3.14E-01	ITGA2	-189311	ITGA1	12071
chr11	59034775	59035046	0.85	3.15E-01	OR5AN1	-97021	MPEG1	-54417
chr5	75698658	75699031	1.07	3.15E-01	IQGAP2	-304		
chr8	52920994	52921304	0.81	3.15E-01	PCMTD1	-109414	ST18	401290
chr6	31633441	31633885	0.81	3.16E-01	BAT4	-500	CSNK2B	6
chr12	12480750	12481026	1.64	3.16E-01	LRP6	-61077	MANSC1	22281
chr2	85044422	85044732	1.70	3.16E-01	C2orf89	63675	DNAH6	300998
chr12	122124850	122125362	1.44	3.16E-01	TMEM120B	-25552	ORAI1	60651
chr3	71470616	71470998	0.90	3.17E-01	FOXP1	162333		
chr18	56531940	56532260	1.32	3.17E-01	SEC11C	-275025	ZNF532	2039
chr6	10838686	10839102	1.23	3.17E-01	MAK	-7784	GCM2	43204
chr4	26863261	26863581	0.72	3.17E-01	STIM2	1057		
chr4	153457099	153457490	1.22	3.17E-01	FBXW7	-1110		
chr1	198567878	198568284	0.81	3.18E-01	ATP6V1G3	-58006	PTPRC	-40056
chr14	24657939	24658340	1.25	3.18E-01	IPO4	874		
chr7	105794351	105794627	0.80	3.18E-01	SYPL1	-41432	NAMPT	131149
chr10	6969559	6969867	0.79	3.18E-01	PRKCQ	-347475	SFMBT2	483737
chr5	55708915	55709248	1.33	3.19E-01	IL6ST	-418319	MAP3K1	-401818
chr15	90437131	90437454	1.31	3.19E-01	ANPEP	-79221	C15orf38	18929
chr17	3571927	3572171	1.14	3.19E-01	TAX1BP3	-76	TMEM93	-41
chr15	101142282	101142791	1.28	3.19E-01	ALDH1A3	-277472	LASS3	-57612
chr17	7738669	7739121	0.78	3.19E-01	KDM6B	-4340		
chr6	31632601	31632985	0.77	3.19E-01	CSNK2B	-864	BAT4	370
chr12	56435244	56435782	1.29	3.19E-01	RPS26	-173		
chr20	18477614	18478038	1.27	3.19E-01	RBBP9	61		
chr13	49343285	49343594	1.48	3.19E-01	FNDC3A	-206608	CYSLTR2	62487
chr14	20922950	20923416	0.77	3.19E-01	APEX1	-107	OSGEP	15
chr2	207630162	207630438	1.52	3.19E-01	MDH1B	-250	FASTKD2	188
chr11	103944680	103945114	0.90	3.19E-01	DDI1	37589	PDGFD	90130
chr5	96232584	96232857	1.74	3.19E-01	LNPEP	-61435	ERAP2	21077
chr20	47835734	47836015	1.21	3.20E-01	DDX27	43		
chr3	141632048	141632366	0.81	3.20E-01	ATP1B3	36737	TFDP2	92179
chr3	186287932	186288420	1.37	3.20E-01	TBCCD1	-3035	DNAJB11	224
chr20	30539751	30540137	1.35	3.20E-01	PDRG1	-61		

chr21	43527975	43528321	0.86	3.20E-01	ABCG1	-111119	UMODL1	36722		
chr8	42660420	42660709	0.82	3.20E-01	CHRNA6	-36946	THAP1	37909		
chr19	56165022	56165601	1.30	3.20E-01	U2AF2	-104				
chr22	36925008	36925293	1.32	3.20E-01	EIF3D	126				
chr1	235491615	235492383	1.42	3.20E-01	ARID4B	-467	GGPS1	246		
chr9	91933263	91933508	1.33	3.20E-01	SECISBP2	-26				
chr15	45492429	45492857	1.39	3.21E-01	SHF	730				
chr11	66610765	66611016	1.28	3.21E-01	RCE1	8				
chrX	47420216	47420533	0.81	3.21E-01	ARAF	-203				
chr19	41955260	41955606	1.27	3.21E-01	CEACAM21	-127098	ATP5SL	-9623		
chr1	53480436	53480757	0.76	3.21E-01	PODN	-47288	SCP2	87649		
chr6	33553480	33553800	0.75	3.22E-01	ITPR3	-35521	BAK1	-5570		
chr6	43682716	43683086	1.57	3.22E-01	VEGFA	-55052	MRPS18A	-27373		
chr12	12867124	12867402	1.25	3.22E-01	CDKN1B	-3039				
chr8	26435299	26435609	0.88	3.23E-01	DPYSL2	33				
chr19	16606802	16607178	0.84	3.23E-01	CALR3	13				
chr22	24830575	24830886	1.32	3.23E-01	UPB1	-59346	ADORA2A	2912		
chr19	10349877	10350149	0.81	3.24E-01	MRPL4	-12627	S1PR2	-8065		
chr1	234908153	234908397	1.22	3.24E-01	IRF2BP2	-163004	TOMM20	383981		
chr6	74230610	74231110	1.41	3.24E-01	EEF1A1	-105				
chr15	65588047	65588663	1.47	3.24E-01	PARP16	-9337	IGDCC3	82023		
chr3	133167398	133167760	1.24	3.24E-01	CDV3	-124855	BFSP2	48789		
chr14	81407949	81408355	0.96	3.24E-01	DIO2	-729627	TSHR	-13717		
chr13	45915252	45915622	1.27	3.25E-01	TPT1	-140				
chr11	72145180	72145451	0.70	3.25E-01	CLPB	252				
chr15	56757197	56757723	0.85	3.25E-01	MNS1	-125				
chr20	35470399	35470709	1.46	3.25E-01	DSN1	-68400	SAMHD1	109622		
chr6	100016477	100016793	0.79	3.25E-01	CCNC	55				
chr7	92157848	92158228	0.74	3.25E-01	PEX1	-193				
chr6	37400728	37401124	0.74	3.25E-01	FTSJD2	19				
chr12	69979172	69979487	0.77	3.26E-01	CCT2	122				
chr5	156998277	156998587	0.85	3.26E-01	ADAM19	4336	NIPAL4	111405		
chr11	65626989	65627233	1.29	3.26E-01	CFL1	-1010	CFL1	-1010	MUS81	-761
chr11	125495611	125495971	1.34	3.26E-01	CHEK1	-521				
chr17	40976074	40976462	1.39	3.26E-01	BECN1	42				
chr11	65819545	65819855	1.19	3.26E-01	GAL3ST3	-3049	SF3B2	-116		
chr1	180471888	180472198	0.90	3.27E-01	ACBD6	-21				
chr20	48227688	48228024	1.27	3.27E-01	PTGIS	-43149	B4GALT5	102565		

chr6	148606	148970	1.50	3.27E-01	DUSP22	-143313		
chr5	123985180	123985739	1.51	3.27E-01	ZNF608	95405		
chr8	60969399	60969740	0.94	3.28E-01	TOX	-937803	CA8	224384
chr1	36653796	36654216	0.75	3.28E-01	THRAP3	-36011	MAP7D1	32203
chr1	161171936	161172394	1.30	3.28E-01	ADAMTS4	-3320	NDUFS2	3060
chr11	65848987	65849297	0.77	3.28E-01	KLC2	-176032	PACS1	11318
chr1	28655354	28655677	1.36	3.28E-01	MED18	3		
chr6	36853558	36853965	0.85	3.28E-01	C6orf89	122		
chr11	111749720	111750265	1.38	3.28E-01	C11orf1	45	FDXACB1	160
chr9	101984202	101984564	0.86	3.28E-01	SEC61B	-187	ALG2	-137
chr17	8054467	8054722	1.24	3.29E-01	HES7	-27193	PER1	1158
chr19	49468297	49468924	1.26	3.29E-01	FTL	45		
chrX	15353717	15354028	1.31	3.29E-01	PIGA	-213		
chr2	190627269	190627579	0.84	3.29E-01	OSGEPL1	500		
chr6	33029803	33030084	0.81	3.29E-01	HLA-DOA	-52555	HLA-DPA1	11605
chrX	21857592	21857838	1.34	3.30E-01	MBTPS2	59		
chr3	188471082	188471527	1.51	3.30E-01	TPRG1	-418458	LPP	540584
chr8	128747430	128747979	0.82	3.30E-01	MYC	-610		
chr21	40684313	40684590	1.04	3.30E-01	PSMG1	-129012	BRWD1	1104
chr16	14448529	14449128	1.43	3.30E-01	PARN	275295	MKL2	283633
chr7	99966855	99967100	0.81	3.31E-01	PILRA	-4090		
chr11	62623201	62623719	1.38	3.31E-01	SLC3A2	-58		
chr20	32580616	32581065	0.82	3.31E-01	RALY	-891		
chr18	43753753	43754116	1.35	3.32E-01	RNF165	-160252	HAUS1	69637
chr13	24789402	24789681	1.45	3.32E-01	C1QTNF9	-94174	SPATA13	235703
chr2	73512272	73512661	0.76	3.32E-01	FBXO41	-14424	EGR4	8362
chr14	23398703	23399065	1.35	3.33E-01	PRMT5	-223		
chr19	54640687	54640996	1.11	3.33E-01	CNOT3	-607		
chr22	23270917	23271273	1.42	3.33E-01	GNAZ	-141574	IGL@	41135
chr12	26309143	26309646	1.36	3.33E-01	SSPN	-39111	BHLHE41	-31392
chr4	667929	668292	1.18	3.33E-01	MYL5	-3600	ATP5I	11
chr1	167657202	167657478	1.31	3.33E-01	CREG1	-134284	MPZL1	-33847
chr9	134615441	134615715	1.11	3.33E-01	RAPGEF1	-30349	MED27	339675
chr16	75467282	75467530	1.28	3.33E-01	CFDP1	-19		
chr14	21151396	21151790	1.39	3.33E-01	OR6S1	-41743	RNASE4	-5339
chr19	10216696	10217087	0.74	3.33E-01	ANGPTL6	-3467	PPAN-P2RY11	-73
chrX	47052955	47053230	1.33	3.34E-01	PCTK1	-25022	UBA1	2894
chr1	44115520	44115858	0.72	3.34E-01	KDM4A	-108		

chr19	17357130	17357452	1.11	3.34E-01	NR2F6	-1140		
chr2	158300347	158300724	1.37	3.34E-01	CYTIP	68		
chr6	35227157	35227572	0.84	3.34E-01	ZNF76	-145		
chr2	46727343	46727590	1.29	3.34E-01	ATP6V1E2	19629	LOC388946	20763
chr2	96814196	96814636	1.21	3.34E-01	DUSP2	-3237		
chr19	2156037	2156281	0.64	3.34E-01	AP3D1	-4603		
chr20	35402071	35402326	1.26	3.34E-01	DSN1	-45		
chr2	65833295	65833694	1.42	3.34E-01	MEIS1	-829037	SPRED2	-173839
chr15	41061833	41062234	0.72	3.34E-01	C15orf62	-125		
chr11	118230087	118230509	1.47	3.34E-01	UBE4A	-4		
chr6	90081755	90082065	0.71	3.34E-01	UBE2J1	-19291	RRAGD	40085
chr1	150601602	150601883	1.26	3.34E-01	ENSA	355		
chr3	188207354	188207688	0.82	3.34E-01	TPRG1	-682242	LPP	276800
chr1	22109906	22110303	1.26	3.34E-01	USP48	-417		
chr16	86588729	86588973	1.40	3.35E-01	MTHFSD	-10		
chr7	101499799	101500071	0.87	3.35E-01	SH2B2	-428470	CUX1	39053
chr14	75229815	75230126	1.30	3.35E-01	YLPM1	-98		
chr1	68150409	68151046	0.64	3.36E-01	GADD45A	-155		
chr14	60797883	60798472	0.85	3.36E-01	PPM1A	85708	C14orf39	154586
chr1	179334789	179335280	0.88	3.36E-01	SOAT1	72018	NPHS2	210049
chr11	67414082	67414448	0.86	3.36E-01	TBX10	-7234	ACY3	3865
chr6	34023081	34023348	0.82	3.36E-01	MLN	-251422	GRM4	78421
chr12	46603752	46604020	1.23	3.36E-01	SFRS2IP	-219485	SLC38A1	59322
chrX	153763052	153763372	1.05	3.37E-01	FAM3A	-18839	IKBKG	-7247
chr1	167586753	167587072	1.29	3.37E-01	MPZL1	-104274	CREG1	-63857
chr14	93170731	93170975	1.50	3.37E-01	LGMN	44159	RIN3	190728
chr19	3505897	3506208	1.19	3.37E-01	FZR1	-16901	DOHH	-5432
chr21	38738820	38739129	1.32	3.37E-01	DSCR3	-99142	DYRK1A	-53626
chr7	65215837	65216095	0.69	3.37E-01	VKORC1L1	-122291	ZNF92	377198
chr1	145610733	145611047	1.46	3.37E-01	RNF115	-146	POLR3C	-6
chr22	24951779	24952024	1.33	3.38E-01	SNRPD3	284		
chr1	9777631	9778153	1.19	3.38E-01	PIK3CD	66102	CLSTN1	106658
chr9	140121983	140122294	1.34	3.38E-01	SLC34A3	-3246		
chr15	101603037	101603292	1.41	3.38E-01	LRRK1	143705	CHSY1	188961
chr6	15299811	15300121	1.31	3.38E-01	JARID2	53439	DTNBP1	363305
chr4	1717204	1717460	1.31	3.38E-01	SLBP	-3302		
chrX	1511031	1511404	1.32	3.38E-01	SLC25A6	-220		
chrX	48433155	48433399	1.37	3.38E-01	RBM3	441		

chr10	14650452	14650728	0.67	3.38E-01	FRMD4A	-277724	CDNF	229393		
chr12	112125146	112125397	0.69	3.38E-01	BRAP	-1482	ACAD10	1415		
chr19	55574460	55574748	1.45	3.38E-01	RDH13	-19				
chr22	22777111	22777352	1.21	3.39E-01	ZNF280B	86273	VPREB1	178032		
chr8	22461936	22462268	1.32	3.39E-01	KIAA1967	-155				
chr1	193028415	193028761	1.39	3.39E-01	TROVE2	-369	UCHL5	-67	UCHL5	601
chr13	41706905	41707175	1.31	3.39E-01	KBTBD6	-104				
chr17	33288399	33288739	1.26	3.39E-01	CCT6B	-63	ZNF830	20		
chr7	100926323	100926570	0.73	3.39E-01	FIS1	-38076	RABL5	38646		
chr1	2231876	2232267	1.10	3.39E-01	RER1	-91142	SKI	71938		
chr10	60028623	60028947	1.13	3.39E-01	IPMK	-1091	CISD1	-110		
chr21	45225375	45225685	1.26	3.40E-01	AGPAT3	-59586	RRP1	16112		
chr22	21356375	21356685	1.46	3.40E-01	THAP7	-126				
chr19	35626321	35626674	1.34	3.40E-01	FXYD1	-3234	LGI4	-320		
chr6	27114744	27115134	1.44	3.41E-01	HIST1H2BK	-320	HIST1H2AH	31		
chr6	88032120	88032491	0.82	3.41E-01	C6orf162	0				
chr5	149867314	149867655	0.71	3.41E-01	RPS14	-38166	NDST1	-20189		
chr16	2390773	2391070	1.15	3.41E-01	ABCA3	-175				
chr5	43042684	43042984	0.71	3.41E-01	C5orf39	-2387				
chrX	14047973	14048255	1.29	3.41E-01	GEMIN8	-79				
chr13	20437565	20437851	1.10	3.41E-01	ZMYM5	68				
chr20	35233797	35234133	1.28	3.41E-01	TGIF2	31955	SLA2	40593		
chr5	118611435	118611887	0.81	3.41E-01	TNFAIP8	-79935				
chr2	100758830	100759287	1.19	3.41E-01	AFF3	-37014	LONRF2	180136		
chr1	100816867	100817177	0.77	3.42E-01	CDC14A	-1001				
chr3	10028407	10028677	1.29	3.42E-01	TMEM111	-20				
chr3	196295162	196295707	1.23	3.42E-01	FBXO45	-290				
chr22	42332247	42332706	1.36	3.42E-01	TNFRSF13C	-9656	CENPM	10671		
chr11	47574699	47575009	0.75	3.42E-01	CUGBP1	-64278	PTPMT1	-12128		
chr11	67276043	67276307	1.39	3.42E-01	PITPNM1	-3332	CDK2AP2	-73		
chr7	132766840	132767162	1.31	3.42E-01	CHCHD3	-173				
chr2	98262242	98262689	1.22	3.42E-01	COX5B	-55				
chr7	64254610	64255014	0.78	3.42E-01	ZNF138	41				
chr6	136571442	136571846	0.78	3.42E-01	BCLAF1	39345	PDE7B	398810		
chr19	1266197	1266590	0.71	3.42E-01	CIRBP	-2873				
chr19	907328	907688	1.27	3.42E-01	MED16	-14290	C19orf22	5717		
chr9	123555590	123555871	1.31	3.43E-01	FBXW2	9				
chr9	99179599	99179839	1.22	3.43E-01	ZNF367	950				

chr15	55489124	55489479	1.28	3.43E-01	RSL24D1	-71		
chr9	139972435	139972683	1.18	3.43E-01	UAP1L1	606		
chr2	71295135	71295901	1.30	3.44E-01	NAGK	110		
chr2	201936164	201936512	0.86	3.44E-01	NDUFB3	-124	FAM126B	54
chr4	26321191	26321478	1.28	3.45E-01	RBPJ	-1113		
chr6	14109241	14109540	1.28	3.45E-01	CD83	-8474	RNF182	184188
chr11	65255090	65255614	0.82	3.45E-01	SCYL1	-37196	FRMD8	101311
chr1	155904081	155904391	1.47	3.45E-01	RIT1	-23059	RXFP4	-7244
chr17	47817105	47817490	0.70	3.45E-01	MYST2	-48773	SLC35B1	-32016
chr19	47747476	47747839	1.36	3.46E-01	PRR24	-30484	BBC3	-11635
chr19	14491919	14492229	1.31	3.46E-01	CD97	-139		
chr17	29814652	29814899	0.64	3.46E-01	RAB11FIP4	96134	C17orf79	371550
chr12	75905280	75905615	1.27	3.46E-01	KRR1	-30		
chr2	102333548	102333835	0.91	3.46E-01	IL1R2	-274614	MAP4K4	19204
chr18	47839099	47839436	1.45	3.47E-01	SKA1	-62124	CXXC1	-24576
chrX	23801094	23801378	1.32	3.47E-01	SAT1	-39		
chr2	33701577	33701823	1.26	3.47E-01	RASGRP3	-37242	LTBP1	529308
chr3	178865719	178865987	1.34	3.47E-01	PIK3CA	-458		
chr5	179402190	179402544	1.28	3.47E-01	TBC1D9B	-67511	RNF130	96742
chr6	25018245	25018521	0.69	3.47E-01	FAM65B	-107188	DKFZp686H12134	119668
chr7	44835677	44836385	0.76	3.47E-01	PPIA	-210		
chr2	11890554	11890854	1.20	3.47E-01	TRIB2	-966294	LPIN1	3964
chr19	16696683	16696996	1.22	3.48E-01	SLC35E1	-13647	MED26	42175
chr11	3818918	3819267	1.18	3.48E-01	NUP98	-201		
chr1	42107189	42107533	0.68	3.48E-01	EDN2	-157017	HIVEP3	277135
chr19	7968374	7968884	1.36	3.48E-01	MAP2K7	-136		
chr9	140095126	140095548	1.25	3.48E-01	NDOR1	-4782	C9orf75	-357
chr1	165362190	165362526	1.35	3.48E-01	LMX1A	-37341	RXRG	52072
chr19	39322199	39322627	1.36	3.49E-01	LGALS4	-18673	HNRNPL	18204
chr7	69029882	69030244	0.70	3.49E-01				
chr2	677256	677610	1.27	3.49E-01	TMEM18	6		
chr5	133268371	133268852	0.71	3.50E-01	FSTL4	-320389	C5orf15	35794
chr16	2570210	2570510	1.49	3.50E-01	AMDHD2	-3		
chr12	31226813	31227229	0.75	3.50E-01	DDX11	242		
chr17	56595446	56595875	0.79	3.50E-01	MTMR4	-410		
chr7	76822336	76822581	1.36	3.50E-01	FGL2	6691	UPK3B	682714
chrX	134654414	134654658	1.22	3.51E-01	ZNF449	175840	MMGT1	401598
chr6	11382352	11382729	0.82	3.51E-01	TMEM170B	-155970	NEDD9	-149626

chr2	197104946	197105241	1.33	3.51E-01	STK17B	-68758	HECW2	352241
chr19	6199418	6199910	1.26	3.51E-01	ACSBG2	63954	MLLT1	80295
chr3	141193073	141193388	1.53	3.51E-01	RASA2	-12695	ZBTB38	150176
chr19	55765202	55765787	1.31	3.51E-01	TMEM86B	-24863	SAPS1	4543
chr19	10346739	10347145	0.83	3.51E-01	S1PR2	-4994		
chr11	64899732	64900260	1.36	3.51E-01	SYVN1	2007	MRPL49	10268
chrX	48456405	48456686	1.25	3.51E-01	WDR13	645		
chr20	5509572	5509841	1.29	3.51E-01	PROKR2	-214684	RP5-1022P6.2	81965
chr15	85923472	85923832	1.33	3.51E-01	AKAP13	-219		
chr14	23057912	23058424	1.24	3.52E-01	DAD1	-25		
chr2	219536549	219536950	0.81	3.52E-01	STK36	-12	RNF25	31
chr11	65420142	65420626	1.36	3.52E-01	RELA	10059	SIPA1	14806
chr7	75495772	75496078	0.81	3.52E-01	CCL24	-52833	RHBDD2	-12392
chr12	111099317	111099627	0.84	3.52E-01	HVCN1	27474	TCTN1	47592
chr4	86646892	86647232	0.72	3.52E-01	ARHGAP24	250778	MAPK10	628735
chr11	67396739	67397109	1.22	3.53E-01	NUDT8	477		
chr5	111093810	111094060	1.23	3.53E-01	STARD4	-245778	C5orf13	218693
chr5	544079	544510	0.76	3.53E-01	CEP72	-68110	SLC9A3	-19746
chr1	151042738	151043296	0.76	3.53E-01	GABPB2	-63		
chr19	50844462	50844772	1.48	3.53E-01	KCNC3	-11983	NAPSB	3388
chr1	150898612	150898886	1.26	3.53E-01	SETDB1	-66		
chr10	70939577	70940108	0.78	3.53E-01	SUPV3L1	-150		
chr19	4457736	4458057	1.32	3.53E-01	UBXN6	-107		
chr3	15247397	15247806	1.18	3.53E-01	CAPN7	-131		
chr19	6292108	6292418	0.85	3.53E-01	MLLT1	-12304	ACER1	41377
chrX	70288248	70288683	1.35	3.53E-01	SNX12	-235		
chr14	93260442	93260862	0.83	3.54E-01	GOLGA5	2		
chr3	195823285	195823767	1.18	3.54E-01	TFRC	-14494	ZDHHC19	114774
chr2	42981140	42981557	0.98	3.54E-01	OXER1	10052	MTA3	185678
chr18	48343217	48343533	1.30	3.54E-01	MRO	3059	MAPK4	256891
chr1	153606340	153606584	1.31	3.54E-01	C1orf77	-63		
chr12	57824687	57825032	1.27	3.54E-01	INHBC	-3683		
chr12	48173164	48173408	0.72	3.54E-01	SLC48A1	6319	HDAC7	40477
chr17	7210319	7210702	1.35	3.55E-01	EIF5A	193		
chr19	2622519	2622982	0.84	3.55E-01	GNG7	79995	GADD45B	146616
chr6	11537778	11538039	0.77	3.55E-01	TMEM170B	-602		
chr15	63484194	63484490	0.86	3.55E-01	APH1B	-85407	RAB8B	2614
chr15	52263733	52264249	1.29	3.56E-01	LEO1	-33		

chr16	89555560	89555825	1.36	3.56E-01	ANKRD11	1276	ZNF778	271582
chr19	38419150	38419472	1.41	3.56E-01	SIPA1L3	21443	DPF1	295579
chr11	77850603	77850896	1.23	3.56E-01	ALG8	-51		
chr7	22491814	22492124	0.80	3.56E-01	RAPGEF5	-95436	MGC87042	47829
chr12	133613759	133614238	1.16	3.56E-01	ZNF84	19		
chr11	67056388	67056754	1.17	3.57E-01	SSH3	-14348	ADRBK1	22666
chr1	161369378	161369663	1.28	3.57E-01	FCGR2A	-105684	SDHC	85355
chr11	78285789	78286033	1.15	3.57E-01	NARS2	-2		
chr5	218141	218480	0.99	3.57E-01	SDHA	-45		
chr13	50019227	50019512	1.44	3.57E-01	CAB39L	-43635	SETDB2	-6319
chr18	30383531	30383952	0.81	3.57E-01	ASXL3	-774799	KLHL14	-30768
chr4	84376927	84377329	1.30	3.57E-01	HELQ	-103	MRPS18C	10
chr9	100700480	100700790	0.75	3.57E-01	HEMGN	-109		
chr9	100772848	100773124	1.22	3.57E-01	NANS	-45696	ANP32B	27497
chr9	112680650	112681011	1.48	3.57E-01	PALM2-AKAP2	138254	TXN	337947
chr6	42896812	42897386	0.77	3.57E-01	CNPY3	239		
chr12	6602741	6603203	0.74	3.57E-01	MRPL51	-501	NCAPD2	-326
chr6	47210181	47210515	1.27	3.57E-01	GPR110	-200266	TNFRSF21	67332
chr12	29534052	29534410	1.26	3.57E-01	ERGIC2	-88		
chr1	23881246	23881498	1.22	3.58E-01	E2F2	-23659	ID3	4950
chr20	3801294	3801604	1.22	3.58E-01	MAVS	-26000	CDC25B	25048
chr17	26684403	26684748	1.28	3.58E-01	TMEM199	-111	POLDIP2	27
chr7	66119214	66119704	0.77	3.58E-01	RABGEF1	-85524	KCTD7	25569
chr1	150241314	150241873	1.32	3.58E-01	C1orf54	-3608	APH1A	-62
chr16	67596065	67596371	1.15	3.58E-01	CTCF	-246		
chr15	70307280	70307590	1.20	3.58E-01	TLE3	82821	RPLP1	562276
chr2	232479441	232479752	0.74	3.58E-01	PTMA	-93638	NMUR1	-84415
chr3	138198221	138198497	1.63	3.58E-01	ESYT3	44944	CEP70	114770
chr7	128337401	128337665	1.39	3.58E-01	CALU	-41813	METTL2B	220750
chr19	2042156	2042492	0.78	3.58E-01	BTBD2	-26622	MKNK2	8919
chr15	64386028	64386305	1.36	3.59E-01	SNX1	-2010		
chr19	38210355	38210999	0.86	3.59E-01	ZNF607	14		
chr19	5288138	5288426	0.64	3.59E-01	PTPRS	52532	KDM4B	319158
chr9	97443800	97444076	1.33	3.59E-01	C9orf3	-45056	FBP1	-42015
chr12	50898541	50898851	0.82	3.59E-01	DIP2B	-72		
chr2	25016143	25016437	1.20	3.59E-01	CENPO	-43	C2orf79	-39
chr3	193310818	193311137	0.82	3.60E-01	OPA1	45		
chr13	113951225	113951592	0.78	3.60E-01	LAMP1	-60		

chr19	55792388	55792628	1.27	3.60E-01	BRSK1	-3026	HSPBP1	-757
chr12	89740045	89740490	0.89	3.60E-01	KITLG	-766030	DUSP6	6028
chr9	37371010	37371615	0.77	3.61E-01	GRHPR	-51394	ZCCHC7	250844
chr17	73663038	73663496	1.19	3.61E-01	SAP30BP	-132	RECQL5	2
chr9	102861230	102861517	1.17	3.61E-01	INVS	-137	ERP44	-44
chr14	24900967	24901277	0.72	3.62E-01	CBLN3	-2391	KIAA0323	1981
chr6	149886759	149887019	0.62	3.62E-01	C6orf72	-639		
chr1	42216965	42217319	1.34	3.62E-01	EDN2	-266798	HIVEP3	167354
chr6	11461089	11461443	1.50	3.62E-01	NEDD9	-228351	TMEM170B	-77245
chr19	42772861	42773101	1.10	3.62E-01	CIC	-15836	ERF	-13672
chr9	86595474	86595881	1.30	3.62E-01	HNRNPK	-109	RMI1	41
chr1	39491672	39492092	0.68	3.62E-01	NDUFS5	-124		
chr7	21466703	21467070	0.73	3.62E-01	SP4	-802		
chr10	73454350	73454693	1.15	3.62E-01	RP11-472K8.2-001	25056	CDH23	297818
chr12	95968696	95968940	0.84	3.62E-01	USP44	-26198	NTN4	215718
chr5	150138336	150138764	1.37	3.62E-01	DCTN4	107		
chr17	37025432	37025830	0.81	3.62E-01	LASP1	-481	LASP1	-481
chr19	6590964	6591289	1.32	3.62E-01	CD70	36		
chr16	69788633	69789023	1.26	3.62E-01	NOB1	1		
chr8	128746245	128746510	0.78	3.62E-01	MYC	-1937		
chr11	44625725	44626037	0.73	3.62E-01	TSPAN18	-302077	CD82	38740
chr19	49298575	49299002	1.35	3.62E-01	BCAT2	15512	FGF21	39641
chr8	22431912	22432250	0.86	3.62E-01	PDLIM2	-5903	SORBS3	22830
chr16	11945249	11945591	1.24	3.62E-01	RSL1D1	22		
chr2	232571553	232572008	1.20	3.62E-01	PTMA	-1454		
chr17	80376354	80376615	1.22	3.62E-01	C17orf101	-23	HEXDC	233
chr8	143484554	143484863	1.19	3.63E-01	TSNARE1	-166		
chr1	25427911	25428237	0.77	3.63E-01	RUNX3	-171304	SYF2	130939
chr14	105512088	105512520	0.81	3.63E-01	CDCA4	-24879	GPR132	19450
chr3	71466178	71466525	0.81	3.63E-01	FOXP1	166788		
chr12	110841531	110842058	0.81	3.63E-01	ANAPC7	-260		
chr12	120638936	120639196	1.17	3.63E-01	RPLP0	-52		
chr7	150065737	150065988	1.35	3.63E-01	REPIN1	-16		
chr18	20811287	20811690	1.31	3.63E-01	CABLES1	95762	C18orf45	206436
chr11	102962726	102963073	1.26	3.63E-01	MMP13	-136438	DYNC2H1	-17260
chr15	34502098	34502527	1.24	3.63E-01	C15orf29	-16		
chr22	24830093	24830396	1.35	3.63E-01	UPB1	-59832	ADORA2A	2426
chr11	615819	616355	1.30	3.64E-01	IRF7	-88		

chr2	88899630	88899914	1.27	3.65E-01	EIF2AK3	27222	C2orf51	75603
chr4	71859003	71859424	1.05	3.65E-01	DCK	-51		
chr1	23921435	23921745	1.23	3.65E-01	RPL11	-96704	ID3	-35268
chr7	44254014	44254290	0.65	3.65E-01	YKT6	13574	CAMK2B	111078
chr12	329063	329324	0.70	3.65E-01	SLC6A12	-5823	SLC6A13	42808
chr19	11593401	11593683	0.91	3.65E-01	ELAVL3	-1739		
chr1	161391704	161392050	1.59	3.65E-01	FCGR2A	-83328	SDHC	107711
chr12	125093660	125093932	0.78	3.65E-01	NCOR2	-73639	SCARB1	254723
chr19	16698458	16698778	1.32	3.65E-01	SLC35E1	-15425	MED26	40397
chr7	39663070	39663347	0.70	3.65E-01	RALA	47		
chr14	77564300	77564631	1.28	3.66E-01	C14orf4	-69432	ZDHHC22	43668
chr7	158648957	158649372	1.20	3.66E-01	ESYT2	-26846	VIPR2	288484
chr2	233925821	233926227	0.80	3.66E-01	INPP5D	988		
chr9	134604425	134604669	1.38	3.67E-01	RAPGEF1	-19318	MED27	350706
chr4	140899797	140900113	1.65	3.67E-01	MAML3	175278	MGST2	313033
chr1	21620772	21621441	0.70	3.67E-01	ECE1	-4341		
chr17	34257439	34258064	0.88	3.67E-01	RDM1	28		
chr7	23510099	23510480	0.73	3.68E-01	IGF2BP3	-295		
chr10	94352546	94352943	1.26	3.68E-01	KIF11	-80		
chr10	102106695	102106950	1.29	3.69E-01	SCD	51		
chr17	78549180	78549451	1.32	3.69E-01	CHMP6	-416325	RPTOR	30691
chr22	50354247	50354546	0.98	3.69E-01	PIM3	254		
chr20	32398782	32399206	1.23	3.69E-01	CHMP4B	-125		
chr16	87417512	87417832	1.38	3.69E-01	FBXO31	-278		
chr2	26467505	26467741	0.93	3.69E-01	HADHA	-29	HADHB	7
chr1	170043754	170044186	1.04	3.69E-01	KIFAP3	-91		
chr17	685438	685730	1.33	3.69E-01	GLOD4	-13	RNMTL1	71
chr17	62097389	62097699	0.96	3.69E-01	ICAM2	450		
chr1	198591650	198592010	1.46	3.70E-01	ATP6V1G3	-81755	PTPRC	-16307
chr12	97300977	97301272	1.36	3.70E-01	NEDD1	124		
chr19	10426526	10426865	1.28	3.70E-01	FDX1L	-5		
chr12	53902386	53902645	0.75	3.70E-01	NPFF	-1094		
chr1	203290056	203290472	1.25	3.71E-01	BTG2	15600	FMOD	30025
chr20	48286195	48286521	1.38	3.71E-01	PTGIS	-101651	B4GALT5	44063
chr16	84548400	84548868	0.90	3.71E-01	KIAA1609	-10346	COTL1	103035
chr1	52831773	52832067	1.22	3.71E-01	ORC1L	38211	ZFYVE9	223874
chr7	139875019	139875456	1.42	3.72E-01	PARP12	-111717	JHDM1D	1503
chr7	23053538	23053947	0.61	3.72E-01	FAM126A	27		

chr2	217363387	217363699	1.20	3.72E-01	RPL37A	23		
chr22	31886447	31886825	1.32	3.72E-01	EIF4ENIF1	-932		
chr6	30655128	30655424	0.91	3.72E-01	KIAA1949	-183		
chr14	102240584	102241029	1.05	3.72E-01	DYNC1H1	-190058	PPP2R5C	12672
chr11	63949046	63949316	1.13	3.72E-01	STIP1	-4406		
chr6	33538888	33539352	0.88	3.72E-01	BAK1	8950	ZBTB9	116764
chr15	75397247	75397599	1.39	3.72E-01	PPCDC	81496	DNM1P33	197883
chr22	43011192	43011581	1.44	3.73E-01	POLDIP3	-425		
chr4	28618	29005	0.81	3.74E-01	ZNF718	-24415		
chr15	41099598	41099942	1.35	3.74E-01	DNAJC17	-115	ZFYVE19	496
chr3	191759955	191760273	1.47	3.74E-01	FGF12	366724	PYDC2	581162
chr5	149815599	149815956	0.77	3.74E-01	CD74	-23446	RPS14	13541
chr20	45825078	45825396	1.24	3.74E-01	ZMYND8	160237	EYA2	301974
chr19	41814408	41814718	1.31	3.74E-01	HNRNPUL1	44443	TGFB1	45253
chr17	28049726	28050008	1.33	3.74E-01	GIT1	-133257	SSH2	207151
chr3	13057876	13058186	1.09	3.75E-01	RPL32	-176082	IQSEC1	56586
chr7	73620965	73621201	0.71	3.75E-01	LAT2	-3004		
chr1	53102745	53103387	1.38	3.75E-01	FAM159A	4000	C1orf163	60972
chr19	8386134	8386446	1.22	3.75E-01	RPS28	-94	NDUFA7	-10
chr15	66446051	66446663	1.13	3.75E-01	MEGF11	99718	RAB11A	284561
chr7	45151161	45151423	0.86	3.75E-01	TBRG4	25		
chr9	35636050	35636454	0.78	3.75E-01	CD72	-17828	SIT1	14695
chr11	59578141	59578451	1.42	3.77E-01	MRPL16	49		
chr2	8681351	8681680	1.27	3.77E-01	ID2	-140468		
chr17	78428338	78428715	1.16	3.77E-01	NPTX1	21877	FLJ35220	39560
chr22	26824782	26825129	1.26	3.77E-01	ASPHD2	-324		
chrX	18692985	18693273	1.28	3.78E-01	RS1	-2906		
chr1	205600238	205600589	1.46	3.78E-01	SLC45A3	49216	MFSD4	62302
chr14	32670279	32670615	1.25	3.78E-01	AKAP6	-128032	ARHGAP5	123952
chr12	96252490	96252855	1.15	3.78E-01	SNRPF	-36		
chr1	77684882	77685247	1.17	3.78E-01	PIGK	67		
chr17	41150134	41150576	1.34	3.78E-01	RPL27	-91		
chr1	203256447	203256950	1.53	3.78E-01	CHIT1	-57839	BTG2	-17965
chr12	124950695	124951096	0.70	3.78E-01	NCOR2	69261	ZNF664	493226
chr7	99063716	99064002	0.76	3.78E-01	ZNF789	-6656	CPSF4	27296
chr17	80250626	80250942	1.31	3.78E-01	CSNK1D	-19211	CD7	24696
chr3	186648892	186649202	0.88	3.78E-01	ST6GAL1	-90618	ADIPOQ	88584
chr16	23347300	23347582	1.29	3.78E-01	SCNN1B	33850	COG7	117062

chr12	48191291	48191567	1.11	3.79E-01	HDAC7	22334	SLC48A1	24462
chr12	92839591	92839928	1.32	3.79E-01	BTG1	-300087	PLEKHG7	-290505
chr5	118677417	118677734	1.31	3.79E-01	TNFAIP8	-14020		
chr19	1665437	1665748	1.23	3.79E-01	ONECUT3	-88069	TCF3	-15307
chr3	191848366	191848676	0.84	3.79E-01	FGF12	278317	PYDC2	669569
chr15	70393925	70394251	1.14	3.80E-01	TLE3	-3832		
chr17	45972982	45973527	1.18	3.80E-01	SP2	-261		
chr2	161263410	161263748	1.47	3.80E-01	ITGB6	-206990	RBMS1	86739
chr14	21081447	21081806	1.27	3.80E-01	RNASE12	-22645	OR6S1	28223
chr14	75741514	75741794	0.80	3.80E-01	FOS	-3827		
chr10	96122469	96122861	0.84	3.80E-01	NOC3L	18		
chr9	109515559	109515941	1.48	3.80E-01	ZNF462	-109628		
chrX	20160008	20160263	1.18	3.80E-01	EIF1AX	-170		
chr2	64441892	64442202	1.08	3.80E-01	HSPC159	-239280	PELI1	-70442
chr22	23284823	23285266	1.31	3.80E-01	GNAZ	-127624	IGL@	55085
chr16	3156438	3156828	1.22	3.81E-01	ZSCAN10	-13772	ZNF205	-5930
chr20	57582748	57583068	1.31	3.81E-01	CTSZ	-599		
chr10	106028481	106029031	0.81	3.81E-01	GSTO2	125		
chr10	14792953	14793315	1.14	3.81E-01	FRMD4A	-420268	CDNF	86849
chr11	122932698	122933137	0.77	3.81E-01	HSPA8	-74		
chr16	10970658	10971132	1.34	3.81E-01	CIITA	-160		
chr19	39826579	39826921	1.43	3.81E-01	GMFG	-24		
chr8	17780103	17780504	1.24	3.81E-01	PCM1	-62		
chr17	34891210	34891533	0.86	3.81E-01	MYO19	-67	PIGW	-31
chr7	90366626	90366933	0.79	3.81E-01	FZD1	-527003	PFTK1	28068
chr9	96031257	96031653	1.26	3.81E-01	FAM120A	-182718	WNK2	84243
chr17	74099792	74100042	1.23	3.81E-01	EXOC7	-49		
chr3	179280479	179281032	1.28	3.82E-01	ACTL6A	48		
chr11	46264801	46265139	1.08	3.82E-01	PHF21A	-121985	CREB3L1	-34258
chr17	29862771	29863074	1.23	3.82E-01	RAB11FIP4	144281	C17orf79	323403
chr12	120570576	120571038	1.32	3.82E-01	RAB35	-16208	GCN1L1	61706
chr12	104359432	104359856	1.32	3.82E-01	TDG	51		
chr7	117823956	117824330	1.17	3.82E-01	LSM8	57		
chr1	1710300	1710566	1.11	3.82E-01	NADK	-524		
chr16	27388993	27389321	0.85	3.82E-01	IL21R	-49422	IL4R	63906
chr2	223836474	223836732	1.35	3.82E-01	KCNE4	-80259	ACSL3	110871
chr17	43225769	43226087	0.73	3.82E-01	HEXIM2	-12336	HEXIM1	1244
chr7	99006014	99006735	0.76	3.83E-01	BUD31	-226	PDAP1	-83

chr12	56601784	56602094	0.72	3.83E-01	SMARCC2	-18588	RNF41	13765
chr7	100861273	100861533	1.19	3.83E-01	PLOD3	-392	ZNHIT1	418
chr2	74426156	74426466	0.75	3.83E-01	MTHFD2	621		
chr11	70283051	70283508	1.38	3.83E-01	CTTN	38668	SHANK2	652562
chr6	27655919	27656252	1.43	3.83E-01	ZNF184	-215189	HIST1H2BL	119623
chr20	49432211	49432472	0.80	3.83E-01	BCAS4	20875	ADNP	115185
chr5	134237935	134238247	0.79	3.83E-01	PCBD2	-2719		
chr3	16592360	16592604	1.27	3.83E-01	RFTN1	-37260	DAZL	54524
chr7	30325646	30325922	1.20	3.83E-01	ZNRF2	1861	NOD1	192609
chr1	150601895	150602255	1.17	3.84E-01	ENSA	23		
chr1	234748428	234748715	1.44	3.84E-01	IRF2BP2	-3301		
chr8	29387333	29387627	0.79	3.84E-01	DUSP4	-179295	TMEM66	553169
chr8	134156755	134157123	1.30	3.84E-01	SLA	-84336	WISP1	-46373
chr12	89919809	89920105	1.27	3.84E-01	GALNT4	-1422		
chr19	42724202	42724498	1.27	3.84E-01	DEDD2	-2537	ZNF526	-142
chr6	3259051	3259331	0.77	3.84E-01	TUBB2B	-31223	SLC22A23	197602
chr7	42951641	42952105	0.74	3.84E-01	C7orf25	278		
chr17	1933300	1933584	1.14	3.85E-01	DPH1	11		
chr5	140893350	140893922	0.80	3.85E-01	PCDHGC5	24828	DIAPH1	104986
chr6	42531423	42532025	0.77	3.85E-01	UBR2	-334		
chr6	14117634	14118089	0.82	3.85E-01	CD83	-3		
chr19	42829329	42829733	1.22	3.85E-01	MEGF8	-230		
chr19	6863562	6863895	1.16	3.86E-01	EMR1	-23853	VAV1	91007
chr7	6556014	6556280	0.65	3.86E-01	KDELR2	-32298	GRID2IP	34920
chr2	9816057	9816307	0.74	3.86E-01	TAF1B	-167389	YWHAQ	-45076
chr22	38240258	38240801	1.15	3.86E-01	EIF3L	-4840	ANKRD54	-227
chr13	27936164	27936408	1.18	3.87E-01	GTF3A	-62395	RASL11A	91822
chr11	6255683	6256130	0.68	3.87E-01	CNGA4	-4423	FAM160A2	34
chr2	136893730	136894162	0.77	3.87E-01	THSD7B	-854516	CXCR4	-18221
chr6	126277738	126278067	0.81	3.88E-01	HINT3	42		
chr3	101292593	101293013	1.32	3.88E-01	PCNP	-239		
chr15	79053714	79054186	0.86	3.88E-01	CHRNB4	-120363	ADAMTS7	49823
chr3	128879726	128880056	1.38	3.88E-01	ISY1	138		
chr12	8085883	8086202	0.78	3.89E-01	SLC2A3	2849	NANOGP1	60509
chr3	193855471	193855760	1.23	3.89E-01	HES1	1682	CPN2	216441
chr12	132568527	132568986	1.21	3.89E-01	DDX51	60123	EP400	134249
chrX	71401355	71401665	0.90	3.89E-01	PIN4	-16		
chr12	113667612	113667889	1.37	3.89E-01	TPCN1	8491	SLC24A6	105174

chr17	41116380	41116631	1.25	3.89E-01	AARSD1	16039	G6PC	63691
chr19	1812929	1813239	0.73	3.89E-01	ATP8B3	-814		
chr11	1858984	1859260	0.84	3.89E-01	TNNI2	-2310		
chr15	90728381	90728691	0.76	3.89E-01	IDH2	-82828	SEMA4B	-16026
chr7	22122531	22122841	0.70	3.89E-01	CDCA7L	-137144	RAPGEF5	273847
chr11	60609385	60609662	1.17	3.89E-01	CCDC86	95		
chr5	17282523	17282781	0.92	3.90E-01	BASP1	64902		
chr3	35706139	35706446	0.76	3.90E-01	STAC	-715804	ARPP-21	22444
chr16	88700511	88700889	1.08	3.90E-01	IL17C	-4301		
chr1	1822404	1822737	1.15	3.90E-01	GNB1	-76		
chr5	67511411	67511718	1.09	3.90E-01	PIK3R1	-10553		
chr12	111531252	111531518	0.70	3.90E-01	SH2B3	-312367	CUX2	59556
chr5	150594169	150594538	0.80	3.90E-01	ANXA6	-56987	GM2A	-38259
chr1	161675493	161675803	0.71	3.91E-01	FCRLA	-1114		
chr14	105956050	105956363	0.70	3.91E-01	TMEM121	-36746	CRIP1	2950
chr2	190525813	190526195	1.26	3.91E-01	ASNSD1	-121		
chr9	6681176	6681422	1.32	3.91E-01	KDM4C	-76342	GLDC	-35607
chr13	28057096	28057370	1.25	3.92E-01	MTIF3	-32522	LNX2	137487
chr17	73286151	73286395	1.24	3.92E-01	SLC25A19	-743		
chr17	19411361	19411649	1.33	3.92E-01	SLC47A1	-25662	RNF112	96982
chr22	37614783	37615280	1.20	3.92E-01	SSTR3	-6679	RAC2	25273
chr17	76712928	76713255	1.37	3.92E-01	DNAH17	-145688	CYTH1	65284
chr6	41700419	41700829	0.72	3.92E-01	TFEB	-9160	TFEB	2174
chr15	65903264	65903639	0.84	3.93E-01	C15orf44	-45		
chr7	128694883	128695358	1.22	3.93E-01	TNPO3	77		
chr10	1496669	1496966	0.68	3.93E-01	IDI1	-401757	ADARB2	282900
chr11	61647590	61647834	0.77	3.93E-01	FADS3	11294	FADS2	51999
chr7	140374145	140374456	0.84	3.93E-01	NDUFB2	-22180	ADCK2	1348
chr20	46040878	46041188	0.86	3.93E-01	NCOA3	-89624	ZMYND8	-55559
chr5	36151960	36152333	1.19	3.93E-01	LMBRD2	-132	SKP2	-42
chr6	30583217	30583576	0.81	3.93E-01	MRPS18B	-2089	PPP1R10	1623
chr17	29421632	29421947	0.82	3.93E-01	NF1	-205		
chr18	56338183	56338862	1.26	3.94E-01	MALT1	-95		
chr5	90677551	90677841	0.74	3.94E-01	ARRDC3	1453	GPR98	823079
chr11	61100512	61100893	1.33	3.94E-01	DDB1	-37	DAK	49
chr17	1303351	1303678	0.66	3.94E-01	YWHAE	41		
chr6	36515041	36515398	0.79	3.94E-01	STK38	27		
chr12	6960438	6960779	0.71	3.95E-01	USP5	-676	CDCA3	-153

chr11	102217745	102218056	1.26	3.95E-01	BIRC2	-65		
chr20	5093437	5093948	0.88	3.95E-01	C20orf30	40		
chr3	167098017	167098278	1.41	3.95E-01	ZBBX	-77		
chr19	36184744	36185054	1.22	3.95E-01	ZBTB32	-18931	UPK1A	27184
chr17	80656520	80656779	0.76	3.96E-01	RAB40B	-52		
chr3	119396224	119396698	1.27	3.96E-01	COX17	-218		
chr3	194412220	194412636	0.77	3.96E-01	LSG1	-19222	C3orf21	579467
chr16	3073990	3074455	1.24	3.96E-01	HCFC1R1	64	THOC6	191
chr8	38815022	38815336	1.40	3.96E-01	HTRA4	-16489	PLEKHA2	56426
chr5	133983895	133984351	0.86	3.97E-01	SEC24A	-356		
chr10	30994205	30994581	1.40	3.97E-01	AK302694	13190	ZNF438	326473
chr11	118740834	118741113	1.27	3.97E-01	DDX6	-79002	CXCR5	-13567
chr12	94633959	94634250	1.52	3.97E-01	PLXNC1	91606	TMCC3	410219
chr3	45429953	45430204	1.37	3.98E-01	LARS2	4		
chr2	112641571	112641961	0.91	3.98E-01	ANAPC1	-25		
chr16	57481246	57481563	0.78	3.98E-01	CIAPIN1	-36	COQ9	3
chr8	133969568	133969985	1.01	3.98E-01	TG	90572	SLA	102826
chr9	126085009	126085388	0.87	3.98E-01	STRBP	-54356	CRB2	-33249
chr20	25299261	25299591	1.28	3.99E-01	PYGB	70720	ABHD12	72051
chr10	7514019	7514363	1.27	3.99E-01	SFMBT2	-60741	ITIH5	194743
chr2	68993469	68993832	1.41	3.99E-01	ARHGAP25	31683	BMP10	104998
chr6	32157958	32158301	0.86	3.99E-01	PBX2	-167		
chr19	1438190	1438552	1.21	3.99E-01	RPS15	8		
chr7	99517084	99517393	0.76	3.99E-01	TRIM4	-85		
chr1	156736849	156737383	1.36	3.99E-01	HDGF	-15573	SH2D2A	49524
chr19	19599999	19600393	0.84	3.99E-01	TSSK6	26273	GATAD2A	103554
chr6	53200236	53200771	0.89	3.99E-01	GCM1	-186880	ELOVL5	13438
chr9	134248686	134249082	1.20	4.00E-01	BAT2L	-56593	PPAPDC3	83803
chr9	131843214	131843457	0.83	4.00E-01	DOLPP1	-47		
chr22	24199824	24200178	1.21	4.00E-01	SLC2A11	-40		
chr3	182833863	182834365	1.23	4.00E-01	MCCC1	-16749	LAMP3	46553
chr6	41392275	41392520	0.62	4.00E-01	FOXP4	-121766	NCR2	88870
chr3	182043089	182043399	0.69	4.00E-01	ATP11B	-468047	SOX2	613522
chr10	134385438	134385773	1.29	4.00E-01	INPP5A	34253	NKX6-2	213931
chr7	89874289	89874590	1.35	4.00E-01	C7orf63	-48		
chr22	39928713	39928996	1.22	4.00E-01	RPS19BP1	5		
chr3	128880117	128880399	1.28	4.00E-01	ISY1	-229		
chr6	160147765	160148106	0.80	4.00E-01	WTAP	-216		

chr9	19380075	19380557	0.81	4.00E-01	RPS6	-81		
chr5	145562007	145562494	1.32	4.00E-01	LARS	43		
chr17	80509753	80510017	0.81	4.00E-01	FOXK2	32291	RAB40B	146713
chr10	88281453	88281754	1.14	4.01E-01	WAPAL	-63		
chr16	15737057	15737341	1.19	4.01E-01	KIAA0430	-190		
chr19	50848027	50848606	1.31	4.01E-01	NAPSB	-312		
chr11	89956151	89956474	0.82	4.01E-01	CHORDC1	219		
chr1	168147913	168148405	1.22	4.02E-01	TIPRL	-12		
chr13	79233134	79233465	1.36	4.02E-01	RNF219	14		
chr5	130970888	130971433	1.23	4.03E-01	RAPGEF6	-232		
chr16	22202350	22202885	0.89	4.03E-01	EEF2K	-14974	VWA3A	98755
chr2	232478573	232478882	0.89	4.03E-01	PTMA	-94507	NMUR1	-83546
chr14	24835877	24836154	1.00	4.03E-01	NFATC4	-129		
chr19	796945	797279	1.35	4.03E-01	PTBP1	-280		
chr11	72533021	72533372	0.80	4.03E-01	ATG16L2	7746	FCHSD2	319946
chr16	87422078	87422420	1.11	4.03E-01	FBXO31	-4855	MAP1LC3B	-3552
chr1	54872967	54873502	0.85	4.04E-01	SSBP3	-1143		
chr11	58869657	58870056	0.82	4.04E-01	FAM111B	-4801		
chr19	14247316	14247761	1.09	4.04E-01	ASF1B	-99		
chr2	220462306	220462626	1.31	4.04E-01	STK11IP	-130		
chr22	50629843	50630287	1.12	4.04E-01	PANX2	20905	TUBGCP6	53335
chr5	36723999	36724297	1.23	4.04E-01	NIPBL	-152713	SLC1A3	117691
chr7	102067022	102067306	1.29	4.04E-01	ORAI2	-6832	PRKRIP1	30360
chr10	102673060	102673304	1.29	4.05E-01	SEMA4G	-59104	PAX2	167714
chr3	10234727	10235054	1.32	4.05E-01	TATDN2	-55286	IRAK2	28328
chr7	99102187	99102576	0.79	4.05E-01	ZNF394	-4505	ZKSCAN5	109
chr1	15852998	15853367	1.26	4.05E-01	CASP9	-2393	DNAJC16	-169
chr7	86848982	86849276	0.83	4.05E-01	C7orf23	-98		
chr19	15574575	15574885	0.73	4.06E-01	RASAL3	652		
chr3	197432589	197432930	1.31	4.06E-01	BDH1	-149902	KIAA0226	31013
chr11	66189102	66189413	1.21	4.06E-01	NPAS4	783		
chr19	55850862	55851266	1.04	4.06E-01	SUV420H2	-157		
chr15	64388022	64388342	1.26	4.06E-01	SNX1	5		
chr2	231533441	231533726	1.27	4.07E-01	CAB39	-43973	SP100	252713
chr22	50908319	50908636	0.77	4.07E-01	SBF1	4986	SAPS2	126718
chr6	52516901	52517158	0.88	4.07E-01	TRAM2	-75168	TMEM14A	-18854
chr19	42388197	42388620	1.22	4.07E-01	ARHGEF1	-37		
chr6	16420192	16420560	0.84	4.07E-01	GMPR	181565	ATXN1	341345

chr1	183439351	183439695	0.87	4.07E-01	SMG7	-2111		
chr12	17092794	17093111	1.27	4.07E-01	LMO3	-331929		
chr11	48131442	48131793	1.27	4.07E-01	OR4B1	-106744	PTPRJ	129508
chr14	62228877	62229138	1.20	4.08E-01	SNAPC1	-67		
chr22	39096911	39097162	1.22	4.08E-01	GTPBP1	-4770		
chr17	38136868	38137226	1.28	4.08E-01	PSMD3	-13		
chr15	98703339	98703746	0.81	4.08E-01	IGF1R	-489218		
chr15	45018841	45019151	1.38	4.08E-01	TRIM69	-9544	B2M	15311
chr2	74153839	74154083	1.21	4.08E-01	DGUOK	8		
chr2	232551770	232552229	0.74	4.08E-01	NMUR1	-156818	PTMA	-21235
chr5	156599430	156599700	0.91	4.08E-01	ITK	-8342	FAM71B	-6286
chr17	33914095	33914440	1.27	4.09E-01	AP2B1	-14		
chr6	30034529	30035192	0.77	4.09E-01	PPP1R11	-71		
chr19	2475973	2476290	1.06	4.09E-01	GADD45B	-3		
chr1	85742273	85742726	1.21	4.09E-01	SYDE2	-75772	BCL10	1083
chr17	78193312	78193577	1.30	4.09E-01	SLC26A11	-792	SGSH	754
chr16	8891419	8891764	1.18	4.10E-01	TMEM186	-87	PMM2	-78
chr2	64978385	64978771	1.20	4.10E-01	SLC1A4	-237878	SERTAD2	-97532
chr6	44046893	44047196	0.75	4.10E-01	MRPL14	48146	VEGFA	309092
chr12	57145491	57145814	0.96	4.10E-01	PRIM1	493		
chr6	43603295	43603774	0.82	4.10E-01	MAD2L1BP	-42		
chr16	81040987	81041317	1.23	4.10E-01	ATMIN	-28306	CENPN	1049
chr17	27055727	27056115	1.26	4.10E-01	TLCD1	-2691	NEK8	89
chr5	64920522	64920957	0.83	4.10E-01	TRIM23	-553		
chr2	201577775	201578099	1.32	4.10E-01	BZW1	-98710	AOX1	127206
chr1	225615759	225616145	1.17	4.11E-01	LBR	-168		
chr18	46987050	46987341	1.21	4.11E-01	DYM	-117		
chr12	113611154	113611472	1.26	4.11E-01	RASAL1	-37292	DDX54	11971
chr17	30936183	30936436	0.89	4.11E-01	CDK5R1	122205	MYO1D	267592
chr19	35814271	35814621	1.47	4.11E-01	CD22	-5633	MAG	31408
chr2	239016044	239016471	0.77	4.11E-01	KLHL30	-33159	SCLY	46626
chr1	26648030	26648359	0.70	4.11E-01	CD52	3784	AIM1L	22248
chr22	41864822	41865145	1.13	4.11E-01	PHF5A	-276	ACO2	-145
chr15	44580659	44580980	0.88	4.11E-01	CASC4	-109		
chr13	41019666	41020009	1.35	4.11E-01	FOXO1	220896	COG6	790074
chr2	202645811	202646081	1.23	4.11E-01	ALS2	-51		
chr6	26171954	26172287	0.83	4.11E-01	HIST1H2BE	-11903	HIST1H2BD	13772
chr19	1592793	1593080	1.12	4.12E-01	MBD3	-285		

chr4	166248312	166248622	0.89	4.12E-01	SC4MOL	-351		
chr19	1383672	1384025	1.36	4.12E-01	NDUFS7	142		
chr3	172428699	172429104	0.90	4.12E-01	NCEH1	106		
chr9	33290287	33290667	1.24	4.12E-01	NFX1	-33		
chr12	6570968	6571474	0.87	4.12E-01	VAMP1	8622	TAPBPL	10044
chr1	203258567	203258999	1.47	4.13E-01	CHIT1	-59923	BTG2	-15881
chr6	134274018	134274405	0.75	4.13E-01	TBPL1	-89		
chr13	33160367	33160676	0.78	4.13E-01	PDS5B	-70		
chr6	75953468	75953778	1.25	4.13E-01	COX7A2	-98		
chr8	128748308	128748611	0.79	4.13E-01	MYC	145		
chr17	7740131	7740460	1.18	4.13E-01	KDM6B	-2939		
chr12	27332779	27333091	0.86	4.14E-01	STK38L	-64143	MED21	157452
chr10	111976089	111976365	1.01	4.14E-01	MXI1	8864	SMNDC1	88480
chr7	102036514	102036887	0.81	4.14E-01	PRKRIP1	-103		
chr22	31885862	31886172	0.72	4.14E-01	EIF4ENIF1	-313		
chr15	44955148	44955613	0.74	4.14E-01	SPG11	495		
chr6	35656540	35656787	0.66	4.14E-01	FKBP5	55		
chr17	66342694	66343033	0.73	4.14E-01	SLC16A6	-55459	WIPI1	110789
chr5	112043025	112043288	1.30	4.14E-01	EPB41L4A	-288147	APC	-30399
chr7	74074145	74074566	0.84	4.15E-01	NCF1	-113953	GTF2I	2326
chr11	67236669	67237007	1.25	4.16E-01	TMEM134	-107		
chr7	102985106	102985453	1.34	4.16E-01	PSMC2	-2809	DNAJC2	40
chr3	186857142	186857727	1.22	4.16E-01	RPL39L	-172		
chr11	59436549	59436865	1.20	4.16E-01	PATL1	-196		
chr12	48551260	48551561	1.21	4.16E-01	ASB8	-34		
chr19	11491588	11492166	0.73	4.17E-01	EPOR	3142	LPPR2	25770
chr16	87840303	87840684	1.17	4.18E-01	KLHDC4	-40952	SLC7A5	62606
chr11	117821653	117821925	1.27	4.18E-01	IL10RA	-35317	TMPRSS13	-21674
chr2	55844640	55844977	1.26	4.18E-01	SMEK2	304		
chr3	25706362	25706720	0.78	4.19E-01	TOP2B	-710		
chr17	36981496	36981806	1.36	4.19E-01	PIP4K2B	-25493	RPL23	28402
chr18	48723920	48724276	0.75	4.19E-01	MEX3C	-408		
chr2	3622617	3622947	1.43	4.19E-01	RPS7	-71		
chr14	21945151	21945473	1.22	4.19E-01	RAB2B	-180	TOX4	-23
chr17	1627868	1628112	1.16	4.19E-01	PRPF8	-39814	SERPINF2	-18140
chr17	28443667	28444168	1.18	4.20E-01	CCDC55	84		
chr7	89975849	89976342	0.77	4.20E-01	GTPBP10	106		
chr1	156252597	156252907	1.28	4.20E-01	TMEM79	-1318	SMG5	-132

chr16	81515009	81515316	1.13	4.20E-01	PLCG2	-297767	CMIP	36388
chr17	30771260	30771580	1.23	4.20E-01	PSMD11	-82		
chr3	142720225	142720508	1.27	4.20E-01	SR140	-5		
chr20	43724436	43724746	0.95	4.20E-01	KCNS1	5162	STK4	129471
chr11	118965996	118966306	1.21	4.20E-01	H2AFX	26		
chr19	4471499	4471850	1.31	4.21E-01	HDGF2	-580		
chr3	37035088	37035560	1.22	4.21E-01	EPM2AIP1	-529	MLH1	345
chr16	12058677	12058981	1.15	4.21E-01	TNFRSF17	-135		
chr2	209130612	209130978	1.33	4.21E-01	PIKFYVE	-196		
chr3	40350773	40351210	1.32	4.21E-01	EIF1B	-181		
chr18	32870057	32870378	1.29	4.21E-01	ZNF397OS	-46	ZNFPHEX133	-18
chr17	47439847	47440157	0.79	4.21E-01	ZNF652	-167		
chr11	119067522	119067911	1.24	4.21E-01	CBL	-9273	PDZD3	11522
chr20	48419127	48419395	1.46	4.21E-01	B4GALT5	-88840	SLC9A8	-9989
chr6	26026518	26026810	0.77	4.22E-01	HIST1H4B	816		
chr12	7079688	7080095	1.22	4.22E-01	EMG1	-52	PHB2	24
chr12	32049531	32049841	1.24	4.22E-01	BICD1	-210499	H3F3C	-104511
chr13	21872104	21872405	1.12	4.22E-01	SKA3	-121545	ZDHHC20	161168
chr2	112917174	112917489	1.50	4.23E-01	FBLN7	21370	ZC3H8	95332
chr6	30524617	30524879	0.82	4.23E-01	PRR3	-8	GNL1	260
chr3	152879109	152879437	0.74	4.23E-01	RAP2B	-756		
chr3	186500837	186501154	1.10	4.23E-01	EIF4A2	-365		
chr17	55682829	55683116	1.27	4.23E-01	MRPS23	244426	MSI2	349042
chr3	183602550	183602865	1.25	4.23E-01	PARL	-15		
chr14	59768117	59768510	1.32	4.23E-01	DAAM1	112915	GPR135	163745
chr2	114647248	114647588	1.12	4.24E-01	ACTR3	-119		
chr12	113646372	113646654	1.12	4.24E-01	DDX54	-23229	TPCN1	-12747
chr5	134389030	134389544	0.87	4.24E-01	PITX1	-19323	H2AFY	345641
chr10	27792957	27793360	1.11	4.24E-01	RAB18	-90		
chr3	41240732	41241119	0.85	4.25E-01	CTNNB1	-16		
chr5	110427804	110428174	0.92	4.25E-01	WDR36	119		
chr14	50329505	50329813	1.32	4.25E-01	ARF6	-30077	SDCCAG1	-10120
chr3	183484823	183485133	1.26	4.25E-01	MAP6D1	58415	YEATS2	69372
chr11	1545083	1545391	0.73	4.25E-01	HCCA2	-43122	DUSP8	47913
chr6	27806028	27806311	0.88	4.25E-01	HIST1H2BN	-270	HIST1H2AK	-53
chr14	106175839	106176084	1.21	4.25E-01	IGHE	153500	TMEM121	183009
chr17	76143416	76144105	1.23	4.25E-01	SYNGR2	-20910	C17orf99	1327
chr20	62689156	62689436	1.33	4.26E-01	TCEA2	857		

chr1	234745817	234746168	1.26	4.26E-01	IRF2BP2	-722		
chr5	109024401	109024670	0.90	4.26E-01	MAN2A1	-620		
chr3	160117243	160117742	1.33	4.26E-01	IFT80	-173	SMC4	63
chr4	17578590	17578941	1.20	4.26E-01	LAP3	-161		
chr6	150067609	150067894	0.71	4.26E-01	PCMT1	-3079	NUP43	-64
chr22	36784086	36784524	1.15	4.26E-01	MYH9	-242		
chr3	105974735	105975078	0.87	4.26E-01	CBLB	-387020		
chr2	175499187	175499853	1.41	4.26E-01	WIPF1	-213		
chr21	38593519	38593826	0.94	4.26E-01	DSCR3	46160	TTC3	148102
chr19	18414310	18414644	1.35	4.27E-01	JUND	-22045	LSM4	19524
chr11	66445816	66446258	1.28	4.27E-01	RBM4B	-762		
chr12	121454206	121454450	1.26	4.27E-01	OASL	22452	HNF1A	37779
chr6	119031251	119031501	1.51	4.27E-01	MCM9	159983	PLN	161934
chr17	27068563	27068931	0.81	4.27E-01	TRAF4	-2276		
chr7	64147664	64147941	0.75	4.27E-01	ZNF138	-106968	ZNF107	21292
chr5	138739571	138739905	0.84	4.28E-01	SPATA24	38		
chr16	69364380	69364670	1.10	4.28E-01	PDF	-27		
chr16	28985898	28986233	1.33	4.29E-01	SPNS1	-30		
chr17	19265804	19266114	0.83	4.29E-01	B9D1	87	b9	87
chr11	67044937	67045276	1.30	4.29E-01	SSH3	-25812	ADRBK1	11202
chr14	67826390	67826724	1.16	4.29E-01	EIF2S1	-477	ATP6V1D	34
chr4	113558282	113558854	1.31	4.29E-01	C4orf21	-417	LARP7	-44
chr11	124543447	124544016	0.91	4.29E-01	SIAE	-15	SPA17	-8
chr5	133702788	133703065	1.38	4.29E-01	UBE2B	-3943	CDKL3	-162
chr16	67694198	67694926	0.76	4.29E-01	PARD6A	-289	ACD	156
chr1	13992210	13992520	0.82	4.29E-01	PRDM2	-38985	PDPN	82113
chr19	45458088	45458646	1.25	4.29E-01	CLPTM1	-271		
chr19	5903638	5904019	1.21	4.29E-01	VMAC	-1023	NDUFA11	196
chr3	139062680	139063008	1.19	4.29E-01	MRPS22	46		
chr16	29984691	29985029	0.87	4.29E-01	TAOK2	-362		
chr6	44205708	44205982	0.74	4.29E-01	HSP90AB1	-9004	SLC29A1	14549
chr17	25659552	25659824	1.20	4.30E-01	KSR1	-139348	WSB1	38582
chr18	74204768	74205118	1.02	4.30E-01	ZNF236	-331173	ZNF516	-29846
chr20	62362074	62362480	1.38	4.30E-01	LIME1	-4856		
chr8	66546453	66546764	0.89	4.31E-01	ARMC1	-177		
chr15	60771205	60771483	1.17	4.31E-01	NARG2	0		
chr12	98987247	98987528	1.22	4.31E-01	SLC25A3	-15		
chr3	182510894	182511260	1.13	4.31E-01	ATP11B	-214		

chr10	93683279	93683663	0.88	4.31E-01	BTAF1	-265		
chr1	181057722	181058054	1.19	4.31E-01	CACNA1E	-394828	MR1	54749
chr16	53537208	53537490	1.09	4.32E-01	AKTIP	-179		
chr20	30865217	30865540	1.21	4.32E-01	KIF3B	-88		
chr11	46260749	46260989	1.26	4.32E-01	PHF21A	-117884	CREB3L1	-38359
chr3	169872263	169872748	0.94	4.32E-01	PHC3	27031	GPR160	116771
chr1	36689979	36690397	1.21	4.32E-01	THRAP3	171		
chr15	85839911	85840258	1.31	4.32E-01	AKAP13	-83786	PDE8A	314880
chr12	54582526	54582974	0.87	4.33E-01	SMUG1	7		
chr6	41336638	41336926	0.80	4.33E-01	FOXP4	-177382	NCR2	33254
chr4	185654450	185654726	1.00	4.33E-01	MLF1IP	698		
chr9	130741685	130741995	0.83	4.34E-01	DPM2	-41077	NAIF1	87759
chr12	125411739	125412050	1.40	4.34E-01	UBC	-12318	DHX37	61772
chr3	169581272	169581531	1.37	4.35E-01	LRRC31	6258	LRRIQ4	41692
chr7	148397866	148398145	0.81	4.35E-01	CUL1	2073	EZH2	183408
chr10	112257432	112257696	0.64	4.35E-01	DUSP5	-61		
chr11	450088	450337	1.32	4.35E-01	PTDSS2	-67		
chr1	184020639	184020902	1.24	4.35E-01	TSEN15	-40		
chr11	10329399	10329957	0.86	4.35E-01	AMPD3	-142546	ADM	3036
chr9	139118006	139118256	1.11	4.35E-01	LHX3	-23127	QSOX2	19556
chr19	12996051	12996323	1.45	4.35E-01	DNASE2	-3852		
chr21	16135482	16135807	1.39	4.36E-01	SAMSN1	-216981	NRIP1	301481
chr6	34855676	34856048	0.89	4.36E-01	ANKS1A	-1176	TAF11	-43
chr1	167189170	167189574	0.92	4.36E-01	POU2F1	-771		
chr1	41134449	41134797	1.24	4.36E-01	RIMS3	-3299		
chr12	46782228	46782604	1.10	4.36E-01	SLC38A2	-15771	SLC38A4	437364
chr7	150594604	150594896	1.38	4.36E-01	ABP1	45177	KCNH2	80264
chr17	40713987	40714264	1.35	4.37E-01	MLX	-4952	COASY	34
chr7	86848153	86848570	0.84	4.37E-01	C7orf23	669		
chr7	30387752	30388000	0.81	4.37E-01	ZNRF2	63953	NOD1	130517
chr5	64777663	64777907	1.20	4.38E-01	ADAMTS6	-81		
chr12	32291955	32292293	1.28	4.38E-01	FGD4	-362917	BICD1	31939
chr1	109969064	109969432	0.79	4.38E-01	PSMA5	-211		
chr20	33680529	33680862	1.20	4.38E-01	TRPC4AP	-78		
chr3	49449491	49449838	1.17	4.39E-01	RHOA	-139		
chr4	6717634	6717990	1.19	4.39E-01	CNO	-30		
chr3	4508788	4509118	0.78	4.39E-01	SUMF1	1		
chr2	220094179	220094666	1.24	4.39E-01	ATG9A	-62	ANKZF1	-56

chr1	6761813	6762093	1.29	4.39E-01	DNAJC11	13		
chr3	10362695	10362969	1.13	4.39E-01	SEC13	26		
chr2	37617548	37617919	1.46	4.39E-01	QPCT	45981	CDC42EP3	281592
chr19	30096983	30097313	1.19	4.39E-01	POP4	-22		
chr15	93461123	93461433	1.21	4.39E-01	CHD2	17727	RGMA	171155
chr4	80979951	80980197	0.91	4.39E-01	GK2	-650702	ANTXR2	14403
chr2	99771239	99771659	1.22	4.40E-01	TSGA10	-262	LIPT1	31
chr11	18127502	18127859	0.88	4.40E-01	SAAL1	-43		
chr1	227015782	227016139	1.33	4.40E-01	ITPKB	-89085	PSEN2	-42312
chr7	142246198	142246434	1.38	4.40E-01	TCRBV1S1A1N1	-6283	TCRBV6S1A1N1	1299
chr9	128023674	128024213	1.16	4.40E-01	GAPVD1	-167		
chr13	41163828	41164138	1.28	4.40E-01	FOXO1	76751	COG6	934219
chr3	195634685	195635019	0.83	4.41E-01	TNK2	-12420	SDHALP1	82298
chr1	32479159	32479559	1.11	4.41E-01	KHDRBS1	-132		
chr17	8095519	8095969	0.76	4.41E-01	C17orf59	-2180		
chr16	3333313	3333623	1.17	4.41E-01	ZNF263	-19		
chr19	35503931	35504207	1.22	4.41E-01	SCN1B	-17465	GRAMD1A	12823
chr7	7291427	7291727	0.79	4.41E-01	C1GALT1	69331	COL28A1	283883
chr16	2097722	2098033	1.26	4.42E-01	TSC2	-112	NTHL1	-11
chr10	46983707	46983984	0.87	4.42E-01	PPYR1	-99688	SYT15	-13245
chr11	65407106	65407386	0.76	4.42E-01	SIPA1	1668	RELA	23197
chr16	2205223	2205682	1.19	4.42E-01	TRAF7	-346		
chr3	156799562	156799862	0.91	4.42E-01	CCNL1	78770	TIPARP	407333
chr5	71597504	71597756	0.91	4.42E-01	MRPS27	18454	MAP1B	194512
chr6	24891273	24891578	0.97	4.42E-01	FAM65B	19769	GMNN	116262
chr15	76196001	76196414	1.19	4.43E-01	FBXO22	8		
chr4	51607	51933	1.62	4.43E-01	ZNF718	-1457		
chr20	13765420	13765730	1.30	4.43E-01	C20orf7	-107	ESF1	-43
chr10	49864320	49864594	0.71	4.43E-01	ARHGAP22	-51319	WDFY4	-29061
chr10	126407744	126407998	1.40	4.43E-01	FAM53B	25059	LHPP	257459
chr15	93363563	93363985	1.28	4.43E-01	FAM174B	-164743	CHD2	-79777
chr11	64084774	64085295	1.32	4.44E-01	PRDX5	-534	TRMT112	-2
chr6	227333	227661	1.13	4.44E-01	DUSP22	-64604		
chr16	21371395	21371822	1.16	4.44E-01	CRYM	-81952	NPIPL3	65090
chr19	17637817	17638093	1.19	4.44E-01	GLT25D1	-28556	PGLS	15523
chr3	188672417	188672706	1.43	4.44E-01	TPRG1	-217201	LPP	741841
chr14	105123394	105123704	0.95	4.44E-01	TMEM179	-52452	INF2	-32394
chr7	152373157	152373448	1.12	4.45E-01	XRCC2	-53		

chr1	204476044	204476414	1.37	4.46E-01	PIK3C2B	-16755	MDM4	-9282
chr6	35435885	35436323	0.83	4.46E-01	RPL10A	-74		
chr9	36487730	36488064	0.67	4.46E-01	RNF38	-87601	MELK	-85008
chr19	14097330	14097607	1.20	4.46E-01	PODNL1	-33265	RFX1	19665
chr2	198299619	198299984	1.25	4.47E-01	SF3B1	-31		
chr19	597519	597907	0.74	4.47E-01	HCN2	7820	POLRMT	35855
chr2	101840257	101840658	0.94	4.47E-01	TBC1D8	-72612	C2orf29	-28887
chr17	42404164	42404515	0.81	4.47E-01	SLC25A39	-2123		
chr1	40851291	40851616	1.32	4.47E-01	ZNF643	-64325	SMAP2	11726
chr19	2613814	2614166	0.70	4.47E-01	GNG7	88756	GADD45B	137855
chr5	94890578	94890940	1.27	4.47E-01	TTC37	-77	ARSK	-66
chr16	3200516	3200875	1.35	4.48E-01	OR1F1	-53551	caspace	6476
chr2	224821829	224822299	1.28	4.48E-01	MRPL44	-57		
chr14	23357801	23358243	0.79	4.48E-01	REM2	5590	RBM23	30374
chr2	214097799	214098167	0.80	4.48E-01	IKZF2	-82925	SPAG16	-51133
chr22	41347120	41347613	1.13	4.48E-01	RBX1	-18		
chr11	18343969	18344253	0.87	4.48E-01	HPS5	-390	GTF2H1	295
chrX	1621183	1621523	0.86	4.48E-01	ASMTL	-49509	P2RY8	34684
chr17	38375311	38375589	1.25	4.48E-01	WIPF2	-124		
chr20	5639582	5639860	1.14	4.48E-01	CHGB	-252253	RP5-1022P6.2	-48049
chr17	4699210	4699622	1.33	4.48E-01	PSMB6	-41		
chr22	31686555	31686976	0.71	4.49E-01	PIK3IP1	1754	LIMK2	78516
chr9	95087421	95087814	1.25	4.49E-01	CENPP	-123	NOL8	258
chr2	29117282	29117601	1.27	4.49E-01	WDR43	-91		
chr8	144598620	144599005	1.06	4.49E-01	MAFA	-86237	ZC3H3	24807
chr7	36429317	36429725	0.79	4.50E-01	ANLN	89		
chr1	155990629	155990887	1.35	4.50E-01	SSR2	0		
chr3	157815929	157816173	1.28	4.50E-01	VEPH1	-598606	SHOX2	7901
chr4	176272664	176273047	1.43	4.50E-01	ADAM29	433347	GPM6A	650792
chr11	3013570	3013814	1.27	4.50E-01	NAP1L4	-85		
chr13	50698506	50698774	1.25	4.50E-01	ST13	-47514	KCNRG	109250
chr3	9404517	9404849	1.36	4.50E-01	THUMPD3	-34		
chr15	31727531	31727911	0.70	4.50E-01	KLF13	108638	OTUD7A	219821
chr15	65596620	65596877	1.31	4.50E-01	PARP16	-17731	IGDCC3	73629
chr3	47323950	47324440	1.11	4.51E-01	KLHL18	-135	KIF9	142
chr15	50716460	50716735	1.11	4.51E-01	USP8	19		
chr7	20259617	20259944	0.77	4.51E-01	MACC1	-2768		
chr11	72889155	72889414	0.78	4.51E-01	P2RY2	-40059	FCHSD2	-36142

chr13	28712197	28712470	1.31	4.51E-01	FLT3	-37605	PAN3	-36075
chr15	59063230	59063546	1.23	4.51E-01	ADAM10	-21211	SLTM	162464
chr14	52313786	52314289	0.94	4.52E-01	GNG2	-13012	FRMD6	195462
chr1	36615114	36615461	1.21	4.52E-01	TRAPPC3	-221		
chr5	141044586	141044929	1.34	4.52E-01	ARAP3	17042	RELL2	28241
chr14	21539543	21539823	1.43	4.52E-01	C14orf176	-27413	FLJ10357	1156
chr20	48429064	48429412	1.26	4.53E-01	SLC9A8	-12		
chr2	32581916	32582270	1.20	4.53E-01	BIRC6	-3		
chr3	121711497	121711824	0.85	4.53E-01	ILDR1	29369	SLC15A2	98490
chr1	44678948	44679258	0.81	4.53E-01	DMAP1	-22		
chr15	72410414	72410960	1.12	4.54E-01	MYO9A	-265	SENP8	-67
chr9	140473245	140473567	1.10	4.54E-01	ZMYND19	11531	MRPL41	27097
chr6	43597688	43598057	0.84	4.54E-01	GTPBP2	-937		
chr10	121066101	121066479	0.79	4.54E-01	GRK5	99093	RGS10	235932
chr20	50157844	50158271	1.04	4.54E-01	KCNG1	-518383	NFATC2	1200
chr7	139876792	139877058	1.11	4.54E-01	JHDM1D	-184		
chr15	42565423	42565882	1.27	4.54E-01	GANC	-713	TMEM87A	102
chr2	26568462	26569066	1.21	4.55E-01	SELI	-190		
chr5	43105796	43106106	0.79	4.55E-01	C5orf39	-65504	ZNF131	-15691
chr13	79968000	79968313	0.88	4.55E-01	RNF219	-734843	RBM26	11766
chr12	132413614	132413976	1.27	4.55E-01	PUS1	6		
chr2	219264284	219264713	0.75	4.55E-01	CTDSP1	21		
chr2	223520657	223521094	0.83	4.55E-01	FARSB	-49		
chr3	14989579	14989919	0.75	4.55E-01	NR2C2	513		
chr13	40700496	40700866	0.80	4.55E-01	COG6	470917	FOXO1	540053
chr3	58335589	58335980	0.91	4.55E-01	PXK	17168	PDHB	83780
chr8	27221337	27221625	0.79	4.55E-01	PTK2B	38427	CHRNA2	115332
chr4	146540316	146540736	1.13	4.56E-01	MMAA	-14		
chr1	205180725	205181047	1.34	4.56E-01	DSTYK	-159		
chr1	118878106	118878378	0.88	4.56E-01	SPAG17	-150394	TBX15	653937
chr1	6403562	6403806	1.14	4.57E-01	GPR153	-82649	ACOT7	42199
chr19	56146138	56146456	1.14	4.57E-01	ZNF784	-10356	ZNF580	-6095
chr9	127952064	127952397	1.15	4.57E-01	PPP6C	-13		
chr5	140699779	140700089	0.80	4.58E-01	TAF7	417		
chr3	128578458	128578694	0.76	4.58E-01	AK294171	5715	RAB7A	133597
chr5	133561699	133562156	1.17	4.58E-01	SKP1	-49204	CDKL3	140837
chr20	5931101	5931480	1.21	4.58E-01	TRMT6	-118	MCM8	-7
chr1	17232134	17232463	0.73	4.58E-01	MSTP9	-141324	CROCC	-16146

chr2	43823096	43823382	1.13	4.58E-01	THADA	-54		
chr12	56334075	56334352	0.75	4.59E-01	DGKA	8402	SILV	25632
chr12	110448992	110449377	1.38	4.59E-01	IFT81	-112955	GIT2	-14991
chr7	103848332	103848730	0.73	4.59E-01	ORC5L	-68		
chr15	63771845	63772201	1.41	4.59E-01	CA12	-97948	USP3	-24787
chr8	101571898	101572175	0.94	4.59E-01	ANKRD46	-25		
chr11	76348499	76348908	0.81	4.59E-01	LRRC32	32340	C11orf30	192635
chr15	79296838	79297220	1.03	4.59E-01	CTSH	-59609	RASGRF1	86186
chr6	27106833	27107307	1.47	4.59E-01	HIST1H4I	-18		
chr19	16695176	16695552	1.20	4.59E-01	SLC35E1	-12171	MED26	43651
chr22	39795636	39795912	1.19	4.60E-01	MAP3K7IP1	15		
chr12	7068170	7068562	1.17	4.60E-01	PHB2	11550	PTPN6	12626
chr1	45452130	45452481	0.81	4.60E-01	EIF2B3	-24		
chr3	101395840	101396195	1.33	4.60E-01	ZBTB11	-30		
chr6	30539113	30539466	0.81	4.60E-01	ABCF1	120		
chr16	30483089	30483339	0.98	4.61E-01	ITGAL	-769		
chr10	3827489	3827785	0.93	4.61E-01	KLF6	-164		
chr22	24094543	24095052	0.79	4.61E-01	ZNF70	-1519		
chr13	28024554	28024847	1.27	4.61E-01	MTIF3	10		
chr11	61666667	61666938	1.12	4.61E-01	FADS3	-7797	RAB3IL1	18194
chr16	85936560	85936836	1.18	4.61E-01	FOXF1	-607435	IRF8	3924
chr19	39881468	39882072	1.31	4.61E-01	MED29	-193	PAF1	-91
chr6	28104502	28104812	0.85	4.61E-01	ZNF192	-5059	ZSCAN16	12270
chr5	44808757	44809006	1.33	4.61E-01	MRPS30	-145		
chr19	5720099	5720883	1.28	4.61E-01	LONP1	-315	TMEM146	-197
chr13	31735941	31736685	0.72	4.61E-01	HSPH1	-196		
chr19	36545152	36545494	1.31	4.61E-01	THAP8	341		
chr8	67564234	67564537	0.81	4.61E-01	MYBL1	-38906	VCPIP1	15066
chr13	20756445	20756721	1.22	4.61E-01	GJA3	-21400	GJB2	10531
chr1	28574949	28575330	0.69	4.61E-01	SESN2	-10866	ATPIF1	12529
chr14	54080247	54080725	1.14	4.61E-01	DDHD1	-460440	BMP4	340784
chr6	37759282	37759526	0.76	4.61E-01	MDGA1	-93638	ZFAND3	-27903
chr6	26533002	26533359	0.87	4.62E-01	HMGN4	-5391	BTN1A1	31686
chr1	100435250	100435642	0.78	4.63E-01	SLC35A3	-94		
chr12	11801617	11801954	1.39	4.63E-01	ETV6	-1002		
chr17	48229346	48229637	0.82	4.63E-01	PPP1R9B	-1615		
chr6	33668291	33668542	0.82	4.63E-01	C6orf125	11087	ITPR3	79256
chr4	48832909	48833171	1.25	4.63E-01	OCIAD1	25		

chr3	188299674	188299939	1.52	4.63E-01	TPRG1	-589956	LPP	369086
chr17	1496182	1496482	1.31	4.63E-01	PITPNA	-30222	SLC43A2	35798
chr4	164415597	164415971	1.41	4.63E-01	TKTL2	-20737	MARCH1	118992
chr19	572228	572612	1.12	4.63E-01	BSG	-117		
chr16	50315940	50316253	0.74	4.63E-01	ADCY7	-5726	PAPD5	128439
chr7	98270965	98271744	0.75	4.64E-01	NPTX2	24758	TMEM130	196318
chr10	112678794	112679149	0.77	4.64E-01	SHOC2	-329		
chr19	45004508	45004920	1.09	4.64E-01	ZNF180	-140		
chr7	105162537	105162781	1.07	4.64E-01	PUS7	26		
chr2	89155064	89155761	0.85	4.64E-01	O1/O11 and JK2	-735149	RPIA	164237
chr7	100472304	100472882	0.82	4.64E-01	SRRT	-108		
chr15	70805131	70805441	0.76	4.64E-01	TLE3	-415030	UACA	250564
chr19	2151605	2151980	1.08	4.64E-01	AP3D1	-237		
chr7	56131834	56132092	0.84	4.65E-01	SUMF2	46		
chr11	66175936	66176213	1.23	4.65E-01	SLC29A2	-36784	NPAS4	-12400
chr16	3450977	3451486	1.20	4.65E-01	ZNF434	-219	ZNF174	42
chr4	148978143	148978469	0.92	4.65E-01	ARHGAP10	324853	NR3C2	385337
chr7	129588626	129589024	0.82	4.65E-01	UBE2H	3964	NRF1	337270
chr4	174290879	174291406	0.80	4.66E-01	SAP30	-950		
chr11	14913128	14913409	1.09	4.66E-01	CYP2R1	482		
chr13	46742602	46742861	1.16	4.66E-01	CPB2	-63521	LCP1	13727
chr19	11616580	11616843	0.76	4.66E-01	ZNF653	-58		
chr3	183165147	183165474	1.32	4.66E-01	MCF2L2	-19456	KLHL6	108188
chr7	44240379	44240685	0.85	4.66E-01	YKT6	-46		
chr11	62572789	62573120	1.20	4.67E-01	NXF1	9		
chr19	58891966	58892264	1.18	4.67E-01	ZNF837	274		
chr8	71520511	71520836	0.73	4.67E-01	TRAM1	-70		
chr1	38478003	38478400	1.29	4.67E-01	UTP11L	-182		
chr19	54704875	54705196	1.25	4.67E-01	RPS9	310		
chr2	30387930	30388200	1.30	4.67E-01	ALK	-243633	LBH	-66332
chr1	227922905	227923265	1.26	4.68E-01	SNAP47	388		
chr6	36842397	36842800	0.88	4.68E-01	PPIL1	201		
chr12	111057307	111057723	0.86	4.68E-01	TCTN1	5635	HVCN1	69431
chr12	124118180	124118491	1.18	4.68E-01	EIF2B1	-89	GTF2H3	-45
chr21	30665873	30666183	0.79	4.68E-01	BACH1	-5192	C21orf7	213155
chr15	75079700	75080506	1.39	4.68E-01	LMAN1L	-25091	CSK	5678
chr6	36270259	36270569	0.93	4.68E-01	PNPLA1	32177	ETV7	85053
chr1	156695653	156695965	0.82	4.69E-01	C1orf66	-2454	ISG20L2	1896

chr11	75400872	75401244	1.58	4.69E-01	MOGAT2	-27876	MAP6	-21579
chr1	151170810	151171377	0.84	4.69E-01	PIP5K1A	73		
chr2	68961872	68962184	1.33	4.69E-01	ARHGAP25	60		
chr21	30375011	30375262	1.14	4.69E-01	USP16	-21801	RNF160	-9860
chr10	104238772	104239087	0.68	4.69E-01	TMEM180	17760	ACTR1A	23582
chr6	26234642	26235265	0.78	4.70E-01	HIST1H1D	262		
chr10	105156246	105156577	1.29	4.70E-01	USMG5	-189	PDCD11	0
chr19	4863878	4864133	0.94	4.70E-01	TICAM1	-32269	PLIN3	3774
chr17	80170605	80170872	1.15	4.70E-01	FASN	-114633	SLC16A3	-15554
chr3	183852915	183853265	1.21	4.70E-01	EIF2B5	280		
chr3	187636865	187637222	1.27	4.71E-01	LPP	-293677	BCL6	-173569
chr6	53036699	53037038	0.83	4.71E-01	GCM1	-23245	ELOVL5	177073
chr1	179923528	179923889	0.90	4.71E-01	CEP350	-199		
chr7	7984467	7984959	1.21	4.71E-01	RPA3	-226475	GLCCI1	-23710
chr6	20401905	20402277	0.77	4.71E-01	E2F3	-46		
chr4	154389757	154390067	1.18	4.72E-01	TLR2	-215529	KIAA0922	2414
chr3	156877816	156878274	1.30	4.72E-01	CCNL1	437		
chr11	82611492	82611882	0.77	4.72E-01	C11orf82	-1050	PRCP	-130
chr17	16905159	16905422	0.79	4.72E-01	KIAA0864	-162683	TNFRSF13B	-29889
chr9	108006482	108006861	1.17	4.72E-01	SLC44A1	-257		
chr4	146970930	146971243	1.26	4.72E-01	LSM6	-125748	ZNF827	-111480
chr9	116344196	116344559	1.12	4.72E-01	ZNF618	-294184	RGS3	137367
chr1	22351682	22352049	0.79	4.73E-01	CDC42	-27254	CELA3B	48448
chr7	24365293	24365650	0.87	4.73E-01	MPP6	-247613	NPY	41663
chr11	118966308	118966699	1.28	4.73E-01	H2AFX	-327		
chr17	62493062	62493342	1.15	4.73E-01	POLG2	-33		
chr20	31366238	31366548	1.12	4.73E-01	MAPRE1	-41306	DNMT3B	16202
chr3	48274163	48274569	1.20	4.73E-01	ZNF589	-8230	CAMP	9504
chr6	28615935	28616183	1.30	4.73E-01	SCAND3	-60947	TRIM27	275709
chr2	182170442	182170691	0.81	4.73E-01	ITGA4	-151052	UBE2E3	325455
chr12	56546006	56546393	1.15	4.73E-01	MYL6B	-135		
chr19	12886383	12886719	0.92	4.73E-01	HOOK2	-117		
chr17	5342277	5342785	1.23	4.73E-01	C1QBP	-60		
chr2	32264789	32265066	1.17	4.73E-01	DPY30	-84		
chr1	157776339	157776919	0.79	4.73E-01	FCRL2	-29707	FCRL1	13311
chr12	125479706	125480104	0.89	4.73E-01	AACS	-70020	BRI3BP	1711
chr6	170101996	170102378	0.75	4.73E-01	C6orf120	-70		
chr12	2364890	2365254	0.79	4.74E-01	FKBP4	-539036	CACNA1C	202656

chr5	145582873	145583349	0.82	4.74E-01	RBM27	-52		
chr12	25205331	25205654	0.84	4.74E-01	LRMP	252		
chr15	52121758	52122087	0.98	4.74E-01	TMOD3	34		
chr15	83474777	83475113	1.37	4.74E-01	WHAMM	-3028		
chr11	67381509	67381849	0.89	4.74E-01	NDUFV1	7356	NUDT8	15722
chr5	180645062	180645505	1.39	4.74E-01	TRIM7	-13107	TRIM41	-5022
chr6	30312818	30313121	0.85	4.74E-01	HLA-E	-144301	TRIM39	17962
chr9	107686113	107686423	0.91	4.74E-01	ABCA1	4168	NIPSNAP3B	159817
chr2	60577913	60578273	1.26	4.75E-01	BCL11A	202540		
chr17	36740558	36740903	0.82	4.75E-01	SNIP	21452	ARHGAP23	127087
chr6	26123106	26123690	1.28	4.75E-01	HIST1H2AC	-975	HIST1H2BC	734
chr11	63953137	63953767	0.66	4.75E-01	STIP1	-135		
chr1	212965020	212965330	1.25	4.75E-01	NSL1	-36	TATDN3	5
chr15	100105870	100106304	1.26	4.75E-01	MEF2A	-46		
chr2	220143691	220144184	1.08	4.75E-01	DNAJB2	-102		
chr20	2451318	2451722	1.29	4.75E-01	SNRPB	-21		
chr3	135915143	135915497	1.24	4.75E-01	MSL2	-632		
chr16	67554990	67555470	0.81	4.75E-01	AGRP	-37514	FAM65A	-7524
chr1	110576999	110577295	1.18	4.75E-01	FAM40A	-94		
chr3	187687424	187687774	0.87	4.75E-01	LPP	-243122	BCL6	-224124
chr11	327076	327461	1.22	4.75E-01	B4GALNT4	-42526	IFITM3	-6355
chr17	45908790	45909075	1.10	4.75E-01	LRRC46	-167	MRPL10	-30
chr3	196014459	196014949	1.14	4.76E-01	PCYT1A	-120		
chr8	67579356	67579605	0.81	4.76E-01	VCPIP1	-29		
chr7	106535639	106535963	1.27	4.76E-01	PRKAR2B	-149377	PIK3CG	29877
chr9	115248870	115249110	1.30	4.76E-01	HSDL2	106639	C9orf80	231397
chr19	14682765	14683122	1.38	4.77E-01	NDUFB7	-58		
chr13	51375514	51375806	1.28	4.77E-01	RNASEH2B	-108232	ST13	629506
chr2	237966569	237966917	1.31	4.77E-01	COPS8	-27341	CXCR7	488363
chr20	37063892	37064202	1.38	4.77E-01	RALGAPB	-37439	LBP	89162
chr3	101443283	101443533	1.44	4.77E-01	CEP97	-86		
chr14	90849649	90849950	1.13	4.77E-01	C14orf102	-51521	CALM1	-13573
chr1	153643345	153643881	1.46	4.77E-01	ILF2	-134		
chr11	121190001	121190285	1.35	4.77E-01	SORL1	-132818	SC5DL	26755
chr7	74064386	74064700	1.15	4.78E-01	GTF2I	-7487	GTF2IRD1	196423
chr8	144623657	144623901	1.04	4.78E-01	ZC3H3	-159		
chr4	13629248	13629631	1.16	4.78E-01	BOD1L	-112		
chr12	32112026	32112365	0.83	4.78E-01	H3F3C	-167021	BICD1	-147989

chr19	47616189	47616608	1.06	4.78E-01	ZC3H4	610		
chr17	27070200	27070626	0.78	4.79E-01	TRAF4	-610		
chr20	5778503	5778996	1.13	4.79E-01	RP5-1022P6.2	-187078	CHGB	-113224
chr14	103851492	103851820	0.95	4.79E-01	MARK3	-45		
chr19	49837035	49837475	1.29	4.79E-01	CD37	-1422		
chr1	16563588	16563861	1.06	4.79E-01	C1orf89	-66		
chr2	170430316	170430573	1.34	4.79E-01	FASTKD1	-21		
chr4	152682000	152682381	1.32	4.79E-01	PET112L	-45		
chr8	37707248	37707634	1.20	4.79E-01	BRF2	-30		
chr16	87735294	87735643	1.16	4.79E-01	KLHDC4	64073	JPH3	98970
chr10	38265250	38265828	1.18	4.79E-01	ZNF25	-86		
chr17	3866864	3867108	0.80	4.79E-01	ATP2A3	750		
chr19	42637340	42637701	1.28	4.79E-01	POU2F2	-891		
chr7	124996065	124996312	1.27	4.79E-01	POT1	-426152		
chr14	74229075	74229444	1.32	4.79E-01	PNMA1	-48132	C14orf43	24636
chr8	38268165	38268450	0.89	4.79E-01	LETM2	24288	FGFR1	58044
chr19	18155851	18156129	1.12	4.79E-01	IL12RB1	41752	KCNN1	93879
chr16	48278059	48278335	0.84	4.80E-01	LONP2	-14		
chr2	198317766	198318100	1.21	4.80E-01	COQ10B	-298		
chr5	132299151	132299439	1.10	4.81E-01	AFF4	59		
chr7	91509844	91510201	0.86	4.81E-01	MTERF	-7		
chr9	95055959	95056334	1.26	4.81E-01	IARS	-109		
chr1	154192967	154193403	1.22	4.81E-01	UBAP2L	-140	C1orf43	88
chr9	102028898	102029233	1.38	4.81E-01	NR4A3	-559943	SEC61B	44496
chr12	26626272	26626616	1.24	4.81E-01	SSPN	277938	ITPR2	359687
chr12	109125273	109125604	1.01	4.82E-01	CORO1C	-144		
chr19	5978189	5978510	1.22	4.82E-01	RANBP3	-30		
chr9	135259779	135260084	1.43	4.82E-01	SETX	-29560	TTF1	22289
chr19	3762701	3763206	1.23	4.82E-01	APBA3	-1281	MRPL54	289
chr14	94444580	94444874	0.93	4.82E-01	OTUB2	-47997	ASB2	-20960
chr7	129650757	129651006	1.19	4.82E-01	UBE2H	-58093	ZC3HC1	40351
chr19	6753273	6753713	1.28	4.82E-01	TRIP10	13786	SH2D3A	14030
chr19	12662349	12662865	0.87	4.82E-01	ZNF709	-66975	ZNF490	59016
chr3	50653344	50653654	0.84	4.82E-01	CISH	-4237	MAPKAPK3	-1102
chr3	112280656	112281343	1.28	4.82E-01	ATG3	-515	SLC35A5	105
chr5	70751294	70751544	1.21	4.82E-01	BDP1	-23		
chr11	64863440	64863755	1.28	4.82E-01	C11orf2	-85		
chr12	12939842	12940093	1.03	4.82E-01	DDX47	-26173	APOLD1	61117

chr20	61585619	61586041	0.90	4.82E-01	SLC17A9	1831	BHLHE23	52557
chr4	39136939	39137214	1.13	4.82E-01	WDR19	-46947	KLHL5	73017
chr22	40446285	40446606	0.92	4.83E-01	TNRC6B	-127483	GRAP2	149360
chr16	66557913	66558182	0.85	4.83E-01	TK2	26267	BEAN	96808
chr2	20850761	20851137	1.17	4.83E-01	HS1BP3	-85		
chr1	78470404	78470673	1.30	4.83E-01	DNAJB4	-97		
chr10	75639436	75639746	0.68	4.83E-01	PLAU	-31271	CAMK2G	-5248
chrX	33201471	33201802	1.28	4.83E-01	DMD	28036		
chr13	20357057	20357301	1.22	4.84E-01	PSPC1	-96		
chr13	31309543	31309797	1.26	4.84E-01	ALOX5AP	1		
chr3	197676661	197677092	0.76	4.84E-01	RPL35A	-175		
chr15	52774111	52774427	1.34	4.84E-01	MYO5C	-186299	MYO5A	46978
chr17	42293629	42293918	1.22	4.84E-01	ATXN7L3	-18245	UBTF	1890
chr7	97831730	97832156	1.20	4.84E-01	BHLHA15	-9625	LMTK2	95746
chr2	20101569	20101876	1.19	4.84E-01	TTC32	21		
chr3	156391858	156392270	1.36	4.84E-01	TIPARP	-315		
chr7	104992658	104992973	1.21	4.84E-01	SRPK2	36525	MLL5	338179
chr11	65382636	65383381	1.24	4.85E-01	MAP3K11	-1289	PCNXL3	-774
chr10	105128363	105128614	1.11	4.85E-01	TAF5	765		
chr19	5690062	5690378	1.29	4.85E-01	RPL36	-52		
chr20	56055849	56056177	1.41	4.85E-01	HMGB1L1	8070	RBM38	89550
chr7	151047114	151047558	1.13	4.85E-01	NUB1	8478	RHEB	169674
chr11	102301190	102301598	1.39	4.85E-01	TMEM123	22381	BIRC2	83428
chr9	140317522	140317843	1.23	4.85E-01	NOXA1	-164	EXD3	31
chr20	4795319	4795936	1.33	4.85E-01	RASSF2	141		
chr15	59430413	59430723	0.95	4.85E-01	LDHAL6B	-68474	CCNB2	33248
chr7	6676785	6677036	0.56	4.85E-01	ZNF853	21384	ZNF12	69655
chr16	87812639	87813028	1.28	4.85E-01	KLHDC4	-13292	SLC7A5	90266
chr2	65173420	65173665	1.15	4.85E-01	SERTAD2	-292497	SLC1A4	-42913
chr19	12807316	12807626	1.22	4.85E-01	FBXW9	-16		
chr14	102605911	102606339	1.19	4.86E-01	HSP90AA1	-39		
chr5	172571286	172571750	0.93	4.86E-01	BNIP1	73		
chr14	77587545	77588112	1.32	4.86E-01	C14orf4	-92795	ZDHHC22	20305
chr6	26195565	26195918	0.82	4.86E-01	HIST1H2BF	-4045	HIST1H3D	3722
chr18	71814940	71815214	1.25	4.86E-01	C18orf55	-669	FBXO15	23
chr7	108056278	108056591	0.91	4.86E-01	LAMB4	-285634	NRCAM	40391
chr17	46985408	46985905	0.93	4.86E-01	UBE2Z	-110		
chr3	141206635	141206909	1.29	4.86E-01	RASA2	846		

chr10	74870103	74870360	0.78	4.87E-01	NUDT13	22		
chr19	40324640	40325056	1.09	4.87E-01	DYRK1B	-7		
chr11	47600133	47600773	0.85	4.87E-01	NDUFS3	-109	KBTBD4	114
chr16	1877073	1877330	1.27	4.87E-01	FAHD1	-23	HAGH	-7
chr12	9913395	9913753	1.32	4.87E-01	CD69	-77		
chr10	93557994	93558264	1.11	4.87E-01	TNKS2	-22		
chr6	30875695	30875951	0.86	4.87E-01	GTF2H4	-154		
chr12	510426	511060	0.74	4.87E-01	B4GALNT3	-58800	KDM5A	-12123
chr12	123717823	123718288	1.08	4.88E-01	C12orf65	215		
chr18	48724333	48724837	0.81	4.88E-01	MEX3C	-895		
chr19	36119780	36120055	1.15	4.88E-01	RBM42	-62		
chr2	187350680	187351033	1.24	4.88E-01	ZC3H15	-28		
chr5	56469213	56469576	1.23	4.88E-01	MIER3	-221441	GPBP1	-40506
chr6	30070895	30071193	0.85	4.88E-01	RNF39	-27416	TRIM31	9823
chr6	30421414	30421658	0.87	4.88E-01	HLA-E	-35735	TRIM39	126528
chr1	47184684	47185119	1.19	4.88E-01	CYP4B1	-79768	C1orf223	47402
chr20	30467857	30468171	1.24	4.88E-01	TTLL9	9509	PDRG1	71869
chr14	45722267	45722681	0.83	4.89E-01	C14orf106	131		
chr10	76995714	76995958	0.70	4.89E-01	UNQ766	-145	COMTD1	-66
chr2	58468335	58468648	1.17	4.89E-01	FANCL	23		
chr7	142985191	142985591	1.15	4.89E-01	CASP2	-11		
chr9	139839011	139839416	1.05	4.89E-01	C8G	-484	FBXW5	-41
chr11	66311215	66311620	0.80	4.89E-01	ACTN3	-2973	ZDHHC24	2253
chr12	53886869	53887132	1.13	4.90E-01	MAP3K12	6270	PCBP2	41115
chr12	49582691	49583074	0.75	4.90E-01	TUBA1C	-75982	TUBA1B	-57579
chr12	66563557	66563979	0.89	4.90E-01	TMBIM4	39		
chr3	141456825	141457191	1.18	4.90E-01	RNF7	-43		
chr22	23623962	23624272	1.14	4.90E-01	BCR	101565	IGLL1	298378
chr7	35840560	35840922	0.78	4.90E-01	SEPT7	114		
chr16	11403718	11404048	0.70	4.91E-01	C16orf75	-35428	PRM1	-28691
chr17	74068573	74068819	1.04	4.91E-01	GALR2	-2196	SRP68	-89
chr20	42939564	42939967	1.17	4.91E-01	FITM2	123		
chr1	17053759	17054090	1.15	4.91E-01	NBPF1	-113943	MSTP9	37050
chr6	141804844	141805129	1.42	4.91E-01	AK097143	546567		
chr2	64067206	64067690	0.89	4.91E-01	UGP2	-1566		
chr6	143234678	143235063	1.03	4.91E-01	HIVEP2	31467	GPR126	611815
chr2	60593315	60593735	1.09	4.92E-01	BCL11A	187108		
chr21	30396870	30397159	1.24	4.92E-01	USP16	77		

chr6	33359052	33359671	0.77	4.92E-01	KIFC1	49		
chr14	69281313	69281689	1.40	4.92E-01	ZFP36L1	-21716	ACTN1	164582
chrX	9309037	9309297	1.64	4.92E-01	FAM9B	-308051	TBL1X	-124034
chr5	78053956	78054253	1.16	4.92E-01	LHFPL2	-109457	ARSB	228252
chr1	170500948	170501258	1.00	4.93E-01	GORAB	-160		
chr1	27524479	27524726	0.88	4.93E-01	SLC9A1	-43152	WDTC1	-36404
chr12	57977138	57977388	1.28	4.93E-01	PIP4K2C	-7679	KIF5A	33416
chr3	42814633	42814921	0.78	4.93E-01	HHATL	-70458	HIGD1A	31157
chr6	41559736	41560018	0.82	4.93E-01	MDFI	-46318	FOXP4	45713
chr11	66383815	66384320	1.19	4.93E-01	RBM14	15		
chr7	30634184	30634559	0.78	4.93E-01	GARS	191		
chr19	42381023	42381384	1.17	4.93E-01	CD79A	14		
chr17	21183362	21183672	0.88	4.93E-01	MAP2K3	-4451		
chr6	30710462	30710968	1.27	4.93E-01	FLOT1	-262		
chr12	6961415	6961860	1.16	4.93E-01	CDCA3	-1182	USP5	353
chr1	32715494	32715987	0.78	4.94E-01	LCK	-23971	EIF3I	27782
chr17	33905499	33905817	0.90	4.94E-01	PEX12	-2		
chr1	38061392	38061685	1.26	4.95E-01	GNL2	47		
chr7	76961252	76961514	0.85	4.95E-01	PTPN12	-205390	FGL2	-132233
chr11	14541846	14542180	0.83	4.95E-01	PSMA1	-22		
chr20	17517521	17517902	1.25	4.96E-01	DSTN	-32887	BFSP1	-5699
chr2	65659740	65660050	1.10	4.96E-01	SPRED2	-239		
chr11	118777699	118778171	1.22	4.97E-01	BCL9L	3678	CXCR5	23394
chr13	50194383	50194634	0.85	4.97E-01	RCBTB1	-34790	ARL11	-8115
chr1	38157937	38158214	1.13	4.97E-01	CDCA8	-83		
chr11	74303463	74303802	1.34	4.97E-01	POLD3	4		
chr19	17716953	17717263	1.09	4.97E-01	GLT25D1	50597	UNC13A	82293
chr8	41909778	41910268	0.90	4.98E-01	MYST3	-518		
chr16	88717707	88718150	1.22	4.98E-01	CYBA	-472		
chr15	57401252	57401531	0.84	4.98E-01	CGNL1	-267313	TCF12	190559
chr16	71929127	71929508	0.89	4.98E-01	KIAA0174	-128		
chr13	53024732	53025033	0.92	4.98E-01	CKAP2	-4612	VPS36	-120
chr3	48507317	48507773	0.86	4.98E-01	TREX1	316		
chr3	184135664	184135940	1.11	4.98E-01	EPHB3	-143785	CHRD	37941
chr11	65625482	65625888	1.13	4.99E-01	MUS81	-2187	CFL1	416
chr16	11422426	11422736	1.20	4.99E-01	PRM1	-47389	C16orf75	-16730
chr22	35773011	35773324	0.81	5.00E-01	HMOX1	-3919		
chr2	96811049	96811593	0.88	5.00E-01	DUSP2	-142		

chr3	15643297	15643701	1.17	5.00E-01	HACL1	-369	BTD	244
chr8	64080848	64081275	0.73	5.00E-01	YTHDF3	-59		
chr16	67880512	67880822	1.17	5.00E-01	NUTF2	-152	CENPT	694
chr19	30101506	30101828	1.22	5.00E-01	PLEKHF1	-54660	POP4	4497
chr3	160167505	160167883	0.88	5.01E-01	TRIM59	-68		
chr17	80455039	80455287	1.14	5.01E-01	FOXK2	-22431	NARF	38623
chr13	37393186	37393488	1.26	5.02E-01	RFXAP	-2		
chr3	13133656	13133947	1.14	5.02E-01	IQSEC1	-19185	NUP210	328007
chr5	55884582	55885094	1.12	5.02E-01	IL6ST	-594075	MAP3K1	-226062
chr17	27054995	27055617	1.28	5.02E-01	TLCD1	-2076	NEK8	-526
chr1	45965617	45966002	1.23	5.03E-01	MMACHC	-46		
chr15	73075965	73076283	1.18	5.03E-01	ADPGK	2		
chr16	2518818	2519138	0.73	5.03E-01	NTN3	-2522		
chr20	46130423	46130786	1.17	5.03E-01	NCOA3	-52		
chr6	27832857	27833287	0.91	5.03E-01	HIST1H2AL	-35		
chr9	132597391	132597703	1.14	5.03E-01	USP20	-149		
chr5	43066870	43067238	0.92	5.03E-01	ZNF131	-54588	C5orf39	-26607
chr11	9635525	9636077	0.84	5.03E-01	SWAP70	-49827	WEE1	40573
chr11	66445259	66445567	1.27	5.03E-01	RBM4B	-138		
chr11	27528169	27528460	0.82	5.04E-01	LIN7C	11		
chr11	72449892	72450189	1.26	5.04E-01	PDE2A	-64547	ARAP1	13393
chr2	85166702	85167034	1.36	5.04E-01	KCMF1	-31363	TMSB10	34105
chr2	170440699	170441009	1.24	5.04E-01	PPIG	4		
chr15	34516998	34517354	1.16	5.04E-01	TMEM85	-69		
chr3	107941189	107941595	1.25	5.04E-01	IFT57	25		
chr12	92987577	92987976	0.91	5.04E-01	BTG1	-448104	PLEKHG7	-142488
chr11	68039476	68039757	1.11	5.04E-01	C11orf24	-148		
chr12	117175905	117176306	1.24	5.04E-01	C12orf49	-263	RNFT2	10
chr9	37324572	37325103	1.00	5.04E-01	GRHPR	-97869	ZCCHC7	204369
chr13	77566066	77566387	1.19	5.05E-01	CLN5	168		
chr2	216973703	216974049	1.19	5.05E-01	XRCC5	-144		
chr6	28696992	28697236	1.33	5.05E-01	SCAND3	-142002	TRIM27	194654
chr11	64645897	64646456	1.22	5.05E-01	EHD1	14		
chr9	37800626	37800904	1.14	5.06E-01	DCAF10	-25		
chr12	65012809	65013132	1.35	5.06E-01	RASSF3	8678	GNS	140255
chr7	24917461	24917841	0.78	5.06E-01	DFNA5	-120568	OSBPL3	102109
chr19	56015360	56015678	0.83	5.06E-01	SSC5D	15649	SBK2	32142
chr1	53704108	53704418	1.14	5.06E-01	MAGOH	-56		

chr6	27094852	27095150	0.86	5.06E-01	ZNF322A	-435038	HIST1H2BJ	5574
chr1	12212150	12212449	1.21	5.06E-01	TNFRSF1B	-14760	TNFRSF8	88866
chr2	233415210	233415520	1.21	5.06E-01	TIGD1	-139	EIF4E2	8
chr7	142491102	142491429	0.86	5.07E-01	TCRBC2	-3101		
chr15	78832611	78833070	1.21	5.07E-01	PSMA4	94		
chr13	33112863	33113327	1.30	5.07E-01	N4BP2L2	-159		
chr6	43138572	43139003	0.83	5.07E-01	SRF	-132		
chr9	35619371	35620189	0.77	5.07E-01	CD72	-1356		
chr19	17850116	17850356	1.13	5.07E-01	B3GNT3	-55683	MAP1S	19933
chr9	117672893	117673282	1.43	5.07E-01	TNFSF15	-104680	TNFSF8	19682
chr16	31044536	31044862	1.35	5.07E-01	STX4	-204		
chr6	106773562	106773865	0.87	5.07E-01	ATG5	-19		
chr15	90418256	90418540	1.31	5.07E-01	ANPEP	-60326	C15orf38	37824
chr17	80416311	80416621	0.75	5.08E-01	NARF	-74		
chr11	18343446	18343885	1.27	5.08E-01	GTF2H1	-150	HPS5	55
chr4	183839063	183839336	1.18	5.08E-01	DCTD	-570		
chr1	203309240	203309516	1.32	5.08E-01	FMOD	10911	BTG2	34714
chr5	87971607	87971873	1.06	5.08E-01	TMEM161B	-407075	MEF2C	207562
chr19	50269491	50269810	1.19	5.08E-01	TSKS	-3136	AP2A1	-529
chr5	180634602	180634912	1.29	5.08E-01	TRIM7	-2580		
chr11	65313568	65314117	0.88	5.08E-01	LTBP3	11856	SCYL1	21295
chr5	125950956	125951393	1.23	5.08E-01	LMNB1	-161658	PHAX	14542
chr19	5803983	5804375	1.01	5.08E-01	NRTN	-19639	DUS3L	-12930
chr10	63659835	63660222	0.89	5.08E-01	ARID5B	-1414		
chr5	179050486	179051056	1.14	5.09E-01	HNRNPH1	-49		
chr7	65447117	65447370	0.88	5.09E-01	GUSB	2		
chr22	41252566	41253160	0.83	5.09E-01	XPNPEP3	-231	ST13	-176
chr2	198318482	198318792	0.99	5.10E-01	COQ10B	406		
chrX	13752424	13752959	1.09	5.10E-01	OFD1	-140	TRAPPC2	50
chr1	1565666	1566019	1.11	5.10E-01	MMP23B	-1717		
chr7	73153188	73153466	1.20	5.10E-01	ABHD11	-137		
chr12	46121317	46121627	1.18	5.11E-01	ARID2	-2148		
chr6	30881894	30882203	0.71	5.11E-01	VARS2	461		
chr12	6741460	6741770	0.79	5.11E-01	LPAR5	-800		
chr12	51762698	51763044	1.22	5.11E-01	CELA1	-22408	GALNT6	22329
chr2	6364417	6364727	0.85	5.11E-01	SOX11	531773	CMPK2	641364
chr12	49504387	49504930	0.72	5.11E-01	LMBR1L	21		
chr3	58173512	58173748	0.72	5.11E-01	DNASE1L3	26768	FLNB	179503

chr18	21032980	21033418	0.83	5.11E-01	RIOK3	412		
chr2	70056538	70056895	1.09	5.12E-01	GMCL1	-101		
chr1	46306467	46306745	1.38	5.12E-01	MAST2	37321	PIK3R3	291774
chr1	235098329	235098635	1.33	5.12E-01	IRF2BP2	-353211	TOMM20	193774
chr6	2891978	2892355	0.86	5.12E-01	SERPINB1	-50086	SERPINB9	11378
chr8	54811363	54811782	0.90	5.12E-01	RGS20	47205	TCEA1	123435
chr1	226374312	226374556	1.35	5.12E-01	ACBD3	-11		
chr19	38864970	38865863	1.31	5.13E-01	PSMD8	227		
chr6	73332816	73333100	0.84	5.13E-01	KCNQ5	1387	KHDC1	639949
chr6	313082	313437	0.85	5.13E-01	IRF4	-78492	DUSP22	21159
chr12	113436647	113437159	1.07	5.13E-01	DTX1	-58759	OAS2	20629
chrX	48595483	48595829	0.87	5.13E-01	GATA1	-49326	SUV39H1	40525
chr16	67850457	67850818	0.82	5.13E-01	THAP11	-25575	TSNAXIP1	9628
chrX	129402723	129403098	0.92	5.14E-01	ZNF280C	-38		
chr14	102414316	102414778	1.30	5.15E-01	DYNC1H1	-16318	PPP2R5C	186412
chr19	18682363	18682730	1.22	5.15E-01	UBA52	-67		
chr4	1283402	1283665	1.13	5.15E-01	MAEA	-138		
chr16	70415138	70415582	0.77	5.15E-01	ST3GAL2	57631	DDX19B	82288
chr6	167001641	167001951	1.09	5.15E-01	BRP44L	-205310	RPS6KA2	273975
chr8	61829042	61829474	0.84	5.15E-01	RLBP1L1	-371267	CHD7	237919
chr15	31556496	31556797	1.17	5.15E-01	TRPM1	-162723	KLF13	-62436
chr13	31191646	31192206	0.69	5.16E-01	USPL1	96		
chr18	32754235	32754481	1.37	5.16E-01	ZNF397	-66640	MAPRE2	196150
chr11	43333327	43333705	1.35	5.16E-01	API5	11		
chr2	176046365	176046704	1.00	5.16E-01	ATP5G3	-144		
chr14	102173651	102173968	1.18	5.17E-01	PPP2R5C	-54325	DIO3	146122
chr9	140135477	140135894	1.10	5.17E-01	TUBB2C	-25		
chr17	8103452	8103743	1.16	5.17E-01	C17orf59	-10034	AURKB	10285
chr1	154155593	154156005	0.97	5.17E-01	NUP210L	-28207	TPM3	8810
chr16	70557302	70557656	0.77	5.17E-01	SF3B3	-212	COG4	-22
chr9	95491058	95491384	0.75	5.17E-01	IPPK	-58674	BICD2	35862
chr14	20811347	20811939	1.29	5.17E-01	PARP2	-130		
chr11	62448240	62448563	0.77	5.17E-01	UBXN1	-1875		
chr11	6624697	6625048	0.94	5.17E-01	ILK	-91	RRP8	-62
chr12	1692695	1692955	1.32	5.17E-01	FBXL14	10506	ERC1	592421
chr15	93449953	93450207	1.33	5.17E-01	CHD2	6529	RGMA	182353
chr19	42069778	42070115	1.24	5.17E-01	ATP5SL	-124137	CEACAM21	-12584
chr7	102987914	102988197	0.79	5.17E-01	DNAJC2	-2736	PSMC2	-33

chr15	35280408	35280673	1.13	5.17E-01	ZNF770	-87		
chr5	118676246	118676624	1.14	5.18E-01	TNFAIP8	-15161		
chr2	85152939	85153254	1.28	5.18E-01	KCMF1	-45134	TMSB10	20334
chr21	47878512	47878856	0.68	5.18E-01	DIP2A	-178		
chr12	111527423	111527665	0.69	5.18E-01	SH2B3	-316208	CUX2	55715
chr1	22778260	22778530	1.22	5.18E-01	ZBTB40	51		
chr19	37808588	37809096	1.23	5.18E-01	HKR1	-16738	ZNF383	91476
chr1	24969295	24969907	1.13	5.19E-01	SRRM1	7		
chr16	89441487	89441740	1.24	5.19E-01	ANKRD11	115355	ZNF778	157503
chr16	28942005	28942356	0.79	5.19E-01	CD19	-1079		
chr17	8042691	8043060	1.29	5.19E-01	HES7	-15474	PER1	12877
chr3	182879358	182879705	1.33	5.19E-01	MCCC1	-62167	LAMP3	1135
chr10	73975677	73976223	0.79	5.19E-01	ASCC1	-256	C10orf104	144
chrX	55744001	55744311	1.31	5.20E-01	RRAGB	46		
chr17	6543979	6544822	0.78	5.20E-01	TXNDC17	179		
chr11	85376075	85376332	1.33	5.20E-01	CREBZF	-22		
chr19	2050117	2050446	1.18	5.20E-01	MKNK2	961		
chr19	4246780	4247447	0.81	5.20E-01	SHD	-31484	EBI3	17574
chr2	70141808	70142132	1.01	5.21E-01	MXD1	-233		
chr1	26615585	26615958	0.80	5.21E-01	SH3BGRL3	9559	UBXN11	17423
chr2	198017168	198017505	0.91	5.21E-01	PGAP1	-225883	ANKRD44	45425
chr3	169755547	169755857	1.19	5.21E-01	GPR160	-33		
chr9	32573020	32573330	1.22	5.21E-01	NDUFB6	7		
chr9	139294557	139294878	1.10	5.21E-01	SNAPC4	-1829		
chr2	8621600	8621997	1.12	5.21E-01	ID2	-200185		
chr5	139049850	139050229	1.16	5.21E-01	PSD2	-125366	CXXC5	21739
chr15	74907901	74908163	1.19	5.21E-01	CLK3	697		
chr15	77835745	77836121	1.13	5.21E-01	LINGO1	88776	HMG20A	122690
chr4	130014434	130014913	1.11	5.21E-01	SCLT1	90		
chr5	142784756	142785177	0.90	5.21E-01	NR3C1	-922		
chr3	39329887	39330249	1.23	5.21E-01	CCR8	-41129	CX3CR1	-8541
chr5	176540952	176541262	0.88	5.21E-01	NSD1	-19726	FGFR4	27186
chr13	51490905	51491244	1.19	5.21E-01	RNASEH2B	7183	GUCY1B2	149268
chr15	41523106	41523572	0.83	5.22E-01	EXD1	-444	CHP	-98
chr10	32635978	32636222	1.31	5.22E-01	EPC1	13		
chr12	113645911	113646238	1.14	5.22E-01	DDX54	-22791	TPCN1	-13185
chr6	27113675	27114013	1.38	5.22E-01	HIST1H2AH	-1064	HIST1H2BK	775
chr11	10830316	10830863	0.84	5.22E-01	EIF4G2	-8		

chr7	44516989	44517265	0.84	5.22E-01	CAMK2B	-151897	NPC1L1	63787
chr13	25861478	25861788	1.10	5.23E-01	MTMR6	71		
chr17	62009570	62009973	0.92	5.23E-01	CD79B	-68		
chr7	130741966	130742343	1.14	5.23E-01	KLF14	-323295	MKLN1	-270440
chr6	33239656	33239938	0.82	5.24E-01	VPS52	-135	RPS18	-55
chr3	195633988	195634267	1.02	5.24E-01	TNK2	-11696	SDHALP1	83022
chr13	98085377	98085711	1.28	5.24E-01	RAP2A	-931		
chr2	196996178	196996483	0.93	5.24E-01	DNAH7	-62795	STK17B	40005
chr7	128065854	128066164	0.90	5.24E-01	C7orf68	-29875	IMPDH1	-15973
chr12	66524445	66524896	0.81	5.24E-01	LLPH	-138		
chr1	27936120	27936449	0.76	5.24E-01	AHDC1	-6142	FGR	25442
chr12	57881496	57882022	1.13	5.24E-01	MARS	39		
chr17	38296458	38296759	1.16	5.24E-01	CASC3	102		
chr18	48556415	48556734	1.08	5.24E-01	SMAD4	-8		
chr14	91833565	91833905	1.06	5.24E-01	GPR68	-113511	CCDC88C	50398
chr3	184080672	184081564	1.27	5.24E-01	CLCN2	-1727	POLR2H	457
chr21	30494108	30494389	1.51	5.24E-01	BACH1	-176971	C21orf7	41376
chr17	1933923	1934359	0.90	5.24E-01	DPH1	710		
chr3	184521099	184521409	1.10	5.24E-01	VPS8	-8677	EPHB3	241667
chr19	797785	798065	0.98	5.24E-01	PTBP1	533		
chr2	68941978	68942462	0.94	5.24E-01	ARHGAP25	-19748	PROKR1	69426
chr9	117349775	117350172	1.21	5.24E-01	ATP6V1G1	-20		
chr14	74237225	74237676	1.39	5.25E-01	PNMA1	-56323	C14orf43	16445
chr3	51703319	51703589	0.83	5.25E-01	TEX264	-1768		
chr11	60253427	60253792	1.39	5.25E-01	MS4A12	-6641	MS4A1	30328
chr17	47785284	47785800	0.86	5.25E-01	SLC35B1	-260		
chr17	79899454	79899822	1.19	5.25E-01	PYCR1	-4670		
chr10	101491789	101492098	1.11	5.26E-01	CUTC	-14	COX15	479
chr15	83518402	83518799	1.07	5.26E-01	WHAMM	40628	HOMER2	102872
chr19	16557843	16558126	0.74	5.26E-01	EPS15L1	24777	KLF2	122334
chr7	32930117	32930399	0.67	5.26E-01	KBTBD2	1210	AVL9	395082
chr3	142314915	142315328	1.15	5.27E-01	PLS1	-27144	ATR	-17454
chr1	160644247	160644555	0.74	5.27E-01	SLAMF1	-27320	CD48	37184
chr9	84742969	84743367	1.20	5.27E-01	FLJ46321	139481	RASEF	934875
chr1	29208534	29208890	0.69	5.27E-01	EPB41	-4916		
chr1	166879676	166879915	1.44	5.27E-01	TADA1	-34142	ILDR2	64765
chr19	46195070	46195434	1.15	5.27E-01	QPCTL	-489	SNRPD2	191
chr5	147763233	147763573	1.30	5.28E-01	FBXO38	-143		

chr11	65210922	65211220	1.14	5.28E-01	SCYL1	-81477	FRMD8	57030
chr1	160458125	160458506	0.86	5.28E-01	SLAMF6	34726	VANGL2	87949
chr21	40752092	40752366	1.20	5.28E-01	WRB	16		
chr22	37258399	37258709	1.08	5.28E-01	CSF2RB	-51121	NCF4	1524
chr3	149470190	149470549	1.37	5.29E-01	COMMD2	-92		
chr3	196669117	196669683	1.19	5.29E-01	NCBP2	64		
chr12	100594389	100594731	1.18	5.30E-01	ACTR6	-14		
chr20	52443617	52443877	1.13	5.30E-01	ZNF217	-244040	BCAS1	243557
chr21	44527657	44527936	1.29	5.30E-01	U2AF1	-109		
chr14	74353109	74353611	0.86	5.30E-01	ZNF410	-226		
chr3	23987669	23987983	1.04	5.30E-01	NR1D2	1075	THRB	548440
chr6	137752089	137752453	1.29	5.30E-01	IFNGR1	-211704	OLIG3	63260
chr6	27440697	27441081	0.88	5.31E-01	ZNF184	8		
chr22	19419650	19420162	1.20	5.31E-01	HIRA	-687	MRPL40	-130
chr9	125667432	125667967	1.13	5.31E-01	RC3H2	-138		
chr21	27107189	27107452	1.26	5.31E-01	GABPA	-8	ATP5J	644
chr9	110046180	110046593	0.99	5.33E-01	RAD23B	843		
chr2	202096970	202097274	1.12	5.33E-01	CASP8	-25632	CASP10	49501
chr6	34725025	34725410	0.81	5.33E-01	SNRPC	-94		
chr19	1040991	1041241	1.21	5.34E-01	HMHA1	-26058	ABCA7	1014
chr3	186714196	186714481	1.35	5.34E-01	ST6GAL1	-25326	ADIPOQ	153876
chr6	28863927	28864255	0.92	5.34E-01	SCAND3	-308979	TRIM27	27677
chr21	45759174	45759527	1.19	5.34E-01	TRPM2	-14133	PFKL	39426
chr10	112680314	112680704	0.79	5.34E-01	SHOC2	1208	RPL13AP6	16504
chr17	8059956	8060356	1.17	5.34E-01	PER1	-4403		
chr3	150320907	150321202	1.29	5.34E-01	SELT	-11		
chr17	2148111	2148347	1.24	5.34E-01	SMG6	58840	HIC1	188625
chr12	111051755	111051997	1.01	5.34E-01	TCTN1	-4		
chr14	50052950	50053300	1.34	5.34E-01	RPS29	-31		
chr16	30669160	30669470	1.09	5.34E-01	ZNF689	-47633	FBRS	-6463
chr14	73493794	73494102	1.18	5.34E-01	ZFYVE1	-109		
chr20	4701323	4701605	1.12	5.34E-01	PRND	-1092		
chr20	31989234	31989520	1.22	5.34E-01	CDK5RAP1	-40		
chr3	152016458	152016768	0.94	5.34E-01	MBNL1	-581		
chr7	96339023	96339348	0.91	5.34E-01	SHFM1	17		
chr11	76337845	76338311	1.12	5.34E-01	LRRC32	42966	C11orf30	182009
chr19	49127422	49127667	1.11	5.34E-01	SPHK2	4997	DBP	13094
chr20	43274983	43275260	1.19	5.34E-01	ADA	5254	PKIG	114686

chr20	57579814	57580129	1.24	5.34E-01	CTSZ	2337	TH1L	23661
chr3	183371015	183371332	0.78	5.34E-01	YEATS2	-44432	KLHL24	17763
chr12	861411	861768	1.26	5.35E-01	WNK1	-635		
chr4	186064305	186064591	0.74	5.35E-01	SLC25A4	50		
chr5	149792332	149792724	0.90	5.35E-01	CD74	-196		
chr11	2721236	2721512	1.05	5.35E-01	CDKN1C	185621	KCNQ1	255153
chr22	23377959	23378384	0.81	5.35E-01	GNAZ	-34497	IGL@	148212
chr2	65615167	65615455	1.17	5.35E-01	SPRED2	44345	ACTR2	160482
chr17	16283894	16284572	0.77	5.36E-01	UBB	-134		
chr1	9687126	9687412	0.80	5.36E-01	PIK3CD	-24521	TMEM201	38292
chr3	169899243	169899638	1.19	5.36E-01	PHC3	96		
chr2	65159630	65159945	0.73	5.36E-01	SERTAD2	-278742	SLC1A4	-56668
chrX	130926393	130926651	1.39	5.36E-01	MST4	-230723	OR13H1	248474
chr4	153587049	153587489	0.86	5.37E-01	FBXW7	-131084	TMEM154	13922
chr20	30310859	30311415	0.74	5.37E-01	BCL2L1	-481		
chr16	2732216	2732622	1.28	5.37E-01	KCTD5	-76		
chr17	49230715	49231044	1.23	5.37E-01	NME1-NME2	-40		
chr22	24142529	24142839	1.17	5.37E-01	SMARCB1	13534	DERL3	38515
chr10	80897599	80897982	0.63	5.37E-01	PPIF	-209429	ZMIZ1	68999
chr1	203830503	203830899	1.15	5.38E-01	SNRPE	-39		
chr16	89008308	89008688	1.16	5.38E-01	LOC390748	-75484	CBFA2T3	34903
chr19	12833375	12833720	1.38	5.38E-01	TNPO2	-86		
chr4	100737774	100738106	1.21	5.38E-01	DAPP1	-41		
chr6	52284684	52285235	0.78	5.38E-01	EFHC1	-34		
chr14	73925444	73925688	1.22	5.38E-01	NUMB	-280		
chr14	20773955	20774284	0.86	5.39E-01	TTC5	33		
chr20	5100295	5100803	1.15	5.39E-01	PCNA	98		
chr1	109584564	109584815	0.75	5.39E-01	CLCC1	-78579	TAF13	33934
chr1	87527476	87527811	1.26	5.39E-01	LMO4	-266507	HS2ST1	147309
chr15	23032920	23033162	1.30	5.39E-01	NIPA2	1367	CYFIP1	140357
chr4	152020502	152020813	1.32	5.40E-01	RPS3A	-96		
chr7	86845951	86846282	0.90	5.40E-01	C7orf23	2914	DMTF1	64440
chr7	101950265	101950674	1.13	5.40E-01	PRKRIP1	-86334	SH2B2	22065
chr14	104029112	104029421	1.23	5.40E-01	BAG5	-653		
chr16	284589	284871	1.10	5.40E-01	ITFG3	-71		
chr16	18801609	18801893	1.21	5.40E-01	RPS15A	-95		
chr3	57541641	57541996	1.29	5.40E-01	PDE12	-162		
chr11	1051095	1051371	0.83	5.40E-01	MUC2	-23642	MUC6	-14527

chr17	1626425	1626650	1.35	5.40E-01	PRPF8	-38362	SERPINF2	-19592
chr12	69080558	69080925	1.23	5.41E-01	NUP107	11		
chr7	158497523	158497790	0.99	5.41E-01	NCAPG2	-137		
chr4	123844164	123844598	1.21	5.41E-01	NUDT6	-622	SPATA5	156
chr7	75157423	75157692	1.06	5.41E-01	PMS2L3	-105		
chr2	6482659	6482951	0.73	5.41E-01	CMPK2	523131	SOX11	650006
chr5	55965859	55966223	1.22	5.41E-01	IL6ST	-675278	MAP3K1	-144859
chr17	63133276	63133541	0.81	5.41E-01	RGS9	-140		
chr5	56469764	56470074	0.91	5.42E-01	MIER3	-221965	GPBP1	-39982
chr8	55014308	55014785	0.84	5.42E-01	LYPLA1	30		
chr3	141088994	141089338	1.32	5.42E-01	RASA2	-116760	ZBTB38	46111
chr17	6554820	6555202	0.99	5.42E-01	MED31	-57		
chr14	95982316	95982626	1.10	5.42E-01	C14orf49	-40298	GLRX5	-18852
chr19	17530411	17530857	1.17	5.42E-01	FAM125A	-278		
chr8	142012309	142012558	1.00	5.42E-01	PTK2	-1102		
chr16	68291150	68291521	0.91	5.43E-01	SLC7A6	-7087	PLA2G15	12089
chr12	54610740	54611094	0.80	5.43E-01	SMUG1	-28160	CBX5	42453
chr5	173172220	173172578	1.14	5.43E-01	CPEB4	-142932	BOD1	-128733
chr2	232259178	232259548	1.18	5.43E-01	B3GNT7	-972		
chrX	49047353	49047634	1.26	5.43E-01	PRICKLE3	-4718		
chr20	17949543	17949853	1.27	5.43E-01	SNX5	-544	C20orf72	-64
chr8	37594983	37595354	1.16	5.44E-01	PROSC	-24932	ERLIN2	1072
chr6	4156168	4156421	0.78	5.44E-01	CDYL	-620385	PECI	-20464
chr6	37071114	37071589	0.76	5.44E-01	PIM1	-66570	FGD2	97929
chr8	103250853	103251309	0.77	5.44E-01	RRM2B	265		
chr6	36561911	36562233	0.79	5.45E-01	SFRS3	-18		
chr17	16438927	16439275	1.28	5.45E-01	ZNF287	33419	C17orf45	96800
chr15	89631151	89631459	1.05	5.45E-01	ABHD2	-76		
chr3	193821287	193821571	1.20	5.45E-01	HES1	-32505	OPA1	510496
chr3	141030509	141030936	1.28	5.45E-01	ZBTB38	-12332	ACPL2	80041
chr2	157257145	157257415	1.35	5.45E-01	NR4A2	-67993	GPD2	-35620
chr12	53553107	53553476	0.83	5.45E-01	CSAD	21138	SOAT2	56018
chr7	26007726	26008091	1.09	5.45E-01	NPVF	-739804	NFE2L3	-183938
chr5	158202332	158202610	0.72	5.46E-01	CLINT1	-916303	EBF1	324317
chr22	21921773	21922073	1.23	5.46E-01	UBE2L3	-34		
chr9	130746143	130746514	0.85	5.46E-01	DPM2	-45566	NAIF1	83270
chr2	85645647	85645887	1.11	5.46E-01	SH2D6	-16151	CAPG	-8091
chr3	121379565	121379843	1.23	5.46E-01	HCLS1	87		

chr12	55378441	55378797	0.89	5.46E-01	NEUROD4	-35110	MUCL1	130320
chr3	170131945	170132239	1.51	5.46E-01	CLDN11	-4561		
chr9	35103018	35103373	0.85	5.46E-01	STOML2	-42		
chr1	42039800	42040077	0.85	5.46E-01	EDN2	-89595	HIVEP3	344557
chr14	50328810	50329059	0.87	5.46E-01	ARF6	-30801	SDCCAG1	-9396
chr22	32058119	32058355	1.09	5.46E-01	YWHAH	-282242	PISD	-31427
chr22	23226176	23226435	0.76	5.47E-01	IGL@	-3654		
chr2	74734226	74734672	1.30	5.48E-01	LBX2	-4006	PCGF1	372
chr11	128480806	128481265	0.90	5.48E-01	FLI1	-82777	ETS1	-23583
chr15	55940604	55940911	1.23	5.48E-01	PYGO1	-59708	PRTG	94419
chr17	27309256	27309627	0.93	5.48E-01	PHF12	-30934	SEZ6	23639
chr4	78077722	78078505	0.81	5.48E-01	CCNG2	-243		
chr12	125401698	125402039	1.30	5.48E-01	UBC	-2292		
chr19	46389660	46390314	0.88	5.48E-01	IRF2BP1	-611		
chr7	66385762	66386247	0.81	5.48E-01	C7orf42	-198		
chr16	81512590	81512997	1.33	5.48E-01	PLCG2	-300136	CMIP	34019
chr17	67411155	67411504	1.21	5.49E-01	MAP2K6	492		
chr4	89205817	89206130	1.10	5.49E-01	PPM1K	-86		
chr19	7985073	7985339	1.07	5.50E-01	SNAPC2	-23		
chr19	11266323	11266980	1.18	5.50E-01	SPC24	-168		
chr2	68585133	68585422	1.18	5.51E-01	CNRIP1	-38095	PLEK	-7044
chr6	10747651	10748191	0.84	5.51E-01	TMEM14B	-74		
chr6	52929526	52930041	0.83	5.51E-01	ICK	-3184	FBXO9	-12
chr1	713887	714210	1.17	5.51E-01	SAMD11	-147072	OR4F16	-92015
chr3	52035573	52035883	0.88	5.51E-01	RPL29	-5770	DUSP7	54733
chr7	148823374	148823684	1.25	5.51E-01	ZNF425	-151		
chr22	42353674	42353957	1.18	5.52E-01	SEPT3	-19115	CENPM	-10668
chr19	36485844	36486225	1.24	5.52E-01	SDHAF1	-66		
chr1	109633168	109633493	1.29	5.52E-01	TMEM167B	-72		
chr17	40086568	40086927	1.19	5.52E-01	TTC25	-140		
chr17	42402128	42402420	0.98	5.52E-01	SLC25A39	-57		
chr19	6530806	6531175	1.16	5.52E-01	TNFSF9	-19		
chr20	37101147	37101457	1.19	5.52E-01	RALGAPB	-184		
chr11	61648014	61648250	0.80	5.52E-01	FADS3	10874	FADS2	52419
chr12	1905178	1905601	1.18	5.52E-01	LRTM2	-24043	ADIPOR2	105143
chr3	195577769	195578080	1.23	5.52E-01	MUC4	-38777	TNK2	44507
chr7	47710317	47710730	0.76	5.52E-01	TNS3	-131325	C7orf69	-124365
chr10	102027286	102027607	1.16	5.53E-01	CWF19L1	-10		

chr7	134855385	134855730	0.82	5.53E-01	C7orf49	-26		
chr11	62606956	62607243	1.17	5.53E-01	WDR74	528		
chr20	19997773	19998064	1.34	5.53E-01	NAT5	-18		
chr20	31560066	31560366	1.21	5.53E-01	SPAG4L	32023	EFCAB8	113487
chr18	44626684	44626946	1.15	5.53E-01	TCEB3B	-64827	HDHD2	50056
chr15	72519093	72519403	0.90	5.54E-01	PKM2	-24243	PKM2	4436
chr21	27107569	27107948	1.22	5.54E-01	ATP5J	206	GABPA	430
chr6	25962912	25963165	0.90	5.54E-01	TRIM38	-32		
chr9	6703996	6704373	0.85	5.54E-01	GLDC	-58493	KDM4C	-53456
chr3	186490443	186490877	1.23	5.55E-01	EIF4A2	-10701	KNG1	55540
chr22	43539295	43539629	1.18	5.55E-01	MCAT	-59		
chr9	35079828	35080217	1.25	5.55E-01	FANCG	-10		
chr11	74959690	74960022	0.95	5.55E-01	SLCO2B1	97816	ARRB1	103017
chr19	54618626	54618870	1.22	5.55E-01	PRPF31	-42	TFPT	307
chr7	92053836	92054144	0.77	5.56E-01	GATAD1	-22775	ANKIB1	178442
chr9	124082423	124082713	1.19	5.56E-01	GSN	20489	STOM	49977
chr7	99680163	99680490	1.28	5.56E-01	ZNF3	-956		
chr20	34330111	34330510	1.19	5.57E-01	RBM39	-118		
chr7	45808538	45808783	0.76	5.57E-01	IGFBP1	-119298	ADCY1	194536
chr1	53168769	53169191	0.87	5.57E-01	C1orf163	-4942		
chr19	15490253	15490623	1.14	5.57E-01	AKAP8	165		
chr4	185305346	185305778	1.21	5.57E-01	ENPP6	-166448	IRF2	90164
chr17	28804223	28804629	1.31	5.57E-01	GOSR1	0		
chr1	225632598	225632908	0.89	5.57E-01	LBR	-16969	ENAH	208092
chr16	85480952	85481388	1.14	5.57E-01	KIAA0182	-165754	KIAA0513	419760
chr4	103940593	103941038	1.17	5.58E-01	NHEDC1	60		
chr1	202976215	202976569	1.22	5.58E-01	TMEM183B	-142		
chr7	20239052	20239314	0.84	5.58E-01	TMEM196	-426779	MACC1	17830
chr16	70464694	70465028	1.16	5.58E-01	ST3GAL2	8130	DDX19B	131789
chr7	25002835	25003206	0.83	5.59E-01	DFNA5	-205938	OSBPL3	16739
chr18	49814833	49815095	1.04	5.59E-01	DCC	-51607		
chr3	31573226	31573673	0.90	5.60E-01	STT3B	-1041		
chr5	169010495	169011024	1.11	5.61E-01	CCDC99	122		
chr5	157621587	157621919	1.00	5.61E-01	CLINT1	-335585	EBF1	905035
chr17	37617483	37617804	1.19	5.61E-01	CRKRS	-95		
chr5	126083853	126084102	0.78	5.61E-01	LMNB1	-28855	PHAX	147345
chr19	41304016	41304447	1.24	5.61E-01	EGLN2	-1949		
chr6	31695344	31695708	0.85	5.62E-01	DDAH2	2513	C6orf25	4365

chr16	81870243	81870636	1.24	5.62E-01	PLCG2	57510	SDR42E1	174653
chr2	89039582	89039858	1.10	5.62E-01	O1/O11 and JK2	-850842	RPIA	48544
chr1	24307273	24307569	1.17	5.62E-01	SFRS13A	-600		
chr2	44588854	44589164	1.18	5.62E-01	PREPL	-2120		
chr20	271174	271566	1.18	5.62E-01	ZCCHC3	-6834	DEFB132	32993
chr7	55954986	55955258	0.71	5.62E-01	ZNF713	-25206	SEPT14	-24640
chr22	22549806	22550355	0.85	5.62E-01	TOP3B	-212934	VPREB1	-49119
chr17	73105902	73106177	0.98	5.63E-01	ARMC7	-42		
chr20	35089414	35089681	1.32	5.63E-01	MYL9	-80349	DLGAP4	94100
chr10	105726680	105726924	1.17	5.63E-01	SLK	-668		
chr11	62446450	62446793	1.16	5.63E-01	UBXN1	-95		
chr15	66789979	66790312	1.32	5.63E-01	SNAPC5	0		
chr2	8716875	8717258	1.29	5.63E-01	ID2	-104917		
chr2	60579657	60579893	0.88	5.63E-01	BCL11A	200858		
chr2	185463140	185463422	1.03	5.63E-01	ZNF804A	188		
chr19	33667795	33668153	1.25	5.63E-01	LRP3	-17625	GPATCH1	96188
chr6	24667068	24667407	0.84	5.63E-01	TTRAP	-123	ACOT13	-25
chr7	42971753	42972099	0.88	5.63E-01	PSMA2	-121	MRPL32	-13
chr20	34686259	34686607	1.20	5.63E-01	SCAND1	-144005	EPB41L1	-56229
chr6	90984182	90984493	0.78	5.63E-01	BACH2	22224	GJA10	380150
chr15	72668233	72668543	1.22	5.63E-01	HEXA	132		
chr21	43955996	43956315	1.18	5.63E-01	PDE9A	-117706	SLC37A1	36414
chr3	158519625	158519962	0.87	5.64E-01	MFSD1	-118		
chr7	44887967	44888237	0.80	5.64E-01	H2AFV	-377		
chr1	205257415	205257802	1.22	5.64E-01	NUAK2	33274	TMCC2	60518
chr1	173836930	173837289	1.24	5.64E-01	ZBTB37	-383		
chr6	36985999	36986243	0.83	5.64E-01	PIM1	-151801	FGD2	12698
chr17	3814571	3815025	1.14	5.65E-01	CAMKK1	-20761	P2RX1	5162
chr6	2842133	2842460	1.15	5.65E-01	SERPINB1	-216		
chr1	28844616	28844929	0.72	5.65E-01	RCC1	28		
chr2	118571902	118572349	0.91	5.66E-01	DDX18	-129		
chr6	31831350	31831837	0.74	5.66E-01	NEU1	-885		
chr5	43313671	43314034	0.79	5.66E-01	HMGCS1	-258		
chr14	35591616	35591966	0.89	5.66E-01	PPP2R3C	-272	KIAA0391	15
chr22	26908335	26908589	1.17	5.66E-01	TFIP11	-25		
chr1	67966182	67966492	0.83	5.66E-01	GADD45A	-184546	SERBP1	-70214
chr10	94516866	94517184	0.76	5.66E-01	EXOC6	-91200	HHEX	67344
chr11	6423976	6424254	1.30	5.66E-01	SMPD1	12460	APBB1	16529

chr15	74257106	74257416	1.19	5.66E-01	STOML1	27374	LOXL1	38472		
chr12	90103078	90103600	1.06	5.67E-01	ATP2B1	-53495				
chr11	61500899	61501235	1.21	5.67E-01	C11orf9	-19054	DAGLA	53157		
chr4	2965060	2965343	1.06	5.68E-01	GRK4	-141	NOP14	-84		
chr1	151138354	151138653	1.25	5.68E-01	LYSMD1	-134	SCNM1	-13		
chr3	121797467	121797712	1.24	5.68E-01	CASR	-104940	CD86	23369		
chr9	92864642	92864905	1.22	5.68E-01	DIRAS2	540334	GADD45G	644847		
chr13	41507728	41508052	0.76	5.68E-01	ELF1	85618	SLC25A15	144343		
chr12	104531866	104532142	1.09	5.68E-01	NFYB	36				
chr19	3185191	3185978	0.84	5.68E-01	NCLN	-290				
chr4	82556876	82557186	0.86	5.68E-01	RASGEF1B	-163970	HNRNPD	738118		
chr12	49110425	49110786	1.27	5.68E-01	CCNT1	175				
chr5	122847750	122848117	0.98	5.69E-01	CSNK1G3	141				
chr14	67955286	67955542	1.13	5.69E-01	PLEK2	-76586	TMEM229B	26607		
chr14	95794171	95794546	1.11	5.69E-01	CLMN	-8114	C14orf49	147814		
chr2	91818607	91818949	1.10	5.69E-01	FKSG73	-310381				
chr19	6801461	6801754	0.97	5.69E-01	EMR1	-85974	VAV1	28886		
chr14	96000797	96001274	1.12	5.70E-01	GLRX5	-287				
chr12	57505935	57506405	0.77	5.70E-01	STAT6	-1009				
chr19	56904885	56905296	1.23	5.70E-01	ZNF582	-202				
chr9	35071954	35072235	0.98	5.70E-01	VCP	644				
chr16	3179573	3179883	0.88	5.71E-01	ZNF213	-5329	ZNF205	17165		
chr19	50320565	50320875	0.75	5.71E-01	FUZ	-4252	MED25	-826		
chr2	192489906	192490242	1.08	5.71E-01	OBFC2A	-52787	MYO1B	379967		
chr19	12992191	12992516	1.11	5.71E-01	DNASE2	-19				
chr1	220219877	220220135	1.33	5.71E-01	EPRS	-6				
chr14	64212705	64213097	1.16	5.71E-01	SYNE2	-106782	SGPP1	-18145		
chr8	59956329	59956625	1.00	5.71E-01	NSMAF	-384511	TOX	75290		
chr6	12008678	12009063	0.90	5.71E-01	HIVEP1	-3853				
chr11	65186141	65186791	1.21	5.71E-01	SCYL1	-106082	FRMD8	32425		
chr19	36630453	36631059	1.25	5.71E-01	CAPNS1	-162				
chr6	33538551	33538832	1.34	5.71E-01	BAK1	9378	ZBTB9	116336		
chr2	241524054	241524454	0.89	5.71E-01	CAPN10	-1891				
chr1	155057185	155057495	0.92	5.71E-01	EFNA1	-43009	EFNA3	5992		
chr4	25915739	25916066	1.08	5.72E-01	C4orf52	89				
chr16	69373376	69373686	1.13	5.72E-01	NIP7	-15	COG8	-5		
chr19	17416102	17416591	1.16	5.72E-01	ABHD8	-2065	MRPL34	-130		
chr19	44079638	44079967	1.10	5.72E-01	BC071811	-4893	AK296854	-1462	XRCC1	-73

chr6	188807	189140	1.19	5.72E-01	DUSP22	-103127		
chr7	44924970	44925355	0.88	5.72E-01	PURB	-203		
chr19	18049103	18049347	0.82	5.72E-01	KCNN1	-12886	SLC5A5	66443
chr3	130465503	130465946	1.22	5.73E-01	PIK3R4	-29		
chr10	127730328	127730740	0.78	5.73E-01	FANK1	145426	ADAM12	346593
chr11	118798749	118799499	1.21	5.73E-01	UPK2	-27902	BCL9L	-17511
chr10	27088335	27088635	1.20	5.73E-01	ABI1	61474	PDSS1	101890
chr19	10827926	10828657	0.82	5.74E-01	DNM2	-463		
chr1	203294088	203294646	0.79	5.74E-01	BTG2	19703	FMOD	25922
chr1	156474747	156475070	1.30	5.74E-01	MEF2D	-4380		
chr12	92839084	92839467	1.10	5.74E-01	BTG1	-299603	PLEKHG7	-290989
chr2	11606271	11606530	0.83	5.74E-01	E2F6	-104		
chr2	64276758	64277165	1.15	5.74E-01	VPS54	-30748	PELI1	94643
chr6	53213764	53214085	0.89	5.75E-01	ELOVL5	17		
chr1	169763981	169764293	1.24	5.75E-01	C1orf156	-106		
chr3	64009056	64009332	1.38	5.75E-01	PSMD6	-74		
chr3	187696442	187697009	0.84	5.75E-01	LPP	-233995	BCL6	-233251
chr10	6128304	6128711	1.05	5.76E-01	RBM17	-2441		
chr4	81796848	81797105	1.33	5.76E-01	BMP3	-155142	FGF5	609235
chr1	24285370	24285805	0.95	5.76E-01	PNRC2	-713		
chr1	23851504	23851927	0.98	5.76E-01	ASAP3	-40966	E2F2	5997
chr10	97849742	97850143	0.88	5.76E-01	ZNF518A	-39529	CCNJ	46784
chr3	13503114	13503424	0.84	5.76E-01	NUP210	-41460	HDAC11	-18446
chr3	187457782	187458196	1.20	5.76E-01	RTP2	-37644	BCL6	5486
chr14	68750177	68750465	0.93	5.76E-01	RAD51L1	463812	ZFP36L1	509464
chr19	6393713	6394093	1.07	5.77E-01	GTF2F1	-612		
chr11	7533776	7534061	1.17	5.77E-01	PPFIBP2	-1082		
chr5	68389560	68389860	1.26	5.77E-01	SLC30A5	-108		
chr9	34126568	34126942	1.12	5.77E-01	DCAF12	16		
chr2	109252824	109253147	0.79	5.77E-01	RANBP2	-82951	LIMS1	48219
chr19	2171324	2171634	0.91	5.78E-01	SF3A2	-65337	DOT1L	7331
chr19	2236596	2236906	1.18	5.78E-01	SF3A2	-65	PLEKHJ1	200
chr14	91708267	91708577	1.37	5.78E-01	GPR68	11802	C14orf159	127443
chr2	219757407	219757719	1.11	5.78E-01	CDK5R2	-66835	WNT10A	12308
chr19	6528023	6528533	0.88	5.79E-01	TNFSF9	-2732		
chr8	84230218	84230567	0.78	5.79E-01	RALYL	-866707		
chr2	38978338	38978715	1.19	5.79E-01	SFRS7	109		
chr16	88860	89170	0.99	5.79E-01	WASH1	-19567	POLR3K	14610

chr16	53132910	53133210	1.07	5.79E-01	RBL2	-335291	CHD9	44115
chr12	122750882	122751466	1.24	5.80E-01	VPS33A	-106		
chr11	121238295	121238645	0.93	5.80E-01	SORL1	-84491	SC5DL	75082
chr3	52259648	52259924	1.14	5.80E-01	TLR9	5461	ALAS1	27670
chr7	105697392	105697702	0.79	5.80E-01	SYPL1	55510	FLJ23834	93890
chr7	99036354	99036622	0.85	5.80E-01	CPSF4	-75	PTCD1	-69
chr17	25544655	25544995	1.27	5.81E-01	WSB1	-76281		
chr17	74477214	74477524	1.16	5.81E-01	AANAT	13718	RHBDF2	20139
chr10	27530991	27531300	0.87	5.81E-01	ACBD5	-1338		
chr14	75745080	75745496	0.77	5.81E-01	FOS	-193		
chr14	65672158	65672484	1.22	5.81E-01	FUT8	-207214	MAX	-103094
chr12	7341980	7342404	1.08	5.82E-01	PEX5	-766		
chr12	69863879	69864282	0.75	5.82E-01	FRS2	-48		
chr3	195163909	195164221	1.06	5.82E-01	ACAP2	-248		
chr12	112856446	112856759	1.05	5.82E-01	PTPN11	67		
chr2	39348082	39348393	1.10	5.83E-01	SOS1	-634		
chr19	39360457	39360789	1.16	5.83E-01	HNRNPL	-20006	RINL	8271
chr3	129295629	129296093	0.82	5.83E-01	PLXND1	29721	H1FOO	33804
chr4	90238093	90238340	1.39	5.83E-01	TIGD2	204249	SNCA	520133
chr17	20946689	20947086	0.81	5.83E-01	USP22	-536		
chr4	17812313	17812665	1.04	5.83E-01	DCAF16	-108	NCAPG	-36
chr19	9938343	9938703	1.19	5.83E-01	UBL5	-45		
chr7	30323719	30324098	1.10	5.83E-01	ZNRF2	-14		
chr10	35668028	35668520	0.87	5.83E-01	GJD4	-226064	CCNY	42472
chr7	150725693	150725937	1.26	5.83E-01	ATG9B	-4229	ABCB8	305
chr16	88522470	88522833	0.75	5.83E-01	ZC3H18	-114137	ZFPM1	2638
chr6	125918771	125919050	1.39	5.83E-01	HDDC2	-295629	HEY2	-151821
chr3	142297550	142297966	1.26	5.83E-01	ATR	-90		
chr5	131892336	131892738	1.22	5.83E-01	RAD50	-93		
chr7	129007443	129007718	0.77	5.84E-01	NRF1	-243974	AHCYL2	142717
chr19	17447996	17448415	1.28	5.84E-01	ANO8	-2568	GTPBP3	-150
chr21	45579650	45579960	0.91	5.84E-01	C21orf33	26311	ICOSLG	81029
chr2	60777456	60777908	1.12	5.84E-01	BCL11A	2951		
chr20	34542215	34542597	1.23	5.84E-01	SCAND1	22		
chr16	57682983	57683293	0.76	5.84E-01	GPR97	-19019	GPR56	29228
chr8	42645440	42645717	1.03	5.84E-01	CHRNA6	-21960	THAP1	52895
chr11	73472161	73472442	1.19	5.84E-01	RAB6A	-101		
chr5	1316438	1316802	0.87	5.85E-01	TERT	-21458	CLPTM1L	28382

chr9	36190608	36190946	0.87	5.85E-01	CLTA	-115		
chr7	72971798	72972238	1.05	5.85E-01	BCL7B	6		
chr20	31331644	31331954	1.30	5.85E-01	COMMD7	15		
chr5	168006539	168006990	1.21	5.85E-01	PANK3	-177		
chr1	169336946	169337209	1.08	5.85E-01	BLZF1	-116	NME7	108
chr3	57102571	57102872	1.19	5.85E-01	ERC2	-600331	ARHGEF3	10614
chr5	176856322	176856673	1.11	5.85E-01	PRR7	-17298	GRK6	2811
chr10	5726458	5726816	0.83	5.85E-01	ASB13	-18079	GDI2	128875
chr16	28857608	28857945	1.16	5.85E-01	TUFM	-48		
chr4	129730657	129731003	1.18	5.86E-01	PHF17	51		
chr12	21654336	21654767	1.23	5.86E-01	GOLT1B	-147	RECQL	51
chr7	100034000	100034455	0.85	5.86E-01	MEPCE	6699	TSC22D4	42674
chr13	31019828	31020086	0.72	5.86E-01	KATNAL1	-138794	HMGB1	20124
chr14	102785841	102786151	1.10	5.87E-01	ZNF839	-100		
chr17	1101381	1101695	0.82	5.87E-01	TUSC5	-81419	ABR	-18407
chr7	2276925	2277254	0.88	5.87E-01	NUDT1	-4767	MAD1L1	-4507
chr15	65185781	65186601	0.82	5.87E-01	PIF1	-68353	ANKDD1A	-17910
chr9	68726301	68726546	1.25	5.87E-01	FOXD4L6	475780	ANKRD20A3	799663
chr11	105892994	105893244	1.15	5.88E-01	KIAA1826	-165		
chr16	85061004	85061385	1.05	5.88E-01	KIAA0513	-215		
chr21	43916252	43916583	0.89	5.88E-01	SLC37A1	-3324	RSPH1	-17
chr21	45563325	45563570	0.81	5.88E-01	C21orf33	9954	ICOSLG	97386
chr15	49103400	49103644	1.19	5.88E-01	CEP152	-338		
chr15	55611272	55611604	0.92	5.88E-01	PIGB	305		
chr12	95397408	95397677	1.06	5.88E-01	NDUFA12	-32		
chr3	127794474	127794774	1.11	5.88E-01	SEC61A1	23412	RUVBL1	48047
chr3	14822465	14822741	0.72	5.88E-01	FGD5	-37866	C3orf20	105949
chr3	121468583	121468981	1.18	5.89E-01	GOLGB1	-180		
chr2	122288375	122288801	1.31	5.90E-01	TFCP2L1	-245810	CLASP1	118464
chr2	170550920	170551273	1.04	5.90E-01	KLHL23	122	PHOSPHO2	122
chr20	46377580	46377877	0.78	5.90E-01	SULF2	37079	NCOA3	247072
chr10	126480369	126480613	0.81	5.90E-01	METTL10	-52		
chr1	199212662	199212901	1.21	5.90E-01	NR5A2	-783988	PTPRC	604645
chr21	11109027	11109317	1.14	5.90E-01	BAGE	-10235		
chr7	127849865	127850131	0.79	5.90E-01	LRRC4	-178996	LEP	-31333
chr19	38826118	38826653	0.92	5.90E-01	CATSPERG	-57		
chr15	101463250	101463619	1.29	5.90E-01	LRRK1	3975	CHSY1	328691
chr3	190335066	190335321	1.30	5.90E-01	IL1RAP	103303	LOC647309	245271

chr22	30787163	30787476	0.81	5.91E-01	RNF215	-4018		
chr22	41697284	41697715	0.93	5.91E-01	ZC3H7B	-67		
chr19	42636808	42637115	1.30	5.91E-01	POU2F2	-332		
chr8	33370981	33371276	1.38	5.91E-01	MAK16	28444	RNF122	53514
chr6	31774564	31774840	1.30	5.91E-01	LSM2	41		
chr15	80216217	80216575	1.09	5.91E-01	MTHFS	-27026	BCL2A1	47247
chr3	57741836	57742170	1.22	5.91E-01	SLMAP	-1171		
chr17	21022970	21023235	1.14	5.92E-01	USP22	-76751	DHRS7B	-7155
chr6	26031765	26032614	1.20	5.92E-01	HIST1H4B	-4710	HIST1H3B	98
chr6	26313178	26313533	0.83	5.92E-01	BTN3A2	-52042	HIST1H4H	-27629
chr6	26473971	26474281	0.95	5.92E-01	BTN1A1	-27369	BTN2A1	15937
chr7	75943557	75943915	0.76	5.92E-01	HSPB1	11861	YWHAG	44606
chr7	98062645	98062928	0.93	5.92E-01	NPTX2	-183810	BAIAP2L1	-32360
chr17	29136011	29136292	1.23	5.92E-01	CRLF3	15626	LRRC37B2	200704
chr19	39108588	39108954	1.15	5.92E-01	EIF3K	-951	MAP4K1	-128
chr2	28113195	28113688	1.26	5.92E-01	RBKS	-219	BRE	-40
chr20	33999632	33999955	1.24	5.92E-01	UQCC	39		
chr1	115323132	115323446	1.23	5.92E-01	SIKE1	19		
chr19	15947600	15948094	0.93	5.92E-01	OR10H1	-28911	CYP4F2	61037
chr1	28201753	28202007	1.10	5.92E-01	C1orf38	2825	RPA2	39356
chr6	26157292	26157627	0.88	5.92E-01	HIST1H2BD	-889	HIST1H1E	901
chr1	36852144	36852396	0.79	5.93E-01	STK40	-785		
chr6	111195811	111196140	0.90	5.93E-01	AMD1	-11		
chr1	234508993	234509523	1.32	5.93E-01	C1orf31	-171		
chr16	89556974	89557355	0.97	5.93E-01	ANKRD11	-196		
chr19	46320387	46320765	1.05	5.93E-01	RSHL1	-1999		
chr3	38038274	38038627	1.44	5.93E-01	VILL	3373	PLCD1	27827
chr3	48672596	48673336	0.84	5.93E-01	TMEM89	-13777	CELSR3	37015
chr2	238512326	238512585	0.84	5.94E-01	LRRFIP1	-88351	RAB17	-12720
chr2	152266227	152266537	0.91	5.94E-01	RIF1	-46		
chr19	50879424	50879739	1.11	5.94E-01	NR1H2	90		
chr2	33824280	33824634	0.99	5.94E-01	RASGRP3	85515	tmp_locus_39	128827
chr1	115124175	115124577	1.17	5.94E-01	BCAS2	-111		
chr13	41345234	41345561	0.94	5.94E-01	MRPS31	-51		
chr2	30934519	30934829	1.24	5.94E-01	CAPN13	95637	LCLAT1	264537
chr7	129710053	129710430	1.24	5.94E-01	ZC3HC1	-19009	TMEM209	135096
chr5	148930944	148931248	1.11	5.95E-01	CSNK1A1	-89		
chr3	194412818	194413128	0.84	5.95E-01	LSG1	-19767	C3orf21	578922

chr17	16120324	16120663	1.23	5.95E-01	NCOR1	-1649	PIGL	-15
chr13	42845778	42846123	1.28	5.95E-01	AKAP11	-338		
chr19	4182428	4182769	1.18	5.95E-01	SIRT6	-3		
chr9	5437703	5438246	0.82	5.95E-01	C9orf46	-115		
chr6	27759958	27760288	1.13	5.95E-01	ZNF184	-319226	HIST1H2BL	15586
chr16	28565088	28565468	1.11	5.95E-01	CCDC101	29		
chr11	76391920	76392164	0.80	5.95E-01	LRRC32	-10998	GUCY2E	40791
chr9	116569765	116570134	1.15	5.95E-01	ZNF618	-68612	RGS3	362939
chr11	125439057	125439332	1.20	5.95E-01	EI24	-103		
chr17	56415278	56415731	1.18	5.95E-01	BZRAP1	-9353	SUPT4H1	14058
chr10	102133028	102133354	1.12	5.95E-01	WNT8B	-89621	SCD	26419
chr18	30502857	30503180	0.77	5.95E-01	ASXL3	-655522	KLHL14	-150045
chr2	144998474	144998878	0.93	5.95E-01	GTDC1	53384		
chr15	34659667	34660037	0.84	5.95E-01	LPCAT4	-457		
chr15	99791364	99791657	1.34	5.95E-01	TTC23	-1696	LRRC28	-141
chr6	41747628	41747941	0.85	5.95E-01	PRICKLE4	-715	FRS3	-155
chr7	150102327	150102607	1.02	5.95E-01	GIMAP8	-45495	ZNF775	26061
chr9	136819976	136820466	0.99	5.95E-01	SARDH	-216742	VAV2	37225
chr2	10262727	10263078	0.84	5.96E-01	RRM2	168		
chr2	27309403	27309728	1.01	5.96E-01	KHK	-45		
chr1	171454405	171454739	1.23	5.96E-01	BAT2D1	-94		
chr10	16859571	16859865	1.05	5.97E-01	RSU1	-265		
chr3	127872003	127872390	0.90	5.97E-01	EEFSEC	-116		
chr19	633490	633810	1.14	5.97E-01	POLRMT	-82		
chr5	173042913	173043195	1.28	5.98E-01	BOD1	612		
chr11	82996962	82997615	1.33	5.98E-01	CCDC90B	88		
chr12	122326359	122326658	1.21	5.98E-01	PSMD9	-137		
chr12	124068839	124069083	1.03	5.98E-01	TMED2	-115		
chr14	104387767	104388053	1.16	5.98E-01	C14orf2	-7		
chr18	19192025	19192598	0.79	5.98E-01	SNRPD1	52		
chr7	43769043	43769312	0.87	5.98E-01	C7orf44	-95		
chr7	72299769	72300082	1.13	5.98E-01	TYW1B	-1113		
chr12	25173523	25173873	1.24	5.98E-01	BCAT1	-71390	LRMP	-31543
chr12	98897154	98897473	1.26	5.98E-01	AX747640	-46391	TMPO	-12095
chr17	55628735	55629257	1.18	5.98E-01	MSI2	295065	MRPS23	298403
chr21	43288441	43288808	0.90	5.98E-01	RIPK4	-101376	PRDM15	10966
chr3	183254023	183254515	1.24	5.98E-01	MCF2L2	-108414	KLHL6	19230
chr21	42693090	42693496	1.16	5.98E-01	MX2	-40657	FAM3B	4632

chr19	36980300	36980772	1.23	5.98E-01	ZNF566	-73		
chr2	201390599	201391195	0.93	5.98E-01	SGOL2	32		
chr19	59070778	59071270	1.24	5.99E-01	CHMP2A	-4538	UBE2M	-681
chr7	143824870	143825114	1.26	5.99E-01	OR2A14	-1214		
chr16	790922	791210	1.19	5.99E-01	NARFL	-69		
chr1	32429649	32429996	1.03	5.99E-01	KHDRBS1	-49668	PTP4A2	-25835
chr1	112289909	112290219	0.96	5.99E-01	DDX20	-8126	RAP1A	127659
chr19	13263615	13264038	0.80	5.99E-01	STX10	-2840	IER2	2545
chr22	39101562	39101882	1.06	5.99E-01	GTPBP1	-85		
chr7	105229953	105230226	1.17	5.99E-01	FLJ23834	-373567	RINT1	57558
chr9	137029433	137029768	1.24	5.99E-01	RXRA	-188715	WDR5	28391
chr15	70390042	70390356	1.12	5.99E-01	TLE3	57		
chr16	90014490	90014862	0.86	5.99E-01	DEF8	-475		
chr1	111756968	111757380	0.82	5.99E-01	DRAM2	-74336	CHI3L2	-13107
chr15	34635174	34635547	0.88	5.99E-01	C15orf55	-2705	NOP10	1
chr15	64302950	64303336	1.13	6.00E-01	HERC1	-176996	DAPK2	35378
chr17	42785835	42786145	1.28	6.00E-01	DBF4B	14		
chr19	4724543	4724874	1.24	6.00E-01	DPP9	-854		
chr3	101441274	101441584	1.20	6.00E-01	CEP97	-2065		
chr12	120729402	120730084	1.36	6.00E-01	PXN	-26180	SIRT4	-10420
chr13	114103347	114103927	0.94	6.00E-01	GRTP1	-85174	ADPRHL1	4202
chr16	30361880	30362167	0.84	6.00E-01	CD2BP2	4658	SULT1A3	151488
chr17	76704598	76704898	1.16	6.00E-01	DNAH17	-137344	CYTH1	73628
chr1	148556596	148556886	1.31	6.00E-01	NBPF15	-4106		
chr14	77923819	77924228	1.11	6.00E-01	AHSA1	-349	C14orf133	-120
chr7	73631996	73632385	1.10	6.00E-01	LAT2	8104	RFC2	36547
chr8	103666219	103666501	0.80	6.00E-01	KLF10	1623	ODF1	102512
chr10	46951327	46951603	1.09	6.00E-01	FRMPD2L1	-275762	SYT15	19136
chr1	230777968	230778259	0.97	6.00E-01	COG2	-88		
chr14	23590072	23590319	1.12	6.00E-01	CEBPE	-1722		
chr17	41400890	41401190	0.72	6.00E-01	ARL4D	-75313	TMEM106A	37146
chr2	65609929	65610189	0.89	6.00E-01	SPRED2	49597	ACTR2	155230
chr2	86263151	86263509	1.14	6.00E-01	ST3GAL5	-147173	POLR1A	69948
chr3	13087871	13088217	1.04	6.00E-01	RPL32	-206095	IQSEC1	26573
chr15	75400915	75401199	1.12	6.01E-01	PPCDC	85130	DNM1P33	194249
chr7	102937686	102937971	0.83	6.01E-01	PMPCB	-44		
chr15	85471485	85471737	0.75	6.02E-01	PDE8A	-53594	SLC28A1	43698
chr9	5626923	5627577	0.84	6.02E-01	KIAA1432	-29333	PDCD1LG2	116705

chr3	72212949	72213391	0.84	6.02E-01	PROK2	-378813	RYBP	282604
chr1	45205278	45205619	1.14	6.02E-01	KIF2C	-41		
chr14	23790065	23790470	1.08	6.02E-01	PABPN1	871		
chr2	86448227	86448474	0.81	6.02E-01	MRPL35	21795	REEP1	116426
chr14	103544663	103544907	1.05	6.03E-01	TNFAIP2	-47879	CDC42BPB	-21043
chr10	27389116	27389591	0.88	6.04E-01	ABI1	-239395	YME1L1	53967
chr15	74753320	74753844	1.22	6.04E-01	UBL7	-53		
chr3	38388061	38388356	1.09	6.04E-01	XYLB	-42		
chr3	52029803	52030132	1.30	6.04E-01	RPL29	-10		
chr4	39979457	39979772	1.02	6.04E-01	PDS5A	-132		
chr5	139554344	139554712	1.05	6.04E-01	C5orf53	49007	PFDN1	128161
chr13	37574700	37575147	0.94	6.04E-01	ALG5	-1420	EXOSC8	246
chr10	89867978	89868387	1.25	6.05E-01	KILLIN	-244989	RNLS	474899
chr4	146859928	146860238	0.96	6.05E-01	ZNF827	-476		
chr3	20227671	20227945	0.91	6.05E-01	SGOL1	-125		
chr5	72861236	72861703	1.09	6.05E-01	UTP15	-128	ANKRA2	28
chr5	25955551	25955833	1.36	6.05E-01				
chr10	65801007	65801275	0.75	6.05E-01	REEP3	520018		
chr1	167178320	167178630	0.94	6.05E-01	POU2F1	-11668	DUSP27	114404
chr15	73928832	73929111	1.14	6.06E-01	NPTN	-3219		
chr19	39832509	39832827	1.22	6.06E-01	SAMD4B	-440		
chr12	53715228	53715576	1.15	6.06E-01	AAAS	10		
chr11	124823807	124824246	0.97	6.06E-01	SLC37A2	-108986	HEPACAM	-17719
chr19	47777393	47777842	1.06	6.06E-01	PRR24	-524		
chr16	30672074	30672318	1.08	6.06E-01	FBRS	-3582		
chr9	100395348	100395810	1.13	6.06E-01	NCBP1	-126		
chr15	91497906	91498177	1.12	6.06E-01	UNC45A	19827	PRC1	39762
chr5	5422368	5422762	0.92	6.06E-01	ADAMTS16	282122	MED10	956074
chr1	151299919	151300229	0.82	6.07E-01	PI4KB	-1225		
chr12	69753355	69753599	1.17	6.07E-01	YEATS4	-55		
chr2	33700785	33701073	1.32	6.07E-01	RASGRP3	-38013	LTBP1	528537
chr3	121264744	121265032	1.21	6.07E-01	POLQ	-35		
chr13	45903812	45904100	1.18	6.08E-01	KCTD4	-128781	TPT1	11341
chr19	8009138	8009524	0.81	6.08E-01	TIMM44	-793		
chr4	190861803	190862080	0.80	6.08E-01	FRG1	-32		
chr10	49880372	49880731	0.81	6.09E-01	ARHGAP22	-67414	WDFY4	-12966
chr1	155197212	155197690	1.12	6.09E-01	GBA	13602	MTX1	18961
chr7	72395440	72395714	0.84	6.09E-01	POM121	-83		

chr1	149223568	149224358	1.40	6.09E-01	PPIAL4B	-329040	NBPF16	484521
chr8	61705460	61705800	0.86	6.09E-01	RLBP1L1	-494895	CHD7	114291
chr14	23025099	23025675	1.16	6.09E-01	DAD1	32756	TCRDV2	97298
chr19	54641116	54641437	0.98	6.09E-01	CNOT3	-172		
chr8	99129355	99129625	1.34	6.09E-01	HRSP12	-72	POP1	-31
chr17	76627930	76628339	1.37	6.09E-01	DNAH17	-60731	CYTH1	150241
chr16	23464365	23464676	1.05	6.10E-01	COG7	-18		
chr19	18439173	18439468	0.84	6.10E-01	PGPEP1	-12087	LSM4	-5320
chr12	57472475	57472711	0.78	6.10E-01	TMEM194A	-19		
chr1	1765473	1765749	0.88	6.10E-01	NADK	-55702	GNB1	56884
chr8	23103781	23104406	1.17	6.10E-01	R3HCC1	-41518	CHMP7	2944
chr19	46010531	46010881	0.94	6.10E-01	VASP	18		
chr3	51428401	51428839	1.23	6.11E-01	RBM15B	-111		
chr4	25314092	25314510	0.89	6.11E-01	ZCCHC4	-95		
chr15	69755059	69755419	1.09	6.11E-01	RPLP1	10080	TLE3	635017
chr1	67461756	67462066	0.83	6.11E-01	SLC35D1	58169	MIER1	71333
chr12	65015013	65015399	1.25	6.12E-01	RASSF3	10913	GNS	138020
chr10	13873892	13874202	0.67	6.12E-01	PRPF18	245108	FRMD4A	498819
chr9	126101546	126101927	1.12	6.12E-01	STRBP	-70894	CRB2	-16711
chr12	49454430	49454759	0.98	6.12E-01	MLL2	-5488	RHEBL1	9180
chr15	85197961	85198271	0.78	6.12E-01	NMB	3686	SCAND2	23425
chr14	82000178	82000575	1.04	6.12E-01	SEL1L	-172		
chr11	118069015	118069299	1.10	6.12E-01	SCN2B	-21820	AMICA1	26652
chr17	7746958	7747434	1.20	6.12E-01	TMEM88	-11188	KDM6B	3961
chr10	101380312	101380624	1.08	6.12E-01	SLC25A28	-247		
chr2	224810010	224810261	1.12	6.12E-01	WDFY1	-84		
chr1	67896128	67896502	1.23	6.12E-01	SERBP1	-192		
chr6	41754918	41755520	0.86	6.12E-01	TOMM6	719		
chr6	18359716	18360026	1.13	6.12E-01	DEK	-95072	RNF144B	-27723
chr6	18155402	18155865	0.77	6.12E-01	TPMT	-260	KDM1B	15
chr12	54891248	54891603	0.81	6.12E-01	NCKAP1L	-69		
chr1	192574170	192574512	1.22	6.12E-01	RGS13	-30941	RGS1	29484
chr11	47475826	47476102	1.14	6.12E-01	RAPSN	-5234	CUGBP1	34612
chr11	75946427	75946726	1.22	6.12E-01	WNT11	-29003	PRKRIR	145303
chr2	27273451	27274661	1.28	6.12E-01	AGBL5	-435		
chr14	23770610	23771052	1.06	6.12E-01	PABPN1	-18566	HOMEZ	-15522
chr20	1305786	1306096	0.85	6.12E-01	SDCBP2	3897	SNPH	58981
chr14	22855893	22856231	0.68	6.13E-01	TCRDV2	-72027	TRA	380298

chr16	88747503	88747813	1.19	6.13E-01	MVD	-18163	SNAI3	5224
chr9	4762577	4762858	1.39	6.13E-01	RCL1	-30116	AK3	-21491
chr1	39724383	39724644	0.80	6.13E-01	MACF1	-72296	NDUFS5	232508
chr10	2742867	2743177	1.28	6.13E-01	ADARB2	-963304	PFKP	-366730
chr1	146643823	146644440	0.89	6.14E-01	PRKAB2	-3		
chr1	12221666	12222175	0.90	6.14E-01	TNFRSF1B	-5139	TNFRSF8	98487
chr9	80911614	80912058	1.15	6.15E-01	PSAT1	-223		
chr8	22526599	22526921	1.33	6.15E-01	BIN3	-99		
chr1	155164591	155164842	0.87	6.15E-01	MUC1	-2017		
chr12	14310075	14310418	1.24	6.15E-01	ATF7IP	-208364	GRIN2B	-177225
chr2	10549250	10549578	1.37	6.15E-01	ODC1	39039	HPCAL1	106374
chr1	235292078	235292516	1.14	6.15E-01	TOMM20	-41		
chr1	153931068	153931320	1.26	6.15E-01	CRTC2	-151		
chr19	11670007	11670295	1.35	6.15E-01	ELOF1	-100		
chr6	157098277	157098586	0.94	6.15E-01	ARID1B	-654		
chr13	114894503	114894767	1.21	6.15E-01	GAS6	-327589	RASA3	3460
chr2	152685022	152685300	1.17	6.16E-01	ARL5A	-152		
chr7	138144947	138145320	0.79	6.16E-01	TRIM24	55		
chr19	50379745	50380484	1.23	6.16E-01	TBC1D17	-714	AKT1S1	497
chr15	90734829	90735363	1.15	6.16E-01	IDH2	-89388	SEMA4B	-9466
chr6	43351532	43351803	1.21	6.16E-01	ABCC10	-43624	ZNF318	-14487
chr7	35770030	35770461	1.18	6.17E-01	SEPT7	-70381	HERPUD2	-35474
chr6	30640661	30641030	0.89	6.17E-01	DHX16	-16		
chr1	161067435	161067788	1.37	6.17E-01	PVRL4	-8227	PFDN2	20254
chr16	57334263	57334657	1.24	6.17E-01	CCL22	-58258	PLLP	-15889
chr7	127877033	127877367	0.84	6.17E-01	LEP	-4131		
chr1	226186911	226187485	1.19	6.17E-01	H3F3B	-63223	LEFTY2	-58278
chr8	37259348	37260003	0.95	6.17E-01	ZNF703	-293625	FKSG2	513679
chr22	50319170	50319446	1.07	6.18E-01	PIM3	-34835	CRELD2	7025
chr2	74699671	74699981	0.97	6.19E-01	MRPL53	97		
chr6	150944692	150944996	0.92	6.19E-01	MTHFD1L	-241847	PLEKHG1	23845
chr15	101835371	101835630	1.26	6.19E-01	SNRPA1	-41		
chr16	3398585	3398865	1.21	6.19E-01	OR2C1	-7164	ZNF75A	43292
chr11	86748707	86748951	1.09	6.19E-01	TMEM135	-236		
chr13	46758364	46758841	0.93	6.19E-01	LCP1	-2144		
chr16	69220448	69220758	0.89	6.19E-01	SNTB2	-447		
chr8	33370556	33370823	1.09	6.19E-01	MAK16	28005	RNF122	53953
chr17	48225771	48226169	1.29	6.19E-01	PPP1R9B	1907	PDK2	53274

chr14	90420983	90421245	1.11	6.20E-01	TDP1	-1132	C14orf143	-25
chr8	42029167	42029471	0.92	6.20E-01	AP3M2	18855	PLAT	35875
chr1	198590056	198590396	1.34	6.20E-01	ATP6V1G3	-80151	PTPRC	-17911
chr14	75725770	75726272	0.83	6.20E-01	TMED10	-82672	FOS	-19460
chr15	50978626	50979411	1.16	6.20E-01	TRPM7	-25		
chr19	56092168	56092445	1.28	6.20E-01	ZNF579	-96		
chr15	75660238	75660648	1.15	6.21E-01	MAN2C1	498		
chr20	34078693	34079003	0.81	6.21E-01	ERGIC3	-50930	CEP250	35698
chr12	102137559	102137823	0.78	6.21E-01	SYCP3	-4444		
chr16	87736005	87736376	1.05	6.21E-01	KLHDC4	63351	JPH3	99692
chr22	22555640	22555950	1.16	6.21E-01	TOP3B	-218648	VPREB1	-43405
chr11	129872938	129873271	0.96	6.21E-01	PRDM10	-375		
chr2	48541325	48541761	1.12	6.21E-01	FOXN2	-252		
chr5	19270293	19270639	1.10	6.22E-01	CDH18	710821		
chr7	139044211	139044659	1.11	6.22E-01	LUC7L2	-199		
chr19	16222098	16222354	1.03	6.22E-01	RAB8A	-264		
chr12	14927012	14927474	1.29	6.23E-01	HIST4H4	-3178	H2AFJ	-27
chr7	104583904	104584453	0.99	6.23E-01	MLL5	-70458	LHFPL3	615075
chr3	196466499	196466940	1.15	6.24E-01	PAK2	-8		
chr5	180633690	180634036	1.32	6.24E-01	TRIM7	-1686		
chr15	81589113	81589423	1.24	6.24E-01	STARD5	27256	IL16	100049
chr16	4475363	4475662	1.18	6.24E-01	DNAJA3	-345		
chr10	98486664	98486922	0.67	6.24E-01	C10orf12	-254248	PIK3AP1	-6514
chr2	161993264	161993591	1.04	6.24E-01	TANK	-38		
chr2	202316094	202316416	1.14	6.24E-01	STRADB	-137	TRAK2	64
chr12	125424369	125424712	1.00	6.25E-01	UBC	-24964	DHX37	49126
chr17	57232521	57232900	1.17	6.25E-01	SKA2	89		
chr7	99933473	99933888	0.86	6.25E-01	PILRB	-21945	PVRIG	116809
chr6	13615384	13615694	0.93	6.26E-01	NOL7	-20		
chr8	90738083	90738329	1.20	6.26E-01	RIPK2	-31769		
chr21	40203497	40203745	1.07	6.26E-01	ETS2	25772	PSMG1	351819
chr22	24552895	24553281	0.86	6.26E-01	SUSD2	-24356	CABIN1	145323
chr17	25620939	25621516	1.13	6.26E-01	WSB1	122		
chr19	42772302	42772702	1.07	6.26E-01	CIC	-16315	ERF	-13193
chr6	26156464	26156844	0.90	6.26E-01	HIST1H2BD	-1695	HIST1H1E	95
chr7	41921231	41921613	1.28	6.26E-01	INHBA	-178716	GLI3	355196
chr3	133524427	133524886	1.11	6.26E-01	SRPRB	-20		
chr8	62627037	62627461	1.04	6.27E-01	ASPH	-50		

chr19	2051523	2051808	0.79	6.27E-01	MKNK2	-423		
chr12	7063017	7063370	1.14	6.27E-01	PTPN6	7454	PHB2	16722
chr12	95611390	95611678	1.06	6.27E-01	FGD6	-294	VEZT	12
chr7	156742227	156742471	1.00	6.28E-01	NOM1	-68		
chr22	43506437	43506755	0.79	6.28E-01	BIK	-161		
chr11	63706084	63706375	0.94	6.28E-01	NAT11	-212		
chr17	37310451	37310963	0.97	6.28E-01	PLXDC1	-2805		
chr18	43753347	43753702	1.17	6.28E-01	RNF165	-160662	HAUS1	69227
chr6	27777621	27778065	0.96	6.28E-01	HIST1H2BM	-4979	HIST1H2BL	-2134
chr11	4115849	4116203	0.86	6.29E-01	RRM1	102		
chr20	49407050	49407519	0.81	6.29E-01	BCAS4	-4182		
chrX	199843	200153	1.01	6.29E-01	PLCXD1	1937	GTPBP6	30889
chr1	45196358	45196861	1.36	6.29E-01	KIF2C	-8880	C1orf228	56216
chr11	506736	507020	1.13	6.29E-01	RNH1	-57		
chr7	106301306	106301904	1.19	6.29E-01	NAMPT	-375967	PIK3CG	-204319
chr16	84674846	84675118	1.11	6.29E-01	COTL1	-23313	KLHL36	-7149
chr2	15731813	15732159	0.91	6.29E-01	DDX1	216		
chr20	57226073	57226411	1.35	6.29E-01	STX16	-86		
chr5	49741123	49741420	0.90	6.30E-01	EMB	-4038		
chr9	37485475	37486103	0.80	6.30E-01	POLR1E	-156		
chr16	58664246	58664567	1.21	6.30E-01	CNOT1	-657		
chr12	57024050	57024360	0.86	6.31E-01	BAZ2A	5958	RBMS2	108596
chr8	125486626	125487061	1.06	6.31E-01	RNF139	-164		
chr17	61920413	61920704	1.05	6.31E-01	SMARCD2	-208		
chrX	47517890	47518298	1.01	6.31E-01	UXT	422		
chr1	167683766	167684094	1.38	6.31E-01	CREG1	-160874	MPZL1	-7257
chr20	18547810	18548185	1.21	6.31E-01	DTD1	-20558	SEC23B	59810
chr3	183967262	183967558	1.11	6.31E-01	ALG3	-651	ECE2	-35
chr19	2749185	2749520	1.18	6.31E-01	SLC39A3	-9279	SGTA	34001
chr4	39183708	39184111	1.16	6.32E-01	WDR19	-114		
chr1	40626846	40627090	0.83	6.32E-01	RLF	-73		
chr16	69599658	69599947	1.02	6.32E-01	NFAT5	806		
chr17	4634433	4634789	1.24	6.32E-01	MED11	-112		
chr7	90033011	90033311	1.26	6.32E-01	CLDN12	365		
chr11	64684804	64685135	1.01	6.32E-01	EHD1	-38779	PPP2R5B	-7210
chr20	10415704	10416175	1.26	6.32E-01	MKKS	-1074	C20orf94	-11
chr20	50211602	50211912	1.26	6.32E-01	NFATC2	-52499	ATP9A	173151
chr8	27224073	27224383	0.91	6.32E-01	PTK2B	41174	CHRNA2	112585

chr1	27070496	27070934	0.82	6.33E-01	PIGV	-43771	ARID1A	48193
chr11	64884953	64885628	1.21	6.33E-01	MRPL49	-4437	ZNHIT2	-121
chr16	67969598	67969851	0.87	6.33E-01	CTRL	-3960		
chr19	11485541	11485869	1.03	6.33E-01	EPOR	9314	LPPR2	19598
chr2	232646082	232646475	1.15	6.33E-01	COPS7B	-4883	PDE6D	-305
chr17	73401765	73402023	1.04	6.34E-01	GRB2	-104		
chr13	52389239	52389564	0.93	6.34E-01	CCDC70	-46715	DHRS12	-11109
chr19	2710426	2710774	1.19	6.34E-01	GNG7	-7854	DIRAS1	10790
chr12	68024553	68024818	0.89	6.34E-01	DYRK2	-17826	CAND1	361625
chr1	119683223	119683524	1.29	6.34E-01	WARS2	-79		
chr10	43278064	43278319	0.94	6.34E-01	BMS1	238		
chr2	209118984	209119389	0.93	6.34E-01	IDH1	619		
chr3	187465839	187466149	1.26	6.34E-01	BCL6	-2519		
chr5	32585511	32585833	0.94	6.34E-01	SUB1	67		
chr5	154237732	154238207	0.89	6.34E-01	CNOT8	-229		
chr8	77912186	77912541	0.90	6.34E-01	PXMP3	160		
chr9	37904179	37904507	1.18	6.34E-01	MCART1	7		
chr14	24768879	24769138	1.10	6.34E-01	C14orf21	-89	DHRS1	30
chr2	20795277	20795530	1.16	6.34E-01	HS1BP3	55460	RHOB	148569
chr4	26828351	26828811	1.25	6.34E-01	STIM2	-33783	TBC1D19	243035
chr7	30278614	30279132	0.85	6.34E-01	ZNRF2	-45050	PLEKHA8	210585
chr7	100485259	100485504	0.92	6.34E-01	UFSP1	1957	SRRT	12681
chr20	2644759	2645013	1.16	6.34E-01	IDH3B	-43		
chr6	28912245	28912505	1.10	6.34E-01	TRIM27	-20607	ZNF311	60660
chr17	56409710	56410298	1.26	6.34E-01	BZRAP1	-3852		
chr1	161195337	161195787	1.38	6.35E-01	APOA2	-2144	TOMM40L	-271
chr19	36505007	36505308	0.82	6.35E-01	ALKBH6	-17		
chr3	58340960	58341272	0.96	6.35E-01	PXK	22499	PDHB	78449
chr11	59051867	59052291	0.83	6.35E-01	OR5AN1	-79853	MPEG1	-71585
chr17	62002921	62003359	0.79	6.36E-01	GH1	-6942	CD79B	6564
chr12	27090814	27091221	1.14	6.36E-01	FGFR1OP2	-298	C12orf11	236
chr19	11071318	11071777	1.08	6.36E-01	SMARCA4	-50		
chr3	194207605	194208063	1.18	6.36E-01	ATP13A3	-18866	TMEM44	146279
chr6	170615584	170615957	1.05	6.37E-01	FAM120B	-73		
chr10	111672090	111672400	0.91	6.37E-01	XPNPEP1	10957		
chr7	107384432	107384741	1.05	6.37E-01	CBLL1	308		
chr1	26690350	26690612	0.87	6.37E-01	AIM1L	-20038	ZNF683	8785
chr1	27191384	27191754	1.17	6.37E-01	SFN	1936	GPN2	25300

chr1	198590581	198590992	1.03	6.37E-01	ATP6V1G3	-80712	PTPRC	-17350
chr21	44081894	44082158	1.10	6.37E-01	PDE9A	8164	WDR4	217652
chr11	62366677	62366987	1.01	6.38E-01	TUT1	-7723	MTA2	2471
chr22	31795270	31795750	1.11	6.38E-01	DRG1	-29		
chr2	201729280	201729606	1.20	6.38E-01	CLK1	24		
chr17	56421663	56422032	1.23	6.38E-01	BZRAP1	-15696	SUPT4H1	7715
chr2	54013994	54014296	1.14	6.38E-01	ERLEC1	77		
chr20	43594913	43595253	1.15	6.38E-01	STK4	-37		
chr1	203296387	203296772	0.89	6.38E-01	BTG2	21916	FMOD	23709
chr2	233989498	233989808	0.83	6.38E-01	ATG16L1	-170564	INPP5D	64617
chr3	47517328	47517728	1.13	6.38E-01	SCAP	-83		
chr3	58291726	58292110	1.14	6.38E-01	RPP14	-56	Rpp14	-56
chr5	180258521	180258849	1.01	6.39E-01	MGAT1	-28637	ZFP62	28999
chr10	14701719	14702275	0.95	6.39E-01	FRMD4A	-329131	CDNF	177986
chr11	128604268	128604639	1.18	6.39E-01	FLI1	40641	KCNJ1	107909
chr15	65426033	65426343	1.25	6.39E-01	PDCD7	-14		
chr6	28317583	28317866	0.99	6.39E-01	ZKSCAN3	34		
chr8	104427293	104427714	0.95	6.39E-01	SLC25A32	-36	DCAF13	562
chr5	36242074	36242602	0.87	6.39E-01	C5orf33	-438		
chr3	113464011	113464416	1.30	6.40E-01	ATP6V1A	-1694	NAT13	882
chr19	38085699	38086183	1.01	6.40E-01	ZNF571	-268		
chr6	17707067	17707418	1.15	6.40E-01	NUP153	-425		
chr14	77279173	77279456	0.88	6.40E-01	C14orf166B	-13410	VASH1	51080
chr4	48343112	48343511	0.81	6.40E-01	SLC10A4	-142048	TEC	-71498
chr12	57030574	57030939	0.84	6.40E-01	BAZ2A	-594		
chr19	17420079	17420418	1.10	6.40E-01	MRPL34	3772	ANO8	25389
chr1	1510153	1510413	1.01	6.40E-01	SSU72	-21		
chr11	64808315	64808676	0.84	6.40E-01	SAC3D1	120		
chr17	57184181	57184499	1.12	6.41E-01	TRIM37	-74		
chr19	2648513	2648919	1.17	6.41E-01	GNG7	54030	GADD45B	172581
chr11	9482440	9482821	0.84	6.41E-01	ZNF143	118		
chr17	41399851	41400501	0.66	6.41E-01	ARL4D	-76177	TMEM106A	36282
chr17	21030070	21030536	1.14	6.41E-01	DHRS7B	45		
chr1	175178301	175178693	1.24	6.41E-01	TNN	141503	TNR	534255
chr19	10362380	10362699	0.81	6.42E-01	MRPL4	-100		
chr15	89089818	89090140	1.14	6.42E-01	DET1	-67		
chr18	48346297	48346632	0.93	6.42E-01	MRO	-31		
chr21	45209073	45209539	1.16	6.42E-01	RRP1	-112		

chr3	195808710	195809150	1.17	6.42E-01	TFRC	102		
chr6	44214380	44215001	0.82	6.42E-01	HSP90AB1	-158		
chr12	31881737	31882302	1.05	6.42E-01	H3F3C	63155	C12orf72	69411
chr13	53542311	53542555	0.83	6.42E-01	PCDH8	-119659	OLFM4	-60539
chr19	45287575	45287892	0.88	6.42E-01	BCAM	-24604	CBLC	6608
chr2	197036162	197036455	1.05	6.43E-01	STK17B	27		
chr20	32077535	32077802	1.21	6.43E-01	CBFA2T2	-259		
chr14	20929676	20930111	1.11	6.43E-01	TMEM55B	-257		
chr9	33024967	33025245	0.85	6.43E-01	DNAJA1	-103		
chr9	123964096	123964515	0.82	6.43E-01	RAB14	59		
chr20	39591491	39591951	1.03	6.43E-01	MAFB	-273845	TOP1	-65741
chr8	30488529	30488778	0.83	6.44E-01	GTF2E2	27084	RBPMS	246710
chr5	43064892	43065285	0.81	6.44E-01	ZNF131	-56553	C5orf39	-24642
chr2	176032631	176033174	1.18	6.45E-01	ATF2	-6		
chr8	146012679	146012943	1.22	6.45E-01	ZNF34	-86		
chr1	212967875	212968296	1.16	6.45E-01	NSL1	-2947	TATDN3	2916
chr11	58347057	58347301	1.23	6.45E-01	LPXN	-3789	ZFP91	592
chr3	195641855	195642178	1.20	6.45E-01	TNK2	-19585	SDHALP1	75133
chr12	50505517	50505823	1.10	6.45E-01	C12orf62	-230		
chr19	19496403	19496713	0.86	6.45E-01	GATAD2A	-84		
chr16	88772712	88773002	1.05	6.45E-01	RNF166	-57	CTU2	-34
chr14	102783292	102783560	0.87	6.46E-01	ZNF839	-2670		
chr11	65274201	65274693	1.33	6.46E-01	SCYL1	-18101	FRMD8	120406
chr12	93389419	93389718	1.19	6.46E-01	NUDT4	-382132	EEA1	-66462
chr14	68746562	68746960	1.28	6.46E-01	RAD51L1	460252	ZFP36L1	513024
chr15	91477554	91477831	0.89	6.46E-01	HDDC3	-1919	UNC45A	-522
chr7	23221445	23221755	0.84	6.46E-01	NUPL2	154		
chr1	109655327	109655592	0.94	6.46E-01	KIAA1324	-1073		
chr5	96204652	96204969	1.24	6.46E-01	ERAP1	-60919	ERAP2	-6833
chr7	86781555	86781860	0.85	6.47E-01	DMTF1	31		
chr16	81772476	81772820	0.98	6.47E-01	PLCG2	-40282	CMIP	293873
chr1	209957729	209958073	1.17	6.47E-01	IRF6	21578	TRAF3IP3	28377
chr14	42898135	42898495	1.40	6.47E-01	LRFN5	821551		
chr14	71786714	71787088	1.24	6.47E-01	SIPA1L1	-209128	PCNX	412779
chr15	68347719	68348011	0.89	6.47E-01	PIAS1	1293	CALML4	150583
chr7	50187923	50188371	0.97	6.47E-01	IKZF1	-156231	ZPBP	-55287
chrX	12974279	12974653	1.41	6.47E-01	TMSB4X	-18759	TLR8	49708
chr19	42970025	42970282	0.85	6.47E-01	CXCL17	-23018	CEACAM1	62485

chr6	90067135	90067479	0.78	6.47E-01	UBE2J1	-4688		
chr10	5847683	5847996	1.20	6.47E-01	ASB13	-139282	GDI2	7672
chr12	125382973	125383284	0.92	6.47E-01	SCARB1	-34610	UBC	16448
chr12	82752087	82752627	1.19	6.47E-01	CCDC59	-158		
chr18	46455183	46455506	1.08	6.48E-01	SMAD7	21736	KIAA0427	389918
chr1	16970900	16971430	1.12	6.48E-01	NBPF1	-31183	MSTP9	119810
chr11	43702014	43702373	0.98	6.48E-01	HSD17B12	51		
chr6	64308539	64308821	1.17	6.49E-01	PHF3	-47752	PTP4A1	26760
chr3	187463162	187463620	0.81	6.49E-01	BCL6	84		
chr6	43737258	43737558	0.99	6.49E-01	VEGFA	-545		
chr12	49412344	49412726	0.80	6.49E-01	PRKAG1	57		
chr17	18684349	18684659	1.13	6.49E-01	FAM18B	-78		
chr15	49913004	49913359	1.08	6.49E-01	FGF7	197807	ATP8B4	498237
chr1	228678210	228678550	1.00	6.50E-01	RHOU	-192489	HIST3H2BB	32572
chr1	27247887	27248170	1.05	6.50E-01	NUDC	-195		
chr5	130500707	130501132	1.12	6.50E-01	HINT1	114		
chr5	133747513	133747764	0.95	6.50E-01	PHF15	-114159	UBE2B	40769
chr17	1945219	1945464	1.05	6.50E-01	OVCA2	65		
chr17	80309724	80310034	0.95	6.51E-01	SECTM1	-17958	TEX19	-7245
chr11	118722732	118723104	1.04	6.51E-01	DDX6	-60946	CXCR5	-31623
chr12	126257630	126257940	1.23	6.51E-01	TMEM132B	446623		
chr17	45144774	45145100	0.90	6.51E-01	RPRML	-88323	CDC27	121728
chr5	14145216	14145473	0.87	6.51E-01	TRIO	1516	ANKH	726542
chr7	93679431	93679753	0.99	6.51E-01	COL1A2	-344281	BET1	-45902
chr12	32635171	32635540	1.16	6.51E-01	FGD4	-19685	BICD1	375171
chr19	45982266	45982635	0.94	6.51E-01	FOSB	11198	RTN2	17862
chr22	39077785	39078030	1.22	6.52E-01	TOMM22	-46		
chr7	47709129	47709405	0.80	6.52E-01	TNS3	-130068	C7orf69	-125622
chr3	127842635	127843044	1.28	6.52E-01	RUVBL1	-169		
chr19	36231821	36232219	1.14	6.52E-01	PSENEN	-4474	TMEM149	1500
chr7	23338748	23339084	1.03	6.52E-01	GPNMB	52600	IGF2BP3	171079
chr15	68132099	68132518	1.46	6.52E-01	PIAS1	-214263	LBXCOR1	14368
chr19	17622097	17622519	1.23	6.52E-01	PGLS	-124		
chr14	102275904	102276254	1.11	6.53E-01	DYNC1H1	-154786	PPP2R5C	47944
chr22	50628938	50629429	0.81	6.53E-01	PANX2	20024	TUBGCP6	54216
chr12	92539432	92539918	1.08	6.53E-01	BTG1	-2		
chr19	6424848	6425147	1.06	6.53E-01	KHSRP	-176		
chr4	178230576	178231060	1.32	6.53E-01	NEIL3	-173		

chr20	50077393	50077738	1.16	6.53E-01	KCNG1	-437891	NFATC2	81692		
chr1	21619599	21619936	0.95	6.53E-01	ECE1	-3002				
chr17	7142966	7143308	1.15	6.53E-01	PHF23	-312				
chr17	27181761	27182167	1.02	6.53E-01	ERAL1	-79				
chr11	95448839	95449258	1.18	6.53E-01	SESN3	-484803	CEP57	-74593		
chr11	134098540	134098807	1.26	6.53E-01	NCAPD3	-4248	VPS26B	4113		
chr5	158533868	158534144	1.14	6.53E-01	EBF1	-7218	RNF145	100828		
chr5	74062855	74063155	1.14	6.54E-01	NSA2	-98	GFM2	37		
chr6	106533940	106534314	0.81	6.54E-01	PRDM1	-68				
chr1	27147313	27147569	0.84	6.54E-01	ZDHHC18	-5760	PIGV	32955		
chr10	112601579	112601911	1.18	6.54E-01	PDCD4	-29851	RBM20	197590		
chr12	121960772	121961069	1.07	6.54E-01	KDM2B	57999	RNF34	123019		
chr11	87063790	87064139	1.12	6.55E-01	TMEM135	314900	RAB38	844634		
chr22	36553159	36553500	0.91	6.55E-01	RBM9	-128745	APOL3	3647		
chr1	28099409	28099756	1.09	6.55E-01	STX12	-111				
chr16	717903	718180	1.13	6.55E-01	RHOT2	-91				
chr11	65728917	65729350	1.20	6.55E-01	SART1	-26				
chr16	58663595	58664063	0.86	6.55E-01	CNOT1	-79				
chr17	73851199	73851921	1.22	6.55E-01	UNC13D	-10762	TRIM47	23096		
chr19	37958273	37958583	1.20	6.56E-01	ZNF570	-1554	ZNF569	-89		
chr8	56843131	56843416	0.87	6.56E-01	LYN	50888	RPS20	143866		
chr4	153771557	153771818	1.33	6.56E-01	FHDC1	-92447	ARFIP1	70576		
chr15	44116820	44117106	1.22	6.56E-01	MFAP1	-12				
chr16	87843916	87844469	1.21	6.56E-01	KLHDC4	-44651	SLC7A5	58907		
chr4	186508582	186508968	1.17	6.56E-01	PDLIM3	-52063	SORBS2	97225		
chr6	26199225	26200052	0.87	6.56E-01	HIST1H3D	-175	HIST1H2AD	-168	HIST1H2BF	-148
chr11	47921416	47921765	1.28	6.57E-01	PTPRJ	-80519	NUP160	-51534		
chr9	37465649	37465912	1.04	6.57E-01	ZBTB5	-374				
chr4	1722834	1723348	1.03	6.58E-01	TACC3	-175	TMEM129	-7		
chr12	121566901	121567236	1.15	6.58E-01	P2RX7	-3609				
chr15	88993385	88993741	1.13	6.58E-01	NTRK3	-193902	MRPL46	17070		
chr2	3699687	3699997	0.91	6.58E-01	ALLC	-5944	COLEC11	57205		
chr2	71357560	71357843	0.93	6.58E-01	MCEE	-308	MPHOSPH10	258		
chr7	99097816	99098084	0.81	6.58E-01	ZKSCAN5	-4323	ZNF394	-73		
chr11	64795077	64795352	0.96	6.58E-01	SNX15	425				
chr13	48611732	48612388	1.22	6.58E-01	NUDT15	357				
chr17	31204166	31204609	0.82	6.59E-01	MYO1D	-486				
chr4	40057160	40057470	1.09	6.59E-01	N4BP2	-1209				

chr9	117135252	117135640	1.31	6.59E-01	AKNA	21239	ORM1	50143			
chr5	81047105	81047461	1.10	6.59E-01	SSBP2	-211					
chr14	96968571	96968935	0.94	6.59E-01	PAPOLA	33					
chr4	68411019	68411463	1.20	6.59E-01	CENPC1	15					
chr1	179851720	179852036	1.21	6.59E-01	TOR1AIP2	-4944	TOR1AIP1	458			
chrX	73163957	73164233	1.20	6.59E-01	ZCCHC13	-359930	CHIC1	381111			
chr13	26796271	26796604	1.06	6.60E-01	RNF6	-460					
chr16	1755894	1756170	1.13	6.60E-01	MAPK8IP3	-189					
chr11	8710322	8710655	0.96	6.60E-01	RPL27A	6494	ST5	121735			
chr6	26250571	26250912	1.10	6.60E-01	HIST1H4G	-3537	HIST1H2BH	-1137	HIST1H3F	93	
chr17	61627397	61627805	1.16	6.60E-01	DCAF7	-221					
chr19	8570597	8570885	0.88	6.60E-01	PRAM1	-3246					
chr19	10679662	10680033	0.78	6.60E-01	CDKN2D	-217					
chr3	123303909	123304433	0.84	6.60E-01	PTPLB	-247					
chr7	4681386	4681882	0.89	6.60E-01	FOXK1	-40296					
chr10	88699173	88699483	0.72	6.60E-01	MMRN2	18097	BMPR1A	182932			
chr14	89897164	89897607	1.33	6.60E-01	FOXN3	188108	TTC8	606468			
chr9	93593407	93593730	1.16	6.60E-01	SYK	29557	AUH	530637			
chr1	206785681	206786022	1.15	6.60E-01	LGTN	52					
chr1	235667845	235668155	1.07	6.60E-01	B3GALNT2	-219					
chr12	50135252	50135639	1.05	6.60E-01	TMBIM6	-146					
chr17	29288197	29288550	1.30	6.60E-01	RNF135	-9582	ADAP2	39620			
chr4	53588371	53588895	1.18	6.60E-01	USP46	-63131	LP9056	29149			
chr15	75746094	75746467	1.11	6.61E-01	SIN3A	-2355					
chr2	89053754	89054130	1.08	6.61E-01	O1/O11 and JK2	-836620	RPIA	62766			
chr1	193506858	193507113	1.09	6.61E-01	B3GALT2	-351243					
chr6	91296867	91297196	0.86	6.61E-01	MAP3K7	-125					
chr17	32688544	32688859	1.13	6.61E-01	CCL1	1550	CCL13	5231			
chr3	178684871	178685247	0.86	6.61E-01	ZMAT3	104525	KCNMB2	430835			
chr7	139024827	139025559	1.18	6.61E-01	LUC7L2	-19441	TTC26	206703			
chr18	3246074	3246384	1.06	6.61E-01	MYL12A	-1299					
chr2	196933081	196933327	1.16	6.61E-01	DNAH7	332					
chr14	50552282	50552666	1.01	6.61E-01	SOS2	145625	ARF6	192738			
chr5	68665332	68665743	0.90	6.61E-01	RAD17	-1743	TAF9	-129			
chr3	152879819	152880059	0.71	6.63E-01	RAP2B	-90					
chr8	144699675	144699962	1.01	6.63E-01	TSTA3	-87					
chr16	58370646	58370956	0.86	6.63E-01	GINS3	-55497	KLKBL4	-41850			
chr17	74478023	74478270	0.85	6.63E-01	AANAT	14496	RHBDF2	19361			

chr5	10607240	10607596	1.25	6.63E-01	DAP	153969	ROPN1L	165409
chr20	2281831	2282188	0.93	6.63E-01	TGM6	-79544	TGM3	5397
chr2	219081511	219081905	1.01	6.63E-01	ARPC2	-166		
chr18	44238169	44238549	0.98	6.63E-01	LOXHD1	-1363		
chr18	47833926	47834310	0.76	6.63E-01	SKA1	-67274	CXXC1	-19426
chr3	154042198	154042466	0.93	6.64E-01	DHX36	-46		
chr2	43446639	43446987	0.80	6.64E-01	HAAO	-427062	ZFP36L2	6932
chr14	24610729	24611124	0.82	6.64E-01	PSME2	4928	PSME1	5549
chr1	203238232	203238500	0.94	6.65E-01	CHIT1	-39506	BTG2	-36298
chr13	26760541	26760899	1.06	6.65E-01	SHISA2	-135522	RNF6	35258
chr6	36634925	36635285	1.13	6.65E-01	CDKN1A	-11354	SFRS3	73015
chr12	19592318	19592606	1.10	6.65E-01	AEBP2	-146		
chr14	62161733	62162170	1.11	6.65E-01	HIF1A	-167		
chr2	27632445	27632895	1.15	6.65E-01	PPM1G	-174		
chr17	35969342	35969723	1.11	6.65E-01	SYNRG	-47		
chrX	84258740	84258998	1.12	6.65E-01	APOOL	-36		
chr1	17231520	17232001	1.22	6.65E-01	MSTP9	-140786	CROCC	-16684
chr12	111021012	111021398	0.97	6.66E-01	PPTC7	-141		
chr10	5671887	5672131	0.80	6.66E-01	ASB13	36549	CALML3	105085
chr1	55181368	55181622	0.83	6.66E-01	TTC4	-34		
chr12	113512867	113513518	1.14	6.66E-01	DTX1	17531	RASAL1	60828
chr19	18402416	18402826	1.03	6.66E-01	JUND	-10189	LSM4	31380
chr6	16322952	16323307	1.07	6.67E-01	GMPR	84319	ATXN1	438591
chr12	56709647	56710265	1.15	6.67E-01	CNPY2	164		
chr6	26553593	26553903	1.00	6.67E-01	ABT1	-43432	HMGN4	15176
chr7	75807478	75807740	0.85	6.67E-01	HSPB1	-124266	MDH2	130216
chr19	10362770	10363015	1.07	6.67E-01	MRPL4	253		
chr19	49339043	49339381	1.13	6.67E-01	HSD17B14	722		
chr12	31478920	31479230	1.01	6.68E-01	OVOS1	-119987	DENND5B	264877
chr2	68478220	68478498	0.90	6.68E-01	PPP3R1	1292	PNO1	93354
chr17	40474836	40475146	0.91	6.68E-01	STAT5A	35426	STAT3	65522
chr19	3987809	3988058	0.92	6.68E-01	EEF2	-2473		
chr9	34048892	34049735	1.14	6.69E-01	DCAF12	77457	UBE2R2	232132
chr11	67084926	67085410	1.15	6.69E-01	SSH3	14249	POLD4	35849
chr4	154265657	154266003	1.17	6.69E-01	MND1	29		
chr9	36008813	36009058	0.75	6.69E-01	OR2S2	-50785	RECK	-27974
chr5	175815817	175816239	1.24	6.69E-01	NOP16	-488	HIGD2A	244
chr11	2554029	2554310	0.91	6.69E-01	KCNQ1	87949	CDKN1C	352825

chr19	41945632	41946053	1.22	6.69E-01	ATP5SL	-33		
chr19	2391324	2391611	0.84	6.69E-01	TMPRSS9	1684	TIMM13	36407
chr10	102820673	102821013	1.21	6.69E-01	KAZALD1	-156		
chr20	45985376	45985645	1.11	6.69E-01	ZMYND8	-37		
chr6	44140497	44140758	0.88	6.69E-01	SLC29A1	-50668	CAPN11	14080
chr9	117147745	117148079	1.14	6.69E-01	AKNA	8773	ORM1	62609
chr19	41304742	41305157	1.06	6.70E-01	EGLN2	-1231		
chr12	860338	860720	0.89	6.70E-01	WNK1	-1696		
chr2	104695037	104695331	0.79	6.70E-01	POU3F3	-776785		
chr9	128003411	128003952	1.06	6.70E-01	HSPA5	-21		
chr19	46220553	46221046	1.07	6.70E-01	FBXO46	13351	QPCTL	25059
chr6	26537518	26537885	0.96	6.70E-01	HMGN4	-870		
chr18	21599606	21599882	1.31	6.70E-01	CABYR	-119211	LAMA3	330182
chr19	6536095	6536405	0.83	6.70E-01	TNFSF9	5240	CD70	54913
chr19	1244471	1244707	1.13	6.70E-01	MIDN	-3963		
chr16	15736297	15736611	0.99	6.70E-01	KIAA0430	555		
chr3	150264155	150264597	1.24	6.70E-01	EIF2A	-198	SERP1	588
chr1	95538299	95538696	1.08	6.70E-01	ALG14	9		
chr15	70272127	70272530	1.18	6.72E-01	TLE3	117927	RPLP1	527170
chr14	67826750	67827258	1.21	6.72E-01	ATP6V1D	-413	EIF2S1	-30
chr16	85942172	85942490	1.24	6.72E-01	FOXF1	-601802	IRF8	9557
chr8	141522006	141522391	1.16	6.72E-01	CHRAC1	798		
chr11	72504615	72505133	1.14	6.73E-01	ARAP1	-41440	ATG16L2	-20577
chr15	91475669	91475984	0.93	6.73E-01	UNC45A	-2388	HDDC3	-53
chr6	52589282	52589604	0.93	6.73E-01	GSTA2	38918	TMEM14A	53559
chr15	45694369	45694872	1.21	6.73E-01	SPATA5L1	102		
chr17	7482727	7483129	1.17	6.73E-01	MPDU1	-4037	CD68	123
chr7	99746409	99746673	0.93	6.73E-01	GAL3ST4	19832	MBLAC1	22221
chr7	130581766	130582036	1.35	6.73E-01	MKLN1	-430694	KLF14	-163041
chr15	59705317	59705627	1.16	6.73E-01	GCNT3	-198510	MYO1E	-40401
chr7	30324100	30324501	1.01	6.73E-01	ZNRF2	378		
chr16	58790315	58790625	0.84	6.73E-01	GOT2	-22224		
chr17	5095161	5095489	0.84	6.73E-01	ZNF594	-147		
chr12	32426757	32427056	0.79	6.73E-01	FGD4	-228134	BICD1	166722
chr2	219122341	219122646	1.19	6.74E-01	GPBAR1	-3244		
chrX	24072638	24073055	1.15	6.74E-01	EIF2S3	-218		
chr16	67840282	67840703	0.98	6.74E-01	TSNAXIP1	-517	RANBP10	62
chr17	20937703	20938068	1.07	6.74E-01	LGALS9B	-567038	USP22	8466

chr2	38735520	38735802	1.26	6.74E-01	ATL2	-131229	HNRPLL	94517
chr6	15691525	15691847	0.85	6.74E-01	MYLIP	-437631	DTNBP1	-28415
chr13	50510478	50510811	1.17	6.75E-01	KPNA3	-143588	TRIM13	-60498
chr9	26892601	26892967	0.90	6.75E-01	PLAA	54684		
chr11	65171057	65171324	1.19	6.75E-01	SCYL1	-121357	FRMD8	17150
chr1	8491109	8491419	0.88	6.75E-01	SLC45A1	106874	RERE	386435
chr12	125425239	125425566	0.75	6.75E-01	UBC	-25826	DHX37	48264
chr22	23238798	23239264	0.95	6.75E-01	GNAZ	-173638	IGL@	9071
chr3	143690723	143691081	1.05	6.76E-01	C3orf58	262		
chr5	121297333	121297760	1.20	6.76E-01	SRFBP1	-109		
chr21	43648283	43648695	0.93	6.76E-01	ABCG1	9222	TFF3	87217
chr1	36554280	36554587	1.12	6.76E-01	ADPRHL2	-19		
chr17	4870640	4871112	0.89	6.76E-01	SPAG7	256		
chr7	32529774	32530241	0.83	6.76E-01	LSM5	15		
chr5	138724228	138724547	0.80	6.76E-01	SLC23A1	-5349	MGC29506	1217
chr15	69113231	69113611	0.72	6.77E-01	ANP32A	-160		
chr11	47270261	47270570	1.15	6.77E-01	ACP2	41		
chr9	272798	273152	0.98	6.77E-01	KANK1	-231728	DOCK8	58110
chr11	9636749	9637059	1.01	6.78E-01	SWAP70	-48724	WEE1	41676
chr6	88635389	88635692	1.13	6.78E-01	AKIRIN2	-223556	SPACA1	-121966
chr1	1447195	1447519	1.10	6.78E-01	ATAD3A	-198		
chr11	925591	925939	0.87	6.78E-01	AP2A2	-76		
chr1	54955092	54955355	0.73	6.78E-01	SSBP3	-83132	ACOT11	-58677
chr12	69684837	69685116	0.96	6.79E-01	LYZ	-57157	CPSF6	51660
chr2	9695830	9696170	1.01	6.79E-01	ADAM17	-83		
chr17	40831775	40832077	1.18	6.79E-01	PLEKHH3	-2878	CNTNAP1	-2706
chr19	1266761	1267225	0.75	6.79E-01	CIRBP	-2274		
chr6	153323854	153324210	1.08	6.79E-01	MTRF1L	-107		
chr17	74488578	74488908	1.03	6.79E-01	RHBDF2	8765	AANAT	25092
chr16	70323222	70323536	1.01	6.79E-01	AARS	33		
chr8	27950488	27951031	1.27	6.79E-01	ELP3	176		
chr19	10870222	10870736	1.15	6.79E-01	DNM2	41724	TMED1	76504
chr1	154244686	154245247	1.13	6.80E-01	HAX1	-72		
chr19	36390590	36390900	0.96	6.80E-01	HCST	-2637	NFKBID	807
chr19	15543495	15543818	1.01	6.80E-01	AKAP8L	-13824	WIZ	17105
chr13	45151834	45152312	0.94	6.80E-01	TSC22D1	-1372		
chr6	24403000	24403265	0.86	6.80E-01	MRS2	-20		
chr1	203264946	203265256	0.80	6.81E-01	CHIT1	-66241	BTG2	-9563

chr12	32908689	32909044	1.08	6.81E-01	YARS2	7		
chr4	148538443	148538772	0.88	6.81E-01	TMEM184C	69		
chr5	76326058	76326341	0.93	6.81E-01	AGGF1	-32		
chr1	167772581	167772870	0.89	6.81E-01	MPZL1	81539	ADCY10	110727
chr5	1801294	1801551	0.84	6.81E-01	MRPL36	-1467	NDUFS6	-86
chr2	86790467	86790748	0.98	6.81E-01	RNF103	60370	KDM3A	122024
chr8	119294186	119294454	1.33	6.81E-01	EXT1	-170262	TNFRSF11B	670063
chr10	105677800	105678108	1.13	6.81E-01	OBFC1	91		
chr9	131486489	131486864	1.07	6.82E-01	ZDHHC12	-269		
chr3	33839943	33840298	1.25	6.82E-01	PDCD6IP	55		
chr2	101618604	101618859	1.25	6.82E-01	RPL31	41		
chr15	101791989	101792443	1.08	6.82E-01	CHSY1	-90		
chr15	78591847	78592100	1.12	6.82E-01	CRABP1	-40692	DNAJA4	35487
chr17	62151727	62152067	1.27	6.82E-01	ICAM2	-53903	ERN1	55605
chr19	10981964	10982242	1.00	6.83E-01	CARM1	-150		
chr1	202317569	202317923	1.14	6.83E-01	PPP1R12B	-90		
chr14	61201305	61201615	0.79	6.83E-01	MNAT1	-10		
chr22	42228835	42229304	1.06	6.84E-01	SREBF2	-36		
chr6	26312696	26312948	1.15	6.84E-01	BTN3A2	-52576	HIST1H4H	-27095
chr5	176881532	176881842	1.12	6.84E-01	PRR7	7891	DBN1	19007
chr8	33412379	33412711	0.82	6.84E-01	RNF122	12098	MAK16	69860
chrX	1587266	1587604	1.08	6.84E-01	ASMTL	-15591	P2RY8	68602
chr7	101500105	101500433	0.86	6.84E-01	SH2B2	-428136	CUX1	39387
chr17	4316532	4316808	1.11	6.84E-01	UBE2G1	-46701	SPNS3	-20549
chr16	2021805	2022152	1.21	6.84E-01	TBL3	-85		
chr15	83419065	83419623	1.23	6.84E-01	WHAMM	-58629	AP3B2	-40709
chr10	17272511	17272896	0.88	6.85E-01	VIM	2446	ST8SIA6	223550
chr14	75469181	75469779	0.91	6.85E-01	EIF2B2	-132		
chr5	140904377	140904713	0.82	6.85E-01	PCDHGC5	35737	DIAPH1	94077
chr9	115555510	115555820	1.10	6.85E-01	SNX30	42531	SLC46A2	97528
chr4	26322181	26322457	0.95	6.86E-01	RBPJ	-129		
chr19	51014059	51014922	1.10	6.86E-01	ASPDH	2651	C19orf63	34755
chr2	214022063	214022336	1.02	6.86E-01	SPAG16	-126916	IKZF2	-7142
chr6	2849144	2849462	0.93	6.86E-01	SERPINB1	-7222	SERPINB9	54242
chr20	32900503	32900850	1.10	6.86E-01	ITCH	-50385	AHCY	-9462
chr11	59521485	59521924	1.09	6.86E-01	STX3	-1184		
chr10	61996741	61997017	0.79	6.86E-01	CCDC6	-330061	ANK3	152609
chr13	25258063	25258318	1.26	6.86E-01	RNF17	-80110	ATP12A	3496

chr17	79212940	79213287	1.05	6.87E-01	C17orf56	-259	C17orf89	3
chr15	62352580	62353087	1.06	6.87E-01	VPS13C	-187		
chr20	34287166	34287432	0.78	6.87E-01	NFS1	-25	ROMO1	67
chr1	20208706	20209068	1.14	6.87E-01	RNF186	-67116	PLA2G2E	41223
chr14	77495045	77495372	1.11	6.87E-01	C14orf4	-175		
chr11	62413965	62414209	1.10	6.87E-01	GANAB	17		
chr17	42147902	42148231	1.18	6.87E-01	LSM12	-3080	G6PC3	-51
chr8	124169374	124169813	0.83	6.87E-01	C8orf76	84023	WDR67	84674
chr12	123011451	123011831	1.15	6.87E-01	KNTC1	-168		
chr3	131221653	131221988	1.26	6.87E-01	MRPL3	8		
chr18	30812057	30812365	0.98	6.87E-01	KLHL14	-459237	ASXL3	-346330
chr12	111351232	111351549	1.05	6.88E-01	PPP1CC	-170634	MYL2	7013
chr1	93427245	93427544	0.77	6.88E-01	FAM69A	-316		
chr12	6579627	6580186	0.79	6.88E-01	VAMP1	-64		
chr2	27851712	27852060	1.07	6.88E-01	GPN1	23		
chr8	101964222	101964466	0.97	6.88E-01	YWHAZ	877		
chr12	122519734	122520044	0.86	6.88E-01	MLXIP	3129	IL31	138857
chr22	34238464	34238840	0.98	6.88E-01	SYN3	-835843	LARGE	77764
chr11	95523489	95523904	1.13	6.88E-01	CEP57	55		
chr5	78023931	78024221	1.15	6.88E-01	LHFPL2	-79428	ARSB	258281
chr7	72349708	72349993	0.86	6.88E-01	TYW1B	-51038	POM121	-45809
chr11	9622974	9623284	0.97	6.88E-01	SWAP70	-62499	WEE1	27901
chr10	26624136	26624507	0.85	6.88E-01	APBB1IP	-102944	GAD2	119086
chr3	32068058	32068443	0.90	6.88E-01	GPD1L	-79893	ZNF860	44985
chr15	66084449	66085229	1.18	6.89E-01	DENND4A	-208		
chr7	130808973	130809457	0.89	6.89E-01	KLF14	-390355	MKLN1	-203380
chr3	182840168	182840444	1.01	6.89E-01	MCCC1	-22941	LAMP3	40361
chr18	19180541	19181002	0.90	6.90E-01	ESCO1	-79		
chr1	16766963	16767347	0.94	6.90E-01	SPATA21	-3236	NECAP2	-12
chr7	130598211	130598610	0.99	6.90E-01	MKLN1	-414184	KLF14	-179551
chr6	143771708	143772090	0.84	6.90E-01	ADAT2	-58	PEX3	-19
chr1	229643961	229644262	1.03	6.90E-01	NUP133	-24		
chr11	65479259	65479624	1.26	6.90E-01	KAT5	-47		
chr3	12525681	12525967	1.11	6.90E-01	TSEN2	-186		
chr3	50396914	50397257	1.18	6.90E-01	TMEM115	-147		
chr6	86353701	86354064	1.16	6.90E-01	SYNCRIP	-840		
chr8	145550349	145550750	1.01	6.90E-01	DGAT1	17		
chr14	59823606	59823902	1.17	6.90E-01	GPR135	108305	DAAM1	168355

chr3	167453161	167453531	1.12	6.90E-01	PDCD10	-695	SERPINI1	-86
chr5	173019100	173019410	0.98	6.90E-01	STC2	-262749	BOD1	24411
chr6	31939636	31940379	0.88	6.90E-01	DOM3Z	24	STK19	362
chr15	41245359	41245748	1.11	6.90E-01	CHAC1	-82		
chr21	45079281	45079631	1.04	6.90E-01	HSF2BP	-82	RRP1B	24
chr12	53895236	53895646	0.90	6.91E-01	MAP3K12	-2170	TARBP2	64
chr5	118689726	118690016	1.07	6.91E-01	TNFAIP8	-1725		
chr16	22206938	22207324	1.13	6.91E-01	EEF2K	-10461	VWA3A	103268
chr1	17036308	17036629	0.87	6.91E-01	NBPF1	-96487	MSTP9	54506
chr13	41885124	41885598	1.02	6.91E-01	NARG1L	20		
chr4	4249941	4250185	1.21	6.91E-01	TMEM128	-129		
chr11	73498779	73499177	1.16	6.92E-01	MRPL48	61		
chr11	85780192	85780432	0.90	6.92E-01	PICALM	-204		
chr14	94425734	94426076	1.24	6.92E-01	ASB2	-2138		
chr17	65821350	65821690	0.90	6.92E-01	BPTF	-260		
chr19	4791248	4791571	1.09	6.92E-01	FEM1A	-318		
chr19	7745498	7745862	1.05	6.92E-01	TRAPPC5	-27		
chr2	242254662	242255179	0.92	6.92E-01	SEPT2	198		
chr9	33001480	33001856	1.16	6.92E-01	APTX	-42		
chr21	43757995	43758250	1.19	6.92E-01	TFF3	-22417	TFF2	13085
chr16	31470610	31470854	1.08	6.92E-01	ARMC5	415		
chr17	38514744	38515060	0.95	6.92E-01	GJD3	-2005		
chr2	88990943	88991337	1.20	6.92E-01	RPIA	-36		
chr3	195621619	195622090	0.86	6.92E-01	TNK2	577		
chr16	56945768	56946156	1.05	6.92E-01	HERPUD1	-19786	SLC12A3	46843
chr17	21191047	21191377	1.10	6.92E-01	KCNJ12	-88487	MAP2K3	3244
chr5	156698706	156699251	0.77	6.92E-01	CYFIP2	5888	C5orf40	73750
chr7	152161028	152161323	1.04	6.92E-01	MLL3	-28086	XRCC2	212074
chr9	100864263	100864551	1.02	6.92E-01	TRIM14	17081	NANS	45725
chr16	89164844	89165282	0.91	6.93E-01	CDH15	-73100	ACSF3	4809
chr5	157625625	157625959	1.14	6.93E-01	CLINT1	-339624	EBF1	900996
chr15	37307284	37307528	1.10	6.93E-01	MEIS2	85298	C15orf41	435594
chr12	83079519	83079904	0.84	6.93E-01	TMTC2	-1222		
chr6	42847539	42847876	0.95	6.93E-01	RPL7L1	37		
chr12	498333	498612	0.84	6.93E-01	KDM5A	147		
chr1	33446798	33447124	1.25	6.94E-01	RNF19B	-16675	AK2	55531
chr2	68694529	68694770	1.01	6.94E-01	APLF	-41		
chr7	99156076	99156329	0.82	6.94E-01	ZNF655	-245		

chr19	39137999	39138262	1.00	6.94E-01	ACTN4	-196		
chr8	81397640	81398120	1.06	6.94E-01	ZBTB10	-568		
chr9	125590871	125591257	1.04	6.95E-01	PDCL	-224		
chr17	7232879	7233477	0.97	6.95E-01	NEURL4	-540		
chr17	7760464	7761418	1.20	6.95E-01	CYB5D1	-123	LSMD1	231
chr3	12598372	12598631	1.09	6.95E-01	MKRN2	-92		
chr12	6862687	6863049	0.82	6.95E-01	MLF2	-786		
chr14	54976427	54976715	0.89	6.95E-01	CGRRF1	-16		
chr11	1543089	1543361	0.92	6.95E-01	HCCA2	-41110	DUSP8	49925
chr5	177019051	177019300	0.98	6.95E-01	TMED9	-37		
chr1	32110641	32110951	0.80	6.95E-01	PEF1	-319		
chr14	77506655	77507267	1.16	6.95E-01	C14orf4	-11927	ZDHHC22	101173
chr17	76732889	76733219	0.85	6.95E-01	DNAH17	-165650	CYTH1	45322
chr16	68056998	68057308	1.07	6.95E-01	DUS2L	-51	DDX28	806
chr3	56950298	56950633	1.28	6.96E-01	ERC2	-448075	ARHGEF3	162870
chrX	70752609	70753050	1.02	6.96E-01	OGT	-103		
chr1	28562494	28562772	0.86	6.96E-01	DNAJC8	-3091	ATPIF1	22
chr12	2994479	2994781	1.05	6.96E-01	FOXM1	-8327	TULP3	-5403
chr2	75873676	75874030	0.90	6.96E-01	MRPL19	-56		
chr6	31587638	31588344	0.87	6.96E-01	BAT2	-459		
chr9	125675194	125675492	0.84	6.97E-01	ZBTB6	266		
chr11	64085462	64085702	0.98	6.97E-01	TRMT112	-549	PRDX5	13
chr14	81685515	81685906	0.87	6.97E-01	GTF2A1	1583	TSHR	263842
chr12	98897627	98898219	0.87	6.97E-01	AX747640	-47000	TMPO	-11486
chr6	27839747	27840253	0.81	6.97E-01	HIST1H1B	-4641	HIST1H3I	99
chr1	109618550	109618860	0.86	6.98E-01	TAF13	-81		
chr14	24685124	24685488	1.12	6.98E-01	MDP1	-65		
chr1	45241061	45241313	0.83	6.98E-01	RPS8	-59		
chr11	66405671	66406023	1.07	6.98E-01	RBM14	21794	RBM4B	39428
chr12	92860279	92860684	1.04	6.98E-01	BTG1	-320809	PLEKHG7	-269783
chr12	96721214	96721536	0.84	6.98E-01	PCTK2	72848	ELK3	133168
chr7	134001672	134001928	1.10	6.99E-01	SLC35B4	27		
chr19	42900969	42901283	0.98	6.99E-01	CNFN	-6682	LIPE	30452
chr16	23568604	23569071	1.05	6.99E-01	EARS2	-142		
chr17	62223027	62223474	1.14	6.99E-01	ERN1	-15749	TEX2	117402
chr2	241524637	241524915	1.02	6.99E-01	CAPN10	-1369		
chr11	94884605	94884978	1.14	6.99E-01	ENDOD1	61775	SESN3	79454
chr14	48398613	48398858	1.01	6.99E-01	MDGA2	-254748		

chr18	47841897	47842146	0.97	6.99E-01	SKA1	-59370	CXXC1	-27330
chr15	78730111	78730493	1.12	7.00E-01	IREB2	-216		
chr1	38941003	38941290	0.81	7.00E-01	POU3F1	-428697	RRAGC	384193
chr2	135497484	135497868	1.17	7.00E-01	ACMSD	-98510	TMEM163	-21105
chr10	94351312	94351584	0.88	7.00E-01	KIF11	-1377		
chr15	91260343	91260743	1.14	7.00E-01	BLM	-36		
chr18	20808750	20809086	0.90	7.00E-01	CABLES1	93191	C18orf45	209007
chr6	43474500	43474748	1.03	7.00E-01	YIPF3	10078	TJAP1	29363
chr3	136581076	136581377	1.14	7.00E-01	NCK1	154		
chr3	23958455	23958783	1.21	7.00E-01	NKIRAS1	-82	RPL15	-20
chrX	54466629	54466904	1.13	7.00E-01	TSR2	-86		
chr6	11459261	11459571	1.28	7.00E-01	NEDD9	-226501	TMEM170B	-79095
chr12	54718440	54718716	0.85	7.00E-01	COPZ1	-333		
chr1	17222576	17222897	1.21	7.00E-01	MSTP9	-131762	CROCC	-25708
chr14	55493647	55494349	0.88	7.00E-01	WDHD1	-191	SOCS4	154
chr12	110906957	110907267	1.14	7.01E-01	GPN3	-1048		
chr10	113943401	113943645	0.83	7.01E-01	GPAM	2		
chr9	130700071	130700368	1.13	7.01E-01	DPM2	543		
chr19	4769620	4769856	1.03	7.01E-01	DPP9	-45883	FEM1A	-21990
chr9	123655045	123655384	1.17	7.01E-01	PHF19	-15609	TRAF1	33958
chr22	27067235	27067573	1.01	7.02E-01	CRYBA4	49476		
chr16	53164465	53164972	0.90	7.02E-01	RBL2	-303632	CHD9	75774
chr13	67804446	67804787	1.01	7.02E-01	PCDH9	-149		
chr11	65769506	65769933	1.22	7.02E-01	EIF1AD	-142	BANF1	170
chr6	42540240	42540516	1.31	7.02E-01	UBR2	8320	PRPH2	149980
chr1	38156032	38156457	1.12	7.02E-01	CDCA8	-1914		
chr1	167297994	167298524	1.08	7.02E-01	POU2F1	108116	CD247	189588
chr1	115300575	115300820	1.09	7.02E-01	CSDE1	-27		
chr19	10490975	10491490	1.09	7.02E-01	TYK2	15		
chr6	53659265	53659823	0.85	7.03E-01	LRRC1	10		
chr3	197509183	197509458	1.26	7.03E-01	LRCH3	-8824	FYTTD1	32697
chr3	124449040	124449360	1.12	7.03E-01	UMPS	-13		
chr10	12237597	12238040	0.75	7.03E-01	CDC123	-142	NUDT5	324
chr15	66797031	66797601	1.02	7.03E-01	RPL4	-132	ZWILCH	-115
chr1	100731471	100731858	0.95	7.03E-01	RTCD1	-49		
chr4	40196460	40196796	0.97	7.03E-01	RHOH	-1899		
chr4	8442350	8442640	1.09	7.03E-01	C4orf23	-554	ACOX3	-43
chr6	135375878	135376298	0.92	7.03E-01	HBS1L	-52		

chr1	36107051	36107387	0.89	7.03E-01	PSMB2	-76		
chr13	20532610	20533052	0.69	7.04E-01	ZMYM2	21		
chrX	2625514	2625758	1.14	7.04E-01	XG	-44457	CD99	16408
chr19	57831528	57831838	1.09	7.05E-01	ZNF543	-194		
chr1	207096456	207096892	1.12	7.05E-01	FAIM3	-1296		
chr7	140714466	140714948	1.20	7.05E-01	MRPS33	74		
chr10	173853	174294	1.16	7.05E-01	TUBB8	-78896	ZMYND11	-6350
chr10	12306276	12306593	1.02	7.05E-01	CAMK1D	-85148	NUDT5	-68292
chr17	27229199	27229654	0.91	7.05E-01	FLOT2	-4712	DHRS13	662
chr1	32573503	32573760	1.22	7.05E-01	KPNA6	-12		
chr1	151128532	151128872	0.80	7.05E-01	TNFAIP8L2	-403		
chr2	68479668	68480210	1.04	7.05E-01	PPP3R1	-288		
chr18	71815372	71816053	0.94	7.05E-01	FBXO15	-613	C18orf55	-33
chr3	150481729	150482070	0.97	7.05E-01	SIAH2	-637		
chr15	70611879	70612296	0.87	7.06E-01	TLE3	-221832	UACA	443762
chr17	19366499	19366818	1.27	7.06E-01	SLC47A1	-70508	RNF112	52136
chr9	37079584	37079985	1.09	7.06E-01	PAX5	-45309	ZCCHC7	-40684
chr6	151773191	151773592	1.15	7.07E-01	RMND1	-76	C6orf211	-30
chr2	227637490	227637798	1.18	7.07E-01	IRS1	25862		
chr12	133263829	133264248	1.05	7.07E-01	PXMP2	-153	POLE	-94
chr17	8089547	8089830	1.13	7.07E-01	TMEM107	-9975	C17orf59	3875
chr3	122102882	122103188	1.09	7.07E-01	CCDC58	-961	FAM162A	12
chr12	50419233	50419512	1.08	7.07E-01	RACGAP1	-66		
chr16	71879695	71879981	1.09	7.07E-01	ATXN1L	-61		
chr2	105593176	105593565	0.94	7.07E-01	MRPS9	-61112	POU3F3	121402
chr15	41196259	41196499	1.00	7.08E-01	DLL4	-25168	VPS18	9751
chr5	139015819	139016128	0.96	7.08E-01	CXXC5	-12327	UBE2D2	75223
chr16	21610713	21611002	1.17	7.08E-01	SLC7A5P1	-79093	IGSF6	53114
chr14	51706759	51707148	1.10	7.08E-01	TMX1	68		
chr19	39420819	39421750	1.05	7.08E-01	MRPS12	-309	SARS2	251
chr11	47429747	47430018	0.88	7.08E-01	SLC39A13	-168		
chr6	16421053	16421389	1.03	7.08E-01	GMPR	182410	ATXN1	340500
chr6	88639554	88639816	0.94	7.08E-01	AKIRIN2	-227700	SPACA1	-117822
chr1	23852191	23852630	0.83	7.09E-01	ASAP3	-41661	E2F2	5302
chr17	21251986	21252390	0.70	7.09E-01	KCNJ12	-27511	MAP2K3	64220
chr8	101162713	101163041	0.88	7.09E-01	FBXO43	-4801	POLR2K	38
chr19	18391095	18391504	1.06	7.09E-01	KIAA1683	-5981	JUND	1132
chr12	113506190	113506501	0.79	7.09E-01	DTX1	10684	RASAL1	67675

chr12	56109698	56109990	1.23	7.10E-01	RDH5	-4307	BLOC1S1	24
chr12	118810756	118811023	0.95	7.10E-01	SUDS3	-3468	TAOK3	-140
chr17	62971474	62971834	1.09	7.10E-01	LRRC37A3	-56666	GNA13	81266
chr19	4204635	4204891	1.06	7.10E-01	EBI3	-24777	SIRT6	-22167
chr6	52255160	52255491	1.18	7.10E-01	EFHC1	-29668	PAQR8	28400
chr17	47269765	47270398	0.89	7.10E-01	GNGT2	16661	B4GALNT2	59753
chr5	137911105	137911680	0.96	7.10E-01	HSPA9	-278		
chr7	75923979	75924274	0.88	7.10E-01	HSPB1	-7748	MDH2	246734
chr16	28961890	28962333	0.98	7.11E-01	NFATC2IP	-206		
chr12	38710346	38710677	1.17	7.11E-01	ALG10B	-45		
chr9	6758881	6759441	1.05	7.12E-01	KDM4C	1520		
chr16	71598543	71599155	1.17	7.12E-01	TAT	12149	CHST4	38788
chr17	4852639	4853003	1.26	7.12E-01	ENO3	-1565	PFN1	-996
chr5	179720467	179720781	0.88	7.12E-01	MAPK9	-1553		
chr19	44259512	44259879	1.12	7.12E-01	C19orf61	-554		
chr11	72759167	72759470	1.33	7.12E-01	FCHSD2	93824	ATG16L2	233868
chr20	62359980	62360275	1.23	7.12E-01	LIME1	-7005	ZGPAT	20737
chr21	34755538	34755921	0.95	7.12E-01	IFNGR2	-19472	IFNAR1	58516
chr11	75525755	75526152	1.12	7.12E-01	UVRAG	-258		
chr7	98978525	98978961	1.16	7.12E-01	ARPC1B	6414	PDAP1	27549
chr3	46530228	46530574	1.09	7.12E-01	LTF	-24006	RTP3	-9084
chr7	128379163	128379679	0.90	7.12E-01	CALU	75		
chr19	47289548	47290080	1.12	7.12E-01	STRN4	-40094	SLC1A5	2028
chr9	101077869	101078213	1.18	7.12E-01	TBC1D2	-60038	GABBR2	393134
chr2	70313905	70314260	1.06	7.12E-01	PCBP1	-502		
chr11	46958091	46958414	1.16	7.12E-01	LRP4	-18177	ARFGAP2	240166
chr16	30007383	30007693	1.07	7.12E-01	HIRIP3	-151		
chr16	115700	115950	1.15	7.12E-01	RHBDF1	6804	SNRNP25	11996
chr13	52368048	52368442	1.00	7.13E-01	DHRS12	10048	WDFY2	209761
chr11	9482079	9482325	0.93	7.13E-01	ZNF143	-311		
chr10	33269491	33269837	0.87	7.13E-01	ITGB1	-22371	NRP1	354169
chr16	740283	740577	1.14	7.13E-01	STUB1	10315	FBXL16	15378
chr12	129314379	129314761	1.05	7.13E-01	GLT1D1	-23511	SLC15A4	-6029
chr7	22149945	22150194	0.83	7.13E-01	CDCA7L	-164528	RAPGEF5	246463
chr19	37569020	37569475	0.98	7.13E-01	ZNF420	-134		
chr12	98910195	98910500	1.00	7.14E-01	TMPO	939		
chr11	111317284	111317653	1.14	7.14E-01	POU2AF1	-67312	BTG4	65595
chr6	26273077	26273482	0.81	7.14E-01	HIST1H3G	-1668	HIST1H2BI	76

chr15	40407987	40408297	1.05	7.14E-01	BUB1B	-45068	BMF	-7067
chr17	40558133	40558507	1.05	7.14E-01	STAT3	-17807	PTRF	16954
chr4	40994023	40994378	0.61	7.14E-01	APBB2	222434	NSUN7	242287
chr5	133968406	133968718	1.16	7.14E-01	SAR1B	-35		
chr5	153418290	153418691	0.96	7.14E-01	MFAP3	-28	FAM114A2	6
chr2	208490059	208490348	1.13	7.14E-01	FAM119A	-231		
chr12	125391315	125391593	1.08	7.15E-01	SCARB1	-42935	UBC	8123
chr17	8028911	8029212	0.96	7.15E-01	HES7	-1660		
chr12	92566406	92566800	0.94	7.15E-01	PLEKHG7	-563662	BTG1	-26930
chr20	61569143	61569405	1.07	7.15E-01	C20orf11	-167	DIDO1	0
chr8	100253056	100253366	0.84	7.15E-01	VPS13B	227717	COX6C	652684
chr12	123464934	123465341	0.95	7.15E-01	ARL6IP4	258		
chr16	66914139	66914620	0.86	7.15E-01	PDP2	-56		
chr17	66015888	66016356	1.20	7.15E-01	C17orf58	-26357	KPNA2	-15726
chr17	56410408	56410912	1.27	7.15E-01	BZRAP1	-4508		
chr1	205225345	205225589	0.92	7.15E-01	TMCC2	28376	NUAK2	65416
chr3	46580261	46580567	1.25	7.15E-01	LRRC2	27597	RTP3	40929
chr12	110939875	110940235	1.21	7.15E-01	VPS29	-139	RAD9B	50
chr19	18263233	18263597	0.99	7.15E-01	PIK3R2	-601		
chr5	1336198	1336483	1.20	7.15E-01	TERT	-41179	CLPTM1L	8661
chr12	54069810	54070170	1.20	7.16E-01	ATP5G2	119		
chr11	118187374	118187643	0.89	7.16E-01	CD3E	12214	CD3D	25950
chr3	47563644	47563920	1.08	7.16E-01	SCAP	-46337	CSPG5	56577
chr1	22109595	22109858	1.14	7.17E-01	USP48	-39		
chr10	556945	557235	0.92	7.17E-01	DIP2C	178518	ZMYND11	376666
chr8	22831713	22832084	0.80	7.17E-01	PEBP4	-46478	RHOBTB2	-21462
chr2	113403061	113403371	0.76	7.17E-01	SLC20A1	-311		
chr14	70081390	70081666	0.92	7.18E-01	SFRS5	-152306	KIAA0247	3218
chr21	30445974	30446312	1.19	7.18E-01	CCT8	-133		
chr16	1401796	1402055	0.97	7.18E-01	C16orf42	-53	GNPTG	-6
chr6	26521294	26521605	1.03	7.18E-01	HMGN4	-17122	BTN1A1	19955
chr12	56320756	56321062	0.90	7.18E-01	DGKA	-4903	WIBG	788
chr3	45079252	45079540	1.25	7.18E-01	CLEC3B	11637	CDCP1	108518
chr1	23345684	23346014	1.12	7.18E-01	KDM1A	-92		
chr12	69139542	69140022	0.90	7.18E-01	SLC35E3	-154		
chr13	59965790	59966100	0.95	7.18E-01	DIAPH3	772174		
chr20	18118252	18118572	0.88	7.18E-01	CSRP2BP	-115		
chr6	33756612	33757183	1.12	7.18E-01	LEMD2	8		

chr18	13612176	13612669	1.14	7.18E-01	C18orf19	114168	C18orf1	393637
chr19	34625087	34625581	1.17	7.18E-01	CR622909	-41121	LSM14A	-38018
chr19	45596041	45596341	1.12	7.18E-01	GEMIN7	13673	NKPD1	67217
chr2	220042746	220042995	0.99	7.18E-01	C2orf24	-1169	FAM134A	-68
chr10	112602863	112603173	0.93	7.19E-01	PDCD4	-28578	RBM20	198863
chr12	98909099	98909471	0.94	7.19E-01	TMPO	-124		
chr7	47614073	47614594	0.95	7.19E-01	C7orf69	-220555	TNS3	-35135
chr12	15114330	15114761	0.89	7.20E-01	ARHGDIB	16		
chr10	70287157	70287483	0.80	7.20E-01	SLC25A16	264		
chr14	23039076	23039458	0.80	7.20E-01	DAD1	18876	TCRDV2	111178
chr4	4291820	4292071	1.11	7.20E-01	LYAR	-50	ZNF509	22
chr2	44395134	44395617	1.01	7.20E-01	PPM1B	-624		
chr11	65266307	65266651	1.32	7.20E-01	SCYL1	-26069	FRMD8	112438
chr17	66031619	66031918	1.04	7.20E-01	KPNA2	-79		
chr21	26979671	26979984	1.27	7.20E-01	MRPL39	-27		
chr2	70520597	70520848	1.17	7.21E-01	SNRPG	146		
chr20	62169128	62169496	1.17	7.21E-01	PTK6	-605		
chr4	154340825	154341112	1.25	7.21E-01	KIAA0922	-46529	MND1	75168
chr2	120124257	120124572	0.82	7.22E-01	DBI	-89		
chr15	76603587	76603955	1.06	7.22E-01	ETFA	39		
chr17	41400587	41400866	0.74	7.22E-01	ARL4D	-75626	TMEM106A	36833
chr13	49684240	49684550	1.42	7.22E-01	MLNR	-110079	FNDC3A	134347
chr4	169931438	169931830	1.16	7.22E-01	CBR4	-212		
chr12	7047129	7047452	1.14	7.23E-01	PTPN6	-8449	ATN1	13665
chr1	51425821	51426164	1.08	7.23E-01	FAF1	-57		
chr19	10230439	10230807	1.11	7.23E-01	EIF3G	-24		
chr20	48225489	48225738	1.34	7.23E-01	PTGIS	-40907	B4GALT5	104807
chr17	29158757	29159074	1.16	7.23E-01	ATAD5	-107		
chr17	34842230	34842543	1.14	7.23E-01	ZNHIT3	-86		
chr19	9546000	9546401	1.12	7.23E-01	ZNF266	33		
chr13	49821898	49822163	1.04	7.23E-01	CDADC1	-72		
chr10	74080553	74080914	0.85	7.23E-01	DNAJB12	34173	DDIT4	47057
chr17	62962036	62962390	1.20	7.23E-01	LRRC37A3	-47225	GNA13	90707
chr6	49430898	49431175	1.15	7.23E-01	CENPQ	-59	MUT	-6
chr1	154989989	154990299	0.95	7.23E-01	ZBTB7B	15032	DCST2	16113
chr10	102046365	102046695	1.02	7.24E-01	BLOC1S2	-91		
chr7	148843973	148844648	0.77	7.24E-01	ZNF398	-249		
chr1	35318290	35318615	1.03	7.24E-01	C1orf212	6939	GJA4	59854

chr10	6389862	6390570	1.26	7.24E-01	PFKFB3	145376	PRKCQ	232022
chr12	4758096	4758361	1.17	7.24E-01	AKAP3	-3871	NDUFA9	-54
chr12	8845283	8845519	0.72	7.24E-01	MFAP5	-29968	RIMKLB	-5117
chr3	57050776	57051142	1.07	7.24E-01	ERC2	-548568	ARHGEF3	62377
chr6	47277653	47278015	1.08	7.24E-01	TNFRSF21	-154		
chr9	35096429	35096771	1.07	7.24E-01	PIGO	-54		
chr15	78251737	78252047	0.94	7.24E-01	LINGO1	-327183	TBC1D2B	118102
chr6	27870521	27870906	1.11	7.24E-01	OR2B2	9383	HIST1H2BO	9511
chr7	26240150	26241053	0.90	7.24E-01	CBX3	-497	HNRNPA2B1	-189
chr14	75593385	75593883	0.96	7.24E-01	NEK9	144		
chr15	90016054	90016364	1.18	7.24E-01	POLG	-138183	RHCG	23590
chr12	106695522	106695799	1.15	7.25E-01	POLR3B	-55775	CKAP4	-53948
chr1	224380188	224380443	1.24	7.25E-01	DEGS1	9388	NVL	137556
chr22	23611657	23612260	1.08	7.25E-01	BCR	89407	IGLL1	310536
chr3	38065785	38066061	0.92	7.25E-01	PLCD1	355		
chr9	6015538	6015786	0.97	7.26E-01	RANBP6	-44		
chr1	93811279	93811555	1.10	7.26E-01	DR1	-61		
chr1	207999209	207999505	1.21	7.26E-01	CD46	73955	CD34	85326
chr7	151065196	151065502	0.87	7.26E-01	NUB1	26491	RHEB	151661
chr17	1620282	1620692	1.11	7.26E-01	PRPF8	-32311	SERPINF2	-25643
chr7	155090744	155091035	1.03	7.26E-01	EN2	-159934	INSIG1	1404
chr17	71187658	71188028	0.84	7.27E-01	COG1	-1330		
chr2	114514228	114514492	0.90	7.27E-01	SLC35F5	40		
chr11	108093483	108094005	1.15	7.27E-01	NPAT	-379	ATM	185
chr19	17326042	17326352	0.96	7.27E-01	USE1	42		
chr6	2862917	2863685	0.90	7.28E-01	SERPINB1	-21220	SERPINB9	40244
chr14	61547343	61547653	0.98	7.28E-01	SLC38A6	99618	TMEM30B	201032
chr19	1174125	1174389	1.06	7.28E-01	SBNO2	25		
chr19	663159	663411	0.90	7.28E-01	RNF126	-58		
chr6	33267026	33267341	0.93	7.28E-01	RGL2	-446		
chr8	95565757	95566067	0.92	7.28E-01	KIAA1429	-224		
chr15	85131248	85131583	1.18	7.28E-01	ZSCAN2	-12833	ADAMTSL3	808578
chr19	7082975	7083254	0.92	7.28E-01	ZNF557	13644	INSR	210896
chr5	118367117	118367361	1.34	7.29E-01	TNFAIP8	-324357		
chr1	205719332	205719674	1.17	7.29E-01	NUCKS1	-142		
chr3	53164339	53164614	0.87	7.29E-01	RFT1	-7		
chr11	58345030	58345367	1.13	7.29E-01	LPXN	-1809	ZFP91	-1388
chr7	134966299	134966550	1.12	7.29E-01	STRA8	49694	CNOT4	228426

chr13	95201066	95201646	0.86	7.29E-01	DCT	-69420	TGDS	47155
chr11	105947737	105948258	0.95	7.29E-01	AASDHPPT	-294	KBTBD3	467
chr2	64977761	64978273	0.99	7.29E-01	SLC1A4	-238439	SERTAD2	-96971
chr12	1058841	1059241	1.16	7.30E-01	RAD52	-178		
chr2	71221923	71222285	0.81	7.30E-01	TEX261	-103		
chr8	96145827	96146083	0.82	7.30E-01	PLEKHF2	-83		
chr8	146017699	146017995	1.04	7.30E-01	RPL8	-42		
chr1	114447462	114448041	0.86	7.30E-01	DCLRE1B	-286	AP4B1	-11
chr10	35485026	35485305	1.11	7.30E-01	CCNY	-140636	CREM	68778
chr10	74079373	74079731	0.82	7.30E-01	DNAJB12	35355	DDIT4	45875
chr10	97516215	97516549	1.41	7.30E-01	CCNJ	-286777	ENTPD1	44846
chr19	11090604	11090976	1.06	7.30E-01	LDLR	-109267	SMARCA4	19192
chr2	201753706	201754205	1.19	7.30E-01	PPIL3	-107	NIF3L1	-94
chr6	26124330	26124661	0.93	7.30E-01	HIST1H2BC	-364	HIST1H2AC	123
chr20	42295331	42295862	1.08	7.30E-01	MYBL2	-112		
chr10	30287286	30287561	0.69	7.30E-01	SVIL	-363523	MTPAP	350843
chr15	38982936	38983254	1.11	7.30E-01	THBS1	-890185	RASGRP1	-126088
chr15	75301342	75301745	1.29	7.30E-01	PPCDC	-14383	SCAMP5	13643
chr17	79227176	79227412	0.85	7.30E-01	C17orf89	14183	SLC38A10	41802
chr9	127027084	127027476	1.16	7.30E-01	NEK6	7037	PSMB7	150441
chr13	21517751	21518014	0.87	7.31E-01	XPO4	-40970	LATS2	117839
chr1	16947559	16947834	0.86	7.31E-01	NBPF1	-7715	MSTP9	143278
chr1	89357153	89357441	1.24	7.31E-01	GTF2B	4		
chr16	70517431	70517762	0.66	7.31E-01	FUK	29099	COG4	39860
chr1	153940138	153940413	0.94	7.32E-01	CREB3L4	-121	SLC39A1	-88
chr1	212458624	212458915	0.93	7.32E-01	PPP2R5A	-109		
chr11	64511128	64511685	0.85	7.32E-01	RASGRP2	922		
chr5	137667246	137667803	1.13	7.32E-01	CDC25C	-9		
chr2	232574964	232575237	1.09	7.32E-01	PTMA	1866	PDE6D	70873
chr4	54232140	54232538	1.06	7.33E-01	SCFD2	-97		
chr6	18387285	18387906	0.84	7.33E-01	RNF144B	2		
chr19	17337165	17337436	1.09	7.33E-01	USE1	11146	NR2F6	18850
chr11	73087165	73087426	1.12	7.33E-01	RELT	-109		
chr20	18488033	18488503	1.20	7.33E-01	SEC23B	80		
chr4	123653512	123653984	1.02	7.33E-01	BBS12	-109		
chr17	79633607	79633851	1.06	7.33E-01	C17orf90	-111		
chr13	41593291	41593643	1.01	7.34E-01	ELF1	41		
chr22	39916337	39916762	1.24	7.34E-01	ATF4	-19		

chr5	163342741	163342995	1.16	7.34E-01	MAT2B	410283		
chr1	206853085	206853443	1.12	7.34E-01	MAPKAPK2	-5025	DYRK3	44383
chr12	4216639	4216912	1.07	7.34E-01	PARP11	-234168	CCND2	-166126
chr17	62502705	62503150	1.11	7.34E-01	DDX5	-444	CCDC45	-230
chr5	131525919	131526229	0.94	7.34E-01	P4HA2	37482	CSF2	116589
chr15	65955929	65956248	1.07	7.34E-01	SLC24A1	41819	DENND4A	128542
chr6	7239064	7239447	0.75	7.34E-01	SSR1	74285	RREB1	131068
chr1	27935213	27935467	1.00	7.34E-01	AHDC1	-5197	FGR	26387
chr15	52519642	52520016	1.24	7.34E-01	GNB5	-36264	MYO5C	68141
chr1	226878992	226879302	0.90	7.34E-01	ITPKB	47729	C1orf95	142646
chr15	40697662	40698028	1.07	7.34E-01	IVD	159		
chr16	30709318	30709567	1.15	7.34E-01	SRCAP	-1019		
chr3	53381507	53381927	0.91	7.34E-01	DCP1A	-80		
chr21	43952940	43953182	0.80	7.34E-01	PDE9A	-120801	SLC37A1	33319
chr17	55927288	55927685	1.10	7.34E-01	MRPS23	-88		
chr2	70314344	70314665	1.08	7.34E-01	PCBP1	-80		
chr17	18528966	18529276	1.17	7.34E-01	TBC1D28	18619	LGALS9C	149022
chr9	37411403	37411716	1.00	7.34E-01	GRHPR	-11147	ZCCHC7	291091
chr22	23301435	23301713	1.17	7.34E-01	GNAZ	-111095	IGL@	71614
chr16	85937170	85937463	0.88	7.35E-01	FOXF1	-606816	IRF8	4543
chr1	174992416	174992762	1.06	7.35E-01	MRPS14	-28		
chr11	59327719	59327999	1.27	7.35E-01	OR4D9	45473	OSBP	55758
chr5	53942526	53942955	1.13	7.35E-01	SNX18	129148	ESM1	338673
chr13	50656551	50656987	1.17	7.36E-01	ST13	-89385	KCNRG	67379
chr14	23476162	23476575	1.15	7.36E-01	JUB	-24521	C14orf93	2991
chr19	45520151	45520474	0.99	7.36E-01	SFRS16	-21985	RELB	15601
chr5	156914416	156914749	1.02	7.36E-01	NIPAL4	27556	ADAM19	88185
chr2	101179180	101179446	1.15	7.36E-01	PDCL3	-105		
chr7	99679312	99679648	0.93	7.36E-01	ZNF3	-109		
chr1	20834560	20834814	0.85	7.36E-01	MUL1	-13		
chr8	11325104	11325425	0.91	7.36E-01	BLK	-26256	AMAC1L2	136770
chr19	54663486	54663802	0.98	7.36E-01	LENG1	-198		
chr20	60961934	60962317	1.07	7.36E-01	RPS21	5		
chrX	119077601	119077911	1.10	7.36E-01	NKAP	-21		
chr11	68034012	68034354	1.15	7.36E-01	SUV420H1	-53399	C11orf24	5286
chr1	10534788	10535120	0.83	7.37E-01	DFFA	-2341	PEX14	-49
chr1	175194641	175194928	1.02	7.37E-01	TNN	157791	TNR	517967
chr2	39005214	39005472	1.06	7.37E-01	GEMIN6	85		

chr16	85343321	85343693	1.05	7.37E-01	KIAA0182	-303417	KIAA0513	282097		
chr3	51536040	51536358	0.93	7.37E-01	VPRBP	-2198				
chr19	12813880	12814162	0.99	7.37E-01	FBXW9	-6566	TNPO2	19441		
chr3	176999162	176999432	1.26	7.37E-01	TBL1XR1	-84249				
chr7	74072737	74073022	0.73	7.37E-01	GTF2I	850				
chr1	47779713	47780199	1.05	7.39E-01	STIL	-137				
chr1	59012360	59012699	1.06	7.39E-01	OMA1	-84				
chr15	40811994	40812340	1.09	7.39E-01	RPUSD2	-49370	CHST14	48954		
chr2	86426469	86426769	1.07	7.39E-01	IMMT	-3726	MRPL35	63		
chr3	193852817	193853097	1.03	7.39E-01	HES1	-977				
chr1	12289889	12290171	0.80	7.39E-01	VPS13D	-83				
chr16	1524944	1525188	1.19	7.39E-01	CLCN7	19				
chr19	39342231	39342647	1.05	7.39E-01	HNRNPL	-1822				
chr2	231921168	231921617	1.03	7.39E-01	PSMD1	-216				
chr19	23456805	23457158	1.14	7.39E-01	AK301230	-23820	ZNF91	121287		
chr3	128336428	128336866	1.09	7.39E-01	GATA2	-124617	RPN1	33072		
chr19	18303552	18303870	1.12	7.40E-01	MPV17L2	-329				
chr15	67401964	67402208	0.99	7.40E-01	SMAD3	43891	AAGAB	144988		
chr12	51477261	51477554	0.84	7.40E-01	CSRNP2	-75				
chr9	130213497	130213935	1.05	7.40E-01	LRSAM1	-76	RPL12	-32		
chr3	152313936	152314246	1.05	7.40E-01	TMEM14E	-255312	P2RY1	-238645		
chr5	140700149	140700529	1.01	7.40E-01	TAF7	12				
chr15	93425405	93425820	1.11	7.40E-01	FAM174B	-226582	CHD2	-17938		
chr10	14879955	14880272	1.09	7.41E-01	HSP60	-147	HSPA14	-147	CDNF	-131
chr6	2932446	2932765	1.04	7.41E-01	SERPINB9	-29061	SERPINB6	39484		
chr17	66168589	66168849	0.90	7.41E-01	AMZ2	-75426	KPNA2	136871		
chr9	123698236	123698663	1.34	7.41E-01	TRAF1	-9277	C5	114104		
chr11	33182907	33183308	1.10	7.41E-01	CSTF3	-71				
chr5	66492491	66492854	1.09	7.41E-01	CD180	-56				
chr20	35471791	35472189	1.16	7.41E-01	DSN1	-69836	SAMHD1	108186		
chr6	13336775	13337019	0.94	7.41E-01	TBC1D7	-8127	GFOD1	150890		
chr7	106808816	106809166	0.88	7.41E-01	HBP1	-469				
chr19	16254214	16254678	1.10	7.41E-01	HSH2D	9608	CIB3	29840		
chr3	196359183	196359686	1.06	7.41E-01	LRRC33	-7221	FBXO45	63710		
chr19	17516368	17516743	0.98	7.41E-01	BST2	-172				
chr20	1447498	1447744	1.17	7.41E-01	NSFL1C	796				
chr11	62432141	62432435	1.05	7.42E-01	METTL12	-491	METTL12	-491		
chr20	42285953	42286302	0.94	7.42E-01	MYBL2	-9581	IFT52	66549		

chr2	68978924	68979270	0.94	7.42E-01	ARHGAP25	17129	BMP10	119552
chr22	43112673	43113094	1.09	7.42E-01	CYB5R3	-67479	A4GALT	3992
chr5	43040349	43040713	0.96	7.42E-01	C5orf39	-84		
chr3	11888257	11888525	1.05	7.43E-01	C3orf31	-39		
chr1	166845525	166845769	1.08	7.43E-01	TADA1	7		
chr2	220159467	220159902	0.86	7.43E-01	PTPRN	14458	DNAJB2	15645
chr1	93544522	93544883	0.91	7.43E-01	MTF2	-89		
chr8	49427065	49427362	0.93	7.44E-01	EFCAB1	220656	UBE2V2	506219
chr4	44680138	44680607	1.15	7.44E-01	GUF1	-60		
chr17	74722843	74723126	1.02	7.44E-01	JMJD6	-104	C17orf95	35
chr1	165737895	165738308	1.22	7.45E-01	TMCO1	33		
chr12	113659004	113659438	0.82	7.45E-01	TPCN1	-39		
chr11	9336043	9336665	1.12	7.45E-01	TMEM41B	-58		
chr1	109102397	109102769	0.86	7.45E-01	PRPF38B	-132349	NBPF6	109679
chr17	18086804	18087229	1.09	7.46E-01	ALKBH5	150		
chr4	3108382	3108626	1.06	7.46E-01	RGS12	-207370	HTT	32096
chr6	31536343	31536698	1.10	7.46E-01	LTA	-3355		
chr1	153769673	153769996	1.14	7.46E-01	SLC27A3	22067	GATAD2B	125616
chr18	42686314	42686616	1.19	7.46E-01	SLC14A2	-508301	SETBP1	425602
chr19	10360785	10361061	0.92	7.46E-01	MRPL4	-1717		
chr1	150848875	150849334	1.00	7.46E-01	ARNT	81		
chr3	52719829	52720258	1.06	7.46E-01	GNL3	108		
chr8	82754335	82755031	0.90	7.47E-01	SNX16	-162		
chr1	28974551	28974861	0.94	7.47E-01	GMEB1	-20538	TAF12	-5102
chr11	67171092	67171499	0.95	7.47E-01	PPP1CA	-1920	TBC1D10C	-116
chr19	1848441	1848914	0.81	7.47E-01	REXO1	-226		
chr15	20561681	20562014	1.15	7.47E-01	HERC2P3	149585		
chr11	118098654	118098918	1.05	7.48E-01	AMICA1	-2977		
chr12	95612606	95612853	1.11	7.48E-01	FGD6	-1490	VEZT	1208
chr11	14521350	14521600	1.09	7.48E-01	COPB1	-60		
chr14	24664502	24665127	1.05	7.49E-01	TM9SF1	67		
chr16	89766622	89766866	1.16	7.49E-01	CDK10	13668	C16orf7	20650
chr6	26554270	26554527	0.95	7.49E-01	ABT1	-42781	HMGN4	15827
chr8	134511547	134511857	1.12	7.49E-01	NDRG1	-202155	ST3GAL1	72481
chr7	127748844	127749215	1.01	7.49E-01	LEP	-132301	LRRC4	-78028
chr16	75589986	75590296	0.84	7.49E-01	TMEM231	29		
chr11	67035070	67035475	0.95	7.49E-01	SSH3	-35646	ADRBK1	1368
chr12	6602292	6602532	1.08	7.49E-01	NCAPD2	-886	MRPL51	59

chr12	108957320	108957654	1.16	7.50E-01	SART3	-2322	ISCU	1193
chr17	2615013	2615276	1.05	7.50E-01	KIAA0664	-218		
chr4	492864	493256	1.11	7.50E-01	PIGG	71	ZNF721	382
chr6	27720983	27721339	0.98	7.51E-01	ZNF184	-280264	HIST1H2BL	54548
chr10	7860240	7860550	0.91	7.51E-01	TAF3	-278		
chr16	89282915	89283193	1.20	7.51E-01	ZNF778	-1057		
chr4	2420506	2420770	1.01	7.51E-01	ZFYVE28	-269		
chr4	2845426	2845666	0.93	7.51E-01	ADD1	-38		
chr1	166059157	166059467	1.43	7.51E-01	POGK	-749412	UCK2	262422
chr6	33168200	33168702	1.10	7.51E-01	SLC39A7	-152	RXRB	-19
chr9	130733083	130733583	1.12	7.51E-01	DPM2	-32570	NAIF1	96266
chr3	9438656	9438939	0.92	7.51E-01	THUMPD3	34081	LHFPL4	156688
chr3	167813457	167813932	1.01	7.51E-01	GOLIM4	-278		
chr3	73551898	73552142	1.31	7.51E-01	PDZRN3	122052	FLJ10213	441210
chr17	17494854	17495260	0.89	7.51E-01	PEMT	-63		
chr5	122759009	122759390	1.16	7.51E-01	CEP120	52		
chr13	73301748	73302076	1.14	7.51E-01	C13orf34	-130	C13orf37	26
chr15	68346195	68346530	0.99	7.52E-01	PIAS1	-209		
chr6	18264881	18265233	1.15	7.52E-01	DEK	-258		
chr7	100025824	100026106	0.91	7.52E-01	MEPCE	-1564	ZCWPW1	337
chr20	1484270	1484580	1.20	7.52E-01	SIRPB2	-12192	SIRPD	53918
chr20	5626014	5626383	0.89	7.52E-01	CHGB	-265775	RP5-1022P6.2	-34527
chr12	57039626	57040019	1.15	7.52E-01	ATP5B	29		
chr12	90274472	90274810	1.15	7.52E-01	ATP2B1	-224797		
chr2	208890146	208890555	1.10	7.52E-01	PLEKHM3	-67		
chr16	84540042	84540356	1.07	7.53E-01	KIAA1609	-1911		
chr19	4638984	4639345	0.99	7.53E-01	SEMA6B	-80664	C19orf10	31250
chr8	110534906	110535162	1.04	7.53E-01	EBAG9	-16895	PKHD1L1	160328
chr12	6772341	6772675	1.16	7.53E-01	ING4	-200		
chr15	34393969	34394332	1.15	7.53E-01	C15orf24	-98		
chr9	34986442	34986752	0.96	7.53E-01	DNAJB5	-3670		
chr3	49058734	49059044	1.01	7.53E-01	DALRD3	-2872	NDUFAF3	-185
chr5	43008705	43009015	0.89	7.53E-01	C5orf39	31587	GHR	584834
chr9	66458015	66458298	1.01	7.53E-01	FAM75A7	-948547		
chr7	127983812	127984142	1.12	7.53E-01	RBM28	-15		
chr1	226862815	226863068	0.98	7.53E-01	ITPKB	63934	C1orf95	126441
chr2	208576038	208576368	1.03	7.53E-01	FAM119A	-86230	FZD5	57940
chr9	37408994	37409583	1.16	7.53E-01	GRHPR	-13418	ZCCHC7	288820

chr15	85174348	85174971	1.13	7.53E-01	SCAND2	-31		
chr10	82231927	82232277	1.04	7.54E-01	SH2D4B	-65556	TSPAN14	18064
chr16	12385886	12386196	0.77	7.54E-01	SNX29	239986	CPPED1	511703
chr18	51795910	51796299	1.19	7.54E-01	POLI	256		
chr20	1099023	1099379	0.94	7.54E-01	PSMF1	-39		
chr19	8645671	8645960	0.95	7.54E-01	MYO1F	-3509		
chr2	223725604	223725946	1.07	7.55E-01	ACSL3	43		
chr3	183229684	183230025	1.09	7.55E-01	MCF2L2	-84000	KLHL6	43644
chr19	13213833	13214228	1.06	7.55E-01	LYL1	-350		
chr4	73935421	73935847	1.19	7.55E-01	COX18	-162		
chr21	43653104	43653473	1.16	7.55E-01	ABCG1	14022	TFF3	82417
chr22	32145894	32146364	0.97	7.55E-01	YWHAH	-194350	PISD	-119319
chr2	183988933	183989305	1.12	7.55E-01	NUP35	36		
chr1	204475521	204475817	1.15	7.55E-01	PIK3C2B	-16195	MDM4	-9842
chr10	74114594	74114838	0.86	7.55E-01	DNAJB12	191		
chr8	56903608	56903918	0.97	7.56E-01	RPS20	83377	LYN	111377
chr11	73881986	73882516	1.13	7.56E-01	C2CD3	-187	PPME1	-117
chr22	46692413	46692756	1.10	7.56E-01	GTSE1	-53		
chr3	31993783	31994100	1.35	7.56E-01	OSBPL10	29296	STT3B	419451
chr7	92861475	92861781	0.92	7.56E-01	HEPACAM2	-5846	CALCR	342190
chr19	46430995	46431305	1.07	7.56E-01	NANOS2	-13114	NOVA2	45507
chr7	105712036	105712400	1.01	7.56E-01	SYPL1	40839	FLJ23834	108561
chr17	40925254	40925568	0.98	7.57E-01	VPS25	-43		
chr3	133380631	133380971	0.98	7.57E-01	TOPBP1	-64		
chr1	24237338	24237703	0.96	7.57E-01	FUCA1	-42700	CNR2	2296
chr1	36929847	36930159	1.01	7.57E-01	MRPS15	37		
chr13	49106754	49107067	0.97	7.57E-01	RCBTB2	405		
chr13	103248517	103248920	0.88	7.57E-01	TPP2	-567		
chr12	50092929	50093272	1.08	7.57E-01	FMNL3	8096	PRPF40B	68773
chr3	187619873	187620465	0.99	7.57E-01	LPP	-310552	BCL6	-156694
chr7	43688342	43688649	1.00	7.57E-01	STK17A	65804	C7orf44	80587
chr12	9102137	9102426	1.15	7.57E-01	M6PR	-30		
chr8	80997334	80997611	1.15	7.57E-01	TPD52	-4463		
chr7	77427853	77428178	0.82	7.57E-01	TMEM60	-269	PHTF2	-93
chr9	123918770	123919039	0.78	7.57E-01	RAB14	45460	CEP110	68331
chr10	73487422	73487858	1.01	7.58E-01	RP11-472K8.2-001	-8062	C10orf54	45697
chr6	3069068	3069563	0.91	7.58E-01	RIPK1	-7682	NQO2	69249
chr16	3018342	3018618	1.01	7.59E-01	PAQR4	-862		

chr16	75551150	75551580	0.82	7.59E-01	CHST6	-22439	CHST5	17703
chr3	53214829	53215101	1.17	7.59E-01	PRKCD	19742	TKT	75103
chr1	1609320	1609630	1.00	7.59E-01	LOC728661	14768	MMP23B	41915
chr14	35590998	35591364	0.98	7.59E-01	KIAA0391	-595	PPP2R3C	338
chr1	161014613	161014958	1.16	7.59E-01	USF1	971		
chr16	70380620	70380951	1.12	7.59E-01	DDX19B	47714	ST3GAL2	92205
chr8	11388384	11388678	0.97	7.59E-01	GATA4	-173186	BLK	37010
chr15	74838640	74838960	0.96	7.59E-01	CLK3	-68535	ARID3B	5252
chr6	8064574	8064914	0.90	7.59E-01	MUTED	-97		
chrX	153191942	153192478	0.93	7.60E-01	ARHGAP4	-512		
chr5	102455781	102456178	1.18	7.60E-01	GIN1	-138		
chr11	64455941	64456360	1.01	7.60E-01	NRXN2	34509	SLC22A12	97869
chr11	2421496	2421963	1.21	7.60E-01	TRPM5	22545	CD81	23183
chr1	1310534	1310940	1.02	7.60E-01	AURKAIP1	-175		
chr17	61920949	61921214	1.17	7.61E-01	SMARCD2	-731		
chr20	46012823	46013133	1.15	7.61E-01	NCOA3	-117679	ZMYND8	-27504
chr7	30067706	30068081	0.97	7.61E-01	FKBP14	-1626	PLEKHA8	-394
chr19	19303227	19303696	0.98	7.61E-01	LOC729991	-62	RFXANK	454
chr1	172787993	172788411	0.96	7.61E-01	FASLG	160017	TNFSF18	231901
chr1	46806283	46806595	0.86	7.62E-01	NSUN4	49		
chr15	75063447	75063771	1.10	7.62E-01	CSK	-10816	CYP1A2	22425
chr8	134461750	134462013	1.10	7.62E-01	NDRG1	-152335	ST3GAL1	122301
chr19	16770733	16771300	1.00	7.62E-01	TMEM38A	-921	C19orf42	-49
chr1	247267460	247267789	1.21	7.63E-01	ZNF669	49		
chr11	73693671	73694040	0.94	7.63E-01	UCP2	33		
chr6	42981696	42982015	1.08	7.63E-01	MEA1	-238	KLHDC3	-121
chr12	120884036	120884387	0.98	7.63E-01	GATC	-72	TRIAP1	3
chr10	35415515	35416183	1.01	7.64E-01	CREM	-539		
chr17	40985280	40985655	1.17	7.64E-01	PSME3	45		
chr21	44313165	44313556	0.98	7.64E-01	NDUFV3	-17		
chr9	2843960	2844379	1.04	7.64E-01	KIAA0020	-40		
chr11	67367836	67368151	1.10	7.64E-01	NDUFV1	-6329	GSTP1	16928
chr6	15901877	15902171	1.04	7.64E-01	DTNBP1	-238753	MYLIP	-227293
chr22	22522255	22522565	1.15	7.64E-01	TOP3B	-185263	VPREB1	-76790
chr10	73610815	73611173	0.84	7.64E-01	PSAP	88		
chr1	45187177	45187550	1.23	7.64E-01	KIF2C	-18126	C1orf228	46970
chr11	111894983	111895293	1.11	7.64E-01	DLAT	-400		
chr11	30364244	30364541	0.96	7.64E-01	FSHB	111830	MPPED2	237648

chr4	87281120	87281480	0.85	7.64E-01	PTPN13	-234168	MAPK10	-5503
chr9	132650683	132651108	0.97	7.65E-01	USP20	53200	FNBP1	154577
chr12	57623249	57623558	1.12	7.65E-01	SHMT2	-148		
chr4	2243534	2244029	0.85	7.65E-01	HAUS3	78		
chr10	27443223	27443473	0.95	7.65E-01	MASTL	-369	YME1L1	-27
chr1	19536083	19536620	0.97	7.66E-01	UBR4	394		
chr11	45951698	45951954	0.95	7.66E-01	GYLTL1B	2355	PHF21A	191159
chr12	113524640	113524888	0.95	7.66E-01	DTX1	29102	RASAL1	49257
chr14	65819860	65820222	0.94	7.67E-01	MAX	-250814	FUT8	-59494
chr17	42422986	42423396	0.96	7.67E-01	GRN	700		
chr19	35815026	35815306	1.31	7.67E-01	CD22	-4913		
chr5	36876597	36876971	1.09	7.67E-01	NIPBL	-77		
chr1	178550355	178550839	1.11	7.67E-01	RALGPS2	-143703	C1orf49	68385
chr19	42449613	42449917	1.06	7.67E-01	RABAC1	13763	ARHGEF1	61319
chr19	56154767	56155077	1.11	7.68E-01	CCDC106	-4032	ZNF581	-64
chr12	53689030	53689565	1.03	7.68E-01	C12orf10	-4172	PFDN5	63
chr20	32950927	32951171	1.09	7.68E-01	ITCH	-13		
chr12	89746364	89746647	0.84	7.68E-01	DUSP6	-210		
chr6	43596978	43597287	0.84	7.68E-01	GTPBP2	-197		
chr12	106751267	106751684	1.06	7.68E-01	POLR3B	40		
chr16	84641412	84641706	0.83	7.68E-01	KIAA1609	-103271	COTL1	10110
chr17	3749501	3749798	1.12	7.68E-01	ITGAE	-45113	CAMKK1	44387
chr6	142468083	142468479	0.93	7.68E-01	VTA1	-129		
chr19	10369725	10369986	1.19	7.69E-01	ICAM1	-11661	MRPL4	7216
chr8	125210132	125210372	0.95	7.69E-01	TMEM65	174688	FER1L6	346025
chr15	59592577	59592848	1.02	7.69E-01	MYO1E	72358	LDHAL6B	93671
chr19	3990259	3990503	1.03	7.69E-01	EEF2	-4920		
chr19	7069571	7069847	1.09	7.69E-01	ZNF557	238		
chr3	135968942	135969252	1.03	7.69E-01	PCCB	-70		
chr8	145597470	145597916	1.09	7.69E-01	ADCK5	-38		
chr15	91236879	91237231	1.05	7.69E-01	BLM	-23524	CRTC3	163857
chr4	154354310	154354596	1.05	7.69E-01	KIAA0922	-33045	MND1	88652
chr14	50100947	50101364	0.93	7.69E-01	C14orf104	792		
chr1	43123854	43124135	1.10	7.69E-01	PPIH	-53		
chr8	23100918	23101297	0.88	7.69E-01	CHMP7	-42		
chr2	48667390	48667696	1.07	7.69E-01	STON1-GTF2A1L	-128616	FOXN2	125748
chr17	16557003	16557247	0.97	7.69E-01	ZNF624	33		
chr13	25496968	25497232	1.16	7.70E-01	CENPJ	-15		

chr5	177026952	177027228	1.12	7.70E-01	B4GALT7	-82		
chr19	47016710	47017004	1.15	7.70E-01	CCDC8	-99938	AK094504	87600
chr1	26872123	26872369	1.12	7.71E-01	RPS6KA1	-97		
chr7	93633549	93633933	1.17	7.71E-01	BET1	-51		
chr9	130788667	130788914	1.26	7.71E-01	DPM2	-88028	NAIF1	40808
chr17	41837810	41838119	1.02	7.71E-01	SOST	-1809		
chr6	29932594	29932939	0.96	7.72E-01	HLA-F	-41450	HLA-A	22436
chr15	42996636	42996912	1.24	7.72E-01	KIAA1300	14981	CDAN1	32643
chr7	65958554	65958808	0.87	7.72E-01	KCTD7	-135209	TPST1	288422
chr14	106178422	106178674	1.04	7.72E-01	IGHE	150914	TMEM121	185595
chr15	45879067	45879641	0.91	7.72E-01	PLDN	-63		
chr19	6522198	6522625	1.13	7.72E-01	TUBB4	-20082	TNFSF9	-8598
chr8	8310197	8310584	0.88	7.73E-01	CLDN23	-249275	PRAGMIN	-71134
chr1	16160845	16161192	0.85	7.73E-01	UQCRHL	-26825	SPEN	-13340
chr1	33116044	33116361	1.12	7.73E-01	RBBP4	-546		
chr13	47169315	47169559	1.08	7.73E-01	LRCH1	42141	ESD	201930
chr6	26311890	26312135	1.27	7.73E-01	BTN3A2	-53385	HIST1H4H	-26286
chr8	42269856	42270167	1.07	7.73E-01	C8orf40	-126286	VDAC3	20666
chr12	56510102	56510628	0.85	7.73E-01	ZC3H10	-1665	RPL41	-9
chr10	11727109	11727374	0.96	7.74E-01	USP6NL	-152968	ECHDC3	-57114
chr17	33416345	33416791	0.98	7.74E-01	RFFL	-274		
chr16	67580908	67581218	0.89	7.74E-01	CTCF	-15401	FAM65A	18309
chr17	76374580	76374836	1.10	7.75E-01	PGS1	-27		
chr2	15282319	15282671	1.13	7.75E-01	DDX1	-449275		
chr7	110723552	110723841	1.11	7.75E-01	LRRN3	-7365		
chr15	49338666	49339033	1.22	7.75E-01	SHC4	-83209	COPS2	109004
chr16	23652581	23652846	0.99	7.75E-01	DCTN5	-93	PALB2	-36
chr2	135208443	135208723	1.02	7.75E-01	MGAT5	196753	TMEM163	267988
chr5	180593892	180594147	1.04	7.75E-01	OR2V2	12077	TRIM7	38157
chr8	145159179	145159423	1.01	7.75E-01	SHARPIN	-161	MAF1	-4
chr15	45003485	45003892	1.03	7.75E-01	B2M	4		
chr11	121319111	121319523	0.87	7.76E-01	SORL1	-3644		
chr8	90996298	90996897	0.90	7.76E-01	NBN	301		
chr19	36248656	36249060	0.92	7.76E-01	HSPB6	-928		
chr1	111764383	111764669	0.87	7.76E-01	DRAM2	-81688	CHI3L2	-5755
chr11	9777990	9778468	1.24	7.76E-01	SWAP70	92601	SBF2	537525
chr12	3913097	3913333	0.74	7.76E-01	EFCAB4B	-50849	PARP11	69393
chr1	94344543	94344960	0.90	7.76E-01	DNTTIP2	-10		

chr5	86708641	86708951	1.01	7.76E-01	CCNH	40		
chr17	74543418	74543715	1.05	7.76E-01	PRCD	7446	ST6GALNAC2	38578
chr18	20938800	20939092	0.79	7.76E-01	C18orf45	78979	CABLES1	223219
chr17	17307235	17307619	1.06	7.77E-01	MED9	-72873	NT5M	100747
chr1	155948243	155948609	0.98	7.77E-01	ARHGEF2	-90		
chr15	67357898	67358252	1.02	7.77E-01	SMAD3	-120		
chr16	5083762	5084072	1.20	7.77E-01	NAGPA	19		
chr19	40727829	40728184	0.93	7.77E-01	TTC9B	-3701		
chr1	94312943	94313253	1.02	7.77E-01	BCAR3	-165713	DNTTIP2	31644
chr14	64970423	64970848	0.97	7.77E-01	ZBTB1	-656	ZBTB25	-82
chr18	13726482	13726915	1.00	7.77E-01	C18orf19	-108	RNMT	-5
chr2	20861244	20861541	1.18	7.77E-01	HS1BP3	-10529	GDF7	-5031
chr9	139117288	139117894	1.21	7.77E-01	LHX3	-22587	QSOX2	20096
chr2	85822615	85822939	1.13	7.77E-01	RNF181	-60		
chr17	62023166	62023437	1.13	7.78E-01	CD79B	-13598	SCN4A	26976
chr10	103113506	103113932	0.98	7.78E-01	BTRC	-106		
chr17	43303488	43303824	1.14	7.78E-01	FMNL1	4364	MAP3K14	90758
chr1	112298168	112298446	0.97	7.78E-01	DDX20	117		
chr1	173793473	173794157	1.22	7.78E-01	DARS2	-143	CENPL	211
chr22	46171394	46171965	1.01	7.78E-01	ATXN10	103993	WNT7B	201328
chr19	45578516	45578836	1.12	7.79E-01	GEMIN7	-3842	ZNF296	1012
chr12	15101607	15102044	1.07	7.79E-01	ERP27	-10363	ARHGDIB	12736
chr2	62115660	62116038	0.95	7.79E-01	CCT4	-58		
chr2	191184435	191184831	0.95	7.79E-01	HIBCH	-74		
chr11	71791710	71792021	1.06	7.79E-01	NUMA1	-293	LRTOMT	484
chr14	51342080	51342422	0.83	7.79E-01	ABHD12B	3373	PYGL	68997
chr17	67577425	67577915	0.88	7.79E-01	KCNJ16	-493756	MAP2K6	166832
chr16	23607502	23607866	0.93	7.80E-01	NDUFAB1	-45		
chr9	127379814	127380130	1.16	7.80E-01	NR5A1	-110273	NR6A1	153604
chr22	38004384	38004762	1.08	7.80E-01	GGA1	70		
chr15	75744200	75744717	0.96	7.80E-01	SIN3A	-533		
chr2	219761211	219761455	1.13	7.80E-01	CDK5R2	-63065	WNT10A	16078
chr11	73490402	73490930	1.11	7.80E-01	RAB6A	-18465	MRPL48	-8251
chr19	4369050	4369477	1.07	7.80E-01	MPND	25740	SH3GL1	31207
chr3	15244221	15244497	0.90	7.81E-01	CAPN7	-3374		
chr15	41408513	41409077	1.10	7.81E-01	INO80	-455		
chr1	226876209	226876519	0.88	7.81E-01	ITPKB	50512	C1orf95	139863
chr14	90865879	90866222	0.82	7.81E-01	CALM1	2678	TTC7B	416710

chr16	28503267	28503563	1.19	7.82E-01	APOB48R	-2578	CLN3	208
chr3	194054550	194054826	1.04	7.82E-01	CPN2	17369	HES1	200754
chr8	145653827	145654085	1.11	7.82E-01	VPS28	-29		
chr1	203296810	203297255	1.02	7.82E-01	BTG2	22369	FMOD	23256
chr19	46026942	46027252	1.02	7.82E-01	VASP	16409	OPA3	61025
chr11	62495003	62495553	0.96	7.83E-01	TTC9C	-674	HNRNPUL2	-457
chr2	27545845	27546247	1.12	7.83E-01	MPV17	-77		
chr1	63988842	63989380	0.88	7.83E-01	ITGB3BP	-276	EFCAB7	98
chr22	36847828	36848072	0.92	7.83E-01	MYH9	-63887	TXN2	29737
chr5	71604126	71604566	0.90	7.83E-01	MRPS27	11738	MAP1B	201228
chr6	7917193	7917493	0.87	7.83E-01	PIP5K1A	-68992	TXNDC5	-6302
chr12	133757816	133758211	0.85	7.84E-01	ZNF268	19		
chr21	44299431	44299839	1.05	7.84E-01	WDR4	43		
chr3	185303694	185304247	1.13	7.84E-01	SENP2	-60		
chr14	24740655	24740930	0.91	7.84E-01	RABGGTA	10		
chr10	94516187	94516620	0.98	7.84E-01	EXOC6	-91821	HHEX	66723
chr5	95144537	95144822	0.94	7.84E-01	GLRX	13897	RHOBTB3	77830
chr20	62495554	62495849	1.09	7.85E-01	TPD52L2	-888		
chr3	13056706	13057016	1.05	7.85E-01	RPL32	-174912	IQSEC1	57756
chr16	67548263	67548584	1.16	7.85E-01	AGRP	-30708	FAM65A	-14330
chr15	101471592	101471970	1.18	7.85E-01	LRRK1	12321	CHSY1	320345
chr16	22308312	22308743	1.07	7.85E-01	POLR3E	-213		
chr21	46359742	46360024	1.18	7.85E-01	C21orf67	-55		
chr5	110074370	110074722	1.04	7.85E-01	SLC25A46	-208		
chr5	78023507	78023848	1.08	7.85E-01	LHFPL2	-79030	ARSB	258679
chr17	78711201	78711521	1.11	7.85E-01	CHMP6	-254280	RPTOR	192736
chr3	57583041	57583708	1.14	7.86E-01	ARF4	-160		
chr3	171757132	171757442	1.04	7.86E-01	FNDC3B	-131		
chr1	51465855	51466116	0.83	7.86E-01	C1orf185	-101920	CDKN2C	30344
chr11	107328489	107328817	1.21	7.86E-01	CWF19L2	-81		
chr3	187653559	187654015	0.93	7.86E-01	LPP	-276934	BCL6	-190312
chr12	69201707	69202068	0.78	7.86E-01	MDM2	-83		
chr5	151151382	151151706	1.13	7.86E-01	G3BP1	68		
chr6	28048546	28048870	0.97	7.87E-01	ZSCAN16	-43679	ZNF165	2136
chr5	180615313	180615654	1.13	7.87E-01	TRIM7	16693	OR2V2	33541
chr6	164088712	164089139	1.24	7.87E-01	QKI	253251		
chr6	37123231	37123658	1.25	7.87E-01	PIM1	-14477	FGD2	150022
chr1	32713826	32714168	0.88	7.88E-01	LCK	-25715	EIF3I	26038

chr22	23227191	23227531	1.02	7.88E-01	IGL@	-2599		
chr18	48778050	48778360	1.00	7.88E-01	MEX3C	-54515		
chr9	139682150	139682439	0.96	7.88E-01	TMEM141	-3482		
chr12	96429341	96429907	1.21	7.88E-01	LTA4H	-259		
chr17	38115240	38115565	1.07	7.88E-01	GSDMA	-3823		
chr5	1792961	1793236	0.93	7.88E-01	LPCAT1	-269023	MRPL36	6857
chr3	123361254	123361535	0.99	7.89E-01	PTPLB	-57471	MYLK	241754
chr16	11775500	11775782	0.86	7.89E-01	SNN	13340	TXNDC11	61007
chr7	8335745	8335995	1.17	7.89E-01	NXPH1	-137715	ICA1	-34188
chr14	65796521	65796899	1.05	7.89E-01	MAX	-227483	FUT8	-82825
chr8	145159697	145159996	1.00	7.90E-01	SHARPIN	-707	MAF1	542
chr7	102066284	102066638	0.90	7.90E-01	ORAI2	-7535	PRKRIP1	29657
chr7	132937612	132937862	1.00	7.90E-01	EXOC4	-86		
chr3	69133922	69134232	1.15	7.90E-01	UBA3	-4553	ARL6IP5	-13
chr10	103124491	103124801	0.90	7.90E-01	BTRC	10821	POLL	223327
chr6	11575810	11576078	1.06	7.91E-01	TMEM170B	37433	C6orf105	203336
chr10	14996055	14996534	0.93	7.91E-01	DCLRE1C	-201		
chr19	48103666	48103964	0.93	7.91E-01	ZNF541	-44702	GLTSCR1	-7638
chr20	30154316	30154871	0.98	7.91E-01	ID1	-38498	HM13	52353
chr22	38054159	38054455	0.90	7.91E-01	LGALS1	-17306	SH3BP1	19250
chr22	40439971	40440824	0.93	7.92E-01	TNRC6B	-133531	GRAP2	143312
chr19	12721445	12721727	0.95	7.92E-01	ZNF791	-146	ZNF490	37
chr14	70826362	70826641	1.03	7.92E-01	COX16	-58		
chrX	48815542	48815981	1.07	7.92E-01	OTUD5	-869		
chr1	23946237	23946663	0.91	7.92E-01	RPL11	-71844	ID3	-60128
chr13	33927426	33927689	0.95	7.92E-01	RFC3	-464648	STARD13	-67657
chr19	49496536	49497298	0.83	7.92E-01	GYS1	-307	RUVBL2	-239
chr13	103498007	103498422	0.99	7.93E-01	ERCC5	38719	SLC10A2	220981
chr20	31073031	31073385	0.87	7.93E-01	ASXL1	127055	COMMD7	258606
chr15	68966335	68966645	0.91	7.93E-01	CORO2B	94917	ANP32A	146771
chr14	35761479	35761832	0.91	7.93E-01	PSMA6	82		
chr1	54303952	54304428	0.93	7.93E-01	TMEM48	-15		
chr11	71689205	71689454	0.90	7.93E-01	IL18BP	-20628	RNF121	49562
chr7	134143858	134144168	0.87	7.93E-01	AKR1B1	-125		
chr14	39583272	39583561	1.13	7.93E-01	SIP1	-71		
chr5	149840873	149841248	1.02	7.94E-01	NDST1	-46613	RPS14	-11742
chr17	57696446	57697399	1.15	7.94E-01	CLTC	-127		
chr6	17600302	17600638	0.95	7.94E-01	FAM8A1	-48		

chr12	54140459	54140727	0.84	7.94E-01	HOXC13	-191983	CALCOCO1	-19286
chr13	74707908	74708218	1.12	7.94E-01	KLF12	3		
chr1	113498712	113499106	1.03	7.94E-01	SLC16A1	66		
chr14	31889855	31890166	1.07	7.94E-01	HEATR5A	-31800	C14orf126	36669
chr19	48866326	48866759	0.87	7.94E-01	SYNGR4	-1114	TMEM143	643
chr20	44600706	44601186	1.04	7.95E-01	ZNF335	-113		
chr17	37309136	37309398	0.94	7.95E-01	PLXDC1	-1365		
chr6	28049223	28049486	1.09	7.95E-01	ZSCAN16	-43032	ZNF165	2783
chr11	17099220	17099521	1.11	7.95E-01	RPS13	-151		
chr15	94831052	94831362	1.04	7.95E-01	MCTP2	-10223		
chr17	27717111	27717402	1.05	7.95E-01	TAOK1	-686		
chr18	11851118	11851516	0.85	7.95E-01	CHMP1B	-72		
chr6	26204650	26205264	0.90	7.95E-01	HIST1H4E	84		
chr13	38407612	38407888	1.10	7.95E-01	POSTN	-234769	TRPC4	36189
chr15	52525612	52526001	1.07	7.95E-01	GNB5	-42242	MYO5C	62163
chr22	39842878	39843189	1.12	7.95E-01	MGAT3	-40195	MAP3K7IP1	47275
chr7	129712053	129712312	1.12	7.95E-01	ZC3HC1	-20950	TMEM209	133155
chr2	128615607	128615952	0.94	7.95E-01	POLR2D	-51		
chr16	12415319	12415637	0.96	7.95E-01	SNX29	269423	CPPED1	482266
chr18	42684047	42684334	1.21	7.95E-01	SLC14A2	-510575	SETBP1	423328
chr4	153700702	153701147	1.10	7.95E-01	ARFIP1	-187	TIGD4	-48
chr10	70480680	70480996	0.93	7.95E-01	CCAR1	-133		
chr11	62521362	62521811	1.06	7.95E-01	ZBTB3	69		
chr9	33722102	33722412	1.29	7.95E-01	PTENP1	-44839	PRSS3	-28258
chr2	68654205	68654638	1.10	7.96E-01	APLF	-40269	PLEK	62100
chr9	27573664	27573967	1.10	7.96E-01	MOBKL2B	-43966		
chr16	30825675	30825962	0.99	7.96E-01	ZNF629	-27296	BCL7C	79580
chr1	70671137	70671470	0.93	7.96E-01	SFRS11	-61	LRRC40	-29
chr17	7155600	7155962	1.08	7.96E-01	DULLARD	-786	C17orf81	230
chr9	36258326	36258619	1.09	7.96E-01	GNE	18568	CLTA	67581
chr7	26438623	26439112	0.89	7.96E-01	SNX10	107353	SKAP2	465473
chr13	41634784	41635099	1.14	7.96E-01	WBP4	-755		
chr1	63249657	63249987	0.80	7.97E-01	ATG4C	19		
chr13	111108288	111108571	0.92	7.97E-01	RAB20	105641	COL4A2	148799
chr12	113658710	113659001	0.78	7.97E-01	TPCN1	-404		
chr10	75012344	75012605	0.88	7.97E-01	MRPS16	-24		
chr12	108959788	108960105	0.93	7.97E-01	SART3	-4782	ISCU	3653
chr14	50426745	50427051	1.03	7.97E-01	ARF6	67162	SOS2	271201

chr20	31490341	31490651	0.94	7.97E-01	EFCAB8	43767	SPAG4L	101743		
chr19	52772589	52772952	0.98	7.97E-01	ZNF766	-53				
chr2	27994495	27994835	1.17	7.97E-01	MRPL33	81				
chr10	104192280	104192621	0.97	7.97E-01	CUEDC2	-28				
chr1	10010446	10010713	0.83	7.97E-01	RBP7	-46675	NMNAT1	7094		
chr10	1505291	1505585	0.74	7.97E-01	IDI1	-410377	ADARB2	274280		
chr15	86018577	86018887	1.22	7.97E-01	AKAP13	94861	KLHL25	319457		
chr16	14379560	14380000	1.24	7.97E-01	MKL2	214584	PARN	344344		
chr16	81865284	81865703	1.19	7.97E-01	PLCG2	52564	SDR42E1	179599		
chr6	26020422	26020866	0.94	7.97E-01	HIST1H1A	-2604	HIST1H4A	-1263	HIST1H3A	-74
chr21	11144414	11144883	1.02	7.97E-01	BAGE	-45712				
chr5	79551875	79552202	1.11	7.97E-01	SERINC5	-169				
chr1	41078012	41078262	0.93	7.98E-01	RIMS3	53187	ZNF684	80904		
chr14	96133650	96134027	0.94	7.99E-01	TCL1A	46694	GLRX5	132516		
chr6	41673904	41674213	1.28	7.99E-01	TFEB	17405	MDFI	67864		
chr3	193290242	193290488	0.98	8.00E-01	OPA1	-20568	ATP13A4	-17669		
chr19	40324174	40324484	0.91	8.00E-01	DYRK1B	512				
chr14	107175134	107175454	0.98	8.00E-01	ADAM6	-736936	IGHV7-81	107989		
chr19	13885061	13885426	1.21	8.00E-01	ZSWIM4	-21030	MRI1	9907		
chr12	123459105	123459359	0.94	8.00E-01	OGFOD2	-122				
chr20	32470839	32471201	1.26	8.00E-01	RALY	-110712	CHMP4B	71901		
chr2	204571164	204571474	1.20	8.00E-01	CD28	121				
chr22	23207962	23208432	0.83	8.00E-01	IGL@	-21763	GGTLC2	219415		
chr16	89160069	89160391	1.08	8.01E-01	ACSF3	-24				
chr1	229694518	229694788	1.00	8.01E-01	ABCB10	-211				
chr7	102004077	102004339	1.05	8.01E-01	PRKRIP1	-32596	SH2B2	75803		
chr22	50630901	50631230	1.00	8.01E-01	PANX2	21906	TUBGCP6	52334		
chr16	75681541	75681868	1.02	8.01E-01	KARS	-120	TERF2IP	21		
chr7	102082100	102082551	0.90	8.01E-01	ORAI2	8330	ALKBH4	22962		
chr1	45477657	45477978	0.88	8.01E-01	HECTD3	-791	UROD	-12		
chr1	155220481	155220791	1.00	8.01E-01	GBA	-9583	FAM189B	4638		
chr15	66161277	66161584	1.09	8.01E-01	RAB11A	-365				
chr20	36322034	36322469	1.22	8.01E-01	CTNNBL1	-182				
chr3	194403342	194403671	0.94	8.01E-01	LSG1	-10301	C3orf21	588388		
chr17	72199550	72199871	1.24	8.02E-01	RPL38	-84				
chr1	153518140	153518554	1.16	8.02E-01	S100A5	-4106	S100A4	-65		
chr1	1167278	1167727	0.98	8.02E-01	B3GALT6	-126	SDF4	-56		
chr15	93447334	93447911	1.15	8.02E-01	CHD2	4072	RGMA	184810		

chr2	47882352	47882614	0.95	8.02E-01	MSH6	-127738	KCNK12	-85013
chr21	36399013	36399427	1.10	8.02E-01	C21orf96	12503	CLIC6	357532
chr7	152038182	152038444	1.07	8.02E-01	MLL3	94777	GALNT11	315535
chr1	23855111	23855468	0.70	8.02E-01	ASAP3	-44540	E2F2	2423
chr1	6614415	6614845	0.89	8.02E-01	TAS1R1	-804	NOL9	-49
chr19	11671379	11671689	1.19	8.04E-01	ELOF1	-1483		
chr10	64413022	64413344	1.10	8.04E-01	ADO	-151333	ZNF365	279267
chr19	14530213	14530563	1.14	8.04E-01	DDX39	-217		
chr14	77843609	77843858	0.79	8.04E-01	C14orf148	45645	GSTZ1	56504
chr16	89131563	89131873	1.10	8.04E-01	CBFA2T3	-88317	ACSF3	-28536
chr3	56528766	56529013	1.15	8.04E-01	ERC2	-26499	ARHGEF3	584446
chr9	124734794	124735349	0.84	8.05E-01	TTLL11	120813	DAB2IP	405673
chr21	11128402	11128680	1.13	8.05E-01	BAGE	-29604		
chr3	99979617	99980145	1.08	8.05E-01	TBC1D23	195		
chr7	122184555	122184865	1.09	8.05E-01	FEZF1	-240145	RNF133	154498
chr22	41682306	41682645	1.02	8.05E-01	RANGAP1	-260		
chr4	122745009	122745319	0.78	8.06E-01	CCNA2	-76		
chr5	177547907	177548297	0.93	8.06E-01	N4BP3	7546	NHP2	32859
chr11	66206273	66206687	1.02	8.06E-01	MRPL11	-170		
chr17	8124131	8124441	1.15	8.06E-01	AURKB	-10403	C17orf68	27127
chr3	8700139	8700416	0.84	8.06E-01	CAV3	-75218	LMCD1	156767
chr17	47824522	47824876	0.88	8.06E-01	MYST2	-41372	SLC35B1	-39417
chr17	26972072	26972316	1.06	8.06E-01	KIAA0100	-21		
chr12	25084673	25084912	1.29	8.06E-01	SOX5	-982156	BCAT1	17515
chr9	139622514	139622800	1.04	8.06E-01	LCN10	14757	FAM69B	15633
chr6	13356266	13356572	0.98	8.07E-01	TBC1D7	-27649	GFOD1	131368
chr7	126336753	126337049	1.35	8.07E-01	GRM8	546668		
chr8	100905815	100906059	1.01	8.07E-01	COX6C	-42		
chr12	46780699	46781028	0.92	8.07E-01	SLC38A2	-14219	SLC38A4	438916
chr12	77157830	77158171	1.15	8.07E-01	ZDHHC17	147		
chr17	66233501	66233848	1.17	8.08E-01	AMZ2	-10470	KPNA2	201827
chr2	225450357	225450730	0.85	8.08E-01	CUL3	-434		
chr2	65144815	65145059	1.04	8.08E-01	SERTAD2	-263891	SLC1A4	-71519
chr11	64616214	64616467	1.05	8.08E-01	CDC42BPG	-4300		
chr11	3078570	3078850	1.11	8.09E-01	CARS	-39		
chr20	30697070	30697485	1.07	8.09E-01	TM9SF4	-31		
chr1	67390696	67391081	0.99	8.09E-01	MIER1	311		
chr10	13203170	13203494	0.87	8.09E-01	MCM10	-249		

chr12	120907332	120907754	0.91	8.09E-01	SFRS9	15		
chr1	114889222	114889498	0.91	8.09E-01	SYT6	-192888	TRIM33	164421
chr5	130718105	130718400	1.02	8.09E-01	CDC42SE2	118551	RAPGEF6	252676
chr18	3297431	3297738	1.00	8.10E-01	TGIF1	-154006	MYL12B	35474
chr19	3986206	3986485	0.98	8.11E-01	EEF2	-885		
chr19	58790104	58790395	1.02	8.11E-01	ZNF8	-68		
chr20	29611681	29612019	1.00	8.11E-01	DEFB115	-233617		
chr22	50683370	50683616	0.92	8.11E-01	TUBGCP6	-93		
chr17	62982082	62982637	1.14	8.11E-01	LRRC37A3	-67372	GNA13	70560
chr20	46045420	46045819	1.18	8.11E-01	NCOA3	-85037	ZMYND8	-60146
chr17	30669067	30669377	1.06	8.11E-01	C17orf75	-33		
chr6	43395118	43395610	0.96	8.12E-01	ABCC10	72		
chr17	75865621	75866095	0.90	8.12E-01	TNRC6C	-134460	SEPT9	588366
chr19	9695027	9695337	1.06	8.12E-01	ZNF121	27		
chr4	47465600	47465936	1.02	8.12E-01	COMMD8	-92		
chr12	54752968	54753328	0.91	8.12E-01	GPR84	5110	COPZ1	34237
chr11	111667820	111668086	0.84	8.12E-01	PPP2R1B	-30784	ALG9	74352
chr13	48669156	48669506	0.97	8.12E-01	MED4	-91		
chr12	109490184	109490523	0.91	8.13E-01	USP30	-26		
chr10	103989572	103989854	0.79	8.13E-01	ELOVL3	3570	PITX3	11518
chr1	231376768	231377258	0.87	8.13E-01	GNPAT	94		
chr15	52971340	52971827	0.85	8.13E-01	ARPP19	-110371	ONECUT1	110625
chr17	56406748	56407492	1.29	8.13E-01	BZRAP1	-968		
chr20	62612216	62612474	1.19	8.13E-01	PRPF6	-86		
chr3	101280539	101280896	1.23	8.14E-01	RG9MTD1	6		
chr17	5372272	5372558	1.21	8.14E-01	DHX33	-234		
chr2	9771282	9771592	0.97	8.14E-01	YWHAQ	-331		
chr6	26285407	26285936	0.96	8.14E-01	HIST1H4H	55		
chr11	126350604	126350914	0.94	8.14E-01	ST3GAL4	124663	KIRREL3	520007
chr22	35653290	35653600	0.92	8.14E-01	HMGXB4	0		
chr11	61584224	61584576	1.15	8.14E-01	FADS1	129		
chr8	8602184	8602581	1.01	8.15E-01	CLDN23	42717	MFHAS1	148748
chr16	29827072	29827659	0.97	8.15E-01	MVP	-4421		
chr7	139045094	139045417	1.06	8.15E-01	LUC7L2	622		
chr1	25566522	25566805	0.90	8.15E-01	RHD	-32317	SYF2	-7651
chr1	25573662	25574206	0.91	8.15E-01	RHD	-25047	SYF2	-14921
chr2	61220241	61220574	1.15	8.15E-01	PEX13	-24404	REL	111656
chr1	205304786	205305097	1.17	8.15E-01	NUAK2	-14059	LEMD1	86239

chr11	66360495	66360768	1.02	8.16E-01	CCS	-58		
chr5	93954184	93954540	0.94	8.16E-01	ANKRD32	-29		
chr19	10755480	10755756	0.90	8.16E-01	ILF3	-9319	SLC44A2	19447
chr12	14956445	14956814	1.08	8.16E-01	WBP11	-229		
chr12	49524404	49524687	1.08	8.16E-01	TUBA1B	758		
chr13	53495297	53495649	1.12	8.16E-01	OLFM4	-107499	PCDH8	-72699
chr19	6460555	6460865	1.16	8.16E-01	CRB3	-3550	SLC25A23	-929
chr8	101450283	101450612	0.95	8.16E-01	RNF19A	-134961	ANKRD46	121564
chr17	46185034	46185294	1.17	8.16E-01	SNX11	244		
chr5	40835259	40835569	1.14	8.16E-01	RPL37	-27		
chr2	174856267	174856659	1.03	8.16E-01	SP3	-26400	OLA1	256902
chr5	138945012	138945349	0.93	8.16E-01	CXXC5	-83120	UBE2D2	4430
chr19	41222385	41222832	0.99	8.16E-01	ITPKC	-399	ADCK4	181
chr16	56998482	56998805	0.90	8.16E-01	NLRC5	-24766	CETP	2809
chr1	182808591	182808912	1.05	8.17E-01	DHX9	301		
chr17	28256774	28257081	1.07	8.17E-01	SSH2	90		
chr11	72865179	72865643	1.08	8.17E-01	P2RY2	-63933	FCHSD2	-12268
chr14	54908060	54908364	0.87	8.18E-01	CNIH	-64		
chr6	24581372	24581648	0.95	8.18E-01	KIAA0319	64873	ALDH5A1	86313
chr12	48226014	48226330	1.12	8.18E-01	HDAC7	-12409	VDR	72642
chr17	74733236	74733598	0.93	8.18E-01	MFSD11	-366	SFRS2	-5
chr8	103302197	103302544	1.30	8.18E-01	RRM2B	-51025	UBR5	122124
chr10	135207484	135207757	0.97	8.18E-01	MTG1	0		
chr10	102772644	102772917	0.94	8.18E-01	LZTS2	15816	PDZD7	18098
chr22	24143145	24143456	1.13	8.18E-01	SMARCB1	14151	DERL3	37898
chr16	50840155	50840557	0.90	8.18E-01	CYLD	64395	SALL1	344827
chr19	17966381	17966713	1.07	8.19E-01	RPL18A	-4180		
chr20	33264955	33265296	1.14	8.19E-01	PIGU	-37		
chr11	121293523	121293773	1.21	8.20E-01	SORL1	-29313	SC5DL	130260
chr19	4911745	4912107	0.99	8.20E-01	KDM4B	-57198	UHRF1	2416
chr12	94567448	94567758	1.11	8.20E-01	PLXNC1	25104	TMCC3	476721
chr5	1140436	1140749	1.06	8.20E-01	SLC6A19	-61117	SLC12A7	-28421
chr1	26758704	26758998	0.90	8.21E-01	DHDDS	49		
chr9	131133470	131133754	1.08	8.21E-01	URM1	14		
chr19	44100371	44100783	1.07	8.21E-01	IRGQ	-290	ZNF576	-161
chr1	207997241	207997527	1.22	8.21E-01	CD46	71982	CD34	87299
chr4	108957444	108957754	0.92	8.21E-01	HADH	46659	LEF1	131979
chr6	122792907	122793178	0.92	8.22E-01	SERINC1	-91		

chr2	146169040	146169541	1.02	8.22E-01	ZEB2	-891375		
chr9	19102770	19103246	1.02	8.22E-01	HAUS6	-106		
chr3	187697374	187697744	1.02	8.22E-01	BCL6	-234084	LPP	-233162
chr3	73497905	73498291	1.18	8.22E-01	PDZRN3	175974	FLJ10213	387288
chr8	38801322	38801566	1.23	8.22E-01	HTRA4	-30224	PLEKHA2	42691
chr7	157095098	157095375	1.13	8.23E-01	DNAJB6	-34473	UBE3C	163582
chr17	56017088	56017387	1.14	8.23E-01	MRPS23	-89839	VEZF1	48377
chr12	49449864	49450146	0.89	8.23E-01	MLL2	-898		
chr15	59691667	59692000	0.94	8.24E-01	GCNT3	-212148	MYO1E	-26763
chr2	212290389	212290642	1.00	8.24E-01	CPS1	869190		
chr8	117886934	117887304	1.06	8.24E-01	RAD21	-14		
chr17	27945005	27945266	0.78	8.24E-01	GIT1	-28526	SSH2	311882
chr3	180319924	180320411	0.95	8.24E-01	TTC14	250		
chr6	26597026	26597337	0.95	8.24E-01	ABT1	2		
chr1	90135690	90135964	1.07	8.24E-01	LRRC8D	-151653	LRRC8C	37183
chr11	236791	237097	0.95	8.24E-01	SIRT3	-582	PSMD13	136
chr5	37379220	37379589	0.99	8.24E-01	NUP155	-8208	GDNF	460377
chr6	36725085	36725364	1.02	8.24E-01	CDKN1A	78766	CPNE5	81995
chr7	55625516	55625951	1.00	8.24E-01	VOPP1	14466	LANCL2	192593
chr17	79995074	79995394	0.96	8.24E-01	DCXR	339		
chr16	81856736	81857064	1.15	8.25E-01	PLCG2	43970	SDR42E1	188193
chr2	149402208	149402454	0.83	8.25E-01	EPC2	-229		
chr19	488893	489224	1.09	8.25E-01	SHC2	-28063	MADCAM1	-7431
chr19	36103428	36103769	1.04	8.25E-01	HAUS5	-47		
chr1	224301667	224301922	1.08	8.25E-01	TP53BP2	-268121	DEGS1	-69133
chr15	68132536	68133057	1.06	8.25E-01	PIAS1	-213775	LBXCOR1	14856
chr22	46173483	46173900	1.22	8.25E-01	ATXN10	106005	WNT7B	199316
chr14	68943288	68943538	0.99	8.25E-01	ZFP36L1	316372	RAD51L1	656904
chr12	121790146	121790532	1.02	8.26E-01	ANAPC5	-74		
chr15	40986722	40987158	1.02	8.26E-01	RAD51	-387		
chr12	53473141	53473418	0.76	8.26E-01	SPRYD3	-142		
chr12	58087479	58087986	0.97	8.26E-01	OS9	-153		
chr21	33144760	33145211	0.99	8.26E-01	HUNK	-100642	SFRS15	-40555
chr1	100598421	100598948	1.00	8.26E-01	SASS6	-174	CCDC76	-21
chr6	27156671	27156919	0.89	8.26E-01	PRSS16	-58713	HIST1H2AH	41887
chr4	29056	29530	0.97	8.27E-01	ZNF718	-23934		
chr2	68384561	68385098	0.98	8.27E-01	PNO1	-175	WDR92	-174
chr7	73588388	73588731	0.94	8.27E-01	EIF4H	-146		

chr7	99149602	99149871	0.93	8.27E-01	C7orf38	-21				
chr12	27167284	27167574	0.96	8.27E-01	TM7SF3	-90				
chr16	1661970	1662214	1.04	8.27E-01	CRAMP1L	-2549	IFT140	17		
chr4	38763192	38763568	1.04	8.27E-01	TLR10	21209	KLF3	97590		
chr12	49658725	49659026	1.01	8.27E-01	TUBA1C	11				
chr3	25831414	25831957	1.10	8.27E-01	OXSM	123				
chr1	95320030	95320294	1.10	8.27E-01	SLC44A3	34261	CNN3	72573		
chr18	10490767	10491077	1.05	8.27E-01	NAPG	-34951	APCDD1	36297		
chr6	154996806	154997077	0.98	8.27E-01	CNKSR3	-165189	RBM16	-57570		
chrX	46696178	46696443	0.81	8.27E-01	RP2	-36				
chr19	13227456	13227706	1.04	8.28E-01	NACC1	-1528	TRMT1	-18		
chr14	65453379	65453750	0.94	8.28E-01	FNTB	58				
chr7	98977811	98978095	1.08	8.28E-01	ARPC1B	5624	PDAP1	28339		
chr5	172199417	172199726	0.89	8.29E-01	DUSP1	-1369				
chr17	7590724	7591034	0.93	8.29E-01	TP53	-16				
chr22	29949735	29950033	1.06	8.29E-01	THOC5	-240				
chr8	11271153	11271523	0.97	8.29E-01	BLK	-80183	AMAC1L2	82843		
chr1	11322685	11322995	1.05	8.29E-01	MTOR	-232				
chr20	54967299	54967760	1.06	8.29E-01	AURKA	-179	CSTF1	103		
chr17	7486963	7487686	0.93	8.29E-01	MPDU1	360				
chr3	186733728	186734032	1.11	8.29E-01	ST6GAL1	-5785	ADIPOQ	173417		
chr14	88851765	88852076	1.00	8.29E-01	SPATA7	179				
chr15	49169933	49170412	1.07	8.29E-01	EID1	-117				
chr17	80023592	80023839	1.12	8.30E-01	DUS1L	-36				
chr11	66139788	66140103	1.10	8.30E-01	SLC29A2	-655				
chr9	66458350	66458688	0.94	8.30E-01	FAM75A7	-948909				
chr11	65655799	65656244	0.99	8.30E-01	CCDC85B	-1853	FIBP	-12		
chr15	74782193	74782503	1.14	8.30E-01	ARID3B	-51200	UBL7	-28819		
chr6	27857875	27858309	1.04	8.30E-01	HIST1H2BO	-3111	HIST1H3J	478		
chr4	39367869	39368259	1.04	8.31E-01	RFC1	-69				
chr17	80606247	80606509	1.08	8.31E-01	RAB40B	50220	FOXK2	128784		
chr8	101506830	101507176	0.96	8.31E-01	RNF19A	-191516	ANKRD46	65009		
chr20	30200658	30201001	0.97	8.31E-01	COX4I2	-24861	ID1	7738		
chr22	41842672	41843029	0.92	8.31E-01	TOB2	176				
chr14	64215531	64215893	1.13	8.31E-01	SYNE2	-103971	SGPP1	-20956		
chr4	154388338	154388989	1.09	8.32E-01	TLR2	-216777	KIAA0922	1166		
chr7	156433089	156433581	1.07	8.32E-01	SHH	-828368	RNF32	-66	C7orf13	13
chr15	37392769	37393051	0.99	8.32E-01	MEIS2	-206				

chr19	10041577	10041830	1.04	8.32E-01	OLFM2	5366	PIN1	95705
chr17	40273284	40273548	1.06	8.32E-01	HSPB9	-1340	KAT2A	-34
chr15	83680296	83680741	1.05	8.32E-01	HOMER2	-59046	BTBD1	55587
chr20	32273080	32273404	1.06	8.33E-01	E2F1	968		
chr12	58176241	58176675	1.13	8.34E-01	TSFM	-78		
chr8	48920905	48921352	1.04	8.34E-01	UBE2V2	134		
chr12	56511759	56512107	1.06	8.35E-01	ZC3H10	-97		
chr15	44955720	44956164	1.11	8.35E-01	SPG11	-66		
chr15	45329331	45329579	0.88	8.35E-01	SORD	14114	DUOX2	76904
chr20	5672605	5672943	1.10	8.35E-01	CHGB	-219200	RP5-1022P6.2	-81102
chr9	36765852	36766189	1.15	8.35E-01	MELK	193116	PAX5	268455
chr1	26324557	26325027	1.07	8.35E-01	PAFAH2	-144		
chr1	46502794	46503039	0.92	8.35E-01	PIK3R3	95463	MAST2	233632
chr8	11659772	11660177	1.05	8.36E-01	FDFT1	-215		
chr5	38453694	38454004	1.13	8.36E-01	LIFR	141658	EGFLAM	195316
chr14	51256530	51256840	0.95	8.36E-01	SAV1	-121662	NIN	41154
chr15	75628218	75628528	0.90	8.36E-01	COMMD4	-1		
chr16	10479643	10479913	0.95	8.36E-01	GRIN2A	-203515	ATF7IP2	-42947
chr17	8286373	8286721	1.00	8.37E-01	RPL26	18		
chr6	44354985	44355416	0.95	8.38E-01	CDC5L	-101		
chr15	64385290	64385600	1.01	8.38E-01	SNX1	-2732		
chr17	4890752	4891063	0.87	8.38E-01	CAMTA2	23		
chr14	23357224	23357534	0.81	8.38E-01	REM2	4947	RBM23	31017
chr5	137371384	137371933	1.05	8.38E-01	FAM13B	-2857		
chr10	121129025	121129389	0.96	8.38E-01	GRK5	162010	RGS10	173015
chr19	1876016	1876307	0.98	8.38E-01	KLF16	-12598	FAM108A1	9356
chr12	76034808	76035118	0.94	8.38E-01	KRR1	-129545	PHLDA1	390593
chr19	35696176	35696541	1.05	8.38E-01	FAM187B	23269	FXYD5	50732
chr7	72298611	72298982	0.94	8.38E-01	TYW1B	16		
chr5	108745687	108746096	0.99	8.38E-01	PJA2	-217		
chr10	65028868	65029246	1.11	8.38E-01	NRBF2	136050	JMJD1C	196665
chr6	26033610	26034033	1.12	8.39E-01	HIST1H3B	-1534	HIST1H2AB	-26
chr5	96519208	96519724	1.08	8.39E-01	RIOK2	-461		
chr16	68344736	68345161	0.88	8.39E-01	PRMT7	4		
chr18	29522874	29523491	0.85	8.39E-01	KIAA1012	-92		
chr9	131084708	131085018	1.04	8.39E-01	TRUB2	-166	COQ4	72
chr1	200379182	200379582	0.92	8.39E-01	ZNF281	-216		
chr7	98923343	98923590	1.02	8.40E-01	ARPC1A	-43		

chr12	102455567	102455917	1.00	8.40E-01	CCDC53	157		
chr1	45956699	45957214	1.07	8.40E-01	TESK2	-117		
chr1	114354807	114355239	0.98	8.40E-01	RSBN1	47		
chr13	99097622	99097925	1.19	8.40E-01	RNF113B	-268253	STK24	131622
chr3	140660302	140660691	1.09	8.40E-01	SLC25A36	-165		
chr6	26103988	26104775	0.94	8.40E-01	HIST1H4C	206		
chr2	198364534	198365011	0.79	8.40E-01	HSPD1	-133	HSPE1	52
chr7	128858447	128858720	0.98	8.40E-01	AHCYL2	-6280	SMO	29871
chr8	37739423	37739778	1.01	8.40E-01	BRF2	-32190	RAB11FIP1	17402
chr15	73925766	73926027	0.98	8.41E-01	NPTN	-144		
chr6	26158073	26158628	1.11	8.41E-01	HIST1H2BD	2		
chr12	57853282	57853637	1.00	8.41E-01	GLI1	-458		
chr5	21058273	21058720	1.28	8.41E-01	GUSBL2	-401092		
chr9	103115090	103115538	1.09	8.41E-01	TEX10	-55		
chr11	65149836	65150163	1.00	8.41E-01	FRMD8	-4041	SLC25A45	142
chr16	66558724	66559047	1.17	8.41E-01	TK2	25429	BEAN	97646
chr17	29876964	29877274	1.17	8.42E-01	RAB11FIP4	158477	C17orf79	309207
chr12	54121241	54121673	0.98	8.42E-01	CALCOCO1	-150		
chr10	97134449	97134703	0.91	8.42E-01	PDLIM1	-83795	SORBS1	66361
chr15	52975800	52976208	0.98	8.42E-01	ARPP19	-114791	ONECUT1	106205
chr1	76251663	76252150	0.93	8.42E-01	RABGGTB	21		
chr15	44719304	44719676	1.00	8.43E-01	CTDSPL2	-89		
chr3	156272715	156273202	1.09	8.43E-01	SSR3	-24		
chr9	71614571	71614905	0.94	8.43E-01	PRKACG	14301	PIP5K1B	294122
chr1	29557316	29557560	0.96	8.43E-01	MECR	16		
chr8	30515697	30515937	1.00	8.43E-01	GTF2E2	-79		
chr6	26189154	26189489	1.16	8.43E-01	HIST1H4D	-18		
chr8	11141779	11142175	0.99	8.43E-01	MTMR9	-23		
chr19	13957397	13957642	0.97	8.44E-01	NANOS3	-30430	ZSWIM4	51246
chr6	30584892	30585495	0.90	8.44E-01	MRPS18B	-292	PPP1R10	-174
chr14	70233636	70233912	1.10	8.44E-01	SFRS5	-60		
chr19	6504843	6505100	1.37	8.44E-01	TUBB4	-2642		
chr11	14913783	14914065	0.84	8.44E-01	CYP2R1	-173		
chr11	64655416	64655662	0.92	8.44E-01	PPP2R5B	-36641	EHD1	-9348
chr16	19533305	19533560	0.91	8.44E-01	CP110	-1746	GDE1	17
chr17	33469081	33469436	1.08	8.44E-01	NLE1	63		
chr2	68589182	68589591	1.03	8.44E-01	PLEK	-2935		
chr3	49377939	49378387	1.05	8.44E-01	USP4	-627		

chr9	2158301	2158649	1.05	8.45E-01	VLDLR	-463318	SMARCA2	143133
chr2	232092087	232092331	1.05	8.45E-01	B3GNT7	-168126	ARMC9	28867
chr2	10518475	10518785	1.02	8.45E-01	ODC1	69823	HPCAL1	75590
chr20	57607308	57607829	1.14	8.45E-01	ATP5E	-147		
chr7	30028072	30028327	1.04	8.46E-01	SCRN1	1705	WIPF3	153859
chr2	61244240	61244550	1.01	8.46E-01	PEX13	-417	PUS10	970
chr6	6584120	6584396	1.12	8.46E-01	LY86	-4676		
chr9	100174093	100174337	1.19	8.46E-01	TDRD7	-87		
chr16	57333716	57334060	1.09	8.46E-01	CCL22	-58830	PLLP	-15317
chr12	50236737	50237016	0.88	8.47E-01	BCDIN3D	35		
chr1	111670939	111671298	1.05	8.48E-01	C1orf103	-164553	CEPT1	-11130
chr7	126341136	126341420	1.19	8.49E-01	GRM8	542291		
chr2	28974188	28974582	1.06	8.49E-01	PPP1CB	-229		
chr7	135662027	135662477	1.25	8.49E-01	MTPN	-48		
chr1	161735841	161736339	1.12	8.49E-01	ATF6	6		
chr17	42610912	42611240	1.11	8.49E-01	GPATCH8	-30274	FZD2	-23849
chr2	233965299	233965775	1.05	8.49E-01	ATG16L1	-194680	INPP5D	40501
chr15	93352361	93352940	1.14	8.50E-01	FAM174B	-153620	CHD2	-90900
chr22	21996323	21996709	1.05	8.50E-01	SDF2L1	-26		
chr7	74071486	74071880	0.78	8.50E-01	GTF2I	-347		
chr19	14191862	14192396	1.03	8.51E-01	PALM3	-22158	SAMD1	9103
chr11	66085478	66085714	0.90	8.51E-01	CD248	-1081		
chr17	80452325	80452855	1.12	8.51E-01	FOXK2	-25004	NARF	36050
chr2	242041607	242041930	1.06	8.51E-01	PASK	47109	SNED1	103514
chr22	41042299	41042540	0.84	8.51E-01	MCHR1	-32762	MKL1	-9730
chr5	176778569	176778925	0.97	8.51E-01	LMAN2	138		
chr6	155502708	155502979	1.19	8.51E-01	CLDN20	-82303	TIAM2	91421
chr8	37489191	37489427	1.01	8.51E-01	ZNF703	-63992	FKSG2	743312
chr13	52027007	52027293	0.98	8.52E-01	INTS6	125		
chr13	29233023	29233308	1.11	8.52E-01	POMP	-75		
chr16	30621690	30621960	0.98	8.52E-01	ZNF689	-143		
chr2	203776172	203776479	1.18	8.53E-01	ALS2CR8	-652	WDR12	623
chr12	53894496	53894829	0.84	8.53E-01	MAP3K12	-1392	TARBP2	-714
chr2	172864642	172864997	1.08	8.53E-01	MAP1D	16		
chr6	42185477	42185778	1.01	8.53E-01	MRPS10	5		
chr8	56757093	56757339	0.87	8.53E-01	TMEM68	-71331	LYN	-35170
chr14	53019285	53019565	0.94	8.53E-01	GPR137C	-441	TXNDC16	-124
chr7	129251339	129251662	0.83	8.53E-01	NRF1	-54		

chr22	24823335	24823633	1.01	8.53E-01	ADORA2A	-4335	ADORA2A	-46
chr20	46041446	46041783	1.08	8.53E-01	NCOA3	-89042	ZMYND8	-56141
chr14	45430943	45431694	1.04	8.54E-01	KLHL28	-140	FAM179B	-97
chr19	17377857	17378312	0.91	8.55E-01	USHBP1	-2541	C19orf62	-147
chr1	220445742	220446115	1.05	8.55E-01	RAB3GAP2	-86		
chr16	14580673	14581048	0.94	8.55E-01	PARN	143263	MKL2	415665
chr14	90422039	90422371	0.97	8.55E-01	C14orf143	-1116	TDP1	-41
chr1	27648418	27648900	0.93	8.56E-01	TMEM222	23		
chr6	28109432	28109728	1.16	8.56E-01	ZNF192	-136		
chr3	111851928	111852262	1.05	8.56E-01	GCET2	22		
chr1	108319956	108320266	0.88	8.56E-01	VAV3	187434	NTNG1	637482
chr1	192578155	192578494	1.11	8.56E-01	RGS13	-26957	RGS1	33468
chr20	35724322	35724702	1.23	8.56E-01	RBL1	-102		
chr12	53845524	53845900	1.04	8.56E-01	PCBP2	-174		
chr6	45645152	45645538	1.10	8.56E-01	SUPT3H	-299675	CLIC5	402740
chr17	42092208	42092542	1.08	8.57E-01	TMEM101	-30		
chr17	73098519	73098788	1.03	8.57E-01	ARMC7	-7428	SLC16A5	14599
chr5	139012905	139013169	1.02	8.57E-01	CXXC5	-15264	UBE2D2	72286
chr10	1531343	1531619	1.06	8.58E-01	IDI1	-436420	ADARB2	248237
chr9	33264850	33265117	0.91	8.58E-01	BAG1	-223	CHMP5	107
chr2	198380525	198380979	0.94	8.58E-01	MOBKL3	-19		
chr17	66243726	66244133	0.96	8.59E-01	AMZ2	-215		
chr5	180687880	180688256	0.96	8.59E-01	TRIM52	51		
chr17	57642682	57643041	1.13	8.59E-01	DHX40	-24		
chr16	1763961	1764252	0.99	8.59E-01	MAPK8IP3	7886	NME3	57603
chr11	82782858	82783254	1.03	8.59E-01	RAB30	-172		
chr6	45345655	45345967	1.04	8.59E-01	SUPT3H	-141		
chr12	112825210	112825455	1.01	8.59E-01	C12orf51	-81295	RPL6	22110
chr4	597980	598256	1.17	8.59E-01	ZNF721	-104676	PDE6B	-21245
chr11	8008469	8008872	0.88	8.60E-01	EIF3F	226		
chr15	40882018	40882317	0.93	8.60E-01	CASC5	-4279		
chr18	54744928	54745377	1.12	8.60E-01	ST8SIA3	-274568	WDR7	426537
chr3	160116811	160117166	1.11	8.60E-01	SMC4	-441	IFT80	331
chr10	104180749	104181059	0.93	8.60E-01	PSD	-2003		
chr12	112450934	112451307	0.97	8.60E-01	TMEM116	-186	ERP29	-31
chr2	231280764	231281436	1.12	8.60E-01	SP100	229		
chr19	4400578	4400921	1.01	8.60E-01	CHAF1A	-1910	SH3GL1	-279
chr5	143567390	143567652	0.90	8.60E-01	KCTD16	17084		

chr5	90678974	90679299	1.03	8.61E-01	ARRDC3	12		
chr7	23636839	23637179	1.11	8.62E-01	CCDC126	11		
chr7	101625269	101625635	1.03	8.62E-01	SH2B2	-302953	CUX1	164570
chr11	62649035	62649304	1.03	8.63E-01	SLC3A2	25652	CHRM1	39842
chr8	37738373	37738633	1.08	8.63E-01	BRF2	-31092	RAB11FIP1	18500
chr3	137906015	137906437	1.22	8.64E-01	ARMC8	78		
chr1	110880934	110881979	0.87	8.64E-01	RBM15	-488		
chr1	35658522	35659037	0.98	8.64E-01	SFPQ	-37		
chr2	27805707	27806145	1.14	8.64E-01	ZNF512	33		
chr12	57856319	57856829	0.90	8.64E-01	GLI1	2656	ARHGAP9	17059
chr3	186734258	186734611	0.94	8.65E-01	ST6GAL1	-5230	ADIPOQ	173972
chr7	105662868	105663337	1.15	8.65E-01	FLJ23834	59446	SYPL1	89954
chr6	136610891	136611197	1.05	8.65E-01	BCLAF1	-55		
chr19	2085333	2085661	1.06	8.65E-01	MKNK2	-34254	MOBKL2A	10772
chr19	18209608	18209979	1.12	8.65E-01	PIK3R2	-54222	MAST3	1191
chr5	68513544	68513905	0.96	8.65E-01	MRPS36	152		
chr13	27803814	27804058	1.17	8.65E-01	USP12	-57907	RPL21	-21756
chr9	140163809	140164192	0.93	8.65E-01	C9orf167	-8279	COBRA1	14242
chrX	149106685	149107167	1.21	8.65E-01	MAMLD1	-424760	HSFX1	-251153
chr20	62496191	62496699	0.88	8.66E-01	TPD52L2	-145		
chr9	32550954	32551428	1.02	8.66E-01	DDX58	-24869	TOPORS	1410
chr3	130612659	130612993	1.11	8.66E-01	ATP2C1	-608		
chr6	35438321	35438729	1.36	8.66E-01	RPL10A	2347	TEAD3	26336
chr6	170863231	170863520	1.04	8.66E-01	PSMB1	-959	TBP	-95
chr2	55496207	55496553	1.07	8.66E-01	MTIF2	4		
chr5	167755785	167756223	1.09	8.67E-01	RARS	-157459	WWC1	36939
chr13	111365275	111365833	1.05	8.67E-01	ING1	-1805		
chr2	219744697	219745032	1.13	8.67E-01	WNT10A	-390		
chr1	149859731	149860230	1.14	8.67E-01	HIST2H2BE	-1749	HIST2H2AB	-515
chr6	15151424	15151750	1.01	8.68E-01	JARID2	-94940		
chr5	171538317	171538564	1.11	8.68E-01	FBXW11	-104564	STK10	76905
chr14	35451498	35451853	0.95	8.68E-01	SRP54	-428		
chr1	36863431	36863685	1.11	8.68E-01	LSM10	-65		
chr15	78730526	78730848	0.89	8.68E-01	IREB2	169		
chr11	43665604	43665914	1.13	8.69E-01	HSD17B12	-36384	TTC17	285268
chr4	2698158	2698553	1.12	8.69E-01	TNIP2	59747	RNF4	227549
chr2	214018416	214018714	0.97	8.69E-01	IKZF2	-3507		
chr11	59040929	59041207	1.05	8.69E-01	OR5AN1	-90864	MPEG1	-60574

chr17	8042134	8042458	1.03	8.69E-01	HES7	-14894	PER1	13457
chr15	90627033	90627434	0.92	8.69E-01	IDH2	18474	ZNF710	82482
chr17	65713756	65714076	1.08	8.69E-01	NOL11	-145		
chr18	10520140	10520382	1.11	8.70E-01	NAPG	-5612	APCDD1	65636
chr1	111991824	111992085	1.00	8.70E-01	WDR77	-125	ATP5F1	212
chr1	46153668	46153931	0.95	8.70E-01	GPBP1L1	-1498	TMEM69	-47
chr15	64648331	64648789	1.08	8.71E-01	CSNK1G1	-118		
chr9	34989090	34989446	0.94	8.71E-01	DNAJB5	-999		
chr3	157827571	157828009	1.10	8.71E-01	SHOX2	-3838	RSRC1	-102
chr3	31267870	31268180	0.84	8.72E-01	GADL1	-331872	STT3B	-306466
chr3	4534753	4535208	1.06	8.72E-01	ITPR1	-53		
chr19	6862750	6863071	1.03	8.72E-01	EMR1	-24671	VAV1	90189
chr1	204485242	204485651	1.08	8.73E-01	MDM4	-64		
chr6	43484530	43484893	0.95	8.73E-01	POLR1C	-79	YIPF3	-10
chr2	43358582	43358949	1.11	8.73E-01	HAAO	-339015	ZFP36L2	94979
chr2	27906397	27906652	1.02	8.73E-01	MRPL33	-88059	SUPT7L	-20076
chr17	8023557	8023828	1.10	8.74E-01	ALOXE3	-1833		
chr1	226111765	226112099	1.00	8.74E-01	PYCR2	33		
chr14	69864994	69865263	1.17	8.74E-01	SLC39A9	-278	ERH	-108
chr14	90722742	90723000	1.10	8.74E-01	PSMC1	-23		
chr17	42767021	42767373	0.99	8.74E-01	DBF4B	-18779	FZD2	132272
chr2	198063571	198063920	1.00	8.74E-01	ANKRD44	-984		
chr9	130477920	130478369	1.09	8.75E-01	TTC16	-213		
chr19	18365797	18366248	0.86	8.75E-01	PDE4C	-7013	KIAA1683	19296
chr14	21560651	21560971	1.04	8.75E-01	C14orf176	-6285	FLJ10357	22284
chr1	205090913	205091648	1.04	8.75E-01	RBBP5	-150		
chr19	35821335	35821732	1.23	8.76E-01	FFAR1	-20911	CD22	1455
chr4	81532021	81532353	0.99	8.76E-01	BMP3	-419932	FGF5	344445
chr4	148605286	148605591	1.19	8.76E-01	PRMT10	-159		
chr17	1123655	1123947	1.07	8.76E-01	TUSC5	-59156	ABR	-40670
chr17	47269245	47269532	0.97	8.76E-01	GNGT2	17354	B4GALNT2	59060
chr2	68954195	68954439	1.11	8.77E-01	ARHGAP25	-7651	PROKR1	81523
chr7	151855898	151856224	1.14	8.77E-01	GALNT11	133283	MLL3	277029
chr5	81046742	81047081	0.99	8.77E-01	SSBP2	160		
chr9	116342864	116343329	0.84	8.77E-01	ZNF618	-295465	RGS3	136086
chr1	167597275	167597647	1.06	8.77E-01	MPZL1	-93726	CREG1	-74405
chr11	62608990	62609304	1.06	8.77E-01	WDR74	-1519		
chr5	14287265	14287637	1.02	8.78E-01	TRIO	143622	ANKH	584436

chr10	11720176	11720451	0.96	8.78E-01	USP6NL	-146040	ECHDC3	-64042
chr16	71496058	71496306	0.92	8.78E-01	ZNF23	-65		
chr18	44711052	44711362	0.88	8.78E-01	IER3IP1	-8462	CORL2	64347
chr16	28956938	28957394	1.05	8.79E-01	NFATC2IP	-5152	CD19	13906
chr6	53275270	53275666	1.15	8.79E-01	ELOVL5	-61526	GCLC	134363
chr6	27446442	27446791	0.91	8.79E-01	ZNF184	-5720	HIST1H2BL	329092
chr1	155034007	155034327	0.97	8.79E-01	EFNA3	-17181	ADAM15	10405
chr2	220109951	220110368	1.07	8.79E-01	STK16	-32	GLB1L	-29
chr19	41283566	41284107	1.02	8.79E-01	RAB4B	-334		
chr17	73008429	73008941	1.01	8.79E-01	ICT1	-95		
chr21	38639595	38640047	1.08	8.79E-01	DSCR3	12		
chr15	79888439	79888793	1.28	8.79E-01	KIAA1024	163758	MTHFS	300754
chr4	83955917	83956339	1.06	8.79E-01	COPS4	-111		
chr7	129691050	129691360	1.15	8.79E-01	ZC3HC1	28		
chr16	18812602	18813468	0.95	8.80E-01	ARL6IP1	-178		
chr12	111742399	111742709	1.14	8.80E-01	SH2B3	-101198	CUX2	270725
chr7	66309842	66310115	1.02	8.80E-01	C7orf42	-76224	RABGEF1	104996
chr3	16475144	16475493	1.11	8.81E-01	RFTN1	79903	OXNAD1	168605
chr3	10289790	10290204	0.99	8.81E-01	TATDN2	-180		
chr6	155054326	155054667	0.81	8.81E-01	RBM16	-15		
chr12	111136103	111136388	1.16	8.81E-01	HVCN1	-9300	PPP1CC	44511
chr11	88070832	88071116	1.02	8.81E-01	CTSC	-33		
chr20	55973283	55973779	1.12	8.81E-01	RBM38	7068	HMGB1L1	90552
chr21	45663858	45664185	1.18	8.81E-01	ICOSLG	-3188		
chr3	133196386	133196683	1.06	8.81E-01	CDV3	-95899	BFSP2	77745
chr3	143247877	143248175	1.11	8.81E-01	SLC9A9	319320	CHST2	409358
chr3	156878360	156878776	1.07	8.81E-01	CCNL1	-86		
chr9	91925507	91926232	1.03	8.81E-01	CKS2	-243		
chr6	27858333	27858672	1.13	8.81E-01	HIST1H2BO	-2700	HIST1H3J	67
chr5	156913320	156913631	0.95	8.82E-01	NIPAL4	26449	ADAM19	89292
chr13	67568414	67568658	0.97	8.82E-01	PCDH9	235932		
chr19	50871815	50872155	0.96	8.82E-01	NAPSA	-3054		
chr3	187802985	187803493	1.02	8.82E-01	BCL6	-339764	LPP	-127482
chr15	91445532	91445894	1.06	8.82E-01	MAN2A2	-1707		
chr11	74204184	74204469	1.02	8.82E-01	MGC12965	-158	LIPT2	428
chr15	77228022	77228350	1.38	8.82E-01	PSTPIP1	-59279	RCN2	4224
chr15	64679851	64680161	0.99	8.82E-01	TRIP4	-14		
chr12	110434063	110434373	1.14	8.82E-01	GIT2	-24		

chr7	142506514	142506828	0.96	8.82E-01	EPHB6	-46121	TCRBC2	12304
chr1	203294952	203295228	1.01	8.82E-01	BTG2	20426	FMOD	25199
chr10	6092810	6093289	1.07	8.82E-01	IL15RA	-73513	IL2RA	11222
chr2	89131278	89131648	0.93	8.82E-01	O1/O11 and JK2	-759099	RPIA	140287
chr11	9595035	9595350	1.05	8.83E-01	WEE1	-35		
chr14	90863192	90863510	0.84	8.83E-01	CALM1	-22		
chr17	40829042	40829368	0.93	8.83E-01	PLEKHH3	-157		
chr5	149829524	149829834	0.97	8.83E-01	RPS14	-360		
chr8	95487271	95487520	1.08	8.83E-01	RAD54B	-86		
chr19	8082730	8083006	1.25	8.83E-01	CCL25	-35016	ELAVL1	-12339
chr2	213727874	213728184	1.10	8.83E-01	ERBB4	-324677	IKZF2	287029
chr6	144537524	144537834	1.00	8.83E-01	UTRN	-75194	STX11	66025
chr4	25161955	25162633	1.01	8.83E-01	SEPSECS	-90		
chr5	154320492	154320835	1.11	8.83E-01	GEMIN5	-2888	MRPL22	31
chr3	53925878	53926263	1.01	8.83E-01	SELK	-82		
chr16	22309570	22309998	1.11	8.83E-01	POLR3E	1043	CDR2	76154
chr3	122101779	122102310	1.01	8.83E-01	FAM162A	-978	CCDC58	29
chr12	50056780	50057102	0.95	8.83E-01	PRPF40B	32613	FMNL3	44256
chr11	121163084	121163516	1.04	8.83E-01	SC5DL	-88		
chr3	150125914	150126182	0.98	8.83E-01	TSC22D2	-740		
chr1	203268354	203268682	0.96	8.83E-01	CHIT1	-69658	BTG2	-6146
chr11	57479487	57480170	1.18	8.83E-01	TMX2	-213	MED19	-156
chr13	111837093	111837441	1.05	8.83E-01	C13orf16	-135748	ARHGEF7	69643
chr12	58146159	58146573	1.03	8.84E-01	MARCH9	-2534	CDK4	-202
chr15	90605215	90605589	1.08	8.84E-01	IDH2	40306	ZNF710	60650
chr17	8125514	8125768	1.06	8.84E-01	AURKB	-11758	C17orf68	25772
chr1	161015621	161015971	1.02	8.84E-01	USF1	-39		
chr17	57287185	57287495	1.19	8.84E-01	C17orf71	-31		
chr10	49879716	49880034	0.93	8.84E-01	ARHGAP22	-66737	WDFY4	-13643
chr14	105957131	105957704	0.99	8.84E-01	TMEM121	-35535	CRIP1	4161
chr19	1068747	1069417	0.99	8.84E-01	HMHA1	1908	POLR2E	26309
chr3	142926164	142926538	1.19	8.84E-01	CHST2	87683	SLC9A9	640995
chr15	40401123	40401422	1.17	8.85E-01	BMF	-198		
chr10	363284	363559	1.00	8.85E-01	ZMYND11	182998	DIP2C	372186
chr19	16251937	16252247	1.02	8.85E-01	HSH2D	7254	CIB3	32194
chr21	45574995	45575378	0.99	8.85E-01	C21orf33	21693	ICOSLG	85647
chr4	4945716	4946099	0.92	8.85E-01	CYTL1	75289	MSX1	84516
chr6	37070237	37070880	0.91	8.85E-01	PIM1	-67363	FGD2	97136

chr8	67025410	67025790	0.92	8.85E-01	TRIM55	-13678	DNAJC5B	91809		
chr2	73461246	73461670	0.99	8.86E-01	C2orf7	-1102	CCT7	53		
chr2	95831333	95831863	0.95	8.86E-01	ZNF2	415				
chr3	184529682	184530059	0.95	8.86E-01	VPS8	-60				
chr7	100808739	100809129	0.92	8.86E-01	VGF	-82				
chr12	54411502	54412021	1.00	8.86E-01	HOXC6	-10432	HOXC8	8872		
chr16	14726467	14726908	0.96	8.86E-01	PARN	-2564	BFAR	20		
chr17	5389706	5390131	1.06	8.86E-01	DERL2	-425	MIS12	-328		
chr5	14664374	14664835	0.93	8.86E-01	ANKH	207282	TRIO	520776		
chr12	32831980	32832224	1.03	8.86E-01	DNM1L	-35				
chr16	2073189	2073458	0.90	8.86E-01	SLC9A3R2	-3564				
chr8	125649776	125650092	1.00	8.86E-01	MTSS1	90796	NDUFB9	98591		
chr11	65756501	65756745	1.01	8.86E-01	BANF1	-12927	SART1	27463		
chr1	111762472	111762777	1.24	8.86E-01	DRAM2	-79787	CHI3L2	-7656		
chr2	47951380	47951796	0.94	8.86E-01	KCNK12	-154118	MSH6	-58633		
chr1	74663971	74664281	0.86	8.87E-01	LRRIQ3	-255	TNNI3K	200		
chr12	59989528	59989804	0.83	8.87E-01	LRIG3	-675404	SLC16A7	-93460		
chr12	58165771	58166040	1.03	8.87E-01	CYP27B1	-4930	FAM119B	-477	METTL1	8
chr9	135282152	135282388	1.01	8.87E-01	TTF1	-49				
chr13	46626795	46627117	0.94	8.87E-01	ZC3H13	-62				
chr17	40730396	40730829	1.10	8.87E-01	PSMC3IP	-866				
chr19	10978652	10978910	0.92	8.87E-01	CARM1	-3472				
chr2	63815455	63816190	0.92	8.87E-01	MDH1	-299				
chr3	183273293	183273701	1.05	8.87E-01	KLHL6	2				
chr7	140396649	140396893	0.90	8.87E-01	NDUFB2	290				
chr10	121632376	121632806	0.87	8.87E-01	SEC23IP	-19632	INPP5F	146982		
chr17	8090037	8090412	1.14	8.87E-01	TMEM107	-10511	C17orf59	3339		
chr17	27053947	27054261	0.99	8.87E-01	NEK8	-1728	TLCD1	-874		
chr17	45214444	45214754	0.84	8.87E-01	RPRML	-157985	CDC27	52066		
chr3	133931813	133932092	1.06	8.87E-01	SLCO2A1	-183033	RYK	37633		
chr19	17622871	17623225	1.09	8.88E-01	PGLS	616				
chr14	55240750	55241036	0.83	8.88E-01	GCH1	128649	SAMD4A	206256		
chr7	97501462	97501897	0.88	8.88E-01	ASNS	174				
chr5	142972207	142972570	0.97	8.88E-01	NR3C1	-188344	YIPF5	577889		
chr16	30064003	30064540	0.96	8.89E-01	FAM57B	-22086	ALDOA	-11328		
chr11	64619257	64619584	1.13	8.89E-01	CDC42BPG	-7380	EHD1	26770		
chr10	97989322	97989632	0.89	8.89E-01	BLNK	41856	ZNF518A	100005		
chr17	10600705	10601024	0.95	8.89E-01	C17orf48	-62	SCO1	20		

chr12	46122527	46122932	0.84	8.89E-01	ARID2	-890		
chr2	145461493	145461803	1.00	8.89E-01	ZEB2	-183732		
chr4	26994294	26994538	0.94	8.89E-01	STIM2	132052		
chr17	56405584	56405894	1.00	8.90E-01	BZRAP1	413		
chr2	85154015	85154392	1.03	8.90E-01	KCMF1	-44027	TMSB10	21441
chr15	75660797	75661160	1.12	8.90E-01	MAN2C1	-38		
chr6	139696390	139696754	0.86	8.90E-01	CITED2	-787		
chr16	75182176	75182438	1.09	8.90E-01	ZFP1	-114		
chr13	37573329	37573613	1.10	8.90E-01	EXOSC8	-1207	ALG5	33
chr12	54762470	54762746	0.96	8.91E-01	GPR84	-4350		
chr1	39338938	39339495	0.96	8.91E-01	MYCBP	-167		
chr17	2304017	2304598	0.97	8.91E-01	MNT	-50		
chr13	41579019	41579289	1.32	8.91E-01	ELF1	14354	SLC25A15	215607
chr14	92572985	92573282	1.02	8.91E-01	ATXN3	-169		
chr19	15529749	15530250	1.04	8.91E-01	AKAP8L	-167		
chr2	97405707	97406037	1.00	8.91E-01	LMAN2L	-59		
chr2	197076046	197076367	1.01	8.91E-01	STK17B	-39871	HECW2	381128
chr3	194785073	194785439	1.12	8.91E-01	LSG1	-392050	C3orf21	206639
chr15	68569885	68570195	0.97	8.91E-01	FEM1B	-101		
chr6	24359941	24360464	0.94	8.91E-01	DCDC2	-1923		
chr5	53606243	53606665	1.10	8.92E-01	ARL15	-51		
chr10	13413972	13414230	0.89	8.92E-01	SEPHS1	-23821	BEND7	130875
chr13	30945779	30946230	1.04	8.92E-01	KATNAL1	-64842	HMGB1	94076
chr16	57220115	57220373	0.84	8.92E-01	FAM192A	-268	RSPRY1	3
chr17	46969962	46970370	1.09	8.92E-01	ATP5G1	18		
chr19	49474182	49474631	0.93	8.92E-01	FTL	5841	GYS1	22203
chr1	24306852	24307118	1.09	8.92E-01	SFRS13A	-164		
chr10	95462272	95462540	0.95	8.92E-01	C10orf4	-77		
chr5	72143781	72144032	0.95	8.92E-01	TNPO1	-23		
chr3	183695274	183695616	1.34	8.92E-01	PARL	-92752	ABCC5	40282
chr5	108804541	108804807	1.05	8.92E-01	MAN2A1	-220482	PJA2	-58999
chr12	7053803	7054113	1.02	8.92E-01	PTPN6	-1782		
chr10	104556165	104556425	0.89	8.92E-01	CYP17A1	40995	C10orf26	52568
chr6	25006815	25007147	1.08	8.92E-01	FAM65B	-95786	DKFZp686H12134	131070
chr11	77790829	77791139	1.16	8.93E-01	NDUFC2	281		
chr8	145634603	145634925	1.03	8.93E-01	CPSF1	-31		
chr4	76598576	76598963	1.05	8.93E-01	G3BP2	-103		
chr6	68160591	68160928	0.99	8.93E-01				

chr1	25071400	25071695	0.82	8.93E-01	CLIC4	-212		
chr7	148663912	148664148	0.87	8.93E-01	EZH2	-82616	PDIA4	61752
chr15	40453015	40453266	1.20	8.93E-01	BUB1B	-69		
chr12	54674317	54674840	0.87	8.94E-01	HNRNPA1	91		
chr12	72079712	72080151	0.88	8.94E-01	TMEM19	54		
chr16	57496372	57496714	1.03	8.94E-01	POLR2C	-8		
chr5	180649246	180649636	1.10	8.94E-01	TRIM41	-865		
chr16	67875749	67876034	0.96	8.94E-01	NUTF2	-4927	THAP11	-321
chr6	33385707	33386415	0.97	8.94E-01	SYNGAP1	-1786	CUTA	4
chr1	43637865	43638134	0.88	8.94E-01	EBNA1BP2	241		
chr17	48474678	48475077	1.06	8.94E-01	LRRC59	-46		
chr19	52531568	52532045	1.15	8.94E-01	ZNF614	-127		
chr2	162164741	162165054	1.08	8.94E-01	PSMD14	112		
chr1	33592744	33593106	1.17	8.94E-01	ADC	46211	TRIM62	54746
chr12	14320042	14320318	1.01	8.94E-01	ATF7IP	-198431	GRIN2B	-187158
chr19	16699224	16699538	1.08	8.94E-01	SLC35E1	-16188	MED26	39634
chr2	113484331	113484650	0.97	8.94E-01	NT5DC4	5428	IL1A	58480
chr2	219433102	219433610	1.05	8.94E-01	RQCD1	-322	USP37	-272
chr6	27100207	27100973	1.09	8.94E-01	HIST1H2AG	-227	HIST1H2BJ	-15
chr1	45285243	45285982	1.08	8.94E-01	BTBD19	11459	PTCH2	23003
chr16	699193	699504	0.94	8.94E-01	RHOT2	-18784	FAM195A	7500
chr20	32307841	32308235	1.07	8.95E-01	PXMP4	98		
chr1	222886554	222886811	1.01	8.95E-01	AIDA	-819	C1orf58	777
chr4	169737989	169738266	0.94	8.96E-01	CBR4	193294	PALLD	319911
chr16	75599032	75599278	1.14	8.97E-01	GABARAPL2	-1094		
chr4	26861973	26862505	1.01	8.97E-01	STIM2	-125		
chr17	45068974	45069322	0.92	8.97E-01	RPRML	-12534	CDC27	197517
chr2	220142393	220142869	0.97	8.97E-01	DNAJB2	-1409		
chr11	65686659	65687054	1.16	8.97E-01	DRAP1	129		
chr12	2999774	3000060	0.78	8.97E-01	TULP3	-116		
chr9	127177657	127177991	1.08	8.97E-01	PSMB7	-103		
chr9	130879948	130880222	0.94	8.97E-01	PTGES2	10389	SLC25A25	49606
chr7	44529959	44530257	0.87	8.97E-01	CAMK2B	-164878	NPC1L1	50806
chr7	73686881	73687207	0.97	8.98E-01	RFC2	-18306	CLIP2	-16761
chr9	74526104	74526675	0.94	8.98E-01	FAM108B1	-242		
chr7	149321735	149322048	0.91	8.98E-01	ZNF746	-126994	ZNF467	148403
chr9	114660181	114660457	1.19	8.98E-01	UGCG	1113	SUSD1	277237
chr5	176852787	176853090	1.05	8.98E-01	GRK6	-748		

chr17	79935170	79935614	1.09	8.98E-01	ASPSCR1	-34		
chr10	74056950	74057408	1.08	8.98E-01	DDIT4	23502	DNAJB12	57728
chr8	22932195	22932447	0.91	8.99E-01	TNFRSF10C	-28113	TNFRSF10B	-5621
chr8	42150225	42150583	1.06	8.99E-01	POLB	-45626	IKBKB	21575
chr16	69458351	69458675	1.04	8.99E-01	CYB5B	15		
chr3	47017870	47018305	1.00	9.00E-01	NBEAL2	-3085		
chr5	150826996	150827322	0.95	9.00E-01	SLC36A1	-4		
chr12	6832891	6833337	0.99	9.00E-01	COPS7A	-66		
chr14	21924370	21924706	1.11	9.00E-01	CHD8	-19134	RAB2B	20594
chr6	41888741	41889022	0.94	9.00E-01	BYSL	-83	MED20	-5
chr13	41556975	41557219	1.13	9.01E-01	ELF1	36411	SLC25A15	193550
chr2	74710021	74710269	1.02	9.01E-01	TTC31	-55	CCDC142	212
chr19	2819648	2819972	1.03	9.01E-01	ZNF554	-62		
chr15	91204512	91204822	1.07	9.01E-01	BLM	-55912	CRTC3	131469
chr17	73341890	73342233	1.04	9.01E-01	SLC25A19	-56532	GRB2	59728
chr16	31128788	31129098	0.95	9.01E-01	MYST1	-42		
chr17	7259397	7259655	1.09	9.01E-01	TNK1	-24839	TMEM95	1029
chr8	82024068	82024429	0.84	9.01E-01	PAG1	54		
chr6	26027052	26027697	1.05	9.02E-01	HIST1H4B	105		
chr14	89850414	89850740	0.99	9.02E-01	FOXN3	234917	TTC8	559659
chr8	71314794	71315059	1.23	9.02E-01	PRDM14	-331365	NCOA2	1093
chr15	40861275	40861632	1.12	9.03E-01	RPUSD2	-83		
chr9	33473874	33474281	0.99	9.03E-01	NOL6	-137		
chr9	131534131	131534393	1.03	9.03E-01	ZER1	-64		
chr1	205601071	205601345	1.00	9.03E-01	SLC45A3	48422	MFSD4	63096
chr10	11205232	11205508	0.90	9.03E-01	CUGBP2	145477	USP6NL	368904
chr11	207238	207742	0.93	9.03E-01	RIC8A	-1040	BET1L	-68
chr12	14460402	14460647	1.06	9.03E-01	GRIN2B	-327503	ATF7IP	-58086
chr7	73097763	73098044	1.03	9.03E-01	DNAJC30	-123	WBSCR22	6
chr12	14518265	14518558	1.05	9.03E-01	ATF7IP	-199		
chr22	38082172	38082482	1.01	9.04E-01	NOL12	-17		
chr8	41685836	41686168	0.99	9.04E-01	NKX6-3	-181127	ANK1	68278
chr10	6244609	6244998	0.89	9.04E-01	PFKFB3	-36		
chr12	111325850	111326160	0.91	9.04E-01	PPP1CC	-145248	MYL2	32399
chr4	170533349	170533832	0.97	9.04E-01	NEK1	23		
chr11	65222472	65222937	1.05	9.04E-01	SCYL1	-69843	FRMD8	68664
chr1	62901935	62902312	0.95	9.05E-01	USP1	149		
chr16	66994958	66995212	0.89	9.05E-01	CES3	-53		

chr7	44162845	44163085	0.96	9.05E-01	POLD2	182		
chr8	27631733	27631996	1.02	9.05E-01	ESCO2	-193		
chr17	73629022	73629337	0.93	9.05E-01	KIAA1783	20125	RECQL5	34089
chr2	162016802	162017220	1.05	9.05E-01	PSMD14	-147775	TANK	23545
chr1	38455569	38455970	0.90	9.05E-01	SF3A3	-9		
chr8	74884362	74884769	1.11	9.05E-01	TMEM70	-3864	TCEB1	-220
chr1	66708431	66708675	0.97	9.05E-01	SGIP1	-291272	PDE4B	449697
chr3	182817215	182817597	1.11	9.06E-01	MCCC1	-41		
chr11	119130547	119131026	1.22	9.06E-01	CBL	53797	MCAM	57053
chr11	46615486	46615796	0.93	9.07E-01	AMBRA1	-2727		
chr17	7591419	7591740	1.09	9.07E-01	TP53	-717		
chr3	71591752	71592187	1.09	9.07E-01	FOXP1	41170		
chr19	3761436	3762138	0.91	9.07E-01	MRPL54	-878	APBA3	-114
chr17	47633693	47634003	0.98	9.07E-01	NXPH3	-19450	NGFR	61193
chr3	18265054	18265375	0.98	9.07E-01	TBC1D5	-480975	SATB1	215037
chrX	149108642	149108892	0.99	9.07E-01	MAMLD1	-422919	HSFX1	-252994
chr10	74927747	74927987	1.03	9.08E-01	ECD	-14		
chr11	6704477	6704780	0.98	9.08E-01	MRPL17	3		
chr6	28863169	28863475	0.96	9.08E-01	SCAND3	-308210	TRIM27	28446
chr8	38820663	38820915	1.17	9.08E-01	HTRA4	-10879	PLEKHA2	62036
chr15	35261867	35262147	1.06	9.08E-01	AQR	-12		
chr2	86667787	86668251	1.08	9.08E-01	KDM3A	-565		
chr2	26256630	26257254	1.02	9.08E-01	RAB10	213		
chr3	39424817	39425127	1.10	9.08E-01	SLC25A38	157		
chr3	155572163	155572661	1.14	9.08E-01	SLC33A1	-245		
chr12	14923853	14924204	1.10	9.08E-01	H2AFJ	-3241	HIST4H4	36
chr8	117778470	117778821	1.00	9.08E-01	UTP23	-96		
chr20	30272452	30272799	1.05	9.08E-01	BCL2L1	38030	COX4I2	46935
chr17	29233112	29233471	1.10	9.08E-01	C17orf42	-6		
chr15	90808554	90809009	1.01	9.09E-01	NGRN	-113		
chr12	133348046	133348406	0.98	9.09E-01	ANKLE2	-9775	GOLGA3	57062
chr12	125406208	125406487	1.22	9.09E-01	UBC	-6771	DHX37	67319
chr11	67007302	67007655	1.05	9.09E-01	ADRBK1	-26426	KDM2A	120739
chr4	13612982	13613226	0.96	9.10E-01	NKX3-2	-66990	BOD1L	16224
chr9	4985044	4985320	1.14	9.10E-01	JAK2	-63		
chr1	155214485	155214801	1.05	9.10E-01	GBA	-3590		
chr17	46048305	46048569	1.03	9.10E-01	CDK5RAP3	8		
chr1	31769653	31770038	0.91	9.10E-01	SNRNP40	-202	ZCCHC17	4

chr7	101705366	101705649	1.01	9.10E-01	SH2B2	-222897	CUX1	244626		
chr6	2947599	2948023	0.99	9.10E-01	SERPINB9	-44266	SERPINB6	24279		
chr2	198010268	198010578	1.15	9.11E-01	PGAP1	-218969	ANKRD44	52339		
chr20	3140420	3140793	1.04	9.11E-01	UBOX5	-67	FASTKD5	-67		
chr3	12705577	12705960	1.05	9.11E-01	RAF1	-69				
chr11	66462203	66462526	0.97	9.11E-01	RBM4B	-17090	SPTBN2	26505		
chr6	44095030	44095301	1.02	9.11E-01	TMEM63B	-210	MRPL14	25		
chr1	63249340	63249584	1.01	9.11E-01	ATG4C	-341				
chr11	114270985	114271750	0.96	9.11E-01	RBM7	-16				
chr2	109335728	109336007	1.06	9.11E-01	RANBP2	-69				
chr10	98561316	98561560	1.03	9.11E-01	C10orf12	-179603	PIK3AP1	-81159		
chr7	130582856	130583211	1.02	9.11E-01	MKLN1	-429561	KLF14	-164174		
chr1	27226816	27227077	1.02	9.11E-01	GPATCH3	15				
chr10	12526852	12527237	1.01	9.11E-01	CAMK1D	135462	CCDC3	516659		
chr9	32552118	32552476	1.09	9.12E-01	TOPORS	304				
chr12	92757556	92757935	1.04	9.12E-01	PLEKHG7	-372519	BTG1	-218073		
chr17	37365104	37365458	1.03	9.12E-01	RPL19	8745	STAC2	16693		
chr2	74425533	74425986	1.03	9.12E-01	MTHFD2	70				
chr4	7072502	7072812	0.96	9.13E-01	GRPEL1	-2857				
chr11	102345249	102345680	1.03	9.13E-01	TMEM123	-21690	MMP7	56013		
chr1	193090919	193091542	1.12	9.13E-01	CDC73	143				
chr20	31408030	31408546	1.00	9.13E-01	MAPRE1	589				
chr1	9689752	9690167	1.02	9.13E-01	PIK3CD	-21830	TMEM201	40983		
chr15	45459581	45459891	1.06	9.13E-01	SHF	33637	DUOX1	37544		
chr12	124938129	124938420	1.06	9.13E-01	NCOR2	81882	ZNF664	480605		
chr7	155517244	155517529	0.95	9.13E-01	RBM33	80184	SHH	87580		
chr17	7835293	7835712	0.97	9.13E-01	KCNAB3	-2750	TRAPPC1	-245	CNTROB	30
chr1	151227047	151227310	0.98	9.14E-01	PSMD4	-18				
chr13	53191387	53191763	1.05	9.14E-01	HNRNPA1L2	-30				
chr2	26205404	26205754	1.02	9.14E-01	KIF3C	-136				
chr7	44621738	44622048	0.87	9.14E-01	TMED4	-66				
chr12	7000125	7000462	1.02	9.14E-01	SPSB2	-17845	LRRC23	-13603		
chr16	88748128	88748546	1.07	9.14E-01	MVD	-18842	SNAI3	4545		
chr17	74553713	74554088	1.09	9.14E-01	PRCD	17780	ST6GALNAC2	28244		
chr19	55791376	55791686	1.01	9.14E-01	BRSK1	-4003	HSPBP1	220		
chr2	3155158	3155556	0.98	9.14E-01	MYT1L	-820312	TSSC1	226296		
chr15	74988296	74988772	1.00	9.14E-01	EDC3	-148				
chr2	172174192	172174531	1.16	9.14E-01	DCAF17	-116497	TLK1	-86538		

chr7	135194853	135195154	1.17	9.14E-01	CNOT4	-153		
chr22	22901532	22901837	0.83	9.14E-01	PRAME	11		
chr20	46096980	46097401	1.02	9.14E-01	ZMYND8	-111717	NCOA3	-33466
chr6	88851325	88851684	1.03	9.14E-01	CNR1	24262	SPACA1	93998
chr20	36871304	36871614	1.00	9.14E-01	TGM2	-77759	BPI	-61093
chr1	94883634	94884007	0.97	9.15E-01	ABCD3	-112		
chr19	2051068	2051506	0.96	9.15E-01	MKNK2	-44		
chr19	5139333	5139569	0.92	9.15E-01	KDM4B	170327	PTPRS	201363
chr4	40198400	40198777	1.08	9.16E-01	RHOH	62		
chr19	45227117	45227416	0.96	9.16E-01	BCL3	-24711	CEACAM16	24909
chr1	155231825	155232450	1.11	9.16E-01	SCAMP3	57		
chr16	30441447	30441762	0.95	9.16E-01	DCTPP1	-232		
chr19	48021419	48021718	0.95	9.16E-01	NAPA	-3072		
chr2	232650941	232651427	1.12	9.16E-01	COPS7B	22		
chr5	138725438	138725785	0.90	9.16E-01	MGC29506	-7		
chr1	26560333	26560589	0.96	9.16E-01	SH3BGRL3	-45752	CATSPER4	43342
chr11	65190028	65190391	1.03	9.16E-01	SCYL1	-102338	FRMD8	36169
chr17	75441482	75441980	1.03	9.16E-01	TNRC6C	-558587	SEPT9	164239
chr20	39620095	39620411	1.11	9.16E-01	MAFB	-302377	TOP1	-37209
chr20	49434861	49435200	1.08	9.16E-01	BCAS4	23564	ADNP	112496
chr6	26568937	26569247	1.08	9.16E-01	ABT1	-28088	HMGN4	30520
chr22	22562315	22562683	0.99	9.17E-01	TOP3B	-225352	VPREB1	-36701
chr17	8124721	8125018	1.00	9.17E-01	AURKB	-10987	C17orf68	26543
chr9	37119972	37120769	0.96	9.18E-01	ZCCHC7	-98		
chr7	142153256	142153515	1.07	9.18E-01	TCRBV6S6A2T	-13571	TCRBV1S1A1N1	86647
chr13	77601093	77601342	1.12	9.19E-01	FBXL3	113		
chr6	51928340	51928650	1.31	9.19E-01	PKHD1	23928		
chr1	28559418	28559662	0.93	9.19E-01	ATPIF1	-3071	DNAJC8	2
chr11	65264881	65265205	0.90	9.19E-01	SCYL1	-27505	FRMD8	111002
chr17	47841540	47841888	0.91	9.19E-01	SLC35B1	-56432	MYST2	-24357
chr3	51975774	51976455	0.91	9.19E-01	PARP3	-246	RRP9	-193
chr2	86263619	86263938	1.12	9.19E-01	ST3GAL5	-147622	POLR1A	69499
chr12	112279644	112279998	1.05	9.19E-01	MAPKAPK5	-458		
chr17	78459598	78459991	0.96	9.19E-01	RPTOR	-58830	NPTX1	-9391
chr22	30820121	30820374	1.08	9.19E-01	SEC14L2	27315	SEC14L3	47786
chr17	38269373	38269722	1.14	9.19E-01	NR1D1	-12575	MSL1	-9242
chr17	75437282	75438098	0.96	9.19E-01	TNRC6C	-562628	SEPT9	160198
chr1	27488170	27488439	1.01	9.19E-01	WDTC1	-72702	SLC9A1	-6854

chr9	4987905	4988214	1.02	9.19E-01	JAK2	2815	INSL6	197558
chr11	82867780	82868344	1.05	9.20E-01	PCF11	-75		
chr3	10067939	10068480	1.08	9.20E-01	FANCD2	97		
chr1	225965294	225965564	1.04	9.20E-01	SRP9	-86		
chr1	212629235	212629550	0.99	9.20E-01	ATF3	-109304	NENF	23164
chr14	104544354	104544630	0.95	9.20E-01	ASPG	-7556	TDRD9	149675
chr1	112050238	112050576	0.95	9.20E-01	ADORA3	-3664		
chr6	28949877	28950121	1.03	9.20E-01	TRIM27	-58231	ZNF311	23036
chr16	72042404	72042847	1.05	9.20E-01	DHODH	-17		
chr17	73285562	73286007	1.11	9.20E-01	SLC25A19	-255		
chr12	95467323	95467598	0.97	9.20E-01	NR2C1	-57		
chr2	98280471	98280887	0.85	9.20E-01	ACTR1B	-118		
chr12	57915805	57916132	0.91	9.21E-01	DDIT3	-1669	MBD6	-690
chr16	16101604	16102060	1.03	9.21E-01	ABCC1	58398	ABCC6	215496
chr8	56798102	56798533	1.03	9.21E-01	LYN	5932	RPS20	188822
chr17	17991032	17991358	1.07	9.21E-01	DRG2	-88		
chr19	6633270	6633544	1.12	9.21E-01	CD70	-42244	TNFSF14	37192
chr15	102192420	102192730	1.03	9.21E-01	TM2D3	19		
chr6	111880375	111880754	0.80	9.22E-01	REV3L	-76133	TRAF3IP2	46909
chr1	112050813	112051132	0.93	9.23E-01	ADORA3	-4230		
chr19	44289506	44289761	1.01	9.23E-01	KCNN4	-4225		
chr17	37309908	37310237	0.97	9.23E-01	PLXDC1	-2171		
chr17	7755066	7755408	0.94	9.23E-01	TMEM88	-3147		
chr1	155826954	155827394	1.11	9.23E-01	SYT11	-2116	GON4L	-202
chr13	49136548	49136805	1.26	9.23E-01	CYSLTR2	-144276	RCBTB2	-29361
chr19	50168756	50169322	0.95	9.23E-01	IRF3	93	BCL2L12	640
chr6	26123858	26124168	1.00	9.23E-01	HIST1H2AC	-360	HIST1H2BC	119
chr1	224622432	224622676	1.00	9.23E-01	WDR26	-822		
chr6	52149347	52150130	1.00	9.23E-01	MCM3	-157		
chr9	101017838	101018343	1.01	9.23E-01	TBC1D2	-88		
chr1	159880269	159880543	1.06	9.23E-01	CCDC19	-10500	TAGLN2	14878
chr13	96032948	96033240	1.04	9.23E-01	CLDN10	-171853	ABCC4	-79407
chr17	66201378	66201647	1.05	9.23E-01	AMZ2	-42632	KPNA2	169665
chr2	206571893	206572176	0.92	9.23E-01	NRP2	24811	NDUFS1	452152
chr6	37019241	37019579	0.93	9.23E-01	PIM1	-118512	FGD2	45987
chr6	126101292	126101625	1.15	9.23E-01	NCOA7	-10542	HEY2	30727
chr17	19281200	19281704	1.01	9.24E-01	MAPK7	-322		
chr6	41040187	41040790	1.02	9.24E-01	NFYA	-218		

chr22	42466754	42467150	0.94	9.24E-01	NAGA	-106		
chr5	76788247	76788557	1.09	9.24E-01	OTP	146120	PDE8B	281696
chr18	33552449	33552714	0.99	9.24E-01	SLC39A6	156775	GALNT1	318049
chr5	32313155	32313429	0.97	9.24E-01	MTMR12	-178		
chr17	79075575	79075826	0.99	9.24E-01	AATK	64171	BAIAP2	66754
chr19	6802383	6802917	1.00	9.24E-01	EMR1	-84932	VAV1	29928
chr17	7387975	7388229	1.01	9.24E-01	ZBTB4	-534	POLR2A	252
chr9	126964760	126965047	1.06	9.24E-01	NEK6	-55339	LHX2	191015
chr11	29549144	29549531	1.02	9.24E-01	KCNA4	489150		
chr7	65670072	65670411	0.83	9.24E-01	TPST1	-17		
chr1	37942882	37943179	1.02	9.24E-01	ZC3H12A	2912	MEAF6	37333
chr10	125163837	125164147	1.12	9.24E-01	GPR26	-261879	BUB3	250232
chr13	111866833	111867110	0.96	9.24E-01	C13orf16	-106043	ARHGEF7	99348
chr15	40566862	40567217	0.98	9.24E-01	PLCB2	33134	PAK6	57411
chr19	39888324	39888645	1.09	9.24E-01	ZFP36	-9002	MED29	6522
chr3	120136196	120136598	0.97	9.24E-01	LRRC58	-68211	FSTL1	33521
chr6	20320168	20320441	1.11	9.24E-01	MBOAT1	-107635	E2F3	-81832
chr6	26322270	26322523	0.99	9.24E-01	BTN3A2	-43001	HIST1H4H	-36670
chr6	28920897	28921238	1.12	9.24E-01	TRIM27	-29300	ZNF311	51967
chr7	76022372	76022710	0.98	9.24E-01	YWHAG	-34199	SRCRB4D	16471
chr7	100026840	100027486	0.93	9.24E-01	ZCWPW1	-861	MEPCE	-366
chr17	73900993	73901353	0.89	9.25E-01	MRPL38	8		
chr17	27065051	27065456	0.93	9.25E-01	TRAF4	-5769	NEK8	9422
chr12	32417858	32418293	0.95	9.25E-01	FGD4	-236965	BICD1	157891
chr10	116697842	116698198	1.07	9.25E-01	TRUB1	68		
chr4	103266316	103266829	1.04	9.25E-01	SLC39A8	82		
chr16	4321692	4322107	1.10	9.25E-01	SRL	-29819	TFAP4	1101
chr4	174255519	174255982	0.93	9.26E-01	HMGB2	-831		
chr1	10458686	10459051	0.83	9.26E-01	PGD	-216		
chr4	41216636	41216925	0.88	9.26E-01	APBB2	-146		
chr11	28131673	28131992	1.18	9.26E-01	KIF18A	-2087	METT5D1	2035
chr12	109058658	109058917	1.05	9.26E-01	SELPLG	-31118	CORO1C	66507
chr1	156571119	156571517	1.00	9.27E-01	GPATCH4	-48		
chr11	124981390	124981751	0.98	9.27E-01	PKNOX2	-52988	TMEM218	-8366
chr1	151162531	151162841	1.07	9.27E-01	VPS72	-46		
chr17	7475722	7476147	1.06	9.27E-01	EIF4A1	333		
chr16	30583090	30583596	1.09	9.27E-01	ZNF688	385		
chr17	61677997	61678372	1.17	9.28E-01	TACO1	-58		

chr19	3985325	3985663	1.13	9.28E-01	EEF2	-33		
chr1	150208202	150208700	1.05	9.28E-01	ANP32E	53		
chr6	163834977	163835221	0.95	9.28E-01	QKI	-576		
chr8	104032916	104033594	0.94	9.28E-01	ATP6V1C1	7		
chr12	25403765	25404093	0.84	9.28E-01	KRAS	-75		
chr15	41694574	41694850	1.04	9.28E-01	NDUFAF1	-70		
chr1	214776189	214776558	1.17	9.28E-01	CENPF	-158		
chr10	97054915	97055301	0.98	9.28E-01	PDLIM1	-4327		
chr19	51869731	51870045	0.94	9.28E-01	ETFB	-11792	CLDND2	2369
chr2	156002300	156002655	1.05	9.28E-01	KCNJ3	447385		
chr17	74137283	74137519	0.83	9.28E-01	FOXJ1	-756		
chr17	42287819	42288095	1.04	9.28E-01	ATXN7L3	-12428	UBTF	7707
chr1	45987386	45987738	1.05	9.29E-01	PRDX1	47		
chr16	68271474	68271923	1.03	9.29E-01	ESRP2	-1563		
chr6	88632586	88632930	0.93	9.29E-01	AKIRIN2	-220773	SPACA1	-124749
chr8	82633476	82633833	0.99	9.29E-01	ZFAND1	-125		
chr14	74220863	74221111	0.99	9.29E-01	PNMA1	-39859	C14orf43	32909
chr17	57784669	57785091	1.09	9.29E-01	PTRH2	-24	TMEM49	17
chr19	49077985	49078264	1.01	9.29E-01	SPACA4	-31875	SULT2B1	22696
chr20	62694259	62694556	1.07	9.29E-01	TCEA2	5969	RGS19	16916
chr19	14640100	14640375	0.92	9.29E-01	TECR	-144		
chr16	70438263	70438624	1.26	9.29E-01	ST3GAL2	34547	DDX19B	105372
chr5	43017982	43018365	1.03	9.29E-01	C5orf39	22273	GHR	594148
chr4	17616088	17616390	0.99	9.29E-01	MED28	-34		
chr11	71639284	71639664	1.07	9.29E-01	RNF121	-294	AK131288	14
chr12	58299161	58299474	1.07	9.29E-01	CTDSP2	-58571	XRCC6BP1	-36127
chr3	72149958	72150203	1.09	9.29E-01	PROK2	-315724	RYBP	345693
chr22	22749009	22749334	0.81	9.29E-01	ZNF280B	114333	VPREB1	149972
chr11	67250495	67251182	1.01	9.29E-01	AIP	334		
chr22	46731420	46731758	1.06	9.29E-01	TRMU	291		
chr19	45972346	45972770	0.91	9.29E-01	FOSB	1305	RTN2	27755
chr12	51157580	51157863	0.93	9.30E-01	ATF1	-97		
chr17	38443715	38444067	0.98	9.30E-01	CDC6	-255		
chr8	146277716	146277960	0.93	9.30E-01	ZNF252	-49553		
chr18	30499796	30500078	1.13	9.30E-01	ASXL3	-658604	KLHL14	-146963
chr7	22862303	22862628	0.90	9.30E-01	TOMM7	-45		
chr7	75115483	75115768	1.03	9.30E-01	POM121C	-58		
chr17	8152371	8152714	0.90	9.30E-01	C17orf68	-1130	PFAS	-53

chr17	38267834	38268161	0.88	9.30E-01	NR1D1	-11025	MSL1	-10792
chr2	172290260	172290794	1.01	9.30E-01	DCAF17	-332	METTL8	785
chr9	139743605	139743857	1.08	9.30E-01	MAMDC4	-3088	PHPT1	475
chr3	32022219	32022529	1.05	9.30E-01	ZNF860	-892	OSBPL10	864
chr20	30308703	30309019	1.10	9.30E-01	BCL2L1	1795	COX4I2	83170
chr8	67026274	67026618	0.97	9.30E-01	TRIM55	-12832	DNAJC5B	92655
chr12	12849514	12849850	1.02	9.30E-01	GPR19	-561		
chr14	73524848	73525283	0.86	9.31E-01	RBM25	-155		
chr8	110346263	110346765	1.01	9.31E-01	ENY2	-122		
chr13	30969614	30969902	1.14	9.31E-01	KATNAL1	-88595	HMGB1	70323
chr20	35474558	35474820	1.06	9.31E-01	DSN1	-72535	SAMHD1	105487
chrX	136520345	136520622	0.85	9.31E-01	GPR101	-406651	ZIC3	-127862
chr9	101998157	101998497	1.08	9.31E-01	NR4A3	-590682	SEC61B	13757
chr16	50058779	50059152	1.02	9.31E-01	TMEM188	-223		
chr10	6130687	6131079	1.05	9.32E-01	RBM17	-66		
chr1	111948948	111949323	0.98	9.32E-01	OVGP1	21263	CHIA	115652
chr3	187650339	187650713	1.09	9.32E-01	LPP	-280195	BCL6	-187051
chr6	159465901	159466250	0.97	9.32E-01	TAGAP	108		
chr15	93382945	93383255	0.82	9.32E-01	FAM174B	-184069	CHD2	-60451
chr14	68303779	68304055	1.09	9.32E-01	RAD51L1	17408	ZFP36L1	955868
chr21	44819421	44819800	1.08	9.32E-01	SIK1	27391	CRYAA	230470
chr14	65380893	65381203	0.93	9.32E-01	SPTB	-91185	GPX2	28483
chr2	48133802	48134291	0.91	9.33E-01	FBXO11	-1233		
chr2	65617255	65617585	1.12	9.33E-01	SPRED2	42236	ACTR2	162591
chr1	151371772	151372086	1.05	9.33E-01	PSMB4	-112		
chr3	49066645	49067125	0.99	9.34E-01	IMPDH2	-10		
chr7	149535232	149535567	0.89	9.34E-01	ZNF862	-109		
chr3	107844401	107844756	1.07	9.34E-01	CD47	-34644	IFT57	96838
chr14	91976787	91977051	0.95	9.34E-01	SMEK1	-275		
chr5	64920053	64920297	0.91	9.34E-01	TRIM23	12		
chr6	149867110	149867350	0.98	9.34E-01	PPIL4	-59		
chr12	122985309	122985768	1.09	9.34E-01	ZCCHC8	-21		
chr1	23885791	23886298	0.90	9.34E-01	ID3	277		
chr13	50018110	50018620	0.96	9.34E-01	CAB39L	-42630	SETDB2	-7324
chr14	91526826	91527454	0.96	9.34E-01	RPS6KA5	-147		
chr13	31191060	31191368	1.00	9.35E-01	USPL1	-616		
chr20	35700082	35700398	1.36	9.35E-01	SAMHD1	-120064	RBL1	24170
chr13	103249148	103249478	0.91	9.35E-01	TPP2	27		

chr9	102790452	102790728	1.02	9.35E-01	ERP44	70740	STX17	121675
chr17	30580096	30580406	1.09	9.35E-01	RHBDL3	-12944	RHOT1	110778
chr7	148936498	148936828	0.92	9.35E-01	ZNF212	-111		
chr15	85931273	85931583	1.04	9.35E-01	AKAP13	7557	KLHL25	406761
chr2	219166019	219166342	0.99	9.35E-01	SLC11A1	-80571	TMBIM1	-8901
chr6	31757223	31757487	1.05	9.35E-01	C6orf27	-12247	VARS	6357
chr20	32319573	32319942	0.94	9.35E-01	ZNF341	-50		
chr7	96746695	96747096	1.07	9.36E-01	ACN9	991		
chr19	19774369	19774852	0.96	9.36E-01	ATP13A1	-108		
chr5	138677305	138677677	1.07	9.36E-01	PAIP2	-28		
chr16	86597451	86597703	0.94	9.36E-01	FOXC2	-3280		
chr1	206299332	206299670	0.99	9.36E-01	CTSE	-17958	C1orf186	-10854
chr11	77348696	77349114	1.02	9.36E-01	CLNS1A	-54		
chr11	62554580	62555078	0.99	9.36E-01	TMEM179B	-45		
chr12	67845896	67846241	1.10	9.36E-01	DYRK2	-196443	CAND1	183008
chr2	70369757	70370079	0.95	9.36E-01	PCBP1	55333	TIA1	105861
chr3	47021100	47021388	1.01	9.36E-01	NBEAL2	71		
chr4	147096670	147096960	0.97	9.36E-01	LSM6	-20		
chr1	85086406	85086846	1.13	9.36E-01	CTBS	-46463	SSX2IP	69554
chr17	8090546	8091169	1.08	9.36E-01	TMEM107	-11144	C17orf59	2706
chr17	79604002	79604356	0.93	9.37E-01	NPLOC4	-41		
chr19	16222427	16222823	0.95	9.37E-01	RAB8A	135		
chr13	32889477	32889822	0.96	9.37E-01	BRCA2	33		
chr6	155635638	155635980	1.05	9.37E-01	TFB1M	-183		
chr11	6502557	6502877	0.97	9.37E-01	ARFIP2	-122	FXC1	40
chr5	133879831	133880115	1.09	9.37E-01	PHF15	18175	SAR1B	88554
chr1	40840170	40840648	1.22	9.38E-01	SMAP2	681		
chr2	171785289	171785657	1.02	9.38E-01	GORASP2	-238		
chr10	74114293	74114541	1.03	9.38E-01	DNAJB12	490		
chr17	7137807	7138051	0.99	9.38E-01	DVL2	-66		
chr17	40810980	40811367	1.01	9.38E-01	TUBG2	-92		
chr19	3435205	3435449	0.99	9.38E-01	C19orf77	45213	NFIC	75711
chr12	48577220	48577595	0.82	9.38E-01	C12orf68	42		
chr16	81348169	81348519	0.93	9.38E-01	GAN	-227		
chr19	17445506	17445863	1.10	9.38E-01	GTPBP3	-2671	ANO8	-47
chr19	10538795	10539160	1.20	9.38E-01	PDE4A	7645	KEAP1	75076
chr3	38159397	38159789	1.04	9.38E-01	ACAA1	19140	DLEC1	78897
chr19	1095316	1095604	1.10	9.39E-01	POLR2E	-69		

chr19	50372736	50373154	0.89	9.39E-01	PNKP	-2127		
chr22	42093123	42093423	0.90	9.39E-01	MEI1	-2245		
chr8	27695098	27695567	0.99	9.39E-01	PBK	16		
chr1	16023444	16023731	0.91	9.39E-01	SLC25A34	-39221	PLEKHM2	12761
chr19	18107680	18107999	1.25	9.39E-01	KCNN1	45729	IL12RB1	89902
chr2	39351447	39351870	0.96	9.39E-01	SOS1	-4055		
chr1	28908190	28908612	0.98	9.39E-01	RAB42	-10311	TRNAU1AP	28872
chr9	7052629	7052886	0.85	9.39E-01	KDM4C	295117	C9orf123	747041
chr3	52188582	52189060	1.19	9.39E-01	WDR51A	-115		
chr5	154317652	154317962	1.08	9.39E-01	MRPL22	-2826	GEMIN5	-31
chr11	60542941	60543230	1.01	9.40E-01	MS4A10	-9735	MS4A15	18746
chr17	56084584	56085142	1.11	9.40E-01	SFRS1	-156		
chr6	144416680	144416986	1.09	9.40E-01	SF3B5	-79		
chr16	30609529	30609767	1.04	9.41E-01	ZNF785	-12638	ZNF689	12034
chr9	95826589	95827061	1.04	9.41E-01	C9orf89	-31625	SUSD3	5836
chr12	48499725	48500255	1.08	9.41E-01	SENP1	-349		
chr11	8704037	8704427	1.12	9.41E-01	RPL27A	237		
chr3	180629745	180630394	0.99	9.41E-01	FXR1	-382		
chr17	21002172	21002570	1.19	9.41E-01	USP22	-56019	DHRS7B	-27887
chr18	32621159	32621581	0.98	9.41E-01	ZNF397	-199628	MAPRE2	63162
chr7	157129479	157129728	0.90	9.41E-01	DNAJB6	-106		
chr10	97453649	97454097	1.02	9.42E-01	TCTN3	27		
chr17	40687884	40688262	0.97	9.42E-01	NAGLU	122		
chr14	91580813	91581064	1.03	9.42E-01	C14orf159	-40		
chr16	89752908	89753344	1.01	9.42E-01	CDK10	50		
chr6	35265383	35265693	0.94	9.42E-01	DEF6	-57		
chr15	51169174	51169535	1.11	9.42E-01	SPPL2A	-111445	AP4E1	-31591
chr19	2052352	2052609	1.00	9.42E-01	MKNK2	-1238		
chr3	39448184	39448527	1.03	9.42E-01	RPSA	152		
chr5	172386206	172386516	1.07	9.42E-01	RPL26L1	-78		
chr7	56174166	56174546	1.01	9.42E-01	CHCHD2	-169		
chr1	14057409	14057831	1.10	9.42E-01	KIAA1026	-867593	PRDM2	26270
chr13	53028543	53028866	0.95	9.42E-01	VPS36	-3942	CKAP2	-790
chr13	114518392	114518664	1.07	9.42E-01	GAS6	48518	FAM70B	56312
chr20	62339177	62339422	1.05	9.42E-01	ZGPAT	-91	ARFRP1	55
chr6	26607671	26607916	0.97	9.42E-01	ABT1	10614	ZNF322A	52169
chr6	159331705	159332017	0.85	9.42E-01	OSTCL	-53197	TAGAP	134323
chr1	183441000	183441310	1.08	9.42E-01	SMG7	-479		

chr18	23501646	23502035	0.90	9.42E-01	ZNF521	-569627	SS18	168770
chr5	177631403	177631791	0.94	9.43E-01	HNRNPAB	89		
chr3	196336199	196336488	1.05	9.43E-01	LRRC33	-30312	FBXO45	40619
chr7	121025868	121026138	1.01	9.43E-01	FAM3C	10419	WNT16	56913
chr16	29816715	29817050	0.91	9.44E-01	MAZ	-972		
chr19	49140508	49140862	0.96	9.44E-01	Sec1	-611	DBP	-46
chr17	7518533	7518808	0.99	9.44E-01	FXR2	-600		
chr12	58329744	58330113	0.95	9.44E-01	CTDSP2	-89182	XRCC6BP1	-5516
chr12	72148514	72148844	0.91	9.44E-01	RAB21	21		
chr1	107599103	107599406	0.90	9.44E-01	PRMT6	-12		
chr19	2446440	2446818	0.98	9.44E-01	TIMM13	-18754	LMNB2	10329
chr8	121822010	121822442	1.06	9.44E-01	SNTB1	2083	MTBP	364560
chr7	35768955	35769344	1.06	9.45E-01	SEPT7	-71477	HERPUD2	-34378
chr19	46009734	46010113	0.94	9.45E-01	VASP	-764		
chr1	212459549	212459859	1.04	9.45E-01	PPP2R5A	825		
chr18	267892	268255	1.03	9.46E-01	THOC1	-15		
chr18	43245076	43245388	1.11	9.46E-01	SLC14A1	-58860	SLC14A2	50466
chr19	42455763	42456082	1.03	9.46E-01	RABAC1	7605	ARHGEF1	67477
chr20	30249318	30249759	0.96	9.46E-01	COX4I2	23848	BCL2L1	61117
chr7	148224745	148225014	0.95	9.46E-01	CUL1	-171053	NM_001126313	93241
chr10	13628708	13629038	1.06	9.46E-01	PRPF18	-66		
chr17	7518125	7518420	0.97	9.46E-01	FXR2	-202		
chr15	75075659	75075980	1.12	9.46E-01	LMAN1L	-29374	CSK	1395
chr16	27410324	27410748	1.07	9.46E-01	IL21R	-28043	IL4R	85285
chr16	68285746	68285995	0.94	9.46E-01	SLC7A6	-12552	PLA2G15	6624
chr9	123605070	123605467	1.06	9.46E-01	PSMD5	-63		
chr9	136202950	136203249	0.96	9.46E-01	SURF6	-53		
chr13	113862852	113863130	1.06	9.46E-01	CUL4A	-95	PCID2	38
chr15	22461047	22461429	0.88	9.46E-01	GOLGA6L1	-275008	OR4N2	47776
chr6	24936921	24937230	1.24	9.46E-01	FAM65B	-25881	DKFZp686H12134	200975
chr15	83478015	83478471	0.95	9.46E-01	WHAMM	270		
chr16	81069177	81069548	0.98	9.46E-01	ATMIN	-95		
chr19	49834909	49835244	1.09	9.46E-01	CD37	-3600		
chr12	125634408	125634924	1.07	9.46E-01	TMEM132B	-176496	AACS	84741
chr3	12761575	12761885	1.06	9.46E-01	RAF1	-56030	TMEM40	39078
chr9	1009157	1009405	1.05	9.46E-01	DMRT2	-41339	DMRT3	32317
chr9	100797004	100797332	1.01	9.46E-01	NANS	-21514	ANP32B	51679
chr17	74667830	74668102	1.00	9.46E-01	ST6GALNAC1	-28072	MXRA7	39090

chr1	109756379	109756628	0.85	9.47E-01	SARS	-36		
chr19	11647041	11647285	1.01	9.47E-01	CNN1	-2416		
chr22	22384989	22385467	1.03	9.47E-01	VPREB1	-213972	TOP3B	-48081
chr8	97247607	97248015	1.05	9.47E-01	UQCRB	51		
chr12	112123644	112124013	0.92	9.47E-01	BRAP	-39	ACAD10	-28
chr18	30340315	30340750	0.94	9.47E-01	KLHL14	12441	MEP1B	570546
chr1	149224429	149224766	1.12	9.47E-01	PPIAL4B	-328405	NBPF16	485156
chr1	40997080	40997405	0.95	9.47E-01	ZNF684	10		
chr4	40303687	40304005	0.97	9.47E-01	CHRNA9	-33623	RHOH	105319
chr10	102819791	102820048	1.00	9.47E-01	KAZALD1	-1079		
chr13	34392040	34392316	0.98	9.47E-01	RFC3	-28		
chr19	50860366	50860700	1.15	9.47E-01	NAPSB	-12528	NAPSA	8398
chr16	8617709	8618013	1.15	9.47E-01	TMEM114	4365		
chr17	66508363	66508628	1.06	9.48E-01	PRKAR1A	386		
chr15	68075683	68075993	0.95	9.48E-01	LBXCOR1	-42103	MAP2K5	240817
chr15	74731104	74731423	1.01	9.49E-01	SEMA7A	-4965		
chr14	75179653	75179989	1.21	9.49E-01	FCF1	-29	KIAA0317	-14
chr15	49447760	49448070	1.08	9.49E-01	COPS2	-61		
chr1	54665628	54665882	0.89	9.49E-01	MRPL37	-85	CYB5RL	-9
chr4	2813779	2814135	0.90	9.49E-01	TNIP2	-55854	SH3BP2	-5955
chr1	27481410	27481808	1.00	9.50E-01	SLC9A1	-158		
chr2	146169829	146170171	0.98	9.50E-01	ZEB2	-892084		
chr1	10490337	10490674	0.96	9.50E-01	CORT	-19465	PGD	31421
chr17	75084474	75084864	1.01	9.50E-01	SEC14L1	-52336	MGAT5B	219871
chr14	58764599	58765280	0.94	9.50E-01	ARID4A	-282		
chr17	38210455	38210765	1.06	9.50E-01	MED24	279		
chr20	30311661	30311994	1.07	9.50E-01	BCL2L1	-1172		
chr8	109260706	109261125	1.00	9.50E-01	EIF3E	43		
chr8	126103789	126104170	0.98	9.50E-01	NSMCE2	-103	KIAA0196	81
chr16	10837521	10838003	1.03	9.50E-01	NUBP1	64		
chr5	116283696	116283940	1.05	9.50E-01	SEMA6A	-373267		
chr17	55038103	55038646	1.06	9.50E-01	COIL	36		
chr1	111682740	111683037	0.99	9.50E-01	DRAM2	-51	CEPT1	640
chr17	43138147	43139132	1.07	9.51E-01	NMT1	-40		
chr2	55237302	55237713	0.89	9.51E-01	RTN4	40226	EML6	285359
chr16	81110561	81110945	1.05	9.51E-01	GCSH	19227	ATMIN	41295
chr1	226543427	226543710	1.21	9.51E-01	LIN9	-46371	PARP1	52232
chr14	77500281	77500591	0.96	9.51E-01	C14orf4	-5402	ZDHHC22	107698

chr6	27841056	27841510	0.92	9.51E-01	HIST1H3I	-1184	HIST1H4L	6
chr17	2304712	2305399	0.99	9.52E-01	MNT	-798		
chr3	176913948	176914189	1.16	9.52E-01	TBL1XR1	979		
chr5	149340096	149340386	0.98	9.52E-01	SLC26A2	-59		
chr7	104652973	104653873	0.98	9.52E-01	MLL5	-1214		
chr15	51200581	51201003	1.01	9.52E-01	AP4E1	-154		
chr3	71471180	71471448	1.04	9.52E-01	FOXP1	161826		
chr15	40346943	40347253	1.12	9.52E-01	SRP14	-15709	BMF	53977
chr12	4644281	4644570	0.99	9.52E-01	RAD51AP1	-3524		
chr11	62432669	62432985	0.99	9.52E-01	METTL12	48	METTL12	48
chr16	4526192	4526476	1.02	9.52E-01	NMRAL1	-1438	HMOX2	-7
chr9	131200935	131201245	1.04	9.53E-01	ODF2	-17342	CERCAM	18331
chr3	197461624	197461913	0.94	9.53E-01	BDH1	-178911	KIAA0226	2004
chr1	149858059	149858757	1.00	9.53E-01	HIST2H2BE	-176	HIST2H2AC	-117
chr3	176778363	176778675	1.25	9.53E-01	TBL1XR1	136529		
chr1	37945245	37945555	0.89	9.53E-01	ZC3H12A	5281	MEAF6	34964
chr19	11052732	11053039	1.18	9.53E-01	SMARCA4	-18712	YIPF2	-13529
chr7	128735303	128735692	1.04	9.53E-01	TSPAN33	-49214	TNPO3	-40300
chr6	64281925	64282363	1.06	9.53E-01	PTP4A1	224		
chr16	89181440	89181967	0.97	9.53E-01	CDH15	-56459	ACSF3	21450
chr19	13261006	13261337	1.21	9.53E-01	STX10	-185	IER2	-110
chr19	18117827	18118071	1.02	9.53E-01	KCNN1	55838	IL12RB1	79793
chr7	112090377	112090642	0.92	9.53E-01	C7orf53	-30398	IFRD1	27284
chr1	24018117	24018517	1.03	9.54E-01	RPL11	23		
chr12	122356222	122356546	1.03	9.54E-01	BCL7A	-103477	PSMD9	29738
chr14	102276327	102276792	0.99	9.54E-01	DYNC1H1	-154305	PPP2R5C	48425
chr2	238583401	238583803	1.05	9.54E-01	RAB17	-83866	LRRFIP1	-17205
chr2	197040686	197041201	0.97	9.54E-01	STK17B	-4608		
chr19	507034	507611	0.94	9.54E-01	C19orf20	-174		
chr11	61129563	61129925	1.04	9.54E-01	TMEM138	-112		
chr11	67273571	67273847	0.99	9.54E-01	PITPNM1	-866		
chr19	19174286	19174798	0.92	9.54E-01	SLC25A42	-266		
chr6	20534549	20534798	1.01	9.55E-01	CDKAL1	-14		
chr19	40696621	40697157	1.20	9.55E-01	MAP3K10	-762		
chr7	44613374	44613738	1.01	9.55E-01	DDX56	581		
chr17	45078534	45078810	0.96	9.55E-01	RPRML	-22058	CDC27	187993
chr1	203242254	203242556	0.97	9.55E-01	CHIT1	-43545	BTG2	-32259
chr17	73775537	73776285	1.02	9.55E-01	UNK	-4770	H3F3B	-51

chr2	219260844	219261146	1.09	9.56E-01	CTDSP1	-3483		
chr12	56862142	56862496	0.96	9.57E-01	SPRYD4	18		
chr19	3971151	3971435	0.89	9.57E-01	DAPK3	-1466		
chr15	72366860	72367152	0.94	9.57E-01	MYO9A	43416	NR2E3	264112
chr1	160765769	160766045	0.98	9.57E-01	LY9	-21		
chr19	19887008	19887639	1.07	9.57E-01	ZNF14	-43403	ZNF506	45236
chr20	34730799	34731171	0.97	9.57E-01	SCAND1	-188557	EPB41L1	-11677
chr6	28806121	28806410	1.02	9.57E-01	SCAND3	-251154	TRIM27	85502
chr19	58077833	58078069	1.03	9.57E-01	ZNF550	-10226	ZNF416	12292
chr12	123717230	123717572	0.93	9.57E-01	C12orf65	-440		
chr3	60541538	60541792	1.29	9.57E-01	FHIT	695468		
chr1	200988576	200988947	1.07	9.57E-01	KIF21B	4066	GPR25	146596
chr17	4118576	4118940	1.01	9.57E-01	ANKFY1	48516	CYB5D2	72296
chr9	125702805	125703231	0.95	9.57E-01	RABGAP1	-270		
chr16	87862099	87862393	0.99	9.57E-01	KLHDC4	-62704	SLC7A5	40854
chr2	65142297	65142709	1.09	9.57E-01	SERTAD2	-261457	SLC1A4	-73953
chr19	18362665	18362920	0.97	9.58E-01	PDE4C	-3783		
chr9	35658193	35658453	0.94	9.58E-01	CCDC107	12		
chr5	141348477	141348859	0.99	9.58E-01	RNF14	2231	GNPDA1	43952
chr7	128580594	128580839	1.00	9.58E-01	IRF5	2723	TNPO3	114481
chr14	69283406	69283722	0.97	9.58E-01	ZFP36L1	-23779	ACTN1	162519
chr17	48785103	48785485	1.06	9.58E-01	LUC7L3	-11683	ABCC3	73076
chr3	180707452	180707874	1.07	9.58E-01	DNAJC19	-133		
chr6	42897641	42898072	1.09	9.58E-01	CNPY3	997		
chr6	30685211	30685530	0.94	9.58E-01	TUBB	-2786	MDC1	87
chr12	129325464	129325744	1.12	9.58E-01	SLC15A4	-17063	GLT1D1	-12477
chr1	206234442	206234737	0.96	9.58E-01	AVPR1B	10307	C1orf186	54057
chr7	148564764	148565011	0.85	9.59E-01	EZH2	16526	CUL1	168955
chr10	94333716	94334020	0.92	9.59E-01	IDE	-16		
chr20	37554689	37555163	1.10	9.59E-01	FAM83D	-29		
chr11	2552652	2552928	0.87	9.59E-01	KCNQ1	86569	CDKN1C	354205
chr3	187618642	187619059	0.91	9.59E-01	LPP	-311870	BCL6	-155376
chr2	220507248	220507580	0.93	9.59E-01	SLC4A3	15122		
chr19	49843593	49843999	0.94	9.59E-01	CD37	5119	TEAD2	21918
chr1	39325446	39325682	0.99	9.59E-01	RRAGC	-224		
chr17	61776609	61776924	1.02	9.60E-01	LIMD2	752	LIMD2	752
chr13	50366856	50367559	1.05	9.60E-01	KPNA3	-151		
chr9	37034710	37035093	0.99	9.60E-01	PAX5	-426		

chr14	77519632	77519876	0.92	9.60E-01	C14orf4	-24720	ZDHHC22	88380
chr16	28833775	28834097	0.99	9.60E-01	ATXN2L	-478		
chr1	28975016	28975337	1.08	9.60E-01	GMEB1	-20067	TAF12	-5573
chr7	100303521	100303831	0.96	9.60E-01	POP7	0		
chr3	191781375	191781616	1.05	9.60E-01	FGF12	345342	PYDC2	602544
chr19	13204655	13204913	0.87	9.60E-01	LYL1	8897	NFIX	98200
chr17	26821785	26822029	0.99	9.60E-01	FOXN1	-29052	SLC13A2	21243
chr9	95527165	95527475	0.85	9.61E-01	BICD2	-237		
chr9	130797444	130797758	0.93	9.61E-01	DPM2	-96838	NAIF1	31998
chr18	24653267	24653711	1.00	9.61E-01	AQP4	-207773	CHST9	111800
chr6	28625905	28626217	1.20	9.61E-01	SCAND3	-70949	TRIM27	265707
chr1	21059518	21059923	0.89	9.61E-01	SH2D5	-588		
chr1	26632984	26633384	0.99	9.61E-01	UBXN11	11		
chr14	51302002	51302387	1.08	9.61E-01	NIN	-4356		
chr11	60222814	60223388	1.21	9.62E-01	MS4A1	-181		
chr3	51572542	51572830	1.10	9.62E-01	RAD54L2	-2910		
chr4	170541381	170541822	0.88	9.62E-01	CLCN3	-120		
chr14	50583155	50583395	1.06	9.62E-01	SOS2	114824	ARF6	223539
chr17	40169357	40169633	1.04	9.62E-01	NKIRAS2	-2592	DNAJC7	176
chr15	81558779	81559108	1.12	9.62E-01	STARD5	57580	IL16	69725
chr5	131761265	131761557	0.96	9.62E-01	SLC22A5	56010	IRF1	65054
chr5	124084001	124084275	0.98	9.62E-01	ZNF608	-3273		
chr10	134145316	134145744	0.79	9.63E-01	LRRC27	-84		
chr10	70660874	70661294	0.94	9.63E-01	DDX50	50		
chr19	10527315	10527646	0.83	9.63E-01	PDE4A	-3852		
chr2	77243619	77243921	1.01	9.63E-01	LRRTM4	505732		
chr2	237994299	237994581	0.96	9.63E-01	COPS8	356		
chr5	139493471	139493762	1.06	9.63E-01	PURA	-91		
chr22	19304279	19304589	1.21	9.63E-01	CLTCL1	-25195	HIRA	114785
chr12	22697339	22697777	1.01	9.63E-01	KIAA0528	-106		
chr7	73082015	73082306	0.88	9.63E-01	VPS37D	-13		
chr19	45922793	45923090	1.01	9.64E-01	ERCC1	3860	CD3EAP	13475
chr13	51486120	51486456	1.09	9.64E-01	RNASEH2B	2396	GUCY1B2	154055
chr21	34915083	34915431	1.14	9.64E-01	GART	-793	SON	-93
chr3	186739482	186739810	1.10	9.64E-01	ST6GAL1	-19		
chr1	45476925	45477190	1.11	9.64E-01	UROD	-772	HECTD3	-31
chr19	46195505	46195962	0.90	9.64E-01	SNRPD2	-291	QPCTL	-7
chr1	40780954	40781292	0.90	9.64E-01	COL9A2	1858	ZMPSTE24	57390

chr19	3180775	3181036	0.90	9.65E-01	NCLN	-4969		
chr6	11374563	11374842	0.98	9.65E-01	TMEM170B	-163808	NEDD9	-141788
chr6	43027021	43027446	0.97	9.65E-01	KLC4	-395	MRPL2	8
chr15	59271634	59272059	1.11	9.65E-01	SLTM	-45995	RNF111	-8018
chr15	75497082	75497413	1.20	9.65E-01	DNM1P33	98058	PPCDC	181321
chr5	43121289	43121701	0.94	9.65E-01	ZNF131	-147		
chr7	93681783	93682028	1.25	9.65E-01	COL1A2	-341967	BET1	-48216
chr20	34359652	34359930	0.99	9.66E-01	PHF20	-132		
chr7	75677187	75677533	1.09	9.66E-01	STYXL1	-39	MDH2	-33
chr7	135347047	135347400	0.97	9.67E-01	SLC13A4	65709	NUP205	104562
chr16	70472991	70473531	0.82	9.67E-01	ST3GAL2	-270		
chr10	125795438	125795778	0.90	9.67E-01	CPXM2	-144108	CHST15	10632
chr6	119558438	119558761	0.74	9.67E-01	MCM9	-302297	MAN1A1	112326
chr5	134209879	134210160	0.94	9.67E-01	TXNDC15	560		
chr12	56520025	56520306	1.05	9.67E-01	ESYT1	-1888		
chr19	11546247	11546563	1.00	9.67E-01	PRKCSH	136		
chr19	4342768	4343018	1.08	9.67E-01	STAP2	-3952	MPND	-631
chr3	133194111	133194542	1.22	9.67E-01	CDV3	-98107	BFSP2	75537
chr8	28479767	28480049	0.88	9.67E-01	EXTL3	-79245	FZD3	128135
chr1	173683831	173684186	1.09	9.67E-01	KLHL20	-71		
chr15	38746176	38746486	1.00	9.67E-01	FAM98B	3		
chr20	26188787	26189001	0.79	9.68E-01	ZNF337	-511425		
chr17	36714438	36714728	1.01	9.68E-01	SNIP	47600	ARHGAP23	100939
chr9	35811982	35812452	0.97	9.69E-01	SPAG8	42		
chr10	64576518	64576838	0.87	9.69E-01	EGR2	-552		
chr11	85956049	85956362	1.00	9.69E-01	EED	391		
chr16	89188990	89189266	0.89	9.69E-01	CDH15	-49035	ACSF3	28874
chr2	25008525	25008797	1.05	9.69E-01	C2orf79	7590	NCOA1	201316
chr3	169683979	169684648	1.06	9.69E-01	SEC62	-266		
chr14	50335018	50335335	1.06	9.69E-01	ARF6	-24559	SDCCAG1	-15638
chr17	36907997	36908252	1.16	9.69E-01	PCGF2	-3564	PSMB3	-877
chr16	20753050	20753372	1.00	9.69E-01	ACSM1	-50633	ACSM3	-22101
chr17	56406206	56406528	1.22	9.70E-01	BZRAP1	-215		
chr6	156600385	156600730	0.91	9.70E-01	NOX3	-823521	ARID1B	-498528
chr7	148787740	148788083	0.91	9.71E-01	ZNF786	-43		
chr6	167526552	167526862	1.15	9.71E-01	CCR6	1412	GPR31	44612
chr1	192777926	192778307	0.97	9.71E-01	RGS2	-52		
chr19	50833985	50834381	0.90	9.71E-01	KCNC3	-1549		

chr20	40321999	40322373	1.05	9.71E-01	CHD6	-75053				
chr11	95745333	95745667	0.97	9.71E-01	MTMR2	-88129	MAML2	330844		
chr19	54694516	54695020	1.01	9.71E-01	MBOAT7	-1035	TSEN34	-336		
chr6	83777213	83777462	1.05	9.71E-01	UBE2CBP	-1793	DOPEY1	-47		
chr7	28855515	28855853	1.00	9.71E-01	TRIL	142345	CREB5	403540		
chr11	62341219	62341740	0.95	9.72E-01	EEF1G	-20				
chr1	151894100	151894410	1.13	9.72E-01	THEM4	-12142	S100A10	72459		
chr20	37491779	37492208	1.03	9.72E-01	FAM83D	-62961	PPP1R16B	57646		
chr18	47807743	47808066	1.05	9.72E-01	MBD1	239				
chr17	79367721	79368151	0.72	9.72E-01	TMEM105	-63462	BAHCC1	-5604		
chr9	126980981	126981288	0.94	9.72E-01	NEK6	-39108	LHX2	207246		
chr16	2653270	2653611	1.15	9.72E-01	PDPK1	39856	PDPK1	65471		
chr19	35704815	35705226	1.08	9.73E-01	FAM187B	14607	FXYD5	59394		
chr14	105282310	105282620	1.01	9.73E-01	KIAA0284	-49189	ZBTB42	15532		
chr6	139695618	139695995	0.96	9.73E-01	CITED2	-22				
chr10	103892467	103892826	0.98	9.73E-01	PPRC1	-140				
chr15	66161701	66162012	1.13	9.73E-01	RAB11A	61				
chr11	116643466	116643971	1.15	9.73E-01	ZNF259	15020				
chr11	65627782	65628094	0.94	9.73E-01	CFL1	-1837	CFL1	-1837	MUS81	66
chr11	47788852	47789359	0.99	9.73E-01	FNBP4	-113				
chr7	141251026	141251364	0.98	9.73E-01	AGK	117				
chr12	120105416	120105838	1.06	9.73E-01	PRKAB1	-134				
chr15	51057778	51058053	0.92	9.73E-01	SPPL2A	-6				
chr15	66994489	66994818	0.84	9.73E-01	SMAD6	-20				
chr19	9879223	9879601	1.20	9.73E-01	ZNF846	-2				
chr9	116172944	116173233	1.06	9.73E-01	POLE3	-60				
chr16	14091808	14092093	1.05	9.73E-01	MKL2	-73245	ERCC4	77937		
chr15	50647233	50647687	1.11	9.74E-01	GABPB1	145				
chr4	174185845	174186308	0.85	9.74E-01	HMGB2	68843	GALNT7	96173		
chr19	6166042	6166358	0.91	9.74E-01	ACSBG2	30490	MLLT1	113759		
chr3	122134789	122135416	1.05	9.74E-01	FAM162A	32080	KPNA1	98683		
chr14	102990924	102991369	0.87	9.74E-01	CINP	-161894	RCOR1	-68086		
chr10	88854469	88854845	0.92	9.75E-01	GLUD1	-34				
chr21	33651125	33651772	1.03	9.75E-01	C21orf45	-73				
chr17	4268473	4268749	1.00	9.75E-01	ANKFY1	-101337	UBE2G1	1358		
chr6	5030946	5031226	1.08	9.75E-01	RPP40	-26815	LYRM4	230082		
chr9	35732246	35732490	1.09	9.75E-01	TLN1	24	CREB3	51		
chr11	7532690	7532937	0.93	9.75E-01	PPFIBP2	-2187				

chr4	7069676	7070041	0.96	9.75E-01	GRPEL1	-59		
chr6	12052120	12052364	0.98	9.75E-01	EDN1	-238287	HIVEP1	39518
chr20	56070327	56070637	0.97	9.75E-01	HMGB1L1	-6399	CTCFL	29701
chr17	41277150	41277702	1.03	9.75E-01	BRCA1	74		
chr12	123237117	123237381	1.12	9.75E-01	DENR	-122		
chr11	76092051	76092312	0.95	9.75E-01	PRKRIR	-302		
chr13	25139117	25139402	1.24	9.75E-01	ATP12A	-115435	PARP4	-52312
chr12	56615664	56616054	0.94	9.76E-01	OBFC2B	-2266	RNF41	-155
chr6	26233797	26234126	1.00	9.76E-01	HIST1H1D	1254	HIST1H3E	8579
chr17	900061	900495	1.00	9.76E-01	TIMM22	-79		
chr4	185185378	185185624	0.89	9.76E-01	ENPP6	-46387	IRF2	210225
chr4	4346568	4346906	0.96	9.76E-01	D4S234E	-40847	ZNF509	54813
chr1	46713231	46713598	0.92	9.76E-01	RAD54L	48		
chr2	27008640	27008955	0.92	9.76E-01	CENPA	-84		
chr14	105947253	105947499	1.06	9.76E-01	CRIP1	-5881	CRIP2	6245
chr5	131755321	131755669	0.88	9.76E-01	SLC22A5	50094	IRF1	70970
chr17	73511817	73512127	0.97	9.77E-01	TSEN54	-637	CASKIN2	-345
chr5	64858936	64859239	0.89	9.77E-01	CENPK	-93	PPWD1	-43
chr7	25164804	25165235	0.95	9.77E-01	CYCS	-65		
chr4	1124421	1124731	0.88	9.77E-01	RNF212	-16994	SPON2	42065
chr1	29211464	29211828	0.92	9.77E-01	EPB41	-1982		
chr15	41047361	41047830	1.04	9.77E-01	FAM82A2	-138		
chr15	66649073	66649492	0.97	9.77E-01	TIPIN	-229		
chr8	125462905	125463149	0.97	9.77E-01	TRMT12	-21		
chr11	35334908	35335187	0.89	9.77E-01	SLC1A2	106057	CD44	174631
chr16	85649590	85649858	0.99	9.77E-01	KIAA0182	2800	GINS2	72864
chr17	7117771	7118039	0.90	9.77E-01	ASGR1	-35022	DLG4	3023
chr1	27433139	27433471	0.92	9.77E-01	SLC9A1	48146	TRNP1	113110
chr16	67906812	67907102	1.01	9.77E-01	EDC4	-42		
chr16	31085483	31085845	1.05	9.78E-01	ZNF668	-160	ZNF646	-79
chr14	88237520	88237838	0.77	9.78E-01	GALC	222228		
chr17	45727099	45727463	0.97	9.78E-01	KPNB1	6		
chr10	22292618	22292992	0.97	9.78E-01	DNAJC1	-155		
chr11	77531734	77532107	1.14	9.78E-01	RSF1	-41		
chr13	111367553	111367947	0.91	9.79E-01	ING1	391		
chr19	15236208	15236666	0.92	9.79E-01	ILVBL	140		
chr13	60971107	60971451	0.82	9.79E-01	TDRD3	-148		
chr11	809758	810086	1.00	9.79E-01	LRDD	-4677	RPLP2	-14

chr16	85969710	85970009	1.21	9.79E-01	FOXF1	-574273	IRF8	37086
chr3	56951633	56952039	1.17	9.79E-01	ERC2	-449445	ARHGEF3	161500
chr7	50477867	50478128	0.87	9.79E-01	FIGNL1	40090	IKZF1	133620
chr11	192054	192371	1.00	9.80E-01	ODF3	-4548	SCGB1C1	-867
chr15	91396075	91396414	1.11	9.80E-01	FURIN	-15640	BLM	135666
chr15	44092571	44092820	0.88	9.81E-01	SERINC4	-441		
chr5	151150847	151151268	1.08	9.81E-01	G3BP1	-418		
chr11	1330802	1331127	1.00	9.82E-01	TOLLIP	-126		
chr14	58711335	58711620	0.99	9.82E-01	PSMA3	-115		
chr15	101458231	101458560	0.90	9.82E-01	LRRK1	-1064		
chr17	58754633	58754939	0.99	9.82E-01	BCAS3	-386		
chr19	17666120	17666437	1.03	9.82E-01	GLT25D1	-232		
chr22	37252413	37252723	1.02	9.82E-01	NCF4	-4462		
chr17	8126124	8126429	1.15	9.82E-01	AURKB	-12394	C17orf68	25136
chr2	11889947	11890231	1.08	9.82E-01	TRIB2	-966909	LPIN1	3349
chr16	58426137	58426447	1.04	9.82E-01	GINS3	-6		
chr19	36705699	36706009	1.04	9.82E-01	ZNF565	-288	ZNF146	350
chr1	89149579	89150180	0.99	9.82E-01	PKN2	-42		
chr7	44645970	44646249	0.93	9.82E-01	OGDH	-61		
chr1	12100732	12101166	1.01	9.82E-01	TNFRSF8	-22485	MFN2	60711
chr11	67415539	67415880	1.06	9.82E-01	TBX10	-8679	ACY3	2420
chr6	15658650	15658926	0.95	9.82E-01	DTNBP1	4483	JARID2	412261
chr6	160210768	160211021	0.96	9.82E-01	MRPL18	-597	TCP1	-160
chr8	10190282	10190540	1.11	9.82E-01	UNQ9391	-192670	MSRA	278581
chr12	122442102	122442568	1.12	9.82E-01	BCL7A	-17526	PSMD9	115689
chr3	183968063	183968373	0.94	9.82E-01	ALG3	-1459	ECE2	773
chr19	42363577	42364077	1.13	9.83E-01	RPS19	-161		
chr6	108879707	108879983	0.91	9.83E-01	FOXO3	-2224		
chr7	141437861	141438239	1.13	9.83E-01	SSBP1	-126		
chr13	31039996	31040356	0.94	9.83E-01	HMGB1	-95		
chr17	37009752	37010218	0.99	9.83E-01	RPL23	68		
chr17	56416259	56416749	1.01	9.83E-01	BZRAP1	-10352	SUPT4H1	13059
chr13	20534216	20534571	1.11	9.83E-01	ZMYM2	1584	GJA3	200789
chr2	74685114	74685496	0.91	9.83E-01	WBP1	-272		
chr5	72793890	72794328	1.04	9.84E-01	BTF3	-141		
chr11	75863262	75863572	1.11	9.84E-01	WNT11	54157	UVRAG	337205
chr14	62547688	62548076	0.93	9.84E-01	SYT16	85341	KCNH5	964073
chr12	57118961	57119419	0.94	9.84E-01	NACA	136		

chr12	93323097	93323459	0.98	9.84E-01	EEA1	-171		
chr15	66678899	66679215	1.01	9.84E-01	MAP2K1	-154		
chr2	232328962	232329638	1.02	9.84E-01	NCL	-95		
chr21	34144149	34144461	1.05	9.84E-01	C21orf66	-136		
chr14	68733709	68734127	1.00	9.84E-01	RAD51L1	447409	ZFP36L1	525867
chr18	33196898	33197148	1.14	9.84E-01	INO80C	-119068	GALNT1	-37510
chr22	23881806	23882080	1.00	9.84E-01	IGLL1	40552	BCR	359391
chr3	39297092	39297425	1.21	9.84E-01	XIRP1	-63182	CX3CR1	24268
chr4	25876913	25877382	1.06	9.84E-01	C4orf52	-38666	SEL1L3	-12538
chr5	38441863	38442174	0.97	9.84E-01	LIFR	153488	EGFLAM	183486
chr13	52768448	52768762	0.97	9.84E-01	NEK3	-34609	THSD1	212024
chr11	73691113	73691449	1.00	9.84E-01	UCP2	2608	DNAJB13	29917
chr9	6412741	6413313	0.89	9.84E-01	UHRF2	-124		
chr7	99214357	99214667	1.03	9.84E-01	ZNF498	-59		
chr1	167905255	167905723	1.03	9.84E-01	DCAF6	-419	BRP44	789
chr6	1869144	1869437	1.13	9.84E-01	FOXC1	258610	GMDS	376555
chr1	160312969	160313261	0.94	9.84E-01	NCSTN	52	COPA	239
chr17	43238770	43239333	0.95	9.85E-01	HEXIM2	788		
chr8	94753132	94753440	0.93	9.85E-01	RBM12B	-62		
chr19	9250910	9251354	0.99	9.85E-01	ZNF317	46	KIAA1588	46
chr6	27099698	27099949	1.06	9.85E-01	HIST1H2AG	-993	HIST1H2BJ	751
chr12	111180777	111181132	0.90	9.85E-01	PPP1CC	-198		
chr7	148565422	148565693	1.09	9.85E-01	EZH2	15856	CUL1	169625
chr16	15859778	15860054	1.14	9.85E-01	MYH11	90971	NDE1	115833
chr1	101491239	101491538	0.98	9.85E-01	DPH5	-27		
chr2	234160015	234160444	0.93	9.85E-01	ATG16L1	13		
chr1	23903893	23904167	0.97	9.85E-01	RPL11	-114264	ID3	-17708
chr19	41903219	41903860	1.08	9.85E-01	EXOSC5	-284	BCKDHA	-164
chr13	54603317	54603680	1.13	9.85E-01				
chr7	97881355	97881713	0.85	9.85E-01	TECPR1	-66		
chr6	170151612	170151849	0.92	9.86E-01	TCTE3	-93	C6orf70	10
chr1	10532407	10532816	1.08	9.86E-01	PEX14	-2391	DFFA	1
chr11	189662	190295	1.01	9.86E-01	SCGB1C1	-3101		
chr14	107147634	107148024	1.15	9.86E-01	ADAM6	-709471	IGHV7-81	135454
chr20	16710438	16710751	1.14	9.86E-01	SNRPB2	-34		
chr5	141340295	141340605	0.94	9.86E-01	PCDH12	-1823		
chr17	39823241	39823551	1.04	9.86E-01	KRT17	-42514	EIF1	-21731
chr3	49059271	49059623	0.96	9.86E-01	DALRD3	-3430	NDUFAF3	373

chr6	27782680	27783117	0.88	9.86E-01	HIST1H2AJ	-381	HIST1H2BM	77
chr6	139483033	139483499	1.17	9.86E-01	HECA	27017	TXLNB	129942
chr3	50654160	50654436	0.97	9.86E-01	MAPKAPK3	-303		
chr14	70092139	70092474	0.87	9.86E-01	SFRS5	-141527	KIAA0247	13997
chr8	47829392	47829669	0.89	9.86E-01	CEBPD	821195		
chr9	123836880	123837249	0.97	9.87E-01	C5	-24511	CEP110	-13509
chr12	80328891	80329156	0.90	9.87E-01	PPP1R12A	-46		
chr16	89045048	89045428	1.01	9.87E-01	CBFA2T3	-1837		
chr17	41323112	41323566	1.07	9.87E-01	NBR1	93		
chr12	104680598	104680895	1.01	9.87E-01	EID3	-16802	TXNRD1	71188
chr12	104682514	104682792	1.06	9.87E-01	EID3	-14896	TXNRD1	73094
chr4	110911810	110912120	1.13	9.87E-01	EGF	77925	ELOVL6	207806
chr9	135258920	135259270	1.09	9.87E-01	SETX	-28723	TTF1	23126
chr17	66288116	66288449	0.99	9.88E-01	SLC16A6	-878		
chr2	54342687	54343203	1.04	9.88E-01	ACYP2	535		
chr12	120524693	120525347	0.88	9.88E-01	RAB35	11945	CCDC64	97372
chr12	1799988	1800244	0.91	9.88E-01	ADIPOR2	-131		
chr1	31222693	31222999	1.07	9.88E-01	MATN1	-26414	LAPTM5	7837
chr12	67662343	67662761	0.93	9.89E-01	CAND1	-509		
chr12	2921890	2922288	0.99	9.89E-01	ITFG2	226		
chr16	3550755	3551069	1.08	9.89E-01	CLUAP1	-51		
chr1	93297438	93297875	1.07	9.89E-01	RPL5	63		
chr1	24862936	24863341	1.11	9.89E-01	C1orf130	-19463	RCAN3	33752
chr17	78518429	78518847	1.06	9.89E-01	RPTOR	13		
chr6	46097436	46097754	0.85	9.89E-01	ENPP4	-106		
chr3	189042522	189042815	1.04	9.90E-01	TP63	-306547	TPRG1	152906
chr14	96130672	96131217	0.89	9.90E-01	TCL1A	49588	GLRX5	129622
chr10	101945738	101946029	0.96	9.90E-01	ERLIN1	-70		
chr3	33482223	33482769	1.09	9.90E-01	UBP1	-599		
chr11	73309237	73309606	1.03	9.90E-01	PLEKHB1	-49172	RELT	222017
chr3	112627148	112627437	1.36	9.93E-01	CD200R1L	-62496	CD200R1	66644
chr7	128116631	128116941	1.02	9.93E-01	METTL2B	3		
chr12	72057455	72058032	0.97	9.93E-01	THAP2	-40	ZFC3H1	5
chr7	32931638	32932230	0.94	9.94E-01	KBTBD2	-466		
chr2	172180773	172181017	1.07	9.94E-01	DCAF17	-109964	TLK1	-93071
chr19	1652637	1653372	1.00	9.94E-01	TCF3	-2719		
chr8	8567284	8567594	0.89	9.94E-01	CLDN23	7773	MFHAS1	183692
chr2	175468072	175468320	1.08	9.94E-01	GPR155	-116385	WIPF1	31111

chr1	40723675	40724111	0.95	9.95E-01	ZMPSTE24	160		
chr20	26189885	26190195	0.77	9.95E-01	ZNF337	-512571		
chr4	25235325	25235650	0.81	9.95E-01	PI4K2B	-165		
chr1	156577912	156578188	1.22	9.95E-01	HAPLN2	-11036	GPATCH4	-6780
chr15	56538162	56538542	1.03	9.95E-01	RFX7	-2869		
chr17	25572348	25572625	0.99	9.95E-01	WSB1	-48619		
chr19	2266599	2266850	0.88	9.95E-01	OAZ1	-2795		
chr9	35116883	35117151	0.97	9.95E-01	KIAA1539	-1124		
chr11	17229864	17230173	1.05	9.95E-01	NUCB2	-68267	PIK3C2A	-38666
chr19	8317933	8318209	0.96	9.95E-01	LASS4	43854	CD320	55168
chr11	64877807	64878077	1.02	9.95E-01	TM7SF2	-1399		
chr13	22245061	22245332	0.93	9.95E-01	FGF9	-18		
chr22	24823846	24824298	1.01	9.95E-01	ADORA2A	-3747	ADORA2A	542
chr12	54585359	54585640	1.02	9.95E-01	SMUG1	-2743		
chr19	18699444	18699701	1.06	9.95E-01	C19orf60	78		
chr11	695599	695964	0.95	9.95E-01	DEAF1	-42	TMEM80	166
chr17	16189291	16189598	1.07	9.95E-01	CENPV	67367	PIGL	68936
chr5	115412163	115412477	1.14	9.95E-01	COMMD10	-8407	LVRN	114169
chr8	6283827	6284163	1.10	9.95E-01	MCPH1	19874	ANGPT2	136789
chr9	71199370	71199714	1.01	9.95E-01	PIP5K1B	-121074	C9orf71	-43759
chr17	47865605	47866123	0.96	9.95E-01	MYST2	-207		
chr17	58677370	58677652	1.05	9.95E-01	PPM1D	-43		
chrX	146992984	146993515	0.94	9.95E-01	FMR1	-259		
chr1	1342560	1342854	0.90	9.95E-01	MRPL20	-14		
chr4	1209296	1209596	0.97	9.95E-01	SPON2	-42805	CTBP1	33462
chr19	2740063	2740319	0.91	9.96E-01	SLC39A3	-117		
chr12	6798676	6799121	1.04	9.96E-01	ZNF384	-223		
chr7	105752880	105753160	0.91	9.96E-01	SYPL1	37		
chr6	43673683	43674004	0.89	9.96E-01	VEGFA	-64109	MRPS18A	-18316
chr5	130545516	130545842	1.01	9.96E-01	CDC42SE2	-54023	LYRM7	39038
chr12	56521587	56522186	0.87	9.96E-01	ESYT1	-167		
chr17	45266439	45267002	0.90	9.96E-01	CDC27	-56		
chr17	74350126	74350578	1.01	9.96E-01	PRPSAP1	-122		
chr2	61698193	61698623	0.90	9.96E-01	USP34	-559		
chr10	11220681	11221005	1.02	9.96E-01	CUGBP2	160950	USP6NL	353431
chr13	45491836	45492304	1.11	9.96E-01	TSC22D1	-341369	NUFIP1	71543
chr22	40888266	40888645	1.00	9.96E-01	SGSM3	121861	MKL1	144234
chr7	105698492	105699182	1.00	9.96E-01	SYPL1	54220	FLJ23834	95180

chr9	98256688	98256983	0.94	9.96E-01	FANCC	-176845	PTCH1	13995
chr9	79087152	79087429	1.01	9.96E-01	GCNT1	13223	PRUNE2	433712
chr1	110090929	110091391	1.07	9.96E-01	GNAI3	-26		
chr15	93443335	93443707	1.05	9.96E-01	CHD2	-30		
chr19	55850409	55850819	0.89	9.96E-01	SUV420H2	-607		
chr16	30886578	30886847	1.04	9.96E-01	ZNF629	-88190	BCL7C	18686
chr4	4543649	4543948	0.98	9.96E-01	STX18	-24		
chr7	120627679	120628037	0.97	9.96E-01	C7orf58	-893		
chr14	50437879	50438194	1.04	9.96E-01	ARF6	78301	SOS2	260062
chr12	95224812	95225131	0.97	9.97E-01	TMCC3	-180648	NDUFA12	172539
chr8	42623726	42624021	1.26	9.97E-01	CHRNA6	-255		
chr12	109197324	109197625	1.03	9.97E-01	CORO1C	-72180	SSH1	53884
chr3	14728887	14729228	1.05	9.97E-01	FGD5	-131411	C3orf20	12404
chr1	180992024	180992374	1.06	9.97E-01	STX6	-153		
chr14	24583714	24584024	0.99	9.97E-01	DCAF11	-37		
chr3	194991786	194992062	0.96	9.97E-01	C3orf21	-29		
chr9	131218260	131218570	0.93	9.97E-01	ODF2	-17		
chr7	26904114	26904706	0.96	9.97E-01	SKAP2	-69		
chr19	4402304	4402751	1.04	9.97E-01	SH3GL1	-2057	CHAF1A	-132
chr2	47403479	47403834	1.04	9.98E-01	CALM2	83		
chr4	26585461	26585771	1.02	9.98E-01	TBC1D19	70		
chr7	102389099	102389476	1.04	9.98E-01	LRRC17	-164056	POLR2J2	-77106
chr12	34175189	34175643	0.90	9.98E-01	ALG10	200		
chr15	85874200	85874712	1.01	9.99E-01	AKAP13	-49415	PDE8A	349251
chr12	56497870	56498326	0.98	9.99E-01	PA2G4	-5		
chr1	17380325	17380834	1.06	1.00E+00	SDHB	85		
chr1	24741963	24742310	0.93	1.00E+00	NIPAL3	-108		
chr1	28969402	28969818	0.96	1.00E+00	TAF12	-6		
chr1	36851387	36851870	0.88	1.00E+00	STK40	-144		
chr1	40042455	40042726	0.91	1.00E+00	PABPC4	-70		
chr1	51700556	51701021	1.03	1.00E+00	RNF11	-1156		
chr1	110527046	110527383	0.94	1.00E+00	AHCYL1	-93		
chr1	227127589	227127996	0.99	1.00E+00	CABC1	-145		
chr11	10772556	10772907	1.05	1.00E+00	CTR9	-79		
chr14	105491217	105491461	1.03	1.00E+00	CDCA4	-3914		
chr15	65282099	65282538	0.97	1.00E+00	SPG21	-68		
chr16	89233822	89234170	1.14	1.00E+00	CDH15	-4167		
chr17	48943536	48943793	1.04	1.00E+00	TOB1	-2252		

chr18	11980931	11981243	0.81	1.00E+00	IMPA2	-368		
chr18	32923803	32924103	1.00	1.00E+00	ZNF24	473		
chr19	11457002	11457376	0.99	1.00E+00	TMEM205	-208		
chr19	39340829	39341133	0.96	1.00E+00	HNRNPL	-364		
chr2	98612689	98612999	1.01	1.00E+00	TMEM131	-490		
chr21	36421473	36421930	1.06	1.00E+00	RUNX1	-107		
chr3	119813699	119814076	1.01	1.00E+00	GSK3B	-624		
chr5	87564585	87564839	1.12	1.00E+00	TMEM161B	-47		
chr8	52438673	52438917	1.10	1.00E+00	PXDNL	283210		
chr9	35603900	35604443	0.96	1.00E+00	TESK1	-1109		
chr9	35657687	35658045	1.15	1.00E+00	CCDC107	-445		
chr9	98638115	98638504	1.06	1.00E+00	C9orf102	410		
chr1	10003359	10003701	0.95	1.00E+00	LZIC	-690	NMNAT1	44
chr1	21844491	21844870	1.02	1.00E+00	ALPL	8823	RAP1GAP	133667
chr1	154832145	154832564	1.13	1.00E+00	ADAR	-251673	KCNN3	10399
chr1	155196392	155196689	0.95	1.00E+00	GBA	14512	MTX1	18051
chr10	27015073	27015383	1.13	1.00E+00	PDSS1	28633	ABI1	134731
chr10	90595461	90595715	0.90	1.00E+00	STAMBPL1	-44438	LIPM	33101
chr11	537194	537465	0.88	1.00E+00	HRAS	-1780	LRRC56	-192
chr11	18417613	18417891	1.08	1.00E+00	LDHC	-16101	LDHA	1816
chr12	31556135	31556420	1.10	1.00E+00	OVOS1	-197190	DENND5B	187674
chr12	31789580	31789917	1.11	1.00E+00	DENND5B	-45797	C12orf72	-22860
chr12	42863075	42863573	0.99	1.00E+00	PRICKLE1	14092	PPHLN1	143377
chr12	113488601	113488939	0.96	1.00E+00	DTX1	-6892	OAS2	72496
chr12	125412281	125412601	1.07	1.00E+00	UBC	-12864	DHX37	61226
chr12	131464385	131464733	0.98	1.00E+00	SFRS8	-731076	GPR133	26107
chr13	28033668	28033946	0.86	1.00E+00	MTIF3	-9096	LNX2	160913
chr13	41495697	41496028	1.11	1.00E+00	ELF1	97645	SLC25A15	132316
chr14	22392757	22393045	0.75	1.00E+00	TRA	-78444	TRA@	30331
chr14	64108129	64108922	1.01	1.00E+00	PPP2R5E	-98447	SGPP1	86230
chr14	104181770	104182030	0.84	1.00E+00	ZFYVE21	-245	XRCC3	-77
chr15	85947413	85947671	1.13	1.00E+00	AKAP13	23671	KLHL25	390647
chr16	81842614	81843074	1.01	1.00E+00	PLCG2	29914	SDR42E1	202249
chr17	3864769	3865117	1.00	1.00E+00	P2RX1	-44983	ATP2A3	2793
chr17	42428173	42428449	0.96	1.00E+00	GRN	5820	FAM171A2	12924
chr18	23770494	23770809	1.01	1.00E+00	TAF4B	-35757	PSMA8	56836
chr19	1087812	1088261	1.00	1.00E+00	POLR2E	7354	HMHA1	20863
chr19	54296220	54296530	0.97	1.00E+00	NLRP12	31273	DPRX	161065

chr2	178128166	178128461	1.05	1.00E+00	NFE2L2	1545	HNRNPA3	50892
chr20	37075062	37075338	1.04	1.00E+00	RALGAPB	-26286	LBP	100315
chr20	55043451	55043852	0.97	1.00E+00	CASS4	56338	GCNT7	57329
chr22	41799825	41800135	0.99	1.00E+00	TEF	22017	TOB2	43047
chr6	27447354	27447693	1.12	1.00E+00	ZNF184	-6627	HIST1H2BL	328185
chr6	137084858	137085332	1.34	1.00E+00	MAP7	-213303	MAP3K5	28561
chr6	151302256	151302562	1.00	1.00E+00	AKAP12	-258725	MTHFD1L	115718
chr7	106358704	106358962	1.16	1.00E+00	NAMPT	-433195	PIK3CG	-147091
chr7	128731604	128731853	0.90	1.00E+00	TSPAN33	-52983	TNPO3	-36531
chr8	144363677	144364050	1.20	1.00E+00	ZNF696	-9695	GLI4	14257
chr9	107537686	107538068	1.08	1.00E+00	NIPSNAP3B	11426	ABCA1	152559
chr9	117501179	117501488	0.93	1.00E+00	TNFSF15	67074	C9orf91	127628
