# Supplementary material for: STAT3 Targets Suggest Mechanisms of Aggressive Tumorigenesis in Diffuse Large B-Cell Lymphoma
Source: G3 (Bethesda). 2013 Oct 18;3(12):2173–85. doi: 10.1534/g3.113.007674 (PMC3852380; doi:10.1534/g3.113.007674)
Supplement: Supporting Information [file supp_g3.113.007674_FigureS1.pdf]

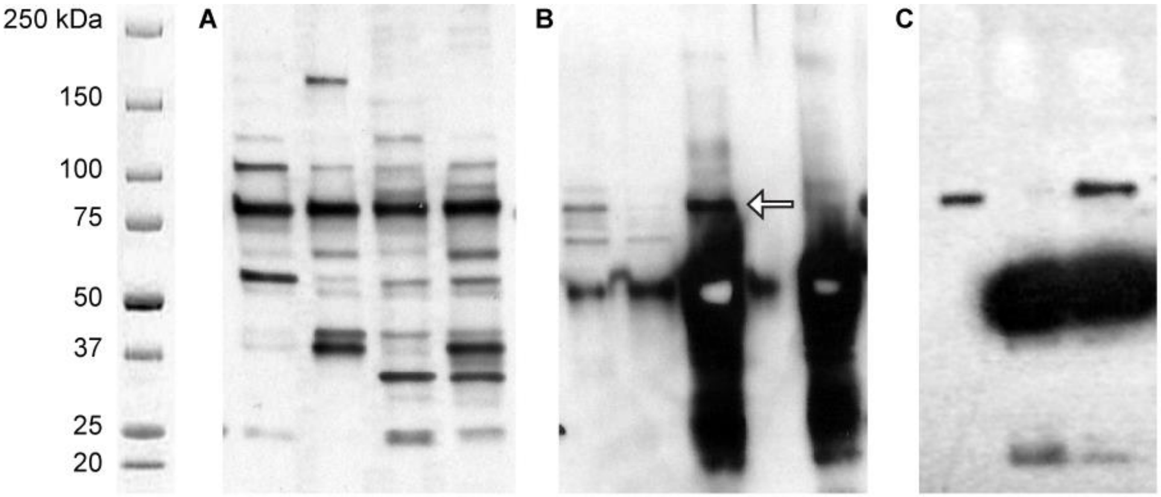

Figure S1 STAT3 immunoblotting and immunoprecipitation with sc-482. Western blot and IPs show a band consistent with expected size (88 kDa) of STAT3. (A) Western blot using antibody sc-482 versus nuclear lysates. Lanes contain (from left to right) lysate from K562 cells, GM12878 cells, HeLa S3 cells, and HepG2 cells. (B) IP of STAT3 using sc-482 in HeLa S3 cells. *Lane 1:* input nuclear lysate; *lane 2:* unbound material from IP with sc-482; *lane 3:* material IP'd with sc-482; *lane 4:* material IP'd using control rabbit IgG. Arrow indicates the band of interest. (C) IP of STAT3 using sc-482 in K562 cells. *Lane 1:* input nuclear lysate; *lane 2:* material IP'd using control rabbit IgG; *lane 3:* material IP'd with sc-482.
