# Supplementary material for: STAT3 Targets Suggest Mechanisms of Aggressive Tumorigenesis in Diffuse Large B-Cell Lymphoma
Source: G3 (Bethesda). 2013 Oct 18;3(12):2173–85. doi: 10.1534/g3.113.007674 (PMC3852380; doi:10.1534/g3.113.007674)
Supplement: Supporting Information [file supp_g3.113.007674_FigureS2.pdf]

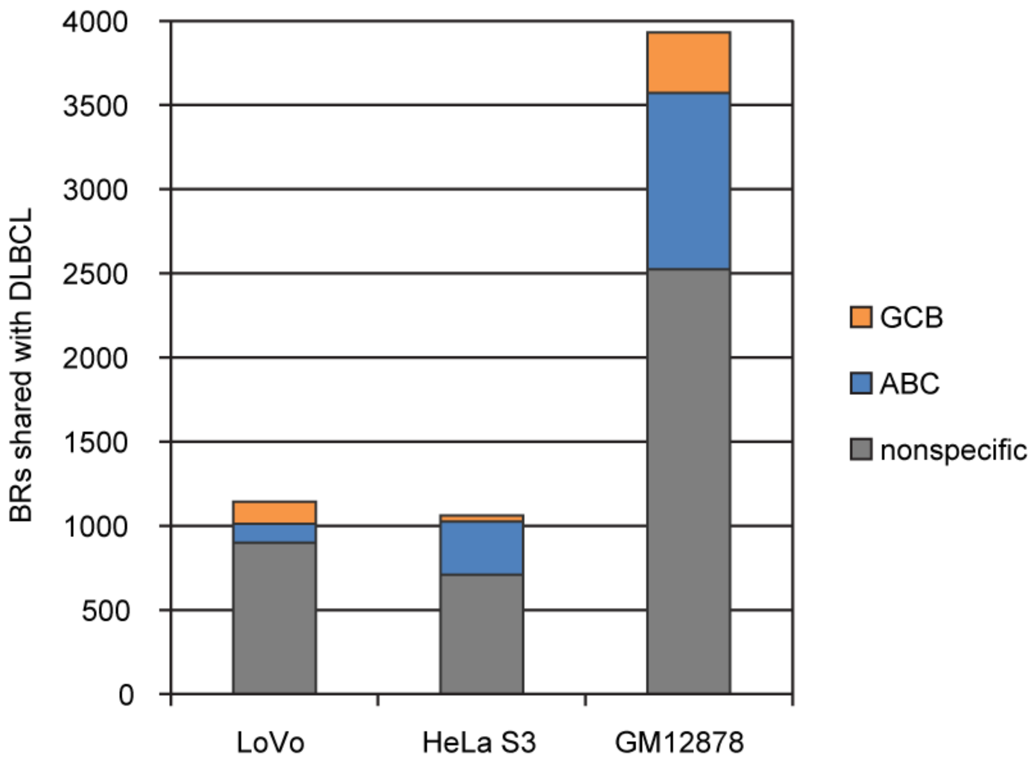

Figure S2 DLBCL STAT3 binding regions shared with other cell lines. 10,337 DLBCL high-confidence STAT3 binding regions (BRs) were compared to STAT3 BR lists generated by other studies in the cell lines LoVo, HeLa S3, and GM12878. Orange bars represent BRs that are preferentially bound by STAT3 in the GCB subtype of DLBCL; blue bars represent BRs preferentially bound in the ABC subtype of DLBLC; gray bars represent BRs that show no DLBCL subtype binding bias.
