# Supplementary material for: STAT3 Targets Suggest Mechanisms of Aggressive Tumorigenesis in Diffuse Large B-Cell Lymphoma
Source: G3 (Bethesda). 2013 Oct 18;3(12):2173–85. doi: 10.1534/g3.113.007674 (PMC3852380; doi:10.1534/g3.113.007674)
Supplement: Supporting Information [file supp_g3.113.007674_FigureS3.pdf]

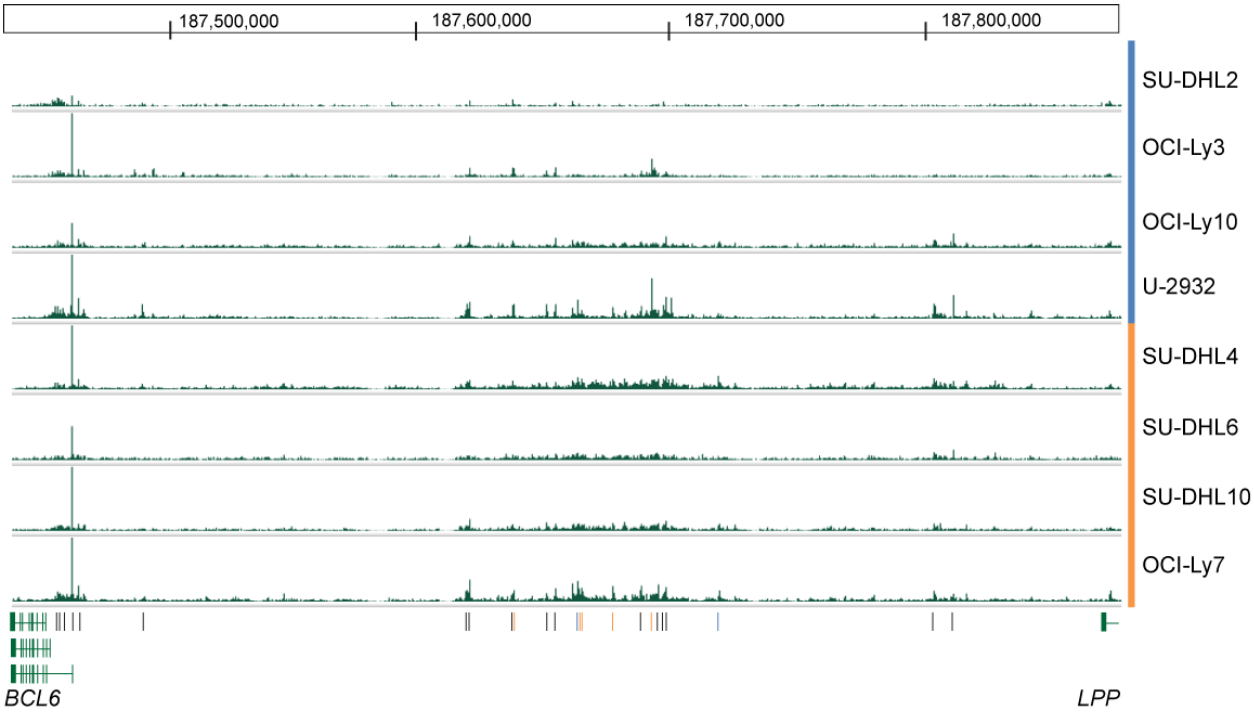

Figure S3 The *BCL6* gene locus with STAT3 ChIP-seq peaks. All 24 STAT3 BRs are shown within the upstream putative regulatory region of *BCL6* (chr3: 187,436,000-187,876,700). Orange bars represent loci with enriched STAT3 binding in the GCB subtype at FDR < 0.05 (*n* = 5); blue bars represent loci with enriched STAT3 binding in the ABC subtype (*n* = 2); dark gray bars represent peaks with no statistically significant difference in binding between the two subtypes (*n* = 17). *BL6* is encoded on the minus strand at left, and *LPP* encoded on the plus strand at right (only TSS shown).
