# Supplementary material for: STAT3 Targets Suggest Mechanisms of Aggressive Tumorigenesis in Diffuse Large B-Cell Lymphoma
Source: G3 (Bethesda). 2013 Oct 18;3(12):2173–85. doi: 10.1534/g3.113.007674 (PMC3852380; doi:10.1534/g3.113.007674)
Supplement: Supporting Information [file supp_g3.113.007674_FileS1.pdf]

File S1

EXTENDED MATERIALS AND METHODS

Cell culture: OCI-Ly7, SU-DHL4, SU-DHL6, SU-DHL10, and U-2932 cell lines were purchased from Deutsche Sammlung von Mikroorganismen und Zellkulturen (DSMZ). OCI-Ly3, OCI-Ly10, and SU-DHL2 cells were generously donated by Dr. Louis Staudt's laboratory at the NIH National Cancer Institute. Cell lines OCI-Ly7, SU-DHL2, SU-DHL4, SU-DHL6, SU-DHL10, and U-2932 were cultured in suspension in RPMI 1640 (Gibco-Invitrogen) containing L-glutamine and HEPES, supplemented with heat-inactivated fetal bovine serum (15%) and antibiotics (Penicillin Streptomycin, Gibco-Invitrogen), at 37° and 5% CO_2. Cell lines OCI-Ly3 and OCI-Ly10 were cultured in suspension in IMDM (Gibco-Invitrogen) containing L-glutamine and HEPES, supplemented with heat-inactivated fetal bovine serum (15%), beta-mercaptoethanol (55 μM, Gibco-Invitrogen), and antibiotics (Penicillin Streptomycin, Gibco-Invitrogen), at 37° and 5% CO_2.

Western blotting: Cells were washed with PBS, then lysed for 30 minutes on ice in cold RIPA buffer containing 1 mM DTT, 0.5 mM PMSF, protease inhibitors (Complete Protease Inhibitor Cocktail Tablets, Roche), and phosphatase inhibitors (Phosphatase Inhibitor Cocktail 2 and 3, Sigma Aldrich). Cell debris was removed by centrifugation. Protein concentration of the resulting whole cell lysate was measured by Bradford assay (Precision Assay solution, BioRad).

Whole cell lysate was mixed with an equal volume of 2x Laemmli SDS loading buffer and incubated at 70° for 15 minutes to denature the proteins. Then, equal amounts of protein were loaded into each lane of a 4-20% SDS-PAGE gel (Mini-PROTEAN TGX Precast Gel, BioRad), which was run at 100 volts for 1 hour (running buffer: 25 mM Tris, 192 mM glycine, 0.1% SDS). The gel was transferred to a PVDF membrane (transfer buffer: 25 mM Tris, 192 mM glycine, 20% methanol, 0.1% SDS) at 250 mAmps for 1 hour. The membrane was then sliced horizontally at the 50 kDa marker to separate the upper portion containing STAT3 protein (88 kDa) from that containing the GAPDH loading control (40 kDa).

PVDF membranes were blocked in 5% BSA at 4° overnight on an orbital shaker. Blots were then probed using the SNAP i.d. 2.0 system (Millipore). Primary antibodies were diluted in 0.5% BSA in PBS-T (0.1% Tween): anti-STAT3 antibody sc-482X and anti-pSTAT3-Y705 antibody sc-8059X (Santa Cruz Biotechnology) were diluted 1:200; anti-GAPDH antibody ab8245 (Abcam) was diluted 1:5000. The membranes were incubated with primary antibody for 20 minutes at room temperature, then washed three times with PBS-T. HRP-conjugated secondary antibodies were diluted in 0.5% BSA in PBS-T: goat anti-rabbit was diluted

1:10,000; rabbit anti-mouse was diluted 1:8000. Blots were incubated with secondary antibody for 10 minutes at room temperature, then washed three times with PBS-T.

Blots were treated with enhanced chemiluminescent (ECL) substrate in a ratio of 70:30 pico to femto (SuperSignal West Chemiluminescent Substrate, Pierce) and visualized using BioMax Maximum Resolution film (Carestream). Fold change was measured by densitometric quanitification using NIH ImageJ software, normalized to the GAPDH loading control (Schneider *et al.* 2012).

Chromatin immunoprecipitation (ChIP): Cells were suspended in their growing media at $1x10^6$ per mL and crosslinked with formaldehyde at a final concentration of 1% for 10 minutes at room temperature, followed by the addition of glycine in PBS at a final concentration of 125 mM for 5 minutes. Cells were collected by centrifugation, washed twice in PBS, then snap frozen in liquid nitrogen and stored at -80° in aliquots of $1x10^8$ cells.

Frozen aliquots were thawed in 5 mL of cold PBS plus protease inhibitors (Complete Protease Inhibitor Cocktail Tablets, Roche) at 4° for 30 minutes with rotation. Cells were collected by centrifugation, then swollen on ice for 10 minutes in a hypotonic buffer (20 mM HEPES, pH 7.9; 10 mM KCl; 1 mM EDTA, pH 8.0; 10% glycerol; 1 mM DTT; 0.5 mM PMSF; and protease inhibitors). Cells were broken by dounce homogenization, and the nuclei were pelleted by mild centrifugation. The nuclei were washed one with hypotonic buffer, then lysed in RIPA buffer (10 mM Tris-Cl, pH 8.0; 140 mM NaCl; 0.025% sodium azide; 1% Triton X-100; 0.1% SDS; 1% deoxycholic acid; 0.5 mM PMSF; 1 mM DTT; 0.1 mM sodium orthovanadate; and protease inhibitors) on ice for at least 30 minutes with repeated vortexing. The nuclear lysate was sonicated with a Branson 250 Sonifier to shear the chromatin (output 20%, 100% duty cycle, 12 20-second pulses each followed by a two minute rest on ice). The samples were clarified by centrifugation at 14,000 rpm at 4° for 15 minutes. 200 μL was removed from each sample to act as a pre-immunoprecipitation genomic DNA control ("input" control) and stored at 4°. Then STAT3-DNA complexes were immunoprecipitated with an anti-STAT3 C-terminal antibody (sc-482X, Santa Cruz Biotechnology) for 16 hours at 4° on a multimix rotator (VWR International).

Each immunoprecipitated sample was incubated with protein A-agarose beads (Upstate Biotechnology) for 2 hours at 4° on a multimix rotator, followed by three washes with RIPA buffer and one wash with PBS. The antibody-STAT3-DNA complexes were eluted from the beads by addition of 1% SDS in TE (10 mM Tris-Cl, pH 7.6; 1 mM EDTA, pH 8.0) and incubation at 65° for 16 minutes with repeated vortexing. The beads were removed by centrifugation and the supernatant reserved. The beads were

resuspended in 0.67% SDS in TE and incubated at 65° for a further 16 minutes with repeated vortexing. The beads were removed by centrifugation and the supernatant combined with that from the 1% SDS elution. The input DNA control aliquots were removed from 4° storage and 300 uL of 1% SDS in TE was added. Both the input controls and the bead elutions were incubated at 65° overnight to reverse the crosslinking. To purify the DNA, RNase A was added to a final concentration of 200 μg/mL, and the samples were incubated at 37° for 30 minutes. Proteinase K was added to a final concentration of 200 μg/mL, and the samples were incubated at 45° for 30 minutes. This was followed by an ethanol precipitation and DNA purification column (Qiagen) to recover the DNA.

To test the efficacy of the IP, ChIP'd DNA was amplified using primers against an empirical STAT3 binding site in its own promoter region, coordinates chr17: 40,540,721-40,540,905 (build hg19). An annealing temperature of 60° and 30 PCR cycles were used. Primer sequence:

Left primer: 5'-GCTGGCTGTTCCGACAGT-3'

Right primer: 5'-CAGGAGGGAGCTGTATCAGG-3'

mRNA isolation for RNA-Seq: An aliquot of 4×10^6 cells were washed once with PBS and lysed. The lysate was incubated with magnetic poly-dT beads (Dynabeads mRNA DIRECT Kit, Invitrogen) to directly isolate poly-A RNA using the "standard" wash volumes. Beads were serially washed in Washing Buffer A (10 mM Tris-HCl, pH 7.5; 0.15 M LiCl; 1 mM EDTA; 0.1% LiDS) and Washing Buffer B (10 mM Tris-HCl, pH 7.5; 0.15 M LiCl; 1 mM EDTA; 10 mM Tris-HCl). The mRNA was eluted from the beads using 10 mM Tris-HCl, pH 7.5.

To reduce contamination by ribosomal RNA, the purified poly-A mRNA was then bound to the same beads, washed, and eluted a second time. After the second purification, it was fragmented to approximately 300 bp in length (RNA Fragmentation Reagents, Ambion), treated with DNase I, and purified via Qiagen's RNeasy MinElute Cleanup kit.

As the kit did not supply enough Buffer B to accommodate all optional additional wash steps, extra buffer was prepared based on the ingredients listed in the kit instructions. There was no difference between samples prepared using the pre-made kit buffer versus those prepared using homemade buffer.

cDNA synthesis: Double-stranded cDNA was synthesized from the isolated mRNA using the Invitrogen SuperScript Double-Stranded cDNA Labeling Kit. mRNA was incubated with random hexamer primers at 70° for 10 minutes, then incubated at 45° for 1 hour with SuperScript II RT to synthesize the first strand of cDNA. RNase H was added to degrade the existing RNA, and then the second strand of cDNA was synthesized in the presence of DNA Polymerase I and DNA Ligase at 16° for 2 hours. The final product was purified using Qiagen's QIAquick PCR Purification kit.

DNA library preparation for sequencing: Sheared DNA was converted to blunt-ended fragments (End-It Repair Kit, Epicenter-Illumina). Then a single overhanging dATP was added via a Klenow fragment, and Illumina adapters were ligated onto the ends. Adapter sequence:

Left primer (1.1): 5'-AATGATACGGCGACCACCGAGATCTACACTCTTTCCCTACACGAC-3'

Right primer (2.1): 5'-CAAGCAGAAGAGGGCATACGAGCTCTTCCGATCT-3'

To remove excess non-ligated adapters, the DNA was gel purified using a 2% agarose gel with a size selection from 150-300 base pairs. Then the DNA was PCR amplified using Illumina primers and Phusion high-fidelity DNA polymerase master mix (New England BioLabs) under the following reaction conditions: 98° for 30 seconds; 15 cycles of 98° for 10 seconds, 65° for 30 seconds, 72° for 30 seconds; and 72° for 5 minutes. The PCR product was gel purified again using a 2% agarose gel with a size selection from 150-300 base pairs.

ChIP-Seq peak calling & replicate analysis: We used the Irreproducible Discovery Rate (IDR) framework (Li *et al.* 2011) with the SPP peak caller (Kharchenko *et al.* 2008) in order to identify high confidence and reliable regions of enrichments (peaks) in the ChIP-seq datasets. Specifically, we followed the ENCODE uniform processing pipeline as outlined at http://anshul.kundaje.net/projects/idr. Briefly, the SPP peak caller was used with a relaxed peak calling threshold (FDR = 0.9) to obtain a large number of peaks (maximum of 300,000) that span true signal as well as noise (false identifications). Peaks in each replicate were ranked based on their SPP signal score. The IDR method analyzes ranked peak calls for a pair of replicates, and considers peaks that are present in both replicates to belong to one of two populations: a reproducible signal group or an irreproducible noise group. The method adaptively learns the degree of peak-rank consistency in the signal component and the proportion of peaks belonging to each component. The model can then be used to infer an IDR score for every peak that is

found in both replicates. The IDR score of a peak represents the expected probability that the peak belongs to the noise component, and is based on its ranks in the two replicates. Hence, low IDR scores represent high-confidence peaks. The number of peaks with IDR scores better than 0.02 (2%) was used as the cross-replicate peak rank threshold. If a dataset had more than two replicates, all pairs of replicates were analyzed using the IDR method. The maximum peak rank threshold across all pairwise analyses was used as the final cross-replicate peak rank threshold. Any thresholds based on reproducibility of peak calling between biological replicates are bounded by the quality and enrichment of the worst replicate.

In cases where a cell line dataset had at least one replicate that was significantly worse in data quality than another replicate, a rescue pipeline was used in order to balance data quality between the set of replicates. Mapped reads were pooled across all replicates of a dataset, and then randomly sampled (without replacement) to generate two pseudo-replicates with equal numbers of reads. This sampling strategy tends to transfer signal from stronger replicates to the weaker replicates, thereby balancing cross-replicate data quality and sequencing depth. SPP was used to with relaxed thresholds (FDR 0.9) to call peaks on these pseudo-replicates. The peaks were ranked by SPP signal score. The two sets of ranked peaks from the pseudo-replicates were then compared using IDR and the number of peaks passing an IDR threshold of 0.0025 (0.25%) was used as a rescue peak rank threshold. Ultimately, for each dataset, the best of the cross-replicate and rescue peak rank thresholds were used to obtain an optimal peak rank threshold. Finally, mapped reads from all replicate datasets were pooled and SPP was once again used to call peaks on the pooled data with a relaxed FDR of 0.9. These peaks on the pooled data were ranked by the SPP signal-score. The optimal peak rank threshold (N) was then used on select the top N peaks ranked set of pooled-data peaks.

Once the characteristic high confidence STAT3 binding sites were identified for each cell line with IDR, all the peak lists were combined. Any overlapping or abutting peaks were merged into broader “binding regions” (BRs) to facilitate comparison between cell lines (as in Kasowski *et al.* 2010). Binding regions that occurred in only one cell line were eliminated from further analysis. The ChIP-Seq data for each line was then rescored to determine how many fragments mapped to each BR.

Next, the ChIP-Seq datasets were normalized using DESeq (Anders and Huber 2010). The effective library size of each sample was estimated based on the pooled count data. First, a reference sample was defined in which the reference count of each BR is its geometric mean over all samples. Second, for each sample, a vector was calculated as the ratios of the read counts over the reference counts for all the BRs. Third, the median of these ratios across all the BRs was defined as the “size factor” of each sample. Lastly, each sample was normalized by dividing the real counts by its size factor.

We then compared the four ABC cell lines to the four GCB cell lines to identity BRs that were more strongly occupied in one subtype of DLBCL. We compared 24 GCB replicates versus 11 ABC replicates. To assess the significance levels of differential

STAT3 binding in ChIP-Seq data, we applied the negative binomial model in DESeq. The dispersion parameter in the model is estimated from the data by examining the relationship between the mean and variance of read counts across all the BRs. To contrast two conditions, we used the parameterized negative binomial model for each gene and obtained the p-values. To correct for multiple comparison, we adjusted the p-values with the Benjamini-Hochberg procedure which controls false discovery rate (BENJAMINI and HOCHBERG 1995).

RNA-Seq replicate analysis: Single-ended 36 bp reads were generated and the raw reads were mapped to the human genome (build hg19) using Bowtie (LANGMEAD *et al.* 2009). The top two best mappings were kept, tolerating two or fewer mismatched bases per read. Then a custom script was used to select the better of these two unique mappings. We also filtered reads that mapped to human ribosomal RNAs. The positions of the filtered reads were intersected with the coordinates of 26,322 annotated RefSeq genes, and the read count for each gene was generated. The RNA-Seq datasets were then normalized similarly to the ChIP-Seq data using DESeq (ANDERS and HUBER 2010).

Two biological replicates were performed for each line, and their normalized read counts were compared on a gene by gene basis. We then compared the four ABC cell lines to the four GCB cell lines to identity genes that were more highly expressed in one subtype of DLBCL. Because of the high degree of similarity between RNA-Seq replicates, we chose to collapse the replicates by summing the read counts of two replicates for each cell line, then compare the four GCB lines versus four ABC lines. To assess the significance levels of differential gene expression in RNA-Seq data, we applied the negative binomial model in DESeq (ANDERS and HUBER 2010). The dispersion parameter in the model is estimated from the data by examining the relationship between the mean and variance of read counts across all the genes. To contrast two conditions, we used the parameterized negative binomial model for each gene and obtained the p-values. To correct for multiple comparison, we adjusted the p-values with the Benjamini-Hochberg procedure which controls false discovery rate (BENJAMINI and HOCHBERG 1995).

ChIP-Seq gene associations: ChIP-Seq binding regions (BRs) were associated with genes that they might regulate via the Genomic Regions Enrichment of Annotations Tool (GREAT, MCLEAN *et al.* 2010). To determine whether a BR and a given gene are linked, GREAT first determines a putative regulatory domain for every gene, which consists of a basal domain plus an extension. The basal regulatory domain (BRD) consists of the region spanning 5 kb upstream to 1 kb downstream of the gene's

transcription start site (TSS), regardless of overlapping nearby genes. The gene's extended regulatory domain (ERD) is then calculated by elongating the BRD both upstream and downstream for 1000 kb or until reaching another gene's BRD, whichever occurs first.

Once this set of extended regulatory domains is established, GREAT associates the list of ChIP-Seq BRs with all of the genes whose regulatory domains they overlap. In many cases, BRs fall in regions where two regulatory domains overlap; consequently, GREAT associates these BRs with both genes. For purposes of downstream comparison, we treated these multiple associations as separate database entries, to allow the greatest sensitivity in detecting associations with gene expression data. We then compared this list of genes associated with significant changes in STAT3 binding to the list of genes with significant expression changes using the online tool Galaxy (Giardine *et al.* 2005; Blankenberg *et al.* 2010; Goecks *et al.* 2010).

REFERENCES FOR EXTENDED METHODS

Anders, S., and W. Huber, 2010 Differential expression analysis for sequence count data. Genome Biol 11**:** R106.

Benjamini, Y., and Y. Hochberg, 1995 Controlling the false discovery rate: a practical and powerful approach to multiple testing. Journal of the Royal Statistical Society Series B (Methodological) 57**:** 289-300.

Blankenberg, D., G. Von Kuster, N. Coraor, G. Ananda, R. Lazarus *et al.*, 2010 Galaxy: a web-based genome analysis tool for experimentalists. Curr Protoc Mol Biol Chapter 19**:** Unit 19 10 11-21.

Giardine, B., C. Riemer, R. C. Hardison, R. Burhans, L. Elnitski *et al.*, 2005 Galaxy: a platform for interactive large-scale genome analysis. Genome Res 15**:** 1451-1455.

Goecks, J., A. Nekrutenko and J. Taylor, 2010 Galaxy: a comprehensive approach for supporting accessible, reproducible, and transparent computational research in the life sciences. Genome Biol 11**:** R86.

Kasowski, M., F. Grubert, C. Heffelfinger, M. Hariharan, A. Asabere *et al.*, 2010 Variation in transcription factor binding among humans. Science 328**:** 232-235.

Kharchenko, P. V., M. Y. Tolstorukov and P. J. Park, 2008 Design and analysis of ChIP-seq experiments for DNA-binding proteins. Nat Biotechnol 26**:** 1351-1359.

Langmead, B., C. Trapnell, M. Pop and S. L. Salzberg, 2009 Ultrafast and memory-efficient alignment of short DNA sequences to the human genome. Genome Biol 10**:** R25.

Li, Q., J. B. Brown, H. Huang and P. J. Bickel, 2011 Measuring reproducibility of high-throughput experiments. Annals of Applied Statistics 5**:** 1752-1779.

McLean, C. Y., D. Bristor, M. Hiller, S. L. Clarke, B. T. Schaar *et al.*, 2010 GREAT improves functional interpretation of cis-regulatory regions. Nat Biotechnol 28**:** 495-501.

Schneider, C. A., W. S. Rasband and K. W. Eliceiri, 2012 NIH Image to ImageJ: 25 years of image analysis. Nat Methods 9**:** 671-675.
