# Supplementary material for: STAT3 Targets Suggest Mechanisms of Aggressive Tumorigenesis in Diffuse Large B-Cell Lymphoma
Source: G3 (Bethesda). 2013 Oct 18;3(12):2173–85. doi: 10.1534/g3.113.007674 (PMC3852380; doi:10.1534/g3.113.007674)
Supplement: Supporting Information [file supp_g3.113.007674_TableS1.pdf]

Table S1 ChIP-Seq sequencing statistics for all replicates

Cell line	Replicate	Total reads	Mapped reads	Percent mapping
**ABC subtype**				
SU-DHL2	1	13,512,260	12,288,255	90.94%
	2	13,362,953	12,424,630	92.98%
OCI-Ly3	1	13,064,810	10,257,726	78.51%
	2	18,089,134	16,571,735	91.61%
	3	18,897,921	17,974,794	95.12%
	4	24,373,222	22,970,681	94.25%
OCI-Ly10	1	37,395,608	33,290,027	89.02%
	2	44,875,156	37,749,797	84.12%
U-2932	1	16,507,416	15,223,231	92.22%
	2	17,272,908	16,148,051	93.49%
	3	18,302,385	14,800,735	80.87%
**GCB subtype**				
SU-DHL4	1	20,378,155	17,826,440	87.48%
	2	20,023,227	18,458,102	92.18%
	3	19,006,085	17,222,037	90.61%
	4	6,186,894	4,950,382	80.01%
	5	26,300,058	21,093,459	80.20%
	6	26,764,265	23,361,452	87.29%
	7	12,400,324	10,817,576	87.24%
	8	22,072,217	20,667,096	93.63%
	9	1,217,646	597,943	49.11%
SU-DHL6	1	10,546,526	8,980,307	85.15%
	2	18,604,899	14,101,371	75.79%
	3	31,175,126	28,350,143	90.94%
	4	21,166,960	19,002,685	89.78%
	5	20,685,171	18,677,393	90.29%
	6	16,917,103	15,262,894	90.22%
	7	16,835,815	15,109,722	89.75%
	8	18,277,246	15,785,997	86.37%
SU-DHL10	1	28,470,759	26,816,509	94.19%
	2	27,785,404	26,145,200	94.10%
	3	16,827,217	15,260,782	90.69%
	4	22,196,262	21,051,264	94.84%
OCI-Ly7	1	25,139,778	23,903,936	95.08%
	2	21,659,272	20,741,470	95.76%
	3	17,519,050	16,590,574	94.70%
