# Supplementary material for: STAT3 Targets Suggest Mechanisms of Aggressive Tumorigenesis in Diffuse Large B-Cell Lymphoma
Source: G3 (Bethesda). 2013 Oct 18;3(12):2173–85. doi: 10.1534/g3.113.007674 (PMC3852380; doi:10.1534/g3.113.007674)
Supplement: Supporting Information [file supp_g3.113.007674_TableS2.pdf]

Table S2 RNA-Seq sequencing statistics for all replicates

Cell line	Replicate	Total reads	Mapped reads	Percent mapping
**ABC subtype**				
SU-DHL2	1	28,204,354	24,615,267	87.27%
	2	25,329,137	21,547,373	85.07%
OCI-Ly3	1	30,939,496	24,755,240	80.01%
	2	28,502,235	21,678,611	76.06%
OCI-Ly10	1	32,056,465	28,628,279	89.31%
	2	29,305,171	26,100,549	89.06%
U-2932	1	17,600,699	14,810,651	84.15%
	2	15,868,465	13,051,886	82.25%
**GCB subtype**				
SU-DHL4	1	18,071,332	15,607,255	86.36%
	2	18,930,836	16,772,051	88.60%
SU-DHL6	1	29,374,231	24,974,330	85.02%
	2	33,740,415	28,752,594	85.22%
SU-DHL10	1	27,520,478	24,673,323	89.65%
	2	28,931,874	25,673,326	88.74%
OCI-Ly7	1	26,992,682	23,664,656	87.67%
	2	27,520,987	24,460,363	88.88%
