# Supplementary material for: STAT3 Targets Suggest Mechanisms of Aggressive Tumorigenesis in Diffuse Large B-Cell Lymphoma
Source: G3 (Bethesda). 2013 Oct 18;3(12):2173–85. doi: 10.1534/g3.113.007674 (PMC3852380; doi:10.1534/g3.113.007674)
Supplement: Supporting Information [file supp_g3.113.007674_TableS3.pdf]

Table S3   BCL6-associated STAT3 binding regions

Coordinates	Relative to TSS	GCB mean	ABC mean	Fold change	FDR
**Proximal to TSS**					
chr3: 187465839-187466149	-2519	23.60	29.70	1.26	6.34E-01
chr3: 187463162-187463620	+84	269.83	217.87	0.81	6.49E-01
chr3: 187459876-187460179	+3447	17.18	21.34	1.24	3.08E-01
chr3: 187457782-187458196	+5486	20.85	25.02	1.20	5.76E-01
chr3: 187456628-187456917	+6702	13.52	21.93	1.62	8.55E-02
**Differential STAT3 binding, FDR < 0.05**					
chr3: 187718164-187718648	-254931	21.57	8.52	0.39	5.39E-05
chr3: 187691802-187692078	-228465	17.20	46.39	2.70	5.67E-08
chr3: 187676418-187676807	-213138	30.48	17.19	0.56	5.18E-03
chr3: 187664324-187664610	-200992	18.02	9.28	0.51	6.92E-04
chr3: 187663823-187664237	-200555	23.78	13.13	0.55	2.81E-03
chr3: 187662495-187662971	-199258	37.25	21.87	0.59	2.16E-02
chr3: 187637464-187637777	-174146	12.62	24.54	1.94	2.94E-03
**No binding change, FDR ≥ 0.05**					
chr3: 187810509-187810889	-347224	21.64	27.38	1.27	1.02E-01
chr3: 187802985-187803493	-339764	26.97	27.44	1.02	8.82E-01
chr3: 187697374-187697744	-234084	31.46	32.09	1.02	8.22E-01
chr3: 187696442-187697009	-233251	31.25	26.35	0.84	5.75E-01
chr3: 187694135-187694445	-230815	21.84	16.36	0.75	9.11E-02
chr3: 187687424-187687774	-224124	26.22	22.90	0.87	4.75E-01
chr3: 187653559-187654015	-190312	32.38	30.23	0.93	7.86E-01
chr3: 187650339-187650713	-187051	20.27	22.18	1.09	9.32E-01
chr3: 187636865-187637222	-173569	19.12	24.24	1.27	4.71E-01
chr3: 187619873-187620465	-156694	34.76	34.35	0.99	7.57E-01
chr3: 187618642-187619059	-155376	26.63	24.31	0.91	9.59E-01
chr3: 187491071-187491347	-27734	10.74	17.00	1.58	7.11E-02
