# Supplementary material for: STAT3 Targets Suggest Mechanisms of Aggressive Tumorigenesis in Diffuse Large B-Cell Lymphoma
Source: G3 (Bethesda). 2013 Oct 18;3(12):2173–85. doi: 10.1534/g3.113.007674 (PMC3852380; doi:10.1534/g3.113.007674)
Supplement: Supporting Information [file supp_g3.113.007674_TableS4.pdf]

Table S4 BRs by binding

Chromosome	Start	End	foldChange	FDR	Gene1	Peak1	Gene2	Peak2	Gene3	Peak3	Gene4	Peak4
**Unique to ABC**												
chr9	5920972	5921248	19.49	1.76E-51	MLANA	30201	RANBP6	94508				
chr6	133383848	133384124	16.78	3.73E-51	EYA4	-178509	RPS12	248278				
chr14	103785780	103785965	37.93	1.60E-46	EIF5	-14620	TNFAIP2	193209				
chr1	59512477	59512749	15.88	3.58E-45	JUN	-262828	FGGY	-250012				
chr12	11802461	11802706	17.46	6.36E-45	ETV6	-204						
chr5	40502911	40503187	14.97	6.36E-45	PTGER4	-176983						
chr9	98614870	98615154	17.90	6.36E-45	PTCH1	-344181	C9orf102	-22888				
chr3	172230265	172230541	11.00	4.49E-42	GHSR	-64200	TNFSF10	10866				
chr2	165610733	165611009	13.24	3.50E-40	GRB14	-132511	SLC38A11	201164				
chr5	95263659	95263935	12.54	6.10E-40	GLRX	-105220	ELL2	33978				
chr2	165766681	165766957	16.01	6.52E-39	GRB14	-288459	SLC38A11	45216				
chr10	24672659	24672970	9.79	2.58E-38	KIAA1217	175095	ARHGAP21	339782				
chr18	55768528	55768814	14.46	4.53E-37	NEDD4L	57052	ALPK2	527518				
chr3	186706720	186706996	11.39	5.07E-37	ST6GAL1	-32807	ADIPOQ	146395				
chr18	42087971	42088270	16.39	2.40E-35	SETBP1	-172742						
chr12	102330026	102330319	11.25	3.06E-35	DRAM1	59068	CCDC53	125726				
chr21	37915128	37915368	11.69	4.54E-35	SIM2	-156743	CLDN14	-76523				
chr18	55618482	55618718	17.64	5.30E-35	ATP8B1	-219561	NEDD4L	-93019				
chr18	55598391	55598656	21.69	3.25E-34	ATP8B1	-199485	NEDD4L	-113095				
chr18	49479891	49480173	7.98	7.01E-34	MEX3C	-756342	DCC	-386539				
chr18	52908371	52908616	11.26	7.01E-34	TCF4	347366	RAB27B	412654				
chr17	76356081	76356364	10.94	2.66E-33	SOCS3	-65						
chr12	103899120	103899424	8.78	4.30E-33	STAB2	-81797	ASCL1	547820				
chr19	57781319	57781595	8.84	4.62E-33	ZNF460	-10396	ZNF805	29404				
chr11	123350217	123350500	9.49	4.17E-32	ASAM	-284352	GRAMD1B	-46169				
chr17	76274427	76274711	8.90	4.17E-32	LOC283999	47178	SOCS3	81589				
chr21	47012333	47012610	7.46	2.83E-31	PCBP3	-257403	SLC19A1	-50121				
chr17	37640480	37640844	13.15	3.83E-31	CRKRS	22923	NEUROD2	123513				
chr17	78228617	78228904	9.17	8.30E-31	RNF213	-84965	SLC26A11	34524				
chr18	53273053	53273434	10.94	9.43E-31	TCF4	-17384						
chr15	64194186	64194462	9.17	1.59E-30	HERC1	-68177	DAPK2	144197				
chr11	27255648	27255970	8.81	2.63E-30	BBOX1	193300	LGR4	238525				
chr12	24895354	24895664	8.83	3.81E-30	SOX5	-792872	BCAT1	206799				
chr20	49133153	49133436	8.09	1.50E-29	PARD6B	-214786	PTPN1	6404				
chr19	47689713	47689953	7.72	1.53E-29	BBC3	46190	SAE1	55753				
chr18	67616215	67616491	7.02	2.16E-29	CD226	7879	DOK6	548062				

chr18	9069588	9069938	9.75	2.79E-29	NDUFV2	-32912	RAB12	460320
chr18	53915940	53916195	8.19	9.96E-29	TCF4	-660208	TXNL1	390202
chr21	23595785	23596105	8.19	1.50E-28				
chr3	146187214	146187502	7.70	1.70E-27	PLSCR4	-218392	PLSCR2	26364
chr16	76610954	76611198	8.60	3.41E-27	LOC100130958	-622273	CNTNAP4	267341
chr19	17958725	17958984	12.46	5.21E-27	JAK3	-14		
chr19	28474885	28475161	11.68	5.21E-27				
chr21	19833298	19833603	7.80	6.93E-27	PRSS7	-57481		
chr12	40707757	40708036	8.67	8.78E-27	CNTN1	-378461	LRRK2	89084
chr3	177061573	177061895	7.80	1.27E-26	TBL1XR1	-146686		
chr7	150019533	150019809	5.85	1.27E-26	LRRC61	-625		
chr20	49163433	49163709	6.28	2.20E-26	PARD6B	-184510	PTPN1	36680
chr18	55816177	55816489	7.52	2.94E-26	NEDD4L	104714	ALPK2	479856
chr18	60952649	60952914	7.44	4.02E-26	BCL2	33831	PHLPP1	570048
chr11	123985983	123986265	9.52	5.72E-26	OR10G7	-76416	X64983	-70227
chr7	30839247	30839523	6.53	6.32E-26	GHRHR	-164251	FAM188B	28352
chr19	45257633	45257915	8.43	6.36E-26	CBLC	-23352	BCL3	5796
chr18	53988700	53989075	7.30	6.90E-26	TCF4	-733028	TXNL1	317382
chr16	10007393	10007669	14.08	1.50E-25	USP7	-950190	GRIN2A	268732
chr4	78508118	78508401	8.24	2.75E-25	CXCL13	75353	CNOT6L	232284
chr19	50426565	50426859	6.94	3.22E-25	IL4I1	-26565	ATF5	-5262
chr21	45274947	45275285	7.72	1.23E-24	AGPAT3	-10000	RRP1	65698
chr2	8518458	8518734	7.76	1.30E-24	ID2	-303388		
chr11	356254	356530	5.87	1.78E-24	IFITM3	-35478	B4GALNT4	-13403
chr1	155163072	155163348	5.89	2.89E-24	MUC1	-510		
chr2	181971576	181971871	12.81	2.91E-24	ITGA4	-349895	UBE2E3	126612
chr20	42379852	42380155	6.71	2.91E-24	TOX2	-163488	GTSF1L	-24362
chr3	194210661	194210934	9.72	3.50E-24	ATP13A3	-21830	TMEM44	143315
chr18	51811452	51811847	5.70	1.16E-23	POLI	15801	STARD6	69293
chr11	35062228	35062511	6.52	4.36E-23	APIP	-124431	CD44	-98047
chr18	53256911	53257261	6.84	4.81E-23	TCF4	-1226		
chr3	186229027	186229271	7.35	5.76E-23	DGKG	-149126	CRYGS	33018
chr11	111238440	111238680	9.14	6.47E-23	C11orf92	-68021	POU2AF1	11597
chr18	77139122	77139372	6.26	6.59E-23	NFATC1	-21079	ATP9B	309850
chr11	118712198	118712510	6.42	6.75E-23	DDX6	-50382	CXCR5	-42187
chr11	35364825	35365101	10.77	8.02E-23	SLC1A2	76142	CD44	204546
chr7	130756852	130757141	6.96	9.81E-23	KLF14	-338137	MKLN1	-255598
chr12	10902813	10903089	10.98	1.18E-22	CSDA	-26998	TAS2R7	52275

chr8	134058583	134058968	5.94	1.41E-22	SLA	13827	TG	179571
chr14	20959936	20960218	7.49	1.60E-22	RNASE10	-18554	NP	22535
chr18	41143819	41144095	6.14	1.63E-22	SYT4	-286342		
chr18	60828337	60828587	8.24	2.75E-22	BCL2	158151	PHLPP1	445728
chr6	45798305	45798591	9.73	3.15E-22	SUPT3H	-452778	CLIC5	249637
chr6	64229244	64229520	7.96	3.82E-22	LGSN	-199500	PTP4A1	-52538
chr19	13010666	13010972	6.10	9.03E-22	GCDH	8845	SYCE2	19267
chr6	471273	471549	5.42	1.79E-21	IRF4	79659	EXOC2	221698
chr2	112236658	112236934	16.33	2.27E-21	BCL2L11	358305	ANAPC1	404945
chr7	130645126	130645370	10.87	2.27E-21	MKLN1	-367347	KLF14	-226388
chr15	101137048	101137324	7.60	3.02E-21	ALDH1A3	-282823	LASS3	-52261
chr16	57023276	57023555	4.82	3.02E-21	NLRC5	6		
chr19	1132195	1132453	10.92	3.73E-21	GPX4	28388	SBNO2	41958
chr1	94108121	94108380	6.33	3.86E-21	BCAR3	39134	FNBP1L	194412
chrX	15866783	15867080	10.79	5.41E-21	AP1S2	6168	ZRSR2	58358
chr4	72978329	72978632	6.37	5.77E-21	NPFFR2	80960	ADAMTS3	456035
chr2	216882193	216882494	5.11	7.95E-21	MREG	-3998		
chr22	50328892	50329181	7.71	8.77E-21	PIM3	-25106	CRELD2	16754
chr8	126614195	126614551	5.97	1.08E-20	TRIB1	171810	FAM84B	956093
chr3	188027437	188027745	7.24	1.90E-20	TPRG1	-862172	LPP	96870
chr14	76009196	76009569	10.06	2.60E-20	FLVCR2	-35557	BATF	20599
chr18	53043702	53043990	5.81	2.85E-20	TCF4	212014	RAB27B	548006
chr5	158325184	158325510	5.60	2.85E-20	EBF1	201441		
chr6	161171259	161171567	5.49	2.85E-20	MAP3K4	-241409	PLG	48139
chr11	26936587	26936863	6.16	3.11E-20	SLC5A12	-193151	FIBIN	-78903
chr17	56756796	56757072	9.17	3.52E-20	SEPT4	-150271	TEX14	12482
chr3	150803233	150803511	6.50	3.69E-20	MED12L	-1213		
chr5	54061661	54061905	10.09	4.23E-20	ESM1	219631	SNX18	248190
chr10	63808265	63808541	6.14	4.70E-20	ARID5B	146960	RTKN2	220063
chr17	37926197	37926473	5.12	4.85E-20	GRB7	32148	IKZF3	94106
chr2	60641027	60641323	6.82	6.44E-20	BCL11A	139458		
chr3	194928900	194929420	4.87	1.05E-19	LSG1	-535954	C3orf21	62735
chr11	35051554	35051835	5.66	1.10E-19	APIP	-113756	CD44	-108722
chr4	140099087	140099416	5.42	1.12E-19	ELF2	-38601	C4orf49	102240
chr22	37518196	37518481	4.93	1.33E-19	TMPRSS6	-18646	IL2RB	27623
chr12	103924220	103924464	5.95	1.35E-19	STAB2	-56727	ASCL1	572890
chr1	118206975	118207304	5.46	1.68E-19	WDR3	-265232	MAN1A2	297055
chr18	52935290	52935745	5.50	1.89E-19	TCF4	320342	RAB27B	439678

chr3	122283022	122283298	6.30	2.20E-19	DTX3L	-25	PARP9	-13
chr19	16453161	16453466	7.40	3.05E-19	KLF2	17663	EPS15L1	129448
chr15	80271082	80271391	5.73	3.07E-19	ZFAND6	-80784	BCL2A1	-7594
chr5	55290877	55291153	5.07	3.38E-19	IL6ST	-252		
chr17	45949642	45949897	5.43	3.77E-19	SP2	-23746	SP6	-16530
chr5	66502878	66503154	6.27	3.87E-19	CD180	-10399		
chr18	24002714	24002999	9.21	7.73E-19	KCTD1	125643	TAF4B	196448
chr19	47685545	47685788	7.12	8.62E-19	BBC3	50356	SAE1	51587
chr2	231526363	231526639	4.39	8.76E-19	CAB39	-51056	SP100	245630
chr1	150539864	150540108	9.88	1.13E-18	MCL1	12150	ADAMTSL4	18088
chr1	75323810	75324086	6.54	1.20E-18	LHX8	-270171	CRYZ	-124856
chr17	74188028	74188315	5.45	1.27E-18	FOXJ1	-51527	RNF157	48218
chr18	57625285	57625595	9.95	1.30E-18	PMAIP1	58248	MC4R	414561
chr3	142800899	142801352	4.98	1.31E-18	CHST2	-37542	SR140	80754
chr15	90325681	90325995	5.25	1.71E-18	MESP2	6249	ANPEP	32234
chr18	55897860	55898357	4.74	1.93E-18	NEDD4L	186490	ALPK2	398080
chr3	150811023	150811319	7.60	2.46E-18	MED12L	6586	GPR171	109817
chr17	57863867	57864130	19.32	2.74E-18	TMEM49	79136	TUBD1	106297
chrX	100663308	100663584	6.73	2.94E-18	GLA	-445	HNRNPH2	255
chr4	167760854	167761222	5.66	3.47E-18	SPOCK3	394703	TLL1	966628
chr4	102728126	102728402	4.78	3.82E-18	BANK1	16500	SLC39A8	538391
chr17	67112134	67112580	5.13	5.01E-18	ABCA9	-55221	ABCA6	25658
chr8	133991289	133991585	5.82	5.73E-18	SLA	81166	TG	112232
chr6	20769220	20769496	6.17	6.29E-18	SOX4	-824614	CDKAL1	234670
chr17	25871616	25871942	5.23	6.47E-18	LGALS9	-86395	KSR1	72743
chr3	195919187	195919427	4.43	1.02E-17	TFRC	-110275	ZDHHC19	18993
chr9	134503373	134503679	6.71	1.13E-17	UCK1	-96864	RAPGEF1	81703
chr7	106065467	106065818	4.42	1.37E-17	PIK3CG	-440281	NAMPT	-140005
chr7	52894253	52894497	6.78	1.56E-17				
chr22	38672278	38672663	6.35	1.59E-17	TMEM184B	-3455		
chr21	36260882	36261158	4.70	1.63E-17	C21orf96	150703	CLIC6	219332
chr18	3586721	3587008	5.60	1.75E-17	TGIF1	135274	DLGAP1	293275
chr5	158883837	158884114	5.25	2.34E-17	ADRA1B	-459764	IL12B	-126495
chr17	63911001	63911277	6.19	2.35E-17	AXIN2	-353399	APOH	314417
chr18	55758445	55758926	4.76	2.43E-17	NEDD4L	47067	ALPK2	537503
chr17	57906607	57906910	5.24	2.62E-17	TUBD1	63537	TMEM49	121896
chr21	44584860	44585253	4.65	3.04E-17	CRYAA	-4084		
chr3	101778418	101778732	5.02	3.45E-17	ZPLD1	-375284	NFKBIZ	210217

chr17	30842915	30843191	6.03	3.60E-17	CDK5R1	28948	MYO1D	360849
chr8	129076524	129076968	4.60	3.60E-17	MYC	328431		
chr8	60060281	60060582	5.45	4.41E-17	TOX	-28665		
chr11	82680949	82681251	5.18	4.82E-17	C11orf82	68363	RAB30	101784
chr9	98585149	98585429	6.52	6.67E-17	PTCH1	-314458	C9orf102	-52611
chr7	17199160	17199534	5.37	7.26E-17	AGR3	-277734	AHR	-138929
chr17	80408637	80408940	5.27	9.13E-17	C17orf62	-95	C17orf62	-95
chr2	208637244	208637520	9.77	9.13E-17	FZD5	-3239		
chr18	55682322	55682624	8.87	1.45E-16	ATP8B1	-283434	NEDD4L	-29146
chr13	107222809	107223143	6.08	1.96E-16	EFNB2	-35639		
chr14	103577238	103577522	5.54	3.35E-16	CDC42BPB	-53638	TNFAIP2	-15284
chr19	947785	948075	5.13	4.95E-16	GRIN3B	-52507	ARID3A	21893
chr18	53052197	53052520	8.08	5.23E-16	TCF4	203501	RAB27B	556519
chr7	17441828	17442104	7.05	6.45E-16	AHR	103690	SNX13	538165
chr3	99657398	99657698	6.81	6.80E-16	C3orf26	120867	FILIP1L	175801
chr3	101568124	101568402	4.90	7.14E-16	NFKBIZ	-95		
chr5	32211270	32211546	4.40	7.14E-16	GOLPH3	-36983	MTMR12	101706
chr12	8782112	8782415	4.28	7.30E-16	AICDA	-16822	MFAP5	33169
chr11	102459815	102460091	5.32	8.21E-16	MMP7	-58475	MMP20	36110
chr3	72034174	72034474	5.17	8.26E-16	PROK2	-199967	RYBP	461450
chr18	51790374	51790662	5.24	9.54E-16	MBD2	-39360	POLI	-5331
chr3	177538503	177538779	6.73	1.30E-15	KCNMB2	-715583	TBL1XR1	-623593
chr10	125157823	125158099	5.05	1.35E-15	GPR26	-267910	BUB3	244201
chr5	54082032	54082317	6.24	1.35E-15	ESM1	199239	SNX18	268582
chr3	186239236	186239578	6.15	1.37E-15	DGKG	-159384	CRYGS	22760
chr3	119041904	119042185	4.57	1.48E-15	CDGAP	28825	TMEM39A	140426
chr8	135575999	135576285	4.66	1.52E-15	ST3GAL1	-991959	ZFAT	149139
chr1	198171533	198171809	7.43	1.75E-15	NEK7	45563	ATP6V1G3	338404
chr18	61185718	61186054	4.57	2.38E-15	SERPINB12	-37507	SERPINB5	41742
chr10	45474119	45474459	6.02	2.45E-15	C10orf10	41		
chr6	4343088	4343332	6.30	2.92E-15	CDYL	-433470	PECI	-207379
chr3	178686801	178687077	4.51	2.96E-15	ZMAT3	102645	KCNMB2	432715
chr15	90931108	90931384	3.86	3.43E-15	IQGAP1	-227		
chr12	103134475	103134751	8.15	3.61E-15	IGF1	-260235	PAH	176768
chr9	127632536	127632820	5.37	4.14E-15	ARPC5L	1194	GOLGA1	70708
chr18	57567119	57567370	4.70	4.16E-15	PMAIP1	53		
chr18	56737727	56737995	5.01	4.31E-15	SEC11C	-69264	ZNF532	207800
chr6	76636067	76636367	6.30	4.83E-15	IMPG1	146118	MYO6	177308

chr9	140732688	140732955	4.59	5.01E-15	CACNA1B	-39419	EHMT1	219378
chr12	122429206	122429550	5.48	5.16E-15	BCL7A	-30483	PSMD9	102732
chr1	236767725	236768005	5.90	6.06E-15	HEATR1	-51		
chr2	8628202	8628579	4.44	6.22E-15	ID2	-193593		
chr18	9066351	9066627	7.63	6.64E-15	NDUFV2	-36186	RAB12	457046
chr18	77134829	77135111	6.26	7.51E-15	NFATC1	-25356	ATP9B	305573
chr22	50978023	50978388	5.89	7.68E-15	TYMP	-9948	CPT1B	38672
chr9	134505751	134506027	5.66	1.12E-14	UCK1	-99227	RAPGEF1	79340
chr10	63752508	63752796	4.86	1.13E-14	ARID5B	91209	RTKN2	275814
chr1	12079252	12079532	4.26	1.14E-14	TNFRSF8	-44042	MFN2	39154
chr20	4793719	4793995	4.52	1.18E-14	RASSF2	1912	PRND	91301
chr2	8685138	8685431	4.23	1.20E-14	ID2	-136699		
chr12	93639397	93639650	4.66	1.22E-14	EEA1	-316417	NUDT4	-132177
chr13	27705357	27705700	4.37	1.34E-14	GPR12	-370607	USP12	40500
chr12	96602599	96602933	4.45	1.44E-14	ELK3	14559	PCTK2	191457
chr14	106284244	106284695	5.69	1.44E-14	IGHE	44992	TMEM121	291517
chr12	57452345	57452623	5.96	1.46E-14	MYO1A	-8594	TMEM194A	20090
chr17	53658151	53658462	8.74	1.50E-14	MMD	-158966	TMEM100	141918
chr5	54198683	54198969	4.67	1.67E-14	ESM1	82588	SNX18	385233
chrX	118751811	118752133	6.21	1.90E-14	NKRF	-12159	SEPT6	75361
chr17	76642055	76642351	5.43	2.00E-14	DNAH17	-74799	CYTH1	136173
chr14	75957936	75958263	4.97	2.13E-14	BATF	-30684	JDP2	59263
chr14	75980794	75981038	4.35	2.50E-14	BATF	-7868	JDP2	82079
chr7	2934119	2934459	4.48	2.98E-14	GNA12	-50330	CARD11	149290
chr18	55626531	55626853	3.86	3.07E-14	ATP8B1	-227653	NEDD4L	-84927
chr1	75277088	75277365	5.67	3.35E-14	LHX8	-316892	CRYZ	-78135
chr18	43554102	43554393	3.96	3.49E-14	PSTPIP2	98002	SIGLEC15	148703
chr4	167583447	167583751	4.28	3.49E-14	SPOCK3	572142	TLL1	789189
chr5	130729884	130730186	4.39	3.57E-14	CDC42SE2	130333	RAPGEF6	240894
chr3	33091609	33091885	8.93	4.22E-14	TMPPE	46567	CCR4	98681
chr9	139298056	139298350	3.98	4.44E-14	SNAPC4	-5314	SDCCAG3	6851
chr12	8801257	8801538	5.12	4.48E-14	AICDA	-35956	MFAP5	14035
chrX	48795970	48796293	4.07	5.12E-14	PIM2	-19719	OTUD5	18761
chr19	893184	893439	11.84	5.32E-14	MED16	-94		
chr22	38852011	38852287	4.32	5.33E-14	KCNJ4	-12116	KDELR3	-11934
chr20	36722770	36723055	4.29	5.78E-14	KIAA0406	-61080	TGM2	70787
chr1	159046984	159047340	11.92	6.12E-14	AIM2	-515		
chr22	35807052	35807328	4.02	6.23E-14	RASD2	-130162	MCM5	11074

chr16	29831611	29831887	3.75	6.83E-14	MVP	-38		
chr16	87731868	87732190	3.99	7.34E-14	KLHDC4	67513	JPH3	95530
chr15	55581878	55582175	4.09	8.17E-14	PIGB	-29106	RAB27A	-18920
chr21	36584792	36585075	6.09	8.38E-14	CBR1	-857351	RUNX1	-163339
chr7	28191532	28191834	4.27	8.82E-14	JAZF1	28754	TAX1BP1	411945
chr18	55879535	55879825	4.12	8.95E-14	NEDD4L	168061	ALPK2	416509
chr2	8683509	8684110	4.07	1.10E-13	ID2	-138174		
chr2	198081273	198081549	3.93	1.10E-13	ANKRD44	-18649	SF3B1	218360
chr4	145556148	145556424	6.51	1.15E-13	GYPA	-494382	HHIP	-10887
chr8	135704533	135704786	6.04	1.30E-13	ZFAT	20621		
chr12	104872163	104872439	4.16	1.33E-13	CHST11	21523	SLC41A2	450171
chr3	126690874	126691201	3.58	1.41E-13	TXNRD3	-317093	PLXNA1	-16464
chr1	42133024	42133344	3.62	1.51E-13	EDN2	-182840	HIVEP3	251312
chr3	194850201	194850615	4.23	1.79E-13	LSG1	-457202	C3orf21	141487
chr18	56459347	56459623	5.22	2.01E-13	ZNF532	-70576	MALT1	120867
chr18	36520830	36521147	4.38	2.15E-13				
chr11	121289397	121289644	4.99	2.34E-13	SORL1	-33440	SC5DL	126133
chr2	160735690	160735966	6.26	2.42E-13	LY75	25434	MARCH7	166818
chr9	140205075	140205362	5.74	2.85E-13	NRARP	-8516	EXD3	112495
chr18	56633584	56633830	4.95	2.88E-13	SEC11C	-173418	ZNF532	103646
chr12	132258048	132258409	3.56	3.25E-13	MMP17	-54712	SFRS8	62594
chr4	105981783	105982087	5.35	3.42E-13	CXXC4	-569468	TET2	-86008
chr11	3071072	3071377	3.82	4.31E-13	NAP1L4	-57618	CARS	7446
chr19	47792751	47793210	3.66	4.64E-13	C5AR1	-20123	PRR24	14839
chr19	57791082	57791392	5.57	4.69E-13	ZNF460	-616		
chr8	133932323	133932606	7.45	4.89E-13	TG	53260	SLA	140138
chr18	60809034	60809314	5.10	4.90E-13	BCL2	177439	PHLPP1	426440
chr14	75988637	75988917	3.84	4.97E-13	BATF	-7		
chr2	25633308	25633584	4.02	5.00E-13	DNMT3A	-68672	DTNB	263057
chr12	125259140	125259419	3.96	5.55E-13	NCOR2	-239123	SCARB1	89239
chr6	2857881	2858303	4.21	6.05E-13	SERPINB1	-16011	SERPINB9	45453
chr2	46137030	46137320	5.00	6.40E-13	EPAS1	-387388	PRKCE	258132
chr6	24940632	24940908	3.78	6.63E-13	FAM65B	-29575	DKFZp686H12134	197281
chr20	21284245	21284521	3.60	6.95E-13	XRN2	441		
chr11	103800264	103800526	4.24	7.06E-13	DDI1	-106913	DYNC2H1	820235
chr9	98029043	98029322	5.32	7.13E-13	FANCC	50808	C9orf3	540189
chr2	55361016	55361315	4.74	7.85E-13	RPS27A	-97899	RTN4	-83432
chr2	10951059	10951358	3.68	1.13E-12	PDIA6	1751	ATP6V1C2	89434

chr3	150886135	150886411	4.61	1.13E-12	GPR171	34715	MED12L	81688
chr12	11903384	11903660	4.33	1.85E-12	BCL2L14	-320356	ETV6	100734
chr18	12839733	12840009	3.97	1.85E-12	PTPN2	44463	PSMG2	136807
chr21	44582645	44583046	3.88	1.85E-12	U2AF1	-55158	CRYAA	-6295
chr12	112568372	112568685	3.80	2.21E-12	TRAFD1	5180	C12orf51	175509
chr18	3593934	3594260	4.80	2.55E-12	TGIF1	142506	DLGAP1	286043
chr19	2475691	2475971	3.93	2.66E-12	GADD45B	-304		
chr21	26561044	26561320	4.11	2.69E-12	MRPL39	418619		
chr19	56632703	56633053	3.71	2.89E-12	ZNF787	-229		
chr19	10381574	10381850	3.91	3.27E-12	ICAM1	195		
chr5	140090676	140090985	3.88	3.91E-12	PCDHAC2	-255281	ZMAT2	10799
chr22	27054911	27055216	3.94	4.35E-12	CRYBA4	37136		
chr17	76339741	76340093	3.98	4.86E-12	SOCS3	16241	LOC283999	112526
chr4	141712037	141712421	4.51	5.70E-12	TBC1D9	-34758	RNF150	342387
chr17	12154233	12154577	4.74	5.71E-12	MYOCD	-414802	MAP2K4	230270
chr9	82749497	82749750	6.86	5.98E-12	TLE4	562746		
chr3	32480507	32480768	4.08	6.27E-12	CMTM7	47475	CMTM6	63765
chr16	48492304	48492580	4.80	7.21E-12	SIAH1	-73213	N4BP1	151678
chr2	242067139	242067386	3.87	8.46E-12	PASK	21615	SNED1	129008
chrX	48794319	48794614	5.56	9.61E-12	PIM2	-18054	OTUD5	20426
chr11	123349780	123350169	4.08	9.88E-12	ASAM	-283968	GRAMD1B	-46553
chr19	36202156	36202502	4.76	9.89E-12	ZBTB32	-1501		
chr1	150551940	150552220	4.04	1.02E-11	MCL1	56		
chr17	12762960	12763279	4.59	1.15E-11	RICH2	70291	ELAC2	158239
chr16	19865609	19865913	5.71	1.20E-11	C16orf88	-136269	GPRC5B	30390
chr18	53073121	53073436	4.86	1.20E-11	TCF4	182581	RAB27B	577439
chr1	205282831	205283155	4.50	1.23E-11	NUAK2	7890	TMCC2	85902
chr17	73872156	73872432	3.56	1.29E-11	UNC13D	-31496	TRIM47	2362
chr3	183309748	183310075	6.15	1.29E-11	KLHL24	-43499	KLHL6	-36413
chr9	130533626	130533902	3.44	1.29E-11	TOR2A	-36160	SH2D3C	7284
chr4	164512786	164513133	4.42	1.32E-11	TKTL2	-117913	MARCH1	21816
chr5	98363130	98363415	3.83	1.33E-11	CHD1	-101035		
chr14	73689739	73690050	4.21	1.37E-11	PAPLN	-14310	PSEN1	86752
chr12	27373199	27373475	5.75	1.62E-11	STK38L	-23741	MED21	197854
chr12	66696106	66696418	3.14	1.67E-11	HELB	-94		
chr5	131826354	131826730	3.87	1.85E-11	IRF1	-77		
chr18	73029727	73030003	5.69	2.20E-11	TSHZ1	107134	C18orf62	109724
chr19	53510549	53510793	3.52	2.58E-11	AK127846	-94		

chr10	12164709	12164977	4.30	2.65E-11	SEC61A2	-6797	DHTKD1	53909
chr20	20432914	20433236	4.24	2.71E-11	INSM1	84310	RALGAPA2	260191
chr1	207752765	207753058	3.98	2.72E-11	CR1L	-65546	CR1	83439
chr8	129115768	129116245	3.36	3.01E-11	MYC	367692		
chr15	31638213	31638489	4.10	3.07E-11	KLF13	19268	OTUD7A	309191
chr22	38656280	38656604	3.38	3.09E-11	TMEM184B	12574	MAFF	46900
chr12	24972604	24972854	5.46	3.18E-11	SOX5	-870092	BCAT1	129579
chr1	54355376	54355670	3.47	3.53E-11	DIO1	-4338	YIPF1	-70
chr11	103751780	103752056	5.25	3.56E-11	DDI1	-155390	DYNC2H1	771758
chr18	33765217	33765493	4.71	3.56E-11	MOCOS	-2125		
chr18	60708352	60708695	4.96	3.56E-11	BCL2	278089	PHLPP1	325790
chr17	80256027	80256343	3.69	3.63E-11	CSNK1D	-24612	CD7	19295
chr5	110590427	110590690	3.64	3.65E-11	CAMK4	30477	STARD4	257598
chr18	57069734	57070046	4.67	3.96E-11	LMAN1	-43382	CCBE1	294754
chr15	40339935	40340280	3.92	4.04E-11	SRP14	-8719	BMF	60967
chr22	37484081	37484357	4.30	4.04E-11	TMPRSS6	15474	KCTD17	36440
chr15	42750126	42750402	3.65	4.78E-11	ZFP106	-534		
chr16	10008221	10008542	4.85	4.78E-11	USP7	-951041	GRIN2A	267881
chr18	56631000	56631311	3.56	4.96E-11	SEC11C	-175969	ZNF532	101095
chr12	46502541	46502857	4.03	4.99E-11	SFRS2IP	-118298	SLC38A1	160509
chr20	51590641	51590911	5.80	5.08E-11	TSHZ2	1899	ZNF217	608931
chr15	85279599	85279996	3.65	5.10E-11	SEC11A	-20124	ZNF592	-12020
chr18	60683545	60683822	3.36	5.24E-11	PHLPP1	300950	BCL2	302929
chr10	53459582	53459858	3.82	5.35E-11	CSTF2T	-365		
chr1	17518908	17519184	6.62	5.64E-11	PADI2	-73098	PADI1	-12575
chr6	221118	221400	3.63	6.83E-11	DUSP22	-70842		
chr19	35838409	35838860	4.65	7.74E-11	FFAR1	-3810		
chr18	55789154	55789539	3.21	8.13E-11	NEDD4L	77728	ALPK2	506842
chr2	58135895	58136173	9.33	9.81E-11	VRK2	-137695		
chr18	12879837	12880113	4.01	1.03E-10	PTPN2	4359	PSMG2	176911
chr7	105410455	105410762	4.07	1.14E-10	FLJ23834	-193048	RINT1	238077
chr18	55549133	55549495	3.39	1.18E-10	NEDD4L	-162305	ATP8B1	-150275
chr3	169774598	169775113	3.68	1.26E-10	GPR160	19121	PHC3	124681
chr1	172750683	172750995	4.12	1.31E-10	FASLG	122654	TNFSF18	269264
chr12	27361042	27361303	4.95	1.36E-10	STK38L	-35905	MED21	185690
chr17	58469540	58469900	3.65	1.41E-10	USP32	-134		
chr11	111921490	111921772	5.03	1.47E-10	DLAT	26093	TIMM8B	35828
chr1	150534560	150534903	22.57	1.51E-10	ADAMTSL4	12834	MCL1	17404

chr17	73316262	73316615	3.45	1.55E-10	SLC25A19	-30909	GRB2	85351
chr5	177659706	177659999	3.22	1.66E-10	AGXT2L2	-50		
chr12	122470522	122470805	3.49	1.79E-10	MLXIP	-46096	BCL7A	10803
chr20	20444732	20445010	3.31	1.95E-10	INSM1	96106	RALGAPA2	248395
chr16	59627200	59627456	5.99	2.10E-10	GOT2	-859082		
chr3	132036014	132036274	4.18	2.16E-10	ACPP	-67		
chr14	81937533	81937853	4.07	2.17E-10	STON2	-72769	SEL1L	62512
chr16	18937824	18938131	3.31	2.30E-10	SMG1	-252		
chr20	49461297	49461541	4.96	2.31E-10	BCAS4	49952	ADNP	86108
chr11	118709620	118710084	3.49	2.33E-10	DDX6	-47880	CXCR5	-44689
chr2	64281274	64281627	2.95	2.54E-10	VPS54	-35237	PELI1	90154
chr17	77005714	77006079	2.99	2.59E-10	CANT1	2		
chr4	77120836	77121100	3.03	2.66E-10	NUP54	-51313	SCARB2	14067
chr19	50850609	50850885	3.30	2.83E-10	NAPSB	-2742		
chr4	185395779	185396086	3.21	3.03E-10	IRF2	-207		
chr18	57364534	57365024	4.49	3.06E-10	CCBE1	-135		
chr16	71887376	71887667	3.39	3.31E-10	ATXN1L	7623	ZNF821	29890
chr20	33735017	33735300	3.06	3.42E-10	EDEM2	2		
chr11	65188730	65189022	5.83	3.50E-10	SCYL1	-103672	FRMD8	34835
chr18	60251605	60251879	3.34	3.76E-10	PHLPP1	-130992	ZCCHC2	61084
chr7	50418962	50419259	3.66	3.92E-10	IKZF1	74733	FIGNL1	98977
chrX	134248462	134248761	7.57	3.98E-10	FAM127A	82279	ZNF75D	229345
chr6	119670952	119671232	3.56	4.12E-10	MAN1A1	-166		
chr1	221219537	221219813	3.52	4.21E-10	HLX	166932	DUSP10	695786
chr6	37018002	37018458	3.83	4.67E-10	PIM1	-119692	FGD2	44807
chr1	185572412	185572724	3.48	4.79E-10	IVNS1ABP	-286107	HMCN1	-131115
chr8	24244967	24245326	3.69	4.98E-10	ADAM7	-53362	ADAMDEC1	3349
chr1	66736898	66737174	3.42	5.18E-10	SGIP1	-262789	PDE4B	478180
chr19	45504370	45504688	2.79	5.21E-10	RELB	-183		
chr13	99959570	99959848	3.57	5.38E-10	GPR183	40		
chr7	28744959	28745240	3.44	5.59E-10	TRIL	252929	CREB5	292956
chr3	69132039	69132354	3.46	5.66E-10	UBA3	-2673	ARL6IP5	-1893
chr8	59864471	59864747	4.23	6.06E-10	NSMAF	-292643	TOX	167158
chr19	28884977	28885253	3.17	6.17E-10	UQCRFS1	819021		
chr14	102241151	102241491	3.01	6.18E-10	DYNC1H1	-189544	PPP2R5C	13186
chr9	6689964	6690243	3.74	6.23E-10	KDM4C	-67537	GLDC	-44412
chr1	204422311	204422556	3.63	6.40E-10	PPP1R15B	-41490	PIK3C2B	37040
chr16	76997540	76997859	3.11	7.21E-10	LOC100130958	-235649	CNTNAP4	653965

chr1	145421707	145422001	3.57	7.30E-10	TXNIP	-16608	HFE2	8663
chr1	226297951	226298264	3.85	8.19E-10	H3F3B	47687	ACBD3	76315
chr1	236319825	236320287	3.53	9.14E-10	GPR137B	14224	ERO1LB	125283
chr6	84937181	84937457	3.26	9.52E-10	KIAA1009	16		
chr15	91137632	91138004	4.33	1.01E-09	BLM	-122761	CRTC3	64620
chr17	40540661	40540981	17.89	1.05E-09	STAT3	-308		
chr6	75308159	75308431	5.67	1.05E-09	COL12A1	607328	CD109	902787
chr11	116933890	116934216	4.17	1.10E-09	APOA1	-225715	SIK3	34940
chr2	10829990	10830311	3.21	1.10E-09	NOL10	-39		
chr19	12904296	12904672	4.06	1.11E-09	JUNB	2174	PRDX2	8210
chr8	129062987	129063334	3.62	1.11E-09	MYC	314846		
chr5	137514518	137514870	3.15	1.15E-09	BRD8	-336	KIF20A	277
chr5	54060028	54060272	3.81	1.24E-09	ESM1	221264	SNX18	246557
chr18	42305616	42305895	3.11	1.26E-09	SLC14A2	-889010	SETBP1	44893
chr3	119357759	119358033	3.65	1.30E-09	POPDC2	21508	PLA1A	41174
chr1	235147342	235147988	4.95	1.37E-09	IRF2BP2	-402394	TOMM20	144591
chr1	245027774	245028122	3.17	1.37E-09	HNRNPU	-121		
chr1	152007086	152007362	3.26	1.38E-09	S100A10	-40510	S100A11	2287
chr12	121665107	121665366	4.37	1.38E-09	P2RX4	17573	CAMKK2	70874
chr2	222419996	222420272	3.65	1.42E-09	EPHA4	16876		
chr18	56203866	56204148	2.94	1.53E-09	ALPK2	92182	NEDD4L	492388
chr1	3573348	3573594	5.54	1.57E-09	TP73	4342	KIAA0495	90415
chr19	1897590	1897897	3.79	1.58E-09	FAM108A1	-12226	SCAMP4	-7629
chr18	60822684	60823034	4.16	1.64E-09	BCL2	163754	PHLPP1	440125
chr18	67861552	67861870	3.71	1.68E-09	CD226	-237479	RTTN	11251
chr3	126630348	126630598	3.20	1.71E-09	TXNRD3	-256528	PLXNA1	-77029
chr3	126673717	126674115	3.40	1.71E-09	TXNRD3	-299971	PLXNA1	-33586
chr4	150959801	150960168	4.65	1.71E-09	DCLK2	-40095		
chr8	24151467	24151772	3.25	1.75E-09	ADAM28	40		
chr3	15403116	15403532	3.21	1.78E-09	SH3BP5	-29220	METTL6	65718
chr16	11269682	11269998	3.63	1.79E-09	SOCS1	80199	CIITA	298785
chr17	49205441	49205717	4.16	1.81E-09	NME1-NME2	-25341	SPAG9	-7353
chr1	205761844	205762120	4.02	1.86E-09	RAB7L1	-17372	SLC41A1	20179
chr18	53100836	53101264	3.53	1.91E-09	TCF4	154810	RAB27B	605210
chr1	156163604	156163903	4.50	1.95E-09	SLC25A44	24		
chr7	134320855	134321138	4.27	1.96E-09	BPGM	-10534	AKR1B15	70764
chrX	71528752	71529028	6.24	2.10E-09	CITED1	-3126		
chr14	24630225	24630563	12.26	2.20E-09	IRF9	665		

chr15	70678921	70679275	3.29	2.20E-09	TLE3	-288842	UACA	376752
chr9	114747789	114748089	4.55	2.20E-09	UGCG	88733	SUSD1	189617
chr17	76272222	76272515	4.03	2.38E-09	LOC283999	44978	SOCS3	83789
chr11	57333640	57333928	3.61	2.44E-09	UBE2L6	1396	SMTNL1	24805
chr15	75016350	75016680	2.96	2.51E-09	EDC3	-28129	CYP1A1	1362
chr19	660313	660795	3.13	2.56E-09	RNF126	2673	FGF22	20628
chr3	88198798	88199089	3.02	2.66E-09	C3orf38	51		
chr2	111977031	111977361	3.46	2.67E-09	BCL2L11	98705	ANAPC1	664545
chr20	42381133	42381460	3.27	2.79E-09	TOX2	-162195	GTSF1L	-25655
chr2	165617783	165618064	4.48	2.80E-09	GRB14	-139564	SLC38A11	194111
chr2	225531724	225532052	3.53	2.80E-09	CUL3	-81778	DOCK10	375442
chr14	75886112	75886397	3.24	2.83E-09	JDP2	-12582	FOS	140774
chr3	112395694	112395971	3.21	2.84E-09	CCDC80	-35856	CD200R1L	168964
chr21	43947941	43948241	2.89	2.93E-09	PDE9A	-125771	SLC37A1	28349
chr18	48362882	48363158	3.78	3.00E-09	ME2	-42415	MRO	-16586
chr2	225906093	225906379	3.62	3.22E-09	CUL3	-456126	DOCK10	1094
chr10	63803225	63803587	3.21	3.26E-09	ARID5B	141963	RTKN2	225060
chr4	84101567	84101849	4.85	3.80E-09	COQ2	104256	COPS4	145469
chr18	44811084	44811445	3.19	3.83E-09	CORL2	-35711	SMAD2	646250
chr8	129186456	129186748	3.97	4.08E-09	MYC	438287		
chr2	145113956	145114206	4.57	4.26E-09	GTDC1	-62021	ZEB2	163835
chr13	42490688	42491071	3.36	4.33E-09	KIAA0564	44341	C13orf15	459338
chr7	105406861	105407114	5.08	4.33E-09	FLJ23834	-196669	RINT1	234456
chr19	49588237	49588531	3.20	4.36E-09	SNRNP70	-81	SNRNP70	-81
chr12	7261732	7262008	2.83	4.55E-09	C1RL	-77		
chr15	76867756	76868035	5.02	5.10E-09	ISL2	238749	SCAPER	308321
chr9	4464909	4465227	3.34	5.40E-09	GLIS3	-165033	SLC1A1	-25376
chr3	191013868	191014178	3.06	5.50E-09	CCDC50	-32851	OSTN	83701
chr13	20438927	20439203	3.90	5.61E-09	ZMYM5	-1289		
chr11	68180307	68180729	2.87	5.66E-09	SAPS3	-47668	LRP5	100410
chr4	40538082	40538372	3.47	5.68E-09	RBM47	93656	CHRNA9	200758
chr22	29999259	29999535	3.19	6.09E-09	NF2	-148		
chr12	94954622	94954995	3.39	6.10E-09	TMCC3	89515	PLXNC1	412310
chr8	66856731	66857060	4.59	6.11E-09	PDE7A	-155575	DNAJC5B	-76895
chr2	201988436	201988887	3.06	6.14E-09	CASP10	-58959	CFLAR	7846
chr18	44471757	44472037	3.12	6.25E-09	ST8SIA5	-134858	PIAS2	25569
chr1	212740495	212740829	3.18	6.93E-09	ATF3	1965	BATF3	132665
chr8	125774149	125774425	4.52	7.02E-09	ZNF572	-211252	MTSS1	-33557

chr14	76015470	76015817	2.85	7.76E-09	FLVCR2	-29296	BATF	26860
chr2	209176057	209176348	3.83	8.21E-09	PTH2R	-95353	PIKFYVE	45212
chr19	35837564	35837836	2.91	8.27E-09	FFAR1	-4745		
chr3	185255112	185255392	3.56	8.41E-09	TMEM41A	-38407	LIPH	15117
chr11	109898061	109898306	3.96	8.46E-09	ZC3H12C	-65903	C11orf87	605309
chr2	61989396	61989674	5.03	8.80E-09	XPO1	-224117	CCT4	126256
chr17	73291754	73292151	4.22	9.02E-09	SLC25A19	-6423	GRB2	109837
chr9	128463169	128463445	3.30	9.02E-09	MAPKAP1	6206	GAPVD1	439196
chr13	100072601	100072951	2.72	9.52E-09	GPR183	-113027	TM9SF2	-80952
chr1	51985027	51985355	2.44	9.82E-09	EPS15	-196		
chr15	70571058	70571357	3.04	9.82E-09	TLE3	-180952	UACA	484642
chr9	138022182	138022469	2.85	9.86E-09	KIAA0649	-349322	OLFM1	55237
chr16	81782040	81782392	3.90	1.06E-08	PLCG2	-30714	CMIP	303441
chr12	105035733	105035984	4.40	1.10E-08	CHST11	185081	SLC41A2	286613
chr6	11461633	11461909	3.26	1.13E-08	NEDD9	-228856	TMEM170B	-76740
chr2	161560800	161561088	3.96	1.24E-08	TANK	-432522	RBMS1	-210626
chr2	185228050	185228340	3.34	1.26E-08	ZNF804A	-234898		
chr15	81403881	81404173	3.35	1.30E-08	MESDC2	-121822	IL16	-85192
chr3	150945556	150945832	2.90	1.33E-08	GPR171	-24706	P2RY14	50536
chr12	26624401	26624711	2.98	1.38E-08	SSPN	276050	ITPR2	361575
chr16	4303781	4304079	3.05	1.38E-08	SRL	-11849	TFAP4	19071
chr2	158325910	158326225	3.43	1.38E-08	CYTIP	-25464	ACVR1C	159331
chr13	99911248	99911522	3.17	1.41E-08	GPR18	-545		
chr11	34675500	34675810	3.43	1.45E-08	PDHX	-262022	EHF	32987
chr8	142192433	142192745	3.01	1.45E-08	PTK2	-181257	SLC45A4	46084
chr7	141310557	141310833	4.81	1.46E-08	WEE2	-97458	AGK	59617
chr1	149969580	149969873	2.99	1.70E-08	MTMR11	-60936	OTUD7B	12959
chr4	3087073	3087349	3.90	1.93E-08	RGS12	-228663	HTT	10803
chr18	55297398	55297687	3.18	2.02E-08	NARS	-8366	ATP8B1	101496
chr3	188002398	188002646	3.58	2.04E-08	TPRG1	-887241	LPP	71801
chr4	130453925	130454201	5.64	2.05E-08	SCLT1	-439299		
chr11	82708249	82708631	3.07	2.10E-08	RAB30	74444	C11orf82	95703
chr12	113508013	113508349	3.18	2.18E-08	DTX1	12519	RASAL1	65840
chr14	96164115	96164459	2.55	2.18E-08	TCL1A	16246	GLRX5	162964
chr2	61034485	61034768	2.42	2.18E-08	REL	-74125	PAPOLG	51244
chr12	11989223	11989779	4.01	2.27E-08	BCL2L14	-234377	ETV6	186713
chr17	56446323	56446606	3.17	2.37E-08	SUPT4H1	-16902	RNF43	48466
chr9	140733682	140734061	3.03	2.43E-08	CACNA1B	-38369	EHMT1	220428

chr7	128603507	128603751	3.39	2.47E-08	IRF5	25635	TNPO3	91569
chr5	75885946	75886222	4.08	2.50E-08	F2RL2	33156	IQGAP2	186935
chr4	144298980	144299270	4.13	2.56E-08	SMARCA5	-135491	GAB1	41142
chr11	65239601	65239910	2.60	2.72E-08	SCYL1	-52792	FRMD8	85715
chr20	32156194	32156482	2.89	2.92E-08	C20orf134	-97966	CBFA2T2	78410
chr11	100551832	100552206	3.27	3.02E-08	FLJ32810	-6388		
chr3	142164885	142165161	4.48	3.17E-08	GK5	-220595	XRN1	1830
chr17	60730748	60731083	3.52	3.31E-08	MRC2	26154	MARCH10	154789
chr2	102239235	102239510	3.40	3.34E-08	RFX8	-148208	MAP4K4	-75115
chr13	50940985	50941333	3.34	3.39E-08	RNASEH2B	-542733	ST13	195005
chr3	107819846	107820164	3.49	3.43E-08	CD47	-10070	IFT57	121412
chr20	39657060	39657353	2.83	3.47E-08	TOP1	-255		
chr12	51751189	51751453	3.07	3.57E-08	CELA1	-10858	GALNT6	33879
chr5	65121780	65122028	3.35	3.66E-08	ERBB2IP	-100480	NLN	103819
chr21	44772532	44773056	3.16	3.83E-08	SIK1	74208	CRYAA	183653
chr8	142168247	142168491	2.83	3.87E-08	PTK2	-157037	SLC45A4	70304
chr2	222067794	222068216	3.55	3.92E-08	EPHA4	369005		
chr4	79671321	79671766	3.37	4.35E-08	BMP2K	-25988	ANXA3	198802
chr16	85449975	85450310	2.82	4.39E-08	KIAA0182	-196781	KIAA0513	388733
chr9	140221137	140221494	3.11	4.46E-08	NRARP	-24613	EXD3	96398
chr1	208351083	208351386	4.18	4.68E-08	CD34	-266552	PLXNA2	66430
chr1	212113305	212113554	3.07	5.32E-08	LPGAT1	-109316	INTS7	95454
chr3	177055507	177055856	2.92	5.42E-08	TBL1XR1	-140634		
chr5	10562498	10562878	2.61	5.44E-08	ROPN1L	120679	DAP	198699
chr16	85932527	85932920	2.87	5.67E-08	IRF8	-50		
chr18	60189950	60190269	3.06	5.67E-08	ZCCHC2	-548		
chr3	187691802	187692078	2.70	5.67E-08	LPP	-238781	BCL6	-228465
chr8	129083285	129083719	2.59	5.82E-08	MYC	335187		
chr2	191884967	191885324	4.89	6.35E-08	STAT1	-6170	STAT4	130779
chr9	33081266	33081542	2.64	6.67E-08	SMU1	-4690		
chr18	22932063	22932339	2.58	6.83E-08	ZNF521	13		
chr2	54865925	54866219	3.60	6.83E-08	EML6	-86077	DKFZp547I014	109713
chr8	136935355	136935621	4.62	7.26E-08	KHDRBS3	465772		
chr20	32829094	32829400	3.60	7.32E-08	EIF2S2	-129162	ASIP	-18924
chr1	17565902	17566193	2.19	7.49E-08	PADI3	-9545	PADI1	34427
chr9	139776192	139776500	2.76	7.49E-08	TRAF2	-4619		
chr21	43889425	43889744	3.31	7.65E-08	RSPH1	26816	UBASH3A	65566
chr3	15598020	15598324	3.56	7.82E-08	COLQ	-34914	HACL1	44958

chr20	50021347	50021623	3.06	7.88E-08	KCNG1	-381810	NFATC2	137773
chr13	100150814	100151183	3.11	8.08E-08	TM9SF2	-2729		
chr20	24404481	24404725	4.24	9.35E-08	GGTLC1	-435187	TMEM90B	-45232
chr3	138048567	138048843	2.90	9.77E-08	TXNDC6	23		
chr9	134604864	134605182	3.26	9.87E-08	RAPGEF1	-19794	MED27	350230
chr12	123893863	123894152	4.23	1.05E-07	SETD8	25304	RILPL2	27256
chr4	139111526	139112060	2.92	1.14E-07	PCDH18	-658164	SLC7A11	51710
chr4	185189151	185189640	3.02	1.14E-07	ENPP6	-50282	IRF2	206330
chr1	110786327	110786610	3.06	1.18E-07	RBM15	-95476	KCNC4	32404
chr11	34255653	34255965	2.97	1.22E-07	ABTB2	122993	NAT10	128698
chr11	70280402	70280696	2.79	1.32E-07	CTTN	35937	SHANK2	655293
chr2	24713502	24713885	3.13	1.33E-07	ITSN2	-130297	NCOA1	-93651
chr12	11912068	11912362	3.58	1.38E-07	BCL2L14	-311663	ETV6	109427
chr1	146555942	146556340	3.20	1.49E-07	PDZK1	-624101	PRKAB2	87988
chr17	16879687	16879977	3.29	1.50E-07	TNFRSF13B	-4430		
chr9	102821102	102821383	3.27	1.50E-07	ERP44	40087	STX17	152328
chr21	37661500	37661741	2.84	1.54E-07	MORC3	-30866	DOPEY2	124782
chr1	195164625	195164911	3.55	1.55E-07				
chr12	92797649	92797939	3.45	1.59E-07	PLEKHG7	-332471	BTG1	-258121
chr15	31558049	31558469	3.00	1.60E-07	TRPM1	-164335	KLF13	-60824
chr18	61089546	61090098	4.43	1.62E-07	VPS4B	-70		
chr20	52276816	52277092	4.10	1.62E-07	ZNF217	-77247	BCAS1	410350
chr17	28033450	28033780	2.90	1.63E-07	GIT1	-117005	SSH2	223403
chr12	24872449	24872696	3.77	1.64E-07	SOX5	-769936	BCAT1	229735
chr5	172483174	172483482	2.71	1.64E-07	C5orf41	-42		
chr8	134070312	134070731	2.49	1.66E-07	SLA	2081	TG	191317
chr18	56317338	56317687	3.06	1.72E-07	ALPK2	-21324	MALT1	-21105
chr20	38458360	38458789	3.00	1.72E-07	MAFB	859301	DHX35	867575
chr10	37717723	37717994	4.70	1.92E-07	ANKRD30A	303074	ZNF248	428627
chr19	12947122	12947403	2.28	1.92E-07	MAST1	-1996	RTBDN	-1033
chr7	22737969	22738312	2.90	1.96E-07	MGC87042	-198343	IL6	-28625
chr5	150439558	150439815	2.83	2.01E-07	TNIP1	21310	GPX3	39688
chr11	73231856	73232132	4.95	2.05E-07	PLEKHB1	-126600	RELT	144589
chr16	10023248	10023524	4.78	2.10E-07	USP7	-966045	GRIN2A	252877
chr2	135498022	135498298	3.66	2.18E-07	ACMSD	-98026	TMEM163	-21589
chr1	192595703	192596030	2.97	2.31E-07	RGS13	-9415	RGS1	51010
chr7	116651960	116652225	3.09	2.36E-07	ST7	58712	WNT2	311250
chr6	134557178	134557472	2.98	2.43E-07	SLC2A12	-183536	SGK1	81871

chr3	13129564	13129870	2.43	2.57E-07	IQSEC1	-15100	NUP210	332092
chr1	169662345	169662632	3.97	2.61E-07	SELP	-63112	SELL	18348
chr16	75109188	75109464	3.19	2.61E-07	LDHD	41339	ZNRF1	76411
chrX	39719541	39719841	3.33	2.61E-07	BCOR	237028		
chr18	2641685	2641984	2.50	2.65E-07	SMCHD1	-14051	NDC80	70325
chr2	69016772	69017107	2.89	2.65E-07	ARHGAP25	54972	BMP10	81709
chr18	60926651	60927142	3.38	2.69E-07	BCL2	59716	PHLPP1	544163
chr2	238795272	238795566	3.45	2.74E-07	UBE2F	-80281	RAMP1	27232
chr16	87942945	87943286	2.84	2.78E-07	SLC7A5	-40016	CA5A	26996
chr11	87070566	87070857	3.66	2.82E-07	TMEM135	321647	RAB38	837887
chr11	61348854	61349179	2.58	2.83E-07	SYT7	-719		
chr7	155137218	155137514	2.43	3.13E-07	EN2	-113458	INSIG1	47880
chr5	157288358	157288646	3.02	3.19E-07	CLINT1	-2334		
chr15	99191499	99191775	2.88	3.31E-07	IGF1R	-1124		
chr9	2241915	2242296	3.54	3.36E-07	VLDLR	-379687	SMARCA2	226764
chr16	28935262	28935601	2.84	3.44E-07	RABEP2	1100	ATP2A1	45623
chr6	157886272	157886548	3.90	3.44E-07	SNX9	-357884	ZDHHC14	83853
chr16	27442359	27442635	3.13	3.48E-07	IL21R	3918	GTF3C1	118754
chr11	75524223	75524490	4.07	3.55E-07	UVRAG	-1855		
chr7	137686939	137687203	2.83	3.67E-07	CREB3L2	-225		
chr20	43367517	43367793	2.99	3.68E-07	KCNK15	-6833	WISP2	23770
chr13	99358785	99359058	3.74	3.72E-07	STK24	-129526	SLC15A1	46007
chr20	42310643	42311045	4.38	3.72E-07	MYBL2	15135	GTSF1L	44798
chr3	175236307	175236641	3.91	3.73E-07	NAALADL2	659363		
chr2	8666941	8667319	2.98	3.74E-07	ID2	-154854		
chr18	54318303	54318684	3.23	3.76E-07	WDR7	-122		
chr1	11741117	11741611	2.76	3.77E-07	MAD2L2	10314	FBXO6	17214
chr7	130645914	130646192	2.38	3.78E-07	MKLN1	-366542	KLF14	-227193
chr16	87889867	87890143	2.81	3.84E-07	KLHDC4	-90463	SLC7A5	13095
chr2	64487988	64488299	2.47	4.11E-07	HSPC159	-193183	PELI1	-116539
chr12	47749984	47750421	2.86	4.12E-07	AMIGO2	-276469	RPAP3	349641
chr7	138802952	138803267	3.12	4.22E-07	TTC26	-15380	ZC3HAV1	-8645
chrX	64062392	64062722	3.43	4.27E-07	MTMR8	-447246	ZC4H2	133779
chr6	70591741	70592027	2.60	4.28E-07	COL19A1	15436	COL9A1	420902
chr12	24992222	24992597	2.76	4.47E-07	SOX5	-889773	BCAT1	109898
chr19	36231289	36231565	2.72	4.50E-07	TMEM149	2093	MLL4	22506
chr11	118754259	118754737	3.34	4.84E-07	CXCR5	-43		
chr15	99396471	99396748	2.08	5.05E-07	LOC145814	152275	IGF1R	203849

chr2	45955608	45955956	2.60	5.05E-07	EPAS1	-568781	PRKCE	76739
chr19	46018192	46018547	3.05	5.26E-07	VASP	7682	OPA3	69752
chr17	33390652	33390922	3.03	5.40E-07	RFFL	25507	LIG3	83249
chr1	8487639	8487985	2.59	5.47E-07	SLC45A1	103422	RERE	389887
chr16	4664859	4665171	2.66	5.55E-07	C16orf5	-76199	MGRN1	-9811
chr20	47541055	47541331	3.72	5.68E-07	CSE1L	-121645	ARFGEF2	2918
chr8	28232897	28233177	2.51	6.00E-07	ZNF395	10940	PNOC	58388
chr2	37862881	37863162	2.60	6.09E-07	CDC42EP3	36304	QPCT	291269
chr12	26241974	26242233	2.78	6.16E-07	BHLHE41	35899	RASSF8	130135
chr19	52068112	52068398	3.94	6.16E-07	SIGLEC6	-33191	ZNF175	-6276
chr5	106724806	106725117	2.58	6.52E-07	EFNA5	281634		
chr15	95128021	95128339	3.76	6.55E-07	MCTP2	286750		
chr15	74695266	74695625	2.81	6.70E-07	CYP11A1	-35365	SEMA7A	30853
chr1	209846654	209846971	3.98	6.85E-07	G0S2	-1857		
chr16	68504459	68504735	3.02	6.95E-07	KIAA1954	-68590	SMPD3	-22188
chr7	37826921	37827288	3.02	7.16E-07	TXNDC3	-61094	GPR141	47109
chr18	9066822	9067355	3.15	7.85E-07	NDUFV2	-35586	RAB12	457646
chr20	61530410	61530686	3.67	7.91E-07	TCFL5	-37433	DIDO1	38726
chr1	208005215	208005459	3.63	7.98E-07	CD34	79346	CD46	79935
chr19	39893279	39893555	2.61	8.18E-07	G0S24	-4081	ZFP36	-4070
chr10	112263920	112264196	3.21	8.27E-07	SMC3	-63391	DUSP5	6433
chr6	378171	378540	2.53	8.35E-07	IRF4	-13396	DUSP22	86255
chr3	16953971	16954247	3.40	8.45E-07	DAZL	-307103	PLCL2	-20473
chr12	71054719	71054968	3.49	8.80E-07	PTPRB	-23625	PTPRR	259740
chr6	135191795	135192075	3.58	9.30E-07	SGK1	-552739	ALDH8A1	79309
chr16	81771642	81771928	2.87	9.40E-07	PLCG2	-41145	CMIP	293010
chr18	10950923	10951238	3.29	9.41E-07	GNAL	-738055	C18orf58	-60391
chr19	47123034	47123274	2.70	9.79E-07	PTGIR	5200	CALM3	18642
chr3	177063420	177063735	2.83	9.94E-07	TBL1XR1	-148530		
chr9	140632642	140632952	2.12	1.01E-06	CACNA1B	-139444	EHMT1	119353
chr1	230259552	230259858	2.91	1.02E-06	GALNT2	56749	PGBD5	253662
chr2	223792334	223792594	4.11	1.02E-06	KCNE4	-124398	ACSL3	66732
chr1	240851163	240851407	3.98	1.03E-06	GREM2	-75823	RGS7	669193
chr15	70785210	70785486	2.48	1.04E-06	TLE3	-395092	UACA	270502
chr16	21518322	21518641	2.65	1.06E-06	DKFZp547E087	-19492	SLC7A5P1	13283
chr18	56806705	56807022	3.68	1.10E-06	SEC11C	-261		
chr12	40015274	40015590	4.33	1.12E-06	ABCD2	-1589		
chr19	47760038	47760331	2.69	1.19E-06	BBC3	-24162	PRR24	-17957

chr10	125151745	125152074	3.76	1.22E-06	GPR26	-273961	BUB3	238150
chr18	12314668	12314949	2.46	1.27E-06	TUBB6	6552	AFG3L2	62385
chr16	29611472	29611748	2.81	1.29E-06	IMAA	-4901		
chr3	171825356	171825636	2.59	1.38E-06	FNDC3B	68078	GHSR	340707
chr18	56197049	56197474	2.37	1.39E-06	ALPK2	98927	NEDD4L	485643
chr18	60191883	60192276	2.32	1.40E-06	PHLPP1	-190654	ZCCHC2	1422
chr19	50845917	50846195	3.33	1.44E-06	KCNC3	-13422	NAPSB	1949
chr12	72233304	72233719	2.38	1.46E-06	TBC1D15	25		
chr19	16438419	16438734	3.18	1.46E-06	KLF2	2926	EPS15L1	144185
chr21	34641136	34641412	3.17	1.47E-06	IFNAR1	-55940	IFNAR2	39043
chrX	23791737	23792019	3.21	1.48E-06	ACOT9	-30471	SAT1	-9397
chr5	17269382	17269632	2.87	1.50E-06	BASP1	51757		
chr3	53782768	53783064	3.40	1.54E-06	CHDH	97503	CACNA1D	253885
chr7	138794414	138794775	2.54	1.59E-06	ZC3HAV1	-130		
chr3	177069582	177069959	3.26	1.65E-06	TBL1XR1	-154723		
chr15	22899417	22899743	3.67	1.70E-06	CYFIP1	6896	NIPA2	134828
chr16	57073059	57073690	3.14	1.73E-06	NLRC5	49965	FAM192A	146601
chr11	84260343	84260619	3.01	1.75E-06				
chr17	26662425	26662823	3.29	1.84E-06	IFT20	-129	TNFAIP1	76
chr17	73110758	73111043	2.65	1.85E-06	ARMC7	4819	NT5C	16976
chr3	150983719	150984047	2.80	1.96E-06	GPR171	-62895	P2RY14	12347
chr3	71179992	71180268	3.53	1.97E-06	FOXP1	453010		
chr19	44258002	44258333	2.59	2.02E-06	C19orf61	974		
chr14	94442468	94442810	2.93	2.07E-06	OTUB2	-50085	ASB2	-18872
chr4	144280193	144280544	3.20	2.10E-06	SMARCA5	-154247	GAB1	22386
chr2	64955141	64955468	2.87	2.12E-06	SLC1A4	-261151	SERTAD2	-74259
chr12	75724023	75724299	2.29	2.14E-06	GLIPR1L1	-4302	CAPS2	-325
chr11	109870843	109871119	3.14	2.15E-06	ZC3H12C	-93106	C11orf87	578106
chr4	109254731	109255010	4.34	2.22E-06	RPL34	-286851	LEF1	-165293
chr17	79702466	79702750	2.94	2.25E-06	GCGR	-59418	SLC25A10	23237
chr4	124897468	124897776	2.76	2.27E-06	SPRY1	579666		
chr9	130673558	130673829	2.91	2.28E-06	ST6GALNAC6	-11823	ST6GALNAC4	5611
chr19	14317584	14317860	1.95	2.29E-06	LPHN1	-725		
chr8	135701345	135701647	2.57	2.32E-06	ZFAT	23785		
chr9	37382689	37382998	2.76	2.32E-06	GRHPR	-39863	ZCCHC7	262375
chr7	148204221	148204465	2.66	2.41E-06	CUL1	-191590	NM_001126313	72704
chr12	24894253	24894532	3.80	2.43E-06	SOX5	-791756	BCAT1	207915
chr7	114706143	114706428	2.69	2.43E-06	MDFIC	144077	TFEC	964512

chr22	36635513	36635843	2.72	2.44E-06	APOL2	322		
chr2	25640733	25641023	2.49	2.50E-06	DNMT3A	-76104	DTNB	255625
chr11	75946831	75947122	2.82	2.58E-06	WNT11	-29403	PRKRIR	144903
chr7	105449664	105449963	2.54	2.59E-06	FLJ23834	-153843	RINT1	277282
chr7	55584896	55585162	2.55	2.65E-06	VOPP1	55171	LANCL2	151888
chr13	51168576	51168852	2.53	2.78E-06	RNASEH2B	-315178	ST13	422560
chr2	74413096	74413378	2.67	2.87E-06	MTHFD2	-12453	MOBKL1B	-7242
chr15	91293461	91293737	3.16	2.90E-06	FURIN	-118286	BLM	33020
chr1	147806576	147806895	3.15	2.99E-06	NBPF11	-196648	PPIAL4A	148683
chr16	87808350	87808658	2.82	3.04E-06	KLHDC4	-8962	SLC7A5	94596
chr19	39894462	39894929	2.47	3.04E-06	G0S24	-2802	ZFP36	-2791
chr13	66712643	66713107	2.94	3.05E-06				
chr20	30945636	30946103	3.43	3.07E-06	ASXL1	-283		
chr2	225893374	225893659	2.85	3.08E-06	CUL3	-443407	DOCK10	13813
chr19	45256567	45256951	3.07	3.09E-06	CBLC	-24367	BCL3	4781
chr1	16291663	16292120	2.49	3.28E-06	ZBTB17	10735	SPEN	117533
chr11	128338904	128339461	2.60	3.34E-06	ETS1	118270		
chr5	95157713	95157989	2.30	3.53E-06	GLRX	726		
chr2	111966723	111967023	2.75	3.54E-06	BCL2L11	88382	ANAPC1	674868
chr10	37718249	37718541	4.47	3.56E-06	ANKRD30A	303610	ZNF248	428091
chr1	173387388	173387635	2.96	3.57E-06	TNFSF4	-211041	PRDX6	-58974
chr11	109855620	109855896	3.20	3.64E-06	ZC3H12C	-108329	C11orf87	562883
chr17	38755560	38755868	2.46	3.78E-06	CCR7	-33990	SMARCE1	48389
chr2	8442152	8442509	2.37	3.89E-06	ID2	-379653		
chr8	126163982	126164226	3.96	3.89E-06	TRIB1	-278459	NSMCE2	60021
chr2	96811819	96812099	2.28	3.95E-06	DUSP2	-780		
chr5	124271548	124271914	2.20	4.06E-06	ZNF608	-190866		
chr12	105368484	105368758	2.53	4.18E-06	SLC41A2	-46149	ALDH1L2	109720
chr1	67223802	67224193	2.94	4.22E-06	INSL5	42941	SGIP1	224173
chr3	101510795	101511159	3.18	4.22E-06	NFKBIZ	-57381	FAM55C	12948
chr1	220267237	220267513	2.53	4.25E-06	BPNT1	-4184	IARS2	-80
chr9	123693846	123694255	2.43	4.28E-06	TRAF1	-4878		
chr13	28054636	28054887	3.12	4.33E-06	MTIF3	-30051	LNX2	139958
chr11	68138220	68138660	2.39	4.36E-06	SAPS3	-89746	LRP5	58332
chr15	85861553	85861918	3.08	4.54E-06	AKAP13	-62135	PDE8A	336531
chr13	99881036	99881293	2.75	4.63E-06	UBAC2	28486	GPR18	29675
chr4	14179690	14179977	2.96	4.63E-06	CPEB2	-825688	BOD1L	-550506
chr1	171221689	171221999	2.70	4.70E-06	FMO4	-61642	FMO1	4181

chr13	34253320	34253692	2.52	4.78E-06	STARD13	-393605	RFC3	-138700
chr22	37618773	37619049	2.61	4.86E-06	SSTR3	-10558	RAC2	21394
chrX	71526720	71526996	3.85	4.87E-06	CITED1	-1094		
chr4	123553887	123554159	3.44	5.02E-06	BBS12	-99834	IL21	-11812
chr15	80263453	80263714	2.77	5.14E-06	BCL2A1	59		
chr20	46115873	46116335	2.90	5.15E-06	ZMYND8	-130630	NCOA3	-14553
chr1	161509925	161510191	2.84	5.21E-06	FCGR3A	9760	HSPA6	16022
chr21	45510290	45510607	2.49	5.29E-06	PWP2	-16759	TRAPPC10	78243
chr9	114774712	114775027	2.55	5.77E-06	UGCG	115664	SUSD1	162686
chr1	206955807	206956197	2.50	5.84E-06	IL19	-16213	IL10	-10163
chr6	52362369	52362736	2.32	5.95E-06	EFHC1	77559	TRAM2	79309
chr4	86478468	86478867	2.76	5.97E-06	ARHGAP24	82384	MAPK10	797129
chr12	40502051	40502420	3.10	6.05E-06	SLC2A13	-2575		
chr12	48201891	48202167	2.03	6.13E-06	HDAC7	11734	SLC48A1	35062
chr1	235243072	235243348	3.55	6.37E-06	IRF2BP2	-497939	TOMM20	49046
chr11	120215121	120215397	2.67	6.54E-06	GRIK4	-315719	ARHGEF12	7313
chr21	43299041	43299324	2.17	6.57E-06	PRDM15	408		
chr3	171818879	171819292	2.55	6.57E-06	FNDC3B	61668	GHSR	347117
chr7	28194590	28194906	1.91	6.67E-06	JAZF1	25689	TAX1BP1	415010
chr17	76333575	76333920	2.41	6.93E-06	SOCS3	22410	LOC283999	106357
chr15	85523509	85523819	2.04	7.17E-06	PDE8A	-1541		
chr14	87533455	87533751	3.34	7.71E-06	GALC	926304		
chr19	14629452	14629747	2.33	8.14E-06	DNAJB1	-399		
chr3	30681482	30681753	3.02	8.16E-06	TGFBR2	33624	GADL1	254535
chr2	238777679	238777923	2.94	8.67E-06	UBE2F	-97899	RAMP1	9614
chr2	172176006	172176303	2.61	8.78E-06	DCAF17	-114704	TLK1	-88331
chr3	13125425	13125788	2.16	8.86E-06	IQSEC1	-10990	NUP210	336202
chr20	24405980	24406421	3.35	9.14E-06	GGTLC1	-436785	TMEM90B	-43634
chr16	27210196	27210440	3.11	9.16E-06	NSMCE1	69795		
chr17	12334654	12334938	3.28	9.41E-06	MYOCD	-234411	MAP2K4	410661
chr3	18464860	18465143	2.23	9.42E-06	TBC1D5	-680762	SATB1	15250
chr3	143747664	143747940	2.40	9.42E-06	C3orf58	57162		
chr6	116340730	116340978	3.06	9.53E-06	FRK	41067		
chr12	108962635	108962881	2.87	9.55E-06	ISCU	6464	TMEM119	29136
chr4	55181759	55182055	3.73	1.02E-05	KIT	-342188	PDGFRA	86643
chr12	11899031	11899391	2.74	1.05E-05	BCL2L14	-324667	ETV6	96423
chr11	57192237	57192541	2.37	1.06E-05	PRG3	-43766	SLC43A3	2664
chr13	42951889	42952204	2.79	1.06E-05	TNFSF11	-196244	AKAP11	105758

chr11	65262035	65262279	3.38	1.07E-05	SCYL1	-30391	FRMD8	108116
chr15	52240926	52241213	2.61	1.10E-05	LEO1	22888	TMOD3	119181
chr16	31885779	31886055	2.39	1.11E-05	ZNF267	838		
chr19	18485205	18485470	2.42	1.12E-05	GDF15	-11630	PGPEP1	33930
chr9	114790934	114791345	2.30	1.15E-05	UGCG	131934	SUSD1	146416
chr7	125301877	125302134	2.86	1.20E-05	POT1	-731969		
chr15	99143155	99143431	4.12	1.24E-05	IGF1R	-49468		
chr1	179050853	179051181	2.19	1.25E-05	TOR3A	-95		
chr15	81316109	81316362	2.87	1.28E-05	IL16	-172983	MESDC2	-34031
chr19	50528496	50529123	3.47	1.28E-05	ZNF473	-402	VRK3	-5
chr22	37838564	37838935	3.66	1.28E-05	ELFN2	-15245	MFNG	43635
chr11	65192665	65192939	2.25	1.29E-05	SCYL1	-99746	FRMD8	38761
chr7	101855189	101855443	2.50	1.32E-05	SH2B2	-73089	CUX1	394434
chr1	199010627	199010996	2.74	1.34E-05	NR5A2	-985958	PTPRC	402675
chr12	93561641	93561887	3.01	1.34E-05	EEA1	-238657	NUDT4	-209937
chr1	207103751	207104035	2.21	1.36E-05	FAIM3	-8515	PIGR	15918
chr17	76272592	76272846	2.33	1.36E-05	LOC283999	45328	SOCS3	83439
chr19	5974904	5975373	2.33	1.41E-05	RANBP3	3181	CAPS	60946
chr18	52901917	52902193	2.28	1.44E-05	TCF4	353805	RAB27B	406215
chr11	9738359	9738638	2.65	1.63E-05	SWAP70	52871	SBF2	577255
chr11	18790023	18790316	2.94	1.64E-05	PTPN5	23219	TMEM86A	69819
chr19	54975277	54975785	2.06	1.64E-05	LENG9	-637		
chr2	170998455	170998728	2.12	1.65E-05	MYO3B	-36063	UBR3	314574
chr4	87919230	87919633	2.43	1.68E-05	SLC10A6	-149016	AFF1	-8721
chr4	170332012	170332259	2.66	1.68E-05	SH3RF1	-139887	NEK1	201478
chr17	16891409	16891783	2.63	1.70E-05	KIAA0864	-176378	TNFRSF13B	-16194
chr5	4879857	4880133	2.65	1.73E-05	ADAMTS16	-260448		
chr21	35392317	35392611	2.33	1.75E-05	SLC5A3	-53359	ITSN1	377680
chr3	50628266	50628572	3.05	1.96E-05	CISH	20843	HEMK1	21510
chr3	185902925	185903293	2.58	1.97E-05	ETV5	-76208	DGKG	176914
chr6	11730876	11731244	2.29	1.97E-05	C6orf105	48220	TMEM170B	192549
chr12	48591845	48592136	2.22	1.99E-05	OR10AD1	5084	C12orf68	14625
chr17	37914201	37914546	2.46	1.99E-05	GRB7	20187	IKZF3	106067
chr9	92761994	92762297	2.99	1.99E-05	GADD45G	542219	DIRAS2	642962
chr17	48226225	48226501	2.33	2.00E-05	PPP1R9B	1514	PDK2	53667
chr22	27006351	27006603	2.86	2.00E-05	TPST2	-45107	CRYBB1	7514
chr2	24299098	24299490	3.14	2.04E-05	SF3B14	20		
chr1	150535239	150535588	4.93	2.07E-05	ADAMTSL4	13516	MCL1	16722

chr13	100028387	100028660	2.23	2.10E-05	TM9SF2	-125204	GPR183	-68775
chrX	41301603	41301867	2.34	2.10E-05	NYX	-4978		
chr15	85855701	85855961	2.88	2.12E-05	AKAP13	-68040	PDE8A	330626
chr1	158985343	158985740	2.40	2.15E-05	IFI16	5860	AIM2	61105
chr15	74677434	74677678	2.26	2.17E-05	CYP11A1	-17475	SEMA7A	48743
chr18	55887673	55887974	2.86	2.21E-05	NEDD4L	176205	ALPK2	408365
chr2	240322602	240322978	1.98	2.21E-05	HDAC4	-147		
chr17	78237237	78237527	2.01	2.29E-05	RNF213	-76344	SLC26A11	43145
chr12	96883149	96883459	2.47	2.31E-05	NEDD1	-417697	PCTK2	-89081
chr4	124246415	124246725	2.86	2.33E-05	SPRY1	-71386	SPATA5	402345
chr11	33964650	33964954	2.97	2.35E-05	CAPRIN1	-108428	LMO2	-50966
chr1	231748526	231748802	2.44	2.43E-05	DISC1	-13897	TSNAX	84265
chr8	81260150	81260410	2.62	2.44E-05	TPD52	-267270	ZBTB10	-138168
chr14	21776900	21777176	2.27	2.54E-05	RPGRIP1	20902	SUPT16H	75387
chr19	3721399	3721692	1.98	2.55E-05	PIP5K1C	-21101	TJP3	-6828
chr7	73157287	73157577	2.03	2.59E-05	ABHD11	-4242		
chr16	87958135	87958473	2.50	2.62E-05	SLC7A5	-55204	CA5A	11808
chr22	35767910	35768160	2.91	2.64E-05	HMOX1	-9052	TOM1	72238
chr14	70163614	70163890	4.21	2.71E-05	SFRS5	-70082	KIAA0247	85442
chr22	29188172	29188448	2.42	2.80E-05	XBP1	8250	HSCB	50267
chr1	212781899	212782205	2.47	2.85E-05	ATF3	43355	BATF3	91275
chr20	16555414	16555690	1.92	2.89E-05	KIF16B	-1474		
chr2	179886051	179886335	2.64	2.90E-05	CCDC141	-136374	SESTD1	243157
chr14	65770449	65771019	2.77	2.91E-05	MAX	-201507	FUT8	-108801
chr12	1663484	1663955	2.10	2.93E-05	FBXL14	39611	ERC1	563316
chr11	76385000	76385285	2.16	3.06E-05	LRRC32	-4099		
chr12	11883824	11884077	2.88	3.06E-05	BCL2L14	-339927	ETV6	81163
chr3	188107747	188108070	2.20	3.09E-05	TPRG1	-781854	LPP	177188
chr3	195906437	195907150	2.48	3.09E-05	TFRC	-97762	ZDHHC19	31506
chr17	73031078	73031378	2.45	3.12E-05	ATP5H	11846	ICT1	22448
chr13	69162845	69163108	2.39	3.13E-05				
chr13	100153431	100153786	2.92	3.14E-05	TM9SF2	-119		
chr15	85524444	85524765	2.10	3.17E-05	PDE8A	-600		
chr19	56116663	56117003	1.90	3.19E-05	ZNF524	5103	ZNF784	19108
chr16	11082260	11082504	3.07	3.25E-05	CIITA	111327	SOCS1	267657
chrX	24531606	24531880	4.22	3.27E-05	PDK3	48399	PCYT1B	133712
chr11	18415887	18416168	2.53	3.32E-05	LDHA	92		
chr7	105925647	105926006	2.13	3.37E-05	NAMPT	-189		

chr19	45542568	45542844	2.44	3.38E-05	SFRS16	408		
chr3	61623108	61623431	2.42	3.40E-05	PTPRG	76027	FEZF2	735920
chr8	74903416	74903715	2.69	3.40E-05	LY96	-21		
chr20	47463457	47463767	3.00	3.42E-05	ARFGEF2	-74663	PREX1	-19192
chr19	48972031	48972342	2.21	3.44E-05	CYTH2	-278		
chr21	42734285	42734566	2.97	3.44E-05	MX2	476		
chr22	24407502	24407957	2.33	3.45E-05	CABIN1	-35		
chr17	76254665	76254945	3.46	3.47E-05	LOC283999	27414	SOCS3	101353
chr18	72728042	72728302	3.28	3.47E-05	ZADH2	193109	ZNF407	385249
chr1	150459561	150459894	2.89	3.50E-05	TARS2	-192		
chr4	10724701	10724983	2.81	3.69E-05	CLNK	-38456	HS3ST1	705695
chr8	103818542	103818832	2.27	3.70E-05	KLF10	-150704	AZIN1	57710
chr12	96972356	96972632	3.39	3.72E-05	NEDD1	-328507	PCTK2	-178271
chr3	183299565	183299899	2.84	3.75E-05	KLHL24	-53679	KLHL6	-26233
chr9	38319073	38319408	2.28	3.81E-05	SHB	-250031	ALDH1B1	-73461
chr15	63796576	63796886	2.38	3.82E-05	USP3	-79		
chr19	52074238	52074616	2.49	3.82E-05	ZNF175	-104		
chr6	27560449	27560754	2.79	3.84E-05	ZNF184	-119705	HIST1H2BL	215107
chr15	91191854	91192142	2.64	3.97E-05	BLM	-68581	CRTC3	118800
chr19	42633490	42634077	5.05	3.97E-05	POU2F2	2846	ZNF574	53494
chr10	31389998	31390279	3.48	3.98E-05	ZEB1	-217962	ZNF438	-69273
chr2	196426087	196426331	2.72	4.01E-05	SLC39A10	-95323		
chr22	49776313	49776787	2.46	4.16E-05	BRD1	441902	FAM19A5	891262
chr9	131902270	131902592	2.26	4.20E-05	METTL11A	-486004	PPP2R4	29187
chr12	26676709	26676971	2.55	4.24E-05	ITPR2	309291	SSPN	328334
chr11	67203880	67204257	3.07	4.27E-05	PTPRCAP	1084	RPS6KB2	8134
chr13	41350894	41351170	2.25	4.41E-05	SLC25A15	-12515	MRPS31	-5685
chr17	29035763	29036089	1.82	4.52E-05	LRRC37B2	100478	CRLF3	115852
chr21	30257362	30257858	2.61	4.55E-05	N6AMT1	83		
chr7	89872280	89872526	2.88	4.56E-05	C7orf63	-2085		
chr2	237478167	237478512	2.63	4.58E-05	CXCR7	-40		
chr1	237899998	237900242	4.18	4.65E-05	ZP4	153815	RYR2	694418
chr1	149982559	149982835	2.19	4.91E-05	OTUD7B	-11		
chr19	54974914	54975238	1.88	4.96E-05	LENG9	-182		
chr17	1620792	1621068	2.55	5.03E-05	PRPF8	-32754	SERPINF2	-25200
chr9	139796926	139797244	2.17	5.10E-05	TRAF2	16120	FBXW5	42088
chr1	157536414	157536704	2.48	5.22E-05	FCRL5	-14249	FCRL4	31311
chr5	133890151	133890417	2.24	5.45E-05	PHF15	28486	SAR1B	78243

chr15	91330079	91330385	2.58	5.50E-05	FURIN	-81653	BLM	69653
chr22	34317106	34317382	2.49	5.51E-05	LARGE	-828		
chr15	81594207	81594490	2.18	5.59E-05	STARD5	22175	IL16	105130
chr19	42625835	42626168	2.05	5.60E-05	POU2F2	10628	ZNF574	45712
chr11	128627789	128628059	2.58	5.63E-05	FLI1	64111	KCNJ1	84439
chr12	14547365	14547649	2.18	5.63E-05	ATF7IP	28896	PLBD1	173284
chr3	15847302	15847580	2.79	5.63E-05	ANKRD28	53612	BTD	204186
chr14	61969946	61970314	2.55	5.64E-05	HIF1A	-191989	PRKCH	181615
chr1	185308407	185308666	2.56	5.86E-05	HMCN1	-395146	IVNS1ABP	-22076
chr14	95731875	95732253	2.59	5.97E-05	DICER1	-123979	CLMN	54181
chr19	38754267	38754511	3.57	6.08E-05	SPINT2	-813		
chr11	125773006	125773255	2.35	6.09E-05	DDX25	-1141	PUS3	-15
chr5	17257900	17258170	2.43	6.28E-05	BASP1	40285		
chr2	25502153	25502444	1.95	6.33E-05	POMC	-110740	DNMT3A	62475
chr3	36953966	36954338	2.47	6.35E-05	TRANK1	-51741	EPM2AIP1	80643
chr1	145455343	145455679	2.17	6.42E-05	RBM8A	-52127	TXNIP	17049
chr13	100089167	100089411	2.01	6.48E-05	GPR183	-129540	TM9SF2	-64439
chr1	87264630	87264888	2.45	6.49E-05	SH3GLB1	94502	SEP15	115348
chr3	169772998	169773398	2.54	6.60E-05	GPR160	17463	PHC3	126339
chr9	101892400	101892693	2.46	6.60E-05	TGFBR1	25135	ALG2	91699
chr2	17994966	17995316	2.63	6.66E-05	MSGN1	-2645		
chr8	66989984	66990236	3.37	6.77E-05	TRIM55	-49168	DNAJC5B	56319
chr2	192482326	192482656	2.66	6.91E-05	OBFC2A	-60370	MYO1B	372384
chr3	186761900	186762400	2.32	6.91E-05	ST6GAL1	22485	RPL39L	95113
chrX	150565389	150565760	2.09	6.91E-05	VMA21	-130		
chr13	48760494	48760738	3.55	6.93E-05	MED4	-91376	ITM2B	-46658
chr16	89126721	89126977	2.25	6.93E-05	CBFA2T3	-83448	ACSF3	-33405
chr12	102155243	102155487	3.69	6.94E-05	SYCP3	-22118	GNPTAB	69267
chr17	36857204	36857452	2.90	7.01E-05	MLLT6	-4545		
chr18	56325450	56325786	4.12	7.09E-05	ALPK2	-29429	MALT1	-13000
chr15	63773328	63773590	3.21	7.10E-05	CA12	-99384	USP3	-23351
chr20	48552634	48552921	1.82	7.13E-05	RNF114	-136		
chr3	170074062	170074331	2.11	7.27E-05	SKIL	-1276		
chr13	115079733	115080074	2.22	7.40E-05	ZNF828	-61		
chr1	235114579	235114867	2.19	7.48E-05	IRF2BP2	-369452	TOMM20	177533
chr3	176914555	176914935	2.28	7.50E-05	TBL1XR1	303		
chr3	127609473	127609728	3.19	7.55E-05	MGLL	-67550	KBTBD12	-32301
chr3	150863869	150864207	2.17	7.71E-05	GPR171	56950	MED12L	59453

chr3	150484135	150484538	2.61	7.83E-05	SIAH2	-3074		
chr2	31479025	31479415	2.33	7.85E-05	EHD3	22340	XDH	158391
chr12	95114990	95115266	2.50	7.89E-05	TMCC3	-70804	NDUFA12	282383
chr2	136891619	136892057	2.50	7.89E-05	THSD7B	-856624	CXCR4	-16113
chr18	55908523	55908799	3.12	7.95E-05	NEDD4L	197042	ALPK2	387528
chr6	134568664	134568942	2.64	7.98E-05	SLC2A12	-195014	SGK1	70393
chr3	5020288	5020574	2.57	7.99E-05	BHLHE40	-666		
chrX	53741741	53742047	2.69	8.06E-05	HUWE1	-28221	PHF8	328713
chr2	97303993	97304303	2.76	9.26E-05	FER1L5	-4426		
chr18	56613474	56613723	2.07	9.33E-05	SEC11C	-193526	ZNF532	83538
chr3	150811780	150812069	2.11	9.33E-05	MED12L	7340	GPR171	109063
chr13	22009997	22010341	2.59	9.49E-05	SKA3	-259459	ZDHHC20	23254
chr3	159706361	159706770	2.52	9.49E-05	IL12A	-63		
chr9	137283737	137284078	2.25	9.67E-05	COL5A1	-249744	RXRA	65592
chr5	88030121	88030378	2.84	9.68E-05	TMEM161B	-465585	MEF2C	149052
chr11	82904586	82904874	2.22	9.71E-05	PCF11	36593	CCDC90B	92647
chr9	33988554	33988804	2.95	9.75E-05	DCAF12	138092	UBE2R2	171497
chr4	71544158	71544434	2.46	1.01E-04	IGJ	-11948	UTP3	-9900
chr18	54305733	54306204	3.02	1.01E-04	TXNL1	301		
chr13	50265515	50265791	2.04	1.02E-04	EBPL	-30		
chr22	40988805	40989069	2.50	1.03E-04	MKL1	43753	SGSM3	222342
chr19	55896879	55897574	2.52	1.05E-04	RPL28	-73		
chr17	57915137	57915447	2.46	1.05E-04	TUBD1	55004	TMEM49	130429
chr3	37949304	37949726	3.10	1.06E-04	VILL	-85563	CTDSPL	45846
chr2	10054829	10055094	2.51	1.06E-04	GRHL1	-36830	TAF1B	71391
chr17	35767201	35767477	2.15	1.08E-04	ACACA	-437	TADA2A	27
chr6	53096537	53096788	3.13	1.08E-04	GCM1	-83039	ELOVL5	117279
chr3	136471129	136471514	2.53	1.10E-04	STAG1	-77		
chr7	138818353	138818636	1.97	1.12E-04	TTC26	5		
chr11	76758649	76758976	2.25	1.12E-04	CAPN5	-19179	B3GNT6	13378
chr14	35873004	35873290	2.06	1.12E-04	NFKBIA	813		
chr11	43902142	43902393	1.87	1.14E-04	ALKBH3	-89		
chr1	20452609	20452886	2.41	1.14E-04	PLA2G2F	-13075	PLA2G2D	-6740
chr22	38857585	38857876	2.09	1.15E-04	KCNJ4	-17698	KDELR3	-6352
chr18	42339336	42339614	2.40	1.16E-04	SLC14A2	-855291	SETBP1	78612
chr2	217087847	217088109	2.72	1.16E-04	XRCC5	113958	MARCH4	148772
chr18	56431567	56431843	2.23	1.19E-04	ZNF532	-98356	MALT1	93087
chr12	92458870	92459162	2.13	1.20E-04	DCN	-885657	BTG1	80657

chr15	101667708	101667984	2.24	1.20E-04	CHSY1	124280	LRRK1	208386
chr15	31645543	31645965	2.21	1.21E-04	KLF13	26671	OTUD7A	301788
chr11	76385698	76386048	2.29	1.21E-04	LRRC32	-4829		
chr3	176919502	176919800	2.22	1.21E-04	TBL1XR1	-4603		
chr19	47791700	47791955	2.08	1.22E-04	C5AR1	-21276	PRR24	13686
chr19	52207430	52207738	2.21	1.23E-04	SIGLEC14	-57452	HAS1	19637
chr1	178575179	178575489	2.28	1.23E-04	RALGPS2	-118966	C1orf49	93122
chr11	85925832	85926144	3.23	1.25E-04	PICALM	-145880	EED	-29827
chr15	80269840	80270102	2.36	1.25E-04	ZFAND6	-82050	BCL2A1	-6328
chr2	38949925	38950169	2.40	1.26E-04	SFRS7	28589	GALM	56995
chr7	142960429	142960725	2.06	1.28E-04	GSTK1	55		
chr8	116439901	116440250	2.15	1.29E-04	TRPS1	241152		
chr14	91830492	91830838	2.20	1.30E-04	GPR68	-110441	CCDC88C	53468
chr2	20251709	20251966	2.08	1.31E-04	LAPTM4A	-49		
chr16	84122355	84122683	1.95	1.32E-04	SLC38A8	-46757	MBTPS1	27998
chr14	75959850	75960215	2.22	1.34E-04	BATF	-28751	JDP2	61196
chr3	156892542	156892923	3.89	1.34E-04	PTX3	-261847	CCNL1	-14251
chr17	36858317	36858658	2.18	1.35E-04	MLLT6	-3385		
chr1	224390950	224391199	2.80	1.36E-04	DEGS1	20147	NVL	126797
chr11	123941665	123942007	3.49	1.39E-04	X64983	-114515	OR10G7	-32128
chr17	74260741	74261093	2.15	1.40E-04	RNF157	-24527	QRICH2	42844
chr19	41035010	41035329	1.96	1.43E-04	SHKBP1	-47587	SPTBN4	62044
chr8	19674610	19675019	2.54	1.43E-04	INTS10	-103		
chr8	30601796	30602072	2.18	1.45E-04	UBXN8	244		
chr9	137282481	137282798	2.66	1.45E-04	COL5A1	-251012	RXRA	64324
chr2	98643539	98643827	2.30	1.47E-04	CNGA3	-318935	TMEM131	-31329
chr11	120796705	120796994	2.79	1.49E-04	TBCEL	-97963	GRIK4	265872
chr2	196424302	196424676	2.33	1.49E-04	SLC39A10	-97043		
chr3	19988397	19988700	1.99	1.54E-04	RAB5A	-23		
chr1	154973227	154973537	1.92	1.56E-04	ZBTB7B	-1730		
chr17	76352510	76352802	2.11	1.56E-04	SOCS3	3502	LOC283999	125265
chr16	23706298	23706628	2.49	1.57E-04	PLK1	16262	ERN2	18358
chr2	20565074	20565422	2.82	1.58E-04	RHOB	-81587	PUM2	-38109
chr2	25642595	25642871	2.40	1.61E-04	DNMT3A	-77959	DTNB	253770
chr1	145382671	145383082	2.97	1.61E-04	HFE2	-30314	NBPF10	89506
chr20	37501713	37502053	2.28	1.63E-04	FAM83D	-53072	PPP1R16B	67535
chr2	191878853	191879311	2.25	1.69E-04	STAT1	-106		
chr9	114794447	114794733	2.45	1.70E-04	UGCG	135384	SUSD1	142966

chr15	74286735	74287113	1.97	1.77E-04	STOML1	-2289	PML	-90
chr8	107072665	107072958	2.50	1.81E-04	OXR1	-209661	ZFPM2	741665
chr4	40187375	40187651	2.35	1.83E-04	ACOT7L	-128694	RHOH	-11014
chr18	56448828	56449104	2.34	1.85E-04	ZNF532	-81095	MALT1	110348
chr15	57591995	57592275	2.36	1.86E-04	CGNL1	-76570	TCF12	381302
chr5	139017055	139017338	2.11	1.89E-04	CXXC5	-11104	UBE2D2	76446
chr9	124412539	124412815	2.94	1.90E-04	DAB2IP	83278	TTLL11	443208
chr19	49866797	49867151	1.99	1.92E-04	TEAD2	-1260	DKKL1	-68
chr19	7198061	7198314	1.79	1.93E-04	INSR	95823	ZNF557	128717
chr1	16292543	16292887	2.22	1.94E-04	ZBTB17	9912	SPEN	118356
chr1	145507294	145507677	2.24	1.95E-04	RBM8A	-152		
chr7	138803748	138804022	1.83	1.96E-04	TTC26	-14605	ZC3HAV1	-9420
chr5	115912778	115913054	2.88	1.99E-04	SEMA6A	-2365		
chr20	37678743	37679024	2.50	2.01E-04	DHX35	87884		
chr3	186251911	186252187	2.34	2.03E-04	DGKG	-172026	CRYGS	10118
chr9	140117878	140118192	1.89	2.03E-04	RNF208	-2260		
chr7	22925574	22925891	1.94	2.03E-04	TOMM7	-63312	FAM126A	128037
chr17	19659170	19659463	3.03	2.04E-04	ALDH3A1	-10545	ULK2	111922
chr9	37197143	37197486	2.02	2.07E-04	GRHPR	-225392	ZCCHC7	76846
chr11	117686050	117686466	2.21	2.09E-04	DSCAML1	-18282	FXYD2	9201
chr8	9008062	9008338	1.97	2.10E-04	PPP1R3B	20		
chr4	40517379	40517655	2.29	2.11E-04	RBM47	114366	CHRNA9	180048
chr5	123370612	123370949	2.34	2.12E-04	CSNK1G3	522988	ZNF608	710084
chr13	109814553	109814879	2.30	2.12E-04	MYO16	566216	IRS2	624198
chr3	186759656	186760103	2.36	2.13E-04	ST6GAL1	20215	RPL39L	97383
chr3	72213543	72213825	2.01	2.14E-04	PROK2	-379327	RYBP	282090
chr3	150317403	150317670	2.67	2.22E-04	SELT	-3529		
chr11	73702386	73703003	2.27	2.22E-04	UCP2	-8806	UCP3	17587
chr14	60794170	60794565	2.12	2.24E-04	PPM1A	81898	C14orf39	158396
chrX	53122380	53122658	2.25	2.24E-04	TSPYL2	10970	KDM5C	132085
chr22	29138452	29138970	2.30	2.26E-04	CHEK2	-889	HSCB	668
chr1	145395448	145395742	2.36	2.27E-04	HFE2	-17596	NBPF10	102224
chr11	60677683	60678045	2.02	2.33E-04	PRPF19	-3803	TMEM109	-3507
chr2	24800377	24800673	2.92	2.34E-04	ITSN2	-217128	NCOA1	-6820
chr11	47236379	47236674	2.05	2.35E-04	DDB2	34		
chr1	53144830	53145128	1.93	2.44E-04	C1orf163	19059	FAM159A	45913
chr3	112532809	112533105	2.84	2.44E-04	CCDC80	-172980	CD200R1L	31840
chr7	70226778	70227071	2.65	2.44E-04	WBSCR17	-370864		

chr19	50400130	50400572	2.04	2.49E-04	IL4I1	-204				
chr4	37140009	37140271	3.09	2.49E-04	RELL1	547859	DTHD1	856896		
chr5	126112279	126112610	2.14	2.49E-04	LMNB1	-388				
chr16	21566123	21566445	2.74	2.51E-04	SLC7A5P1	-34519	IGSF6	97688		
chr19	5827874	5828252	1.62	2.51E-04	NRTN	4245	FUT6	11679		
chr2	8692637	8693051	2.36	2.52E-04	ID2	-129140				
chr5	110087960	110088303	2.72	2.52E-04	TSLP	-319258	SLC25A46	13378		
chr3	9210046	9210290	2.39	2.61E-04	RAD18	-205022	SRGAP3	81143		
chr7	155534537	155534838	1.97	2.62E-04	SHH	70279	RBM33	97485		
chr2	55459577	55459933	2.39	2.65E-04	C2orf63	-306	RPS27A	690		
chr2	17935180	17935463	1.81	2.68E-04	SMC6	-226	GEN1	145		
chr19	44269609	44269950	2.14	2.68E-04	C19orf61	-10638	KCNN4	15629		
chr13	50705316	50705597	2.12	2.68E-04	ST13	-40697	KCNRG	116067		
chr10	104153742	104154018	2.04	2.69E-04	NFKB2	-459				
chr3	13053084	13053399	2.01	2.82E-04	RPL32	-171293	IQSEC1	61375		
chr2	239762938	239763182	2.30	2.86E-04	TWIST2	6334	HDAC4	559583		
chr14	103993757	103993999	2.57	2.88E-04	CKB	-4708	TRMT61A	-1631	TRMT61A	-1631
chr15	31558605	31558938	2.09	2.90E-04	TRPM1	-164848	KLF13	-60311		
chr9	136651561	136651868	1.93	2.97E-04	SARDH	-48236	VAV2	205731		
chr16	78133276	78133586	2.31	2.98E-04	WWOX	-120				
chr4	2747919	2748167	1.98	3.04E-04	TNIP2	10060	RNF4	277236		
chr4	140222435	140222725	1.85	3.05E-04	NARG1	-96				
chr18	56563565	56563882	2.17	3.10E-04	SEC11C	-243401	ZNF532	33663		
chr9	112736728	112737034	1.99	3.17E-04	PALM2-AKAP2	194304	TXN	281897		
chr9	37403095	37403522	2.64	3.19E-04	GRHPR	-19398	ZCCHC7	282840		
chr2	194340882	194341158	2.53	3.22E-04						
chr22	34317843	34318119	2.61	3.35E-04	LARGE	-1565				
chr19	44037339	44037595	1.78	3.40E-04	ZNF575	127				
chr1	153935883	153936162	2.11	3.50E-04	CRTC2	-4980	CREB3L4	-4374		
chr19	6244017	6244293	2.42	3.64E-04	MLLT1	35804	ACSBG2	108445		
chr8	1904145	1904389	3.01	3.72E-04	KBTBD11	-17777	ARHGEF10	132118		
chr6	7313354	7313713	2.21	3.74E-04	SSR1	7				
chr4	103748939	103749550	2.40	3.89E-04	UBE2D3	-537				
chr8	53206801	53207060	2.49	3.96E-04	PCMTD1	-395196	ST18	115508		
chr2	11893655	11894000	2.29	3.99E-04	TRIB2	-963170	LPIN1	7088		
chr1	203259424	203259940	2.32	4.06E-04	CHIT1	-60822	BTG2	-14982		
chr7	116660104	116660348	2.60	4.08E-04	ST7	66845	WNT2	303117		
chr8	131054592	131055005	2.19	4.09E-04	GSDMC	-255665	ASAP1	359417		

chr15	52554100	52554350	2.22	4.14E-04	GNB5	-70660	MYO5C	33745
chr3	141217697	141217995	2.08	4.15E-04	RNF7	-239205	RASA2	11920
chr6	12011378	12011783	2.03	4.25E-04	HIVEP1	-1143		
chr7	116223805	116224049	3.18	4.25E-04	MET	-88532	CAV1	59088
chr17	4278290	4278741	2.24	4.26E-04	SPNS3	-58703	UBE2G1	-8547
chr12	91775165	91775441	2.74	4.32E-04	DCN	-201944	BTG1	764370
chr16	11391374	11391731	2.37	4.33E-04	C16orf75	-47758	PRM1	-16361
chr6	154674062	154674378	2.95	4.34E-04	IPCEF1	3680	OPRM1	313777
chr11	128159960	128160380	2.24	4.35E-04	ETS1	297283		
chr9	96725250	96725542	1.91	4.35E-04	PTPDC1	-121350	BARX1	-7788
chr7	50308605	50308913	2.19	4.42E-04	ZPBP	-175899	IKZF1	-35619
chr2	45957102	45957385	2.10	4.42E-04	EPAS1	-567319	PRKCE	78201
chr11	115093871	115094147	2.38	4.44E-04	CADM1	281232	FAM55B	544809
chr11	65355636	65356020	1.77	4.45E-04	KCNK7	7639	SSSCA1	17885
chr1	45276081	45276391	1.81	4.46E-04	BTBD19	2082	PTCH2	32380
chr4	8393743	8394012	2.04	4.50E-04	ACOX3	48574	HTRA3	122386
chr5	32583741	32583990	2.03	4.58E-04	SUB1	-1739		
chr13	48878677	48879012	2.40	4.63E-04	RB1	962		
chr3	67050441	67050769	2.07	4.64E-04	KBTBD8	1878	SUCLG2	654433
chr15	78441334	78441622	1.78	4.65E-04	IDH3A	-241		
chr3	157147562	157147824	2.97	4.66E-04	CCNL1	-269211	PTX3	-6887
chr1	9486340	9486686	2.17	4.66E-04	SLC25A33	-113015	SPSB1	133572
chr11	128421735	128422158	2.29	4.75E-04	ETS1	35506		
chr19	16413155	16413413	2.64	4.76E-04	KLF2	-22367	AP1M1	104619
chr19	12902016	12902428	1.99	4.77E-04	JUNB	-88		
chr18	23795638	23795966	2.03	4.77E-04	TAF4B	-10607	PSMA8	81986
chr13	100195088	100195585	2.30	4.78E-04	CLYBL	-63599	TM9SF2	41609
chr2	176033408	176033688	2.48	4.80E-04	ATF2	-651		
chr14	59642183	59642485	2.40	4.82E-04	DAAM1	-13065	DACT1	537577
chr3	106021412	106021657	2.79	5.04E-04	CBLB	-433648		
chr1	174126371	174126616	2.26	5.05E-04	RABGAP1L	-2140		
chr19	16189680	16189979	2.14	5.13E-04	RAB8A	-32660	TPM4	11513
chr11	62389486	62389873	2.07	5.16E-04	B3GAT3	-232		
chr12	77171504	77171780	2.02	5.16E-04	ZDHHC17	13788	CSRP2	101157
chr3	185960388	185960664	2.75	5.22E-04	ETV5	-133625	DGKG	119497
chr1	8763370	8763658	1.50	5.23E-04	RERE	114185	SLC45A1	379124
chr2	101769340	101769576	2.46	5.28E-04	TBC1D8	-1612		
chr13	99626341	99626633	3.16	5.37E-04	SLC15A1	-221558	DOCK9	112173

chr19	37288171	37288475	2.24	5.40E-04	BC052603	-24607	ZNF790	40961
chr6	27125726	27126100	2.11	5.52E-04	PRSS16	-89595	HIST1H2AH	11005
chr20	49186984	49187250	2.17	5.52E-04	PARD6B	-160964	PTPN1	60226
chr12	125264329	125264670	1.86	5.57E-04	NCOR2	-244343	SCARB1	84019
chr13	91709108	91709372	2.70	5.58E-04	GPC5	-341695		
chr1	2322936	2323212	1.83	5.65E-04	RER1	-140		
chr1	25348771	25349242	2.20	5.78E-04	RUNX3	-92237	SYF2	210006
chr20	43331467	43331880	1.96	5.78E-04	ADA	-51298	WISP2	-12211
chr17	37911036	37911331	2.33	5.97E-04	GRB7	16997	IKZF3	109257
chr19	1038120	1038564	2.09	6.10E-04	ABCA7	-1760		
chr7	64146884	64147228	2.11	6.21E-04	ZNF138	-107715	ZNF107	20545
chr11	35187538	35187895	2.57	6.31E-04	CD44	27300	SLC1A2	253388
chr17	72510506	72510775	2.27	6.33E-04	CD300LB	16972	CD300A	48119
chr2	100851561	100852000	1.96	6.33E-04	AFF3	-129736	LONRF2	87414
chr5	118754485	118754754	2.29	6.34E-04	HSD17B4	-33528	TNFAIP8	63024
chr16	22218427	22218766	1.95	6.40E-04	POLR3E	-90144	EEF2K	1005
chr3	159640866	159641157	2.59	6.41E-04	IL12A	-65617	SCHIP1	649468
chr17	38771460	38771792	1.95	6.51E-04	CCR7	-49902	SMARCE1	32477
chr17	76640575	76640819	2.19	6.51E-04	DNAH17	-73293	CYTH1	137679
chr2	68917067	68917457	2.33	6.55E-04	ARHGAP25	-44706	PROKR1	44468
chrX	1599679	1600148	2.29	6.61E-04	ASMTL	-28070	P2RY8	56123
chr3	186244136	186244383	1.97	6.61E-04	DGKG	-164237	CRYGS	17907
chr12	53297179	53297455	2.67	6.62E-04	KRT78	-54539	KRT8	1551
chr14	76006510	76006765	2.14	6.68E-04	FLVCR2	-38302	BATF	17854
chr7	44530315	44530681	2.05	6.72E-04	CAMK2B	-165268	NPC1L1	50416
chr7	45020088	45020415	2.00	6.72E-04	MYO1G	-1548		
chr1	206291766	206292103	2.44	6.72E-04	C1orf186	-3288		
chr19	52110332	52110608	2.03	6.74E-04	hCG_2008157	-12840	SIGLEC5	23257
chr7	26004139	26004614	2.10	6.76E-04	NPVF	-736272	NFE2L3	-187470
chr11	62380421	62380708	2.04	6.91E-04	EML3	-328	ROM1	352
chr17	79450301	79450545	2.05	6.95E-04	ACTG1	29404	BAHCC1	76883
chr1	27021988	27022346	1.97	7.13E-04	ARID1A	-355		
chr20	48236835	48237261	2.34	7.26E-04	PTGIS	-52341	B4GALT5	93373
chr11	115093203	115093496	2.37	7.27E-04	CADM1	281891	FAM55B	544150
chr17	56736299	56736696	2.29	7.45E-04	SEPT4	-129835	TEX14	32918
chr1	206939833	206940078	2.62	7.48E-04	IL10	5883	MAPKAPK2	81667
chrX	48538894	48539199	2.53	7.56E-04	WAS	-3139		
chr8	141467747	141468023	1.69	7.60E-04	TRAPPC9	793		

chr6	15662538	15662929	2.24	7.61E-04	DTNBP1	537		
chr11	130036388	130036661	2.14	7.66E-04	ST14	6843	ZBTB44	148082
chr13	99828857	99829133	1.84	7.66E-04	DOCK9	-90335	UBAC2	-23684
chr21	44710291	44710635	2.25	7.84E-04	CRYAA	121322	SIK1	136539
chr5	95672142	95672418	1.98	7.98E-04	ELL2	-374505	PCSK1	96672
chr12	9803319	9803629	2.02	8.07E-04	KLRB1	-42977	CLEC2D	-18835
chr1	161359620	161359896	2.22	8.08E-04	FCGR2A	-115447	SDHC	75592
chr1	22192486	22192915	2.01	8.09E-04	LDLRAD2	53943	HSPG2	71049
chr3	113724335	113724604	2.18	8.10E-04	QTRTD1	-51141	ZDHHC23	57722
chr2	20876620	20876929	1.81	8.22E-04	GDF7	10351	APOB	390170
chr19	42101678	42101967	2.15	8.36E-04	CEACAM21	19292	CEACAM4	31619
chr8	131000355	131000614	2.29	8.61E-04	GSDMC	-201351	ASAP1	413731
chr19	6604860	6605191	2.21	8.74E-04	CD70	-13863	TNFSF14	65573
chr6	414639	414875	2.49	8.74E-04	IRF4	23005	EXOC2	278352
chr18	77385186	77385476	2.44	8.80E-04	CTDP1	-54470	NFATC1	225005
chr7	102065270	102065577	1.82	9.01E-04	ORAI2	-8572	PRKRIP1	28620
chr18	33709668	33709966	1.86	9.13E-04	SLC39A6	-460	ELP2	-70
chr3	169762747	169763023	2.03	9.13E-04	GPR160	7150	PHC3	136652
chr13	31613412	31613694	2.72	9.46E-04	HSPH1	122564	ALOX5AP	303884
chr20	62611365	62611614	2.20	9.52E-04	PRPF6	-941		
chr1	150336011	150336409	2.05	9.58E-04	RPRD2	-780		
chr1	207098645	207098951	2.07	9.66E-04	FAIM3	-3420		
chr1	161582390	161582656	1.93	9.72E-04	HSPA7	6674	FCGR3B	18635
chr16	4012776	4013037	2.58	9.73E-04	CREBBP	-82786	ADCY9	153279
chr17	61919985	61920334	2.25	9.73E-04	SMARCD2	191		
chr10	26661801	26662210	1.98	9.79E-04	APBB1IP	-65260	GAD2	156770
chr8	67005479	67005732	2.70	9.83E-04	TRIM55	-33672	DNAJC5B	71815
chr17	60802246	60802582	2.05	9.85E-04	MARCH10	83291	MRC2	97652
chr12	9917423	9917667	1.90	9.85E-04	CD69	-4048		
chr12	92865396	92865672	2.23	9.91E-04	BTG1	-325861	PLEKHG7	-264731
chr8	26429599	26429921	1.99	9.93E-04	PNMA2	-58277	DPYSL2	-5661
chr19	40926648	40927060	1.98	9.98E-04	PRX	-7583	SERTAD1	5078
chr18	33709264	33709589	1.80	1.01E-03	ELP2	-460	SLC39A6	-70
chr3	71060779	71061053	2.97	1.01E-03	FOXP1	572224		
chr22	27011198	27011478	2.01	1.03E-03	TPST2	-49968	CRYBB1	2653
chr8	56885616	56885865	2.09	1.04E-03	LYN	93355	RPS20	101399
chr10	91648545	91648931	2.58	1.05E-03	KIF20B	187371	HTR7	968933
chr1	211700390	211700685	2.84	1.06E-03	RD3	-34279	SLC30A1	51561

chr11	60134505	60134833	2.39	1.10E-03	MS4A7	-11289	MS4A6E	32314
chr5	124040609	124040949	2.04	1.10E-03	ZNF608	40086		
chrX	53100837	53101247	1.88	1.10E-03	TSPYL2	-10507	GPR173	22536
chr19	55166422	55166669	2.13	1.11E-03	LILRB4	-7578	LILRB1	24578
chr2	86221135	86221449	2.17	1.13E-03	ST3GAL5	-105135	POLR1A	111986
chr6	106545581	106545982	2.21	1.16E-03	PRDM1	11587	ATG5	227913
chr2	25584973	25585286	1.93	1.16E-03	DNMT3A	-20356	DTNB	311373
chr3	195633244	195633520	1.97	1.17E-03	TNK2	-10950	SDHALP1	83768
chr20	23331181	23331458	1.97	1.17E-03	NXT1	-53		
chr5	77656082	77656411	1.79	1.18E-03	SCAMP1	-92		
chr15	31751257	31751601	1.87	1.18E-03	KLF13	132346	OTUD7A	196113
chr17	259826	260265	1.88	1.18E-03	RPH3AL	-57470	VPS53	358050
chr13	95201807	95202118	2.05	1.19E-03	DCT	-70027	TGDS	46548
chr19	42618622	42618919	1.77	1.19E-03	POU2F2	17859	ZNF574	38481
chr18	56435686	56435997	2.08	1.20E-03	ZNF532	-94219	MALT1	97224
chr19	50401011	50401289	1.92	1.22E-03	IL4I1	-1003		
chr7	69204231	69204483	2.21	1.23E-03				
chr1	206912640	206913088	2.08	1.23E-03	IL10	32975	MAPKAPK2	54575
chr19	46209701	46210036	1.98	1.23E-03	QPCTL	14128	FBXO46	24282
chr9	12958765	12959071	2.55	1.25E-03	TYRP1	265532	MPDZ	291453
chr12	4647515	4647803	1.70	1.25E-03	RAD51AP1	-291		
chr17	8130237	8130481	2.66	1.28E-03	AURKB	-16476	C17orf68	21054
chr3	58182238	58182501	2.07	1.28E-03	DNASE1L3	18028	FLNB	188243
chr9	140655489	140655777	2.54	1.28E-03	CACNA1B	-116608	EHMT1	142189
chr4	114604324	114604707	2.84	1.29E-03	CAMK2D	78567	ANK2	633731
chr17	80828905	80829447	1.77	1.30E-03	ZNF750	-31245	B3GNTL1	180510
chr3	183245144	183245396	2.18	1.32E-03	MCF2L2	-99415	KLHL6	28229
chr1	36814922	36815244	1.80	1.36E-03	FAM176B	-25328	STK40	36402
chr4	124317723	124317999	2.02	1.39E-03	SPRY1	-95		
chr13	78596391	78596733	2.09	1.39E-03	EDNRB	-46898	POU4F1	581133
chr19	50529166	50529476	2.04	1.40E-03	VRK3	-516	ZNF473	109
chr3	183272113	183272439	1.97	1.41E-03	MCF2L2	-126421	KLHL6	1223
chr22	22307125	22307375	2.26	1.42E-03	PPM1F	-33		
chr11	58984357	58984655	2.02	1.42E-03	MPEG1	-4012		
chr11	65189537	65189881	1.86	1.45E-03	SCYL1	-102839	FRMD8	35668
chr15	41708998	41709344	1.88	1.46E-03	RTF1	-131		
chr3	177038667	177038974	2.02	1.46E-03	TBL1XR1	-123773		
chr1	9129690	9130086	2.10	1.46E-03	SLC2A5	-1		

chr19	14317995	14318469	1.32	1.47E-03	LPHN1	-1235		
chrX	152127365	152127653	1.85	1.47E-03	PNMA3	-97257	ZNF185	44512
chr3	138296877	138297378	2.05	1.47E-03	CEP70	16001	ESYT3	143713
chr15	83735874	83736159	1.71	1.49E-03	BTBD1	89		
chr2	136964223	136964620	2.23	1.53E-03	THSD7B	-784040	CXCR4	-88697
chr14	22750386	22750736	2.11	1.55E-03	TCRDV2	-177528	TRA	274797
chr4	102711663	102711928	1.80	1.56E-03	BANK1	32		
chr1	205744227	205744760	2.19	1.59E-03	RAB7L1	116		
chr9	130732692	130732990	1.93	1.60E-03	DPM2	-32078	NAIF1	96758
chr17	43430466	43430731	2.47	1.61E-03	MAP3K14	-36185	ARHGAP27	72413
chr3	185000593	185000837	2.11	1.61E-03	MAP3K13	-80255	EHHADH	-28878
chr1	157010672	157010932	3.33	1.62E-03	ARHGEF11	4360	PEAR1	147279
chr19	49588584	49588884	1.74	1.62E-03	SNRNP70	269	SNRNP70	269
chr12	53765023	53765299	1.86	1.64E-03	SP7	-35624	SP1	-8818
chr1	79658909	79659388	2.21	1.64E-03	ELTD1	-186654		
chr4	87857329	87857597	2.43	1.69E-03	SLC10A6	-87047	AFF1	-70690
chr1	175158069	175158573	2.10	1.75E-03	TNN	121327	TNR	554431
chr15	90324379	90324654	2.04	1.75E-03	MESP2	4928	ANPEP	33555
chr15	70796970	70797299	1.81	1.75E-03	TLE3	-406879	UACA	258715
chr20	48315885	48316190	2.23	1.79E-03	PTGIS	-131331	B4GALT5	14383
chr16	87734591	87734924	1.73	1.80E-03	KLHDC4	64784	JPH3	98259
chr19	49256099	49256375	2.27	1.85E-03	FGF21	-2911	FUT1	2410
chr4	56814790	56815091	1.86	1.85E-03	CEP135	-96		
chr3	179156581	179156852	2.27	1.85E-03	GNB4	12654	MFN1	91237
chr1	110950195	110950521	1.94	1.86E-03	HBXIP	188		
chr17	62502224	62502550	1.98	1.87E-03	CCDC45	-771	DDX5	97
chr22	18278557	18279001	2.45	1.88E-03	BID	-22000	MICAL3	228546
chr6	291589	292060	1.98	1.89E-03	DUSP22	-276		
chr7	73515790	73516078	1.92	1.91E-03	EIF4H	-72772	LIMK1	17778
chr11	117857864	117858182	2.06	1.91E-03	IL10RA	917		
chr3	15387279	15387575	1.69	1.94E-03	SH3BP5	-13323	METTL6	81615
chr12	96589362	96589638	2.21	1.94E-03	ELK3	1293	PCTK2	204723
chr22	20104644	20104884	1.67	1.95E-03	RANBP1	-260	TRMT2A	4
chr10	27026627	27026920	2.03	1.96E-03	PDSS1	40179	ABI1	123185
chr12	104322801	104323077	2.03	1.97E-03	HSP90B1	-1250	GNN	1050
chr14	102342913	102343259	1.96	2.00E-03	DYNC1H1	-87779	PPP2R5C	114951
chr11	117881734	117882048	2.19	2.00E-03	TMPRSS4	-65836	IL10RA	24785
chr9	140082936	140083246	1.84	2.02E-03	ANAPC2	-34	SSNA1	37

chr12	90320940	90321242	2.06	2.05E-03	ATP2B1	-271247		
chr4	143326555	143326799	2.79	2.05E-03	INPP4B	440927	IL15	768923
chr1	28879303	28879613	1.70	2.10E-03	TRNAU1AP	-71		
chr2	202022846	202023090	2.01	2.11E-03	CASP10	-24653	CFLAR	42152
chr6	13274059	13274416	2.04	2.11E-03	TBC1D7	54532	PHACTR1	556405
chr4	185655264	185655574	2.51	2.11E-03	MLF1IP	-133		
chr19	42612707	42612990	1.80	2.18E-03	POU2F2	23781	ZNF574	32559
chr22	21355953	21356245	1.89	2.20E-03	THAP7	305		
chr15	89183378	89183624	1.76	2.21E-03	ACAN	-163173	ISG20	1462
chr12	90343717	90343993	2.25	2.22E-03	ATP2B1	-294011		
chr18	72166955	72167258	2.06	2.22E-03	CNDP1	-34585	CNDP2	3510
chr11	111259717	111259993	2.08	2.24E-03	POU2AF1	-9698	BTG4	123209
chr19	7770557	7770861	2.25	2.24E-03	FCER2	-3677		
chr9	37378256	37378528	2.14	2.24E-03	GRHPR	-44315	ZCCHC7	257923
chr3	171824820	171825084	1.91	2.27E-03	FNDC3B	67534	GHSR	341251
chr17	40215742	40216083	1.74	2.31E-03	ZNF385C	-35206	DHX58	48838
chr6	159128951	159129228	2.25	2.32E-03	SYTL3	58044	EZR	110250
chr3	121733612	121733917	2.23	2.35E-03	ILDR1	7265	SLC15A2	120594
chr11	118972755	118973040	1.99	2.35E-03	DPAGT1	-113		
chrX	52963879	52964219	1.98	2.37E-03	FAM156B	21904	FAM156B	35964
chr3	195264826	195265191	1.92	2.39E-03	ACAP2	-101192	PPP1R2	5215
chr5	88028037	88028357	1.96	2.40E-03	TMEM161B	-463532	MEF2C	151105
chr19	14693675	14693966	2.05	2.42E-03	CLEC17A	-75		
chr3	194854319	194854655	2.15	2.42E-03	LSG1	-461281	C3orf21	137408
chr19	58962717	58963038	1.69	2.42E-03	ZNF324B	-93		
chr7	47977820	47978119	1.82	2.45E-03	PKD1L1	10067	C7orf69	143081
chr1	36770171	36770424	2.08	2.46E-03	FAM176B	19457	THRAP3	80281
chr2	233946933	233947269	2.06	2.47E-03	ATG16L1	-213116	INPP5D	22065
chr10	52384261	52384572	1.69	2.47E-03	SGMS1	-680		
chr11	86013041	86013370	2.02	2.49E-03	C11orf73	-47		
chr13	52188700	52188976	2.10	2.51E-03	WDFY2	30354	DHRS12	189455
chr18	76828983	76829292	1.80	2.55E-03	ATP9B	-259		
chr19	47730794	47731110	1.83	2.58E-03	BBC3	5071	SAE1	96872
chr18	55288920	55289248	1.98	2.58E-03	NARS	93		
chr1	212661978	212662302	2.22	2.60E-03	ATF3	-76557	NENF	55911
chr3	150804448	150804807	1.84	2.62E-03	MED12L	43		
chr7	150132433	150132720	2.00	2.63E-03	GIMAP8	-15385	ZNF775	56171
chr2	158325357	158325700	2.12	2.63E-03	CYTIP	-24925	ACVR1C	159870

chr18	12407779	12408110	1.49	2.65E-03	SLMO1	50		
chr12	104321996	104322272	1.95	2.69E-03	HSP90B1	-2055	GNN	1855
chr3	184231552	184231828	2.29	2.70E-03	EPHB3	-47897	CHRD	133829
chr4	164585044	164585320	2.09	2.71E-03	MARCH1	-50406		
chrX	48776232	48776644	1.81	2.74E-03	PIM2	-25		
chr2	9563448	9563807	1.68	2.74E-03	CPSF3	-240	ITGB1BP1	15
chr1	234658332	234658699	1.91	2.77E-03	TARBP1	-43667	IRF2BP2	86755
chr9	139662591	139662924	1.63	2.77E-03	LCN15	-3793		
chr8	130951952	130952249	1.65	2.78E-03	GSDMC	-152967	ASAP1	462115
chr1	235098956	235099266	1.97	2.79E-03	IRF2BP2	-353840	TOMM20	193145
chr3	186718480	186718918	2.15	2.84E-03	ST6GAL1	-20966	ADIPOQ	158236
chr20	16554000	16554295	1.65	2.85E-03	KIF16B	-70		
chr3	42632021	42632300	1.69	2.90E-03	NKTR	-9986	SEC22C	-8733
chr10	80827039	80827338	1.76	2.91E-03	ZMIZ1	-1603		
chr1	192544625	192544958	1.85	2.93E-03	RGS1	-65		
chr4	74124640	74125046	1.92	2.93E-03	ANKRD17	-341		
chr3	187637464	187637777	1.94	2.94E-03	LPP	-293100	BCL6	-174146
chr8	103836554	103836871	2.11	2.95E-03	KLF10	-168730	AZIN1	39684
chr5	34499164	34499431	2.57	2.99E-03	C1QTNF3	-455981	RAI14	-157298
chr19	58987297	58987775	1.86	3.00E-03	ZNF446	-259		
chr7	148198841	148199165	1.79	3.01E-03	CUL1	-196930	NM_001126313	67364
chr22	18077412	18077703	1.98	3.03E-03	ATP6V1E1	34030	SLC25A18	34375
chr7	134832616	134832918	2.02	3.05E-03	TMEM140	1		
chr19	56189497	56189881	1.91	3.06E-03	EPN1	1698	NLRP9	60079
chr3	169658263	169658608	1.98	3.07E-03	LRRC31	-70776	SEC62	-26144
chr4	74455907	74456347	2.16	3.08E-03	RASSF6	30213	AFM	108665
chr21	48055231	48055601	1.79	3.09E-03	PRMT2	-111		
chr1	150132556	150132942	2.07	3.14E-03	PLEKHO1	10579	ANP32E	75755
chr9	132175901	132176201	1.60	3.14E-03	METTL11A	-212384	PPP2R4	302807
chr11	118691354	118691845	2.57	3.15E-03	CXCR5	-62941	DDX6	-29628
chrX	347842	348156	1.76	3.16E-03	PPP2R3B	-372		
chr3	47823362	47823619	1.78	3.17E-03	SMARCC1	-86		
chr1	145396850	145397126	1.96	3.17E-03	HFE2	-16203	NBPF10	103617
chr3	190963874	190964123	1.90	3.18E-03	CCDC50	-82875	OSTN	33677
chr2	220363479	220363786	1.71	3.19E-03	GMPPA	20		
chr1	145399174	145399494	2.01	3.20E-03	HFE2	-13857	NBPF10	105963
chr12	50505880	50506272	1.74	3.21E-03	C12orf62	176		
chr16	77224581	77225246	2.04	3.26E-03	LOC100130958	-8435	CNTNAP4	881179

chr2	112462829	112463144	1.68	3.31E-03	ANAPC1	178754	BCL2L11	584496
chr12	46384440	46384765	1.89	3.33E-03	SFRS2IP	-202		
chr19	57862485	57862738	1.86	3.35E-03	ZNF304	-33		
chr10	67261028	67261289	2.33	3.35E-03				
chr9	139434133	139434569	1.97	3.39E-03	NOTCH1	5887	C9orf163	56404
chr8	53626928	53627233	1.71	3.42E-03	RB1CC1	-55		
chr5	58423523	58423799	1.67	3.49E-03	RAB3C	544722	PDE4D	765960
chr9	95640211	95640576	1.90	3.49E-03	ZNF484	-104		
chr12	12878273	12878676	1.53	3.51E-03	APOLD1	-376		
chr6	18328843	18329119	1.94	3.51E-03	DEK	-64182	RNF144B	-58613
chr8	96036933	96037209	1.86	3.58E-03	C8orf38	-150		
chr4	87882822	87883098	1.91	3.58E-03	SLC10A6	-112544	AFF1	-45193
chr11	117356943	117357243	1.93	3.60E-03	CEP164	158522	DSCAML1	310883
chr1	197170520	197170911	1.79	3.69E-03	ZBTB41	-1044		
chr15	23033425	23033701	2.06	3.72E-03	NIPA2	845		
chr17	76702407	76702657	1.77	3.74E-03	DNAH17	-135128	CYTH1	75844
chr11	63993604	63993884	1.73	3.75E-03	DNAJC4	-4009	TRPT1	-18
chr7	140624715	140624992	1.81	3.80E-03	BRAF	-290		
chr19	19266343	19266705	2.33	3.86E-03	TMEM161A	-17257	MEF2B	14574
chr19	5791094	5791357	1.79	3.91E-03	DUS3L	23		
chr2	218087301	218087577	1.92	3.93E-03	TNP1	-362657	TNS1	721357
chr2	232572762	232573105	1.58	3.94E-03	PTMA	-301		
chr19	4581733	4582009	1.94	3.95E-03	SEMA6B	-23370	C19orf10	88544
chr4	146981054	146981370	1.87	3.99E-03	ZNF827	-121605	LSM6	-115623
chr1	2232533	2232875	1.97	4.00E-03	RER1	-90510	SKI	72570
chr10	52383633	52383931	1.58	4.00E-03	SGMS1	-45		
chr9	88555634	88555880	1.73	4.04E-03	MAK10	-300		
chr1	220263029	220263394	2.03	4.10E-03	IARS2	-4243	BPNT1	-21
chr1	144533961	144534325	3.26	4.10E-03	PPIAL4A	-169897	PDE4DIP	460879
chr19	947094	947401	1.83	4.11E-03	GRIN3B	-53189	ARID3A	21211
chr2	98644103	98644429	1.57	4.12E-03	CNGA3	-318352	TMEM131	-31912
chr5	77590368	77590837	1.70	4.13E-03	AP3B1	-75		
chr8	133288601	133288911	1.80	4.15E-03	HHLA1	-171244	KCNQ3	204248
chr5	64357289	64357649	2.07	4.15E-03	SDCCAG10	292714	ADAMTS6	420235
chrX	70585693	70585971	1.82	4.20E-03	TAF1	-282		
chr1	235123380	235123628	2.39	4.21E-03	IRF2BP2	-378233	TOMM20	168752
chr12	58292965	58293337	1.71	4.28E-03	CTDSP2	-52404	XRCC6BP1	-42294
chr17	77336754	77337298	1.99	4.32E-03	HRNBP3	141654	ENGASE	266007

chr2	10554148	10554472	1.74	4.36E-03	ODC1	34143	HPCAL1	111270		
chr16	89831320	89831652	1.68	4.36E-03	ZNF276	43534	FANCA	51579		
chr7	50332012	50332256	2.03	4.36E-03	ZPBP	-199274	IKZF1	-12244		
chr10	101754168	101754412	1.81	4.38E-03	DNMBP	15386	ABCC2	211827		
chr2	74756660	74757085	1.84	4.39E-03	DQX1	-3465	AUP1	101	HTRA2	341
chr3	15419692	15420042	1.80	4.39E-03	SH3BP5	-45763	METTL6	49175		
chr1	31226586	31226889	1.79	4.48E-03	MATN1	-30306	LAPTM5	3945		
chr2	70417700	70417987	1.74	4.50E-03	TIA1	57935	PCBP1	103259		
chr19	39050229	39050584	1.68	4.50E-03	MAP4K1	58236	RYR1	126067		
chr17	41445179	41445456	2.05	4.55E-03	ARL4D	-31035	TMEM106A	81424		
chr19	59084313	59084666	1.48	4.56E-03	MZF1	452				
chr4	144325777	144326021	2.12	4.67E-03	SMARCA5	-108717	GAB1	67916		
chr18	43830396	43830737	1.78	4.69E-03	RNF165	-83620	HAUS1	146269		
chr1	40652194	40652470	1.82	4.72E-03	TMCO2	-61241	RLF	25291		
chr11	65641059	65641410	1.59	4.75E-03	EFEMP2	-895				
chr7	105332520	105332817	2.01	4.78E-03	FLJ23834	-270988	RINT1	160137		
chr3	178789103	178789380	2.21	4.78E-03	ZMAT3	342				
chr20	37471192	37471520	2.13	4.82E-03	FAM83D	-83599	PPP1R16B	37008		
chr6	43142252	43142599	1.80	4.85E-03	CUL9	-7496	SRF	3506		
chr1	221951137	221951413	1.93	4.85E-03	DUSP10	-35814	HHIPL2	770169		
chr2	69875329	69875626	1.99	4.87E-03	AAK1	-4501				
chr2	185309023	185309344	1.97	4.87E-03	ZNF804A	-153909				
chr12	26266794	26267109	1.94	4.91E-03	BHLHE41	11051	RASSF8	154983		
chr1	206290163	206290588	2.38	4.93E-03	C1orf186	-1729				
chr19	35949907	35950234	1.89	4.96E-03	FFAR2	9454	KRTDAP	31285		
chr11	118741356	118741830	1.92	4.96E-03	DDX6	-79621	CXCR5	-12948		
chr15	80310021	80310274	2.21	4.99E-03	BCL2A1	-46505	ZFAND6	-41873		
chr1	249168218	249168608	1.92	5.01E-03	ZNF692	-15142				
chr12	12764615	12764894	1.68	5.03E-03	CREBL2	-76				
chr2	232526884	232527235	1.82	5.03E-03	NMUR1	-131878	PTMA	-46175		
chr15	22541907	22542314	1.88	5.06E-03	GOLGA6L1	-194135	OR4N2	128649		
chr4	109541524	109541808	1.86	5.12E-03	RPL34	-56				
chr4	184427339	184427669	1.65	5.12E-03	ING2	1284	ENPP6	711610		
chr9	128395007	128395322	1.96	5.13E-03	MAPKAP1	74348	GAPVD1	371054		
chr17	76635122	76635568	1.79	5.16E-03	DNAH17	-67941	CYTH1	143031		
chr3	15689160	15689470	2.23	5.18E-03	BTD	46060	ANKRD28	211738		
chrX	24168434	24168692	2.03	5.18E-03	ZFX	-1245				
chr5	77655651	77655939	1.68	5.28E-03	SCAMP1	-544				

chr2	39719234	39719565	2.11	5.30E-03	TMEM178	-173693	MAP4K3	-55181
chr12	56584009	56584281	2.10	5.30E-03	SMARCC2	-794		
chr17	1359501	1359763	1.56	5.36E-03	CRK	-88		
chr1	145589284	145589630	1.90	5.37E-03	NUDT17	-22		
chr8	8610515	8610881	2.12	5.45E-03	CLDN23	51032	MFHAS1	140433
chr16	85532132	85532564	1.61	5.49E-03	KIAA0182	-114576	KIAA0513	470938
chr17	8099437	8099701	2.17	5.50E-03	C17orf59	-6005	AURKB	14314
chr5	60615601	60615917	1.76	5.53E-03	C5orf43	-157457	ZSWIM6	-12341
chr19	49956249	49956561	1.57	5.53E-03	PIH1D1	-1290	ALDH16A1	-68
chr1	15898248	15898583	2.04	5.67E-03	AGMAT	13189	DNAJC16	45064
chr4	141075292	141075572	1.54	5.69E-03	MAML3	-199		
chr22	17738876	17739239	1.97	5.70E-03	CECR2	-217572	CECR1	-48279
chr15	63766286	63766577	1.66	5.71E-03	CA12	-92357	USP3	-30378
chr16	56950940	56951242	1.81	5.76E-03	HERPUD1	-14657	SLC12A3	51972
chr7	151168800	151169100	1.87	5.78E-03	RHEB	48060	NUB1	130092
chr4	23335610	23336081	1.98	5.83E-03	PPARGC1A	555854	GBA3	641298
chr7	22485063	22485401	1.63	5.84E-03	RAPGEF5	-88699	MGC87042	54566
chr7	47976914	47977278	1.88	5.87E-03	PKD1L1	10941	C7orf69	142207
chr12	25150986	25151389	1.94	5.89E-03	LRMP	-54053	BCAT1	-48880
chr1	235116767	235117051	2.18	5.89E-03	IRF2BP2	-371638	TOMM20	175347
chr11	128422917	128423248	1.95	5.89E-03	ETS1	34370		
chr2	65280408	65280688	1.73	5.98E-03	CEP68	-2947		
chr2	197024786	197025138	1.98	6.02E-03	DNAH7	-91426	STK17B	11374
chr12	59507616	59507892	2.16	6.04E-03	SLC16A7	-575372	LRIG3	-193492
chr18	9102341	9102663	1.84	6.04E-03	NDUFV2	-173		
chr2	97202813	97203091	1.87	6.07E-03	ARID5A	488		
chr11	62319233	62319695	2.13	6.08E-03	AHNAK	-5132	EEF1G	21996
chr11	82773012	82773278	2.16	6.10E-03	RAB30	9739	C11orf82	160408
chr2	39355179	39355448	1.69	6.10E-03	SOS1	-7710	CDKL4	101359
chr16	27388720	27388967	1.98	6.11E-03	IL21R	-49735	IL4R	63593
chr7	29317166	29317444	1.75	6.11E-03	PRR15	-286122	CHN2	83184
chr17	72510947	72511191	1.93	6.19E-03	CD300LB	16544	CD300A	48547
chr10	96996105	96996455	2.02	6.26E-03	C10orf129	42323	PDLIM1	54501
chr5	118679470	118679746	1.88	6.31E-03	TNFAIP8	-11988		
chr4	87995037	87995288	1.77	6.31E-03	AFF1	67010	KLHL8	146511
chr3	58477531	58477819	1.78	6.34E-03	KCTD6	-148		
chr2	21022750	21023132	1.78	6.34E-03	GDF7	156517	APOB	244004
chr19	58919673	58920106	1.42	6.38E-03	ZNF584	-173		

chr13	99128765	99129017	2.05	6.40E-03	RNF113B	-299370	STK24	100505
chr9	112178471	112178741	1.93	6.48E-03	EPB41L4B	-95585	PTPN3	81987
chr7	128786235	128786679	1.94	6.73E-03	SMO	-42256	TSPAN33	1745
chr6	53409734	53410050	1.65	6.76E-03	GCLC	-61		
chr4	119199568	119200119	1.80	6.76E-03	PRSS12	74078	NDST3	244344
chr9	130878080	130878325	1.97	6.82E-03	PTGES2	12271	SLC25A25	47724
chr3	122417137	122417381	2.16	6.84E-03	PARP14	17587	HSPBAP1	95391
chr10	70319909	70320185	1.98	6.89E-03	TET1	-70		
chrX	47077075	47077502	1.62	6.98E-03	PCTK1	-826		
chr12	113772740	113773081	1.64	6.99E-03	SLC24A6	14		
chr12	1913725	1914001	1.59	7.00E-03	LRTM2	-15570	ADIPOR2	113616
chr19	7744182	7744585	1.75	7.00E-03	TRAPPC5	-1323		
chr8	142216658	142216906	1.80	7.06E-03	PTK2	-205450	SLC45A4	21891
chr17	45569950	45570220	2.05	7.08E-03	NPEPPS	-38359	C17orf57	168735
chr2	97523600	97523900	1.47	7.10E-03	ANKRD23	-13992	SEMA4C	11985
chr5	124270762	124271107	1.74	7.18E-03	ZNF608	-190070		
chr9	117160066	117160371	1.88	7.22E-03	AKNA	-3534		
chr3	152016798	152017237	1.68	7.32E-03	MBNL1	-176		
chr9	101943818	101944285	1.83	7.35E-03	ALG2	40194	TGFBR1	76640
chr1	52344461	52344737	1.71	7.36E-03	NRD1	10		
chr20	5059164	5059459	1.82	7.36E-03	SLC23A2	-68373	C20orf30	34421
chr8	135613841	135614465	1.67	7.53E-03	ZFAT	111128		
chr19	58978220	58978471	1.66	7.54E-03	ZNF324	-117		
chr21	36599556	36599922	1.89	7.57E-03	CBR1	-842546	RUNX1	-178144
chr5	68628501	68628745	1.82	7.61E-03	TAF9	36786	CDK7	98001
chr6	28180625	28180958	2.05	7.62E-03	ZNF193	-12281	ZNF192	71076
chr17	36908750	36909162	1.94	7.66E-03	PCGF2	-4395	PSMB3	-46
chr15	30917875	30918174	2.07	7.70E-03	ARHGAP11B	-854		
chr14	32030424	32030760	1.76	7.72E-03	NUBPL	1		
chr17	3461877	3462153	1.76	7.74E-03	TRPV3	-726		
chr17	77397208	77397484	1.87	7.86E-03	HRNBP3	81334	ENGASE	326327
chr18	39694333	39694609	1.72	7.88E-03	PIK3C3	159272		
chr10	102672386	102672648	1.78	7.92E-03	SEMA4G	-59769	PAX2	167049
chr2	25585634	25585878	1.69	7.96E-03	DNMT3A	-20982	DTNB	310747
chr1	161016398	161016678	2.18	7.98E-03	USF1	-781		
chr14	24836387	24836663	1.47	8.02E-03	NFATC4	380		
chr17	55681347	55681643	1.97	8.04E-03	MRPS23	245904	MSI2	347564
chrX	77359209	77359599	1.86	8.04E-03	PGK1	-262		

chr19	909174	909557	1.75	8.12E-03	MED16	-16148	C19orf22	3859
chr19	55629041	55629326	1.53	8.14E-03	PPP1R12C	-257		
chr16	85790002	85790293	1.81	8.18E-03	GINS2	-67560	COX4NB	43000
chr14	65818560	65818866	2.07	8.23E-03	MAX	-249486	FUT8	-60822
chr12	66626115	66626408	1.98	8.39E-03	HELB	-70094	IRAK3	43284
chr3	138089962	138090218	2.02	8.40E-03	ESYT3	-63325	MRAS	22582
chr11	75484607	75484948	1.91	8.40E-03	UVRAG	-41434	DGAT2	5000
chr21	43955154	43955576	1.84	8.41E-03	PDE9A	-118497	SLC37A1	35623
chr20	52543087	52543500	1.93	8.53E-03	ZNF217	-343587	BCAS1	144010
chr2	68989821	68990214	1.75	8.60E-03	ARHGAP25	28050	BMP10	108631
chr2	240189126	240189402	1.65	8.61E-03	HDAC4	133379	TWIST2	432538
chr7	73625266	73625598	1.49	8.63E-03	LAT2	1345	RFC2	43306
chr9	114424647	114424923	2.20	8.73E-03	UGCG	-234421	DNAJC25	31153
chr2	216880706	216881011	1.79	8.75E-03	MREG	-2513		
chr19	51399901	51400205	1.67	8.77E-03	KLK4	13941	KLK2	23364
chr3	122824479	122824774	1.70	8.82E-03	SEC22A	-96147	PDIA5	38662
chr1	26947298	26947608	1.55	8.90E-03	ARID1A	-75069	RPS6KA1	75110
chr2	3714873	3715137	1.76	8.94E-03	ALLC	9219		
chr3	177077539	177078016	2.20	8.94E-03	TBL1XR1	-162730		
chr3	186703865	186704146	1.94	8.94E-03	ST6GAL1	-35659	ADIPOQ	143543
chr1	154531168	154531574	1.96	9.00E-03	UBE2Q1	-251		
chr6	134700246	134700678	1.71	9.00E-03	SGK1	-61266	ALDH8A1	570782
chr1	31227545	31227972	2.29	9.11E-03	MATN1	-31327	LAPTM5	2924
chr2	100863964	100864335	1.79	9.17E-03	AFF3	-142105	LONRF2	75045
chr2	88315793	88316092	1.97	9.20E-03	RGPD1	-190628	SMYD1	-51439
chr7	55601110	55601408	1.85	9.27E-03	VOPP1	38941	LANCL2	168118
chrX	153607212	153607629	1.25	9.35E-03	FLNA	-4415	EMD	-176
chr12	108079356	108079645	1.68	9.41E-03	PWP1	-89		
chr11	128343482	128343888	1.66	9.45E-03	ETS1	113768		
chr19	38524247	38524807	1.88	9.53E-03	SIPA1L3	126659	DPF1	190363
chr2	98629081	98629423	1.90	9.60E-03	CNGA3	-333366	TMEM131	-16898
chr1	66867747	66868269	1.68	9.64E-03	SGIP1	-131817	PDE4B	609152
chr17	17566627	17567327	1.63	9.65E-03	PEMT	-71983	RAI1	-17810
chr19	17633941	17634502	1.97	9.78E-03	GLT25D1	-32289	PGLS	11790
chr16	22203587	22203865	1.84	9.80E-03	EEF2K	-13866	VWA3A	99863
chr1	211870716	211871109	1.51	9.83E-03	NEK2	-21946	LPGAT1	133201
chr6	132819763	132820055	2.14	9.96E-03	MOXD1	-97245	STX7	14428
chr5	65850082	65850358	1.75	9.97E-03	MAST4	-274384	SFRS12	410135

chr22	39541546	39541818	1.91	1.00E-02	CBX7	6856	APOBEC3H	48392
chr6	4776455	4776731	1.68	1.01E-02	CDYL	-87		
chr19	10676605	10676874	1.52	1.02E-02	CDKN2D	2891	ATG4D	22093
chr15	75339281	75339754	1.66	1.02E-02	PPCDC	23591	DNM1P33	255788
chr4	185235517	185235793	1.81	1.02E-02	ENPP6	-96541	IRF2	160071
chr12	111882730	111883037	1.84	1.02E-02	SH2B3	39132	ATXN2	154596
chr5	169758190	169758462	2.23	1.03E-02	LCP2	-33504	KCNIP1	-22555
chr1	150265913	150266470	1.84	1.03E-02	MRPS21	-77		
chr13	48809222	48809508	2.08	1.03E-02	RB1	-68518	ITM2B	2091
chr11	121440741	121441065	1.60	1.04E-02	SORL1	117942	BLID	546020
chr2	70702455	70702743	2.13	1.04E-02	FAM136A	-173379	TGFA	78506
chr5	10700127	10700502	1.62	1.05E-02	DAP	61072	ROPN1L	258306
chr5	139018000	139018278	1.61	1.07E-02	CXXC5	-10162	UBE2D2	77388
chr6	53412697	53412987	1.56	1.07E-02	GCLC	-3011		
chr17	42425572	42425933	1.72	1.07E-02	GRN	3262	FAM171A2	15482
chr19	59055357	59055834	1.49	1.07E-02	TRIM28	-240		
chr11	121460678	121461251	1.97	1.07E-02	SORL1	138004	BLID	525958
chr18	31382994	31383275	2.07	1.07E-02	ASXL3	224594	NOL4	420311
chr17	40400575	40401027	1.78	1.07E-02	GHDC	-54251	STAT5B	27623
chr15	33162700	33162976	1.86	1.07E-02	GREM1	152633	FMN1	197247
chr1	32421650	32421969	1.70	1.08E-02	KHDRBS1	-57681	PTP4A2	-17822
chr12	123868360	123868648	1.63	1.09E-02	SETD8	-200		
chr15	91329374	91329826	1.79	1.10E-02	FURIN	-82285	BLM	69021
chr12	104322453	104322697	1.78	1.11E-02	HSP90B1	-1614	GNN	1414
chr22	39925188	39925498	1.87	1.11E-02	RPS19BP1	3517	ATF4	8774
chr15	44068330	44068735	1.68	1.12E-02	ELL3	969		
chr3	100427964	100428465	1.79	1.13E-02	TFG	55		
chr15	43212986	43213391	1.59	1.13E-02	TTBK2	-182		
chr19	45519610	45520057	1.65	1.14E-02	SFRS16	-22464	RELB	15122
chr2	8713733	8714009	1.60	1.14E-02	ID2	-108113		
chr2	231467386	231467687	1.92	1.14E-02	CAB39	-110020	SP100	186666
chr5	109257497	109257807	2.16	1.15E-02	MAN2A1	232496	TMEM232	804729
chr2	96890816	96891154	1.69	1.15E-02	STARD7	-16412	TMEM127	40747
chr11	117688973	117689250	2.13	1.16E-02	DSCAML1	-21136	FXYD2	6347
chr15	66111282	66111565	1.87	1.17E-02	RAB11A	-50372	DENND4A	-26793
chr10	105437954	105438233	1.63	1.18E-02	SH3PXD2A	177070	NEURL	184359
chr3	17236699	17237111	1.99	1.18E-02	PLCL2	262323	TBC1D5	547335
chr12	90250298	90250574	1.46	1.18E-02	ATP2B1	-200592		

chr3	122295034	122295365	2.01	1.18E-02	PARP15	-1249		
chr11	64633091	64633504	1.73	1.19E-02	CDC42BPG	-21257	EHD1	12893
chr19	11146509	11146985	1.91	1.19E-02	LDLR	-53310	SMARCA4	75149
chr5	33440691	33440971	1.78	1.20E-02	TARS	-67		
chr19	49955016	49955371	1.88	1.20E-02	ALDH16A1	-1279	PIH1D1	-79
chr1	159037709	159038319	1.98	1.20E-02	AIM2	8633	IFI16	58332
chr19	56825753	56826103	1.39	1.20E-02	ZSCAN5A	-86269	ZNF542	-53540
chr2	89137752	89138205	1.69	1.20E-02	O1/O11 and JK2	-752583	RPIA	146803
chr16	2961748	2962018	1.41	1.20E-02	FLYWCH1	-97		
chr14	24578045	24578389	1.66	1.21E-02	DCAF11	-5689	PCK2	14734
chr6	30456933	30457298	1.77	1.21E-02	HLA-E	-155		
chr13	79768481	79768776	1.82	1.22E-02	RNF219	-535315	RBM26	211294
chr17	56708946	56709245	1.91	1.22E-02	SEPT4	-102433	TEX14	60320
chr2	3522726	3523036	1.54	1.23E-02	ADI1	469		
chr18	77580121	77580424	1.80	1.23E-02	KCNG2	-43395	CTDP1	140472
chr1	80133585	80133861	2.07	1.24E-02	ELTD1	-661228		
chr8	33330584	33330933	1.46	1.24E-02	FUT10	-95		
chr19	58666445	58666767	1.74	1.24E-02	ZNF329	-4458		
chr3	197439219	197439548	1.78	1.24E-02	BDH1	-156526	KIAA0226	24389
chr19	36606307	36606637	1.53	1.27E-02	POLR2I	-266	TBCB	584
chr2	42332512	42332832	1.76	1.27E-02	EML4	-63818	SGK493	57511
chr3	186236758	186237174	1.63	1.27E-02	DGKG	-156943	CRYGS	25201
chr3	195620386	195620680	1.64	1.28E-02	MUC4	-81385	TNK2	1899
chr3	120314933	120315333	1.64	1.29E-02	NDUFB4	5		
chr15	45020905	45021419	2.42	1.29E-02	TRIM69	-7378	B2M	17477
chr17	53315651	53315904	1.86	1.30E-02	HLF	-26543	STXBP4	269652
chr2	6485693	6486026	1.68	1.31E-02	CMPK2	520076	SOX11	653061
chr3	188156876	188157244	1.67	1.32E-02	TPRG1	-732703	LPP	226339
chr5	1499042	1499374	1.67	1.32E-02	SLC6A3	-53670	LPCAT1	24868
chr16	3202823	3203124	1.88	1.32E-02	OR1F1	-51273	caspace	8754
chr9	102016113	102016543	1.66	1.34E-02	NR4A3	-572681	SEC61B	31758
chr19	54605905	54606206	1.67	1.35E-02	OSCAR	-1814	NDUFA3	-104
chr13	95253617	95254083	1.69	1.36E-02	GPR180	-254		
chr15	86233838	86234109	1.85	1.36E-02	KLHL25	104215	AKAP13	310103
chr17	37778214	37778524	1.66	1.37E-02	PPP1R1B	-4810		
chr1	154580537	154580947	1.56	1.38E-02	ADAR	-60		
chr14	102310025	102310475	1.51	1.38E-02	DYNC1H1	-120615	PPP2R5C	82115
chr7	55637485	55637761	1.76	1.40E-02	VOPP1	2577	LANCL2	204482

chr1	245084313	245084603	2.30	1.40E-02	HNRNPU	-56631	EFCAB2	-48713
chr12	14518796	14519076	1.63	1.40E-02	ATF7IP	325		
chr19	49999404	49999708	1.60	1.41E-02	RPS11	-78		
chr14	103572140	103572416	1.74	1.43E-02	CDC42BPB	-48536	TNFAIP2	-20386
chr2	173941046	173941353	2.18	1.43E-02	ZAK	635		
chr9	138853102	138853464	1.41	1.43E-02	UBAC1	-57		
chr13	52158332	52158595	1.47	1.44E-02	WDFY2	-20		
chr14	62129233	62129511	1.93	1.44E-02	HIF1A	-32747	PRKCH	340857
chr3	186250597	186250907	2.05	1.44E-02	DGKG	-170729	CRYGS	11415
chr16	89638488	89638732	2.13	1.45E-02	CPNE7	-3566		
chr17	3614240	3614739	2.36	1.45E-02	P2RX5	-14907	GSG2	-12707
chr3	181418270	181418534	1.99	1.46E-02	DNAJC19	-710872	SOX2	-11320
chr5	96271542	96271852	1.60	1.46E-02	LNPEP	-22459	ERAP2	60053
chr8	141474244	141474713	1.72	1.47E-02	CHRAC1	-46922	TRAPPC9	-5801
chr2	211035370	211035634	1.63	1.50E-02	ACADL	54713	RPE	168150
chr2	201983150	201983515	1.67	1.50E-02	CASP10	-64288	CFLAR	2517
chr19	45970816	45971064	1.43	1.50E-02	FOSB	-313		
chr11	93474542	93474921	1.75	1.51E-02	TAF1D	-70	C11orf54	-61
chr8	135703566	135703898	1.66	1.51E-02	ZFAT	21549		
chr11	67035896	67036195	1.77	1.52E-02	SSH3	-34873	ADRBK1	2141
chr2	192419826	192420102	1.75	1.52E-02	OBFC2A	-122897	MYO1B	309857
chr2	24148887	24149178	1.59	1.53E-02	ATAD2B	901		
chr16	2557069	2557329	1.86	1.53E-02	ATP6V0C	-6754	TBC1D24	32052
chr16	87843176	87843526	1.57	1.54E-02	KLHDC4	-43809	SLC7A5	59749
chr19	6502345	6502602	2.16	1.54E-02	TUBB4	-144		
chr3	188414919	188415237	2.13	1.54E-02	TPRG1	-474685	LPP	484357
chr1	42928752	42929028	1.93	1.55E-02	PPIH	-195158	PPCS	6717
chr20	48285064	48285322	2.05	1.57E-02	PTGIS	-100486	B4GALT5	45228
chr19	47616945	47617191	1.51	1.57E-02	ZC3H4	-59		
chr2	3643673	3643923	1.96	1.57E-02	ALLC	-61988	COLEC11	1161
chr19	1269529	1269821	1.63	1.57E-02	CIRBP	408		
chr12	31812094	31812389	1.61	1.58E-02	C12orf72	-367		
chr19	38469433	38469714	1.77	1.61E-02	SIPA1L3	71706	DPF1	245316
chr19	11375151	11375405	1.84	1.61E-02	DOCK6	-2121		
chr17	61851132	61851478	1.71	1.61E-02	DDX42	-262	CCDC47	-217
chr7	4721684	4721970	1.57	1.62E-02	FOXK1	-103		
chr18	51750879	51751490	1.63	1.65E-02	MBD2	-27		
chr18	45206303	45206562	1.92	1.65E-02	CORL2	-430879	SMAD2	251082

chr2	25524375	25524654	1.72	1.66E-02	POMC	-132956	DNMT3A	40259
chrX	1640560	1640832	1.83	1.67E-02	ASMTL	-68852	P2RY8	15341
chr11	62476956	62477323	1.61	1.68E-02	BSCL2	-94		
chr3	107241595	107241871	1.57	1.68E-02	BBX	-50		
chr11	119039283	119039572	1.80	1.68E-02	NLRX1	-12		
chr18	3178002	3178278	1.60	1.69E-02	LPIN2	-166195	MYOM1	41966
chr16	57578212	57578569	1.63	1.69E-02	GPR56	-75519	GPR114	1790
chr7	76831779	76832083	1.94	1.69E-02	FGL2	-2781		
chr1	221950093	221950393	1.47	1.70E-02	DUSP10	-34782	HHIPL2	771201
chr19	57900923	57901332	1.63	1.71E-02	ZNF548	-90		
chr13	41006172	41006469	1.67	1.72E-02	FOXO1	234413	COG6	776557
chr4	99850183	99850707	1.74	1.72E-02	EIF4E	-202		
chr22	29102904	29103161	2.31	1.73E-02	TTC28	-27180	CHEK2	34789
chr12	2113561	2113833	1.48	1.73E-02	DCP1B	-20		
chr19	56652148	56652566	1.50	1.74E-02	ZNF444	-199		
chr11	34018612	34018892	1.99	1.74E-02	LMO2	-104916	CAPRIN1	-54478
chr12	116997618	116997887	1.83	1.75E-02	MAP1LC3B2	567		
chr15	74676158	74676480	1.74	1.75E-02	CYP11A1	-16238	SEMA7A	49980
chr4	114486142	114486463	1.66	1.77E-02	CAMK2D	196780	ANK2	515518
chr4	1340485	1340960	1.47	1.78E-02	CRIPAK	-44617	MAEA	57051
chr9	135257500	135257902	1.82	1.78E-02	SETX	-27329	TTF1	24520
chr11	76383726	76384155	2.03	1.78E-02	LRRC32	-2897		
chr19	57874683	57874995	1.58	1.79E-02	ZNF547	-40		
chr20	50016951	50017199	1.73	1.80E-02	KCNG1	-377400	NFATC2	142183
chr1	145397743	145398052	1.73	1.80E-02	HFE2	-15293	NBPF10	104527
chr2	8617973	8618333	1.70	1.80E-02	ID2	-203831		
chr11	133765179	133765470	1.81	1.81E-02	SPATA19	-49933	IGSF9B	61555
chr19	49990617	49990994	1.62	1.81E-02	RPL13A	-59		
chr3	49275742	49276200	1.66	1.81E-02	KLHDC8B	66903	USP4	101565
chr4	81912085	81912361	1.72	1.82E-02	BMP3	-39896	FGF5	724481
chr6	28849079	28849380	2.16	1.84E-02	SCAND3	-294118	TRIM27	42538
chr2	54326952	54327280	1.64	1.84E-02	PSME4	-129139	ACYP2	-15294
chr7	105332176	105332430	1.64	1.84E-02	FLJ23834	-271354	RINT1	159771
chr18	72264980	72265220	1.78	1.86E-02	ZNF407	-77823	CNDP1	63408
chr12	90647603	90647952	1.50	1.87E-02	ATP2B1	-597934	EPYC	751025
chr21	26946216	26946470	1.74	1.87E-02	MRPL39	33458		
chr15	80748204	80748503	1.80	1.88E-02	FAM108C1	-239298	ARNT2	51662
chr7	138915719	138916057	1.48	1.89E-02	LUC7L2	-128746	TTC26	97398

chr9	88969046	88969518	1.58	1.90E-02	ZCCHC6	87		
chr9	124705839	124706132	1.65	1.91E-02	TTLL11	149899	DAB2IP	376587
chr19	58874148	58874389	1.24	1.92E-02	ZNF497	-149		
chr1	192485968	192486285	1.87	1.92E-02	RGS1	-58730	RGS21	200005
chr18	77748493	77748796	1.94	1.92E-02	TXNL4A	-113		
chr1	161500896	161501173	1.70	1.93E-02	HSPA6	6999	FCGR3A	18783
chr19	37019107	37019515	1.68	1.95E-02	ZNF260	-141		
chr17	30850294	30850676	1.71	1.95E-02	CDK5R1	36380	MYO1D	353417
chr22	19842367	19842616	1.57	1.96E-02	GNB1L	-30		
chr10	97457225	97457469	2.28	2.00E-02	TCTN3	-3447		
chr9	34098341	34098704	1.75	2.02E-02	DCAF12	28248	UBE2R2	281341
chr3	183892380	183892674	1.55	2.03E-02	AP2M1	-107		
chr1	207510558	207510865	1.92	2.03E-02	CR2	-116958	CD55	15895
chr19	41770121	41770361	1.43	2.03E-02	HNRNPUL1	121		
chr9	137828646	137828906	1.73	2.04E-02	OLFM1	-138313	FCN1	-18967
chr12	69202555	69202831	1.59	2.05E-02	MDM2	722		
chrX	129300076	129300352	1.41	2.05E-02	AIFM1	-406		
chr22	35806203	35806530	1.64	2.05E-02	RASD2	-130985	MCM5	10251
chr4	113656590	113656869	2.35	2.06E-02	ANK2	-314055	LARP7	98118
chr2	31392963	31393275	1.79	2.08E-02	GALNT14	-31548	CAPN14	47292
chr15	63767107	63767463	1.66	2.09E-02	CA12	-93210	USP3	-29525
chr19	55919532	55919816	1.58	2.10E-02	UBE2S	-349		
chr7	140098231	140098515	1.68	2.10E-02	SLC37A3	-62		
chr1	37754437	37754895	1.58	2.14E-02	GRIK3	-254822	ZC3H12A	-185453
chr3	143393026	143393503	1.78	2.15E-02	SLC9A9	174081	CHST2	554597
chr13	76270010	76270286	2.23	2.17E-02	LMO7	-64649	UCHL3	146221
chr17	77967381	77967689	2.01	2.18E-02	CBX4	-154322	TBC1D16	42112
chr3	186783139	186783621	1.96	2.20E-02	ST6GAL1	43715	RPL39L	73883
chr16	2014614	2015146	1.73	2.20E-02	RNF151	-1995	RPS2	-53
chr19	40790328	40790604	2.12	2.22E-02	AKT2	799		
chr17	75136447	75136784	1.59	2.22E-02	SEC14L1	-389		
chr9	139001495	139001808	1.48	2.23E-02	NACC2	-14521	LHX3	93352
chr12	123850010	123850490	1.73	2.23E-02	SETD8	-18454	SBNO1	-15262
chr2	177628634	177629120	1.56	2.24E-02	HNRNPA3	-448545	MTX2	494754
chr15	45684339	45684691	1.75	2.30E-02	GATM	-13535	SPATA5L1	-10004
chr3	57261593	57261903	1.70	2.30E-02	APPL1	-17		
chr7	11579966	11580244	1.47	2.30E-02	THSD7A	291719	PHF14	566589
chr12	56754086	56754396	2.12	2.31E-02	STAT2	-332		

chrX	30907547	30907859	1.46	2.31E-02	MAP3K7IP3	-192		
chr16	15684733	15685074	1.92	2.32E-02	KIAA0430	52105	C16orf45	156579
chr14	68973478	68973766	1.68	2.34E-02	ZFP36L1	286163	RAD51L1	687113
chr12	57916484	57916736	1.43	2.37E-02	DDIT3	-2310	MBD6	-49
chr16	2770946	2771228	1.89	2.37E-02	PRSS27	-535		
chr10	89622023	89622355	1.43	2.37E-02	PTEN	-1006		
chr3	38179941	38180312	1.67	2.39E-02	ACAA1	-1394	MYD88	158
chr5	118625963	118626373	1.58	2.40E-02	TNFAIP8	-65428		
chr14	104073491	104073767	1.73	2.40E-02	BAG5	-45015	KLC1	-21896
chr4	57844875	57845119	1.46	2.42E-02	C4orf14	-1171	POLR2B	-112
chr12	31835100	31835417	1.62	2.42E-02	C12orf72	22650	H3F3C	109916
chr19	48248590	48249049	1.58	2.42E-02	GLTSCR2	27		
chr9	31519020	31519348	1.72	2.45E-02	ACO1	-865417		
chr12	12509877	12510209	1.58	2.47E-02	MANSC1	-6874	DUSP16	205274
chr22	24186640	24186931	1.45	2.48E-02	SLC2A11	-13255	DERL3	-5587
chr12	46465990	46466266	1.68	2.48E-02	SFRS2IP	-81727	SLC38A1	197080
chr7	55602025	55602420	2.08	2.49E-02	VOPP1	37977	LANCL2	169082
chr2	46717781	46718038	1.86	2.51E-02	LOC388946	11206	ATP6V1E2	29186
chr12	43505304	43505697	1.70	2.51E-02	PRICKLE1	-628085	ADAMTS20	440223
chr19	1021054	1021314	1.68	2.52E-02	C19orf6	-43		
chr6	37016447	37016816	1.72	2.52E-02	PIM1	-121290	FGD2	43209
chr16	85981462	85981825	1.61	2.54E-02	FOXF1	-562489	IRF8	48870
chr9	95900677	95901103	1.48	2.54E-02	NINJ1	-4320		
chr7	97829705	97830048	1.71	2.54E-02	BHLHA15	-11691	LMTK2	93680
chr7	26008325	26008599	1.45	2.55E-02	NPVF	-740357	NFE2L3	-183385
chr5	173314625	173314939	1.64	2.57E-02	CPEB4	-549		
chr11	65341611	65342055	1.54	2.57E-02	SSSCA1	3890	KCNK7	21634
chr17	77778326	77778611	2.02	2.57E-02	CBX8	-7579	CBX4	34744
chr6	87864941	87865304	1.59	2.58E-02	ZNF292	-146		
chr12	123911571	123911914	1.56	2.58E-02	RILPL2	9521	SETD8	43039
chr9	36993852	36994236	1.64	2.60E-02	PAX5	40432	MELK	421139
chr12	121991439	121991742	1.60	2.60E-02	KDM2B	27329	RNF34	153689
chr6	347291	347589	1.90	2.60E-02	IRF4	-44312	DUSP22	55339
chr11	119194234	119194510	1.86	2.61E-02	RNF26	-10865	MCAM	-6532
chr2	65662835	65663126	1.49	2.62E-02	SPRED2	-3325		
chr3	98241744	98242055	1.81	2.62E-02	CLDND1	10		
chr4	170679024	170679334	1.77	2.66E-02	C4orf27	-86		
chr2	27712528	27712902	1.57	2.66E-02	IFT172	-144		

chr15	74258214	74258474	1.92	2.67E-02	STOML1	26291	LOXL1	39555
chr19	50143480	50143880	1.32	2.67E-02	SCAF1	-1702	RRAS	-280
chr3	184190884	184191150	1.57	2.67E-02	EPHB3	-88570	CHRD	93156
chr2	3652966	3653312	1.34	2.67E-02	ALLC	-52647	COLEC11	10502
chr9	136810392	136810997	1.72	2.68E-02	SARDH	-207216	VAV2	46751
chr3	183003651	183003895	1.72	2.69E-02	B3GNT5	32741	MCF2L2	142082
chr1	169863015	169863495	1.77	2.70E-02	SCYL3	-179		
chr20	49996954	49997264	1.78	2.70E-02	KCNG1	-357434	NFATC2	162149
chr19	39109559	39110008	1.63	2.72E-02	MAP4K1	-1141	EIF3K	62
chr17	80477152	80477557	1.44	2.72E-02	FOXK2	-239		
chr5	88567331	88567575	1.93	2.73E-02	MEF2C	-388151		
chr8	8860072	8860481	1.59	2.73E-02	ERI1	-37		
chr20	30946819	30947216	1.80	2.74E-02	ASXL1	865		
chr20	5591481	5591949	1.52	2.75E-02	RP5-1022P6.2	-43		
chr20	43996860	43997236	1.82	2.76E-02	SYS1	5347	TP53TG5	9909
chr15	93375657	93375960	1.76	2.76E-02	FAM174B	-176778	CHD2	-67742
chr8	28198601	28198918	1.84	2.77E-02	PNOC	24111	ZNF395	45217
chr15	90640809	90641056	1.81	2.77E-02	IDH2	4775	ZNF710	96181
chr19	1240306	1240578	1.55	2.78E-02	C19orf26	-2452	ATP5D	-1307
chr22	20861694	20861939	1.74	2.78E-02	MED15	-69		
chr11	64667776	64668020	2.23	2.79E-02	PPP2R5B	-24282	EHD1	-21707
chr7	135665538	135665869	1.79	2.80E-02	MTPN	-3500		
chr1	27018675	27019154	1.59	2.81E-02	ARID1A	-3607		
chr12	56617985	56618274	1.49	2.81E-02	RNF41	-2426	OBFC2B	5
chr22	45683886	45684130	1.52	2.81E-02	FAM118A	-21777	UPK3A	3119
chr4	122722290	122722600	1.46	2.82E-02	EXOSC9	-27		
chr8	125539513	125539793	1.47	2.82E-02	TATDN1	11676	RNF139	52645
chr2	185235797	185236046	1.87	2.84E-02	ZNF804A	-227171		
chr13	78271707	78271986	1.48	2.84E-02	SCEL	162038	EDNRB	277817
chr9	126973085	126973365	1.72	2.85E-02	NEK6	-47018	LHX2	199336
chr4	693233	693488	1.66	2.85E-02	MFSD7	-10388	PCGF3	-6212
chr19	1344752	1345060	1.55	2.86E-02	MUM1	-10070	EFNA2	58738
chr9	129233377	129233635	1.76	2.86E-02	LMX1B	-143242	FAM125B	144378
chr19	38495222	38495505	1.52	2.87E-02	SIPA1L3	97496	DPF1	219526
chr19	46149333	46149649	1.69	2.89E-02	EML2	-765		
chr2	8443585	8443977	1.66	2.90E-02	ID2	-378203		
chr18	44702571	44702882	1.62	2.91E-02	IER3IP1	18		
chr15	85956718	85957036	1.54	2.93E-02	AKAP13	33006	KLHL25	381312

chr12	122418084	122418455	1.77	2.94E-02	BCL7A	-41591	PSMD9	91624
chr7	50356947	50357223	1.72	2.94E-02	IKZF1	12707	FIGNL1	161003
chr1	226850600	226851053	2.00	2.95E-02	ITPKB	76049	C1orf95	114326
chr10	329790	330149	1.72	2.95E-02	ZMYND11	149546	DIP2C	405638
chr19	57751858	57752137	1.87	2.96E-02	ZNF805	-55		
chr20	60982602	60982940	1.75	2.96E-02	CABLES2	-432		
chr8	33342543	33342841	1.80	2.96E-02	MAK16	7		
chr11	122588123	122588477	1.66	2.97E-02	CRTAM	-120955	UBASH3B	61902
chr5	179233797	179234073	1.25	2.97E-02	MGAT4B	17		
chr22	19132163	19132427	1.54	2.98E-02	DGCR14	-105		
chr19	58898346	58898806	1.86	3.01E-02	RPS5	-60		
chrX	16737506	16737799	1.72	3.02E-02	SYAP1	-102		
chr5	114545006	114545389	1.83	3.06E-02	TRIM36	-28955	PGGT1B	53371
chr11	70116501	70116809	1.60	3.07E-02	PPFIA1	-168		
chr11	117198377	117198653	1.60	3.07E-02	CEP164	-56		
chr11	118938086	118938587	1.62	3.09E-02	VPS11	-156		
chr10	18948071	18948470	1.47	3.11E-02	ARL5B	-42		
chr19	16739889	16740429	1.57	3.11E-02	MED26	-1144		
chr9	130541566	130541831	1.51	3.11E-02	SH2D3C	-651		
chr3	150102718	150103003	1.90	3.12E-02	PFN2	-414120	TSC22D2	-23927
chr17	37910349	37910942	1.73	3.13E-02	GRB7	16459	IKZF3	109795
chr5	60954403	60954679	2.02	3.13E-02	KIF2A	-647448	FLJ37543	20905
chr12	26266250	26266537	1.80	3.15E-02	BHLHE41	11609	RASSF8	154425
chr7	127291850	127292174	1.51	3.16E-02	SND1	-190		
chr15	45747090	45747400	1.66	3.18E-02	C15orf48	24482	SLC30A4	67757
chr1	101713480	101713817	1.60	3.18E-02	S1PR1	11344	OLFM3	749141
chrX	41593198	41593520	1.75	3.18E-02	GPR82	9951	CASK	188928
chr7	150132816	150133177	1.57	3.19E-02	GIMAP8	-14965	ZNF775	56591
chr7	43689449	43689725	1.95	3.20E-02	STK17A	66895	C7orf44	79496
chr18	43547064	43547523	1.81	3.22E-02	PSTPIP2	104956	SIGLEC15	141749
chr15	65596987	65597487	1.88	3.23E-02	PARP16	-18219	IGDCC3	73141
chr8	126656743	126657124	1.87	3.23E-02	TRIB1	214371	FAM84B	913532
chr5	141704537	141704786	1.76	3.25E-02	SPRY4	-42		
chr19	3180384	3180660	1.90	3.26E-02	NCLN	-5353	S1PR4	1756
chr11	116969151	116969401	1.50	3.26E-02	SIK3	-283		
chr3	191846733	191847007	1.92	3.26E-02	FGF12	279968	PYDC2	667918
chr20	42356889	42357169	1.73	3.28E-02	GTSF1L	-1387		
chr6	149079653	149080024	1.90	3.28E-02	MAP3K7IP2	-559224	UST	11568

chr3	185973360	185973716	1.49	3.29E-02	ETV5	-146637	DGKG	106485
chr2	242275754	242276108	1.96	3.29E-02	FARP2	-19780	SEPT2	21208
chr12	40620078	40620361	1.86	3.29E-02	CNTN1	-466138	LRRK2	1407
chr2	174739636	174739995	1.69	3.31E-02	SP3	90247	CDCA7	520255
chr19	37157631	37157920	2.07	3.31E-02	ZNF461	-37		
chr9	139981167	139981445	1.56	3.32E-02	MAN1B1	-111		
chr17	73030502	73030746	1.71	3.33E-02	ATP5H	12450	ICT1	21844
chr11	111957345	111957643	1.66	3.33E-02	SDHD	-77	TIMM8B	-35
chr15	81586179	81586681	1.89	3.33E-02	STARD5	30094	IL16	97211
chr2	89145311	89145802	1.58	3.33E-02	O1/O11 and JK2	-745005	RPIA	154381
chr5	43020159	43020490	1.54	3.34E-02	C5orf39	20122	GHR	596299
chr15	79299325	79299749	1.48	3.37E-02	CTSH	-62117	RASGRF1	83678
chr15	43398150	43398478	1.71	3.38E-02	UBR1	-28		
chr2	133339586	133339925	2.66	3.38E-02	LYPD1	88725	GPR39	165609
chr4	77069450	77069760	1.64	3.38E-02	NUP54	50		
chr1	118148259	118148566	1.52	3.38E-02	WDR3	-323959	MAN1A2	238328
chr17	34417496	34417954	1.83	3.39E-02	CCL3	-219		
chr7	154997318	154997588	1.68	3.40E-02	INSIG1	-92033	HTR5A	134907
chr1	157670603	157670913	1.68	3.41E-02	FCRL3	-111		
chr10	105439172	105439470	1.60	3.43E-02	SH3PXD2A	175843	NEURL	185586
chr11	61809576	61809913	1.67	3.44E-02	INCENP	-81700	FTH1	-74613
chr17	36754846	36755186	1.39	3.44E-02	SNIP	7167	ARHGAP23	141372
chr11	118991679	118992278	1.72	3.45E-02	HINFP	-309		
chr9	130565141	130565453	1.85	3.46E-02	FPGS	143		
chr7	106511546	106511791	1.92	3.48E-02	PRKAR2B	-173509	PIK3CG	5745
chr2	74408858	74409146	1.66	3.48E-02	MOBKL1B	-3007		
chr12	107303961	107304358	1.59	3.51E-02	C12orf23	-45384	RIC8B	135761
chr1	27019498	27019838	1.67	3.52E-02	ARID1A	-2854		
chr5	61601492	61601914	1.64	3.54E-02	KIF2A	-286		
chr19	1042231	1042517	1.87	3.54E-02	HMHA1	-24800	ABCA7	2272
chr19	41282983	41283357	1.55	3.57E-02	RAB4B	-1001		
chr3	13373716	13374108	1.66	3.59E-02	IQSEC1	-259295	NUP210	87897
chr20	30286925	30287260	1.77	3.60E-02	BCL2L1	23563	COX4I2	61402
chr11	118766376	118766628	1.56	3.61E-02	CXCR5	11961	BCL9L	15111
chr2	16894387	16894648	1.71	3.61E-02	VSNL1	-827289	FAM49A	-47422
chr2	122407417	122407789	1.61	3.63E-02	CLASP1	-551		
chr3	111258605	111258881	1.91	3.64E-02	CD96	-2183		
chr19	48833364	48833747	1.44	3.64E-02	EMP3	4927	TMEM143	33630

chr17	41560993	41561411	1.75	3.64E-02	DHX8	-132		
chr19	50922823	50923113	1.77	3.64E-02	SPIB	773		
chr19	50181531	50181910	1.43	3.64E-02	C19orf76	-10221	PRMT1	1225
chr22	42840118	42840394	1.29	3.69E-02	SERHL	-56329	NFAM1	-11855
chr6	6856817	6857068	1.63	3.69E-02	RREB1	-251245	LY86	268009
chr14	55569036	55569327	1.71	3.70E-02	LGALS3	-26690	SOCS4	75338
chr12	31899759	31900051	1.58	3.71E-02	H3F3C	45270	C12orf72	87296
chr19	1940718	1941140	1.35	3.72E-02	CSNK1G2	-232		
chr5	178971532	178971808	1.63	3.74E-02	RUFY1	-5901	AX747985	20222
chr3	193411658	193411977	1.78	3.75E-02	HES1	-442116	OPA1	100885
chr2	96829524	96829834	1.63	3.76E-02	DUSP2	-18500	STARD7	44894
chr12	124086516	124086962	1.55	3.81E-02	DDX55	67		
chr19	2289755	2290036	1.62	3.83E-02	AX747191	-103		
chr2	25433119	25433475	1.74	3.85E-02	POMC	-41738	DNMT3A	131477
chrX	100662887	100663303	1.69	3.86E-02	HNRNPH2	-96	GLA	-94
chr1	247095432	247095691	1.56	3.88E-02	AHCTF1	-13883	ZNF695	75793
chr12	58008446	58008722	1.43	3.89E-02	SLC26A10	-5109	GEFT	4621
chr3	184925708	184926186	1.56	3.89E-02	EHHADH	45890	VPS8	396016
chr12	133485053	133485509	1.44	3.90E-02	CHFR	-21077	ZNF605	47587
chr3	156202119	156202411	1.76	3.90E-02	SSR3	70670	KCNAB1	363928
chr1	51972262	51972596	1.75	3.90E-02	TTC39A	-161644	EPS15	12566
chr14	55541364	55541676	1.90	3.92E-02	LGALS3	-54352	SOCS4	47676
chr2	61198551	61198808	1.90	3.92E-02	PEX13	-46132	REL	89928
chr10	23012178	23012493	1.67	3.94E-02	ARMC3	-204618	PIP4K2A	-8833
chr7	94250062	94250316	1.59	3.94E-02	SGCE	35332	CASD1	111019
chr12	9822126	9822429	1.70	3.94E-02	CLEC2D	-31		
chr7	25894723	25894976	1.67	3.96E-02	NPVF	-626745	NFE2L3	-296997
chr11	126081432	126081799	1.43	3.96E-02	RPUSD4	-29		
chr4	40843048	40843330	1.54	3.99E-02	NSUN7	91275	APBB2	373446
chr20	23342426	23342792	1.41	4.02E-02	GZF1	-2391		
chr9	136688795	136689147	1.56	4.03E-02	SARDH	-85492	VAV2	168475
chr5	175843488	175843793	1.49	4.07E-02	CLTB	-101		
chr3	37000195	37000519	1.63	4.10E-02	TRANK1	-97946	EPM2AIP1	34438
chr18	51884078	51884664	1.54	4.11E-02	STARD6	-3428	C18orf54	-2526
chr13	99830900	99831243	1.55	4.13E-02	DOCK9	-92412	UBAC2	-21607
chr4	3049239	3049571	1.55	4.13E-02	HTT	-27003	GRK4	84062
chr5	94417210	94417529	2.14	4.14E-02	MCTP1	202909	ANKRD32	462979
chr19	58816527	58816818	1.51	4.14E-02	ZSCAN22	-21712	ZNF8	26355

chr19	45873813	45874067	1.50	4.15E-02	ERCC2	-95		
chr19	49375397	49375791	1.37	4.15E-02	PLEKHA4	-3710	PPP1R15A	-55
chr11	65208358	65208734	1.66	4.15E-02	SCYL1	-84002	FRMD8	54505
chr13	46739676	46739943	1.67	4.18E-02	CPB2	-60599	LCP1	16649
chr17	74264344	74264632	1.60	4.18E-02	RNF157	-28098	QRICH2	39273
chr19	1904345	1904734	1.65	4.20E-02	ADAT3	-833	SCAMP4	-833
chr2	231454261	231454526	1.70	4.20E-02	CAB39	-123163	SP100	173523
chr16	64355850	64356132	1.62	4.21E-02	CDH11	799928		
chr11	125462243	125462722	1.64	4.24E-02	STT3A	-256		
chr17	61819095	61819508	1.45	4.24E-02	STRADA	-87		
chr7	129133958	129134280	1.81	4.24E-02	NRF1	-117436	AHCYL2	269255
chr8	10850883	10851170	1.58	4.25E-02	SOX7	-263005	XKR6	207848
chr3	51534108	51534380	1.40	4.26E-02	VPRBP	-243		
chr16	89877726	89878056	1.41	4.27E-02	FANCA	5174	ZNF276	89939
chr7	148680681	148680957	1.81	4.27E-02	EZH2	-99405	PDIA4	44963
chr15	72371295	72371611	1.67	4.29E-02	MYO9A	38969	NR2E3	268559
chr20	37504150	37504624	1.83	4.31E-02	FAM83D	-50568	PPP1R16B	70039
chrX	15692861	15693105	1.58	4.31E-02	CA5BP	-56		
chr19	50145034	50145426	1.53	4.33E-02	RRAS	-1830	SCAF1	-152
chr20	4970261	4970574	1.61	4.35E-02	RASSF2	-174649	SLC23A2	20521
chr1	167691015	167691291	1.74	4.35E-02	MPZL1	-34		
chr9	114688854	114689123	1.51	4.35E-02	UGCG	29783	SUSD1	248567
chr17	79068751	79068998	2.29	4.36E-02	BAIAP2	59928	AATK	70997
chr21	37692286	37692612	1.41	4.37E-02	MORC3	-38		
chr12	44152567	44152863	1.44	4.38E-02	PUS7L	-153	IRAK4	-32
chr19	40791325	40791849	1.79	4.39E-02	AKT2	-322		
chr3	46120363	46120639	1.63	4.39E-02	XCR1	-51522	CCR1	129331
chr5	123919511	123919780	1.75	4.41E-02	ZNF608	161219		
chr22	32026371	32026647	1.69	4.42E-02	PISD	301		
chr2	216979416	216979744	1.58	4.44E-02	XRCC5	5560	MARCH4	257170
chr17	73150478	73150903	1.54	4.47E-02	HN1	84		
chr7	22486638	22486915	1.80	4.48E-02	RAPGEF5	-90244	MGC87042	53021
chr19	40949071	40949371	1.50	4.49E-02	SERTAD1	-17289	SERTAD3	1061
chr16	17510572	17511153	1.65	4.52E-02	XYLT1	53875		
chr5	143569483	143569727	1.81	4.52E-02	KCTD16	19168		
chr7	129254928	129255194	1.61	4.53E-02	NRF1	3506	UBE2H	337728
chr7	111202508	111202826	1.30	4.54E-02	IMMP2L	-320		
chr11	10879223	10879499	1.57	4.56E-02	ZBED5	259		

chr1	235114097	235114454	1.63	4.56E-02	IRF2BP2	-369005	TOMM20	177980
chr4	175849845	175850335	1.64	4.56E-02	ADAM29	10581		
chr15	89019848	89020168	2.00	4.56E-02	MRPS11	9324	DET1	69904
chr13	99852761	99853130	1.50	4.58E-02	UBAC2	267		
chr2	27593234	27593510	1.67	4.58E-02	EIF2B4	-453	SNX17	-17
chr7	108210019	108210439	1.52	4.61E-02	DNAJB9	-127	THAP5	-62
chr6	14910954	14911190	1.76	4.61E-02	JARID2	-335455	CD83	793207
chr8	109455439	109455862	1.52	4.61E-02	TTC35	-202		
chr1	212588205	212588654	1.60	4.63E-02	TMEM206	-163		
chr22	19159033	19159308	1.58	4.65E-02	GSC2	-21375	SLC25A1	7130
chr3	61880738	61881044	1.74	4.65E-02	PTPRG	333648	FEZF2	478299
chr5	124082973	124083293	1.64	4.65E-02	ZNF608	-2268		
chr6	27640152	27640438	1.87	4.66E-02	ZNF184	-199398	HIST1H2BL	135414
chr8	29087058	29087392	2.00	4.66E-02	KIF13B	33385	HMBOX1	339314
chr4	110736415	110736765	1.50	4.68E-02	GAR1	-76		
chr2	37458671	37458930	1.66	4.68E-02	CEBPZ	-61	C2orf56	27
chr1	206304394	206304639	1.80	4.69E-02	C1orf186	-15870	CTSE	-12942
chr10	79789213	79789520	1.50	4.72E-02	RPS24	-4151	POLR3A	-108
chr7	36325905	36326181	1.76	4.74E-02	ANLN	-103389	EEPD1	133207
chr4	3079608	3079907	1.61	4.76E-02	RGS12	-236116	HTT	3350
chr15	75082393	75082722	1.66	4.77E-02	LMAN1L	-22636	CSK	8133
chr4	57253444	57253777	1.61	4.79E-02	AASDH	27		
chr19	6801862	6802106	1.64	4.80E-02	EMR1	-85598	VAV1	29262
chr14	81425829	81426123	1.58	4.82E-02	TSHR	4107	GTF2A1	261318
chr19	45829431	45829689	1.63	4.83E-02	CKM	-3426		
chr1	241696247	241696497	1.81	4.85E-02	KMO	692		
chr2	64751222	64751470	1.41	4.85E-02	AFTPH	-119		
chr19	10535983	10536268	1.68	4.91E-02	PDE4A	4793	KEAP1	77928
chr22	29168514	29168856	1.46	4.91E-02	XBP1	27875	HSCB	30642
chr17	73179007	73179268	1.61	4.93E-02	HN1	-28363	NUP85	-22459
chr17	1726758	1727067	1.90	4.94E-02	SMYD4	6262	SERPINF1	61654
chr22	40573740	40574068	1.76	4.96E-02	TNRC6B	-25		
chr18	9475243	9475617	1.31	4.96E-02	RALBP1	-100		
chr11	118757427	118757813	1.60	4.99E-02	CXCR5	3079	BCL9L	23993
chr6	119763935	119764260	1.74	4.99E-02	MAN1A1	-93172		
chr7	116502331	116502677	1.40	4.99E-02	CAPZA2	-59		
chr12	25539066	25539310	1.34	4.99E-02	KRAS	-135334	IFLTD1	167029
chr2	33497086	33497375	1.70	4.99E-02	RASGRP3	-241711	LTBP1	324839

Unique to GCB								
chr10	71712594	71712830	0.06	2.96E-28	H2AFY2	-99645	COL13A1	151068
chr2	234135329	234135613	0.06	7.65E-26	ATG16L1	-24746	INPP5D	210435
chr1	115885979	115886289	0.06	9.24E-25	VANGL1	-298440	NGF	-5277
chr3	48700231	48700578	0.08	5.64E-24	TMEM89	-41216	CELSR3	9576
chr7	26137839	26138170	0.08	6.41E-24	NPVF	-869900	NFE2L3	-53842
chr18	5978408	5978718	0.07	1.45E-23	TMEM200C	-86460	L3MBTL4	436347
chr2	120939650	120939886	0.11	2.87E-23	TMEM185A	41216	EPB41L5	169099
chr3	34304544	34304854	0.08	1.03E-22	PDCD6IP	464633		
chr1	110166411	110166721	0.09	1.60E-22	GSTM4	-32132	AMPD2	3290
chr1	54821700	54821900	0.07	1.62E-22	SSBP3	50292	MRPL37	155960
chr20	62918217	62918527	0.09	5.99E-22	PCMTD2	31324		
chr16	24113514	24113763	0.16	9.77E-22	CACNG3	-153237	PRKCB	266339
chr3	12758081	12758318	0.14	1.03E-21	RAF1	-52500	TMEM40	42608
chr3	15259407	15259587	0.13	1.94E-21	CAPN7	11764	SH3BP5	114607
chr3	11623776	11624007	0.04	3.73E-21	VGLL4	61506	ATG7	309882
chrX	48754406	48754649	0.13	9.81E-21	PQBP1	-667	TIMM17B	898
chr16	4511348	4511608	0.12	1.36E-20	NMRAL1	13418	DNAJA3	35620
chr15	37392240	37392440	0.09	1.88E-19	MEIS2	364		
chr19	12991419	12991701	0.17	3.07E-19	DNASE2	775		
chr19	55737020	55737291	0.09	3.55E-19	PTPRH	-16282	TMEM86B	3476
chr10	112483725	112484035	0.09	6.08E-19	PDCD4	-147716	RBM20	79725
chr7	99967329	99967665	0.11	1.24E-18	PILRA	-3571		
chr2	145427230	145427540	0.13	1.70E-18	ZEB2	-149469		
chr14	95953760	95954020	0.10	2.14E-18	GLRX5	-47433	C14orf49	-11717
chr5	692251	692561	0.10	6.27E-18	TPPP	1104	CEP72	80001
chr7	16955788	16955988	0.10	6.94E-18	AHR	-382388	AGR3	-34275
chrX	33146367	33146620	0.16	9.04E-18	DMD	83179		
chr7	10032	10276	0.07	1.09E-17	FAM20C	-182815		
chr7	22213949	22214149	0.14	1.09E-17	CDCA7L	-228507	RAPGEF5	182484
chr6	35231302	35231612	0.09	1.13E-17	DEF6	-34138	ZNF76	3947
chr5	130967696	130967937	0.18	2.04E-17	RAPGEF6	3112	CDC42SE2	368115
chr12	56696472	56696683	0.14	2.48E-17	CS	-2403		
chr19	49652931	49653277	0.08	3.34E-17	HRC	5577	PPFIA3	30441
chr1	198607119	198607364	0.20	4.33E-17	PTPRC	-895		
chr14	75699356	75699666	0.13	4.94E-17	TMED10	-56162	FOS	-45970
chr13	50074476	50074786	0.14	5.07E-17	PHF11	4830	RCBTB1	85088
chr4	186401482	186401734	0.16	6.78E-17	CCDC110	-8695	PDLIM3	55104

chr18	10033	10281	0.13	7.52E-17	USP14	-148326		
chr10	121920644	121920954	0.11	9.70E-17	PPAPDC1A	-295667	SEC23IP	268576
chr2	66661998	66662258	0.15	1.16E-16	MEIS1	-404		
chr20	62918583	62918893	0.12	1.30E-16	PCMTD2	31690		
chr11	83393368	83393633	0.09	2.36E-16	CCDC90B	-396124		
chr11	76093316	76093590	0.21	3.66E-16	PRKRIR	-1573		
chr19	12917616	12917868	0.23	4.78E-16	RNASEH2A	314		
chr8	56922647	56922947	0.17	5.01E-16	RPS20	64343	LYN	130411
chr1	20730816	20731142	0.07	5.18E-16	CAMK2N1	81749	VWA5B1	113567
chr6	139337557	139337793	0.22	7.23E-16	HECA	-118574	REPS1	-28277
chr13	49942913	49943149	0.15	7.98E-16	CAB39L	32704	CDADC1	120928
chr17	30466148	30466381	0.25	1.18E-15	RHOT1	-3208		
chr1	214601269	214601505	0.18	1.35E-15	PTPN14	123255	SMYD2	146822
chr2	122246953	122247263	0.18	1.35E-15	TFCP2L1	-204330	CLASP1	159944
chr10	115720472	115720838	0.06	1.46E-15	ADRB1	-83151	NHLRC2	106235
chr19	44084545	44084855	0.17	2.21E-15	XRCC1	-4970	BC071811	4
chr1	26437509	26437745	0.15	2.23E-15	PDIK1L	-641		
chrX	48857998	48858260	0.19	2.33E-15	GRIPAP1	546		
chr6	159477130	159477421	0.19	2.38E-15	FNDC1	-113153	TAGAP	-11092
chr15	64889460	64889683	0.14	2.43E-15	ZNF609	97953	OAZ2	105890
chr10	94003108	94003334	0.14	4.83E-15	CPEB3	47623	BTAF1	319485
chr16	25771331	25771567	0.17	5.17E-15	HS3ST4	68102		
chr6	45485797	45486033	0.13	5.46E-15	SUPT3H	-140245	CLIC5	562170
chr19	42721166	42721408	0.16	6.23E-15	ZNF526	-3205	DEDD2	526
chr2	89534569	89534879	0.05	6.28E-15	O1/O11 and JK2	-355838	RPIA	543548
chr12	109747504	109747748	0.17	8.47E-15	FOXN4	-601		
chr18	12703863	12704102	0.16	8.69E-15	CEP76	-1280	PSMG2	919
chr20	61069632	61069911	0.22	1.02E-14	SLCO4A1	-204025	GATA5	-18746
chr13	41226762	41227008	0.15	1.95E-14	FOXO1	13849	COG6	997121
chr2	143629080	143629327	0.16	5.21E-14	LRP1B	-739934	KYNU	-5991
chr8	48109337	48109690	0.16	5.24E-14	CEBPD	541212		
chr20	57738831	57739132	0.24	6.57E-14	SLMO2	-121081	ZNF831	-27093
chr16	788144	788388	0.20	7.67E-14	NARFL	2731	HAGHL	11000
chr7	101497750	101498060	0.19	8.96E-14	SH2B2	-430500	CUX1	37023
chrX	19002520	19002756	0.21	9.16E-14	PHKA2	-158		
chr2	75062388	75062700	0.22	1.08E-13	POLE4	-123231	HK2	2762
chr5	110221682	110221992	0.30	1.21E-13	TSLP	-185553	SLC25A46	147083
chr14	52118144	52118434	0.14	1.55E-13	FRMD6	-287		

chr8	42128468	42128704	0.23	1.69E-13	IKBKB	-243		
chr12	113772146	113772390	0.30	1.75E-13	SLC24A6	657		
chr10	76349059	76349369	0.14	1.75E-13	MYST4	-237165	ADK	438249
chr12	49594931	49595241	0.22	2.28E-13	TUBA1B	-69782	TUBA1C	-63779
chr8	29743626	29743862	0.18	2.36E-13	DUSP4	-535559	TMEM66	196905
chr17	70506592	70506828	0.16	2.58E-13	SOX9	389549	SLC39A11	582143
chr7	10395515	10395825	0.11	3.42E-13	NDUFA4	584143		
chr1	3636696	3636932	0.17	3.51E-13	KIAA0495	27072	TP73	67685
chr1	6550557	6550820	0.19	3.65E-13	TNFRSF25	-24434	PLEKHG5	6795
chr11	118799894	118800111	0.24	3.84E-13	UPK2	-27023	BCL9L	-18390
chr3	44040583	44040864	0.06	3.86E-13	C3orf23	-339220	ABHD5	308349
chr5	154137060	154137370	0.23	4.10E-13	LARP1	2326	C5orf4	92998
chr17	61926317	61926582	0.13	4.35E-13	SMARCD2	-6099	CSH2	24639
chr12	56615074	56615316	0.21	4.64E-13	OBFC2B	-2930	RNF41	509
chrX	37853094	37853442	0.04	5.80E-13	SYTL5	-39519	CXorf27	3198
chrX	43513845	43514151	0.21	6.05E-13	MAOA	-1411		
chr8	41435838	41436082	0.13	6.38E-13	AGPAT6	253		
chr21	40875065	40875375	0.22	6.59E-13	B3GALT5	-154034	SH3BGR	51440
chr19	19517209	19517445	0.22	7.07E-13	GATAD2A	20685	TSSK6	109142
chrX	49020301	49020537	0.23	7.24E-13	PLP2	-7854	MAGIX	1238
chr18	74207095	74207584	0.15	7.85E-13	ZNF236	-328776	ZNF516	-32243
chr11	57103015	57103215	0.21	8.09E-13	P2RX3	-2834	SSRP1	236
chr12	95833495	95833816	0.19	8.55E-13	METAP2	-34166	VEZT	222134
chr9	119522900	119523228	0.18	9.20E-13	TRIM32	73483	ASTN2	654253
chr13	30989760	30990009	0.14	9.23E-13	KATNAL1	-108722	HMGB1	50196
chr16	70099879	70100140	0.23	9.26E-13	PDXDC2	-75899	PDPR	-47519
chr7	99789329	99789639	0.16	1.04E-12	PVRIG	-27388	STAG3	13946
chr15	38943978	38944251	0.18	1.09E-12	THBS1	-929165	RASGRP1	-87108
chr5	148737146	148737406	0.16	1.10E-12	PCYOX1L	-294		
chr1	6052055	6052343	0.22	1.13E-12	NPHP4	332		
chr5	147162255	147162608	0.22	1.19E-12	JAKMIP2	-180		
chr11	66034752	66035025	0.17	1.29E-12	RAB1B	-1167		
chr7	99790549	99790859	0.12	1.42E-12	PVRIG	-26168	STAG3	15166
chr6	150325657	150326005	0.19	2.24E-12	RAET1L	20837	ULBP1	40688
chr1	24872202	24872512	0.11	2.33E-12	C1orf130	-10245	RCAN3	42970
chr3	31986673	31986932	0.21	2.42E-12	OSBPL10	36435	STT3B	412312
chrX	48768356	48768599	0.32	2.50E-12	SLC35A2	757		
chr15	42411146	42411456	0.21	2.60E-12	PLA2G4D	-24549	PLA2G4F	37509

chr1	206732659	206732903	0.25	2.85E-12	RASSF5	51902	LGTN	53123
chr14	56046523	56046833	0.24	3.04E-12	KTN1	-247		
chr18	658156	658416	0.20	3.50E-12	C18orf56	54	TYMS	682
chr7	134832157	134832395	0.11	3.61E-12	TMEM140	-490		
chr1	179335556	179335866	0.19	3.68E-12	SOAT1	72694	NPHS2	209373
chr3	126105525	126105835	0.23	3.84E-12	KLF15	-29444	ZXDC	89082
chr19	10203844	10204154	0.25	4.18E-12	ANGPTL6	9426	RDH8	80074
chr7	142503655	142503965	0.17	5.16E-12	EPHB6	-48982	TCRBC2	9443
chr11	45921389	45921683	0.17	5.54E-12	C11orf94	7297	MAPK8IP1	14334
chr17	7137432	7137680	0.26	6.14E-12	DVL2	307		
chr10	73868615	73868925	0.19	7.21E-12	SPOCK2	-19980	ASCC1	106924
chr8	30659229	30659539	0.25	7.26E-12	PPP2CB	10968	UBXN8	57694
chr1	19922891	19923128	0.30	8.40E-12	C1orf151	-457		
chr2	87303435	87303715	0.22	8.72E-12	PLGLB1	-744031	LOC285074	-25886
chr12	105745389	105745627	0.17	9.70E-12	APPL2	-115500	NUAK1	788303
chr14	22902192	22902592	0.17	1.09E-11	TCRDV2	-25697	TRA	426628
chr18	78016187	78016436	0.13	1.13E-11	PARD6G	-10915		
chr11	62474952	62475202	0.26	1.15E-11	GNG3	-37		
chr17	62031864	62032174	0.27	1.18E-11	CD79B	-22315	SCN4A	18259
chr7	25016715	25017025	0.13	1.20E-11	DFNA5	-219787	OSBPL3	2890
chr1	78295660	78295896	0.10	1.21E-11	NEXN	-58422	FAM73A	50469
chr12	113569378	113569642	0.27	1.21E-11	RASAL1	4511	DTX1	73848
chr11	67123796	67124181	0.28	1.22E-11	POLD4	-2972		
chr13	115077452	115077784	0.19	1.23E-11	ZNF828	-2347		
chr18	2570806	2571116	0.24	1.32E-11	NDC80	-549	METTL4	528
chr1	155349484	155349735	0.04	1.47E-11	RUSC1	58970	ASH1L	182714
chrX	131623217	131623527	0.21	1.50E-11	MBNL3	-49030	HS6ST2	472051
chr1	31364698	31365008	0.14	1.83E-11	LAPTM5	-134170	SDC3	16627
chr10	64028340	64028607	0.20	1.84E-11	RTKN2	-8		
chr10	90640603	90640857	0.22	2.26E-11	STAMBPL1	704		
chr22	24129644	24129919	0.19	2.39E-11	SMARCB1	632		
chr14	22984483	22984784	0.14	2.39E-11	TCRDV2	56545	DAD1	73509
chr1	87916339	87916649	0.24	2.47E-11	LMO4	122343		
chr5	66300262	66300540	0.24	2.77E-11	MAST4	175797	CD180	192216
chr20	55819946	55820277	0.08	2.98E-11	BMP7	21595	TFAP2C	615754
chr18	12420992	12421308	0.14	3.54E-11	SLMO1	13255	SPIRE1	236762
chr17	56199307	56199617	0.24	3.60E-11	OR4D1	-33053	DYNLL2	38682
chr19	55678138	55678521	0.07	3.62E-11	TNNI3	-9230	SYT5	13390

chr2	219741653	219741969	0.27	3.66E-11	WNT10A	-3444		
chr17	58042180	58042424	0.18	3.85E-11	RNFT1	-185		
chr4	38818140	38818551	0.26	4.07E-11	TLR1	-11934	TLR6	12812
chr10	111768443	111768753	0.20	4.12E-11	ADD3	887		
chr17	7743288	7743624	0.05	4.66E-11	KDM6B	221		
chrX	129304624	129304890	0.22	4.96E-11	AIFM1	-4949	RAB33A	-1016
chr19	49649470	49649720	0.13	5.00E-11	HRC	9086	PPFIA3	26932
chr1	150244732	150245042	0.24	5.35E-11	APH1A	-3355	C1orf54	-315
chr13	115078731	115079005	0.19	6.17E-11	ZNF828	-1097		
chr10	112201476	112201796	0.12	6.78E-11	SMNDC1	-136929	DUSP5	-55989
chr4	41258692	41259005	0.05	7.22E-11	UCHL1	-49		
chr6	160114377	160114665	0.15	7.34E-11	SOD2	-168		
chrX	49022733	49023065	0.10	7.74E-11	PLP2	-5374	MAGIX	3718
chr12	52517568	52518033	0.27	7.85E-11	C12orf44	54043	KRT80	67983
chr10	103879292	103879602	0.25	9.23E-11	LDB1	763		
chr1	9910301	9910611	0.26	9.23E-11	CLSTN1	-25906	CTNNBIP1	59860
chr16	31880808	31881125	0.28	1.02E-10	ZNF267	-4112		
chr2	87303889	87304199	0.26	1.02E-10	PLGLB1	-743562	LOC285074	-26355
chr12	32286586	32286902	0.17	1.05E-10	FGD4	-368297	BICD1	26559
chr6	35104031	35104341	0.20	1.12E-10	TCP11	5001	ANKS1A	247148
chr9	135285272	135285594	0.23	1.13E-10	TTF1	-3212		
chr2	219151818	219152070	0.34	1.14E-10	TMBIM1	5336	PNKD	16829
chr12	120104298	120104534	0.22	1.25E-10	PRKAB1	-1345		
chr1	161691191	161691501	0.28	1.27E-10	FCRLB	-1097		
chr11	111749425	111749661	0.32	1.31E-10	C11orf1	-405	FDXACB1	610
chr8	10857080	10857416	0.20	1.37E-10	SOX7	-269226	XKR6	201627
chr22	46984311	46984628	0.15	1.41E-10	CELSR1	-51403	GRAMD4	-38178
chr2	71357871	71358229	0.20	1.49E-10	MCEE	-656	MPHOSPH10	606
chr7	73089113	73089423	0.24	1.57E-10	VPS37D	7094	DNAJC30	8513
chr6	57037344	57037594	0.13	1.65E-10	BAG2	365		
chr12	54149300	54149610	0.27	1.65E-10	HOXC13	-183121	CALCOCO1	-28148
chr12	121974430	121974630	0.18	1.75E-10	KDM2B	44390	RNF34	136628
chrX	48595889	48596179	0.21	1.76E-10	GATA1	-48948	SUV39H1	40903
chr1	193506539	193506849	0.33	1.78E-10	B3GALT2	-350951		
chr1	100817777	100817973	0.20	1.81E-10	CDC14A	-148		
chr14	76657670	76657980	0.26	1.96E-10	ESRRB	-179865	C14orf118	39566
chr2	86946773	86947180	0.25	1.99E-10	RMND5A	-437		
chr1	1677446	1678011	0.06	1.99E-10	CDC2L1	-21954	NADK	32180

chr19	1490482	1490760	0.14	2.05E-10	REEP6	-544	PCSK4	-214
chrX	40856435	40856745	0.27	2.49E-10	MED14	-261786	USP9X	-88298
chr2	86947262	86947406	0.29	2.50E-10	RMND5A	-80		
chr5	40679466	40679666	0.27	2.57E-10	PTGER4	-466		
chr13	44849644	44849954	0.23	2.81E-10	ENOX1	-646186	SERP2	-98179
chr14	68750691	68751001	0.24	2.85E-10	RAD51L1	464337	ZFP36L1	508939
chr12	32086529	32086839	0.24	2.96E-10	BICD1	-173501	H3F3C	-141509
chr16	29678019	29678270	0.17	3.14E-10	QPRT	-12296	SPN	3845
chr6	157138635	157138871	0.16	3.69E-10	ARID1B	39667	C6orf35	606040
chrX	47479226	47479470	0.20	3.83E-10	SYN1	-92		
chr2	66794118	66794354	0.23	3.92E-10	ETAA1	-830206	MEIS1	131704
chr3	57262507	57262799	0.18	4.30E-10	APPL1	888		
chr5	180671155	180671425	0.20	4.37E-10	GNB2L1	-384		
chr7	8009504	8009814	0.25	4.47E-10	GLCCI1	1236	ICA1	292023
chrY	22897227	22897552	0.17	4.51E-10	RPS4Y2	-20564	EIF1AY	159779
chr17	36955628	36955938	0.19	4.61E-10	PIP4K2B	375		
chr17	7476709	7477098	0.23	5.19E-10	CD68	-5901	EIF4A1	1302
chr6	31797511	31797821	0.23	5.38E-10	HSPA1B	2154	NEU1	33043
chr12	67010166	67010476	0.26	5.69E-10	GRIP1	62604	HELB	313965
chr20	56477369	56477679	0.21	5.72E-10	PMEPA1	-192493	PPP4R1L	406971
chr6	155739544	155739953	0.24	5.72E-10	TFB1M	-104123	NOX3	37288
chr6	32121583	32121909	0.13	5.92E-10	PRRT1	-2026	PPT2	445
chr6	33291061	33291297	0.20	6.06E-10	DAXX	-386		
chr1	182614109	182614419	0.28	6.23E-10	RGS16	-40716	RGS8	27803
chr6	35300175	35300485	0.19	6.33E-10	PPARD	-10005	DEF6	34735
chr8	29743294	29743530	0.24	6.70E-10	DUSP4	-535227	TMEM66	197237
chr1	19229014	19229386	0.06	7.65E-10	ALDH4A1	93		
chr7	99792882	99793192	0.24	7.67E-10	PVRIG	-23835	STAG3	17499
chr1	36621275	36621606	0.20	8.72E-10	MAP7D1	-362		
chr7	98667788	98668024	0.18	9.84E-10	SMURF1	73817	TRRAP	191793
chr17	48980951	48981430	0.27	1.05E-09	TOB1	-39778	SPAG9	217035
chr7	68958982	68959278	0.23	1.08E-09				
chr6	160183220	160183599	0.30	1.16E-09	ACAT2	421		
chr7	7222453	7222763	0.28	1.25E-09	C1GALT1	362		
chr1	205717949	205718259	0.35	1.35E-09	SLC45A3	-68474	NUCKS1	1257
chr1	204283440	204283689	0.32	1.40E-09	GOLT1A	-100345	PPP1R15B	97379
chr22	37988561	37988798	0.34	1.47E-09	GGA1	-15823	LGALS2	-12656
chr15	74340719	74340955	0.27	1.54E-09	ISLR2	-81906	PML	53823

chr8	61824308	61824549	0.29	1.62E-09	RLBP1L1	-376096	CHD7	233090
chr4	153589026	153589262	0.33	1.68E-09	FBXW7	-132959	TMEM154	12047
chr2	230999416	230999652	0.30	1.93E-09	SLC16A14	-65915	SP110	85293
chr7	40873618	40873930	0.18	1.94E-09	C7orf10	699199	INHBA	868932
chr9	119526035	119526345	0.21	1.99E-09	TRIM32	76609	ASTN2	651127
chr2	89985855	89986381	0.20	2.01E-09	O1/O11 and JK2	95556		
chr8	79428243	79428561	0.15	2.08E-09	PKIA	66		
chr14	100070576	100070844	0.22	2.20E-09	HHIPL1	-40770	CCNK	122971
chr7	140396235	140396490	0.27	2.31E-09	NDUFB2	-118		
chr2	217277312	217277556	0.21	2.38E-09	SMARCAL1	-39		
chr1	147013117	147013364	0.32	2.42E-09	BCL9	59		
chr1	10125	10395	0.17	2.43E-09	OR4F5	-58831		
chr8	23077251	23077561	0.37	2.47E-09	TNFRSF10D	-55866	TNFRSF10A	5233
chr16	30196400	30196644	0.27	2.57E-09	GIYD2	-8695	CORO1A	1596
chr7	90338955	90339231	0.14	2.67E-09	PFTK1	381		
chr19	18108599	18108966	0.15	2.67E-09	KCNN1	46672	IL12RB1	88959
chr3	71191606	71191864	0.38	2.68E-09	FOXP1	441405		
chr18	10446	10756	0.17	2.69E-09	USP14	-147882		
chr17	62770406	62770716	0.23	2.70E-09	SMURF2	-112175	LOC146880	7061
chrX	47880760	47881070	0.23	2.76E-09	SPACA5	17181	ZNF630	49593
chr20	52551088	52551398	0.28	2.79E-09	ZNF217	-351536	BCAS1	136061
chr12	56224351	56224638	0.16	2.80E-09	DNAJC14	-1997		
chr17	40594279	40594589	0.26	2.81E-09	PTRF	-19160	ATP6V0A1	-16428
chr17	16189918	16190228	0.32	3.03E-09	CENPV	66739	PIGL	69564
chr5	65220511	65220821	0.35	3.08E-09	ERBB2IP	-1718		
chr2	42793198	42793508	0.28	3.36E-09	MTA3	-2318		
chr6	154582828	154583064	0.16	3.42E-09	IPCEF1	94954	OPRM1	222503
chr5	153569905	153570215	0.25	3.71E-09	GALNT10	-235		
chr19	18633440	18633702	0.31	4.02E-09	ELL	-634		
chr1	38259466	38259776	0.23	4.14E-09	MANEAL	-153		
chr7	129073919	129074158	0.31	4.17E-09	NRF1	-177516	AHCYL2	209175
chr20	633826	634136	0.29	4.18E-09	SRXN1	-91		
chr7	136088390	136088700	0.23	4.54E-09	CHRM2	-465287	MTPN	-426341
chr1	87221549	87221859	0.33	4.94E-09	SH3GLB1	51447	SEP15	158403
chr7	27774243	27774509	0.26	4.99E-09	HIBADH	-71774	TAX1BP1	-5362
chr5	177557800	177558070	0.20	5.04E-09	N4BP3	17379	NHP2	23026
chr14	96119085	96119395	0.30	5.10E-09	TCL1A	61293	GLRX5	117917
chr3	10029004	10029240	0.39	5.23E-09	TMEM111	-600		

chr2	191513273	191513583	0.31	5.66E-09	NAB1	-420		
chr19	58326051	58326292	0.29	5.68E-09	ZNF552	109		
chr5	79571246	79571556	0.25	6.11E-09	SPZ1	-44389	SERINC5	-19531
chr1	52869227	52869537	0.26	6.19E-09	PRPF38A	-837	ORC1L	749
chr1	26437965	26438233	0.29	6.33E-09	PDIK1L	-169		
chr11	47290634	47290942	0.21	6.41E-09	MADD	-411		
chr13	67460285	67460595	0.32	6.41E-09	PCDH9	344028		
chrX	129065452	129065688	0.25	6.64E-09	BCORL1	-51099	UTP14A	25411
chr11	911036	911449	0.28	6.72E-09	CHID1	-369		
chr8	62844218	62844463	0.21	7.51E-09	NKAIN3	-317160	ASPH	-217142
chr17	55629539	55629816	0.36	7.81E-09	MSI2	295747	MRPS23	297721
chr17	58428455	58428765	0.29	7.89E-09	USP32	40976	CA4	201308
chr1	204476873	204477183	0.39	8.07E-09	PIK3C2B	-17554	MDM4	-8483
chr19	42927265	42927591	0.12	8.58E-09	CNFN	-32984	LIPE	4150
chr2	97628179	97628489	0.27	8.98E-09	SEMA4C	-92599	FAHD2B	132248
chr11	34460697	34460933	0.29	9.02E-09	CAT	337		
chr3	150127782	150128029	0.30	9.28E-09	EIF2A	-136668	TSC22D2	1118
chr12	6570515	6570825	0.35	9.82E-09	VAMP1	9173	TAPBPL	9493
chr6	152623349	152623585	0.26	1.07E-08	SYNE1	335067	NR3A1	459735
chr2	39102818	39103084	0.35	1.10E-08	DHX57	70		
chr9	100749836	100750146	0.27	1.26E-08	NANS	-68691	ANP32B	4502
chr1	15944398	15944727	0.31	1.30E-08	DDI2	493		
chr14	21944571	21944826	0.38	1.31E-08	TOX4	-636	RAB2B	433
chr3	31975586	31975896	0.18	1.36E-08	OSBPL10	47497	STT3B	401250
chr6	33378494	33378730	0.32	1.37E-08	PHF1	-161		
chr16	82608986	82609231	0.16	1.43E-08	MPHOSPH6	-405280	CDH13	-51469
chr9	112676641	112676954	0.30	1.44E-08	PALM2-AKAP2	134221	TXN	341980
chr12	54892217	54892527	0.32	1.48E-08	NCKAP1L	877		
chr15	45997735	45998009	0.31	1.58E-08	SQRDL	70616		
chr8	70025944	70026188	0.24	1.71E-08	SULF1	-352793		
chr7	99699522	99699809	0.31	1.81E-08	MCM7	-239	AP4M1	536
chr8	42995232	42995544	0.30	1.83E-08	HGSNAT	-204		
chr7	64712221	64712658	0.29	1.86E-08	ERV3	-245319	ZNF92	-126328
chr17	4851991	4852227	0.31	2.01E-08	ENO3	-2277	PFN1	-284
chr16	15736655	15736936	0.32	2.04E-08	KIAA0430	213		
chr7	27963370	27963606	0.19	2.10E-08	TAX1BP1	183750	JAZF1	256949
chr9	112888351	112888897	0.26	2.15E-08	TXN	130154	PALM2-AKAP2	346047
chr10	99078707	99079055	0.12	2.18E-08	FRAT1	-141		

chr11	67417513	67417829	0.41	2.18E-08	ACY3	459		
chr6	13262009	13262274	0.29	2.21E-08	TBC1D7	66628	PHACTR1	544309
chr19	55690657	55690967	0.26	2.25E-08	SYT5	908		
chr10	99094457	99094798	0.15	2.27E-08	FRAT2	-170		
chr18	63533	63843	0.16	2.38E-08	USP14	-94795		
chr11	63535179	63535489	0.34	2.42E-08	C11orf95	779		
chr17	76151007	76151293	0.23	2.42E-08	SYNGR2	-13521	C17orf99	8716
chr10	63656598	63656882	0.30	2.47E-08	ARID5B	-4703		
chrX	49969025	49969358	0.35	2.55E-08	AKAP4	-3528		
chr8	59562280	59562516	0.33	2.59E-08	NSMAF	9568	SDCBP	96670
chr7	43691290	43691622	0.26	2.83E-08	STK17A	68764	C7orf44	77627
chr1	68201644	68201883	0.34	2.98E-08	GADD45A	50881	GNG12	97378
chr8	107669769	107670093	0.14	3.08E-08	ABRA	112541	OXR1	387458
chr1	198633340	198633650	0.39	3.13E-08	PTPRC	25358		
chr2	166450833	166451143	0.26	3.16E-08	CSRNP3	22098	GALNT3	199815
chr22	26828588	26828898	0.32	3.27E-08	ASPHD2	3463	HPS4	46808
chr9	126959523	126959901	0.31	3.37E-08	NEK6	-60531	LHX2	185823
chr1	31224958	31225268	0.33	3.41E-08	MATN1	-28681	LAPTM5	5570
chrX	47225338	47225843	0.14	3.62E-08	ZNF157	-4408		
chr12	121974117	121974353	0.29	3.84E-08	KDM2B	44685	RNF34	136333
chr3	196949745	196949981	0.26	3.86E-08	MFI2	-193177	DLG1	75584
chr9	141054147	141054457	0.23	4.04E-08	TUBB4Q	-15149	CACNA1B	282061
chr3	42201200	42201653	0.36	4.06E-08	TRAK1	68681	CCK	104972
chr12	54120982	54121218	0.37	4.11E-08	CALCOCO1	207		
chr5	137203311	137203621	0.26	4.76E-08	MYOT	-79		
chr11	75479467	75479791	0.16	4.85E-08	DGAT2	-149		
chr7	101497208	101497547	0.24	5.10E-08	SH2B2	-431027	CUX1	36496
chr14	88470807	88471117	0.39	5.41E-08	GPR65	-534		
chr3	127278870	127279180	0.37	5.44E-08	TPRA1	30543	PLXNA1	571523
chr15	38369137	38369447	0.29	5.77E-08	SPRED1	-175760	TMCO5A	141834
chrX	129220928	129221238	0.30	5.80E-08	ELF4	23605	BCORL1	104414
chr18	21596620	21596930	0.40	5.86E-08	CABYR	-122180	LAMA3	327213
chr7	4792927	4793173	0.22	5.89E-08	KIAA0415	-22214	FOXK1	71120
chr12	50643628	50643938	0.37	6.14E-08	LASS5	-82686	LIMA1	33570
chr7	92219100	92219534	0.24	6.29E-08	PEX1	-61472	CDK6	246624
chr17	21189891	21190201	0.22	6.53E-08	KCNJ12	-89653	MAP2K3	2078
chr15	42787190	42787434	0.29	6.68E-08	SNAP23	-523		
chr1	64060072	64060382	0.34	7.17E-08	ROR1	-179463	PGM1	1280

chr15	98689770	98689970	0.37	7.20E-08	IGF1R	-502891		
chr11	121246623	121246936	0.30	7.23E-08	SORL1	-76181	SC5DL	83392
chr12	106721294	106721604	0.25	7.49E-08	CKAP4	-79736	POLR3B	-29987
chr12	111538597	111538907	0.24	7.49E-08	SH2B3	-305000	CUX2	66923
chr6	132816731	132817041	0.39	8.07E-08	MOXD1	-94222	STX7	17451
chr7	101532054	101532290	0.18	8.15E-08	SH2B2	-396233	CUX1	71290
chr3	20227417	20227662	0.35	8.20E-08	SGOL1	143		
chr20	57365394	57365633	0.26	8.70E-08	GNAS	-49281	NPEPL1	97652
chr18	23878291	23878589	0.23	9.09E-08	TAF4B	72031	KCTD1	250060
chr9	695233	695543	0.25	9.18E-08	DMRT1	-146302	KANK1	190685
chr7	5862418	5862889	0.15	9.34E-08	OCM	-57775	RNF216	-41362
chr9	27386852	27387162	0.33	9.90E-08	IFNK	-137305	C9orf11	-89870
chr19	2398779	2399089	0.29	1.01E-07	TMPRSS9	9150	TIMM13	28941
chrX	48755245	48755595	0.17	1.02E-07	TIMM17B	6	PQBP1	225
chr19	2678175	2678485	0.22	1.07E-07	GNG7	24416	GADD45B	202195
chr12	49628968	49629212	0.26	1.10E-07	TUBA1B	-103786	TUBA1C	-29775
chr7	67079885	67080195	0.16	1.18E-07	TYW1	618223		
chr19	35819821	35820060	0.30	1.21E-07	CD22	-138		
chr8	38644496	38644745	0.18	1.33E-07	TACC1	-101		
chr8	96960636	96960946	0.30	1.37E-07	GDF6	212229	PLEKHF2	814753
chr19	53898358	53898682	0.37	1.45E-07	ZNF765	123		
chr8	17013247	17013528	0.13	1.49E-07	ZDHHC2	-448		
chr20	25667403	25667715	0.33	1.51E-07	NANP	-62911	ZNF337	9910
chr15	70600760	70601077	0.28	1.55E-07	TLE3	-210663	UACA	454931
chr12	56769384	56769772	0.33	1.59E-07	APOF	-12995	TIMELESS	73622
chrX	12976609	12976919	0.20	1.60E-07	TMSB4X	-16461	TLR8	52006
chr1	53924470	53924721	0.17	1.61E-07	DMRTB1	-476		
chr1	228325969	228326279	0.30	1.61E-07	GUK1	-1805		
chr15	40972387	40972732	0.28	1.61E-07	RAD51	-14767	CASC5	86113
chr12	13254096	13254406	0.28	1.66E-07	EMP1	-95351	GSG1	-5542
chr14	45603423	45603709	0.18	1.66E-07	FANCM	-1570	FKBP3	443
chr7	104909474	104909803	0.18	1.69E-07	SRPK2	119702	MLL5	255002
chrX	118827060	118827458	0.31	1.74E-07	SEPT6	74		
chr1	24235411	24235680	0.27	1.88E-07	FUCA1	-40725	CNR2	4271
chr10	5882911	5883324	0.21	1.88E-07	FBXO18	-53231	GDI2	-27606
chr11	85021591	85021830	0.17	2.17E-07	DLG2	316603		
chr17	40439364	40439627	0.31	2.17E-07	STAT5A	-69		
chr1	43609182	43609500	0.30	2.18E-07	SLC2A1	-184494	EBNA1BP2	28900

chr19	59118741	59119051	0.24	2.25E-07	MZF1	-33954		
chr17	64487385	64487695	0.25	2.26E-07	CACNG5	-385808	PRKCA	188614
chr11	65667816	65668126	0.25	2.33E-07	FOSL1	26		
chr16	85587297	85587533	0.36	2.37E-07	KIAA0182	-59509	KIAA0513	526005
chr16	31707001	31707311	0.27	2.44E-07	CSDAP1	-126311	ZNF720	-17410
chr11	71938757	71938993	0.38	2.45E-07	INPPL1	2993	PHOX2A	16345
chr19	44123790	44124031	0.30	2.60E-07	ZNF428	103		
chr3	46968392	46968775	0.28	2.69E-07	NBEAL2	-52589	PTH1R	49348
chrX	155260155	155260399	0.19	2.76E-07	WASH1	10310		
chr1	9970711	9970955	0.33	2.79E-07	CTNNBIP1	-517		
chr19	17866760	17867059	0.31	2.91E-07	B3GNT3	-39009	MAP1S	36607
chr21	45196135	45196419	0.35	2.99E-07	CSTB	-18		
chr10	126336445	126336755	0.30	2.99E-07	FAM53B	96330	LHPP	186188
chr12	44200270	44200580	0.33	3.23E-07	TWF1	-247		
chr4	74088760	74088996	0.41	3.27E-07	COX18	-153406	ANKRD17	35624
chr22	18539055	18539367	0.37	3.29E-07	MICAL3	-31886	PEX26	-21475
chr17	7741785	7742095	0.41	3.38E-07	KDM6B	-1295		
chr15	44580003	44580313	0.46	3.47E-07	CASC4	-771		
chr10	131733033	131733283	0.24	3.53E-07	EBF3	28933	MGMT	467704
chr9	130667483	130667793	0.34	3.55E-07	ST6GALNAC6	-5767	ST6GALNAC4	11667
chr10	112033977	112034333	0.21	3.61E-07	SMNDC1	30552	MXI1	66792
chr1	43833963	43834273	0.30	3.80E-07	ELOVL1	-419		
chr19	3854321	3854582	0.35	4.05E-07	MATK	-68037	ZFR2	14575
chr12	1704438	1704682	0.29	4.08E-07	FBXL14	-1229		
chr6	15586142	15586459	0.24	4.08E-07	DTNBP1	76970	JARID2	339774
chrX	48858635	48858940	0.21	4.13E-07	GRIPAP1	-113		
chr8	59988715	59989087	0.35	4.24E-07	NSMAF	-416935	TOX	42866
chr1	11538921	11539200	0.25	4.44E-07	PTCHD2	-234		
chr16	24550502	24550810	0.19	4.44E-07	RBBP6	-252		
chr9	98268179	98268453	0.33	4.63E-07	FANCC	-188325	PTCH1	2515
chr2	179313645	179313955	0.30	4.72E-07	DFNB59	-2363	PRKRA	2158
chr12	65059309	65059509	0.34	4.75E-07	RASSF3	55116	GNS	93817
chr21	34863791	34864119	0.22	5.15E-07	DNAJC28	68		
chr6	159638769	159639104	0.32	5.36E-07	FNDC1	48508	SOD2	475416
chr17	75720699	75721009	0.23	5.40E-07	TNRC6C	-279464	SEPT9	443362
chr10	116286633	116286991	0.19	5.46E-07	AFAP1L2	-122297	ABLIM1	131246
chr19	36869827	36870107	0.37	5.48E-07	ZFP14	-11094	ZFP82	39583
chr17	56065722	56066032	0.33	5.68E-07	VEZF1	-262		

chr16	3301220	3301476	0.21	5.79E-07	ZNF200	-15892	MEFV	5279
chr7	101458285	101458650	0.38	5.89E-07	CUX1	-2414		
chr1	44302037	44302482	0.21	6.12E-07	ARTN	-99394	ST3GAL3	129042
chr7	7310813	7311113	0.21	6.20E-07	C1GALT1	88717	COL28A1	264497
chr1	84211947	84212257	0.39	6.26E-07	TTLL7	252731		
chr5	156644554	156645081	0.40	6.47E-07	CYFIP2	-48273	ITK	36911
chr19	56150477	56150713	0.37	6.78E-07	ZNF581	-4391	ZNF580	-1797
chr16	89227745	89228055	0.27	6.83E-07	CDH15	-10263	ACSF3	67646
chr6	36087072	36087334	0.21	6.84E-07	MAPK13	-11059	MAPK14	91749
chr4	42634724	42635034	0.31	6.89E-07	ATP8A1	24243	SHISA3	235023
chr16	17463298	17463747	0.33	7.23E-07	XYLT1	101215	AK310228	998526
chr9	130769962	130770331	0.46	7.77E-07	DPM2	-69384	NAIF1	59452
chr10	75571515	75571792	0.13	7.89E-07	NDST2	-65		
chr6	107437824	107438134	0.44	7.96E-07	C6orf203	88572	PDSS2	342800
chr1	93426096	93426348	0.30	8.15E-07	FAM69A	857		
chr2	153574505	153574741	0.30	8.35E-07	PRPF40A	-648	ARL6IP6	200
chr8	125538875	125539125	0.36	8.89E-07	TATDN1	12329	RNF139	51992
chr15	48624076	48624360	0.20	8.91E-07	DUT	597		
chr3	9438296	9438596	0.16	9.07E-07	THUMPD3	33729	LHFPL4	157040
chrX	48534942	48535252	0.26	9.25E-07	WAS	-7089	WDR13	79196
chr1	160807327	160807656	0.31	9.30E-07	CD244	25153	LY9	41564
chr17	37123525	37123809	0.33	9.52E-07	LASP1	97555	PLXDC1	184235
chrX	53024645	53025136	0.20	9.81E-07	GPR173	-53615	FAM156B	-38938
chr10	95255914	95256224	0.32	1.02E-06	CEP55	-300		
chr6	111804323	111804779	0.31	1.03E-06	REV3L	-119		
chr5	1313088	1313398	0.34	1.12E-06	TERT	-18081	CLPTM1L	31759
chr9	6757103	6757384	0.32	1.23E-06	KDM4C	-397		
chr16	29972998	29973319	0.18	1.26E-06	TMEM219	-192		
chr3	105975433	105975743	0.38	1.30E-06	CBLB	-387701		
chrX	47420671	47420999	0.19	1.40E-06	ARAF	257		
chr10	27530621	27530931	0.42	1.43E-06	ACBD5	-968		
chr1	32757334	32757619	0.15	1.44E-06	HDAC1	-231		
chr17	77774363	77774673	0.37	1.49E-06	CBX8	-3628		
chr19	35739208	35739934	0.06	1.49E-06	LSR	12		
chrX	47077788	47078220	0.24	1.49E-06	PCTK1	-111		
chr17	73043144	73043411	0.29	1.52E-06	ATP5H	-204	KCTD2	-1
chr5	40755289	40755607	0.37	1.55E-06	TTC33	624		
chr3	4544799	4545177	0.38	1.55E-06	BHLHE40	-476109	ITPR1	9954

chr17	62963768	62964078	0.29	1.58E-06	LRRC37A3	-48935	GNA13	88997
chr19	40790908	40791261	0.31	1.63E-06	AKT2	180		
chr1	24648820	24649130	0.41	1.68E-06	NIPAL3	-93270	GRHL3	3094
chr5	137088520	137088830	0.31	1.68E-06	KLHL3	-16896	HNRNPA0	1364
chr7	5436160	5436509	0.27	1.68E-06	TNRC18	26842	SLC29A4	113774
chr7	10979778	10980088	0.36	1.72E-06	NDUFA4	-120		
chr3	55760355	55760672	0.39	1.78E-06	WNT5A	-239183	ERC2	741877
chr11	72525088	72525447	0.19	1.79E-06	ATG16L2	-183		
chr2	27603503	27603757	0.42	1.82E-06	ZNF513	-37		
chr14	39736295	39736619	0.21	1.87E-06	CTAGE5	129		
chr2	166532001	166532350	0.34	1.87E-06	CSRNP3	103286	GALNT3	118627
chr15	102520957	102521270	0.23	1.95E-06	WASH2P	8010		
chr2	113299430	113299787	0.21	1.95E-06	POLR1B	117		
chr6	112408759	112409069	0.39	1.95E-06	TUBE1	-163		
chr3	133292566	133292876	0.32	2.02E-06	CDV3	287		
chr1	208037246	208037556	0.47	2.03E-06	CD34	47282	CD46	111999
chr3	152005787	152006038	0.24	2.13E-06	MBNL1	-11281	SUCNR1	414476
chr1	39456727	39456994	0.13	2.14E-06	AKIRIN1	-55		
chr1	35667672	35668010	0.38	2.14E-06	ZMYM4	-66727	SFPQ	-9098
chr6	41990137	41990562	0.30	2.15E-06	CCND3	-80798	TAF8	-27901
chr2	46925604	46925927	0.26	2.19E-06	SOCS5	-333		
chr6	43214327	43214637	0.37	2.36E-06	SLC22A7	-51516	TTBK1	3260
chr12	1703905	1704236	0.23	2.40E-06	FBXL14	-740		
chr3	32726271	32726557	0.20	2.42E-06	CNOT10	-284		
chr22	50690637	50691039	0.31	2.43E-06	HDAC10	-1004		
chr16	81897414	81897848	0.36	2.43E-06	PLCG2	84701	SDR42E1	147462
chr16	23875022	23875474	0.35	2.47E-06	CACNG3	-391628	PRKCB	27948
chr16	85148564	85148800	0.40	2.48E-06	KIAA0182	-498242	KIAA0513	87272
chr6	36971819	36972193	0.36	2.66E-06	FGD2	-1417		
chr8	81050846	81051156	0.44	2.69E-06	ZBTB10	-347447	TPD52	-57991
chr7	43688046	43688282	0.38	2.92E-06	STK17A	65472	C7orf44	80919
chr8	99058274	99058596	0.32	2.94E-06	RPL30	-662		
chr3	33700526	33700930	0.12	2.95E-06	UBP1	-218831	CLASP2	59120
chr4	87927981	87928259	0.22	3.01E-06	AFF1	-33		
chrX	128977703	128978013	0.34	3.02E-06	ZDHHC9	-390		
chr8	49860126	49860436	0.25	3.08E-06	SNTG1	-964316	SNAI2	-26293
chr15	94774285	94774592	0.40	3.12E-06	MCTP2	-66991		
chr2	131863717	131864027	0.42	3.15E-06	POTEE	-112052	PLEKHB2	1452

chr7	2595109	2595556	0.13	3.28E-06	IQCE	-3299	C7orf27	-187
chr12	124272594	124272838	0.20	3.35E-06	ZNF664	-184954	DNAH10	25674
chr19	10339134	10339444	0.26	3.35E-06	DNMT1	-33534	S1PR2	2659
chr5	612106	612416	0.32	3.41E-06	CEP72	-144		
chr1	228651417	228651838	0.38	3.41E-06	RHOU	-219241	HIST3H2BB	5820
chr10	85974355	85974665	0.41	3.50E-06	LRIT2	10774	PCDH21	19993
chr22	26702618	26702935	0.41	3.50E-06	ASPHD2	-122503	SEZ6L	137297
chr16	22368857	22369103	0.30	3.53E-06	CDR2	16958	POLR3E	60239
chr4	104119522	104119838	0.20	3.57E-06	CENPE	-114		
chr16	11903923	11904289	0.18	3.64E-06	ZC3H7A	-27698	RSL1D1	41336
chr10	126228393	126228942	0.40	3.74E-06	LHPP	78256	FAM53B	204262
chr1	46016412	46016727	0.36	3.75E-06	AKR1A1	72		
chr18	46472798	46473108	0.31	3.76E-06	SMAD7	4128	KIAA0427	407526
chr11	47291467	47291746	0.30	3.93E-06	MADD	408		
chr5	1525163	1525473	0.42	4.41E-06	LPCAT1	-1242		
chr3	194406307	194406678	0.14	4.46E-06	LSG1	-13287	C3orf21	585402
chr19	49653516	49653954	0.16	4.49E-06	HRC	4946	PPFIA3	31072
chr16	85855209	85855596	0.38	4.61E-06	IRF8	-77371	COX4I1	22207
chr19	5864065	5864375	0.37	4.67E-06	FUT3	-12735	FUT5	6414
chr1	32404024	32404274	0.21	4.74E-06	PTP4A2	-161		
chr19	35738545	35739067	0.36	4.86E-06	LSR	-753		
chr21	34426895	34427139	0.30	4.92E-06	OLIG1	-15433	OLIG2	28778
chr11	85913828	85914198	0.43	4.93E-06	PICALM	-133905	EED	-41802
chr9	21802429	21802827	0.33	5.03E-06	MTAP	-7		
chr16	11633548	11633859	0.38	5.03E-06	LITAF	47102	C16orf75	194393
chr6	44215410	44215729	0.27	5.11E-06	HSP90AB1	721		
chr12	113623261	113623564	0.23	5.14E-06	DDX54	-129		
chrX	118708320	118708572	0.25	5.14E-06	UBE2A	-53		
chr12	56727550	56727855	0.31	5.14E-06	IL23A	-4960	PAN2	-174
chr2	86790038	86790376	0.23	5.14E-06	RNF103	60771	KDM3A	121623
chr10	135524491	135524801	0.29	5.26E-06	LOC653545	40968		
chr3	67096528	67096863	0.37	5.31E-06	KBTBD8	47969	SUCLG2	608342
chr11	568595	568844	0.22	5.44E-06	PHRF1	-7763	RASSF7	7270
chr11	3876027	3876263	0.43	5.66E-06	STIM1	-788		
chr13	43701994	43702242	0.23	5.82E-06	DNAJC15	104756	ENOX1	501495
chr20	36661581	36661903	0.29	5.84E-06	KIAA0406	91		
chr19	48972862	48973172	0.35	5.85E-06	CYTH2	552		
chr10	61645424	61645734	0.42	5.92E-06	SLC16A9	-175930	CCDC6	21239

chr9	112729287	112729747	0.43	6.10E-06	PALM2-AKAP2	186940	TXN	289261
chr1	85459809	85460119	0.33	6.13E-06	LPAR3	-101068	MCOLN2	2832
chr7	154794820	154795084	0.19	6.33E-06	PAXIP1	-270		
chr16	31119656	31120079	0.38	6.37E-06	BCKDK	206		
chr20	814201	814527	0.28	6.37E-06	FAM110A	8		
chr4	36252	36638	0.33	6.60E-06	ZNF718	-16782		
chr1	183442124	183442537	0.44	6.65E-06	SMG7	697		
chr2	12856699	12857009	0.35	6.71E-06	TRIB2	-144		
chr4	42629906	42630216	0.28	6.76E-06	ATP8A1	29061	SHISA3	230205
chr22	42765333	42765643	0.34	7.04E-06	TCF20	-154043	NFAM1	62913
chr15	65020385	65020696	0.45	7.30E-06	OAZ2	-25079	RBPMS2	47229
chr19	898707	899017	0.36	7.41E-06	MED16	-5644	C19orf22	14363
chr19	1650587	1650897	0.32	7.45E-06	TCF3	-456		
chr7	50161003	50161260	0.25	7.50E-06	IKZF1	-183246	ZPBP	-28272
chr12	50794224	50794599	0.12	7.71E-06	LARP4	-238		
chr17	8093329	8093749	0.32	7.91E-06	C17orf59	25		
chrX	47050006	47050289	0.18	7.98E-06	UBA1	-51		
chr20	57463993	57464307	0.39	8.08E-06	TH1L	-92161	GNAS	36114
chr1	6259746	6260042	0.24	8.13E-06	RPL22	-215		
chr8	54755717	54756085	0.42	8.17E-06	ATP6V1H	-51		
chr2	134877034	134877424	0.47	8.30E-06	NCKAP5	-551198	MGAT5	-134601
chr6	42018226	42018551	0.29	8.34E-06	TAF8	138		
chrX	19002911	19003250	0.42	8.67E-06	PHKA2	-601		
chr19	23945424	23945792	0.30	8.72E-06	ZNF681	-3915	RPSA	-208
chr7	87849144	87849676	0.18	8.76E-06	SRI	-17		
chr8	62661038	62661348	0.37	8.95E-06	NKAIN3	-500308	ASPH	-33994
chr16	81861600	81861923	0.38	9.49E-06	PLCG2	48832	SDR42E1	183331
chr20	3335141	3335415	0.29	9.65E-06	SLC4A11	-116905	ATRN	-116387
chr8	17941616	17941913	0.20	1.01E-05	ASAH1	114		
chr9	131903915	131904225	0.44	1.01E-05	METTL11A	-484365	PPP2R4	30826
chr11	44626928	44627238	0.30	1.05E-05	TSPAN18	-300875	CD82	39942
chr19	2679482	2679792	0.24	1.05E-05	GNG7	23109	GADD45B	203502
chr8	142106635	142106987	0.39	1.05E-05	PTK2	-95479	SLC45A4	131862
chr16	15068552	15068873	0.35	1.06E-05	PDXDC1	-120		
chr19	38826993	38827433	0.36	1.06E-05	CATSPERG	770		
chr7	16685467	16685777	0.10	1.06E-05	ANKMY2	-224	BZW2	-137
chr17	65990614	65990942	0.29	1.08E-05	C17orf58	-1013		
chr11	118796569	118796855	0.15	1.09E-05	UPK2	-30314	BCL9L	-15099

chr2	173145654	173145967	0.41	1.09E-05	DLX2	-178333	ITGA6	-146503
chr12	106696251	106696579	0.20	1.13E-05	POLR3B	-55021	CKAP4	-54702
chr1	31223742	31224069	0.40	1.15E-05	MATN1	-27474	LAPTM5	6777
chr14	21571909	21572250	0.18	1.17E-05	ZNF219	-4907		
chr6	114292271	114292720	0.17	1.18E-05	HDAC2	-142		
chr15	40074952	40075267	0.39	1.25E-05	FSIP1	-71		
chr7	101513004	101513525	0.37	1.32E-05	SH2B2	-415140	CUX1	52383
chr17	71228306	71228598	0.37	1.37E-05	C17orf80	-324		
chr7	130754046	130754356	0.24	1.37E-05	KLF14	-335341	MKLN1	-258394
chr2	230038186	230038496	0.17	1.38E-05	SPHKAP	-991980	PID1	97716
chr2	60779702	60780090	0.44	1.40E-05	BCL11A	737		
chr12	113377709	113378067	0.45	1.42E-05	OAS2	-38386	OAS3	1639
chr11	11283841	11284351	0.48	1.44E-05	ZBED5	-404476	CSNK2A1	90808
chr14	74208607	74208917	0.31	1.44E-05	PNMA1	-27634	C14orf43	45134
chr2	220408488	220408767	0.35	1.46E-05	CHPF	-141	TMEM198	-117
chr22	38710971	38711219	0.35	1.46E-05	TMEM184B	-42079	CSNK1E	2994
chr4	13486004	13486322	0.19	1.49E-05	RAB28	-174		
chr3	12791008	12791318	0.48	1.49E-05	RAF1	-85463	TMEM40	9645
chr6	36953935	36954215	0.26	1.52E-05	MTCH1	-126		
chr2	153574818	153575123	0.26	1.53E-05	PRPF40A	-996	ARL6IP6	548
chr16	25061945	25062255	0.49	1.57E-05	LCMT1	-60947	ARHGAP17	-35425
chr8	110551522	110551859	0.42	1.58E-05	EBAG9	-238		
chr14	31494508	31494818	0.42	1.58E-05	AP4S1	-20	STRN3	944
chr17	2614009	2614319	0.38	1.65E-05	KIAA0664	763		
chr10	23055140	23055514	0.42	1.65E-05	ARMC3	-161627	PIP4K2A	-51824
chr1	145122825	145123061	0.44	1.68E-05	NOTCH2NL	-86168	SEC22B	26536
chr16	10037187	10037456	0.47	1.68E-05	USP7	-979981	GRIN2A	238941
chr2	136889506	136889770	0.43	1.70E-05	THSD7B	-858824	CXCR4	-13913
chr22	24530155	24530465	0.32	1.73E-05	SUSD2	-47134	CABIN1	122545
chr10	43913604	43914109	0.33	1.74E-05	ZNF487	-18717	HNRNPF	-10558
chr15	72978202	72978555	0.38	1.77E-05	BBS4	-147		
chr4	186347079	186347430	0.14	1.83E-05	UFSP2	-116		
chr6	32590290	32590581	0.33	1.87E-05	HLA-DRB5	-92430	HLA-DQA1	-14747
chr7	97601394	97601773	0.36	1.91E-05	ASNS	-99730	OCM2	17832
chr2	239197484	239197813	0.25	1.92E-05	PER2	-442		
chr10	35379416	35379689	0.30	1.97E-05	CUL2	-259		
chr20	48247016	48247458	0.43	1.97E-05	PTGIS	-62530	B4GALT5	83184
chr7	127743849	127744159	0.33	1.99E-05	LEP	-137327	LRRC4	-73002

chrX	153640911	153641221	0.25	2.07E-05	DNASE1L1	-639		
chr1	156119412	156119687	0.39	2.09E-05	SEMA4A	-3838		
chr10	98031096	98031428	0.44	2.10E-05	BLNK	71		
chr3	113462129	113462439	0.31	2.12E-05	ATP6V1A	-3624	NAT13	2812
chr1	236447707	236448047	0.55	2.13E-05	ERO1LB	-2538		
chr1	11072402	11072760	0.14	2.17E-05	TARDBP	-98		
chr1	26197537	26197848	0.22	2.17E-05	PAQR7	51		
chr15	53022397	53022688	0.33	2.20E-05	ARPP19	-161330	ONECUT1	59666
chr16	20911795	20912154	0.20	2.20E-05	DCUN1D3	-414	LYRM1	-100
chr2	171738402	171738712	0.38	2.20E-05	GORASP2	-47154	GAD1	65357
chr18	3261823	3262113	0.27	2.21E-05	MYL12B	-143		
chr16	47526460	47526779	0.40	2.30E-05	PHKB	31410	ABCC12	654061
chr11	129817398	129817708	0.43	2.31E-05	NFRKB	-54649	PRDM10	55177
chr7	151329365	151329639	0.33	2.33E-05	RHEB	-112492	PRKAG2	244814
chr6	137416771	137417007	0.51	2.35E-05	IL20RA	-50591	IL22RA2	77896
chr8	97273839	97274142	0.17	2.38E-05	MTERFD1	-195	PTDSS1	-176
chr10	81947266	81947680	0.37	2.39E-05	ANXA11	17855	PLAC9	55215
chr12	27031733	27032043	0.43	2.39E-05	ITPR2	-45757	C12orf11	59366
chr18	3448973	3449287	0.44	2.45E-05	TGIF1	-2461		
chr7	21985517	21985842	0.17	2.53E-05	CDCA7L	-138		
chr14	92587983	92588367	0.18	2.60E-05	CPSF2	-154	NDUFB1	-22
chr17	7748108	7748520	0.41	2.67E-05	TMEM88	-10070	KDM6B	5079
chr17	36956038	36956427	0.16	2.71E-05	PIP4K2B	-75		
chr9	130953864	130954202	0.38	2.73E-05	CIZ1	-165		
chr21	45144666	45144977	0.44	2.73E-05	PDXK	5844	CSTB	51437
chr20	32581424	32581698	0.27	2.80E-05	RALY	-171		
chr7	50352249	50352609	0.38	2.84E-05	IKZF1	8051	FIGNL1	165659
chr17	66221284	66221560	0.35	2.90E-05	AMZ2	-22723	KPNA2	189574
chr12	104850510	104850754	0.50	2.91E-05	CHST11	-146		
chr1	147142405	147142758	0.39	2.94E-05	ACP6	52		
chr19	4763126	4763370	0.42	3.00E-05	DPP9	-39393	FEM1A	-28480
chr7	99724730	99725047	0.16	3.02E-05	MBLAC1	569		
chr5	1255146	1255456	0.30	3.05E-05	SLC6A18	29831	TERT	39861
chr19	12900780	12901070	0.21	3.11E-05	JUNB	-1385		
chr5	81147337	81148024	0.30	3.11E-05	ATG10	-120163	SSBP2	-100609
chr19	48281642	48281960	0.22	3.12E-05	SEPW1	-41		
chr12	49947348	49947658	0.41	3.17E-05	MCRS1	12719	KCNH3	14563
chr6	114179941	114180190	0.39	3.29E-05	MARCKS	1539	HDAC2	112288

chr8	27951245	27951481	0.32	3.38E-05	ELP3	779		
chr10	112327085	112327481	0.14	3.43E-05	SMC3	-166		
chr7	43909016	43909275	0.19	3.45E-05	MRPS24	-1		
chr7	28609885	28610162	0.43	3.45E-05	CREB5	157880	TRIL	388005
chr12	113500564	113500874	0.38	3.46E-05	DTX1	5057	RASAL1	73302
chr14	57046213	57046570	0.20	3.47E-05	C14orf101	-119		
chr3	44087398	44087732	0.35	3.47E-05	C3orf23	-292379	ABHD5	355190
chr22	27905799	27906111	0.30	3.48E-05	PITPNB	409300	CRYBA4	888027
chr1	154934231	154934676	0.36	3.49E-05	PYGO2	-196		
chr22	40574546	40574856	0.50	3.49E-05	TNRC6B	772		
chr6	109703686	109704043	0.13	3.56E-05	CD164	-103		
chr12	119984216	119984526	0.32	3.60E-05	LOC387890	-46893	HSPB8	367776
chr1	54291068	54291378	0.36	3.61E-05	GLIS1	-91346	TMEM48	12952
chr1	203267878	203268188	0.42	3.61E-05	CHIT1	-69173	BTG2	-6631
chr1	177091996	177092313	0.39	3.69E-05	ASTN1	41869	PAPPA2	659848
chr10	104040048	104040358	0.34	3.78E-05	NFKB2	-114136	GBF1	34894
chr19	5865212	5865456	0.44	3.79E-05	FUT3	-13849	FUT5	5300
chr3	9821445	9821755	0.38	3.88E-05	CAMK1	-9939	TADA3	12820
chr20	60718569	60718805	0.39	3.97E-05	PSMA7	-213	SS18L1	-135
chr6	31550115	31550425	0.43	4.16E-05	LST1	-3701	LTB	-68
chr16	21530331	21530641	0.41	4.18E-05	DKFZp547E087	-31496	SLC7A5P1	1279
chr5	173070952	173071262	0.52	4.34E-05	CPEB4	-244224	BOD1	-27441
chr14	102834086	102834396	0.40	4.40E-05	CINP	-4988		
chr7	139025874	139026209	0.23	4.49E-05	LUC7L2	-18592	TTC26	207552
chr19	10205073	10205383	0.32	4.52E-05	ANGPTL6	8197	RDH8	81303
chr22	39052331	39052690	0.19	4.69E-05	CBY1	-147		
chr16	16001753	16002209	0.48	4.70E-05	ABCC1	-41453	C16orf63	-19534
chr2	149893086	149893535	0.45	4.94E-05	LYPD6B	-1670		
chr22	37415398	37415908	0.39	4.99E-05	TST	-162	MPST	-49
chr13	76111958	76112285	0.18	5.03E-05	COMMD6	-131		
chr2	73441071	73441381	0.46	5.03E-05	SMYD5	-140		
chr9	34637697	34638070	0.25	5.04E-05	SIGMAR1	-116		
chr10	13850975	13851215	0.31	5.04E-05	PRPF18	222156	FRMD4A	521771
chr1	37420571	37420925	0.30	5.06E-05	CSF3R	-472239	GRIK3	79096
chr19	50167510	50167839	0.43	5.27E-05	BCL2L12	-724		
chr5	149790023	149790291	0.45	5.27E-05	CD74	2175	TCOF1	52955
chr6	30524212	30524478	0.26	5.34E-05	PRR3	-411	GNL1	663
chr8	50097413	50097723	0.25	5.35E-05	SNTG1	-727029	SNAI2	-263580

chr19	10443501	10444021	0.16	5.36E-05	RAVER1	553		
chr3	187718164	187718648	0.39	5.39E-05	BCL6	-254931	LPP	-212315
chr8	22926552	22926788	0.52	5.41E-05	TNFRSF10B	30		
chr19	36134369	36134716	0.21	5.42E-05	COX6B1	-4612		
chr19	18315061	18315509	0.30	5.49E-05	RAB3A	-411		
chr10	76970271	76970581	0.42	5.80E-05	VDAC2	-137		
chr8	144679584	144679874	0.22	5.93E-05	TIGD5	-492	EEF1D	116
chr6	10533990	10534369	0.46	6.01E-05	TFAP2A	-118710	GCNT2	-51813
chr3	50358887	50359228	0.21	6.05E-05	HYAL1	-18054	HYAL2	1223
chrX	118699223	118699594	0.38	6.07E-05	CXorf56	-45		
chr6	32909318	32909626	0.46	6.16E-05	HLA-DMB	-655		
chr3	191900884	191901274	0.38	6.23E-05	FGF12	225759	PYDC2	722127
chr16	75143644	75143884	0.34	6.33E-05	LDHD	6901	ZNRF1	110849
chr1	151431839	151432401	0.46	6.38E-05	POGZ	-188		
chr19	12912566	12912954	0.15	6.50E-05	RNASEH2A	-4668	PRDX2	-66
chr14	31926004	31926314	0.49	6.56E-05	C14orf126	521		
chr9	91537060	91537441	0.38	6.60E-05	C9orf47	-68527	SPIN1	533954
chr11	842550	842867	0.26	6.84E-05	POLR2L	-180	TSPAN4	-115
chr16	30363153	30363463	0.28	7.13E-05	CD2BP2	3374	SULT1A3	152772
chr6	35888700	35889160	0.34	7.25E-05	SRPK1	26		
chr8	41502523	41502833	0.28	7.35E-05	NKX6-3	2197	AGPAT6	66971
chr2	120070548	120070801	0.27	7.40E-05	DBI	-53829	STEAP3	89291
chr10	99894165	99894401	0.46	7.41E-05	CRTAC1	-103698	LOXL4	133724
chr16	2563659	2564054	0.28	7.46E-05	ATP6V0C	-96		
chr4	42659228	42659595	0.16	7.63E-05	ATP8A1	-290		
chr14	107259167	107259477	0.39	7.73E-05	ADAM6	-820964	IGHV7-81	23961
chr6	74171241	74171624	0.46	7.74E-05	MTO1	-21		
chr14	39901338	39901708	0.18	7.89E-05	CTAGE5	165195		
chr9	127624180	127624450	0.23	7.89E-05	RPL35	-75		
chr8	86102109	86102563	0.20	7.93E-05	CA13	-55380	E2F5	12717
chr12	46797176	46797486	0.38	8.04E-05	SLC38A2	-30686	SLC38A4	422449
chr13	49945818	49946128	0.47	8.07E-05	CAB39L	29762	CDADC1	123870
chr19	49621984	49622297	0.33	8.34E-05	PPFIA3	-522		
chr12	32260875	32261185	0.43	8.57E-05	BICD1	845		
chr3	129813036	129813415	0.52	8.59E-05	COL29A1	-251133	ALG1L2	12552
chr14	23000529	23000839	0.53	8.60E-05	DAD1	57459	TCRDV2	72595
chr14	105124220	105124530	0.49	8.91E-05	TMEM179	-53278	INF2	-31568
chr8	131675372	131675628	0.23	9.12E-05	ASAP1	-261284	ADCY8	377335

chr1	174231978	174232329	0.47	9.67E-05	GPR52	-185058	RABGAP1L	103520
chr19	7410517	7410991	0.37	9.96E-05	INSR	-116743	ARHGEF18	-93820
chr10	127511808	127512165	0.16	1.02E-04	UROS	-150	BCCIP	-117
chr1	1624096	1624743	0.09	1.02E-04	LOC728661	-177		
chr1	175194144	175194613	0.40	1.02E-04	TNN	157385	TNR	518373
chr8	27326027	27326337	0.44	1.03E-04	CHRNA2	10631	PTK2B	143128
chr7	138791783	138792035	0.45	1.04E-04	KIAA1549	-187339	ZC3HAV1	2556
chr1	241933541	241933777	0.54	1.05E-04	OPN3	-129958	EXO1	-77876
chr3	160472883	160473269	0.17	1.08E-04	PPM1L	-920		
chr22	19466686	19466977	0.25	1.08E-04	CDC45L	-584	UFD1L	-94
chr19	18334779	18335015	0.23	1.09E-04	RAB3A	-20023	PDE4C	24113
chr12	53594612	53594922	0.40	1.09E-04	ITGB7	6233	ZNF740	20232
chr6	57036831	57037141	0.46	1.09E-04	BAG2	-118		
chr8	59570989	59571299	0.39	1.09E-04	NSMAF	822		
chr12	6536092	6536438	0.31	1.12E-04	CD27	-17786	LTBR	42908
chr19	9945335	9945645	0.46	1.13E-04	PIN1	-509		
chr4	48917609	48917913	0.41	1.14E-04	CWH43	-70504	OCIAD2	-8946
chr8	43018259	43018588	0.39	1.15E-04	HGSNAT	22832		
chr7	107204135	107204444	0.44	1.17E-04	DUS4L	-142	COG5	669
chr9	137000827	137001227	0.43	1.18E-04	WDR5	-183		
chr17	76730880	76731387	0.47	1.21E-04	DNAH17	-163730	CYTH1	47242
chr5	88179272	88179582	0.44	1.22E-04	MEF2C	-125		
chr8	48873182	48873505	0.12	1.23E-04	PRKDC	-601	MCM4	-150
chr5	140887063	140887333	0.37	1.25E-04	PCDHGC5	18390	DIAPH1	111424
chr4	37070	37357	0.25	1.26E-04	ZNF718	-16013		
chr22	24820798	24821141	0.40	1.28E-04	ADORA2A	-2560		
chr8	61824712	61824991	0.34	1.30E-04	RLBP1L1	-375673	CHD7	233513
chr6	106958372	106958757	0.42	1.31E-04	AIM1	-740		
chr19	46850594	46850904	0.53	1.31E-04	PPP5C	455		
chr6	31795150	31795466	0.19	1.32E-04	HSPA1B	-204		
chr5	10250005	10250449	0.26	1.32E-04	FAM173B	-213	CCT5	-55
chr5	179921127	179921406	0.21	1.36E-04	CNOT6	-150		
chr8	22832849	22833233	0.42	1.40E-04	PEBP4	-47620	RHOBTB2	-20320
chr16	70285727	70286133	0.38	1.42E-04	EXOSC6	-97		
chr19	36391492	36391802	0.49	1.46E-04	HCST	-1735	NFKBID	-95
chr12	25104743	25105057	0.63	1.47E-04	BCAT1	-2592		
chr7	158312139	158312400	0.44	1.52E-04	PTPRN2	68212		
chr1	98386892	98387208	0.32	1.56E-04	DPYD	-435		

chr9	117150158	117150511	0.51	1.57E-04	AKNA	6350	ORM1	65032
chr6	42858495	42858794	0.21	1.57E-04	PTCRA	-25082	RPL7L1	10974
chr1	28501826	28502202	0.38	1.63E-04	EYA3	-86883	PTAFR	1177
chr11	576121	576471	0.17	1.64E-04	PHRF1	-187		
chr19	24006698	24007023	0.43	1.66E-04	ZNF254	-263115	RPSA	61045
chr7	1199745	1200127	0.35	1.67E-04	ZFAND2A	-81		
chr1	154916717	154917027	0.53	1.76E-04	PMVK	-7388	PBXIP1	11695
chr6	27791550	27791862	0.52	1.76E-04	HIST1H4J	-197		
chr14	93579709	93580019	0.51	1.80E-04	ITPK1	2399	CHGA	190419
chrX	153686359	153686693	0.41	1.86E-04	PLXNA3	-97		
chr6	31869850	31870434	0.41	1.86E-04	EHMT2	-4678	ZBTB12	-373
chr22	18484273	18484602	0.30	1.88E-04	BID	-227659	MICAL3	22887
chr8	8800161	8800471	0.48	1.88E-04	ERI1	-59998	MFHAS1	-49185
chr6	151006999	151007347	0.55	1.92E-04	MTHFD1L	-179518	PLEKHG1	86174
chr1	91487707	91487943	0.50	1.92E-04	ZNF644	-154		
chr18	9136399	9136716	0.25	1.94E-04	ANKRD12	-200		
chr12	70300111	70300549	0.46	1.94E-04	C12orf28	-20107	RAB3IP	167699
chr17	40518709	40519019	0.48	1.97E-04	STAT3	21649	STAT5A	79299
chr17	49008318	49008628	0.49	1.98E-04	TOB1	-67060	SPAG9	189753
chr19	17865659	17866151	0.42	2.01E-04	B3GNT3	-40014	MAP1S	35602
chr6	42237311	42237655	0.39	2.01E-04	MRPS10	-51850	TRERF1	182300
chr11	111284975	111285350	0.47	2.19E-04	POU2AF1	-35006	BTG4	97901
chr19	7124857	7125167	0.28	2.22E-04	ZNF557	55541	INSR	168999
chr14	75348235	75348695	0.21	2.25E-04	DLST	-129		
chr12	120739958	120740374	0.26	2.27E-04	SIRT4	3		
chr1	155658553	155658903	0.26	2.28E-04	DAP3	-146	YY1AP1	63
chr5	132202098	132202385	0.29	2.29E-04	GDF9	-1765	UQCRQ	-77
chr19	7413586	7414063	0.33	2.32E-04	INSR	-119814	ARHGEF18	-90749
chr1	42512475	42512785	0.30	2.35E-04	HIVEP3	-128134	GUCA2B	-106462
chr13	51483841	51484151	0.48	2.39E-04	RNASEH2B	104		
chr15	89786961	89787289	0.28	2.40E-04	FANCI	-69		
chr18	32738969	32739279	0.55	2.41E-04	ZNF397	-81874	MAPRE2	180916
chr7	8230059	8230354	0.54	2.48E-04	ICA1	71475	GLCCI1	221784
chr8	74903852	74904275	0.56	2.60E-04	LY96	477		
chr8	125616841	125617151	0.43	2.65E-04	NDUFB9	65653	MTSS1	123734
chr11	126334849	126335159	0.49	2.68E-04	ST3GAL4	108908	KIRREL3	535762
chr10	75385616	75385926	0.44	2.80E-04	USP54	-50338	MYOZ1	15744
chr17	6915557	6915975	0.22	2.80E-04	C17orf49	-1698	RNASEK	-32

chr1	54335163	54335473	0.50	2.83E-04	TMEM48	-31143	YIPF1	20135
chr3	195636236	195636556	0.33	2.86E-04	TNK2	-13964	SDHALP1	80754
chr21	44394069	44394412	0.20	2.87E-04	PKNOX1	-402		
chr10	15001045	15001355	0.41	2.90E-04	MEIG1	-238		
chr7	5270753	5271093	0.36	2.97E-04	SLC29A4	-51638	WIPI2	41088
chr2	227770469	227770786	0.45	3.00E-04	RHBDD1	69855	COL4A4	258647
chr5	114880833	114881143	0.49	3.04E-04	FEM1C	-397		
chr22	21368527	21368837	0.39	3.07E-04	P2RX6	-782		
chr17	80550601	80551323	0.04	3.09E-04	FOXK2	73368	RAB40B	105636
chr6	33289802	33290235	0.56	3.11E-04	ZBTB22	-4300	DAXX	774
chr6	31670603	31671335	0.20	3.19E-04	LY6G6F	-3715	BAT5	119
chr1	213224280	213224663	0.53	3.28E-04	RPS6KC1	-116		
chr9	140162437	140162747	0.42	3.28E-04	C9orf167	-9688	COBRA1	12833
chr6	107939876	107940233	0.44	3.32E-04	SOBP	128738	SCML4	205466
chr22	30988121	30988403	0.29	3.34E-04	PES1	-368		
chr6	116593491	116593801	0.42	3.35E-04	TSPYL4	-18385	TSPYL1	7634
chr17	7258318	7258562	0.44	3.48E-04	TMEM95	-57		
chr8	8245770	8246084	0.42	3.49E-04	CLDN23	-313739	PRAGMIN	-6670
chr10	72335731	72336158	0.43	3.52E-04	NODAL	-134480	PRF1	26586
chr12	62653895	62654184	0.24	3.60E-04	USP15	-147		
chr19	50353849	50354492	0.22	3.60E-04	PTOV1	-245		
chr10	98038815	98039228	0.42	3.66E-04	DNTT	-25063	BLNK	-7689
chrX	134478174	134478484	0.35	3.74E-04	ZNF75D	-372	ZNF449	-367
chr12	92801803	92802113	0.42	3.78E-04	PLEKHG7	-328307	BTG1	-262285
chr19	45909587	45909954	0.19	3.84E-04	PPP1R13L	-1459	CD3EAP	304
chr1	46598338	46598901	0.21	3.88E-04	PIK3R3	-240		
chr1	21845598	21845908	0.42	3.93E-04	ALPL	9895	RAP1GAP	132595
chr4	103746719	103747298	0.48	4.08E-04	MANBA	-64858	UBE2D3	1699
chr12	53614414	53614775	0.52	4.09E-04	ITGB7	-13595	RARG	11441
chr16	81040488	81040847	0.22	4.15E-04	CENPN	565		
chr7	30066011	30066542	0.33	4.18E-04	PLEKHA8	-2011	FKBP14	-9
chr6	2886230	2886540	0.46	4.20E-04	SERPINB1	-44304	SERPINB9	17160
chr3	15248396	15248772	0.54	4.26E-04	CAPN7	851		
chr19	8275391	8275775	0.40	4.34E-04	LASS4	1366	CD320	97656
chr2	198571132	198571442	0.43	4.44E-04	MARS2	1200	BOLL	79651
chr3	9690760	9691303	0.24	4.47E-04	MTMR14	-112		
chr12	1313885	1314195	0.56	4.47E-04	ERC1	213636	FBXL14	389291
chr14	68141325	68141648	0.41	4.60E-04	VTI1B	115		

chr17	67651082	67651374	0.37	4.68E-04	KCNJ16	-420198	MAP2K6	240390
chr16	4674309	4674731	0.24	4.70E-04	MGRN1	-306		
chr22	48466650	48466964	0.41	4.70E-04	FAM19A5	-418481		
chr11	3915505	3915824	0.43	4.73E-04	RRM1	-200259	STIM1	38732
chr8	67974311	67974635	0.26	4.77E-04	CSPP1	-2130	COPS5	89
chr21	44782090	44782400	0.63	4.82E-04	SIK1	64757	CRYAA	193104
chr1	27816605	27817135	0.20	4.88E-04	WASF2	-201		
chr19	18740669	18740979	0.42	4.90E-04	KLHL26	-7014	TMEM59L	17142
chr9	35815121	35815494	0.47	4.94E-04	SPAG8	-3049	HINT2	-266
chr5	159895049	159895434	0.36	4.94E-04	PTTG1	46377	ATP10B	383977
chr14	69259857	69260178	0.49	4.97E-04	ZFP36L1	-233		
chr1	158217960	158218270	0.57	5.04E-04	CD1A	-5812	CD1D	68378
chr2	135169448	135169893	0.44	5.10E-04	MGAT5	157841	TMEM163	306900
chr1	217804387	217804736	0.25	5.26E-04	GPATCH2	-153	SPATA17	-133
chr7	101528196	101528600	0.50	5.27E-04	SH2B2	-400007	CUX1	67516
chr6	33245660	33245965	0.35	5.31E-04	B3GALT4	896		
chr12	56551941	56552295	0.39	5.32E-04	MYL6	18		
chr22	39926687	39926997	0.48	5.46E-04	RPS19BP1	2018	ATF4	10273
chr17	28258319	28258627	0.47	5.48E-04	SSH2	-1455		
chr1	151319440	151319754	0.50	5.59E-04	RFX5	172		
chr19	18100324	18100569	0.41	5.61E-04	KCNN1	38336	IL12RB1	97295
chr7	55433620	55434082	0.27	5.68E-04	LANCL2	710		
chr2	64044131	64044441	0.29	5.69E-04	UGP2	-24728	MDH1	228164
chr9	95087895	95088230	0.29	5.74E-04	NOL8	-187	CENPP	322
chr14	39644140	39644468	0.18	5.76E-04	TRAPPC6B	-4670	PNN	-83
chr12	96537277	96537587	0.50	6.10E-04	LTA4H	-108067	ELK3	-50775
chr5	173344906	173345276	0.25	6.11E-04	HMP19	-127633	CPEB4	29760
chr19	1648405	1648794	0.46	6.26E-04	UQCR	-43169	TCF3	1686
chr1	32292911	32293283	0.43	6.30E-04	SPOCD1	-11517	PTP4A2	110891
chr3	169939794	169940297	0.12	6.36E-04	PRKCI	-174		
chr1	85461137	85461373	0.62	6.40E-04	LPAR3	-102359	MCOLN2	1541
chr3	30694495	30694812	0.54	6.42E-04	TGFBR2	46660	GADL1	241499
chr8	38625808	38626131	0.48	6.42E-04	RNF5	-167195	TACC1	-18752
chr19	36050158	36050409	0.29	6.54E-04	ATP4A	4276	TMEM147	13739
chr17	54974379	54974773	0.42	6.58E-04	TRIM25	16833	DGKE	63116
chr1	62902450	62902686	0.53	6.60E-04	USP1	593		
chr20	35807413	35807758	0.32	6.67E-04	RPN2	130	C20orf132	388
chr1	231115173	231115483	0.51	6.70E-04	TTC13	-721	ARV1	505

chr3	197900190	197900444	0.35	6.76E-04	LMLN	213246		
chr5	71615975	71616404	0.30	6.76E-04	MRPS27	-106	PTCD2	-10
chrX	147691519	147691829	0.55	6.78E-04	AFF2	109535	IDS	895190
chr12	56498408	56498718	0.49	6.91E-04	PA2G4	460		
chr3	187664324	187664610	0.51	6.92E-04	LPP	-266254	BCL6	-200992
chr11	36153342	36153604	0.49	6.99E-04	PRR5L	-244062	LDLRAD3	187861
chr2	146508234	146508516	0.50	7.02E-04				
chr6	32862086	32862448	0.51	7.02E-04	PSMB9	40329	HLA-DMB	46550
chr22	51059507	51059817	0.47	7.08E-04	ARSA	6945	MAPK8IP2	18100
chr5	115177076	115177511	0.39	7.14E-04	AP3S1	-325	ATG12	205
chr19	10613376	10613640	0.24	7.16E-04	KEAP1	546		
chr2	128378417	128378727	0.29	7.26E-04	GPR17	-24867	MYO7B	85194
chr7	100202114	100202487	0.42	7.49E-04	MOSPD3	-7813	PCOLCE	2419
chr6	32915652	32915909	0.52	7.55E-04	HLA-DMB	-6964	HLA-DMA	5118
chr1	8021456	8021773	0.21	7.56E-04	PARK7	-99		
chr12	125399903	125400157	0.48	7.57E-04	UBC	-453		
chr9	140567501	140567811	0.45	7.66E-04	CACNA1B	-204585	EHMT1	54212
chrX	48900700	48901015	0.52	7.67E-04	TFE3	132		
chr7	50306442	50306752	0.54	7.74E-04	ZPBP	-173737	IKZF1	-37781
chr4	670082	670427	0.42	7.77E-04	ATP5I	-2133	MYL5	-1456
chr1	222817408	222817757	0.39	7.85E-04	MIA3	26139	AIDA	68281
chr5	140998615	140998886	0.37	7.98E-04	DIAPH1	-129		
chr5	64807986	64808296	0.48	8.04E-04	ADAMTS6	-30437	CENPK	50854
chr5	6717874	6718184	0.39	8.07E-04	ADCY2	-678314	POLS	3311
chr11	85955471	85955877	0.20	8.07E-04	EED	-141		
chr16	58451671	58452107	0.49	8.09E-04	NDRG4	-45660	GINS3	25591
chr18	29523551	29523861	0.48	8.09E-04	KIAA1012	-615		
chr4	81355351	81355661	0.25	8.10E-04	BMP3	-596613	FGF5	167764
chr11	57434878	57435256	0.26	8.16E-04	ZDHHC5	-407		
chr3	179040560	179040920	0.47	8.21E-04	ZNF639	-811		
chr5	163343297	163343607	0.64	8.34E-04	MAT2B	410867		
chr12	40618740	40619181	0.48	8.56E-04	LRRK2	148		
chr11	8935949	8936199	0.37	8.73E-04	C11orf17	3373	ASCL3	28506
chr13	45009348	45009654	0.42	8.73E-04	SERP2	61523	TSC22D1	141200
chr13	27359348	27359739	0.47	8.74E-04	GPR12	-24622	USP12	386485
chr7	98973681	98974083	0.41	8.74E-04	ARPC1B	1553	PDAP1	32410
chr10	6389270	6389580	0.48	8.79E-04	PFKFB3	144585	PRKCQ	232813
chr3	135912137	135912447	0.41	8.81E-04	MSL2	2396	PPP2R3A	227725

chr10	75403868	75404180	0.47	9.03E-04	MYOZ1	-2509		
chr11	126174157	126174467	0.51	9.12E-04	DCPS	665		
chr1	46768997	46769457	0.24	9.17E-04	UQCRH	-153		
chr20	2633017	2633313	0.31	9.18E-04	NOP56	-89		
chr18	24018891	24019368	0.54	9.20E-04	KCTD1	109370	TAF4B	212721
chr15	76136551	76136861	0.46	9.20E-04	FBXO22	-59494	UBE2Q2	1084
chr16	81554747	81555057	0.41	9.26E-04	PLCG2	-258028	CMIP	76127
chr8	52849976	52850272	0.50	9.32E-04	PCMTD1	-38389	ST18	472315
chr19	14607017	14607327	0.42	9.36E-04	GIPC1	-228		
chr16	4555425	4555831	0.59	9.38E-04	HMOX2	29287	C16orf5	33188
chr19	49141439	49141928	0.21	9.41E-04	DBP	-1045	Sec1	388
chr4	153001090	153001379	0.40	9.44E-04	PET112L	-319089	FBXW7	454950
chr3	9834386	9834786	0.20	9.66E-04	TADA3	-166		
chr12	4257582	4257921	0.57	9.80E-04	PARP11	-275144	CCND2	-125150
chr8	134512317	134512627	0.57	1.07E-03	NDRG1	-202925	ST3GAL1	71711
chr17	8079012	8079322	0.42	1.09E-03	TMEM107	547		
chr7	8007930	8008240	0.48	1.10E-03	GLCCI1	-338		
chr7	123388952	123389331	0.28	1.10E-03	WASL	-26		
chr10	17628733	17629043	0.44	1.10E-03	ST8SIA6	-132634	PTPLA	30485
chr15	67546924	67547235	0.17	1.11E-03	AAGAB	-6		
chr7	111846380	111846708	0.44	1.12E-03	ZNF277	-99	DOCK4	-82
chr2	6641974	6642284	0.48	1.12E-03	CMPK2	363807	SOX11	809330
chr10	90146932	90147216	0.41	1.13E-03	KILLIN	-523880	RNLS	196008
chr16	7403706	7403966	0.31	1.14E-03	A2BP1	21085		
chr16	13484004	13484398	0.43	1.15E-03	ERCC4	-529813	SHISA9	488724
chr2	44271368	44271678	0.40	1.15E-03	PPM1B	-124477	LRPPRC	-48379
chr10	126107372	126107796	0.36	1.15E-03	OAT	-65		
chr9	15510916	15511306	0.24	1.17E-03	PSIP1	-108		
chr12	42076803	42077113	0.50	1.18E-03	PDZRN4	245417	GLT8D3	461715
chr16	88869899	88870204	0.51	1.20E-03	CDT1	-134		
chr4	114315919	114316229	0.54	1.21E-03	ANK2	345289	CAMK2D	367009
chr2	201676069	201676611	0.27	1.22E-03	BZW1	-307		
chr15	66252968	66253278	0.48	1.23E-03	RAB11A	91327	MEGF11	292952
chr20	5751653	5751949	0.40	1.23E-03	RP5-1022P6.2	-160129	CHGB	-140173
chr20	52824360	52824691	0.30	1.26E-03	PFDN4	24		
chr17	71952261	71952518	0.39	1.26E-03	SDK2	-312163	RPL38	-247405
chr12	105352446	105352757	0.52	1.26E-03	SLC41A2	-30130	ALDH1L2	125739
chr6	31461599	31461891	0.43	1.27E-03	MICB	-4110		

chr3	11313924	11314327	0.49	1.27E-03	ATG7	116		
chr4	83933972	83934482	0.34	1.27E-03	LIN54	-2245		
chr2	20297913	20298272	0.53	1.29E-03	LAPTM4A	-46304	SDC1	126834
chr17	17653235	17653584	0.56	1.31E-03	RAI1	68623	SREBF1	86915
chr6	34759419	34759898	0.47	1.36E-03	SNRPC	34347	TAF11	96160
chrX	53449084	53449556	0.48	1.37E-03	SMC1A	298		
chr19	6737216	6737600	0.17	1.37E-03	TRIP10	-2299	GPR108	225
chrX	47221488	47221798	0.48	1.38E-03	ZNF157	-8356	USP11	129329
chr5	176433723	176434000	0.33	1.39E-03	UIMC1	-419		
chr17	72451363	72451687	0.47	1.39E-03	CD300A	-10997	GPRC5C	21689
chr19	18210238	18210582	0.57	1.40E-03	PIK3R2	-53606	MAST3	1807
chr10	122062060	122062370	0.32	1.40E-03	PPAPDC1A	-154251	SEC23IP	409992
chr3	182762338	182762660	0.54	1.41E-03	DCUN1D1	-64173	MCCC1	54866
chr16	58764978	58765292	0.48	1.42E-03	SLC38A7	-46461	GOT2	3111
chr8	120927555	120927838	0.25	1.42E-03	COL14A1	-209655	DEPDC6	41797
chr17	9479061	9479421	0.53	1.43E-03	WDR16	-703	STX8	34
chr7	87503428	87503738	0.50	1.44E-03	DBF4	-1961		
chr11	47421839	47422269	0.52	1.44E-03	SPI1	-21927	SLC39A13	-7997
chr1	226841113	226841536	0.50	1.45E-03	ITPKB	85551	C1orf95	104824
chr19	2163737	2163985	0.27	1.51E-03	DOT1L	-287		
chr19	50381161	50381397	0.59	1.51E-03	AKT1S1	-667	TBC1D17	450
chr11	82612438	82612855	0.26	1.53E-03	PRCP	-1090	C11orf82	-90
chr5	111496321	111496632	0.18	1.54E-03	TIGA1	251		
chr19	35786898	35787216	0.50	1.56E-03	CD22	-33022	MAG	4019
chr12	133396684	133397080	0.47	1.59E-03	ANKLE2	-58431	GOLGA3	8406
chr11	117049382	117049692	0.55	1.61E-03	SIDT2	-402		
chr10	104172841	104173151	0.36	1.61E-03	PSD	5905	NFKB2	18657
chr14	71122174	71122506	0.47	1.62E-03	TTC9	13836	MAP3K9	153548
chr19	7600464	7600849	0.42	1.62E-03	KIAA1543	-60131	PNPLA6	1614
chr21	19191702	19192271	0.24	1.64E-03	CHODL	-425163	BTG3	-206719
chr8	67443979	67444340	0.42	1.68E-03	MYBL1	81320	ADHFE1	99442
chr6	24910644	24911104	0.50	1.69E-03	FAM65B	321		
chr22	41777464	41777809	0.48	1.70E-03	TEF	-326		
chr7	22005539	22005933	0.51	1.74E-03	CDCA7L	-20194	RAPGEF5	390797
chr8	38243546	38244040	0.46	1.75E-03	WHSC1L1	-4003	LETM2	-227
chr6	32122434	32122744	0.54	1.79E-03	PRRT1	-2869	PPT2	1288
chr13	30948661	30949002	0.41	1.80E-03	KATNAL1	-67669	HMGB1	91249
chr19	50354740	50355050	0.38	1.81E-03	PTOV1	479		

chr6	116892286	116892670	0.50	1.86E-03	RWDD1	-105		
chr9	130538553	130538984	0.24	1.86E-03	TOR2A	-41165	SH2D3C	2279
chr16	21964296	21964776	0.23	1.89E-03	UQCRC2	-73		
chr20	44600078	44600577	0.26	1.90E-03	ZNF335	505		
chr10	103578111	103578744	0.14	1.91E-03	MGEA5	-206		
chr22	21213048	21213326	0.28	1.93E-03	PI4KA	-117	SNAP29	-105
chr4	114374362	114374672	0.47	1.94E-03	CAMK2D	308566	ANK2	403732
chr10	74091709	74092058	0.43	1.96E-03	DNAJB12	23023	DDIT4	58207
chr7	64868995	64869305	0.39	1.97E-03	VKORC1L1	-469107	ZNF92	30382
chr7	27170445	27170693	0.43	1.98E-03	HOXA4	-170		
chr11	125932702	125933240	0.26	1.99E-03	CDON	216		
chr6	6610579	6611002	0.53	2.00E-03	RREB1	-497397	LY86	21857
chr1	155532702	155533070	0.37	2.02E-03	ASH1L	-562		
chr5	137673390	137673976	0.17	2.04E-03	KDM3B	-14602	CDC25C	-6167
chr17	17140447	17141116	0.19	2.05E-03	FLCN	-280		
chr7	6487538	6487950	0.49	2.07E-03	DAGLB	-101		
chr6	150326201	150326587	0.57	2.07E-03	RAET1L	20274	ULBP1	41251
chr1	154910092	154910402	0.55	2.08E-03	PMVK	-763		
chr20	42219216	42219610	0.31	2.08E-03	IFT52	-166		
chr19	871559	871869	0.39	2.10E-03	CFD	12049	MED16	21504
chr19	12847870	12848373	0.32	2.15E-03	ASNA1	-184		
chr1	114414208	114414764	0.46	2.15E-03	PTPN22	-111		
chr2	29503360	29503658	0.51	2.15E-03	C2orf71	-206382	ALK	640923
chr6	36410527	36410834	0.32	2.18E-03	KCTD20	137		
chr7	2393704	2394496	0.10	2.21E-03	EIF3B	-374		
chr12	129306561	129306871	0.58	2.23E-03	SLC15A4	1825	TMEM132C	407425
chr11	129872182	129872804	0.18	2.24E-03	PRDM10	237		
chr2	33302920	33303230	0.55	2.24E-03	RASGRP3	-435867	LTBP1	130683
chr12	122241080	122241403	0.54	2.25E-03	SETD1B	-1388		
chr1	174245268	174245539	0.50	2.26E-03	GPR52	-171808	RABGAP1L	116770
chr17	7589286	7590038	0.15	2.28E-03	WRAP53	273		
chr4	139936578	139936924	0.30	2.28E-03	CCRN4L	-192		
chr1	151319779	151320107	0.50	2.28E-03	RFX5	-174		
chr4	57301852	57302385	0.40	2.32E-03	PPAT	-274	PAICS	-150
chr18	3247504	3247856	0.22	2.33E-03	MYL12A	152		
chr19	10305560	10305917	0.16	2.35E-03	DNMT1	16		
chr7	44086277	44086587	0.45	2.37E-03	DBNL	2193	PGAM2	18731
chr3	113933975	113934309	0.59	2.38E-03	DRD3	-36243	ZNF80	22283

chr9	130007541	130007851	0.56	2.38E-03	ANGPTL2	-122652	GARNL3	-19060
chrX	4264814	4265058	0.33	2.38E-03	PRKX	-633275		
chr7	66144021	66144331	0.49	2.40E-03	RABGEF1	-60807	KCTD7	50286
chr6	32952603	32952913	0.44	2.40E-03	BRD2	16321	HLA-DOA	24631
chr19	10522905	10523218	0.57	2.40E-03	CDC37	-8791	PDE4A	-8271
chr1	172501538	172502098	0.39	2.42E-03	C1orf9	-442		
chr14	107154857	107155207	0.46	2.46E-03	ADAM6	-716674	IGHV7-81	128251
chr1	153571333	153571704	0.37	2.48E-03	S100A2	-33213	S100A16	13995
chr2	86226323	86226633	0.53	2.52E-03	ST3GAL5	-110321	POLR1A	106800
chr5	156310274	156310784	0.57	2.52E-03	PPP1R2P3	32980	TIMD4	79737
chr3	16647465	16647775	0.51	2.54E-03	DAZL	-614		
chr8	68305090	68305400	0.58	2.56E-03	ARFGEF1	-49333	CPA6	353375
chr11	3926378	3926688	0.32	2.57E-03	RRM1	-189391	STIM1	49600
chr1	186051497	186051741	0.58	2.62E-03	PRG4	-213786	HMCN1	347936
chr3	118960284	118960594	0.51	2.63E-03	B4GALT4	-687		
chr4	140684861	140685174	0.62	2.70E-03	MGST2	98096	MAML3	390215
chr7	69161071	69161409	0.52	2.71E-03				
chr5	64064383	64064819	0.34	2.72E-03	SDCCAG10	-154	SFRS12IP1	-105
chr3	194457268	194457622	0.46	2.72E-03	LSG1	-64239	C3orf21	534450
chr12	54019853	54020506	0.23	2.77E-03	ATF7	19		
chr17	56429480	56429789	0.44	2.78E-03	SUPT4H1	-72		
chr3	12748005	12748372	0.55	2.80E-03	RAF1	-42489	TMEM40	52619
chr3	187663823	187664237	0.55	2.81E-03	LPP	-266691	BCL6	-200555
chr9	132346425	132346755	0.68	2.91E-03	METTL11A	-41845	PPP2R4	473346
chr12	110436753	110437252	0.17	2.92E-03	GIT2	-2809		
chr16	84759460	84759941	0.57	2.92E-03	CRISPLD2	-93886	USP10	26146
chr19	3500568	3500999	0.23	2.93E-03	DOHH	-163		
chr22	38967008	38967320	0.48	2.94E-03	DMC1	-975		
chr20	524456	524810	0.28	3.01E-03	CSNK2A1	-151		
chr15	89181920	89182338	0.35	3.01E-03	ISG20	90		
chr7	2354269	2354563	0.52	3.03E-03	SNX8	-317		
chr6	33290486	33290815	0.57	3.04E-03	ZBTB22	-4932	DAXX	142
chr17	49337306	49337834	0.24	3.06E-03	UTP18	-327	MBTD1	-143
chr2	233940657	233941091	0.61	3.07E-03	ATG16L1	-219343	INPP5D	15838
chr8	54934805	54935115	0.52	3.08E-03	TCEA1	48		
chr5	149790854	149791240	0.56	3.09E-03	CD74	1285	TCOF1	53845
chr2	86332977	86333351	0.27	3.14E-03	PTCD3	-141	POLR1A	114
chr16	2255222	2255822	0.35	3.16E-03	MLST8	72		

chr6	6587344	6587699	0.51	3.16E-03	LY86	-1412		
chr6	43149598	43149960	0.54	3.18E-03	CUL9	-143		
chr6	3356509	3356819	0.58	3.19E-03	TUBB2B	-128696	SLC22A23	100129
chr11	9623330	9623714	0.57	3.19E-03	SWAP70	-62106	WEE1	28294
chr19	50316793	50317153	0.38	3.20E-03	MED25	-4573	FUZ	-505
chr6	42016177	42016615	0.49	3.21E-03	TAF8	-1855		
chr19	42806521	42807232	0.17	3.27E-03	PAFAH1B3	75		
chr5	157825704	157826014	0.36	3.34E-03	CLINT1	-539691	EBF1	700929
chr1	2574520	2574844	0.60	3.37E-03	ACTRT2	-363364	MMEL1	-10201
chr10	75910523	75910833	0.49	3.46E-03	ADK	-287	AP3M1	148
chr18	47883606	47883916	0.51	3.48E-03	CXXC1	-69069	SKA1	-17631
chr4	77870704	77870940	0.49	3.48E-03	SEPT11	-73		
chr9	140470522	140470859	0.62	3.51E-03	ZMYND19	14246	MRPL41	24382
chr18	74827500	74828138	0.38	3.52E-03	MBP	16955	ZNF236	291703
chr6	57086897	57087207	0.65	3.56E-03	RAB23	26		
chr2	97589986	97590296	0.51	3.56E-03	SEMA4C	-54406	FAHD2B	170441
chr10	49876138	49876528	0.50	3.59E-03	ARHGAP22	-63195	WDFY4	-17185
chr6	139696997	139697355	0.55	3.60E-03	CITED2	-1391		
chr6	31620336	31620742	0.45	3.67E-03	APOM	-3132	BAT3	-369
chr15	38364647	38364957	0.56	3.67E-03	SPRED1	-180250	TMCO5A	137344
chr14	36325215	36325612	0.61	3.72E-03	BRMS1L	29817	MBIP	464468
chr3	148961248	148961671	0.56	3.72E-03	CP	-21628	TM4SF18	89959
chr10	11706139	11706439	0.59	3.74E-03	USP6NL	-132015	ECHDC3	-78067
chr17	64563352	64563670	0.51	3.74E-03	CACNG5	-309837	PRKCA	264585
chr21	46530138	46530448	0.55	3.75E-03	ADARB1	35800	POFUT2	177518
chr7	75544221	75544524	0.17	3.76E-03	POR	-47		
chr4	31292501	31292832	0.52	3.76E-03	PCDH7	570630		
chr13	114100197	114100664	0.40	3.81E-03	GRTP1	-81968	ADPRHL1	7408
chr12	68382963	68383284	0.51	3.86E-03	IFNG	170397	DYRK2	340612
chr5	79486721	79487075	0.49	3.88E-03	SERINC5	64972	THBS4	155907
chr12	67295014	67295324	0.50	3.90E-03	CAND1	-367892	GRIP1	-222244
chr9	71255197	71255608	0.49	3.90E-03	C9orf71	-99620	PIP5K1B	-65213
chr18	20808228	20808613	0.53	3.91E-03	CABLES1	92694	C18orf45	209504
chr10	25304961	25305271	0.65	4.04E-03	THNSL1	-392	ENKUR	-86
chr9	129483830	129484078	0.46	4.05E-03	ZBTB43	-83331	LMX1B	107206
chr15	65117795	65118467	0.31	4.06E-03	PIF1	-293		
chr7	66202721	66202990	0.42	4.08E-03	RABGEF1	-2127		
chr2	234240298	234240608	0.50	4.14E-03	DGKD	-22700	SAG	24144

chr18	18551790	18552117	0.47	4.15E-03	ROCK1	139858		
chr19	13215186	13215779	0.44	4.15E-03	LYL1	-1802		
chr2	85173272	85173634	0.62	4.23E-03	KCMF1	-24778	TMSB10	40690
chr20	56255352	56255662	0.55	4.24E-03	ZBP1	-59875	PMEPA1	29524
chr9	92116319	92116674	0.39	4.27E-03	GADD45G	-103430	SEMA4D	-21886
chr7	8215830	8216274	0.55	4.28E-03	ICA1	85630	GLCCI1	207629
chr20	31332721	31333090	0.65	4.32E-03	COMMD7	-1092		
chr6	30484358	30484693	0.59	4.34E-03	PRR3	-40230	HLA-E	27255
chr16	29874544	29875049	0.23	4.34E-03	CDIPT	-219		
chr2	164202409	164202811	0.64	4.37E-03	KCNH7	-507370	FIGN	389903
chr5	158239407	158239717	0.48	4.38E-03	CLINT1	-953394	EBF1	287226
chrX	152602501	152602737	0.30	4.38E-03	ZFP92	-81162	ZNF275	3006
chr15	65136815	65137150	0.53	4.45E-03	ANKDD1A	-67118	PIF1	-19145
chr12	105131170	105131496	0.33	4.48E-03	SLC41A2	191139	CHST11	280555
chr3	51533361	51533608	0.73	4.52E-03	VPRBP	516		
chr5	137438650	137438974	0.57	4.55E-03	WNT8A	19038	NME5	36320
chr7	102920444	102920680	0.39	4.59E-03	DPY19L2P2	197		
chr15	57551678	57551992	0.58	4.64E-03	CGNL1	-116870	TCF12	341002
chr1	158921745	158922078	0.55	4.65E-03	IFI16	-57770	PYHIN1	20570
chr16	17554097	17554464	0.56	4.68E-03	XYLT1	10457		
chr12	122101657	122102035	0.62	4.72E-03	TMEM120B	-48812	ORAI1	37391
chr1	226537731	226538074	0.48	4.79E-03	LIN9	-40705	PARP1	57898
chr7	45018636	45018947	0.56	4.81E-03	MYO1G	-88		
chr22	23296927	23297171	0.56	4.82E-03	GNAZ	-115620	IGL@	67089
chr12	113859716	113860026	0.53	4.83E-03	SDSL	-345		
chr11	111285470	111286014	0.63	4.85E-03	POU2AF1	-35585	BTG4	97322
chr9	117379328	117379646	0.53	4.86E-03	C9orf91	5781	TNFSF15	188921
chr2	85765852	85766336	0.31	4.88E-03	MAT2A	-194		
chr19	1354510	1354754	0.46	4.89E-03	MUM1	-344		
chr12	6873431	6873758	0.53	4.95E-03	PTMS	-1946		
chrX	77041539	77042003	0.58	4.96E-03	ATRX	-52		
chr6	2989794	2990147	0.31	4.98E-03	SERPINB6	-17881	NQO2	-10096
chr17	34135981	34136520	0.18	5.03E-03	TAF15	-237		
chr7	1032822	1033108	0.54	5.08E-03	GPR146	-64176	CYP2W1	10130
chr3	187676418	187676807	0.56	5.18E-03	LPP	-254108	BCL6	-213138
chr19	17186205	17186543	0.22	5.21E-03	MYO9B	-217	HAUS8	-31
chr8	103825946	103826399	0.48	5.28E-03	KLF10	-158190	AZIN1	50224
chr17	73257690	73258010	0.61	5.32E-03	GGA3	-173	MRPS7	101

chr16	31191129	31191598	0.25	5.42E-03	FUS	-89		
chr8	42979587	42979823	0.43	5.42E-03	HGSNAT	-15887	SGK196	31048
chr6	106967895	106968313	0.41	5.46E-03	AIM1	8799	RTN4IP1	109269
chr3	121744243	121744518	0.55	5.49E-03	ILDR1	-3351		
chr1	207981068	207981378	0.54	5.51E-03	CD46	55821	CD34	103460
chr9	127533613	127533973	0.29	5.63E-03	NR6A1	-217		
chr2	12172558	12172857	0.53	5.64E-03	TRIB2	-684290	LPIN1	285968
chr1	24239715	24240129	0.46	5.65E-03	CNR2	-105		
chr19	38538817	38539317	0.44	5.73E-03	SIPA1L3	141199	DPF1	175823
chr11	44578508	44578818	0.65	5.74E-03	ALX4	-246947	CD82	-8478
chr14	60558346	60558832	0.53	5.80E-03	C14orf135	-40		
chr6	28219905	28220232	0.54	5.81E-03	ZKSCAN4	-67		
chr20	311166	311516	0.61	5.85E-03	NRSN2	-16029	SOX12	5102
chr4	963176	963486	0.36	5.87E-03	DGKQ	4013	TMEM175	37069
chr12	45937608	45937918	0.58	5.95E-03	ARID2	-185857	ANO6	327887
chr19	42927679	42928108	0.53	6.04E-03	CNFN	-33450	LIPE	3684
chr12	116309598	116309908	0.59	6.06E-03	MED13L	405238		
chr9	134270116	134270426	0.45	6.07E-03	BAT2L	-35206	PPAPDC3	105190
chr19	1449951	1450282	0.48	6.08E-03	APC2	-31		
chr13	76123464	76123937	0.27	6.10E-03	UCHL3	-226		
chr8	67578405	67578820	0.57	6.10E-03	VCPIP1	839		
chr2	234327255	234327531	0.44	6.10E-03	DGKD	64240	USP40	146843
chr1	147071572	147071886	0.53	6.14E-03	BCL9	58547	ACP6	70905
chr14	69152136	69152446	0.57	6.15E-03	ZFP36L1	107494	RAD51L1	865782
chr11	64901950	64902744	0.22	6.17E-03	SYVN1	-344		
chr6	31703947	31704374	0.52	6.25E-03	MSH5	-3613		
chr10	18483238	18483494	0.48	6.25E-03	CACNB2	53760	NSUN6	457184
chr20	8000350	8000660	0.55	6.26E-03	TMX4	-112		
chr11	66049923	66050240	0.41	6.31E-03	CNIH2	4386	YIF1A	6556
chr6	37786734	37787325	0.25	6.32E-03	ZFAND3	-277		
chr12	53493183	53493493	0.51	6.32E-03	SOAT2	-3936		
chr19	56330278	56330588	0.54	6.35E-03	NLRP4	-17511	RFPL4A	59926
chr17	73781050	73781360	0.41	6.35E-03	UNK	524		
chr8	48110226	48110514	0.48	6.37E-03	CEBPD	540356		
chr3	42003486	42003807	0.23	6.44E-03	ULK4	13		
chr11	11251675	11251985	0.35	6.48E-03	ZBED5	-372210	CSNK2A1	123074
chr3	193822024	193822446	0.28	6.49E-03	HES1	-31699	OPA1	511302
chr17	43225039	43225593	0.36	6.55E-03	HEXIM1	632		

chr17	1388198	1388508	0.55	6.57E-03	CRK	-28809	MYO1C	7648
chr15	75642247	75642551	0.58	6.74E-03	NEIL1	3068	MAN2C1	18542
chr19	7402263	7402574	0.45	6.74E-03	INSR	-108408	ARHGEF18	-102155
chr8	30013776	30014086	0.55	6.74E-03	DCTN6	118		
chr15	43802790	43803341	0.50	6.75E-03	TP53BP1	-17712	MAP1A	-6740
chr13	111366364	111366674	0.60	6.90E-03	ING1	-840		
chr7	5571518	5571780	0.52	6.93E-03	ACTB	-1417		
chr1	154833062	154833342	0.42	6.96E-03	ADAR	-252520	KCNN3	9552
chr15	63792562	63792798	0.44	7.08E-03	USP3	-4130		
chr2	44589708	44590018	0.59	7.17E-03	PREPL	-2974		
chr5	78907838	78908176	0.54	7.32E-03	PAPD4	-236		
chr2	114361682	114361992	0.54	7.46E-03	RABL2A	-22980	WASH1	8192
chr13	115046020	115046295	0.58	7.54E-03	UPF3A	-920		
chr17	16341923	16342406	0.25	7.59E-03	C17orf45	-136		
chr6	31528204	31528455	0.53	7.60E-03	LTA	-11546	NFKBIL1	12977
chr1	150039664	150040002	0.58	7.60E-03	VPS45	491		
chr19	53607006	53607317	0.69	7.62E-03	ZNF160	-475		
chr15	89010332	89011059	0.31	7.66E-03	MRPL46	-63	MRPS11	12
chr18	77384645	77384977	0.70	7.74E-03	CTDP1	-54990	NFATC1	224485
chr19	46975137	46975447	0.52	7.77E-03	CCDC8	-58373	AK094504	129165
chr7	1067908	1068177	0.41	7.77E-03	GPR146	-29098	CYP2W1	45208
chr17	21226215	21226585	0.62	7.80E-03	KCNJ12	-53299	MAP2K3	38432
chr8	57154531	57154777	0.63	7.86E-03	CHCHD7	30339	SDR16C5	78587
chr11	117103078	117103388	0.56	7.88E-03	PCSK7	-422	RNF214	-171
chr2	220019761	220020071	0.55	7.88E-03	IHH	-94727	NHEJ1	5671
chr2	69135017	69135339	0.55	7.89E-03	BMP10	-36529	GKN2	44924
chr17	37356071	37356636	0.29	7.93E-03	CACNB1	-2453	RPL19	-182
chr11	114030601	114030990	0.60	7.94E-03	NNMT	-135739	ZBTB16	99508
chr14	107259683	107260110	0.53	7.94E-03	ADAM6	-821539	IGHV7-81	23386
chr2	44223084	44223566	0.30	7.97E-03	LRPPRC	-181		
chr8	96281744	96282322	0.44	8.03E-03	PLEKHF2	135995	GDF6	890987
chr17	7108126	7108475	0.66	8.05E-03	ASGR1	-25418	DLG4	12627
chr22	29663674	29664328	0.31	8.16E-03	RHBDD3	-87	EWSR1	3
chr1	91966367	91966631	0.58	8.16E-03	CDC7	95		
chr15	89878009	89878398	0.22	8.20E-03	POLG	-178		
chr7	101582433	101582945	0.55	8.21E-03	SH2B2	-345716	CUX1	121807
chr2	214092819	214093129	0.46	8.21E-03	IKZF2	-77916	SPAG16	-56142
chr6	150943931	150944241	0.67	8.31E-03	MTHFD1L	-242605	PLEKHG1	23087

chr17	76126647	76126957	0.66	8.56E-03	TMC6	-1941	TMC8	-57
chr6	111580162	111580627	0.47	8.60E-03	KIAA1919	-87		
chr17	64188243	64188563	0.57	8.66E-03	AXIN2	-630663	APOH	37153
chr1	114471897	114472333	0.52	8.75E-03	HIPK1	119		
chr22	47010285	47010771	0.54	8.86E-03	CELSR1	-77461	GRAMD4	-12120
chr7	5572505	5572974	0.56	9.06E-03	ACTB	-2508		
chr9	116698625	116698887	0.59	9.27E-03	ZNF618	60194	AMBP	141996
chr10	126290033	126290288	0.48	9.35E-03	LHPP	139749	FAM53B	142769
chr13	46869560	46869889	0.64	9.38E-03	LRRC63	83642	C13orf18	91910
chr19	5130385	5130771	0.49	9.39E-03	KDM4B	161454	PTPRS	210236
chr8	67579712	67580031	0.60	9.43E-03	VCPIP1	-420		
chr1	150583984	150584294	0.54	9.45E-03	MCL1	-32003	ENSA	17959
chr14	55658175	55658634	0.57	9.49E-03	DLGAP5	-9		
chr10	14644009	14644319	0.59	9.49E-03	FRMD4A	-271298	CDNF	235819
chr13	42033076	42033320	0.50	9.49E-03	C13orf15	1656	KIAA0564	502023
chr6	32920723	32921266	0.50	9.57E-03	HLA-DMA	-96		
chr1	76250285	76250595	0.54	9.78E-03	RABGGTB	-1446		
chr1	26185977	26186283	0.49	9.87E-03	PAQR7	11614	FAM54B	39733
chr19	50093437	50093848	0.56	9.89E-03	PRR12	-1269		
chr1	32014600	32014910	0.59	1.00E-02	TINAGL1	-27331	SERINC2	128792
chr11	72853476	72853908	0.29	1.00E-02	FCHSD2	-549		
chr8	30014343	30014674	0.51	1.00E-02	DCTN6	696		
chr14	64702713	64703163	0.65	1.02E-02	ESR2	58190	SYNE2	383255
chr22	20817536	20817846	0.47	1.04E-02	SCARF2	-25545	KLHL22	32431
chr6	32939919	32940159	0.47	1.04E-02	BRD2	3602	HLA-DOA	37350
chr1	178994906	178995409	0.53	1.05E-02	FAM20B	84		
chr6	144164072	144164548	0.60	1.05E-02	PHACTR2	165208	PLAGL1	165231
chr16	84544907	84545217	0.60	1.05E-02	KIAA1609	-6774	COTL1	106607
chr17	76248420	76248715	0.60	1.06E-02	LOC283999	21177	SOCS3	107590
chr14	52642715	52642966	0.69	1.06E-02	NID2	-106895	PTGDR	-91590
chr14	93472870	93473180	0.47	1.07E-02	CHGA	83580	ITPK1	109238
chr12	53693326	53693764	0.54	1.07E-02	C12orf10	75		
chr6	88889445	88889751	0.57	1.07E-02	CNR1	-13831	RNGTT	783750
chr18	23670531	23671058	0.32	1.10E-02	SS18	-184		
chr12	95325	95647	0.12	1.10E-02	IQSEC3	-91056		
chr7	63781418	63781728	0.57	1.10E-02	AK301806	7181	ZNF680	241932
chr20	3748966	3749276	0.57	1.11E-02	SPEF1	12981	HSPA12B	35765
chr19	21105886	21106263	0.52	1.11E-02	ZNF85	-5		

chr18	47339908	47340218	0.61	1.12E-02	ACAA2	188		
chr10	33260615	33260929	0.52	1.12E-02	ITGB1	-13479	NRP1	363061
chr17	9953986	9954263	0.40	1.13E-02	RCVRN	-145441	GAS7	147743
chr6	43192117	43192502	0.57	1.13E-02	C6orf108	4901	CUL9	42388
chr2	19547708	19548049	0.62	1.14E-02	NT5C1B	-777041	OSR1	10493
chr16	22637920	22638208	0.42	1.14E-02	HS3ST2	-187796	NPIPL3	113180
chr15	64455160	64455726	0.36	1.14E-02	PPIB	-89		
chr13	41271891	41272171	0.61	1.15E-02	FOXO1	-31297	MRPS31	73316
chr4	38862469	38862782	0.58	1.15E-02	TLR6	-31468	FAM114A1	-6811
chr14	45366051	45366369	0.65	1.15E-02	FSCB	-389711	KLHL28	64969
chr7	37392536	37392783	0.54	1.16E-02	AOAH	-628507	ELMO1	95851
chr10	1095383	1095768	0.58	1.17E-02	IDI1	-515		
chr20	55892962	55893292	0.54	1.17E-02	BMP7	-51420	SPO11	-11704
chr8	38627617	38627861	0.56	1.18E-02	RNF5	-168964	TACC1	-16983
chr12	130443013	130443261	0.38	1.18E-02	FZD10	-203895	TMEM132D	-54925
chr10	111967178	111967470	0.70	1.19E-02	MXI1	-39		
chr6	116600990	116601362	0.58	1.19E-02	DSE	-107	TSPYL1	104
chr6	150338113	150338362	0.56	1.20E-02	RAET1L	8430	ULBP1	53095
chr5	150613307	150613596	0.58	1.20E-02	ANXA6	-76085	GM2A	-19161
chr19	36544703	36545058	0.53	1.20E-02	THAP8	783		
chr8	103540746	103541092	0.60	1.23E-02	UBR5	-116424	ODF1	-22929
chr1	154909128	154909815	0.22	1.24E-02	PMVK	12		
chr12	52463112	52463796	0.29	1.25E-02	C12orf44	-304		
chr4	149903321	149903640	0.52	1.25E-02	NR3C2	-539838		
chr21	43483274	43483638	0.59	1.25E-02	ZNF295	-52960	UMODL1	-7970
chr1	32729339	32729604	0.49	1.27E-02	LCK	-10240	EIF3I	41513
chr16	67581694	67582004	0.61	1.27E-02	CTCF	-14615	FAM65A	19095
chr8	95961551	95961828	0.53	1.28E-02	TP53INP1	-75		
chr10	17685859	17686169	0.65	1.28E-02	STAM	-110		
chr3	184374765	184375075	0.43	1.28E-02	VPS8	-155011	EPHB3	95333
chr11	29540349	29540726	0.71	1.31E-02	KCNA4	497950		
chr12	51319027	51319337	0.68	1.31E-02	METTL7A	648		
chr19	17246213	17246523	0.55	1.31E-02	USE1	-79787	MYO9B	59777
chr6	36733702	36734057	0.60	1.32E-02	CPNE5	73340	CDKN1A	87421
chr6	109416344	109416775	0.59	1.32E-02	SESN1	-1283		
chr12	68385080	68385356	0.65	1.33E-02	IFNG	168303	DYRK2	342706
chr9	126979043	126979326	0.70	1.33E-02	NEK6	-41058	LHX2	205296
chr7	99588554	99588805	0.60	1.35E-02	ZKSCAN1	-24539	BC022382	10295

chr13	47170212	47170522	0.63	1.35E-02	LRCH1	43071	ESD	201000
chr7	135242429	135242706	0.61	1.35E-02	NUP205	-94		
chr7	24860574	24861403	0.48	1.38E-02	DFNA5	-63906	OSBPL3	158771
chr17	71587538	71587863	0.64	1.39E-02	CDC42EP4	-279558	SDK2	52526
chr7	27779303	27779888	0.52	1.40E-02	TAX1BP1	-142		
chr10	496902	497175	0.46	1.43E-02	DIP2C	238569	ZMYND11	316615
chr7	100270899	100271403	0.26	1.43E-02	GNB2	-212		
chr8	74206324	74206654	0.58	1.43E-02	RDH10	-776	RPL7	-620
chr17	7387287	7387916	0.34	1.43E-02	POLR2A	-248	ZBTB4	-34
chr5	1134568	1134912	0.60	1.44E-02	SLC6A19	-66970	SLC12A7	-22568
chr2	47917255	47917565	0.51	1.46E-02	KCNK12	-119940	MSH6	-92811
chr5	133706606	133707152	0.38	1.47E-02	CDKL3	-4114	UBE2B	9
chr6	31543217	31543502	0.53	1.49E-02	TNF	-932	TNF	10
chr2	88927318	88927882	0.54	1.50E-02	EIF2AK3	-606		
chr19	47103868	47104392	0.30	1.50E-02	CALM3	-382	AK094504	327
chr1	165567264	165567740	0.69	1.51E-02	MGST3	-32948	LRRC52	54024
chr15	56830090	56830442	0.54	1.51E-02	MNS1	-72931	ZNF280D	195521
chr2	61765229	61765590	0.41	1.52E-02	XPO1	8		
chr12	109592505	109592815	0.59	1.52E-02	ACACB	15458	FOXN4	154365
chr3	16474192	16474520	0.65	1.52E-02	RFTN1	80866	OXNAD1	167642
chr1	44412317	44412627	0.63	1.53E-02	IPO13	-6		
chr7	75947278	75947589	0.50	1.53E-02	HSPB1	15559	YWHAG	40908
chr17	61774863	61775173	0.67	1.55E-02	LIMD2	2501	MAP3K3	75217
chr1	26613963	26614273	0.54	1.58E-02	SH3BGRL3	7905	UBXN11	19077
chr4	1714069	1714583	0.44	1.60E-02	SLBP	-296		
chr14	107119936	107120449	0.60	1.60E-02	ADAM6	-681835	IGHV7-81	163090
chr20	52226223	52226667	0.59	1.61E-02	ZNF217	-26738	BCAS1	460859
chr6	161794945	161795204	0.43	1.62E-02	AGPAT4	-99968		
chr6	34436254	34436506	0.69	1.62E-02	PACSIN1	2475	SPDEF	87711
chr1	161146835	161147218	0.58	1.62E-02	B4GALT3	287		
chr6	44214062	44214337	0.54	1.63E-02	HSP90AB1	-649		
chr2	10588340	10588702	0.55	1.63E-02	ODC1	-68		
chr11	85780433	85780810	0.59	1.65E-02	PICALM	-514		
chr6	14211228	14211551	0.51	1.65E-02	CD83	93525		
chr7	1177945	1178181	0.51	1.66E-02	C7orf50	-170		
chr1	228354036	228354378	0.52	1.67E-02	C1orf69	778		
chr2	153032236	153032546	0.69	1.68E-02	STAM2	115		
chr14	102553288	102553615	0.40	1.68E-02	HSP90AA1	52634	DYNC1H1	122587

chr6	43741109	43741419	0.58	1.70E-02	VEGFA	3311	MRPL14	353927
chr19	20149953	20150197	0.50	1.71E-02	ZNF682	202		
chr20	8256059	8256575	0.68	1.72E-02	PLCB4	-820615	PLCB1	143021
chr17	3595703	3596013	0.62	1.73E-02	P2RX5	3725	TMEM93	23768
chr22	30831675	30832046	0.57	1.73E-02	SEC14L3	36173	SEC14L2	38928
chr11	94964429	94964899	0.51	1.74E-02	SESN3	-418		
chr6	6588842	6589152	0.66	1.74E-02	LY86	63		
chr6	42714685	42715039	0.60	1.74E-02	TBCC	-978		
chr2	47922171	47922804	0.47	1.74E-02	KCNK12	-125018	MSH6	-87733
chr16	9184996	9185384	0.56	1.74E-02	USP7	-127849		
chr6	27805117	27805385	0.59	1.75E-02	HIST1H2BN	-1189	HIST1H2AK	866
chr7	138212420	138212730	0.48	1.77E-02	TRIM24	67496	SVOPL	151215
chr6	24721100	24721679	0.29	1.79E-02	C6orf62	-1987		
chr19	47939969	47940279	0.63	1.80E-02	MEIS3	-17339	SLC8A2	35183
chr10	90145406	90145966	0.58	1.80E-02	KILLIN	-522492	RNLS	197396
chr1	224517811	224518163	0.68	1.81E-02	NVL	-115		
chr7	39989290	39989746	0.62	1.81E-02	CDC2L5	-441		
chr1	209929309	209929619	0.68	1.81E-02	TRAF3IP3	-60		
chr16	23529655	23530113	0.70	1.81E-02	GGA2	-8076	EARS2	38812
chr18	12702586	12703034	0.55	1.82E-02	PSMG2	-254	CEP76	-107
chr19	4723805	4724175	0.28	1.83E-02	DPP9	-135		
chr6	31548645	31548919	0.57	1.88E-02	LTB	1420	TNF	4490
chr6	35704101	35704367	0.59	1.88E-02	C6orf81	-625		
chr6	33560986	33561253	0.63	1.89E-02	ITPR3	-28041	BAK1	-13050
chr1	157743844	157744154	0.65	1.91E-02	FCRL3	-73352	FCRL2	2923
chr17	56591810	56592120	0.58	1.91E-02	HSF5	-26206	MTMR4	3286
chr12	117240870	117241281	0.54	1.92E-02	RNFT2	64980	HRK	78156
chr11	19454949	19455259	0.51	1.93E-02	NAV2	-279777	E2F8	-192597
chr14	107256632	107256900	0.61	1.94E-02	ADAM6	-818408	IGHV7-81	26517
chr1	155949393	155949703	0.55	1.94E-02	ARHGEF2	-1212		
chr22	38029042	38029484	0.53	1.95E-02	SH3BP1	-5794	GGA1	24760
chr19	19477546	19477856	0.58	1.95E-02	GATAD2A	-18941	KIAA0892	46071
chr8	57124216	57124512	0.65	1.96E-02	PLAG1	-505	CHCHD7	49
chr7	101673966	101674229	0.57	1.96E-02	SH2B2	-254307	CUX1	213216
chr9	106856281	106856591	0.58	1.98E-02	SMC2	-105		
chr7	87562972	87563397	0.58	1.98E-02	ADAM22	-517		
chr19	36706419	36706729	0.62	1.99E-02	ZNF565	-1008		
chr3	191849739	191849984	0.53	2.00E-02	FGF12	276976	PYDC2	670910

chr6	36727006	36727279	0.49	2.00E-02	CPNE5	80077	CDKN1A	80684
chr7	18560191	18560501	0.62	2.00E-02	HDAC9	24461	TWIST1	596949
chr7	5371034	5371319	0.52	2.02E-02	SLC29A4	48616	TNRC18	92000
chr1	52499365	52499675	0.68	2.02E-02	KTI12	-48		
chr8	22832149	22832401	0.57	2.02E-02	PEBP4	-46854	RHOBTB2	-21086
chr18	21589671	21589980	0.59	2.02E-02	CABYR	-129129	LAMA3	320264
chr18	3247211	3247503	0.54	2.04E-02	MYL12A	-171		
chr6	3024105	3024362	0.66	2.04E-02	RIPK1	-52764	NQO2	24167
chr1	39735377	39735703	0.63	2.05E-02	MACF1	-61270	NDUFS5	243534
chr17	39852271	39852590	0.69	2.05E-02	GAST	-16182	EIF1	7304
chr7	33102602	33102988	0.60	2.05E-02	NT5C3	-386		
chr7	66205374	66205731	0.56	2.08E-02	RABGEF1	570		
chr1	11029217	11029500	0.56	2.08E-02	CASZ1	-172652	TARDBP	-43320
chr17	18804262	18804573	0.67	2.09E-02	SLC5A10	-51060	PRPSAP2	42926
chr14	71583171	71583593	0.64	2.09E-02	SIPA1L1	-412647	PCNX	209260
chr2	84686369	84686736	0.55	2.11E-02	SUCLG1	33		
chr19	14629143	14629387	0.51	2.11E-02	DNAJB1	-64		
chr15	60973556	60973828	0.47	2.13E-02	RORA	-53963		
chr7	36231505	36231941	0.60	2.14E-02	ANLN	-197709	EEPD1	38887
chr3	16557346	16557844	0.70	2.15E-02	RFTN1	-2373		
chr17	65362600	65362910	0.75	2.16E-02	PSMD12	-34		
chr7	7221733	7222129	0.53	2.16E-02	C1GALT1	-315		
chr3	187662495	187662971	0.59	2.16E-02	LPP	-267988	BCL6	-199258
chr20	49575031	49575379	0.43	2.18E-02	MOCS3	-158	DPM1	-145
chr11	102980041	102980313	0.61	2.20E-02	DYNC2H1	17		
chr15	68522166	68522609	0.61	2.20E-02	CLN6	-308		
chr6	30698601	30698960	0.54	2.24E-02	TUBB	10624	FLOT1	11672
chr19	50179598	50180064	0.36	2.26E-02	PRMT1	-665		
chr6	31409456	31409702	0.50	2.26E-02	HCP5	-21378	MICA	38208
chr13	41722872	41723182	0.51	2.26E-02	KBTBD6	-16091	KBTBD7	45675
chr6	32659868	32660112	0.58	2.27E-02	HLA-DQA2	-49173	HLA-DQB1	-25524
chr17	56083834	56084144	0.61	2.31E-02	SFRS1	718		
chr3	56817942	56818196	0.82	2.31E-02	ERC2	-315678	ARHGEF3	295267
chr4	47916441	47916784	0.65	2.31E-02	NFXL1	20		
chr8	42249804	42250152	0.59	2.37E-02	VDAC3	632		
chr2	136874550	136875038	0.67	2.38E-02	CXCR4	931		
chr6	41286576	41286885	0.55	2.38E-02	TREM1	-32274	NCR2	-16797
chr10	3853662	3853972	0.68	2.39E-02	KLF6	-26344		

chr14	89290690	89291000	0.59	2.39E-02	TTC8	-73		
chr7	150594934	150595300	0.60	2.42E-02	ABP1	45544	KCNH2	79897
chr1	175288633	175288943	0.76	2.42E-02	TNN	251794	TNR	423964
chr3	134204488	134205101	0.36	2.44E-02	CEP63	-252	ANAPC13	68
chr7	915778	916171	0.53	2.45E-02	C7orf20	-216		
chr8	42195884	42196212	0.52	2.46E-02	POLB	18		
chr1	162467389	162467699	0.63	2.48E-02	UHMK1	-111		
chr11	60929105	60929544	0.68	2.49E-02	VPS37C	-409		
chr3	39425623	39426109	0.56	2.56E-02	RPSA	-22338	SLC25A38	1051
chr5	150535126	150535375	0.61	2.58E-02	TNIP1	-74254	ANXA6	2116
chr11	67423822	67424132	0.64	2.58E-02	ACY3	-5847	ALDH3B2	24708
chr17	55979060	55979306	0.63	2.59E-02	MRPS23	-51784	VEZF1	86432
chr6	5260820	5261301	0.61	2.60E-02	FARS2	-523	LYRM4	107
chr19	11190711	11190963	0.44	2.60E-02	LDLR	-9220	SMARCA4	119239
chr6	99873180	99873563	0.60	2.61E-02	SFRS18	-165		
chr11	111779939	111780189	0.68	2.64E-02	HSPB2	-3396	CRYAB	2409
chr1	40282698	40283298	0.61	2.67E-02	BMP8B	-28465	TRIT1	66179
chr12	56321689	56321951	0.59	2.67E-02	DGKA	-3992	WIBG	-123
chr5	140892655	140892974	0.60	2.69E-02	PCDHGC5	24007	DIAPH1	105807
chr11	94987490	94987817	0.61	2.70E-02	CEP57	-535988	SESN3	-23408
chr6	31763518	31763829	0.56	2.71E-02	VARS	38		
chr9	130497619	130497929	0.60	2.71E-02	TOR2A	-170		
chr13	51086192	51086547	0.70	2.71E-02	RNASEH2B	-397522	ST13	340216
chr8	48872516	48872951	0.62	2.76E-02	MCM4	-760	PRKDC	9
chr6	5261496	5261806	0.62	2.77E-02	LYRM4	-483	FARS2	67
chr10	104404631	104405065	0.57	2.77E-02	TRIM8	596		
chr2	232537693	232538022	0.54	2.78E-02	NMUR1	-142676	PTMA	-35377
chr3	194392900	194393401	0.35	2.83E-02	LSG1	55		
chr8	41480475	41480843	0.59	2.85E-02	NKX6-3	24216	AGPAT6	44952
chr6	155739126	155739462	0.62	2.85E-02	TFB1M	-103668	NOX3	37743
chr10	60144854	60145413	0.63	2.86E-02	TFAM	-42		
chr1	76192276	76192586	0.61	2.87E-02	RABGGTB	-59455	ACADM	2388
chr3	48342594	48343055	0.63	2.88E-02	NME6	23		
chr6	13526826	13527389	0.56	2.88E-02	SIRT5	-47767	GFOD1	-39321
chr11	22647337	22647693	0.68	2.92E-02	FANCF	-128		
chr5	90676792	90677105	0.54	2.95E-02	ARRDC3	2200	GPR98	822332
chr4	180911257	180911567	0.57	2.95E-02				
chr10	122610658	122611313	0.59	2.97E-02	BRWD2	291		

chr6	32383276	32383521	0.60	2.97E-02	HLA-DRA	-24248	BTNL2	-8499
chr8	120921463	120921773	0.49	3.01E-02	COL14A1	-215734	DEPDC6	35718
chr2	60782394	60782902	0.58	3.01E-02	BCL11A	-2015		
chr10	74094664	74094969	0.58	3.04E-02	DNAJB12	20090	DDIT4	61140
chr3	52444980	52445290	0.56	3.04E-02	BAP1	-1126	PHF7	608
chr15	68076698	68077014	0.62	3.05E-02	LBXCOR1	-41085	MAP2K5	241835
chr5	150503659	150504164	0.63	3.08E-02	TNIP1	-42915	ANXA6	33455
chr13	50071109	50071419	0.81	3.08E-02	PHF11	1463	RCBTB1	88455
chr16	30470121	30470465	0.62	3.09E-02	ITGAL	-13690	SEPHS2	-13069
chr6	89791274	89791604	0.51	3.09E-02	PNRC1	1010	SFRS13B	36361
chr12	94815945	94816255	0.54	3.09E-02	TMCC3	228224	PLXNC1	273601
chr6	24919843	24920153	0.66	3.13E-02	FAM65B	-8803	DKFZp686H12134	218053
chr5	40755784	40756228	0.64	3.13E-02	TTC33	66		
chr14	64970111	64970421	0.66	3.14E-02	ZBTB1	-1026	ZBTB25	288
chr5	6633174	6633441	0.68	3.14E-02	SRD5A1	-192	NSUN2	-151
chr2	6450063	6450400	0.54	3.15E-02	CMPK2	555704	SOX11	617433
chr7	66386979	66387404	0.56	3.17E-02	C7orf42	989		
chr19	42784441	42784751	0.66	3.18E-02	CIC	-4221		
chr7	64459687	64460102	0.66	3.19E-02	ZNF117	-8481	ERV3	7226
chr5	95159933	95160243	0.57	3.19E-02	GLRX	-1511		
chr17	73201535	73201859	0.69	3.19E-02	NUP85	100		
chr12	125348254	125348564	0.55	3.19E-02	SCARB1	110		
chr17	77970866	77971252	0.77	3.26E-02	CBX4	-157846	TBC1D16	38588
chr6	33216108	33216384	0.60	3.26E-02	VPS52	23416	RING1	39960
chr10	103815863	103816173	0.59	3.27E-02	C10orf76	-86		
chr6	24936016	24936326	0.66	3.29E-02	FAM65B	-24976	DKFZp686H12134	201880
chr1	32254116	32254426	0.68	3.34E-02	BAI2	-24623	SPOCD1	27309
chr2	61067985	61068686	0.48	3.35E-02	REL	-40416	PAPOLG	84953
chr16	84633372	84633744	0.58	3.38E-02	KIAA1609	-95270	COTL1	18111
chr6	89351163	89351554	0.55	3.39E-02	CNR1	-475592	RNGTT	321989
chr7	35577063	35577373	0.64	3.41E-02	TBX20	-283976	HERPUD2	157554
chr16	69830251	69830561	0.73	3.42E-02	CLEC18A	-154220	WWP2	34132
chr7	2749368	2749736	0.58	3.42E-02	AMZ1	30389	GNA12	134407
chr18	77782749	77783002	0.76	3.42E-02	TXNL4A	-34344	C18orf22	-11482
chr20	62580539	62580910	0.55	3.45E-02	UCKL1	7043	DNAJC5	54207
chr10	38299198	38299508	0.68	3.46E-02	ZNF33A	-225		
chr8	67525476	67525881	0.65	3.48E-02	MYBL1	-199		
chr11	3875624	3875917	0.68	3.51E-02	STIM1	-1162		

chr6	41701857	41702217	0.63	3.51E-02	TFEB	761		
chr1	24286075	24286418	0.55	3.53E-02	PNRC2	-54		
chr6	34216839	34217124	0.67	3.59E-02	C6orf1	-78		
chr2	27579318	27579782	0.57	3.60E-02	GTF3C2	318		
chr6	47215240	47215550	0.72	3.60E-02	GPR110	-205313	TNFRSF21	62285
chr4	25225396	25225706	0.62	3.60E-02	SEPSECS	-63347	PI4K2B	-10102
chr12	121124194	121124830	0.47	3.61E-02	MLEC	-437		
chr7	5534320	5534596	0.53	3.63E-02	TNRC18	-71281	FBXL18	18941
chr7	90338157	90338449	0.66	3.68E-02	PFTK1	-409		
chr11	64615678	64616012	0.54	3.69E-02	CDC42BPG	-3804		
chr12	8113308	8113670	0.67	3.75E-02	FOXJ2	-71870	SLC2A3	-24597
chr10	102289546	102289951	0.66	3.81E-02	NDUFB8	-113		
chr6	42713947	42714495	0.65	3.83E-02	TBCC	-337		
chr20	34252765	34253120	0.69	3.87E-02	RBM12	-95	CPNE1	-95
chr14	94429399	94429739	0.69	3.88E-02	OTUB2	-63155	ASB2	-5802
chr5	132387361	132387823	0.42	3.90E-02	HSPA4	-70		
chr6	159515557	159516032	0.59	3.90E-02	FNDC1	-74634	TAGAP	-49611
chr12	2921513	2921823	0.68	3.92E-02	ITFG2	-195		
chr7	100183648	100184218	0.46	3.94E-02	FBXO24	-3263	LRCH4	-157
chr12	656192	656447	0.54	3.95E-02	B4GALNT3	86777	NINJ2	116435
chr3	52755022	52755277	0.45	3.99E-02	SPCS1	15293	NEK4	49801
chr7	91875147	91875507	0.60	4.01E-02	ANKIB1	-221	KRIT1	87
chr19	7405939	7406273	0.58	4.02E-02	INSR	-112095	ARHGEF18	-98468
chr17	7117255	7117565	0.63	4.05E-02	ASGR1	-34527	DLG4	3518
chr7	24859905	24860236	0.57	4.06E-02	DFNA5	-62988	OSBPL3	159689
chr10	5882240	5882596	0.62	4.09E-02	FBXO18	-53931	GDI2	-26906
chr2	65597807	65598117	0.58	4.10E-02	SPRED2	61694	ACTR2	143133
chr7	73148870	73149327	0.61	4.14E-02	STX1A	-15111	ABHD11	4091
chr1	205564572	205564926	0.68	4.14E-02	MFSD4	26637	SLC45A3	84881
chr11	57508680	57508938	0.65	4.15E-02	C11orf31	87		
chr19	2086587	2086897	0.65	4.15E-02	MKNK2	-35499	MOBKL2A	9527
chr12	46660745	46661050	0.75	4.18E-02	SFRS2IP	-276497	SLC38A1	2310
chr3	9791409	9791734	0.60	4.20E-02	OGG1	-56		
chr19	10946955	10947265	0.66	4.20E-02	TMED1	-127		
chr12	118813924	118814322	0.68	4.21E-02	TAOK3	-3373	SUDS3	-235
chr17	74456697	74456954	0.59	4.24E-02	UBE2O	-7538	AANAT	-6825
chr8	59720535	59720771	0.61	4.25E-02	NSMAF	-148687	TOX	311114
chr7	31162235	31162495	0.51	4.25E-02	ADCYAP1R1	70223	NEUROD6	218173

chr21	35014038	35014348	0.70	4.30E-02	ITSN1	-591	CRYZL1	-33
chr19	44281962	44282272	0.49	4.33E-02	C19orf61	-22975	KCNN4	3292
chr12	6532656	6533095	0.67	4.36E-02	CD27	-21175	LTBR	39519
chr13	44817382	44817714	0.64	4.38E-02	ENOX1	-613935	SERP2	-130430
chr17	40168367	40168616	0.48	4.43E-02	NKIRAS2	-3595	DNAJC7	1179
chr7	92219587	92219864	0.61	4.50E-02	PEX1	-61881	CDK6	246215
chr1	36689290	36689976	0.57	4.53E-02	THRAP3	-384		
chr8	96243546	96243814	0.69	4.54E-02	PLEKHF2	97642	GDF6	929340
chr11	48040166	48040476	0.60	4.56E-02	OR4B1	-198041	PTPRJ	38211
chr6	15901310	15901718	0.66	4.57E-02	DTNBP1	-238243	MYLIP	-227803
chr1	115259262	115259792	0.62	4.58E-02	NRAS	-12		
chr8	102063952	102064220	0.50	4.63E-02	YWHAZ	-98865	ZNF706	153874
chr8	38853799	38854231	0.59	4.64E-02	ADAM9	-490	TM2D2	26
chr20	34042392	34042765	0.66	4.74E-02	CEP250	-571		
chr13	53226822	53227233	0.59	4.74E-02	SUGT1	197		
chr1	204490360	204490791	0.66	4.74E-02	MDM4	5065	LRRN2	164021
chr5	74632606	74633077	0.57	4.77E-02	HMGCR	-151		
chr7	5271433	5271697	0.59	4.77E-02	SLC29A4	-50996	WIPI2	41730
chr8	38324185	38324732	0.67	4.77E-02	FGFR1	1893	LETM2	80439
chr20	57987907	57988217	0.63	4.79E-02	PHACTR3	-191541	EDN3	112563
chr10	69834585	69834936	0.70	4.81E-02	HERC4	342		
chr12	131355441	131355751	0.71	4.90E-02	RAN	-1021		
chr8	42911303	42911550	0.62	4.90E-02	FNTA	-15		
chr2	27651117	27651608	0.65	4.90E-02	NRBP1	178		
chr6	30181105	30181449	0.62	4.91E-02	TRIM26	-124		
chr6	41978131	41978566	0.54	4.93E-02	CCND3	-68797	TAF8	-39902
chr6	31509612	31510213	0.58	4.95E-02	BAT1	312		
chr8	98655781	98656146	0.71	4.96E-02	MTDH	-443		
chr6	111136343	111136757	0.55	4.98E-02	CDC2L6	-138		
chr17	47287858	47288168	0.60	4.98E-02	GNGT2	-1270	ABI3	424
chr19	7239592	7239997	0.61	4.99E-02	INSR	54216	ZNF557	170324

Nonsignificant								
chr5	140943769	140944022	1.59	5.00E-02	DIAPH1	54726	PCDHGC5	75088
chr20	30640591	30640887	1.54	5.00E-02	HCK	682		
chrX	155259740	155260033	0.68	5.02E-02	WASH1	9920		
chr12	94070928	94071221	1.45	5.03E-02	CRADD	-76		
chr17	16877172	16877416	1.66	5.05E-02	TNFRSF13B	-1892		
chr19	50380554	50381084	0.39	5.05E-02	AKT1S1	-207	TBC1D17	-10
chr17	6939034	6939293	1.44	5.06E-02	SLC16A13	-230		
chr19	21688206	21688482	0.57	5.07E-02	ZNF429	-93		
chr6	134568316	134568603	1.65	5.07E-02	SLC2A12	-194671	SGK1	70736
chr4	185202564	185202864	1.75	5.07E-02	ENPP6	-63600	IRF2	193012
chr13	92001186	92001488	1.40	5.07E-02	GPC5	-49598		
chr19	48763379	48763684	1.67	5.08E-02	ZNF114	-10970	CARD8	-10607
chr19	17862079	17862484	0.63	5.08E-02	B3GNT3	-43637	MAP1S	31979
chr6	1861080	1861519	1.71	5.09E-02	FOXC1	250619	GMDS	384546
chr11	66568550	66568853	1.81	5.09E-02	SPTBN2	-79832	RCE1	-42181
chr17	80415912	80416192	1.52	5.10E-02	NARF	-488		
chr17	39844723	39845274	0.62	5.11E-02	EIF1	-128		
chr19	50934885	50935195	1.62	5.11E-02	MYBPC2	-1120		
chr4	106629687	106630058	1.49	5.11E-02	GSTCD	-2097	INTS12	8
chr6	26197307	26197703	0.72	5.11E-02	HIST1H2BF	-2282	HIST1H3D	1959
chr3	38178426	38178890	1.42	5.13E-02	MYD88	-1311	ACAA1	75
chr12	94542959	94543203	0.47	5.13E-02	PLXNC1	582		
chr12	132628798	132629091	1.48	5.13E-02	DDX51	-65	NOC4L	-48
chr14	107287443	107287687	0.56	5.15E-02	IGHV7-81	-4282		
chr17	45000216	45000633	0.74	5.16E-02	GOSR2	-61		
chr11	110198870	110199202	2.24	5.16E-02	FDX1	-101558	RDX	-31599
chr7	140179138	140179433	1.60	5.17E-02	MKRN1	83		
chr8	41521399	41521709	0.53	5.18E-02	NKX6-3	-16679	ANK1	232726
chr9	116037536	116037986	1.54	5.18E-02	PRPF4	-213	CDC26	108
chr7	101948772	101949051	0.58	5.19E-02	PRKRIP1	-87892	SH2B2	20507
chr11	66035741	66036100	1.52	5.20E-02	RAB1B	-135		
chr3	38125871	38126107	0.66	5.20E-02	DLEC1	45293	ACAA1	52744
chr20	2082737	2083356	0.36	5.25E-02	STK35	-367		
chr11	269224	269645	0.56	5.25E-02	NLRP6	-9135	PSMD13	32627
chr15	100052206	100052542	1.51	5.25E-02	MEF2A	-53759	LRRC28	260722
chr15	72766388	72766727	0.68	5.26E-02	ARIH1	-109		
chr13	99739848	99740204	1.32	5.27E-02	DOCK9	-1366		

chr20	57482868	57483187	0.60	5.28E-02	TH1L	-73283	GNAS	54992
chr12	48276969	48277303	1.56	5.29E-02	HDAC7	-63373	VDR	21678
chr1	45189161	45189435	0.54	5.30E-02	KIF2C	-16192	C1orf228	48904
chr6	43692852	43693096	0.64	5.30E-02	VEGFA	-44979	MRPS18A	-37446
chr7	108166345	108167093	1.62	5.31E-02	PNPLA8	-81		
chr17	4920214	4920648	0.66	5.37E-02	GPR172B	18296	KIF1C	19155
chr2	179279401	179279677	1.54	5.38E-02	PRKRA	36419	OSBPL6	94568
chr10	105230937	105231285	0.73	5.39E-02	CALHM1	-12463	CALHM3	7886
chr8	52814691	52814977	0.74	5.40E-02	PCMTD1	-3099		
chr1	144918027	144918303	1.88	5.40E-02	PPIAL4A	-553919	PDE4DIP	76857
chr20	43214984	43215362	1.64	5.41E-02	PKIG	54737	ADA	65203
chr19	47734503	47734826	1.45	5.41E-02	BBC3	1358	SAE1	100585
chr14	77428116	77428541	1.69	5.44E-02	C14orf4	66705	C14orf166B	135604
chr16	74401812	74402289	0.57	5.44E-02	LOC283922	102		
chr12	120730644	120731107	2.00	5.45E-02	PXN	-27313	SIRT4	-9287
chr1	6086213	6086519	1.50	5.45E-02	KCNAB2	-14		
chr2	27294467	27294781	1.33	5.46E-02	LOC100128731	-57		
chr1	38388546	38388892	0.62	5.46E-02	MTF1	-63427	INPP5B	24010
chr1	173379837	173380368	1.56	5.46E-02	TNFSF4	-203632	PRDX6	-66383
chr20	30623230	30623519	1.59	5.46E-02	HCK	-16682	XKR7	67570
chr7	128095584	128096043	1.63	5.47E-02	C7orf68	-70		
chr12	8796938	8797250	1.65	5.48E-02	AICDA	-31652	MFAP5	18339
chr9	140024070	140024342	1.46	5.49E-02	DPP7	-15011	GRIN1	-9403
chr3	170075170	170075520	1.44	5.50E-02	SKIL	-128		
chr11	130051331	130051621	1.60	5.50E-02	ST14	21794	ZBTB44	133131
chr7	44790614	44790911	1.47	5.51E-02	PPIA	-45478	ZMIZ2	2233
chr19	37019597	37020064	1.62	5.51E-02	ZNF260	-661		
chr4	25880327	25880655	0.70	5.54E-02	C4orf52	-35323	SEL1L3	-15881
chr7	140090722	140090985	1.50	5.54E-02	JHDM1D	-214113	SLC37A3	7457
chr14	74181082	74181403	0.46	5.55E-02	PNMA1	-115		
chr10	105880649	105880915	1.59	5.55E-02	GSTO1	-133896	COL17A1	-35144
chr2	26101229	26101772	1.41	5.56E-02	ASXL2	-189		
chr19	1407195	1407590	1.42	5.56E-02	DAZAP1	-191		
chr19	57791584	57791931	1.42	5.56E-02	ZNF460	-95		
chr22	43583507	43583855	1.52	5.56E-02	TTLL12	-574		
chr3	177076500	177076744	1.59	5.57E-02	TBL1XR1	-161574		
chr2	8822076	8822319	1.49	5.57E-02	ID2	214		
chrX	148622375	148622619	1.68	5.57E-02	CXorf40A	-60	CXorf40A	-46

chr2	32235856	32236340	1.49	5.58E-02	MEMO1	-400		
chr10	101774149	101774434	1.55	5.59E-02	DNMBP	-4616		
chr1	247553549	247553825	1.56	5.59E-02	ZNF496	-58642	NLRP3	-25771
chr17	79829134	79829475	1.59	5.62E-02	ARHGDIA	-67		
chr7	92439078	92439351	1.62	5.63E-02	PEX1	-281370	CDK6	26726
chr5	173248181	173248470	1.81	5.63E-02	BOD1	-204660	CPEB4	-67005
chr5	180402381	180402684	1.77	5.64E-02	BTNL3	-13368	BTNL8	76456
chr3	133207817	133208088	0.64	5.64E-02	CDV3	-84481	BFSP2	89163
chr11	64618690	64619096	1.61	5.65E-02	CDC42BPG	-6852	EHD1	27298
chr4	185249187	185249851	1.53	5.65E-02	ENPP6	-110405	IRF2	146207
chr1	14029298	14029638	1.48	5.65E-02	PRDM2	-1882		
chr12	117299247	117299598	1.84	5.66E-02	HRK	19809	RNFT2	123327
chr1	112912289	112912618	0.58	5.67E-02	KCND3	-380677	CTTNBP2NL	-26346
chr15	65184418	65184696	1.65	5.67E-02	PIF1	-66719	ANKDD1A	-19544
chr4	57623944	57624254	0.76	5.68E-02	HOPX	-101411	SPINK2	63794
chr15	77480161	77480471	1.92	5.69E-02	TSPAN3	-116803	SGK269	97017
chr1	145516101	145516377	1.64	5.70E-02	PEX11B	-144		
chr11	118077230	118077540	0.51	5.70E-02	SCN2B	-30048	AMICA1	18424
chr16	30645446	30645967	0.68	5.70E-02	FBRS	-30071	ZNF689	-24025
chr4	76439374	76439795	0.68	5.71E-02	THAP6	-69	RCHY1	43
chr1	65329700	65330025	0.63	5.72E-02	JAK1	102324	RAVER2	119085
chr6	53658399	53658684	1.40	5.77E-02	LRRC1	-992		
chr2	20550620	20550877	1.50	5.77E-02	RHOB	-96086	PUM2	-23610
chr1	54738706	54738979	1.45	5.77E-02	MRPL37	73003	SSBP3	133249
chr6	21593755	21594157	0.64	5.80E-02	SOX4	-16		
chr3	319975	320258	1.68	5.81E-02	CNTN6	-814512	CHL1	81467
chr19	5968040	5968289	0.71	5.82E-02	RANBP3	10155	CAPS	53972
chr7	46508427	46508663	0.63	5.82E-02	IGFBP3	-547674		
chr12	14766704	14767039	1.61	5.85E-02	PLBD1	-46081	GUCY2C	82647
chr18	20513134	20513725	0.65	5.85E-02	RBBP8	135		
chr5	124084379	124084689	0.66	5.85E-02	ZNF608	-3669		
chr15	64752507	64752858	1.39	5.86E-02	ZNF609	-38936	TRIP4	72663
chr4	129209152	129209454	1.44	5.88E-02	PGRMC2	-355		
chr20	36156254	36156564	0.69	5.88E-02	BLCAP	-106		
chr20	43093924	43094172	0.55	5.89E-02	TTPAL	-10499	HNF4A	64124
chr6	151086371	151086840	1.50	5.89E-02	MTHFD1L	-100085	PLEKHG1	165607
chr14	22749534	22749873	0.64	5.89E-02	TCRDV2	-178385	TRA	273940
chr10	101605635	101605879	1.52	5.89E-02	ABCC2	63294	DNMBP	163919

chr11	121300943	121301310	1.56	5.90E-02	SORL1	-21834	SC5DL	137739
chr19	16370436	16370827	1.49	5.90E-02	KLF2	-65019	AP1M1	61967
chr4	82483342	82483618	1.81	5.90E-02	RASGEF1B	-90419	HNRNPD	811669
chr2	226140979	226141271	2.12	5.90E-02	DOCK10	-233795		
chr3	196369978	196370483	1.60	5.91E-02	PIGX	-69015	LRRC33	3575
chr9	115141905	115142364	1.52	5.91E-02	HSDL2	-216		
chr19	45594792	45595045	0.62	5.92E-02	GEMIN7	12401	NKPD1	68489
chr4	84133654	84134077	1.32	5.93E-02	COQ2	72098	COPS4	177627
chr18	46986220	46986530	0.70	5.94E-02	DYM	704		
chr13	27998540	27998913	1.37	5.94E-02	GTF3A	46		
chr2	44706562	44706853	1.44	5.95E-02	SIX3	-462329	PREPL	-119819
chr7	105697916	105698290	0.75	5.99E-02	SYPL1	54954	FLJ23834	94446
chrX	1600645	1600994	1.57	6.04E-02	ASMTL	-28976	P2RY8	55217
chr11	66886454	66886855	1.31	6.04E-02	KDM2A	-85		
chr11	19575336	19575646	0.58	6.05E-02	E2F8	-312984	NAV2	-159390
chr6	138132585	138132831	1.58	6.05E-02	OLIG3	-317177	TNFAIP3	-55873
chr19	57946500	57946837	1.33	6.06E-02	ZNF749	-24		
chr9	37294083	37294386	1.50	6.06E-02	GRHPR	-128472	ZCCHC7	173766
chr19	48018334	48018939	1.50	6.06E-02	NAPA	-140		
chr3	42054160	42054809	0.58	6.07E-02	TRAK1	-78261	ULK4	-50825
chr12	14537833	14538129	1.38	6.08E-02	ATF7IP	19370	PLBD1	182810
chr11	119205051	119205295	1.55	6.09E-02	RNF26	-64		
chr10	14640449	14640740	1.61	6.09E-02	FRMD4A	-267729	CDNF	239388
chr21	34587894	34588204	1.64	6.13E-02	IFNAR2	-14182	OLIG1	145599
chr6	33575588	33575845	0.55	6.13E-02	BAK1	-27647	ITPR3	-13444
chr3	23705417	23705727	0.44	6.13E-02	UBE2E1	-141867	UBE2E2	460788
chr7	135194425	135194787	0.70	6.14E-02	CNOT4	245		
chr11	66115444	66115817	1.44	6.18E-02	BRMS1	-3049	B3GNT1	-470
chr16	27437744	27438054	1.72	6.20E-02	IL21R	-680		
chr6	107978974	107979241	0.66	6.21E-02	SCML4	166413	SOBP	167791
chr2	10588774	10589018	1.29	6.22E-02	ODC1	-443		
chr16	85603893	85604169	1.62	6.22E-02	KIAA0182	-42893	KIAA0513	542621
chr2	88301252	88301562	0.71	6.23E-02	RGPD1	-176092	SMYD1	-65975
chr19	8260052	8260401	0.68	6.26E-02	FBN3	-47846	LASS4	-13990
chr7	104585523	104585914	0.56	6.28E-02	MLL5	-68918	LHFPL3	616615
chr19	40030689	40030959	1.47	6.28E-02	EID2	14		
chr11	17297927	17298410	0.67	6.29E-02	NUCB2	-117		
chr10	74008576	74008913	0.61	6.29E-02	DDIT4	-24932	C10orf104	32939

chr4	139163397	139163695	1.65	6.31E-02	SLC7A11	-43		
chr7	30485484	30485788	0.62	6.32E-02	NOD1	32757	ZNRF2	161713
chr3	71613596	71613864	1.60	6.32E-02	FOXP1	19410		
chr22	48494099	48494452	1.80	6.33E-02	FAM19A5	-391012		
chr11	128563712	128564041	1.78	6.34E-02	FLI1	64		
chr15	57025721	57025997	1.76	6.38E-02	ZNF280D	-72		
chr18	72166454	72166716	1.75	6.39E-02	CNDP1	-35107	CNDP2	2988
chr14	60679264	60679574	1.77	6.39E-02	DHRS7	-47208	PPM1A	-33051
chr20	55926137	55926394	1.48	6.40E-02	RAE1	-352		
chr14	107238866	107239256	0.65	6.40E-02	ADAM6	-800703	IGHV7-81	44222
chr15	40857158	40857434	1.58	6.43E-02	RPUSD2	-4241		
chr20	18774536	18774819	1.51	6.43E-02	C20orf79	-19692	DTD1	206122
chr11	95524126	95524479	0.80	6.43E-02	CEP57	661		
chr1	6052683	6052959	1.45	6.44E-02	NPHP4	-290		
chr2	8374799	8375185	1.53	6.44E-02	ID2	-446992		
chr13	21714473	21714739	1.38	6.46E-02	SAP18	-47		
chr3	111697694	111697958	1.47	6.46E-02	ABHD10	-2		
chr5	157023467	157023799	1.68	6.46E-02	ADAM19	-20865	SOX30	55795
chr7	100157431	100157702	0.65	6.46E-02	SAP25	13874	AGFG2	20733
chr3	42623564	42623874	0.60	6.48E-02	SEC22C	-291		
chrX	53449684	53449960	1.49	6.49E-02	SMC1A	-204		
chr19	19030183	19030567	1.52	6.49E-02	COPE	-176	DDX49	-119
chr10	4178401	4178659	0.57	6.49E-02	AKR1E2	-689872	KLF6	-351057
chr22	43165705	43166029	1.50	6.49E-02	A4GALT	-48991	ARFGAP3	87541
chr3	13493268	13493569	1.49	6.51E-02	NUP210	-31610	HDAC11	-28296
chr14	74238430	74238918	0.63	6.51E-02	PNMA1	-57546	C14orf43	15222
chr11	111944617	111945150	1.62	6.53E-02	TIMM8B	12575	DLAT	49346
chr19	47291683	47291955	1.44	6.54E-02	SLC1A5	23		
chr19	51611335	51611886	1.42	6.56E-02	CTU1	36		
chr9	135817924	135818234	0.77	6.58E-02	C9orf98	-63881	TSC1	1941
chr6	137540453	137540731	1.58	6.60E-02	IFNGR1	-25		
chr15	62637153	62637397	1.61	6.60E-02	TLN2	-302235	C2CD4B	-179793
chr7	105172262	105172943	0.59	6.63E-02	RINT1	71		
chr19	54960131	54960468	1.45	6.65E-02	LENG8	235		
chr19	7294269	7294550	1.25	6.68E-02	INSR	-399		
chr8	145026257	145026542	1.42	6.68E-02	PLEC1	-1356		
chr1	158979475	158979862	1.71	6.69E-02	IFI16	-13		
chr19	49378543	49379073	1.48	6.71E-02	PPP1R15A	3159	TULP2	23188

chr17	4046182	4046601	0.63	6.71E-02	ZZEF1	-139	CYB5D2	-70
chr15	31522984	31523594	1.79	6.71E-02	TRPM1	-129365	KLF13	-95794
chr13	99141022	99141507	1.48	6.71E-02	RNF113B	-311744	STK24	88131
chr6	30582653	30583047	0.72	6.72E-02	MRPS18B	-2636	PPP1R10	2170
chr20	44563766	44564042	1.31	6.75E-02	PCIF1	587		
chr1	212208299	212208720	1.67	6.76E-02	DTL	-409	INTS7	374
chr17	63096425	63096903	0.73	6.76E-02	GNA13	-43744	RGS9	-36885
chr2	43360127	43360426	1.50	6.76E-02	HAAO	-340526	ZFP36L2	93468
chr6	70506692	70507151	0.61	6.77E-02	LMBRD1	-34		
chr10	70360028	70360304	1.43	6.77E-02	CCAR1	-120805	TET1	40049
chr14	106048121	106048712	1.53	6.78E-02	TMEM121	55464	IGHE	281045
chr1	11968177	11968696	1.86	6.78E-02	NPPB	-49445	KIAA2013	18043
chr7	10980174	10980454	0.66	6.79E-02	NDUFA4	-501		
chr10	91403610	91404016	0.62	6.79E-02	SLC16A12	-108500	PANK1	1402
chr14	65753214	65753496	1.54	6.81E-02	MAX	-184128	FUT8	-126180
chr5	135547898	135548144	0.63	6.86E-02	SMAD5	79485	TRPC7	145052
chr7	73482156	73482469	0.68	6.86E-02	LIMK1	-15843	ELN	39886
chr8	91640054	91640338	0.74	6.86E-02	CALB1	-545089	TMEM64	17937
chr15	50736871	50737143	1.56	6.88E-02	USP8	20428	USP50	101895
chr19	3626767	3627221	0.69	6.89E-02	C19orf29	-181		
chr11	120042984	120043260	1.77	6.89E-02	POU2F3	-67829	TRIM29	-34259
chr20	31533646	31534055	0.69	6.93E-02	SPAG4L	58388	EFCAB8	87122
chr17	76238552	76238873	0.62	6.93E-02	LOC283999	11322	SOCS3	117445
chr8	61911668	61912000	0.67	6.93E-02	RLBP1L1	-288691	CHD7	320495
chr16	75498400	75498893	0.65	6.94E-02	TMEM170A	-63		
chr2	182177185	182177658	1.48	7.00E-02	ITGA4	-144197	UBE2E3	332310
chr9	139378169	139378502	1.29	7.02E-02	SEC16A	-829	C9orf163	389
chr2	60666598	60666886	1.62	7.02E-02	BCL11A	113891		
chr1	178577611	178577917	0.88	7.04E-02	RALGPS2	-116536	C1orf49	95552
chr17	48944742	48945157	0.61	7.04E-02	TOB1	-3537		
chr9	893741	894216	1.53	7.07E-02	DMRT3	-82985	DMRT1	52289
chr1	197115759	197116214	0.72	7.07E-02	ASPM	-163		
chr4	120375714	120376012	1.45	7.07E-02	AX746903	-173082	FABP2	-132547
chrX	11381219	11381463	1.46	7.08E-02	AMELX	69808	ARHGAP6	302480
chrX	13105402	13105669	1.78	7.09E-02	FAM9C	-42736	ATXN3L	232982
chr3	187491071	187491347	1.58	7.11E-02	LPP	-439512	BCL6	-27734
chr8	22831249	22831635	0.66	7.13E-02	PEBP4	-46021	RHOBTB2	-21919
chr11	64851434	64851748	1.53	7.14E-02	ZFPL1	-103	CDCA5	24

chr5	90675755	90676126	0.60	7.15E-02	ARRDC3	3208	GPR98	821324
chr19	19144510	19144857	1.53	7.16E-02	SFRS14	-304	ARMC6	213
chr1	87379786	87380228	0.70	7.17E-02	HS2ST1	-328	SEP15	100
chr17	7739136	7739455	0.64	7.18E-02	KDM6B	-3939		
chr1	31971645	31971953	1.56	7.19E-02	TINAGL1	-70287	SERINC2	85836
chr6	30524904	30525274	0.70	7.23E-02	GNL1	-81	PRR3	333
chr8	67419473	67419749	1.51	7.23E-02	ADHFE1	74893	MYBL1	105869
chr1	2516873	2517155	1.52	7.26E-02	C1orf93	-1235		
chr6	43139047	43139294	0.62	7.26E-02	SRF	251		
chr9	82186638	82186986	1.42	7.26E-02	TLE4	-66		
chr13	111124153	111124464	1.65	7.26E-02	RAB20	89762	COL4A2	164678
chr19	16229367	16229719	1.52	7.27E-02	HSH2D	-15295	RAB8A	7053
chr1	33502531	33502876	0.65	7.28E-02	AK2	-212		
chr15	42066278	42066834	0.56	7.28E-02	MAPKBP1	-76		
chr17	79670238	79670593	1.49	7.28E-02	SLC25A10	-8955	HGS	19396
chr2	232180656	232180945	0.77	7.28E-02	B3GNT7	-79534	ARMC9	117459
chr7	107844015	107844405	1.38	7.29E-02	LAMB4	-73409	NRCAM	252616
chr16	67701818	67702148	0.76	7.30E-02	C16orf48	-1355		
chr19	42746830	42747107	1.45	7.31E-02	GSK3A	-233		
chr19	59084677	59085102	1.36	7.32E-02	MZF1	52		
chr14	58894189	58894682	0.54	7.33E-02	TIMM9	-204		
chr11	85339318	85339817	1.58	7.36E-02	DLG2	-1254	TMEM126B	-94
chr7	2681036	2681334	1.45	7.36E-02	AMZ1	-37978	TTYH3	9582
chr5	17278060	17278405	2.04	7.36E-02	BASP1	60483		
chr17	29648554	29649032	0.79	7.36E-02	EVI2A	-76		
chr19	50321205	50321640	1.28	7.36E-02	FUZ	-4955	MED25	-123
chr12	64943308	64943618	0.76	7.39E-02	RASSF3	-60830	TBK1	97526
chr11	119076478	119076754	1.49	7.40E-02	CBL	-374		
chr4	175849374	175849729	1.70	7.41E-02	ADAM29	10043		
chr6	8435677	8436147	0.68	7.41E-02	SLC35B3	-118		
chr5	179398012	179398279	1.81	7.42E-02	TBC1D9B	-63290	RNF130	100963
chr6	24721757	24722184	0.61	7.43E-02	C6orf62	-2568		
chr11	34126921	34127252	1.57	7.44E-02	NAT10	-24		
chr6	32812306	32812649	0.76	7.44E-02	PSMB8	-662		
chr3	112709847	112710269	1.52	7.45E-02	GTPBP8	258		
chr8	11349449	11349920	1.83	7.45E-02	BLK	-1836		
chr12	112820186	112820608	0.72	7.45E-02	C12orf51	-76359	RPL6	27046
chr12	6339951	6340249	1.51	7.46E-02	PLEKHG6	-79502	CD9	30545

chr7	50186268	50186616	0.64	7.48E-02	IKZF1	-157936	ZPBP	-53582
chr5	137688034	137688420	0.69	7.48E-02	KDM3B	-58		
chr11	81580729	81581084	1.77	7.48E-02				
chr1	156389770	156390046	1.65	7.48E-02	C1orf61	9432	RHBG	50905
chr16	3285084	3285334	1.61	7.49E-02	ZNF200	247		
chr2	74259626	74259999	1.39	7.50E-02	TET3	-13637	DGUOK	105860
chr4	190628401	190628645	1.52	7.51E-02	FRG1	-233451		
chr3	14166241	14166610	1.55	7.51E-02	CHCHD4	-55	TMEM43	-14
chr19	45251685	45251991	1.43	7.53E-02	BCL3	-140		
chr6	75994532	75994822	1.51	7.56E-02	TMEM30A	-45		
chr12	129163043	129163317	1.66	7.56E-02	SLC15A4	145361	TMEM132C	263889
chr1	25256733	25257140	0.60	7.57E-02	RUNX3	-167		
chr3	16472722	16473050	0.63	7.58E-02	RFTN1	82336	OXNAD1	166172
chr19	46365831	46366119	1.78	7.59E-02	FOXA3	-1543	SYMPK	573
chr20	52456041	52456400	0.58	7.60E-02	ZNF217	-256514	BCAS1	231083
chr11	67121064	67121516	1.50	7.60E-02	POLD4	-273		
chr11	58912117	58912411	1.53	7.61E-02	FAM111A	12		
chr1	243419189	243419452	1.59	7.62E-02	CEP170	-613	SDCCAG8	1
chr11	66379094	66379404	0.64	7.64E-02	RBM14	-4804		
chr20	40246911	40247243	0.76	7.66E-02	CHD6	56		
chr2	64954200	64954476	1.49	7.66E-02	SLC1A4	-262118	SERTAD2	-73292
chr11	73096158	73096527	1.55	7.67E-02	PLEKHB1	-262251	RELT	8938
chr17	73029866	73030118	1.60	7.68E-02	ATP5H	13082	ICT1	21212
chr7	100209384	100209841	0.62	7.69E-02	MOSPD3	-501		
chr9	34376680	34377186	0.64	7.69E-02	KIAA1161	-39		
chr1	176176675	176176980	1.63	7.71E-02	RFWD2	-458		
chr19	16427485	16427721	0.56	7.71E-02	KLF2	-8048	AP1M1	118938
chr9	93536988	93537310	1.58	7.71E-02	DIRAS2	-132041	SYK	-26863
chr11	71814389	71814744	1.57	7.75E-02	C11orf59	-245		
chr3	71613067	71613403	1.41	7.75E-02	FOXP1	19905		
chr14	23340847	23341157	0.63	7.76E-02	LRP10	42		
chr17	79565503	79565824	1.68	7.76E-02	C17orf70	-46238	NPLOC4	38474
chr2	37193904	37194178	1.30	7.77E-02	STRN	-426		
chr1	148241412	148241731	2.04	7.77E-02	NBPF15	-319275	NBPF14	-215709
chr3	113871354	113871689	1.70	7.78E-02	DRD3	26377	QTRTD1	95911
chr3	101617684	101618010	1.69	7.79E-02	ZPLD1	-536012	NFKBIZ	49489
chr8	6565651	6565969	1.43	7.80E-02	AGPAT5	-68		
chr7	35946084	35946363	0.65	7.82E-02	EEPD1	-246612	SEPT7	105597

chr14	24483221	24483497	1.71	7.82E-02	DHRS4L1	-22351	DHRS4	60394
chr1	146714019	146714355	1.33	7.85E-02	CHD1L	-104		
chr1	202858186	202858430	1.46	7.85E-02	RABIF	-45		
chr9	127703291	127703531	1.44	7.85E-02	GOLGA1	-25		
chr3	57969569	57969852	0.71	7.85E-02	FLNB	-24416	SLMAP	226537
chr18	67872789	67873100	1.50	7.86E-02	RTTN	17		
chr7	27702368	27702803	0.58	7.86E-02	HIBADH	16		
chr3	179322385	179322637	1.54	7.87E-02	MRPL47	-77	NDUFB5	-64
chr20	52556489	52556734	1.46	7.90E-02	ZNF217	-356905	BCAS1	130692
chr5	124238030	124238340	1.47	7.93E-02	ZNF608	-157320		
chr2	37311448	37311726	1.42	7.93E-02	HEATR5B	-102	CCDC75	-7
chr4	186134967	186135280	0.58	7.93E-02	SNX25	3840	LRP2BP	165028
chr7	44499951	44500213	0.73	7.93E-02	CAMK2B	-134852	NPC1L1	80832
chr1	111758808	111759118	0.68	7.94E-02	DRAM2	-76125	CHI3L2	-11318
chr1	1655692	1655986	1.50	7.98E-02	CDC2L1	-64		
chr3	194024154	194024488	0.81	8.02E-02	CPN2	47736	HES1	170387
chr6	33280546	33280853	1.51	8.07E-02	RGL2	-13962	TAPBP	1289
chr14	95961922	95962202	1.59	8.09E-02	GLRX5	-39261	C14orf49	-19889
chr19	55690084	55690394	1.42	8.09E-02	TNNI3	-21139	SYT5	1481
chr9	132751389	132751636	1.52	8.09E-02	FNBP1	53960	USP20	153817
chr7	100459245	100459514	1.55	8.10E-02	TRIP6	-5570	SLC12A9	9022
chr20	47131838	47132172	1.34	8.11E-02	SULF2	-717197	PREX1	312415
chr20	32700010	32700317	1.42	8.11E-02	EIF2S2	-79		
chr19	49894208	49894627	1.51	8.11E-02	CCDC155	2912	PTH2	32280
chr3	105572984	105573299	1.78	8.11E-02	CBLB	14745	ALCAM	487429
chr5	150576195	150576598	0.58	8.11E-02	GM2A	-56216	ANXA6	-39030
chr1	210001132	210001497	1.60	8.12E-02	C1orf107	-18		
chr20	34824194	34824470	1.66	8.14E-02	C20orf4	-115		
chr3	69101394	69101714	1.49	8.15E-02	TMF1	-70		
chr1	9686240	9686611	1.43	8.15E-02	PIK3CD	-25364	TMEM201	37449
chr5	179245969	179246252	1.39	8.19E-02	SQSTM1	-1731		
chr1	974130	974440	0.56	8.19E-02	AGRN	18782	C1orf159	77451
chr19	14685080	14685337	1.39	8.21E-02	NDUFB7	-2323		
chr3	169775894	169776240	1.69	8.22E-02	GPR160	20332	PHC3	123470
chr17	12921291	12921735	1.42	8.22E-02	ELAC2	-154		
chr6	157019449	157019744	1.69	8.24E-02	ARID1B	-79489		
chr5	134240097	134240463	0.65	8.24E-02	PCBD2	-530		
chr9	34171806	34172082	1.60	8.24E-02	DCAF12	-45173	UBAP1	-7067

chr3	101947960	101948236	1.68	8.24E-02	ZPLD1	-205761	NFKBIZ	379740
chr6	26055638	26055938	0.71	8.25E-02	HIST1H1C	911		
chr3	194304586	194304862	1.35	8.25E-02	ATP13A3	-115756	TMEM44	49389
chr2	69135602	69135928	1.55	8.26E-02	BMP10	-37116	GKN2	44337
chr3	186784351	186784808	1.60	8.29E-02	ST6GAL1	44915	RPL39L	72683
chr10	12084894	12085351	0.71	8.29E-02	UPF2	-315		
chr1	228296882	228297126	1.53	8.32E-02	MRPL55	9		
chr2	47168784	47169242	0.74	8.33E-02	TTC7A	700		
chr11	117817714	117818023	1.65	8.33E-02	IL10RA	-39237	TMPRSS13	-17754
chr17	43212696	43213066	1.39	8.35E-02	PLCD3	-2990	ACBD4	-133
chr9	3525769	3526064	0.62	8.37E-02	RFX3	66		
chr7	48031374	48031764	0.58	8.37E-02	HUS1	-12323	SUNC1	37147
chr10	74033431	74033759	0.58	8.39E-02	DDIT4	-82		
chr3	37284558	37284885	1.41	8.40E-02	GOLGA4	-16		
chr11	119544599	119545114	1.61	8.40E-02	THY1	-250611	PVRL1	54578
chr12	3334681	3334937	1.71	8.41E-02	PRMT8	-265616	TSPAN9	148252
chr14	23355940	23356310	0.59	8.41E-02	REM2	3693	RBM23	32271
chr12	12878686	12878935	1.33	8.41E-02	APOLD1	-40		
chr16	85587694	85588048	1.50	8.44E-02	KIAA0182	-59053	KIAA0513	526461
chr15	75335207	75335839	1.63	8.44E-02	PPCDC	19596	DNM1P33	259783
chr8	135844308	135844777	1.44	8.44E-02	KHDRBS3	-625173	ZFAT	-119262
chr2	131099769	131100079	0.68	8.44E-02	IMP4	-565	CCDC115	-2
chr19	40502764	40503077	1.52	8.45E-02	ZNF546	-22		
chr15	85872841	85873204	1.65	8.45E-02	AKAP13	-50848	PDE8A	347818
chr17	66494075	66494426	1.76	8.47E-02	WIPI1	-40598	PRKAR1A	-13859
chr15	66585598	66586103	0.57	8.49E-02	DIS3L	-69		
chr2	3381498	3381832	1.55	8.49E-02	TTC15	-1781	TSSC1	-12
chr1	150669493	150669915	1.51	8.49E-02	GOLPH3L	-32		
chr11	65893630	65893908	1.70	8.49E-02	KLC2	-131405	PACS1	55945
chr7	50307782	50308058	1.62	8.49E-02	ZPBP	-175060	IKZF1	-36458
chr2	182275800	182276110	0.63	8.49E-02	ITGA4	-45664	UBE2E3	430843
chr1	156710863	156711178	1.67	8.51E-02	MRPL24	-98		
chr19	46295934	46296462	0.68	8.51E-02	DMWD	-138		
chr2	25194590	25195185	1.43	8.51E-02	DNAJC27	-64		
chr14	104145722	104146062	0.74	8.51E-02	XRCC3	35931	KLC1	50367
chr19	47551828	47552191	1.33	8.51E-02	TMEM160	-128		
chr3	187456628	187456917	1.62	8.55E-02	RTP2	-36428	BCL6	6702
chr2	99952846	99953128	1.47	8.55E-02	EIF5B	-847	TXNDC9	-127

chr16	87425643	87425909	1.46	8.57E-02	MAP1LC3B	-25		
chr13	41038527	41038776	0.59	8.57E-02	FOXO1	202082	COG6	808888
chr19	48836034	48836624	1.43	8.57E-02	EMP3	7700	TMEM143	30857
chr21	44596572	44597066	1.41	8.57E-02	CRYAA	7678	SIK1	250183
chr19	59010799	59011109	1.45	8.58E-02	SLC27A5	12478	ZNF446	23159
chr8	56685815	56686125	0.63	8.58E-02	TMEM68	-85	TGS1	179
chr12	123849646	123849982	1.31	8.61E-02	SETD8	-18890	SBNO1	-14826
chr11	60128264	60128574	0.51	8.62E-02	MS4A7	-17539	MS4A6E	26064
chr6	47445073	47445487	0.68	8.63E-02	CD2AP	-245		
chr16	20915804	20916114	1.76	8.67E-02	DCUN1D3	-4398	LYRM1	3884
chr1	23670770	23671122	1.40	8.73E-02	HNRNPR	-93		
chr1	36396649	36397010	0.73	8.73E-02	EIF2C3	58		
chr6	32811637	32812018	1.46	8.73E-02	PSMB8	-12		
chr15	40885907	40886560	1.64	8.75E-02	CASC5	-213		
chr3	152094423	152094779	1.94	8.76E-02	P2RY1	-458135	TMEM14E	-35822
chr12	53773075	53773434	0.67	8.77E-02	SP1	-724		
chr11	60244476	60244838	1.56	8.78E-02	MS4A12	-15594	MS4A1	21375
chr13	74541949	74542200	1.68	8.78E-02	KLF12	165991	KLF5	908933
chr8	135612393	135612904	1.64	8.79E-02	ZFAT	112632		
chr19	47164415	47164705	1.11	8.80E-02	GNG8	-26621	PRKD2	55824
chr3	127317109	127317369	1.38	8.89E-02	MCM2	-14		
chr19	47354119	47354491	1.61	8.94E-02	AP2S1	-102		
chr1	16939970	16940439	0.52	8.97E-02	NBPF1	-223		
chr11	61582599	61583121	1.51	8.97E-02	FADS1	1669	FEN1	22710
chr2	47211063	47211674	0.63	8.98E-02	TTC7A	43056	CALM2	192371
chr9	14688314	14688624	0.75	9.02E-02	NFIB	-374524	ZDHHC21	5000
chr11	67195721	67196051	1.49	9.02E-02	RPS6KB2	-49		
chr7	4815025	4815383	0.63	9.02E-02	KIAA0415	-60		
chr8	142046133	142046481	0.76	9.03E-02	PTK2	-34975	SLC45A4	192366
chr1	1072116	1072380	0.49	9.06E-02	TTLL10	-37038	C1orf159	-20512
chr11	117924134	117924418	1.59	9.07E-02	TMPRSS4	-23451	IL10RA	67170
chr15	55977489	55977799	1.71	9.09E-02	PYGO1	-96594	PRTG	57533
chr9	101946762	101947191	1.50	9.09E-02	ALG2	37269	TGFBR1	79565
chr11	66112496	66112769	1.45	9.09E-02	BRMS1	-51		
chr8	38571855	38572107	0.76	9.10E-02	RNF5	-113206	TACC1	-72741
chr19	19517802	19518056	0.77	9.10E-02	GATAD2A	21287	TSSK6	108540
chr3	187694135	187694445	0.75	9.11E-02	LPP	-236431	BCL6	-230815
chr1	156426348	156426675	1.56	9.13E-02	C1orf61	-27172	MEF2D	44017

chr8	61821344	61821695	0.68	9.15E-02	RLBP1L1	-379005	CHD7	230181
chr8	87520852	87521124	0.62	9.17E-02	FAM82B	21		
chr22	22337050	22337417	1.69	9.18E-02	TOP3B	-87		
chr12	102333641	102333958	0.74	9.21E-02	DRAM1	62695	CCDC53	122099
chr15	44084070	44084314	1.34	9.21E-02	ELL3	-14690	SERINC4	8063
chr3	151986341	151987002	0.60	9.21E-02	MBNL1	-30522	SUCNR1	395235
chr2	32390708	32391055	1.61	9.22E-02	SLC30A6	-51		
chr17	56709343	56709616	1.41	9.23E-02	SEPT4	-102817	TEX14	59936
chr7	1543950	1544206	0.62	9.24E-02	INTS1	-60		
chr11	118306965	118307250	1.48	9.26E-02	MLL	-97		
chr2	3605858	3606161	1.45	9.29E-02	RNASEH1	-70		
chr15	75069750	75070137	1.58	9.31E-02	CSK	-4481		
chr3	174991883	174992254	1.74	9.31E-02	NAALADL2	414958		
chr17	8076590	8076988	1.89	9.31E-02	VAMP2	-10496	TMEM107	2925
chr16	66785504	66785814	0.67	9.32E-02	DYNC1LI2	-134		
chr12	16351801	16352171	1.48	9.32E-02	MGST1	-148593	DERA	287800
chr17	40761321	40761583	1.36	9.34E-02	FAM134C	-7	TUBG1	94
chr20	30135012	30135311	1.48	9.35E-02	ID1	-57930	HM13	32921
chr11	64781272	64782098	0.66	9.35E-02	ARL2	99		
chr3	128444748	128445043	1.48	9.36E-02	RAB7A	-83		
chr12	9860562	9861061	0.66	9.38E-02	CLECL1	25048	CLEC2D	38503
chr9	9612254	9612646	1.35	9.38E-02				
chr8	73920723	73921270	0.70	9.41E-02	TERF1	-100		
chr7	107383849	107384160	0.66	9.42E-02	CBLL1	-274		
chr20	30956783	30957186	1.56	9.42E-02	ASXL1	10832	COMMD7	374829
chr14	20895906	20896240	1.77	9.42E-02	TEP1	-14494	KLHL33	2521
chr4	8430096	8430349	1.32	9.43E-02	ACOX3	12229	HTRA3	158731
chr9	114837064	114837320	1.76	9.43E-02	SUSD1	100364	UGCG	177986
chr22	40831901	40832327	1.65	9.44E-02	SGSM3	65519	MKL1	200576
chr1	154600332	154600651	1.36	9.44E-02	ADAR	-19810	KCNN3	242262
chr19	14714885	14715140	1.70	9.44E-02	CLEC17A	21117	EMR3	70717
chr2	24270249	24270524	1.32	9.44E-02	FKBP1B	-2197	C2orf44	-91
chr9	102581566	102582002	1.43	9.44E-02	NR4A3	-7225	SEC61B	597214
chr15	31750031	31750356	1.44	9.45E-02	KLF13	131111	OTUD7A	197348
chr13	73356121	73356541	1.39	9.45E-02	DIS3	13		
chr12	25158187	25158691	1.55	9.47E-02	BCAT1	-56131	LRMP	-46802
chr20	19299501	19299747	0.61	9.47E-02	RIN2	-570586	SLC24A3	106334
chr19	6577346	6578175	1.75	9.48E-02	CD70	13402	TNFSF9	46751

chr6	44281001	44281311	0.69	9.49E-02	AARS2	-93		
chr22	23484050	23484409	0.69	9.49E-02	RAB36	-3283	RTDR1	11
chr8	28243630	28244081	1.15	9.51E-02	ZNF395	121		
chr9	137822290	137822667	1.66	9.51E-02	OLFM1	-144610	FCN1	-12670
chr7	73668584	73669009	1.29	9.52E-02	RFC2	-59		
chr2	68290040	68290289	1.50	9.52E-02	C1D	-6		
chr2	153573834	153574352	0.77	9.53E-02	ARL6IP6	-330	PRPF40A	-118
chr11	34072887	34073197	0.64	9.53E-02	CAPRIN1	-188		
chr1	246729415	246729840	1.56	9.53E-02	TFB2M	-63	C1orf71	-11
chr1	228353319	228353598	1.46	9.54E-02	C1orf69	30		
chr17	79818719	79819012	1.35	9.54E-02	P4HB	-322		
chr1	4404109	4404419	0.71	9.54E-02	AJAP1	-310841	DFFB	630419
chr17	75461689	75462106	0.71	9.56E-02	TNRC6C	-538420	SEPT9	184406
chr6	30650322	30650643	0.68	9.56E-02	DHX16	-9653	KIAA1949	4610
chr12	110905938	110906455	1.53	9.59E-02	GPN3	-133		
chr19	42747231	42747528	1.48	9.60E-02	GSK3A	-644		
chr2	60581016	60581387	1.57	9.63E-02	BCL11A	199431		
chr7	20256944	20257212	0.63	9.64E-02	MACC1	-65		
chr16	2802193	2802437	1.56	9.65E-02	SRRM2	-15		
chr19	10330391	10330698	0.67	9.65E-02	DNMT1	-24790	S1PR2	11403
chr13	31017999	31018312	0.64	9.66E-02	KATNAL1	-136993	HMGB1	21925
chr17	62103174	62103418	0.68	9.73E-02	ICAM2	-5302	ERN1	104206
chr2	32502681	32503140	1.57	9.74E-02	YIPF4	-47		
chr21	36237925	36238538	1.50	9.74E-02	C21orf96	173491	CLIC6	196544
chr4	8194484	8194827	1.45	9.74E-02	ABLIM2	-34097	SH3TC1	-6404
chr1	45275198	45275527	1.55	9.74E-02	BTBD19	1209	PTCH2	33253
chr6	27636286	27636530	1.86	9.74E-02	ZNF184	-195511	HIST1H2BL	139301
chr1	155210962	155211227	1.53	9.76E-02	GBA	-42		
chr6	26055994	26056247	0.77	9.76E-02	HIST1H1C	578		
chr5	81574148	81574458	0.74	9.77E-02	RPS23	-68		
chr11	65275015	65275301	1.62	9.81E-02	SCYL1	-17390	FRMD8	121117
chr18	2972197	2972457	1.60	9.82E-02	LOC727896	-25706	LPIN2	39618
chr1	1590403	1590645	1.25	9.83E-02	MMP23B	22964	LOC728661	33719
chr15	83513510	83513867	0.62	9.83E-02	WHAMM	35716	HOMER2	107784
chr20	25677404	25677666	1.45	9.83E-02	ZNF337	-66		
chr3	45730195	45730690	1.58	9.83E-02	SACM1L	-311		
chr3	169531554	169531896	1.57	9.83E-02	LRRC34	-1413		
chr13	26760229	26760537	1.53	9.83E-02	SHISA2	-135185	RNF6	35595

chr1	145208845	145209212	1.47	9.83E-02	NOTCH2NL	-82		
chr4	177116547	177116900	1.34	9.84E-02	SPATA4	98		
chr1	161155080	161155375	0.74	9.84E-02	B4GALT3	-7914	ADAMTS4	13617
chr5	56471451	56471716	2.11	9.85E-02	MIER3	-223630	GPBP1	-38317
chr9	130981050	130981297	1.25	9.88E-02	DNM1	15511	GOLGA2	57094
chr4	74454957	74455352	1.52	9.90E-02	RASSF6	31185	AFM	107693
chr20	49547800	49548227	0.67	9.92E-02	ADNP	-487		
chr12	14436384	14436725	1.76	9.92E-02	GRIN2B	-303533	ATF7IP	-82056
chr3	67048400	67048665	1.44	9.97E-02	KBTBD8	-194		
chr17	80407120	80407707	1.78	9.97E-02	C17orf101	-30952	C17orf62	1280
chr17	57439749	57440025	1.46	9.97E-02	DHX40	-202999	YPEL2	30834
chr10	98478067	98478399	0.50	9.99E-02	TM9SF3	-131424	PIK3AP1	2046
chr3	185216519	185217017	1.46	1.00E-01	TMEM41A	77		
chr19	45640535	45640782	1.53	1.00E-01	NKPD1	22749	GEMIN7	58141
chrX	153626429	153626895	1.45	1.00E-01	RPL10	91		
chr1	42800905	42801366	0.66	1.00E-01	FOXJ3	-233		
chr13	28051436	28051860	1.48	1.00E-01	MTIF3	-26937	LNX2	143072
chr5	180600566	180600842	1.52	1.00E-01	OR2V2	18761	TRIM7	31473
chr16	11836694	11837151	1.31	1.00E-01	TXNDC11	-275		
chr11	118122663	118123183	1.65	1.01E-01	MPZL3	88		
chr15	68502196	68502506	0.62	1.01E-01	CALML4	-3903		
chr17	74268154	74268669	1.65	1.01E-01	RNF157	-32022	QRICH2	35349
chr2	111814735	111815045	0.75	1.01E-01	BCL2L11	-63601	ACOXL	324740
chr7	72722729	72723066	0.62	1.01E-01	NSUN5	-75		
chr1	28832145	28832665	1.65	1.01E-01	RCC1	-12340	PHACTR4	136312
chr11	118888793	118889435	1.55	1.01E-01	TRAPPC4	-127	RPS25	-57
chr16	84627730	84628222	1.68	1.01E-01	KIAA1609	-89688	COTL1	23693
chr20	20032993	20033416	1.31	1.01E-01	CRNKL1	3485	NAT5	35268
chr7	25902045	25902399	1.32	1.01E-01	NPVF	-634117	NFE2L3	-289625
chr15	51199169	51199517	1.46	1.01E-01	AP4E1	-1603		
chr16	2318022	2318501	1.44	1.01E-01	RNPS1	-465		
chr2	38834920	38835236	1.52	1.02E-01	HNRPLL	-4900		
chr19	12792620	12792886	1.46	1.02E-01	DHPS	-76		
chr5	271451	271709	1.39	1.02E-01	PDCD6	-156		
chr9	35618290	35618612	1.45	1.02E-01	CD72	-27		
chr4	114683730	114684135	1.42	1.02E-01	CAMK2D	-850		
chr19	47852370	47852668	1.33	1.02E-01	DHX34	-27		
chr17	29035235	29035537	1.21	1.02E-01	LRRC37B2	99938	CRLF3	116392

chr3	187810509	187810889	1.27	1.02E-01	BCL6	-347224	LPP	-120022		
chr4	141805636	141805903	1.47	1.02E-01	TBC1D9	-128299	RNF150	248846		
chr7	64940700	64941058	0.60	1.03E-01	VKORC1L1	-397378	ZNF92	102111		
chr1	150185850	150186395	1.44	1.03E-01	ANP32E	22381	PLEKHO1	63953		
chr1	236135118	236135402	1.63	1.03E-01	LYST	-105040	NID1	93221		
chr18	43266812	43267106	0.76	1.03E-01	SLC14A1	-37133	SLC14A2	72193		
chr7	87505670	87505967	0.69	1.03E-01	SLC25A40	-151	DBF4	275		
chr2	106342331	106342685	2.19	1.03E-01	FHL2	-326827	NCK2	-125797		
chr5	95194448	95194724	1.64	1.03E-01	GLRX	-36009	ELL2	103189		
chr8	135724785	135725058	1.52	1.03E-01	ZFAT	359				
chr8	103823087	103823422	0.70	1.03E-01	KLF10	-155272	AZIN1	53142		
chr19	14116970	14117280	0.68	1.03E-01	RFX1	9				
chr2	152628102	152628402	1.48	1.03E-01	NEB	-37263	ARL5A	56757		
chr3	48754571	48754881	0.62	1.03E-01	DKFZp564L0678	194	IP6K2	194		
chr4	76870299	76870568	1.64	1.03E-01	NAAA	-8268	SDAD1	41679		
chr8	126446920	126447196	1.37	1.03E-01	TRIB1	4495				
chrX	12968276	12968556	1.54	1.04E-01	TMSB4X	-24809	TLR8	43658		
chr13	39612048	39612486	0.68	1.04E-01	NHLRC3	-188				
chr2	179387689	179388029	1.56	1.04E-01	PLEKHA3	42660	TTN	284291		
chr7	99942472	99942731	1.43	1.04E-01	PILRB	-13024	PVRIG	125730		
chr16	3124945	3125189	0.61	1.04E-01	IL32	9754	ZSCAN10	17794		
chr3	133185823	133186347	0.70	1.04E-01	CDV3	-106349	BFSP2	67295		
chr21	43430005	43430437	1.62	1.05E-01	ZNF295	275				
chr17	41393077	41393458	1.51	1.05E-01	ARL4D	-83085	TMEM106A	29374		
chr1	42194422	42194790	1.48	1.05E-01	EDN2	-244262	HIVEP3	189890		
chr13	50656111	50656420	1.49	1.05E-01	ST13	-89888	KCNRG	66876		
chr6	10460392	10460682	0.75	1.05E-01	GCNT2	-125456	TFAP2A	-45067		
chr20	44519753	44520107	1.46	1.05E-01	C20orf165	-3692	NEURL2	-29	CTSA	339
chr12	112452386	112452660	1.48	1.05E-01	TMEM116	-1588	ERP29	1371		
chr19	39348076	39348392	1.51	1.05E-01	HNRNPL	-7617	RINL	20660		
chr19	13001634	13001986	0.71	1.06E-01	KLF1	-3793	GCDH	-164		
chr16	1470660	1471175	1.49	1.06E-01	UNKL	-6218	C16orf91	8427		
chr6	31036663	31036921	0.56	1.06E-01	C6orf15	43540	MUC21	85307		
chr11	6256266	6256577	0.61	1.06E-01	CNGA4	-3908	FAM160A2	-481		
chr11	65278270	65278514	0.70	1.06E-01	SCYL1	-14156	FRMD8	124351		
chr4	76912025	76912348	1.38	1.06E-01	SDAD1	-74				
chr12	57636516	57636936	0.73	1.06E-01	STAC3	8250	SHMT2	13174		
chr14	58666532	58666868	0.59	1.06E-01	ACTR10	-133				

chr6	79577163	79577778	0.74	1.06E-01	IRAK1BP1	282		
chr11	111234790	111235029	0.97	1.06E-01	C11orf92	-64371	POU2AF1	15247
chr11	67253323	67253633	0.69	1.07E-01	AIP	2973	PITPNM1	19365
chr7	1063942	1064245	0.61	1.07E-01	GPR146	-33047	CYP2W1	41259
chr1	243418592	243418919	1.60	1.07E-01	SDCCAG8	-564	CEP170	-48
chr13	77903232	77903624	1.58	1.07E-01	MYCBP2	-2251		
chr7	99818881	99819225	0.65	1.07E-01	PILRB	-136573	PVRIG	2181
chr12	12966096	12966470	1.44	1.07E-01	DDX47	142		
chr11	108535416	108535885	1.38	1.07E-01	DDX10	-165		
chr2	100867151	100867486	1.33	1.07E-01	AFF3	-145274	LONRF2	71876
chr1	156308143	156308568	1.53	1.07E-01	CCT3	-161		
chr1	198650948	198651272	1.69	1.07E-01	PTPRC	42973		
chr1	203274217	203274497	1.47	1.08E-01	BTG2	-307		
chr9	4792488	4792853	0.60	1.08E-01	RCL1	-163		
chr17	27065685	27066034	1.53	1.08E-01	TRAF4	-5163	NEK8	10028
chr16	27415829	27416139	0.71	1.08E-01	IL21R	-22595	IL4R	90733
chr11	76155808	76156188	1.50	1.09E-01	C11orf30	-71		
chr12	109027450	109027797	1.48	1.09E-01	SELPLG	46		
chr15	65133537	65134006	1.36	1.09E-01	ANKDD1A	-70329	PIF1	-15934
chr9	127420509	127420838	1.33	1.09E-01	NR5A1	-150975	NR6A1	112902
chr2	65610998	65611246	0.80	1.09E-01	SPRED2	48534	ACTR2	156293
chr7	139616290	139616581	1.29	1.09E-01	TBXAS1	87413	PARP12	147085
chr2	70313444	70313754	0.69	1.09E-01	PCBP1	-986		
chr7	99686388	99686664	0.71	1.09E-01	COPS6	-57		
chr3	187956872	187957257	0.69	1.09E-01	TPRG1	-932698	LPP	26344
chr12	30848656	30849088	1.38	1.09E-01	IPO8	-121		
chr14	106328224	106328600	1.58	1.09E-01	IGHE	1050	TMEM121	335459
chr15	40397383	40397627	1.49	1.10E-01	SRP14	-66116	BMF	3570
chr5	177540315	177540693	1.23	1.10E-01	N4BP3	-52		
chr12	96608962	96609337	1.48	1.10E-01	ELK3	20943	PCTK2	185073
chr20	32366829	32367078	0.64	1.10E-01	CHMP4B	-32165	ZNF341	47146
chr4	148787996	148788254	1.71	1.10E-01	ARHGAP10	134672	NR3C2	575518
chr19	13278440	13278765	0.71	1.10E-01	IER2	17321	CACNA1A	338671
chr15	52758355	52758697	1.45	1.11E-01	MYO5C	-170556	MYO5A	62721
chr20	47894975	47895399	1.60	1.11E-01	ZNFX1	-431		
chr1	11866040	11866343	1.42	1.11E-01	MTHFR	-77	CLCN6	-15
chr14	68734895	68735223	1.57	1.11E-01	RAD51L1	448550	ZFP36L1	524726
chr1	61542507	61542994	0.62	1.11E-01	NFIA	-4783		

chr17	56582305	56582668	1.63	1.11E-01	HSF5	-16728	MTMR4	12764
chr8	81209269	81209649	0.65	1.12E-01	TPD52	-216449	ZBTB10	-188989
chr11	2407130	2407395	0.60	1.12E-01	CD81	8716	TRPM5	37012
chr8	103543024	103543347	0.62	1.12E-01	UBR5	-118691	ODF1	-20662
chr7	29961099	29961368	0.64	1.12E-01	SCRN1	68671	WIPF3	86893
chr1	155278218	155278768	1.44	1.12E-01	FDPS	-46		
chr1	45265809	45266279	1.29	1.12E-01	PLK3	8		
chr7	100781840	100782086	1.44	1.12E-01	AP1S1	-15723	SERPINE1	11584
chr10	75541594	75541917	0.73	1.12E-01	KIAA0913	-3849	CHCHD1	-52
chr1	183447198	183447442	0.63	1.12E-01	SMG7	5686	NCF2	112726
chr12	32059701	32060107	0.73	1.13E-01	BICD1	-200281	H3F3C	-114729
chr7	44646447	44646918	0.71	1.13E-01	OGDH	512		
chr1	194258684	194259087	1.61	1.13E-01				
chr16	2301542	2301787	1.43	1.13E-01	DCI	-62		
chr19	40023409	40023725	1.35	1.14E-01	EID2B	-73		
chr6	31830570	31830894	0.65	1.14E-01	NEU1	-23		
chr17	73660061	73660433	1.61	1.14E-01	SAP30BP	-3152	RECQL5	3022
chr4	57843674	57843931	1.59	1.14E-01	POLR2B	-1306	C4orf14	23
chr11	128341074	128341540	1.49	1.14E-01	ETS1	116146		
chr16	56985321	56985747	1.45	1.14E-01	CETP	-10301	HERPUD1	19786
chr17	56407559	56407901	1.48	1.14E-01	BZRAP1	-1578		
chr11	67275370	67275882	0.67	1.14E-01	PITPNM1	-2783	CDK2AP2	476
chr12	57632913	57633177	0.68	1.14E-01	SHMT2	9493	STAC3	11931
chr3	121721943	121722368	1.52	1.14E-01	ILDR1	18874	SLC15A2	108985
chr14	102771417	102771661	1.41	1.14E-01	RAGE	-8		
chr10	105881687	105882184	0.71	1.14E-01	GSTO1	-132742	COL17A1	-36298
chr5	99870848	99871133	1.30	1.14E-01	FAM174A	-133		
chr6	32982600	32982844	0.76	1.15E-01	HLA-DOA	-5333	HLA-DPA1	58827
chr12	1193089	1193325	0.71	1.16E-01	ERC1	92803	FBXL14	510124
chr5	96270733	96271159	1.38	1.16E-01	LNPEP	-23210	ERAP2	59302
chr2	190305661	190305952	1.43	1.16E-01	WDR75	-352		
chr7	43622294	43622604	0.70	1.16E-01	STK17A	-243		
chr6	36630138	36630448	0.63	1.16E-01	CDKN1A	-16166	SFRS3	68203
chr15	85293791	85294175	0.82	1.17E-01	ALPK3	-65928	ZNF592	2165
chr19	10541861	10542180	1.34	1.17E-01	PDE4A	10688	KEAP1	72033
chr16	734250	734596	1.42	1.17E-01	STUB1	4308	FBXL16	21385
chr9	95432383	95432789	1.39	1.17E-01	IPPK	-39		
chr2	240323017	240323484	1.32	1.17E-01	HDAC4	-608		

chr15	85197274	85197606	0.69	1.18E-01	NMB	4362	SCAND2	22749
chr9	130217783	130218215	0.85	1.18E-01	RPL12	-4315	LRSAM1	4207
chr2	157192103	157192361	1.58	1.18E-01	NR4A2	-2945		
chr20	18447789	18448133	0.75	1.18E-01	C20orf12	-132	POLR3F	-72
chr1	200589927	200590203	1.40	1.18E-01	KIF14	-203		
chr18	42683363	42683619	1.51	1.19E-01	SLC14A2	-511275	SETBP1	422628
chr22	28315142	28315508	1.36	1.19E-01	PITPNB	-70		
chr7	54826821	54827125	1.52	1.19E-01	SEC61G	-34		
chr7	35767311	35767566	0.65	1.19E-01	SEPT7	-73188	HERPUD2	-32667
chr16	30197145	30197468	0.79	1.19E-01	GIYD2	-7910	CORO1A	2381
chr12	109531222	109531544	1.50	1.19E-01	UNG	-4032	ALKBH2	-90
chr4	140216827	140217137	1.52	1.19E-01	NDUFC1	132		
chr13	47371203	47371629	1.45	1.19E-01	ESD	-49		
chr7	99613006	99613316	0.70	1.19E-01	ZKSCAN1	-58		
chr6	31527718	31528067	0.70	1.19E-01	LTA	-11983	NFKBIL1	12540
chr7	45040763	45041358	0.61	1.19E-01	CCM2	1274	NACAD	87432
chr7	128084422	128084751	1.31	1.20E-01	IMPDH1	-34551	C7orf68	-11297
chr9	130890472	130890782	0.77	1.20E-01	LOC389791	-181	PTGES2	-153
chr7	50346905	50347215	0.72	1.20E-01	IKZF1	2682	FIGNL1	171028
chr5	150635484	150635805	0.68	1.20E-01	GM2A	3032	SLC36A3	47689
chr19	18811278	18811539	1.41	1.20E-01	CRTC1	16984	COMP	90705
chr10	14690220	14690526	0.70	1.20E-01	FRMD4A	-317507	CDNF	189610
chr10	97517674	97518013	1.39	1.20E-01	CCNJ	-285315	ENTPD1	46308
chr12	68151976	68152234	0.67	1.20E-01	DYRK2	109593	IFNG	401416
chr15	91593067	91593334	1.91	1.20E-01	SV2B	-175902	VPS33B	-27368
chr7	23145127	23145720	0.58	1.20E-01	KLHL7	71		
chr8	119291731	119292025	1.51	1.20E-01	EXT1	-167820	TNFRSF11B	672505
chr2	170655247	170655557	0.84	1.21E-01	SSB	13		
chr11	102187840	102188506	1.70	1.21E-01	BIRC3	-21		
chr5	81045434	81045744	0.71	1.21E-01	ACOT12	-355601	SSBP2	1483
chr14	56662463	56662749	1.48	1.21E-01	C14orf101	-383905	PELI2	77513
chr19	49217597	49217884	1.28	1.21E-01	MAMSTR	5235	FUT2	18513
chr2	113931262	113931703	0.65	1.21E-01	PSD4	-77		
chr14	102305582	102305858	1.60	1.22E-01	DYNC1H1	-125145	PPP2R5C	77585
chr4	140004027	140004286	1.52	1.22E-01	ELF2	56494	CCRN4L	67214
chr3	13152143	13152425	1.37	1.22E-01	IQSEC1	-37667	NUP210	309525
chr4	128886280	128886524	1.37	1.22E-01	C4orf29	-59	MFSD8	737
chr22	24236370	24236729	1.51	1.22E-01	MIF	-15		

chr17	65809358	65809657	0.67	1.22E-01	BPTF	-12272	NOL11	95447
chr8	47868637	47868946	0.60	1.22E-01	CEBPD	781934		
chr16	29674861	29675171	0.61	1.23E-01	SPN	716		
chr7	12250744	12250987	0.67	1.23E-01	TMEM106B	18		
chr12	15942299	15942575	1.26	1.23E-01	EPS8	73		
chr17	7210725	7210987	1.42	1.23E-01	EIF5A	538		
chr3	113775209	113775797	1.52	1.23E-01	QTRTD1	-108		
chr11	65430262	65430836	1.51	1.23E-01	RELA	-106		
chr2	192540508	192540881	1.50	1.23E-01	OBFC2A	-2166		
chr3	169482818	169483179	1.56	1.24E-01	MECOM	-101436	ARPM1	4684
chr9	140149593	140149857	1.41	1.24E-01	COBRA1	-34		
chr3	10157196	10157440	1.32	1.24E-01	C3orf10	-15		
chr8	28195982	28196278	1.54	1.24E-01	PNOC	21481	ZNF395	47847
chr7	75272270	75272529	0.66	1.24E-01	PMS2L3	-114947	HIP1	95879
chr1	167468994	167469274	1.45	1.24E-01	CD247	18713	POU2F1	278991
chr2	128577670	128577982	0.58	1.24E-01	WDR33	-9081	POLR2D	37903
chr1	37349812	37350122	0.63	1.25E-01	CSF3R	-401458	GRIK3	149877
chr4	140187915	140188194	1.47	1.25E-01	ELF2	-127404	C4orf49	13437
chr19	12251896	12252243	0.65	1.25E-01	ZNF20	-930		
chr19	59066420	59066673	1.26	1.25E-01	CHMP2A	-61		
chr19	56110931	56111497	1.37	1.25E-01	ZNF524	-516	FIZ1	-321
chr6	11144641	11144895	0.71	1.25E-01	HERV-FRD	-32809	NEDD9	88147
chr15	69744736	69745203	0.77	1.25E-01	RPLP1	-189		
chr19	36422441	36422859	1.48	1.25E-01	TYROBP	-23464	LRFN3	-5372
chr1	9488888	9489180	1.35	1.26E-01	SLC25A33	-110494	SPSB1	136093
chr19	42636342	42636787	0.73	1.26E-01	POU2F2	65		
chr19	5680536	5680793	1.36	1.26E-01	HSD11B1L	-370	C19orf70	246
chr11	65990162	65990472	1.47	1.26E-01	KLC2	-34857	PACS1	152493
chr22	39898179	39898468	1.54	1.26E-01	SMCR7L	40		
chr19	54694082	54694426	1.25	1.26E-01	TSEN34	-850	MBOAT7	-521
chr19	8258824	8259134	0.83	1.26E-01	FBN3	-46598	LASS4	-15238
chr6	31164756	31165066	0.72	1.26E-01	POU5F1	-26460	HLA-C	74944
chr15	40390971	40391336	1.54	1.26E-01	SRP14	-59765	BMF	9921
chr6	33560293	33560615	0.72	1.26E-01	ITPR3	-28707	BAK1	-12384
chr6	57181504	57181755	0.73	1.27E-01	PRIM2	-792		
chr7	1126159	1126524	0.70	1.27E-01	GPER	-1381		
chr3	44803030	44803307	1.47	1.27E-01	KIF15	-40		
chr11	118271756	118272293	0.76	1.27E-01	ATP5L	-79		

chr5	96152120	96152430	0.88	1.28E-01	ERAP2	-59369	ERAP1	-8383		
chr4	78783698	78784164	1.50	1.28E-01	MRPL1	126				
chr5	139646397	139646857	1.41	1.28E-01	PFDN1	36062	C5orf53	141106		
chr6	474850	475110	1.52	1.29E-01	IRF4	83228	EXOC2	218129		
chr1	185126007	185126459	1.42	1.29E-01	C1orf25	-117				
chr19	55770540	55770893	1.43	1.29E-01	SAPS1	-679				
chr7	1550462	1550702	1.31	1.29E-01	MAFK	-19786	INTS1	-6564		
chr4	300359	300740	1.50	1.29E-01	ZNF141	-31046	ZNF732	-10606		
chr7	99717133	99717851	0.58	1.29E-01	TAF6	-513	CNPY4	227		
chr10	80828486	80828862	1.08	1.29E-01	ZMIZ1	-118				
chr19	47729463	47729952	1.33	1.29E-01	BBC3	6315	SAE1	95628		
chr6	27860729	27861156	1.42	1.30E-01	HIST1H3J	-2373	HIST1H2BO	-260	HIST1H2AM	20
chr20	44509699	44509976	1.49	1.30E-01	ZSWIM1	-10				
chr1	32782149	32782395	0.58	1.30E-01	MARCKSL1	19562	HDAC1	24564		
chr11	62358960	62359344	1.64	1.30E-01	TUT1	-43				
chr7	44121893	44122146	0.66	1.30E-01	POLM	109				
chr9	100684420	100684692	1.51	1.30E-01	C9orf156	296				
chr2	45878091	45878401	1.36	1.30E-01	PRKCE	-797				
chr3	194992100	194992602	0.63	1.30E-01	C3orf21	-456				
chr7	5553201	5553600	0.65	1.30E-01	FBXL18	-2				
chr12	56040006	56040260	1.36	1.30E-01	METTL7B	-35197	OR10P1	9457		
chr19	47759637	47759949	1.36	1.30E-01	BBC3	-23770	PRR24	-18349		
chr11	63334613	63335056	1.49	1.30E-01	RARRES3	30562	PLA2G16	47106		
chr7	128502682	128502973	1.48	1.31E-01	ATP6V1F	-70				
chr6	4018797	4019419	0.68	1.31E-01	PRPF4B	-2461				
chr11	59318419	59318858	1.46	1.32E-01	OR4D9	36253	OSBP	64978		
chr19	46405859	46406259	1.28	1.32E-01	MYPOP	-197				
chr5	180615830	180616084	1.51	1.32E-01	TRIM7	16220	OR2V2	34014		
chr1	153918484	153918794	1.50	1.32E-01	GATAD2B	-23188	CRTC2	12404		
chr19	17887362	17887637	1.44	1.32E-01	B3GNT3	-18419	MAP1S	57197		
chr6	26595997	26596307	0.71	1.33E-01	ABT1	-1028				
chr19	49137687	49137940	1.29	1.33E-01	Sec1	-3482	DBP	2825		
chr19	46234062	46234354	1.28	1.33E-01	FBXO46	-57				
chr6	42694312	42694622	0.74	1.33E-01	PRPH2	-4109				
chr5	61708433	61708681	1.40	1.33E-01	DIMT1L	-8829	IPO11	-6154		
chr2	68942555	68942847	1.50	1.33E-01	ARHGAP25	-19267	PROKR1	69907		
chr16	31471141	31471411	1.33	1.34E-01	ARMC5	959				
chr13	45694446	45694715	1.46	1.34E-01	GTF2F2	-50				

chr8	102217932	102218233	0.55	1.34E-01	ZNF706	-123		
chr3	176915594	176916077	1.44	1.35E-01	TBL1XR1	-788		
chr11	62420703	62421013	1.69	1.35E-01	INTS5	-84		
chr4	54614528	54614941	1.39	1.35E-01	LNX1	-157011	CHIC2	316053
chr11	102158764	102159011	1.91	1.35E-01	BIRC3	-29306	YAP1	177678
chr15	101739882	101740241	1.67	1.35E-01	CHSY1	52064	LRRK1	280602
chr8	118452006	118452451	0.63	1.35E-01	MED30	-80736	SLC30A8	304892
chr14	65879100	65879508	0.71	1.35E-01	FUT8	-231		
chr17	17942380	17942757	1.32	1.35E-01	ATPAF2	-89		
chr6	24774881	24775377	0.65	1.35E-01	GMNN	-35		
chrX	153990784	153991110	1.35	1.35E-01	DKC1	-84		
chr12	125399038	125399664	1.43	1.35E-01	UBC	226		
chr21	43954557	43954954	0.75	1.36E-01	PDE9A	-119106	SLC37A1	35014
chr9	124921853	124922211	0.78	1.36E-01	NDUFA8	66		
chr1	218458403	218458748	1.45	1.36E-01	RRP15	-53		
chr19	1241421	1241831	1.43	1.36E-01	C19orf26	-3636	ATP5D	-123
chr9	136283052	136283441	1.38	1.36E-01	ADAMTS13	-3873	REXO4	-83
chr2	46796345	46796764	1.44	1.36E-01	RHOQ	26688	PIGF	47696
chr7	23412860	23413666	0.56	1.37E-01	IGF2BP3	96732	GPNMB	126947
chr9	102582040	102582395	1.17	1.37E-01	NR4A3	-6791	SEC61B	597648
chr6	14095697	14095968	1.48	1.38E-01	CD83	-22032	RNF182	170630
chr6	30646774	30647051	0.63	1.38E-01	DHX16	-6083	KIAA1949	8180
chr11	65029128	65029446	1.48	1.38E-01	POLA2	-145		
chr12	114404035	114404325	1.38	1.38E-01	RBM19	-4		
chr2	73460346	73460596	1.18	1.38E-01	CCT7	-934	C2orf7	-115
chr9	114798094	114798387	1.38	1.38E-01	UGCG	139035	SUSD1	139315
chr7	130792751	130793010	1.28	1.39E-01	KLF14	-374021	MKLN1	-219714
chr20	62259697	62259976	1.52	1.39E-01	GMEB2	-1456		
chr6	27834338	27834738	0.71	1.39E-01	HIST1H1B	821		
chr20	39640239	39640697	1.55	1.39E-01	MAFB	-322592	TOP1	-16994
chr3	32509299	32509599	1.37	1.40E-01	CMTM6	34954	CMTM7	76286
chr6	33281638	33281993	0.77	1.40E-01	TAPBP	173		
chr13	46756134	46756453	1.54	1.40E-01	LCP1	165		
chr20	23338631	23338892	1.30	1.40E-01	GZF1	-6238	NXT1	7389
chr9	139010439	139010949	1.35	1.40E-01	NACC2	-23563	LHX3	84310
chr7	152133751	152134027	1.18	1.41E-01	MLL3	-799		
chr4	787934	788370	0.60	1.41E-01	CPLX1	31793	PCGF3	88579
chr21	45626630	45626991	0.79	1.41E-01	ICOSLG	34023	C21orf33	73317

chr2	232092968	232093296	0.69	1.41E-01	B3GNT7	-167203	ARMC9	29790
chr1	145058991	145059284	1.50	1.41E-01	PDE4DIP	-64116	SEC22B	-37269
chr7	64023286	64023606	0.66	1.41E-01	ZNF680	59		
chr17	38738524	38738849	0.67	1.41E-01	CCR7	-16963	SMARCE1	65416
chr2	6985514	6985824	0.73	1.41E-01	CMPK2	20267		
chr3	5163729	5164085	1.33	1.41E-01	ARL8B	-23		
chr6	159230018	159230310	0.65	1.42E-01	EZR	9176	SYTL3	159118
chr11	1873998	1874414	0.68	1.42E-01	LSP1	6		
chr12	48206958	48207348	1.28	1.42E-01	HDAC7	6610	SLC48A1	40186
chr19	55787525	55788060	1.37	1.42E-01	SAPS1	-17755	HSPBP1	3958
chr6	130005163	130005469	1.65	1.42E-01	ARHGAP18	26054	LAMA2	801030
chr8	49862139	49862446	0.75	1.42E-01	SNTG1	-962304	SNAI2	-28305
chr6	41301749	41302042	0.77	1.42E-01	NCR2	-1632		
chr1	228674589	228674899	0.66	1.42E-01	RHOU	-196125	HIST3H2BB	28936
chr11	111712221	111712497	1.33	1.42E-01	PPP2R1B	-75190	ALG9	29946
chr1	19536730	19537204	1.36	1.42E-01	UBR4	-221		
chr15	70489048	70489294	1.40	1.43E-01	TLE3	-98915	UACA	566679
chr2	61371961	61372299	1.23	1.43E-01	PUS10	-126765	C2orf74	-17499
chr1	16162372	16162657	1.37	1.43E-01	UQCRHL	-28321	SPEN	-11844
chr3	14692961	14693435	1.59	1.43E-01	GRIP2	-109610	C3orf20	-23456
chr1	153895586	153895869	1.42	1.43E-01	GATAD2B	-277		
chr7	912968	913278	0.70	1.43E-01	C7orf20	-3068		
chr20	48732413	48732701	1.61	1.43E-01	TMEM189-UBE2V1	37778	SNAI1	133030
chr3	128399544	128399801	1.53	1.43E-01	RAB7A	-45306	RPN1	-29954
chr19	46366392	46366717	1.32	1.43E-01	FOXA3	-963	SYMPK	-7
chr7	23312739	23312989	0.71	1.44E-01	GPNMB	26548	IGF2BP3	197131
chr3	119182339	119182637	1.45	1.44E-01	TMEM39A	-17		
chr11	9779625	9779921	0.75	1.44E-01	SWAP70	94145	SBF2	535981
chr3	169489829	169490180	1.43	1.44E-01	ARPM1	-2322	MYNN	-848
chr7	48019050	48019375	0.72	1.44E-01	HUS1	33		
chr14	75716227	75716488	0.74	1.45E-01	TMED10	-73009	FOS	-29123
chr1	1550642	1550886	1.20	1.45E-01	MIB2	-120		
chr8	41347794	41348166	0.62	1.45E-01	GOLGA7	-101		
chr3	124714309	124714562	1.62	1.45E-01	MUC13	-60856	HEG1	60366
chr6	53583718	53584002	1.67	1.45E-01	LRRC1	-75674	KLHL31	-53354
chr17	29865156	29865467	0.73	1.46E-01	RAB11FIP4	146670	C17orf79	321014
chr5	43043242	43043583	0.59	1.46E-01	C5orf39	-2966		
chr8	95731899	95732205	0.77	1.46E-01	DPY19L4	-51		

chr1	25075130	25075586	1.55	1.46E-01	CLIC4	3598	RUNX3	181412				
chr19	36236312	36236623	1.43	1.46E-01	LIN37	-3044	TMEM149	-2948	U2AF1L4	-132	PSENEN	-26
chr11	108338115	108338373	1.28	1.46E-01	KDELC2	30915	ATM	244685				
chr7	5013421	5013893	0.74	1.46E-01	RBAK	-71896	MMD2	-14813				
chr1	226315190	226315541	1.47	1.47E-01	ACBD3	59057	H3F3B	64945				
chr8	38662850	38663249	0.71	1.47E-01	PLEKHA2	-95703	TACC1	18328				
chr4	79697002	79697249	1.32	1.47E-01	BMP2K	-406						
chr1	20142272	20142661	1.45	1.47E-01	RNF186	-696						
chr5	140079756	140080029	1.50	1.47E-01	ZMAT2	-139						
chr7	129845117	129845399	1.29	1.47E-01	C7orf45	-2446	TMEM209	80				
chr13	28024062	28024524	1.44	1.47E-01	MTIF3	418						
chr19	1021329	1021837	1.63	1.47E-01	CNN2	-4715	C19orf6	-442				
chr6	131520886	131521214	0.66	1.47E-01	ARG1	-373315	AKAP7	54625				
chr1	206808538	206808926	1.55	1.48E-01	DYRK3	-149						
chr7	106193258	106193539	1.33	1.48E-01	PIK3CG	-312525	NAMPT	-267761				
chr13	27825315	27825691	0.74	1.48E-01	RPL21	-189						
chr18	18691635	18691968	0.64	1.48E-01	ROCK1	10						
chr9	134553904	134554148	1.49	1.48E-01	UCK1	-147364	RAPGEF1	31203				
chr1	185286530	185286884	1.37	1.48E-01	IVNS1ABP	-246						
chr6	42713585	42713923	0.67	1.48E-01	TBCC	130						
chr2	136964871	136965336	1.30	1.48E-01	THSD7B	-783358	CXCR4	-89379				
chr10	121356518	121356763	0.59	1.48E-01	TIAL1	-100						
chr7	25219804	25220062	0.60	1.48E-01	CYCS	-54978	NPVF	48172				
chr19	1103672	1103945	1.28	1.48E-01	GPX4	-127						
chr22	43010678	43011043	1.26	1.48E-01	POLDIP3	101						
chr2	30369117	30369744	1.31	1.48E-01	ALK	-224999	LBH	-84966				
chr2	37899292	37899532	1.41	1.48E-01	CDC42EP3	-86						
chr9	132032494	132032787	1.55	1.48E-01	METTL11A	-355794	PPP2R4	159397				
chr3	150583164	150583437	1.53	1.49E-01	SIAH2	-102038	CLRN1	107485				
chr7	140623932	140624175	1.39	1.49E-01	BRAF	510						
chr19	49457828	49458188	1.42	1.49E-01	BAX	-109						
chr11	74660059	74660390	1.36	1.49E-01	SPCS2	-67	XRRA1	7				
chr20	25262293	25262569	1.40	1.49E-01	PYGB	33725	ABHD12	109046				
chr1	155915996	155916336	1.66	1.50E-01	RXFP4	4686	ARHGEF2	32170				
chr14	91782283	91782527	1.53	1.50E-01	GPR68	-62181	CCDC88C	101728				
chr10	69609073	69609360	1.58	1.51E-01	SIRT1	-35210	DNAJC12	-11280				
chr19	39971266	39971542	1.37	1.51E-01	TIMM50	352						
chr6	91085244	91085620	0.60	1.51E-01	BACH2	-78870	MAP3K7	211475				

chr1	26868691	26869047	1.29	1.52E-01	RPS6KA1	-3474		
chr4	121670691	121671015	1.23	1.52E-01	MAD2L1	-682840	PRDM5	173160
chr1	93441146	93441501	1.51	1.52E-01	MTF2	-103468	FAM69A	-14245
chr19	45981715	45982236	0.69	1.52E-01	FOSB	10723	RTN2	18337
chr1	70876592	70876921	0.72	1.53E-01	CTH	-198		
chr9	140356355	140356614	1.62	1.53E-01	NELF	-2699		
chr15	75315729	75316099	1.34	1.53E-01	PPCDC	-13		
chr17	77752639	77752953	1.29	1.53E-01	CBX2	803		
chr15	64445641	64446037	0.70	1.53E-01	SNX22	1923	PPIB	9515
chr7	128649522	128649778	1.45	1.53E-01	TNPO3	45548	IRF5	71656
chr18	42792733	42793012	1.54	1.53E-01	SLC14A2	-401893	SETBP1	532010
chr4	2263952	2264375	0.55	1.53E-01	MXD4	-425		
chr20	43607963	43608214	1.45	1.53E-01	STK4	12969	KCNS1	121664
chr17	76836922	76837204	1.32	1.53E-01	USP36	-94		
chr9	77703775	77704140	0.64	1.53E-01	C9orf95	-825	OSTF1	560
chr1	70820373	70820649	1.47	1.54E-01	ANKRD13C	-94	HHLA3	18
chr15	52548612	52548917	1.50	1.54E-01	GNB5	-65200	MYO5C	39205
chr2	231466258	231466598	1.64	1.54E-01	CAB39	-111129	SP100	185557
chr8	103875152	103875581	0.74	1.54E-01	KLF10	-207384	AZIN1	1030
chr3	128598201	128598556	1.40	1.54E-01	ACAD9	46		
chr4	164509024	164509342	1.54	1.54E-01	TKTL2	-114136	MARCH1	25593
chr19	5790445	5790946	1.57	1.54E-01	DUS3L	553		
chr22	37641484	37641867	1.53	1.54E-01	RAC2	-1371		
chr13	100027658	100028087	1.55	1.54E-01	TM9SF2	-125855	GPR183	-68124
chr3	177075679	177075972	1.57	1.54E-01	TBL1XR1	-160778		
chr2	69066414	69066727	0.67	1.55E-01	BMP10	32078	ARHGAP25	104603
chr11	93517204	93517537	1.36	1.55E-01	MED17	-34		
chr4	71553918	71554339	1.44	1.55E-01	UTP3	-67		
chr15	59279712	59279960	1.46	1.55E-01	RNF111	-29		
chr19	16683181	16683512	1.64	1.56E-01	SLC35E1	-154		
chrX	9431124	9431535	1.37	1.56E-01	TBL1X	-1871		
chr11	134094365	134094601	1.42	1.56E-01	VPS26B	-78	NCAPD3	-57
chr8	54983541	54983837	0.69	1.56E-01	TCEA1	-48681	LYPLA1	30888
chr3	187934896	187935310	0.70	1.56E-01	TPRG1	-954660	LPP	4382
chr3	40498616	40498878	1.43	1.56E-01	RPL14	-54		
chr12	113634799	113635073	0.62	1.56E-01	TPCN1	-24324	DDX54	-11652
chr4	26809050	26809297	0.68	1.57E-01	STIM2	-53190	TBC1D19	223628
chr3	177043048	177043317	1.58	1.57E-01	TBL1XR1	-128135		

chr17	38256911	38257155	1.33	1.57E-01	NR1D1	-60		
chr12	31559158	31559468	0.87	1.57E-01	OVOS1	-200225	DENND5B	184639
chr8	75262543	75262918	0.67	1.57E-01	GDAP1	113		
chr17	17380136	17380406	1.28	1.57E-01	MED9	-29		
chr20	43991550	43992154	0.68	1.57E-01	SYS1	151		
chr3	128968142	128968510	1.57	1.57E-01	COPG	-127		
chr6	41691248	41691558	0.72	1.57E-01	TFEB	61		
chr3	169663118	169663470	1.43	1.57E-01	LRRC31	-75634	SEC62	-21286
chr7	81475747	81476058	0.69	1.57E-01	HGF	-76451	CACNA2D1	597128
chr9	137029898	137030237	1.40	1.57E-01	RXRA	-188248	WDR5	28858
chr17	7338442	7338732	1.24	1.58E-01	FGF11	-4102	TMEM102	-175
chr11	64546263	64546709	1.43	1.58E-01	SF1	-245		
chr11	125757401	125757661	1.44	1.58E-01	HYLS1	4022	PUS3	15585
chr20	52539472	52539798	1.64	1.58E-01	ZNF217	-339928	BCAS1	147669
chr21	30364821	30365175	0.76	1.58E-01	RNF160	279		
chr17	74259145	74259393	0.57	1.58E-01	RNF157	-22879	QRICH2	44492
chr3	45637790	45638227	1.31	1.58E-01	SACM1L	-92745	LIMD1	1686
chr8	101965485	101965863	0.66	1.58E-01	YWHAZ	-453		
chr12	262348	262658	0.70	1.58E-01	SLC6A12	60868	IQSEC3	75961
chr9	96328704	96329098	1.19	1.58E-01	PHF2	-10008	FAM120A	114728
chr2	42588333	42588657	0.77	1.59E-01	COX7A2L	493		
chr1	28499163	28499484	0.61	1.59E-01	EYA3	-84193	PTAFR	3867
chr1	145096177	145096498	1.58	1.59E-01	SEC22B	-69		
chr9	37374913	37375249	0.69	1.59E-01	GRHPR	-47626	ZCCHC7	254612
chr11	119978952	119979199	1.44	1.59E-01	PVRL1	-379641	TRIM29	29787
chr9	71394783	71395038	1.31	1.59E-01	PIP5K1B	74295	PRKACG	234128
chr2	122512975	122513285	0.74	1.59E-01	TSN	9		
chr11	46722099	46722548	1.50	1.60E-01	ARHGAP1	-204	ZNF408	-44
chr13	53029366	53029718	0.61	1.60E-01	VPS36	-4779	CKAP2	47
chr1	231473519	231473863	1.44	1.60E-01	EXOC8	-113	C1orf124	9
chr17	19209599	19210106	1.54	1.60E-01	b9	56193	EPN2	69163
chr5	176784694	176784982	1.50	1.60E-01	RGS14	-6		
chr17	37912049	37912682	1.84	1.60E-01	GRB7	18179	IKZF3	108075
chr22	39842397	39842795	0.76	1.60E-01	MGAT3	-40633	MAP3K7IP1	46837
chr3	133209931	133210329	0.69	1.60E-01	CDV3	-82304	BFSP2	91340
chr5	159507898	159508313	0.61	1.60E-01	FABP6	-106268	TTC1	71926
chr3	62804005	62804383	1.51	1.60E-01	FEZF2	-445004	CADPS	56870
chr9	116172424	116172867	1.55	1.61E-01	POLE3	383		

chr9	94877500	94877922	1.41	1.62E-01	SPTLC1	-21		
chr5	133436216	133436472	1.36	1.62E-01	VDAC1	-95911	TCF7	-15256
chr6	56224114	56224390	1.44	1.62E-01	COL21A1	-111874	DST	595161
chr3	14732832	14733142	0.74	1.62E-01	FGD5	-127482	C3orf20	16333
chr10	37177967	37178214	1.29	1.62E-01	ANKRD30A	-236694		
chr10	74020521	74020802	0.65	1.62E-01	DDIT4	-13015	C10orf104	44856
chr16	89355883	89356123	1.20	1.62E-01	ZNF778	71892	ANKRD11	200966
chr19	2631464	2631753	0.64	1.62E-01	GNG7	71137	GADD45B	155474
chr7	99102628	99102938	0.68	1.62E-01	ZNF394	-4906	ZKSCAN5	510
chr18	24229663	24230000	1.50	1.63E-01	KCTD1	-101332	AQP4	215884
chr15	57511405	57511715	1.87	1.63E-01	CGNL1	-157145	TCF12	300727
chr2	70418022	70418339	1.20	1.63E-01	TIA1	57598	PCBP1	103596
chr14	94434025	94434340	1.50	1.64E-01	OTUB2	-58541	ASB2	-10416
chr16	11782978	11783258	1.52	1.64E-01	SNN	20817	TXNDC11	53530
chr3	101405492	101405777	1.55	1.64E-01	RPL24	-72		
chr13	103683811	103684280	0.81	1.64E-01	SLC10A2	35150	ERCC5	224550
chr1	27668308	27668584	1.17	1.64E-01	SYTL1	-67		
chr10	98479547	98479857	0.60	1.65E-01	PIK3AP1	577		
chr7	19748495	19748797	0.63	1.65E-01	TWISTNB	14		
chr19	36822569	36822845	0.77	1.65E-01	ZNF565	-117141	ZFP14	36166
chr1	156024319	156024732	1.45	1.65E-01	UBQLN4	-1010	ROBLD3	9
chr17	7307374	7307954	1.37	1.65E-01	NLGN2	-3838	C17orf61	-246
chr8	56852179	56852459	0.71	1.65E-01	LYN	59933	RPS20	134821
chr6	83924902	83925201	1.63	1.65E-01	PGM3	-22117	ME1	215727
chr9	35072493	35072900	0.70	1.66E-01	VCP	42		
chr7	26404712	26405030	0.66	1.66E-01	SNX10	73356	SKAP2	499470
chr12	133562770	133563236	1.41	1.67E-01	ZNF26	-53		
chr4	113174573	113174883	0.63	1.67E-01	AP1AR	21833	TIFA	32331
chrX	1510463	1510707	1.43	1.67E-01	SLC25A6	413		
chr2	202122954	202123316	1.49	1.67E-01	CASP8	381		
chr3	8398941	8399204	1.23	1.67E-01	LMCD1	-144438		
chr13	47557233	47557664	0.68	1.67E-01	HTR2A	-87080		
chr19	8454759	8455009	1.52	1.68E-01	RAB11B	-321		
chr17	7155308	7155588	1.31	1.68E-01	DULLARD	-453	C17orf81	-103
chr3	15091952	15092199	1.30	1.68E-01	MRPS25	14740	NR2C2	102840
chr3	185655061	185655408	1.37	1.68E-01	TRA2B	689		
chr14	64805490	64805929	0.79	1.69E-01	MTHFD1	-49049	ESR2	-44582
chr9	134955186	134955437	1.36	1.69E-01	MED27	-59		

chr10	102295394	102295760	1.56	1.69E-01	HIF1AN	-64		
chr5	139015286	139015562	1.37	1.69E-01	CXXC5	-12877	UBE2D2	74673
chr10	112174366	112174744	0.77	1.70E-01	SMNDC1	-109848	DUSP5	-83070
chr9	131038311	131038654	1.31	1.70E-01	GOLGA2	-215		
chr1	40071840	40072150	0.74	1.70E-01	PABPC4	-29474	HEYL	33353
chr1	153962969	153963363	0.72	1.70E-01	RAB13	-4360	RPS27	-73
chr12	10766049	10766359	1.28	1.70E-01	MAGOHB	-21		
chr2	232578485	232578815	0.70	1.70E-01	PTMA	5415	PDE6D	67324
chr3	189679289	189679633	1.58	1.70E-01	LEPREL1	159447	TP63	330245
chr6	143206676	143206920	1.63	1.70E-01	HIVEP2	59540	GPR126	583742
chr19	52692598	52693688	1.42	1.70E-01	PPP2R1A	-48		
chr10	89623088	89623514	0.65	1.70E-01	KILLIN	-107	PTEN	106
chr16	74729801	74730045	1.43	1.70E-01	RFWD3	-29144	MLKL	4866
chr7	24758848	24759182	0.58	1.70E-01	DFNA5	38068	MPP6	145930
chr16	11877222	11877651	1.37	1.70E-01	ZC3H7A	-1029		
chr16	89034538	89035041	1.34	1.70E-01	LOC390748	-101776	CBFA2T3	8611
chr12	56323188	56323514	0.73	1.71E-01	DGKA	-2461	WIBG	-1654
chr11	118800912	118801297	1.57	1.71E-01	UPK2	-25921	BCL9L	-19492
chr2	74007122	74007475	1.23	1.71E-01	DUSP11	-15		
chr19	8334018	8334338	1.47	1.71E-01	CD320	39061	LASS4	59961
chr6	26216597	26216973	0.64	1.71E-01	HIST1H2AE	-363	HIST1H2BG	87
chr19	41120447	41120723	1.24	1.72E-01	LTBP4	17444	NUMBL	75971
chr16	30546302	30546584	1.35	1.72E-01	ZNF747	-249		
chr7	3018095	3018517	1.34	1.72E-01	GNA12	-134347	CARD11	65273
chr13	49789177	49789447	0.84	1.72E-01	MLNR	-5162	FNDC3A	239264
chr2	47935930	47936240	1.57	1.72E-01	KCNK12	-138615	MSH6	-74136
chr13	46734679	46735040	1.64	1.73E-01	CPB2	-55649	LCP1	21599
chr7	134331375	134331724	1.26	1.73E-01	BPGM	19		
chr16	30420793	30421071	0.72	1.73E-01	ZNF771	2197	DCTPP1	20441
chr22	23277754	23278152	1.46	1.73E-01	GNAZ	-134716	IGL@	47993
chr22	29137613	29137951	1.48	1.73E-01	HSCB	-261	CHEK2	40
chr19	16295984	16296229	1.28	1.73E-01	FAM32A	-128		
chr17	27279312	27279721	1.57	1.73E-01	PHF12	-1009		
chr17	72869336	72869633	1.22	1.73E-01	FDXR	-329		
chr2	27357325	27357682	1.33	1.73E-01	PREB	38		
chr3	119217148	119217409	1.47	1.73E-01	C3orf1	-89		
chr8	96149933	96150256	0.85	1.73E-01	PLEKHF2	4057		
chr1	186344326	186344720	1.41	1.73E-01	C1orf27	-367	TPR	-66

chr3	14220065	14220546	1.33	1.74E-01	XPC	-134	LSM3	-31
chr12	22778689	22779065	0.78	1.74E-01	ETNK1	801		
chr10	26745959	26746255	1.40	1.74E-01	PDSS1	-240488	APBB1IP	18841
chr3	169530699	169530943	1.54	1.74E-01	LRRC34	-509		
chr4	170581219	170581553	0.81	1.74E-01	CLCN3	39664	C4orf27	97707
chr19	50490417	50490752	1.22	1.75E-01	SIGLEC11	-26156	VRK3	38220
chr18	54684405	54684715	1.43	1.75E-01	ST8SIA3	-335161	WDR7	365944
chr15	67378104	67378631	1.57	1.75E-01	SMAD3	20173	AAGAB	168706
chr11	67416685	67416995	0.78	1.75E-01	TBX10	-9809	ACY3	1290
chr16	30705160	30705505	0.66	1.75E-01	SRCAP	-5129	FBRS	29555
chr3	129158728	129159152	1.40	1.75E-01	MBD4	-88	IFT122	-28
chr6	26305596	26305863	1.59	1.76E-01	BTN3A2	-59668	HIST1H4H	-20003
chr17	4272894	4273318	1.45	1.76E-01	UBE2G1	-3137		
chr15	66681881	66682127	0.66	1.76E-01	MAP2K1	2793	SNAPC5	108142
chr8	67341070	67341380	0.61	1.76E-01	ADHFE1	-3493	RRS1	-38
chr3	9005076	9005355	1.20	1.77E-01	RAD18	-70		
chr11	118436659	118436968	1.31	1.77E-01	C11orf60	-64		
chr12	111872747	111873221	1.19	1.77E-01	SH2B3	29232	ATXN2	164496
chr22	38022089	38022362	1.54	1.77E-01	SH3BP1	-12831	GGA1	17723
chr15	40397824	40398119	1.39	1.77E-01	SRP14	-66583	BMF	3103
chr19	18392473	18392709	1.23	1.77E-01	JUND	-159		
chr2	242383306	242383597	1.52	1.77E-01	STK25	64582	FARP2	87741
chr19	34850116	34850612	1.38	1.77E-01	GPI	-5705	LSM14A	187012
chr6	88464548	88464917	0.78	1.78E-01	SPACA1	-292774	AKIRIN2	-52748
chr12	132434342	132434595	1.22	1.78E-01	EP400	-39		
chr8	86089264	86089612	0.73	1.78E-01	E2F5	-181		
chr1	171282969	171283290	1.66	1.78E-01	FMO4	-356		
chr1	161493547	161493852	1.66	1.79E-01	HSPA6	-336		
chr1	173446081	173446338	1.43	1.79E-01	PRDX6	-276		
chr19	12792106	12792382	1.42	1.79E-01	DHPS	433		
chr19	12035334	12035750	0.81	1.79E-01	ZNF700	-358		
chrX	40943631	40944069	1.25	1.79E-01	USP9X	-1038		
chr7	150020130	150020611	0.70	1.79E-01	LRRC61	75		
chr13	67798350	67798635	1.35	1.79E-01	PCDH9	5975		
chr11	20408834	20409269	0.72	1.79E-01	PRMT3	-24		
chr2	65258417	65258751	1.27	1.79E-01	CEP68	-24911	SLC1A4	42128
chr16	21369596	21369998	1.39	1.79E-01	CRYM	-80140	NPIPL3	66902
chr17	44270014	44270395	0.81	1.80E-01	LRRC37A	-102292	KIAA1267	-20607

chr11	68823404	68823991	1.36	1.80E-01	CCND1	-632175	TPCN2	7348
chr16	56390352	56390707	1.22	1.80E-01	AMFR	68914	GNAO1	165279
chr16	24025610	24025909	1.41	1.80E-01	CACNG3	-241116	PRKCB	178460
chr19	11039269	11039579	1.37	1.80E-01	YIPF2	-67		
chr19	52328082	52328392	0.65	1.81E-01	FPR3	29826	ZNF577	62992
chr3	167452525	167453044	1.62	1.81E-01	SERPINI1	-647	PDCD10	-134
chr5	131758449	131758744	1.38	1.81E-01	SLC22A5	53196	IRF1	67868
chr11	67159211	67159488	1.41	1.81E-01	RAD9A	-73		
chr9	98256305	98256572	1.28	1.81E-01	FANCC	-176448	PTCH1	14392
chr16	30933675	30934029	1.26	1.81E-01	FBXL19	-2044		
chr16	67260850	67261131	1.49	1.81E-01	LRRC29	-90	TMEM208	-25
chr20	43150701	43151048	0.76	1.81E-01	SERINC3	-149		
chr7	124569809	124570058	1.31	1.82E-01	POT1	103		
chr7	37375853	37376168	1.52	1.82E-01	AOAH	-611858	ELMO1	112500
chr10	112116370	112116716	1.42	1.82E-01	DUSP5	-141082	SMNDC1	-51836
chr2	207024195	207024565	1.32	1.82E-01	NDUFS1	-193	EEF1B2	62
chr1	26946837	26947202	1.39	1.82E-01	ARID1A	-75502	RPS6KA1	74677
chr6	131949268	131949628	0.74	1.82E-01	MED23	-85		
chr11	45053507	45053958	0.83	1.82E-01	TP53I11	-81125	SYT13	254151
chrX	12974916	12975284	0.92	1.82E-01	TMSB4X	-18125	TLR8	50342
chr1	40804400	40804745	0.75	1.82E-01	SMAP2	-35155	COL9A2	-21592
chr9	97488824	97489077	1.29	1.82E-01	C9orf3	-43		
chr1	66797256	66797532	1.21	1.82E-01	SGIP1	-202431	PDE4B	538538
chr11	63741801	63742180	1.43	1.83E-01	COX8A	-88		
chr8	38590089	38590380	0.69	1.83E-01	RNF5	-131460	TACC1	-54487
chr22	23480149	23480405	1.40	1.83E-01	RTDR1	3964	GNAZ	67608
chr5	126113962	126114285	1.31	1.83E-01	LMNB1	1291	MARCH3	252316
chr5	159435945	159436340	1.42	1.83E-01	TTC1	-37		
chr20	4954303	4954547	1.21	1.84E-01	RASSF2	-158656	SLC23A2	36514
chr19	7694481	7694767	1.34	1.84E-01	XAB2	-185		
chr17	27475913	27476157	1.38	1.84E-01	TIAF1	-73408	MYO18A	31372
chr1	234659372	234659624	1.61	1.84E-01	TARBP1	-44649	IRF2BP2	85773
chr9	37004657	37005181	1.43	1.84E-01	PAX5	29557	MELK	432014
chr7	77326724	77326975	0.69	1.84E-01	RSBN1L	1107	TMEM60	100897
chr13	99551370	99551637	0.65	1.84E-01	SLC15A1	-146575	DOCK9	187156
chr13	77901215	77901561	1.38	1.84E-01	MYCBP2	-211		
chr11	9710761	9711071	0.76	1.84E-01	SWAP70	25288	SBF2	604838
chr9	33297302	33297804	1.47	1.84E-01	NFX1	7043	AQP7	104964

chr19	2783288	2783772	1.34	1.85E-01	THOP1	-1976	SGTA	-176
chr8	61839149	61839437	0.64	1.85E-01	RLBP1L1	-361232	CHD7	247954
chr2	234312954	234313270	0.57	1.85E-01	DGKD	49959	USP40	161124
chr3	16553411	16553815	1.51	1.85E-01	RFTN1	1609	OXNAD1	246899
chr6	16205625	16206050	0.68	1.85E-01	GMPR	-32973	MYLIP	76521
chr7	100895370	100895844	0.80	1.85E-01	FIS1	-7236	RABL5	69486
chr6	32096032	32096299	0.76	1.86E-01	ATF6B	-149		
chr15	69167988	69168499	1.47	1.86E-01	ANP32A	-54983	NOX5	-54620
chr19	5622632	5623141	1.40	1.87E-01	SAFB	-277	SAFB2	51
chr16	66550709	66551081	1.49	1.87E-01	TK2	33420	BEAN	89655
chr2	197664303	197664608	1.51	1.87E-01	GTF3C3	-31		
chr13	51374606	51374864	1.57	1.88E-01	RNASEH2B	-109157	ST13	628581
chr7	130804246	130804556	0.63	1.89E-01	KLF14	-385541	MKLN1	-208194
chr8	134512856	134513268	1.38	1.89E-01	NDRG1	-203515	ST3GAL1	71121
chr3	129118376	129118693	1.56	1.89E-01	H1FX	-83415	MBD4	40317
chr17	7386590	7386869	1.57	1.89E-01	POLR2A	-1120	ZBTB4	838
chr3	185378265	185378555	1.48	1.89E-01	SENP2	74379	IGF2BP2	164417
chr1	244615433	244615747	1.54	1.89E-01	ADSS	-177		
chr12	118454307	118454802	0.66	1.89E-01	RFC5	47		
chr15	72343642	72343927	0.63	1.89E-01	MYO9A	66637	NR2E3	240891
chr14	21077476	21077752	1.34	1.90E-01	RNASE12	-18632	OR6S1	32236
chr12	133656656	133657333	1.49	1.90E-01	ZNF140	-42		
chr1	163291450	163291782	1.62	1.90E-01	NUF2	-107		
chr8	56792199	56792493	0.60	1.90E-01	LYN	-40		
chr14	91882601	91882889	1.46	1.90E-01	GPR68	-162521	CCDC88C	1388
chr20	10414771	10415047	1.27	1.90E-01	C20orf94	-1042	MKKS	-43
chr1	161135644	161136424	0.73	1.90E-01	PPOX	-147		
chr17	884273	884600	0.68	1.90E-01	NXN	-1427		
chr13	114519991	114520249	0.69	1.91E-01	GAS6	46926	FAM70B	57904
chr3	52481092	52481348	0.68	1.91E-01	SEMA3G	-2177		
chr18	33077515	33078089	1.64	1.91E-01	INO80C	153		
chr3	140911077	140911390	1.21	1.91E-01	ACPL2	-39448	SPSB4	140491
chr15	67813260	67813568	1.22	1.92E-01	C15orf61	-108		
chr16	68108586	68108916	0.71	1.92E-01	DDX28	-50792	NFATC3	-10624
chr19	52154472	52154718	1.44	1.92E-01	SIGLEC14	-4463		
chr16	71518305	71518710	1.34	1.92E-01	ZNF23	-22391	ZNF19	4746
chr6	20291983	20292293	0.83	1.92E-01	E2F3	-109999	MBOAT1	-79468
chr7	99698799	99699321	0.61	1.92E-01	AP4M1	-70	MCM7	367

chr5	167913270	167913580	1.49	1.93E-01	RARS	-38		
chr2	203130274	203130580	1.30	1.93E-01	NOP58	-88		
chr2	85581589	85581902	1.36	1.93E-01	ELMOD3	-97	RETSAT	75
chr21	35288034	35288278	1.33	1.93E-01	SLC5A3	-157667	ITSN1	273372
chr11	118797032	118797329	1.54	1.93E-01	UPK2	-29845	BCL9L	-15568
chr17	34454642	34454974	0.59	1.93E-01	CCL4	23588	CCL3L1	69348
chr1	109642559	109642937	0.69	1.93E-01	KIAA1324	-13785	TMEM167B	9345
chr10	74124074	74124384	0.75	1.93E-01	DNAJB12	-9322	CBARA1	261670
chr7	42923503	42923913	0.71	1.94E-01	GLI3	-647090	C7orf25	28443
chr12	122430136	122430412	1.59	1.94E-01	BCL7A	-29587	PSMD9	103628
chr19	1383091	1383591	1.51	1.94E-01	NDUFS7	-366		
chr20	1415564	1415877	1.37	1.94E-01	FKBP1A	-41905	NSFL1C	32696
chr5	1485140	1485417	1.07	1.94E-01	SLC6A3	-39741	LPCAT1	38797
chr9	86322953	86323321	1.47	1.95E-01	AK300656	-99	UBQLN1	31
chr11	102906754	102907030	1.65	1.95E-01	MMP13	-80430	DYNC2H1	-73268
chr19	29080937	29081240	1.53	1.95E-01	UQCRFS1	623047		
chr2	1618469	1618713	1.76	1.95E-01	PXDN	129700	TPO	201358
chr8	123685748	123686006	0.74	1.95E-01	ZHX2	-108024		
chr19	29110782	29111092	1.23	1.95E-01	UQCRFS1	593199		
chr10	5894326	5894602	1.39	1.95E-01	FBXO18	-41885	GDI2	-38952
chr17	8022131	8022664	0.82	1.96E-01	ALOXE3	-538		
chr7	140376167	140376463	1.28	1.96E-01	NDUFB2	-20166	ADCK2	3362
chr19	18668329	18668662	1.37	1.96E-01	C19orf50	-108		
chr9	125693765	125694088	1.18	1.96E-01	ZBTB26	-148		
chr2	234257843	234258108	1.24	1.96E-01	DGKD	-5177	SAG	41667
chr6	28908708	28909036	1.48	1.96E-01	TRIM27	-17104	ZNF311	64163
chr11	34073671	34073911	1.47	1.96E-01	CAPRIN1	561		
chr5	117842336	117842624	0.58	1.97E-01	TNFAIP8	-849116		
chr7	100026372	100026809	0.76	1.97E-01	MEPCE	-938	ZCWPW1	-289
chr7	156685847	156686088	1.16	1.97E-01	LMBR1	-66		
chr17	27278521	27278962	1.46	1.97E-01	PHF12	-234		
chr1	230031300	230031630	0.66	1.97E-01	GALNT2	-171491	URB2	269484
chr19	36618668	36619041	1.32	1.97E-01	POLR2I	-12649	CAPNS1	-12063
chr2	190648722	190649265	0.69	1.97E-01	ORMDL1	103	PMS1	183
chr19	16181265	16181597	0.76	1.97E-01	RAB8A	-41059	TPM4	3114
chr11	72862981	72863327	0.71	1.97E-01	P2RY2	-66190	FCHSD2	-10011
chr15	75230474	75230726	1.34	1.97E-01	COX5A	-105		
chr2	89157545	89158020	1.26	1.98E-01	O1/O11 and JK2	-732779	RPIA	166607

chr2	182321345	182321656	0.75	1.98E-01	ITGA4	-118		
chr13	34320071	34320478	0.80	1.98E-01	STARD13	-460374	RFC3	-71931
chr10	27531878	27532498	1.43	1.98E-01	ACBD5	-2380		
chr19	41816077	41816502	1.45	1.98E-01	TGFB1	43526	HNRNPUL1	46170
chr16	19535009	19535259	1.32	1.98E-01	GDE1	-1684	CP110	-45
chr7	100076755	100077155	0.63	1.98E-01	TSC22D4	-53		
chr21	43880973	43881323	1.48	1.98E-01	RSPH1	35253	UBASH3A	57129
chr13	101327190	101327544	1.27	1.98E-01	TMTC4	-264		
chr15	84624780	84625091	0.75	1.98E-01	ZSCAN2	-519313	ADAMTSL3	302098
chr1	249167877	249168192	1.51	1.98E-01	ZNF692	-14764		
chr3	133293173	133293483	0.66	1.99E-01	CDV3	894		
chr19	59031120	59031452	1.30	1.99E-01	ZBTB45	-365		
chr5	142814860	142815162	1.52	1.99E-01	NR3C1	-30966	YIPF5	735267
chr2	232539681	232540121	0.66	1.99E-01	NMUR1	-144719	PTMA	-33334
chr11	67124318	67124621	1.49	1.99E-01	POLD4	-3453		
chr14	105531755	105532014	1.60	1.99E-01	GPR132	-131		
chr9	133919141	133919451	0.91	1.99E-01	AIF1L	-52616	LAMC3	34792
chr5	108744629	108745214	0.75	1.99E-01	PJA2	753		
chr1	244816017	244816354	1.37	1.99E-01	FAM36A	-182453	C1orf101	191513
chr3	160603533	160603865	1.62	2.00E-01	PPM1L	129703	B3GALNT1	218984
chr6	52860333	52860843	0.93	2.00E-01	GSTA4	-410		
chr9	128469444	128469702	1.22	2.00E-01	MAPKAP1	-60		
chr1	197898088	197898398	0.87	2.00E-01	NEK7	-227865	LHX9	11726
chr6	26286636	26286911	0.78	2.01E-01	HIST1H4H	-1047		
chr6	139540446	139540756	0.68	2.01E-01	TXLNB	72607	HECA	84352
chr2	150444098	150444571	0.83	2.01E-01	MMADHC	-5		
chr1	208108546	208108896	1.80	2.02E-01	CD34	-24038	PLXNA2	308944
chr1	109288932	109289273	1.25	2.02E-01	STXBP3	-182		
chr8	102149357	102149750	0.74	2.02E-01	YWHAZ	-184333	ZNF706	68406
chr13	49066674	49067037	0.80	2.02E-01	LPAR6	-79203	RCBTB2	40460
chr1	24127247	24127525	1.38	2.02E-01	GALE	-1326		
chr12	9102550	9102892	1.33	2.02E-01	M6PR	-469		
chr2	29093043	29093400	1.38	2.02E-01	TRMT61B	-47		
chr1	33282783	33283156	1.34	2.02E-01	S100PBP	-206	YARS	663
chr1	226216709	226216968	1.43	2.02E-01	LEFTY2	-87919	H3F3B	-33582
chr12	110486271	110486640	1.39	2.02E-01	IFT81	-75684	GIT2	-52262
chr9	125027005	125027252	1.30	2.02E-01	MRRF	-18	RBM18	14
chr9	140130929	140131239	1.30	2.03E-01	TUBB2C	-4627		

chr7	120590845	120591364	1.31	2.03E-01	ING3	288		
chr20	49545850	49546235	1.43	2.03E-01	ADNP	1484	BCAS4	134576
chr5	180618546	180618950	1.53	2.03E-01	TRIM7	13429	OR2V2	36805
chrX	152110050	152110331	1.22	2.04E-01	PNMA3	-114575	ZNF185	27194
chr5	123985846	123986270	1.46	2.04E-01	ZNF608	94807		
chr9	139702151	139702470	1.37	2.04E-01	C9orf86	119		
chr1	154947274	154947657	1.24	2.04E-01	SHC1	-4243	CKS1B	348
chr11	64037373	64037732	1.38	2.04E-01	BAD	14623	PLCB3	18431
chr18	21977880	21978215	0.73	2.04E-01	OSBPL1A	-258		
chr1	153763556	153763980	1.52	2.04E-01	SLC27A3	16000	GATAD2B	131683
chr11	125757958	125758268	0.74	2.05E-01	HYLS1	4604	PUS3	15003
chr22	37638490	37638823	1.35	2.05E-01	SSTR3	-30304	RAC2	1648
chr9	136890411	136890667	1.15	2.05E-01	VAV2	-33093	BRD3	42602
chr2	228189800	228190078	1.56	2.05E-01	MFF	-2289		
chr17	2907571	2907964	0.72	2.05E-01	OR1D4	59187	RAP1GAP2	208036
chr3	52443918	52444173	1.29	2.05E-01	PHF7	-481	BAP1	-37
chr3	48229790	48230258	0.72	2.05E-01	CDC25A	-223		
chr19	36438350	36438660	1.52	2.05E-01	SDHAF1	-47596	LRFN3	10483
chr19	11302532	11302939	0.67	2.06E-01	LOC55908	-46081	SPC24	-36252
chr20	45891245	45891706	0.77	2.06E-01	ZMYND8	93998	EYA2	368213
chr6	26045521	26045831	1.29	2.06E-01	HIST1H2BB	-1791	HIST1H3C	37
chr19	18632877	18633187	0.72	2.06E-01	ELL	-95		
chr14	81444783	81445171	1.33	2.06E-01	TSHR	23108	GTF2A1	242317
chr1	19578013	19578323	0.76	2.06E-01	KIAA0090	-122	MRTO4	93
chr6	157744457	157744707	0.77	2.07E-01	C6orf35	211		
chr14	106873540	106873864	0.91	2.07E-01	ADAM6	-435344	IGHV7-81	409581
chr6	56954648	56955049	0.73	2.07E-01	ZNF451	21		
chr10	12285925	12286207	1.33	2.07E-01	CAMK1D	-105517	NUDT5	-47923
chr11	60673848	60674276	1.30	2.07E-01	PRPF19	-1		
chr15	77197638	77197898	1.39	2.07E-01	RCN2	-26194	SCAPER	-21551
chr12	76741946	76742455	0.69	2.07E-01	BBS10	21		
chr19	6588037	6588313	1.17	2.08E-01	CD70	2988	TNFSF9	57165
chr7	66093379	66093751	0.76	2.08E-01	KCTD7	-325		
chr15	52526699	52527034	0.75	2.08E-01	GNB5	-43302	MYO5C	61103
chr12	50038390	50038635	0.77	2.08E-01	PRPF40B	14185	FMNL3	62684
chr7	44678836	44679080	1.28	2.08E-01	ZMIZ2	-109572	OGDH	32787
chr14	55737859	55738169	0.77	2.09E-01	DLGAP5	-79618	KIAA0831	140562
chr11	65868190	65868546	0.72	2.09E-01	KLC2	-156806	PACS1	30544

chr14	23388215	23388798	0.70	2.09E-01	RBM23	-111		
chr19	34801242	34801552	0.61	2.09E-01	GPI	-54672	LSM14A	138045
chr19	42721862	42722287	1.38	2.09E-01	ZNF526	-2417	DEDD2	-262
chr17	47755292	47755627	1.34	2.09E-01	SPOP	65		
chr11	72863903	72864268	1.50	2.09E-01	P2RY2	-65258	FCHSD2	-10943
chr6	143265577	143265913	1.55	2.09E-01	HIVEP2	593		
chr19	41256155	41256573	1.26	2.09E-01	SNRPA	-415		
chr11	61730624	61730919	1.41	2.09E-01	FTH1	4360	BEST1	13416
chr7	101504448	101504860	0.66	2.09E-01	SH2B2	-423751	CUX1	43772
chr17	1552074	1552417	1.24	2.10E-01	SCARF1	-3205		
chr22	38859361	38859637	1.60	2.10E-01	KDELR3	-4584		
chr19	984146	984458	1.23	2.10E-01	GRIN3B	-16135	ARID3A	58265
chr22	37703086	37703330	0.58	2.10E-01	CYTH4	24713	ELFN2	120297
chr17	42976777	42977162	0.77	2.10E-01	YQ028	-3157	EFTUD2	23
chr7	48149806	48150050	0.72	2.10E-01	ABCA13	-61129	UPP1	21573
chr19	50979446	50979820	1.21	2.10E-01	C19orf63	-103		
chr2	223776765	223777196	1.48	2.10E-01	KCNE4	-139881	ACSL3	51249
chr6	150262911	150263193	1.39	2.10E-01	ULBP2	-84		
chr5	122733975	122734316	0.72	2.11E-01	CEP120	25106	PRDM6	309305
chr18	33729914	33730348	0.81	2.11E-01	MOCOS	-37349	ELP2	20244
chr12	49245879	49246317	1.37	2.11E-01	DDX23	-141		
chr2	11970212	11970654	1.21	2.11E-01	TRIB2	-886565	LPIN1	83693
chr11	130786297	130786593	1.36	2.11E-01	SNX19	-63		
chr12	1425035	1425312	1.66	2.11E-01	FBXL14	278157	ERC1	324770
chr3	126793714	126794143	0.81	2.11E-01	PLXNA1	86427	TPRA1	515639
chr14	99947603	99947847	1.40	2.12E-01	CCNK	-14		
chr19	58090192	58090551	1.35	2.12E-01	ZNF416	-129		
chr9	95491598	95491908	0.72	2.12E-01	IPPK	-59206	BICD2	35330
chr17	18759488	18759761	1.45	2.12E-01	PRPSAP2	-1867		
chr11	111288520	111288862	1.49	2.13E-01	POU2AF1	-38534	BTG4	94373
chr19	3133476	3133872	1.27	2.13E-01	GNA15	-2517		
chr3	3221265	3221565	1.52	2.13E-01	CRBN	-25		
chr5	98264875	98265491	1.37	2.13E-01	CHD1	-2945		
chr1	154810121	154810435	1.27	2.13E-01	ADAR	-229596	KCNN3	32476
chr2	131862516	131862899	0.70	2.13E-01	PLEKHB2	288		
chr6	43238957	43239269	1.45	2.13E-01	SLC22A7	-26885	TTBK1	27891
chr11	118955265	118955704	1.37	2.13E-01	HMBS	-102		
chr1	160752079	160752389	1.25	2.13E-01	LY9	-13694	SLAMF7	43157

chr1	222885970	222886339	1.33	2.14E-01	AIDA	-291	C1orf58	249
chr1	202896303	202896622	1.50	2.14E-01	KLHL12	-92		
chr17	35305997	35306267	1.21	2.14E-01	AATF	-43		
chr18	72219236	72219480	1.77	2.14E-01	ZNF407	-123565	CNDP1	17666
chr2	43037487	43037862	1.35	2.14E-01	HAAO	-17924	ZFP36L2	416070
chr2	8714419	8714694	1.17	2.14E-01	ID2	-107427		
chr17	40608859	40609116	1.39	2.14E-01	ATP6V0A1	-1874		
chr15	67155221	67155531	0.78	2.14E-01	SMAD3	-202819	SMAD6	160702
chr16	67514952	67515385	1.24	2.14E-01	ATP6V0D1	-80		
chr3	185826843	185827136	1.35	2.14E-01	ETV5	-89		
chr8	42698124	42698781	0.72	2.14E-01	THAP1	21		
chr17	29821258	29821595	1.53	2.15E-01	RAB11FIP4	102785	C17orf79	364899
chr17	7464320	7464768	0.74	2.15E-01	SENP3	-775		
chr11	47884755	47885191	1.28	2.15E-01	PTPRJ	-117137	NUP160	-14916
chr2	8821022	8821627	1.48	2.15E-01	ID2	-659		
chr18	48680276	48680683	1.31	2.15E-01	MEX3C	43210	SMAD4	123897
chr8	71314252	71314684	0.76	2.16E-01	PRDM14	-330906	NCOA2	1552
chr20	305555	306150	0.70	2.16E-01	SOX12	-386		
chr22	26931457	26931804	0.79	2.16E-01	TFIP11	-23194	TPST2	29739
chr19	47730312	47730588	1.35	2.16E-01	BBC3	5573	SAE1	96370
chr7	150755032	150755318	1.25	2.17E-01	SLC4A2	-1482	CDK5	-179
chr10	98460450	98460760	0.74	2.17E-01	TM9SF3	-113796	PIK3AP1	19674
chr14	107239538	107239913	0.82	2.17E-01	ADAM6	-801368	IGHV7-81	43557
chr1	40264900	40265245	0.91	2.17E-01	BMP8B	-10540	TRIT1	84104
chr7	102072284	102072569	0.71	2.17E-01	ORAI2	-1569		
chr15	90602764	90603121	1.41	2.17E-01	IDH2	42765	ZNF710	58191
chr20	42839327	42839774	1.37	2.17E-01	JPH2	-23333	FITM2	100338
chr7	45026237	45026555	0.67	2.17E-01	CCM2	-13391	MYO1G	-7692
chr2	231069780	231070125	1.53	2.18E-01	SLC16A14	-136334	SP110	14874
chr17	78388854	78389134	1.27	2.18E-01	FLJ35220	27		
chr17	7146209	7146487	1.38	2.18E-01	PHF23	-3523	GABARAP	-595
chr14	62019622	62019923	1.32	2.18E-01	HIF1A	-142346	PRKCH	231258
chr3	113233916	113234246	1.27	2.18E-01	CCDC52	-47		
chr2	70120893	70121213	1.45	2.18E-01	SNRNP27	-37		
chr19	59025265	59025559	1.46	2.18E-01	SLC27A5	-1980		
chr9	3526215	3526673	0.78	2.18E-01	RFX3	-461		
chr1	149870947	149871223	1.36	2.19E-01	BOLA1	-70		
chr7	150924214	150924598	1.38	2.19E-01	ABCF2	-89		

chr1	231347112	231347461	1.47	2.19E-01	GNPAT	-29632	TRIM67	48613
chr17	78120892	78121178	1.25	2.19E-01	EIF4A3	-97		
chr1	26868400	26868682	0.73	2.19E-01	RPS6KA1	-3802		
chr4	169753034	169753283	0.72	2.19E-01	CBR4	178263	PALLD	334942
chr12	7060255	7060580	0.72	2.19E-01	PTPN6	4678	PHB2	19498
chr17	74178371	74178673	1.43	2.19E-01	FOXJ1	-41877	RNF157	57868
chr22	26879664	26879938	1.35	2.19E-01	HPS4	-4250	SRRD	-49
chr13	92000690	92000997	1.34	2.19E-01	GPC5	-50091		
chr6	36648227	36648503	1.51	2.19E-01	CDKN1A	1906	CPNE5	158855
chr17	43394678	43395279	1.36	2.19E-01	MAP3K14	-565		
chr7	106810117	106810442	1.36	2.19E-01	HBP1	820		
chr4	89241842	89242155	1.35	2.20E-01	HERC6	-57892	PPM1K	-36111
chr7	66767467	66767900	0.61	2.20E-01	TYW1	305867		
chr6	44306308	44306588	0.83	2.20E-01	CDC5L	-48854	AARS2	-25385
chr10	111985489	111985780	1.38	2.20E-01	MXI1	18272	SMNDC1	79072
chr9	116341172	116341575	0.68	2.20E-01	ZNF618	-297188	RGS3	134363
chr6	26056415	26056792	0.76	2.20E-01	HIST1H1C	95		
chr6	43337200	43337560	0.64	2.20E-01	ZNF318	-199		
chr14	50065423	50065983	0.75	2.20E-01	PPIL5	288		
chr2	148778301	148778654	1.43	2.21E-01	ORC4L	658		
chr7	23571623	23571897	0.65	2.21E-01	TRA2A	-104		
chr16	2009369	2009702	1.38	2.21E-01	RPL3L	-4857	NDUFB10	19
chr16	72206248	72206579	0.89	2.21E-01	PMFBP1	-350		
chr6	6008747	6009090	0.63	2.21E-01	NRN1	-1286		
chr4	107237413	107237723	0.88	2.21E-01	TBCK	-145	AIMP1	-96
chr10	104941071	104941315	1.54	2.21E-01	NT5C2	11863	CNNM2	263079
chr7	50485788	50486041	0.69	2.21E-01	FIGNL1	32173	IKZF1	141537
chr11	117820975	117821341	1.66	2.21E-01	IL10RA	-35948	TMPRSS13	-21043
chr1	25345925	25346229	1.35	2.22E-01	RUNX3	-89307	SYF2	212936
chr4	77997387	77997641	1.37	2.22E-01	CCNI	-389		
chr11	44622623	44622867	1.35	2.22E-01	TSPAN18	-305213	CD82	35604
chr17	1619735	1620015	1.26	2.23E-01	PRPF8	-31699	SERPINF2	-26255
chr3	188139653	188140021	1.45	2.23E-01	TPRG1	-749926	LPP	209116
chr19	39926509	39926763	1.38	2.23E-01	RPS16	-18		
chr6	28909283	28909559	1.58	2.23E-01	TRIM27	-17653	ZNF311	63614
chr1	16694148	16694458	0.73	2.23E-01	FBXO42	-15355	SPATA21	69616
chr6	32163530	32163974	0.70	2.23E-01	GPSM3	-452		
chr12	1900326	1900740	0.64	2.23E-01	LRTM2	-28900	ADIPOR2	100286

chr2	118813680	118813990	1.44	2.23E-01	INSIG2	-32215	DDX18	241580
chr19	54704448	54704764	1.26	2.23E-01	RPS9	-120		
chr2	112463388	112463939	1.51	2.23E-01	ANAPC1	178077	BCL2L11	585173
chr3	44379780	44380402	0.74	2.23E-01	C3orf23	147		
chr17	17876025	17876344	1.26	2.23E-01	TOM1L2	-401	LRRC48	58
chr8	107282205	107282520	1.93	2.24E-01	OXR1	-110		
chr1	110927807	110928182	0.73	2.24E-01	SLC16A4	5641	RBM15	46050
chr19	13056398	13056806	1.42	2.24E-01	RAD23A	-52		
chr17	16440117	16440467	0.69	2.24E-01	ZNF287	32228	C17orf45	97991
chr21	46707792	46708036	1.32	2.24E-01	POFUT2	-103		
chr18	58546001	58546275	1.29	2.25E-01	CDH20	-611497	MC4R	-506137
chr8	67039017	67039352	0.77	2.25E-01	TRIM55	-93		
chr11	57424909	57425307	1.41	2.25E-01	CLP1	-108		
chr19	7402696	7403015	0.75	2.25E-01	INSR	-108845	ARHGEF18	-101718
chr5	131722310	131722672	0.88	2.25E-01	SLC22A5	17090	IRF1	103974
chr7	40174069	40174581	0.73	2.25E-01	C7orf10	-250	C7orf11	-67
chr10	71267654	71267898	0.74	2.25E-01	TSPAN15	56550	NEUROG3	65346
chr3	14473690	14474139	1.29	2.25E-01	SLC6A6	29809	GRIP2	109673
chr19	37663509	37663870	1.31	2.25E-01	ZNF585A	-75		
chr8	24224813	24225202	1.51	2.26E-01	ADAMDEC1	-16790	ADAM28	73428
chr13	91999681	92000082	1.28	2.26E-01	GPC5	-51053		
chr13	114992263	114992523	0.83	2.26E-01	RASA3	-94298	CDC16	-7969
chr22	27068557	27069182	1.46	2.26E-01	CRYBA4	50942		
chr8	87354558	87354838	1.20	2.26E-01	WWP1	-296		
chr19	39390192	39390686	1.30	2.27E-01	NFKBIB	-176	SIRT2	-78
chr13	41635468	41635891	0.85	2.27E-01	WBP4	-17		
chr22	42062675	42062937	1.34	2.27E-01	NHP2L1	22107	XRCC6	45511
chr3	114866429	114866689	1.59	2.27E-01	ZBTB20	-432		
chr16	70834926	70835218	1.24	2.28E-01	VAC14	-11		
chr2	65357296	65357570	1.29	2.28E-01	RAB1A	2		
chr1	155145667	155145927	1.35	2.28E-01	TRIM46	-563	KRTCAP2	7
chrX	1620174	1620452	0.71	2.28E-01	ASMTL	-48469	P2RY8	35724
chr4	26859124	26859430	1.29	2.28E-01	STIM2	-3087		
chr6	52528496	52528786	0.68	2.28E-01	TRAM2	-86779	TMEM14A	-7243
chr8	42010138	42010499	1.43	2.28E-01	AP3M2	-145		
chr1	99337084	99337443	1.26	2.28E-01	PAP2D	133185	SNX7	210028
chr8	37593998	37594260	1.28	2.28E-01	ERLIN2	32		
chr7	22893638	22893990	1.20	2.28E-01	TOMM7	-31393	FAM126A	159956

chr5	180478018	180478328	0.77	2.28E-01	OR2V2	-103770	BTNL9	10948
chr6	159290906	159291160	1.29	2.28E-01	OSTCL	-12369	TAGAP	175151
chr6	33176018	33176409	0.65	2.29E-01	RING1	-72		
chr1	24517448	24517770	0.71	2.29E-01	IL28RA	-3858		
chr12	7052963	7053554	1.62	2.29E-01	PTPN6	-2481		
chr3	170587839	170588223	1.41	2.29E-01	RPL22L1	14		
chr14	65785421	65785701	0.61	2.29E-01	MAX	-216334	FUT8	-93974
chr14	91885152	91885435	0.76	2.30E-01	CCDC88C	-1161		
chr7	64838627	64839053	0.72	2.30E-01	ZNF92	72		
chr10	104401692	104402136	0.68	2.30E-01	TRIM8	-2338		
chr12	52512102	52512529	1.30	2.30E-01	C12orf44	48558	KRT80	73468
chr2	120934714	120935099	0.82	2.30E-01	TMEM185A	46077	EPB41L5	164238
chr11	111250284	111250661	1.26	2.30E-01	POU2AF1	-316		
chr20	30102012	30102457	1.25	2.30E-01	HM13	-6		
chr20	60876886	60877196	0.75	2.30E-01	ADRM1	-986		
chr8	56986975	56987308	0.77	2.30E-01	RPS20	-2		
chr6	44144881	44145125	1.48	2.31E-01	SLC29A1	-46293	CAPN11	18455
chr19	38405587	38405977	0.86	2.31E-01	SIPA1L3	7914	DPF1	309108
chr15	86232926	86233236	0.82	2.31E-01	KLHL25	105108	AKAP13	309210
chr5	143550149	143550433	1.40	2.31E-01	KCTD16	-146	YIPF5	-13
chr1	211556002	211556288	1.32	2.31E-01	TRAF5	55997	RD3	110114
chr8	78042887	78043220	0.57	2.31E-01	PXMP3	-130530		
chr19	39902622	39903376	1.48	2.32E-01	PLEKHG2	-751		
chr9	37290430	37290688	0.59	2.32E-01	GRHPR	-132148	ZCCHC7	170090
chr12	129019503	129019748	1.49	2.32E-01	TMEM132C	120335	SLC15A4	288915
chr2	231068070	231068346	1.56	2.32E-01	SLC16A14	-134589	SP110	16619
chr5	78810337	78810631	1.44	2.32E-01	HOMER1	-784		
chr6	159137014	159137324	0.77	2.32E-01	SYTL3	66123	EZR	102171
chr1	155907938	155908266	0.84	2.33E-01	RXFP4	-3378		
chr19	10514147	10514428	1.42	2.33E-01	CDC37	-17		
chr22	41844435	41844794	0.66	2.33E-01	TOB2	-1588		
chr22	43486198	43486516	1.27	2.33E-01	TTLL1	-923		
chr9	130186427	130186827	1.37	2.33E-01	ZNF79	-26		
chr17	48981602	48981856	0.76	2.33E-01	TOB1	-40316	SPAG9	216497
chr19	50220791	50221101	0.67	2.33E-01	CPT1C	26545	TSKS	45569
chr2	27502543	27503053	0.71	2.33E-01	TRIM54	-2802		
chr14	51280295	51280551	0.69	2.34E-01	SAV1	-145400	NIN	17416
chr6	2765565	2765927	0.77	2.34E-01	WRNIP1	80		

chr2	95787503	95787813	0.81	2.34E-01	MRPS5	96		
chr3	193788334	193788805	1.30	2.34E-01	HES1	-65364	OPA1	477637
chr3	196594584	196594878	1.23	2.34E-01	SENP5	4		
chr1	23866268	23866578	0.68	2.34E-01	E2F2	-8710	ID3	19899
chr11	67416309	67416683	0.69	2.34E-01	TBX10	-9465	ACY3	1634
chr10	94050780	94051079	1.21	2.35E-01	CPEB3	-86	MARCH5	10
chr5	130506408	130506694	1.29	2.35E-01	LYRM7	-90		
chr17	27224244	27224715	1.26	2.35E-01	FLOT2	235		
chr9	134127974	134128284	1.55	2.35E-01	PPAPDC3	-36952	NUP214	127148
chr1	28573726	28574086	0.65	2.35E-01	SESN2	-12100	ATPIF1	11295
chr18	21498466	21498776	0.76	2.35E-01	CABYR	-220334	LAMA3	229059
chr11	111293780	111294056	1.39	2.35E-01	POU2AF1	-43761	BTG4	89146
chr10	105127568	105127864	1.48	2.36E-01	TAF5	-8		
chr18	77438964	77439557	1.39	2.36E-01	CTDP1	-540		
chr2	3383281	3383708	1.35	2.36E-01	TSSC1	-1842	TTC15	49
chr22	22465206	22465669	1.45	2.36E-01	VPREB1	-133762	TOP3B	-128291
chr6	32912182	32912449	0.83	2.36E-01	HLA-DMB	-3499		
chr10	103911849	103912186	0.70	2.36E-01	NOLC1	85		
chr15	74833146	74833480	1.38	2.36E-01	ARID3B	-235		
chr19	45393602	45393904	1.39	2.36E-01	TOMM40	-724		
chr6	159290363	159290826	0.79	2.36E-01	OSTCL	-11931	TAGAP	175589
chrX	53710991	53711470	1.18	2.36E-01	HSD17B10	-249908	HUWE1	2442
chr17	30676978	30677284	1.36	2.36E-01	ZNF207	-26		
chr12	49463623	49464019	1.17	2.37E-01	RHEBL1	-46		
chr16	47175619	47175897	0.64	2.37E-01	DNAJA2	-168133	NETO2	2150
chr15	90777191	90777466	1.19	2.37E-01	CIB1	-50		
chr7	73515445	73515770	1.36	2.37E-01	EIF4H	-73098	LIMK1	17452
chr1	236118004	236118520	1.32	2.37E-01	LYST	-88042	NID1	110219
chr15	59698669	59698963	1.36	2.37E-01	GCNT3	-205166	MYO1E	-33745
chr7	75988553	75989004	1.34	2.37E-01	YWHAG	-437		
chr4	83295105	83295540	1.32	2.37E-01	HNRNPD	-174		
chr7	24875668	24876010	0.72	2.37E-01	DFNA5	-78756	OSBPL3	143921
chr6	17706189	17706491	0.80	2.37E-01	NUP153	478		
chr9	80912310	80912761	0.81	2.37E-01	PSAT1	477		
chr1	45196923	45197257	1.50	2.37E-01	KIF2C	-8400	C1orf228	56696
chr1	181056653	181056944	1.38	2.37E-01	CACNA1E	-395917	MR1	53660
chr19	47633791	47634303	1.31	2.37E-01	SAE1	-33		
chr13	41054776	41055226	0.79	2.37E-01	FOXO1	185733	COG6	825237

chr11	111287905	111288270	1.46	2.38E-01	POU2AF1	-37931	BTG4	94976
chr22	41985661	41986207	0.69	2.39E-01	PMM1	-63		
chr14	68162223	68162590	0.70	2.39E-01	RDH11	103		
chr11	85753021	85753283	1.52	2.39E-01	SYTL2	-315641	PICALM	26956
chr5	74807513	74807818	1.25	2.39E-01	POLK	9	COL4A3BP	140
chr2	160106563	160106807	1.59	2.39E-01	WDSUB1	36551	TANC1	281539
chr12	6982385	6983021	0.73	2.39E-01	SPSB2	-254		
chr9	130668815	130669074	1.38	2.39E-01	ST6GALNAC6	-7074	ST6GALNAC4	10360
chr1	108440112	108440354	0.75	2.39E-01	VAV3	67312	NTNG1	757604
chr2	70475688	70476035	1.21	2.39E-01	TIA1	-83		
chr14	74868054	74868576	0.70	2.40E-01	TMEM90A	24490	C14orf115	53149
chr1	174968346	174968985	1.35	2.40E-01	CACYBP	-226		
chr19	2061541	2061851	0.69	2.40E-01	MKNK2	-10453	MOBKL2A	34573
chr15	41583330	41583603	1.47	2.40E-01	OIP5	41352	CHP	60030
chr20	44746746	44747112	1.41	2.41E-01	CD40	23		
chr11	18655822	18656127	0.78	2.41E-01	TMEM86A	-64376	UEVLD	-45694
chr8	56756655	56756981	0.86	2.41E-01	TMEM68	-70933	LYN	-35568
chr21	43298037	43298582	0.82	2.41E-01	RIPK4	-111061	PRDM15	1281
chrX	64754690	64754988	1.30	2.41E-01	LAS1L	-172		
chr19	13318970	13319231	1.42	2.42E-01	IER2	57819	CACNA1A	298173
chr3	169649643	169650299	1.52	2.42E-01	LRRC31	-62311	SEC62	-34609
chr3	16306254	16306682	0.76	2.42E-01	OXNAD1	-246	DPH3	28
chr12	25209603	25210034	0.80	2.42E-01	LYRM5	-138331	LRMP	4578
chr22	21983612	21983869	1.26	2.42E-01	SDF2L1	-12801	UBE2L3	61784
chr6	31515165	31515518	0.65	2.42E-01	ATP6V1G2	-715	NFKBIL1	-11
chr20	39570845	39571155	0.96	2.43E-01	MAFB	-253124	TOP1	-86462
chr19	56186441	56186699	1.26	2.43E-01	EPN1	-1421		
chr17	4843281	4843542	1.17	2.43E-01	RNF167	-218	SLC25A11	-94
chr2	27886588	27886848	1.45	2.43E-01	SUPT7L	-269	SLC4A1AP	380
chr21	45358922	45359221	1.43	2.43E-01	TRAPPC10	-73134	AGPAT3	73956
chr6	35995167	35995681	0.67	2.43E-01	SLC26A8	-3047	MAPK14	-30
chr17	38689614	38689950	1.41	2.44E-01	TNS4	-31928	CCR7	31942
chr6	84569061	84569428	1.39	2.44E-01	CYB5R4	-125		
chr3	15106705	15106983	1.37	2.44E-01	MRPS25	-28		
chr2	204103516	204103923	1.47	2.44E-01	CYP20A1	556		
chr21	46529707	46530003	1.58	2.44E-01	ADARB1	35362	POFUT2	177956
chr16	1358957	1359252	1.19	2.45E-01	UBE2I	-75		
chr11	111742148	111742424	1.54	2.45E-01	ALG9	19		

chr17	30228603	30228934	1.29	2.45E-01	UTP6	-40		
chr9	565529	565914	0.82	2.46E-01	DMRT1	-275968	KANK1	61019
chr12	133287165	133287505	1.23	2.46E-01	PGAM5	-101		
chr2	24149893	24150206	1.24	2.46E-01	ATAD2B	-116		
chr5	180648949	180649197	1.57	2.47E-01	TRIM41	-1233		
chr16	4524477	4524835	0.88	2.47E-01	HMOX2	-1685	NMRAL1	240
chr3	14443698	14444094	1.21	2.47E-01	SLC6A6	-210		
chr11	71823597	71824021	1.46	2.48E-01	C11orf51	13		
chr6	58287638	58287931	0.73	2.48E-01				
chr19	54618877	54619286	1.40	2.48E-01	TFPT	-27	PRPF31	292
chr13	28713432	28713676	1.20	2.48E-01	FLT3	-38825	PAN3	-34855
chr11	18548320	18548630	0.84	2.48E-01	TSG101	14		
chr2	74618983	74619383	0.74	2.48E-01	DCTN1	-11708	RTKN	49877
chr11	59383644	59384218	0.83	2.48E-01	OSBP	-314		
chr12	12868780	12869107	1.51	2.49E-01	CDKN1B	-1358		
chr2	183943340	183943658	1.19	2.49E-01	DUSP19	212		
chr12	96455715	96455995	1.51	2.50E-01	ELK3	-132352	LTA4H	-26490
chr4	1857812	1858248	1.20	2.50E-01	LETM1	-56		
chr5	139937032	139937320	1.26	2.50E-01	SRA1	502		
chr17	28050654	28050980	0.73	2.50E-01	GIT1	-134207	SSH2	206201
chr5	173043678	173044011	1.26	2.50E-01	BOD1	-179		
chr20	1373706	1374107	1.38	2.50E-01	FKBP1A	-91		
chr16	4401227	4401517	1.32	2.50E-01	VASN	-20477	GLIS2	19156
chr12	57081956	57082446	0.63	2.51E-01	PTGES3	-123		
chr7	139910085	139910479	1.47	2.51E-01	JHDM1D	-33541	SLC37A3	188029
chr1	84464764	84465028	0.59	2.51E-01	TTLL7	-63		
chr1	160759476	160759827	1.18	2.51E-01	LY9	-6276	SLAMF7	50575
chr22	42486854	42487167	0.74	2.51E-01	NDUFA6	-123		
chr7	35749124	35749369	0.82	2.51E-01	SEPT7	-91380	HERPUD2	-14475
chr9	114393356	114393828	1.25	2.51E-01	DNAJC25	-40		
chr19	46087818	46088445	1.36	2.52E-01	OPA3	-10		
chr6	53174352	53174777	0.76	2.52E-01	GCM1	-160941	ELOVL5	39377
chr9	125987676	125988057	1.27	2.53E-01	STRBP	42976	GPR21	191021
chr8	11275375	11275771	0.69	2.53E-01	BLK	-75948	AMAC1L2	87078
chr16	30934301	30934789	1.54	2.54E-01	FBXL19	-1351		
chr7	157179905	157180201	1.51	2.54E-01	DNAJB6	50343		
chr7	56019380	56019693	0.75	2.54E-01	GBAS	-12759	ZNF713	39209
chr11	6640537	6640908	0.83	2.54E-01	TPP1	-31		

chr6	53109827	53110137	0.75	2.54E-01	GCM1	-96358	ELOVL5	103960
chr15	75917977	75918302	0.82	2.54E-01	SNUPN	579		
chr21	34681110	34681473	0.72	2.54E-01	IFNAR1	-15922	IFNAR2	79061
chr11	72526751	72527074	0.77	2.55E-01	ATG16L2	1462	FCHSD2	326230
chr16	68318835	68319145	0.77	2.55E-01	PRMT7	-25955	SLC7A6	20567
chr3	107843250	107843507	1.68	2.55E-01	CD47	-33444	IFT57	98038
chr1	144932168	144932581	1.36	2.55E-01	PPIAL4A	-568129	PDE4DIP	62647
chr11	61124649	61124978	1.43	2.56E-01	CYBASC3	-453		
chr15	63795442	63795738	1.22	2.56E-01	USP3	-1220		
chr10	63759903	63760260	1.23	2.56E-01	ARID5B	98639	RTKN2	268384
chr2	220114263	220114575	0.82	2.57E-01	GLB1L	-4288	TUBA4B	-3546
chr2	201828370	201828615	1.42	2.57E-01	ORC2L	-83		
chr1	110359152	110359426	1.49	2.57E-01	CSF1	-93944	EPS8L3	-52725
chr13	28021112	28021422	0.83	2.57E-01	MTIF3	3444	GTF3A	22586
chr2	86422460	86422746	1.42	2.57E-01	MRPL35	-3953	IMMT	290
chr19	49956610	49957004	1.22	2.57E-01	PIH1D1	-1692	ALDH16A1	334
chr20	52346132	52346450	1.24	2.57E-01	ZNF217	-146584	BCAS1	341013
chr11	57298174	57298565	1.35	2.58E-01	TIMM10	-138		
chr9	100684720	100684992	1.24	2.58E-01	C9orf156	-4		
chr1	41174829	41175199	0.73	2.58E-01	KCNQ4	-74670	NFYC	17772
chr3	142937695	142938012	1.47	2.58E-01	CHST2	99186	SLC9A9	629492
chr5	149380057	149380301	1.39	2.58E-01	HMGXB3	10	TIGD6	38
chr11	36310856	36311166	1.34	2.58E-01	PRR5L	-86524	LDLRAD3	345399
chr3	108308096	108308736	0.75	2.58E-01	KIAA1524	75	DZIP3	79
chr3	9773077	9773509	1.24	2.59E-01	BRPF1	-141		
chr16	87756893	87757203	1.39	2.59E-01	KLHDC4	42494	JPH3	120549
chr13	46038828	46039126	1.21	2.59E-01	COG3	-94		
chr19	1076978	1077327	1.33	2.59E-01	HMHA1	9979	POLR2E	18238
chr9	116385177	116385549	1.38	2.59E-01	ZNF618	-253199	RGS3	178352
chr17	74523657	74523928	1.23	2.59E-01	RHBDF2	-26285	CYGB	9989
chr6	26538190	26538685	0.78	2.59E-01	HMGN4	-134		
chr22	43019512	43019863	1.17	2.59E-01	POLDIP3	-8726	CYB5R3	25717
chr13	47180364	47180664	1.48	2.59E-01	LRCH1	53218	ESD	190853
chr7	2178023	2178299	1.17	2.59E-01	MAD1L1	94422	ELFN1	429363
chr22	43507211	43507500	1.20	2.59E-01	BIK	599		
chr21	47706054	47706329	1.39	2.60E-01	MCM3AP	-956	C21orf57	-75
chr19	42364092	42364587	1.36	2.60E-01	RPS19	352		
chr19	58912647	58913017	1.10	2.60E-01	ZNF584	-7231	RPS5	14196

chr3	183966585	183966906	1.36	2.60E-01	ECE2	-699	ALG3	13
chr6	88589276	88589649	1.26	2.60E-01	AKIRIN2	-177478	SPACA1	-168044
chr5	157156938	157157188	1.81	2.61E-01	THG1L	-1260		
chr17	27046791	27047150	1.37	2.61E-01	RAB34	-1685	RPL23A	-29
chr6	42710160	42710524	0.80	2.61E-01	PRPH2	-19984	TBCC	3542
chr5	151129200	151129448	1.11	2.61E-01	SPARC	-62807	ATOX1	8886
chr3	179065217	179065628	1.25	2.61E-01	MFN1	-57		
chr1	93645865	93646270	0.66	2.62E-01	TMED5	178		
chr1	16212769	16213013	0.64	2.62E-01	SPEN	38532	ZBTB17	89736
chr14	92905985	92906268	1.24	2.63E-01	RIN3	-73998	SLC24A4	115975
chr19	46061727	46062014	1.27	2.63E-01	OPA3	26251	VASP	51183
chr3	133291995	133292239	1.42	2.63E-01	CDV3	-317		
chr11	65307652	65307968	0.70	2.63E-01	SCYL1	15262	LTBP3	17889
chr20	30628173	30628502	1.33	2.63E-01	HCK	-11719	XKR7	72533
chr19	41769355	41770068	0.64	2.63E-01	HNRNPUL1	-408		
chr10	70091408	70091922	0.72	2.63E-01	HNRNPH3	-103		
chr1	11969598	11970034	1.38	2.63E-01	NPPB	-50824	KIAA2013	16664
chr2	27304099	27304358	1.33	2.63E-01	KHK	-5382	EMILIN1	2723
chr16	28835318	28835753	1.23	2.63E-01	ATXN2L	1122	TUFM	22193
chr6	44064426	44064744	0.71	2.63E-01	MRPL14	30606	VEGFA	326632
chr18	12990958	12991268	0.66	2.63E-01	CEP192	-248		
chr3	169846336	169846693	1.39	2.63E-01	PHC3	53022	GPR160	90780
chr12	62996583	62996938	1.31	2.63E-01	MON2	136164	PPM1H	331904
chr3	15140551	15140829	1.33	2.63E-01	ZFYVE20	-35		
chr1	203734096	203734406	0.85	2.63E-01	LAX1	-33		
chr2	55920812	55921272	1.57	2.63E-01	PNPT1	-31		
chr3	42641778	42642097	1.31	2.63E-01	NKTR	-209		
chr15	45490702	45491038	1.33	2.63E-01	SHF	2503	DUOX1	68678
chr3	172468239	172468580	1.42	2.64E-01	ECT2	-3889		
chr1	222628509	222628767	1.45	2.64E-01	DUSP10	-713177	HHIPL2	92806
chr19	45681425	45681937	1.20	2.64E-01	BLOC1S3	-322	TRAPPC6A	-196
chr2	99797345	99797743	1.35	2.64E-01	MITD1	-52	MRPL30	-34
chr7	112580019	112580314	1.31	2.65E-01	TMEM168	-149689	GPR85	146375
chr7	129592502	129593063	1.18	2.65E-01	UBE2H	6		
chr4	378151	378725	0.68	2.65E-01	PIGG	-114551	ZNF141	46842
chr5	139944168	139944434	1.41	2.65E-01	SLC35A4	-119	APBB3	-112
chr7	65579686	65579934	0.82	2.65E-01	CRCP	5		
chr1	228290942	228291229	1.49	2.66E-01	MRPL55	5927	ARF1	20725

chr1	160001690	160001975	1.24	2.66E-01	PIGM	-50		
chr19	50919005	50919246	1.36	2.66E-01	SPIB	-3069		
chr6	31802210	31802820	0.77	2.66E-01	HSPA1B	7003	NEU1	28194
chr5	112312085	112312425	1.46	2.67E-01	DCP2	-178		
chr4	114468726	114469129	1.31	2.67E-01	CAMK2D	214155	ANK2	498143
chr14	90187142	90187542	0.70	2.67E-01	FOXN3	-101848	C14orf143	233747
chr6	133135307	133135690	0.73	2.67E-01	RPS12	-209		
chr7	11251802	11252087	0.67	2.67E-01	PHF14	238429	THSD7A	619879
chr11	96076387	96076697	1.40	2.68E-01	MAML2	-198		
chr4	159593138	159593792	1.37	2.68E-01	ETFDH	188		
chr1	16302485	16302785	1.37	2.68E-01	ZBTB17	-8		
chr12	54673825	54674135	1.40	2.68E-01	HNRNPA1	-508		
chr12	101673741	101673989	1.38	2.68E-01	UTP20	-40		
chr3	158362100	158362560	1.39	2.68E-01	GFM1	13		
chr4	113152520	113152774	1.36	2.68E-01	AP1AR	-248		
chr4	119512655	119512965	0.81	2.68E-01	METTL14	-93764	CEP170L	75315
chr13	31182068	31182364	1.48	2.69E-01	HMGB1	-142135	USPL1	-9614
chr17	56494892	56495216	1.20	2.69E-01	RNF43	-123		
chr18	47814018	47814493	1.45	2.70E-01	CXXC1	436		
chr5	37371067	37371320	1.31	2.70E-01	NUP155	3		
chr1	167684697	167684987	1.44	2.70E-01	CREG1	-161786	MPZL1	-6345
chr6	126102026	126102414	0.79	2.70E-01	NCOA7	-9781	HEY2	31488
chr19	13990971	13991363	1.32	2.70E-01	NANOS3	3217	C19orf57	25742
chr3	71493178	71493460	0.72	2.70E-01	FOXP1	139821		
chr1	244898763	244899096	0.73	2.70E-01	FAM36A	-99709	C1orf101	274257
chr2	158380286	158380613	0.80	2.70E-01	CYTIP	-79846	ACVR1C	104949
chr22	19406777	19407087	0.68	2.70E-01	CLTCL1	-127693	HIRA	12287
chr6	42738646	42739117	0.79	2.70E-01	RPL7L1	-108789	TBCC	-24998
chr1	245205998	245206308	1.47	2.70E-01	KIF26B	-112134	EFCAB2	72982
chr7	158387819	158388029	0.49	2.70E-01	PTPRN2	-7442	NCAPG2	109596
chr19	51307737	51308174	0.83	2.70E-01	ACPT	14284	KLK1	19087
chr17	46018622	46018979	0.86	2.71E-01	PNPO	-88		
chr21	35296373	35296665	0.66	2.71E-01	SLC5A3	-149304	ITSN1	281735
chr6	30689079	30689385	0.76	2.71E-01	MDC1	-3774	TUBB	1075
chr19	40476639	40477087	1.29	2.71E-01	PSMC4	-210		
chr6	1739923	1740219	1.44	2.71E-01	FOXC1	129390	GMDS	505775
chr9	136811592	136811845	1.52	2.71E-01	SARDH	-208240	VAV2	45727
chr11	114309944	114310189	1.32	2.71E-01	REXO2	-41		

chr4	146019082	146019533	1.27	2.71E-01	ANAPC10	60	ABCE1	152
chr11	67771552	67771796	1.42	2.72E-01	UNC93B1	-81		
chr2	55746558	55746883	1.22	2.72E-01	CCDC88A	-99664	SMEK2	98392
chr19	50934512	50934879	1.30	2.72E-01	MYBPC2	-1464		
chr19	50083729	50084086	1.40	2.72E-01	PRRG2	-679	NOSIP	-105
chr16	15949188	15949720	0.87	2.72E-01	MYH11	1433	NDE1	205371
chr11	67374227	67374514	1.31	2.72E-01	NDUFV1	48		
chr3	129143138	129143475	1.56	2.72E-01	H1FX	-108187	MBD4	15545
chr10	102241286	102241591	0.66	2.73E-01	WNT8B	18627	SEC31B	38156
chr17	7212213	7212545	0.73	2.73E-01	EIF5A	2061	GPS2	6595
chr8	38240334	38240927	0.84	2.73E-01	LETM2	-3389	WHSC1L1	-841
chr12	49208377	49208683	0.70	2.73E-01	CACNB3	-3982		
chr16	30483820	30484181	0.77	2.73E-01	ITGAL	18		
chr13	77913296	77913725	0.81	2.74E-01	SCEL	-196298	MYCBP2	-12334
chr22	23408742	23409009	1.49	2.75E-01	GNAZ	-3793		
chr6	43196933	43197360	0.75	2.75E-01	C6orf108	64		
chr19	48867103	48867893	1.39	2.75E-01	TMEM143	-312	SYNGR4	-159
chr12	104350850	104351449	1.37	2.75E-01	C12orf73	-157		
chr11	73000496	73000806	1.30	2.75E-01	ARHGEF17	-19012	P2RY6	17404
chr2	136996389	136996712	1.26	2.76E-01	THSD7B	-751911	CXCR4	-120826
chr20	10415129	10415380	1.19	2.76E-01	C20orf94	-696	MKKS	-389
chr7	106505687	106506015	0.84	2.76E-01	PIK3CG	-73		
chr5	10607763	10608307	1.22	2.76E-01	DAP	153352	ROPN1L	166026
chr8	145133322	145133632	1.45	2.76E-01	GPAA1	-4047	EXOSC4	-45
chrX	2439360	2439710	1.59	2.76E-01	CD99	-169693	ZBED1	-20520
chr19	52097551	52097955	1.19	2.76E-01	hCG_2008157	-123		
chr19	6587374	6587811	0.69	2.76E-01	CD70	3570	TNFSF9	56583
chr7	104624269	104624707	0.71	2.77E-01	MLL5	-30149	LHFPL3	655384
chr3	112361804	112362088	1.45	2.77E-01	CCDC80	-1969		
chr6	24522639	24523067	0.65	2.77E-01	ALDH5A1	27656	KIAA0319	123530
chr6	25992773	25993020	0.78	2.78E-01	HIST1H1A	25143	TRIM38	29826
chr16	89038229	89038523	1.35	2.78E-01	LOC390748	-105362	CBFA2T3	5025
chr17	4167086	4167566	0.81	2.78E-01	ANKFY1	-52		
chr14	76127436	76127682	1.24	2.78E-01	TTLL5	-62	C14orf1	-27
chr17	1990974	1991294	1.46	2.78E-01	HIC1	31530	SMG6	215935
chrX	102941505	102941904	1.34	2.78E-01	MORF4L2	1381	TCEAL1	57813
chr16	85752267	85752650	1.38	2.78E-01	GINS2	-29871	COX4NB	80689
chr11	65337422	65338041	1.47	2.78E-01	SSSCA1	-211		

chr1	165797311	165797621	0.81	2.79E-01	UCK2	576		
chr8	1840636	1840892	1.60	2.79E-01	KBTBD11	-81280	ARHGEF10	68615
chr1	182602970	182603248	1.18	2.79E-01	RGS16	-29561	RGS8	38958
chr13	46951686	46951996	1.44	2.79E-01	C13orf18	9794	LRRC63	165758
chr8	41402595	41402932	0.67	2.79E-01	AGPAT6	-32943	GINS4	16039
chr1	224180230	224180474	1.35	2.79E-01	DEGS1	-190576	TP53BP2	-146678
chr4	113208287	113208597	0.75	2.80E-01	TIFA	-1383		
chr5	154134404	154134809	0.71	2.80E-01	LARP1	-282		
chr6	153304437	153304757	1.21	2.80E-01	FBXO5	-394		
chr3	27410853	27411163	0.71	2.80E-01	NEK10	-96		
chr3	10431881	10432205	0.80	2.80E-01	SEC13	-69185	ATP2B2	115225
chr7	5258265	5258541	0.79	2.80E-01	SLC29A4	-64158	WIPI2	28568
chr1	203764539	203764830	1.34	2.80E-01	ZC3H11A	-66		
chr8	125551194	125551527	0.72	2.80E-01	TATDN1	-32	NDUFB9	18
chr10	112115795	112116079	1.38	2.81E-01	DUSP5	-141688	SMNDC1	-51230
chr1	165599940	165600339	1.48	2.81E-01	MGST3	-310		
chr11	118901492	118901895	1.34	2.81E-01	SLC37A4	-78		
chr1	156391377	156391713	1.17	2.81E-01	C1orf61	7795	RHBG	52542
chr8	103819759	103820027	0.93	2.81E-01	KLF10	-151910	AZIN1	56504
chr22	22711710	22712039	1.37	2.81E-01	VPREB1	112675	ZNF280B	151630
chr11	77705662	77706312	1.38	2.81E-01	INTS4	-270		
chr3	128995287	128995637	1.34	2.81E-01	COPG	27009	H1FX	39658
chr9	115983403	115983821	1.16	2.81E-01	SLC31A1	-196	FKBP15	64
chr6	159525193	159525433	0.65	2.81E-01	FNDC1	-65116	TAGAP	-59129
chr22	41682854	41683130	1.47	2.81E-01	RANGAP1	-776		
chr16	23345784	23346190	0.92	2.82E-01	SCNN1B	32396	COG7	118516
chr2	61108002	61108364	1.28	2.82E-01	REL	-569		
chr20	44718323	44718744	1.28	2.83E-01	NCOA5	46		
chr7	144532949	144533313	1.31	2.83E-01	TPK1	15		
chr3	32501621	32501963	0.72	2.83E-01	CMTM6	42611	CMTM7	68629
chr9	2242456	2242732	1.24	2.83E-01	VLDLR	-379199	SMARCA2	227252
chr2	68668812	68669146	1.42	2.83E-01	APLF	-25712	PLEK	76657
chr1	231664065	231664380	0.84	2.83E-01	TSNAX	-176		
chr5	139781279	139781616	1.23	2.83E-01	ANKHD1-EIF4EBP3	19		
chr2	10885743	10886021	1.45	2.83E-01	ATP6V1C2	24107	PDIA6	67078
chr11	47198356	47198751	1.20	2.84E-01	ARFGAP2	-135		
chr3	187960190	187960551	1.33	2.84E-01	TPRG1	-929392	LPP	29650
chr22	24256082	24256514	1.41	2.84E-01	BC036909	-15181	GSTT2	47095

chr6	36409218	36409564	0.74	2.84E-01	KCTD20	-1153				
chr12	53574198	53574458	1.38	2.85E-01	ZNF740	-207	CSAD	102		
chr8	67837592	67837890	0.66	2.85E-01	C8orf45	54757	LRRC67	103045		
chr20	56051219	56051491	0.79	2.85E-01	HMGB1L1	12728	RBM38	84892		
chr4	115524674	115524936	1.35	2.85E-01	UGT8	5194	NDST4	510227		
chr7	7680161	7680595	0.65	2.85E-01	COL28A1	-104918	RPA3	77860		
chr6	156885557	156885814	0.65	2.86E-01	ARID1B	-213400				
chr19	11545831	11546141	0.81	2.86E-01	PRKCSH	-283				
chr15	67385835	67386208	0.71	2.86E-01	SMAD3	27827	AAGAB	161052		
chr19	1258983	1259447	1.34	2.86E-01	CIRBP	-10052	MIDN	10663		
chr14	21457972	21458251	1.34	2.86E-01	METT11D1	147				
chr6	167527397	167527728	0.72	2.86E-01	CCR6	2268	GPR31	43756		
chr7	123197778	123198035	1.31	2.87E-01	NDUFA5	51				
chr16	89787213	89787491	1.42	2.87E-01	ZNF276	-600	C16orf7	42		
chr9	130829989	130830340	1.22	2.87E-01	NAIF1	-566	SLC25A25	-314		
chr12	14410571	14410990	1.36	2.87E-01	GRIN2B	-277759	ATF7IP	-107830		
chr6	10694734	10695242	0.71	2.88E-01	PAK1IP1	-200				
chr14	77787567	77787820	1.31	2.88E-01	POMT2	-469	GSTZ1	464		
chr16	11402721	11403050	1.21	2.88E-01	C16orf75	-36425	PRM1	-27694		
chr22	50946488	50946762	1.06	2.88E-01	LMF2	-490	NCAPH2	-20		
chr7	98976914	98977172	0.70	2.88E-01	ARPC1B	4714	PDAP1	29249		
chr2	62266496	62266783	1.53	2.88E-01	B3GNT2	-156622	COMMD1	133837		
chr2	241981203	241981513	0.76	2.88E-01	SNED1	43103	PASK	107520		
chr17	17739457	17739833	1.23	2.89E-01	SREBF1	680				
chr11	64889536	64889838	1.30	2.89E-01	ZNHIT2	-4517	MRPL49	-41	FAU	-27
chr3	58626403	58626651	0.65	2.89E-01	FAM107A	-63036	FAM3D	26034		
chr19	42787734	42788233	1.42	2.89E-01	CIC	-833				
chr2	47630155	47630465	1.41	2.89E-01	MSH2	47				
chr6	32098026	32098325	0.77	2.89E-01	ATF6B	-2159	FKBPL	-109		
chr8	28258590	28259008	1.17	2.89E-01	FZD3	-92974	ZNF395	-14822		
chr9	139131842	139132086	1.25	2.89E-01	LHX3	-36960	QSOX2	5723		
chr5	115420964	115421285	1.28	2.90E-01	COMMD10	398				
chr1	174998888	174999198	1.41	2.90E-01	TNN	-37951	MRPS14	-6482		
chr6	43635032	43635276	0.68	2.90E-01	MRPS18A	20374	RSPH9	22344		
chr1	156698481	156698773	1.56	2.91E-01	ISG20L2	-922	C1orf66	364		
chr11	63448726	63449017	1.24	2.91E-01	RTN3	-50				
chr17	7961104	7961388	1.37	2.91E-01	ALOX15B	18888	ALOX12B	29775		
chr16	81855119	81855465	0.73	2.91E-01	PLCG2	42362	SDR42E1	189801		

chr2	6483173	6483563	1.25	2.91E-01	CMPK2	522568	SOX11	650569
chr3	186524086	186524599	1.34	2.92E-01	RFC4	-53		
chr19	7587182	7587513	1.29	2.92E-01	MCOLN1	-164		
chr6	30028828	30029113	0.87	2.92E-01	ZNRD1	-65		
chr16	3208775	3209019	1.73	2.92E-01	OR1F1	-45350	caspace	14677
chr8	12622846	12623158	1.41	2.92E-01	C8orf79	-180181	LONRF1	-10010
chr9	93589455	93589790	1.51	2.92E-01	SYK	25611	AUH	534583
chr21	43483739	43484146	0.66	2.92E-01	ZNF295	-53447	UMODL1	-7483
chr13	25875595	25875893	1.38	2.92E-01	NUPL1	78		
chr22	46983602	46983912	1.29	2.92E-01	CELSR1	-50690	GRAMD4	-38891
chr18	52989000	52989236	0.76	2.93E-01	TCF4	266742	RAB27B	493278
chr16	80574675	80574919	1.33	2.93E-01	DYNLRB2	-57		
chr17	37950319	37950914	1.65	2.93E-01	GRB7	56430	IKZF3	69824
chr9	5438827	5439111	0.81	2.93E-01	C9orf46	-1109		
chr11	19454095	19454405	1.10	2.93E-01	NAV2	-280631	E2F8	-191743
chr10	12110619	12110924	1.19	2.93E-01	DHTKD1	-162		
chr13	74864521	74864856	0.83	2.93E-01	KLF12	-156623		
chr17	4736525	4736775	1.33	2.93E-01	MINK1	-33		
chr2	74681897	74682269	1.51	2.94E-01	WBP1	-3494	INO80B	-116
chr3	187954145	187954525	0.77	2.94E-01	TPRG1	-935428	LPP	23614
chr19	39935910	39936379	1.32	2.94E-01	SUPT5H	-41		
chr9	98224847	98225167	1.26	2.94E-01	FANCC	-145016	PTCH1	45824
chr2	9747966	9748378	1.45	2.94E-01	ADAM17	-52255	YWHAQ	22934
chr9	133454786	133455041	1.39	2.95E-01	FUBP3	-46		
chr15	91361868	91362184	1.22	2.95E-01	FURIN	-49859	BLM	101447
chr12	67835626	67835877	0.87	2.95E-01	DYRK2	-206760	CAND1	172691
chr13	45563493	45563810	1.47	2.95E-01	NUFIP1	-39		
chr15	64943784	64944060	0.66	2.95E-01	OAZ2	51540	ZNF609	152303
chr7	148638097	148638766	1.36	2.96E-01	EZH2	-57018	PDIA4	87350
chr9	34899625	34899869	0.69	2.96E-01	C9orf144B	-170212	KIAA1045	-58445
chr15	93374913	93375360	1.39	2.96E-01	FAM174B	-176106	CHD2	-68414
chr3	122512570	122512844	1.38	2.96E-01	DIRC2	-1194	HSPBAP1	-57
chr15	75081089	75081498	1.43	2.96E-01	LMAN1L	-23900	CSK	6869
chr20	30795520	30795764	1.09	2.97E-01	PLAGL2	-96	POFUT1	-54
chr11	33942800	33943168	1.32	2.97E-01	CAPRIN1	-130246	LMO2	-29148
chr1	22366310	22366642	0.70	2.97E-01	CDC42	-12644	CELA3B	63058
chr5	43008138	43008464	0.80	2.97E-01	C5orf39	32146	GHR	584275
chr12	93381773	93382085	1.20	2.98E-01	NUDT4	-389772	EEA1	-58822

chr3	183903412	183903899	1.30	2.98E-01	ABCF3	-207		
chr8	74888329	74888573	0.70	2.98E-01	TCEB1	-4105	TMEM70	21
chr7	100136287	100136808	1.31	2.98E-01	AGFG2	-286		
chr15	57998684	57999026	1.28	2.98E-01	GCOM1	114741	ALDH1A2	359051
chr15	67373166	67373811	1.36	2.98E-01	SMAD3	15294	AAGAB	173585
chr22	43263735	43264041	1.22	2.99E-01	ARFGAP3	-10480	PACSIN2	79159
chr10	6205504	6205910	1.07	2.99E-01	PFKFB3	-39133	RBM17	74758
chr15	90590410	90590655	1.30	2.99E-01	ZNF710	45781	IDH2	55175
chr19	45157725	45157973	1.32	2.99E-01	CEACAM19	-16875	PVR	10751
chr2	238877293	238877606	1.21	2.99E-01	SCLY	-92182	UBE2F	1750
chr12	57852742	57853050	0.72	2.99E-01	GLI1	-1022		
chr4	40307149	40307646	1.41	2.99E-01	CHRNA9	-30071	RHOH	108871
chr4	6641943	6642395	1.28	2.99E-01	MRFAP1	-276		
chr9	131266802	131267052	1.20	2.99E-01	GLE1	-44		
chr6	31125943	31126435	0.73	3.00E-01	CCHCR1	-623	TCF19	-114
chr11	67252373	67252864	0.81	3.00E-01	AIP	2114	PITPNM1	20224
chr8	123799976	123800291	0.70	3.00E-01	ZHX2	6233	DERL1	254514
chr7	35734669	35734996	0.66	3.00E-01	HERPUD2	-61		
chr3	127293758	127294086	1.22	3.00E-01	TPRA1	15646	PLXNA1	586420
chr15	91565746	91566015	1.54	3.00E-01	VPS33B	-48		
chr17	79908480	79908891	1.53	3.01E-01	MYADML2	-3577		
chr3	14473425	14473669	1.30	3.01E-01	SLC6A6	29441	GRIP2	110041
chr15	68923925	68924245	1.12	3.01E-01	CORO2B	52512	ANP32A	189176
chr5	131746491	131746864	1.28	3.01E-01	SLC22A5	41277	IRF1	79787
chr8	28259254	28259623	1.18	3.01E-01	FZD3	-92334	ZNF395	-15462
chr6	26271365	26271728	0.80	3.01E-01	HIST1H2BI	-1657	HIST1H3G	65
chr10	11726748	11727088	0.79	3.01E-01	USP6NL	-152644	ECHDC3	-57438
chr4	141444947	141445392	1.25	3.01E-01	ELMOD2	-182		
chr3	128952397	128952740	1.29	3.01E-01	CNBP	-49759	COPG	-15884
chr19	13029993	13030363	1.23	3.01E-01	SYCE2	-92		
chr20	2854012	2854252	1.12	3.02E-01	PTPRA	-49721	VPS16	32759
chr13	34116578	34117079	1.41	3.02E-01	RFC3	-275377	STARD13	-256928
chr1	160162928	160163226	1.26	3.02E-01	PEA15	-12048	CASQ1	2792
chr16	17395493	17395913	1.19	3.02E-01	XYLT1	169035	AK310228	930706
chr20	60757864	60758209	1.40	3.02E-01	GTPBP5	-44		
chr12	108954860	108955178	0.79	3.02E-01	ISCU	-1275	SART3	146
chr16	88103073	88103404	1.27	3.02E-01	ZNF469	-390640	BANP	118201
chr17	41438383	41438824	1.54	3.02E-01	ARL4D	-37749	TMEM106A	74710

chr9	134000836	134001090	1.22	3.02E-01	NUP214	-18		
chr2	38760334	38760653	1.24	3.02E-01	ATL2	-156062	HNRPLL	69684
chr16	71842869	71843390	1.54	3.03E-01	AP1G1	-154		
chr19	49122393	49122920	1.31	3.03E-01	RPL18	-224	SPHK2	109
chr1	156182635	156182991	0.88	3.03E-01	PMF1	29		
chr16	87984449	87985119	1.36	3.03E-01	BANP	-254		
chr4	39460307	39460741	1.27	3.03E-01	RPL9	-317	LIAS	-141
chr19	19779094	19779504	0.70	3.04E-01	ATP13A1	-4796	ZNF101	-364
chr10	43951069	43951456	1.27	3.04E-01	ZNF487	18689	ZNF239	118803
chr5	176943893	176944176	1.29	3.04E-01	DDX41	-68		
chr1	212208770	212209207	1.28	3.04E-01	INTS7	-105	DTL	70
chr2	85843087	85843354	1.37	3.04E-01	USP39	-62		
chr1	219347011	219347610	1.37	3.04E-01	LYPLAL1	119		
chr6	149753150	149753460	0.79	3.05E-01	TFL	52725	MAP3K7IP2	114242
chr17	38083744	38084061	1.31	3.05E-01	ORMDL3	-49		
chr3	121977734	121978015	1.45	3.05E-01	CSTA	-66136	CASR	75345
chr7	102045044	102045354	0.85	3.05E-01	ORAI2	-28797	PRKRIP1	8395
chr19	10764381	10764852	1.51	3.05E-01	ILF3	-320		
chr6	139613101	139613528	0.78	3.06E-01	TXLNB	-107		
chr9	139685601	139685883	1.25	3.06E-01	TMEM141	-35		
chr1	169454901	169455343	0.76	3.06E-01	SLC19A2	86		
chr17	57970120	57970556	1.37	3.06E-01	RPS6KB1	-105	TUBD1	-42
chrX	52949852	52950260	1.00	3.06E-01	FAM156B	21971	FAM156B	35897
chr12	102224453	102224799	1.36	3.07E-01	GNPTAB	6		
chr4	108951559	108951893	1.37	3.07E-01	HADH	40786	LEF1	137852
chr20	44420291	44420660	1.38	3.07E-01	DNTTIP1	-100	WFDC3	71
chr21	46369742	46370018	1.31	3.08E-01	ADARB1	-124613	C21orf67	-10052
chr3	187459876	187460179	1.24	3.08E-01	RTP2	-39683	BCL6	3447
chr12	104323150	104323661	1.20	3.08E-01	HSP90B1	-783	GNN	583
chr15	59978582	59978864	1.40	3.08E-01	GTF2A2	-28986	BNIP2	2919
chr19	42924161	42924471	0.91	3.08E-01	CNFN	-29872	LIPE	7262
chr22	17700175	17700487	1.17	3.08E-01	CECR2	-256299	CECR1	-9552
chr19	19249230	19249496	1.36	3.08E-01	TMEM161A	-96		
chr1	45251818	45252088	0.63	3.08E-01	BEST4	1473	RPS8	10707
chr20	49416714	49417127	0.72	3.08E-01	BCAS4	5454	ADNP	130606
chr2	120517211	120517628	0.71	3.09E-01	PTPN4	213		
chr17	73780604	73780861	1.15	3.09E-01	H3F3B	-4873	UNK	52
chr3	186285087	186285430	1.31	3.09E-01	DNAJB11	-2693	TBCCD1	-118

chr14	68283325	68283626	1.32	3.09E-01	RAD51L1	-3033	ZFYVE26	-170
chr7	25109644	25109911	0.75	3.09E-01	OSBPL3	-90018	CYCS	55177
chr12	9800758	9801292	1.29	3.09E-01	KLRB1	-40528	CLEC2D	-21284
chr1	1838913	1839219	1.21	3.10E-01	GNB1	-16571	CALML6	-7200
chr9	126102467	126102792	0.76	3.10E-01	STRBP	-71787	CRB2	-15818
chr9	139780635	139780920	1.22	3.10E-01	TRAF2	-187		
chr6	29933768	29934076	0.71	3.10E-01	HLA-F	-40295	HLA-A	23591
chr11	118763180	118763456	1.49	3.11E-01	CXCR5	8777	BCL9L	18295
chr19	18518872	18519116	1.23	3.11E-01	SSBP4	-11227	LRRC25	-10579
chr21	33984777	33985169	0.74	3.11E-01	C21orf59	-307		
chr22	39715424	39715848	1.29	3.11E-01	RPL3	34		
chr17	77770866	77771111	1.13	3.11E-01	CBX8	-99		
chr8	101734629	101735069	0.69	3.11E-01	PABPC1	-534		
chr3	10266537	10266853	1.21	3.11E-01	TATDN2	-23482	IRAK2	60132
chr14	75937150	75937548	0.68	3.11E-01	BATF	-51435	JDP2	38512
chr21	45627301	45627611	0.78	3.11E-01	ICOSLG	33378	C21orf33	73962
chr13	80622989	80623250	1.67	3.12E-01	SPRY2	291966	NDFIP2	567861
chr19	6772434	6772840	1.38	3.12E-01	VAV1	-85		
chr19	40931562	40931918	1.22	3.12E-01	SERTAD1	192		
chr1	52000739	52000983	0.62	3.12E-01	OSBPL9	-81685	EPS15	-15866
chr19	2236249	2236507	1.22	3.12E-01	SF3A2	-438	PLEKHJ1	573
chr1	71546690	71547074	0.78	3.12E-01	ZRANB2	-137		
chr9	110045311	110045900	1.21	3.12E-01	RAD23B	62		
chr6	45649561	45649930	0.88	3.12E-01	SUPT3H	-304076	CLIC5	398339
chr16	27214639	27215326	1.33	3.13E-01	NSMCE1	65130		
chr8	142099590	142099840	0.73	3.13E-01	PTK2	-88383	SLC45A4	138958
chr6	52926559	52926803	0.82	3.13E-01	FBXO9	-3115	ICK	-81
chr7	50535752	50536056	0.82	3.13E-01	FIGNL1	-17816	DDC	92864
chr2	64834161	64834535	1.30	3.13E-01	SERTAD2	46698	AFTPH	82883
chr7	26416644	26416920	0.67	3.13E-01	SNX10	85267	SKAP2	487559
chr6	88411547	88412052	0.74	3.13E-01	AKIRIN2	185		
chr7	35839974	35840298	0.71	3.13E-01	SEPT7	-491		
chr12	116997284	116997528	1.33	3.13E-01	MAP1LC3B2	220		
chr11	59317921	59318231	1.39	3.13E-01	OR4D9	35690	OSBP	65541
chr13	34302926	34303253	1.53	3.13E-01	STARD13	-443189	RFC3	-89116
chr17	79791063	79791334	1.21	3.14E-01	P4HB	27345	GCGR	29173
chr15	74913404	74913699	1.27	3.14E-01	CLK3	6217	EDC3	74834
chrX	10087584	10087905	1.32	3.14E-01	CLCN4	-37240	SHROOM2	333249

chr19	42748861	42749187	1.32	3.14E-01	GSK3A	-2288		
chr9	114799771	114800324	0.89	3.14E-01	SUSD1	137508	UGCG	140842
chr11	33499809	33500165	0.77	3.14E-01	C11orf41	-63890	HIPK3	220819
chr2	166049760	166050105	1.24	3.14E-01	SLC38A11	-237898	SCN3A	10644
chr5	52095697	52095992	1.22	3.14E-01	ITGA2	-189311	ITGA1	12071
chr11	59034775	59035046	0.85	3.15E-01	OR5AN1	-97021	MPEG1	-54417
chr5	75698658	75699031	1.07	3.15E-01	IQGAP2	-304		
chr8	52920994	52921304	0.81	3.15E-01	PCMTD1	-109414	ST18	401290
chr6	31633441	31633885	0.81	3.16E-01	BAT4	-500	CSNK2B	6
chr12	12480750	12481026	1.64	3.16E-01	LRP6	-61077	MANSC1	22281
chr2	85044422	85044732	1.70	3.16E-01	C2orf89	63675	DNAH6	300998
chr12	122124850	122125362	1.44	3.16E-01	TMEM120B	-25552	ORAI1	60651
chr3	71470616	71470998	0.90	3.17E-01	FOXP1	162333		
chr18	56531940	56532260	1.32	3.17E-01	SEC11C	-275025	ZNF532	2039
chr6	10838686	10839102	1.23	3.17E-01	MAK	-7784	GCM2	43204
chr4	26863261	26863581	0.72	3.17E-01	STIM2	1057		
chr4	153457099	153457490	1.22	3.17E-01	FBXW7	-1110		
chr1	198567878	198568284	0.81	3.18E-01	ATP6V1G3	-58006	PTPRC	-40056
chr14	24657939	24658340	1.25	3.18E-01	IPO4	874		
chr7	105794351	105794627	0.80	3.18E-01	SYPL1	-41432	NAMPT	131149
chr10	6969559	6969867	0.79	3.18E-01	PRKCQ	-347475	SFMBT2	483737
chr5	55708915	55709248	1.33	3.19E-01	IL6ST	-418319	MAP3K1	-401818
chr15	90437131	90437454	1.31	3.19E-01	ANPEP	-79221	C15orf38	18929
chr17	3571927	3572171	1.14	3.19E-01	TAX1BP3	-76	TMEM93	-41
chr15	101142282	101142791	1.28	3.19E-01	ALDH1A3	-277472	LASS3	-57612
chr17	7738669	7739121	0.78	3.19E-01	KDM6B	-4340		
chr6	31632601	31632985	0.77	3.19E-01	CSNK2B	-864	BAT4	370
chr12	56435244	56435782	1.29	3.19E-01	RPS26	-173		
chr20	18477614	18478038	1.27	3.19E-01	RBBP9	61		
chr13	49343285	49343594	1.48	3.19E-01	FNDC3A	-206608	CYSLTR2	62487
chr14	20922950	20923416	0.77	3.19E-01	APEX1	-107	OSGEP	15
chr2	207630162	207630438	1.52	3.19E-01	MDH1B	-250	FASTKD2	188
chr11	103944680	103945114	0.90	3.19E-01	DDI1	37589	PDGFD	90130
chr5	96232584	96232857	1.74	3.19E-01	LNPEP	-61435	ERAP2	21077
chr20	47835734	47836015	1.21	3.20E-01	DDX27	43		
chr3	141632048	141632366	0.81	3.20E-01	ATP1B3	36737	TFDP2	92179
chr3	186287932	186288420	1.37	3.20E-01	TBCCD1	-3035	DNAJB11	224
chr20	30539751	30540137	1.35	3.20E-01	PDRG1	-61		

chr21	43527975	43528321	0.86	3.20E-01	ABCG1	-111119	UMODL1	36722		
chr8	42660420	42660709	0.82	3.20E-01	CHRNA6	-36946	THAP1	37909		
chr19	56165022	56165601	1.30	3.20E-01	U2AF2	-104				
chr22	36925008	36925293	1.32	3.20E-01	EIF3D	126				
chr1	235491615	235492383	1.42	3.20E-01	ARID4B	-467	GGPS1	246		
chr9	91933263	91933508	1.33	3.20E-01	SECISBP2	-26				
chr15	45492429	45492857	1.39	3.21E-01	SHF	730				
chr11	66610765	66611016	1.28	3.21E-01	RCE1	8				
chrX	47420216	47420533	0.81	3.21E-01	ARAF	-203				
chr19	41955260	41955606	1.27	3.21E-01	CEACAM21	-127098	ATP5SL	-9623		
chr1	53480436	53480757	0.76	3.21E-01	PODN	-47288	SCP2	87649		
chr6	33553480	33553800	0.75	3.22E-01	ITPR3	-35521	BAK1	-5570		
chr6	43682716	43683086	1.57	3.22E-01	VEGFA	-55052	MRPS18A	-27373		
chr12	12867124	12867402	1.25	3.22E-01	CDKN1B	-3039				
chr8	26435299	26435609	0.88	3.23E-01	DPYSL2	33				
chr19	16606802	16607178	0.84	3.23E-01	CALR3	13				
chr22	24830575	24830886	1.32	3.23E-01	UPB1	-59346	ADORA2A	2912		
chr19	10349877	10350149	0.81	3.24E-01	MRPL4	-12627	S1PR2	-8065		
chr1	234908153	234908397	1.22	3.24E-01	IRF2BP2	-163004	TOMM20	383981		
chr6	74230610	74231110	1.41	3.24E-01	EEF1A1	-105				
chr15	65588047	65588663	1.47	3.24E-01	PARP16	-9337	IGDCC3	82023		
chr3	133167398	133167760	1.24	3.24E-01	CDV3	-124855	BFSP2	48789		
chr14	81407949	81408355	0.96	3.24E-01	DIO2	-729627	TSHR	-13717		
chr13	45915252	45915622	1.27	3.25E-01	TPT1	-140				
chr11	72145180	72145451	0.70	3.25E-01	CLPB	252				
chr15	56757197	56757723	0.85	3.25E-01	MNS1	-125				
chr20	35470399	35470709	1.46	3.25E-01	DSN1	-68400	SAMHD1	109622		
chr6	100016477	100016793	0.79	3.25E-01	CCNC	55				
chr7	92157848	92158228	0.74	3.25E-01	PEX1	-193				
chr6	37400728	37401124	0.74	3.25E-01	FTSJD2	19				
chr12	69979172	69979487	0.77	3.26E-01	CCT2	122				
chr5	156998277	156998587	0.85	3.26E-01	ADAM19	4336	NIPAL4	111405		
chr11	65626989	65627233	1.29	3.26E-01	CFL1	-1010	CFL1	-1010	MUS81	-761
chr11	125495611	125495971	1.34	3.26E-01	CHEK1	-521				
chr17	40976074	40976462	1.39	3.26E-01	BECN1	42				
chr11	65819545	65819855	1.19	3.26E-01	GAL3ST3	-3049	SF3B2	-116		
chr1	180471888	180472198	0.90	3.27E-01	ACBD6	-21				
chr20	48227688	48228024	1.27	3.27E-01	PTGIS	-43149	B4GALT5	102565		

chr6	148606	148970	1.50	3.27E-01	DUSP22	-143313		
chr5	123985180	123985739	1.51	3.27E-01	ZNF608	95405		
chr8	60969399	60969740	0.94	3.28E-01	TOX	-937803	CA8	224384
chr1	36653796	36654216	0.75	3.28E-01	THRAP3	-36011	MAP7D1	32203
chr1	161171936	161172394	1.30	3.28E-01	ADAMTS4	-3320	NDUFS2	3060
chr11	65848987	65849297	0.77	3.28E-01	KLC2	-176032	PACS1	11318
chr1	28655354	28655677	1.36	3.28E-01	MED18	3		
chr6	36853558	36853965	0.85	3.28E-01	C6orf89	122		
chr11	111749720	111750265	1.38	3.28E-01	C11orf1	45	FDXACB1	160
chr9	101984202	101984564	0.86	3.28E-01	SEC61B	-187	ALG2	-137
chr17	8054467	8054722	1.24	3.29E-01	HES7	-27193	PER1	1158
chr19	49468297	49468924	1.26	3.29E-01	FTL	45		
chrX	15353717	15354028	1.31	3.29E-01	PIGA	-213		
chr2	190627269	190627579	0.84	3.29E-01	OSGEPL1	500		
chr6	33029803	33030084	0.81	3.29E-01	HLA-DOA	-52555	HLA-DPA1	11605
chrX	21857592	21857838	1.34	3.30E-01	MBTPS2	59		
chr3	188471082	188471527	1.51	3.30E-01	TPRG1	-418458	LPP	540584
chr8	128747430	128747979	0.82	3.30E-01	MYC	-610		
chr21	40684313	40684590	1.04	3.30E-01	PSMG1	-129012	BRWD1	1104
chr16	14448529	14449128	1.43	3.30E-01	PARN	275295	MKL2	283633
chr7	99966855	99967100	0.81	3.31E-01	PILRA	-4090		
chr11	62623201	62623719	1.38	3.31E-01	SLC3A2	-58		
chr20	32580616	32581065	0.82	3.31E-01	RALY	-891		
chr18	43753753	43754116	1.35	3.32E-01	RNF165	-160252	HAUS1	69637
chr13	24789402	24789681	1.45	3.32E-01	C1QTNF9	-94174	SPATA13	235703
chr2	73512272	73512661	0.76	3.32E-01	FBXO41	-14424	EGR4	8362
chr14	23398703	23399065	1.35	3.33E-01	PRMT5	-223		
chr19	54640687	54640996	1.11	3.33E-01	CNOT3	-607		
chr22	23270917	23271273	1.42	3.33E-01	GNAZ	-141574	IGL@	41135
chr12	26309143	26309646	1.36	3.33E-01	SSPN	-39111	BHLHE41	-31392
chr4	667929	668292	1.18	3.33E-01	MYL5	-3600	ATP5I	11
chr1	167657202	167657478	1.31	3.33E-01	CREG1	-134284	MPZL1	-33847
chr9	134615441	134615715	1.11	3.33E-01	RAPGEF1	-30349	MED27	339675
chr16	75467282	75467530	1.28	3.33E-01	CFDP1	-19		
chr14	21151396	21151790	1.39	3.33E-01	OR6S1	-41743	RNASE4	-5339
chr19	10216696	10217087	0.74	3.33E-01	ANGPTL6	-3467	PPAN-P2RY11	-73
chrX	47052955	47053230	1.33	3.34E-01	PCTK1	-25022	UBA1	2894
chr1	44115520	44115858	0.72	3.34E-01	KDM4A	-108		

chr19	17357130	17357452	1.11	3.34E-01	NR2F6	-1140		
chr2	158300347	158300724	1.37	3.34E-01	CYTIP	68		
chr6	35227157	35227572	0.84	3.34E-01	ZNF76	-145		
chr2	46727343	46727590	1.29	3.34E-01	ATP6V1E2	19629	LOC388946	20763
chr2	96814196	96814636	1.21	3.34E-01	DUSP2	-3237		
chr19	2156037	2156281	0.64	3.34E-01	AP3D1	-4603		
chr20	35402071	35402326	1.26	3.34E-01	DSN1	-45		
chr2	65833295	65833694	1.42	3.34E-01	MEIS1	-829037	SPRED2	-173839
chr15	41061833	41062234	0.72	3.34E-01	C15orf62	-125		
chr11	118230087	118230509	1.47	3.34E-01	UBE4A	-4		
chr6	90081755	90082065	0.71	3.34E-01	UBE2J1	-19291	RRAGD	40085
chr1	150601602	150601883	1.26	3.34E-01	ENSA	355		
chr3	188207354	188207688	0.82	3.34E-01	TPRG1	-682242	LPP	276800
chr1	22109906	22110303	1.26	3.34E-01	USP48	-417		
chr16	86588729	86588973	1.40	3.35E-01	MTHFSD	-10		
chr7	101499799	101500071	0.87	3.35E-01	SH2B2	-428470	CUX1	39053
chr14	75229815	75230126	1.30	3.35E-01	YLPM1	-98		
chr1	68150409	68151046	0.64	3.36E-01	GADD45A	-155		
chr14	60797883	60798472	0.85	3.36E-01	PPM1A	85708	C14orf39	154586
chr1	179334789	179335280	0.88	3.36E-01	SOAT1	72018	NPHS2	210049
chr11	67414082	67414448	0.86	3.36E-01	TBX10	-7234	ACY3	3865
chr6	34023081	34023348	0.82	3.36E-01	MLN	-251422	GRM4	78421
chr12	46603752	46604020	1.23	3.36E-01	SFRS2IP	-219485	SLC38A1	59322
chrX	153763052	153763372	1.05	3.37E-01	FAM3A	-18839	IKBKG	-7247
chr1	167586753	167587072	1.29	3.37E-01	MPZL1	-104274	CREG1	-63857
chr14	93170731	93170975	1.50	3.37E-01	LGMN	44159	RIN3	190728
chr19	3505897	3506208	1.19	3.37E-01	FZR1	-16901	DOHH	-5432
chr21	38738820	38739129	1.32	3.37E-01	DSCR3	-99142	DYRK1A	-53626
chr7	65215837	65216095	0.69	3.37E-01	VKORC1L1	-122291	ZNF92	377198
chr1	145610733	145611047	1.46	3.37E-01	RNF115	-146	POLR3C	-6
chr22	24951779	24952024	1.33	3.38E-01	SNRPD3	284		
chr1	9777631	9778153	1.19	3.38E-01	PIK3CD	66102	CLSTN1	106658
chr9	140121983	140122294	1.34	3.38E-01	SLC34A3	-3246		
chr15	101603037	101603292	1.41	3.38E-01	LRRK1	143705	CHSY1	188961
chr6	15299811	15300121	1.31	3.38E-01	JARID2	53439	DTNBP1	363305
chr4	1717204	1717460	1.31	3.38E-01	SLBP	-3302		
chrX	1511031	1511404	1.32	3.38E-01	SLC25A6	-220		
chrX	48433155	48433399	1.37	3.38E-01	RBM3	441		

chr10	14650452	14650728	0.67	3.38E-01	FRMD4A	-277724	CDNF	229393		
chr12	112125146	112125397	0.69	3.38E-01	BRAP	-1482	ACAD10	1415		
chr19	55574460	55574748	1.45	3.38E-01	RDH13	-19				
chr22	22777111	22777352	1.21	3.39E-01	ZNF280B	86273	VPREB1	178032		
chr8	22461936	22462268	1.32	3.39E-01	KIAA1967	-155				
chr1	193028415	193028761	1.39	3.39E-01	TROVE2	-369	UCHL5	-67	UCHL5	601
chr13	41706905	41707175	1.31	3.39E-01	KBTBD6	-104				
chr17	33288399	33288739	1.26	3.39E-01	CCT6B	-63	ZNF830	20		
chr7	100926323	100926570	0.73	3.39E-01	FIS1	-38076	RABL5	38646		
chr1	2231876	2232267	1.10	3.39E-01	RER1	-91142	SKI	71938		
chr10	60028623	60028947	1.13	3.39E-01	IPMK	-1091	CISD1	-110		
chr21	45225375	45225685	1.26	3.40E-01	AGPAT3	-59586	RRP1	16112		
chr22	21356375	21356685	1.46	3.40E-01	THAP7	-126				
chr19	35626321	35626674	1.34	3.40E-01	FXYD1	-3234	LGI4	-320		
chr6	27114744	27115134	1.44	3.41E-01	HIST1H2BK	-320	HIST1H2AH	31		
chr6	88032120	88032491	0.82	3.41E-01	C6orf162	0				
chr5	149867314	149867655	0.71	3.41E-01	RPS14	-38166	NDST1	-20189		
chr16	2390773	2391070	1.15	3.41E-01	ABCA3	-175				
chr5	43042684	43042984	0.71	3.41E-01	C5orf39	-2387				
chrX	14047973	14048255	1.29	3.41E-01	GEMIN8	-79				
chr13	20437565	20437851	1.10	3.41E-01	ZMYM5	68				
chr20	35233797	35234133	1.28	3.41E-01	TGIF2	31955	SLA2	40593		
chr5	118611435	118611887	0.81	3.41E-01	TNFAIP8	-79935				
chr2	100758830	100759287	1.19	3.41E-01	AFF3	-37014	LONRF2	180136		
chr1	100816867	100817177	0.77	3.42E-01	CDC14A	-1001				
chr3	10028407	10028677	1.29	3.42E-01	TMEM111	-20				
chr3	196295162	196295707	1.23	3.42E-01	FBXO45	-290				
chr22	42332247	42332706	1.36	3.42E-01	TNFRSF13C	-9656	CENPM	10671		
chr11	47574699	47575009	0.75	3.42E-01	CUGBP1	-64278	PTPMT1	-12128		
chr11	67276043	67276307	1.39	3.42E-01	PITPNM1	-3332	CDK2AP2	-73		
chr7	132766840	132767162	1.31	3.42E-01	CHCHD3	-173				
chr2	98262242	98262689	1.22	3.42E-01	COX5B	-55				
chr7	64254610	64255014	0.78	3.42E-01	ZNF138	41				
chr6	136571442	136571846	0.78	3.42E-01	BCLAF1	39345	PDE7B	398810		
chr19	1266197	1266590	0.71	3.42E-01	CIRBP	-2873				
chr19	907328	907688	1.27	3.42E-01	MED16	-14290	C19orf22	5717		
chr9	123555590	123555871	1.31	3.43E-01	FBXW2	9				
chr9	99179599	99179839	1.22	3.43E-01	ZNF367	950				

chr15	55489124	55489479	1.28	3.43E-01	RSL24D1	-71		
chr9	139972435	139972683	1.18	3.43E-01	UAP1L1	606		
chr2	71295135	71295901	1.30	3.44E-01	NAGK	110		
chr2	201936164	201936512	0.86	3.44E-01	NDUFB3	-124	FAM126B	54
chr4	26321191	26321478	1.28	3.45E-01	RBPJ	-1113		
chr6	14109241	14109540	1.28	3.45E-01	CD83	-8474	RNF182	184188
chr11	65255090	65255614	0.82	3.45E-01	SCYL1	-37196	FRMD8	101311
chr1	155904081	155904391	1.47	3.45E-01	RIT1	-23059	RXFP4	-7244
chr17	47817105	47817490	0.70	3.45E-01	MYST2	-48773	SLC35B1	-32016
chr19	47747476	47747839	1.36	3.46E-01	PRR24	-30484	BBC3	-11635
chr19	14491919	14492229	1.31	3.46E-01	CD97	-139		
chr17	29814652	29814899	0.64	3.46E-01	RAB11FIP4	96134	C17orf79	371550
chr12	75905280	75905615	1.27	3.46E-01	KRR1	-30		
chr2	102333548	102333835	0.91	3.46E-01	IL1R2	-274614	MAP4K4	19204
chr18	47839099	47839436	1.45	3.47E-01	SKA1	-62124	CXXC1	-24576
chrX	23801094	23801378	1.32	3.47E-01	SAT1	-39		
chr2	33701577	33701823	1.26	3.47E-01	RASGRP3	-37242	LTBP1	529308
chr3	178865719	178865987	1.34	3.47E-01	PIK3CA	-458		
chr5	179402190	179402544	1.28	3.47E-01	TBC1D9B	-67511	RNF130	96742
chr6	25018245	25018521	0.69	3.47E-01	FAM65B	-107188	DKFZp686H12134	119668
chr7	44835677	44836385	0.76	3.47E-01	PPIA	-210		
chr2	11890554	11890854	1.20	3.47E-01	TRIB2	-966294	LPIN1	3964
chr19	16696683	16696996	1.22	3.48E-01	SLC35E1	-13647	MED26	42175
chr11	3818918	3819267	1.18	3.48E-01	NUP98	-201		
chr1	42107189	42107533	0.68	3.48E-01	EDN2	-157017	HIVEP3	277135
chr19	7968374	7968884	1.36	3.48E-01	MAP2K7	-136		
chr9	140095126	140095548	1.25	3.48E-01	NDOR1	-4782	C9orf75	-357
chr1	165362190	165362526	1.35	3.48E-01	LMX1A	-37341	RXRG	52072
chr19	39322199	39322627	1.36	3.49E-01	LGALS4	-18673	HNRNPL	18204
chr7	69029882	69030244	0.70	3.49E-01				
chr2	677256	677610	1.27	3.49E-01	TMEM18	6		
chr5	133268371	133268852	0.71	3.50E-01	FSTL4	-320389	C5orf15	35794
chr16	2570210	2570510	1.49	3.50E-01	AMDHD2	-3		
chr12	31226813	31227229	0.75	3.50E-01	DDX11	242		
chr17	56595446	56595875	0.79	3.50E-01	MTMR4	-410		
chr7	76822336	76822581	1.36	3.50E-01	FGL2	6691	UPK3B	682714
chrX	134654414	134654658	1.22	3.51E-01	ZNF449	175840	MMGT1	401598
chr6	11382352	11382729	0.82	3.51E-01	TMEM170B	-155970	NEDD9	-149626

chr2	197104946	197105241	1.33	3.51E-01	STK17B	-68758	HECW2	352241
chr19	6199418	6199910	1.26	3.51E-01	ACSBG2	63954	MLLT1	80295
chr3	141193073	141193388	1.53	3.51E-01	RASA2	-12695	ZBTB38	150176
chr19	55765202	55765787	1.31	3.51E-01	TMEM86B	-24863	SAPS1	4543
chr19	10346739	10347145	0.83	3.51E-01	S1PR2	-4994		
chr11	64899732	64900260	1.36	3.51E-01	SYVN1	2007	MRPL49	10268
chrX	48456405	48456686	1.25	3.51E-01	WDR13	645		
chr20	5509572	5509841	1.29	3.51E-01	PROKR2	-214684	RP5-1022P6.2	81965
chr15	85923472	85923832	1.33	3.51E-01	AKAP13	-219		
chr14	23057912	23058424	1.24	3.52E-01	DAD1	-25		
chr2	219536549	219536950	0.81	3.52E-01	STK36	-12	RNF25	31
chr11	65420142	65420626	1.36	3.52E-01	RELA	10059	SIPA1	14806
chr7	75495772	75496078	0.81	3.52E-01	CCL24	-52833	RHBDD2	-12392
chr12	111099317	111099627	0.84	3.52E-01	HVCN1	27474	TCTN1	47592
chr4	86646892	86647232	0.72	3.52E-01	ARHGAP24	250778	MAPK10	628735
chr11	67396739	67397109	1.22	3.53E-01	NUDT8	477		
chr5	111093810	111094060	1.23	3.53E-01	STARD4	-245778	C5orf13	218693
chr5	544079	544510	0.76	3.53E-01	CEP72	-68110	SLC9A3	-19746
chr1	151042738	151043296	0.76	3.53E-01	GABPB2	-63		
chr19	50844462	50844772	1.48	3.53E-01	KCNC3	-11983	NAPSB	3388
chr1	150898612	150898886	1.26	3.53E-01	SETDB1	-66		
chr10	70939577	70940108	0.78	3.53E-01	SUPV3L1	-150		
chr19	4457736	4458057	1.32	3.53E-01	UBXN6	-107		
chr3	15247397	15247806	1.18	3.53E-01	CAPN7	-131		
chr19	6292108	6292418	0.85	3.53E-01	MLLT1	-12304	ACER1	41377
chrX	70288248	70288683	1.35	3.53E-01	SNX12	-235		
chr14	93260442	93260862	0.83	3.54E-01	GOLGA5	2		
chr3	195823285	195823767	1.18	3.54E-01	TFRC	-14494	ZDHHC19	114774
chr2	42981140	42981557	0.98	3.54E-01	OXER1	10052	MTA3	185678
chr18	48343217	48343533	1.30	3.54E-01	MRO	3059	MAPK4	256891
chr1	153606340	153606584	1.31	3.54E-01	C1orf77	-63		
chr12	57824687	57825032	1.27	3.54E-01	INHBC	-3683		
chr12	48173164	48173408	0.72	3.54E-01	SLC48A1	6319	HDAC7	40477
chr17	7210319	7210702	1.35	3.55E-01	EIF5A	193		
chr19	2622519	2622982	0.84	3.55E-01	GNG7	79995	GADD45B	146616
chr6	11537778	11538039	0.77	3.55E-01	TMEM170B	-602		
chr15	63484194	63484490	0.86	3.55E-01	APH1B	-85407	RAB8B	2614
chr15	52263733	52264249	1.29	3.56E-01	LEO1	-33		

chr16	89555560	89555825	1.36	3.56E-01	ANKRD11	1276	ZNF778	271582
chr19	38419150	38419472	1.41	3.56E-01	SIPA1L3	21443	DPF1	295579
chr11	77850603	77850896	1.23	3.56E-01	ALG8	-51		
chr7	22491814	22492124	0.80	3.56E-01	RAPGEF5	-95436	MGC87042	47829
chr12	133613759	133614238	1.16	3.56E-01	ZNF84	19		
chr11	67056388	67056754	1.17	3.57E-01	SSH3	-14348	ADRBK1	22666
chr1	161369378	161369663	1.28	3.57E-01	FCGR2A	-105684	SDHC	85355
chr11	78285789	78286033	1.15	3.57E-01	NARS2	-2		
chr5	218141	218480	0.99	3.57E-01	SDHA	-45		
chr13	50019227	50019512	1.44	3.57E-01	CAB39L	-43635	SETDB2	-6319
chr18	30383531	30383952	0.81	3.57E-01	ASXL3	-774799	KLHL14	-30768
chr4	84376927	84377329	1.30	3.57E-01	HELQ	-103	MRPS18C	10
chr9	100700480	100700790	0.75	3.57E-01	HEMGN	-109		
chr9	100772848	100773124	1.22	3.57E-01	NANS	-45696	ANP32B	27497
chr9	112680650	112681011	1.48	3.57E-01	PALM2-AKAP2	138254	TXN	337947
chr6	42896812	42897386	0.77	3.57E-01	CNPY3	239		
chr12	6602741	6603203	0.74	3.57E-01	MRPL51	-501	NCAPD2	-326
chr6	47210181	47210515	1.27	3.57E-01	GPR110	-200266	TNFRSF21	67332
chr12	29534052	29534410	1.26	3.57E-01	ERGIC2	-88		
chr1	23881246	23881498	1.22	3.58E-01	E2F2	-23659	ID3	4950
chr20	3801294	3801604	1.22	3.58E-01	MAVS	-26000	CDC25B	25048
chr17	26684403	26684748	1.28	3.58E-01	TMEM199	-111	POLDIP2	27
chr7	66119214	66119704	0.77	3.58E-01	RABGEF1	-85524	KCTD7	25569
chr1	150241314	150241873	1.32	3.58E-01	C1orf54	-3608	APH1A	-62
chr16	67596065	67596371	1.15	3.58E-01	CTCF	-246		
chr15	70307280	70307590	1.20	3.58E-01	TLE3	82821	RPLP1	562276
chr2	232479441	232479752	0.74	3.58E-01	PTMA	-93638	NMUR1	-84415
chr3	138198221	138198497	1.63	3.58E-01	ESYT3	44944	CEP70	114770
chr7	128337401	128337665	1.39	3.58E-01	CALU	-41813	METTL2B	220750
chr19	2042156	2042492	0.78	3.58E-01	BTBD2	-26622	MKNK2	8919
chr15	64386028	64386305	1.36	3.59E-01	SNX1	-2010		
chr19	38210355	38210999	0.86	3.59E-01	ZNF607	14		
chr19	5288138	5288426	0.64	3.59E-01	PTPRS	52532	KDM4B	319158
chr9	97443800	97444076	1.33	3.59E-01	C9orf3	-45056	FBP1	-42015
chr12	50898541	50898851	0.82	3.59E-01	DIP2B	-72		
chr2	25016143	25016437	1.20	3.59E-01	CENPO	-43	C2orf79	-39
chr3	193310818	193311137	0.82	3.60E-01	OPA1	45		
chr13	113951225	113951592	0.78	3.60E-01	LAMP1	-60		

chr19	55792388	55792628	1.27	3.60E-01	BRSK1	-3026	HSPBP1	-757
chr12	89740045	89740490	0.89	3.60E-01	KITLG	-766030	DUSP6	6028
chr9	37371010	37371615	0.77	3.61E-01	GRHPR	-51394	ZCCHC7	250844
chr17	73663038	73663496	1.19	3.61E-01	SAP30BP	-132	RECQL5	2
chr9	102861230	102861517	1.17	3.61E-01	INVS	-137	ERP44	-44
chr14	24900967	24901277	0.72	3.62E-01	CBLN3	-2391	KIAA0323	1981
chr6	149886759	149887019	0.62	3.62E-01	C6orf72	-639		
chr1	42216965	42217319	1.34	3.62E-01	EDN2	-266798	HIVEP3	167354
chr6	11461089	11461443	1.50	3.62E-01	NEDD9	-228351	TMEM170B	-77245
chr19	42772861	42773101	1.10	3.62E-01	CIC	-15836	ERF	-13672
chr9	86595474	86595881	1.30	3.62E-01	HNRNPK	-109	RMI1	41
chr1	39491672	39492092	0.68	3.62E-01	NDUFS5	-124		
chr7	21466703	21467070	0.73	3.62E-01	SP4	-802		
chr10	73454350	73454693	1.15	3.62E-01	RP11-472K8.2-001	25056	CDH23	297818
chr12	95968696	95968940	0.84	3.62E-01	USP44	-26198	NTN4	215718
chr5	150138336	150138764	1.37	3.62E-01	DCTN4	107		
chr17	37025432	37025830	0.81	3.62E-01	LASP1	-481	LASP1	-481
chr19	6590964	6591289	1.32	3.62E-01	CD70	36		
chr16	69788633	69789023	1.26	3.62E-01	NOB1	1		
chr8	128746245	128746510	0.78	3.62E-01	MYC	-1937		
chr11	44625725	44626037	0.73	3.62E-01	TSPAN18	-302077	CD82	38740
chr19	49298575	49299002	1.35	3.62E-01	BCAT2	15512	FGF21	39641
chr8	22431912	22432250	0.86	3.62E-01	PDLIM2	-5903	SORBS3	22830
chr16	11945249	11945591	1.24	3.62E-01	RSL1D1	22		
chr2	232571553	232572008	1.20	3.62E-01	PTMA	-1454		
chr17	80376354	80376615	1.22	3.62E-01	C17orf101	-23	HEXDC	233
chr8	143484554	143484863	1.19	3.63E-01	TSNARE1	-166		
chr1	25427911	25428237	0.77	3.63E-01	RUNX3	-171304	SYF2	130939
chr14	105512088	105512520	0.81	3.63E-01	CDCA4	-24879	GPR132	19450
chr3	71466178	71466525	0.81	3.63E-01	FOXP1	166788		
chr12	110841531	110842058	0.81	3.63E-01	ANAPC7	-260		
chr12	120638936	120639196	1.17	3.63E-01	RPLP0	-52		
chr7	150065737	150065988	1.35	3.63E-01	REPIN1	-16		
chr18	20811287	20811690	1.31	3.63E-01	CABLES1	95762	C18orf45	206436
chr11	102962726	102963073	1.26	3.63E-01	MMP13	-136438	DYNC2H1	-17260
chr15	34502098	34502527	1.24	3.63E-01	C15orf29	-16		
chr22	24830093	24830396	1.35	3.63E-01	UPB1	-59832	ADORA2A	2426
chr11	615819	616355	1.30	3.64E-01	IRF7	-88		

chr2	88899630	88899914	1.27	3.65E-01	EIF2AK3	27222	C2orf51	75603
chr4	71859003	71859424	1.05	3.65E-01	DCK	-51		
chr1	23921435	23921745	1.23	3.65E-01	RPL11	-96704	ID3	-35268
chr7	44254014	44254290	0.65	3.65E-01	YKT6	13574	CAMK2B	111078
chr12	329063	329324	0.70	3.65E-01	SLC6A12	-5823	SLC6A13	42808
chr19	11593401	11593683	0.91	3.65E-01	ELAVL3	-1739		
chr1	161391704	161392050	1.59	3.65E-01	FCGR2A	-83328	SDHC	107711
chr12	125093660	125093932	0.78	3.65E-01	NCOR2	-73639	SCARB1	254723
chr19	16698458	16698778	1.32	3.65E-01	SLC35E1	-15425	MED26	40397
chr7	39663070	39663347	0.70	3.65E-01	RALA	47		
chr14	77564300	77564631	1.28	3.66E-01	C14orf4	-69432	ZDHHC22	43668
chr7	158648957	158649372	1.20	3.66E-01	ESYT2	-26846	VIPR2	288484
chr2	233925821	233926227	0.80	3.66E-01	INPP5D	988		
chr9	134604425	134604669	1.38	3.67E-01	RAPGEF1	-19318	MED27	350706
chr4	140899797	140900113	1.65	3.67E-01	MAML3	175278	MGST2	313033
chr1	21620772	21621441	0.70	3.67E-01	ECE1	-4341		
chr17	34257439	34258064	0.88	3.67E-01	RDM1	28		
chr7	23510099	23510480	0.73	3.68E-01	IGF2BP3	-295		
chr10	94352546	94352943	1.26	3.68E-01	KIF11	-80		
chr10	102106695	102106950	1.29	3.69E-01	SCD	51		
chr17	78549180	78549451	1.32	3.69E-01	CHMP6	-416325	RPTOR	30691
chr22	50354247	50354546	0.98	3.69E-01	PIM3	254		
chr20	32398782	32399206	1.23	3.69E-01	CHMP4B	-125		
chr16	87417512	87417832	1.38	3.69E-01	FBXO31	-278		
chr2	26467505	26467741	0.93	3.69E-01	HADHA	-29	HADHB	7
chr1	170043754	170044186	1.04	3.69E-01	KIFAP3	-91		
chr17	685438	685730	1.33	3.69E-01	GLOD4	-13	RNMTL1	71
chr17	62097389	62097699	0.96	3.69E-01	ICAM2	450		
chr1	198591650	198592010	1.46	3.70E-01	ATP6V1G3	-81755	PTPRC	-16307
chr12	97300977	97301272	1.36	3.70E-01	NEDD1	124		
chr19	10426526	10426865	1.28	3.70E-01	FDX1L	-5		
chr12	53902386	53902645	0.75	3.70E-01	NPFF	-1094		
chr1	203290056	203290472	1.25	3.71E-01	BTG2	15600	FMOD	30025
chr20	48286195	48286521	1.38	3.71E-01	PTGIS	-101651	B4GALT5	44063
chr16	84548400	84548868	0.90	3.71E-01	KIAA1609	-10346	COTL1	103035
chr1	52831773	52832067	1.22	3.71E-01	ORC1L	38211	ZFYVE9	223874
chr7	139875019	139875456	1.42	3.72E-01	PARP12	-111717	JHDM1D	1503
chr7	23053538	23053947	0.61	3.72E-01	FAM126A	27		

chr2	217363387	217363699	1.20	3.72E-01	RPL37A	23		
chr22	31886447	31886825	1.32	3.72E-01	EIF4ENIF1	-932		
chr6	30655128	30655424	0.91	3.72E-01	KIAA1949	-183		
chr14	102240584	102241029	1.05	3.72E-01	DYNC1H1	-190058	PPP2R5C	12672
chr11	63949046	63949316	1.13	3.72E-01	STIP1	-4406		
chr6	33538888	33539352	0.88	3.72E-01	BAK1	8950	ZBTB9	116764
chr15	75397247	75397599	1.39	3.72E-01	PPCDC	81496	DNM1P33	197883
chr22	43011192	43011581	1.44	3.73E-01	POLDIP3	-425		
chr4	28618	29005	0.81	3.74E-01	ZNF718	-24415		
chr15	41099598	41099942	1.35	3.74E-01	DNAJC17	-115	ZFYVE19	496
chr3	191759955	191760273	1.47	3.74E-01	FGF12	366724	PYDC2	581162
chr5	149815599	149815956	0.77	3.74E-01	CD74	-23446	RPS14	13541
chr20	45825078	45825396	1.24	3.74E-01	ZMYND8	160237	EYA2	301974
chr19	41814408	41814718	1.31	3.74E-01	HNRNPUL1	44443	TGFB1	45253
chr17	28049726	28050008	1.33	3.74E-01	GIT1	-133257	SSH2	207151
chr3	13057876	13058186	1.09	3.75E-01	RPL32	-176082	IQSEC1	56586
chr7	73620965	73621201	0.71	3.75E-01	LAT2	-3004		
chr1	53102745	53103387	1.38	3.75E-01	FAM159A	4000	C1orf163	60972
chr19	8386134	8386446	1.22	3.75E-01	RPS28	-94	NDUFA7	-10
chr15	66446051	66446663	1.13	3.75E-01	MEGF11	99718	RAB11A	284561
chr7	45151161	45151423	0.86	3.75E-01	TBRG4	25		
chr9	35636050	35636454	0.78	3.75E-01	CD72	-17828	SIT1	14695
chr11	59578141	59578451	1.42	3.77E-01	MRPL16	49		
chr2	8681351	8681680	1.27	3.77E-01	ID2	-140468		
chr17	78428338	78428715	1.16	3.77E-01	NPTX1	21877	FLJ35220	39560
chr22	26824782	26825129	1.26	3.77E-01	ASPHD2	-324		
chrX	18692985	18693273	1.28	3.78E-01	RS1	-2906		
chr1	205600238	205600589	1.46	3.78E-01	SLC45A3	49216	MFSD4	62302
chr14	32670279	32670615	1.25	3.78E-01	AKAP6	-128032	ARHGAP5	123952
chr12	96252490	96252855	1.15	3.78E-01	SNRPF	-36		
chr1	77684882	77685247	1.17	3.78E-01	PIGK	67		
chr17	41150134	41150576	1.34	3.78E-01	RPL27	-91		
chr1	203256447	203256950	1.53	3.78E-01	CHIT1	-57839	BTG2	-17965
chr12	124950695	124951096	0.70	3.78E-01	NCOR2	69261	ZNF664	493226
chr7	99063716	99064002	0.76	3.78E-01	ZNF789	-6656	CPSF4	27296
chr17	80250626	80250942	1.31	3.78E-01	CSNK1D	-19211	CD7	24696
chr3	186648892	186649202	0.88	3.78E-01	ST6GAL1	-90618	ADIPOQ	88584
chr16	23347300	23347582	1.29	3.78E-01	SCNN1B	33850	COG7	117062

chr12	48191291	48191567	1.11	3.79E-01	HDAC7	22334	SLC48A1	24462
chr12	92839591	92839928	1.32	3.79E-01	BTG1	-300087	PLEKHG7	-290505
chr5	118677417	118677734	1.31	3.79E-01	TNFAIP8	-14020		
chr19	1665437	1665748	1.23	3.79E-01	ONECUT3	-88069	TCF3	-15307
chr3	191848366	191848676	0.84	3.79E-01	FGF12	278317	PYDC2	669569
chr15	70393925	70394251	1.14	3.80E-01	TLE3	-3832		
chr17	45972982	45973527	1.18	3.80E-01	SP2	-261		
chr2	161263410	161263748	1.47	3.80E-01	ITGB6	-206990	RBMS1	86739
chr14	21081447	21081806	1.27	3.80E-01	RNASE12	-22645	OR6S1	28223
chr14	75741514	75741794	0.80	3.80E-01	FOS	-3827		
chr10	96122469	96122861	0.84	3.80E-01	NOC3L	18		
chr9	109515559	109515941	1.48	3.80E-01	ZNF462	-109628		
chrX	20160008	20160263	1.18	3.80E-01	EIF1AX	-170		
chr2	64441892	64442202	1.08	3.80E-01	HSPC159	-239280	PELI1	-70442
chr22	23284823	23285266	1.31	3.80E-01	GNAZ	-127624	IGL@	55085
chr16	3156438	3156828	1.22	3.81E-01	ZSCAN10	-13772	ZNF205	-5930
chr20	57582748	57583068	1.31	3.81E-01	CTSZ	-599		
chr10	106028481	106029031	0.81	3.81E-01	GSTO2	125		
chr10	14792953	14793315	1.14	3.81E-01	FRMD4A	-420268	CDNF	86849
chr11	122932698	122933137	0.77	3.81E-01	HSPA8	-74		
chr16	10970658	10971132	1.34	3.81E-01	CIITA	-160		
chr19	39826579	39826921	1.43	3.81E-01	GMFG	-24		
chr8	17780103	17780504	1.24	3.81E-01	PCM1	-62		
chr17	34891210	34891533	0.86	3.81E-01	MYO19	-67	PIGW	-31
chr7	90366626	90366933	0.79	3.81E-01	FZD1	-527003	PFTK1	28068
chr9	96031257	96031653	1.26	3.81E-01	FAM120A	-182718	WNK2	84243
chr17	74099792	74100042	1.23	3.81E-01	EXOC7	-49		
chr3	179280479	179281032	1.28	3.82E-01	ACTL6A	48		
chr11	46264801	46265139	1.08	3.82E-01	PHF21A	-121985	CREB3L1	-34258
chr17	29862771	29863074	1.23	3.82E-01	RAB11FIP4	144281	C17orf79	323403
chr12	120570576	120571038	1.32	3.82E-01	RAB35	-16208	GCN1L1	61706
chr12	104359432	104359856	1.32	3.82E-01	TDG	51		
chr7	117823956	117824330	1.17	3.82E-01	LSM8	57		
chr1	1710300	1710566	1.11	3.82E-01	NADK	-524		
chr16	27388993	27389321	0.85	3.82E-01	IL21R	-49422	IL4R	63906
chr2	223836474	223836732	1.35	3.82E-01	KCNE4	-80259	ACSL3	110871
chr17	43225769	43226087	0.73	3.82E-01	HEXIM2	-12336	HEXIM1	1244
chr7	99006014	99006735	0.76	3.83E-01	BUD31	-226	PDAP1	-83

chr12	56601784	56602094	0.72	3.83E-01	SMARCC2	-18588	RNF41	13765
chr7	100861273	100861533	1.19	3.83E-01	PLOD3	-392	ZNHIT1	418
chr2	74426156	74426466	0.75	3.83E-01	MTHFD2	621		
chr11	70283051	70283508	1.38	3.83E-01	CTTN	38668	SHANK2	652562
chr6	27655919	27656252	1.43	3.83E-01	ZNF184	-215189	HIST1H2BL	119623
chr20	49432211	49432472	0.80	3.83E-01	BCAS4	20875	ADNP	115185
chr5	134237935	134238247	0.79	3.83E-01	PCBD2	-2719		
chr3	16592360	16592604	1.27	3.83E-01	RFTN1	-37260	DAZL	54524
chr7	30325646	30325922	1.20	3.83E-01	ZNRF2	1861	NOD1	192609
chr1	150601895	150602255	1.17	3.84E-01	ENSA	23		
chr1	234748428	234748715	1.44	3.84E-01	IRF2BP2	-3301		
chr8	29387333	29387627	0.79	3.84E-01	DUSP4	-179295	TMEM66	553169
chr8	134156755	134157123	1.30	3.84E-01	SLA	-84336	WISP1	-46373
chr12	89919809	89920105	1.27	3.84E-01	GALNT4	-1422		
chr19	42724202	42724498	1.27	3.84E-01	DEDD2	-2537	ZNF526	-142
chr6	3259051	3259331	0.77	3.84E-01	TUBB2B	-31223	SLC22A23	197602
chr7	42951641	42952105	0.74	3.84E-01	C7orf25	278		
chr17	1933300	1933584	1.14	3.85E-01	DPH1	11		
chr5	140893350	140893922	0.80	3.85E-01	PCDHGC5	24828	DIAPH1	104986
chr6	42531423	42532025	0.77	3.85E-01	UBR2	-334		
chr6	14117634	14118089	0.82	3.85E-01	CD83	-3		
chr19	42829329	42829733	1.22	3.85E-01	MEGF8	-230		
chr19	6863562	6863895	1.16	3.86E-01	EMR1	-23853	VAV1	91007
chr7	6556014	6556280	0.65	3.86E-01	KDELR2	-32298	GRID2IP	34920
chr2	9816057	9816307	0.74	3.86E-01	TAF1B	-167389	YWHAQ	-45076
chr22	38240258	38240801	1.15	3.86E-01	EIF3L	-4840	ANKRD54	-227
chr13	27936164	27936408	1.18	3.87E-01	GTF3A	-62395	RASL11A	91822
chr11	6255683	6256130	0.68	3.87E-01	CNGA4	-4423	FAM160A2	34
chr2	136893730	136894162	0.77	3.87E-01	THSD7B	-854516	CXCR4	-18221
chr6	126277738	126278067	0.81	3.88E-01	HINT3	42		
chr3	101292593	101293013	1.32	3.88E-01	PCNP	-239		
chr15	79053714	79054186	0.86	3.88E-01	CHRNB4	-120363	ADAMTS7	49823
chr3	128879726	128880056	1.38	3.88E-01	ISY1	138		
chr12	8085883	8086202	0.78	3.89E-01	SLC2A3	2849	NANOGP1	60509
chr3	193855471	193855760	1.23	3.89E-01	HES1	1682	CPN2	216441
chr12	132568527	132568986	1.21	3.89E-01	DDX51	60123	EP400	134249
chrX	71401355	71401665	0.90	3.89E-01	PIN4	-16		
chr12	113667612	113667889	1.37	3.89E-01	TPCN1	8491	SLC24A6	105174

chr17	41116380	41116631	1.25	3.89E-01	AARSD1	16039	G6PC	63691
chr19	1812929	1813239	0.73	3.89E-01	ATP8B3	-814		
chr11	1858984	1859260	0.84	3.89E-01	TNNI2	-2310		
chr15	90728381	90728691	0.76	3.89E-01	IDH2	-82828	SEMA4B	-16026
chr7	22122531	22122841	0.70	3.89E-01	CDCA7L	-137144	RAPGEF5	273847
chr11	60609385	60609662	1.17	3.89E-01	CCDC86	95		
chr5	17282523	17282781	0.92	3.90E-01	BASP1	64902		
chr3	35706139	35706446	0.76	3.90E-01	STAC	-715804	ARPP-21	22444
chr16	88700511	88700889	1.08	3.90E-01	IL17C	-4301		
chr1	1822404	1822737	1.15	3.90E-01	GNB1	-76		
chr5	67511411	67511718	1.09	3.90E-01	PIK3R1	-10553		
chr12	111531252	111531518	0.70	3.90E-01	SH2B3	-312367	CUX2	59556
chr5	150594169	150594538	0.80	3.90E-01	ANXA6	-56987	GM2A	-38259
chr1	161675493	161675803	0.71	3.91E-01	FCRLA	-1114		
chr14	105956050	105956363	0.70	3.91E-01	TMEM121	-36746	CRIP1	2950
chr2	190525813	190526195	1.26	3.91E-01	ASNSD1	-121		
chr9	6681176	6681422	1.32	3.91E-01	KDM4C	-76342	GLDC	-35607
chr13	28057096	28057370	1.25	3.92E-01	MTIF3	-32522	LNX2	137487
chr17	73286151	73286395	1.24	3.92E-01	SLC25A19	-743		
chr17	19411361	19411649	1.33	3.92E-01	SLC47A1	-25662	RNF112	96982
chr22	37614783	37615280	1.20	3.92E-01	SSTR3	-6679	RAC2	25273
chr17	76712928	76713255	1.37	3.92E-01	DNAH17	-145688	CYTH1	65284
chr6	41700419	41700829	0.72	3.92E-01	TFEB	-9160	TFEB	2174
chr15	65903264	65903639	0.84	3.93E-01	C15orf44	-45		
chr7	128694883	128695358	1.22	3.93E-01	TNPO3	77		
chr10	1496669	1496966	0.68	3.93E-01	IDI1	-401757	ADARB2	282900
chr11	61647590	61647834	0.77	3.93E-01	FADS3	11294	FADS2	51999
chr7	140374145	140374456	0.84	3.93E-01	NDUFB2	-22180	ADCK2	1348
chr20	46040878	46041188	0.86	3.93E-01	NCOA3	-89624	ZMYND8	-55559
chr5	36151960	36152333	1.19	3.93E-01	LMBRD2	-132	SKP2	-42
chr6	30583217	30583576	0.81	3.93E-01	MRPS18B	-2089	PPP1R10	1623
chr17	29421632	29421947	0.82	3.93E-01	NF1	-205		
chr18	56338183	56338862	1.26	3.94E-01	MALT1	-95		
chr5	90677551	90677841	0.74	3.94E-01	ARRDC3	1453	GPR98	823079
chr11	61100512	61100893	1.33	3.94E-01	DDB1	-37	DAK	49
chr17	1303351	1303678	0.66	3.94E-01	YWHAE	41		
chr6	36515041	36515398	0.79	3.94E-01	STK38	27		
chr12	6960438	6960779	0.71	3.95E-01	USP5	-676	CDCA3	-153

chr11	102217745	102218056	1.26	3.95E-01	BIRC2	-65		
chr20	5093437	5093948	0.88	3.95E-01	C20orf30	40		
chr3	167098017	167098278	1.41	3.95E-01	ZBBX	-77		
chr19	36184744	36185054	1.22	3.95E-01	ZBTB32	-18931	UPK1A	27184
chr17	80656520	80656779	0.76	3.96E-01	RAB40B	-52		
chr3	119396224	119396698	1.27	3.96E-01	COX17	-218		
chr3	194412220	194412636	0.77	3.96E-01	LSG1	-19222	C3orf21	579467
chr16	3073990	3074455	1.24	3.96E-01	HCFC1R1	64	THOC6	191
chr8	38815022	38815336	1.40	3.96E-01	HTRA4	-16489	PLEKHA2	56426
chr5	133983895	133984351	0.86	3.97E-01	SEC24A	-356		
chr10	30994205	30994581	1.40	3.97E-01	AK302694	13190	ZNF438	326473
chr11	118740834	118741113	1.27	3.97E-01	DDX6	-79002	CXCR5	-13567
chr12	94633959	94634250	1.52	3.97E-01	PLXNC1	91606	TMCC3	410219
chr3	45429953	45430204	1.37	3.98E-01	LARS2	4		
chr2	112641571	112641961	0.91	3.98E-01	ANAPC1	-25		
chr16	57481246	57481563	0.78	3.98E-01	CIAPIN1	-36	COQ9	3
chr8	133969568	133969985	1.01	3.98E-01	TG	90572	SLA	102826
chr9	126085009	126085388	0.87	3.98E-01	STRBP	-54356	CRB2	-33249
chr20	25299261	25299591	1.28	3.99E-01	PYGB	70720	ABHD12	72051
chr10	7514019	7514363	1.27	3.99E-01	SFMBT2	-60741	ITIH5	194743
chr2	68993469	68993832	1.41	3.99E-01	ARHGAP25	31683	BMP10	104998
chr6	32157958	32158301	0.86	3.99E-01	PBX2	-167		
chr19	1438190	1438552	1.21	3.99E-01	RPS15	8		
chr7	99517084	99517393	0.76	3.99E-01	TRIM4	-85		
chr1	156736849	156737383	1.36	3.99E-01	HDGF	-15573	SH2D2A	49524
chr19	19599999	19600393	0.84	3.99E-01	TSSK6	26273	GATAD2A	103554
chr6	53200236	53200771	0.89	3.99E-01	GCM1	-186880	ELOVL5	13438
chr9	134248686	134249082	1.20	4.00E-01	BAT2L	-56593	PPAPDC3	83803
chr9	131843214	131843457	0.83	4.00E-01	DOLPP1	-47		
chr22	24199824	24200178	1.21	4.00E-01	SLC2A11	-40		
chr3	182833863	182834365	1.23	4.00E-01	MCCC1	-16749	LAMP3	46553
chr6	41392275	41392520	0.62	4.00E-01	FOXP4	-121766	NCR2	88870
chr3	182043089	182043399	0.69	4.00E-01	ATP11B	-468047	SOX2	613522
chr10	134385438	134385773	1.29	4.00E-01	INPP5A	34253	NKX6-2	213931
chr7	89874289	89874590	1.35	4.00E-01	C7orf63	-48		
chr22	39928713	39928996	1.22	4.00E-01	RPS19BP1	5		
chr3	128880117	128880399	1.28	4.00E-01	ISY1	-229		
chr6	160147765	160148106	0.80	4.00E-01	WTAP	-216		

chr9	19380075	19380557	0.81	4.00E-01	RPS6	-81		
chr5	145562007	145562494	1.32	4.00E-01	LARS	43		
chr17	80509753	80510017	0.81	4.00E-01	FOXK2	32291	RAB40B	146713
chr10	88281453	88281754	1.14	4.01E-01	WAPAL	-63		
chr16	15737057	15737341	1.19	4.01E-01	KIAA0430	-190		
chr19	50848027	50848606	1.31	4.01E-01	NAPSB	-312		
chr11	89956151	89956474	0.82	4.01E-01	CHORDC1	219		
chr1	168147913	168148405	1.22	4.02E-01	TIPRL	-12		
chr13	79233134	79233465	1.36	4.02E-01	RNF219	14		
chr5	130970888	130971433	1.23	4.03E-01	RAPGEF6	-232		
chr16	22202350	22202885	0.89	4.03E-01	EEF2K	-14974	VWA3A	98755
chr2	232478573	232478882	0.89	4.03E-01	PTMA	-94507	NMUR1	-83546
chr14	24835877	24836154	1.00	4.03E-01	NFATC4	-129		
chr19	796945	797279	1.35	4.03E-01	PTBP1	-280		
chr11	72533021	72533372	0.80	4.03E-01	ATG16L2	7746	FCHSD2	319946
chr16	87422078	87422420	1.11	4.03E-01	FBXO31	-4855	MAP1LC3B	-3552
chr1	54872967	54873502	0.85	4.04E-01	SSBP3	-1143		
chr11	58869657	58870056	0.82	4.04E-01	FAM111B	-4801		
chr19	14247316	14247761	1.09	4.04E-01	ASF1B	-99		
chr2	220462306	220462626	1.31	4.04E-01	STK11IP	-130		
chr22	50629843	50630287	1.12	4.04E-01	PANX2	20905	TUBGCP6	53335
chr5	36723999	36724297	1.23	4.04E-01	NIPBL	-152713	SLC1A3	117691
chr7	102067022	102067306	1.29	4.04E-01	ORAI2	-6832	PRKRIP1	30360
chr10	102673060	102673304	1.29	4.05E-01	SEMA4G	-59104	PAX2	167714
chr3	10234727	10235054	1.32	4.05E-01	TATDN2	-55286	IRAK2	28328
chr7	99102187	99102576	0.79	4.05E-01	ZNF394	-4505	ZKSCAN5	109
chr1	15852998	15853367	1.26	4.05E-01	CASP9	-2393	DNAJC16	-169
chr7	86848982	86849276	0.83	4.05E-01	C7orf23	-98		
chr19	15574575	15574885	0.73	4.06E-01	RASAL3	652		
chr3	197432589	197432930	1.31	4.06E-01	BDH1	-149902	KIAA0226	31013
chr11	66189102	66189413	1.21	4.06E-01	NPAS4	783		
chr19	55850862	55851266	1.04	4.06E-01	SUV420H2	-157		
chr15	64388022	64388342	1.26	4.06E-01	SNX1	5		
chr2	231533441	231533726	1.27	4.07E-01	CAB39	-43973	SP100	252713
chr22	50908319	50908636	0.77	4.07E-01	SBF1	4986	SAPS2	126718
chr6	52516901	52517158	0.88	4.07E-01	TRAM2	-75168	TMEM14A	-18854
chr19	42388197	42388620	1.22	4.07E-01	ARHGEF1	-37		
chr6	16420192	16420560	0.84	4.07E-01	GMPR	181565	ATXN1	341345

chr1	183439351	183439695	0.87	4.07E-01	SMG7	-2111		
chr12	17092794	17093111	1.27	4.07E-01	LMO3	-331929		
chr11	48131442	48131793	1.27	4.07E-01	OR4B1	-106744	PTPRJ	129508
chr14	62228877	62229138	1.20	4.08E-01	SNAPC1	-67		
chr22	39096911	39097162	1.22	4.08E-01	GTPBP1	-4770		
chr17	38136868	38137226	1.28	4.08E-01	PSMD3	-13		
chr15	98703339	98703746	0.81	4.08E-01	IGF1R	-489218		
chr15	45018841	45019151	1.38	4.08E-01	TRIM69	-9544	B2M	15311
chr2	74153839	74154083	1.21	4.08E-01	DGUOK	8		
chr2	232551770	232552229	0.74	4.08E-01	NMUR1	-156818	PTMA	-21235
chr5	156599430	156599700	0.91	4.08E-01	ITK	-8342	FAM71B	-6286
chr17	33914095	33914440	1.27	4.09E-01	AP2B1	-14		
chr6	30034529	30035192	0.77	4.09E-01	PPP1R11	-71		
chr19	2475973	2476290	1.06	4.09E-01	GADD45B	-3		
chr1	85742273	85742726	1.21	4.09E-01	SYDE2	-75772	BCL10	1083
chr17	78193312	78193577	1.30	4.09E-01	SLC26A11	-792	SGSH	754
chr16	8891419	8891764	1.18	4.10E-01	TMEM186	-87	PMM2	-78
chr2	64978385	64978771	1.20	4.10E-01	SLC1A4	-237878	SERTAD2	-97532
chr6	44046893	44047196	0.75	4.10E-01	MRPL14	48146	VEGFA	309092
chr12	57145491	57145814	0.96	4.10E-01	PRIM1	493		
chr6	43603295	43603774	0.82	4.10E-01	MAD2L1BP	-42		
chr16	81040987	81041317	1.23	4.10E-01	ATMIN	-28306	CENPN	1049
chr17	27055727	27056115	1.26	4.10E-01	TLCD1	-2691	NEK8	89
chr5	64920522	64920957	0.83	4.10E-01	TRIM23	-553		
chr2	201577775	201578099	1.32	4.10E-01	BZW1	-98710	AOX1	127206
chr1	225615759	225616145	1.17	4.11E-01	LBR	-168		
chr18	46987050	46987341	1.21	4.11E-01	DYM	-117		
chr12	113611154	113611472	1.26	4.11E-01	RASAL1	-37292	DDX54	11971
chr17	30936183	30936436	0.89	4.11E-01	CDK5R1	122205	MYO1D	267592
chr19	35814271	35814621	1.47	4.11E-01	CD22	-5633	MAG	31408
chr2	239016044	239016471	0.77	4.11E-01	KLHL30	-33159	SCLY	46626
chr1	26648030	26648359	0.70	4.11E-01	CD52	3784	AIM1L	22248
chr22	41864822	41865145	1.13	4.11E-01	PHF5A	-276	ACO2	-145
chr15	44580659	44580980	0.88	4.11E-01	CASC4	-109		
chr13	41019666	41020009	1.35	4.11E-01	FOXO1	220896	COG6	790074
chr2	202645811	202646081	1.23	4.11E-01	ALS2	-51		
chr6	26171954	26172287	0.83	4.11E-01	HIST1H2BE	-11903	HIST1H2BD	13772
chr19	1592793	1593080	1.12	4.12E-01	MBD3	-285		

chr4	166248312	166248622	0.89	4.12E-01	SC4MOL	-351		
chr19	1383672	1384025	1.36	4.12E-01	NDUFS7	142		
chr3	172428699	172429104	0.90	4.12E-01	NCEH1	106		
chr9	33290287	33290667	1.24	4.12E-01	NFX1	-33		
chr12	6570968	6571474	0.87	4.12E-01	VAMP1	8622	TAPBPL	10044
chr1	203258567	203258999	1.47	4.13E-01	CHIT1	-59923	BTG2	-15881
chr6	134274018	134274405	0.75	4.13E-01	TBPL1	-89		
chr13	33160367	33160676	0.78	4.13E-01	PDS5B	-70		
chr6	75953468	75953778	1.25	4.13E-01	COX7A2	-98		
chr8	128748308	128748611	0.79	4.13E-01	MYC	145		
chr17	7740131	7740460	1.18	4.13E-01	KDM6B	-2939		
chr12	27332779	27333091	0.86	4.14E-01	STK38L	-64143	MED21	157452
chr10	111976089	111976365	1.01	4.14E-01	MXI1	8864	SMNDC1	88480
chr7	102036514	102036887	0.81	4.14E-01	PRKRIP1	-103		
chr22	31885862	31886172	0.72	4.14E-01	EIF4ENIF1	-313		
chr15	44955148	44955613	0.74	4.14E-01	SPG11	495		
chr6	35656540	35656787	0.66	4.14E-01	FKBP5	55		
chr17	66342694	66343033	0.73	4.14E-01	SLC16A6	-55459	WIPI1	110789
chr5	112043025	112043288	1.30	4.14E-01	EPB41L4A	-288147	APC	-30399
chr7	74074145	74074566	0.84	4.15E-01	NCF1	-113953	GTF2I	2326
chr11	67236669	67237007	1.25	4.16E-01	TMEM134	-107		
chr7	102985106	102985453	1.34	4.16E-01	PSMC2	-2809	DNAJC2	40
chr3	186857142	186857727	1.22	4.16E-01	RPL39L	-172		
chr11	59436549	59436865	1.20	4.16E-01	PATL1	-196		
chr12	48551260	48551561	1.21	4.16E-01	ASB8	-34		
chr19	11491588	11492166	0.73	4.17E-01	EPOR	3142	LPPR2	25770
chr16	87840303	87840684	1.17	4.18E-01	KLHDC4	-40952	SLC7A5	62606
chr11	117821653	117821925	1.27	4.18E-01	IL10RA	-35317	TMPRSS13	-21674
chr2	55844640	55844977	1.26	4.18E-01	SMEK2	304		
chr3	25706362	25706720	0.78	4.19E-01	TOP2B	-710		
chr17	36981496	36981806	1.36	4.19E-01	PIP4K2B	-25493	RPL23	28402
chr18	48723920	48724276	0.75	4.19E-01	MEX3C	-408		
chr2	3622617	3622947	1.43	4.19E-01	RPS7	-71		
chr14	21945151	21945473	1.22	4.19E-01	RAB2B	-180	TOX4	-23
chr17	1627868	1628112	1.16	4.19E-01	PRPF8	-39814	SERPINF2	-18140
chr17	28443667	28444168	1.18	4.20E-01	CCDC55	84		
chr7	89975849	89976342	0.77	4.20E-01	GTPBP10	106		
chr1	156252597	156252907	1.28	4.20E-01	TMEM79	-1318	SMG5	-132

chr16	81515009	81515316	1.13	4.20E-01	PLCG2	-297767	CMIP	36388
chr17	30771260	30771580	1.23	4.20E-01	PSMD11	-82		
chr3	142720225	142720508	1.27	4.20E-01	SR140	-5		
chr20	43724436	43724746	0.95	4.20E-01	KCNS1	5162	STK4	129471
chr11	118965996	118966306	1.21	4.20E-01	H2AFX	26		
chr19	4471499	4471850	1.31	4.21E-01	HDGF2	-580		
chr3	37035088	37035560	1.22	4.21E-01	EPM2AIP1	-529	MLH1	345
chr16	12058677	12058981	1.15	4.21E-01	TNFRSF17	-135		
chr2	209130612	209130978	1.33	4.21E-01	PIKFYVE	-196		
chr3	40350773	40351210	1.32	4.21E-01	EIF1B	-181		
chr18	32870057	32870378	1.29	4.21E-01	ZNF397OS	-46	ZNFPHEX133	-18
chr17	47439847	47440157	0.79	4.21E-01	ZNF652	-167		
chr11	119067522	119067911	1.24	4.21E-01	CBL	-9273	PDZD3	11522
chr20	48419127	48419395	1.46	4.21E-01	B4GALT5	-88840	SLC9A8	-9989
chr6	26026518	26026810	0.77	4.22E-01	HIST1H4B	816		
chr12	7079688	7080095	1.22	4.22E-01	EMG1	-52	PHB2	24
chr12	32049531	32049841	1.24	4.22E-01	BICD1	-210499	H3F3C	-104511
chr13	21872104	21872405	1.12	4.22E-01	SKA3	-121545	ZDHHC20	161168
chr2	112917174	112917489	1.50	4.23E-01	FBLN7	21370	ZC3H8	95332
chr6	30524617	30524879	0.82	4.23E-01	PRR3	-8	GNL1	260
chr3	152879109	152879437	0.74	4.23E-01	RAP2B	-756		
chr3	186500837	186501154	1.10	4.23E-01	EIF4A2	-365		
chr17	55682829	55683116	1.27	4.23E-01	MRPS23	244426	MSI2	349042
chr3	183602550	183602865	1.25	4.23E-01	PARL	-15		
chr14	59768117	59768510	1.32	4.23E-01	DAAM1	112915	GPR135	163745
chr2	114647248	114647588	1.12	4.24E-01	ACTR3	-119		
chr12	113646372	113646654	1.12	4.24E-01	DDX54	-23229	TPCN1	-12747
chr5	134389030	134389544	0.87	4.24E-01	PITX1	-19323	H2AFY	345641
chr10	27792957	27793360	1.11	4.24E-01	RAB18	-90		
chr3	41240732	41241119	0.85	4.25E-01	CTNNB1	-16		
chr5	110427804	110428174	0.92	4.25E-01	WDR36	119		
chr14	50329505	50329813	1.32	4.25E-01	ARF6	-30077	SDCCAG1	-10120
chr3	183484823	183485133	1.26	4.25E-01	MAP6D1	58415	YEATS2	69372
chr11	1545083	1545391	0.73	4.25E-01	HCCA2	-43122	DUSP8	47913
chr6	27806028	27806311	0.88	4.25E-01	HIST1H2BN	-270	HIST1H2AK	-53
chr14	106175839	106176084	1.21	4.25E-01	IGHE	153500	TMEM121	183009
chr17	76143416	76144105	1.23	4.25E-01	SYNGR2	-20910	C17orf99	1327
chr20	62689156	62689436	1.33	4.26E-01	TCEA2	857		

chr1	234745817	234746168	1.26	4.26E-01	IRF2BP2	-722		
chr5	109024401	109024670	0.90	4.26E-01	MAN2A1	-620		
chr3	160117243	160117742	1.33	4.26E-01	IFT80	-173	SMC4	63
chr4	17578590	17578941	1.20	4.26E-01	LAP3	-161		
chr6	150067609	150067894	0.71	4.26E-01	PCMT1	-3079	NUP43	-64
chr22	36784086	36784524	1.15	4.26E-01	MYH9	-242		
chr3	105974735	105975078	0.87	4.26E-01	CBLB	-387020		
chr2	175499187	175499853	1.41	4.26E-01	WIPF1	-213		
chr21	38593519	38593826	0.94	4.26E-01	DSCR3	46160	TTC3	148102
chr19	18414310	18414644	1.35	4.27E-01	JUND	-22045	LSM4	19524
chr11	66445816	66446258	1.28	4.27E-01	RBM4B	-762		
chr12	121454206	121454450	1.26	4.27E-01	OASL	22452	HNF1A	37779
chr6	119031251	119031501	1.51	4.27E-01	MCM9	159983	PLN	161934
chr17	27068563	27068931	0.81	4.27E-01	TRAF4	-2276		
chr7	64147664	64147941	0.75	4.27E-01	ZNF138	-106968	ZNF107	21292
chr5	138739571	138739905	0.84	4.28E-01	SPATA24	38		
chr16	69364380	69364670	1.10	4.28E-01	PDF	-27		
chr16	28985898	28986233	1.33	4.29E-01	SPNS1	-30		
chr17	19265804	19266114	0.83	4.29E-01	B9D1	87	b9	87
chr11	67044937	67045276	1.30	4.29E-01	SSH3	-25812	ADRBK1	11202
chr14	67826390	67826724	1.16	4.29E-01	EIF2S1	-477	ATP6V1D	34
chr4	113558282	113558854	1.31	4.29E-01	C4orf21	-417	LARP7	-44
chr11	124543447	124544016	0.91	4.29E-01	SIAE	-15	SPA17	-8
chr5	133702788	133703065	1.38	4.29E-01	UBE2B	-3943	CDKL3	-162
chr16	67694198	67694926	0.76	4.29E-01	PARD6A	-289	ACD	156
chr1	13992210	13992520	0.82	4.29E-01	PRDM2	-38985	PDPN	82113
chr19	45458088	45458646	1.25	4.29E-01	CLPTM1	-271		
chr19	5903638	5904019	1.21	4.29E-01	VMAC	-1023	NDUFA11	196
chr3	139062680	139063008	1.19	4.29E-01	MRPS22	46		
chr16	29984691	29985029	0.87	4.29E-01	TAOK2	-362		
chr6	44205708	44205982	0.74	4.29E-01	HSP90AB1	-9004	SLC29A1	14549
chr17	25659552	25659824	1.20	4.30E-01	KSR1	-139348	WSB1	38582
chr18	74204768	74205118	1.02	4.30E-01	ZNF236	-331173	ZNF516	-29846
chr20	62362074	62362480	1.38	4.30E-01	LIME1	-4856		
chr8	66546453	66546764	0.89	4.31E-01	ARMC1	-177		
chr15	60771205	60771483	1.17	4.31E-01	NARG2	0		
chr12	98987247	98987528	1.22	4.31E-01	SLC25A3	-15		
chr3	182510894	182511260	1.13	4.31E-01	ATP11B	-214		

chr10	93683279	93683663	0.88	4.31E-01	BTAF1	-265		
chr1	181057722	181058054	1.19	4.31E-01	CACNA1E	-394828	MR1	54749
chr16	53537208	53537490	1.09	4.32E-01	AKTIP	-179		
chr20	30865217	30865540	1.21	4.32E-01	KIF3B	-88		
chr11	46260749	46260989	1.26	4.32E-01	PHF21A	-117884	CREB3L1	-38359
chr3	169872263	169872748	0.94	4.32E-01	PHC3	27031	GPR160	116771
chr1	36689979	36690397	1.21	4.32E-01	THRAP3	171		
chr15	85839911	85840258	1.31	4.32E-01	AKAP13	-83786	PDE8A	314880
chr12	54582526	54582974	0.87	4.33E-01	SMUG1	7		
chr6	41336638	41336926	0.80	4.33E-01	FOXP4	-177382	NCR2	33254
chr4	185654450	185654726	1.00	4.33E-01	MLF1IP	698		
chr9	130741685	130741995	0.83	4.34E-01	DPM2	-41077	NAIF1	87759
chr12	125411739	125412050	1.40	4.34E-01	UBC	-12318	DHX37	61772
chr3	169581272	169581531	1.37	4.35E-01	LRRC31	6258	LRRIQ4	41692
chr7	148397866	148398145	0.81	4.35E-01	CUL1	2073	EZH2	183408
chr10	112257432	112257696	0.64	4.35E-01	DUSP5	-61		
chr11	450088	450337	1.32	4.35E-01	PTDSS2	-67		
chr1	184020639	184020902	1.24	4.35E-01	TSEN15	-40		
chr11	10329399	10329957	0.86	4.35E-01	AMPD3	-142546	ADM	3036
chr9	139118006	139118256	1.11	4.35E-01	LHX3	-23127	QSOX2	19556
chr19	12996051	12996323	1.45	4.35E-01	DNASE2	-3852		
chr21	16135482	16135807	1.39	4.36E-01	SAMSN1	-216981	NRIP1	301481
chr6	34855676	34856048	0.89	4.36E-01	ANKS1A	-1176	TAF11	-43
chr1	167189170	167189574	0.92	4.36E-01	POU2F1	-771		
chr1	41134449	41134797	1.24	4.36E-01	RIMS3	-3299		
chr12	46782228	46782604	1.10	4.36E-01	SLC38A2	-15771	SLC38A4	437364
chr7	150594604	150594896	1.38	4.36E-01	ABP1	45177	KCNH2	80264
chr17	40713987	40714264	1.35	4.37E-01	MLX	-4952	COASY	34
chr7	86848153	86848570	0.84	4.37E-01	C7orf23	669		
chr7	30387752	30388000	0.81	4.37E-01	ZNRF2	63953	NOD1	130517
chr5	64777663	64777907	1.20	4.38E-01	ADAMTS6	-81		
chr12	32291955	32292293	1.28	4.38E-01	FGD4	-362917	BICD1	31939
chr1	109969064	109969432	0.79	4.38E-01	PSMA5	-211		
chr20	33680529	33680862	1.20	4.38E-01	TRPC4AP	-78		
chr3	49449491	49449838	1.17	4.39E-01	RHOA	-139		
chr4	6717634	6717990	1.19	4.39E-01	CNO	-30		
chr3	4508788	4509118	0.78	4.39E-01	SUMF1	1		
chr2	220094179	220094666	1.24	4.39E-01	ATG9A	-62	ANKZF1	-56

chr1	6761813	6762093	1.29	4.39E-01	DNAJC11	13		
chr3	10362695	10362969	1.13	4.39E-01	SEC13	26		
chr2	37617548	37617919	1.46	4.39E-01	QPCT	45981	CDC42EP3	281592
chr19	30096983	30097313	1.19	4.39E-01	POP4	-22		
chr15	93461123	93461433	1.21	4.39E-01	CHD2	17727	RGMA	171155
chr4	80979951	80980197	0.91	4.39E-01	GK2	-650702	ANTXR2	14403
chr2	99771239	99771659	1.22	4.40E-01	TSGA10	-262	LIPT1	31
chr11	18127502	18127859	0.88	4.40E-01	SAAL1	-43		
chr1	227015782	227016139	1.33	4.40E-01	ITPKB	-89085	PSEN2	-42312
chr7	142246198	142246434	1.38	4.40E-01	TCRBV1S1A1N1	-6283	TCRBV6S1A1N1	1299
chr9	128023674	128024213	1.16	4.40E-01	GAPVD1	-167		
chr13	41163828	41164138	1.28	4.40E-01	FOXO1	76751	COG6	934219
chr3	195634685	195635019	0.83	4.41E-01	TNK2	-12420	SDHALP1	82298
chr1	32479159	32479559	1.11	4.41E-01	KHDRBS1	-132		
chr17	8095519	8095969	0.76	4.41E-01	C17orf59	-2180		
chr16	3333313	3333623	1.17	4.41E-01	ZNF263	-19		
chr19	35503931	35504207	1.22	4.41E-01	SCN1B	-17465	GRAMD1A	12823
chr7	7291427	7291727	0.79	4.41E-01	C1GALT1	69331	COL28A1	283883
chr16	2097722	2098033	1.26	4.42E-01	TSC2	-112	NTHL1	-11
chr10	46983707	46983984	0.87	4.42E-01	PPYR1	-99688	SYT15	-13245
chr11	65407106	65407386	0.76	4.42E-01	SIPA1	1668	RELA	23197
chr16	2205223	2205682	1.19	4.42E-01	TRAF7	-346		
chr3	156799562	156799862	0.91	4.42E-01	CCNL1	78770	TIPARP	407333
chr5	71597504	71597756	0.91	4.42E-01	MRPS27	18454	MAP1B	194512
chr6	24891273	24891578	0.97	4.42E-01	FAM65B	19769	GMNN	116262
chr15	76196001	76196414	1.19	4.43E-01	FBXO22	8		
chr4	51607	51933	1.62	4.43E-01	ZNF718	-1457		
chr20	13765420	13765730	1.30	4.43E-01	C20orf7	-107	ESF1	-43
chr10	49864320	49864594	0.71	4.43E-01	ARHGAP22	-51319	WDFY4	-29061
chr10	126407744	126407998	1.40	4.43E-01	FAM53B	25059	LHPP	257459
chr15	93363563	93363985	1.28	4.43E-01	FAM174B	-164743	CHD2	-79777
chr11	64084774	64085295	1.32	4.44E-01	PRDX5	-534	TRMT112	-2
chr6	227333	227661	1.13	4.44E-01	DUSP22	-64604		
chr16	21371395	21371822	1.16	4.44E-01	CRYM	-81952	NPIPL3	65090
chr19	17637817	17638093	1.19	4.44E-01	GLT25D1	-28556	PGLS	15523
chr3	188672417	188672706	1.43	4.44E-01	TPRG1	-217201	LPP	741841
chr14	105123394	105123704	0.95	4.44E-01	TMEM179	-52452	INF2	-32394
chr7	152373157	152373448	1.12	4.45E-01	XRCC2	-53		

chr1	204476044	204476414	1.37	4.46E-01	PIK3C2B	-16755	MDM4	-9282
chr6	35435885	35436323	0.83	4.46E-01	RPL10A	-74		
chr9	36487730	36488064	0.67	4.46E-01	RNF38	-87601	MELK	-85008
chr19	14097330	14097607	1.20	4.46E-01	PODNL1	-33265	RFX1	19665
chr2	198299619	198299984	1.25	4.47E-01	SF3B1	-31		
chr19	597519	597907	0.74	4.47E-01	HCN2	7820	POLRMT	35855
chr2	101840257	101840658	0.94	4.47E-01	TBC1D8	-72612	C2orf29	-28887
chr17	42404164	42404515	0.81	4.47E-01	SLC25A39	-2123		
chr1	40851291	40851616	1.32	4.47E-01	ZNF643	-64325	SMAP2	11726
chr19	2613814	2614166	0.70	4.47E-01	GNG7	88756	GADD45B	137855
chr5	94890578	94890940	1.27	4.47E-01	TTC37	-77	ARSK	-66
chr16	3200516	3200875	1.35	4.48E-01	OR1F1	-53551	caspace	6476
chr2	224821829	224822299	1.28	4.48E-01	MRPL44	-57		
chr14	23357801	23358243	0.79	4.48E-01	REM2	5590	RBM23	30374
chr2	214097799	214098167	0.80	4.48E-01	IKZF2	-82925	SPAG16	-51133
chr22	41347120	41347613	1.13	4.48E-01	RBX1	-18		
chr11	18343969	18344253	0.87	4.48E-01	HPS5	-390	GTF2H1	295
chrX	1621183	1621523	0.86	4.48E-01	ASMTL	-49509	P2RY8	34684
chr17	38375311	38375589	1.25	4.48E-01	WIPF2	-124		
chr20	5639582	5639860	1.14	4.48E-01	CHGB	-252253	RP5-1022P6.2	-48049
chr17	4699210	4699622	1.33	4.48E-01	PSMB6	-41		
chr22	31686555	31686976	0.71	4.49E-01	PIK3IP1	1754	LIMK2	78516
chr9	95087421	95087814	1.25	4.49E-01	CENPP	-123	NOL8	258
chr2	29117282	29117601	1.27	4.49E-01	WDR43	-91		
chr8	144598620	144599005	1.06	4.49E-01	MAFA	-86237	ZC3H3	24807
chr7	36429317	36429725	0.79	4.50E-01	ANLN	89		
chr1	155990629	155990887	1.35	4.50E-01	SSR2	0		
chr3	157815929	157816173	1.28	4.50E-01	VEPH1	-598606	SHOX2	7901
chr4	176272664	176273047	1.43	4.50E-01	ADAM29	433347	GPM6A	650792
chr11	3013570	3013814	1.27	4.50E-01	NAP1L4	-85		
chr13	50698506	50698774	1.25	4.50E-01	ST13	-47514	KCNRG	109250
chr3	9404517	9404849	1.36	4.50E-01	THUMPD3	-34		
chr15	31727531	31727911	0.70	4.50E-01	KLF13	108638	OTUD7A	219821
chr15	65596620	65596877	1.31	4.50E-01	PARP16	-17731	IGDCC3	73629
chr3	47323950	47324440	1.11	4.51E-01	KLHL18	-135	KIF9	142
chr15	50716460	50716735	1.11	4.51E-01	USP8	19		
chr7	20259617	20259944	0.77	4.51E-01	MACC1	-2768		
chr11	72889155	72889414	0.78	4.51E-01	P2RY2	-40059	FCHSD2	-36142

chr13	28712197	28712470	1.31	4.51E-01	FLT3	-37605	PAN3	-36075
chr15	59063230	59063546	1.23	4.51E-01	ADAM10	-21211	SLTM	162464
chr14	52313786	52314289	0.94	4.52E-01	GNG2	-13012	FRMD6	195462
chr1	36615114	36615461	1.21	4.52E-01	TRAPPC3	-221		
chr5	141044586	141044929	1.34	4.52E-01	ARAP3	17042	RELL2	28241
chr14	21539543	21539823	1.43	4.52E-01	C14orf176	-27413	FLJ10357	1156
chr20	48429064	48429412	1.26	4.53E-01	SLC9A8	-12		
chr2	32581916	32582270	1.20	4.53E-01	BIRC6	-3		
chr3	121711497	121711824	0.85	4.53E-01	ILDR1	29369	SLC15A2	98490
chr1	44678948	44679258	0.81	4.53E-01	DMAP1	-22		
chr15	72410414	72410960	1.12	4.54E-01	MYO9A	-265	SENP8	-67
chr9	140473245	140473567	1.10	4.54E-01	ZMYND19	11531	MRPL41	27097
chr6	43597688	43598057	0.84	4.54E-01	GTPBP2	-937		
chr10	121066101	121066479	0.79	4.54E-01	GRK5	99093	RGS10	235932
chr20	50157844	50158271	1.04	4.54E-01	KCNG1	-518383	NFATC2	1200
chr7	139876792	139877058	1.11	4.54E-01	JHDM1D	-184		
chr15	42565423	42565882	1.27	4.54E-01	GANC	-713	TMEM87A	102
chr2	26568462	26569066	1.21	4.55E-01	SELI	-190		
chr5	43105796	43106106	0.79	4.55E-01	C5orf39	-65504	ZNF131	-15691
chr13	79968000	79968313	0.88	4.55E-01	RNF219	-734843	RBM26	11766
chr12	132413614	132413976	1.27	4.55E-01	PUS1	6		
chr2	219264284	219264713	0.75	4.55E-01	CTDSP1	21		
chr2	223520657	223521094	0.83	4.55E-01	FARSB	-49		
chr3	14989579	14989919	0.75	4.55E-01	NR2C2	513		
chr13	40700496	40700866	0.80	4.55E-01	COG6	470917	FOXO1	540053
chr3	58335589	58335980	0.91	4.55E-01	PXK	17168	PDHB	83780
chr8	27221337	27221625	0.79	4.55E-01	PTK2B	38427	CHRNA2	115332
chr4	146540316	146540736	1.13	4.56E-01	MMAA	-14		
chr1	205180725	205181047	1.34	4.56E-01	DSTYK	-159		
chr1	118878106	118878378	0.88	4.56E-01	SPAG17	-150394	TBX15	653937
chr1	6403562	6403806	1.14	4.57E-01	GPR153	-82649	ACOT7	42199
chr19	56146138	56146456	1.14	4.57E-01	ZNF784	-10356	ZNF580	-6095
chr9	127952064	127952397	1.15	4.57E-01	PPP6C	-13		
chr5	140699779	140700089	0.80	4.58E-01	TAF7	417		
chr3	128578458	128578694	0.76	4.58E-01	AK294171	5715	RAB7A	133597
chr5	133561699	133562156	1.17	4.58E-01	SKP1	-49204	CDKL3	140837
chr20	5931101	5931480	1.21	4.58E-01	TRMT6	-118	MCM8	-7
chr1	17232134	17232463	0.73	4.58E-01	MSTP9	-141324	CROCC	-16146

chr2	43823096	43823382	1.13	4.58E-01	THADA	-54		
chr12	56334075	56334352	0.75	4.59E-01	DGKA	8402	SILV	25632
chr12	110448992	110449377	1.38	4.59E-01	IFT81	-112955	GIT2	-14991
chr7	103848332	103848730	0.73	4.59E-01	ORC5L	-68		
chr15	63771845	63772201	1.41	4.59E-01	CA12	-97948	USP3	-24787
chr8	101571898	101572175	0.94	4.59E-01	ANKRD46	-25		
chr11	76348499	76348908	0.81	4.59E-01	LRRC32	32340	C11orf30	192635
chr15	79296838	79297220	1.03	4.59E-01	CTSH	-59609	RASGRF1	86186
chr6	27106833	27107307	1.47	4.59E-01	HIST1H4I	-18		
chr19	16695176	16695552	1.20	4.59E-01	SLC35E1	-12171	MED26	43651
chr22	39795636	39795912	1.19	4.60E-01	MAP3K7IP1	15		
chr12	7068170	7068562	1.17	4.60E-01	PHB2	11550	PTPN6	12626
chr1	45452130	45452481	0.81	4.60E-01	EIF2B3	-24		
chr3	101395840	101396195	1.33	4.60E-01	ZBTB11	-30		
chr6	30539113	30539466	0.81	4.60E-01	ABCF1	120		
chr16	30483089	30483339	0.98	4.61E-01	ITGAL	-769		
chr10	3827489	3827785	0.93	4.61E-01	KLF6	-164		
chr22	24094543	24095052	0.79	4.61E-01	ZNF70	-1519		
chr13	28024554	28024847	1.27	4.61E-01	MTIF3	10		
chr11	61666667	61666938	1.12	4.61E-01	FADS3	-7797	RAB3IL1	18194
chr16	85936560	85936836	1.18	4.61E-01	FOXF1	-607435	IRF8	3924
chr19	39881468	39882072	1.31	4.61E-01	MED29	-193	PAF1	-91
chr6	28104502	28104812	0.85	4.61E-01	ZNF192	-5059	ZSCAN16	12270
chr5	44808757	44809006	1.33	4.61E-01	MRPS30	-145		
chr19	5720099	5720883	1.28	4.61E-01	LONP1	-315	TMEM146	-197
chr13	31735941	31736685	0.72	4.61E-01	HSPH1	-196		
chr19	36545152	36545494	1.31	4.61E-01	THAP8	341		
chr8	67564234	67564537	0.81	4.61E-01	MYBL1	-38906	VCPIP1	15066
chr13	20756445	20756721	1.22	4.61E-01	GJA3	-21400	GJB2	10531
chr1	28574949	28575330	0.69	4.61E-01	SESN2	-10866	ATPIF1	12529
chr14	54080247	54080725	1.14	4.61E-01	DDHD1	-460440	BMP4	340784
chr6	37759282	37759526	0.76	4.61E-01	MDGA1	-93638	ZFAND3	-27903
chr6	26533002	26533359	0.87	4.62E-01	HMGN4	-5391	BTN1A1	31686
chr1	100435250	100435642	0.78	4.63E-01	SLC35A3	-94		
chr12	11801617	11801954	1.39	4.63E-01	ETV6	-1002		
chr17	48229346	48229637	0.82	4.63E-01	PPP1R9B	-1615		
chr6	33668291	33668542	0.82	4.63E-01	C6orf125	11087	ITPR3	79256
chr4	48832909	48833171	1.25	4.63E-01	OCIAD1	25		

chr3	188299674	188299939	1.52	4.63E-01	TPRG1	-589956	LPP	369086
chr17	1496182	1496482	1.31	4.63E-01	PITPNA	-30222	SLC43A2	35798
chr4	164415597	164415971	1.41	4.63E-01	TKTL2	-20737	MARCH1	118992
chr19	572228	572612	1.12	4.63E-01	BSG	-117		
chr16	50315940	50316253	0.74	4.63E-01	ADCY7	-5726	PAPD5	128439
chr7	98270965	98271744	0.75	4.64E-01	NPTX2	24758	TMEM130	196318
chr10	112678794	112679149	0.77	4.64E-01	SHOC2	-329		
chr19	45004508	45004920	1.09	4.64E-01	ZNF180	-140		
chr7	105162537	105162781	1.07	4.64E-01	PUS7	26		
chr2	89155064	89155761	0.85	4.64E-01	O1/O11 and JK2	-735149	RPIA	164237
chr7	100472304	100472882	0.82	4.64E-01	SRRT	-108		
chr15	70805131	70805441	0.76	4.64E-01	TLE3	-415030	UACA	250564
chr19	2151605	2151980	1.08	4.64E-01	AP3D1	-237		
chr7	56131834	56132092	0.84	4.65E-01	SUMF2	46		
chr11	66175936	66176213	1.23	4.65E-01	SLC29A2	-36784	NPAS4	-12400
chr16	3450977	3451486	1.20	4.65E-01	ZNF434	-219	ZNF174	42
chr4	148978143	148978469	0.92	4.65E-01	ARHGAP10	324853	NR3C2	385337
chr7	129588626	129589024	0.82	4.65E-01	UBE2H	3964	NRF1	337270
chr4	174290879	174291406	0.80	4.66E-01	SAP30	-950		
chr11	14913128	14913409	1.09	4.66E-01	CYP2R1	482		
chr13	46742602	46742861	1.16	4.66E-01	CPB2	-63521	LCP1	13727
chr19	11616580	11616843	0.76	4.66E-01	ZNF653	-58		
chr3	183165147	183165474	1.32	4.66E-01	MCF2L2	-19456	KLHL6	108188
chr7	44240379	44240685	0.85	4.66E-01	YKT6	-46		
chr11	62572789	62573120	1.20	4.67E-01	NXF1	9		
chr19	58891966	58892264	1.18	4.67E-01	ZNF837	274		
chr8	71520511	71520836	0.73	4.67E-01	TRAM1	-70		
chr1	38478003	38478400	1.29	4.67E-01	UTP11L	-182		
chr19	54704875	54705196	1.25	4.67E-01	RPS9	310		
chr2	30387930	30388200	1.30	4.67E-01	ALK	-243633	LBH	-66332
chr1	227922905	227923265	1.26	4.68E-01	SNAP47	388		
chr6	36842397	36842800	0.88	4.68E-01	PPIL1	201		
chr12	111057307	111057723	0.86	4.68E-01	TCTN1	5635	HVCN1	69431
chr12	124118180	124118491	1.18	4.68E-01	EIF2B1	-89	GTF2H3	-45
chr21	30665873	30666183	0.79	4.68E-01	BACH1	-5192	C21orf7	213155
chr15	75079700	75080506	1.39	4.68E-01	LMAN1L	-25091	CSK	5678
chr6	36270259	36270569	0.93	4.68E-01	PNPLA1	32177	ETV7	85053
chr1	156695653	156695965	0.82	4.69E-01	C1orf66	-2454	ISG20L2	1896

chr11	75400872	75401244	1.58	4.69E-01	MOGAT2	-27876	MAP6	-21579
chr1	151170810	151171377	0.84	4.69E-01	PIP5K1A	73		
chr2	68961872	68962184	1.33	4.69E-01	ARHGAP25	60		
chr21	30375011	30375262	1.14	4.69E-01	USP16	-21801	RNF160	-9860
chr10	104238772	104239087	0.68	4.69E-01	TMEM180	17760	ACTR1A	23582
chr6	26234642	26235265	0.78	4.70E-01	HIST1H1D	262		
chr10	105156246	105156577	1.29	4.70E-01	USMG5	-189	PDCD11	0
chr19	4863878	4864133	0.94	4.70E-01	TICAM1	-32269	PLIN3	3774
chr17	80170605	80170872	1.15	4.70E-01	FASN	-114633	SLC16A3	-15554
chr3	183852915	183853265	1.21	4.70E-01	EIF2B5	280		
chr3	187636865	187637222	1.27	4.71E-01	LPP	-293677	BCL6	-173569
chr6	53036699	53037038	0.83	4.71E-01	GCM1	-23245	ELOVL5	177073
chr1	179923528	179923889	0.90	4.71E-01	CEP350	-199		
chr7	7984467	7984959	1.21	4.71E-01	RPA3	-226475	GLCCI1	-23710
chr6	20401905	20402277	0.77	4.71E-01	E2F3	-46		
chr4	154389757	154390067	1.18	4.72E-01	TLR2	-215529	KIAA0922	2414
chr3	156877816	156878274	1.30	4.72E-01	CCNL1	437		
chr11	82611492	82611882	0.77	4.72E-01	C11orf82	-1050	PRCP	-130
chr17	16905159	16905422	0.79	4.72E-01	KIAA0864	-162683	TNFRSF13B	-29889
chr9	108006482	108006861	1.17	4.72E-01	SLC44A1	-257		
chr4	146970930	146971243	1.26	4.72E-01	LSM6	-125748	ZNF827	-111480
chr9	116344196	116344559	1.12	4.72E-01	ZNF618	-294184	RGS3	137367
chr1	22351682	22352049	0.79	4.73E-01	CDC42	-27254	CELA3B	48448
chr7	24365293	24365650	0.87	4.73E-01	MPP6	-247613	NPY	41663
chr11	118966308	118966699	1.28	4.73E-01	H2AFX	-327		
chr17	62493062	62493342	1.15	4.73E-01	POLG2	-33		
chr20	31366238	31366548	1.12	4.73E-01	MAPRE1	-41306	DNMT3B	16202
chr3	48274163	48274569	1.20	4.73E-01	ZNF589	-8230	CAMP	9504
chr6	28615935	28616183	1.30	4.73E-01	SCAND3	-60947	TRIM27	275709
chr2	182170442	182170691	0.81	4.73E-01	ITGA4	-151052	UBE2E3	325455
chr12	56546006	56546393	1.15	4.73E-01	MYL6B	-135		
chr19	12886383	12886719	0.92	4.73E-01	HOOK2	-117		
chr17	5342277	5342785	1.23	4.73E-01	C1QBP	-60		
chr2	32264789	32265066	1.17	4.73E-01	DPY30	-84		
chr1	157776339	157776919	0.79	4.73E-01	FCRL2	-29707	FCRL1	13311
chr12	125479706	125480104	0.89	4.73E-01	AACS	-70020	BRI3BP	1711
chr6	170101996	170102378	0.75	4.73E-01	C6orf120	-70		
chr12	2364890	2365254	0.79	4.74E-01	FKBP4	-539036	CACNA1C	202656

chr5	145582873	145583349	0.82	4.74E-01	RBM27	-52		
chr12	25205331	25205654	0.84	4.74E-01	LRMP	252		
chr15	52121758	52122087	0.98	4.74E-01	TMOD3	34		
chr15	83474777	83475113	1.37	4.74E-01	WHAMM	-3028		
chr11	67381509	67381849	0.89	4.74E-01	NDUFV1	7356	NUDT8	15722
chr5	180645062	180645505	1.39	4.74E-01	TRIM7	-13107	TRIM41	-5022
chr6	30312818	30313121	0.85	4.74E-01	HLA-E	-144301	TRIM39	17962
chr9	107686113	107686423	0.91	4.74E-01	ABCA1	4168	NIPSNAP3B	159817
chr2	60577913	60578273	1.26	4.75E-01	BCL11A	202540		
chr17	36740558	36740903	0.82	4.75E-01	SNIP	21452	ARHGAP23	127087
chr6	26123106	26123690	1.28	4.75E-01	HIST1H2AC	-975	HIST1H2BC	734
chr11	63953137	63953767	0.66	4.75E-01	STIP1	-135		
chr1	212965020	212965330	1.25	4.75E-01	NSL1	-36	TATDN3	5
chr15	100105870	100106304	1.26	4.75E-01	MEF2A	-46		
chr2	220143691	220144184	1.08	4.75E-01	DNAJB2	-102		
chr20	2451318	2451722	1.29	4.75E-01	SNRPB	-21		
chr3	135915143	135915497	1.24	4.75E-01	MSL2	-632		
chr16	67554990	67555470	0.81	4.75E-01	AGRP	-37514	FAM65A	-7524
chr1	110576999	110577295	1.18	4.75E-01	FAM40A	-94		
chr3	187687424	187687774	0.87	4.75E-01	LPP	-243122	BCL6	-224124
chr11	327076	327461	1.22	4.75E-01	B4GALNT4	-42526	IFITM3	-6355
chr17	45908790	45909075	1.10	4.75E-01	LRRC46	-167	MRPL10	-30
chr3	196014459	196014949	1.14	4.76E-01	PCYT1A	-120		
chr8	67579356	67579605	0.81	4.76E-01	VCPIP1	-29		
chr7	106535639	106535963	1.27	4.76E-01	PRKAR2B	-149377	PIK3CG	29877
chr9	115248870	115249110	1.30	4.76E-01	HSDL2	106639	C9orf80	231397
chr19	14682765	14683122	1.38	4.77E-01	NDUFB7	-58		
chr13	51375514	51375806	1.28	4.77E-01	RNASEH2B	-108232	ST13	629506
chr2	237966569	237966917	1.31	4.77E-01	COPS8	-27341	CXCR7	488363
chr20	37063892	37064202	1.38	4.77E-01	RALGAPB	-37439	LBP	89162
chr3	101443283	101443533	1.44	4.77E-01	CEP97	-86		
chr14	90849649	90849950	1.13	4.77E-01	C14orf102	-51521	CALM1	-13573
chr1	153643345	153643881	1.46	4.77E-01	ILF2	-134		
chr11	121190001	121190285	1.35	4.77E-01	SORL1	-132818	SC5DL	26755
chr7	74064386	74064700	1.15	4.78E-01	GTF2I	-7487	GTF2IRD1	196423
chr8	144623657	144623901	1.04	4.78E-01	ZC3H3	-159		
chr4	13629248	13629631	1.16	4.78E-01	BOD1L	-112		
chr12	32112026	32112365	0.83	4.78E-01	H3F3C	-167021	BICD1	-147989

chr19	47616189	47616608	1.06	4.78E-01	ZC3H4	610			
chr17	27070200	27070626	0.78	4.79E-01	TRAF4	-610			
chr20	5778503	5778996	1.13	4.79E-01	RP5-1022P6.2	-187078	CHGB	-113224	
chr14	103851492	103851820	0.95	4.79E-01	MARK3	-45			
chr19	49837035	49837475	1.29	4.79E-01	CD37	-1422			
chr1	16563588	16563861	1.06	4.79E-01	C1orf89	-66			
chr2	170430316	170430573	1.34	4.79E-01	FASTKD1	-21			
chr4	152682000	152682381	1.32	4.79E-01	PET112L	-45			
chr8	37707248	37707634	1.20	4.79E-01	BRF2	-30			
chr16	87735294	87735643	1.16	4.79E-01	KLHDC4	64073	JPH3	98970	
chr10	38265250	38265828	1.18	4.79E-01	ZNF25	-86			
chr17	3866864	3867108	0.80	4.79E-01	ATP2A3	750			
chr19	42637340	42637701	1.28	4.79E-01	POU2F2	-891			
chr7	124996065	124996312	1.27	4.79E-01	POT1	-426152			
chr14	74229075	74229444	1.32	4.79E-01	PNMA1	-48132	C14orf43	24636	
chr8	38268165	38268450	0.89	4.79E-01	LETM2	24288	FGFR1	58044	
chr19	18155851	18156129	1.12	4.79E-01	IL12RB1	41752	KCNN1	93879	
chr16	48278059	48278335	0.84	4.80E-01	LONP2	-14			
chr2	198317766	198318100	1.21	4.80E-01	COQ10B	-298			
chr5	132299151	132299439	1.10	4.81E-01	AFF4	59			
chr7	91509844	91510201	0.86	4.81E-01	MTERF	-7			
chr9	95055959	95056334	1.26	4.81E-01	IARS	-109			
chr1	154192967	154193403	1.22	4.81E-01	UBAP2L	-140	C1orf43	88	
chr9	102028898	102029233	1.38	4.81E-01	NR4A3	-559943	SEC61B	44496	
chr12	26626272	26626616	1.24	4.81E-01	SSPN	277938	ITPR2	359687	
chr12	109125273	109125604	1.01	4.82E-01	CORO1C	-144			
chr19	5978189	5978510	1.22	4.82E-01	RANBP3	-30			
chr9	135259779	135260084	1.43	4.82E-01	SETX	-29560	TTF1	22289	
chr19	3762701	3763206	1.23	4.82E-01	APBA3	-1281	MRPL54	289	
chr14	94444580	94444874	0.93	4.82E-01	OTUB2	-47997	ASB2	-20960	
chr7	129650757	129651006	1.19	4.82E-01	UBE2H	-58093	ZC3HC1	40351	
chr19	6753273	6753713	1.28	4.82E-01	TRIP10	13786	SH2D3A	14030	
chr19	12662349	12662865	0.87	4.82E-01	ZNF709	-66975	ZNF490	59016	
chr3	50653344	50653654	0.84	4.82E-01	CISH	-4237	MAPKAPK3	-1102	
chr3	112280656	112281343	1.28	4.82E-01	ATG3	-515	SLC35A5	105	
chr5	70751294	70751544	1.21	4.82E-01	BDP1	-23			
chr11	64863440	64863755	1.28	4.82E-01	C11orf2	-85			
chr12	12939842	12940093	1.03	4.82E-01	DDX47	-26173	APOLD1	61117	

chr20	61585619	61586041	0.90	4.82E-01	SLC17A9	1831	BHLHE23	52557
chr4	39136939	39137214	1.13	4.82E-01	WDR19	-46947	KLHL5	73017
chr22	40446285	40446606	0.92	4.83E-01	TNRC6B	-127483	GRAP2	149360
chr16	66557913	66558182	0.85	4.83E-01	TK2	26267	BEAN	96808
chr2	20850761	20851137	1.17	4.83E-01	HS1BP3	-85		
chr1	78470404	78470673	1.30	4.83E-01	DNAJB4	-97		
chr10	75639436	75639746	0.68	4.83E-01	PLAU	-31271	CAMK2G	-5248
chrX	33201471	33201802	1.28	4.83E-01	DMD	28036		
chr13	20357057	20357301	1.22	4.84E-01	PSPC1	-96		
chr13	31309543	31309797	1.26	4.84E-01	ALOX5AP	1		
chr3	197676661	197677092	0.76	4.84E-01	RPL35A	-175		
chr15	52774111	52774427	1.34	4.84E-01	MYO5C	-186299	MYO5A	46978
chr17	42293629	42293918	1.22	4.84E-01	ATXN7L3	-18245	UBTF	1890
chr7	97831730	97832156	1.20	4.84E-01	BHLHA15	-9625	LMTK2	95746
chr2	20101569	20101876	1.19	4.84E-01	TTC32	21		
chr3	156391858	156392270	1.36	4.84E-01	TIPARP	-315		
chr7	104992658	104992973	1.21	4.84E-01	SRPK2	36525	MLL5	338179
chr11	65382636	65383381	1.24	4.85E-01	MAP3K11	-1289	PCNXL3	-774
chr10	105128363	105128614	1.11	4.85E-01	TAF5	765		
chr19	5690062	5690378	1.29	4.85E-01	RPL36	-52		
chr20	56055849	56056177	1.41	4.85E-01	HMGB1L1	8070	RBM38	89550
chr7	151047114	151047558	1.13	4.85E-01	NUB1	8478	RHEB	169674
chr11	102301190	102301598	1.39	4.85E-01	TMEM123	22381	BIRC2	83428
chr9	140317522	140317843	1.23	4.85E-01	NOXA1	-164	EXD3	31
chr20	4795319	4795936	1.33	4.85E-01	RASSF2	141		
chr15	59430413	59430723	0.95	4.85E-01	LDHAL6B	-68474	CCNB2	33248
chr7	6676785	6677036	0.56	4.85E-01	ZNF853	21384	ZNF12	69655
chr16	87812639	87813028	1.28	4.85E-01	KLHDC4	-13292	SLC7A5	90266
chr2	65173420	65173665	1.15	4.85E-01	SERTAD2	-292497	SLC1A4	-42913
chr19	12807316	12807626	1.22	4.85E-01	FBXW9	-16		
chr14	102605911	102606339	1.19	4.86E-01	HSP90AA1	-39		
chr5	172571286	172571750	0.93	4.86E-01	BNIP1	73		
chr14	77587545	77588112	1.32	4.86E-01	C14orf4	-92795	ZDHHC22	20305
chr6	26195565	26195918	0.82	4.86E-01	HIST1H2BF	-4045	HIST1H3D	3722
chr18	71814940	71815214	1.25	4.86E-01	C18orf55	-669	FBXO15	23
chr7	108056278	108056591	0.91	4.86E-01	LAMB4	-285634	NRCAM	40391
chr17	46985408	46985905	0.93	4.86E-01	UBE2Z	-110		
chr3	141206635	141206909	1.29	4.86E-01	RASA2	846		

chr10	74870103	74870360	0.78	4.87E-01	NUDT13	22		
chr19	40324640	40325056	1.09	4.87E-01	DYRK1B	-7		
chr11	47600133	47600773	0.85	4.87E-01	NDUFS3	-109	KBTBD4	114
chr16	1877073	1877330	1.27	4.87E-01	FAHD1	-23	HAGH	-7
chr12	9913395	9913753	1.32	4.87E-01	CD69	-77		
chr10	93557994	93558264	1.11	4.87E-01	TNKS2	-22		
chr6	30875695	30875951	0.86	4.87E-01	GTF2H4	-154		
chr12	510426	511060	0.74	4.87E-01	B4GALNT3	-58800	KDM5A	-12123
chr12	123717823	123718288	1.08	4.88E-01	C12orf65	215		
chr18	48724333	48724837	0.81	4.88E-01	MEX3C	-895		
chr19	36119780	36120055	1.15	4.88E-01	RBM42	-62		
chr2	187350680	187351033	1.24	4.88E-01	ZC3H15	-28		
chr5	56469213	56469576	1.23	4.88E-01	MIER3	-221441	GPBP1	-40506
chr6	30070895	30071193	0.85	4.88E-01	RNF39	-27416	TRIM31	9823
chr6	30421414	30421658	0.87	4.88E-01	HLA-E	-35735	TRIM39	126528
chr1	47184684	47185119	1.19	4.88E-01	CYP4B1	-79768	C1orf223	47402
chr20	30467857	30468171	1.24	4.88E-01	TTLL9	9509	PDRG1	71869
chr14	45722267	45722681	0.83	4.89E-01	C14orf106	131		
chr10	76995714	76995958	0.70	4.89E-01	UNQ766	-145	COMTD1	-66
chr2	58468335	58468648	1.17	4.89E-01	FANCL	23		
chr7	142985191	142985591	1.15	4.89E-01	CASP2	-11		
chr9	139839011	139839416	1.05	4.89E-01	C8G	-484	FBXW5	-41
chr11	66311215	66311620	0.80	4.89E-01	ACTN3	-2973	ZDHHC24	2253
chr12	53886869	53887132	1.13	4.90E-01	MAP3K12	6270	PCBP2	41115
chr12	49582691	49583074	0.75	4.90E-01	TUBA1C	-75982	TUBA1B	-57579
chr12	66563557	66563979	0.89	4.90E-01	TMBIM4	39		
chr3	141456825	141457191	1.18	4.90E-01	RNF7	-43		
chr22	23623962	23624272	1.14	4.90E-01	BCR	101565	IGLL1	298378
chr7	35840560	35840922	0.78	4.90E-01	SEPT7	114		
chr16	11403718	11404048	0.70	4.91E-01	C16orf75	-35428	PRM1	-28691
chr17	74068573	74068819	1.04	4.91E-01	GALR2	-2196	SRP68	-89
chr20	42939564	42939967	1.17	4.91E-01	FITM2	123		
chr1	17053759	17054090	1.15	4.91E-01	NBPF1	-113943	MSTP9	37050
chr6	141804844	141805129	1.42	4.91E-01	AK097143	546567		
chr2	64067206	64067690	0.89	4.91E-01	UGP2	-1566		
chr6	143234678	143235063	1.03	4.91E-01	HIVEP2	31467	GPR126	611815
chr2	60593315	60593735	1.09	4.92E-01	BCL11A	187108		
chr21	30396870	30397159	1.24	4.92E-01	USP16	77		

chr6	33359052	33359671	0.77	4.92E-01	KIFC1	49		
chr14	69281313	69281689	1.40	4.92E-01	ZFP36L1	-21716	ACTN1	164582
chrX	9309037	9309297	1.64	4.92E-01	FAM9B	-308051	TBL1X	-124034
chr5	78053956	78054253	1.16	4.92E-01	LHFPL2	-109457	ARSB	228252
chr1	170500948	170501258	1.00	4.93E-01	GORAB	-160		
chr1	27524479	27524726	0.88	4.93E-01	SLC9A1	-43152	WDTC1	-36404
chr12	57977138	57977388	1.28	4.93E-01	PIP4K2C	-7679	KIF5A	33416
chr3	42814633	42814921	0.78	4.93E-01	HHATL	-70458	HIGD1A	31157
chr6	41559736	41560018	0.82	4.93E-01	MDFI	-46318	FOXP4	45713
chr11	66383815	66384320	1.19	4.93E-01	RBM14	15		
chr7	30634184	30634559	0.78	4.93E-01	GARS	191		
chr19	42381023	42381384	1.17	4.93E-01	CD79A	14		
chr17	21183362	21183672	0.88	4.93E-01	MAP2K3	-4451		
chr6	30710462	30710968	1.27	4.93E-01	FLOT1	-262		
chr12	6961415	6961860	1.16	4.93E-01	CDCA3	-1182	USP5	353
chr1	32715494	32715987	0.78	4.94E-01	LCK	-23971	EIF3I	27782
chr17	33905499	33905817	0.90	4.94E-01	PEX12	-2		
chr1	38061392	38061685	1.26	4.95E-01	GNL2	47		
chr7	76961252	76961514	0.85	4.95E-01	PTPN12	-205390	FGL2	-132233
chr11	14541846	14542180	0.83	4.95E-01	PSMA1	-22		
chr20	17517521	17517902	1.25	4.96E-01	DSTN	-32887	BFSP1	-5699
chr2	65659740	65660050	1.10	4.96E-01	SPRED2	-239		
chr11	118777699	118778171	1.22	4.97E-01	BCL9L	3678	CXCR5	23394
chr13	50194383	50194634	0.85	4.97E-01	RCBTB1	-34790	ARL11	-8115
chr1	38157937	38158214	1.13	4.97E-01	CDCA8	-83		
chr11	74303463	74303802	1.34	4.97E-01	POLD3	4		
chr19	17716953	17717263	1.09	4.97E-01	GLT25D1	50597	UNC13A	82293
chr8	41909778	41910268	0.90	4.98E-01	MYST3	-518		
chr16	88717707	88718150	1.22	4.98E-01	CYBA	-472		
chr15	57401252	57401531	0.84	4.98E-01	CGNL1	-267313	TCF12	190559
chr16	71929127	71929508	0.89	4.98E-01	KIAA0174	-128		
chr13	53024732	53025033	0.92	4.98E-01	CKAP2	-4612	VPS36	-120
chr3	48507317	48507773	0.86	4.98E-01	TREX1	316		
chr3	184135664	184135940	1.11	4.98E-01	EPHB3	-143785	CHRD	37941
chr11	65625482	65625888	1.13	4.99E-01	MUS81	-2187	CFL1	416
chr16	11422426	11422736	1.20	4.99E-01	PRM1	-47389	C16orf75	-16730
chr22	35773011	35773324	0.81	5.00E-01	HMOX1	-3919		
chr2	96811049	96811593	0.88	5.00E-01	DUSP2	-142		

chr3	15643297	15643701	1.17	5.00E-01	HACL1	-369	BTD	244
chr8	64080848	64081275	0.73	5.00E-01	YTHDF3	-59		
chr16	67880512	67880822	1.17	5.00E-01	NUTF2	-152	CENPT	694
chr19	30101506	30101828	1.22	5.00E-01	PLEKHF1	-54660	POP4	4497
chr3	160167505	160167883	0.88	5.01E-01	TRIM59	-68		
chr17	80455039	80455287	1.14	5.01E-01	FOXK2	-22431	NARF	38623
chr13	37393186	37393488	1.26	5.02E-01	RFXAP	-2		
chr3	13133656	13133947	1.14	5.02E-01	IQSEC1	-19185	NUP210	328007
chr5	55884582	55885094	1.12	5.02E-01	IL6ST	-594075	MAP3K1	-226062
chr17	27054995	27055617	1.28	5.02E-01	TLCD1	-2076	NEK8	-526
chr1	45965617	45966002	1.23	5.03E-01	MMACHC	-46		
chr15	73075965	73076283	1.18	5.03E-01	ADPGK	2		
chr16	2518818	2519138	0.73	5.03E-01	NTN3	-2522		
chr20	46130423	46130786	1.17	5.03E-01	NCOA3	-52		
chr6	27832857	27833287	0.91	5.03E-01	HIST1H2AL	-35		
chr9	132597391	132597703	1.14	5.03E-01	USP20	-149		
chr5	43066870	43067238	0.92	5.03E-01	ZNF131	-54588	C5orf39	-26607
chr11	9635525	9636077	0.84	5.03E-01	SWAP70	-49827	WEE1	40573
chr11	66445259	66445567	1.27	5.03E-01	RBM4B	-138		
chr11	27528169	27528460	0.82	5.04E-01	LIN7C	11		
chr11	72449892	72450189	1.26	5.04E-01	PDE2A	-64547	ARAP1	13393
chr2	85166702	85167034	1.36	5.04E-01	KCMF1	-31363	TMSB10	34105
chr2	170440699	170441009	1.24	5.04E-01	PPIG	4		
chr15	34516998	34517354	1.16	5.04E-01	TMEM85	-69		
chr3	107941189	107941595	1.25	5.04E-01	IFT57	25		
chr12	92987577	92987976	0.91	5.04E-01	BTG1	-448104	PLEKHG7	-142488
chr11	68039476	68039757	1.11	5.04E-01	C11orf24	-148		
chr12	117175905	117176306	1.24	5.04E-01	C12orf49	-263	RNFT2	10
chr9	37324572	37325103	1.00	5.04E-01	GRHPR	-97869	ZCCHC7	204369
chr13	77566066	77566387	1.19	5.05E-01	CLN5	168		
chr2	216973703	216974049	1.19	5.05E-01	XRCC5	-144		
chr6	28696992	28697236	1.33	5.05E-01	SCAND3	-142002	TRIM27	194654
chr11	64645897	64646456	1.22	5.05E-01	EHD1	14		
chr9	37800626	37800904	1.14	5.06E-01	DCAF10	-25		
chr12	65012809	65013132	1.35	5.06E-01	RASSF3	8678	GNS	140255
chr7	24917461	24917841	0.78	5.06E-01	DFNA5	-120568	OSBPL3	102109
chr19	56015360	56015678	0.83	5.06E-01	SSC5D	15649	SBK2	32142
chr1	53704108	53704418	1.14	5.06E-01	MAGOH	-56		

chr6	27094852	27095150	0.86	5.06E-01	ZNF322A	-435038	HIST1H2BJ	5574
chr1	12212150	12212449	1.21	5.06E-01	TNFRSF1B	-14760	TNFRSF8	88866
chr2	233415210	233415520	1.21	5.06E-01	TIGD1	-139	EIF4E2	8
chr7	142491102	142491429	0.86	5.07E-01	TCRBC2	-3101		
chr15	78832611	78833070	1.21	5.07E-01	PSMA4	94		
chr13	33112863	33113327	1.30	5.07E-01	N4BP2L2	-159		
chr6	43138572	43139003	0.83	5.07E-01	SRF	-132		
chr9	35619371	35620189	0.77	5.07E-01	CD72	-1356		
chr19	17850116	17850356	1.13	5.07E-01	B3GNT3	-55683	MAP1S	19933
chr9	117672893	117673282	1.43	5.07E-01	TNFSF15	-104680	TNFSF8	19682
chr16	31044536	31044862	1.35	5.07E-01	STX4	-204		
chr6	106773562	106773865	0.87	5.07E-01	ATG5	-19		
chr15	90418256	90418540	1.31	5.07E-01	ANPEP	-60326	C15orf38	37824
chr17	80416311	80416621	0.75	5.08E-01	NARF	-74		
chr11	18343446	18343885	1.27	5.08E-01	GTF2H1	-150	HPS5	55
chr4	183839063	183839336	1.18	5.08E-01	DCTD	-570		
chr1	203309240	203309516	1.32	5.08E-01	FMOD	10911	BTG2	34714
chr5	87971607	87971873	1.06	5.08E-01	TMEM161B	-407075	MEF2C	207562
chr19	50269491	50269810	1.19	5.08E-01	TSKS	-3136	AP2A1	-529
chr5	180634602	180634912	1.29	5.08E-01	TRIM7	-2580		
chr11	65313568	65314117	0.88	5.08E-01	LTBP3	11856	SCYL1	21295
chr5	125950956	125951393	1.23	5.08E-01	LMNB1	-161658	PHAX	14542
chr19	5803983	5804375	1.01	5.08E-01	NRTN	-19639	DUS3L	-12930
chr10	63659835	63660222	0.89	5.08E-01	ARID5B	-1414		
chr5	179050486	179051056	1.14	5.09E-01	HNRNPH1	-49		
chr7	65447117	65447370	0.88	5.09E-01	GUSB	2		
chr22	41252566	41253160	0.83	5.09E-01	XPNPEP3	-231	ST13	-176
chr2	198318482	198318792	0.99	5.10E-01	COQ10B	406		
chrX	13752424	13752959	1.09	5.10E-01	OFD1	-140	TRAPPC2	50
chr1	1565666	1566019	1.11	5.10E-01	MMP23B	-1717		
chr7	73153188	73153466	1.20	5.10E-01	ABHD11	-137		
chr12	46121317	46121627	1.18	5.11E-01	ARID2	-2148		
chr6	30881894	30882203	0.71	5.11E-01	VARS2	461		
chr12	6741460	6741770	0.79	5.11E-01	LPAR5	-800		
chr12	51762698	51763044	1.22	5.11E-01	CELA1	-22408	GALNT6	22329
chr2	6364417	6364727	0.85	5.11E-01	SOX11	531773	CMPK2	641364
chr12	49504387	49504930	0.72	5.11E-01	LMBR1L	21		
chr3	58173512	58173748	0.72	5.11E-01	DNASE1L3	26768	FLNB	179503

chr18	21032980	21033418	0.83	5.11E-01	RIOK3	412		
chr2	70056538	70056895	1.09	5.12E-01	GMCL1	-101		
chr1	46306467	46306745	1.38	5.12E-01	MAST2	37321	PIK3R3	291774
chr1	235098329	235098635	1.33	5.12E-01	IRF2BP2	-353211	TOMM20	193774
chr6	2891978	2892355	0.86	5.12E-01	SERPINB1	-50086	SERPINB9	11378
chr8	54811363	54811782	0.90	5.12E-01	RGS20	47205	TCEA1	123435
chr1	226374312	226374556	1.35	5.12E-01	ACBD3	-11		
chr19	38864970	38865863	1.31	5.13E-01	PSMD8	227		
chr6	73332816	73333100	0.84	5.13E-01	KCNQ5	1387	KHDC1	639949
chr6	313082	313437	0.85	5.13E-01	IRF4	-78492	DUSP22	21159
chr12	113436647	113437159	1.07	5.13E-01	DTX1	-58759	OAS2	20629
chrX	48595483	48595829	0.87	5.13E-01	GATA1	-49326	SUV39H1	40525
chr16	67850457	67850818	0.82	5.13E-01	THAP11	-25575	TSNAXIP1	9628
chrX	129402723	129403098	0.92	5.14E-01	ZNF280C	-38		
chr14	102414316	102414778	1.30	5.15E-01	DYNC1H1	-16318	PPP2R5C	186412
chr19	18682363	18682730	1.22	5.15E-01	UBA52	-67		
chr4	1283402	1283665	1.13	5.15E-01	MAEA	-138		
chr16	70415138	70415582	0.77	5.15E-01	ST3GAL2	57631	DDX19B	82288
chr6	167001641	167001951	1.09	5.15E-01	BRP44L	-205310	RPS6KA2	273975
chr8	61829042	61829474	0.84	5.15E-01	RLBP1L1	-371267	CHD7	237919
chr15	31556496	31556797	1.17	5.15E-01	TRPM1	-162723	KLF13	-62436
chr13	31191646	31192206	0.69	5.16E-01	USPL1	96		
chr18	32754235	32754481	1.37	5.16E-01	ZNF397	-66640	MAPRE2	196150
chr11	43333327	43333705	1.35	5.16E-01	API5	11		
chr2	176046365	176046704	1.00	5.16E-01	ATP5G3	-144		
chr14	102173651	102173968	1.18	5.17E-01	PPP2R5C	-54325	DIO3	146122
chr9	140135477	140135894	1.10	5.17E-01	TUBB2C	-25		
chr17	8103452	8103743	1.16	5.17E-01	C17orf59	-10034	AURKB	10285
chr1	154155593	154156005	0.97	5.17E-01	NUP210L	-28207	TPM3	8810
chr16	70557302	70557656	0.77	5.17E-01	SF3B3	-212	COG4	-22
chr9	95491058	95491384	0.75	5.17E-01	IPPK	-58674	BICD2	35862
chr14	20811347	20811939	1.29	5.17E-01	PARP2	-130		
chr11	62448240	62448563	0.77	5.17E-01	UBXN1	-1875		
chr11	6624697	6625048	0.94	5.17E-01	ILK	-91	RRP8	-62
chr12	1692695	1692955	1.32	5.17E-01	FBXL14	10506	ERC1	592421
chr15	93449953	93450207	1.33	5.17E-01	CHD2	6529	RGMA	182353
chr19	42069778	42070115	1.24	5.17E-01	ATP5SL	-124137	CEACAM21	-12584
chr7	102987914	102988197	0.79	5.17E-01	DNAJC2	-2736	PSMC2	-33

chr15	35280408	35280673	1.13	5.17E-01	ZNF770	-87		
chr5	118676246	118676624	1.14	5.18E-01	TNFAIP8	-15161		
chr2	85152939	85153254	1.28	5.18E-01	KCMF1	-45134	TMSB10	20334
chr21	47878512	47878856	0.68	5.18E-01	DIP2A	-178		
chr12	111527423	111527665	0.69	5.18E-01	SH2B3	-316208	CUX2	55715
chr1	22778260	22778530	1.22	5.18E-01	ZBTB40	51		
chr19	37808588	37809096	1.23	5.18E-01	HKR1	-16738	ZNF383	91476
chr1	24969295	24969907	1.13	5.19E-01	SRRM1	7		
chr16	89441487	89441740	1.24	5.19E-01	ANKRD11	115355	ZNF778	157503
chr16	28942005	28942356	0.79	5.19E-01	CD19	-1079		
chr17	8042691	8043060	1.29	5.19E-01	HES7	-15474	PER1	12877
chr3	182879358	182879705	1.33	5.19E-01	MCCC1	-62167	LAMP3	1135
chr10	73975677	73976223	0.79	5.19E-01	ASCC1	-256	C10orf104	144
chrX	55744001	55744311	1.31	5.20E-01	RRAGB	46		
chr17	6543979	6544822	0.78	5.20E-01	TXNDC17	179		
chr11	85376075	85376332	1.33	5.20E-01	CREBZF	-22		
chr19	2050117	2050446	1.18	5.20E-01	MKNK2	961		
chr19	4246780	4247447	0.81	5.20E-01	SHD	-31484	EBI3	17574
chr2	70141808	70142132	1.01	5.21E-01	MXD1	-233		
chr1	26615585	26615958	0.80	5.21E-01	SH3BGRL3	9559	UBXN11	17423
chr2	198017168	198017505	0.91	5.21E-01	PGAP1	-225883	ANKRD44	45425
chr3	169755547	169755857	1.19	5.21E-01	GPR160	-33		
chr9	32573020	32573330	1.22	5.21E-01	NDUFB6	7		
chr9	139294557	139294878	1.10	5.21E-01	SNAPC4	-1829		
chr2	8621600	8621997	1.12	5.21E-01	ID2	-200185		
chr5	139049850	139050229	1.16	5.21E-01	PSD2	-125366	CXXC5	21739
chr15	74907901	74908163	1.19	5.21E-01	CLK3	697		
chr15	77835745	77836121	1.13	5.21E-01	LINGO1	88776	HMG20A	122690
chr4	130014434	130014913	1.11	5.21E-01	SCLT1	90		
chr5	142784756	142785177	0.90	5.21E-01	NR3C1	-922		
chr3	39329887	39330249	1.23	5.21E-01	CCR8	-41129	CX3CR1	-8541
chr5	176540952	176541262	0.88	5.21E-01	NSD1	-19726	FGFR4	27186
chr13	51490905	51491244	1.19	5.21E-01	RNASEH2B	7183	GUCY1B2	149268
chr15	41523106	41523572	0.83	5.22E-01	EXD1	-444	CHP	-98
chr10	32635978	32636222	1.31	5.22E-01	EPC1	13		
chr12	113645911	113646238	1.14	5.22E-01	DDX54	-22791	TPCN1	-13185
chr6	27113675	27114013	1.38	5.22E-01	HIST1H2AH	-1064	HIST1H2BK	775
chr11	10830316	10830863	0.84	5.22E-01	EIF4G2	-8		

chr7	44516989	44517265	0.84	5.22E-01	CAMK2B	-151897	NPC1L1	63787
chr13	25861478	25861788	1.10	5.23E-01	MTMR6	71		
chr17	62009570	62009973	0.92	5.23E-01	CD79B	-68		
chr7	130741966	130742343	1.14	5.23E-01	KLF14	-323295	MKLN1	-270440
chr6	33239656	33239938	0.82	5.24E-01	VPS52	-135	RPS18	-55
chr3	195633988	195634267	1.02	5.24E-01	TNK2	-11696	SDHALP1	83022
chr13	98085377	98085711	1.28	5.24E-01	RAP2A	-931		
chr2	196996178	196996483	0.93	5.24E-01	DNAH7	-62795	STK17B	40005
chr7	128065854	128066164	0.90	5.24E-01	C7orf68	-29875	IMPDH1	-15973
chr12	66524445	66524896	0.81	5.24E-01	LLPH	-138		
chr1	27936120	27936449	0.76	5.24E-01	AHDC1	-6142	FGR	25442
chr12	57881496	57882022	1.13	5.24E-01	MARS	39		
chr17	38296458	38296759	1.16	5.24E-01	CASC3	102		
chr18	48556415	48556734	1.08	5.24E-01	SMAD4	-8		
chr14	91833565	91833905	1.06	5.24E-01	GPR68	-113511	CCDC88C	50398
chr3	184080672	184081564	1.27	5.24E-01	CLCN2	-1727	POLR2H	457
chr21	30494108	30494389	1.51	5.24E-01	BACH1	-176971	C21orf7	41376
chr17	1933923	1934359	0.90	5.24E-01	DPH1	710		
chr3	184521099	184521409	1.10	5.24E-01	VPS8	-8677	EPHB3	241667
chr19	797785	798065	0.98	5.24E-01	PTBP1	533		
chr2	68941978	68942462	0.94	5.24E-01	ARHGAP25	-19748	PROKR1	69426
chr9	117349775	117350172	1.21	5.24E-01	ATP6V1G1	-20		
chr14	74237225	74237676	1.39	5.25E-01	PNMA1	-56323	C14orf43	16445
chr3	51703319	51703589	0.83	5.25E-01	TEX264	-1768		
chr11	60253427	60253792	1.39	5.25E-01	MS4A12	-6641	MS4A1	30328
chr17	47785284	47785800	0.86	5.25E-01	SLC35B1	-260		
chr17	79899454	79899822	1.19	5.25E-01	PYCR1	-4670		
chr10	101491789	101492098	1.11	5.26E-01	CUTC	-14	COX15	479
chr15	83518402	83518799	1.07	5.26E-01	WHAMM	40628	HOMER2	102872
chr19	16557843	16558126	0.74	5.26E-01	EPS15L1	24777	KLF2	122334
chr7	32930117	32930399	0.67	5.26E-01	KBTBD2	1210	AVL9	395082
chr3	142314915	142315328	1.15	5.27E-01	PLS1	-27144	ATR	-17454
chr1	160644247	160644555	0.74	5.27E-01	SLAMF1	-27320	CD48	37184
chr9	84742969	84743367	1.20	5.27E-01	FLJ46321	139481	RASEF	934875
chr1	29208534	29208890	0.69	5.27E-01	EPB41	-4916		
chr1	166879676	166879915	1.44	5.27E-01	TADA1	-34142	ILDR2	64765
chr19	46195070	46195434	1.15	5.27E-01	QPCTL	-489	SNRPD2	191
chr5	147763233	147763573	1.30	5.28E-01	FBXO38	-143		

chr11	65210922	65211220	1.14	5.28E-01	SCYL1	-81477	FRMD8	57030
chr1	160458125	160458506	0.86	5.28E-01	SLAMF6	34726	VANGL2	87949
chr21	40752092	40752366	1.20	5.28E-01	WRB	16		
chr22	37258399	37258709	1.08	5.28E-01	CSF2RB	-51121	NCF4	1524
chr3	149470190	149470549	1.37	5.29E-01	COMMD2	-92		
chr3	196669117	196669683	1.19	5.29E-01	NCBP2	64		
chr12	100594389	100594731	1.18	5.30E-01	ACTR6	-14		
chr20	52443617	52443877	1.13	5.30E-01	ZNF217	-244040	BCAS1	243557
chr21	44527657	44527936	1.29	5.30E-01	U2AF1	-109		
chr14	74353109	74353611	0.86	5.30E-01	ZNF410	-226		
chr3	23987669	23987983	1.04	5.30E-01	NR1D2	1075	THRB	548440
chr6	137752089	137752453	1.29	5.30E-01	IFNGR1	-211704	OLIG3	63260
chr6	27440697	27441081	0.88	5.31E-01	ZNF184	8		
chr22	19419650	19420162	1.20	5.31E-01	HIRA	-687	MRPL40	-130
chr9	125667432	125667967	1.13	5.31E-01	RC3H2	-138		
chr21	27107189	27107452	1.26	5.31E-01	GABPA	-8	ATP5J	644
chr9	110046180	110046593	0.99	5.33E-01	RAD23B	843		
chr2	202096970	202097274	1.12	5.33E-01	CASP8	-25632	CASP10	49501
chr6	34725025	34725410	0.81	5.33E-01	SNRPC	-94		
chr19	1040991	1041241	1.21	5.34E-01	HMHA1	-26058	ABCA7	1014
chr3	186714196	186714481	1.35	5.34E-01	ST6GAL1	-25326	ADIPOQ	153876
chr6	28863927	28864255	0.92	5.34E-01	SCAND3	-308979	TRIM27	27677
chr21	45759174	45759527	1.19	5.34E-01	TRPM2	-14133	PFKL	39426
chr10	112680314	112680704	0.79	5.34E-01	SHOC2	1208	RPL13AP6	16504
chr17	8059956	8060356	1.17	5.34E-01	PER1	-4403		
chr3	150320907	150321202	1.29	5.34E-01	SELT	-11		
chr17	2148111	2148347	1.24	5.34E-01	SMG6	58840	HIC1	188625
chr12	111051755	111051997	1.01	5.34E-01	TCTN1	-4		
chr14	50052950	50053300	1.34	5.34E-01	RPS29	-31		
chr16	30669160	30669470	1.09	5.34E-01	ZNF689	-47633	FBRS	-6463
chr14	73493794	73494102	1.18	5.34E-01	ZFYVE1	-109		
chr20	4701323	4701605	1.12	5.34E-01	PRND	-1092		
chr20	31989234	31989520	1.22	5.34E-01	CDK5RAP1	-40		
chr3	152016458	152016768	0.94	5.34E-01	MBNL1	-581		
chr7	96339023	96339348	0.91	5.34E-01	SHFM1	17		
chr11	76337845	76338311	1.12	5.34E-01	LRRC32	42966	C11orf30	182009
chr19	49127422	49127667	1.11	5.34E-01	SPHK2	4997	DBP	13094
chr20	43274983	43275260	1.19	5.34E-01	ADA	5254	PKIG	114686

chr20	57579814	57580129	1.24	5.34E-01	CTSZ	2337	TH1L	23661
chr3	183371015	183371332	0.78	5.34E-01	YEATS2	-44432	KLHL24	17763
chr12	861411	861768	1.26	5.35E-01	WNK1	-635		
chr4	186064305	186064591	0.74	5.35E-01	SLC25A4	50		
chr5	149792332	149792724	0.90	5.35E-01	CD74	-196		
chr11	2721236	2721512	1.05	5.35E-01	CDKN1C	185621	KCNQ1	255153
chr22	23377959	23378384	0.81	5.35E-01	GNAZ	-34497	IGL@	148212
chr2	65615167	65615455	1.17	5.35E-01	SPRED2	44345	ACTR2	160482
chr17	16283894	16284572	0.77	5.36E-01	UBB	-134		
chr1	9687126	9687412	0.80	5.36E-01	PIK3CD	-24521	TMEM201	38292
chr3	169899243	169899638	1.19	5.36E-01	PHC3	96		
chr2	65159630	65159945	0.73	5.36E-01	SERTAD2	-278742	SLC1A4	-56668
chrX	130926393	130926651	1.39	5.36E-01	MST4	-230723	OR13H1	248474
chr4	153587049	153587489	0.86	5.37E-01	FBXW7	-131084	TMEM154	13922
chr20	30310859	30311415	0.74	5.37E-01	BCL2L1	-481		
chr16	2732216	2732622	1.28	5.37E-01	KCTD5	-76		
chr17	49230715	49231044	1.23	5.37E-01	NME1-NME2	-40		
chr22	24142529	24142839	1.17	5.37E-01	SMARCB1	13534	DERL3	38515
chr10	80897599	80897982	0.63	5.37E-01	PPIF	-209429	ZMIZ1	68999
chr1	203830503	203830899	1.15	5.38E-01	SNRPE	-39		
chr16	89008308	89008688	1.16	5.38E-01	LOC390748	-75484	CBFA2T3	34903
chr19	12833375	12833720	1.38	5.38E-01	TNPO2	-86		
chr4	100737774	100738106	1.21	5.38E-01	DAPP1	-41		
chr6	52284684	52285235	0.78	5.38E-01	EFHC1	-34		
chr14	73925444	73925688	1.22	5.38E-01	NUMB	-280		
chr14	20773955	20774284	0.86	5.39E-01	TTC5	33		
chr20	5100295	5100803	1.15	5.39E-01	PCNA	98		
chr1	109584564	109584815	0.75	5.39E-01	CLCC1	-78579	TAF13	33934
chr1	87527476	87527811	1.26	5.39E-01	LMO4	-266507	HS2ST1	147309
chr15	23032920	23033162	1.30	5.39E-01	NIPA2	1367	CYFIP1	140357
chr4	152020502	152020813	1.32	5.40E-01	RPS3A	-96		
chr7	86845951	86846282	0.90	5.40E-01	C7orf23	2914	DMTF1	64440
chr7	101950265	101950674	1.13	5.40E-01	PRKRIP1	-86334	SH2B2	22065
chr14	104029112	104029421	1.23	5.40E-01	BAG5	-653		
chr16	284589	284871	1.10	5.40E-01	ITFG3	-71		
chr16	18801609	18801893	1.21	5.40E-01	RPS15A	-95		
chr3	57541641	57541996	1.29	5.40E-01	PDE12	-162		
chr11	1051095	1051371	0.83	5.40E-01	MUC2	-23642	MUC6	-14527

chr17	1626425	1626650	1.35	5.40E-01	PRPF8	-38362	SERPINF2	-19592
chr12	69080558	69080925	1.23	5.41E-01	NUP107	11		
chr7	158497523	158497790	0.99	5.41E-01	NCAPG2	-137		
chr4	123844164	123844598	1.21	5.41E-01	NUDT6	-622	SPATA5	156
chr7	75157423	75157692	1.06	5.41E-01	PMS2L3	-105		
chr2	6482659	6482951	0.73	5.41E-01	CMPK2	523131	SOX11	650006
chr5	55965859	55966223	1.22	5.41E-01	IL6ST	-675278	MAP3K1	-144859
chr17	63133276	63133541	0.81	5.41E-01	RGS9	-140		
chr5	56469764	56470074	0.91	5.42E-01	MIER3	-221965	GPBP1	-39982
chr8	55014308	55014785	0.84	5.42E-01	LYPLA1	30		
chr3	141088994	141089338	1.32	5.42E-01	RASA2	-116760	ZBTB38	46111
chr17	6554820	6555202	0.99	5.42E-01	MED31	-57		
chr14	95982316	95982626	1.10	5.42E-01	C14orf49	-40298	GLRX5	-18852
chr19	17530411	17530857	1.17	5.42E-01	FAM125A	-278		
chr8	142012309	142012558	1.00	5.42E-01	PTK2	-1102		
chr16	68291150	68291521	0.91	5.43E-01	SLC7A6	-7087	PLA2G15	12089
chr12	54610740	54611094	0.80	5.43E-01	SMUG1	-28160	CBX5	42453
chr5	173172220	173172578	1.14	5.43E-01	CPEB4	-142932	BOD1	-128733
chr2	232259178	232259548	1.18	5.43E-01	B3GNT7	-972		
chrX	49047353	49047634	1.26	5.43E-01	PRICKLE3	-4718		
chr20	17949543	17949853	1.27	5.43E-01	SNX5	-544	C20orf72	-64
chr8	37594983	37595354	1.16	5.44E-01	PROSC	-24932	ERLIN2	1072
chr6	4156168	4156421	0.78	5.44E-01	CDYL	-620385	PECI	-20464
chr6	37071114	37071589	0.76	5.44E-01	PIM1	-66570	FGD2	97929
chr8	103250853	103251309	0.77	5.44E-01	RRM2B	265		
chr6	36561911	36562233	0.79	5.45E-01	SFRS3	-18		
chr17	16438927	16439275	1.28	5.45E-01	ZNF287	33419	C17orf45	96800
chr15	89631151	89631459	1.05	5.45E-01	ABHD2	-76		
chr3	193821287	193821571	1.20	5.45E-01	HES1	-32505	OPA1	510496
chr3	141030509	141030936	1.28	5.45E-01	ZBTB38	-12332	ACPL2	80041
chr2	157257145	157257415	1.35	5.45E-01	NR4A2	-67993	GPD2	-35620
chr12	53553107	53553476	0.83	5.45E-01	CSAD	21138	SOAT2	56018
chr7	26007726	26008091	1.09	5.45E-01	NPVF	-739804	NFE2L3	-183938
chr5	158202332	158202610	0.72	5.46E-01	CLINT1	-916303	EBF1	324317
chr22	21921773	21922073	1.23	5.46E-01	UBE2L3	-34		
chr9	130746143	130746514	0.85	5.46E-01	DPM2	-45566	NAIF1	83270
chr2	85645647	85645887	1.11	5.46E-01	SH2D6	-16151	CAPG	-8091
chr3	121379565	121379843	1.23	5.46E-01	HCLS1	87		

chr12	55378441	55378797	0.89	5.46E-01	NEUROD4	-35110	MUCL1	130320
chr3	170131945	170132239	1.51	5.46E-01	CLDN11	-4561		
chr9	35103018	35103373	0.85	5.46E-01	STOML2	-42		
chr1	42039800	42040077	0.85	5.46E-01	EDN2	-89595	HIVEP3	344557
chr14	50328810	50329059	0.87	5.46E-01	ARF6	-30801	SDCCAG1	-9396
chr22	32058119	32058355	1.09	5.46E-01	YWHAH	-282242	PISD	-31427
chr22	23226176	23226435	0.76	5.47E-01	IGL@	-3654		
chr2	74734226	74734672	1.30	5.48E-01	LBX2	-4006	PCGF1	372
chr11	128480806	128481265	0.90	5.48E-01	FLI1	-82777	ETS1	-23583
chr15	55940604	55940911	1.23	5.48E-01	PYGO1	-59708	PRTG	94419
chr17	27309256	27309627	0.93	5.48E-01	PHF12	-30934	SEZ6	23639
chr4	78077722	78078505	0.81	5.48E-01	CCNG2	-243		
chr12	125401698	125402039	1.30	5.48E-01	UBC	-2292		
chr19	46389660	46390314	0.88	5.48E-01	IRF2BP1	-611		
chr7	66385762	66386247	0.81	5.48E-01	C7orf42	-198		
chr16	81512590	81512997	1.33	5.48E-01	PLCG2	-300136	CMIP	34019
chr17	67411155	67411504	1.21	5.49E-01	MAP2K6	492		
chr4	89205817	89206130	1.10	5.49E-01	PPM1K	-86		
chr19	7985073	7985339	1.07	5.50E-01	SNAPC2	-23		
chr19	11266323	11266980	1.18	5.50E-01	SPC24	-168		
chr2	68585133	68585422	1.18	5.51E-01	CNRIP1	-38095	PLEK	-7044
chr6	10747651	10748191	0.84	5.51E-01	TMEM14B	-74		
chr6	52929526	52930041	0.83	5.51E-01	ICK	-3184	FBXO9	-12
chr1	713887	714210	1.17	5.51E-01	SAMD11	-147072	OR4F16	-92015
chr3	52035573	52035883	0.88	5.51E-01	RPL29	-5770	DUSP7	54733
chr7	148823374	148823684	1.25	5.51E-01	ZNF425	-151		
chr22	42353674	42353957	1.18	5.52E-01	SEPT3	-19115	CENPM	-10668
chr19	36485844	36486225	1.24	5.52E-01	SDHAF1	-66		
chr1	109633168	109633493	1.29	5.52E-01	TMEM167B	-72		
chr17	40086568	40086927	1.19	5.52E-01	TTC25	-140		
chr17	42402128	42402420	0.98	5.52E-01	SLC25A39	-57		
chr19	6530806	6531175	1.16	5.52E-01	TNFSF9	-19		
chr20	37101147	37101457	1.19	5.52E-01	RALGAPB	-184		
chr11	61648014	61648250	0.80	5.52E-01	FADS3	10874	FADS2	52419
chr12	1905178	1905601	1.18	5.52E-01	LRTM2	-24043	ADIPOR2	105143
chr3	195577769	195578080	1.23	5.52E-01	MUC4	-38777	TNK2	44507
chr7	47710317	47710730	0.76	5.52E-01	TNS3	-131325	C7orf69	-124365
chr10	102027286	102027607	1.16	5.53E-01	CWF19L1	-10		

chr7	134855385	134855730	0.82	5.53E-01	C7orf49	-26		
chr11	62606956	62607243	1.17	5.53E-01	WDR74	528		
chr20	19997773	19998064	1.34	5.53E-01	NAT5	-18		
chr20	31560066	31560366	1.21	5.53E-01	SPAG4L	32023	EFCAB8	113487
chr18	44626684	44626946	1.15	5.53E-01	TCEB3B	-64827	HDHD2	50056
chr15	72519093	72519403	0.90	5.54E-01	PKM2	-24243	PKM2	4436
chr21	27107569	27107948	1.22	5.54E-01	ATP5J	206	GABPA	430
chr6	25962912	25963165	0.90	5.54E-01	TRIM38	-32		
chr9	6703996	6704373	0.85	5.54E-01	GLDC	-58493	KDM4C	-53456
chr3	186490443	186490877	1.23	5.55E-01	EIF4A2	-10701	KNG1	55540
chr22	43539295	43539629	1.18	5.55E-01	MCAT	-59		
chr9	35079828	35080217	1.25	5.55E-01	FANCG	-10		
chr11	74959690	74960022	0.95	5.55E-01	SLCO2B1	97816	ARRB1	103017
chr19	54618626	54618870	1.22	5.55E-01	PRPF31	-42	TFPT	307
chr7	92053836	92054144	0.77	5.56E-01	GATAD1	-22775	ANKIB1	178442
chr9	124082423	124082713	1.19	5.56E-01	GSN	20489	STOM	49977
chr7	99680163	99680490	1.28	5.56E-01	ZNF3	-956		
chr20	34330111	34330510	1.19	5.57E-01	RBM39	-118		
chr7	45808538	45808783	0.76	5.57E-01	IGFBP1	-119298	ADCY1	194536
chr1	53168769	53169191	0.87	5.57E-01	C1orf163	-4942		
chr19	15490253	15490623	1.14	5.57E-01	AKAP8	165		
chr4	185305346	185305778	1.21	5.57E-01	ENPP6	-166448	IRF2	90164
chr17	28804223	28804629	1.31	5.57E-01	GOSR1	0		
chr1	225632598	225632908	0.89	5.57E-01	LBR	-16969	ENAH	208092
chr16	85480952	85481388	1.14	5.57E-01	KIAA0182	-165754	KIAA0513	419760
chr4	103940593	103941038	1.17	5.58E-01	NHEDC1	60		
chr1	202976215	202976569	1.22	5.58E-01	TMEM183B	-142		
chr7	20239052	20239314	0.84	5.58E-01	TMEM196	-426779	MACC1	17830
chr16	70464694	70465028	1.16	5.58E-01	ST3GAL2	8130	DDX19B	131789
chr7	25002835	25003206	0.83	5.59E-01	DFNA5	-205938	OSBPL3	16739
chr18	49814833	49815095	1.04	5.59E-01	DCC	-51607		
chr3	31573226	31573673	0.90	5.60E-01	STT3B	-1041		
chr5	169010495	169011024	1.11	5.61E-01	CCDC99	122		
chr5	157621587	157621919	1.00	5.61E-01	CLINT1	-335585	EBF1	905035
chr17	37617483	37617804	1.19	5.61E-01	CRKRS	-95		
chr5	126083853	126084102	0.78	5.61E-01	LMNB1	-28855	PHAX	147345
chr19	41304016	41304447	1.24	5.61E-01	EGLN2	-1949		
chr6	31695344	31695708	0.85	5.62E-01	DDAH2	2513	C6orf25	4365

chr16	81870243	81870636	1.24	5.62E-01	PLCG2	57510	SDR42E1	174653
chr2	89039582	89039858	1.10	5.62E-01	O1/O11 and JK2	-850842	RPIA	48544
chr1	24307273	24307569	1.17	5.62E-01	SFRS13A	-600		
chr2	44588854	44589164	1.18	5.62E-01	PREPL	-2120		
chr20	271174	271566	1.18	5.62E-01	ZCCHC3	-6834	DEFB132	32993
chr7	55954986	55955258	0.71	5.62E-01	ZNF713	-25206	SEPT14	-24640
chr22	22549806	22550355	0.85	5.62E-01	TOP3B	-212934	VPREB1	-49119
chr17	73105902	73106177	0.98	5.63E-01	ARMC7	-42		
chr20	35089414	35089681	1.32	5.63E-01	MYL9	-80349	DLGAP4	94100
chr10	105726680	105726924	1.17	5.63E-01	SLK	-668		
chr11	62446450	62446793	1.16	5.63E-01	UBXN1	-95		
chr15	66789979	66790312	1.32	5.63E-01	SNAPC5	0		
chr2	8716875	8717258	1.29	5.63E-01	ID2	-104917		
chr2	60579657	60579893	0.88	5.63E-01	BCL11A	200858		
chr2	185463140	185463422	1.03	5.63E-01	ZNF804A	188		
chr19	33667795	33668153	1.25	5.63E-01	LRP3	-17625	GPATCH1	96188
chr6	24667068	24667407	0.84	5.63E-01	TTRAP	-123	ACOT13	-25
chr7	42971753	42972099	0.88	5.63E-01	PSMA2	-121	MRPL32	-13
chr20	34686259	34686607	1.20	5.63E-01	SCAND1	-144005	EPB41L1	-56229
chr6	90984182	90984493	0.78	5.63E-01	BACH2	22224	GJA10	380150
chr15	72668233	72668543	1.22	5.63E-01	HEXA	132		
chr21	43955996	43956315	1.18	5.63E-01	PDE9A	-117706	SLC37A1	36414
chr3	158519625	158519962	0.87	5.64E-01	MFSD1	-118		
chr7	44887967	44888237	0.80	5.64E-01	H2AFV	-377		
chr1	205257415	205257802	1.22	5.64E-01	NUAK2	33274	TMCC2	60518
chr1	173836930	173837289	1.24	5.64E-01	ZBTB37	-383		
chr6	36985999	36986243	0.83	5.64E-01	PIM1	-151801	FGD2	12698
chr17	3814571	3815025	1.14	5.65E-01	CAMKK1	-20761	P2RX1	5162
chr6	2842133	2842460	1.15	5.65E-01	SERPINB1	-216		
chr1	28844616	28844929	0.72	5.65E-01	RCC1	28		
chr2	118571902	118572349	0.91	5.66E-01	DDX18	-129		
chr6	31831350	31831837	0.74	5.66E-01	NEU1	-885		
chr5	43313671	43314034	0.79	5.66E-01	HMGCS1	-258		
chr14	35591616	35591966	0.89	5.66E-01	PPP2R3C	-272	KIAA0391	15
chr22	26908335	26908589	1.17	5.66E-01	TFIP11	-25		
chr1	67966182	67966492	0.83	5.66E-01	GADD45A	-184546	SERBP1	-70214
chr10	94516866	94517184	0.76	5.66E-01	EXOC6	-91200	HHEX	67344
chr11	6423976	6424254	1.30	5.66E-01	SMPD1	12460	APBB1	16529

chr15	74257106	74257416	1.19	5.66E-01	STOML1	27374	LOXL1	38472		
chr12	90103078	90103600	1.06	5.67E-01	ATP2B1	-53495				
chr11	61500899	61501235	1.21	5.67E-01	C11orf9	-19054	DAGLA	53157		
chr4	2965060	2965343	1.06	5.68E-01	GRK4	-141	NOP14	-84		
chr1	151138354	151138653	1.25	5.68E-01	LYSMD1	-134	SCNM1	-13		
chr3	121797467	121797712	1.24	5.68E-01	CASR	-104940	CD86	23369		
chr9	92864642	92864905	1.22	5.68E-01	DIRAS2	540334	GADD45G	644847		
chr13	41507728	41508052	0.76	5.68E-01	ELF1	85618	SLC25A15	144343		
chr12	104531866	104532142	1.09	5.68E-01	NFYB	36				
chr19	3185191	3185978	0.84	5.68E-01	NCLN	-290				
chr4	82556876	82557186	0.86	5.68E-01	RASGEF1B	-163970	HNRNPD	738118		
chr12	49110425	49110786	1.27	5.68E-01	CCNT1	175				
chr5	122847750	122848117	0.98	5.69E-01	CSNK1G3	141				
chr14	67955286	67955542	1.13	5.69E-01	PLEK2	-76586	TMEM229B	26607		
chr14	95794171	95794546	1.11	5.69E-01	CLMN	-8114	C14orf49	147814		
chr2	91818607	91818949	1.10	5.69E-01	FKSG73	-310381				
chr19	6801461	6801754	0.97	5.69E-01	EMR1	-85974	VAV1	28886		
chr14	96000797	96001274	1.12	5.70E-01	GLRX5	-287				
chr12	57505935	57506405	0.77	5.70E-01	STAT6	-1009				
chr19	56904885	56905296	1.23	5.70E-01	ZNF582	-202				
chr9	35071954	35072235	0.98	5.70E-01	VCP	644				
chr16	3179573	3179883	0.88	5.71E-01	ZNF213	-5329	ZNF205	17165		
chr19	50320565	50320875	0.75	5.71E-01	FUZ	-4252	MED25	-826		
chr2	192489906	192490242	1.08	5.71E-01	OBFC2A	-52787	MYO1B	379967		
chr19	12992191	12992516	1.11	5.71E-01	DNASE2	-19				
chr1	220219877	220220135	1.33	5.71E-01	EPRS	-6				
chr14	64212705	64213097	1.16	5.71E-01	SYNE2	-106782	SGPP1	-18145		
chr8	59956329	59956625	1.00	5.71E-01	NSMAF	-384511	TOX	75290		
chr6	12008678	12009063	0.90	5.71E-01	HIVEP1	-3853				
chr11	65186141	65186791	1.21	5.71E-01	SCYL1	-106082	FRMD8	32425		
chr19	36630453	36631059	1.25	5.71E-01	CAPNS1	-162				
chr6	33538551	33538832	1.34	5.71E-01	BAK1	9378	ZBTB9	116336		
chr2	241524054	241524454	0.89	5.71E-01	CAPN10	-1891				
chr1	155057185	155057495	0.92	5.71E-01	EFNA1	-43009	EFNA3	5992		
chr4	25915739	25916066	1.08	5.72E-01	C4orf52	89				
chr16	69373376	69373686	1.13	5.72E-01	NIP7	-15	COG8	-5		
chr19	17416102	17416591	1.16	5.72E-01	ABHD8	-2065	MRPL34	-130		
chr19	44079638	44079967	1.10	5.72E-01	BC071811	-4893	AK296854	-1462	XRCC1	-73

chr6	188807	189140	1.19	5.72E-01	DUSP22	-103127		
chr7	44924970	44925355	0.88	5.72E-01	PURB	-203		
chr19	18049103	18049347	0.82	5.72E-01	KCNN1	-12886	SLC5A5	66443
chr3	130465503	130465946	1.22	5.73E-01	PIK3R4	-29		
chr10	127730328	127730740	0.78	5.73E-01	FANK1	145426	ADAM12	346593
chr11	118798749	118799499	1.21	5.73E-01	UPK2	-27902	BCL9L	-17511
chr10	27088335	27088635	1.20	5.73E-01	ABI1	61474	PDSS1	101890
chr19	10827926	10828657	0.82	5.74E-01	DNM2	-463		
chr1	203294088	203294646	0.79	5.74E-01	BTG2	19703	FMOD	25922
chr1	156474747	156475070	1.30	5.74E-01	MEF2D	-4380		
chr12	92839084	92839467	1.10	5.74E-01	BTG1	-299603	PLEKHG7	-290989
chr2	11606271	11606530	0.83	5.74E-01	E2F6	-104		
chr2	64276758	64277165	1.15	5.74E-01	VPS54	-30748	PELI1	94643
chr6	53213764	53214085	0.89	5.75E-01	ELOVL5	17		
chr1	169763981	169764293	1.24	5.75E-01	C1orf156	-106		
chr3	64009056	64009332	1.38	5.75E-01	PSMD6	-74		
chr3	187696442	187697009	0.84	5.75E-01	LPP	-233995	BCL6	-233251
chr10	6128304	6128711	1.05	5.76E-01	RBM17	-2441		
chr4	81796848	81797105	1.33	5.76E-01	BMP3	-155142	FGF5	609235
chr1	24285370	24285805	0.95	5.76E-01	PNRC2	-713		
chr1	23851504	23851927	0.98	5.76E-01	ASAP3	-40966	E2F2	5997
chr10	97849742	97850143	0.88	5.76E-01	ZNF518A	-39529	CCNJ	46784
chr3	13503114	13503424	0.84	5.76E-01	NUP210	-41460	HDAC11	-18446
chr3	187457782	187458196	1.20	5.76E-01	RTP2	-37644	BCL6	5486
chr14	68750177	68750465	0.93	5.76E-01	RAD51L1	463812	ZFP36L1	509464
chr19	6393713	6394093	1.07	5.77E-01	GTF2F1	-612		
chr11	7533776	7534061	1.17	5.77E-01	PPFIBP2	-1082		
chr5	68389560	68389860	1.26	5.77E-01	SLC30A5	-108		
chr9	34126568	34126942	1.12	5.77E-01	DCAF12	16		
chr2	109252824	109253147	0.79	5.77E-01	RANBP2	-82951	LIMS1	48219
chr19	2171324	2171634	0.91	5.78E-01	SF3A2	-65337	DOT1L	7331
chr19	2236596	2236906	1.18	5.78E-01	SF3A2	-65	PLEKHJ1	200
chr14	91708267	91708577	1.37	5.78E-01	GPR68	11802	C14orf159	127443
chr2	219757407	219757719	1.11	5.78E-01	CDK5R2	-66835	WNT10A	12308
chr19	6528023	6528533	0.88	5.79E-01	TNFSF9	-2732		
chr8	84230218	84230567	0.78	5.79E-01	RALYL	-866707		
chr2	38978338	38978715	1.19	5.79E-01	SFRS7	109		
chr16	88860	89170	0.99	5.79E-01	WASH1	-19567	POLR3K	14610

chr16	53132910	53133210	1.07	5.79E-01	RBL2	-335291	CHD9	44115
chr12	122750882	122751466	1.24	5.80E-01	VPS33A	-106		
chr11	121238295	121238645	0.93	5.80E-01	SORL1	-84491	SC5DL	75082
chr3	52259648	52259924	1.14	5.80E-01	TLR9	5461	ALAS1	27670
chr7	105697392	105697702	0.79	5.80E-01	SYPL1	55510	FLJ23834	93890
chr7	99036354	99036622	0.85	5.80E-01	CPSF4	-75	PTCD1	-69
chr17	25544655	25544995	1.27	5.81E-01	WSB1	-76281		
chr17	74477214	74477524	1.16	5.81E-01	AANAT	13718	RHBDF2	20139
chr10	27530991	27531300	0.87	5.81E-01	ACBD5	-1338		
chr14	75745080	75745496	0.77	5.81E-01	FOS	-193		
chr14	65672158	65672484	1.22	5.81E-01	FUT8	-207214	MAX	-103094
chr12	7341980	7342404	1.08	5.82E-01	PEX5	-766		
chr12	69863879	69864282	0.75	5.82E-01	FRS2	-48		
chr3	195163909	195164221	1.06	5.82E-01	ACAP2	-248		
chr12	112856446	112856759	1.05	5.82E-01	PTPN11	67		
chr2	39348082	39348393	1.10	5.83E-01	SOS1	-634		
chr19	39360457	39360789	1.16	5.83E-01	HNRNPL	-20006	RINL	8271
chr3	129295629	129296093	0.82	5.83E-01	PLXND1	29721	H1FOO	33804
chr4	90238093	90238340	1.39	5.83E-01	TIGD2	204249	SNCA	520133
chr17	20946689	20947086	0.81	5.83E-01	USP22	-536		
chr4	17812313	17812665	1.04	5.83E-01	DCAF16	-108	NCAPG	-36
chr19	9938343	9938703	1.19	5.83E-01	UBL5	-45		
chr7	30323719	30324098	1.10	5.83E-01	ZNRF2	-14		
chr10	35668028	35668520	0.87	5.83E-01	GJD4	-226064	CCNY	42472
chr7	150725693	150725937	1.26	5.83E-01	ATG9B	-4229	ABCB8	305
chr16	88522470	88522833	0.75	5.83E-01	ZC3H18	-114137	ZFPM1	2638
chr6	125918771	125919050	1.39	5.83E-01	HDDC2	-295629	HEY2	-151821
chr3	142297550	142297966	1.26	5.83E-01	ATR	-90		
chr5	131892336	131892738	1.22	5.83E-01	RAD50	-93		
chr7	129007443	129007718	0.77	5.84E-01	NRF1	-243974	AHCYL2	142717
chr19	17447996	17448415	1.28	5.84E-01	ANO8	-2568	GTPBP3	-150
chr21	45579650	45579960	0.91	5.84E-01	C21orf33	26311	ICOSLG	81029
chr2	60777456	60777908	1.12	5.84E-01	BCL11A	2951		
chr20	34542215	34542597	1.23	5.84E-01	SCAND1	22		
chr16	57682983	57683293	0.76	5.84E-01	GPR97	-19019	GPR56	29228
chr8	42645440	42645717	1.03	5.84E-01	CHRNA6	-21960	THAP1	52895
chr11	73472161	73472442	1.19	5.84E-01	RAB6A	-101		
chr5	1316438	1316802	0.87	5.85E-01	TERT	-21458	CLPTM1L	28382

chr9	36190608	36190946	0.87	5.85E-01	CLTA	-115		
chr7	72971798	72972238	1.05	5.85E-01	BCL7B	6		
chr20	31331644	31331954	1.30	5.85E-01	COMMD7	15		
chr5	168006539	168006990	1.21	5.85E-01	PANK3	-177		
chr1	169336946	169337209	1.08	5.85E-01	BLZF1	-116	NME7	108
chr3	57102571	57102872	1.19	5.85E-01	ERC2	-600331	ARHGEF3	10614
chr5	176856322	176856673	1.11	5.85E-01	PRR7	-17298	GRK6	2811
chr10	5726458	5726816	0.83	5.85E-01	ASB13	-18079	GDI2	128875
chr16	28857608	28857945	1.16	5.85E-01	TUFM	-48		
chr4	129730657	129731003	1.18	5.86E-01	PHF17	51		
chr12	21654336	21654767	1.23	5.86E-01	GOLT1B	-147	RECQL	51
chr7	100034000	100034455	0.85	5.86E-01	MEPCE	6699	TSC22D4	42674
chr13	31019828	31020086	0.72	5.86E-01	KATNAL1	-138794	HMGB1	20124
chr14	102785841	102786151	1.10	5.87E-01	ZNF839	-100		
chr17	1101381	1101695	0.82	5.87E-01	TUSC5	-81419	ABR	-18407
chr7	2276925	2277254	0.88	5.87E-01	NUDT1	-4767	MAD1L1	-4507
chr15	65185781	65186601	0.82	5.87E-01	PIF1	-68353	ANKDD1A	-17910
chr9	68726301	68726546	1.25	5.87E-01	FOXD4L6	475780	ANKRD20A3	799663
chr11	105892994	105893244	1.15	5.88E-01	KIAA1826	-165		
chr16	85061004	85061385	1.05	5.88E-01	KIAA0513	-215		
chr21	43916252	43916583	0.89	5.88E-01	SLC37A1	-3324	RSPH1	-17
chr21	45563325	45563570	0.81	5.88E-01	C21orf33	9954	ICOSLG	97386
chr15	49103400	49103644	1.19	5.88E-01	CEP152	-338		
chr15	55611272	55611604	0.92	5.88E-01	PIGB	305		
chr12	95397408	95397677	1.06	5.88E-01	NDUFA12	-32		
chr3	127794474	127794774	1.11	5.88E-01	SEC61A1	23412	RUVBL1	48047
chr3	14822465	14822741	0.72	5.88E-01	FGD5	-37866	C3orf20	105949
chr3	121468583	121468981	1.18	5.89E-01	GOLGB1	-180		
chr2	122288375	122288801	1.31	5.90E-01	TFCP2L1	-245810	CLASP1	118464
chr2	170550920	170551273	1.04	5.90E-01	KLHL23	122	PHOSPHO2	122
chr20	46377580	46377877	0.78	5.90E-01	SULF2	37079	NCOA3	247072
chr10	126480369	126480613	0.81	5.90E-01	METTL10	-52		
chr1	199212662	199212901	1.21	5.90E-01	NR5A2	-783988	PTPRC	604645
chr21	11109027	11109317	1.14	5.90E-01	BAGE	-10235		
chr7	127849865	127850131	0.79	5.90E-01	LRRC4	-178996	LEP	-31333
chr19	38826118	38826653	0.92	5.90E-01	CATSPERG	-57		
chr15	101463250	101463619	1.29	5.90E-01	LRRK1	3975	CHSY1	328691
chr3	190335066	190335321	1.30	5.90E-01	IL1RAP	103303	LOC647309	245271

chr22	30787163	30787476	0.81	5.91E-01	RNF215	-4018		
chr22	41697284	41697715	0.93	5.91E-01	ZC3H7B	-67		
chr19	42636808	42637115	1.30	5.91E-01	POU2F2	-332		
chr8	33370981	33371276	1.38	5.91E-01	MAK16	28444	RNF122	53514
chr6	31774564	31774840	1.30	5.91E-01	LSM2	41		
chr15	80216217	80216575	1.09	5.91E-01	MTHFS	-27026	BCL2A1	47247
chr3	57741836	57742170	1.22	5.91E-01	SLMAP	-1171		
chr17	21022970	21023235	1.14	5.92E-01	USP22	-76751	DHRS7B	-7155
chr6	26031765	26032614	1.20	5.92E-01	HIST1H4B	-4710	HIST1H3B	98
chr6	26313178	26313533	0.83	5.92E-01	BTN3A2	-52042	HIST1H4H	-27629
chr6	26473971	26474281	0.95	5.92E-01	BTN1A1	-27369	BTN2A1	15937
chr7	75943557	75943915	0.76	5.92E-01	HSPB1	11861	YWHAG	44606
chr7	98062645	98062928	0.93	5.92E-01	NPTX2	-183810	BAIAP2L1	-32360
chr17	29136011	29136292	1.23	5.92E-01	CRLF3	15626	LRRC37B2	200704
chr19	39108588	39108954	1.15	5.92E-01	EIF3K	-951	MAP4K1	-128
chr2	28113195	28113688	1.26	5.92E-01	RBKS	-219	BRE	-40
chr20	33999632	33999955	1.24	5.92E-01	UQCC	39		
chr1	115323132	115323446	1.23	5.92E-01	SIKE1	19		
chr19	15947600	15948094	0.93	5.92E-01	OR10H1	-28911	CYP4F2	61037
chr1	28201753	28202007	1.10	5.92E-01	C1orf38	2825	RPA2	39356
chr6	26157292	26157627	0.88	5.92E-01	HIST1H2BD	-889	HIST1H1E	901
chr1	36852144	36852396	0.79	5.93E-01	STK40	-785		
chr6	111195811	111196140	0.90	5.93E-01	AMD1	-11		
chr1	234508993	234509523	1.32	5.93E-01	C1orf31	-171		
chr16	89556974	89557355	0.97	5.93E-01	ANKRD11	-196		
chr19	46320387	46320765	1.05	5.93E-01	RSHL1	-1999		
chr3	38038274	38038627	1.44	5.93E-01	VILL	3373	PLCD1	27827
chr3	48672596	48673336	0.84	5.93E-01	TMEM89	-13777	CELSR3	37015
chr2	238512326	238512585	0.84	5.94E-01	LRRFIP1	-88351	RAB17	-12720
chr2	152266227	152266537	0.91	5.94E-01	RIF1	-46		
chr19	50879424	50879739	1.11	5.94E-01	NR1H2	90		
chr2	33824280	33824634	0.99	5.94E-01	RASGRP3	85515	tmp_locus_39	128827
chr1	115124175	115124577	1.17	5.94E-01	BCAS2	-111		
chr13	41345234	41345561	0.94	5.94E-01	MRPS31	-51		
chr2	30934519	30934829	1.24	5.94E-01	CAPN13	95637	LCLAT1	264537
chr7	129710053	129710430	1.24	5.94E-01	ZC3HC1	-19009	TMEM209	135096
chr5	148930944	148931248	1.11	5.95E-01	CSNK1A1	-89		
chr3	194412818	194413128	0.84	5.95E-01	LSG1	-19767	C3orf21	578922

chr17	16120324	16120663	1.23	5.95E-01	NCOR1	-1649	PIGL	-15
chr13	42845778	42846123	1.28	5.95E-01	AKAP11	-338		
chr19	4182428	4182769	1.18	5.95E-01	SIRT6	-3		
chr9	5437703	5438246	0.82	5.95E-01	C9orf46	-115		
chr6	27759958	27760288	1.13	5.95E-01	ZNF184	-319226	HIST1H2BL	15586
chr16	28565088	28565468	1.11	5.95E-01	CCDC101	29		
chr11	76391920	76392164	0.80	5.95E-01	LRRC32	-10998	GUCY2E	40791
chr9	116569765	116570134	1.15	5.95E-01	ZNF618	-68612	RGS3	362939
chr11	125439057	125439332	1.20	5.95E-01	EI24	-103		
chr17	56415278	56415731	1.18	5.95E-01	BZRAP1	-9353	SUPT4H1	14058
chr10	102133028	102133354	1.12	5.95E-01	WNT8B	-89621	SCD	26419
chr18	30502857	30503180	0.77	5.95E-01	ASXL3	-655522	KLHL14	-150045
chr2	144998474	144998878	0.93	5.95E-01	GTDC1	53384		
chr15	34659667	34660037	0.84	5.95E-01	LPCAT4	-457		
chr15	99791364	99791657	1.34	5.95E-01	TTC23	-1696	LRRC28	-141
chr6	41747628	41747941	0.85	5.95E-01	PRICKLE4	-715	FRS3	-155
chr7	150102327	150102607	1.02	5.95E-01	GIMAP8	-45495	ZNF775	26061
chr9	136819976	136820466	0.99	5.95E-01	SARDH	-216742	VAV2	37225
chr2	10262727	10263078	0.84	5.96E-01	RRM2	168		
chr2	27309403	27309728	1.01	5.96E-01	KHK	-45		
chr1	171454405	171454739	1.23	5.96E-01	BAT2D1	-94		
chr10	16859571	16859865	1.05	5.97E-01	RSU1	-265		
chr3	127872003	127872390	0.90	5.97E-01	EEFSEC	-116		
chr19	633490	633810	1.14	5.97E-01	POLRMT	-82		
chr5	173042913	173043195	1.28	5.98E-01	BOD1	612		
chr11	82996962	82997615	1.33	5.98E-01	CCDC90B	88		
chr12	122326359	122326658	1.21	5.98E-01	PSMD9	-137		
chr12	124068839	124069083	1.03	5.98E-01	TMED2	-115		
chr14	104387767	104388053	1.16	5.98E-01	C14orf2	-7		
chr18	19192025	19192598	0.79	5.98E-01	SNRPD1	52		
chr7	43769043	43769312	0.87	5.98E-01	C7orf44	-95		
chr7	72299769	72300082	1.13	5.98E-01	TYW1B	-1113		
chr12	25173523	25173873	1.24	5.98E-01	BCAT1	-71390	LRMP	-31543
chr12	98897154	98897473	1.26	5.98E-01	AX747640	-46391	TMPO	-12095
chr17	55628735	55629257	1.18	5.98E-01	MSI2	295065	MRPS23	298403
chr21	43288441	43288808	0.90	5.98E-01	RIPK4	-101376	PRDM15	10966
chr3	183254023	183254515	1.24	5.98E-01	MCF2L2	-108414	KLHL6	19230
chr21	42693090	42693496	1.16	5.98E-01	MX2	-40657	FAM3B	4632

chr19	36980300	36980772	1.23	5.98E-01	ZNF566	-73		
chr2	201390599	201391195	0.93	5.98E-01	SGOL2	32		
chr19	59070778	59071270	1.24	5.99E-01	CHMP2A	-4538	UBE2M	-681
chr7	143824870	143825114	1.26	5.99E-01	OR2A14	-1214		
chr16	790922	791210	1.19	5.99E-01	NARFL	-69		
chr1	32429649	32429996	1.03	5.99E-01	KHDRBS1	-49668	PTP4A2	-25835
chr1	112289909	112290219	0.96	5.99E-01	DDX20	-8126	RAP1A	127659
chr19	13263615	13264038	0.80	5.99E-01	STX10	-2840	IER2	2545
chr22	39101562	39101882	1.06	5.99E-01	GTPBP1	-85		
chr7	105229953	105230226	1.17	5.99E-01	FLJ23834	-373567	RINT1	57558
chr9	137029433	137029768	1.24	5.99E-01	RXRA	-188715	WDR5	28391
chr15	70390042	70390356	1.12	5.99E-01	TLE3	57		
chr16	90014490	90014862	0.86	5.99E-01	DEF8	-475		
chr1	111756968	111757380	0.82	5.99E-01	DRAM2	-74336	CHI3L2	-13107
chr15	34635174	34635547	0.88	5.99E-01	C15orf55	-2705	NOP10	1
chr15	64302950	64303336	1.13	6.00E-01	HERC1	-176996	DAPK2	35378
chr17	42785835	42786145	1.28	6.00E-01	DBF4B	14		
chr19	4724543	4724874	1.24	6.00E-01	DPP9	-854		
chr3	101441274	101441584	1.20	6.00E-01	CEP97	-2065		
chr12	120729402	120730084	1.36	6.00E-01	PXN	-26180	SIRT4	-10420
chr13	114103347	114103927	0.94	6.00E-01	GRTP1	-85174	ADPRHL1	4202
chr16	30361880	30362167	0.84	6.00E-01	CD2BP2	4658	SULT1A3	151488
chr17	76704598	76704898	1.16	6.00E-01	DNAH17	-137344	CYTH1	73628
chr1	148556596	148556886	1.31	6.00E-01	NBPF15	-4106		
chr14	77923819	77924228	1.11	6.00E-01	AHSA1	-349	C14orf133	-120
chr7	73631996	73632385	1.10	6.00E-01	LAT2	8104	RFC2	36547
chr8	103666219	103666501	0.80	6.00E-01	KLF10	1623	ODF1	102512
chr10	46951327	46951603	1.09	6.00E-01	FRMPD2L1	-275762	SYT15	19136
chr1	230777968	230778259	0.97	6.00E-01	COG2	-88		
chr14	23590072	23590319	1.12	6.00E-01	CEBPE	-1722		
chr17	41400890	41401190	0.72	6.00E-01	ARL4D	-75313	TMEM106A	37146
chr2	65609929	65610189	0.89	6.00E-01	SPRED2	49597	ACTR2	155230
chr2	86263151	86263509	1.14	6.00E-01	ST3GAL5	-147173	POLR1A	69948
chr3	13087871	13088217	1.04	6.00E-01	RPL32	-206095	IQSEC1	26573
chr15	75400915	75401199	1.12	6.01E-01	PPCDC	85130	DNM1P33	194249
chr7	102937686	102937971	0.83	6.01E-01	PMPCB	-44		
chr15	85471485	85471737	0.75	6.02E-01	PDE8A	-53594	SLC28A1	43698
chr9	5626923	5627577	0.84	6.02E-01	KIAA1432	-29333	PDCD1LG2	116705

chr3	72212949	72213391	0.84	6.02E-01	PROK2	-378813	RYBP	282604
chr1	45205278	45205619	1.14	6.02E-01	KIF2C	-41		
chr14	23790065	23790470	1.08	6.02E-01	PABPN1	871		
chr2	86448227	86448474	0.81	6.02E-01	MRPL35	21795	REEP1	116426
chr14	103544663	103544907	1.05	6.03E-01	TNFAIP2	-47879	CDC42BPB	-21043
chr10	27389116	27389591	0.88	6.04E-01	ABI1	-239395	YME1L1	53967
chr15	74753320	74753844	1.22	6.04E-01	UBL7	-53		
chr3	38388061	38388356	1.09	6.04E-01	XYLB	-42		
chr3	52029803	52030132	1.30	6.04E-01	RPL29	-10		
chr4	39979457	39979772	1.02	6.04E-01	PDS5A	-132		
chr5	139554344	139554712	1.05	6.04E-01	C5orf53	49007	PFDN1	128161
chr13	37574700	37575147	0.94	6.04E-01	ALG5	-1420	EXOSC8	246
chr10	89867978	89868387	1.25	6.05E-01	KILLIN	-244989	RNLS	474899
chr4	146859928	146860238	0.96	6.05E-01	ZNF827	-476		
chr3	20227671	20227945	0.91	6.05E-01	SGOL1	-125		
chr5	72861236	72861703	1.09	6.05E-01	UTP15	-128	ANKRA2	28
chr5	25955551	25955833	1.36	6.05E-01				
chr10	65801007	65801275	0.75	6.05E-01	REEP3	520018		
chr1	167178320	167178630	0.94	6.05E-01	POU2F1	-11668	DUSP27	114404
chr15	73928832	73929111	1.14	6.06E-01	NPTN	-3219		
chr19	39832509	39832827	1.22	6.06E-01	SAMD4B	-440		
chr12	53715228	53715576	1.15	6.06E-01	AAAS	10		
chr11	124823807	124824246	0.97	6.06E-01	SLC37A2	-108986	HEPACAM	-17719
chr19	47777393	47777842	1.06	6.06E-01	PRR24	-524		
chr16	30672074	30672318	1.08	6.06E-01	FBRS	-3582		
chr9	100395348	100395810	1.13	6.06E-01	NCBP1	-126		
chr15	91497906	91498177	1.12	6.06E-01	UNC45A	19827	PRC1	39762
chr5	5422368	5422762	0.92	6.06E-01	ADAMTS16	282122	MED10	956074
chr1	151299919	151300229	0.82	6.07E-01	PI4KB	-1225		
chr12	69753355	69753599	1.17	6.07E-01	YEATS4	-55		
chr2	33700785	33701073	1.32	6.07E-01	RASGRP3	-38013	LTBP1	528537
chr3	121264744	121265032	1.21	6.07E-01	POLQ	-35		
chr13	45903812	45904100	1.18	6.08E-01	KCTD4	-128781	TPT1	11341
chr19	8009138	8009524	0.81	6.08E-01	TIMM44	-793		
chr4	190861803	190862080	0.80	6.08E-01	FRG1	-32		
chr10	49880372	49880731	0.81	6.09E-01	ARHGAP22	-67414	WDFY4	-12966
chr1	155197212	155197690	1.12	6.09E-01	GBA	13602	MTX1	18961
chr7	72395440	72395714	0.84	6.09E-01	POM121	-83		

chr1	149223568	149224358	1.40	6.09E-01	PPIAL4B	-329040	NBPF16	484521
chr8	61705460	61705800	0.86	6.09E-01	RLBP1L1	-494895	CHD7	114291
chr14	23025099	23025675	1.16	6.09E-01	DAD1	32756	TCRDV2	97298
chr19	54641116	54641437	0.98	6.09E-01	CNOT3	-172		
chr8	99129355	99129625	1.34	6.09E-01	HRSP12	-72	POP1	-31
chr17	76627930	76628339	1.37	6.09E-01	DNAH17	-60731	CYTH1	150241
chr16	23464365	23464676	1.05	6.10E-01	COG7	-18		
chr19	18439173	18439468	0.84	6.10E-01	PGPEP1	-12087	LSM4	-5320
chr12	57472475	57472711	0.78	6.10E-01	TMEM194A	-19		
chr1	1765473	1765749	0.88	6.10E-01	NADK	-55702	GNB1	56884
chr8	23103781	23104406	1.17	6.10E-01	R3HCC1	-41518	CHMP7	2944
chr19	46010531	46010881	0.94	6.10E-01	VASP	18		
chr3	51428401	51428839	1.23	6.11E-01	RBM15B	-111		
chr4	25314092	25314510	0.89	6.11E-01	ZCCHC4	-95		
chr15	69755059	69755419	1.09	6.11E-01	RPLP1	10080	TLE3	635017
chr1	67461756	67462066	0.83	6.11E-01	SLC35D1	58169	MIER1	71333
chr12	65015013	65015399	1.25	6.12E-01	RASSF3	10913	GNS	138020
chr10	13873892	13874202	0.67	6.12E-01	PRPF18	245108	FRMD4A	498819
chr9	126101546	126101927	1.12	6.12E-01	STRBP	-70894	CRB2	-16711
chr12	49454430	49454759	0.98	6.12E-01	MLL2	-5488	RHEBL1	9180
chr15	85197961	85198271	0.78	6.12E-01	NMB	3686	SCAND2	23425
chr14	82000178	82000575	1.04	6.12E-01	SEL1L	-172		
chr11	118069015	118069299	1.10	6.12E-01	SCN2B	-21820	AMICA1	26652
chr17	7746958	7747434	1.20	6.12E-01	TMEM88	-11188	KDM6B	3961
chr10	101380312	101380624	1.08	6.12E-01	SLC25A28	-247		
chr2	224810010	224810261	1.12	6.12E-01	WDFY1	-84		
chr1	67896128	67896502	1.23	6.12E-01	SERBP1	-192		
chr6	41754918	41755520	0.86	6.12E-01	TOMM6	719		
chr6	18359716	18360026	1.13	6.12E-01	DEK	-95072	RNF144B	-27723
chr6	18155402	18155865	0.77	6.12E-01	TPMT	-260	KDM1B	15
chr12	54891248	54891603	0.81	6.12E-01	NCKAP1L	-69		
chr1	192574170	192574512	1.22	6.12E-01	RGS13	-30941	RGS1	29484
chr11	47475826	47476102	1.14	6.12E-01	RAPSN	-5234	CUGBP1	34612
chr11	75946427	75946726	1.22	6.12E-01	WNT11	-29003	PRKRIR	145303
chr2	27273451	27274661	1.28	6.12E-01	AGBL5	-435		
chr14	23770610	23771052	1.06	6.12E-01	PABPN1	-18566	HOMEZ	-15522
chr20	1305786	1306096	0.85	6.12E-01	SDCBP2	3897	SNPH	58981
chr14	22855893	22856231	0.68	6.13E-01	TCRDV2	-72027	TRA	380298

chr16	88747503	88747813	1.19	6.13E-01	MVD	-18163	SNAI3	5224
chr9	4762577	4762858	1.39	6.13E-01	RCL1	-30116	AK3	-21491
chr1	39724383	39724644	0.80	6.13E-01	MACF1	-72296	NDUFS5	232508
chr10	2742867	2743177	1.28	6.13E-01	ADARB2	-963304	PFKP	-366730
chr1	146643823	146644440	0.89	6.14E-01	PRKAB2	-3		
chr1	12221666	12222175	0.90	6.14E-01	TNFRSF1B	-5139	TNFRSF8	98487
chr9	80911614	80912058	1.15	6.15E-01	PSAT1	-223		
chr8	22526599	22526921	1.33	6.15E-01	BIN3	-99		
chr1	155164591	155164842	0.87	6.15E-01	MUC1	-2017		
chr12	14310075	14310418	1.24	6.15E-01	ATF7IP	-208364	GRIN2B	-177225
chr2	10549250	10549578	1.37	6.15E-01	ODC1	39039	HPCAL1	106374
chr1	235292078	235292516	1.14	6.15E-01	TOMM20	-41		
chr1	153931068	153931320	1.26	6.15E-01	CRTC2	-151		
chr19	11670007	11670295	1.35	6.15E-01	ELOF1	-100		
chr6	157098277	157098586	0.94	6.15E-01	ARID1B	-654		
chr13	114894503	114894767	1.21	6.15E-01	GAS6	-327589	RASA3	3460
chr2	152685022	152685300	1.17	6.16E-01	ARL5A	-152		
chr7	138144947	138145320	0.79	6.16E-01	TRIM24	55		
chr19	50379745	50380484	1.23	6.16E-01	TBC1D17	-714	AKT1S1	497
chr15	90734829	90735363	1.15	6.16E-01	IDH2	-89388	SEMA4B	-9466
chr6	43351532	43351803	1.21	6.16E-01	ABCC10	-43624	ZNF318	-14487
chr7	35770030	35770461	1.18	6.17E-01	SEPT7	-70381	HERPUD2	-35474
chr6	30640661	30641030	0.89	6.17E-01	DHX16	-16		
chr1	161067435	161067788	1.37	6.17E-01	PVRL4	-8227	PFDN2	20254
chr16	57334263	57334657	1.24	6.17E-01	CCL22	-58258	PLLP	-15889
chr7	127877033	127877367	0.84	6.17E-01	LEP	-4131		
chr1	226186911	226187485	1.19	6.17E-01	H3F3B	-63223	LEFTY2	-58278
chr8	37259348	37260003	0.95	6.17E-01	ZNF703	-293625	FKSG2	513679
chr22	50319170	50319446	1.07	6.18E-01	PIM3	-34835	CRELD2	7025
chr2	74699671	74699981	0.97	6.19E-01	MRPL53	97		
chr6	150944692	150944996	0.92	6.19E-01	MTHFD1L	-241847	PLEKHG1	23845
chr15	101835371	101835630	1.26	6.19E-01	SNRPA1	-41		
chr16	3398585	3398865	1.21	6.19E-01	OR2C1	-7164	ZNF75A	43292
chr11	86748707	86748951	1.09	6.19E-01	TMEM135	-236		
chr13	46758364	46758841	0.93	6.19E-01	LCP1	-2144		
chr16	69220448	69220758	0.89	6.19E-01	SNTB2	-447		
chr8	33370556	33370823	1.09	6.19E-01	MAK16	28005	RNF122	53953
chr17	48225771	48226169	1.29	6.19E-01	PPP1R9B	1907	PDK2	53274

chr14	90420983	90421245	1.11	6.20E-01	TDP1	-1132	C14orf143	-25
chr8	42029167	42029471	0.92	6.20E-01	AP3M2	18855	PLAT	35875
chr1	198590056	198590396	1.34	6.20E-01	ATP6V1G3	-80151	PTPRC	-17911
chr14	75725770	75726272	0.83	6.20E-01	TMED10	-82672	FOS	-19460
chr15	50978626	50979411	1.16	6.20E-01	TRPM7	-25		
chr19	56092168	56092445	1.28	6.20E-01	ZNF579	-96		
chr15	75660238	75660648	1.15	6.21E-01	MAN2C1	498		
chr20	34078693	34079003	0.81	6.21E-01	ERGIC3	-50930	CEP250	35698
chr12	102137559	102137823	0.78	6.21E-01	SYCP3	-4444		
chr16	87736005	87736376	1.05	6.21E-01	KLHDC4	63351	JPH3	99692
chr22	22555640	22555950	1.16	6.21E-01	TOP3B	-218648	VPREB1	-43405
chr11	129872938	129873271	0.96	6.21E-01	PRDM10	-375		
chr2	48541325	48541761	1.12	6.21E-01	FOXN2	-252		
chr5	19270293	19270639	1.10	6.22E-01	CDH18	710821		
chr7	139044211	139044659	1.11	6.22E-01	LUC7L2	-199		
chr19	16222098	16222354	1.03	6.22E-01	RAB8A	-264		
chr12	14927012	14927474	1.29	6.23E-01	HIST4H4	-3178	H2AFJ	-27
chr7	104583904	104584453	0.99	6.23E-01	MLL5	-70458	LHFPL3	615075
chr3	196466499	196466940	1.15	6.24E-01	PAK2	-8		
chr5	180633690	180634036	1.32	6.24E-01	TRIM7	-1686		
chr15	81589113	81589423	1.24	6.24E-01	STARD5	27256	IL16	100049
chr16	4475363	4475662	1.18	6.24E-01	DNAJA3	-345		
chr10	98486664	98486922	0.67	6.24E-01	C10orf12	-254248	PIK3AP1	-6514
chr2	161993264	161993591	1.04	6.24E-01	TANK	-38		
chr2	202316094	202316416	1.14	6.24E-01	STRADB	-137	TRAK2	64
chr12	125424369	125424712	1.00	6.25E-01	UBC	-24964	DHX37	49126
chr17	57232521	57232900	1.17	6.25E-01	SKA2	89		
chr7	99933473	99933888	0.86	6.25E-01	PILRB	-21945	PVRIG	116809
chr6	13615384	13615694	0.93	6.26E-01	NOL7	-20		
chr8	90738083	90738329	1.20	6.26E-01	RIPK2	-31769		
chr21	40203497	40203745	1.07	6.26E-01	ETS2	25772	PSMG1	351819
chr22	24552895	24553281	0.86	6.26E-01	SUSD2	-24356	CABIN1	145323
chr17	25620939	25621516	1.13	6.26E-01	WSB1	122		
chr19	42772302	42772702	1.07	6.26E-01	CIC	-16315	ERF	-13193
chr6	26156464	26156844	0.90	6.26E-01	HIST1H2BD	-1695	HIST1H1E	95
chr7	41921231	41921613	1.28	6.26E-01	INHBA	-178716	GLI3	355196
chr3	133524427	133524886	1.11	6.26E-01	SRPRB	-20		
chr8	62627037	62627461	1.04	6.27E-01	ASPH	-50		

chr19	2051523	2051808	0.79	6.27E-01	MKNK2	-423		
chr12	7063017	7063370	1.14	6.27E-01	PTPN6	7454	PHB2	16722
chr12	95611390	95611678	1.06	6.27E-01	FGD6	-294	VEZT	12
chr7	156742227	156742471	1.00	6.28E-01	NOM1	-68		
chr22	43506437	43506755	0.79	6.28E-01	BIK	-161		
chr11	63706084	63706375	0.94	6.28E-01	NAT11	-212		
chr17	37310451	37310963	0.97	6.28E-01	PLXDC1	-2805		
chr18	43753347	43753702	1.17	6.28E-01	RNF165	-160662	HAUS1	69227
chr6	27777621	27778065	0.96	6.28E-01	HIST1H2BM	-4979	HIST1H2BL	-2134
chr11	4115849	4116203	0.86	6.29E-01	RRM1	102		
chr20	49407050	49407519	0.81	6.29E-01	BCAS4	-4182		
chrX	199843	200153	1.01	6.29E-01	PLCXD1	1937	GTPBP6	30889
chr1	45196358	45196861	1.36	6.29E-01	KIF2C	-8880	C1orf228	56216
chr11	506736	507020	1.13	6.29E-01	RNH1	-57		
chr7	106301306	106301904	1.19	6.29E-01	NAMPT	-375967	PIK3CG	-204319
chr16	84674846	84675118	1.11	6.29E-01	COTL1	-23313	KLHL36	-7149
chr2	15731813	15732159	0.91	6.29E-01	DDX1	216		
chr20	57226073	57226411	1.35	6.29E-01	STX16	-86		
chr5	49741123	49741420	0.90	6.30E-01	EMB	-4038		
chr9	37485475	37486103	0.80	6.30E-01	POLR1E	-156		
chr16	58664246	58664567	1.21	6.30E-01	CNOT1	-657		
chr12	57024050	57024360	0.86	6.31E-01	BAZ2A	5958	RBMS2	108596
chr8	125486626	125487061	1.06	6.31E-01	RNF139	-164		
chr17	61920413	61920704	1.05	6.31E-01	SMARCD2	-208		
chrX	47517890	47518298	1.01	6.31E-01	UXT	422		
chr1	167683766	167684094	1.38	6.31E-01	CREG1	-160874	MPZL1	-7257
chr20	18547810	18548185	1.21	6.31E-01	DTD1	-20558	SEC23B	59810
chr3	183967262	183967558	1.11	6.31E-01	ALG3	-651	ECE2	-35
chr19	2749185	2749520	1.18	6.31E-01	SLC39A3	-9279	SGTA	34001
chr4	39183708	39184111	1.16	6.32E-01	WDR19	-114		
chr1	40626846	40627090	0.83	6.32E-01	RLF	-73		
chr16	69599658	69599947	1.02	6.32E-01	NFAT5	806		
chr17	4634433	4634789	1.24	6.32E-01	MED11	-112		
chr7	90033011	90033311	1.26	6.32E-01	CLDN12	365		
chr11	64684804	64685135	1.01	6.32E-01	EHD1	-38779	PPP2R5B	-7210
chr20	10415704	10416175	1.26	6.32E-01	MKKS	-1074	C20orf94	-11
chr20	50211602	50211912	1.26	6.32E-01	NFATC2	-52499	ATP9A	173151
chr8	27224073	27224383	0.91	6.32E-01	PTK2B	41174	CHRNA2	112585

chr1	27070496	27070934	0.82	6.33E-01	PIGV	-43771	ARID1A	48193
chr11	64884953	64885628	1.21	6.33E-01	MRPL49	-4437	ZNHIT2	-121
chr16	67969598	67969851	0.87	6.33E-01	CTRL	-3960		
chr19	11485541	11485869	1.03	6.33E-01	EPOR	9314	LPPR2	19598
chr2	232646082	232646475	1.15	6.33E-01	COPS7B	-4883	PDE6D	-305
chr17	73401765	73402023	1.04	6.34E-01	GRB2	-104		
chr13	52389239	52389564	0.93	6.34E-01	CCDC70	-46715	DHRS12	-11109
chr19	2710426	2710774	1.19	6.34E-01	GNG7	-7854	DIRAS1	10790
chr12	68024553	68024818	0.89	6.34E-01	DYRK2	-17826	CAND1	361625
chr1	119683223	119683524	1.29	6.34E-01	WARS2	-79		
chr10	43278064	43278319	0.94	6.34E-01	BMS1	238		
chr2	209118984	209119389	0.93	6.34E-01	IDH1	619		
chr3	187465839	187466149	1.26	6.34E-01	BCL6	-2519		
chr5	32585511	32585833	0.94	6.34E-01	SUB1	67		
chr5	154237732	154238207	0.89	6.34E-01	CNOT8	-229		
chr8	77912186	77912541	0.90	6.34E-01	PXMP3	160		
chr9	37904179	37904507	1.18	6.34E-01	MCART1	7		
chr14	24768879	24769138	1.10	6.34E-01	C14orf21	-89	DHRS1	30
chr2	20795277	20795530	1.16	6.34E-01	HS1BP3	55460	RHOB	148569
chr4	26828351	26828811	1.25	6.34E-01	STIM2	-33783	TBC1D19	243035
chr7	30278614	30279132	0.85	6.34E-01	ZNRF2	-45050	PLEKHA8	210585
chr7	100485259	100485504	0.92	6.34E-01	UFSP1	1957	SRRT	12681
chr20	2644759	2645013	1.16	6.34E-01	IDH3B	-43		
chr6	28912245	28912505	1.10	6.34E-01	TRIM27	-20607	ZNF311	60660
chr17	56409710	56410298	1.26	6.34E-01	BZRAP1	-3852		
chr1	161195337	161195787	1.38	6.35E-01	APOA2	-2144	TOMM40L	-271
chr19	36505007	36505308	0.82	6.35E-01	ALKBH6	-17		
chr3	58340960	58341272	0.96	6.35E-01	PXK	22499	PDHB	78449
chr11	59051867	59052291	0.83	6.35E-01	OR5AN1	-79853	MPEG1	-71585
chr17	62002921	62003359	0.79	6.36E-01	GH1	-6942	CD79B	6564
chr12	27090814	27091221	1.14	6.36E-01	FGFR1OP2	-298	C12orf11	236
chr19	11071318	11071777	1.08	6.36E-01	SMARCA4	-50		
chr3	194207605	194208063	1.18	6.36E-01	ATP13A3	-18866	TMEM44	146279
chr6	170615584	170615957	1.05	6.37E-01	FAM120B	-73		
chr10	111672090	111672400	0.91	6.37E-01	XPNPEP1	10957		
chr7	107384432	107384741	1.05	6.37E-01	CBLL1	308		
chr1	26690350	26690612	0.87	6.37E-01	AIM1L	-20038	ZNF683	8785
chr1	27191384	27191754	1.17	6.37E-01	SFN	1936	GPN2	25300

chr1	198590581	198590992	1.03	6.37E-01	ATP6V1G3	-80712	PTPRC	-17350
chr21	44081894	44082158	1.10	6.37E-01	PDE9A	8164	WDR4	217652
chr11	62366677	62366987	1.01	6.38E-01	TUT1	-7723	MTA2	2471
chr22	31795270	31795750	1.11	6.38E-01	DRG1	-29		
chr2	201729280	201729606	1.20	6.38E-01	CLK1	24		
chr17	56421663	56422032	1.23	6.38E-01	BZRAP1	-15696	SUPT4H1	7715
chr2	54013994	54014296	1.14	6.38E-01	ERLEC1	77		
chr20	43594913	43595253	1.15	6.38E-01	STK4	-37		
chr1	203296387	203296772	0.89	6.38E-01	BTG2	21916	FMOD	23709
chr2	233989498	233989808	0.83	6.38E-01	ATG16L1	-170564	INPP5D	64617
chr3	47517328	47517728	1.13	6.38E-01	SCAP	-83		
chr3	58291726	58292110	1.14	6.38E-01	RPP14	-56	Rpp14	-56
chr5	180258521	180258849	1.01	6.39E-01	MGAT1	-28637	ZFP62	28999
chr10	14701719	14702275	0.95	6.39E-01	FRMD4A	-329131	CDNF	177986
chr11	128604268	128604639	1.18	6.39E-01	FLI1	40641	KCNJ1	107909
chr15	65426033	65426343	1.25	6.39E-01	PDCD7	-14		
chr6	28317583	28317866	0.99	6.39E-01	ZKSCAN3	34		
chr8	104427293	104427714	0.95	6.39E-01	SLC25A32	-36	DCAF13	562
chr5	36242074	36242602	0.87	6.39E-01	C5orf33	-438		
chr3	113464011	113464416	1.30	6.40E-01	ATP6V1A	-1694	NAT13	882
chr19	38085699	38086183	1.01	6.40E-01	ZNF571	-268		
chr6	17707067	17707418	1.15	6.40E-01	NUP153	-425		
chr14	77279173	77279456	0.88	6.40E-01	C14orf166B	-13410	VASH1	51080
chr4	48343112	48343511	0.81	6.40E-01	SLC10A4	-142048	TEC	-71498
chr12	57030574	57030939	0.84	6.40E-01	BAZ2A	-594		
chr19	17420079	17420418	1.10	6.40E-01	MRPL34	3772	ANO8	25389
chr1	1510153	1510413	1.01	6.40E-01	SSU72	-21		
chr11	64808315	64808676	0.84	6.40E-01	SAC3D1	120		
chr17	57184181	57184499	1.12	6.41E-01	TRIM37	-74		
chr19	2648513	2648919	1.17	6.41E-01	GNG7	54030	GADD45B	172581
chr11	9482440	9482821	0.84	6.41E-01	ZNF143	118		
chr17	41399851	41400501	0.66	6.41E-01	ARL4D	-76177	TMEM106A	36282
chr17	21030070	21030536	1.14	6.41E-01	DHRS7B	45		
chr1	175178301	175178693	1.24	6.41E-01	TNN	141503	TNR	534255
chr19	10362380	10362699	0.81	6.42E-01	MRPL4	-100		
chr15	89089818	89090140	1.14	6.42E-01	DET1	-67		
chr18	48346297	48346632	0.93	6.42E-01	MRO	-31		
chr21	45209073	45209539	1.16	6.42E-01	RRP1	-112		

chr3	195808710	195809150	1.17	6.42E-01	TFRC	102		
chr6	44214380	44215001	0.82	6.42E-01	HSP90AB1	-158		
chr12	31881737	31882302	1.05	6.42E-01	H3F3C	63155	C12orf72	69411
chr13	53542311	53542555	0.83	6.42E-01	PCDH8	-119659	OLFM4	-60539
chr19	45287575	45287892	0.88	6.42E-01	BCAM	-24604	CBLC	6608
chr2	197036162	197036455	1.05	6.43E-01	STK17B	27		
chr20	32077535	32077802	1.21	6.43E-01	CBFA2T2	-259		
chr14	20929676	20930111	1.11	6.43E-01	TMEM55B	-257		
chr9	33024967	33025245	0.85	6.43E-01	DNAJA1	-103		
chr9	123964096	123964515	0.82	6.43E-01	RAB14	59		
chr20	39591491	39591951	1.03	6.43E-01	MAFB	-273845	TOP1	-65741
chr8	30488529	30488778	0.83	6.44E-01	GTF2E2	27084	RBPMS	246710
chr5	43064892	43065285	0.81	6.44E-01	ZNF131	-56553	C5orf39	-24642
chr2	176032631	176033174	1.18	6.45E-01	ATF2	-6		
chr8	146012679	146012943	1.22	6.45E-01	ZNF34	-86		
chr1	212967875	212968296	1.16	6.45E-01	NSL1	-2947	TATDN3	2916
chr11	58347057	58347301	1.23	6.45E-01	LPXN	-3789	ZFP91	592
chr3	195641855	195642178	1.20	6.45E-01	TNK2	-19585	SDHALP1	75133
chr12	50505517	50505823	1.10	6.45E-01	C12orf62	-230		
chr19	19496403	19496713	0.86	6.45E-01	GATAD2A	-84		
chr16	88772712	88773002	1.05	6.45E-01	RNF166	-57	CTU2	-34
chr14	102783292	102783560	0.87	6.46E-01	ZNF839	-2670		
chr11	65274201	65274693	1.33	6.46E-01	SCYL1	-18101	FRMD8	120406
chr12	93389419	93389718	1.19	6.46E-01	NUDT4	-382132	EEA1	-66462
chr14	68746562	68746960	1.28	6.46E-01	RAD51L1	460252	ZFP36L1	513024
chr15	91477554	91477831	0.89	6.46E-01	HDDC3	-1919	UNC45A	-522
chr7	23221445	23221755	0.84	6.46E-01	NUPL2	154		
chr1	109655327	109655592	0.94	6.46E-01	KIAA1324	-1073		
chr5	96204652	96204969	1.24	6.46E-01	ERAP1	-60919	ERAP2	-6833
chr7	86781555	86781860	0.85	6.47E-01	DMTF1	31		
chr16	81772476	81772820	0.98	6.47E-01	PLCG2	-40282	CMIP	293873
chr1	209957729	209958073	1.17	6.47E-01	IRF6	21578	TRAF3IP3	28377
chr14	42898135	42898495	1.40	6.47E-01	LRFN5	821551		
chr14	71786714	71787088	1.24	6.47E-01	SIPA1L1	-209128	PCNX	412779
chr15	68347719	68348011	0.89	6.47E-01	PIAS1	1293	CALML4	150583
chr7	50187923	50188371	0.97	6.47E-01	IKZF1	-156231	ZPBP	-55287
chrX	12974279	12974653	1.41	6.47E-01	TMSB4X	-18759	TLR8	49708
chr19	42970025	42970282	0.85	6.47E-01	CXCL17	-23018	CEACAM1	62485

chr6	90067135	90067479	0.78	6.47E-01	UBE2J1	-4688		
chr10	5847683	5847996	1.20	6.47E-01	ASB13	-139282	GDI2	7672
chr12	125382973	125383284	0.92	6.47E-01	SCARB1	-34610	UBC	16448
chr12	82752087	82752627	1.19	6.47E-01	CCDC59	-158		
chr18	46455183	46455506	1.08	6.48E-01	SMAD7	21736	KIAA0427	389918
chr1	16970900	16971430	1.12	6.48E-01	NBPF1	-31183	MSTP9	119810
chr11	43702014	43702373	0.98	6.48E-01	HSD17B12	51		
chr6	64308539	64308821	1.17	6.49E-01	PHF3	-47752	PTP4A1	26760
chr3	187463162	187463620	0.81	6.49E-01	BCL6	84		
chr6	43737258	43737558	0.99	6.49E-01	VEGFA	-545		
chr12	49412344	49412726	0.80	6.49E-01	PRKAG1	57		
chr17	18684349	18684659	1.13	6.49E-01	FAM18B	-78		
chr15	49913004	49913359	1.08	6.49E-01	FGF7	197807	ATP8B4	498237
chr1	228678210	228678550	1.00	6.50E-01	RHOU	-192489	HIST3H2BB	32572
chr1	27247887	27248170	1.05	6.50E-01	NUDC	-195		
chr5	130500707	130501132	1.12	6.50E-01	HINT1	114		
chr5	133747513	133747764	0.95	6.50E-01	PHF15	-114159	UBE2B	40769
chr17	1945219	1945464	1.05	6.50E-01	OVCA2	65		
chr17	80309724	80310034	0.95	6.51E-01	SECTM1	-17958	TEX19	-7245
chr11	118722732	118723104	1.04	6.51E-01	DDX6	-60946	CXCR5	-31623
chr12	126257630	126257940	1.23	6.51E-01	TMEM132B	446623		
chr17	45144774	45145100	0.90	6.51E-01	RPRML	-88323	CDC27	121728
chr5	14145216	14145473	0.87	6.51E-01	TRIO	1516	ANKH	726542
chr7	93679431	93679753	0.99	6.51E-01	COL1A2	-344281	BET1	-45902
chr12	32635171	32635540	1.16	6.51E-01	FGD4	-19685	BICD1	375171
chr19	45982266	45982635	0.94	6.51E-01	FOSB	11198	RTN2	17862
chr22	39077785	39078030	1.22	6.52E-01	TOMM22	-46		
chr7	47709129	47709405	0.80	6.52E-01	TNS3	-130068	C7orf69	-125622
chr3	127842635	127843044	1.28	6.52E-01	RUVBL1	-169		
chr19	36231821	36232219	1.14	6.52E-01	PSENEN	-4474	TMEM149	1500
chr7	23338748	23339084	1.03	6.52E-01	GPNMB	52600	IGF2BP3	171079
chr15	68132099	68132518	1.46	6.52E-01	PIAS1	-214263	LBXCOR1	14368
chr19	17622097	17622519	1.23	6.52E-01	PGLS	-124		
chr14	102275904	102276254	1.11	6.53E-01	DYNC1H1	-154786	PPP2R5C	47944
chr22	50628938	50629429	0.81	6.53E-01	PANX2	20024	TUBGCP6	54216
chr12	92539432	92539918	1.08	6.53E-01	BTG1	-2		
chr19	6424848	6425147	1.06	6.53E-01	KHSRP	-176		
chr4	178230576	178231060	1.32	6.53E-01	NEIL3	-173		

chr20	50077393	50077738	1.16	6.53E-01	KCNG1	-437891	NFATC2	81692		
chr1	21619599	21619936	0.95	6.53E-01	ECE1	-3002				
chr17	7142966	7143308	1.15	6.53E-01	PHF23	-312				
chr17	27181761	27182167	1.02	6.53E-01	ERAL1	-79				
chr11	95448839	95449258	1.18	6.53E-01	SESN3	-484803	CEP57	-74593		
chr11	134098540	134098807	1.26	6.53E-01	NCAPD3	-4248	VPS26B	4113		
chr5	158533868	158534144	1.14	6.53E-01	EBF1	-7218	RNF145	100828		
chr5	74062855	74063155	1.14	6.54E-01	NSA2	-98	GFM2	37		
chr6	106533940	106534314	0.81	6.54E-01	PRDM1	-68				
chr1	27147313	27147569	0.84	6.54E-01	ZDHHC18	-5760	PIGV	32955		
chr10	112601579	112601911	1.18	6.54E-01	PDCD4	-29851	RBM20	197590		
chr12	121960772	121961069	1.07	6.54E-01	KDM2B	57999	RNF34	123019		
chr11	87063790	87064139	1.12	6.55E-01	TMEM135	314900	RAB38	844634		
chr22	36553159	36553500	0.91	6.55E-01	RBM9	-128745	APOL3	3647		
chr1	28099409	28099756	1.09	6.55E-01	STX12	-111				
chr16	717903	718180	1.13	6.55E-01	RHOT2	-91				
chr11	65728917	65729350	1.20	6.55E-01	SART1	-26				
chr16	58663595	58664063	0.86	6.55E-01	CNOT1	-79				
chr17	73851199	73851921	1.22	6.55E-01	UNC13D	-10762	TRIM47	23096		
chr19	37958273	37958583	1.20	6.56E-01	ZNF570	-1554	ZNF569	-89		
chr8	56843131	56843416	0.87	6.56E-01	LYN	50888	RPS20	143866		
chr4	153771557	153771818	1.33	6.56E-01	FHDC1	-92447	ARFIP1	70576		
chr15	44116820	44117106	1.22	6.56E-01	MFAP1	-12				
chr16	87843916	87844469	1.21	6.56E-01	KLHDC4	-44651	SLC7A5	58907		
chr4	186508582	186508968	1.17	6.56E-01	PDLIM3	-52063	SORBS2	97225		
chr6	26199225	26200052	0.87	6.56E-01	HIST1H3D	-175	HIST1H2AD	-168	HIST1H2BF	-148
chr11	47921416	47921765	1.28	6.57E-01	PTPRJ	-80519	NUP160	-51534		
chr9	37465649	37465912	1.04	6.57E-01	ZBTB5	-374				
chr4	1722834	1723348	1.03	6.58E-01	TACC3	-175	TMEM129	-7		
chr12	121566901	121567236	1.15	6.58E-01	P2RX7	-3609				
chr15	88993385	88993741	1.13	6.58E-01	NTRK3	-193902	MRPL46	17070		
chr2	3699687	3699997	0.91	6.58E-01	ALLC	-5944	COLEC11	57205		
chr2	71357560	71357843	0.93	6.58E-01	MCEE	-308	MPHOSPH10	258		
chr7	99097816	99098084	0.81	6.58E-01	ZKSCAN5	-4323	ZNF394	-73		
chr11	64795077	64795352	0.96	6.58E-01	SNX15	425				
chr13	48611732	48612388	1.22	6.58E-01	NUDT15	357				
chr17	31204166	31204609	0.82	6.59E-01	MYO1D	-486				
chr4	40057160	40057470	1.09	6.59E-01	N4BP2	-1209				

chr9	117135252	117135640	1.31	6.59E-01	AKNA	21239	ORM1	50143		
chr5	81047105	81047461	1.10	6.59E-01	SSBP2	-211				
chr14	96968571	96968935	0.94	6.59E-01	PAPOLA	33				
chr4	68411019	68411463	1.20	6.59E-01	CENPC1	15				
chr1	179851720	179852036	1.21	6.59E-01	TOR1AIP2	-4944	TOR1AIP1	458		
chrX	73163957	73164233	1.20	6.59E-01	ZCCHC13	-359930	CHIC1	381111		
chr13	26796271	26796604	1.06	6.60E-01	RNF6	-460				
chr16	1755894	1756170	1.13	6.60E-01	MAPK8IP3	-189				
chr11	8710322	8710655	0.96	6.60E-01	RPL27A	6494	ST5	121735		
chr6	26250571	26250912	1.10	6.60E-01	HIST1H4G	-3537	HIST1H2BH	-1137	HIST1H3F	93
chr17	61627397	61627805	1.16	6.60E-01	DCAF7	-221				
chr19	8570597	8570885	0.88	6.60E-01	PRAM1	-3246				
chr19	10679662	10680033	0.78	6.60E-01	CDKN2D	-217				
chr3	123303909	123304433	0.84	6.60E-01	PTPLB	-247				
chr7	4681386	4681882	0.89	6.60E-01	FOXK1	-40296				
chr10	88699173	88699483	0.72	6.60E-01	MMRN2	18097	BMPR1A	182932		
chr14	89897164	89897607	1.33	6.60E-01	FOXN3	188108	TTC8	606468		
chr9	93593407	93593730	1.16	6.60E-01	SYK	29557	AUH	530637		
chr1	206785681	206786022	1.15	6.60E-01	LGTN	52				
chr1	235667845	235668155	1.07	6.60E-01	B3GALNT2	-219				
chr12	50135252	50135639	1.05	6.60E-01	TMBIM6	-146				
chr17	29288197	29288550	1.30	6.60E-01	RNF135	-9582	ADAP2	39620		
chr4	53588371	53588895	1.18	6.60E-01	USP46	-63131	LP9056	29149		
chr15	75746094	75746467	1.11	6.61E-01	SIN3A	-2355				
chr2	89053754	89054130	1.08	6.61E-01	O1/O11 and JK2	-836620	RPIA	62766		
chr1	193506858	193507113	1.09	6.61E-01	B3GALT2	-351243				
chr6	91296867	91297196	0.86	6.61E-01	MAP3K7	-125				
chr17	32688544	32688859	1.13	6.61E-01	CCL1	1550	CCL13	5231		
chr3	178684871	178685247	0.86	6.61E-01	ZMAT3	104525	KCNMB2	430835		
chr7	139024827	139025559	1.18	6.61E-01	LUC7L2	-19441	TTC26	206703		
chr18	3246074	3246384	1.06	6.61E-01	MYL12A	-1299				
chr2	196933081	196933327	1.16	6.61E-01	DNAH7	332				
chr14	50552282	50552666	1.01	6.61E-01	SOS2	145625	ARF6	192738		
chr5	68665332	68665743	0.90	6.61E-01	RAD17	-1743	TAF9	-129		
chr3	152879819	152880059	0.71	6.63E-01	RAP2B	-90				
chr8	144699675	144699962	1.01	6.63E-01	TSTA3	-87				
chr16	58370646	58370956	0.86	6.63E-01	GINS3	-55497	KLKBL4	-41850		
chr17	74478023	74478270	0.85	6.63E-01	AANAT	14496	RHBDF2	19361		

chr5	10607240	10607596	1.25	6.63E-01	DAP	153969	ROPN1L	165409
chr20	2281831	2282188	0.93	6.63E-01	TGM6	-79544	TGM3	5397
chr2	219081511	219081905	1.01	6.63E-01	ARPC2	-166		
chr18	44238169	44238549	0.98	6.63E-01	LOXHD1	-1363		
chr18	47833926	47834310	0.76	6.63E-01	SKA1	-67274	CXXC1	-19426
chr3	154042198	154042466	0.93	6.64E-01	DHX36	-46		
chr2	43446639	43446987	0.80	6.64E-01	HAAO	-427062	ZFP36L2	6932
chr14	24610729	24611124	0.82	6.64E-01	PSME2	4928	PSME1	5549
chr1	203238232	203238500	0.94	6.65E-01	CHIT1	-39506	BTG2	-36298
chr13	26760541	26760899	1.06	6.65E-01	SHISA2	-135522	RNF6	35258
chr6	36634925	36635285	1.13	6.65E-01	CDKN1A	-11354	SFRS3	73015
chr12	19592318	19592606	1.10	6.65E-01	AEBP2	-146		
chr14	62161733	62162170	1.11	6.65E-01	HIF1A	-167		
chr2	27632445	27632895	1.15	6.65E-01	PPM1G	-174		
chr17	35969342	35969723	1.11	6.65E-01	SYNRG	-47		
chrX	84258740	84258998	1.12	6.65E-01	APOOL	-36		
chr1	17231520	17232001	1.22	6.65E-01	MSTP9	-140786	CROCC	-16684
chr12	111021012	111021398	0.97	6.66E-01	PPTC7	-141		
chr10	5671887	5672131	0.80	6.66E-01	ASB13	36549	CALML3	105085
chr1	55181368	55181622	0.83	6.66E-01	TTC4	-34		
chr12	113512867	113513518	1.14	6.66E-01	DTX1	17531	RASAL1	60828
chr19	18402416	18402826	1.03	6.66E-01	JUND	-10189	LSM4	31380
chr6	16322952	16323307	1.07	6.67E-01	GMPR	84319	ATXN1	438591
chr12	56709647	56710265	1.15	6.67E-01	CNPY2	164		
chr6	26553593	26553903	1.00	6.67E-01	ABT1	-43432	HMGN4	15176
chr7	75807478	75807740	0.85	6.67E-01	HSPB1	-124266	MDH2	130216
chr19	10362770	10363015	1.07	6.67E-01	MRPL4	253		
chr19	49339043	49339381	1.13	6.67E-01	HSD17B14	722		
chr12	31478920	31479230	1.01	6.68E-01	OVOS1	-119987	DENND5B	264877
chr2	68478220	68478498	0.90	6.68E-01	PPP3R1	1292	PNO1	93354
chr17	40474836	40475146	0.91	6.68E-01	STAT5A	35426	STAT3	65522
chr19	3987809	3988058	0.92	6.68E-01	EEF2	-2473		
chr9	34048892	34049735	1.14	6.69E-01	DCAF12	77457	UBE2R2	232132
chr11	67084926	67085410	1.15	6.69E-01	SSH3	14249	POLD4	35849
chr4	154265657	154266003	1.17	6.69E-01	MND1	29		
chr9	36008813	36009058	0.75	6.69E-01	OR2S2	-50785	RECK	-27974
chr5	175815817	175816239	1.24	6.69E-01	NOP16	-488	HIGD2A	244
chr11	2554029	2554310	0.91	6.69E-01	KCNQ1	87949	CDKN1C	352825

chr19	41945632	41946053	1.22	6.69E-01	ATP5SL	-33		
chr19	2391324	2391611	0.84	6.69E-01	TMPRSS9	1684	TIMM13	36407
chr10	102820673	102821013	1.21	6.69E-01	KAZALD1	-156		
chr20	45985376	45985645	1.11	6.69E-01	ZMYND8	-37		
chr6	44140497	44140758	0.88	6.69E-01	SLC29A1	-50668	CAPN11	14080
chr9	117147745	117148079	1.14	6.69E-01	AKNA	8773	ORM1	62609
chr19	41304742	41305157	1.06	6.70E-01	EGLN2	-1231		
chr12	860338	860720	0.89	6.70E-01	WNK1	-1696		
chr2	104695037	104695331	0.79	6.70E-01	POU3F3	-776785		
chr9	128003411	128003952	1.06	6.70E-01	HSPA5	-21		
chr19	46220553	46221046	1.07	6.70E-01	FBXO46	13351	QPCTL	25059
chr6	26537518	26537885	0.96	6.70E-01	HMGN4	-870		
chr18	21599606	21599882	1.31	6.70E-01	CABYR	-119211	LAMA3	330182
chr19	6536095	6536405	0.83	6.70E-01	TNFSF9	5240	CD70	54913
chr19	1244471	1244707	1.13	6.70E-01	MIDN	-3963		
chr16	15736297	15736611	0.99	6.70E-01	KIAA0430	555		
chr3	150264155	150264597	1.24	6.70E-01	EIF2A	-198	SERP1	588
chr1	95538299	95538696	1.08	6.70E-01	ALG14	9		
chr15	70272127	70272530	1.18	6.72E-01	TLE3	117927	RPLP1	527170
chr14	67826750	67827258	1.21	6.72E-01	ATP6V1D	-413	EIF2S1	-30
chr16	85942172	85942490	1.24	6.72E-01	FOXF1	-601802	IRF8	9557
chr8	141522006	141522391	1.16	6.72E-01	CHRAC1	798		
chr11	72504615	72505133	1.14	6.73E-01	ARAP1	-41440	ATG16L2	-20577
chr15	91475669	91475984	0.93	6.73E-01	UNC45A	-2388	HDDC3	-53
chr6	52589282	52589604	0.93	6.73E-01	GSTA2	38918	TMEM14A	53559
chr15	45694369	45694872	1.21	6.73E-01	SPATA5L1	102		
chr17	7482727	7483129	1.17	6.73E-01	MPDU1	-4037	CD68	123
chr7	99746409	99746673	0.93	6.73E-01	GAL3ST4	19832	MBLAC1	22221
chr7	130581766	130582036	1.35	6.73E-01	MKLN1	-430694	KLF14	-163041
chr15	59705317	59705627	1.16	6.73E-01	GCNT3	-198510	MYO1E	-40401
chr7	30324100	30324501	1.01	6.73E-01	ZNRF2	378		
chr16	58790315	58790625	0.84	6.73E-01	GOT2	-22224		
chr17	5095161	5095489	0.84	6.73E-01	ZNF594	-147		
chr12	32426757	32427056	0.79	6.73E-01	FGD4	-228134	BICD1	166722
chr2	219122341	219122646	1.19	6.74E-01	GPBAR1	-3244		
chrX	24072638	24073055	1.15	6.74E-01	EIF2S3	-218		
chr16	67840282	67840703	0.98	6.74E-01	TSNAXIP1	-517	RANBP10	62
chr17	20937703	20938068	1.07	6.74E-01	LGALS9B	-567038	USP22	8466

chr2	38735520	38735802	1.26	6.74E-01	ATL2	-131229	HNRPLL	94517
chr6	15691525	15691847	0.85	6.74E-01	MYLIP	-437631	DTNBP1	-28415
chr13	50510478	50510811	1.17	6.75E-01	KPNA3	-143588	TRIM13	-60498
chr9	26892601	26892967	0.90	6.75E-01	PLAA	54684		
chr11	65171057	65171324	1.19	6.75E-01	SCYL1	-121357	FRMD8	17150
chr1	8491109	8491419	0.88	6.75E-01	SLC45A1	106874	RERE	386435
chr12	125425239	125425566	0.75	6.75E-01	UBC	-25826	DHX37	48264
chr22	23238798	23239264	0.95	6.75E-01	GNAZ	-173638	IGL@	9071
chr3	143690723	143691081	1.05	6.76E-01	C3orf58	262		
chr5	121297333	121297760	1.20	6.76E-01	SRFBP1	-109		
chr21	43648283	43648695	0.93	6.76E-01	ABCG1	9222	TFF3	87217
chr1	36554280	36554587	1.12	6.76E-01	ADPRHL2	-19		
chr17	4870640	4871112	0.89	6.76E-01	SPAG7	256		
chr7	32529774	32530241	0.83	6.76E-01	LSM5	15		
chr5	138724228	138724547	0.80	6.76E-01	SLC23A1	-5349	MGC29506	1217
chr15	69113231	69113611	0.72	6.77E-01	ANP32A	-160		
chr11	47270261	47270570	1.15	6.77E-01	ACP2	41		
chr9	272798	273152	0.98	6.77E-01	KANK1	-231728	DOCK8	58110
chr11	9636749	9637059	1.01	6.78E-01	SWAP70	-48724	WEE1	41676
chr6	88635389	88635692	1.13	6.78E-01	AKIRIN2	-223556	SPACA1	-121966
chr1	1447195	1447519	1.10	6.78E-01	ATAD3A	-198		
chr11	925591	925939	0.87	6.78E-01	AP2A2	-76		
chr1	54955092	54955355	0.73	6.78E-01	SSBP3	-83132	ACOT11	-58677
chr12	69684837	69685116	0.96	6.79E-01	LYZ	-57157	CPSF6	51660
chr2	9695830	9696170	1.01	6.79E-01	ADAM17	-83		
chr17	40831775	40832077	1.18	6.79E-01	PLEKHH3	-2878	CNTNAP1	-2706
chr19	1266761	1267225	0.75	6.79E-01	CIRBP	-2274		
chr6	153323854	153324210	1.08	6.79E-01	MTRF1L	-107		
chr17	74488578	74488908	1.03	6.79E-01	RHBDF2	8765	AANAT	25092
chr16	70323222	70323536	1.01	6.79E-01	AARS	33		
chr8	27950488	27951031	1.27	6.79E-01	ELP3	176		
chr19	10870222	10870736	1.15	6.79E-01	DNM2	41724	TMED1	76504
chr1	154244686	154245247	1.13	6.80E-01	HAX1	-72		
chr19	36390590	36390900	0.96	6.80E-01	HCST	-2637	NFKBID	807
chr19	15543495	15543818	1.01	6.80E-01	AKAP8L	-13824	WIZ	17105
chr13	45151834	45152312	0.94	6.80E-01	TSC22D1	-1372		
chr6	24403000	24403265	0.86	6.80E-01	MRS2	-20		
chr1	203264946	203265256	0.80	6.81E-01	CHIT1	-66241	BTG2	-9563

chr12	32908689	32909044	1.08	6.81E-01	YARS2	7		
chr4	148538443	148538772	0.88	6.81E-01	TMEM184C	69		
chr5	76326058	76326341	0.93	6.81E-01	AGGF1	-32		
chr1	167772581	167772870	0.89	6.81E-01	MPZL1	81539	ADCY10	110727
chr5	1801294	1801551	0.84	6.81E-01	MRPL36	-1467	NDUFS6	-86
chr2	86790467	86790748	0.98	6.81E-01	RNF103	60370	KDM3A	122024
chr8	119294186	119294454	1.33	6.81E-01	EXT1	-170262	TNFRSF11B	670063
chr10	105677800	105678108	1.13	6.81E-01	OBFC1	91		
chr9	131486489	131486864	1.07	6.82E-01	ZDHHC12	-269		
chr3	33839943	33840298	1.25	6.82E-01	PDCD6IP	55		
chr2	101618604	101618859	1.25	6.82E-01	RPL31	41		
chr15	101791989	101792443	1.08	6.82E-01	CHSY1	-90		
chr15	78591847	78592100	1.12	6.82E-01	CRABP1	-40692	DNAJA4	35487
chr17	62151727	62152067	1.27	6.82E-01	ICAM2	-53903	ERN1	55605
chr19	10981964	10982242	1.00	6.83E-01	CARM1	-150		
chr1	202317569	202317923	1.14	6.83E-01	PPP1R12B	-90		
chr14	61201305	61201615	0.79	6.83E-01	MNAT1	-10		
chr22	42228835	42229304	1.06	6.84E-01	SREBF2	-36		
chr6	26312696	26312948	1.15	6.84E-01	BTN3A2	-52576	HIST1H4H	-27095
chr5	176881532	176881842	1.12	6.84E-01	PRR7	7891	DBN1	19007
chr8	33412379	33412711	0.82	6.84E-01	RNF122	12098	MAK16	69860
chrX	1587266	1587604	1.08	6.84E-01	ASMTL	-15591	P2RY8	68602
chr7	101500105	101500433	0.86	6.84E-01	SH2B2	-428136	CUX1	39387
chr17	4316532	4316808	1.11	6.84E-01	UBE2G1	-46701	SPNS3	-20549
chr16	2021805	2022152	1.21	6.84E-01	TBL3	-85		
chr15	83419065	83419623	1.23	6.84E-01	WHAMM	-58629	AP3B2	-40709
chr10	17272511	17272896	0.88	6.85E-01	VIM	2446	ST8SIA6	223550
chr14	75469181	75469779	0.91	6.85E-01	EIF2B2	-132		
chr5	140904377	140904713	0.82	6.85E-01	PCDHGC5	35737	DIAPH1	94077
chr9	115555510	115555820	1.10	6.85E-01	SNX30	42531	SLC46A2	97528
chr4	26322181	26322457	0.95	6.86E-01	RBPJ	-129		
chr19	51014059	51014922	1.10	6.86E-01	ASPDH	2651	C19orf63	34755
chr2	214022063	214022336	1.02	6.86E-01	SPAG16	-126916	IKZF2	-7142
chr6	2849144	2849462	0.93	6.86E-01	SERPINB1	-7222	SERPINB9	54242
chr20	32900503	32900850	1.10	6.86E-01	ITCH	-50385	AHCY	-9462
chr11	59521485	59521924	1.09	6.86E-01	STX3	-1184		
chr10	61996741	61997017	0.79	6.86E-01	CCDC6	-330061	ANK3	152609
chr13	25258063	25258318	1.26	6.86E-01	RNF17	-80110	ATP12A	3496

chr17	79212940	79213287	1.05	6.87E-01	C17orf56	-259	C17orf89	3
chr15	62352580	62353087	1.06	6.87E-01	VPS13C	-187		
chr20	34287166	34287432	0.78	6.87E-01	NFS1	-25	ROMO1	67
chr1	20208706	20209068	1.14	6.87E-01	RNF186	-67116	PLA2G2E	41223
chr14	77495045	77495372	1.11	6.87E-01	C14orf4	-175		
chr11	62413965	62414209	1.10	6.87E-01	GANAB	17		
chr17	42147902	42148231	1.18	6.87E-01	LSM12	-3080	G6PC3	-51
chr8	124169374	124169813	0.83	6.87E-01	C8orf76	84023	WDR67	84674
chr12	123011451	123011831	1.15	6.87E-01	KNTC1	-168		
chr3	131221653	131221988	1.26	6.87E-01	MRPL3	8		
chr18	30812057	30812365	0.98	6.87E-01	KLHL14	-459237	ASXL3	-346330
chr12	111351232	111351549	1.05	6.88E-01	PPP1CC	-170634	MYL2	7013
chr1	93427245	93427544	0.77	6.88E-01	FAM69A	-316		
chr12	6579627	6580186	0.79	6.88E-01	VAMP1	-64		
chr2	27851712	27852060	1.07	6.88E-01	GPN1	23		
chr8	101964222	101964466	0.97	6.88E-01	YWHAZ	877		
chr12	122519734	122520044	0.86	6.88E-01	MLXIP	3129	IL31	138857
chr22	34238464	34238840	0.98	6.88E-01	SYN3	-835843	LARGE	77764
chr11	95523489	95523904	1.13	6.88E-01	CEP57	55		
chr5	78023931	78024221	1.15	6.88E-01	LHFPL2	-79428	ARSB	258281
chr7	72349708	72349993	0.86	6.88E-01	TYW1B	-51038	POM121	-45809
chr11	9622974	9623284	0.97	6.88E-01	SWAP70	-62499	WEE1	27901
chr10	26624136	26624507	0.85	6.88E-01	APBB1IP	-102944	GAD2	119086
chr3	32068058	32068443	0.90	6.88E-01	GPD1L	-79893	ZNF860	44985
chr15	66084449	66085229	1.18	6.89E-01	DENND4A	-208		
chr7	130808973	130809457	0.89	6.89E-01	KLF14	-390355	MKLN1	-203380
chr3	182840168	182840444	1.01	6.89E-01	MCCC1	-22941	LAMP3	40361
chr18	19180541	19181002	0.90	6.90E-01	ESCO1	-79		
chr1	16766963	16767347	0.94	6.90E-01	SPATA21	-3236	NECAP2	-12
chr7	130598211	130598610	0.99	6.90E-01	MKLN1	-414184	KLF14	-179551
chr6	143771708	143772090	0.84	6.90E-01	ADAT2	-58	PEX3	-19
chr1	229643961	229644262	1.03	6.90E-01	NUP133	-24		
chr11	65479259	65479624	1.26	6.90E-01	KAT5	-47		
chr3	12525681	12525967	1.11	6.90E-01	TSEN2	-186		
chr3	50396914	50397257	1.18	6.90E-01	TMEM115	-147		
chr6	86353701	86354064	1.16	6.90E-01	SYNCRIP	-840		
chr8	145550349	145550750	1.01	6.90E-01	DGAT1	17		
chr14	59823606	59823902	1.17	6.90E-01	GPR135	108305	DAAM1	168355

chr3	167453161	167453531	1.12	6.90E-01	PDCD10	-695	SERPINI1	-86
chr5	173019100	173019410	0.98	6.90E-01	STC2	-262749	BOD1	24411
chr6	31939636	31940379	0.88	6.90E-01	DOM3Z	24	STK19	362
chr15	41245359	41245748	1.11	6.90E-01	CHAC1	-82		
chr21	45079281	45079631	1.04	6.90E-01	HSF2BP	-82	RRP1B	24
chr12	53895236	53895646	0.90	6.91E-01	MAP3K12	-2170	TARBP2	64
chr5	118689726	118690016	1.07	6.91E-01	TNFAIP8	-1725		
chr16	22206938	22207324	1.13	6.91E-01	EEF2K	-10461	VWA3A	103268
chr1	17036308	17036629	0.87	6.91E-01	NBPF1	-96487	MSTP9	54506
chr13	41885124	41885598	1.02	6.91E-01	NARG1L	20		
chr4	4249941	4250185	1.21	6.91E-01	TMEM128	-129		
chr11	73498779	73499177	1.16	6.92E-01	MRPL48	61		
chr11	85780192	85780432	0.90	6.92E-01	PICALM	-204		
chr14	94425734	94426076	1.24	6.92E-01	ASB2	-2138		
chr17	65821350	65821690	0.90	6.92E-01	BPTF	-260		
chr19	4791248	4791571	1.09	6.92E-01	FEM1A	-318		
chr19	7745498	7745862	1.05	6.92E-01	TRAPPC5	-27		
chr2	242254662	242255179	0.92	6.92E-01	SEPT2	198		
chr9	33001480	33001856	1.16	6.92E-01	APTX	-42		
chr21	43757995	43758250	1.19	6.92E-01	TFF3	-22417	TFF2	13085
chr16	31470610	31470854	1.08	6.92E-01	ARMC5	415		
chr17	38514744	38515060	0.95	6.92E-01	GJD3	-2005		
chr2	88990943	88991337	1.20	6.92E-01	RPIA	-36		
chr3	195621619	195622090	0.86	6.92E-01	TNK2	577		
chr16	56945768	56946156	1.05	6.92E-01	HERPUD1	-19786	SLC12A3	46843
chr17	21191047	21191377	1.10	6.92E-01	KCNJ12	-88487	MAP2K3	3244
chr5	156698706	156699251	0.77	6.92E-01	CYFIP2	5888	C5orf40	73750
chr7	152161028	152161323	1.04	6.92E-01	MLL3	-28086	XRCC2	212074
chr9	100864263	100864551	1.02	6.92E-01	TRIM14	17081	NANS	45725
chr16	89164844	89165282	0.91	6.93E-01	CDH15	-73100	ACSF3	4809
chr5	157625625	157625959	1.14	6.93E-01	CLINT1	-339624	EBF1	900996
chr15	37307284	37307528	1.10	6.93E-01	MEIS2	85298	C15orf41	435594
chr12	83079519	83079904	0.84	6.93E-01	TMTC2	-1222		
chr6	42847539	42847876	0.95	6.93E-01	RPL7L1	37		
chr12	498333	498612	0.84	6.93E-01	KDM5A	147		
chr1	33446798	33447124	1.25	6.94E-01	RNF19B	-16675	AK2	55531
chr2	68694529	68694770	1.01	6.94E-01	APLF	-41		
chr7	99156076	99156329	0.82	6.94E-01	ZNF655	-245		

chr19	39137999	39138262	1.00	6.94E-01	ACTN4	-196		
chr8	81397640	81398120	1.06	6.94E-01	ZBTB10	-568		
chr9	125590871	125591257	1.04	6.95E-01	PDCL	-224		
chr17	7232879	7233477	0.97	6.95E-01	NEURL4	-540		
chr17	7760464	7761418	1.20	6.95E-01	CYB5D1	-123	LSMD1	231
chr3	12598372	12598631	1.09	6.95E-01	MKRN2	-92		
chr12	6862687	6863049	0.82	6.95E-01	MLF2	-786		
chr14	54976427	54976715	0.89	6.95E-01	CGRRF1	-16		
chr11	1543089	1543361	0.92	6.95E-01	HCCA2	-41110	DUSP8	49925
chr5	177019051	177019300	0.98	6.95E-01	TMED9	-37		
chr1	32110641	32110951	0.80	6.95E-01	PEF1	-319		
chr14	77506655	77507267	1.16	6.95E-01	C14orf4	-11927	ZDHHC22	101173
chr17	76732889	76733219	0.85	6.95E-01	DNAH17	-165650	CYTH1	45322
chr16	68056998	68057308	1.07	6.95E-01	DUS2L	-51	DDX28	806
chr3	56950298	56950633	1.28	6.96E-01	ERC2	-448075	ARHGEF3	162870
chrX	70752609	70753050	1.02	6.96E-01	OGT	-103		
chr1	28562494	28562772	0.86	6.96E-01	DNAJC8	-3091	ATPIF1	22
chr12	2994479	2994781	1.05	6.96E-01	FOXM1	-8327	TULP3	-5403
chr2	75873676	75874030	0.90	6.96E-01	MRPL19	-56		
chr6	31587638	31588344	0.87	6.96E-01	BAT2	-459		
chr9	125675194	125675492	0.84	6.97E-01	ZBTB6	266		
chr11	64085462	64085702	0.98	6.97E-01	TRMT112	-549	PRDX5	13
chr14	81685515	81685906	0.87	6.97E-01	GTF2A1	1583	TSHR	263842
chr12	98897627	98898219	0.87	6.97E-01	AX747640	-47000	TMPO	-11486
chr6	27839747	27840253	0.81	6.97E-01	HIST1H1B	-4641	HIST1H3I	99
chr1	109618550	109618860	0.86	6.98E-01	TAF13	-81		
chr14	24685124	24685488	1.12	6.98E-01	MDP1	-65		
chr1	45241061	45241313	0.83	6.98E-01	RPS8	-59		
chr11	66405671	66406023	1.07	6.98E-01	RBM14	21794	RBM4B	39428
chr12	92860279	92860684	1.04	6.98E-01	BTG1	-320809	PLEKHG7	-269783
chr12	96721214	96721536	0.84	6.98E-01	PCTK2	72848	ELK3	133168
chr7	134001672	134001928	1.10	6.99E-01	SLC35B4	27		
chr19	42900969	42901283	0.98	6.99E-01	CNFN	-6682	LIPE	30452
chr16	23568604	23569071	1.05	6.99E-01	EARS2	-142		
chr17	62223027	62223474	1.14	6.99E-01	ERN1	-15749	TEX2	117402
chr2	241524637	241524915	1.02	6.99E-01	CAPN10	-1369		
chr11	94884605	94884978	1.14	6.99E-01	ENDOD1	61775	SESN3	79454
chr14	48398613	48398858	1.01	6.99E-01	MDGA2	-254748		

chr18	47841897	47842146	0.97	6.99E-01	SKA1	-59370	CXXC1	-27330
chr15	78730111	78730493	1.12	7.00E-01	IREB2	-216		
chr1	38941003	38941290	0.81	7.00E-01	POU3F1	-428697	RRAGC	384193
chr2	135497484	135497868	1.17	7.00E-01	ACMSD	-98510	TMEM163	-21105
chr10	94351312	94351584	0.88	7.00E-01	KIF11	-1377		
chr15	91260343	91260743	1.14	7.00E-01	BLM	-36		
chr18	20808750	20809086	0.90	7.00E-01	CABLES1	93191	C18orf45	209007
chr6	43474500	43474748	1.03	7.00E-01	YIPF3	10078	TJAP1	29363
chr3	136581076	136581377	1.14	7.00E-01	NCK1	154		
chr3	23958455	23958783	1.21	7.00E-01	NKIRAS1	-82	RPL15	-20
chrX	54466629	54466904	1.13	7.00E-01	TSR2	-86		
chr6	11459261	11459571	1.28	7.00E-01	NEDD9	-226501	TMEM170B	-79095
chr12	54718440	54718716	0.85	7.00E-01	COPZ1	-333		
chr1	17222576	17222897	1.21	7.00E-01	MSTP9	-131762	CROCC	-25708
chr14	55493647	55494349	0.88	7.00E-01	WDHD1	-191	SOCS4	154
chr12	110906957	110907267	1.14	7.01E-01	GPN3	-1048		
chr10	113943401	113943645	0.83	7.01E-01	GPAM	2		
chr9	130700071	130700368	1.13	7.01E-01	DPM2	543		
chr19	4769620	4769856	1.03	7.01E-01	DPP9	-45883	FEM1A	-21990
chr9	123655045	123655384	1.17	7.01E-01	PHF19	-15609	TRAF1	33958
chr22	27067235	27067573	1.01	7.02E-01	CRYBA4	49476		
chr16	53164465	53164972	0.90	7.02E-01	RBL2	-303632	CHD9	75774
chr13	67804446	67804787	1.01	7.02E-01	PCDH9	-149		
chr11	65769506	65769933	1.22	7.02E-01	EIF1AD	-142	BANF1	170
chr6	42540240	42540516	1.31	7.02E-01	UBR2	8320	PRPH2	149980
chr1	38156032	38156457	1.12	7.02E-01	CDCA8	-1914		
chr1	167297994	167298524	1.08	7.02E-01	POU2F1	108116	CD247	189588
chr1	115300575	115300820	1.09	7.02E-01	CSDE1	-27		
chr19	10490975	10491490	1.09	7.02E-01	TYK2	15		
chr6	53659265	53659823	0.85	7.03E-01	LRRC1	10		
chr3	197509183	197509458	1.26	7.03E-01	LRCH3	-8824	FYTTD1	32697
chr3	124449040	124449360	1.12	7.03E-01	UMPS	-13		
chr10	12237597	12238040	0.75	7.03E-01	CDC123	-142	NUDT5	324
chr15	66797031	66797601	1.02	7.03E-01	RPL4	-132	ZWILCH	-115
chr1	100731471	100731858	0.95	7.03E-01	RTCD1	-49		
chr4	40196460	40196796	0.97	7.03E-01	RHOH	-1899		
chr4	8442350	8442640	1.09	7.03E-01	C4orf23	-554	ACOX3	-43
chr6	135375878	135376298	0.92	7.03E-01	HBS1L	-52		

chr1	36107051	36107387	0.89	7.03E-01	PSMB2	-76		
chr13	20532610	20533052	0.69	7.04E-01	ZMYM2	21		
chrX	2625514	2625758	1.14	7.04E-01	XG	-44457	CD99	16408
chr19	57831528	57831838	1.09	7.05E-01	ZNF543	-194		
chr1	207096456	207096892	1.12	7.05E-01	FAIM3	-1296		
chr7	140714466	140714948	1.20	7.05E-01	MRPS33	74		
chr10	173853	174294	1.16	7.05E-01	TUBB8	-78896	ZMYND11	-6350
chr10	12306276	12306593	1.02	7.05E-01	CAMK1D	-85148	NUDT5	-68292
chr17	27229199	27229654	0.91	7.05E-01	FLOT2	-4712	DHRS13	662
chr1	32573503	32573760	1.22	7.05E-01	KPNA6	-12		
chr1	151128532	151128872	0.80	7.05E-01	TNFAIP8L2	-403		
chr2	68479668	68480210	1.04	7.05E-01	PPP3R1	-288		
chr18	71815372	71816053	0.94	7.05E-01	FBXO15	-613	C18orf55	-33
chr3	150481729	150482070	0.97	7.05E-01	SIAH2	-637		
chr15	70611879	70612296	0.87	7.06E-01	TLE3	-221832	UACA	443762
chr17	19366499	19366818	1.27	7.06E-01	SLC47A1	-70508	RNF112	52136
chr9	37079584	37079985	1.09	7.06E-01	PAX5	-45309	ZCCHC7	-40684
chr6	151773191	151773592	1.15	7.07E-01	RMND1	-76	C6orf211	-30
chr2	227637490	227637798	1.18	7.07E-01	IRS1	25862		
chr12	133263829	133264248	1.05	7.07E-01	PXMP2	-153	POLE	-94
chr17	8089547	8089830	1.13	7.07E-01	TMEM107	-9975	C17orf59	3875
chr3	122102882	122103188	1.09	7.07E-01	CCDC58	-961	FAM162A	12
chr12	50419233	50419512	1.08	7.07E-01	RACGAP1	-66		
chr16	71879695	71879981	1.09	7.07E-01	ATXN1L	-61		
chr2	105593176	105593565	0.94	7.07E-01	MRPS9	-61112	POU3F3	121402
chr15	41196259	41196499	1.00	7.08E-01	DLL4	-25168	VPS18	9751
chr5	139015819	139016128	0.96	7.08E-01	CXXC5	-12327	UBE2D2	75223
chr16	21610713	21611002	1.17	7.08E-01	SLC7A5P1	-79093	IGSF6	53114
chr14	51706759	51707148	1.10	7.08E-01	TMX1	68		
chr19	39420819	39421750	1.05	7.08E-01	MRPS12	-309	SARS2	251
chr11	47429747	47430018	0.88	7.08E-01	SLC39A13	-168		
chr6	16421053	16421389	1.03	7.08E-01	GMPR	182410	ATXN1	340500
chr6	88639554	88639816	0.94	7.08E-01	AKIRIN2	-227700	SPACA1	-117822
chr1	23852191	23852630	0.83	7.09E-01	ASAP3	-41661	E2F2	5302
chr17	21251986	21252390	0.70	7.09E-01	KCNJ12	-27511	MAP2K3	64220
chr8	101162713	101163041	0.88	7.09E-01	FBXO43	-4801	POLR2K	38
chr19	18391095	18391504	1.06	7.09E-01	KIAA1683	-5981	JUND	1132
chr12	113506190	113506501	0.79	7.09E-01	DTX1	10684	RASAL1	67675

chr12	56109698	56109990	1.23	7.10E-01	RDH5	-4307	BLOC1S1	24
chr12	118810756	118811023	0.95	7.10E-01	SUDS3	-3468	TAOK3	-140
chr17	62971474	62971834	1.09	7.10E-01	LRRC37A3	-56666	GNA13	81266
chr19	4204635	4204891	1.06	7.10E-01	EBI3	-24777	SIRT6	-22167
chr6	52255160	52255491	1.18	7.10E-01	EFHC1	-29668	PAQR8	28400
chr17	47269765	47270398	0.89	7.10E-01	GNGT2	16661	B4GALNT2	59753
chr5	137911105	137911680	0.96	7.10E-01	HSPA9	-278		
chr7	75923979	75924274	0.88	7.10E-01	HSPB1	-7748	MDH2	246734
chr16	28961890	28962333	0.98	7.11E-01	NFATC2IP	-206		
chr12	38710346	38710677	1.17	7.11E-01	ALG10B	-45		
chr9	6758881	6759441	1.05	7.12E-01	KDM4C	1520		
chr16	71598543	71599155	1.17	7.12E-01	TAT	12149	CHST4	38788
chr17	4852639	4853003	1.26	7.12E-01	ENO3	-1565	PFN1	-996
chr5	179720467	179720781	0.88	7.12E-01	MAPK9	-1553		
chr19	44259512	44259879	1.12	7.12E-01	C19orf61	-554		
chr11	72759167	72759470	1.33	7.12E-01	FCHSD2	93824	ATG16L2	233868
chr20	62359980	62360275	1.23	7.12E-01	LIME1	-7005	ZGPAT	20737
chr21	34755538	34755921	0.95	7.12E-01	IFNGR2	-19472	IFNAR1	58516
chr11	75525755	75526152	1.12	7.12E-01	UVRAG	-258		
chr7	98978525	98978961	1.16	7.12E-01	ARPC1B	6414	PDAP1	27549
chr3	46530228	46530574	1.09	7.12E-01	LTF	-24006	RTP3	-9084
chr7	128379163	128379679	0.90	7.12E-01	CALU	75		
chr19	47289548	47290080	1.12	7.12E-01	STRN4	-40094	SLC1A5	2028
chr9	101077869	101078213	1.18	7.12E-01	TBC1D2	-60038	GABBR2	393134
chr2	70313905	70314260	1.06	7.12E-01	PCBP1	-502		
chr11	46958091	46958414	1.16	7.12E-01	LRP4	-18177	ARFGAP2	240166
chr16	30007383	30007693	1.07	7.12E-01	HIRIP3	-151		
chr16	115700	115950	1.15	7.12E-01	RHBDF1	6804	SNRNP25	11996
chr13	52368048	52368442	1.00	7.13E-01	DHRS12	10048	WDFY2	209761
chr11	9482079	9482325	0.93	7.13E-01	ZNF143	-311		
chr10	33269491	33269837	0.87	7.13E-01	ITGB1	-22371	NRP1	354169
chr16	740283	740577	1.14	7.13E-01	STUB1	10315	FBXL16	15378
chr12	129314379	129314761	1.05	7.13E-01	GLT1D1	-23511	SLC15A4	-6029
chr7	22149945	22150194	0.83	7.13E-01	CDCA7L	-164528	RAPGEF5	246463
chr19	37569020	37569475	0.98	7.13E-01	ZNF420	-134		
chr12	98910195	98910500	1.00	7.14E-01	TMPO	939		
chr11	111317284	111317653	1.14	7.14E-01	POU2AF1	-67312	BTG4	65595
chr6	26273077	26273482	0.81	7.14E-01	HIST1H3G	-1668	HIST1H2BI	76

chr15	40407987	40408297	1.05	7.14E-01	BUB1B	-45068	BMF	-7067
chr17	40558133	40558507	1.05	7.14E-01	STAT3	-17807	PTRF	16954
chr4	40994023	40994378	0.61	7.14E-01	APBB2	222434	NSUN7	242287
chr5	133968406	133968718	1.16	7.14E-01	SAR1B	-35		
chr5	153418290	153418691	0.96	7.14E-01	MFAP3	-28	FAM114A2	6
chr2	208490059	208490348	1.13	7.14E-01	FAM119A	-231		
chr12	125391315	125391593	1.08	7.15E-01	SCARB1	-42935	UBC	8123
chr17	8028911	8029212	0.96	7.15E-01	HES7	-1660		
chr12	92566406	92566800	0.94	7.15E-01	PLEKHG7	-563662	BTG1	-26930
chr20	61569143	61569405	1.07	7.15E-01	C20orf11	-167	DIDO1	0
chr8	100253056	100253366	0.84	7.15E-01	VPS13B	227717	COX6C	652684
chr12	123464934	123465341	0.95	7.15E-01	ARL6IP4	258		
chr16	66914139	66914620	0.86	7.15E-01	PDP2	-56		
chr17	66015888	66016356	1.20	7.15E-01	C17orf58	-26357	KPNA2	-15726
chr17	56410408	56410912	1.27	7.15E-01	BZRAP1	-4508		
chr1	205225345	205225589	0.92	7.15E-01	TMCC2	28376	NUAK2	65416
chr3	46580261	46580567	1.25	7.15E-01	LRRC2	27597	RTP3	40929
chr12	110939875	110940235	1.21	7.15E-01	VPS29	-139	RAD9B	50
chr19	18263233	18263597	0.99	7.15E-01	PIK3R2	-601		
chr5	1336198	1336483	1.20	7.15E-01	TERT	-41179	CLPTM1L	8661
chr12	54069810	54070170	1.20	7.16E-01	ATP5G2	119		
chr11	118187374	118187643	0.89	7.16E-01	CD3E	12214	CD3D	25950
chr3	47563644	47563920	1.08	7.16E-01	SCAP	-46337	CSPG5	56577
chr1	22109595	22109858	1.14	7.17E-01	USP48	-39		
chr10	556945	557235	0.92	7.17E-01	DIP2C	178518	ZMYND11	376666
chr8	22831713	22832084	0.80	7.17E-01	PEBP4	-46478	RHOBTB2	-21462
chr2	113403061	113403371	0.76	7.17E-01	SLC20A1	-311		
chr14	70081390	70081666	0.92	7.18E-01	SFRS5	-152306	KIAA0247	3218
chr21	30445974	30446312	1.19	7.18E-01	CCT8	-133		
chr16	1401796	1402055	0.97	7.18E-01	C16orf42	-53	GNPTG	-6
chr6	26521294	26521605	1.03	7.18E-01	HMGN4	-17122	BTN1A1	19955
chr12	56320756	56321062	0.90	7.18E-01	DGKA	-4903	WIBG	788
chr3	45079252	45079540	1.25	7.18E-01	CLEC3B	11637	CDCP1	108518
chr1	23345684	23346014	1.12	7.18E-01	KDM1A	-92		
chr12	69139542	69140022	0.90	7.18E-01	SLC35E3	-154		
chr13	59965790	59966100	0.95	7.18E-01	DIAPH3	772174		
chr20	18118252	18118572	0.88	7.18E-01	CSRP2BP	-115		
chr6	33756612	33757183	1.12	7.18E-01	LEMD2	8		

chr18	13612176	13612669	1.14	7.18E-01	C18orf19	114168	C18orf1	393637
chr19	34625087	34625581	1.17	7.18E-01	CR622909	-41121	LSM14A	-38018
chr19	45596041	45596341	1.12	7.18E-01	GEMIN7	13673	NKPD1	67217
chr2	220042746	220042995	0.99	7.18E-01	C2orf24	-1169	FAM134A	-68
chr10	112602863	112603173	0.93	7.19E-01	PDCD4	-28578	RBM20	198863
chr12	98909099	98909471	0.94	7.19E-01	TMPO	-124		
chr7	47614073	47614594	0.95	7.19E-01	C7orf69	-220555	TNS3	-35135
chr12	15114330	15114761	0.89	7.20E-01	ARHGDIB	16		
chr10	70287157	70287483	0.80	7.20E-01	SLC25A16	264		
chr14	23039076	23039458	0.80	7.20E-01	DAD1	18876	TCRDV2	111178
chr4	4291820	4292071	1.11	7.20E-01	LYAR	-50	ZNF509	22
chr2	44395134	44395617	1.01	7.20E-01	PPM1B	-624		
chr11	65266307	65266651	1.32	7.20E-01	SCYL1	-26069	FRMD8	112438
chr17	66031619	66031918	1.04	7.20E-01	KPNA2	-79		
chr21	26979671	26979984	1.27	7.20E-01	MRPL39	-27		
chr2	70520597	70520848	1.17	7.21E-01	SNRPG	146		
chr20	62169128	62169496	1.17	7.21E-01	PTK6	-605		
chr4	154340825	154341112	1.25	7.21E-01	KIAA0922	-46529	MND1	75168
chr2	120124257	120124572	0.82	7.22E-01	DBI	-89		
chr15	76603587	76603955	1.06	7.22E-01	ETFA	39		
chr17	41400587	41400866	0.74	7.22E-01	ARL4D	-75626	TMEM106A	36833
chr13	49684240	49684550	1.42	7.22E-01	MLNR	-110079	FNDC3A	134347
chr4	169931438	169931830	1.16	7.22E-01	CBR4	-212		
chr12	7047129	7047452	1.14	7.23E-01	PTPN6	-8449	ATN1	13665
chr1	51425821	51426164	1.08	7.23E-01	FAF1	-57		
chr19	10230439	10230807	1.11	7.23E-01	EIF3G	-24		
chr20	48225489	48225738	1.34	7.23E-01	PTGIS	-40907	B4GALT5	104807
chr17	29158757	29159074	1.16	7.23E-01	ATAD5	-107		
chr17	34842230	34842543	1.14	7.23E-01	ZNHIT3	-86		
chr19	9546000	9546401	1.12	7.23E-01	ZNF266	33		
chr13	49821898	49822163	1.04	7.23E-01	CDADC1	-72		
chr10	74080553	74080914	0.85	7.23E-01	DNAJB12	34173	DDIT4	47057
chr17	62962036	62962390	1.20	7.23E-01	LRRC37A3	-47225	GNA13	90707
chr6	49430898	49431175	1.15	7.23E-01	CENPQ	-59	MUT	-6
chr1	154989989	154990299	0.95	7.23E-01	ZBTB7B	15032	DCST2	16113
chr10	102046365	102046695	1.02	7.24E-01	BLOC1S2	-91		
chr7	148843973	148844648	0.77	7.24E-01	ZNF398	-249		
chr1	35318290	35318615	1.03	7.24E-01	C1orf212	6939	GJA4	59854

chr10	6389862	6390570	1.26	7.24E-01	PFKFB3	145376	PRKCQ	232022
chr12	4758096	4758361	1.17	7.24E-01	AKAP3	-3871	NDUFA9	-54
chr12	8845283	8845519	0.72	7.24E-01	MFAP5	-29968	RIMKLB	-5117
chr3	57050776	57051142	1.07	7.24E-01	ERC2	-548568	ARHGEF3	62377
chr6	47277653	47278015	1.08	7.24E-01	TNFRSF21	-154		
chr9	35096429	35096771	1.07	7.24E-01	PIGO	-54		
chr15	78251737	78252047	0.94	7.24E-01	LINGO1	-327183	TBC1D2B	118102
chr6	27870521	27870906	1.11	7.24E-01	OR2B2	9383	HIST1H2BO	9511
chr7	26240150	26241053	0.90	7.24E-01	CBX3	-497	HNRNPA2B1	-189
chr14	75593385	75593883	0.96	7.24E-01	NEK9	144		
chr15	90016054	90016364	1.18	7.24E-01	POLG	-138183	RHCG	23590
chr12	106695522	106695799	1.15	7.25E-01	POLR3B	-55775	CKAP4	-53948
chr1	224380188	224380443	1.24	7.25E-01	DEGS1	9388	NVL	137556
chr22	23611657	23612260	1.08	7.25E-01	BCR	89407	IGLL1	310536
chr3	38065785	38066061	0.92	7.25E-01	PLCD1	355		
chr9	6015538	6015786	0.97	7.26E-01	RANBP6	-44		
chr1	93811279	93811555	1.10	7.26E-01	DR1	-61		
chr1	207999209	207999505	1.21	7.26E-01	CD46	73955	CD34	85326
chr7	151065196	151065502	0.87	7.26E-01	NUB1	26491	RHEB	151661
chr17	1620282	1620692	1.11	7.26E-01	PRPF8	-32311	SERPINF2	-25643
chr7	155090744	155091035	1.03	7.26E-01	EN2	-159934	INSIG1	1404
chr17	71187658	71188028	0.84	7.27E-01	COG1	-1330		
chr2	114514228	114514492	0.90	7.27E-01	SLC35F5	40		
chr11	108093483	108094005	1.15	7.27E-01	NPAT	-379	ATM	185
chr19	17326042	17326352	0.96	7.27E-01	USE1	42		
chr6	2862917	2863685	0.90	7.28E-01	SERPINB1	-21220	SERPINB9	40244
chr14	61547343	61547653	0.98	7.28E-01	SLC38A6	99618	TMEM30B	201032
chr19	1174125	1174389	1.06	7.28E-01	SBNO2	25		
chr19	663159	663411	0.90	7.28E-01	RNF126	-58		
chr6	33267026	33267341	0.93	7.28E-01	RGL2	-446		
chr8	95565757	95566067	0.92	7.28E-01	KIAA1429	-224		
chr15	85131248	85131583	1.18	7.28E-01	ZSCAN2	-12833	ADAMTSL3	808578
chr19	7082975	7083254	0.92	7.28E-01	ZNF557	13644	INSR	210896
chr5	118367117	118367361	1.34	7.29E-01	TNFAIP8	-324357		
chr1	205719332	205719674	1.17	7.29E-01	NUCKS1	-142		
chr3	53164339	53164614	0.87	7.29E-01	RFT1	-7		
chr11	58345030	58345367	1.13	7.29E-01	LPXN	-1809	ZFP91	-1388
chr7	134966299	134966550	1.12	7.29E-01	STRA8	49694	CNOT4	228426

chr13	95201066	95201646	0.86	7.29E-01	DCT	-69420	TGDS	47155
chr11	105947737	105948258	0.95	7.29E-01	AASDHPPT	-294	KBTBD3	467
chr2	64977761	64978273	0.99	7.29E-01	SLC1A4	-238439	SERTAD2	-96971
chr12	1058841	1059241	1.16	7.30E-01	RAD52	-178		
chr2	71221923	71222285	0.81	7.30E-01	TEX261	-103		
chr8	96145827	96146083	0.82	7.30E-01	PLEKHF2	-83		
chr8	146017699	146017995	1.04	7.30E-01	RPL8	-42		
chr1	114447462	114448041	0.86	7.30E-01	DCLRE1B	-286	AP4B1	-11
chr10	35485026	35485305	1.11	7.30E-01	CCNY	-140636	CREM	68778
chr10	74079373	74079731	0.82	7.30E-01	DNAJB12	35355	DDIT4	45875
chr10	97516215	97516549	1.41	7.30E-01	CCNJ	-286777	ENTPD1	44846
chr19	11090604	11090976	1.06	7.30E-01	LDLR	-109267	SMARCA4	19192
chr2	201753706	201754205	1.19	7.30E-01	PPIL3	-107	NIF3L1	-94
chr6	26124330	26124661	0.93	7.30E-01	HIST1H2BC	-364	HIST1H2AC	123
chr20	42295331	42295862	1.08	7.30E-01	MYBL2	-112		
chr10	30287286	30287561	0.69	7.30E-01	SVIL	-363523	MTPAP	350843
chr15	38982936	38983254	1.11	7.30E-01	THBS1	-890185	RASGRP1	-126088
chr15	75301342	75301745	1.29	7.30E-01	PPCDC	-14383	SCAMP5	13643
chr17	79227176	79227412	0.85	7.30E-01	C17orf89	14183	SLC38A10	41802
chr9	127027084	127027476	1.16	7.30E-01	NEK6	7037	PSMB7	150441
chr13	21517751	21518014	0.87	7.31E-01	XPO4	-40970	LATS2	117839
chr1	16947559	16947834	0.86	7.31E-01	NBPF1	-7715	MSTP9	143278
chr1	89357153	89357441	1.24	7.31E-01	GTF2B	4		
chr16	70517431	70517762	0.66	7.31E-01	FUK	29099	COG4	39860
chr1	153940138	153940413	0.94	7.32E-01	CREB3L4	-121	SLC39A1	-88
chr1	212458624	212458915	0.93	7.32E-01	PPP2R5A	-109		
chr11	64511128	64511685	0.85	7.32E-01	RASGRP2	922		
chr5	137667246	137667803	1.13	7.32E-01	CDC25C	-9		
chr2	232574964	232575237	1.09	7.32E-01	PTMA	1866	PDE6D	70873
chr4	54232140	54232538	1.06	7.33E-01	SCFD2	-97		
chr6	18387285	18387906	0.84	7.33E-01	RNF144B	2		
chr19	17337165	17337436	1.09	7.33E-01	USE1	11146	NR2F6	18850
chr11	73087165	73087426	1.12	7.33E-01	RELT	-109		
chr20	18488033	18488503	1.20	7.33E-01	SEC23B	80		
chr4	123653512	123653984	1.02	7.33E-01	BBS12	-109		
chr17	79633607	79633851	1.06	7.33E-01	C17orf90	-111		
chr13	41593291	41593643	1.01	7.34E-01	ELF1	41		
chr22	39916337	39916762	1.24	7.34E-01	ATF4	-19		

chr5	163342741	163342995	1.16	7.34E-01	MAT2B	410283		
chr1	206853085	206853443	1.12	7.34E-01	MAPKAPK2	-5025	DYRK3	44383
chr12	4216639	4216912	1.07	7.34E-01	PARP11	-234168	CCND2	-166126
chr17	62502705	62503150	1.11	7.34E-01	DDX5	-444	CCDC45	-230
chr5	131525919	131526229	0.94	7.34E-01	P4HA2	37482	CSF2	116589
chr15	65955929	65956248	1.07	7.34E-01	SLC24A1	41819	DENND4A	128542
chr6	7239064	7239447	0.75	7.34E-01	SSR1	74285	RREB1	131068
chr1	27935213	27935467	1.00	7.34E-01	AHDC1	-5197	FGR	26387
chr15	52519642	52520016	1.24	7.34E-01	GNB5	-36264	MYO5C	68141
chr1	226878992	226879302	0.90	7.34E-01	ITPKB	47729	C1orf95	142646
chr15	40697662	40698028	1.07	7.34E-01	IVD	159		
chr16	30709318	30709567	1.15	7.34E-01	SRCAP	-1019		
chr3	53381507	53381927	0.91	7.34E-01	DCP1A	-80		
chr21	43952940	43953182	0.80	7.34E-01	PDE9A	-120801	SLC37A1	33319
chr17	55927288	55927685	1.10	7.34E-01	MRPS23	-88		
chr2	70314344	70314665	1.08	7.34E-01	PCBP1	-80		
chr17	18528966	18529276	1.17	7.34E-01	TBC1D28	18619	LGALS9C	149022
chr9	37411403	37411716	1.00	7.34E-01	GRHPR	-11147	ZCCHC7	291091
chr22	23301435	23301713	1.17	7.34E-01	GNAZ	-111095	IGL@	71614
chr16	85937170	85937463	0.88	7.35E-01	FOXF1	-606816	IRF8	4543
chr1	174992416	174992762	1.06	7.35E-01	MRPS14	-28		
chr11	59327719	59327999	1.27	7.35E-01	OR4D9	45473	OSBP	55758
chr5	53942526	53942955	1.13	7.35E-01	SNX18	129148	ESM1	338673
chr13	50656551	50656987	1.17	7.36E-01	ST13	-89385	KCNRG	67379
chr14	23476162	23476575	1.15	7.36E-01	JUB	-24521	C14orf93	2991
chr19	45520151	45520474	0.99	7.36E-01	SFRS16	-21985	RELB	15601
chr5	156914416	156914749	1.02	7.36E-01	NIPAL4	27556	ADAM19	88185
chr2	101179180	101179446	1.15	7.36E-01	PDCL3	-105		
chr7	99679312	99679648	0.93	7.36E-01	ZNF3	-109		
chr1	20834560	20834814	0.85	7.36E-01	MUL1	-13		
chr8	11325104	11325425	0.91	7.36E-01	BLK	-26256	AMAC1L2	136770
chr19	54663486	54663802	0.98	7.36E-01	LENG1	-198		
chr20	60961934	60962317	1.07	7.36E-01	RPS21	5		
chrX	119077601	119077911	1.10	7.36E-01	NKAP	-21		
chr11	68034012	68034354	1.15	7.36E-01	SUV420H1	-53399	C11orf24	5286
chr1	10534788	10535120	0.83	7.37E-01	DFFA	-2341	PEX14	-49
chr1	175194641	175194928	1.02	7.37E-01	TNN	157791	TNR	517967
chr2	39005214	39005472	1.06	7.37E-01	GEMIN6	85		

chr16	85343321	85343693	1.05	7.37E-01	KIAA0182	-303417	KIAA0513	282097		
chr3	51536040	51536358	0.93	7.37E-01	VPRBP	-2198				
chr19	12813880	12814162	0.99	7.37E-01	FBXW9	-6566	TNPO2	19441		
chr3	176999162	176999432	1.26	7.37E-01	TBL1XR1	-84249				
chr7	74072737	74073022	0.73	7.37E-01	GTF2I	850				
chr1	47779713	47780199	1.05	7.39E-01	STIL	-137				
chr1	59012360	59012699	1.06	7.39E-01	OMA1	-84				
chr15	40811994	40812340	1.09	7.39E-01	RPUSD2	-49370	CHST14	48954		
chr2	86426469	86426769	1.07	7.39E-01	IMMT	-3726	MRPL35	63		
chr3	193852817	193853097	1.03	7.39E-01	HES1	-977				
chr1	12289889	12290171	0.80	7.39E-01	VPS13D	-83				
chr16	1524944	1525188	1.19	7.39E-01	CLCN7	19				
chr19	39342231	39342647	1.05	7.39E-01	HNRNPL	-1822				
chr2	231921168	231921617	1.03	7.39E-01	PSMD1	-216				
chr19	23456805	23457158	1.14	7.39E-01	AK301230	-23820	ZNF91	121287		
chr3	128336428	128336866	1.09	7.39E-01	GATA2	-124617	RPN1	33072		
chr19	18303552	18303870	1.12	7.40E-01	MPV17L2	-329				
chr15	67401964	67402208	0.99	7.40E-01	SMAD3	43891	AAGAB	144988		
chr12	51477261	51477554	0.84	7.40E-01	CSRNP2	-75				
chr9	130213497	130213935	1.05	7.40E-01	LRSAM1	-76	RPL12	-32		
chr3	152313936	152314246	1.05	7.40E-01	TMEM14E	-255312	P2RY1	-238645		
chr5	140700149	140700529	1.01	7.40E-01	TAF7	12				
chr15	93425405	93425820	1.11	7.40E-01	FAM174B	-226582	CHD2	-17938		
chr10	14879955	14880272	1.09	7.41E-01	HSP60	-147	HSPA14	-147	CDNF	-131
chr6	2932446	2932765	1.04	7.41E-01	SERPINB9	-29061	SERPINB6	39484		
chr17	66168589	66168849	0.90	7.41E-01	AMZ2	-75426	KPNA2	136871		
chr9	123698236	123698663	1.34	7.41E-01	TRAF1	-9277	C5	114104		
chr11	33182907	33183308	1.10	7.41E-01	CSTF3	-71				
chr5	66492491	66492854	1.09	7.41E-01	CD180	-56				
chr20	35471791	35472189	1.16	7.41E-01	DSN1	-69836	SAMHD1	108186		
chr6	13336775	13337019	0.94	7.41E-01	TBC1D7	-8127	GFOD1	150890		
chr7	106808816	106809166	0.88	7.41E-01	HBP1	-469				
chr19	16254214	16254678	1.10	7.41E-01	HSH2D	9608	CIB3	29840		
chr3	196359183	196359686	1.06	7.41E-01	LRRC33	-7221	FBXO45	63710		
chr19	17516368	17516743	0.98	7.41E-01	BST2	-172				
chr20	1447498	1447744	1.17	7.41E-01	NSFL1C	796				
chr11	62432141	62432435	1.05	7.42E-01	METTL12	-491	METTL12	-491		
chr20	42285953	42286302	0.94	7.42E-01	MYBL2	-9581	IFT52	66549		

chr2	68978924	68979270	0.94	7.42E-01	ARHGAP25	17129	BMP10	119552
chr22	43112673	43113094	1.09	7.42E-01	CYB5R3	-67479	A4GALT	3992
chr5	43040349	43040713	0.96	7.42E-01	C5orf39	-84		
chr3	11888257	11888525	1.05	7.43E-01	C3orf31	-39		
chr1	166845525	166845769	1.08	7.43E-01	TADA1	7		
chr2	220159467	220159902	0.86	7.43E-01	PTPRN	14458	DNAJB2	15645
chr1	93544522	93544883	0.91	7.43E-01	MTF2	-89		
chr8	49427065	49427362	0.93	7.44E-01	EFCAB1	220656	UBE2V2	506219
chr4	44680138	44680607	1.15	7.44E-01	GUF1	-60		
chr17	74722843	74723126	1.02	7.44E-01	JMJD6	-104	C17orf95	35
chr1	165737895	165738308	1.22	7.45E-01	TMCO1	33		
chr12	113659004	113659438	0.82	7.45E-01	TPCN1	-39		
chr11	9336043	9336665	1.12	7.45E-01	TMEM41B	-58		
chr1	109102397	109102769	0.86	7.45E-01	PRPF38B	-132349	NBPF6	109679
chr17	18086804	18087229	1.09	7.46E-01	ALKBH5	150		
chr4	3108382	3108626	1.06	7.46E-01	RGS12	-207370	HTT	32096
chr6	31536343	31536698	1.10	7.46E-01	LTA	-3355		
chr1	153769673	153769996	1.14	7.46E-01	SLC27A3	22067	GATAD2B	125616
chr18	42686314	42686616	1.19	7.46E-01	SLC14A2	-508301	SETBP1	425602
chr19	10360785	10361061	0.92	7.46E-01	MRPL4	-1717		
chr1	150848875	150849334	1.00	7.46E-01	ARNT	81		
chr3	52719829	52720258	1.06	7.46E-01	GNL3	108		
chr8	82754335	82755031	0.90	7.47E-01	SNX16	-162		
chr1	28974551	28974861	0.94	7.47E-01	GMEB1	-20538	TAF12	-5102
chr11	67171092	67171499	0.95	7.47E-01	PPP1CA	-1920	TBC1D10C	-116
chr19	1848441	1848914	0.81	7.47E-01	REXO1	-226		
chr15	20561681	20562014	1.15	7.47E-01	HERC2P3	149585		
chr11	118098654	118098918	1.05	7.48E-01	AMICA1	-2977		
chr12	95612606	95612853	1.11	7.48E-01	FGD6	-1490	VEZT	1208
chr11	14521350	14521600	1.09	7.48E-01	COPB1	-60		
chr14	24664502	24665127	1.05	7.49E-01	TM9SF1	67		
chr16	89766622	89766866	1.16	7.49E-01	CDK10	13668	C16orf7	20650
chr6	26554270	26554527	0.95	7.49E-01	ABT1	-42781	HMGN4	15827
chr8	134511547	134511857	1.12	7.49E-01	NDRG1	-202155	ST3GAL1	72481
chr7	127748844	127749215	1.01	7.49E-01	LEP	-132301	LRRC4	-78028
chr16	75589986	75590296	0.84	7.49E-01	TMEM231	29		
chr11	67035070	67035475	0.95	7.49E-01	SSH3	-35646	ADRBK1	1368
chr12	6602292	6602532	1.08	7.49E-01	NCAPD2	-886	MRPL51	59

chr12	108957320	108957654	1.16	7.50E-01	SART3	-2322	ISCU	1193
chr17	2615013	2615276	1.05	7.50E-01	KIAA0664	-218		
chr4	492864	493256	1.11	7.50E-01	PIGG	71	ZNF721	382
chr6	27720983	27721339	0.98	7.51E-01	ZNF184	-280264	HIST1H2BL	54548
chr10	7860240	7860550	0.91	7.51E-01	TAF3	-278		
chr16	89282915	89283193	1.20	7.51E-01	ZNF778	-1057		
chr4	2420506	2420770	1.01	7.51E-01	ZFYVE28	-269		
chr4	2845426	2845666	0.93	7.51E-01	ADD1	-38		
chr1	166059157	166059467	1.43	7.51E-01	POGK	-749412	UCK2	262422
chr6	33168200	33168702	1.10	7.51E-01	SLC39A7	-152	RXRB	-19
chr9	130733083	130733583	1.12	7.51E-01	DPM2	-32570	NAIF1	96266
chr3	9438656	9438939	0.92	7.51E-01	THUMPD3	34081	LHFPL4	156688
chr3	167813457	167813932	1.01	7.51E-01	GOLIM4	-278		
chr3	73551898	73552142	1.31	7.51E-01	PDZRN3	122052	FLJ10213	441210
chr17	17494854	17495260	0.89	7.51E-01	PEMT	-63		
chr5	122759009	122759390	1.16	7.51E-01	CEP120	52		
chr13	73301748	73302076	1.14	7.51E-01	C13orf34	-130	C13orf37	26
chr15	68346195	68346530	0.99	7.52E-01	PIAS1	-209		
chr6	18264881	18265233	1.15	7.52E-01	DEK	-258		
chr7	100025824	100026106	0.91	7.52E-01	MEPCE	-1564	ZCWPW1	337
chr20	1484270	1484580	1.20	7.52E-01	SIRPB2	-12192	SIRPD	53918
chr20	5626014	5626383	0.89	7.52E-01	CHGB	-265775	RP5-1022P6.2	-34527
chr12	57039626	57040019	1.15	7.52E-01	ATP5B	29		
chr12	90274472	90274810	1.15	7.52E-01	ATP2B1	-224797		
chr2	208890146	208890555	1.10	7.52E-01	PLEKHM3	-67		
chr16	84540042	84540356	1.07	7.53E-01	KIAA1609	-1911		
chr19	4638984	4639345	0.99	7.53E-01	SEMA6B	-80664	C19orf10	31250
chr8	110534906	110535162	1.04	7.53E-01	EBAG9	-16895	PKHD1L1	160328
chr12	6772341	6772675	1.16	7.53E-01	ING4	-200		
chr15	34393969	34394332	1.15	7.53E-01	C15orf24	-98		
chr9	34986442	34986752	0.96	7.53E-01	DNAJB5	-3670		
chr3	49058734	49059044	1.01	7.53E-01	DALRD3	-2872	NDUFAF3	-185
chr5	43008705	43009015	0.89	7.53E-01	C5orf39	31587	GHR	584834
chr9	66458015	66458298	1.01	7.53E-01	FAM75A7	-948547		
chr7	127983812	127984142	1.12	7.53E-01	RBM28	-15		
chr1	226862815	226863068	0.98	7.53E-01	ITPKB	63934	C1orf95	126441
chr2	208576038	208576368	1.03	7.53E-01	FAM119A	-86230	FZD5	57940
chr9	37408994	37409583	1.16	7.53E-01	GRHPR	-13418	ZCCHC7	288820

chr15	85174348	85174971	1.13	7.53E-01	SCAND2	-31			
chr10	82231927	82232277	1.04	7.54E-01	SH2D4B	-65556	TSPAN14	18064
chr16	12385886	12386196	0.77	7.54E-01	SNX29	239986	CPPED1	511703
chr18	51795910	51796299	1.19	7.54E-01	POLI	256		
chr20	1099023	1099379	0.94	7.54E-01	PSMF1	-39		
chr19	8645671	8645960	0.95	7.54E-01	MYO1F	-3509		
chr2	223725604	223725946	1.07	7.55E-01	ACSL3	43		
chr3	183229684	183230025	1.09	7.55E-01	MCF2L2	-84000	KLHL6	43644
chr19	13213833	13214228	1.06	7.55E-01	LYL1	-350		
chr4	73935421	73935847	1.19	7.55E-01	COX18	-162		
chr21	43653104	43653473	1.16	7.55E-01	ABCG1	14022	TFF3	82417
chr22	32145894	32146364	0.97	7.55E-01	YWHAH	-194350	PISD	-119319
chr2	183988933	183989305	1.12	7.55E-01	NUP35	36		
chr1	204475521	204475817	1.15	7.55E-01	PIK3C2B	-16195	MDM4	-9842
chr10	74114594	74114838	0.86	7.55E-01	DNAJB12	191		
chr8	56903608	56903918	0.97	7.56E-01	RPS20	83377	LYN	111377
chr11	73881986	73882516	1.13	7.56E-01	C2CD3	-187	PPME1	-117
chr22	46692413	46692756	1.10	7.56E-01	GTSE1	-53		
chr3	31993783	31994100	1.35	7.56E-01	OSBPL10	29296	STT3B	419451
chr7	92861475	92861781	0.92	7.56E-01	HEPACAM2	-5846	CALCR	342190
chr19	46430995	46431305	1.07	7.56E-01	NANOS2	-13114	NOVA2	45507
chr7	105712036	105712400	1.01	7.56E-01	SYPL1	40839	FLJ23834	108561
chr17	40925254	40925568	0.98	7.57E-01	VPS25	-43		
chr3	133380631	133380971	0.98	7.57E-01	TOPBP1	-64		
chr1	24237338	24237703	0.96	7.57E-01	FUCA1	-42700	CNR2	2296
chr1	36929847	36930159	1.01	7.57E-01	MRPS15	37		
chr13	49106754	49107067	0.97	7.57E-01	RCBTB2	405		
chr13	103248517	103248920	0.88	7.57E-01	TPP2	-567		
chr12	50092929	50093272	1.08	7.57E-01	FMNL3	8096	PRPF40B	68773
chr3	187619873	187620465	0.99	7.57E-01	LPP	-310552	BCL6	-156694
chr7	43688342	43688649	1.00	7.57E-01	STK17A	65804	C7orf44	80587
chr12	9102137	9102426	1.15	7.57E-01	M6PR	-30		
chr8	80997334	80997611	1.15	7.57E-01	TPD52	-4463		
chr7	77427853	77428178	0.82	7.57E-01	TMEM60	-269	PHTF2	-93
chr9	123918770	123919039	0.78	7.57E-01	RAB14	45460	CEP110	68331
chr10	73487422	73487858	1.01	7.58E-01	RP11-472K8.2-001	-8062	C10orf54	45697
chr6	3069068	3069563	0.91	7.58E-01	RIPK1	-7682	NQO2	69249
chr16	3018342	3018618	1.01	7.59E-01	PAQR4	-862		

chr16	75551150	75551580	0.82	7.59E-01	CHST6	-22439	CHST5	17703
chr3	53214829	53215101	1.17	7.59E-01	PRKCD	19742	TKT	75103
chr1	1609320	1609630	1.00	7.59E-01	LOC728661	14768	MMP23B	41915
chr14	35590998	35591364	0.98	7.59E-01	KIAA0391	-595	PPP2R3C	338
chr1	161014613	161014958	1.16	7.59E-01	USF1	971		
chr16	70380620	70380951	1.12	7.59E-01	DDX19B	47714	ST3GAL2	92205
chr8	11388384	11388678	0.97	7.59E-01	GATA4	-173186	BLK	37010
chr15	74838640	74838960	0.96	7.59E-01	CLK3	-68535	ARID3B	5252
chr6	8064574	8064914	0.90	7.59E-01	MUTED	-97		
chrX	153191942	153192478	0.93	7.60E-01	ARHGAP4	-512		
chr5	102455781	102456178	1.18	7.60E-01	GIN1	-138		
chr11	64455941	64456360	1.01	7.60E-01	NRXN2	34509	SLC22A12	97869
chr11	2421496	2421963	1.21	7.60E-01	TRPM5	22545	CD81	23183
chr1	1310534	1310940	1.02	7.60E-01	AURKAIP1	-175		
chr17	61920949	61921214	1.17	7.61E-01	SMARCD2	-731		
chr20	46012823	46013133	1.15	7.61E-01	NCOA3	-117679	ZMYND8	-27504
chr7	30067706	30068081	0.97	7.61E-01	FKBP14	-1626	PLEKHA8	-394
chr19	19303227	19303696	0.98	7.61E-01	LOC729991	-62	RFXANK	454
chr1	172787993	172788411	0.96	7.61E-01	FASLG	160017	TNFSF18	231901
chr1	46806283	46806595	0.86	7.62E-01	NSUN4	49		
chr15	75063447	75063771	1.10	7.62E-01	CSK	-10816	CYP1A2	22425
chr8	134461750	134462013	1.10	7.62E-01	NDRG1	-152335	ST3GAL1	122301
chr19	16770733	16771300	1.00	7.62E-01	TMEM38A	-921	C19orf42	-49
chr1	247267460	247267789	1.21	7.63E-01	ZNF669	49		
chr11	73693671	73694040	0.94	7.63E-01	UCP2	33		
chr6	42981696	42982015	1.08	7.63E-01	MEA1	-238	KLHDC3	-121
chr12	120884036	120884387	0.98	7.63E-01	GATC	-72	TRIAP1	3
chr10	35415515	35416183	1.01	7.64E-01	CREM	-539		
chr17	40985280	40985655	1.17	7.64E-01	PSME3	45		
chr21	44313165	44313556	0.98	7.64E-01	NDUFV3	-17		
chr9	2843960	2844379	1.04	7.64E-01	KIAA0020	-40		
chr11	67367836	67368151	1.10	7.64E-01	NDUFV1	-6329	GSTP1	16928
chr6	15901877	15902171	1.04	7.64E-01	DTNBP1	-238753	MYLIP	-227293
chr22	22522255	22522565	1.15	7.64E-01	TOP3B	-185263	VPREB1	-76790
chr10	73610815	73611173	0.84	7.64E-01	PSAP	88		
chr1	45187177	45187550	1.23	7.64E-01	KIF2C	-18126	C1orf228	46970
chr11	111894983	111895293	1.11	7.64E-01	DLAT	-400		
chr11	30364244	30364541	0.96	7.64E-01	FSHB	111830	MPPED2	237648

chr4	87281120	87281480	0.85	7.64E-01	PTPN13	-234168	MAPK10	-5503
chr9	132650683	132651108	0.97	7.65E-01	USP20	53200	FNBP1	154577
chr12	57623249	57623558	1.12	7.65E-01	SHMT2	-148		
chr4	2243534	2244029	0.85	7.65E-01	HAUS3	78		
chr10	27443223	27443473	0.95	7.65E-01	MASTL	-369	YME1L1	-27
chr1	19536083	19536620	0.97	7.66E-01	UBR4	394		
chr11	45951698	45951954	0.95	7.66E-01	GYLTL1B	2355	PHF21A	191159
chr12	113524640	113524888	0.95	7.66E-01	DTX1	29102	RASAL1	49257
chr14	65819860	65820222	0.94	7.67E-01	MAX	-250814	FUT8	-59494
chr17	42422986	42423396	0.96	7.67E-01	GRN	700		
chr19	35815026	35815306	1.31	7.67E-01	CD22	-4913		
chr5	36876597	36876971	1.09	7.67E-01	NIPBL	-77		
chr1	178550355	178550839	1.11	7.67E-01	RALGPS2	-143703	C1orf49	68385
chr19	42449613	42449917	1.06	7.67E-01	RABAC1	13763	ARHGEF1	61319
chr19	56154767	56155077	1.11	7.68E-01	CCDC106	-4032	ZNF581	-64
chr12	53689030	53689565	1.03	7.68E-01	C12orf10	-4172	PFDN5	63
chr20	32950927	32951171	1.09	7.68E-01	ITCH	-13		
chr12	89746364	89746647	0.84	7.68E-01	DUSP6	-210		
chr6	43596978	43597287	0.84	7.68E-01	GTPBP2	-197		
chr12	106751267	106751684	1.06	7.68E-01	POLR3B	40		
chr16	84641412	84641706	0.83	7.68E-01	KIAA1609	-103271	COTL1	10110
chr17	3749501	3749798	1.12	7.68E-01	ITGAE	-45113	CAMKK1	44387
chr6	142468083	142468479	0.93	7.68E-01	VTA1	-129		
chr19	10369725	10369986	1.19	7.69E-01	ICAM1	-11661	MRPL4	7216
chr8	125210132	125210372	0.95	7.69E-01	TMEM65	174688	FER1L6	346025
chr15	59592577	59592848	1.02	7.69E-01	MYO1E	72358	LDHAL6B	93671
chr19	3990259	3990503	1.03	7.69E-01	EEF2	-4920		
chr19	7069571	7069847	1.09	7.69E-01	ZNF557	238		
chr3	135968942	135969252	1.03	7.69E-01	PCCB	-70		
chr8	145597470	145597916	1.09	7.69E-01	ADCK5	-38		
chr15	91236879	91237231	1.05	7.69E-01	BLM	-23524	CRTC3	163857
chr4	154354310	154354596	1.05	7.69E-01	KIAA0922	-33045	MND1	88652
chr14	50100947	50101364	0.93	7.69E-01	C14orf104	792		
chr1	43123854	43124135	1.10	7.69E-01	PPIH	-53		
chr8	23100918	23101297	0.88	7.69E-01	CHMP7	-42		
chr2	48667390	48667696	1.07	7.69E-01	STON1-GTF2A1L	-128616	FOXN2	125748
chr17	16557003	16557247	0.97	7.69E-01	ZNF624	33		
chr13	25496968	25497232	1.16	7.70E-01	CENPJ	-15		

chr5	177026952	177027228	1.12	7.70E-01	B4GALT7	-82		
chr19	47016710	47017004	1.15	7.70E-01	CCDC8	-99938	AK094504	87600
chr1	26872123	26872369	1.12	7.71E-01	RPS6KA1	-97		
chr7	93633549	93633933	1.17	7.71E-01	BET1	-51		
chr9	130788667	130788914	1.26	7.71E-01	DPM2	-88028	NAIF1	40808
chr17	41837810	41838119	1.02	7.71E-01	SOST	-1809		
chr6	29932594	29932939	0.96	7.72E-01	HLA-F	-41450	HLA-A	22436
chr15	42996636	42996912	1.24	7.72E-01	KIAA1300	14981	CDAN1	32643
chr7	65958554	65958808	0.87	7.72E-01	KCTD7	-135209	TPST1	288422
chr14	106178422	106178674	1.04	7.72E-01	IGHE	150914	TMEM121	185595
chr15	45879067	45879641	0.91	7.72E-01	PLDN	-63		
chr19	6522198	6522625	1.13	7.72E-01	TUBB4	-20082	TNFSF9	-8598
chr8	8310197	8310584	0.88	7.73E-01	CLDN23	-249275	PRAGMIN	-71134
chr1	16160845	16161192	0.85	7.73E-01	UQCRHL	-26825	SPEN	-13340
chr1	33116044	33116361	1.12	7.73E-01	RBBP4	-546		
chr13	47169315	47169559	1.08	7.73E-01	LRCH1	42141	ESD	201930
chr6	26311890	26312135	1.27	7.73E-01	BTN3A2	-53385	HIST1H4H	-26286
chr8	42269856	42270167	1.07	7.73E-01	C8orf40	-126286	VDAC3	20666
chr12	56510102	56510628	0.85	7.73E-01	ZC3H10	-1665	RPL41	-9
chr10	11727109	11727374	0.96	7.74E-01	USP6NL	-152968	ECHDC3	-57114
chr17	33416345	33416791	0.98	7.74E-01	RFFL	-274		
chr16	67580908	67581218	0.89	7.74E-01	CTCF	-15401	FAM65A	18309
chr17	76374580	76374836	1.10	7.75E-01	PGS1	-27		
chr2	15282319	15282671	1.13	7.75E-01	DDX1	-449275		
chr7	110723552	110723841	1.11	7.75E-01	LRRN3	-7365		
chr15	49338666	49339033	1.22	7.75E-01	SHC4	-83209	COPS2	109004
chr16	23652581	23652846	0.99	7.75E-01	DCTN5	-93	PALB2	-36
chr2	135208443	135208723	1.02	7.75E-01	MGAT5	196753	TMEM163	267988
chr5	180593892	180594147	1.04	7.75E-01	OR2V2	12077	TRIM7	38157
chr8	145159179	145159423	1.01	7.75E-01	SHARPIN	-161	MAF1	-4
chr15	45003485	45003892	1.03	7.75E-01	B2M	4		
chr11	121319111	121319523	0.87	7.76E-01	SORL1	-3644		
chr8	90996298	90996897	0.90	7.76E-01	NBN	301		
chr19	36248656	36249060	0.92	7.76E-01	HSPB6	-928		
chr1	111764383	111764669	0.87	7.76E-01	DRAM2	-81688	CHI3L2	-5755
chr11	9777990	9778468	1.24	7.76E-01	SWAP70	92601	SBF2	537525
chr12	3913097	3913333	0.74	7.76E-01	EFCAB4B	-50849	PARP11	69393
chr1	94344543	94344960	0.90	7.76E-01	DNTTIP2	-10		

chr5	86708641	86708951	1.01	7.76E-01	CCNH	40		
chr17	74543418	74543715	1.05	7.76E-01	PRCD	7446	ST6GALNAC2	38578
chr18	20938800	20939092	0.79	7.76E-01	C18orf45	78979	CABLES1	223219
chr17	17307235	17307619	1.06	7.77E-01	MED9	-72873	NT5M	100747
chr1	155948243	155948609	0.98	7.77E-01	ARHGEF2	-90		
chr15	67357898	67358252	1.02	7.77E-01	SMAD3	-120		
chr16	5083762	5084072	1.20	7.77E-01	NAGPA	19		
chr19	40727829	40728184	0.93	7.77E-01	TTC9B	-3701		
chr1	94312943	94313253	1.02	7.77E-01	BCAR3	-165713	DNTTIP2	31644
chr14	64970423	64970848	0.97	7.77E-01	ZBTB1	-656	ZBTB25	-82
chr18	13726482	13726915	1.00	7.77E-01	C18orf19	-108	RNMT	-5
chr2	20861244	20861541	1.18	7.77E-01	HS1BP3	-10529	GDF7	-5031
chr9	139117288	139117894	1.21	7.77E-01	LHX3	-22587	QSOX2	20096
chr2	85822615	85822939	1.13	7.77E-01	RNF181	-60		
chr17	62023166	62023437	1.13	7.78E-01	CD79B	-13598	SCN4A	26976
chr10	103113506	103113932	0.98	7.78E-01	BTRC	-106		
chr17	43303488	43303824	1.14	7.78E-01	FMNL1	4364	MAP3K14	90758
chr1	112298168	112298446	0.97	7.78E-01	DDX20	117		
chr1	173793473	173794157	1.22	7.78E-01	DARS2	-143	CENPL	211
chr22	46171394	46171965	1.01	7.78E-01	ATXN10	103993	WNT7B	201328
chr19	45578516	45578836	1.12	7.79E-01	GEMIN7	-3842	ZNF296	1012
chr12	15101607	15102044	1.07	7.79E-01	ERP27	-10363	ARHGDIB	12736
chr2	62115660	62116038	0.95	7.79E-01	CCT4	-58		
chr2	191184435	191184831	0.95	7.79E-01	HIBCH	-74		
chr11	71791710	71792021	1.06	7.79E-01	NUMA1	-293	LRTOMT	484
chr14	51342080	51342422	0.83	7.79E-01	ABHD12B	3373	PYGL	68997
chr17	67577425	67577915	0.88	7.79E-01	KCNJ16	-493756	MAP2K6	166832
chr16	23607502	23607866	0.93	7.80E-01	NDUFAB1	-45		
chr9	127379814	127380130	1.16	7.80E-01	NR5A1	-110273	NR6A1	153604
chr22	38004384	38004762	1.08	7.80E-01	GGA1	70		
chr15	75744200	75744717	0.96	7.80E-01	SIN3A	-533		
chr2	219761211	219761455	1.13	7.80E-01	CDK5R2	-63065	WNT10A	16078
chr11	73490402	73490930	1.11	7.80E-01	RAB6A	-18465	MRPL48	-8251
chr19	4369050	4369477	1.07	7.80E-01	MPND	25740	SH3GL1	31207
chr3	15244221	15244497	0.90	7.81E-01	CAPN7	-3374		
chr15	41408513	41409077	1.10	7.81E-01	INO80	-455		
chr1	226876209	226876519	0.88	7.81E-01	ITPKB	50512	C1orf95	139863
chr14	90865879	90866222	0.82	7.81E-01	CALM1	2678	TTC7B	416710

chr16	28503267	28503563	1.19	7.82E-01	APOB48R	-2578	CLN3	208
chr3	194054550	194054826	1.04	7.82E-01	CPN2	17369	HES1	200754
chr8	145653827	145654085	1.11	7.82E-01	VPS28	-29		
chr1	203296810	203297255	1.02	7.82E-01	BTG2	22369	FMOD	23256
chr19	46026942	46027252	1.02	7.82E-01	VASP	16409	OPA3	61025
chr11	62495003	62495553	0.96	7.83E-01	TTC9C	-674	HNRNPUL2	-457
chr2	27545845	27546247	1.12	7.83E-01	MPV17	-77		
chr1	63988842	63989380	0.88	7.83E-01	ITGB3BP	-276	EFCAB7	98
chr22	36847828	36848072	0.92	7.83E-01	MYH9	-63887	TXN2	29737
chr5	71604126	71604566	0.90	7.83E-01	MRPS27	11738	MAP1B	201228
chr6	7917193	7917493	0.87	7.83E-01	PIP5K1A	-68992	TXNDC5	-6302
chr12	133757816	133758211	0.85	7.84E-01	ZNF268	19		
chr21	44299431	44299839	1.05	7.84E-01	WDR4	43		
chr3	185303694	185304247	1.13	7.84E-01	SENP2	-60		
chr14	24740655	24740930	0.91	7.84E-01	RABGGTA	10		
chr10	94516187	94516620	0.98	7.84E-01	EXOC6	-91821	HHEX	66723
chr5	95144537	95144822	0.94	7.84E-01	GLRX	13897	RHOBTB3	77830
chr20	62495554	62495849	1.09	7.85E-01	TPD52L2	-888		
chr3	13056706	13057016	1.05	7.85E-01	RPL32	-174912	IQSEC1	57756
chr16	67548263	67548584	1.16	7.85E-01	AGRP	-30708	FAM65A	-14330
chr15	101471592	101471970	1.18	7.85E-01	LRRK1	12321	CHSY1	320345
chr16	22308312	22308743	1.07	7.85E-01	POLR3E	-213		
chr21	46359742	46360024	1.18	7.85E-01	C21orf67	-55		
chr5	110074370	110074722	1.04	7.85E-01	SLC25A46	-208		
chr5	78023507	78023848	1.08	7.85E-01	LHFPL2	-79030	ARSB	258679
chr17	78711201	78711521	1.11	7.85E-01	CHMP6	-254280	RPTOR	192736
chr3	57583041	57583708	1.14	7.86E-01	ARF4	-160		
chr3	171757132	171757442	1.04	7.86E-01	FNDC3B	-131		
chr1	51465855	51466116	0.83	7.86E-01	C1orf185	-101920	CDKN2C	30344
chr11	107328489	107328817	1.21	7.86E-01	CWF19L2	-81		
chr3	187653559	187654015	0.93	7.86E-01	LPP	-276934	BCL6	-190312
chr12	69201707	69202068	0.78	7.86E-01	MDM2	-83		
chr5	151151382	151151706	1.13	7.86E-01	G3BP1	68		
chr6	28048546	28048870	0.97	7.87E-01	ZSCAN16	-43679	ZNF165	2136
chr5	180615313	180615654	1.13	7.87E-01	TRIM7	16693	OR2V2	33541
chr6	164088712	164089139	1.24	7.87E-01	QKI	253251		
chr6	37123231	37123658	1.25	7.87E-01	PIM1	-14477	FGD2	150022
chr1	32713826	32714168	0.88	7.88E-01	LCK	-25715	EIF3I	26038

chr22	23227191	23227531	1.02	7.88E-01	IGL@	-2599		
chr18	48778050	48778360	1.00	7.88E-01	MEX3C	-54515		
chr9	139682150	139682439	0.96	7.88E-01	TMEM141	-3482		
chr12	96429341	96429907	1.21	7.88E-01	LTA4H	-259		
chr17	38115240	38115565	1.07	7.88E-01	GSDMA	-3823		
chr5	1792961	1793236	0.93	7.88E-01	LPCAT1	-269023	MRPL36	6857
chr3	123361254	123361535	0.99	7.89E-01	PTPLB	-57471	MYLK	241754
chr16	11775500	11775782	0.86	7.89E-01	SNN	13340	TXNDC11	61007
chr7	8335745	8335995	1.17	7.89E-01	NXPH1	-137715	ICA1	-34188
chr14	65796521	65796899	1.05	7.89E-01	MAX	-227483	FUT8	-82825
chr8	145159697	145159996	1.00	7.90E-01	SHARPIN	-707	MAF1	542
chr7	102066284	102066638	0.90	7.90E-01	ORAI2	-7535	PRKRIP1	29657
chr7	132937612	132937862	1.00	7.90E-01	EXOC4	-86		
chr3	69133922	69134232	1.15	7.90E-01	UBA3	-4553	ARL6IP5	-13
chr10	103124491	103124801	0.90	7.90E-01	BTRC	10821	POLL	223327
chr6	11575810	11576078	1.06	7.91E-01	TMEM170B	37433	C6orf105	203336
chr10	14996055	14996534	0.93	7.91E-01	DCLRE1C	-201		
chr19	48103666	48103964	0.93	7.91E-01	ZNF541	-44702	GLTSCR1	-7638
chr20	30154316	30154871	0.98	7.91E-01	ID1	-38498	HM13	52353
chr22	38054159	38054455	0.90	7.91E-01	LGALS1	-17306	SH3BP1	19250
chr22	40439971	40440824	0.93	7.92E-01	TNRC6B	-133531	GRAP2	143312
chr19	12721445	12721727	0.95	7.92E-01	ZNF791	-146	ZNF490	37
chr14	70826362	70826641	1.03	7.92E-01	COX16	-58		
chrX	48815542	48815981	1.07	7.92E-01	OTUD5	-869		
chr1	23946237	23946663	0.91	7.92E-01	RPL11	-71844	ID3	-60128
chr13	33927426	33927689	0.95	7.92E-01	RFC3	-464648	STARD13	-67657
chr19	49496536	49497298	0.83	7.92E-01	GYS1	-307	RUVBL2	-239
chr13	103498007	103498422	0.99	7.93E-01	ERCC5	38719	SLC10A2	220981
chr20	31073031	31073385	0.87	7.93E-01	ASXL1	127055	COMMD7	258606
chr15	68966335	68966645	0.91	7.93E-01	CORO2B	94917	ANP32A	146771
chr14	35761479	35761832	0.91	7.93E-01	PSMA6	82		
chr1	54303952	54304428	0.93	7.93E-01	TMEM48	-15		
chr11	71689205	71689454	0.90	7.93E-01	IL18BP	-20628	RNF121	49562
chr7	134143858	134144168	0.87	7.93E-01	AKR1B1	-125		
chr14	39583272	39583561	1.13	7.93E-01	SIP1	-71		
chr5	149840873	149841248	1.02	7.94E-01	NDST1	-46613	RPS14	-11742
chr17	57696446	57697399	1.15	7.94E-01	CLTC	-127		
chr6	17600302	17600638	0.95	7.94E-01	FAM8A1	-48		

chr12	54140459	54140727	0.84	7.94E-01	HOXC13	-191983	CALCOCO1	-19286
chr13	74707908	74708218	1.12	7.94E-01	KLF12	3		
chr1	113498712	113499106	1.03	7.94E-01	SLC16A1	66		
chr14	31889855	31890166	1.07	7.94E-01	HEATR5A	-31800	C14orf126	36669
chr19	48866326	48866759	0.87	7.94E-01	SYNGR4	-1114	TMEM143	643
chr20	44600706	44601186	1.04	7.95E-01	ZNF335	-113		
chr17	37309136	37309398	0.94	7.95E-01	PLXDC1	-1365		
chr6	28049223	28049486	1.09	7.95E-01	ZSCAN16	-43032	ZNF165	2783
chr11	17099220	17099521	1.11	7.95E-01	RPS13	-151		
chr15	94831052	94831362	1.04	7.95E-01	MCTP2	-10223		
chr17	27717111	27717402	1.05	7.95E-01	TAOK1	-686		
chr18	11851118	11851516	0.85	7.95E-01	CHMP1B	-72		
chr6	26204650	26205264	0.90	7.95E-01	HIST1H4E	84		
chr13	38407612	38407888	1.10	7.95E-01	POSTN	-234769	TRPC4	36189
chr15	52525612	52526001	1.07	7.95E-01	GNB5	-42242	MYO5C	62163
chr22	39842878	39843189	1.12	7.95E-01	MGAT3	-40195	MAP3K7IP1	47275
chr7	129712053	129712312	1.12	7.95E-01	ZC3HC1	-20950	TMEM209	133155
chr2	128615607	128615952	0.94	7.95E-01	POLR2D	-51		
chr16	12415319	12415637	0.96	7.95E-01	SNX29	269423	CPPED1	482266
chr18	42684047	42684334	1.21	7.95E-01	SLC14A2	-510575	SETBP1	423328
chr4	153700702	153701147	1.10	7.95E-01	ARFIP1	-187	TIGD4	-48
chr10	70480680	70480996	0.93	7.95E-01	CCAR1	-133		
chr11	62521362	62521811	1.06	7.95E-01	ZBTB3	69		
chr9	33722102	33722412	1.29	7.95E-01	PTENP1	-44839	PRSS3	-28258
chr2	68654205	68654638	1.10	7.96E-01	APLF	-40269	PLEK	62100
chr9	27573664	27573967	1.10	7.96E-01	MOBKL2B	-43966		
chr16	30825675	30825962	0.99	7.96E-01	ZNF629	-27296	BCL7C	79580
chr1	70671137	70671470	0.93	7.96E-01	SFRS11	-61	LRRC40	-29
chr17	7155600	7155962	1.08	7.96E-01	DULLARD	-786	C17orf81	230
chr9	36258326	36258619	1.09	7.96E-01	GNE	18568	CLTA	67581
chr7	26438623	26439112	0.89	7.96E-01	SNX10	107353	SKAP2	465473
chr13	41634784	41635099	1.14	7.96E-01	WBP4	-755		
chr1	63249657	63249987	0.80	7.97E-01	ATG4C	19		
chr13	111108288	111108571	0.92	7.97E-01	RAB20	105641	COL4A2	148799
chr12	113658710	113659001	0.78	7.97E-01	TPCN1	-404		
chr10	75012344	75012605	0.88	7.97E-01	MRPS16	-24		
chr12	108959788	108960105	0.93	7.97E-01	SART3	-4782	ISCU	3653
chr14	50426745	50427051	1.03	7.97E-01	ARF6	67162	SOS2	271201

chr20	31490341	31490651	0.94	7.97E-01	EFCAB8	43767	SPAG4L	101743		
chr19	52772589	52772952	0.98	7.97E-01	ZNF766	-53				
chr2	27994495	27994835	1.17	7.97E-01	MRPL33	81				
chr10	104192280	104192621	0.97	7.97E-01	CUEDC2	-28				
chr1	10010446	10010713	0.83	7.97E-01	RBP7	-46675	NMNAT1	7094		
chr10	1505291	1505585	0.74	7.97E-01	IDI1	-410377	ADARB2	274280		
chr15	86018577	86018887	1.22	7.97E-01	AKAP13	94861	KLHL25	319457		
chr16	14379560	14380000	1.24	7.97E-01	MKL2	214584	PARN	344344		
chr16	81865284	81865703	1.19	7.97E-01	PLCG2	52564	SDR42E1	179599		
chr6	26020422	26020866	0.94	7.97E-01	HIST1H1A	-2604	HIST1H4A	-1263	HIST1H3A	-74
chr21	11144414	11144883	1.02	7.97E-01	BAGE	-45712				
chr5	79551875	79552202	1.11	7.97E-01	SERINC5	-169				
chr1	41078012	41078262	0.93	7.98E-01	RIMS3	53187	ZNF684	80904		
chr14	96133650	96134027	0.94	7.99E-01	TCL1A	46694	GLRX5	132516		
chr6	41673904	41674213	1.28	7.99E-01	TFEB	17405	MDFI	67864		
chr3	193290242	193290488	0.98	8.00E-01	OPA1	-20568	ATP13A4	-17669		
chr19	40324174	40324484	0.91	8.00E-01	DYRK1B	512				
chr14	107175134	107175454	0.98	8.00E-01	ADAM6	-736936	IGHV7-81	107989		
chr19	13885061	13885426	1.21	8.00E-01	ZSWIM4	-21030	MRI1	9907		
chr12	123459105	123459359	0.94	8.00E-01	OGFOD2	-122				
chr20	32470839	32471201	1.26	8.00E-01	RALY	-110712	CHMP4B	71901		
chr2	204571164	204571474	1.20	8.00E-01	CD28	121				
chr22	23207962	23208432	0.83	8.00E-01	IGL@	-21763	GGTLC2	219415		
chr16	89160069	89160391	1.08	8.01E-01	ACSF3	-24				
chr1	229694518	229694788	1.00	8.01E-01	ABCB10	-211				
chr7	102004077	102004339	1.05	8.01E-01	PRKRIP1	-32596	SH2B2	75803		
chr22	50630901	50631230	1.00	8.01E-01	PANX2	21906	TUBGCP6	52334		
chr16	75681541	75681868	1.02	8.01E-01	KARS	-120	TERF2IP	21		
chr7	102082100	102082551	0.90	8.01E-01	ORAI2	8330	ALKBH4	22962		
chr1	45477657	45477978	0.88	8.01E-01	HECTD3	-791	UROD	-12		
chr1	155220481	155220791	1.00	8.01E-01	GBA	-9583	FAM189B	4638		
chr15	66161277	66161584	1.09	8.01E-01	RAB11A	-365				
chr20	36322034	36322469	1.22	8.01E-01	CTNNBL1	-182				
chr3	194403342	194403671	0.94	8.01E-01	LSG1	-10301	C3orf21	588388		
chr17	72199550	72199871	1.24	8.02E-01	RPL38	-84				
chr1	153518140	153518554	1.16	8.02E-01	S100A5	-4106	S100A4	-65		
chr1	1167278	1167727	0.98	8.02E-01	B3GALT6	-126	SDF4	-56		
chr15	93447334	93447911	1.15	8.02E-01	CHD2	4072	RGMA	184810		

chr2	47882352	47882614	0.95	8.02E-01	MSH6	-127738	KCNK12	-85013
chr21	36399013	36399427	1.10	8.02E-01	C21orf96	12503	CLIC6	357532
chr7	152038182	152038444	1.07	8.02E-01	MLL3	94777	GALNT11	315535
chr1	23855111	23855468	0.70	8.02E-01	ASAP3	-44540	E2F2	2423
chr1	6614415	6614845	0.89	8.02E-01	TAS1R1	-804	NOL9	-49
chr19	11671379	11671689	1.19	8.04E-01	ELOF1	-1483		
chr10	64413022	64413344	1.10	8.04E-01	ADO	-151333	ZNF365	279267
chr19	14530213	14530563	1.14	8.04E-01	DDX39	-217		
chr14	77843609	77843858	0.79	8.04E-01	C14orf148	45645	GSTZ1	56504
chr16	89131563	89131873	1.10	8.04E-01	CBFA2T3	-88317	ACSF3	-28536
chr3	56528766	56529013	1.15	8.04E-01	ERC2	-26499	ARHGEF3	584446
chr9	124734794	124735349	0.84	8.05E-01	TTLL11	120813	DAB2IP	405673
chr21	11128402	11128680	1.13	8.05E-01	BAGE	-29604		
chr3	99979617	99980145	1.08	8.05E-01	TBC1D23	195		
chr7	122184555	122184865	1.09	8.05E-01	FEZF1	-240145	RNF133	154498
chr22	41682306	41682645	1.02	8.05E-01	RANGAP1	-260		
chr4	122745009	122745319	0.78	8.06E-01	CCNA2	-76		
chr5	177547907	177548297	0.93	8.06E-01	N4BP3	7546	NHP2	32859
chr11	66206273	66206687	1.02	8.06E-01	MRPL11	-170		
chr17	8124131	8124441	1.15	8.06E-01	AURKB	-10403	C17orf68	27127
chr3	8700139	8700416	0.84	8.06E-01	CAV3	-75218	LMCD1	156767
chr17	47824522	47824876	0.88	8.06E-01	MYST2	-41372	SLC35B1	-39417
chr17	26972072	26972316	1.06	8.06E-01	KIAA0100	-21		
chr12	25084673	25084912	1.29	8.06E-01	SOX5	-982156	BCAT1	17515
chr9	139622514	139622800	1.04	8.06E-01	LCN10	14757	FAM69B	15633
chr6	13356266	13356572	0.98	8.07E-01	TBC1D7	-27649	GFOD1	131368
chr7	126336753	126337049	1.35	8.07E-01	GRM8	546668		
chr8	100905815	100906059	1.01	8.07E-01	COX6C	-42		
chr12	46780699	46781028	0.92	8.07E-01	SLC38A2	-14219	SLC38A4	438916
chr12	77157830	77158171	1.15	8.07E-01	ZDHHC17	147		
chr17	66233501	66233848	1.17	8.08E-01	AMZ2	-10470	KPNA2	201827
chr2	225450357	225450730	0.85	8.08E-01	CUL3	-434		
chr2	65144815	65145059	1.04	8.08E-01	SERTAD2	-263891	SLC1A4	-71519
chr11	64616214	64616467	1.05	8.08E-01	CDC42BPG	-4300		
chr11	3078570	3078850	1.11	8.09E-01	CARS	-39		
chr20	30697070	30697485	1.07	8.09E-01	TM9SF4	-31		
chr1	67390696	67391081	0.99	8.09E-01	MIER1	311		
chr10	13203170	13203494	0.87	8.09E-01	MCM10	-249		

chr12	120907332	120907754	0.91	8.09E-01	SFRS9	15		
chr1	114889222	114889498	0.91	8.09E-01	SYT6	-192888	TRIM33	164421
chr5	130718105	130718400	1.02	8.09E-01	CDC42SE2	118551	RAPGEF6	252676
chr18	3297431	3297738	1.00	8.10E-01	TGIF1	-154006	MYL12B	35474
chr19	3986206	3986485	0.98	8.11E-01	EEF2	-885		
chr19	58790104	58790395	1.02	8.11E-01	ZNF8	-68		
chr20	29611681	29612019	1.00	8.11E-01	DEFB115	-233617		
chr22	50683370	50683616	0.92	8.11E-01	TUBGCP6	-93		
chr17	62982082	62982637	1.14	8.11E-01	LRRC37A3	-67372	GNA13	70560
chr20	46045420	46045819	1.18	8.11E-01	NCOA3	-85037	ZMYND8	-60146
chr17	30669067	30669377	1.06	8.11E-01	C17orf75	-33		
chr6	43395118	43395610	0.96	8.12E-01	ABCC10	72		
chr17	75865621	75866095	0.90	8.12E-01	TNRC6C	-134460	SEPT9	588366
chr19	9695027	9695337	1.06	8.12E-01	ZNF121	27		
chr4	47465600	47465936	1.02	8.12E-01	COMMD8	-92		
chr12	54752968	54753328	0.91	8.12E-01	GPR84	5110	COPZ1	34237
chr11	111667820	111668086	0.84	8.12E-01	PPP2R1B	-30784	ALG9	74352
chr13	48669156	48669506	0.97	8.12E-01	MED4	-91		
chr12	109490184	109490523	0.91	8.13E-01	USP30	-26		
chr10	103989572	103989854	0.79	8.13E-01	ELOVL3	3570	PITX3	11518
chr1	231376768	231377258	0.87	8.13E-01	GNPAT	94		
chr15	52971340	52971827	0.85	8.13E-01	ARPP19	-110371	ONECUT1	110625
chr17	56406748	56407492	1.29	8.13E-01	BZRAP1	-968		
chr20	62612216	62612474	1.19	8.13E-01	PRPF6	-86		
chr3	101280539	101280896	1.23	8.14E-01	RG9MTD1	6		
chr17	5372272	5372558	1.21	8.14E-01	DHX33	-234		
chr2	9771282	9771592	0.97	8.14E-01	YWHAQ	-331		
chr6	26285407	26285936	0.96	8.14E-01	HIST1H4H	55		
chr11	126350604	126350914	0.94	8.14E-01	ST3GAL4	124663	KIRREL3	520007
chr22	35653290	35653600	0.92	8.14E-01	HMGXB4	0		
chr11	61584224	61584576	1.15	8.14E-01	FADS1	129		
chr8	8602184	8602581	1.01	8.15E-01	CLDN23	42717	MFHAS1	148748
chr16	29827072	29827659	0.97	8.15E-01	MVP	-4421		
chr7	139045094	139045417	1.06	8.15E-01	LUC7L2	622		
chr1	25566522	25566805	0.90	8.15E-01	RHD	-32317	SYF2	-7651
chr1	25573662	25574206	0.91	8.15E-01	RHD	-25047	SYF2	-14921
chr2	61220241	61220574	1.15	8.15E-01	PEX13	-24404	REL	111656
chr1	205304786	205305097	1.17	8.15E-01	NUAK2	-14059	LEMD1	86239

chr11	66360495	66360768	1.02	8.16E-01	CCS	-58		
chr5	93954184	93954540	0.94	8.16E-01	ANKRD32	-29		
chr19	10755480	10755756	0.90	8.16E-01	ILF3	-9319	SLC44A2	19447
chr12	14956445	14956814	1.08	8.16E-01	WBP11	-229		
chr12	49524404	49524687	1.08	8.16E-01	TUBA1B	758		
chr13	53495297	53495649	1.12	8.16E-01	OLFM4	-107499	PCDH8	-72699
chr19	6460555	6460865	1.16	8.16E-01	CRB3	-3550	SLC25A23	-929
chr8	101450283	101450612	0.95	8.16E-01	RNF19A	-134961	ANKRD46	121564
chr17	46185034	46185294	1.17	8.16E-01	SNX11	244		
chr5	40835259	40835569	1.14	8.16E-01	RPL37	-27		
chr2	174856267	174856659	1.03	8.16E-01	SP3	-26400	OLA1	256902
chr5	138945012	138945349	0.93	8.16E-01	CXXC5	-83120	UBE2D2	4430
chr19	41222385	41222832	0.99	8.16E-01	ITPKC	-399	ADCK4	181
chr16	56998482	56998805	0.90	8.16E-01	NLRC5	-24766	CETP	2809
chr1	182808591	182808912	1.05	8.17E-01	DHX9	301		
chr17	28256774	28257081	1.07	8.17E-01	SSH2	90		
chr11	72865179	72865643	1.08	8.17E-01	P2RY2	-63933	FCHSD2	-12268
chr14	54908060	54908364	0.87	8.18E-01	CNIH	-64		
chr6	24581372	24581648	0.95	8.18E-01	KIAA0319	64873	ALDH5A1	86313
chr12	48226014	48226330	1.12	8.18E-01	HDAC7	-12409	VDR	72642
chr17	74733236	74733598	0.93	8.18E-01	MFSD11	-366	SFRS2	-5
chr8	103302197	103302544	1.30	8.18E-01	RRM2B	-51025	UBR5	122124
chr10	135207484	135207757	0.97	8.18E-01	MTG1	0		
chr10	102772644	102772917	0.94	8.18E-01	LZTS2	15816	PDZD7	18098
chr22	24143145	24143456	1.13	8.18E-01	SMARCB1	14151	DERL3	37898
chr16	50840155	50840557	0.90	8.18E-01	CYLD	64395	SALL1	344827
chr19	17966381	17966713	1.07	8.19E-01	RPL18A	-4180		
chr20	33264955	33265296	1.14	8.19E-01	PIGU	-37		
chr11	121293523	121293773	1.21	8.20E-01	SORL1	-29313	SC5DL	130260
chr19	4911745	4912107	0.99	8.20E-01	KDM4B	-57198	UHRF1	2416
chr12	94567448	94567758	1.11	8.20E-01	PLXNC1	25104	TMCC3	476721
chr5	1140436	1140749	1.06	8.20E-01	SLC6A19	-61117	SLC12A7	-28421
chr1	26758704	26758998	0.90	8.21E-01	DHDDS	49		
chr9	131133470	131133754	1.08	8.21E-01	URM1	14		
chr19	44100371	44100783	1.07	8.21E-01	IRGQ	-290	ZNF576	-161
chr1	207997241	207997527	1.22	8.21E-01	CD46	71982	CD34	87299
chr4	108957444	108957754	0.92	8.21E-01	HADH	46659	LEF1	131979
chr6	122792907	122793178	0.92	8.22E-01	SERINC1	-91		

chr2	146169040	146169541	1.02	8.22E-01	ZEB2	-891375		
chr9	19102770	19103246	1.02	8.22E-01	HAUS6	-106		
chr3	187697374	187697744	1.02	8.22E-01	BCL6	-234084	LPP	-233162
chr3	73497905	73498291	1.18	8.22E-01	PDZRN3	175974	FLJ10213	387288
chr8	38801322	38801566	1.23	8.22E-01	HTRA4	-30224	PLEKHA2	42691
chr7	157095098	157095375	1.13	8.23E-01	DNAJB6	-34473	UBE3C	163582
chr17	56017088	56017387	1.14	8.23E-01	MRPS23	-89839	VEZF1	48377
chr12	49449864	49450146	0.89	8.23E-01	MLL2	-898		
chr15	59691667	59692000	0.94	8.24E-01	GCNT3	-212148	MYO1E	-26763
chr2	212290389	212290642	1.00	8.24E-01	CPS1	869190		
chr8	117886934	117887304	1.06	8.24E-01	RAD21	-14		
chr17	27945005	27945266	0.78	8.24E-01	GIT1	-28526	SSH2	311882
chr3	180319924	180320411	0.95	8.24E-01	TTC14	250		
chr6	26597026	26597337	0.95	8.24E-01	ABT1	2		
chr1	90135690	90135964	1.07	8.24E-01	LRRC8D	-151653	LRRC8C	37183
chr11	236791	237097	0.95	8.24E-01	SIRT3	-582	PSMD13	136
chr5	37379220	37379589	0.99	8.24E-01	NUP155	-8208	GDNF	460377
chr6	36725085	36725364	1.02	8.24E-01	CDKN1A	78766	CPNE5	81995
chr7	55625516	55625951	1.00	8.24E-01	VOPP1	14466	LANCL2	192593
chr17	79995074	79995394	0.96	8.24E-01	DCXR	339		
chr16	81856736	81857064	1.15	8.25E-01	PLCG2	43970	SDR42E1	188193
chr2	149402208	149402454	0.83	8.25E-01	EPC2	-229		
chr19	488893	489224	1.09	8.25E-01	SHC2	-28063	MADCAM1	-7431
chr19	36103428	36103769	1.04	8.25E-01	HAUS5	-47		
chr1	224301667	224301922	1.08	8.25E-01	TP53BP2	-268121	DEGS1	-69133
chr15	68132536	68133057	1.06	8.25E-01	PIAS1	-213775	LBXCOR1	14856
chr22	46173483	46173900	1.22	8.25E-01	ATXN10	106005	WNT7B	199316
chr14	68943288	68943538	0.99	8.25E-01	ZFP36L1	316372	RAD51L1	656904
chr12	121790146	121790532	1.02	8.26E-01	ANAPC5	-74		
chr15	40986722	40987158	1.02	8.26E-01	RAD51	-387		
chr12	53473141	53473418	0.76	8.26E-01	SPRYD3	-142		
chr12	58087479	58087986	0.97	8.26E-01	OS9	-153		
chr21	33144760	33145211	0.99	8.26E-01	HUNK	-100642	SFRS15	-40555
chr1	100598421	100598948	1.00	8.26E-01	SASS6	-174	CCDC76	-21
chr6	27156671	27156919	0.89	8.26E-01	PRSS16	-58713	HIST1H2AH	41887
chr4	29056	29530	0.97	8.27E-01	ZNF718	-23934		
chr2	68384561	68385098	0.98	8.27E-01	PNO1	-175	WDR92	-174
chr7	73588388	73588731	0.94	8.27E-01	EIF4H	-146		

chr7	99149602	99149871	0.93	8.27E-01	C7orf38	-21					
chr12	27167284	27167574	0.96	8.27E-01	TM7SF3	-90					
chr16	1661970	1662214	1.04	8.27E-01	CRAMP1L	-2549	IFT140	17			
chr4	38763192	38763568	1.04	8.27E-01	TLR10	21209	KLF3	97590			
chr12	49658725	49659026	1.01	8.27E-01	TUBA1C	11					
chr3	25831414	25831957	1.10	8.27E-01	OXSM	123					
chr1	95320030	95320294	1.10	8.27E-01	SLC44A3	34261	CNN3	72573			
chr18	10490767	10491077	1.05	8.27E-01	NAPG	-34951	APCDD1	36297			
chr6	154996806	154997077	0.98	8.27E-01	CNKSR3	-165189	RBM16	-57570			
chrX	46696178	46696443	0.81	8.27E-01	RP2	-36					
chr19	13227456	13227706	1.04	8.28E-01	NACC1	-1528	TRMT1	-18			
chr14	65453379	65453750	0.94	8.28E-01	FNTB	58					
chr7	98977811	98978095	1.08	8.28E-01	ARPC1B	5624	PDAP1	28339			
chr5	172199417	172199726	0.89	8.29E-01	DUSP1	-1369					
chr17	7590724	7591034	0.93	8.29E-01	TP53	-16					
chr22	29949735	29950033	1.06	8.29E-01	THOC5	-240					
chr8	11271153	11271523	0.97	8.29E-01	BLK	-80183	AMAC1L2	82843			
chr1	11322685	11322995	1.05	8.29E-01	MTOR	-232					
chr20	54967299	54967760	1.06	8.29E-01	AURKA	-179	CSTF1	103			
chr17	7486963	7487686	0.93	8.29E-01	MPDU1	360					
chr3	186733728	186734032	1.11	8.29E-01	ST6GAL1	-5785	ADIPOQ	173417			
chr14	88851765	88852076	1.00	8.29E-01	SPATA7	179					
chr15	49169933	49170412	1.07	8.29E-01	EID1	-117					
chr17	80023592	80023839	1.12	8.30E-01	DUS1L	-36					
chr11	66139788	66140103	1.10	8.30E-01	SLC29A2	-655					
chr9	66458350	66458688	0.94	8.30E-01	FAM75A7	-948909					
chr11	65655799	65656244	0.99	8.30E-01	CCDC85B	-1853	FIBP	-12			
chr15	74782193	74782503	1.14	8.30E-01	ARID3B	-51200	UBL7	-28819			
chr6	27857875	27858309	1.04	8.30E-01	HIST1H2BO	-3111	HIST1H3J	478			
chr4	39367869	39368259	1.04	8.31E-01	RFC1	-69					
chr17	80606247	80606509	1.08	8.31E-01	RAB40B	50220	FOXK2	128784			
chr8	101506830	101507176	0.96	8.31E-01	RNF19A	-191516	ANKRD46	65009			
chr20	30200658	30201001	0.97	8.31E-01	COX4I2	-24861	ID1	7738			
chr22	41842672	41843029	0.92	8.31E-01	TOB2	176					
chr14	64215531	64215893	1.13	8.31E-01	SYNE2	-103971	SGPP1	-20956			
chr4	154388338	154388989	1.09	8.32E-01	TLR2	-216777	KIAA0922	1166			
chr7	156433089	156433581	1.07	8.32E-01	SHH	-828368	RNF32	-66	C7orf13	13	
chr15	37392769	37393051	0.99	8.32E-01	MEIS2	-206					

chr19	10041577	10041830	1.04	8.32E-01	OLFM2	5366	PIN1	95705
chr17	40273284	40273548	1.06	8.32E-01	HSPB9	-1340	KAT2A	-34
chr15	83680296	83680741	1.05	8.32E-01	HOMER2	-59046	BTBD1	55587
chr20	32273080	32273404	1.06	8.33E-01	E2F1	968		
chr12	58176241	58176675	1.13	8.34E-01	TSFM	-78		
chr8	48920905	48921352	1.04	8.34E-01	UBE2V2	134		
chr12	56511759	56512107	1.06	8.35E-01	ZC3H10	-97		
chr15	44955720	44956164	1.11	8.35E-01	SPG11	-66		
chr15	45329331	45329579	0.88	8.35E-01	SORD	14114	DUOX2	76904
chr20	5672605	5672943	1.10	8.35E-01	CHGB	-219200	RP5-1022P6.2	-81102
chr9	36765852	36766189	1.15	8.35E-01	MELK	193116	PAX5	268455
chr1	26324557	26325027	1.07	8.35E-01	PAFAH2	-144		
chr1	46502794	46503039	0.92	8.35E-01	PIK3R3	95463	MAST2	233632
chr8	11659772	11660177	1.05	8.36E-01	FDFT1	-215		
chr5	38453694	38454004	1.13	8.36E-01	LIFR	141658	EGFLAM	195316
chr14	51256530	51256840	0.95	8.36E-01	SAV1	-121662	NIN	41154
chr15	75628218	75628528	0.90	8.36E-01	COMMD4	-1		
chr16	10479643	10479913	0.95	8.36E-01	GRIN2A	-203515	ATF7IP2	-42947
chr17	8286373	8286721	1.00	8.37E-01	RPL26	18		
chr6	44354985	44355416	0.95	8.38E-01	CDC5L	-101		
chr15	64385290	64385600	1.01	8.38E-01	SNX1	-2732		
chr17	4890752	4891063	0.87	8.38E-01	CAMTA2	23		
chr14	23357224	23357534	0.81	8.38E-01	REM2	4947	RBM23	31017
chr5	137371384	137371933	1.05	8.38E-01	FAM13B	-2857		
chr10	121129025	121129389	0.96	8.38E-01	GRK5	162010	RGS10	173015
chr19	1876016	1876307	0.98	8.38E-01	KLF16	-12598	FAM108A1	9356
chr12	76034808	76035118	0.94	8.38E-01	KRR1	-129545	PHLDA1	390593
chr19	35696176	35696541	1.05	8.38E-01	FAM187B	23269	FXYD5	50732
chr7	72298611	72298982	0.94	8.38E-01	TYW1B	16		
chr5	108745687	108746096	0.99	8.38E-01	PJA2	-217		
chr10	65028868	65029246	1.11	8.38E-01	NRBF2	136050	JMJD1C	196665
chr6	26033610	26034033	1.12	8.39E-01	HIST1H3B	-1534	HIST1H2AB	-26
chr5	96519208	96519724	1.08	8.39E-01	RIOK2	-461		
chr16	68344736	68345161	0.88	8.39E-01	PRMT7	4		
chr18	29522874	29523491	0.85	8.39E-01	KIAA1012	-92		
chr9	131084708	131085018	1.04	8.39E-01	TRUB2	-166	COQ4	72
chr1	200379182	200379582	0.92	8.39E-01	ZNF281	-216		
chr7	98923343	98923590	1.02	8.40E-01	ARPC1A	-43		

chr12	102455567	102455917	1.00	8.40E-01	CCDC53	157		
chr1	45956699	45957214	1.07	8.40E-01	TESK2	-117		
chr1	114354807	114355239	0.98	8.40E-01	RSBN1	47		
chr13	99097622	99097925	1.19	8.40E-01	RNF113B	-268253	STK24	131622
chr3	140660302	140660691	1.09	8.40E-01	SLC25A36	-165		
chr6	26103988	26104775	0.94	8.40E-01	HIST1H4C	206		
chr2	198364534	198365011	0.79	8.40E-01	HSPD1	-133	HSPE1	52
chr7	128858447	128858720	0.98	8.40E-01	AHCYL2	-6280	SMO	29871
chr8	37739423	37739778	1.01	8.40E-01	BRF2	-32190	RAB11FIP1	17402
chr15	73925766	73926027	0.98	8.41E-01	NPTN	-144		
chr6	26158073	26158628	1.11	8.41E-01	HIST1H2BD	2		
chr12	57853282	57853637	1.00	8.41E-01	GLI1	-458		
chr5	21058273	21058720	1.28	8.41E-01	GUSBL2	-401092		
chr9	103115090	103115538	1.09	8.41E-01	TEX10	-55		
chr11	65149836	65150163	1.00	8.41E-01	FRMD8	-4041	SLC25A45	142
chr16	66558724	66559047	1.17	8.41E-01	TK2	25429	BEAN	97646
chr17	29876964	29877274	1.17	8.42E-01	RAB11FIP4	158477	C17orf79	309207
chr12	54121241	54121673	0.98	8.42E-01	CALCOCO1	-150		
chr10	97134449	97134703	0.91	8.42E-01	PDLIM1	-83795	SORBS1	66361
chr15	52975800	52976208	0.98	8.42E-01	ARPP19	-114791	ONECUT1	106205
chr1	76251663	76252150	0.93	8.42E-01	RABGGTB	21		
chr15	44719304	44719676	1.00	8.43E-01	CTDSPL2	-89		
chr3	156272715	156273202	1.09	8.43E-01	SSR3	-24		
chr9	71614571	71614905	0.94	8.43E-01	PRKACG	14301	PIP5K1B	294122
chr1	29557316	29557560	0.96	8.43E-01	MECR	16		
chr8	30515697	30515937	1.00	8.43E-01	GTF2E2	-79		
chr6	26189154	26189489	1.16	8.43E-01	HIST1H4D	-18		
chr8	11141779	11142175	0.99	8.43E-01	MTMR9	-23		
chr19	13957397	13957642	0.97	8.44E-01	NANOS3	-30430	ZSWIM4	51246
chr6	30584892	30585495	0.90	8.44E-01	MRPS18B	-292	PPP1R10	-174
chr14	70233636	70233912	1.10	8.44E-01	SFRS5	-60		
chr19	6504843	6505100	1.37	8.44E-01	TUBB4	-2642		
chr11	14913783	14914065	0.84	8.44E-01	CYP2R1	-173		
chr11	64655416	64655662	0.92	8.44E-01	PPP2R5B	-36641	EHD1	-9348
chr16	19533305	19533560	0.91	8.44E-01	CP110	-1746	GDE1	17
chr17	33469081	33469436	1.08	8.44E-01	NLE1	63		
chr2	68589182	68589591	1.03	8.44E-01	PLEK	-2935		
chr3	49377939	49378387	1.05	8.44E-01	USP4	-627		

chr9	2158301	2158649	1.05	8.45E-01	VLDLR	-463318	SMARCA2	143133
chr2	232092087	232092331	1.05	8.45E-01	B3GNT7	-168126	ARMC9	28867
chr2	10518475	10518785	1.02	8.45E-01	ODC1	69823	HPCAL1	75590
chr20	57607308	57607829	1.14	8.45E-01	ATP5E	-147		
chr7	30028072	30028327	1.04	8.46E-01	SCRN1	1705	WIPF3	153859
chr2	61244240	61244550	1.01	8.46E-01	PEX13	-417	PUS10	970
chr6	6584120	6584396	1.12	8.46E-01	LY86	-4676		
chr9	100174093	100174337	1.19	8.46E-01	TDRD7	-87		
chr16	57333716	57334060	1.09	8.46E-01	CCL22	-58830	PLLP	-15317
chr12	50236737	50237016	0.88	8.47E-01	BCDIN3D	35		
chr1	111670939	111671298	1.05	8.48E-01	C1orf103	-164553	CEPT1	-11130
chr7	126341136	126341420	1.19	8.49E-01	GRM8	542291		
chr2	28974188	28974582	1.06	8.49E-01	PPP1CB	-229		
chr7	135662027	135662477	1.25	8.49E-01	MTPN	-48		
chr1	161735841	161736339	1.12	8.49E-01	ATF6	6		
chr17	42610912	42611240	1.11	8.49E-01	GPATCH8	-30274	FZD2	-23849
chr2	233965299	233965775	1.05	8.49E-01	ATG16L1	-194680	INPP5D	40501
chr15	93352361	93352940	1.14	8.50E-01	FAM174B	-153620	CHD2	-90900
chr22	21996323	21996709	1.05	8.50E-01	SDF2L1	-26		
chr7	74071486	74071880	0.78	8.50E-01	GTF2I	-347		
chr19	14191862	14192396	1.03	8.51E-01	PALM3	-22158	SAMD1	9103
chr11	66085478	66085714	0.90	8.51E-01	CD248	-1081		
chr17	80452325	80452855	1.12	8.51E-01	FOXK2	-25004	NARF	36050
chr2	242041607	242041930	1.06	8.51E-01	PASK	47109	SNED1	103514
chr22	41042299	41042540	0.84	8.51E-01	MCHR1	-32762	MKL1	-9730
chr5	176778569	176778925	0.97	8.51E-01	LMAN2	138		
chr6	155502708	155502979	1.19	8.51E-01	CLDN20	-82303	TIAM2	91421
chr8	37489191	37489427	1.01	8.51E-01	ZNF703	-63992	FKSG2	743312
chr13	52027007	52027293	0.98	8.52E-01	INTS6	125		
chr13	29233023	29233308	1.11	8.52E-01	POMP	-75		
chr16	30621690	30621960	0.98	8.52E-01	ZNF689	-143		
chr2	203776172	203776479	1.18	8.53E-01	ALS2CR8	-652	WDR12	623
chr12	53894496	53894829	0.84	8.53E-01	MAP3K12	-1392	TARBP2	-714
chr2	172864642	172864997	1.08	8.53E-01	MAP1D	16		
chr6	42185477	42185778	1.01	8.53E-01	MRPS10	5		
chr8	56757093	56757339	0.87	8.53E-01	TMEM68	-71331	LYN	-35170
chr14	53019285	53019565	0.94	8.53E-01	GPR137C	-441	TXNDC16	-124
chr7	129251339	129251662	0.83	8.53E-01	NRF1	-54		

chr22	24823335	24823633	1.01	8.53E-01	ADORA2A	-4335	ADORA2A	-46
chr20	46041446	46041783	1.08	8.53E-01	NCOA3	-89042	ZMYND8	-56141
chr14	45430943	45431694	1.04	8.54E-01	KLHL28	-140	FAM179B	-97
chr19	17377857	17378312	0.91	8.55E-01	USHBP1	-2541	C19orf62	-147
chr1	220445742	220446115	1.05	8.55E-01	RAB3GAP2	-86		
chr16	14580673	14581048	0.94	8.55E-01	PARN	143263	MKL2	415665
chr14	90422039	90422371	0.97	8.55E-01	C14orf143	-1116	TDP1	-41
chr1	27648418	27648900	0.93	8.56E-01	TMEM222	23		
chr6	28109432	28109728	1.16	8.56E-01	ZNF192	-136		
chr3	111851928	111852262	1.05	8.56E-01	GCET2	22		
chr1	108319956	108320266	0.88	8.56E-01	VAV3	187434	NTNG1	637482
chr1	192578155	192578494	1.11	8.56E-01	RGS13	-26957	RGS1	33468
chr20	35724322	35724702	1.23	8.56E-01	RBL1	-102		
chr12	53845524	53845900	1.04	8.56E-01	PCBP2	-174		
chr6	45645152	45645538	1.10	8.56E-01	SUPT3H	-299675	CLIC5	402740
chr17	42092208	42092542	1.08	8.57E-01	TMEM101	-30		
chr17	73098519	73098788	1.03	8.57E-01	ARMC7	-7428	SLC16A5	14599
chr5	139012905	139013169	1.02	8.57E-01	CXXC5	-15264	UBE2D2	72286
chr10	1531343	1531619	1.06	8.58E-01	IDI1	-436420	ADARB2	248237
chr9	33264850	33265117	0.91	8.58E-01	BAG1	-223	CHMP5	107
chr2	198380525	198380979	0.94	8.58E-01	MOBKL3	-19		
chr17	66243726	66244133	0.96	8.59E-01	AMZ2	-215		
chr5	180687880	180688256	0.96	8.59E-01	TRIM52	51		
chr17	57642682	57643041	1.13	8.59E-01	DHX40	-24		
chr16	1763961	1764252	0.99	8.59E-01	MAPK8IP3	7886	NME3	57603
chr11	82782858	82783254	1.03	8.59E-01	RAB30	-172		
chr6	45345655	45345967	1.04	8.59E-01	SUPT3H	-141		
chr12	112825210	112825455	1.01	8.59E-01	C12orf51	-81295	RPL6	22110
chr4	597980	598256	1.17	8.59E-01	ZNF721	-104676	PDE6B	-21245
chr11	8008469	8008872	0.88	8.60E-01	EIF3F	226		
chr15	40882018	40882317	0.93	8.60E-01	CASC5	-4279		
chr18	54744928	54745377	1.12	8.60E-01	ST8SIA3	-274568	WDR7	426537
chr3	160116811	160117166	1.11	8.60E-01	SMC4	-441	IFT80	331
chr10	104180749	104181059	0.93	8.60E-01	PSD	-2003		
chr12	112450934	112451307	0.97	8.60E-01	TMEM116	-186	ERP29	-31
chr2	231280764	231281436	1.12	8.60E-01	SP100	229		
chr19	4400578	4400921	1.01	8.60E-01	CHAF1A	-1910	SH3GL1	-279
chr5	143567390	143567652	0.90	8.60E-01	KCTD16	17084		

chr5	90678974	90679299	1.03	8.61E-01	ARRDC3	12		
chr7	23636839	23637179	1.11	8.62E-01	CCDC126	11		
chr7	101625269	101625635	1.03	8.62E-01	SH2B2	-302953	CUX1	164570
chr11	62649035	62649304	1.03	8.63E-01	SLC3A2	25652	CHRM1	39842
chr8	37738373	37738633	1.08	8.63E-01	BRF2	-31092	RAB11FIP1	18500
chr3	137906015	137906437	1.22	8.64E-01	ARMC8	78		
chr1	110880934	110881979	0.87	8.64E-01	RBM15	-488		
chr1	35658522	35659037	0.98	8.64E-01	SFPQ	-37		
chr2	27805707	27806145	1.14	8.64E-01	ZNF512	33		
chr12	57856319	57856829	0.90	8.64E-01	GLI1	2656	ARHGAP9	17059
chr3	186734258	186734611	0.94	8.65E-01	ST6GAL1	-5230	ADIPOQ	173972
chr7	105662868	105663337	1.15	8.65E-01	FLJ23834	59446	SYPL1	89954
chr6	136610891	136611197	1.05	8.65E-01	BCLAF1	-55		
chr19	2085333	2085661	1.06	8.65E-01	MKNK2	-34254	MOBKL2A	10772
chr19	18209608	18209979	1.12	8.65E-01	PIK3R2	-54222	MAST3	1191
chr5	68513544	68513905	0.96	8.65E-01	MRPS36	152		
chr13	27803814	27804058	1.17	8.65E-01	USP12	-57907	RPL21	-21756
chr9	140163809	140164192	0.93	8.65E-01	C9orf167	-8279	COBRA1	14242
chrX	149106685	149107167	1.21	8.65E-01	MAMLD1	-424760	HSFX1	-251153
chr20	62496191	62496699	0.88	8.66E-01	TPD52L2	-145		
chr9	32550954	32551428	1.02	8.66E-01	DDX58	-24869	TOPORS	1410
chr3	130612659	130612993	1.11	8.66E-01	ATP2C1	-608		
chr6	35438321	35438729	1.36	8.66E-01	RPL10A	2347	TEAD3	26336
chr6	170863231	170863520	1.04	8.66E-01	PSMB1	-959	TBP	-95
chr2	55496207	55496553	1.07	8.66E-01	MTIF2	4		
chr5	167755785	167756223	1.09	8.67E-01	RARS	-157459	WWC1	36939
chr13	111365275	111365833	1.05	8.67E-01	ING1	-1805		
chr2	219744697	219745032	1.13	8.67E-01	WNT10A	-390		
chr1	149859731	149860230	1.14	8.67E-01	HIST2H2BE	-1749	HIST2H2AB	-515
chr6	15151424	15151750	1.01	8.68E-01	JARID2	-94940		
chr5	171538317	171538564	1.11	8.68E-01	FBXW11	-104564	STK10	76905
chr14	35451498	35451853	0.95	8.68E-01	SRP54	-428		
chr1	36863431	36863685	1.11	8.68E-01	LSM10	-65		
chr15	78730526	78730848	0.89	8.68E-01	IREB2	169		
chr11	43665604	43665914	1.13	8.69E-01	HSD17B12	-36384	TTC17	285268
chr4	2698158	2698553	1.12	8.69E-01	TNIP2	59747	RNF4	227549
chr2	214018416	214018714	0.97	8.69E-01	IKZF2	-3507		
chr11	59040929	59041207	1.05	8.69E-01	OR5AN1	-90864	MPEG1	-60574

chr17	8042134	8042458	1.03	8.69E-01	HES7	-14894	PER1	13457
chr15	90627033	90627434	0.92	8.69E-01	IDH2	18474	ZNF710	82482
chr17	65713756	65714076	1.08	8.69E-01	NOL11	-145		
chr18	10520140	10520382	1.11	8.70E-01	NAPG	-5612	APCDD1	65636
chr1	111991824	111992085	1.00	8.70E-01	WDR77	-125	ATP5F1	212
chr1	46153668	46153931	0.95	8.70E-01	GPBP1L1	-1498	TMEM69	-47
chr15	64648331	64648789	1.08	8.71E-01	CSNK1G1	-118		
chr9	34989090	34989446	0.94	8.71E-01	DNAJB5	-999		
chr3	157827571	157828009	1.10	8.71E-01	SHOX2	-3838	RSRC1	-102
chr3	31267870	31268180	0.84	8.72E-01	GADL1	-331872	STT3B	-306466
chr3	4534753	4535208	1.06	8.72E-01	ITPR1	-53		
chr19	6862750	6863071	1.03	8.72E-01	EMR1	-24671	VAV1	90189
chr1	204485242	204485651	1.08	8.73E-01	MDM4	-64		
chr6	43484530	43484893	0.95	8.73E-01	POLR1C	-79	YIPF3	-10
chr2	43358582	43358949	1.11	8.73E-01	HAAO	-339015	ZFP36L2	94979
chr2	27906397	27906652	1.02	8.73E-01	MRPL33	-88059	SUPT7L	-20076
chr17	8023557	8023828	1.10	8.74E-01	ALOXE3	-1833		
chr1	226111765	226112099	1.00	8.74E-01	PYCR2	33		
chr14	69864994	69865263	1.17	8.74E-01	SLC39A9	-278	ERH	-108
chr14	90722742	90723000	1.10	8.74E-01	PSMC1	-23		
chr17	42767021	42767373	0.99	8.74E-01	DBF4B	-18779	FZD2	132272
chr2	198063571	198063920	1.00	8.74E-01	ANKRD44	-984		
chr9	130477920	130478369	1.09	8.75E-01	TTC16	-213		
chr19	18365797	18366248	0.86	8.75E-01	PDE4C	-7013	KIAA1683	19296
chr14	21560651	21560971	1.04	8.75E-01	C14orf176	-6285	FLJ10357	22284
chr1	205090913	205091648	1.04	8.75E-01	RBBP5	-150		
chr19	35821335	35821732	1.23	8.76E-01	FFAR1	-20911	CD22	1455
chr4	81532021	81532353	0.99	8.76E-01	BMP3	-419932	FGF5	344445
chr4	148605286	148605591	1.19	8.76E-01	PRMT10	-159		
chr17	1123655	1123947	1.07	8.76E-01	TUSC5	-59156	ABR	-40670
chr17	47269245	47269532	0.97	8.76E-01	GNGT2	17354	B4GALNT2	59060
chr2	68954195	68954439	1.11	8.77E-01	ARHGAP25	-7651	PROKR1	81523
chr7	151855898	151856224	1.14	8.77E-01	GALNT11	133283	MLL3	277029
chr5	81046742	81047081	0.99	8.77E-01	SSBP2	160		
chr9	116342864	116343329	0.84	8.77E-01	ZNF618	-295465	RGS3	136086
chr1	167597275	167597647	1.06	8.77E-01	MPZL1	-93726	CREG1	-74405
chr11	62608990	62609304	1.06	8.77E-01	WDR74	-1519		
chr5	14287265	14287637	1.02	8.78E-01	TRIO	143622	ANKH	584436

chr10	11720176	11720451	0.96	8.78E-01	USP6NL	-146040	ECHDC3	-64042
chr16	71496058	71496306	0.92	8.78E-01	ZNF23	-65		
chr18	44711052	44711362	0.88	8.78E-01	IER3IP1	-8462	CORL2	64347
chr16	28956938	28957394	1.05	8.79E-01	NFATC2IP	-5152	CD19	13906
chr6	53275270	53275666	1.15	8.79E-01	ELOVL5	-61526	GCLC	134363
chr6	27446442	27446791	0.91	8.79E-01	ZNF184	-5720	HIST1H2BL	329092
chr1	155034007	155034327	0.97	8.79E-01	EFNA3	-17181	ADAM15	10405
chr2	220109951	220110368	1.07	8.79E-01	STK16	-32	GLB1L	-29
chr19	41283566	41284107	1.02	8.79E-01	RAB4B	-334		
chr17	73008429	73008941	1.01	8.79E-01	ICT1	-95		
chr21	38639595	38640047	1.08	8.79E-01	DSCR3	12		
chr15	79888439	79888793	1.28	8.79E-01	KIAA1024	163758	MTHFS	300754
chr4	83955917	83956339	1.06	8.79E-01	COPS4	-111		
chr7	129691050	129691360	1.15	8.79E-01	ZC3HC1	28		
chr16	18812602	18813468	0.95	8.80E-01	ARL6IP1	-178		
chr12	111742399	111742709	1.14	8.80E-01	SH2B3	-101198	CUX2	270725
chr7	66309842	66310115	1.02	8.80E-01	C7orf42	-76224	RABGEF1	104996
chr3	16475144	16475493	1.11	8.81E-01	RFTN1	79903	OXNAD1	168605
chr3	10289790	10290204	0.99	8.81E-01	TATDN2	-180		
chr6	155054326	155054667	0.81	8.81E-01	RBM16	-15		
chr12	111136103	111136388	1.16	8.81E-01	HVCN1	-9300	PPP1CC	44511
chr11	88070832	88071116	1.02	8.81E-01	CTSC	-33		
chr20	55973283	55973779	1.12	8.81E-01	RBM38	7068	HMGB1L1	90552
chr21	45663858	45664185	1.18	8.81E-01	ICOSLG	-3188		
chr3	133196386	133196683	1.06	8.81E-01	CDV3	-95899	BFSP2	77745
chr3	143247877	143248175	1.11	8.81E-01	SLC9A9	319320	CHST2	409358
chr3	156878360	156878776	1.07	8.81E-01	CCNL1	-86		
chr9	91925507	91926232	1.03	8.81E-01	CKS2	-243		
chr6	27858333	27858672	1.13	8.81E-01	HIST1H2BO	-2700	HIST1H3J	67
chr5	156913320	156913631	0.95	8.82E-01	NIPAL4	26449	ADAM19	89292
chr13	67568414	67568658	0.97	8.82E-01	PCDH9	235932		
chr19	50871815	50872155	0.96	8.82E-01	NAPSA	-3054		
chr3	187802985	187803493	1.02	8.82E-01	BCL6	-339764	LPP	-127482
chr15	91445532	91445894	1.06	8.82E-01	MAN2A2	-1707		
chr11	74204184	74204469	1.02	8.82E-01	MGC12965	-158	LIPT2	428
chr15	77228022	77228350	1.38	8.82E-01	PSTPIP1	-59279	RCN2	4224
chr15	64679851	64680161	0.99	8.82E-01	TRIP4	-14		
chr12	110434063	110434373	1.14	8.82E-01	GIT2	-24		

chr7	142506514	142506828	0.96	8.82E-01	EPHB6	-46121	TCRBC2	12304
chr1	203294952	203295228	1.01	8.82E-01	BTG2	20426	FMOD	25199
chr10	6092810	6093289	1.07	8.82E-01	IL15RA	-73513	IL2RA	11222
chr2	89131278	89131648	0.93	8.82E-01	O1/O11 and JK2	-759099	RPIA	140287
chr11	9595035	9595350	1.05	8.83E-01	WEE1	-35		
chr14	90863192	90863510	0.84	8.83E-01	CALM1	-22		
chr17	40829042	40829368	0.93	8.83E-01	PLEKHH3	-157		
chr5	149829524	149829834	0.97	8.83E-01	RPS14	-360		
chr8	95487271	95487520	1.08	8.83E-01	RAD54B	-86		
chr19	8082730	8083006	1.25	8.83E-01	CCL25	-35016	ELAVL1	-12339
chr2	213727874	213728184	1.10	8.83E-01	ERBB4	-324677	IKZF2	287029
chr6	144537524	144537834	1.00	8.83E-01	UTRN	-75194	STX11	66025
chr4	25161955	25162633	1.01	8.83E-01	SEPSECS	-90		
chr5	154320492	154320835	1.11	8.83E-01	GEMIN5	-2888	MRPL22	31
chr3	53925878	53926263	1.01	8.83E-01	SELK	-82		
chr16	22309570	22309998	1.11	8.83E-01	POLR3E	1043	CDR2	76154
chr3	122101779	122102310	1.01	8.83E-01	FAM162A	-978	CCDC58	29
chr12	50056780	50057102	0.95	8.83E-01	PRPF40B	32613	FMNL3	44256
chr11	121163084	121163516	1.04	8.83E-01	SC5DL	-88		
chr3	150125914	150126182	0.98	8.83E-01	TSC22D2	-740		
chr1	203268354	203268682	0.96	8.83E-01	CHIT1	-69658	BTG2	-6146
chr11	57479487	57480170	1.18	8.83E-01	TMX2	-213	MED19	-156
chr13	111837093	111837441	1.05	8.83E-01	C13orf16	-135748	ARHGEF7	69643
chr12	58146159	58146573	1.03	8.84E-01	MARCH9	-2534	CDK4	-202
chr15	90605215	90605589	1.08	8.84E-01	IDH2	40306	ZNF710	60650
chr17	8125514	8125768	1.06	8.84E-01	AURKB	-11758	C17orf68	25772
chr1	161015621	161015971	1.02	8.84E-01	USF1	-39		
chr17	57287185	57287495	1.19	8.84E-01	C17orf71	-31		
chr10	49879716	49880034	0.93	8.84E-01	ARHGAP22	-66737	WDFY4	-13643
chr14	105957131	105957704	0.99	8.84E-01	TMEM121	-35535	CRIP1	4161
chr19	1068747	1069417	0.99	8.84E-01	HMHA1	1908	POLR2E	26309
chr3	142926164	142926538	1.19	8.84E-01	CHST2	87683	SLC9A9	640995
chr15	40401123	40401422	1.17	8.85E-01	BMF	-198		
chr10	363284	363559	1.00	8.85E-01	ZMYND11	182998	DIP2C	372186
chr19	16251937	16252247	1.02	8.85E-01	HSH2D	7254	CIB3	32194
chr21	45574995	45575378	0.99	8.85E-01	C21orf33	21693	ICOSLG	85647
chr4	4945716	4946099	0.92	8.85E-01	CYTL1	75289	MSX1	84516
chr6	37070237	37070880	0.91	8.85E-01	PIM1	-67363	FGD2	97136

chr8	67025410	67025790	0.92	8.85E-01	TRIM55	-13678	DNAJC5B	91809		
chr2	73461246	73461670	0.99	8.86E-01	C2orf7	-1102	CCT7	53		
chr2	95831333	95831863	0.95	8.86E-01	ZNF2	415				
chr3	184529682	184530059	0.95	8.86E-01	VPS8	-60				
chr7	100808739	100809129	0.92	8.86E-01	VGF	-82				
chr12	54411502	54412021	1.00	8.86E-01	HOXC6	-10432	HOXC8	8872		
chr16	14726467	14726908	0.96	8.86E-01	PARN	-2564	BFAR	20		
chr17	5389706	5390131	1.06	8.86E-01	DERL2	-425	MIS12	-328		
chr5	14664374	14664835	0.93	8.86E-01	ANKH	207282	TRIO	520776		
chr12	32831980	32832224	1.03	8.86E-01	DNM1L	-35				
chr16	2073189	2073458	0.90	8.86E-01	SLC9A3R2	-3564				
chr8	125649776	125650092	1.00	8.86E-01	MTSS1	90796	NDUFB9	98591		
chr11	65756501	65756745	1.01	8.86E-01	BANF1	-12927	SART1	27463		
chr1	111762472	111762777	1.24	8.86E-01	DRAM2	-79787	CHI3L2	-7656		
chr2	47951380	47951796	0.94	8.86E-01	KCNK12	-154118	MSH6	-58633		
chr1	74663971	74664281	0.86	8.87E-01	LRRIQ3	-255	TNNI3K	200		
chr12	59989528	59989804	0.83	8.87E-01	LRIG3	-675404	SLC16A7	-93460		
chr12	58165771	58166040	1.03	8.87E-01	CYP27B1	-4930	FAM119B	-477	METTL1	8
chr9	135282152	135282388	1.01	8.87E-01	TTF1	-49				
chr13	46626795	46627117	0.94	8.87E-01	ZC3H13	-62				
chr17	40730396	40730829	1.10	8.87E-01	PSMC3IP	-866				
chr19	10978652	10978910	0.92	8.87E-01	CARM1	-3472				
chr2	63815455	63816190	0.92	8.87E-01	MDH1	-299				
chr3	183273293	183273701	1.05	8.87E-01	KLHL6	2				
chr7	140396649	140396893	0.90	8.87E-01	NDUFB2	290				
chr10	121632376	121632806	0.87	8.87E-01	SEC23IP	-19632	INPP5F	146982		
chr17	8090037	8090412	1.14	8.87E-01	TMEM107	-10511	C17orf59	3339		
chr17	27053947	27054261	0.99	8.87E-01	NEK8	-1728	TLCD1	-874		
chr17	45214444	45214754	0.84	8.87E-01	RPRML	-157985	CDC27	52066		
chr3	133931813	133932092	1.06	8.87E-01	SLCO2A1	-183033	RYK	37633		
chr19	17622871	17623225	1.09	8.88E-01	PGLS	616				
chr14	55240750	55241036	0.83	8.88E-01	GCH1	128649	SAMD4A	206256		
chr7	97501462	97501897	0.88	8.88E-01	ASNS	174				
chr5	142972207	142972570	0.97	8.88E-01	NR3C1	-188344	YIPF5	577889		
chr16	30064003	30064540	0.96	8.89E-01	FAM57B	-22086	ALDOA	-11328		
chr11	64619257	64619584	1.13	8.89E-01	CDC42BPG	-7380	EHD1	26770		
chr10	97989322	97989632	0.89	8.89E-01	BLNK	41856	ZNF518A	100005		
chr17	10600705	10601024	0.95	8.89E-01	C17orf48	-62	SCO1	20		

chr12	46122527	46122932	0.84	8.89E-01	ARID2	-890		
chr2	145461493	145461803	1.00	8.89E-01	ZEB2	-183732		
chr4	26994294	26994538	0.94	8.89E-01	STIM2	132052		
chr17	56405584	56405894	1.00	8.90E-01	BZRAP1	413		
chr2	85154015	85154392	1.03	8.90E-01	KCMF1	-44027	TMSB10	21441
chr15	75660797	75661160	1.12	8.90E-01	MAN2C1	-38		
chr6	139696390	139696754	0.86	8.90E-01	CITED2	-787		
chr16	75182176	75182438	1.09	8.90E-01	ZFP1	-114		
chr13	37573329	37573613	1.10	8.90E-01	EXOSC8	-1207	ALG5	33
chr12	54762470	54762746	0.96	8.91E-01	GPR84	-4350		
chr1	39338938	39339495	0.96	8.91E-01	MYCBP	-167		
chr17	2304017	2304598	0.97	8.91E-01	MNT	-50		
chr13	41579019	41579289	1.32	8.91E-01	ELF1	14354	SLC25A15	215607
chr14	92572985	92573282	1.02	8.91E-01	ATXN3	-169		
chr19	15529749	15530250	1.04	8.91E-01	AKAP8L	-167		
chr2	97405707	97406037	1.00	8.91E-01	LMAN2L	-59		
chr2	197076046	197076367	1.01	8.91E-01	STK17B	-39871	HECW2	381128
chr3	194785073	194785439	1.12	8.91E-01	LSG1	-392050	C3orf21	206639
chr15	68569885	68570195	0.97	8.91E-01	FEM1B	-101		
chr6	24359941	24360464	0.94	8.91E-01	DCDC2	-1923		
chr5	53606243	53606665	1.10	8.92E-01	ARL15	-51		
chr10	13413972	13414230	0.89	8.92E-01	SEPHS1	-23821	BEND7	130875
chr13	30945779	30946230	1.04	8.92E-01	KATNAL1	-64842	HMGB1	94076
chr16	57220115	57220373	0.84	8.92E-01	FAM192A	-268	RSPRY1	3
chr17	46969962	46970370	1.09	8.92E-01	ATP5G1	18		
chr19	49474182	49474631	0.93	8.92E-01	FTL	5841	GYS1	22203
chr1	24306852	24307118	1.09	8.92E-01	SFRS13A	-164		
chr10	95462272	95462540	0.95	8.92E-01	C10orf4	-77		
chr5	72143781	72144032	0.95	8.92E-01	TNPO1	-23		
chr3	183695274	183695616	1.34	8.92E-01	PARL	-92752	ABCC5	40282
chr5	108804541	108804807	1.05	8.92E-01	MAN2A1	-220482	PJA2	-58999
chr12	7053803	7054113	1.02	8.92E-01	PTPN6	-1782		
chr10	104556165	104556425	0.89	8.92E-01	CYP17A1	40995	C10orf26	52568
chr6	25006815	25007147	1.08	8.92E-01	FAM65B	-95786	DKFZp686H12134	131070
chr11	77790829	77791139	1.16	8.93E-01	NDUFC2	281		
chr8	145634603	145634925	1.03	8.93E-01	CPSF1	-31		
chr4	76598576	76598963	1.05	8.93E-01	G3BP2	-103		
chr6	68160591	68160928	0.99	8.93E-01				

chr1	25071400	25071695	0.82	8.93E-01	CLIC4	-212		
chr7	148663912	148664148	0.87	8.93E-01	EZH2	-82616	PDIA4	61752
chr15	40453015	40453266	1.20	8.93E-01	BUB1B	-69		
chr12	54674317	54674840	0.87	8.94E-01	HNRNPA1	91		
chr12	72079712	72080151	0.88	8.94E-01	TMEM19	54		
chr16	57496372	57496714	1.03	8.94E-01	POLR2C	-8		
chr5	180649246	180649636	1.10	8.94E-01	TRIM41	-865		
chr16	67875749	67876034	0.96	8.94E-01	NUTF2	-4927	THAP11	-321
chr6	33385707	33386415	0.97	8.94E-01	SYNGAP1	-1786	CUTA	4
chr1	43637865	43638134	0.88	8.94E-01	EBNA1BP2	241		
chr17	48474678	48475077	1.06	8.94E-01	LRRC59	-46		
chr19	52531568	52532045	1.15	8.94E-01	ZNF614	-127		
chr2	162164741	162165054	1.08	8.94E-01	PSMD14	112		
chr1	33592744	33593106	1.17	8.94E-01	ADC	46211	TRIM62	54746
chr12	14320042	14320318	1.01	8.94E-01	ATF7IP	-198431	GRIN2B	-187158
chr19	16699224	16699538	1.08	8.94E-01	SLC35E1	-16188	MED26	39634
chr2	113484331	113484650	0.97	8.94E-01	NT5DC4	5428	IL1A	58480
chr2	219433102	219433610	1.05	8.94E-01	RQCD1	-322	USP37	-272
chr6	27100207	27100973	1.09	8.94E-01	HIST1H2AG	-227	HIST1H2BJ	-15
chr1	45285243	45285982	1.08	8.94E-01	BTBD19	11459	PTCH2	23003
chr16	699193	699504	0.94	8.94E-01	RHOT2	-18784	FAM195A	7500
chr20	32307841	32308235	1.07	8.95E-01	PXMP4	98		
chr1	222886554	222886811	1.01	8.95E-01	AIDA	-819	C1orf58	777
chr4	169737989	169738266	0.94	8.96E-01	CBR4	193294	PALLD	319911
chr16	75599032	75599278	1.14	8.97E-01	GABARAPL2	-1094		
chr4	26861973	26862505	1.01	8.97E-01	STIM2	-125		
chr17	45068974	45069322	0.92	8.97E-01	RPRML	-12534	CDC27	197517
chr2	220142393	220142869	0.97	8.97E-01	DNAJB2	-1409		
chr11	65686659	65687054	1.16	8.97E-01	DRAP1	129		
chr12	2999774	3000060	0.78	8.97E-01	TULP3	-116		
chr9	127177657	127177991	1.08	8.97E-01	PSMB7	-103		
chr9	130879948	130880222	0.94	8.97E-01	PTGES2	10389	SLC25A25	49606
chr7	44529959	44530257	0.87	8.97E-01	CAMK2B	-164878	NPC1L1	50806
chr7	73686881	73687207	0.97	8.98E-01	RFC2	-18306	CLIP2	-16761
chr9	74526104	74526675	0.94	8.98E-01	FAM108B1	-242		
chr7	149321735	149322048	0.91	8.98E-01	ZNF746	-126994	ZNF467	148403
chr9	114660181	114660457	1.19	8.98E-01	UGCG	1113	SUSD1	277237
chr5	176852787	176853090	1.05	8.98E-01	GRK6	-748		

chr17	79935170	79935614	1.09	8.98E-01	ASPSCR1	-34		
chr10	74056950	74057408	1.08	8.98E-01	DDIT4	23502	DNAJB12	57728
chr8	22932195	22932447	0.91	8.99E-01	TNFRSF10C	-28113	TNFRSF10B	-5621
chr8	42150225	42150583	1.06	8.99E-01	POLB	-45626	IKBKB	21575
chr16	69458351	69458675	1.04	8.99E-01	CYB5B	15		
chr3	47017870	47018305	1.00	9.00E-01	NBEAL2	-3085		
chr5	150826996	150827322	0.95	9.00E-01	SLC36A1	-4		
chr12	6832891	6833337	0.99	9.00E-01	COPS7A	-66		
chr14	21924370	21924706	1.11	9.00E-01	CHD8	-19134	RAB2B	20594
chr6	41888741	41889022	0.94	9.00E-01	BYSL	-83	MED20	-5
chr13	41556975	41557219	1.13	9.01E-01	ELF1	36411	SLC25A15	193550
chr2	74710021	74710269	1.02	9.01E-01	TTC31	-55	CCDC142	212
chr19	2819648	2819972	1.03	9.01E-01	ZNF554	-62		
chr15	91204512	91204822	1.07	9.01E-01	BLM	-55912	CRTC3	131469
chr17	73341890	73342233	1.04	9.01E-01	SLC25A19	-56532	GRB2	59728
chr16	31128788	31129098	0.95	9.01E-01	MYST1	-42		
chr17	7259397	7259655	1.09	9.01E-01	TNK1	-24839	TMEM95	1029
chr8	82024068	82024429	0.84	9.01E-01	PAG1	54		
chr6	26027052	26027697	1.05	9.02E-01	HIST1H4B	105		
chr14	89850414	89850740	0.99	9.02E-01	FOXN3	234917	TTC8	559659
chr8	71314794	71315059	1.23	9.02E-01	PRDM14	-331365	NCOA2	1093
chr15	40861275	40861632	1.12	9.03E-01	RPUSD2	-83		
chr9	33473874	33474281	0.99	9.03E-01	NOL6	-137		
chr9	131534131	131534393	1.03	9.03E-01	ZER1	-64		
chr1	205601071	205601345	1.00	9.03E-01	SLC45A3	48422	MFSD4	63096
chr10	11205232	11205508	0.90	9.03E-01	CUGBP2	145477	USP6NL	368904
chr11	207238	207742	0.93	9.03E-01	RIC8A	-1040	BET1L	-68
chr12	14460402	14460647	1.06	9.03E-01	GRIN2B	-327503	ATF7IP	-58086
chr7	73097763	73098044	1.03	9.03E-01	DNAJC30	-123	WBSCR22	6
chr12	14518265	14518558	1.05	9.03E-01	ATF7IP	-199		
chr22	38082172	38082482	1.01	9.04E-01	NOL12	-17		
chr8	41685836	41686168	0.99	9.04E-01	NKX6-3	-181127	ANK1	68278
chr10	6244609	6244998	0.89	9.04E-01	PFKFB3	-36		
chr12	111325850	111326160	0.91	9.04E-01	PPP1CC	-145248	MYL2	32399
chr4	170533349	170533832	0.97	9.04E-01	NEK1	23		
chr11	65222472	65222937	1.05	9.04E-01	SCYL1	-69843	FRMD8	68664
chr1	62901935	62902312	0.95	9.05E-01	USP1	149		
chr16	66994958	66995212	0.89	9.05E-01	CES3	-53		

chr7	44162845	44163085	0.96	9.05E-01	POLD2	182		
chr8	27631733	27631996	1.02	9.05E-01	ESCO2	-193		
chr17	73629022	73629337	0.93	9.05E-01	KIAA1783	20125	RECQL5	34089
chr2	162016802	162017220	1.05	9.05E-01	PSMD14	-147775	TANK	23545
chr1	38455569	38455970	0.90	9.05E-01	SF3A3	-9		
chr8	74884362	74884769	1.11	9.05E-01	TMEM70	-3864	TCEB1	-220
chr1	66708431	66708675	0.97	9.05E-01	SGIP1	-291272	PDE4B	449697
chr3	182817215	182817597	1.11	9.06E-01	MCCC1	-41		
chr11	119130547	119131026	1.22	9.06E-01	CBL	53797	MCAM	57053
chr11	46615486	46615796	0.93	9.07E-01	AMBRA1	-2727		
chr17	7591419	7591740	1.09	9.07E-01	TP53	-717		
chr3	71591752	71592187	1.09	9.07E-01	FOXP1	41170		
chr19	3761436	3762138	0.91	9.07E-01	MRPL54	-878	APBA3	-114
chr17	47633693	47634003	0.98	9.07E-01	NXPH3	-19450	NGFR	61193
chr3	18265054	18265375	0.98	9.07E-01	TBC1D5	-480975	SATB1	215037
chrX	149108642	149108892	0.99	9.07E-01	MAMLD1	-422919	HSFX1	-252994
chr10	74927747	74927987	1.03	9.08E-01	ECD	-14		
chr11	6704477	6704780	0.98	9.08E-01	MRPL17	3		
chr6	28863169	28863475	0.96	9.08E-01	SCAND3	-308210	TRIM27	28446
chr8	38820663	38820915	1.17	9.08E-01	HTRA4	-10879	PLEKHA2	62036
chr15	35261867	35262147	1.06	9.08E-01	AQR	-12		
chr2	86667787	86668251	1.08	9.08E-01	KDM3A	-565		
chr2	26256630	26257254	1.02	9.08E-01	RAB10	213		
chr3	39424817	39425127	1.10	9.08E-01	SLC25A38	157		
chr3	155572163	155572661	1.14	9.08E-01	SLC33A1	-245		
chr12	14923853	14924204	1.10	9.08E-01	H2AFJ	-3241	HIST4H4	36
chr8	117778470	117778821	1.00	9.08E-01	UTP23	-96		
chr20	30272452	30272799	1.05	9.08E-01	BCL2L1	38030	COX4I2	46935
chr17	29233112	29233471	1.10	9.08E-01	C17orf42	-6		
chr15	90808554	90809009	1.01	9.09E-01	NGRN	-113		
chr12	133348046	133348406	0.98	9.09E-01	ANKLE2	-9775	GOLGA3	57062
chr12	125406208	125406487	1.22	9.09E-01	UBC	-6771	DHX37	67319
chr11	67007302	67007655	1.05	9.09E-01	ADRBK1	-26426	KDM2A	120739
chr4	13612982	13613226	0.96	9.10E-01	NKX3-2	-66990	BOD1L	16224
chr9	4985044	4985320	1.14	9.10E-01	JAK2	-63		
chr1	155214485	155214801	1.05	9.10E-01	GBA	-3590		
chr17	46048305	46048569	1.03	9.10E-01	CDK5RAP3	8		
chr1	31769653	31770038	0.91	9.10E-01	SNRNP40	-202	ZCCHC17	4

chr7	101705366	101705649	1.01	9.10E-01	SH2B2	-222897	CUX1	244626		
chr6	2947599	2948023	0.99	9.10E-01	SERPINB9	-44266	SERPINB6	24279		
chr2	198010268	198010578	1.15	9.11E-01	PGAP1	-218969	ANKRD44	52339		
chr20	3140420	3140793	1.04	9.11E-01	UBOX5	-67	FASTKD5	-67		
chr3	12705577	12705960	1.05	9.11E-01	RAF1	-69				
chr11	66462203	66462526	0.97	9.11E-01	RBM4B	-17090	SPTBN2	26505		
chr6	44095030	44095301	1.02	9.11E-01	TMEM63B	-210	MRPL14	25		
chr1	63249340	63249584	1.01	9.11E-01	ATG4C	-341				
chr11	114270985	114271750	0.96	9.11E-01	RBM7	-16				
chr2	109335728	109336007	1.06	9.11E-01	RANBP2	-69				
chr10	98561316	98561560	1.03	9.11E-01	C10orf12	-179603	PIK3AP1	-81159		
chr7	130582856	130583211	1.02	9.11E-01	MKLN1	-429561	KLF14	-164174		
chr1	27226816	27227077	1.02	9.11E-01	GPATCH3	15				
chr10	12526852	12527237	1.01	9.11E-01	CAMK1D	135462	CCDC3	516659		
chr9	32552118	32552476	1.09	9.12E-01	TOPORS	304				
chr12	92757556	92757935	1.04	9.12E-01	PLEKHG7	-372519	BTG1	-218073		
chr17	37365104	37365458	1.03	9.12E-01	RPL19	8745	STAC2	16693		
chr2	74425533	74425986	1.03	9.12E-01	MTHFD2	70				
chr4	7072502	7072812	0.96	9.13E-01	GRPEL1	-2857				
chr11	102345249	102345680	1.03	9.13E-01	TMEM123	-21690	MMP7	56013		
chr1	193090919	193091542	1.12	9.13E-01	CDC73	143				
chr20	31408030	31408546	1.00	9.13E-01	MAPRE1	589				
chr1	9689752	9690167	1.02	9.13E-01	PIK3CD	-21830	TMEM201	40983		
chr15	45459581	45459891	1.06	9.13E-01	SHF	33637	DUOX1	37544		
chr12	124938129	124938420	1.06	9.13E-01	NCOR2	81882	ZNF664	480605		
chr7	155517244	155517529	0.95	9.13E-01	RBM33	80184	SHH	87580		
chr17	7835293	7835712	0.97	9.13E-01	KCNAB3	-2750	TRAPPC1	-245	CNTROB	30
chr1	151227047	151227310	0.98	9.14E-01	PSMD4	-18				
chr13	53191387	53191763	1.05	9.14E-01	HNRNPA1L2	-30				
chr2	26205404	26205754	1.02	9.14E-01	KIF3C	-136				
chr7	44621738	44622048	0.87	9.14E-01	TMED4	-66				
chr12	7000125	7000462	1.02	9.14E-01	SPSB2	-17845	LRRC23	-13603		
chr16	88748128	88748546	1.07	9.14E-01	MVD	-18842	SNAI3	4545		
chr17	74553713	74554088	1.09	9.14E-01	PRCD	17780	ST6GALNAC2	28244		
chr19	55791376	55791686	1.01	9.14E-01	BRSK1	-4003	HSPBP1	220		
chr2	3155158	3155556	0.98	9.14E-01	MYT1L	-820312	TSSC1	226296		
chr15	74988296	74988772	1.00	9.14E-01	EDC3	-148				
chr2	172174192	172174531	1.16	9.14E-01	DCAF17	-116497	TLK1	-86538		

chr7	135194853	135195154	1.17	9.14E-01	CNOT4	-153		
chr22	22901532	22901837	0.83	9.14E-01	PRAME	11		
chr20	46096980	46097401	1.02	9.14E-01	ZMYND8	-111717	NCOA3	-33466
chr6	88851325	88851684	1.03	9.14E-01	CNR1	24262	SPACA1	93998
chr20	36871304	36871614	1.00	9.14E-01	TGM2	-77759	BPI	-61093
chr1	94883634	94884007	0.97	9.15E-01	ABCD3	-112		
chr19	2051068	2051506	0.96	9.15E-01	MKNK2	-44		
chr19	5139333	5139569	0.92	9.15E-01	KDM4B	170327	PTPRS	201363
chr4	40198400	40198777	1.08	9.16E-01	RHOH	62		
chr19	45227117	45227416	0.96	9.16E-01	BCL3	-24711	CEACAM16	24909
chr1	155231825	155232450	1.11	9.16E-01	SCAMP3	57		
chr16	30441447	30441762	0.95	9.16E-01	DCTPP1	-232		
chr19	48021419	48021718	0.95	9.16E-01	NAPA	-3072		
chr2	232650941	232651427	1.12	9.16E-01	COPS7B	22		
chr5	138725438	138725785	0.90	9.16E-01	MGC29506	-7		
chr1	26560333	26560589	0.96	9.16E-01	SH3BGRL3	-45752	CATSPER4	43342
chr11	65190028	65190391	1.03	9.16E-01	SCYL1	-102338	FRMD8	36169
chr17	75441482	75441980	1.03	9.16E-01	TNRC6C	-558587	SEPT9	164239
chr20	39620095	39620411	1.11	9.16E-01	MAFB	-302377	TOP1	-37209
chr20	49434861	49435200	1.08	9.16E-01	BCAS4	23564	ADNP	112496
chr6	26568937	26569247	1.08	9.16E-01	ABT1	-28088	HMGN4	30520
chr22	22562315	22562683	0.99	9.17E-01	TOP3B	-225352	VPREB1	-36701
chr17	8124721	8125018	1.00	9.17E-01	AURKB	-10987	C17orf68	26543
chr9	37119972	37120769	0.96	9.18E-01	ZCCHC7	-98		
chr7	142153256	142153515	1.07	9.18E-01	TCRBV6S6A2T	-13571	TCRBV1S1A1N1	86647
chr13	77601093	77601342	1.12	9.19E-01	FBXL3	113		
chr6	51928340	51928650	1.31	9.19E-01	PKHD1	23928		
chr1	28559418	28559662	0.93	9.19E-01	ATPIF1	-3071	DNAJC8	2
chr11	65264881	65265205	0.90	9.19E-01	SCYL1	-27505	FRMD8	111002
chr17	47841540	47841888	0.91	9.19E-01	SLC35B1	-56432	MYST2	-24357
chr3	51975774	51976455	0.91	9.19E-01	PARP3	-246	RRP9	-193
chr2	86263619	86263938	1.12	9.19E-01	ST3GAL5	-147622	POLR1A	69499
chr12	112279644	112279998	1.05	9.19E-01	MAPKAPK5	-458		
chr17	78459598	78459991	0.96	9.19E-01	RPTOR	-58830	NPTX1	-9391
chr22	30820121	30820374	1.08	9.19E-01	SEC14L2	27315	SEC14L3	47786
chr17	38269373	38269722	1.14	9.19E-01	NR1D1	-12575	MSL1	-9242
chr17	75437282	75438098	0.96	9.19E-01	TNRC6C	-562628	SEPT9	160198
chr1	27488170	27488439	1.01	9.19E-01	WDTC1	-72702	SLC9A1	-6854

chr9	4987905	4988214	1.02	9.19E-01	JAK2	2815	INSL6	197558
chr11	82867780	82868344	1.05	9.20E-01	PCF11	-75		
chr3	10067939	10068480	1.08	9.20E-01	FANCD2	97		
chr1	225965294	225965564	1.04	9.20E-01	SRP9	-86		
chr1	212629235	212629550	0.99	9.20E-01	ATF3	-109304	NENF	23164
chr14	104544354	104544630	0.95	9.20E-01	ASPG	-7556	TDRD9	149675
chr1	112050238	112050576	0.95	9.20E-01	ADORA3	-3664		
chr6	28949877	28950121	1.03	9.20E-01	TRIM27	-58231	ZNF311	23036
chr16	72042404	72042847	1.05	9.20E-01	DHODH	-17		
chr17	73285562	73286007	1.11	9.20E-01	SLC25A19	-255		
chr12	95467323	95467598	0.97	9.20E-01	NR2C1	-57		
chr2	98280471	98280887	0.85	9.20E-01	ACTR1B	-118		
chr12	57915805	57916132	0.91	9.21E-01	DDIT3	-1669	MBD6	-690
chr16	16101604	16102060	1.03	9.21E-01	ABCC1	58398	ABCC6	215496
chr8	56798102	56798533	1.03	9.21E-01	LYN	5932	RPS20	188822
chr17	17991032	17991358	1.07	9.21E-01	DRG2	-88		
chr19	6633270	6633544	1.12	9.21E-01	CD70	-42244	TNFSF14	37192
chr15	102192420	102192730	1.03	9.21E-01	TM2D3	19		
chr6	111880375	111880754	0.80	9.22E-01	REV3L	-76133	TRAF3IP2	46909
chr1	112050813	112051132	0.93	9.23E-01	ADORA3	-4230		
chr19	44289506	44289761	1.01	9.23E-01	KCNN4	-4225		
chr17	37309908	37310237	0.97	9.23E-01	PLXDC1	-2171		
chr17	7755066	7755408	0.94	9.23E-01	TMEM88	-3147		
chr1	155826954	155827394	1.11	9.23E-01	SYT11	-2116	GON4L	-202
chr13	49136548	49136805	1.26	9.23E-01	CYSLTR2	-144276	RCBTB2	-29361
chr19	50168756	50169322	0.95	9.23E-01	IRF3	93	BCL2L12	640
chr6	26123858	26124168	1.00	9.23E-01	HIST1H2AC	-360	HIST1H2BC	119
chr1	224622432	224622676	1.00	9.23E-01	WDR26	-822		
chr6	52149347	52150130	1.00	9.23E-01	MCM3	-157		
chr9	101017838	101018343	1.01	9.23E-01	TBC1D2	-88		
chr1	159880269	159880543	1.06	9.23E-01	CCDC19	-10500	TAGLN2	14878
chr13	96032948	96033240	1.04	9.23E-01	CLDN10	-171853	ABCC4	-79407
chr17	66201378	66201647	1.05	9.23E-01	AMZ2	-42632	KPNA2	169665
chr2	206571893	206572176	0.92	9.23E-01	NRP2	24811	NDUFS1	452152
chr6	37019241	37019579	0.93	9.23E-01	PIM1	-118512	FGD2	45987
chr6	126101292	126101625	1.15	9.23E-01	NCOA7	-10542	HEY2	30727
chr17	19281200	19281704	1.01	9.24E-01	MAPK7	-322		
chr6	41040187	41040790	1.02	9.24E-01	NFYA	-218		

chr22	42466754	42467150	0.94	9.24E-01	NAGA	-106		
chr5	76788247	76788557	1.09	9.24E-01	OTP	146120	PDE8B	281696
chr18	33552449	33552714	0.99	9.24E-01	SLC39A6	156775	GALNT1	318049
chr5	32313155	32313429	0.97	9.24E-01	MTMR12	-178		
chr17	79075575	79075826	0.99	9.24E-01	AATK	64171	BAIAP2	66754
chr19	6802383	6802917	1.00	9.24E-01	EMR1	-84932	VAV1	29928
chr17	7387975	7388229	1.01	9.24E-01	ZBTB4	-534	POLR2A	252
chr9	126964760	126965047	1.06	9.24E-01	NEK6	-55339	LHX2	191015
chr11	29549144	29549531	1.02	9.24E-01	KCNA4	489150		
chr7	65670072	65670411	0.83	9.24E-01	TPST1	-17		
chr1	37942882	37943179	1.02	9.24E-01	ZC3H12A	2912	MEAF6	37333
chr10	125163837	125164147	1.12	9.24E-01	GPR26	-261879	BUB3	250232
chr13	111866833	111867110	0.96	9.24E-01	C13orf16	-106043	ARHGEF7	99348
chr15	40566862	40567217	0.98	9.24E-01	PLCB2	33134	PAK6	57411
chr19	39888324	39888645	1.09	9.24E-01	ZFP36	-9002	MED29	6522
chr3	120136196	120136598	0.97	9.24E-01	LRRC58	-68211	FSTL1	33521
chr6	20320168	20320441	1.11	9.24E-01	MBOAT1	-107635	E2F3	-81832
chr6	26322270	26322523	0.99	9.24E-01	BTN3A2	-43001	HIST1H4H	-36670
chr6	28920897	28921238	1.12	9.24E-01	TRIM27	-29300	ZNF311	51967
chr7	76022372	76022710	0.98	9.24E-01	YWHAG	-34199	SRCRB4D	16471
chr7	100026840	100027486	0.93	9.24E-01	ZCWPW1	-861	MEPCE	-366
chr17	73900993	73901353	0.89	9.25E-01	MRPL38	8		
chr17	27065051	27065456	0.93	9.25E-01	TRAF4	-5769	NEK8	9422
chr12	32417858	32418293	0.95	9.25E-01	FGD4	-236965	BICD1	157891
chr10	116697842	116698198	1.07	9.25E-01	TRUB1	68		
chr4	103266316	103266829	1.04	9.25E-01	SLC39A8	82		
chr16	4321692	4322107	1.10	9.25E-01	SRL	-29819	TFAP4	1101
chr4	174255519	174255982	0.93	9.26E-01	HMGB2	-831		
chr1	10458686	10459051	0.83	9.26E-01	PGD	-216		
chr4	41216636	41216925	0.88	9.26E-01	APBB2	-146		
chr11	28131673	28131992	1.18	9.26E-01	KIF18A	-2087	METT5D1	2035
chr12	109058658	109058917	1.05	9.26E-01	SELPLG	-31118	CORO1C	66507
chr1	156571119	156571517	1.00	9.27E-01	GPATCH4	-48		
chr11	124981390	124981751	0.98	9.27E-01	PKNOX2	-52988	TMEM218	-8366
chr1	151162531	151162841	1.07	9.27E-01	VPS72	-46		
chr17	7475722	7476147	1.06	9.27E-01	EIF4A1	333		
chr16	30583090	30583596	1.09	9.27E-01	ZNF688	385		
chr17	61677997	61678372	1.17	9.28E-01	TACO1	-58		

chr19	3985325	3985663	1.13	9.28E-01	EEF2	-33		
chr1	150208202	150208700	1.05	9.28E-01	ANP32E	53		
chr6	163834977	163835221	0.95	9.28E-01	QKI	-576		
chr8	104032916	104033594	0.94	9.28E-01	ATP6V1C1	7		
chr12	25403765	25404093	0.84	9.28E-01	KRAS	-75		
chr15	41694574	41694850	1.04	9.28E-01	NDUFAF1	-70		
chr1	214776189	214776558	1.17	9.28E-01	CENPF	-158		
chr10	97054915	97055301	0.98	9.28E-01	PDLIM1	-4327		
chr19	51869731	51870045	0.94	9.28E-01	ETFB	-11792	CLDND2	2369
chr2	156002300	156002655	1.05	9.28E-01	KCNJ3	447385		
chr17	74137283	74137519	0.83	9.28E-01	FOXJ1	-756		
chr17	42287819	42288095	1.04	9.28E-01	ATXN7L3	-12428	UBTF	7707
chr1	45987386	45987738	1.05	9.29E-01	PRDX1	47		
chr16	68271474	68271923	1.03	9.29E-01	ESRP2	-1563		
chr6	88632586	88632930	0.93	9.29E-01	AKIRIN2	-220773	SPACA1	-124749
chr8	82633476	82633833	0.99	9.29E-01	ZFAND1	-125		
chr14	74220863	74221111	0.99	9.29E-01	PNMA1	-39859	C14orf43	32909
chr17	57784669	57785091	1.09	9.29E-01	PTRH2	-24	TMEM49	17
chr19	49077985	49078264	1.01	9.29E-01	SPACA4	-31875	SULT2B1	22696
chr20	62694259	62694556	1.07	9.29E-01	TCEA2	5969	RGS19	16916
chr19	14640100	14640375	0.92	9.29E-01	TECR	-144		
chr16	70438263	70438624	1.26	9.29E-01	ST3GAL2	34547	DDX19B	105372
chr5	43017982	43018365	1.03	9.29E-01	C5orf39	22273	GHR	594148
chr4	17616088	17616390	0.99	9.29E-01	MED28	-34		
chr11	71639284	71639664	1.07	9.29E-01	RNF121	-294	AK131288	14
chr12	58299161	58299474	1.07	9.29E-01	CTDSP2	-58571	XRCC6BP1	-36127
chr3	72149958	72150203	1.09	9.29E-01	PROK2	-315724	RYBP	345693
chr22	22749009	22749334	0.81	9.29E-01	ZNF280B	114333	VPREB1	149972
chr11	67250495	67251182	1.01	9.29E-01	AIP	334		
chr22	46731420	46731758	1.06	9.29E-01	TRMU	291		
chr19	45972346	45972770	0.91	9.29E-01	FOSB	1305	RTN2	27755
chr12	51157580	51157863	0.93	9.30E-01	ATF1	-97		
chr17	38443715	38444067	0.98	9.30E-01	CDC6	-255		
chr8	146277716	146277960	0.93	9.30E-01	ZNF252	-49553		
chr18	30499796	30500078	1.13	9.30E-01	ASXL3	-658604	KLHL14	-146963
chr7	22862303	22862628	0.90	9.30E-01	TOMM7	-45		
chr7	75115483	75115768	1.03	9.30E-01	POM121C	-58		
chr17	8152371	8152714	0.90	9.30E-01	C17orf68	-1130	PFAS	-53

chr17	38267834	38268161	0.88	9.30E-01	NR1D1	-11025	MSL1	-10792
chr2	172290260	172290794	1.01	9.30E-01	DCAF17	-332	METTL8	785
chr9	139743605	139743857	1.08	9.30E-01	MAMDC4	-3088	PHPT1	475
chr3	32022219	32022529	1.05	9.30E-01	ZNF860	-892	OSBPL10	864
chr20	30308703	30309019	1.10	9.30E-01	BCL2L1	1795	COX4I2	83170
chr8	67026274	67026618	0.97	9.30E-01	TRIM55	-12832	DNAJC5B	92655
chr12	12849514	12849850	1.02	9.30E-01	GPR19	-561		
chr14	73524848	73525283	0.86	9.31E-01	RBM25	-155		
chr8	110346263	110346765	1.01	9.31E-01	ENY2	-122		
chr13	30969614	30969902	1.14	9.31E-01	KATNAL1	-88595	HMGB1	70323
chr20	35474558	35474820	1.06	9.31E-01	DSN1	-72535	SAMHD1	105487
chrX	136520345	136520622	0.85	9.31E-01	GPR101	-406651	ZIC3	-127862
chr9	101998157	101998497	1.08	9.31E-01	NR4A3	-590682	SEC61B	13757
chr16	50058779	50059152	1.02	9.31E-01	TMEM188	-223		
chr10	6130687	6131079	1.05	9.32E-01	RBM17	-66		
chr1	111948948	111949323	0.98	9.32E-01	OVGP1	21263	CHIA	115652
chr3	187650339	187650713	1.09	9.32E-01	LPP	-280195	BCL6	-187051
chr6	159465901	159466250	0.97	9.32E-01	TAGAP	108		
chr15	93382945	93383255	0.82	9.32E-01	FAM174B	-184069	CHD2	-60451
chr14	68303779	68304055	1.09	9.32E-01	RAD51L1	17408	ZFP36L1	955868
chr21	44819421	44819800	1.08	9.32E-01	SIK1	27391	CRYAA	230470
chr14	65380893	65381203	0.93	9.32E-01	SPTB	-91185	GPX2	28483
chr2	48133802	48134291	0.91	9.33E-01	FBXO11	-1233		
chr2	65617255	65617585	1.12	9.33E-01	SPRED2	42236	ACTR2	162591
chr1	151371772	151372086	1.05	9.33E-01	PSMB4	-112		
chr3	49066645	49067125	0.99	9.34E-01	IMPDH2	-10		
chr7	149535232	149535567	0.89	9.34E-01	ZNF862	-109		
chr3	107844401	107844756	1.07	9.34E-01	CD47	-34644	IFT57	96838
chr14	91976787	91977051	0.95	9.34E-01	SMEK1	-275		
chr5	64920053	64920297	0.91	9.34E-01	TRIM23	12		
chr6	149867110	149867350	0.98	9.34E-01	PPIL4	-59		
chr12	122985309	122985768	1.09	9.34E-01	ZCCHC8	-21		
chr1	23885791	23886298	0.90	9.34E-01	ID3	277		
chr13	50018110	50018620	0.96	9.34E-01	CAB39L	-42630	SETDB2	-7324
chr14	91526826	91527454	0.96	9.34E-01	RPS6KA5	-147		
chr13	31191060	31191368	1.00	9.35E-01	USPL1	-616		
chr20	35700082	35700398	1.36	9.35E-01	SAMHD1	-120064	RBL1	24170
chr13	103249148	103249478	0.91	9.35E-01	TPP2	27		

chr9	102790452	102790728	1.02	9.35E-01	ERP44	70740	STX17	121675
chr17	30580096	30580406	1.09	9.35E-01	RHBDL3	-12944	RHOT1	110778
chr7	148936498	148936828	0.92	9.35E-01	ZNF212	-111		
chr15	85931273	85931583	1.04	9.35E-01	AKAP13	7557	KLHL25	406761
chr2	219166019	219166342	0.99	9.35E-01	SLC11A1	-80571	TMBIM1	-8901
chr6	31757223	31757487	1.05	9.35E-01	C6orf27	-12247	VARS	6357
chr20	32319573	32319942	0.94	9.35E-01	ZNF341	-50		
chr7	96746695	96747096	1.07	9.36E-01	ACN9	991		
chr19	19774369	19774852	0.96	9.36E-01	ATP13A1	-108		
chr5	138677305	138677677	1.07	9.36E-01	PAIP2	-28		
chr16	86597451	86597703	0.94	9.36E-01	FOXC2	-3280		
chr1	206299332	206299670	0.99	9.36E-01	CTSE	-17958	C1orf186	-10854
chr11	77348696	77349114	1.02	9.36E-01	CLNS1A	-54		
chr11	62554580	62555078	0.99	9.36E-01	TMEM179B	-45		
chr12	67845896	67846241	1.10	9.36E-01	DYRK2	-196443	CAND1	183008
chr2	70369757	70370079	0.95	9.36E-01	PCBP1	55333	TIA1	105861
chr3	47021100	47021388	1.01	9.36E-01	NBEAL2	71		
chr4	147096670	147096960	0.97	9.36E-01	LSM6	-20		
chr1	85086406	85086846	1.13	9.36E-01	CTBS	-46463	SSX2IP	69554
chr17	8090546	8091169	1.08	9.36E-01	TMEM107	-11144	C17orf59	2706
chr17	79604002	79604356	0.93	9.37E-01	NPLOC4	-41		
chr19	16222427	16222823	0.95	9.37E-01	RAB8A	135		
chr13	32889477	32889822	0.96	9.37E-01	BRCA2	33		
chr6	155635638	155635980	1.05	9.37E-01	TFB1M	-183		
chr11	6502557	6502877	0.97	9.37E-01	ARFIP2	-122	FXC1	40
chr5	133879831	133880115	1.09	9.37E-01	PHF15	18175	SAR1B	88554
chr1	40840170	40840648	1.22	9.38E-01	SMAP2	681		
chr2	171785289	171785657	1.02	9.38E-01	GORASP2	-238		
chr10	74114293	74114541	1.03	9.38E-01	DNAJB12	490		
chr17	7137807	7138051	0.99	9.38E-01	DVL2	-66		
chr17	40810980	40811367	1.01	9.38E-01	TUBG2	-92		
chr19	3435205	3435449	0.99	9.38E-01	C19orf77	45213	NFIC	75711
chr12	48577220	48577595	0.82	9.38E-01	C12orf68	42		
chr16	81348169	81348519	0.93	9.38E-01	GAN	-227		
chr19	17445506	17445863	1.10	9.38E-01	GTPBP3	-2671	ANO8	-47
chr19	10538795	10539160	1.20	9.38E-01	PDE4A	7645	KEAP1	75076
chr3	38159397	38159789	1.04	9.38E-01	ACAA1	19140	DLEC1	78897
chr19	1095316	1095604	1.10	9.39E-01	POLR2E	-69		

chr19	50372736	50373154	0.89	9.39E-01	PNKP	-2127		
chr22	42093123	42093423	0.90	9.39E-01	MEI1	-2245		
chr8	27695098	27695567	0.99	9.39E-01	PBK	16		
chr1	16023444	16023731	0.91	9.39E-01	SLC25A34	-39221	PLEKHM2	12761
chr19	18107680	18107999	1.25	9.39E-01	KCNN1	45729	IL12RB1	89902
chr2	39351447	39351870	0.96	9.39E-01	SOS1	-4055		
chr1	28908190	28908612	0.98	9.39E-01	RAB42	-10311	TRNAU1AP	28872
chr9	7052629	7052886	0.85	9.39E-01	KDM4C	295117	C9orf123	747041
chr3	52188582	52189060	1.19	9.39E-01	WDR51A	-115		
chr5	154317652	154317962	1.08	9.39E-01	MRPL22	-2826	GEMIN5	-31
chr11	60542941	60543230	1.01	9.40E-01	MS4A10	-9735	MS4A15	18746
chr17	56084584	56085142	1.11	9.40E-01	SFRS1	-156		
chr6	144416680	144416986	1.09	9.40E-01	SF3B5	-79		
chr16	30609529	30609767	1.04	9.41E-01	ZNF785	-12638	ZNF689	12034
chr9	95826589	95827061	1.04	9.41E-01	C9orf89	-31625	SUSD3	5836
chr12	48499725	48500255	1.08	9.41E-01	SENP1	-349		
chr11	8704037	8704427	1.12	9.41E-01	RPL27A	237		
chr3	180629745	180630394	0.99	9.41E-01	FXR1	-382		
chr17	21002172	21002570	1.19	9.41E-01	USP22	-56019	DHRS7B	-27887
chr18	32621159	32621581	0.98	9.41E-01	ZNF397	-199628	MAPRE2	63162
chr7	157129479	157129728	0.90	9.41E-01	DNAJB6	-106		
chr10	97453649	97454097	1.02	9.42E-01	TCTN3	27		
chr17	40687884	40688262	0.97	9.42E-01	NAGLU	122		
chr14	91580813	91581064	1.03	9.42E-01	C14orf159	-40		
chr16	89752908	89753344	1.01	9.42E-01	CDK10	50		
chr6	35265383	35265693	0.94	9.42E-01	DEF6	-57		
chr15	51169174	51169535	1.11	9.42E-01	SPPL2A	-111445	AP4E1	-31591
chr19	2052352	2052609	1.00	9.42E-01	MKNK2	-1238		
chr3	39448184	39448527	1.03	9.42E-01	RPSA	152		
chr5	172386206	172386516	1.07	9.42E-01	RPL26L1	-78		
chr7	56174166	56174546	1.01	9.42E-01	CHCHD2	-169		
chr1	14057409	14057831	1.10	9.42E-01	KIAA1026	-867593	PRDM2	26270
chr13	53028543	53028866	0.95	9.42E-01	VPS36	-3942	CKAP2	-790
chr13	114518392	114518664	1.07	9.42E-01	GAS6	48518	FAM70B	56312
chr20	62339177	62339422	1.05	9.42E-01	ZGPAT	-91	ARFRP1	55
chr6	26607671	26607916	0.97	9.42E-01	ABT1	10614	ZNF322A	52169
chr6	159331705	159332017	0.85	9.42E-01	OSTCL	-53197	TAGAP	134323
chr1	183441000	183441310	1.08	9.42E-01	SMG7	-479		

chr18	23501646	23502035	0.90	9.42E-01	ZNF521	-569627	SS18	168770
chr5	177631403	177631791	0.94	9.43E-01	HNRNPAB	89		
chr3	196336199	196336488	1.05	9.43E-01	LRRC33	-30312	FBXO45	40619
chr7	121025868	121026138	1.01	9.43E-01	FAM3C	10419	WNT16	56913
chr16	29816715	29817050	0.91	9.44E-01	MAZ	-972		
chr19	49140508	49140862	0.96	9.44E-01	Sec1	-611	DBP	-46
chr17	7518533	7518808	0.99	9.44E-01	FXR2	-600		
chr12	58329744	58330113	0.95	9.44E-01	CTDSP2	-89182	XRCC6BP1	-5516
chr12	72148514	72148844	0.91	9.44E-01	RAB21	21		
chr1	107599103	107599406	0.90	9.44E-01	PRMT6	-12		
chr19	2446440	2446818	0.98	9.44E-01	TIMM13	-18754	LMNB2	10329
chr8	121822010	121822442	1.06	9.44E-01	SNTB1	2083	MTBP	364560
chr7	35768955	35769344	1.06	9.45E-01	SEPT7	-71477	HERPUD2	-34378
chr19	46009734	46010113	0.94	9.45E-01	VASP	-764		
chr1	212459549	212459859	1.04	9.45E-01	PPP2R5A	825		
chr18	267892	268255	1.03	9.46E-01	THOC1	-15		
chr18	43245076	43245388	1.11	9.46E-01	SLC14A1	-58860	SLC14A2	50466
chr19	42455763	42456082	1.03	9.46E-01	RABAC1	7605	ARHGEF1	67477
chr20	30249318	30249759	0.96	9.46E-01	COX4I2	23848	BCL2L1	61117
chr7	148224745	148225014	0.95	9.46E-01	CUL1	-171053	NM_001126313	93241
chr10	13628708	13629038	1.06	9.46E-01	PRPF18	-66		
chr17	7518125	7518420	0.97	9.46E-01	FXR2	-202		
chr15	75075659	75075980	1.12	9.46E-01	LMAN1L	-29374	CSK	1395
chr16	27410324	27410748	1.07	9.46E-01	IL21R	-28043	IL4R	85285
chr16	68285746	68285995	0.94	9.46E-01	SLC7A6	-12552	PLA2G15	6624
chr9	123605070	123605467	1.06	9.46E-01	PSMD5	-63		
chr9	136202950	136203249	0.96	9.46E-01	SURF6	-53		
chr13	113862852	113863130	1.06	9.46E-01	CUL4A	-95	PCID2	38
chr15	22461047	22461429	0.88	9.46E-01	GOLGA6L1	-275008	OR4N2	47776
chr6	24936921	24937230	1.24	9.46E-01	FAM65B	-25881	DKFZp686H12134	200975
chr15	83478015	83478471	0.95	9.46E-01	WHAMM	270		
chr16	81069177	81069548	0.98	9.46E-01	ATMIN	-95		
chr19	49834909	49835244	1.09	9.46E-01	CD37	-3600		
chr12	125634408	125634924	1.07	9.46E-01	TMEM132B	-176496	AACS	84741
chr3	12761575	12761885	1.06	9.46E-01	RAF1	-56030	TMEM40	39078
chr9	1009157	1009405	1.05	9.46E-01	DMRT2	-41339	DMRT3	32317
chr9	100797004	100797332	1.01	9.46E-01	NANS	-21514	ANP32B	51679
chr17	74667830	74668102	1.00	9.46E-01	ST6GALNAC1	-28072	MXRA7	39090

chr1	109756379	109756628	0.85	9.47E-01	SARS	-36		
chr19	11647041	11647285	1.01	9.47E-01	CNN1	-2416		
chr22	22384989	22385467	1.03	9.47E-01	VPREB1	-213972	TOP3B	-48081
chr8	97247607	97248015	1.05	9.47E-01	UQCRB	51		
chr12	112123644	112124013	0.92	9.47E-01	BRAP	-39	ACAD10	-28
chr18	30340315	30340750	0.94	9.47E-01	KLHL14	12441	MEP1B	570546
chr1	149224429	149224766	1.12	9.47E-01	PPIAL4B	-328405	NBPF16	485156
chr1	40997080	40997405	0.95	9.47E-01	ZNF684	10		
chr4	40303687	40304005	0.97	9.47E-01	CHRNA9	-33623	RHOH	105319
chr10	102819791	102820048	1.00	9.47E-01	KAZALD1	-1079		
chr13	34392040	34392316	0.98	9.47E-01	RFC3	-28		
chr19	50860366	50860700	1.15	9.47E-01	NAPSB	-12528	NAPSA	8398
chr16	8617709	8618013	1.15	9.47E-01	TMEM114	4365		
chr17	66508363	66508628	1.06	9.48E-01	PRKAR1A	386		
chr15	68075683	68075993	0.95	9.48E-01	LBXCOR1	-42103	MAP2K5	240817
chr15	74731104	74731423	1.01	9.49E-01	SEMA7A	-4965		
chr14	75179653	75179989	1.21	9.49E-01	FCF1	-29	KIAA0317	-14
chr15	49447760	49448070	1.08	9.49E-01	COPS2	-61		
chr1	54665628	54665882	0.89	9.49E-01	MRPL37	-85	CYB5RL	-9
chr4	2813779	2814135	0.90	9.49E-01	TNIP2	-55854	SH3BP2	-5955
chr1	27481410	27481808	1.00	9.50E-01	SLC9A1	-158		
chr2	146169829	146170171	0.98	9.50E-01	ZEB2	-892084		
chr1	10490337	10490674	0.96	9.50E-01	CORT	-19465	PGD	31421
chr17	75084474	75084864	1.01	9.50E-01	SEC14L1	-52336	MGAT5B	219871
chr14	58764599	58765280	0.94	9.50E-01	ARID4A	-282		
chr17	38210455	38210765	1.06	9.50E-01	MED24	279		
chr20	30311661	30311994	1.07	9.50E-01	BCL2L1	-1172		
chr8	109260706	109261125	1.00	9.50E-01	EIF3E	43		
chr8	126103789	126104170	0.98	9.50E-01	NSMCE2	-103	KIAA0196	81
chr16	10837521	10838003	1.03	9.50E-01	NUBP1	64		
chr5	116283696	116283940	1.05	9.50E-01	SEMA6A	-373267		
chr17	55038103	55038646	1.06	9.50E-01	COIL	36		
chr1	111682740	111683037	0.99	9.50E-01	DRAM2	-51	CEPT1	640
chr17	43138147	43139132	1.07	9.51E-01	NMT1	-40		
chr2	55237302	55237713	0.89	9.51E-01	RTN4	40226	EML6	285359
chr16	81110561	81110945	1.05	9.51E-01	GCSH	19227	ATMIN	41295
chr1	226543427	226543710	1.21	9.51E-01	LIN9	-46371	PARP1	52232
chr14	77500281	77500591	0.96	9.51E-01	C14orf4	-5402	ZDHHC22	107698

chr6	27841056	27841510	0.92	9.51E-01	HIST1H3I	-1184	HIST1H4L	6
chr17	2304712	2305399	0.99	9.52E-01	MNT	-798		
chr3	176913948	176914189	1.16	9.52E-01	TBL1XR1	979		
chr5	149340096	149340386	0.98	9.52E-01	SLC26A2	-59		
chr7	104652973	104653873	0.98	9.52E-01	MLL5	-1214		
chr15	51200581	51201003	1.01	9.52E-01	AP4E1	-154		
chr3	71471180	71471448	1.04	9.52E-01	FOXP1	161826		
chr15	40346943	40347253	1.12	9.52E-01	SRP14	-15709	BMF	53977
chr12	4644281	4644570	0.99	9.52E-01	RAD51AP1	-3524		
chr11	62432669	62432985	0.99	9.52E-01	METTL12	48	METTL12	48
chr16	4526192	4526476	1.02	9.52E-01	NMRAL1	-1438	HMOX2	-7
chr9	131200935	131201245	1.04	9.53E-01	ODF2	-17342	CERCAM	18331
chr3	197461624	197461913	0.94	9.53E-01	BDH1	-178911	KIAA0226	2004
chr1	149858059	149858757	1.00	9.53E-01	HIST2H2BE	-176	HIST2H2AC	-117
chr3	176778363	176778675	1.25	9.53E-01	TBL1XR1	136529		
chr1	37945245	37945555	0.89	9.53E-01	ZC3H12A	5281	MEAF6	34964
chr19	11052732	11053039	1.18	9.53E-01	SMARCA4	-18712	YIPF2	-13529
chr7	128735303	128735692	1.04	9.53E-01	TSPAN33	-49214	TNPO3	-40300
chr6	64281925	64282363	1.06	9.53E-01	PTP4A1	224		
chr16	89181440	89181967	0.97	9.53E-01	CDH15	-56459	ACSF3	21450
chr19	13261006	13261337	1.21	9.53E-01	STX10	-185	IER2	-110
chr19	18117827	18118071	1.02	9.53E-01	KCNN1	55838	IL12RB1	79793
chr7	112090377	112090642	0.92	9.53E-01	C7orf53	-30398	IFRD1	27284
chr1	24018117	24018517	1.03	9.54E-01	RPL11	23		
chr12	122356222	122356546	1.03	9.54E-01	BCL7A	-103477	PSMD9	29738
chr14	102276327	102276792	0.99	9.54E-01	DYNC1H1	-154305	PPP2R5C	48425
chr2	238583401	238583803	1.05	9.54E-01	RAB17	-83866	LRRFIP1	-17205
chr2	197040686	197041201	0.97	9.54E-01	STK17B	-4608		
chr19	507034	507611	0.94	9.54E-01	C19orf20	-174		
chr11	61129563	61129925	1.04	9.54E-01	TMEM138	-112		
chr11	67273571	67273847	0.99	9.54E-01	PITPNM1	-866		
chr19	19174286	19174798	0.92	9.54E-01	SLC25A42	-266		
chr6	20534549	20534798	1.01	9.55E-01	CDKAL1	-14		
chr19	40696621	40697157	1.20	9.55E-01	MAP3K10	-762		
chr7	44613374	44613738	1.01	9.55E-01	DDX56	581		
chr17	45078534	45078810	0.96	9.55E-01	RPRML	-22058	CDC27	187993
chr1	203242254	203242556	0.97	9.55E-01	CHIT1	-43545	BTG2	-32259
chr17	73775537	73776285	1.02	9.55E-01	UNK	-4770	H3F3B	-51

chr2	219260844	219261146	1.09	9.56E-01	CTDSP1	-3483		
chr12	56862142	56862496	0.96	9.57E-01	SPRYD4	18		
chr19	3971151	3971435	0.89	9.57E-01	DAPK3	-1466		
chr15	72366860	72367152	0.94	9.57E-01	MYO9A	43416	NR2E3	264112
chr1	160765769	160766045	0.98	9.57E-01	LY9	-21		
chr19	19887008	19887639	1.07	9.57E-01	ZNF14	-43403	ZNF506	45236
chr20	34730799	34731171	0.97	9.57E-01	SCAND1	-188557	EPB41L1	-11677
chr6	28806121	28806410	1.02	9.57E-01	SCAND3	-251154	TRIM27	85502
chr19	58077833	58078069	1.03	9.57E-01	ZNF550	-10226	ZNF416	12292
chr12	123717230	123717572	0.93	9.57E-01	C12orf65	-440		
chr3	60541538	60541792	1.29	9.57E-01	FHIT	695468		
chr1	200988576	200988947	1.07	9.57E-01	KIF21B	4066	GPR25	146596
chr17	4118576	4118940	1.01	9.57E-01	ANKFY1	48516	CYB5D2	72296
chr9	125702805	125703231	0.95	9.57E-01	RABGAP1	-270		
chr16	87862099	87862393	0.99	9.57E-01	KLHDC4	-62704	SLC7A5	40854
chr2	65142297	65142709	1.09	9.57E-01	SERTAD2	-261457	SLC1A4	-73953
chr19	18362665	18362920	0.97	9.58E-01	PDE4C	-3783		
chr9	35658193	35658453	0.94	9.58E-01	CCDC107	12		
chr5	141348477	141348859	0.99	9.58E-01	RNF14	2231	GNPDA1	43952
chr7	128580594	128580839	1.00	9.58E-01	IRF5	2723	TNPO3	114481
chr14	69283406	69283722	0.97	9.58E-01	ZFP36L1	-23779	ACTN1	162519
chr17	48785103	48785485	1.06	9.58E-01	LUC7L3	-11683	ABCC3	73076
chr3	180707452	180707874	1.07	9.58E-01	DNAJC19	-133		
chr6	42897641	42898072	1.09	9.58E-01	CNPY3	997		
chr6	30685211	30685530	0.94	9.58E-01	TUBB	-2786	MDC1	87
chr12	129325464	129325744	1.12	9.58E-01	SLC15A4	-17063	GLT1D1	-12477
chr1	206234442	206234737	0.96	9.58E-01	AVPR1B	10307	C1orf186	54057
chr7	148564764	148565011	0.85	9.59E-01	EZH2	16526	CUL1	168955
chr10	94333716	94334020	0.92	9.59E-01	IDE	-16		
chr20	37554689	37555163	1.10	9.59E-01	FAM83D	-29		
chr11	2552652	2552928	0.87	9.59E-01	KCNQ1	86569	CDKN1C	354205
chr3	187618642	187619059	0.91	9.59E-01	LPP	-311870	BCL6	-155376
chr2	220507248	220507580	0.93	9.59E-01	SLC4A3	15122		
chr19	49843593	49843999	0.94	9.59E-01	CD37	5119	TEAD2	21918
chr1	39325446	39325682	0.99	9.59E-01	RRAGC	-224		
chr17	61776609	61776924	1.02	9.60E-01	LIMD2	752	LIMD2	752
chr13	50366856	50367559	1.05	9.60E-01	KPNA3	-151		
chr9	37034710	37035093	0.99	9.60E-01	PAX5	-426		

chr14	77519632	77519876	0.92	9.60E-01	C14orf4	-24720	ZDHHC22	88380
chr16	28833775	28834097	0.99	9.60E-01	ATXN2L	-478		
chr1	28975016	28975337	1.08	9.60E-01	GMEB1	-20067	TAF12	-5573
chr7	100303521	100303831	0.96	9.60E-01	POP7	0		
chr3	191781375	191781616	1.05	9.60E-01	FGF12	345342	PYDC2	602544
chr19	13204655	13204913	0.87	9.60E-01	LYL1	8897	NFIX	98200
chr17	26821785	26822029	0.99	9.60E-01	FOXN1	-29052	SLC13A2	21243
chr9	95527165	95527475	0.85	9.61E-01	BICD2	-237		
chr9	130797444	130797758	0.93	9.61E-01	DPM2	-96838	NAIF1	31998
chr18	24653267	24653711	1.00	9.61E-01	AQP4	-207773	CHST9	111800
chr6	28625905	28626217	1.20	9.61E-01	SCAND3	-70949	TRIM27	265707
chr1	21059518	21059923	0.89	9.61E-01	SH2D5	-588		
chr1	26632984	26633384	0.99	9.61E-01	UBXN11	11		
chr14	51302002	51302387	1.08	9.61E-01	NIN	-4356		
chr11	60222814	60223388	1.21	9.62E-01	MS4A1	-181		
chr3	51572542	51572830	1.10	9.62E-01	RAD54L2	-2910		
chr4	170541381	170541822	0.88	9.62E-01	CLCN3	-120		
chr14	50583155	50583395	1.06	9.62E-01	SOS2	114824	ARF6	223539
chr17	40169357	40169633	1.04	9.62E-01	NKIRAS2	-2592	DNAJC7	176
chr15	81558779	81559108	1.12	9.62E-01	STARD5	57580	IL16	69725
chr5	131761265	131761557	0.96	9.62E-01	SLC22A5	56010	IRF1	65054
chr5	124084001	124084275	0.98	9.62E-01	ZNF608	-3273		
chr10	134145316	134145744	0.79	9.63E-01	LRRC27	-84		
chr10	70660874	70661294	0.94	9.63E-01	DDX50	50		
chr19	10527315	10527646	0.83	9.63E-01	PDE4A	-3852		
chr2	77243619	77243921	1.01	9.63E-01	LRRTM4	505732		
chr2	237994299	237994581	0.96	9.63E-01	COPS8	356		
chr5	139493471	139493762	1.06	9.63E-01	PURA	-91		
chr22	19304279	19304589	1.21	9.63E-01	CLTCL1	-25195	HIRA	114785
chr12	22697339	22697777	1.01	9.63E-01	KIAA0528	-106		
chr7	73082015	73082306	0.88	9.63E-01	VPS37D	-13		
chr19	45922793	45923090	1.01	9.64E-01	ERCC1	3860	CD3EAP	13475
chr13	51486120	51486456	1.09	9.64E-01	RNASEH2B	2396	GUCY1B2	154055
chr21	34915083	34915431	1.14	9.64E-01	GART	-793	SON	-93
chr3	186739482	186739810	1.10	9.64E-01	ST6GAL1	-19		
chr1	45476925	45477190	1.11	9.64E-01	UROD	-772	HECTD3	-31
chr19	46195505	46195962	0.90	9.64E-01	SNRPD2	-291	QPCTL	-7
chr1	40780954	40781292	0.90	9.64E-01	COL9A2	1858	ZMPSTE24	57390

chr19	3180775	3181036	0.90	9.65E-01	NCLN	-4969		
chr6	11374563	11374842	0.98	9.65E-01	TMEM170B	-163808	NEDD9	-141788
chr6	43027021	43027446	0.97	9.65E-01	KLC4	-395	MRPL2	8
chr15	59271634	59272059	1.11	9.65E-01	SLTM	-45995	RNF111	-8018
chr15	75497082	75497413	1.20	9.65E-01	DNM1P33	98058	PPCDC	181321
chr5	43121289	43121701	0.94	9.65E-01	ZNF131	-147		
chr7	93681783	93682028	1.25	9.65E-01	COL1A2	-341967	BET1	-48216
chr20	34359652	34359930	0.99	9.66E-01	PHF20	-132		
chr7	75677187	75677533	1.09	9.66E-01	STYXL1	-39	MDH2	-33
chr7	135347047	135347400	0.97	9.67E-01	SLC13A4	65709	NUP205	104562
chr16	70472991	70473531	0.82	9.67E-01	ST3GAL2	-270		
chr10	125795438	125795778	0.90	9.67E-01	CPXM2	-144108	CHST15	10632
chr6	119558438	119558761	0.74	9.67E-01	MCM9	-302297	MAN1A1	112326
chr5	134209879	134210160	0.94	9.67E-01	TXNDC15	560		
chr12	56520025	56520306	1.05	9.67E-01	ESYT1	-1888		
chr19	11546247	11546563	1.00	9.67E-01	PRKCSH	136		
chr19	4342768	4343018	1.08	9.67E-01	STAP2	-3952	MPND	-631
chr3	133194111	133194542	1.22	9.67E-01	CDV3	-98107	BFSP2	75537
chr8	28479767	28480049	0.88	9.67E-01	EXTL3	-79245	FZD3	128135
chr1	173683831	173684186	1.09	9.67E-01	KLHL20	-71		
chr15	38746176	38746486	1.00	9.67E-01	FAM98B	3		
chr20	26188787	26189001	0.79	9.68E-01	ZNF337	-511425		
chr17	36714438	36714728	1.01	9.68E-01	SNIP	47600	ARHGAP23	100939
chr9	35811982	35812452	0.97	9.69E-01	SPAG8	42		
chr10	64576518	64576838	0.87	9.69E-01	EGR2	-552		
chr11	85956049	85956362	1.00	9.69E-01	EED	391		
chr16	89188990	89189266	0.89	9.69E-01	CDH15	-49035	ACSF3	28874
chr2	25008525	25008797	1.05	9.69E-01	C2orf79	7590	NCOA1	201316
chr3	169683979	169684648	1.06	9.69E-01	SEC62	-266		
chr14	50335018	50335335	1.06	9.69E-01	ARF6	-24559	SDCCAG1	-15638
chr17	36907997	36908252	1.16	9.69E-01	PCGF2	-3564	PSMB3	-877
chr16	20753050	20753372	1.00	9.69E-01	ACSM1	-50633	ACSM3	-22101
chr17	56406206	56406528	1.22	9.70E-01	BZRAP1	-215		
chr6	156600385	156600730	0.91	9.70E-01	NOX3	-823521	ARID1B	-498528
chr7	148787740	148788083	0.91	9.71E-01	ZNF786	-43		
chr6	167526552	167526862	1.15	9.71E-01	CCR6	1412	GPR31	44612
chr1	192777926	192778307	0.97	9.71E-01	RGS2	-52		
chr19	50833985	50834381	0.90	9.71E-01	KCNC3	-1549		

chr20	40321999	40322373	1.05	9.71E-01	CHD6	-75053					
chr11	95745333	95745667	0.97	9.71E-01	MTMR2	-88129	MAML2	330844			
chr19	54694516	54695020	1.01	9.71E-01	MBOAT7	-1035	TSEN34	-336			
chr6	83777213	83777462	1.05	9.71E-01	UBE2CBP	-1793	DOPEY1	-47			
chr7	28855515	28855853	1.00	9.71E-01	TRIL	142345	CREB5	403540			
chr11	62341219	62341740	0.95	9.72E-01	EEF1G	-20					
chr1	151894100	151894410	1.13	9.72E-01	THEM4	-12142	S100A10	72459			
chr20	37491779	37492208	1.03	9.72E-01	FAM83D	-62961	PPP1R16B	57646			
chr18	47807743	47808066	1.05	9.72E-01	MBD1	239					
chr17	79367721	79368151	0.72	9.72E-01	TMEM105	-63462	BAHCC1	-5604			
chr9	126980981	126981288	0.94	9.72E-01	NEK6	-39108	LHX2	207246			
chr16	2653270	2653611	1.15	9.72E-01	PDPK1	39856	PDPK1	65471			
chr19	35704815	35705226	1.08	9.73E-01	FAM187B	14607	FXYD5	59394			
chr14	105282310	105282620	1.01	9.73E-01	KIAA0284	-49189	ZBTB42	15532			
chr6	139695618	139695995	0.96	9.73E-01	CITED2	-22					
chr10	103892467	103892826	0.98	9.73E-01	PPRC1	-140					
chr15	66161701	66162012	1.13	9.73E-01	RAB11A	61					
chr11	116643466	116643971	1.15	9.73E-01	ZNF259	15020					
chr11	65627782	65628094	0.94	9.73E-01	CFL1	-1837	CFL1	-1837	MUS81	66	
chr11	47788852	47789359	0.99	9.73E-01	FNBP4	-113					
chr7	141251026	141251364	0.98	9.73E-01	AGK	117					
chr12	120105416	120105838	1.06	9.73E-01	PRKAB1	-134					
chr15	51057778	51058053	0.92	9.73E-01	SPPL2A	-6					
chr15	66994489	66994818	0.84	9.73E-01	SMAD6	-20					
chr19	9879223	9879601	1.20	9.73E-01	ZNF846	-2					
chr9	116172944	116173233	1.06	9.73E-01	POLE3	-60					
chr16	14091808	14092093	1.05	9.73E-01	MKL2	-73245	ERCC4	77937			
chr15	50647233	50647687	1.11	9.74E-01	GABPB1	145					
chr4	174185845	174186308	0.85	9.74E-01	HMGB2	68843	GALNT7	96173			
chr19	6166042	6166358	0.91	9.74E-01	ACSBG2	30490	MLLT1	113759			
chr3	122134789	122135416	1.05	9.74E-01	FAM162A	32080	KPNA1	98683			
chr14	102990924	102991369	0.87	9.74E-01	CINP	-161894	RCOR1	-68086			
chr10	88854469	88854845	0.92	9.75E-01	GLUD1	-34					
chr21	33651125	33651772	1.03	9.75E-01	C21orf45	-73					
chr17	4268473	4268749	1.00	9.75E-01	ANKFY1	-101337	UBE2G1	1358			
chr6	5030946	5031226	1.08	9.75E-01	RPP40	-26815	LYRM4	230082			
chr9	35732246	35732490	1.09	9.75E-01	TLN1	24	CREB3	51			
chr11	7532690	7532937	0.93	9.75E-01	PPFIBP2	-2187					

chr4	7069676	7070041	0.96	9.75E-01	GRPEL1	-59		
chr6	12052120	12052364	0.98	9.75E-01	EDN1	-238287	HIVEP1	39518
chr20	56070327	56070637	0.97	9.75E-01	HMGB1L1	-6399	CTCFL	29701
chr17	41277150	41277702	1.03	9.75E-01	BRCA1	74		
chr12	123237117	123237381	1.12	9.75E-01	DENR	-122		
chr11	76092051	76092312	0.95	9.75E-01	PRKRIR	-302		
chr13	25139117	25139402	1.24	9.75E-01	ATP12A	-115435	PARP4	-52312
chr12	56615664	56616054	0.94	9.76E-01	OBFC2B	-2266	RNF41	-155
chr6	26233797	26234126	1.00	9.76E-01	HIST1H1D	1254	HIST1H3E	8579
chr17	900061	900495	1.00	9.76E-01	TIMM22	-79		
chr4	185185378	185185624	0.89	9.76E-01	ENPP6	-46387	IRF2	210225
chr4	4346568	4346906	0.96	9.76E-01	D4S234E	-40847	ZNF509	54813
chr1	46713231	46713598	0.92	9.76E-01	RAD54L	48		
chr2	27008640	27008955	0.92	9.76E-01	CENPA	-84		
chr14	105947253	105947499	1.06	9.76E-01	CRIP1	-5881	CRIP2	6245
chr5	131755321	131755669	0.88	9.76E-01	SLC22A5	50094	IRF1	70970
chr17	73511817	73512127	0.97	9.77E-01	TSEN54	-637	CASKIN2	-345
chr5	64858936	64859239	0.89	9.77E-01	CENPK	-93	PPWD1	-43
chr7	25164804	25165235	0.95	9.77E-01	CYCS	-65		
chr4	1124421	1124731	0.88	9.77E-01	RNF212	-16994	SPON2	42065
chr1	29211464	29211828	0.92	9.77E-01	EPB41	-1982		
chr15	41047361	41047830	1.04	9.77E-01	FAM82A2	-138		
chr15	66649073	66649492	0.97	9.77E-01	TIPIN	-229		
chr8	125462905	125463149	0.97	9.77E-01	TRMT12	-21		
chr11	35334908	35335187	0.89	9.77E-01	SLC1A2	106057	CD44	174631
chr16	85649590	85649858	0.99	9.77E-01	KIAA0182	2800	GINS2	72864
chr17	7117771	7118039	0.90	9.77E-01	ASGR1	-35022	DLG4	3023
chr1	27433139	27433471	0.92	9.77E-01	SLC9A1	48146	TRNP1	113110
chr16	67906812	67907102	1.01	9.77E-01	EDC4	-42		
chr16	31085483	31085845	1.05	9.78E-01	ZNF668	-160	ZNF646	-79
chr14	88237520	88237838	0.77	9.78E-01	GALC	222228		
chr17	45727099	45727463	0.97	9.78E-01	KPNB1	6		
chr10	22292618	22292992	0.97	9.78E-01	DNAJC1	-155		
chr11	77531734	77532107	1.14	9.78E-01	RSF1	-41		
chr13	111367553	111367947	0.91	9.79E-01	ING1	391		
chr19	15236208	15236666	0.92	9.79E-01	ILVBL	140		
chr13	60971107	60971451	0.82	9.79E-01	TDRD3	-148		
chr11	809758	810086	1.00	9.79E-01	LRDD	-4677	RPLP2	-14

chr16	85969710	85970009	1.21	9.79E-01	FOXF1	-574273	IRF8	37086
chr3	56951633	56952039	1.17	9.79E-01	ERC2	-449445	ARHGEF3	161500
chr7	50477867	50478128	0.87	9.79E-01	FIGNL1	40090	IKZF1	133620
chr11	192054	192371	1.00	9.80E-01	ODF3	-4548	SCGB1C1	-867
chr15	91396075	91396414	1.11	9.80E-01	FURIN	-15640	BLM	135666
chr15	44092571	44092820	0.88	9.81E-01	SERINC4	-441		
chr5	151150847	151151268	1.08	9.81E-01	G3BP1	-418		
chr11	1330802	1331127	1.00	9.82E-01	TOLLIP	-126		
chr14	58711335	58711620	0.99	9.82E-01	PSMA3	-115		
chr15	101458231	101458560	0.90	9.82E-01	LRRK1	-1064		
chr17	58754633	58754939	0.99	9.82E-01	BCAS3	-386		
chr19	17666120	17666437	1.03	9.82E-01	GLT25D1	-232		
chr22	37252413	37252723	1.02	9.82E-01	NCF4	-4462		
chr17	8126124	8126429	1.15	9.82E-01	AURKB	-12394	C17orf68	25136
chr2	11889947	11890231	1.08	9.82E-01	TRIB2	-966909	LPIN1	3349
chr16	58426137	58426447	1.04	9.82E-01	GINS3	-6		
chr19	36705699	36706009	1.04	9.82E-01	ZNF565	-288	ZNF146	350
chr1	89149579	89150180	0.99	9.82E-01	PKN2	-42		
chr7	44645970	44646249	0.93	9.82E-01	OGDH	-61		
chr1	12100732	12101166	1.01	9.82E-01	TNFRSF8	-22485	MFN2	60711
chr11	67415539	67415880	1.06	9.82E-01	TBX10	-8679	ACY3	2420
chr6	15658650	15658926	0.95	9.82E-01	DTNBP1	4483	JARID2	412261
chr6	160210768	160211021	0.96	9.82E-01	MRPL18	-597	TCP1	-160
chr8	10190282	10190540	1.11	9.82E-01	UNQ9391	-192670	MSRA	278581
chr12	122442102	122442568	1.12	9.82E-01	BCL7A	-17526	PSMD9	115689
chr3	183968063	183968373	0.94	9.82E-01	ALG3	-1459	ECE2	773
chr19	42363577	42364077	1.13	9.83E-01	RPS19	-161		
chr6	108879707	108879983	0.91	9.83E-01	FOXO3	-2224		
chr7	141437861	141438239	1.13	9.83E-01	SSBP1	-126		
chr13	31039996	31040356	0.94	9.83E-01	HMGB1	-95		
chr17	37009752	37010218	0.99	9.83E-01	RPL23	68		
chr17	56416259	56416749	1.01	9.83E-01	BZRAP1	-10352	SUPT4H1	13059
chr13	20534216	20534571	1.11	9.83E-01	ZMYM2	1584	GJA3	200789
chr2	74685114	74685496	0.91	9.83E-01	WBP1	-272		
chr5	72793890	72794328	1.04	9.84E-01	BTF3	-141		
chr11	75863262	75863572	1.11	9.84E-01	WNT11	54157	UVRAG	337205
chr14	62547688	62548076	0.93	9.84E-01	SYT16	85341	KCNH5	964073
chr12	57118961	57119419	0.94	9.84E-01	NACA	136		

chr12	93323097	93323459	0.98	9.84E-01	EEA1	-171		
chr15	66678899	66679215	1.01	9.84E-01	MAP2K1	-154		
chr2	232328962	232329638	1.02	9.84E-01	NCL	-95		
chr21	34144149	34144461	1.05	9.84E-01	C21orf66	-136		
chr14	68733709	68734127	1.00	9.84E-01	RAD51L1	447409	ZFP36L1	525867
chr18	33196898	33197148	1.14	9.84E-01	INO80C	-119068	GALNT1	-37510
chr22	23881806	23882080	1.00	9.84E-01	IGLL1	40552	BCR	359391
chr3	39297092	39297425	1.21	9.84E-01	XIRP1	-63182	CX3CR1	24268
chr4	25876913	25877382	1.06	9.84E-01	C4orf52	-38666	SEL1L3	-12538
chr5	38441863	38442174	0.97	9.84E-01	LIFR	153488	EGFLAM	183486
chr13	52768448	52768762	0.97	9.84E-01	NEK3	-34609	THSD1	212024
chr11	73691113	73691449	1.00	9.84E-01	UCP2	2608	DNAJB13	29917
chr9	6412741	6413313	0.89	9.84E-01	UHRF2	-124		
chr7	99214357	99214667	1.03	9.84E-01	ZNF498	-59		
chr1	167905255	167905723	1.03	9.84E-01	DCAF6	-419	BRP44	789
chr6	1869144	1869437	1.13	9.84E-01	FOXC1	258610	GMDS	376555
chr1	160312969	160313261	0.94	9.84E-01	NCSTN	52	COPA	239
chr17	43238770	43239333	0.95	9.85E-01	HEXIM2	788		
chr8	94753132	94753440	0.93	9.85E-01	RBM12B	-62		
chr19	9250910	9251354	0.99	9.85E-01	ZNF317	46	KIAA1588	46
chr6	27099698	27099949	1.06	9.85E-01	HIST1H2AG	-993	HIST1H2BJ	751
chr12	111180777	111181132	0.90	9.85E-01	PPP1CC	-198		
chr7	148565422	148565693	1.09	9.85E-01	EZH2	15856	CUL1	169625
chr16	15859778	15860054	1.14	9.85E-01	MYH11	90971	NDE1	115833
chr1	101491239	101491538	0.98	9.85E-01	DPH5	-27		
chr2	234160015	234160444	0.93	9.85E-01	ATG16L1	13		
chr1	23903893	23904167	0.97	9.85E-01	RPL11	-114264	ID3	-17708
chr19	41903219	41903860	1.08	9.85E-01	EXOSC5	-284	BCKDHA	-164
chr13	54603317	54603680	1.13	9.85E-01				
chr7	97881355	97881713	0.85	9.85E-01	TECPR1	-66		
chr6	170151612	170151849	0.92	9.86E-01	TCTE3	-93	C6orf70	10
chr1	10532407	10532816	1.08	9.86E-01	PEX14	-2391	DFFA	1
chr11	189662	190295	1.01	9.86E-01	SCGB1C1	-3101		
chr14	107147634	107148024	1.15	9.86E-01	ADAM6	-709471	IGHV7-81	135454
chr20	16710438	16710751	1.14	9.86E-01	SNRPB2	-34		
chr5	141340295	141340605	0.94	9.86E-01	PCDH12	-1823		
chr17	39823241	39823551	1.04	9.86E-01	KRT17	-42514	EIF1	-21731
chr3	49059271	49059623	0.96	9.86E-01	DALRD3	-3430	NDUFAF3	373

chr6	27782680	27783117	0.88	9.86E-01	HIST1H2AJ	-381	HIST1H2BM	77
chr6	139483033	139483499	1.17	9.86E-01	HECA	27017	TXLNB	129942
chr3	50654160	50654436	0.97	9.86E-01	MAPKAPK3	-303		
chr14	70092139	70092474	0.87	9.86E-01	SFRS5	-141527	KIAA0247	13997
chr8	47829392	47829669	0.89	9.86E-01	CEBPD	821195		
chr9	123836880	123837249	0.97	9.87E-01	C5	-24511	CEP110	-13509
chr12	80328891	80329156	0.90	9.87E-01	PPP1R12A	-46		
chr16	89045048	89045428	1.01	9.87E-01	CBFA2T3	-1837		
chr17	41323112	41323566	1.07	9.87E-01	NBR1	93		
chr12	104680598	104680895	1.01	9.87E-01	EID3	-16802	TXNRD1	71188
chr12	104682514	104682792	1.06	9.87E-01	EID3	-14896	TXNRD1	73094
chr4	110911810	110912120	1.13	9.87E-01	EGF	77925	ELOVL6	207806
chr9	135258920	135259270	1.09	9.87E-01	SETX	-28723	TTF1	23126
chr17	66288116	66288449	0.99	9.88E-01	SLC16A6	-878		
chr2	54342687	54343203	1.04	9.88E-01	ACYP2	535		
chr12	120524693	120525347	0.88	9.88E-01	RAB35	11945	CCDC64	97372
chr12	1799988	1800244	0.91	9.88E-01	ADIPOR2	-131		
chr1	31222693	31222999	1.07	9.88E-01	MATN1	-26414	LAPTM5	7837
chr12	67662343	67662761	0.93	9.89E-01	CAND1	-509		
chr12	2921890	2922288	0.99	9.89E-01	ITFG2	226		
chr16	3550755	3551069	1.08	9.89E-01	CLUAP1	-51		
chr1	93297438	93297875	1.07	9.89E-01	RPL5	63		
chr1	24862936	24863341	1.11	9.89E-01	C1orf130	-19463	RCAN3	33752
chr17	78518429	78518847	1.06	9.89E-01	RPTOR	13		
chr6	46097436	46097754	0.85	9.89E-01	ENPP4	-106		
chr3	189042522	189042815	1.04	9.90E-01	TP63	-306547	TPRG1	152906
chr14	96130672	96131217	0.89	9.90E-01	TCL1A	49588	GLRX5	129622
chr10	101945738	101946029	0.96	9.90E-01	ERLIN1	-70		
chr3	33482223	33482769	1.09	9.90E-01	UBP1	-599		
chr11	73309237	73309606	1.03	9.90E-01	PLEKHB1	-49172	RELT	222017
chr3	112627148	112627437	1.36	9.93E-01	CD200R1L	-62496	CD200R1	66644
chr7	128116631	128116941	1.02	9.93E-01	METTL2B	3		
chr12	72057455	72058032	0.97	9.93E-01	THAP2	-40	ZFC3H1	5
chr7	32931638	32932230	0.94	9.94E-01	KBTBD2	-466		
chr2	172180773	172181017	1.07	9.94E-01	DCAF17	-109964	TLK1	-93071
chr19	1652637	1653372	1.00	9.94E-01	TCF3	-2719		
chr8	8567284	8567594	0.89	9.94E-01	CLDN23	7773	MFHAS1	183692
chr2	175468072	175468320	1.08	9.94E-01	GPR155	-116385	WIPF1	31111

chr1	40723675	40724111	0.95	9.95E-01	ZMPSTE24	160		
chr20	26189885	26190195	0.77	9.95E-01	ZNF337	-512571		
chr4	25235325	25235650	0.81	9.95E-01	PI4K2B	-165		
chr1	156577912	156578188	1.22	9.95E-01	HAPLN2	-11036	GPATCH4	-6780
chr15	56538162	56538542	1.03	9.95E-01	RFX7	-2869		
chr17	25572348	25572625	0.99	9.95E-01	WSB1	-48619		
chr19	2266599	2266850	0.88	9.95E-01	OAZ1	-2795		
chr9	35116883	35117151	0.97	9.95E-01	KIAA1539	-1124		
chr11	17229864	17230173	1.05	9.95E-01	NUCB2	-68267	PIK3C2A	-38666
chr19	8317933	8318209	0.96	9.95E-01	LASS4	43854	CD320	55168
chr11	64877807	64878077	1.02	9.95E-01	TM7SF2	-1399		
chr13	22245061	22245332	0.93	9.95E-01	FGF9	-18		
chr22	24823846	24824298	1.01	9.95E-01	ADORA2A	-3747	ADORA2A	542
chr12	54585359	54585640	1.02	9.95E-01	SMUG1	-2743		
chr19	18699444	18699701	1.06	9.95E-01	C19orf60	78		
chr11	695599	695964	0.95	9.95E-01	DEAF1	-42	TMEM80	166
chr17	16189291	16189598	1.07	9.95E-01	CENPV	67367	PIGL	68936
chr5	115412163	115412477	1.14	9.95E-01	COMMD10	-8407	LVRN	114169
chr8	6283827	6284163	1.10	9.95E-01	MCPH1	19874	ANGPT2	136789
chr9	71199370	71199714	1.01	9.95E-01	PIP5K1B	-121074	C9orf71	-43759
chr17	47865605	47866123	0.96	9.95E-01	MYST2	-207		
chr17	58677370	58677652	1.05	9.95E-01	PPM1D	-43		
chrX	146992984	146993515	0.94	9.95E-01	FMR1	-259		
chr1	1342560	1342854	0.90	9.95E-01	MRPL20	-14		
chr4	1209296	1209596	0.97	9.95E-01	SPON2	-42805	CTBP1	33462
chr19	2740063	2740319	0.91	9.96E-01	SLC39A3	-117		
chr12	6798676	6799121	1.04	9.96E-01	ZNF384	-223		
chr7	105752880	105753160	0.91	9.96E-01	SYPL1	37		
chr6	43673683	43674004	0.89	9.96E-01	VEGFA	-64109	MRPS18A	-18316
chr5	130545516	130545842	1.01	9.96E-01	CDC42SE2	-54023	LYRM7	39038
chr12	56521587	56522186	0.87	9.96E-01	ESYT1	-167		
chr17	45266439	45267002	0.90	9.96E-01	CDC27	-56		
chr17	74350126	74350578	1.01	9.96E-01	PRPSAP1	-122		
chr2	61698193	61698623	0.90	9.96E-01	USP34	-559		
chr10	11220681	11221005	1.02	9.96E-01	CUGBP2	160950	USP6NL	353431
chr13	45491836	45492304	1.11	9.96E-01	TSC22D1	-341369	NUFIP1	71543
chr22	40888266	40888645	1.00	9.96E-01	SGSM3	121861	MKL1	144234
chr7	105698492	105699182	1.00	9.96E-01	SYPL1	54220	FLJ23834	95180

chr9	98256688	98256983	0.94	9.96E-01	FANCC	-176845	PTCH1	13995
chr9	79087152	79087429	1.01	9.96E-01	GCNT1	13223	PRUNE2	433712
chr1	110090929	110091391	1.07	9.96E-01	GNAI3	-26		
chr15	93443335	93443707	1.05	9.96E-01	CHD2	-30		
chr19	55850409	55850819	0.89	9.96E-01	SUV420H2	-607		
chr16	30886578	30886847	1.04	9.96E-01	ZNF629	-88190	BCL7C	18686
chr4	4543649	4543948	0.98	9.96E-01	STX18	-24		
chr7	120627679	120628037	0.97	9.96E-01	C7orf58	-893		
chr14	50437879	50438194	1.04	9.96E-01	ARF6	78301	SOS2	260062
chr12	95224812	95225131	0.97	9.97E-01	TMCC3	-180648	NDUFA12	172539
chr8	42623726	42624021	1.26	9.97E-01	CHRNA6	-255		
chr12	109197324	109197625	1.03	9.97E-01	CORO1C	-72180	SSH1	53884
chr3	14728887	14729228	1.05	9.97E-01	FGD5	-131411	C3orf20	12404
chr1	180992024	180992374	1.06	9.97E-01	STX6	-153		
chr14	24583714	24584024	0.99	9.97E-01	DCAF11	-37		
chr3	194991786	194992062	0.96	9.97E-01	C3orf21	-29		
chr9	131218260	131218570	0.93	9.97E-01	ODF2	-17		
chr7	26904114	26904706	0.96	9.97E-01	SKAP2	-69		
chr19	4402304	4402751	1.04	9.97E-01	SH3GL1	-2057	CHAF1A	-132
chr2	47403479	47403834	1.04	9.98E-01	CALM2	83		
chr4	26585461	26585771	1.02	9.98E-01	TBC1D19	70		
chr7	102389099	102389476	1.04	9.98E-01	LRRC17	-164056	POLR2J2	-77106
chr12	34175189	34175643	0.90	9.98E-01	ALG10	200		
chr15	85874200	85874712	1.01	9.99E-01	AKAP13	-49415	PDE8A	349251
chr12	56497870	56498326	0.98	9.99E-01	PA2G4	-5		
chr1	17380325	17380834	1.06	1.00E+00	SDHB	85		
chr1	24741963	24742310	0.93	1.00E+00	NIPAL3	-108		
chr1	28969402	28969818	0.96	1.00E+00	TAF12	-6		
chr1	36851387	36851870	0.88	1.00E+00	STK40	-144		
chr1	40042455	40042726	0.91	1.00E+00	PABPC4	-70		
chr1	51700556	51701021	1.03	1.00E+00	RNF11	-1156		
chr1	110527046	110527383	0.94	1.00E+00	AHCYL1	-93		
chr1	227127589	227127996	0.99	1.00E+00	CABC1	-145		
chr11	10772556	10772907	1.05	1.00E+00	CTR9	-79		
chr14	105491217	105491461	1.03	1.00E+00	CDCA4	-3914		
chr15	65282099	65282538	0.97	1.00E+00	SPG21	-68		
chr16	89233822	89234170	1.14	1.00E+00	CDH15	-4167		
chr17	48943536	48943793	1.04	1.00E+00	TOB1	-2252		

chr18	11980931	11981243	0.81	1.00E+00	IMPA2	-368		
chr18	32923803	32924103	1.00	1.00E+00	ZNF24	473		
chr19	11457002	11457376	0.99	1.00E+00	TMEM205	-208		
chr19	39340829	39341133	0.96	1.00E+00	HNRNPL	-364		
chr2	98612689	98612999	1.01	1.00E+00	TMEM131	-490		
chr21	36421473	36421930	1.06	1.00E+00	RUNX1	-107		
chr3	119813699	119814076	1.01	1.00E+00	GSK3B	-624		
chr5	87564585	87564839	1.12	1.00E+00	TMEM161B	-47		
chr8	52438673	52438917	1.10	1.00E+00	PXDNL	283210		
chr9	35603900	35604443	0.96	1.00E+00	TESK1	-1109		
chr9	35657687	35658045	1.15	1.00E+00	CCDC107	-445		
chr9	98638115	98638504	1.06	1.00E+00	C9orf102	410		
chr1	10003359	10003701	0.95	1.00E+00	LZIC	-690	NMNAT1	44
chr1	21844491	21844870	1.02	1.00E+00	ALPL	8823	RAP1GAP	133667
chr1	154832145	154832564	1.13	1.00E+00	ADAR	-251673	KCNN3	10399
chr1	155196392	155196689	0.95	1.00E+00	GBA	14512	MTX1	18051
chr10	27015073	27015383	1.13	1.00E+00	PDSS1	28633	ABI1	134731
chr10	90595461	90595715	0.90	1.00E+00	STAMBPL1	-44438	LIPM	33101
chr11	537194	537465	0.88	1.00E+00	HRAS	-1780	LRRC56	-192
chr11	18417613	18417891	1.08	1.00E+00	LDHC	-16101	LDHA	1816
chr12	31556135	31556420	1.10	1.00E+00	OVOS1	-197190	DENND5B	187674
chr12	31789580	31789917	1.11	1.00E+00	DENND5B	-45797	C12orf72	-22860
chr12	42863075	42863573	0.99	1.00E+00	PRICKLE1	14092	PPHLN1	143377
chr12	113488601	113488939	0.96	1.00E+00	DTX1	-6892	OAS2	72496
chr12	125412281	125412601	1.07	1.00E+00	UBC	-12864	DHX37	61226
chr12	131464385	131464733	0.98	1.00E+00	SFRS8	-731076	GPR133	26107
chr13	28033668	28033946	0.86	1.00E+00	MTIF3	-9096	LNX2	160913
chr13	41495697	41496028	1.11	1.00E+00	ELF1	97645	SLC25A15	132316
chr14	22392757	22393045	0.75	1.00E+00	TRA	-78444	TRA@	30331
chr14	64108129	64108922	1.01	1.00E+00	PPP2R5E	-98447	SGPP1	86230
chr14	104181770	104182030	0.84	1.00E+00	ZFYVE21	-245	XRCC3	-77
chr15	85947413	85947671	1.13	1.00E+00	AKAP13	23671	KLHL25	390647
chr16	81842614	81843074	1.01	1.00E+00	PLCG2	29914	SDR42E1	202249
chr17	3864769	3865117	1.00	1.00E+00	P2RX1	-44983	ATP2A3	2793
chr17	42428173	42428449	0.96	1.00E+00	GRN	5820	FAM171A2	12924
chr18	23770494	23770809	1.01	1.00E+00	TAF4B	-35757	PSMA8	56836
chr19	1087812	1088261	1.00	1.00E+00	POLR2E	7354	HMHA1	20863
chr19	54296220	54296530	0.97	1.00E+00	NLRP12	31273	DPRX	161065

chr2	178128166	178128461	1.05	1.00E+00	NFE2L2	1545	HNRNPA3	50892
chr20	37075062	37075338	1.04	1.00E+00	RALGAPB	-26286	LBP	100315
chr20	55043451	55043852	0.97	1.00E+00	CASS4	56338	GCNT7	57329
chr22	41799825	41800135	0.99	1.00E+00	TEF	22017	TOB2	43047
chr6	27447354	27447693	1.12	1.00E+00	ZNF184	-6627	HIST1H2BL	328185
chr6	137084858	137085332	1.34	1.00E+00	MAP7	-213303	MAP3K5	28561
chr6	151302256	151302562	1.00	1.00E+00	AKAP12	-258725	MTHFD1L	115718
chr7	106358704	106358962	1.16	1.00E+00	NAMPT	-433195	PIK3CG	-147091
chr7	128731604	128731853	0.90	1.00E+00	TSPAN33	-52983	TNPO3	-36531
chr8	144363677	144364050	1.20	1.00E+00	ZNF696	-9695	GLI4	14257
chr9	107537686	107538068	1.08	1.00E+00	NIPSNAP3B	11426	ABCA1	152559
chr9	117501179	117501488	0.93	1.00E+00	TNFSF15	67074	C9orf91	127628
